# Identification of a novel interaction between corticotropin releasing hormone (Crh) and macroautophagy

**DOI:** 10.1038/srep23342

**Published:** 2016-03-18

**Authors:** Panagiotis Giannogonas, Athanasia Apostolou, Antigoni Manousopoulou, Stamatis Theocharis, Sofia A. Macari, Stelios Psarras, Spiros D. Garbis, Charalabos Pothoulakis, Katia P. Karalis

**Affiliations:** 1 Clinical, Experimental Surgery, & Translational Research, Biomedical Research Foundation of the Academy of Athens, 11527, Athens, Greece; 2 Faculty of Medicine, University of Crete, 71003, Iraklion, Crete, Greece; 3 Faculty of Medicine, School of Health Sciences, University of Athens, 11527, Athens, Greece; 4 Centre for Proteomic Research, Institute for Life Sciences, University of Southampton, SO17 1BJ, Southampton, UK; 5 Clinical and Experimental Sciences Unit, University of Southampton, Faculty of Medicine, SO16 6YD, Southampton, UK; 6 Cancer Sciences Unit, University of Southampton, Faculty of Medicine, Southampton SO16 6YD, United Kingdom; 7 Cell Biology Division, Center of Basic Research I, Biomedical Research Foundation of the Academy of Athens, 11527, Athens, Greece; 8 Center for Inflammatory Bowel Diseases, Division of Digestive Diseases, David Geffen School of Medicine, University of California at Los Angeles, 90095, Los Angeles, CA, USA; 9 Division of Endocrinology, Children’s Hospital, 02115, Boston, MA, USA

## Abstract

In inflammatory bowel disease (IBD), compromised restitution of the epithelial barrier contributes to disease severity. Owing to the complexity in the pathogenesis of IBD, a variety of factors have been implicated in its progress. In this study, we report a functional interaction between macroautophagy and Corticotropin Releasing Hormone (Crh) in the gut. For this purpose we used DSS colitis model on *Crh −/−* or wild-type (wt) with pharmacological inhibition of autophagy. We uncovered sustained basal autophagy in the gut of *Crh −/−* mice, which persisted over the course of DSS administration. Autophagy inhibition resulted in partial rescue of *Crh −/−* mice, while it increased the expression of Crh in the wt gut. Similarly, Crh deficiency was associated with sustained activation of base line autophagy. *In vitro* models of amino acid deprivation- and LPS-induced autophagy confirmed the *in vivo* findings. Our results indicate a novel role for Crh in the intestinal epithelium that involves regulation of autophagy, while suggesting the complementary action of the two pathways. These data suggest the intriguing possibility that targeting Crh stimulation in the intestine may provide a novel therapeutic approach to support the integrity of the epithelial barrier and to protect from chronic colitis.

A basic objective of therapeutics is maintenance of tissue homeostasis^1^. Major obstacles remain for such an objective in common diseases such as inflammatory bowel disease (IBD), primarily stemming from current gaps to its pathogenesis etiology. Genome wide association studies (GWAS) have provided insight on possible distinct pathways and mechanisms involved in this regard. IBD has a clear, albeit complicated, genetic background implicating over 150 genes^2^, whose significant component is linked to innate and adaptive immunity, or to homeostatic processes such as endoplasmic reticulum or oxidative stress. A consistent finding of GWAS in IBD patients confirmed by experimental studies, is the significance of autophagy in the progression of IBD^3^. Macroautophagy, from now on referred as autophagy, is a conserved process across different species that preserves cell survival and tissue homeostasis in states of stress by promoting energy availability^4^,^5^. Emerging evidence demonstrates the contribution of autophagy in a spectrum of human pathological conditions including cancer, infection, inflammatory, cardiovascular and neurodegenerative diseases^4^,^6^.

At the systemic level, homeostatic regulation is attributed to the coordinated function of the endocrine and the autonomic nervous systems^7^,^8^. Corticotropin Releasing Hormone/Factor (CRH or CRF) is secreted by the hypothalamus in response to endogenous and external stressors to promote the release of glucocorticoid by the adrenals and coordinate the systemic response^9^,^10^,^11^. Of interest, Crh is also expressed in a number of peripheral tissues including the mammalian gut, where it exerts immunomodulatory effects^12^. Intestinal biopsies from patients with IBD identified significantly induced Crh levels compared to control samples, similarly to findings from relevant experimental models^13^,^14^,^15^. In line with the later, Crh-null (*Crh −/−*) mice show increased susceptibility to the development of acute dextran sodium sulfate (DSS)-induced colitis^16^. Our working hypothesis is that tissue Crh plays a critical role in the maintenance of tissue homeostasis in response to specific challenges such as inflammatory stimuli.

Stress has been thought as a major mechanism driving induction of autophagy^17^. As Crh plays a central role in the elicitation of the stress response^8^, we raised the hypothesis for its putative role in the regulation of autophagy. To investigate this hypothesis, we chose the mouse model of DSS-induced colitis, a disease associated with altered autophagy^18^. In this model, development of colitis is driven by the activation of innate immunity due to disruption of the epithelial integrity^19^,^20^. *Crh −/−* mice show increased susceptibility to the development of DSS-induced colitis characterized by severely compromised ability to advance to the repair phase^16^.

This current study examined the possible interaction between Crh and autophagy in the regulation of intestinal inflammation using the DSS model of colitis. The study also examined the possible functional interplay between Crh and autophagy in the gut and its effect on the development of severe colitis relative to baseline conditions. Our study demonstrated the close interaction between Crh and autophagy in the progression of colitis and repair of the injured epithelium. We also indicate the role of Crh in the induction of autophagy in relevant *in vitro* systems. In total, our study provides clear evidence for the tight interaction between intestinal homeostatic processes, such as autophagy, and the “stress factor” Crh in the maintenance of the epithelial barrier and its restitution following innate immunity-driven colitis.

## Results

### Altered autophagy activation in the *Crh −/−* gut

**Figure 1:**
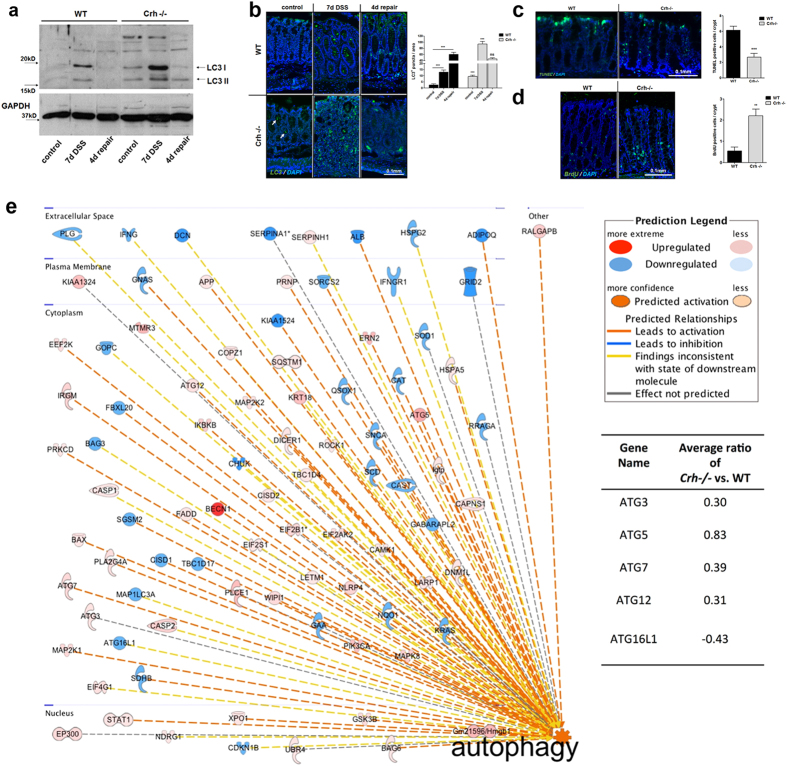
Sustained formation of autophagosomes in the *Crh −/−* gut. (**a**) Western blot analysis of LC3 I and II in whole lysates from colonic tissue of wt and *Crh −/−* mice in baseline conditions (control), following 7 days DSS administration and following 4d repair, indicating increased autophagy in the *Crh −/−* tissue (n = 3, 2 individual experiments). (**b**) Representative immunofluorescence images in colon from wt and *Crh −/−* mice under baseline conditions, following 7 days DSS administration and following 4d repair. Sections were stained for LC3 (green) and DAPI (blue). Quantification was performed using the image analysis software ImageJ v.1.46r. ***p < 0.001 (n = 6–10, 4 individual experiments) Representative images of colon from TUNEL assay (n = 12–14, 2 individual experiments) (**c**) and BrdU immunofluorescence staining (n = 5, 4 individual experiments) (**d**) from wt and *Crh −/−* mice under baseline conditions. Quantification is expressed as a number of BrdU or TUNEL positive cells per crypt. **p < 0.01, ***p < 0.001 (**e**) In silico interpretation of the proteomic results using the Ingenuity Pathway Analysis software tool showed increased autophagy in the intestine of Crh*−/−* mice compared to WT mice (p-value = 1.29E-7; activation z-score = 2.557). A number of autophagy-related proteins (ATGs) were identified, of which ATG3, ATG5, ATG7 and ATG12 were analyzed to be up-regulated whereas ATG16L1 down-regulated in the intestine of Crh*−/−* vs WT mice.

**Figure 2:**
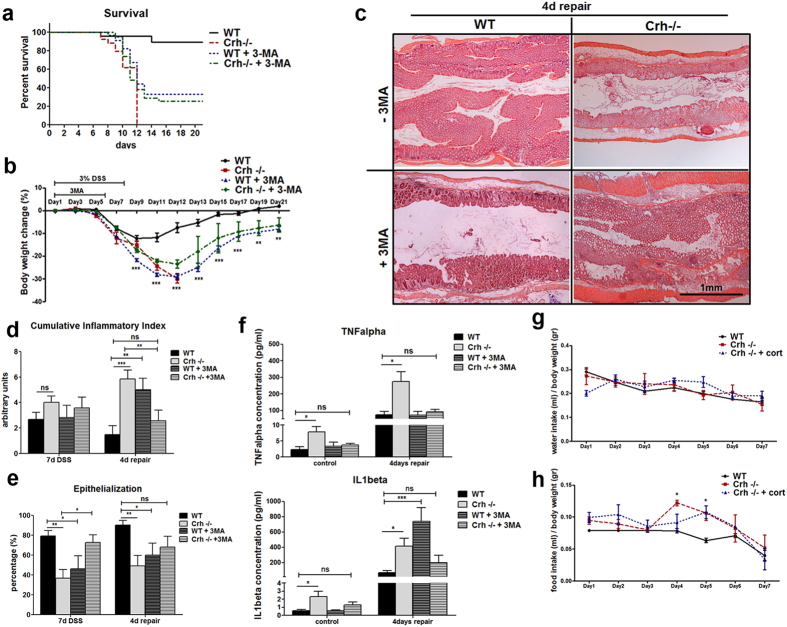
Pharmacological inhibition of autophagy (3-methyladenine) in *Crh −/−* mice during DSS administration, prolongs survival and ameliorates the inflammatory response. (**a**) Kaplan-Meier survival curve from wt (n = 53) and *Crh −/−* (n = 42) administered DSS, and wt (n = 34) and *Crh −/−* (n = 39) mice administered DSS and receiving daily injections of 3-MA during days 1–6 of DSS administration. (**b**) Body weight loss in wt and *Crh −/−* mice administered DSS, and wt and *Crh −/−* mice administered DSS and receiving daily injections of 3-MA (d1–6) of DSS administration. (**c**) Representative H&E images from wt and *Crh −/−* colon 4 days after 3% DSS administration (repair) with or without 3MA, depicting the extend of epithelial regeneration of the tissue. (**d**) Cumulative inflammatory index based on edema (scale 0–3), inflammatory indices (scale 0–3) and necrosis (scale 0–3), assessed at the completion (7 days) or 4 days after (repair) of 3% DSS administration. **p < 0.01, ***p < 0.001 (**e**) Epithelialization is expressed as a percentage of the tissue area covered by crypts at the completion (7 days) or 4 days after (repair) of 3% DSS administration. *p < 0.05, **p < 0.01 (**f**) Cytokines secreted from wt and *Crh −/−* colonic tissue explants from the indicated experimental groups above, isolated at the indicated times following the completion of DSS administration. *p < 0.05, ***p < 0.001 Water (**g**) and food (**h**) consumption of wt, *Crh −/−* and *Crh −/−* mice with corticosterone replacement during the course of DSS administration. *p < 0.05.

We have previously reported that *Crh −/−* mice show increased susceptibility to the development of DSS colitis and failure to survive the disease for more than 4 days after the completion of DSS administration^16^. Compromised autophagy predisposes to the development of severe colitis in mice^21^,^22^ whereas polymorphisms in autophagy genes have been found in patients with IBD^3^. Here, we assessed the possibility that the severe response of the *Crh −/−* mice to DSS might be also associated with defective activation of autophagy, in line with the role of stress in the activation of colitis^17^. To address this hypothesis, we first assessed the abundance of the autophagosome marker LC3 (isoforms I and II) in *Crh −/−* colonic tissues. LC3 is a ubiquitin-like protein incorporated in the newly forming inner and outer autophagosomal membranes^23^. The fusion of autophagosomes with endosomes or lysosomes, a central event in autophagy activation, is characterized by the rapid degradation of LC3 I to the LC3 II form. Therefore, assessment of the lysosomal turnover of LC3 provides a well established measurement of the macroautophagic activity^24^. We evaluated the levels of LC3 II in colonic tissues of wild-type (wt) and *Crh −/−* mice, seven days after the initiation of DSS administration (inflamed state), and four days following the cessation of DSS (repair phase)^25^ by Western blot analysis. Colitis resulted in significant induction of LC3 II formation, which declined as the disease advanced towards the repair phase (Fig. 1a). Contrary to our initial hypothesis, we identified increased levels of LC3 II in the DSS-treated *Crh −/−* colonic tissues, as compared to the wt samples, with no evidence for decline over time. Further, the *Crh −/−* mice did not progress to repair ((Fig. 2a–e), a process actively in place, as soon as 4 days after completion of the 7 days long DSS treatment. Completion of DSS treatment coincides with the peaking of inflammatory activity in both genotypes (Supplementary Figure 1a), significantly increased in the *Crh −/−* mice as we have previously reported^16^. As shown for the repair phase, in contrast to the active regeneration in the wt tissue, H&E analysis of the *Crh −/−* colon depicted ongoing inflammatory process with minimal evidence of repair in the injured epithelium (Fig. 2c), further indicated by the sustained activation of inflammatory markers (Supplementary Table 3). As previously reported, the administration of DSS in drinking water induces a colitis that is characterized by hematochezia (blood in the stool), body weight loss, mucosal ulceration, and infiltration with neutrophil granulocytes^25^,^26^. In accordance with the histological features described^25^, the histopathological analysis in our DSS treated groups, reveals focal epithelial erosion with crypt atrophy and diffuse chronic inflammatory infiltration, indicative of the induction of DSS colitis. Mucosal damage and inflammatory signs are profound in the colonic tissue in both wild-type and *Crh −/−* mice during the acute phase of DSS-induced colitis (7days DSS, Supplementary Figure 1), whereas during the repair phase of colitis only the *Crh −/−* mice continue to exhibit signs of active inflammation (4 days repair, Fig. 2c). Cumulative disease index calculated based on edema (scale 0–3), inflammatory indices (scale 0–3) and necrosis (scale 0–3) reflects the impaired repair process in the intestinal epithelial barrier associated with Crh deficiency (Fig. 2d). Notably, the increase in LC3 II expression in the *Crh−/−* intestinal tissues was evident even in the non-inflamed tissues (base line), in contrast to the almost undetectable expression of LC3 II in the wt tissues. Collectively, these findings suggest that Crh participates in the regulation of autophagy in the colon in basal and inflamed states (Fig. 1a,b). We have no evidence that this finding may be associated with altered microbial exposure, since treatment with broad-spectrum antibiotics (Supplementary Figure 2b,c) did not normalize the LC3 II immunoreactivity in the *Crh −/−* tissues. Impaired induction of autophagy in the intestine was shown to result in hyperplasia of goblet cells, compromised mucus secretion and reduced expression of mucin 2, all hallmark traits for inadequate host defense against invading microbes^27^,^28^. In agreement with the above, we found reduced number and hypoplastic appearance of Goblet cells in the *Crh −/−* colonic samples as compared to the wt tissues (Supplementary Figure 3)^28^. The constitutive activation of autophagy in the *Crh −/−* mouse gut was also confirmed by immunofluorescence, as use of an antibody that recognizes all isoforms of LC3 (Fig. 1b) revealed increase in the characteristic punctuated LC3 signal in the *Crh −/−* tissue samples^28^,^29^. In line with this observation are the induced proliferation and reduced apoptosis in the *Crh −/−* tissues^17^ (Fig. 1c,d). The effects of Crh-deficiency in the induction of autophagy are not restricted in the colon, as depicted by the significant increase in the characteristic LC3 punctuate staining in the bottom of the *Crh −/−* small intestine crypts^30^ (Supplementary Figure 2a). Finally, significantly lower levels of phosphorylated Akt in the *Crh −/−* colon in base line argue for Akt – independent induction of autophagy in this case^31^ (Supplementary Figure 4a,b). Further, no differences were detected between the inflammation-induced Akt phosphorylation in the colonic tissues of Crh*−/−* and wt mice, indicative of the minor contribution, if any, of Crh deficiency in this process.

Next, we set up to evaluate the impact of *Crh* deficiency itself, as opposed to the associated glucocorticoid insufficiency of the *Crh −/−* mice, in the regulation of autophagy. Corticosterone supplementation in the drinking water, 10 μg/ml for 1 week, maintains stable circulating corticosterone close to normal low levels, as we have previously shown^32^. There was no difference in food intake between the wt, *Crh −/−* and *Crh −/−* with corticosterone supplementation over the course of DSS administration (Fig. 2g,h). Notably, corticosterone administration reduced the punctate staining for LC3 of the *Crh −/−* colon tissue samples (Supplementary Figure 2b,c), although its levels remained significantly higher compared to those of the wt colon samples. This finding implies that locally secreted Crh in the colon has direct impact in controlling the formation of autophagosomes. These observations suggest that an increase in baseline autophagy in the *Crh −/−* gut, may be a mechanistic component recruited to overcome potential dysregulation in intestinal function due to compromised Crh expression. Interestingly, this adaptation cannot confer protection of the intestinal tissue from any additional superimposed stressors, such as DSS (Fig. 2a).

For the unbiased and in-depth evaluation of the Crh deficiency-associated changes to the autophagic “machinery”, we used high-precision global quantitative proteomics with *in silico* bioinformatics analysis to uncover related pathways and networks (Fig. 1e). A total of 6975 proteins were profiled at >95% confidence (q < 0.05) (Supplementary Table 1). Of these, 3774 were modulated in the *Crh−/−* vs. WT intestinal tissue (Supplementary Table 2). Bioinformatics interpretation of the modulated proteins using Ingenuity Pathway Analysis showed that autophagy was significantly increased in the intestine of *Crh−/−* vs. WT mice (p-value = 1.29E-7; activation z-score = 2.557). Three thousand nine hundred and sixty six (3966) proteins were modulated in the intestine of *Crh −/−* 7d DSS vs. WT 7d DSS mice (Supplementary Table 3) whereas 4299 were differentially expressed between WT 7d DSS vs. WT control and *Crh −/−* 7d DSS vs. *Crh −/−* control (Supplementary Table 4). Validation of this approach was provided by the obtained differences in inflammatory markers, in line with our previous studies. As such, myeloperoxidase levels were significantly increased in the intestine of *Crh−/−* 7d DSS vs. WT 7d DSS (Supplementary Figure 1b), whereas a number of cytokines associated with colitis, were expressed at higher levels in the intestine of *Crh −/−* 7d DSS vs. WT 7d DSS mice (Supplementary Table 3). These findings are also confirmed by the secreted cytokines from wt and *Crh −/−* colon explants following 7 days of DSS administration (Supplementary Figure 1c).

### Effects of pharmacological inhibition of autophagy in DSS colitis

**Figure 3:**
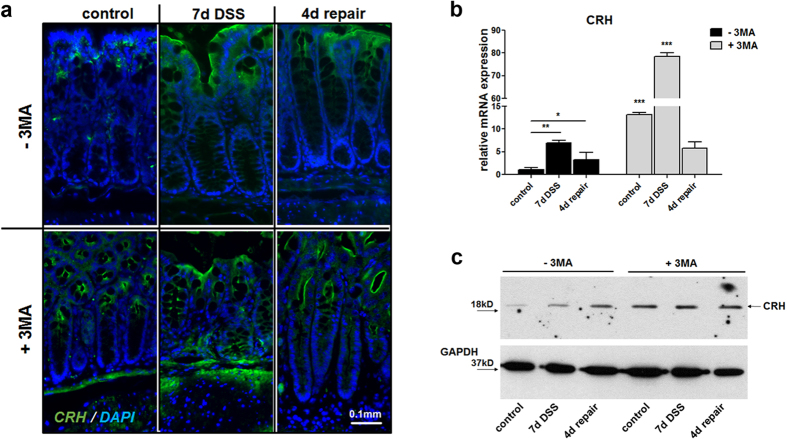
Autophagy inhibition positive regulates Crh expression and Crh protein levels. (**a**) Representative images from immunohistochemistry in colon from wt mice under baseline conditions (control), following 7 days DSS administration and following 4d repair, indicating increased Crh in 3MA-treated mice. Sections were stained for Crh (green) and DAPI (blue). (**b**) Quantitative RT-PCR for mCRH conducted on colon homogenates from wt and *Crh−/−* under baseline conditions (control), during (7d DSS) or 4 days after (4d repair) 3% DSS administration. *p < 0.05, **p < 0.01, ***p < 0.001 (**c**) Western blot analysis of CRH protein levels. Whole protein lysates were extracted from wt and *Crh −/−* colons under baseline conditions (control), during (7d DSS) or 4 days after (4d repair) 3% DSS administration with or without 3MA administration.

Although the impact of deficient activation of autophagy in colitis is well documented, there is no prior knowledge on the contribution of sustained activation of autophagy in this disease. We set up to evaluate the possible effect of induced autophagy in the progress of DSS colitis, by use of a common, non-specific, autophagy inhibitor 3-Methyladenine (3-MA)^33^. We evaluated two different dosages of 3-MA administered daily in DSS-treated mice, i.e. 10 mg/kg as per a previous study^34^ or 20 mg/kg (~4 mM) that is closer to the maximal recommended dosage of 5 mM, and remained with the later due to its reproducible efficacy in this model. Wild-type mice administered DSS and 3-MA worsened the disease as shown by increased body weight loss, lethality (34%)^35^ and IL1 beta (Fig. 2a,b,f). These findings agree with the increased susceptibility to colitis of mice with genetic deficiencies in autophagy-related genes^28^. Notably, DSS administered *Crh −/−* mice treated with 3-MA showed dramatic improvement in survival (50% on day 12 as opposed to 100% lethality in the vehicle-treated group) (Fig. 2a). As shown, their inflammatory response was significantly reduced as shown by the increased formation of new crypts, and reduced edema, cellular infiltration (Fig. 2c, Supplementary Figure 1a), and locally released TNFalpha and IL1beta^36^ (Fig. 2f). Thus, the overall clinical picture of colitis was indistinguishable between similarly treated wt and *Crh −/−* mice. These findings indicate that in states of Crh deficiency, inhibition of autophagy during colitis prevents the development of severe, lethal disease (Fig. 2c) in further support of the dynamic interaction between Crh and autophagy. Building on these observations, we assessed the effect of autophagy inhibition during colitis in the regulation of Crh in the colon of wt mice. Treatment with 3MA of mice with DSS-induced colitis resulted in further increase of Crh immunoreactivity in the inflamed colon (Fig. 3a–c). Crh was particularly expressed in myeloid cells, but not in the macrophages (F4/80 positive cells), surrounded by Crh positive cells (white arrows, Supplementary Figure 5). Our findings highlight the dynamic regulation of autophagy over the progress of colitis and the need to further clarify the specific factors implicated in the epithelial regeneration^5^.

### *In vitro* confirmation of the interaction between Crh and macroautophagy

**Figure 4:**
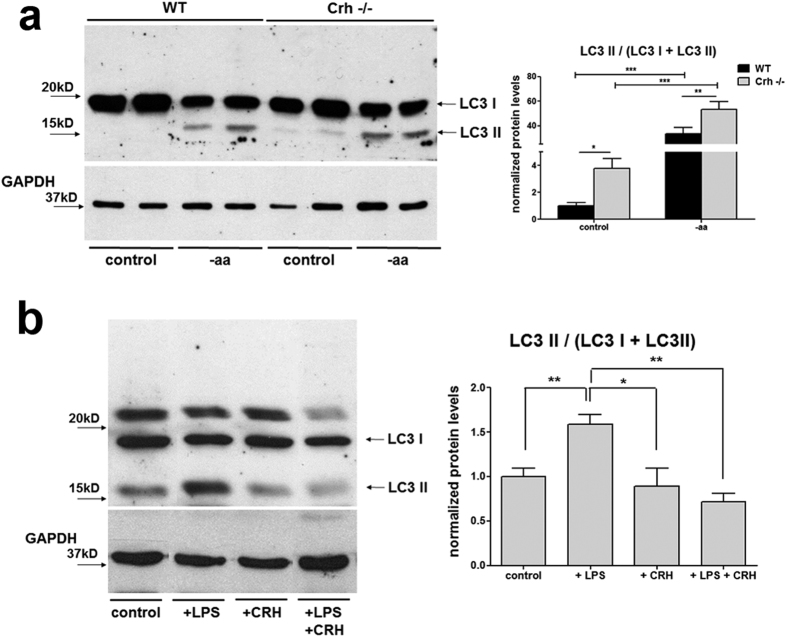
The effect of Crh in the induction of autophagy *in vitro*. (**a**) Western blot analysis and quantification of LC3 I and II protein levels in whole lysates from wt and *Crh −/−* MEFs in baseline and following 4 h incubation with aminoacid deprived medium. Autophagic activity is induced during aminoacid deprivation, as expected, in both wt and *Crh −/−* MEFs but the ratio of LC3 II/(LC3 I + LC3 II) in *Crh −/−* MEFs is continuously higher than in wt MEFs. *p < 0.05, **p < 0.01, ***p < 0.001 (n = 6, 3 individual experiments) (**b**) Western blot analysis and quantification of LC3 in a model of LPS induced autophagy in Raw264.7 cells. Following 16 h of LPS stimulation (1 μg/ml), CRH coadministration (10^−7^ M) reduces the protein levels of LC3 II compared to cells treated with LPS alone. *p < 0.05, **p < 0.01 (n = 8, 2 individual experiments).

To identify the potential impact of Crh deficiency in autophagy induction beyond the gut we used an *in vitro* model based on amino acid (aa) deprivation of mouse embryonic fibroblasts (MEF). This model relies on starvation, the prototypic stimulus, for the induction of autophagy in a very reproducible manner^37^,^38^. LC3 II was assessed by western blot analysis in wt and *Crh −/−* MEFs subjected to amino acid deprivation for 4 h. As shown (Fig. 4a), the ratio of LC3 II /LC3 I isoforms, both at baseline and following aa deprivation, was significantly increased in the *Crh −/−*, compared to the wt, MEFs. This finding confirms the hypothesis generated by our *in vivo* findings above, that Crh deficiency has direct implications in the regulation of autophagic activity. To further elucidate the effects of Crh on autophagy in states of immune activation, we used murine Raw264.7 an *in vitro* model of LPS-induced autophagy^39^. Following 16 h of LPS stimulation (1 μg/ml), there is significant induction of LC3 II, abolished by co-administration of LPS and Crh (10^−7^ M) (Fig. 4b). Crh treatment alone had no effect on the constitutive activation of LC3 II. Possible reasons for this include the endogenous Crh leading to the maximal possible effect in base line autophagy or that Crh is only effective when superimposed to additional challenges, such as the LPS-induced immune activation. The latter would be in line with the physiological role of Crh in the control of stress-induced responses.

## Discussion

Autophagy, a physiological process recruited to maintain cellular energy homeostasis and repair^40^, is induced by starvation and other stressors such as infections and inflammatory diseases, including colitis. Here we studied the effect of Crh, a major mediator of the systemic response to stressors, in the regulation of autophagy activation in the mouse gut following DSS colitis. Crh exerts immunomodulatory effects both *in vivo* and *in vitro*^9^,^10^,^11^. Crh and its receptors are expressed in the intestine of humans and rodents^12^, in increased abundance during inflammation and colitis^41^,^42^. Crh deficiency in mice is associated with higher susceptibility to innate immune responses and the development of associated colitis. Along these lines, we have previously reported that *Crh −/−* mice develop severe DSS colitis^16^, in contrast to the protective effects of *Crh* deficiency in other models of inflammation not dependent on innate immunity such as carrageenan^43^ or TNBS-induced enteritis^14^. Crh deficiency results in glucocorticoid insufficiency, which is normalized by administration of corticosterone in their drinking water^44^. We have previously shown that intestinal Crh deficiency is associated with altered innate immune responses and infectious enteritis, independent of their glucocorticoid insufficiency^45^.

Autophagy is induced to promote cell survival by providing energy, and thus contribute in the maintenance of homeostasis^4^,^46^. To our surprise, we found sustained activation of autophagy in the *Crh −/−* mouse colon at baseline and its further induction by DSS, as depicted by increased formation of LC3 II (Fig. 1a,b). Sustained activation of basal autophagy was only partially corrected by normalization of the corticosterone levels of the *Crh −/−* mice (Supplementary Figure 2b,c), indicating a direct Crh deficiency-dependent effect on autophagy. The inhibitory effects of glucocorticoid on autophagy have been previously reported in other pathophysiological settings^47^.

Our finding of activation of autophagy in the *Crh −/−* colon was not in line with our hypothesis of compromised autophagy, potentially underlying the severe colitis in the *Crh −/−* mice. The non-targeted examination of the causative agents implicated with this phenomenon using global and in-depth proteomics analysis confirmed the association between *Crh* deficiency and the autophagy pathway in the intestinal tissue. Of interest, in contrast to the induced autophagy in the *Crh −/−* intestinal tissue, Atg16l1 identified by proteomics to be significantly down regulated. As previously shown, mice with Atg16l1 gene deletion have resistance to bacterial infection-induced enteric disease^48^, whereas polymorphisms in Atg16l1 are associated with increased susceptibility to IBD in humans^49^. These findings raise the hypothesis that Atg16l1 may not be part of the cascade of factors driving the induction of autophagy in the *Crh −/−* gut, modestly surprising given the multimodal regulation of this process. Similarly, we show compromised Akt phosphorylation, that is upstream of Atg16l1, in the Crh*−/−* colon in base line (Supplementary Figure 3), in line with the reported Crh – mediated induction of PI3K-Akt^50^,^51^. The exact level of interaction of Crh and Atg16l1 will be addressed in future studies in primary cells genetically engineered to carry the polymorphism associated with IBD or not.

Blockade of autophagy via administration of 3-MA rescued around 50% of the *Crh −/−* mice from detrimental colitis. Notably, the progress of DSS colitis was similar between wt mice treated with 3-MA, and *Crh −/−* non- 3-MA treated mice, based on the increased lethality during the repair phase. These data support the hypothesis that autophagy is necessary to support mucosal repair and regeneration following injury, possibly by recycling the dying cells’ catabolism products. Defects in this process may drive additional immune activation that interferes with proper development of the newly formed crypts promoting the chronicity, rather than the resolution of inflammation^52^. These results agree with studies in mice with mutations in autophagy genes that develop severe colitis^21^,^22^. Similar mutations were found by genome-wide association studies (GWAS) in IBD patients highlighting the significance of proper activation of autophagy in the pathogenesis of colitis^3^. The severe colitis in the *Crh −/−* mice, or in the wt mice with blockade of autophagy, and the significant amelioration of colitis in *Crh −/−* mice treated with 3MA, indicate a dynamic interplay between the pathways driving the activation of Crh and autophagy in the intestinal epithelia. Notably, optimal regulation of colitis occurred in the presence or absence of both mechanisms, suggesting their cooperation is instrumental to keep in balance both the active and the resolution phases of colitis. This data highlights the complex, time- and inflammation-phase dependent regulation of autophagy induction while they provide evidence for the contribution of additional tissue expressed factors in this process.

Finally, the findings with the starvation-induced autophagy in MEFs^37^,^38^ (Fig. 4a) demonstrate the impact of Crh deficiency in LC3 II regulation beyond the intestinal tissue. Further, Crh blocked the LPS induced autophagy in the RAW murine line of macrophages, a model of innate immune activation^39^ (Fig. 4b). A number of reports have shown induction of autophagy in macrophages following LPS-activation of TLR4 signaling^39^,^53^,^54^ or simply, exposure to proinflammatory cytokines, such as TNFalpha and IL1beta^55^,^56^. The inhibitory effects of Crh in this process could also underlie the significantly lower LPS-induced IL1 activation in the *Crh −/−* macrophages and other tissues^57^,^58^. Collectively, our current findings suggest that Crh and autophagy operate primarily via non-overlapping, complementary pathways to regulate the development and the outcome of DSS-induced colitis. Furthermore, these data raise the possibility that Crh operates as a brake for the sustained activation of autophagy, an effect in line with its role in the adaptive response to stressors.

In summary, here we describe a missing function of the stress hormone Crh in the regulation of autophagy activation. We present evidence that this effect is linked to maintenance of gut homeostasis under basal conditions, as shown by our findings in Crh deficient mice. Our data from the DSS colitis uncover the contribution of tissue Crh in the regulation of innate immunity-induced autophagy activation, similar to its HPA axis-mediated effects in the systemic adaptation to stressors^59^,^60^. Future studies are required to address the potential beneficial effects of Crh agonists in the support of epithelial restitution in IBD, and other intestinal diseases. These studies may contribute in the ongoing efforts for identification of small molecules targeting tissue-specific activation of autophagy for a spectrum of therapeutic applications^60^.

## Materials and Methods

### Animals and animal care

wt and *Crh −/−* male mice of 129xC57BL/6 genetic background, weighing 25–30 g and aged 10–12 wks were used in this study. Animal housing and care was performed according to NIH guidelines; all experimental procedures were approved by the Animal Care and Use Committee of Boston Children’s Hospital, and of BRFAA in Athens, Greece. The animals were housed under controlled conditions (temperature 22 °C; 12 h light/dark cycle; lights on at 7 am) and were given free access to standard laboratory pellet formula and tap water. Daily inspection of cages for food spillage and monitoring of body weight and food intake was done for the duration of the study.

### DSS colitis

Mice (n = 5–10/group, four independent cohorts) were given 3% DSS (M.W. = 36,000–50,000 kDa; MP Biomedicals) in their drinking water for 7 days. To study the repair phase following colitis, the DSS solution was replaced with clean water for 4 days. Mice were evaluated twice/day for signs of moribund state, diarrhea and rectal bleeding. At day 3 or 7 post-DSS administration, or as described in the Results, the mouse colon was excised, and segments of the transverse colon (1 cm) were fixed in 4% paraformaldehyde (PFA) and processed for hematoxylin and eosin (H&E) staining. For the autophagy inhibition experiments, 20 mg/kg 3-methyladenine (3MA, M9281, Sigma) in w.f.i., was administered daily (day1–6) by intraperitoneal injections. Vehicle (w.f.i.)-injected wt and *Crh −/−* mice served as controls.

### Corticosterone replacement

Corticosterone (27840, Sigma) was added in the drinking water at final concentration of 10 μg/ml for 1 week, replaced with fresh solution every 2 days.

### Histological analysis

Colons were fixed in 4% PFA and embedded in paraffin. Blocks were serially sectioned vertical to the axis of the lumen. For H&E and Alcian Blue/PAS stainings, tissues were processed according to standard protocols. The histologic severity of colitis was graded in a “blinded” fashion by a pathologist blinded to samples’ identity, on a 0–3 ascending scale. Cumulative inflammatory index, a descriptive term, represents the average score of each section following characterization and rating for based on edema, inflammatory cells and necrosis in most affected regions. For the goblet cells characterization, well longitudinally sectioned crypts were selected and examined by counting the number of Alcian Blue/PAS stained cells along the crypt and expressed as number of goblet cells per crypt. The size of goblet cells was determined by randomly selecting 100 Alcian Blue/PAS stained cells per condition and the area was measured with ImageJ v1.46 software.

### Cytokine measurement

Intestinal tissue segments (2 cm) from the descending colon were collected from all experimental groups, and incubated for 24 hours in RPMI medium supplemented with 10% fetal bovine serum (FBS) and 1% penicillin/streptomycin. The cultures were incubated at 37 °C in a humidified 95% O_2_/5% CO_2_ atmosphere. At the end of each culture period, the supernatants were collected and stored at −80 °C until they were assayed. Quantification of secreted cytokines was performed using ELISA kits (Ready-Set-Go!® ELISA, eBioscience) according to manufacturer’s instructions.

### Quantitative proteomics

Colon from WT mice (n = 6), *Crh*−/− mice (n = 6), WT mice after 7 days exposure to DSS (n = 6) and *Crh*−/− mice after 7 days exposure to DSS (n = 6) were dissolved in 200 μL of 0.5 M triethylammonium bicarbonate, 0.05% sodium dodecyl sulfate and homogenised using the FastPrep®-24 Instrument (MP Biomedicals, Santa Ana, CA, USA). Lysates were subjected to pulsed probe sonication (Misonix, Farmingdale, NY, USA) and centrifuged (16,000 g, 10 min, 4 °C). The supernatant was measured for its protein content using the Direct Detect^TM^ system (Merck Millipore, Darmstadt, Germany). From each lysate, 33.3 μg of protein was pooled to form two biological replicates for each of the four conditions (WT, *Crh−/−*, WT 7d DSS, *Crh−/−* 7d DSS). Lysates were then subjected to reduction, alkylation, trypsin proteolysis, 8-plex iTRAQ labeling and two-dimensional liquid chromatography, tandem mass spectrometry analysis as described previously^61^,^62^,^63^,^64^. The unprocessed raw data files were submitted to Proteome Discoverer 1.4 for target decoy searching with SequestHT as reported^61^,^62^,^63^,^64^. Quantification ratios were median-normalized and log_2_ transformed. The threshold of percent co-isolation excluding peptides from quantification was set at 30. A one-group T-Test was performed to identify proteins that were modulated in the intestine from *Crh −/−* vs. WT and *Crh −/−* 7d DSS vs. WT 7d DSS mice. A two-group T-Test was performed to identify proteins that were differentially expressed between WT 7d DSS vs. WT control and *Crh −/−* 7d DSS vs. *Crh −/−* control. A *p-value* ≤ 0.05 was considered significant. Proteomics data were deposited to the ProteomeXchange Consortium via the PRIDE partner repository (dataset identifier PXD002886).

The *Diseases and Functions* feature of the Ingenuity Pathway Analysis software tool (http://www.ingenuity.com) was used to identify biological processes significantly over-represented in the modulated proteome. *P-values* ≤ 0.05 were considered significant.

### Western blot

Western blot analysis was performed by standard methodology. Antibodies against LC3 (1:1000, PM036, MBL), phospho-Akt (1:1000, 4060P, Cell Signaling), total-Akt (1:1000, 9272, Cell Signaling), CRH (1:500, NBP1-35703, NOVUS Biologicals) and GAPDH (1:20.000, AM4300, Ambion) were commercially available.

### Immunofluorescence staining

Paraffin embedded sections of intestine from wt and *Crh−/−* mice were processed according to the manufacturer’s protocol. Briefly, sections were deparaffinized, washed and antigen retrieved with 10 mM citrate buffer (pH 6.3). The tissues were then washed, covered with blocking buffer (20 mM HEPES, 1% bovine serum albumin (BSA), 135 mM NaCl) for 5 minutes and incubated overnight with the primary antibody (LC3, 1:2000, PM036, MBL; Crh, 1:100, NBP1-35703, Novus Biologicals; F4/80, 1:100, ab6640, Abcam; CD11b, 1:50, 553307, BD Pharmingen) diluted in blocking buffer. For BrdU immunohistochemistry (1:50 anti-BrdU antibody, ab6326, Abcam), following antigen retrieval, sections were sequentially incubated in 2N HCl for 30 minutes at 37 °C, in 0.1 M borate buffer for 10 min and in blocking solution (10% Normal Goat Serum). Finally, sections were washed and incubated with a secondary antibody (1:500 Alexa Fluor® 488 Goat Anti – rabbit IgG, Invitrogen) and mounted with VECTASHIELD® Mounting Medium with DAPI (H-1200, Vector Laboratories). F4/80/CD11b

### BrdU labeling and TUNEL assay

5-Bromo-2′-deoxyuridine (BrdU, B5002, Sigma Aldrich) uptake experiments were done by intraperitoneal injection (100 μg/g body weight) of nucleotides into mice for 3 hours before sacrifice. Tissues of BrdU-injected mice were processed for paraffin embedding. Subsequently, 5 μm sections were used for immunohistochemistry.

TUNEL assay was performed on 5 μm paraffin sections using DeadEnd™ Fluorometric TUNEL System (G3250, Promega) following the manufacturer’s instructions.

### Semi quantitative real-time PCR

RNA was extracted from colonic tissue using TRI reagent (Sigma). cDNA was made from 2 ug total RNA with MMLV reverse transcriptase and random hexamer primers (Life Technologies Inc., Rockville, MD, USA). PCR products from semi quantitative real-time PCR with primers for CRH (forward primer: 5′- CGC AGC CCT TGA ATT TCT TG -3′, reverse primer: 5′- GCG GGA CTT CTG TTG AGA TT -3′) were detected with SYBR Green (Molecular Probes, Life Technologies) on an ABI PRISM 7000 Sequence Detection System (Applied Biosystems). Real-time qPCR data were converted to linear data by calculating 2-ΔCt values for 18S normalized data.

### Cell lines and cultures

Mouse embryonic fibroblasts (MEFs) were isolated by female wt and *Crh −/−* mice, when embryos were 13.5–14.5 days old as previously described^64^. Isolated fibroblasts were cultured in Dulbecco’s modified Eagle’s medium (DMEM) with 10% fetal bovine serum (FBS), 1% penicillin/streptomycin and 1% L-glutamine. For the aminoacid deprivation experiments, cells were incubated for 4 h with a special formulation of Invitrogen DMEM-12320. RAW264.7 cells were maintained in Dulbecco’s modified Eagle’s medium containing 10% FBS. For LPS induced autophagy experiments, RAW264.7 cells were treated with LPS (1 μg/ml, L2630, Sigma Aldrich), Crh (10^−7^M, C3042, Sigma Aldrich) or both.

### Statistical Analysis

Results are expressed as mean ± SEM. Data was analyzed by two-tailed, unpaired, equal variance student’s t-test, one-way ANOVA followed by Bonferroni’s post hoc multiple comparison tests, or repeated measures ANOVA, as appropriate, using GraphPad Prism, version 5.00 (GraphPad Software Inc.).

## Additional Information

**How to cite this article**: Giannogonas, P. *et al.* Identification of a novel interaction between corticotropin releasing hormone (Crh) and macroautophagy. *Sci. Rep.* **6**, 23342; doi: 10.1038/srep23342 (2016).

## Supplementary Material

Supplementary Information
