# Supplementary material for: Identification of a novel interaction between corticotropin releasing hormone (Crh) and macroautophagy
Source: Sci Rep. 2016 Mar 18;6:23342. doi: 10.1038/srep23342 (PMC4796911; doi:10.1038/srep23342)
Supplement: Supplementary Information [file srep23342-s1.pdf]

Identification of a novel interaction between corticotropin releasing hormone (Crh) and macroautophagy

Panagiotis Giannogonas[1, 2], Athanasia Apostolou[1, 3], Antigoni Manousopoulou[4,5], Stamatis Theocharis[3], Sofia A. Macari[4,6], Stelios Psarras[7], Spiros D. Garbis[4,5,6], Charalabos Pothoulakis[8], Katia P. Karalis[1, 9]*

1. Clinical, Experimental Surgery, & Translational Research, Biomedical Research Foundation of the Academy of Athens, 11527, Athens, Greece
2. Faculty of Medicine, University of Crete, 71003, Iraklion, Crete, Greece
3. Faculty of Medicine, School of Health Sciences, University of Athens, 11527, Athens, Greece
4. Centre for Proteomic Research, Institute for Life Sciences, University of Southampton, SO17 1BJ, Southampton, UK
5. Clinical and Experimental Sciences Unit, University of Southampton, Faculty of Medicine, , SO16 6YD, Southampton, UK
6. Cancer Sciences Unit, University of Southampton, Faculty of Medicine, Southampton SO16 6YD, United Kingdom
7. Cell Biology Division, Center of Basic Research I, Biomedical Research Foundation of the Academy of Athens, 11527, Athens, Greece
8. Center for Inflammatory Bowel Diseases, Division of Digestive Diseases, David Geffen School of Medicine, University of California at Los Angeles, 90095, Los Angeles, CA
9. Division of Endocrinology, Children's Hospital, 02115, Boston, MA

***Corresponding author:** Katia Catherine Karalis, MD, PhD

Senior Investigator - Professor level

Biomedical Research Foundation of the Academy of Athens

4 Soranou Ephessiou St., 115 27 Athens, Greece

Telephone: +30 210 6597 465

e-mail: kkarali@bioacademy.gr, katia.karalis@childrens.harvard.edu

Corresponding author for proteomics: Spyros Garbis

Associate Professor / Head of Proteomics Group

Cancer Sciences Unit, University of Southampton, Faculty of Medicine, Southampton SO16 6YD, United Kingdom

e-mail: S.D.Garbis@soton.ac.uk

SUPPLEMENTARY FIGURES

Supplementary Figure 1

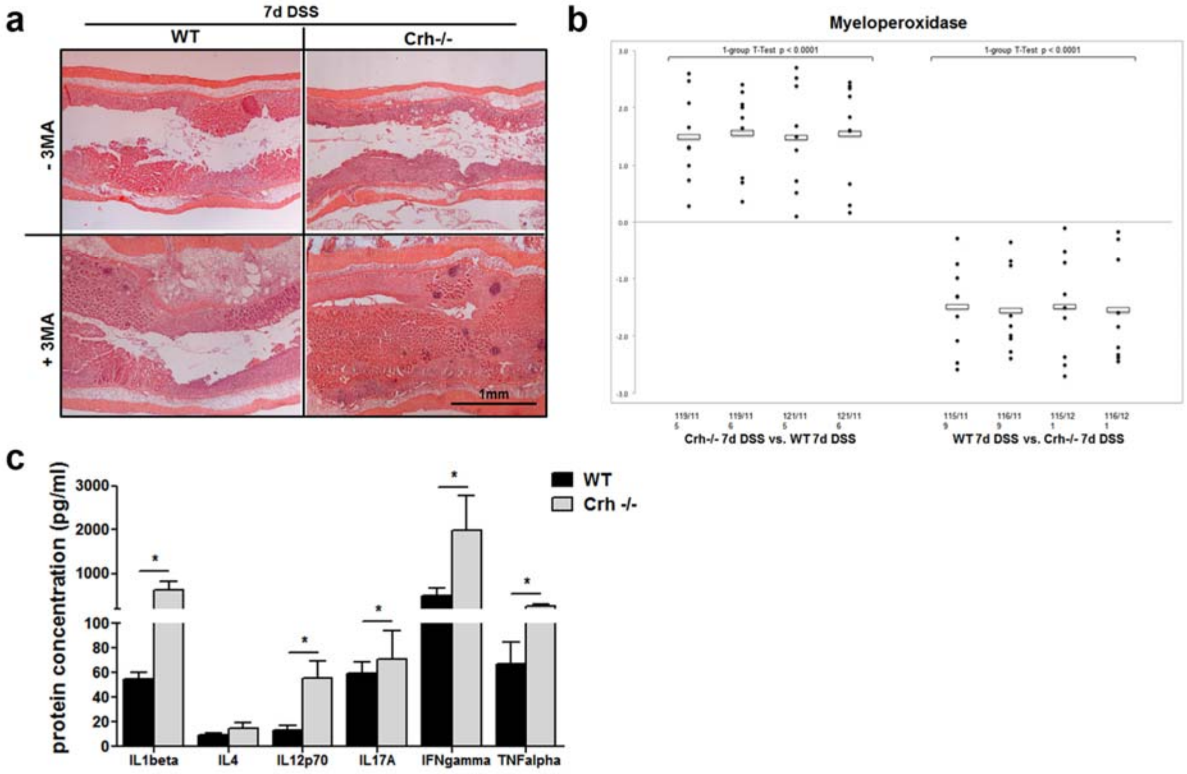

a. Representative H&E stained colons from WT and *Crh -/-* mice after 7 days of 3% DSS administration with or without 3MA, depicting focal epithelial erosion with crypt atrophy and diffuse chronic inflammatory infiltration characteristic of the acute phase of DSS induced colitis.

b. Myeloperoxidase levels were analyzed to be significantly increased in the intestines of the Crh-/- 7d DSS vs. WT 7d DSS (one-group T-test).

c. Cytokines secreted from wt and *Crh -/-* colonic tissue explants following 7 days of DSS administration. *$p < 0.05$

Supplementary Figure 2

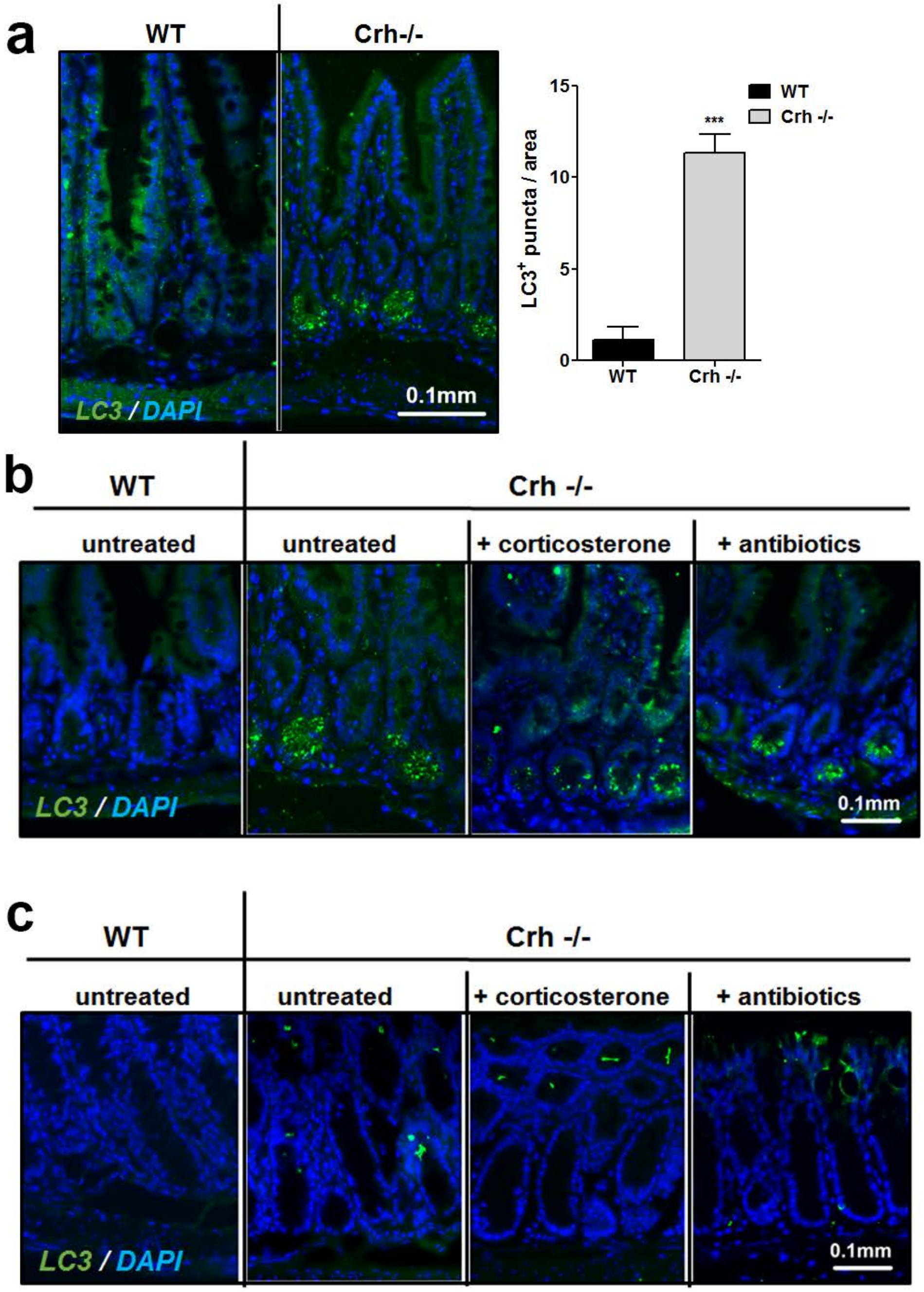

a. Representative images from immunohistochemistry in small intestine from wt and *Crh -/-* mice under baseline conditions. Sections were stained for LC3 (green) and DAPI (blue). Quantification was performed using the image analysis software ImageJ v.1.46r. $^{***}p < 0.001$

b. Representative images from immunohistochemistry in small intestine from wt and *Crh -/-* mice under baseline conditions (untreated), and following 1 week of corticosterone replacement or 10 days of broad spectrum antibiotics administration (200 mg/L ciprofloxacin and 500 mg/L metronidazole in drinking water). Sections were stained for LC3 (green) and DAPI (blue).

c. Representative images from immunohistochemistry in colon from wt and *Crh -/-* mice under baseline conditions (untreated), and following 1 week of corticosterone replacement or 10 days of broad spectrum antibiotics administration (200 mg/L ciprofloxacin and 500 mg/L metronidazole in drinking water). Sections were stained for LC3 (green) and DAPI (blue).

Supplementary Figure 3

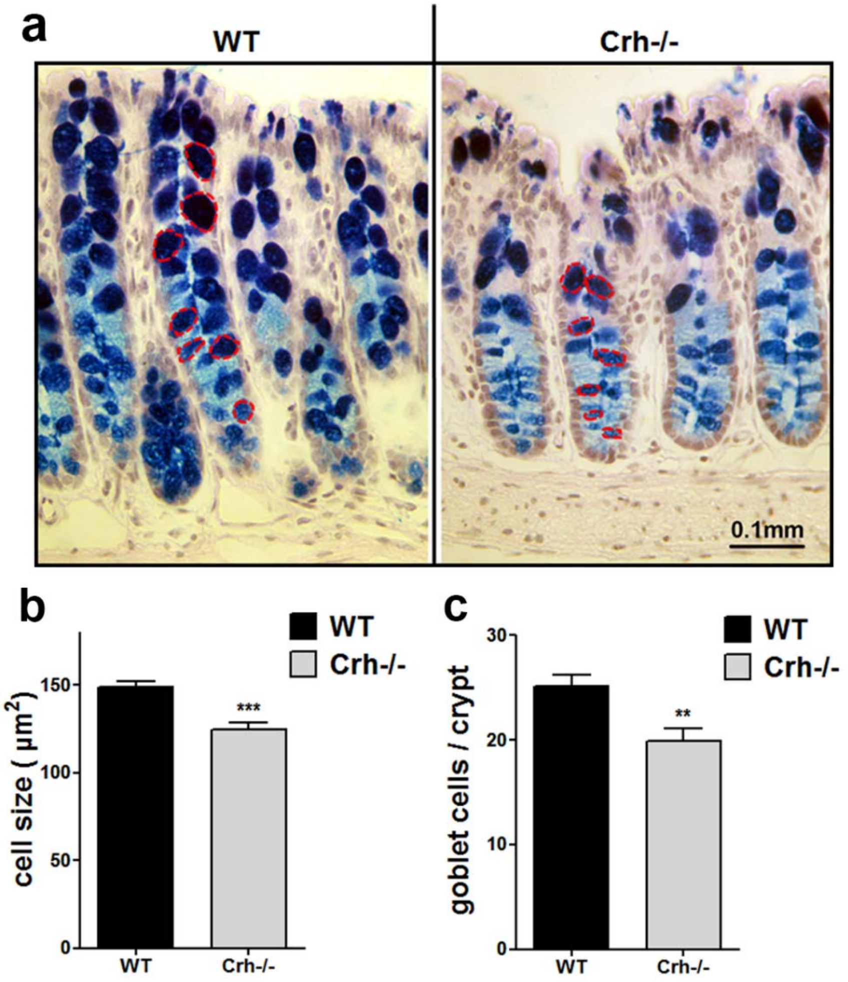

a. Alcian Blue / PAS stained colon sections of wt and *Crh -/-* mice showing the goblet cells (red dashed line).

b. Quantification of goblet cells size in the colon. *Crh -/-* mice exhibit mild goblet cell hypoplasia compared to wt mice. ***p < 0.001

c. Quantification of goblet cell number in the colon. Number is expressed as amount of cells per crypt, indicating reduced numbers of goblet cells in the *Crh -/-* mice. **p < 0.01

Supplementary Figure 4

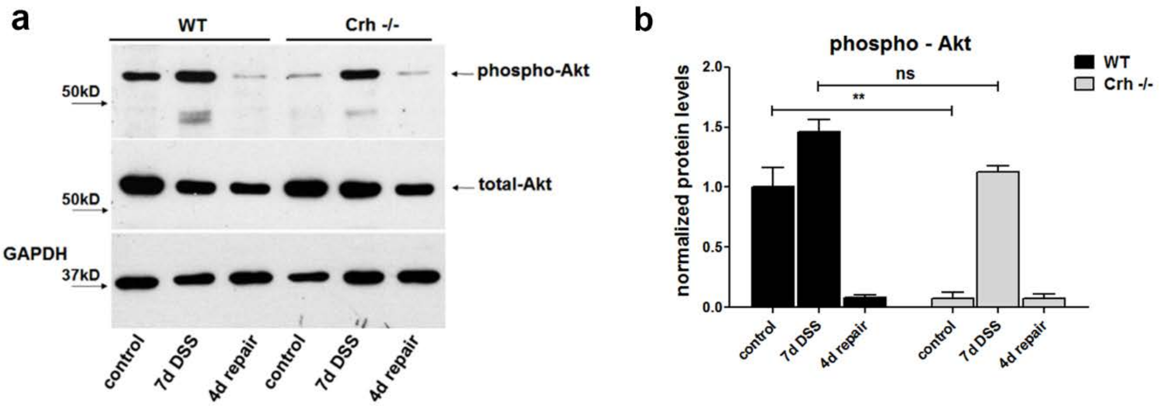

Western blot analysis (**a**) and quantification (**b**) of phospho-Akt protein levels in whole lysates from colonic tissue of WT and *Crh -/-* mice in baseline conditions (control), following 7 days DSS administration or 4d repair. phospho-Akt is highly suppressed in base line in the Crh deficient tissues, but not in tissues following 7 days DSS administration or 4 days repair compared to wt mice. **p < 0.01

Supplementary Figure 5

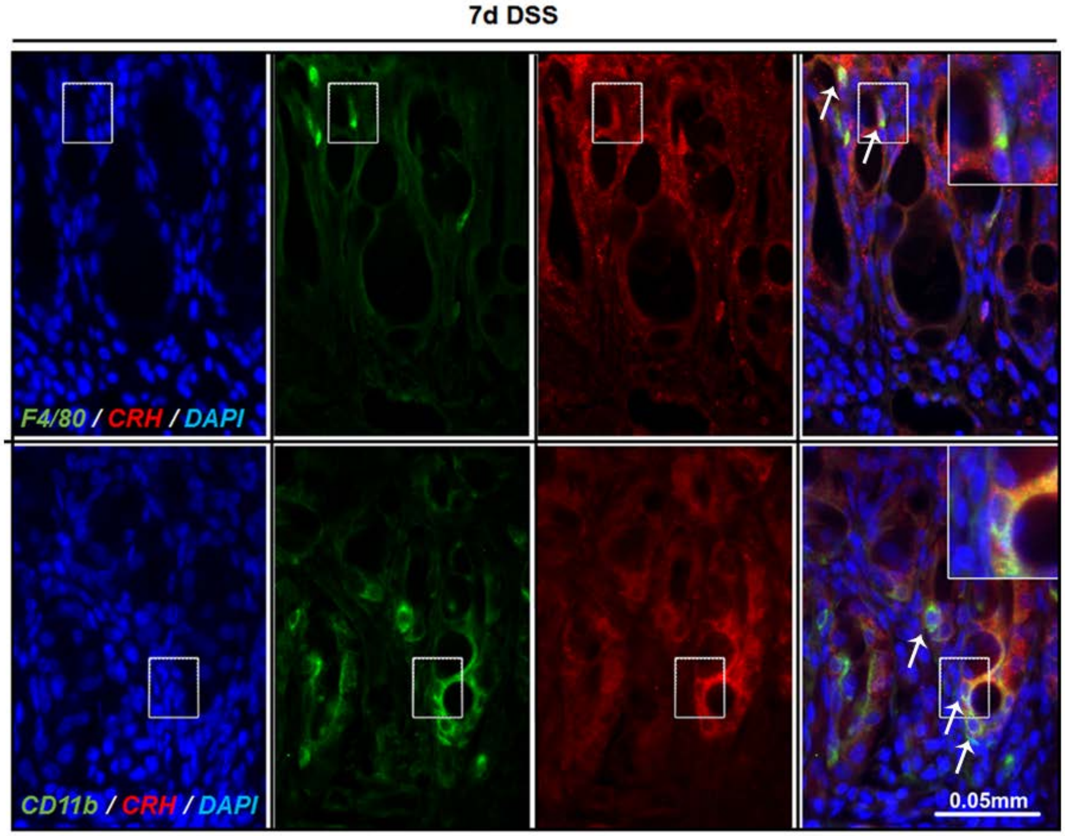

Representative images from double immunofluorescence staining in colon from wt and *Crh -/-* mice under baseline conditions, immediately (7 days) or 4 days after (repair) 3% DSS administration. Sections were stained for markers of macrophages, F4/80 or CD11b (green), CRH (red) and DAPI (blue).

SUPPLEMENTARY TABLES

Supplementary Table 1. Analysed proteome at over 95% confidence (q < 0.05)

Accession	Description	Σ Coverage	Σ# Proteins	Σ# Unique Peptides	Σ# Peptides	Σ# PSMs	Crh-/- (1) vs. WT (1)	Crh-/- (1) vs. WT (2)	Crh-/-(2) vs. WT (1)	Crh-/- (2) vs. WT (2)
Q8R1M8	Mucosal pentraxin OS=Mus musculus GN=Mptx1 PE=2 SV=1 - [MPTX_MOUSE]	17.81	2	2	2	77	2.81	2.27	2.67	2.08
F7CVL9	Beclin-1 (Fragment) OS=Mus musculus GN=Becn1 PE=2 SV=1 - [F7CVL9_MOUSE]	11.20	1	1	1	1	3.04	3.45	2.43	2.83
E9Q9Z6	Protein Olfr235 OS=Mus musculus GN=Olfr235 PE=2 SV=1 - [E9Q9Z6_MOUSE]	10.90	1	1	1	1	2.36	2.12	1.62	1.38
Q3TMQ6	Angiogenin-4 OS=Mus musculus GN=Ang4 PE=1 SV=1 - [ANG4_MOUSE]	23.61	3	5	5	14	2.73	2.34	2.72	2.33
O88310	Intelectin-1a OS=Mus musculus GN=Itln1 PE=1 SV=1 - [ITL1A_MOUSE]	6.71	3	2	2	3	2.35	2.67	2.27	2.59
P09602	Non-histone chromosomal protein HMG-17 OS=Mus musculus GN=Hmgn2 PE=1 SV=2 - [HMGN2_MOUSE]	15.56	4	1	1	2	0.17	1.01	0.43	1.27
P01629	Ig kappa chain V-II region 2S1.3 OS=Mus musculus PE=1 SV=1 - [KV2A4_MOUSE]	23.21	1	1	1	2	0.47	0.35	0.54	0.41
Q08EC7	Pla2g4c protein OS=Mus musculus GN=Pla2g4c PE=2 SV=1 - [Q08EC7_MOUSE]	24.55	6	15	16	104	2.24	2.13	2.17	2.08
Q6P8U6	Pancreatic triacylglycero lipase OS=Mus musculu GN=Pnlip PE=1 SV=1 - [LIPP_MOUSE]	23.23	1	6	6	54	2.43	2.72	2.52	2.88
P18419	Seminal vesicle secretory protein 4 OS=Mus musculus GN=Svs4 PE=1 SV=2 - [SVS4_MOUSE]	25.66	1	1	1	5	1.77	0.77	1.64	0.67
Q920R0	Alsin OS=Mus musculus GN=Als2 PE=1 SV=3 - [ALS2_MOUSE]	0.97	2	1	2	7	-0.92	-0.80	4.43	5.35
Q8VIB3	Type 2 lactosamine alpha-2,3-sialyltransferase OS=Mus musculus GN=St3gal6 PE=2 SV=3 - [SIA10_MOUSE]	16.41	1	4	4	21	1.09	1.04	1.13	1.09
Q8R3C6	Probable RNA-binding protein 19 OS=Mus musculus GN=Rbm19 PE=1 SV=1 - [RBM19_MOUSE]	3.78	2	3	3	4	1.57	1.95	1.07	1.45
Q7TPZ8	Carboxypeptidase A1 OS=Mus musculus GN=Cpa1 PE=2 SV=1 - [CBPA1_MOUSE]	12.89	1	4	4	9	1.40	2.36	1.31	2.42
Q9R1X5	Multidrug resistance-associated protein 5 OS=Mus musculus GN=Abcc5 PE=1 SV=2 - [MRP5_MOUSE]	3.69	2	3	3	4	1.07	0.92	1.15	1.00

D3Z3N3	RAS guanyl-releasing protein 2 (Fragment) OS=Mus musculus GN=Rasgrp2 PE=2 SV=1 - [D3Z3N3_MOUSE]	13.79	9	1	1	1	-0.48	1.13	-0.41	1.19
Q62472	Vomeronasal secretory protein 2 OS=Mus musculus GN=Lcn4 PE=2 SV=1 - [VNS2_MOUSE]	7.03	1	1	1	2	1.78	2.11	1.78	2.10
Q9D7Z6	Calcium-activated chloride channel regulator 1 OS=Mus musculus GN=Clca1 PE=1 SV=2 - [CLCA1_MOUSE]	55.20	1	39	39	1052	1.94	1.73	1.87	1.65
P97474-4	Isoform Pitx2Calpha of Pituitary homeobox 2 OS=Mus musculus GN=Pitx2 - [PITX2_MOUSE]	7.24	9	2	2	2	-1.41	-1.18	0.70	0.93
E9Q0D4	Protein Sbf2 OS=Mus musculus GN=Sbf2 PE=2 SV=1 - [E9Q0D4_MOUSE]	0.99	5	1	2	3	-0.07	-0.56	0.46	-0.04
D3Z7U6	Calpain-9 (Fragment) OS=Mus musculus GN=Capn9 PE=2 SV=1 - [D3Z7U6_MOUSE]	21.88	2	1	1	2	1.49	0.53	1.88	0.93
D3Z5X2	RAC-beta serine/threonine-protein kinase (Fragment) OS=Mus musculus GN=Akt2 PE=2 SV=1 - [D3Z5X2_MOUSE]	9.38	1	1	1	27	0.18	1.50	-0.54	0.67
Q8VHJ5	Serine/threonine-protein kinase MARK1 OS=Mus musculus GN=Mark1 PE=1 SV=2 - [MARK1_MOUSE]	8.18	2	2	5	10	-0.15	0.10	0.88	1.12
Q3USF0	UDP-GlcNAc:betaGal beta-1,3-N-acetylglucosaminyltransferase 6 OS=Mus musculus GN=B3gnt6 PE=2 SV=2 - [B3GN6_MOUSE]	8.70	1	3	3	4	0.14	0.94	0.22	1.01
Q80YA3-2	Isoform 2 of Phospholipase DDHD1 OS=Mus musculus GN=Ddhd1 - [DDHD1_MOUSE]	3.47	5	1	1	1	1.25	0.82	1.12	0.68
Q64285	Bile salt-activated lipase OS=Mus musculus GN=Cel PE=1 SV=1 - [CEL_MOUSE]	8.01	1	5	5	11	1.82	1.66	1.76	1.60
P70298	Homeobox protein cut-like 2 OS=Mus musculus GN=Cux2 PE=2 SV=2 - [CUX2_MOUSE]	2.73	1	2	2	2	1.36	0.99	1.28	0.91
Q8C790	Protein FAM221A OS=Mus musculus GN=Fam221a PE=2 SV=1 - [F221A_MOUSE]	15.61	2	2	2	7	1.22	1.13	1.46	1.50
Q91YE8	Synaptopodin-2 OS=Mus musculus GN=Synpo2 PE=1 SV=2 - [SYNP2_MOUSE]	53.91	2	2	41	460	2.25	1.77	2.17	1.69
Q7TPS0-2	Isoform 2 of Ribosomal protein S6 kinase alpha-6 OS=Mus musculus GN=Rps6ka6 - [KS6A6_MOUSE]	9.44	28	1	6	20	0.41	1.00	0.51	1.09
Q9ESL4-2	Isoform 2 of Mitogen-activated protein kinase kinase kinase MLT OS=Mus musculus GN=Mltk - [MLTK_MOUSE]	12.11	2	2	4	12	1.48	1.01	1.46	0.99

Q99L59	X-linked lymphocyte-regulated protein 3A OS=Mus musculus GN=Xlr3a PE=2 SV=1 - [Q99L59_MOUSE]	6.79	5	1	1	2	0.56	0.82	0.54	0.80
P03976	Ig kappa chain V-II region 17S29.1 OS=Mus musculus PE=1 SV=1 - [KV2A5_MOUSE]	11.50	1	1	1	1	-0.65	-0.90	-0.46	-0.71
P00688	Pancreatic alpha-amylase OS=Mus musculus GN=Amy2 PE=1 SV=2 - [AMYP_MOUSE]	47.24	4	19	19	226	1.93	1.97	1.93	1.95
Q0VDN7	RUN domain-containing protein 1 OS=Mus musculus GN=Rundc1 PE=2 SV=1 - [RUND1_MOUSE]	1.30	1	1	1	2	0.73	0.51	0.91	0.68
F6XZQ5	CMP-N-acetylneuraminate-beta-1,4-galactoside alpha-2,3-sialyltransferase (Fragment) OS=Mus musculus GN=St3gal3 PE=4 SV=1 - [F6XZQ5_MOUSE]	8.52	4	2	2	6	1.26	1.21	1.35	1.30
Q6NSU3-2	Isoform 2 of Glycosyltransferase 8 domain-containing protein 1 OS=Mus musculus GN=Glt8d1 - [GL8D1_MOUSE]	4.85	2	1	1	1	-0.41	0.33	2.17	2.91
Q9CQC2	Colipase OS=Mus musculus GN=Clps PE=2 SV=1 - [COL_MOUSE]	25.66	2	2	2	3	1.24	1.13	1.17	1.05
A2ANX3	Protein Atp8b4 OS=Mus musculus GN=Atp8b4 PE=4 SV=1 - [A2ANX3_MOUSE]	3.52	3	2	3	6	-0.34	-3.57	1.46	-1.77
Q9Z1T2	Thrombospondin-4 OS=Mus musculus GN=Thbs4 PE=1 SV=1 - [TSP4_MOUSE]	8.52	1	5	6	15	1.24	1.33	1.31	1.13
Q8BYR1-2	Isoform 2 of Leucine carboxyl methyltransferase 2 OS=Mus musculus GN=Lcmt2 - [LCMT2_MOUSE]	2.27	2	1	1	1	-0.11	-0.07	0.28	0.33
Q8VCW2	Keratin, type I cytoskeletal 25 OS=Mus musculus GN=Krt25 PE=1 SV=1 [K1C25_MOUSE]	13.68	2	2	3	7	0.59	0.20	-0.07	-0.47
Q8R071	Inositol-trisphosphate 3-kinase A OS=Mus musculus GN=Itpka PE=2 SV=1 - [IP3KA_MOUSE]	4.58	1	1	1	1	0.96	1.32	0.74	1.10
Q9D8U3	Endoplasmic reticulum resident protein 27 OS=Mus musculus GN=Erp27 PE=2 SV=1 [ERP27_MOUSE]	10.66	1	2	2	6	2.38	2.28	2.32	2.15
Q8BGN8-2	Isoform 2 of Synaptoporin OS=Mus musculus GN=Synpr - [SYNPR_MOUSE]	4.91	1	1	1	7	0.59	0.13	0.57	0.33

A3KGF9	Uncharacterized protein C15orf52 homolog OS=Mus musculus PE=1 SV=1 - [CO052_MOUSE]	8.07	1	3	3	7	1.01	0.91	1.00	0.96
Q9WUG6	Insulin-like peptide INSL5 OS=Mus musculus GN=Insl5 PE=2 SV=1 - [INSL5_MOUSE]	24.44	2	4	4	17	1.08	0.58	1.22	0.87
Q91Y74	CMP-N-acetylneuraminate-beta-galactosamide-alpha-2,3-sialyltransferase 4 OS=Mus musculus GN=St3gal4 PE=2 SV=1 - [SIA4C_MOUSE]	12.61	1	4	4	11	1.07	1.00	1.21	1.14
P01635	Ig kappa chain V-V region K2 (Fragment) OS=Mus musculus PE=1 SV=1 - [KV5A3_MOUSE]	27.83	1	3	3	16	0.31	0.58	0.34	0.49
P70458	Plasma serine protease inhibitor OS=Mus musculus GN=Serpina5 PE=1 SV=2 - [IPSP_MOUSE]	7.41	1	2	2	3	0.97	1.34	0.62	0.99
Q8K1S3-2	Isoform 2 of Netrin receptor UNC5B OS=Mus musculus GN=Unc5b - [UNC5B_MOUSE]	1.28	2	1	1	3	0.43	0.62	0.37	0.60
Q80XP8	Protein FAM76B OS=Mus musculus GN=Fam76b PE=2 SV=1 - [FA76B_MOUSE]	14.45	2	2	2	4	0.31	0.86	0.09	0.64
P01644	Ig kappa chain V-V region HP R16.7 OS=Mus musculus PE=1 SV=1 - [KV5AB_MOUSE]	36.11	5	2	2	28	1.01	1.09	0.63	0.82
P05208	Chymotrypsin-like elastase family member 2A OS=Mus musculus GN=Cela2a PE=2 SV=1 - [CEL2A_MOUSE]	3.32	1	1	1	7	1.69	0.50	1.70	0.40
P70351	Histone-lysine N-methyltransferase EZH1 OS=Mus musculus GN=Ezh1 PE=1 SV=1 - [EZH1_MOUSE]	4.42	2	2	2	4	0.17	0.20	0.20	0.21
Q922S4	cGMP-dependent 3',5'-cyclic phosphodiesterase OS=Mus musculus GN=Pde2a PE=1 SV=3 [PDE2A_MOUSE]	13.97	3	9	10	22	0.35	0.68	0.51	0.81
Q91X79	Chymotrypsin-like elastase family member 1 OS=Mus musculus GN=Cela1 PE=2 SV=1 [CELA1_MOUSE]	26.69	1	5	5	43	1.52	0.93	1.58	1.11
Q9CR35	Chymotrypsinogen B OS=Mus musculus GN=Ctrb1 PE=2 SV=1 [CTRB1_MOUSE]	36.12	1	8	8	48	2.69	2.69	2.48	2.51
P30933	Seminal vesicle secretory protein 5 OS=Mus musculus GN=Svs5 PE=2 SV=1 - [SVS5_MOUSE]	22.95	2	2	2	3	3.05	0.96	2.92	1.40

Q7TSG0	Protein 1700065D16Rik OS=Mus musculus GN=1700065D16Rik PE=2 SV=1 - [Q7TSG0_MOUSE]	20.10	1	1	1	1	1.02	-0.35	0.98	-0.39
F8VQ54	Endoribonuclease Dicer OS=Mus musculus GN=Dicer1 PE=2 SV=1 - [F8VQ54_MOUSE]	3.57	2	3	3	6	0.31	0.22	0.24	0.14
Q8BYI9-2	Isoform 2 of Tenascin-R OS=Mus musculus GN=Tnr - [TENR_MOUSE]	4.73	2	2	2	2	0.01	0.37	1.37	1.73
Q8R2Q8	Bone marrow stromal antigen 2 OS=Mus musculus GN=Bst2 PE=1 SV=1 - [BST2_MOUSE]	11.63	1	3	3	8	0.50	0.48	0.68	0.46
Q6P1E8-3	Isoform 2 of EF-hand calcium-binding domain-containing protein 6 OS=Mus musculus GN=Efcab6 - [EFCB6_MOUSE]	2.42	3	2	2	3	-1.29	-2.78	-1.10	-2.59
Q9DB16	Calcium-binding protein 39-like OS=Mus musculus GN=Cab39l PE=1 SV=3 - [CB39L_MOUSE]	13.65	2	3	5	29	0.59	1.01	0.53	0.95
O88312	Anterior gradient protein 2 homolog OS=Mus musculus GN=Agr2 PE=2 SV=1 - [AGR2_MOUSE]	65.14	2	12	12	722	0.17	0.29	0.18	0.26
Q0VEK2	MCG3056, isoform CRA_b OS=Mus musculus GN=Chp2 PE=2 SV=1 - [Q0VEK2_MOUSE]	5.10	2	1	1	3	0.77	0.57	1.01	0.80
M0QW74	MCG9626, isoform CRA_c OS=Mus musculus GN=Mtmr3 PE=4 SV=1 - [M0QW74_MOUSE]	3.16	6	2	2	7	0.71	0.61	0.80	0.70
Q9CPW5	Translocon-associated protein subunit beta OS=Mus musculus GN=Ssr2 PE=1 SV=1 - [SSRB_MOUSE]	4.37	1	1	1	1	1.86	2.22	1.57	1.93
D3Z6F0	Protein Gm14446 OS=Mus musculus GN=Gm14446 PE=4 SV=1 - [D3Z6F0_MOUSE]	8.30	2	2	4	10	0.67	0.70	0.60	0.63
F6ZSM3	Annexin OS=Mus musculus GN=Anxa13 PE=3 SV=2 - [F6ZSM3_MOUSE]	23.03	2	5	5	13	0.53	0.44	0.33	0.15
Q99K74-3	Isoform 3 of Mediator of RNA polymerase II transcription subunit 24 OS=Mus musculus GN=Med24 - [MED24_MOUSE]	3.03	3	1	2	4	0.46	0.27	1.13	0.94
P14142	Solute carrier family 2, facilitated glucose transporter member 4 OS=Mus musculus GN=Slc2a4 PE=1 SV=3 - [GTR4_MOUSE]	7.47	3	2	2	5	0.13	0.01	0.54	0.42
A2A3Z2	Transcriptional regulator Kaiso (Fragment) OS=Mus musculus GN=Zbtb33 PE=2 SV=1 - [A2A3Z2_MOUSE]	5.02	2	2	2	11	0.82	0.65	0.93	0.64

A2AD83	FERM domain-containing protein 7 OS=Mus musculus GN=Frmd7 PE=1 SV=1 - [FRMD7_MOUSE]	0.85	1	1	1	1	1.85	1.99	2.63	2.77
D6RCW3	Cholinephosphotransferase 1 OS=Mus musculus GN=Chpt1 PE=2 SV=1 - [D6RCW3_MOUSE]	5.50	6	1	1	3	0.80	0.31	1.28	0.80
Q70IV5-2	Isoform 2 of Synemin OS=Mus musculus GN=Synm - [SYNEM_MOUSE]	52.74	2	1	56	569	0.67	0.77	0.72	0.73
P50608	Fibromodulin OS=Mus musculus GN=Fmod PE=2 SV=1 - [FMOD_MOUSE]	2.93	1	1	1	2	0.90	0.78	0.99	0.87
Q60604	Adseverin OS=Mus musculus GN=Scin PE=1 SV=3 - [ADSV_MOUSE]	49.51	2	30	30	334	0.76	0.80	0.81	0.83
P97805	Protein FAM3D OS=Mus musculus GN=Fam3d PE=2 SV=2 - [FAM3D_MOUSE]	47.09	1	8	9	111	0.65	0.59	0.64	0.65
Q8CD92-2	Isoform 2 of Tetratricopeptide repeat protein 27 OS=Mus musculus GN=Ttc27 - [TTC27_MOUSE]	5.79	2	4	4	5	0.43	0.39	0.86	0.83
P01631	Ig kappa chain V-II region 26-10 OS=Mus musculus PE=1 SV=1 - [KV2A7_MOUSE]	17.70	2	2	2	5	0.20	0.33	0.20	0.50
D3Z6P0-2	Isoform 2 of Protein disulfide-isomerase A2 OS=Mus musculus GN=Pdia2 - [PDIA2_MOUSE]	17.37	3	9	9	16	1.95	1.81	2.05	1.96
E9Q7Q0	Mucin-4 OS=Mus musculus GN=Muc4 PE=2 SV=1 - [E9Q7Q0_MOUSE]	3.78	3	5	5	6	0.69	1.10	0.14	0.94
P03893	NADH-ubiquinone oxidoreductase chain 2 OS=Mus musculus GN=Mtnd2 PE=1 SV=1 - [NU2M_MOUSE]	4.93	2	1	1	5	0.40	0.62	-1.21	-0.26
B1AR69	Protein Myh13 OS=Mus musculus GN=Myh13 PE=4 SV=1 - [B1AR69_MOUSE]	4.44	2	3	8	101	0.32	-0.02	0.59	0.33
Q71M36-3	Isoform 3 of Chondroitin sulfate proteoglycan 5 OS=Mus musculus GN=Cspg5 - [CSPG5_MOUSE]	7.42	4	1	1	4	1.21	1.92	1.30	2.01
A2AFS3	UPF0577 protein KIAA1324 OS=Mus musculus GN=Kiaa1324 PE=2 SV=1 - [K1324_MOUSE]	8.03	3	7	7	25	0.64	0.67	0.56	0.61
E0CY52	Uncharacterized protein OS=Mus musculus GN=Slc25a54 PE=2 SV=1 - [E0CY52_MOUSE]	2.35	2	1	1	1	1.23	1.08	1.87	1.72
Q7TME2	Sperm-associated antigen 5 OS=Mus musculus GN=Spag5 PE=1 SV=1 - [SPAG5_MOUSE]	0.77	1	1	1	16	0.35	0.18	0.16	0.08

F6T2U3	Protein Gm9925 OS=Mus musculus GN=Gm9925 PE=4 SV=1 - [F6T2U3_MOUSE]	10.00	1	1	1	1	0.61	0.01	0.70	0.10
Q9ER05	Chymopasin OS=Mus musculus GN=Ctrl PE=2 SV=1 - [Q9ER05_MOUSE]	22.73	1	4	4	14	1.56	1.25	1.47	1.50
Q9Z2E3	Serine/threonine-protein kinase/endoribonuclease IRE2 OS=Mus musculus GN=Ern2 PE=2 SV=2 - [ERN2_MOUSE]	5.60	1	4	4	10	0.36	1.11	0.41	0.89
Q80V62	Fanconi anemia group D2 protein homolog OS=Mus musculus GN=Fancd2 PE=1 SV=2 - [FACD2_MOUSE]	2.41	3	3	3	11	0.79	0.84	0.79	1.06
J3QK03	Uncharacterized protein OS=Mus musculus GN=Ighv6-5 PE=4 SV=1 - [J3QK03_MOUSE]	16.16	8	1	1	4	0.49	0.45	0.49	0.45
P47856-2	Isoform 2 of Glutamine-fructose-6-phosphate aminotransferase [isomerizing] 1 OS=Mus musculus GN=Gfpt1 - [GFPT1_MOUSE]	57.71	4	27	33	734	0.57	0.61	0.54	0.63
Q91W27	Tuberoinfundibular peptide of 39 residues OS=Mus musculus GN=Pth2 PE=1 SV=1 - [TIP39_MOUSE]	15.00	1	2	2	4	0.46	0.07	0.70	0.31
E0CXB6	Sorting nexin-31 (Fragment) OS=Mus musculus GN=Snx31 PE=2 SV=1 - [E0CXB6_MOUSE]	17.16	3	1	1	1	-0.35	0.08	-0.37	0.07
G5E817	BTB (POZ) domain containing 6, isoform CRA_b OS=Mus musculus GN=Btbd6 PE=4 SV=1 - [G5E817_MOUSE]	10.02	2	2	2	5	-0.17	0.34	0.16	0.94
Q9CRB0	Sorting nexin-24 OS=Mus musculus GN=Snx24 PE=2 SV=1 - [SNX24_MOUSE]	5.33	1	1	1	1	0.62	0.40	0.62	0.40
F6R7Y0	CMP-sialic acid transporter (Fragment) OS=Mus musculus GN=Slc35a1 PE=4 SV=1 - [F6R7Y0_MOUSE]	8.40	2	2	2	14	0.44	0.49	0.50	0.58
Q8K0J2	UDP-GlcNAc:betaGal beta-1,3-N-acetylglucosaminyltransferase 7 OS=Mus musculus GN=B3gnt7 PE=2 SV=2 - [B3GN7_MOUSE]	12.59	1	5	5	14	0.98	0.78	0.94	0.69
Q9WTS2	Alpha-(1,6)-fucosyltransferase OS=Mus musculus GN=Fut8 PE=2 SV=2 - [FUT8_MOUSE]	15.13	1	8	8	17	0.28	0.47	0.22	0.48
P01674	Ig kappa chain V-III region PC 2154 OS=Mus musculus PE=1 SV=1 - [KV3AM_MOUSE]	11.11	1	1	1	2	1.75	1.02	1.90	1.16
O08785-2	Isoform Short of Circadian locomoter output cycles protein kaput OS=Mus musculus GN=Clock - [CLOCK_MOUSE]	4.12	2	2	2	13	0.20	-0.62	1.05	1.14

Q8BW66	Solute carrier family 25 member 48 OS=Mus musculus GN=Slc25a48 PE=2 SV=2 - [S2548_MOUSE]	8.17	1	1	1	4	0.71	0.50	0.89	0.67
P01878	Ig alpha chain C region OS=Mus musculus PE=1 SV=1 - [IGHA_MOUSE]	50.00	1	13	13	180	0.59	0.76	0.57	0.74
E9Q4Y1	Protein Adh6a OS=Mus musculus GN=Adh6a PE=2 SV=1 - [E9Q4Y1_MOUSE]	5.43	2	1	1	1	-0.13	-0.85	1.72	1.00
P01636	Ig kappa chain V-V region MOPC 149 OS=Mus musculus PE=1 SV=1 - [KV5A4_MOUSE]	12.04	1	1	1	3	-0.01	0.04	0.36	0.42
Q5U4C1	G-protein coupled receptor-associated sorting protein 1 OS=Mus musculus GN=Gprasp1 PE=2 SV=1 - [GASP1_MOUSE]	1.11	1	1	1	1	-0.42	0.22	-0.39	0.25
Q3UW64	Bifunctional UDP-N-acetylglucosamine 2-epimerase/N-acetylmannosamine kinase OS=Mus musculus GN=Gne PE=2 SV=1 - [Q3UW64_MOUSE]	34.13	9	22	22	187	0.69	0.65	0.71	0.65
G3X8Q1	Calcineurin binding protein 1, isoform CRA_a OS=Mus musculus GN=Cabin1 PE=4 SV=1 - [G3X8Q1_MOUSE]	3.06	1	3	3	6	0.06	-0.16	0.25	0.03
P12979	Myogenin OS=Mus musculus GN=Myog PE=2 SV=2 - [MYOG_MOUSE]	5.80	1	1	1	1	-0.49	-0.44	1.56	1.61
P97820	Mitogen-activated protein kinase kinase kinase kinase 4 OS=Mus musculus GN=Map4k4 PE=1 SV=1 - [M4K4_MOUSE]	3.89	3	2	2	2	-0.42	-0.28	-0.06	0.08
Q5JCS9	UDP-GlcNAc:betaGal beta-1,3-N-acetylglucosaminyltransferase 3 OS=Mus musculus GN=B3gnt3 PE=2 SV=1 - [B3GN3_MOUSE]	12.10	1	5	5	30	0.89	0.62	0.93	0.64
Q91VN0	Low-density lipoprotein receptor-related protein 5 OS=Mus musculus GN=Lrp5 PE=1 SV=3 - [LRP5_MOUSE]	1.05	1	2	2	3	1.10	0.92	0.97	0.79
G3UW36	MCG117244, isoform CRA_a OS=Mus musculus GN=4932443I19Rik PE=4 SV=1 - [G3UW36_MOUSE]	14.29	1	1	1	1	0.47	0.68	0.65	0.85
Q80WQ8-3	Isoform 3 of Mis18-binding protein 1 OS=Mus musculus GN=Mis18bp1 - [M18BP_MOUSE]	5.09	3	1	2	3	0.69	0.36	0.65	0.31
B1ATS4	Sarcoplasmic/endoplasmic reticulum calcium ATPase 3 OS=Mus musculus GN=Atp2a3 PE=2 SV=1 - [B1ATS4_MOUSE]	31.91	7	23	30	205	0.59	0.60	0.56	0.48

Q80V26	Inositol monophosphatase 3 OS=Mus musculus GN=Impad1 PE=1 SV=1 - [IMPA3_MOUSE]	10.67	1	4	4	9	0.42	0.37	0.61	0.48
Q6DI86-3	Isoform 3 of FAST kinase domain-containing protein 1 OS=Mus musculus GN=Fastkd1 - [FAKD1_MOUSE]	3.38	3	2	2	4	-0.05	-1.07	0.39	-0.64
Q8BHP7	Olfactomedin-like protein 2A OS=Mus musculus GN=Olfml2a PE=1 SV=1 - [OLM2A_MOUSE]	1.91	1	1	1	5	1.42	0.43	1.34	0.35
Q8K330	Protein phosphatase Slingshot homolog 3 OS=Mus musculus GN=Ssh3 PE=1 SV=1 - [SSH3_MOUSE]	7.24	2	4	4	15	0.18	0.17	0.16	0.58
O70138	Neutrophil collagenase OS=Mus musculus GN=Mmp8 PE=2 SV=2 - [MMP8_MOUSE]	3.01	1	1	1	1	-0.01	0.52	0.89	1.41
D3YXZ7	UDP-N-acetylglucosamine transporter (Fragment) OS=Mus musculus GN=Slc35a3 PE=2 SV=1 - [D3YXZ7_MOUSE]	7.08	2	1	1	8	0.17	0.50	0.06	0.39
Q922Q5	Adenosine 3'-phospho 5'-phosphosulfate transporter 2 OS=Mus musculus GN=Slc35b3 PE=2 SV=1 - [S35B3_MOUSE]	1.63	2	1	1	1	0.53	0.83	0.40	0.70
B9EJX2	Nance-Horan syndrome (Human) OS=Mus musculus GN=Nhs PE=2 SV=1 - [B9EJX2_MOUSE]	1.54	2	2	2	8	0.71	0.64	0.70	0.64
Q8K221	Arfaptin-2 OS=Mus musculus GN=Arfip2 PE=2 SV=2 - [ARFP2_MOUSE]	21.11	3	6	6	13	0.41	0.28	0.31	0.33
Q5F226	Protocadherin Fat 2 OS=Mus musculus GN=Fat2 PE=2 SV=1 - [FAT2_MOUSE]	1.17	1	2	3	6	0.44	0.07	1.12	0.75
Q05BQ1	Protein turtle homolog A OS=Mus musculus GN=Igsf9 PE=1 SV=2 - [TUTLA_MOUSE]	1.44	1	1	1	1	-0.11	0.01	0.26	0.38
Q3TL54	Tripartite motif-containing protein 43A OS=Mus musculus GN=Trim43a PE=2 SV=1 - [TR43A_MOUSE]	2.25	1	1	1	2	-0.05	-0.22	0.13	-0.04
D3YXA1	Lebercilin (Fragment) OS=Mus musculus GN=Lca5 PE=2 SV=1 - [D3YXA1_MOUSE]	11.36	5	1	2	2	0.67	0.52	1.00	0.86
D3YVR7	L-lactate dehydrogenase (Fragment) OS=Mus musculus GN=Ldhc PE=2 SV=1 - [D3YVR7_MOUSE]	11.49	3	1	2	68	0.16	0.12	0.06	0.17
Q9WU22	Tyrosine-protein phosphatase non-receptor type 4 OS=Mus musculus GN=Ptpn4 PE=1 SV=1 [PTN4_MOUSE]	4.64	3	2	2	2	0.36	0.39	0.65	0.68

B2RT41	Protein Zfc3h1 OS=Mus musculus GN=Zfc3h1 PE=2 SV=1 - [B2RT41_MOUSE]	1.20	1	2	2	2	-1.69	-1.48	-1.07	-0.86
Q3UTQ8	Cyclin-dependent kinase-like 5 OS=Mus musculus GN=Cdkl5 PE=2 SV=1 - [CDKL5_MOUSE]	1.28	1	1	1	2	0.13	0.67	0.38	0.91
Q921L5	Conserved oligomeric Golgi complex subunit 2 OS=Mus musculus GN=Cog2 PE=2 SV=2 - [COG2_MOUSE]	23.39	3	14	15	36	0.57	0.49	0.64	0.55
Q6GQS1	Calcium-binding mitochondrial carrier protein SCaMC-3 OS=Mus musculus GN=Slc25a23 PE=2 SV=1 - [SCMC3_MOUSE]	1.71	1	1	1	1	-1.87	-1.59	-0.07	0.21
Q9CR20	Immediate early response 3-interacting protein 1 OS=Mus musculus GN=Ier3ip1 PE=2 SV=1 - [IR3IP_MOUSE]	24.39	2	1	1	10	0.39	0.54	0.75	0.91
Q11127-2	Isoform Short of Alpha-(1,3)-fucosyltransferase OS=Mus musculus GN=Fut4 - [FUT4_MOUSE]	11.00	2	3	3	8	0.30	0.28	0.49	0.33
Q8C407	Protein YIPF4 OS=Mus musculus GN=Yipf4 PE=2 SV=1 - [YIPF4_MOUSE]	3.66	2	1	1	2	0.51	0.24	0.63	0.36
Q80Y83-8	Isoform 8 of Dixin OS=Mus musculus GN=Dixdc1 - [DIXC1_MOUSE]	3.06	8	1	1	1	-0.56	-2.66	1.66	-0.45
Q3V1D7	BPI fold-containing family C protein OS=Mus musculus GN=Bpifc PE=2 SV=1 - [Q3V1D7_MOUSE]	1.78	2	1	1	1	-0.20	-0.23	0.16	0.14
Q9CZV8-4	Isoform 2 of F-box/LRR-repeat protein 20 OS=Mus musculus GN=Fbxl20 - [FXL20_MOUSE]	3.32	5	1	2	5	-0.56	-0.37	-0.29	-0.09
Q91VT8	Small integral membrane protein 14 OS=Mus musculus GN=Smim14 PE=2 SV=1 - [SIM14_MOUSE]	29.29	3	1	2	5	0.01	0.37	-0.17	0.18
Q3USW5	FAD-dependent oxidoreductase domain-containing protein 2 OS=Mus musculus GN=Foxred2 PE=2 SV=1 - [FXRD2_MOUSE]	3.16	1	1	1	1	0.96	1.28	0.74	1.06
Q5M6W3	Clathrin heavy chain linker domain-containing protein 1 OS=Mus musculus GN=Clhc1 PE=2 SV=1 [CLHC1_MOUSE]	3.52	1	2	2	3	-1.27	-0.98	0.63	0.92
D3YUR8	Protein 0610007P14Rik (Fragment) OS=Mus musculus GN=0610007P14Rik PE=2 SV=1 - [D3YUR8_MOUSE]	27.78	2	1	1	3	0.58	0.78	0.65	0.86

G3UZE6	Protein Obox5 (Fragment) OS=Mus musculus GN=Obox5 PE=2 SV=1 - [G3UZE6_MOUSE]	6.18	3	1	1	1	0.02	0.12	-0.06	0.04
Q91WA3	Histone deacetylase 11 OS=Mus musculus GN=Hdac11 PE=2 SV=1 - [HDA11_MOUSE]	6.63	1	1	1	3	0.24	0.50	0.26	0.53
O35682	Myeloid-associated differentiation marker OS=Mus musculus GN=Myadm PE=2 SV=2 - [MYADM_MOUSE]	7.81	1	2	2	23	-0.17	-0.45	-0.04	-0.24
E0CXV3	Alcohol dehydrogenase 1 OS=Mus musculus GN=Adh1 PE=2 SV=1 - [E0CXV3_MOUSE]	43.18	1	1	1	3	0.55	0.79	0.51	0.75
Q78KK3	Solute carrier family 22 member 18 OS=Mus musculus GN=Slc22a18 PE=2 SV=2 - [S22AI_MOUSE]	3.20	1	1	1	5	0.05	0.39	0.85	1.56
O54818	Tumor protein D53 OS=Mus musculus GN=Tpd52l1 PE=2 SV=1 - [TPD53_MOUSE]	13.24	1	2	2	2	-0.25	-1.43	-0.21	-1.38
Q9ER71-2	Isoform 2 of Rho-related GTP-binding protein RhoJ OS=Mus musculus GN=Rhoj - [RHOJ_MOUSE]	12.25	7	1	2	18	0.82	0.90	0.72	0.81
P69744	Transient receptor potential cation channel subfamily V member 5 OS=Mus musculus GN=Trpv5 PE=1 SV=2 [TRPV5_MOUSE]	1.80	1	1	1	1	0.62	1.28	0.39	1.06
Q9Z0D9	CX3C chemokine receptor 1 OS=Mus musculus GN=Cx3cr1 PE=2 SV=1 - [CX3C1_MOUSE]	7.63	1	1	1	4	0.30	0.38	0.86	0.94
F6X9Z6	4-hydroxyphenylpyruvate dioxygenase (Fragment) OS=Mus musculus GN=Hpd PE=4 SV=1 - [F6X9Z6_MOUSE]	9.38	2	1	1	2	-0.05	-0.52	0.00	-0.48
Q5BKQ4	Inactive pancreatic lipase-related protein 1 OS=Mus musculus GN=Pnliprp1 PE=2 SV=2 - [LIPR1_MOUSE]	34.46	1	10	10	28	2.35	2.01	2.22	1.94
E9QPE7	Myosin-11 OS=Mus musculus GN=Myh11 PE=4 SV=1 - [E9QPE7_MOUSE]	64.81	2	4	183	12039	0.32	0.25	0.45	0.42
Q9JHH9	Coatomer subunit zeta-2 OS=Mus musculus GN=Copz2 PE=2 SV=1 - [COPZ2_MOUSE]	19.02	2	3	3	19	0.31	-0.03	0.31	0.13
Q69ZA1-2	Isoform 2 of Cyclin-dependent kinase 13 OS=Mus musculus GN=Cdk13 - [CDK13_MOUSE]	4.34	32	4	5	26	-0.38	0.04	-0.06	0.36
Q8BGT9	Polypeptide N-acetylgalactosaminyltransferase 12 OS=Mus musculus GN=Galnt12 PE=2 SV=1 - [GLT12_MOUSE]	15.63	2	7	8	24	0.31	0.36	0.34	0.38

O35382	Exocyst complex component 4 OS=Mus musculus GN=Exoc4 PE=1 SV=2 - [EXOC4_MOUSE]	8.92	3	7	7	14	0.41	-0.20	0.38	-0.23
Q8R2S8	CD177 antigen OS=Mus musculus GN=Cd177 PE=2 SV=1 - [CD177_MOUSE]	14.44	1	10	10	37	1.30	1.11	1.25	1.31
Q9JMG2	C1GALT1-specific chaperone 1 OS=Mus musculus GN=C1galt1c1 PE=2 SV=1 - [C1GLC_MOUSE]	15.19	1	4	4	7	0.33	0.49	0.29	0.38
Q80Y44	Probable ATP-dependent RNA helicase DDX10 OS=Mus musculus GN=Ddx10 PE=1 SV=2 - [DDX10_MOUSE]	4.34	1	3	3	4	0.17	-0.26	0.34	-0.09
Q0KL01	UBX domain-containing protein 2B OS=Mus musculus GN=Ubxn2b PE=1 SV=2 - [UBX2B_MOUSE]	5.14	1	2	2	4	-0.06	-2.65	0.64	-1.95
E9QAN4	Kinesin-like protein KIF1A OS=Mus musculus GN=Kif1a PE=2 SV=1 - [E9QAN4_MOUSE]	3.55	6	1	6	21	0.55	0.84	0.21	0.49
F6YRQ2	DnaJ homolog subfamily B member 6 (Fragment) OS=Mus musculus GN=Dnajb6 PE=4 SV=1 - [F6YRQ2_MOUSE]	26.41	1	1	4	5	0.81	0.64	0.74	0.57
Q14DK4-2	Isoform 2 of Glycerol-3-phosphate acyltransferase 2, mitochondrial OS=Mus musculus GN=Gpat2 - [GPAT2_MOUSE]	1.88	3	1	1	6	0.77	0.92	1.34	1.49
P54265-10	Isoform 10 of Myotonin protein kinase OS=Mus musculus GN=Dmpk - [DMPK_MOUSE]	19.75	12	7	10	26	0.08	-0.07	-0.01	-0.08
E9QL72	Uncharacterized aarF domain-containing protein kinase 2 OS=Mus musculus GN=Adck2 PE=2 SV=1 - [E9QL72_MOUSE]	3.52	2	1	1	1	-0.11	-0.79	2.76	2.09
P50446	Keratin, type II cytoskeletal 6A OS=Mus musculus GN=Krt6a PE=2 SV=3 [K2C6A_MOUSE]	13.92	4	2	9	229	0.82	0.38	0.84	0.40
Q8R1U2	Cell growth regulator with EF hand domain protein 1 OS=Mus musculus GN=Cgref1 PE=2 SV=1 - [CGRE1_MOUSE]	10.32	2	2	2	6	-0.17	-0.02	0.15	0.30
F7A6H4	E3 ubiquitin-protein ligase RNF213 OS=Mus musculus GN=Rnf213 PE=4 SV=2 - [F7A6H4_MOUSE]	7.94	2	35	37	82	0.53	0.51	0.64	0.55
D3YY41	Phosphatidylinositol 3-kinase, catalytic, alpha polypeptide, isoform CRA_a OS=Mus musculus GN=Pik3ca PE=4 SV=1 - [D3YY41_MOUSE]	3.17	2	3	3	8	0.58	0.66	0.57	0.64

F7AC41	Protein Pus7 (Fragment) OS=Mus musculus GN=Pus7 PE=4 SV=1 - [F7AC41_MOUSE]	10.19	4	4	4	5	0.18	0.10	0.44	0.01
Q9CT10	Ran-binding protein 3 OS=Mus musculus GN=Ranbp3 PE=1 SV=2 - [RANB3_MOUSE]	11.41	1	6	6	18	0.35	0.58	0.48	0.52
Q99PL5-10	Isoform RRp2 of Ribosome-binding protein 1 OS=Mus musculus GN=Rrbp1 - [RRBP1_MOUSE]	50.00	4	1	10	133	-0.48	-0.28	-0.15	0.05
Q9CQ25	Mitotic-spindle organizing protein 2 OS=Mus musculus GN=Mzt2 PE=1 SV=1 - [MZT2_MOUSE]	8.81	1	1	1	1	1.13	0.38	1.62	0.87
Q7M6Y6	Maestro heat-like repeat containing protein family member 2B OS=Mus musculus GN=Mroh2b PE=1 SV=2 - [MRO2B_MOUSE]	2.40	1	2	2	2	0.70	0.63	0.64	0.57
P00683	Ribonuclease pancreatic OS=Mus musculus GN=Rnase1 PE=1 SV=2 - [RNAS1_MOUSE]	32.21	2	3	3	10	0.54	1.12	0.70	1.06
D6RI62	Putative Polycomb group protein ASXL2 (Fragment) OS=Mus musculus GN=Asxl2 PE=2 SV=1 - [D6RI62_MOUSE]	2.23	5	1	1	1	-0.25	-0.51	1.74	1.48
P61014	Cardiac phospholamban OS=Mus musculus GN=Pln PE=1 SV=1 - [PPLA_MOUSE]	32.69	1	2	2	6	0.03	0.26	0.09	0.25
Q8VCZ6	Small G protein signaling modulator 3 OS=Mus musculus GN=Sgsm3 PE=1 SV=1 - [SGSM3_MOUSE]	11.73	4	7	7	20	0.14	0.16	0.26	0.19
P70186	Epiphycan OS=Mus musculus GN=Epyc PE=2 SV=1 - [EPYC_MOUSE]	5.28	1	1	1	1	0.53	0.94	2.12	2.52
P01723	Ig lambda-1 chain V region OS=Mus musculus PE=1 SV=2 - [LV1A_MOUSE]	35.90	5	2	2	7	1.44	1.34	1.37	1.27
B2RQY6	BC035947 protein OS=Mus musculus GN=BC035947 PE=2 SV=1 - [B2RQY6_MOUSE]	3.17	1	1	3	15	0.73	0.58	0.85	0.69
A1BN54	Alpha actinin 1a OS=Mus musculus GN=Actn1 PE=2 SV=1 [A1BN54_MOUSE]	61.67	1	2	51	1947	0.55	0.58	0.55	0.55
Q3TML0	Protein disulfide-isomerase A6 OS=Mus musculus GN=Pdia6 PE=2 SV=1 - [Q3TML0_MOUSE]	41.12	2	17	17	246	0.74	0.70	0.66	0.73
Q08091	Calponin-1 OS=Mus musculus GN=Cnn1 PE=2 SV=1 - [CNN1_MOUSE]	79.46	2	21	24	1506	0.92	0.94	0.85	0.90

P01902	H-2 class I histocompatibility antigen, K-D alpha chain OS=Mus musculus GN=H2-K1 PE=1 SV=1 - [HA1D_MOUSE]	11.14	1	1	3	19	-0.94	-1.20	0.19	-0.07
Q3UVL4	Vacuolar protein sorting associated protein 51 homolog OS=Mus musculus GN=Vps51 PE=2 SV=2 - [VPS51_MOUSE]	11.38	2	5	6	13	0.35	0.25	0.41	0.15
Q3UW68	Calpain-13 OS=Mus musculus GN=Capn13 PE=2 SV=1 - [CAN13_MOUSE]	12.63	1	7	7	19	1.20	1.18	1.25	0.90
P00329	Alcohol dehydrogenase 1 OS=Mus musculus GN=Adh1 PE=2 SV=2 - [ADH1_MOUSE]	52.53	2	14	14	263	0.54	0.59	0.57	0.52
Q8C078	Calcium/calmodulin-dependent protein kinase kinase 2 OS=Mus musculus GN=Camkk2 PE=1 SV=2 - [KKCC2_MOUSE]	2.21	1	1	1	1	0.84	1.10	0.84	1.10
Q8K4S1	1-phosphatidylinositol 4,5-bisphosphate phosphodiesterase epsilon-1 OS=Mus musculus GN=Plce1 PE=1 SV=3 - [PLCE1_MOUSE]	1.40	1	2	2	10	0.57	0.58	0.74	0.65
A2AMD2	Protein Ankrd60 OS=Mus musculus GN=Ankrd60 PE=2 SV=2 - [A2AMD2_MOUSE]	4.72	1	1	1	1	-1.10	-0.35	-1.17	-0.43
Q8K273	Membrane magnesium transporter 1 OS=Mus musculus GN=Mmgt1 PE=2 SV=1 - [MMGT1_MOUSE]	18.32	1	1	1	9	0.41	0.25	0.25	0.10
Q810B7-2	Isoform 2 of SLIT and NTRK-like protein 5 OS=Mus musculus GN=Slitrk5 - [SLIK5_MOUSE]	3.58	2	3	3	3	0.59	0.75	0.43	-0.01
D3Z667	Dynein heavy chain 2, axonemal OS=Mus musculus GN=Dnah2 PE=2 SV=1 - [D3Z667_MOUSE]	1.32	2	5	5	14	0.19	-0.63	0.87	0.03
D3Z080	Sorbin and SH3 domain containing protein 2 OS=Mus musculus GN=Sorbs2 PE=2 SV=1 - [D3Z080_MOUSE]	49.93	12	1	29	214	0.67	0.60	0.51	0.44
O08638	Myosin-11 OS=Mus musculus GN=Myh11 PE=1 SV=1 - [MYH11_MOUSE]	62.42	2	5	181	11645	0.26	0.22	0.34	0.17
B2RUF0	Y box protein 2 OS=Mus musculus GN=Ybx2 PE=2 SV=1 - [B2RUF0_MOUSE]	27.86	2	2	7	42	-1.15	-1.27	-0.47	-0.88
Q8K3G9	DCC-interacting protein 13-beta OS=Mus musculus GN=Appl2 PE=2 SV=1 - [DP13B_MOUSE]	15.41	3	5	8	27	0.67	0.59	0.62	0.56
Q9R0N9	Synaptotagmin-9 OS=Mus musculus GN=Syt9 PE=1 SV=2 - [SYT9_MOUSE]	6.92	1	2	2	27	0.26	-0.01	0.10	-0.05

Q8BKA3	Serine/arginine repetitive matrix protein 4 OS=Mus musculus GN=Srrm4 PE=1 SV=1 - [SRRM4_MOUSE]	2.80	1	1	1	1	0.67	1.16	0.43	0.92
Q80UQ2	Ras association domain-containing protein 6 OS=Mus musculus GN=Rassf6 PE=1 SV=1 - [RASF6_MOUSE]	11.61	3	2	2	40	0.47	0.68	0.21	0.60
E9QPE8	Plectin OS=Mus musculus GN=Plec PE=2 SV=1 - [E9QPE8_MOUSE]	47.59	28	1	204	1474	0.59	0.18	1.08	0.67
A2AHJ4	Bromodomain and WD repeat-containing protein 3 OS=Mus musculus GN=Brwd3 PE=2 SV=1 - [BRWD3_MOUSE]	1.83	7	2	3	10	0.35	0.17	0.05	-0.10
Q6ZQH8	Nucleoporin NUP188 homolog OS=Mus musculus GN=Nup188 PE=1 SV=2 - [NU188_MOUSE]	5.00	5	5	5	45	0.55	0.62	0.57	0.67
Q3UKX1	Protein arginine N-methyltransferase 2 OS=Mus musculus GN=Prmt2 PE=2 SV=1 [Q3UKX1_MOUSE]	3.37	3	1	1	1	0.21	0.10	0.37	0.26
P51162	Gastrotropin OS=Mus musculus GN=Fabp6 PE=2 SV=2 - [FABP6_MOUSE]	32.81	1	3	3	4	-1.27	-1.43	-0.21	-0.37
D3YXN9	MCG59255 OS=Mus musculus GN=5830403L16Rik PE=4 SV=1 - [D3YXN9_MOUSE]	13.04	2	1	1	1	2.24	2.53	2.29	2.57
F6RJC1	Teneurin-3 (Fragment) OS=Mus musculus GN=Tenm3 PE=4 SV=1 - [F6RJC1_MOUSE]	1.49	4	2	2	6	0.71	0.68	0.60	0.56
Q3UHH1-5	Isoform 5 of Zinc finger SWIM domain-containing protein 8 OS=Mus musculus GN=Zswim8 - [ZSWM8_MOUSE]	2.62	4	2	2	3	0.40	0.48	0.55	0.62
O88455	7-dehydrocholesterol reductase OS=Mus musculus GN=Dhcr7 PE=2 SV=1 - [DHCR7_MOUSE]	4.46	1	2	2	4	0.29	0.53	0.18	0.53
Q91VX9	Transmembrane protein 168 OS=Mus musculus GN=Tmem168 PE=2 SV=1 - [TM168_MOUSE]	3.44	1	2	2	9	0.61	0.37	0.75	0.52
P58404-2	Isoform 2 of Striatin-4 OS=Mus musculus GN=Strn4 - [STRN4_MOUSE]	14.21	2	3	6	17	1.12	0.73	1.20	0.81
Q8BH24	Transmembrane 9 superfamily member 4 OS=Mus musculus GN=Tm9sf4 PE=2 SV=1 - [TM9S4_MOUSE]	15.55	1	8	8	93	0.39	0.40	0.41	0.37
O88987	A-kinase anchor protein 3 OS=Mus musculus GN=Akap3 PE=2 SV=2 - [AKAP3_MOUSE]	1.50	1	1	1	1	1.03	1.03	1.09	1.09
P41183	B-cell lymphoma 6 protein homolog OS=Mus musculus GN=Bcl6 PE=1 SV=1 - [BCL6_MOUSE]	1.27	1	1	1	2	0.96	0.37	1.20	0.61

D3Z6B9	Mitochondrial 10-formyltetrahydrofolate dehydrogenase OS=Mus musculus GN=Aldh1l2 PE=2 SV=1 - [D3Z6B9_MOUSE]	12.47	2	6	8	22	0.53	0.33	0.64	0.54
P01680	Ig kappa chain V-IV region S107B OS=Mus musculus PE=4 SV=1 - [KV4A1_MOUSE]	8.53	1	1	1	4	0.35	0.11	0.16	-0.08
Q61263	Sterol O-acyltransferase 1 OS=Mus musculus GN=Soat1 PE=2 SV=2 [SOAT1_MOUSE]	4.07	1	2	2	33	0.60	0.67	0.54	0.61
Q3UTJ2-2	Isoform 2 of Sorbin and SH3 domain-containing protein 2 OS=Mus musculus GN=Sorbs2 - [SRBS2_MOUSE]	29.96	13	1	32	210	0.18	-0.20	0.40	0.02
G3UXC2	Disintegrin and metalloproteinase domain-containing protein 32 (Fragment) OS=Mus musculus GN=Adam32 PE=2 SV=1 - [G3UXC2_MOUSE]	15.00	3	1	1	1	-0.22	0.13	0.15	0.50
Q9JJ06	Glycoprotein-N-acetylgalactosamine 3-beta-galactosyltransferase 1 OS=Mus musculus GN=C1galt1 PE=1 SV=1 - [C1GLT_MOUSE]	26.17	1	7	7	44	0.18	0.29	0.25	0.28
Q99PF4-2	Isoform 2 of Cadherin-23 OS=Mus musculus GN=Cdh23 - [CAD23_MOUSE]	2.26	5	4	4	10	1.01	1.07	0.80	0.96
D3YWH4	Transmembrane 9 superfamily member 1 (Fragment) OS=Mus musculus GN=Tm9sf1 PE=2 SV=1 - [D3YWH4_MOUSE]	16.32	6	7	7	28	0.23	0.23	0.29	0.21
Q9CRY7	Glycerophosphodiester phosphodiesterase domain-containing protein 1 OS=Mus musculus GN=Gdpd1 PE=2 SV=1 - [GDPD1_MOUSE]	33.12	1	10	10	30	0.47	0.59	0.37	0.55
Q4VC33	Macrophage erythroblast attacher OS=Mus musculus GN=Maea PE=1 SV=1 - [MAEA_MOUSE]	2.02	2	1	1	1	1.59	0.94	1.41	0.75
P01837	Ig kappa chain C region OS=Mus musculus PE=1 SV=1 - [IGKC_MOUSE]	49.06	1	5	5	162	0.17	0.36	0.17	0.34
Q9CU24	Protein THEMIS3 OS=Mus musculus GN=Themis3 PE=2 SV=1 - [THMS3_MOUSE]	2.46	1	1	1	1	1.04	1.23	0.84	1.02
P01734	T-cell receptor beta chain V region 3H.25 OS=Mus musculus PE=4 SV=1 - [TVB1_MOUSE]	14.07	1	1	1	1	0.70	0.54	0.66	0.50
Q80YS5	Leucine-rich repeat-containing protein 27 OS=Mus musculus GN=Lrrc27 PE=2 SV=1 - [LRC27_MOUSE]	5.54	2	2	2	4	-0.34	0.14	0.28	0.76

F6YIY0	Protein YIPF3 (Fragment) OS=Mus musculus GN=Yipf3 PE=4 SV=1 - [F6YIY0_MOUSE]	7.96	3	2	2	3	0.31	0.39	0.31	0.30
Q8BNW9	Kelch repeat and BTB domain-containing protein 11 OS=Mus musculus GN=Kbtbd11 PE=1 SV=3 - [KBTBB_MOUSE]	5.85	1	3	3	4	-0.29	-0.11	0.79	0.97
Q99KD5	Protein unc-45 homolog A OS=Mus musculus GN=Unc45a PE=1 SV=2 - [UN45A_MOUSE]	20.55	4	16	16	53	0.41	0.19	0.48	0.40
Q9D7T1	Rab15 effector protein OS=Mus musculus GN=Rep15 PE=2 SV=1 - [REP15_MOUSE]	12.17	1	3	3	4	0.12	0.08	0.17	0.13
Q9D8I1	Marginal zone B- and B1-cell-specific protein OS=Mus musculus GN=Mzb1 PE=1 SV=2 [MZB1_MOUSE]	54.79	1	7	7	23	0.51	0.40	0.48	0.71
P22361	Hepatocyte nuclear factor 1-alpha OS=Mus musculus GN=Hnf1a PE=1 SV=2 - [HNF1A_MOUSE]	7.32	2	3	3	3	0.91	0.94	0.84	0.87
Q8K4X7	1-acyl-sn-glycerol-3-phosphate acyltransferase delta OS=Mus musculus GN=Agpat4 PE=2 SV=1 - [PLCD_MOUSE]	14.55	1	6	6	16	0.22	0.28	0.07	0.25
B1AU74	Motile sperm domain-containing protein 2 OS=Mus musculus GN=Mospd2 PE=2 SV=1 - [B1AU74_MOUSE]	2.12	2	1	1	1	0.26	-0.01	0.32	0.04
P18528	Ig heavy chain V region 6.96 OS=Mus musculus PE=4 SV=1 - [HVM57_MOUSE]	30.61	6	2	2	5	0.71	0.55	0.94	0.78
O09105	Neurogenic differentiation factor 4 OS=Mus musculus GN=Neurod4 PE=1 SV=1 - [NDF4_MOUSE]	13.03	1	2	2	6	0.45	0.46	0.39	0.45
P55088-2	Isoform 1 of Aquaporin-4 OS=Mus musculus GN=Aqp4 - [AQP4_MOUSE]	23.26	3	3	3	5	1.38	1.42	1.25	1.29
F8VQF9	Trafficking protein particle complex subunit 10 OS=Mus musculus GN=Trappc10 PE=4 SV=1 - [F8VQF9_MOUSE]	3.50	2	3	3	4	0.64	-0.66	0.68	0.52
Q8R1F6-2	Isoform 2 of Protein HID1 OS=Mus musculus GN=Hid1 - [HID1_MOUSE]	11.94	3	8	8	20	0.31	0.14	0.21	0.17
P55012	Solute carrier family 12 member 2 OS=Mus musculus GN=Slc12a2 PE=1 SV=2 - [S12A2_MOUSE]	20.58	3	16	18	117	0.08	0.20	0.15	0.27
O08576-2	Isoform 2 of RUN domain-containing protein 3A OS=Mus musculus GN=Rundc3a - [RUN3A_MOUSE]	2.50	4	1	1	1	-0.50	-2.09	1.76	0.16

Q924C5	Alpha-protein kinase 3 OS=Mus musculus GN=Alpk3 PE=1 SV=1 [ALPK3_MOUSE]	1.19	2	1	1	2	0.56	1.52	0.64	1.60
Q8BK62	Olfactomedin-like protein 3 OS=Mus musculus GN=Olfml3 PE=2 SV=2 - [OLFL3_MOUSE]	4.93	1	1	1	1	0.91	0.80	0.59	0.47
O88351	Inhibitor of nuclear factor kappa-B kinase subunit beta OS=Mus musculus GN=Ikbkb PE=1 SV=1 - [IKKB_MOUSE]	5.68	2	3	3	7	0.34	0.63	0.25	0.74
E9Q411	Protein Nbas OS=Mus musculus GN=Nbas PE=2 SV=1 - [E9Q411_MOUSE]	2.80	1	3	4	5	0.54	0.03	0.82	0.26
E9Q0E2	Protein 2700049A03Rik OS=Mus musculus GN=2700049A03Rik PE=2 SV=1 - [E9Q0E2_MOUSE]	1.47	2	1	1	1	0.46	0.40	0.84	0.78
Q9D1M7	Peptidyl-prolyl cis-trans isomerase FKBP11 OS=Mus musculus GN=Fkbp11 PE=2 SV=1 - [FKB11_MOUSE]	9.45	1	2	2	9	0.78	0.70	0.63	0.58
A2ACM0	Regulatory-associated protein of mTOR OS=Mus musculus GN=Rptor PE=4 SV=1 [A2ACM0_MOUSE]	3.07	5	3	3	3	0.59	-0.09	0.26	-0.42
Q9R0M8	UDP-galactose translocator OS=Mus musculus GN=Slc35a2 PE=2 SV=1 - [S35A2_MOUSE]	6.41	2	2	2	11	0.35	0.46	0.26	0.33
Q8BY35-2	Isoform 2 of FYVE, RhoGEF and PH domain-containing protein 2 OS=Mus musculus GN=Fgd2 - [FGD2_MOUSE]	3.47	2	1	1	1	0.38	-0.24	2.02	1.40
Q60960	Importin subunit alpha-5 OS=Mus musculus GN=Kpna1 PE=1 SV=2 - [IMA5_MOUSE]	7.25	4	1	3	19	0.54	0.88	0.45	0.79
J3QPC9	CTD nuclear envelope phosphatase 1 (Fragment) OS=Mus musculus GN=Ctdnep1 PE=4 SV=1 - [J3QPC9_MOUSE]	12.36	1	1	1	1	-0.86	0.35	-0.76	0.45
Q91WD0-2	Isoform 2 of Protein GPR108 OS=Mus musculus GN=Gpr108 - [GP108_MOUSE]	7.30	2	3	3	7	0.63	0.39	0.42	0.17
A2AI93	DMRT-like family C1b OS=Mus musculus GN=Dmrtc1b PE=2 SV=1 - [A2AI93_MOUSE]	14.52	3	2	2	2	0.01	0.28	0.12	0.40
G3UX45	Protein Urb2 OS=Mus musculus GN=Urb2 PE=2 SV=1 - [G3UX45_MOUSE]	1.77	2	2	3	10	0.41	0.36	0.63	0.55
Q3V117	ATP-citrate synthase OS=Mus musculus GN=Acly PE=2 SV=1 - [Q3V117_MOUSE]	48.41	2	1	49	474	0.41	1.12	0.54	1.01
B7ZCP5	Copine-1 (Fragment) OS=Mus musculus GN=Cpne1 PE=2 SV=1 - [B7ZCP5_MOUSE]	16.92	4	1	5	23	-1.28	-0.45	1.04	1.87

Q8R238	Serine dehydratase-like OS=Mus musculus GN=Sdsl PE=2 SV=1 - [SDSL_MOUSE]	6.08	2	2	2	7	0.32	0.65	0.41	0.74
Q69Z69	N-acetyltransferase ESCO1 OS=Mus musculus GN=Esco1 PE=2 SV=2 - [ESCO1_MOUSE]	2.02	3	1	2	4	1.00	1.06	1.05	1.13
Q9D8V7	Signal peptidase complex catalytic subunit SEC11C OS=Mus musculus GN=Sec11c PE=2 SV=3 - [SC11C_MOUSE]	11.98	1	3	3	8	0.82	0.71	0.83	0.72
Q8VD65	Phosphoinositide 3-kinase regulatory subunit 4 OS=Mus musculus GN=Pik3r4 PE=1 SV=3 - [PI3R4_MOUSE]	2.50	1	2	3	3	0.41	0.14	0.53	0.26
Q921U8-2	Isoform L2 of Smoothelin OS=Mus musculus GN=Smtn - [SMTN_MOUSE]	37.02	8	29	29	364	0.39	0.42	0.38	0.38
Q8CDI6	Coiled-coil domain-containing protein 158 OS=Mus musculus GN=Ccdc158 PE=2 SV=1 - [CD158_MOUSE]	3.07	1	2	4	7	0.66	0.54	0.54	0.42
E9Q0S6	Protein Tns1 OS=Mus musculus GN=Tns1 PE=2 SV=1 - [E9Q0S6_MOUSE]	41.10	1	54	58	447	0.36	0.32	0.38	0.31
P61750	ADP-ribosylation factor 4 OS=Mus musculus GN=Arf4 PE=2 SV=2 - [ARF4_MOUSE]	67.78	6	4	10	217	0.39	0.30	0.38	0.36
Q7TQK1	Integrator complex subunit 7 OS=Mus musculus GN=Ints7 PE=1 SV=1 - [INT7_MOUSE]	1.04	1	1	1	4	0.15	0.26	0.45	0.48
Q3UHQ6	Protein dopey-2 OS=Mus musculus GN=Dopey2 PE=2 SV=3 - [DOP2_MOUSE]	1.22	5	2	3	4	0.05	0.09	0.23	0.27
Q8VI78	Phospholipase A1 member A OS=Mus musculus GN=Pla1a PE=2 SV=3 - [PLA1A_MOUSE]	5.26	1	2	2	2	0.31	0.32	0.31	0.32
Q8C0V9-2	Isoform 2 of FERM domain-containing protein 6 OS=Mus musculus GN=Frmd6 - [FRMD6_MOUSE]	3.89	3	2	2	3	-0.22	-0.68	0.21	-0.26
Q8K3F6	Potassium voltage-gated channel subfamily KQT member 3 OS=Mus musculus GN=Kcnq3 PE=1 SV=2 - [KCNQ3_MOUSE]	1.15	1	1	1	1	0.40	-0.24	0.54	-0.11
F7CC56	RB1-inducible coiled-coil protein 1 (Fragment) OS=Mus musculus GN=Rb1cc1 PE=4 SV=1 - [F7CC56_MOUSE]	2.66	4	2	4	5	0.64	0.25	0.21	-0.18
P46978	Dolichyl-diphosphooligosaccharide--protein glycosyltransferase subunit STT3A OS=Mus musculus GN=Stt3a PE=1 SV=1 - [STT3A_MOUSE]	10.64	2	9	9	50	0.42	0.51	0.44	0.52

Q8C102	Polypeptide N-acetylgalactosaminyltransferase 5 OS=Mus musculus GN=Galnt5 PE=2 SV=2 - [GALT5_MOUSE]	4.73	2	4	5	7	-0.07	-0.20	-0.09	-0.22
P26039	Talin-1 OS=Mus musculus GN=Tln1 PE=1 SV=2 - [TLN1_MOUSE]	52.66	8	95	114	1996	0.61	0.63	0.61	0.62
A3KGB4	TBC1 domain family member 8B OS=Mus musculus GN=Tbc1d8b PE=2 SV=1 - [TBC8B_MOUSE]	4.85	1	3	4	23	0.45	0.31	0.47	-0.04
E9Q1F5	Protein Myo5c OS=Mus musculus GN=Myo5c PE=2 SV=1 - [E9Q1F5_MOUSE]	5.22	6	6	10	20	0.28	0.42	0.18	0.54
Q99J83	Autophagy protein 5 OS=Mus musculus GN=Atg5 PE=1 SV=1 - [ATG5_MOUSE]	4.73	1	1	1	2	0.71	1.07	0.58	0.94
Q8BWY7-3	Isoform 3 of Zinc transporter ZIP11 OS=Mus musculus GN=Slc39a11 - [S39AB_MOUSE]	2.99	3	1	1	2	0.33	0.22	0.43	0.32
O55222	Integrin-linked protein kinase OS=Mus musculus GN=Ilk PE=1 SV=2 - [ILK_MOUSE]	36.50	3	15	15	153	0.57	0.61	0.49	0.66
Q9JIF7	Coatomer subunit beta OS=Mus musculus GN=Copb1 PE=1 SV=1 - [COPB_MOUSE]	35.89	1	29	29	176	0.54	0.57	0.45	0.48
G5E8L8	Mitogen activated protein kinase kinase kinase 2 OS=Mus musculus GN=Map3k2 PE=4 SV=1 - [G5E8L8_MOUSE]	4.36	2	2	3	4	0.69	0.76	0.53	0.60
Q9EQQ2	Protein YIPF5 OS=Mus musculus GN=Yipf5 PE=2 SV=1 - [YIPF5_MOUSE]	4.67	1	1	1	2	0.44	0.97	0.55	1.07
Q9DC23	DnaJ homolog subfamily C member 10 OS=Mus musculus GN=Dnajc10 PE=1 SV=2 - [DJC10_MOUSE]	12.61	1	9	9	33	0.84	0.66	0.79	0.67
Q91ZU9	Glutamate receptor ionotropic, NMDA 3B OS=Mus musculus GN=Grin3b PE=1 SV=1 - [NMD3B_MOUSE]	1.20	1	1	1	1	0.10	0.91	0.18	0.99
Q9QZE5	Coatomer subunit gamma-1 OS=Mus musculus GN=Copg1 PE=2 SV=1 - [COPG1_MOUSE]	35.58	2	20	23	170	0.47	0.57	0.46	0.51
Q9QZI9	Serine incorporator 3 OS=Mus musculus GN=Serinc3 PE=1 SV=2 - [SERC3_MOUSE]	8.47	1	2	2	4	0.61	0.53	0.35	0.39
Q08879	Fibulin-1 OS=Mus musculus GN=Fbln1 PE=1 SV=2 - [FBLN1_MOUSE]	18.44	2	12	12	68	0.53	0.55	0.47	0.55
Q8K248	4-hydroxyphenylpyruvate dioxygenase-like protein OS=Mus musculus GN=Hpdl PE=2 SV=1 - [HPDL_MOUSE]	3.23	1	1	1	2	-0.58	-0.93	-0.11	-0.46

Q64685	Beta-galactoside alpha-2,6-sialyltransferase 1 OS=Mus musculus GN=St6gal1 PE=1 SV=2 - [SIAT1_MOUSE]	9.18	1	3	3	4	-0.02	0.22	0.09	0.33
Q3UCQ1-2	Isoform 2 of Forkhead box protein K2 OS=Mus musculus GN=Foxk2 - [FOXK2_MOUSE]	3.27	2	1	2	4	0.09	0.03	0.39	0.33
Q99K01-2	Isoform 2 of Pyridoxal-dependent decarboxylase domain-containing protein 1 OS=Mus musculus GN=Pdxdc1 - [PDXD1_MOUSE]	43.27	6	25	25	223	0.23	0.30	0.21	0.28
Q9DCS9	NADH dehydrogenase [ubiquinone] 1 beta subcomplex subunit 10 OS=Mus musculus GN=Ndufb10 PE=1 SV=3 - [NDUBA_MOUSE]	38.07	2	7	7	61	0.36	0.31	0.26	0.24
Q6PDK8	E3 ubiquitin-protein ligase DTX4 OS=Mus musculus GN=Dtx4 PE=1 SV=2 - [DTX4_MOUSE]	2.11	1	1	1	1	-0.89	-1.20	-0.40	-0.71
P70419	Polypeptide N-acetylgalactosaminyltransferase 3 OS=Mus musculus GN=Galnt3 PE=2 SV=3 - [GALT3_MOUSE]	12.48	1	9	9	20	0.42	0.32	0.26	0.19
Q9ERL9	Guanylate cyclase soluble subunit alpha-3 OS=Mus musculus GN=Gucy1a3 PE=2 SV=2 - [GCYA3_MOUSE]	2.89	1	2	2	3	0.43	0.08	0.42	0.08
E9Q9A0	Type I inositol 3,4-bisphosphate 4-phosphatase OS=Mus musculus GN=Inpp4a PE=2 SV=1 - [E9Q9A0_MOUSE]	6.86	9	3	4	8	0.63	0.60	0.91	0.71
Q810B8	SLIT and NTRK-like protein 4 OS=Mus musculus GN=Slitrk4 PE=2 SV=2 - [SLIK4_MOUSE]	1.79	1	1	1	1	0.16	0.01	0.30	0.15
B2RS76	Carboxypeptidase B1 (Tissue) OS=Mus musculus GN=Cpb1 PE=2 SV=1 - [B2RS76_MOUSE]	18.07	1	6	6	14	0.64	0.72	0.57	0.63
P58021	Transmembrane 9 superfamily member 2 OS=Mus musculus GN=Tm9sf2 PE=2 SV=1 - [TM9S2_MOUSE]	21.90	2	10	10	93	0.25	0.27	0.31	0.22
P55288	Cadherin-11 OS=Mus musculus GN=Cdh11 PE=1 SV=1 - [CAD11_MOUSE]	6.16	3	4	4	6	0.05	-0.08	0.33	0.20
Q9CZX7	Transmembrane protein 55A OS=Mus musculus GN=Tmem55a PE=1 SV=1 - [TM55A_MOUSE]	10.12	1	1	1	4	-0.34	-0.13	0.11	0.32
Q02780-6	Isoform 6 of Nuclear factor 1 A-type OS=Mus musculus GN=Nfia - [NFIA_MOUSE]	9.26	13	1	3	6	0.54	0.87	0.59	0.92

Q3URS9-2	Isoform 2 of Coiled-coil domain-containing protein 51 OS=Mus musculus GN=Ccdc51 - [CCD51_MOUSE]	15.20	2	5	5	17	-0.36	-0.32	1.08	0.60
Q80ZQ3	Uncharacterized protein C8orf74 homolog OS=Mus musculus PE=2 SV=1 - [CH074_MOUSE]	5.03	1	1	1	1	0.29	-0.14	0.31	-0.12
Q8CIE6	Coatomer subunit alpha OS=Mus musculus GN=Copa PE=1 SV=2 - [COPA_MOUSE]	33.09	3	36	36	262	0.52	0.54	0.47	0.55
E9PUV7	Protein Gm872 OS=Mus musculus GN=Gm872 PE=2 SV=1 - [E9PUV7_MOUSE]	1.93	4	2	4	7	0.28	0.24	0.10	0.06
Q9Z0S9	Prenylated Rab acceptor protein 1 OS=Mus musculus GN=Rabac1 PE=1 SV=1 - [PRAF1_MOUSE]	19.46	1	4	4	19	0.35	0.30	0.34	0.45
Q9D666-4	Isoform 4 of SUN domain-containing protein 1 OS=Mus musculus GN=Sun1 - [SUN1_MOUSE]	6.60	6	4	4	6	0.15	0.20	-0.10	0.03
Q8R123	FAD synthase OS=Mus musculus GN=Flad1 PE=1 SV=1 - [FAD1_MOUSE]	10.57	3	5	5	10	0.41	0.32	0.52	0.39
Q6P9S7	Polypeptide N-acetylgalactosaminyltransferase 10 OS=Mus musculus GN=Galnt10 PE=2 SV=1 - [GLT10_MOUSE]	11.44	3	6	6	17	0.18	0.29	0.21	-0.03
F6W623	Histone-lysine N-methyltransferase MLL4 (Fragment) OS=Mus musculus GN=Kmt2b PE=4 SV=1 - [F6W623_MOUSE]	0.84	3	1	2	2	0.14	0.05	0.28	0.18
A6NAU3	Alpha26-takusan OS=Mus musculus GN=Gm3591 PE=2 SV=1 - [A6NAU3_MOUSE]	6.11	61	1	1	2	-0.05	0.03	0.41	0.49
B1ART1	Protein Vps13d OS=Mus musculus GN=Vps13d PE=2 SV=1 - [B1ART1_MOUSE]	2.04	3	6	6	8	-0.80	-0.99	-0.18	-0.37
P0C605-2	Isoform Beta of cGMP-dependent protein kinase 1 OS=Mus musculus GN=Prkg1 - [KGP1_MOUSE]	12.68	1	2	9	23	0.14	0.43	0.43	0.50
Q9CR89-2	Isoform 2 of Endoplasmic reticulum-Golgi intermediate compartment protein 2 OS=Mus musculus GN=Ergic2 - [ERGI2_MOUSE]	4.30	2	1	1	2	0.71	0.44	0.87	0.61
Q8BHB9	Chloride intracellular channel protein 6 OS=Mus musculus GN=Clic6 PE=2 SV=1 - [CLIC6_MOUSE]	32.05	4	8	9	44	0.54	0.40	0.42	0.50

Q8CBY1-2	Isoform 2 of Protein Smaug homolog 1 OS=Mus musculus GN=Samd4a - [SMAG1_MOUSE]	3.44	4	2	2	4	-0.73	0.16	-0.33	0.56
F6SXR7	Dual 3',5'-cyclic-AMP and -GMP phosphodiesterase 11A (Fragment) OS=Mus musculus GN=Pde11a PE=4 SV=1 - [F6SXR7_MOUSE]	3.15	2	1	1	1	0.14	0.09	-0.07	-0.12
Q9JKN1	Zinc transporter 7 OS=Mus musculus GN=Slc30a7 PE=1 SV=1 - [ZNT7_MOUSE]	9.52	1	3	3	14	0.83	0.73	0.59	0.35
Q9ET30	Transmembrane 9 superfamily member 3 OS=Mus musculus GN=Tm9sf3 PE=1 SV=1 - [TM9S3_MOUSE]	17.72	1	8	9	18	0.35	0.06	0.27	0.07
D3YWT0	Signal peptidase complex catalytic subunit SEC11A OS=Mus musculus GN=Sec11a PE=2 SV=1 - [D3YWT0_MOUSE]	25.29	4	5	5	33	0.36	0.20	0.30	0.14
Q921X9	Protein disulfide-isomerase A5 OS=Mus musculus GN=Pdia5 PE=2 SV=1 - [PDIA5_MOUSE]	37.33	1	17	17	108	0.45	0.63	0.51	0.49
Q80VA0	N-acetylgalactosaminyltransferase 7 OS=Mus musculus GN=Galnt7 PE=2 SV=2 - [GALT7_MOUSE]	39.27	3	24	24	137	0.26	0.27	0.25	0.25
Q3UG93	Heat shock factor protein 4 OS=Mus musculus GN=Hsf4 PE=2 SV=1 - [Q3UG93_MOUSE]	5.79	6	2	2	4	0.59	0.59	0.48	0.48
Q8BNI4	Derlin-2 OS=Mus musculus GN=Derl2 PE=2 SV=2 - [DERL2_MOUSE]	17.15	5	2	2	22	-0.49	0.11	-0.44	0.21
Q80UK7-2	Isoform 2 of Spindle assembly abnormal protein 6 homolog OS=Mus musculus GN=Sass6 - [SAS6_MOUSE]	8.04	2	2	4	32	0.30	0.46	0.17	0.34
Q9JHU4	Cytoplasmic dynein 1 heavy chain 1 OS=Mus musculus GN=Dync1h1 PE=1 SV=2 - [DYHC1_MOUSE]	35.59	2	146	149	769	0.33	0.25	0.34	0.28
Q70IV5	Synemin OS=Mus musculus GN=Synm PE=1 SV=2 - [SYNEM_MOUSE]	53.75	2	12	67	599	0.69	0.67	0.70	0.54
P01747	Ig heavy chain V region 36-65 OS=Mus musculus PE=1 SV=1 - [HVM03_MOUSE]	11.67	2	1	1	1	-0.16	-0.31	-0.32	-0.48
Q9D5V2	Kelch-like protein 10 OS=Mus musculus GN=Klhl10 PE=1 SV=1 - [KLH10_MOUSE]	3.13	1	1	1	1	0.64	1.48	0.53	1.37
Q8CI94	Glycogen phosphorylase, brain form OS=Mus musculus GN=Pygb PE=1 SV=3 - [PYGB_MOUSE]	47.09	1	29	37	512	0.37	0.34	0.37	0.41

A6X940	Fermitin family homolog 2 (Fragment) OS=Mus musculus GN=Fermt2 PE=2 SV=1 - [A6X940_MOUSE]	54.26	1	1	11	46	0.98	1.24	0.97	0.95
Q91V92	ATP-citrate synthase OS=Mus musculus GN=Acly PE=1 SV=1 - [ACLY_MOUSE]	48.40	1	1	49	466	0.53	-0.95	0.30	-1.18
Q9JIB3	Baculoviral IAP repeat-containing protein 1g OS=Mus musculus GN=Naip7 PE=3 SV=1 [BIR1G_MOUSE]	4.49	2	1	5	12	0.77	1.10	0.89	1.22
Q9Z1Z0	General vesicular transport factor p115 OS=Mus musculus GN=Uso1 PE=1 SV=2 - [USO1_MOUSE]	32.01	4	23	23	144	0.37	0.38	0.43	0.41
M0QWY7	N-acetylglucosaminyl-phosphatidylinositol biosynthetic protein OS=Mus musculus GN=Piga PE=4 SV=1 - [M0QWY7_MOUSE]	7.49	2	1	1	9	-0.25	-0.01	-0.46	-0.19
F6U4F1	Protein Sh3tc1 (Fragment) OS=Mus musculus GN=Sh3tc1 PE=4 SV=1 - [F6U4F1_MOUSE]	2.27	2	2	2	4	0.37	1.47	-0.07	1.03
P62737	Actin, aortic smooth muscle OS=Mus musculus GN=Acta2 PE=1 SV=1 - [ACTA_MOUSE]	72.68	6	4	31	7898	0.49	0.46	0.47	0.45
Q8VCG3	WD repeat-containing protein 74 OS=Mus musculus GN=Wdr74 PE=2 SV=1 - [WDR74_MOUSE]	6.77	1	2	2	3	-0.38	-0.75	0.35	-0.02
Q3UM29	Conserved oligomeric Golgi complex subunit 7 OS=Mus musculus GN=Cog7 PE=2 SV=1 - [COG7_MOUSE]	5.71	1	4	4	7	0.16	0.21	0.25	0.34
P05784	Keratin, type I cytoskeletal 18 OS=Mus musculus GN=Krt18 PE=1 SV=5 [K1C18_MOUSE]	71.63	3	28	31	625	0.63	0.61	0.61	0.62
Q9D0F3	Protein ERGIC-53 OS=Mus musculus GN=Lman1 PE=2 SV=1 - [LMAN1_MOUSE]	40.43	2	18	19	137	0.43	0.24	0.41	0.31
Q69ZX8	Actin-binding LIM protein 3 OS=Mus musculus GN=Ablim3 PE=1 SV=2 - [ABLM3_MOUSE]	8.06	1	2	3	3	0.51	0.22	0.63	0.33
Q6PFQ7-2	Isoform 2 of Ras GTPase-activating protein 4 OS=Mus musculus GN=Rasa4 - [RASL2_MOUSE]	2.51	3	2	2	3	0.28	0.29	0.75	0.77
P01633	Ig kappa chain V19-17 OS=Mus musculus GN=Igk-V19-17 PE=1 SV=1 - [KV5A1_MOUSE]	38.26	1	3	3	6	-0.11	-0.31	0.30	0.10
Q91XA2	Golgi membrane protein 1 OS=Mus musculus GN=Golm1 PE=1 SV=2 - [GOLM1_MOUSE]	52.16	1	15	16	243	0.36	0.45	0.38	0.48

Q9JIP7	Solute carrier family 15 member 1 OS=Mus musculus GN=Slc15a1 PE=1 SV=2 - [S15A1_MOUSE]	1.27	1	1	1	1	0.88	0.61	0.95	0.68
D3YU58	Anaphase-promoting complex subunit 7 OS=Mus musculus GN=Anapc7 PE=2 SV=1 - [D3YU58_MOUSE]	4.77	2	2	2	4	0.47	0.55	0.51	0.58
Q7TQE6	Macoilin OS=Mus musculus GN=Tmem57 PE=2 SV=1 - [MACOI_MOUSE]	4.67	1	2	3	10	-0.69	0.03	-0.04	0.78
Q8BS95	Golgi pH regulator OS=Mus musculus GN=Gpr89a PE=2 SV=2 - [GPHR_MOUSE]	5.93	1	2	2	4	0.48	0.76	0.36	0.63
Q7TMC8	L-fucose kinase OS=Mus musculus GN=Fuk PE=2 SV=1 - [Q7TMC8_MOUSE]	12.02	1	8	8	35	0.41	0.27	0.37	0.16
E9PUI0	Alpha-N-acetylgalactosaminide alpha-2,6-sialyltransferase 6 OS=Mus musculus GN=St6galnac6 PE=2 SV=1 - [E9PUI0_MOUSE]	18.06	5	6	6	40	0.29	0.27	0.38	0.22
E9PUB9	Chondroitin sulfate synthase 2 OS=Mus musculus GN=Chpf PE=2 SV=1 - [E9PUB9_MOUSE]	1.63	2	1	1	4	-0.16	-0.11	-0.16	-0.12
Q8BHJ6	Serine incorporator 5 OS=Mus musculus GN=Serinc5 PE=2 SV=1 - [SERC5_MOUSE]	13.45	1	2	2	3	-0.66	-0.28	-0.74	-0.36
O88200	C-type lectin domain family 11 member A OS=Mus musculus GN=Clec11a PE=2 SV=1 - [CLC11_MOUSE]	2.44	1	1	1	1	-0.53	-0.19	-0.05	0.29
Q8VC42	Uncharacterized protein C18orf8 homolog OS=Mus musculus GN=Mic1 PE=2 SV=1 - [MIC1_MOUSE]	3.65	1	2	2	2	0.04	0.10	0.33	0.38
G3UXG8	All-trans retinoic acid-induced differentiation factor (Fragment) OS=Mus musculus GN=Atraid PE=2 SV=1 - [G3UXG8_MOUSE]	4.90	4	1	1	1	-0.87	-0.71	-0.16	0.00
G3UZJ5	Casein kinase II subunit beta (Fragment) OS=Mus musculus GN=Csnk2b PE=2 SV=2 - [G3UZJ5_MOUSE]	35.44	4	1	7	31	-0.62	-0.26	0.31	0.67
D6RDS1	Lipase maturation factor 1 OS=Mus musculus GN=Lmf1 PE=2 SV=1 - [D6RDS1_MOUSE]	26.56	5	2	2	3	0.12	0.42	0.24	0.53
Q8K0T7	Protein unc-13 homolog C OS=Mus musculus GN=Unc13c PE=1 SV=3 - [UN13C_MOUSE]	0.72	1	1	1	10	1.09	0.83	0.98	0.67
Q9CYN9	Renin receptor OS=Mus musculus GN=Atp6ap2 PE=2 SV=2 - [RENR_MOUSE]	13.71	1	4	4	6	0.28	0.20	0.28	0.17

Q3V3Q7	Phosphofurin acidic cluster sorting protein 2 OS=Mus musculus GN=Pacs2 PE=1 SV=2 [PACS2_MOUSE]	3.02	2	1	1	1	0.27	0.69	0.16	0.58
Q2LKU9	NACHT-, LRR-, and PYD-containing protein 1 paralog a OS=Mus musculus GN=Nlrp1a PE=2 SV=1 - [Q2LKU9_MOUSE]	1.18	2	1	2	4	1.06	0.87	0.88	0.69
Q3U2P1	Protein transport protein Sec24A OS=Mus musculus GN=Sec24a PE=1 SV=1 - [SC24A_MOUSE]	10.64	4	9	10	33	0.39	0.12	0.43	0.24
Q61151	Serine/threonine-protein phosphatase 2A 56 kDa regulatory subunit epsilon isoform OS=Mus musculus GN=Ppp2r5e PE=2 SV=3 - [2A5E_MOUSE]	19.70	1	4	7	23	0.46	0.03	0.48	0.18
Q9QZC7-2	Isoform 2 of Pleckstrin homology domain-containing family B member 2 OS=Mus musculus GN=Plekhb2 - [PKHB2_MOUSE]	8.96	4	2	2	2	0.10	0.29	0.16	0.34
P14094	Sodium/potassium-transporting ATPase subunit beta-1 OS=Mus musculus GN=Atp1b1 PE=1 SV=1 - [AT1B1_MOUSE]	35.53	1	12	12	254	0.38	0.38	0.31	0.36
Q9JJ78	Lymphokine-activated killer T-cell-originated protein kinase OS=Mus musculus GN=Pbk PE=2 SV=1 - [TOPK_MOUSE]	2.12	1	1	1	1	0.87	-0.58	0.87	-0.58
E9PVA8	Protein Gcn1l1 OS=Mus musculus GN=Gcn1l1 PE=2 SV=1 - [E9PVA8_MOUSE]	12.80	2	30	32	80	0.55	0.45	0.55	0.56
Q6XQH0	Galactose-3-O-sulfotransferase 2 OS=Mus musculus GN=Gal3st2 PE=2 SV=1 - [G3ST2_MOUSE]	7.11	4	3	3	3	0.71	0.53	0.71	0.52
E9Q9Y4	Methylcytosine dioxygenase TET1 OS=Mus musculus GN=Tet1 PE=2 SV=1 - [E9Q9Y4_MOUSE]	1.62	2	2	3	3	-0.06	-0.07	0.33	0.31
Q05922	Dual specificity protein phosphatase 2 OS=Mus musculus GN=Dusp2 PE=2 SV=2 - [DUS2_MOUSE]	7.86	1	1	1	2	0.65	0.85	0.48	0.68
Q8C2S0	Ubiquitin carboxyl-terminal hydrolase 44 OS=Mus musculus GN=Usp44 PE=2 SV=3 - [UBP44_MOUSE]	1.97	1	1	1	3	-0.33	-0.16	-0.58	0.06
Q9EPN1-3	Isoform 3 of Neurobeachin OS=Mus musculus GN=Nbea - [NBEA_MOUSE]	3.27	4	3	8	12	-0.46	0.04	-0.44	0.05

E9PX70	Collagen alpha-1(XII) chain OS=Mus musculus GN=Col12a1 PE=2 SV=1 - [E9PX70_MOUSE]	30.03	8	68	69	465	0.54	0.51	0.49	0.49
D3YWQ8	Protein kinase C eta type OS=Mus musculus GN=Prkch PE=2 SV=1 [D3YWQ8_MOUSE]	4.33	2	1	2	2	0.47	0.53	0.56	0.62
A2ASX1	Growth factor receptor-bound protein 14 (Fragment) OS=Mus musculus GN=Grb14 PE=2 SV=1 - [A2ASX1_MOUSE]	4.98	2	1	1	2	0.33	0.01	0.53	0.20
Q68FM6	Protein phosphatase 1 regulatory subunit 29 OS=Mus musculus GN=Elfn2 PE=1 SV=1 - [PPR29_MOUSE]	5.10	2	2	2	6	0.95	0.77	0.72	0.60
E9PX48	Dedicator of cytokinesis protein 7 OS=Mus musculus GN=Dock7 PE=2 SV=1 - [E9PX48_MOUSE]	9.49	5	15	16	34	0.56	0.64	0.46	0.54
Q8BRK8	5'-AMP-activated protein kinase catalytic subunit alpha-2 OS=Mus musculus GN=Prkaa2 PE=1 SV=3 - [AAPK2_MOUSE]	18.84	1	4	6	22	0.67	0.24	0.40	0.01
Q9CYR6	Phosphoacetylglucosamine mutase OS=Mus musculus GN=Pgm3 PE=1 SV=1 - [AGM1_MOUSE]	35.42	2	15	15	90	0.28	0.31	0.17	0.31
Q8K3E5-2	Isoform 2 of Jouberin OS=Mus musculus GN=Ahi1 - [AHI1_MOUSE]	1.49	3	1	1	1	0.22	0.18	0.23	0.19
E9Q8Q5	Breast carcinoma-amplified sequence 1 homolog OS=Mus musculus GN=Bcas1 PE=2 SV=1 - [E9Q8Q5_MOUSE]	44.46	3	1	18	70	0.08	0.37	0.22	0.18
Q8C3Y4	Kinetochore-associated protein 1 OS=Mus musculus GN=Kntc1 PE=1 SV=2 - [KNTC1_MOUSE]	3.04	1	2	2	2	0.28	0.62	0.60	0.94
F6UUZ3	Protein Dgkk (Fragment) OS=Mus musculus GN=Dgkk PE=4 SV=1 - [F6UUZ3_MOUSE]	1.69	2	1	2	6	-0.30	-0.14	-0.11	0.05
Q9QXP7	Complement C1q tumor necrosis factor-related protein 1 OS=Mus musculus GN=C1qtnf1 PE=2 SV=1 - [C1QT1_MOUSE]	2.85	1	1	1	1	0.43	0.82	0.40	0.78
Q9JI59	Junctional adhesion molecule B OS=Mus musculus GN=Jam2 PE=1 SV=1 - [JAM2_MOUSE]	3.36	1	1	1	2	-0.17	0.05	0.28	0.51
Q99JH8	ER lumen protein retaining receptor 1 OS=Mus musculus GN=Kdelr1 PE=2 SV=1 - [ERD21_MOUSE]	12.74	5	2	2	6	0.76	0.79	0.55	0.58

Q811I0	ATP synthase mitochondrial F1 complex assembly factor 1 OS=Mus musculus GN=Atpaf1 PE=2 SV=1 - [ATPF1_MOUSE]	10.80	5	2	2	3	0.01	-0.14	-0.04	-0.20
D3YWJ5	Tumor necrosis factor alpha-induced protein 8 OS=Mus musculus GN=Tnfaip8 PE=2 SV=1 - [D3YWJ5_MOUSE]	18.62	9	3	3	11	0.60	0.52	0.62	0.59
Q9DBE8	Alpha-1,3/1,6-mannosyltransferase ALG2 OS=Mus musculus GN=Alg2 PE=2 SV=2 - [ALG2_MOUSE]	14.70	2	5	5	10	-0.04	-0.15	-0.15	-0.07
Q9D958	Signal peptidase complex subunit 1 OS=Mus musculus GN=Spcs1 PE=2 SV=3 [SPCS1_MOUSE]	8.07	1	2	2	5	0.79	1.10	0.56	0.84
I7HPY1	Novel protein OS=Mus musculus GN=Fam3a PE=4 SV=1 - [I7HPY1_MOUSE]	17.71	5	2	3	12	0.68	0.68	0.61	0.61
Q9D1N9	39S ribosomal protein L21, mitochondrial OS=Mus musculus GN=Mrpl21 PE=2 SV=1 - [RM21_MOUSE]	26.79	3	3	3	6	-0.13	-0.24	0.50	0.39
Q8R088	Golgi phosphoprotein 3-like OS=Mus musculus GN=Golph3l PE=2 SV=1 - [GLP3L_MOUSE]	44.91	12	12	13	85	0.47	0.50	0.43	0.49
Q9EPT5	Solute carrier organic anion transporter family member 2A1 OS=Mus musculus GN=Slco2a1 PE=1 SV=2 - [SO2A1_MOUSE]	2.02	1	1	1	2	0.59	0.57	0.46	0.44
Q8BLN6	Protein unc-80 homolog OS=Mus musculus GN=Unc80 PE=1 SV=2 - [UNC80_MOUSE]	0.31	2	1	1	2	0.15	0.32	0.09	0.24
Q924C1	Exportin-5 OS=Mus musculus GN=Xpo5 PE=2 SV=1 - [XPO5_MOUSE]	7.72	3	8	8	16	0.47	0.15	0.65	0.35
Q8BGB8	Ubiquinone biosynthesis protein COQ4 homolog, mitochondrial OS=Mus musculus GN=Coq4 PE=2 SV=1 - [COQ4_MOUSE]	18.05	3	4	4	13	0.33	0.15	0.58	0.40
Q6ZWQ7	Signal peptidase complex subunit 3 OS=Mus musculus GN=Spcs3 PE=2 SV=1 [Q6ZWQ7_MOUSE]	21.67	1	4	4	37	0.34	0.25	0.33	0.26
P01900	H-2 class I histocompatibility antigen, D-D alpha chain OS=Mus musculus GN=H2-D1 PE=1 SV=1 - [HA12_MOUSE]	10.68	5	2	3	38	-0.03	-0.34	0.53	0.21
Q3TKT4	Transcription activator BRG1 OS=Mus musculus GN=Smarca4 PE=1 SV=1 - [SMCA4_MOUSE]	6.82	9	3	8	29	0.32	0.40	0.45	0.63

Q8BL00	Cadherin-related family member 3 OS=Mus musculus GN=Cdhr3 PE=2 SV=2 - [CDHR3_MOUSE]	2.41	1	1	1	2	-0.13	0.57	0.16	0.86
Q91VS8	FERM, RhoGEF and pleckstrin domain-containing protein 2 OS=Mus musculus GN=Farp2 PE=1 SV=2 [FARP2_MOUSE]	9.01	1	5	8	12	-0.68	-0.23	-0.60	-0.37
D3Z183	Major facilitator superfamily domain-containing protein 6 OS=Mus musculus GN=Mfsd6 PE=2 SV=1 - [D3Z183_MOUSE]	7.68	4	3	4	9	0.48	0.00	0.10	-0.38
Q60967	Bifunctional 3'-phosphoadenosine 5'-phosphosulfate synthase 1 OS=Mus musculus GN=Papss1 PE=2 SV=1 - [PAPS1_MOUSE]	19.39	1	12	14	87	0.31	0.34	0.37	0.30
Q64444	Carbonic anhydrase 4 OS=Mus musculus GN=Ca4 PE=1 SV=1 - [CAH4_MOUSE]	22.95	2	7	7	88	0.34	0.29	0.20	0.46
Q6A099	MKIAA0248 protein (Fragment) OS=Mus musculus GN=Gbf1 PE=2 SV=1 - [Q6A099_MOUSE]	9.93	7	12	14	51	0.39	0.32	0.39	0.31
Q9EQJ0	Two pore calcium channel protein 1 OS=Mus musculus GN=Tpcn1 PE=2 SV=1 - [TPC1_MOUSE]	2.69	2	2	2	3	0.20	-0.32	0.32	-0.20
Q6NXL1	Protein Sec24d OS=Mus musculus GN=Sec24d PE=2 SV=1 - [Q6NXL1_MOUSE]	16.28	1	13	14	106	0.28	0.49	0.57	0.56
Q3UPH1	Protein PRRC1 OS=Mus musculus GN=Prrc1 PE=2 SV=1 - [PRRC1_MOUSE]	15.80	1	6	6	33	0.56	0.46	0.49	0.48
P47746	Cannabinoid receptor 1 OS=Mus musculus GN=Cnr1 PE=2 SV=1 - [CNR1_MOUSE]	2.11	1	1	1	2	-0.97	-0.87	-0.82	-0.73
E9Q3V2	UDP-N-acetylglucosamine transporter (Fragment) OS=Mus musculus GN=Slc35a3 PE=2 SV=1 - [E9Q3V2_MOUSE]	7.43	1	1	1	5	0.07	0.08	-0.14	-0.06
O54692	Centromere/kinetochore protein zw10 homolog OS=Mus musculus GN=Zw10 PE=2 SV=3 [ZW10_MOUSE]	8.86	1	5	5	9	-0.02	0.69	-0.01	0.60
P15306	Thrombomodulin OS=Mus musculus GN=Thbd PE=1 SV=1 - [TRBM_MOUSE]	1.39	1	1	1	3	0.31	0.64	0.38	0.71
Q8BZJ8	Uncharacterized protein C8orf34 homolog OS=Mus musculus PE=2 SV=1 - [CH034_MOUSE]	5.88	2	1	1	2	-0.25	-0.01	-0.17	0.08
G3X922	MCG115602 OS=Mus musculus GN=Dnajc13 PE=4 SV=1 - [G3X922_MOUSE]	11.19	1	17	17	53	0.19	0.22	0.25	0.23

Q8K3S0	MCG55901 OS=Mus musculus GN=4930519F16Rik PE=3 SV=1 - [Q8K3S0_MOUSE]	4.83	1	1	1	1	1.18	1.09	0.94	0.85
F2Z3Z1	Hippocalcin-like protein 4 (Fragment) OS=Mus musculus GN=Hpcal4 PE=2 SV=1 - [F2Z3Z1_MOUSE]	7.10	2	1	1	1	-0.28	-0.48	0.04	-0.16
E9QMI9	Serine/threonine-protein kinase WNK2 OS=Mu musculus GN=Wnk2 PE=2 SV=1 - [E9QMI9_MOUSE]	2.82	19	2	4	7	-0.09	0.08	-0.03	0.14
Q8BI84	Melanoma inhibitory activity protein 3 OS=Mus musculus GN=Mia3 PE=1 SV=2 - [MIA3_MOUSE]	29.64	5	43	43	169	0.39	0.38	0.28	0.30
F8WIE5	E3 ubiquitin-protein ligase HECTD1 OS=Mus musculus GN=Hectd1 PE=2 SV=1 - [F8WIE5_MOUSE]	4.44	2	9	10	14	0.40	0.04	0.44	0.29
Q8BUR4	Dedicator of cytokinesis protein 1 OS=Mus musculus GN=Dock1 PE=1 SV=3 - [DOCK1_MOUSE]	6.17	2	9	10	24	0.22	0.09	0.07	-0.01
E9PZP8	Protein Herc1 OS=Mus musculus GN=Herc1 PE=2 SV=1 - [E9PZP8_MOUSE]	1.77	1	3	5	5	-0.43	-0.43	-0.41	-0.42
P20615	Homeobox protein Hox-B9 OS=Mus musculus GN=Hoxb9 PE=2 SV=3 - [HXB9_MOUSE]	2.80	1	1	1	3	0.22	0.10	0.15	0.03
Q8R373	CXADR-like membrane protein OS=Mus musculus GN=Clmp PE=1 SV=1 - [CLMP_MOUSE]	5.63	1	2	2	3	-0.38	-0.74	-0.09	-0.44
Q9WV71	Ankyrin repeat and SOCS box protein 4 OS=Mus musculus GN=Asb4 PE=1 SV=1 - [ASB4_MOUSE]	3.05	1	1	1	1	-0.52	-3.45	0.64	-2.29
Q91XD7	Cysteine-rich with EGF-like domain protein 1 OS=Mus musculus GN=Creld1 PE=2 SV=1 - [CREL1_MOUSE]	4.52	1	2	2	9	-0.05	-0.25	0.16	-0.23
Q9JIA6	Galactokinase OS=Mus musculus GN=Galk1 PE=2 SV=1 - [Q9JIA6_MOUSE]	26.02	2	9	9	61	-0.01	-0.08	0.13	0.08
A6PWX1	Calcium channel flower homolog OS=Mus musculus GN=Cacfd1 PE=2 SV=1 - [A6PWX1_MOUSE]	8.26	2	1	1	3	0.23	0.39	0.06	0.22
Q9Z2V4	Phosphoenolpyruvate carboxykinase, cytosolic [GTP] OS=Mus musculus GN=Pck1 PE=2 SV=1 - [PCKGC_MOUSE]	25.24	1	12	12	44	-0.49	-0.23	-0.38	-0.24

P61620	Protein transport protein Sec61 subunit alpha isoform 1 OS=Mus musculus GN=Sec61a1 PE=2 SV=2 - [S61A1_MOUSE]	11.97	3	6	6	38	0.53	0.36	0.40	0.48
Q8BGT5	Alanine aminotransferase 2 OS=Mus musculus GN=Gpt2 PE=2 SV=1 - [ALAT2_MOUSE]	13.03	2	4	5	7	-0.17	-0.04	-0.08	0.05
P01675	Ig kappa chain V-VI region XRPC 44 OS=Mus musculus PE=1 SV=1 - [KV6A1_MOUSE]	7.48	8	1	1	6	0.55	0.56	0.60	0.65
P53995	Anaphase-promoting complex subunit 1 OS=Mus musculus GN=Anapc1 PE=1 SV=2 - [APC1_MOUSE]	5.76	2	7	7	18	0.33	0.34	0.39	0.42
Q61070	Etoposide-induced protein 2.4 OS=Mus musculus GN=Ei24 PE=1 SV=3 - [EI24_MOUSE]	9.41	1	3	3	5	0.16	0.06	0.36	0.26
Q3UGY8	Brefeldin A-inhibited guanine nucleotide-exchange protein 3 OS=Mus musculus GN=Arfgef3 PE=1 SV=1 - [BIG3_MOUSE]	2.21	1	3	3	9	0.14	0.64	-0.03	0.47
Q9JL04	Formin-2 OS=Mus musculus GN=Fmn2 PE=1 SV=2 - [FMN2_MOUSE]	1.33	1	1	2	7	0.05	0.37	1.36	1.14
A2A9I0	Golgi SNAP receptor complex member 2 OS=Mus musculus GN=Gosr2 PE=4 SV=1 [A2A9I0_MOUSE]	13.33	2	2	2	5	-0.06	0.07	0.11	0.24
Q9EPC1	Alpha-parvin OS=Mus musculus GN=Parva PE=1 SV=1 - [PARVA_MOUSE]	29.03	6	10	12	115	0.39	0.32	0.46	0.45
F8VPK0	Protein Ttc37 OS=Mus musculus GN=Ttc37 PE=2 SV=1 - [F8VPK0_MOUSE]	7.55	1	7	7	14	0.24	0.44	0.33	0.52
Q7TMB8	Cytoplasmic FMR1-interacting protein 1 OS=Mus musculus GN=Cyfip1 PE=1 SV=1 - [CYFP1_MOUSE]	12.37	4	9	14	40	0.24	0.21	0.30	0.17
Q9Z2F2	2'-5'-oligoadenylate synthase-like protein 2 OS=Mus musculus GN=Oasl2 PE=1 SV=2 [OASL2_MOUSE]	17.52	4	5	6	16	0.58	0.82	0.43	0.64
Q99PG0	Arylacetamide deacetylase OS=Mus musculus GN=Aadac PE=1 SV=3 - [AAAD_MOUSE]	14.57	1	4	4	11	1.01	0.78	0.95	1.03
E9PYJ8	Histone acetyltransferase p300 OS=Mus musculus GN=Ep300 PE=4 SV=1 - [E9PYJ8_MOUSE]	5.68	4	7	7	8	0.15	0.18	0.56	0.59
P35276	Ras-related protein Rab-3D OS=Mus musculus GN=Rab3d PE=1 SV=1 - [RAB3D_MOUSE]	44.29	16	4	9	82	0.16	0.37	0.00	0.37

E9Q8T1	Transforming acidic coiled-coil-containing protein 2 OS=Mus musculus GN=Tacc2 PE=2 SV=1 - [E9Q8T1_MOUSE]	3.44	5	2	7	30	0.60	0.40	0.59	0.54
P61979-3	Isoform 3 of Heterogeneous nuclear ribonucleoprotein K OS=Mus musculus GN=Hnrnpk - [HNRPK_MOUSE]	48.75	9	3	20	334	0.10	0.31	0.22	0.49
P35428	Transcription factor HES-1 OS=Mus musculus GN=Hes1 PE=1 SV=1 - [HES1_MOUSE]	4.96	1	1	1	1	-0.79	-0.34	-0.23	0.22
Q8VDN2	Sodium/potassium-transporting ATPase subunit alpha-1 OS=Mus musculus GN=Atp1a1 PE=1 SV=1 - [AT1A1_MOUSE]	49.76	1	31	52	1298	0.27	0.27	0.26	0.28
A9C437	Chloride channel protein 2 OS=Mus musculus GN=Clcn2 PE=2 SV=1 - [A9C437_MOUSE]	10.33	3	6	6	7	0.52	0.19	0.37	0.17
Q8C0Z1	Protein ITFG3 OS=Mus musculus GN=Itfg3 PE=1 SV=1 - [ITFG3_MOUSE]	15.14	3	7	7	18	0.41	0.35	0.33	0.27
Q6PHU5	Sortilin OS=Mus musculus GN=Sort1 PE=1 SV=1 - [SORT_MOUSE]	13.09	3	11	12	26	0.16	0.25	0.22	0.33
Q8BH79	Anoctamin-10 OS=Mus musculus GN=Ano10 PE=2 SV=1 - [ANO10_MOUSE]	11.38	4	7	7	21	0.32	0.29	0.22	0.31
E9PXX7	Thioredoxin domain-containing protein 5 OS=Mus musculus GN=Txndc5 PE=2 SV=1 - [E9PXX7_MOUSE]	64.24	2	19	20	459	-0.09	0.01	-0.21	-0.01
E9Q9P4	Calcium-activated potassium channel subunit alpha-1 OS=Mus musculus GN=Kcnma1 PE=4 SV=2 - [E9Q9P4_MOUSE]	3.04	14	2	2	4	0.22	0.07	0.11	-0.05
Q06138	Calcium-binding protein 39 OS=Mus musculus GN=Cab39 PE=1 SV=2 - [CAB39_MOUSE]	38.12	3	12	14	80	0.98	0.64	0.86	0.67
A2AVA0-2	Isoform 2 of Sushi, von Willebrand factor type A, EGF and pentraxin domain-containing protein 1 OS=Mus musculus GN=Svep1 - [SVEP1_MOUSE]	6.36	2	2	2	4	0.95	0.54	1.33	0.92
Q8K1E0	Syntaxin-5 OS=Mus musculus GN=Stx5 PE=1 SV=3 - [STX5_MOUSE]	26.48	11	9	10	27	0.46	0.45	0.61	0.56
A2AEX8	Four and a half LIM domains 1, isoform CRA_b OS=Mus musculus GN=Fhl1 PE=4 SV=1 - [A2AEX8_MOUSE]	54.39	10	16	16	233	0.55	0.63	0.48	0.58
Q8VHX6	Filamin-C OS=Mus musculus GN=Flnc PE=1 SV=3 - [FLNC_MOUSE]	58.44	4	113	131	1773	0.52	0.53	0.49	0.50

F6UFZ5	Protein Pcf11 (Fragment) OS=Mus musculus GN=Pcf11 PE=4 SV=1 - [F6UFZ5_MOUSE]	0.54	2	1	1	1	0.57	0.81	0.48	0.72
Q8K0F1-2	Isoform 2 of TBC1 domain family member 23 OS=Mus musculus GN=Tbc1d23 - [TBC23_MOUSE]	22.66	2	3	3	5	0.17	-0.09	0.52	0.26
P69566	Ran-binding protein 9 OS=Mus musculus GN=Ranbp9 PE=1 SV=1 - [RANB9_MOUSE]	3.68	3	2	2	5	-0.26	0.22	0.08	0.56
Q8VD29	Sodium/potassium/calcium exchanger 3 OS=Mus musculus GN=Slc24a3 PE=2 SV=1 - [Q8VD29_MOUSE]	4.20	2	2	2	3	-0.50	-0.66	0.14	-0.06
Q80TL7-2	Isoform 2 of Protein MON2 homolog OS=Mus musculus GN=Mon2 - [MON2_MOUSE]	5.80	3	9	9	23	0.28	0.25	0.38	0.36
Q9DAG7	Protein Styxl1 OS=Mus musculus GN=Styxl1 PE=2 SV=1 - [Q9DAG7_MOUSE]	8.47	3	1	1	2	-0.37	-0.85	0.11	-0.37
P51655	Glypican-4 OS=Mus musculus GN=Gpc4 PE=2 SV=2 - [GPC4_MOUSE]	13.11	3	6	6	13	0.52	0.69	0.66	0.68
Q8K327	Chromosome alignment maintaining phosphoprotein 1 OS=Mus musculus GN=Champ1 PE=1 SV=1 - [CHAP1_MOUSE]	3.37	1	2	2	2	0.09	0.53	-0.01	0.43
Q9DCF9-2	Isoform 2 of Translocon associated protein subunit gamma OS=Mus musculus GN=Ssr3 - [SSRG_MOUSE]	7.82	2	1	1	28	0.29	0.27	0.34	0.40
Q6PD28	Protein Ppp2r5b OS=Mus musculus GN=Ppp2r5b PE=2 SV=1 - [Q6PD28_MOUSE]	10.87	1	1	3	7	0.21	0.02	0.62	0.43
A2AGS2	Dual-specificity mitogen-activated protein kinase kinase 6 OS=Mus musculus GN=Map2k6 PE=2 SV=1 - [A2AGS2_MOUSE]	30.72	2	5	8	19	0.21	0.16	0.25	-0.13
Q68FD5	Clathrin heavy chain 1 OS=Mus musculus GN=Cltc PE=1 SV=3 - [CLH1_MOUSE]	42.87	3	63	63	1015	0.27	0.31	0.31	0.30
Q9DB10	Single-pass membrane protein with aspartate-rich tail 1, mitochondrial OS=Mus musculus GN=Smdt1 PE=2 SV=1 - [SMDT1_MOUSE]	9.35	1	1	1	4	0.74	0.71	0.52	0.49
Q8BXN9	Transmembrane protein 87A OS=Mus musculus GN=Tmem87a PE=1 SV=1 - [TM87A_MOUSE]	10.63	6	5	5	21	0.59	0.58	0.67	0.77
A2AD84	Protein 2610018G03Rik OS=Mus musculus GN=2610018G03Rik PE=2 SV=1 - [A2AD84_MOUSE]	16.58	2	1	7	17	0.42	0.38	0.60	0.56

P97411-2	Isoform 2 of Islet cell autoantigen 1 OS=Mus musculus GN=Ica1 - [ICA69_MOUSE]	4.16	7	2	2	4	0.95	0.70	0.44	0.19
D3Z784	Probable UDP-sugar transporter protein SLC35A5 OS=Mus musculus GN=Slc35a5 PE=2 SV=1 - [D3Z784_MOUSE]	7.26	3	1	1	1	-0.36	-0.08	0.37	0.65
O09117-2	Isoform 2 of Synaptophysin-like protein 1 OS=Mus musculus GN=Sypl1 - [SYPL1_MOUSE]	17.28	3	4	4	29	0.47	0.63	0.48	0.60
Q8R3I3	Conserved oligomeric Golgi complex subunit 6 OS=Mus musculus GN=Cog6 PE=2 SV=2 - [COG6_MOUSE]	3.65	1	2	3	10	0.44	0.54	0.14	0.24
Q99LM2	CDK5 regulatory subunit-associated protein 3 OS=Mus musculus GN=Cdk5rap3 PE=2 SV=1 - [CK5P3_MOUSE]	16.30	4	6	6	30	0.58	0.49	0.60	0.57
E9QKZ2	Importin-9 OS=Mus musculus GN=Ipo9 PE=2 SV=1 - [E9QKZ2_MOUSE]	4.23	5	4	4	10	0.48	0.15	0.38	0.17
B5X0E4	ATP-binding cassette sub-family B member 5 variant OS=Mus musculus GN=Abcb5 PE=2 SV=1 - [B5X0E4_MOUSE]	1.83	1	1	2	4	0.38	1.23	-0.08	0.76
F8VPU2	FERM, RhoGEF and pleckstrin domain-containing protein 1 OS=Mus musculus GN=Farp1 PE=1 SV=1 [FARP1_MOUSE]	17.46	4	14	14	44	0.13	-0.02	0.30	0.11
Q10470	Beta-1,4-mannosyl-glycoprotein 4-beta-N-acetylglucosaminyltransferase OS=Mus musculus GN=Mgat3 PE=1 SV=2 - [MGAT3_MOUSE]	13.20	2	5	5	16	0.30	0.15	0.37	0.12
Q64520	Guanylate kinase OS=Mus musculus GN=Guk1 PE=1 SV=2 - [KGUA_MOUSE]	49.49	3	8	8	48	0.09	0.13	0.00	0.07
Q9Z160	Conserved oligomeric Golgi complex subunit 1 OS=Mus musculus GN=Cog1 PE=2 SV=3 - [COG1_MOUSE]	7.86	6	6	6	23	0.19	0.33	0.44	0.24
Q8VHI6	Wiskott-Aldrich syndrome protein family member 3 OS=Mus musculus GN=Wasf3 PE=2 SV=1 - [WASF3_MOUSE]	2.59	1	1	1	2	-0.35	-0.22	-0.11	0.02
Q9JJA2	Conserved oligomeric Golgi complex subunit 8 OS=Mus musculus GN=Cog8 PE=2 SV=3 - [COG8_MOUSE]	12.81	2	6	6	22	0.31	0.54	0.30	0.48
Q920E5	Farnesyl pyrophosphate synthase OS=Mus musculus GN=Fdps PE=2 SV=1 - [FPPS_MOUSE]	36.26	1	12	12	98	1.15	1.12	1.02	1.09

P97333	Neuropilin-1 OS=Mus musculus GN=Nrp1 PE=1 SV=2 - [NRP1_MOUSE]	4.77	1	2	2	3	-0.04	-0.62	1.27	0.68
Q8BPM0-2	Isoform 2 of Disheveled associated activator of morphogenesis 1 OS=Mus musculus GN=Daam1 - [DAAM1_MOUSE]	7.12	5	4	7	31	-0.04	0.31	0.26	0.62
Q7TSE6	Serine/threonine-protein kinase 38-like OS=Mus musculus GN=Stk38l PE=1 SV=2 - [ST38L_MOUSE]	17.03	2	5	7	9	0.59	0.57	0.41	0.39
Q64287-2	Isoform 2 of Interferon regulatory factor 4 OS=Mus musculus GN=Irf4 - [IRF4_MOUSE]	5.12	2	1	1	1	-0.22	-0.55	0.73	0.40
Q6ZQ38	Cullin-associated NEDD8-dissociated protein 1 OS=Mus musculus GN=Cand1 PE=2 SV=2 - [CAND1_MOUSE]	23.98	2	23	25	143	0.39	0.45	0.30	0.37
D3Z4E3	DEAH (Asp-Glu-Ala-His) box polypeptide 32, isoform CRA_b OS=Mus musculus GN=Dhx32 PE=4 SV=1 - [D3Z4E3_MOUSE]	18.33	3	7	7	16	0.21	0.28	0.54	0.38
Q80WR5	UPF0688 protein C1orf174 homolog OS=Mus musculus PE=1 SV=1 - [CA174_MOUSE]	12.17	2	2	2	4	0.11	0.05	0.44	0.38
P10630	Eukaryotic initiation factor 4A-II OS=Mus musculus GN=Eif4a2 PE=2 SV=2 - [IF4A2_MOUSE]	48.89	6	9	21	365	0.24	0.35	0.14	0.18
O54890	Integrin beta-3 OS=Mus musculus GN=Itgb3 PE=1 SV=2 - [ITB3_MOUSE]	9.78	1	7	7	49	0.04	0.22	-0.15	-0.03
Q8BM55	Transmembrane protein 214 OS=Mus musculus GN=Tmem214 PE=2 SV=1 - [TM214_MOUSE]	16.59	4	12	12	36	0.53	0.47	0.60	0.51
Q8K0C4	Lanosterol 14-alpha demethylase OS=Mus musculus GN=Cyp51a1 PE=2 SV=1 - [CP51A_MOUSE]	16.30	1	6	6	33	0.58	0.77	0.67	0.83
Q8CDU6	Probable E3 ubiquitin-protein ligase HECTD2 OS=Mus musculus GN=Hectd2 PE=2 SV=2 - [HECD2_MOUSE]	1.81	2	1	1	1	0.90	-0.29	0.94	-0.25
Q6RT24	Centromere-associated protein E OS=Mus musculus GN=Cenpe PE=1 SV=1 - [CENPE_MOUSE]	1.98	2	3	4	10	0.39	-0.01	0.56	-0.06
E9Q828	Protein Atp2b4 OS=Mus musculus GN=Atp2b4 PE=2 SV=1 - [E9Q828_MOUSE]	28.75	4	16	27	278	-0.10	-0.19	-0.07	-0.15
Q6PDI5	Proteasome-associated protein ECM29 homolog OS=Mus musculus GN=Ecm29 PE=1 SV=3 - [ECM29_MOUSE]	11.74	7	17	17	71	0.29	0.37	0.33	0.37

Q8VE47	Ubiquitin-like modifier-activating enzyme 5 OS=Mus musculus GN=Uba5 PE=2 SV=2 - [UBA5_MOUSE]	16.38	3	4	4	21	0.64	0.83	0.66	0.72
Q04690-4	Isoform 4 of Neurofibromin OS=Mus musculus GN=Nf1 - [NF1_MOUSE]	1.93	4	2	2	6	0.04	-0.04	-0.13	-0.21
Q3UHH8-2	Isoform 2 of Glucoside xylosyltransferase 1 OS=Mus musculus GN=Gxylt1 - [GXLT1_MOUSE]	8.60	3	2	2	5	-0.18	0.22	0.11	0.42
A2AG06-2	Isoform 2 of Uncharacterized protein C17orf104 homolog OS=Mus musculus GN=Gm1564 - [CQ104_MOUSE]	0.99	2	1	1	1	0.62	0.44	0.63	0.45
E9Q8T7	Protein Dnah1 OS=Mus musculus GN=Dnah1 PE=4 SV=1 - [E9Q8T7_MOUSE]	1.11	1	4	5	8	0.47	0.81	0.41	0.75
P28660-2	Isoform 2 of Nck-associated protein 1 OS=Mus musculus GN=Nckap1 - [NCKP1_MOUSE]	17.36	3	19	20	73	0.30	0.38	0.36	0.44
Q8C0V1-2	Isoform 2 of Coiled-coil domain-containing protein 79 OS=Mus musculus GN=Ccdc79 - [CCD79_MOUSE]	0.99	3	1	1	13	0.13	-0.54	0.59	-0.09
Q9D500	Protein 4930533L02Rik OS=Mus musculus GN=4930533L02Rik PE=2 SV=1 - [Q9D500_MOUSE]	12.90	1	1	1	4	0.20	-1.55	0.28	-1.47
Q8CIV8	Tubulin-specific chaperone E OS=Mus musculus GN=Tbce PE=1 SV=1 - [TBCE_MOUSE]	3.44	2	1	2	5	0.03	0.06	0.23	0.26
Q9WUQ2	Prolactin regulatory element-binding protein OS=Mus musculus GN=Preb PE=1 SV=1 - [PREB_MOUSE]	24.94	3	8	8	24	0.12	0.38	0.21	0.39
A2ARI0	Thioredoxin-related transmembrane protein 4 OS=Mus musculus GN=Tmx4 PE=2 SV=1 [A2ARI0_MOUSE]	14.75	2	2	2	5	0.16	0.33	0.34	0.51
D3YVP5	Glutathione S-transferase Mu 7 OS=Mus musculus GN=Gstm7 PE=2 SV=1 - [D3YVP5_MOUSE]	50.93	6	3	11	230	0.13	0.16	0.13	0.16
E9QL65	Conserved oligomeric Golgi complex subunit 3 OS=Mus musculus GN=Cog3 PE=4 SV=1 - [E9QL65_MOUSE]	16.18	2	11	11	30	0.64	0.21	0.68	0.26
Q8C2E7	WASH complex subunit strumpellin OS=Mus musculus GN=Kiaa0196 PE=2 SV=2 - [STRUM_MOUSE]	8.71	2	9	9	23	0.08	0.20	0.42	0.34

Q9D1D4	Transmembrane emp24 domain-containing protein 10 OS=Mus musculus GN=Tmed10 PE=2 SV=1 - [TMEDA_MOUSE]	35.16	2	6	6	174	0.41	0.45	0.25	0.38
Q6GQR8	Zinc finger protein 329 OS=Mus musculus GN=Znf329 PE=2 SV=2 - [ZN329_MOUSE]	5.94	120	2	3	10	-0.49	-0.18	-0.48	-0.14
P59017	Bcl-2-like protein 13 OS=Mus musculus GN=Bcl2l13 PE=1 SV=2 - [B2L13_MOUSE]	6.68	1	3	3	4	0.25	-0.11	0.13	-0.07
D3Z7V3	Sarcolemmal membrane associated protein OS=Mus musculus GN=Slmap PE=2 SV=1 - [D3Z7V3_MOUSE]	34.43	15	30	32	277	0.23	0.26	0.24	0.21
Q8C3X8	Lipase maturation factor 2 OS=Mus musculus GN=Lmf2 PE=2 SV=1 - [LMF2_MOUSE]	1.57	1	1	1	3	0.10	0.15	0.19	0.24
E9QAE4	Histone-lysine N-methyltransferase, H3 lysine-36 and H4 lysine-20-specific OS=Mus musculus GN=Nsd1 PE=2 SV=1 - [E9QAE4_MOUSE]	1.34	6	1	3	5	0.38	-0.22	0.65	0.04
P09036	Serine protease inhibitor Kazal-type 3 OS=Mus musculus GN=Spink3 PE=1 SV=1 - [ISK3_MOUSE]	25.00	1	2	2	9	1.50	1.51	1.44	1.57
Q03141-2	Isoform 2 of MAP/microtubule affinity-regulating kinase 3 OS=Mus musculus GN=Mark3 - [MARK3_MOUSE]	9.88	4	3	7	15	-0.47	0.66	-0.22	0.31
Q9JI67	Beta-1,3-galactosyltransferase 5 OS=Mus musculus GN=B3galt5 PE=2 SV=1 - [B3GT5_MOUSE]	19.48	1	4	4	22	0.09	0.03	0.04	-0.01
Q62190	Macrophage-stimulating protein receptor OS=Mus musculus GN=Mst1r PE=1 SV=3 [RON_MOUSE]	2.10	1	1	2	5	0.12	0.69	0.38	0.95
Q6P5F9	Exportin-1 OS=Mus musculus GN=Xpo1 PE=1 SV=1 - [XPO1_MOUSE]	15.03	3	11	12	58	0.13	0.32	0.20	0.32
G3UW57	Protein Rab11fip2 OS=Mus musculus GN=Rab11fip2 PE=4 SV=1 - [G3UW57_MOUSE]	3.78	3	2	2	3	-0.37	-0.54	-0.19	-0.36
A2AR60	Protein CASC4 (Fragment) OS=Mus musculus GN=Casc4 PE=2 SV=1 - [A2AR60_MOUSE]	7.87	5	2	2	4	-0.01	0.31	-0.07	0.25
Q6ZWQ5	Sorting nexin 12, isoform CRA_c OS=Mus musculus GN=Snx12 PE=2 SV=1 - [Q6ZWQ5_MOUSE]	35.80	4	5	6	18	0.39	0.48	0.43	0.54
Q9ESJ0	Exportin-4 OS=Mus musculus GN=Xpo4 PE=1 SV=2 - [XPO4_MOUSE]	8.08	2	6	6	19	0.63	0.37	0.76	0.51

P55086	Proteinase-activated receptor 2 OS=Mus musculus GN=F2rl1 PE=1 SV=1 - [PAR2_MOUSE]	3.26	1	1	1	1	0.76	0.95	0.54	0.73
Q8BMK4	Cytoskeleton-associated protein 4 OS=Mus musculus GN=Ckap4 PE=2 SV=2 - [CKAP4_MOUSE]	51.13	1	30	31	229	0.31	0.36	0.33	0.31
B2RT54	Apolipoprotein L 7a OS=Mus musculus GN=Apol7a PE=2 SV=1 - [B2RT54_MOUSE]	3.59	1	1	2	3	-0.04	-0.37	0.27	-0.06
O35400	Sulfotransferase family cytosolic 2B member 1 OS=Mus musculus GN=Sult2b1 PE=2 SV=2 - [ST2B1_MOUSE]	10.65	3	2	5	21	0.28	0.49	0.18	0.41
Q62431	AT-rich interactive domain-containing protein 3A OS=Mus musculus GN=Arid3a PE=1 SV=1 - [ARI3A_MOUSE]	3.33	1	1	2	4	0.97	-0.48	1.05	-0.40
P68134	Actin, alpha skeletal muscle OS=Mus musculus GN=Acta1 PE=1 SV=1 - [ACTS_MOUSE]	72.68	3	2	30	6079	0.19	0.39	0.21	0.36
Q9Z0I6	Protein Slfn2 OS=Mus musculus GN=Slfn2 PE=2 SV=1 - [Q9Z0I6_MOUSE]	5.82	1	1	1	6	0.19	-0.44	0.30	-0.33
Q8VC49	Interferon alpha-inducible protein 27-like protein 2B OS=Mus musculus GN=Ifi27l2b PE=2 SV=1 - [IF27B_MOUSE]	15.90	1	4	4	15	-0.17	-0.19	-0.18	-0.09
Q9CQU3	Protein RER1 OS=Mus musculus GN=Rer1 PE=1 SV=1 - [RER1_MOUSE]	9.18	1	2	2	8	0.67	0.21	0.60	0.25
Q8BW10	RNA-binding protein NOB1 OS=Mus musculus GN=Nob1 PE=1 SV=1 - [NOB1_MOUSE]	4.47	1	1	1	1	0.53	0.79	0.58	0.85
P49135	TFIIH basal transcription factor complex helicase XPB subunit OS=Mus musculus GN=Ercc3 PE=2 SV=1 - [ERCC3_MOUSE]	1.92	1	1	1	2	-0.11	0.22	0.27	0.60
Q8R3Q0	Store-operated calcium entry-associated regulatory factor OS=Mus musculus GN=Tmem66 PE=2 SV=2 - [SARAF_MOUSE]	3.89	1	1	1	3	0.18	0.16	0.57	0.55
Q01341	Adenylate cyclase type 6 OS=Mus musculus GN=Adcy6 PE=1 SV=1 - [ADCY6_MOUSE]	4.89	2	4	5	10	-0.02	0.18	0.02	0.19
P37804	Transgelin OS=Mus musculus GN=Tagln PE=1 SV=3 - [TAGL_MOUSE]	78.11	1	22	23	1615	0.59	0.66	0.55	0.62

Q8BJ64	Choline dehydrogenase, mitochondrial OS=Mus musculus GN=Chdh PE=1 SV=1 - [CHDH_MOUSE]	31.54	5	15	15	58	0.24	0.20	0.24	0.28
P70280	Vesicle-associated membrane protein 7 OS=Mus musculus GN=Vamp7 PE=1 SV=1 - [VAMP7_MOUSE]	11.36	1	3	3	5	0.07	-0.34	0.13	0.13
Q923S9	Ras-related protein Rab-30 OS=Mus musculus GN=Rab30 PE=2 SV=1 - [RAB30_MOUSE]	19.21	12	2	4	30	0.11	0.32	0.23	0.45
Q9CZW4	Long-chain-fatty-acid--CoA ligase 3 OS=Mus musculus GN=Acsl3 PE=2 SV=2 - [ACSL3_MOUSE]	14.86	3	8	9	26	0.27	0.59	0.25	0.59
A2ACP1-2	Isoform 2 of Tetratricopeptide repeat protein 39A OS=Mus musculus GN=Ttc39a - [TT39A_MOUSE]	9.55	2	5	5	18	0.22	0.18	0.17	0.17
E9QLM4	Mismatch repair endonuclease PMS2 OS=Mus musculus GN=Pms2 PE=2 SV=1 - [E9QLM4_MOUSE]	4.12	3	1	1	1	0.26	-1.13	0.18	-1.20
O89051	Integral membrane protein 2B OS=Mus musculus GN=Itm2b PE=2 SV=1 - [ITM2B_MOUSE]	10.53	1	2	2	5	0.48	0.55	0.41	0.57
Q8C863-2	Isoform 2 of E3 ubiquitin-protein ligase Itchy OS=Mus musculus GN=Itch - [ITCH_MOUSE]	5.53	2	3	4	5	-0.11	-0.09	0.23	0.07
Q8VF20	MCG142237 OS=Mus musculus GN=Olfr509 PE=2 SV=1 - [Q8VF20_MOUSE]	2.18	1	1	1	1	0.52	0.26	0.63	0.36
Q60850-4	Isoform 4 of Solute carrier family 23 member 3 OS=Mus musculus GN=Slc23a3 - [S23A3_MOUSE]	21.62	4	1	1	1	-0.78	-0.92	0.00	-0.14
Q9DBG7	Signal recognition particle receptor subunit alpha OS=Mus musculus GN=Srpr PE=1 SV=1 - [SRPR_MOUSE]	12.58	2	7	7	24	0.47	0.26	0.56	0.39
Q3U186	Probable arginine--tRNA ligase, mitochondrial OS=Mus musculus GN=Rars2 PE=2 SV=1 - [SYRM_MOUSE]	14.53	2	8	8	16	-0.13	0.01	0.04	0.11
E9PXM6	Equilibrative nucleoside transporter 1 OS=Mus musculus GN=Slc29a1 PE=2 SV=1 - [E9PXM6_MOUSE]	7.54	11	2	2	5	0.08	-0.35	0.26	-0.17
B8JK39	Protein Itga9 OS=Mus musculus GN=Itga9 PE=2 SV=1 - [B8JK39_MOUSE]	8.88	2	6	6	12	0.26	0.18	0.32	0.18
E0CZE8	Bax inhibitor 1 (Fragment) OS=Mus musculus GN=Tmbim6 PE=2 SV=1 - [E0CZE8_MOUSE]	28.13	4	1	1	1	0.81	0.97	0.77	0.93

Q61738-3	Isoform Alpha-7X1B of Integrin alpha-7 OS=Mus musculus GN=Itga7 - [ITA7_MOUSE]	22.48	7	19	19	104	-0.05	0.05	-0.04	0.04
E9PYT3	Atlastin-3 OS=Mus musculus GN=Atl3 PE=2 SV=1 - [E9PYT3_MOUSE]	32.09	3	12	12	95	0.21	0.24	0.15	0.07
Q9EQG7	Ectonucleotide pyrophosphatase/phosphodiesterase family member 5 OS=Mus musculus GN=Enpp5 PE=2 SV=3 - [ENPP5_MOUSE]	4.19	4	3	3	3	-0.65	-0.53	-0.67	-0.58
O35375-5	Isoform B0 of Neuropilin-2 OS=Mus musculus GN=Nrp2 - [NRP2_MOUSE]	9.10	6	4	4	28	0.06	0.17	0.23	0.10
Q8C1Q4	Meteorin OS=Mus musculus GN=Metrn PE=2 SV=2 - [METRN_MOUSE]	16.15	1	2	2	3	0.63	0.65	0.59	0.60
E9Q174	Unconventional myosin-VI OS=Mus musculus GN=Myo6 PE=2 SV=1 [E9Q174_MOUSE]	39.56	2	1	48	213	0.83	0.86	0.58	0.60
P70121	Zinc fingers and homeoboxes protein 1 OS=Mus musculus GN=Zhx1 PE=1 SV=2 - [ZHX1_MOUSE]	1.37	1	1	1	1	0.42	0.83	-0.76	-0.36
Q8VI94	2'-5'-oligoadenylate synthase-like protein 1 OS=Mus musculus GN=Oasl1 PE=2 SV=1 [OASL1_MOUSE]	9.00	1	3	4	13	0.46	0.47	0.38	0.39
Q8BGE6	Cysteine protease ATG4B OS=Mus musculus GN=Atg4b PE=1 SV=2 - [ATG4B_MOUSE]	8.14	1	2	2	9	0.93	1.15	0.80	1.01
Q6PD03	Serine/threonine-protein phosphatase 2A 56 kDa regulatory subunit alpha isoform OS=Mus musculus GN=Ppp2r5a PE=1 SV=1 - [2A5A_MOUSE]	18.11	1	6	7	21	0.33	0.47	0.35	0.37
Q99MB7	RING finger protein 141 OS=Mus musculus GN=Rnf141 PE=2 SV=2 - [RN141_MOUSE]	24.35	5	2	2	2	-0.35	0.44	0.38	1.17
Q0P543-2	Isoform 2 of Gastric inhibitory polypeptide receptor OS=Mus musculus GN=Gipr - [GIPR_MOUSE]	10.87	1	1	1	1	0.36	0.55	0.57	0.76
Q9D6K5-4	Isoform 4 of Synaptojanin-2-binding protein OS=Mus musculus GN=Synj2bp - [SYJ2B_MOUSE]	28.10	12	4	4	20	-0.08	0.28	-0.18	0.20
Q791V5	Mitochondrial carrier homolog 2 OS=Mus musculus GN=Mtch2 PE=1 SV=1 - [MTCH2_MOUSE]	14.52	3	3	3	22	-0.11	0.11	-0.03	0.08
D3Z6T3	Cathepsin E OS=Mus musculus GN=Ctse PE=2 SV=1 - [D3Z6T3_MOUSE]	4.95	2	2	2	6	0.56	0.82	0.38	0.74
Q8BYA0	Tubulin-specific chaperone D OS=Mus musculus GN=Tbcd PE=2 SV=1 - [TBCD_MOUSE]	4.43	2	5	5	16	0.36	0.70	0.46	0.59

Q6DFV5	Probable helicase with zinc finger domain OS=Mus musculus GN=Helz PE=1 SV=2 - [HELZ_MOUSE]	0.66	2	1	1	1	0.17	-0.77	0.88	-0.06
Q3UNX5	Acyl-coenzyme A synthetase ACSM3, mitochondrial OS=Mus musculus GN=Acsm3 PE=1 SV=2 - [ACSM3_MOUSE]	45.00	3	20	20	144	0.21	0.09	0.14	0.17
Q91Z67	SLIT-ROBO Rho GTPase-activating protein 2 OS=Mus musculus GN=Srgap2 PE=1 SV=2 - [SRGP2_MOUSE]	4.20	8	3	4	8	0.67	-0.16	1.01	0.13
O08547	Vesicle-trafficking protein SEC22b OS=Mus musculus GN=Sec22b PE=1 SV=3 - [SC22B_MOUSE]	34.42	2	6	6	104	0.26	0.21	0.22	0.19
Q9CRA5	Golgi phosphoprotein 3 OS=Mus musculus GN=Golph3 PE=2 SV=1 - [GOLP3_MOUSE]	25.17	2	5	6	45	0.61	0.74	0.64	0.70
Q9JLI2	Collagen type V alpha 3 chain OS=Mus musculus GN=Col5a3 PE=2 SV=1 - [Q9JLI2_MOUSE]	1.09	1	2	2	3	0.48	-0.10	0.72	0.14
Q9JKD3	Secretory carrier-associated membrane protein 5 OS=Mus musculus GN=Scamp5 PE=2 SV=1 - [SCAM5_MOUSE]	4.68	1	1	1	2	0.05	-0.01	0.01	-0.05
Q9WVC8	Chloride anion exchanger OS=Mus musculus GN=Slc26a3 PE=2 SV=1 - [S26A3_MOUSE]	10.17	4	8	8	30	1.17	1.01	0.91	0.87
Q7TSQ9	Component of oligomeric golgi complex 4 OS=Mus musculus GN=Cog4 PE=2 SV=1 - [Q7TSQ9_MOUSE]	8.01	5	3	5	10	0.15	-0.10	0.27	0.02
Q7TS75	APC membrane recruitment protein 1 OS=Mus musculus GN=Amer1 PE=2 SV=3 - [AMER1_MOUSE]	1.24	1	1	1	2	0.22	0.35	0.23	0.35
Q91W34	UPF0420 protein C16orf58 homolog OS=Mus musculus PE=2 SV=1 - [CP058_MOUSE]	13.52	3	4	4	9	0.06	0.30	0.04	0.29
Q9QXK3-4	Isoform 4 of Coatomer subunit gamma-2 OS=Mus musculus GN=Copg2 - [COPG2_MOUSE]	12.30	6	7	10	39	0.52	0.36	0.44	0.03
A2ARJ3	Transmembrane protein 236 OS=Mus musculus GN=Tmem236 PE=3 SV=1 - [TM236_MOUSE]	9.30	1	3	3	6	0.07	-0.01	-0.03	-0.11
B1AXB9	Predicted gene, EG236749 OS=Mus musculus GN=Gm4907 PE=2 SV=1 - [B1AXB9_MOUSE]	16.30	1	2	7	28	-0.12	0.53	0.13	0.58

E9PWK4	Immunoglobulin J chain (Fragment) OS=Mus musculus GN=Igj PE=2 SV=1 - [E9PWK4_MOUSE]	28.57	2	3	3	28	0.43	0.31	0.43	0.39
Q8BU33-3	Isoform 3 of Acetolactate synthase-like protein OS=Mus musculus GN=Ilvbl - [ILVBL_MOUSE]	3.49	3	2	2	4	-0.08	0.01	0.08	0.06
B7ZWL1	CCR4-NOT transcription complex subunit 1 OS=Mus musculus GN=Cnot1 PE=2 SV=1 - [B7ZWL1_MOUSE]	5.20	5	9	9	19	0.07	0.05	0.21	0.26
Q91ZB5	Mas-related G-protein coupled receptor member G OS=Mus musculus GN=Mrgprg PE=2 SV=1 - [MRGRG_MOUSE]	5.54	1	1	1	1	0.28	-2.61	0.53	-2.37
Q3TRP8	Cartilage acidic protein 1 OS=Mus musculus GN=Cep68 PE=2 SV=1 - [Q3TRP8_MOUSE]	2.05	3	1	1	1	-0.05	-0.44	0.50	0.10
Q80YX1-2	Isoform 2 of Tenascin OS=Mus musculus GN=Tnc - [TENA_MOUSE]	25.66	6	41	41	241	0.49	0.48	0.53	0.47
Q4VA53	Sister chromatid cohesion protein PDS5 homolog B OS=Mus musculus GN=Pds5b PE=1 SV=1 - [PDS5B_MOUSE]	9.61	4	11	13	33	0.37	0.50	0.18	0.19
E9PYY7	Carboxypeptidase A5 OS=Mus musculus GN=Cpa5 PE=2 SV=1 - [E9PYY7_MOUSE]	8.41	3	2	2	3	-0.28	0.11	0.00	0.39
Q3TJZ6	Protein FAM98A OS=Mus musculus GN=Fam98a PE=2 SV=1 - [FA98A_MOUSE]	25.44	5	5	8	48	0.07	-0.01	0.25	0.19
F8WIB1	ADP-ribosylation factor like protein 1 (Fragment) OS=Mus musculus GN=Arl1 PE=2 SV=1 - [F8WIB1_MOUSE]	18.12	3	3	3	19	0.09	0.20	0.20	0.21
Q64282	Interferon-induced protein with tetratricopeptide repeats 1 OS=Mus musculus GN=Ifit1 PE=1 SV=2 - [IFIT1_MOUSE]	19.44	1	8	9	21	0.33	0.42	0.21	0.37
A2A3V2	Solute carrier family 25 member 43 OS=Mus musculus GN=Slc25a43 PE=2 SV=1 - [S2543_MOUSE]	4.69	1	1	1	1	0.51	0.25	0.38	0.11
F8VQ05	Protein Fryl OS=Mus musculus GN=Fryl PE=4 SV=1 - [F8VQ05_MOUSE]	2.19	2	5	5	6	0.54	0.64	0.46	0.55
A2A485	Protein Zmynd8 OS=Mus musculus GN=Zmynd8 PE=2 SV=1 - [A2A485_MOUSE]	2.47	8	2	2	3	0.24	-0.01	0.33	0.07
Q64374	Regucalcin OS=Mus musculus GN=Rgn PE=1 SV=1 - [RGN_MOUSE]	5.69	1	1	1	1	-0.10	-0.17	0.15	0.08
Q3TCH7	Cullin-4A OS=Mus musculus GN=Cul4a PE=1 SV=1 - [CUL4A_MOUSE]	10.94	2	3	9	13	0.59	0.59	0.50	0.55

M0QWS4	Ubiquitin-fold modifier conjugating enzyme 1, isoform CRA_c OS=Mus musculus GN=Ufc1 PE=4 SV=1 - [M0QWS4_MOUSE]	37.63	2	5	5	17	0.49	0.65	0.42	0.29
E9QNX9	Tyrosine-protein kinase receptor OS=Mus musculus GN=Igf1r PE=2 SV=1 - [E9QNX9_MOUSE]	3.14	2	3	3	5	-0.31	-0.14	-0.06	0.10
Q91WK7	Ankyrin repeat domain-containing protein 54 OS=Mus musculus GN=Ankrd54 PE=1 SV=1 - [ANR54_MOUSE]	14.38	3	2	2	5	-0.36	-0.02	-0.37	-0.04
E9PUM4	Talin-2 OS=Mus musculus GN=Tln2 PE=4 SV=1 - [E9PUM4_MOUSE]	25.65	3	33	52	343	0.26	0.19	0.26	0.16
D3Z414	Lymphocyte-specific helicase (Fragment) OS=Mus musculus GN=Hells PE=2 SV=1 - [D3Z414_MOUSE]	3.35	3	1	1	1	0.73	0.76	0.41	0.44
Q9Z1K6	E3 ubiquitin-protein ligase ARIH2 OS=Mus musculus GN=Arih2 PE=2 SV=1 - [ARI2_MOUSE]	3.46	1	1	1	3	0.44	0.35	0.34	0.25
Q03173-6	Isoform 6 of Protein enabled homolog OS=Mus musculus GN=Enah - [ENAH_MOUSE]	22.09	10	7	7	23	0.74	0.89	0.62	0.77
O88520	Leucine-rich repeat protein SHOC-2 OS=Mus musculus GN=Shoc2 PE=2 SV=2 - [SHOC2_MOUSE]	8.93	1	3	3	10	0.61	0.49	0.48	0.35
Q62186	Translocon-associated protein subunit delta OS=Mus musculus GN=Ssr4 PE=2 SV=1 - [SSRD_MOUSE]	34.30	2	5	5	49	0.32	0.28	0.40	0.38
Q4FE56	Ubiquitin carboxyl-terminal hydrolase OS=Mus musculus GN=Usp9x PE=2 SV=1 - [Q4FE56_MOUSE]	10.92	4	16	20	62	0.35	0.44	0.31	0.42
Q5DU09-2	Isoform 2 of Zinc finger protein 652 OS=Mus musculus GN=Znf652 - [ZN652_MOUSE]	4.00	3	1	1	1	-0.57	-0.54	0.17	0.20
L7N451	Interferon-induced very large GTPase 1 OS=Mus musculus GN=Gvin1 PE=4 SV=1 - [L7N451_MOUSE]	4.45	5	8	9	31	-0.47	-0.54	-0.52	-0.47
Q8K377	Leucine-rich repeat transmembrane neuronal protein 1 OS=Mus musculus GN=Lrrtm1 PE=2 SV=1 - [LRRT1_MOUSE]	3.64	1	2	2	4	0.28	0.41	0.43	0.48
D3Z1D3	Protein 3425401B19Rik OS=Mus musculus GN=3425401B19Rik PE=4 SV=1 - [D3Z1D3_MOUSE]	1.35	1	1	1	1	-0.38	-0.44	-0.41	-0.47

Q9DAV9	Trimeric intracellular cation channel type B OS=Mus musculus GN=Tmem38b PE=2 SV=1 - [TM38B_MOUSE]	5.82	1	1	1	4	0.31	0.81	0.01	0.51
K7N744	Protein Gm8104 OS=Mus musculus GN=Gm8104 PE=4 SV=1 - [K7N744_MOUSE]	8.08	1	1	1	1	0.87	-1.33	1.12	-1.09
Q8R138	Transmembrane protein 119 OS=Mus musculus GN=Tmem119 PE=2 SV=1 - [TM119_MOUSE]	12.86	1	3	3	8	-0.30	0.00	-0.25	0.08
F6ZED4	Protein Gm9970 OS=Mus musculus GN=Gm9970 PE=4 SV=1 - [F6ZED4_MOUSE]	5.95	1	1	1	3	0.62	0.44	0.60	0.42
Q5PRF0	HEAT repeat-containing protein 5A OS=Mus musculus GN=Heatr5a PE=2 SV=2 - [HTR5A_MOUSE]	0.29	1	1	1	1	0.05	0.58	-0.18	0.36
O89079	Coatomer subunit epsilon OS=Mus musculus GN=Cope PE=2 SV=3 - [COPE_MOUSE]	36.69	5	8	9	73	0.11	0.04	0.15	0.07
Q64727	Vinculin OS=Mus musculus GN=Vcl PE=1 SV=4 - [VINC_MOUSE]	66.79	1	68	68	2169	0.52	0.57	0.52	0.56
O89001	Carboxypeptidase D OS=Mus musculus GN=Cpd PE=1 SV=2 - [CBPD_MOUSE]	15.47	1	16	16	46	0.22	0.20	0.26	0.22
Q8BXA5	Cleft lip and palate transmembrane protein 1-like protein OS=Mus musculus GN=Clptm1l PE=2 SV=1 - [CLP1L_MOUSE]	5.38	1	2	2	4	0.15	0.17	0.16	0.18
P47740	Fatty aldehyde dehydrogenase OS=Mus musculus GN=Aldh3a2 PE=2 SV=2 - [AL3A2_MOUSE]	24.38	3	9	11	87	0.19	0.23	0.17	0.25
Q923T9-3	Isoform 3 of Calcium/calmodulin-dependent protein kinase type II subunit gamma OS=Mus musculus GN=Camk2g [KCC2G_MOUSE]	26.06	14	7	12	70	0.60	0.47	0.57	0.56
Q9JKV5	Secretory carrier-associated membrane protein 4 OS=Mus musculus GN=Scamp4 PE=2 SV=1 - [SCAM4_MOUSE]	4.78	1	1	1	4	0.13	0.11	0.35	0.32
Q6GTM0	Ifit2 protein OS=Mus musculus GN=Ifit2 PE=2 SV=1 - [Q6GTM0_MOUSE]	4.26	4	1	2	6	0.28	0.28	0.54	0.56
D3Z3E2	Glycerophosphodiester phosphodiesterase 1 (Fragment) OS=Mus musculus GN=Gde1 PE=2 SV=1 - [D3Z3E2_MOUSE]	12.14	2	2	2	3	0.33	0.15	0.25	0.06

Q9D136	2-oxoglutarate and iron-dependent oxygenase domain-containing protein 3 OS=Mus musculus GN=Ogfod3 PE=2 SV=1 - [OGFD3_MOUSE]	2.86	1	1	1	2	0.17	0.17	0.28	0.27
F6UD05	A disintegrin and metalloproteinase with thrombospondin motifs 7 (Fragment) OS=Mus musculus GN=Adamts7 PE=4 SV=1 - [F6UD05_MOUSE]	1.17	4	1	1	1	0.12	0.55	0.16	0.59
Q8C0L8	Conserved oligomeric Golgi complex subunit 5 OS=Mus musculus GN=Cog5 PE=2 SV=3 - [COG5_MOUSE]	9.29	1	7	7	17	0.21	0.02	0.43	0.16
D3YW03	Steroid hormone receptor ERR2 OS=Mus musculus GN=Esrrb PE=2 SV=2 - [D3YW03_MOUSE]	5.82	4	2	2	3	0.10	0.26	-0.02	0.27
Q3UMC0	Spermatogenesis-associated protein 5 OS=Mus musculus GN=Spata5 PE=2 SV=2 - [SPAT5_MOUSE]	4.70	6	2	4	35	-1.08	-1.09	0.00	-0.24
F8WHL1	Zinc transporter 9 OS=Mus musculus GN=Slc30a9 PE=2 SV=1 - [F8WHL1_MOUSE]	1.10	3	1	1	1	-0.21	-0.10	0.14	0.25
Q64695	Endothelial protein C receptor OS=Mus musculus GN=Procr PE=2 SV=3 - [EPCR_MOUSE]	5.37	1	1	1	2	-0.28	0.15	-0.25	0.18
Q922Q4	Pyrroline-5-carboxylate reductase 2 OS=Mus musculus GN=Pycr2 PE=2 SV=1 - [P5CR2_MOUSE]	22.50	1	5	6	18	0.16	0.12	0.13	0.04
Q8BXK9	Chloride intracellular channel protein 5 OS=Mus musculus GN=Clic5 PE=1 SV=1 - [CLIC5_MOUSE]	37.45	1	4	6	49	0.03	0.07	0.06	0.11
Q5SSP3	Neutral amino acid transporter A OS=Mus musculus GN=Slc1a4 PE=2 SV=1 - [Q5SSP3_MOUSE]	5.97	2	1	1	6	0.08	0.30	0.27	0.50
Q8BH59	Calcium-binding mitochondrial carrier protein Aralar1 OS=Mus musculus GN=Slc25a12 PE=1 SV=1 - [CMC1_MOUSE]	33.38	2	15	20	142	0.13	0.13	0.12	0.15
P08113	Endoplasmin OS=Mus musculus GN=Hsp90b1 PE=1 SV=2 - [ENPL_MOUSE]	55.74	2	44	46	1394	0.73	0.76	0.69	0.72
Q2KHK6	Gasdermin-C2 OS=Mus musculus GN=Gsdmc2 PE=2 SV=2 - [GSDC2_MOUSE]	40.83	4	8	20	142	1.69	1.41	1.65	1.41
Q8R1V4	Transmembrane emp24 domain-containing protein 4 OS=Mus musculus GN=Tmed4 PE=2 SV=1 - [TMED4_MOUSE]	25.11	2	4	6	93	0.25	0.38	0.11	0.29

Q3TDQ1	Dolichyl-diphosphooligosaccharide--protein glycosyltransferase subunit STT3B OS=Mus musculus GN=Stt3b PE=1 SV=2 - [STT3B_MOUSE]	9.84	1	7	7	36	0.02	-0.10	0.12	0.05
Q99K73	Nrf1 protein OS=Mus musculus GN=Nrf1 PE=2 SV=1 - [Q99K73_MOUSE]	3.93	7	1	2	11	0.40	0.31	0.44	0.35
P30306	M-phase inducer phosphatase 2 OS=Mus musculus GN=Cdc25b PE=2 SV=1 - [MPIP2_MOUSE]	6.60	2	2	2	3	-0.22	-0.28	0.55	0.49
Q64735-2	Isoform 2 of Complement component receptor 1-like protein OS=Mus musculus GN=Cr1l - [CR1L_MOUSE]	17.27	2	7	7	21	0.44	0.55	0.40	0.56
Q9CQA9	Cancer-related nucleoside-triphosphatase homolog OS=Mus musculus GN=Ntpcr PE=2 SV=1 - [NTPCR_MOUSE]	11.05	3	2	2	2	0.62	-0.12	0.56	-0.19
Q9CPP6	NADH dehydrogenase [ubiquinone] 1 alpha subcomplex subunit 5 OS=Mus musculus GN=Ndufa5 PE=1 SV=3 - [NDUA5_MOUSE]	65.52	1	4	6	84	0.25	0.40	0.06	0.25
Q80Y55	BSD domain-containing protein 1 OS=Mus musculus GN=Bsdc1 PE=2 SV=1 - [BSDC1_MOUSE]	11.24	1	4	4	15	0.14	0.57	0.20	0.63
Q91YQ5	Dolichyl-diphosphooligosaccharide--protein glycosyltransferase subunit 1 OS=Mus musculus GN=Rpn1 PE=2 SV=1 - [RPN1_MOUSE]	56.25	1	27	28	519	0.39	0.44	0.36	0.44
Q91YN9	BAG family molecular chaperone regulator 2 OS=Mus musculus GN=Bag2 PE=1 SV=1 - [BAG2_MOUSE]	53.33	5	10	10	43	0.28	0.26	0.35	0.34
O35566	CD151 antigen OS=Mus musculus GN=Cd151 PE=2 SV=2 - [CD151_MOUSE]	9.09	1	2	2	2	0.40	0.58	0.31	0.49
Q80XL6	Acyl-CoA dehydrogenase family member 11 OS=Mus musculus GN=Acad11 PE=1 SV=2 - [ACD11_MOUSE]	15.66	3	10	11	38	0.21	-0.29	0.44	-0.04
Q8BRM2	RAB6-interacting golgin OS=Mus musculus GN=Gorab PE=1 SV=1 - [GORAB_MOUSE]	6.79	2	2	2	11	0.20	-0.60	0.40	-0.32
Q921N6-2	Isoform 2 of Probable ATP-dependent RNA helicase DDX27 OS=Mus musculus GN=Ddx27 - [DDX27_MOUSE]	3.77	3	1	1	1	0.33	-0.07	1.15	0.74

P42859-2	Isoform Short of Huntingtin OS=Mus musculus GN=Htt - [HD_MOUSE]	1.89	3	6	6	8	0.06	-0.11	0.11	0.10
Q99MZ6	Unconventional myosin VIIb OS=Mus musculu GN=Myo7b PE=1 SV=3 - [MYO7B_MOUSE]	16.85	1	30	32	82	0.29	0.01	0.27	0.16
Q8VDJ3	Vigilin OS=Mus musculus GN=Hdlbp PE=1 SV=1 - [VIGLN_MOUSE]	53.94	2	57	58	423	0.52	0.57	0.49	0.54
B9EJ54	MCG21756, isoform CRA_b OS=Mus musculus GN=Nup205 PE=2 SV=1 - [B9EJ54_MOUSE]	5.08	2	7	9	22	0.37	0.32	0.24	0.19
Q99J77	N-acetylneuraminic acid synthase (Sialic acid synthase) OS=Mus musculus GN=Nans PE=2 SV=1 - [Q99J77_MOUSE]	49.86	1	16	16	410	0.27	0.32	0.18	0.23
Q9CQH0	PDZK1-interacting protein 1 OS=Mus musculus GN=Pdzk1ip1 PE=2 SV=1 - [PDZ1I_MOUSE]	9.65	2	1	1	4	0.36	0.03	0.29	-0.04
Q9ERS2	NADH dehydrogenase [ubiquinone] 1 alpha subcomplex subunit 13 OS=Mus musculus GN=Ndufa13 PE=1 SV=3 - [NDUAD_MOUSE]	51.39	1	7	7	104	0.12	0.20	0.01	0.10
Q571I4	Tyrosine-protein kinase SgK223 OS=Mus musculus GN=Sgk223 PE=2 SV=2 - [SG223_MOUSE]	9.08	2	5	5	6	0.16	0.21	0.05	0.21
O88712-3	Isoform 3 of C-terminal-binding protein 1 OS=Mus musculus GN=Ctbp1 - [CTBP1_MOUSE]	19.89	1	3	7	36	-0.02	-0.02	0.12	-0.03
Q8CGY7	Protein Cd163l1 (Precursor) OS=Mus musculus GN=Cd163l1 PE=2 SV=1 - [Q8CGY7_MOUSE]	2.73	1	1	1	1	0.41	0.98	0.09	0.66
E9PYS3	Protein Adam3 OS=Mus musculus GN=Adam3 PE=2 SV=1 - [E9PYS3_MOUSE]	26.15	1	1	1	1	0.47	0.89	0.34	0.75
Q9JK83-2	Isoform 2 of Partitioning defective 6 homolog beta OS=Mus musculus GN=Pard6b - [PAR6B_MOUSE]	4.70	2	1	1	1	0.05	-0.31	0.54	0.18
D3Z6W2	Tyrosine-protein phosphatase non-receptor type OS=Mus musculus GN=Ptpn2 PE=2 SV=1 - [D3Z6W2_MOUSE]	5.23	3	1	1	2	0.16	0.18	0.57	0.58
P59113	Fermitin family homolog 1 OS=Mus musculus GN=Fermt1 PE=1 SV=4 - [FERM1_MOUSE]	7.68	1	3	5	7	0.53	0.32	0.61	0.39
D3Z7C5	Ski-like protein OS=Mus musculus GN=Skil PE=2 SV=1 - [D3Z7C5_MOUSE]	2.00	3	1	1	3	0.40	0.32	0.39	0.51

O89114	DnaJ homolog subfamily B member 5 OS=Mus musculus GN=Dnajb5 PE=2 SV=1 - [DNJB5_MOUSE]	29.31	2	9	9	25	0.47	0.54	0.38	0.47
A2AQ99	Protein Duox2 OS=Mus musculus GN=Duox2 PE=4 SV=1 - [A2AQ99_MOUSE]	8.57	1	11	13	36	0.45	0.65	0.55	0.63
A2A8S2	Protein YIPF1 (Fragment) OS=Mus musculus GN=Yipf1 PE=2 SV=1 - [A2A8S2_MOUSE]	3.69	3	1	1	4	0.43	0.45	0.29	0.31
M0QWG8	CAS1 domain-containing protein 1 (Fragment) OS=Mus musculus GN=Casd1 PE=4 SV=1 - [M0QWG8_MOUSE]	7.21	3	3	3	4	0.30	0.17	0.18	0.04
Q8BX02	KN motif and ankyrin repeat domain-containing protein 2 OS=Mus musculus GN=Kank2 PE=1 SV=1 - [KANK2_MOUSE]	44.84	7	24	25	172	0.17	0.21	0.15	0.22
D3YVM8	Biogenesis of lysosome-related organelles complex 1 subunit 1 OS=Mus musculus GN=Bloc1s1 PE=2 SV=1 - [D3YVM8_MOUSE]	18.18	3	2	2	5	-0.04	-0.27	0.04	-0.33
Q3UR39	Rab proteins geranylgeranyltransferase component A 1 OS=Mus musculus GN=Chm PE=2 SV=1 - [Q3UR39_MOUSE]	2.09	3	1	1	1	0.54	0.95	0.55	0.96
Q8VC19	5-aminolevulinate synthase, nonspecific, mitochondrial OS=Mus musculus GN=Alas1 PE=2 SV=2 - [HEM1_MOUSE]	3.27	1	1	1	1	0.24	-0.88	1.14	0.02
F6TVP2	Deoxynucleoside triphosphate triphosphohydrolase SAMHD1 (Fragment) OS=Mus musculus GN=Samhd1 PE=4 SV=1 - [F6TVP2_MOUSE]	42.16	4	1	22	278	0.08	-0.62	0.15	-0.55
P24610	Paired box protein Pax-3 OS=Mus musculus GN=Pax3 PE=1 SV=2 - [PAX3_MOUSE]	1.88	2	1	1	1	-0.84	-1.00	-0.62	-0.78
A2AG36	GPI ethanolamine phosphate transferase 3 OS=Mus musculus GN=Pigo PE=2 SV=1 - [A2AG36_MOUSE]	2.53	4	2	2	4	0.30	0.48	-0.15	0.02
Q80WG5	Leucine-rich repeat-containing protein 8A OS=Mus musculus GN=Lrrc8a PE=1 SV=1 - [LRC8A_MOUSE]	2.10	1	1	1	1	0.58	0.57	0.31	0.29
Q3TJ55	Syntaxin-2 OS=Mus musculus GN=Stx2 PE=2 SV=1 - [Q3TJ55_MOUSE]	7.29	4	1	2	2	0.20	0.14	0.65	0.58
A2BDP0	Mediator of RNA polymerase II transcription subunit 14 OS=Mus musculus GN=Med14 PE=2 SV=1 - [A2BDP0_MOUSE]	4.39	2	2	2	4	0.39	0.58	-0.09	0.11

Q6IFZ6	Keratin, type II cytoskeletal 1b OS=Mus musculus GN=Krt77 PE=1 SV=1 [K2C1B_MOUSE]	11.71	1	2	9	107	1.36	1.36	-0.25	-0.24
G5E8T3	Signal recognition particle 19 kDa protein OS=Mus musculus GN=Srp19 PE=4 SV=1 [G5E8T3_MOUSE]	27.50	3	2	2	3	0.63	0.93	0.38	0.67
O70572	Sphingomyelin phosphodiesterase 2 OS=Mus musculus GN=Smpd2 PE=1 SV=1 - [NSMA_MOUSE]	10.26	1	4	4	16	-0.02	-0.10	0.25	0.00
F2Z3X3	CUE domain-containing protein 1 OS=Mus musculus GN=Cuedc1 PE=2 SV=1 - [F2Z3X3_MOUSE]	6.65	6	2	2	5	-0.23	-0.14	0.40	0.50
Q9CZP3	Protein Zfp655 OS=Mus musculus GN=Zfp655 PE=2 SV=1 - [Q9CZP3_MOUSE]	1.85	1	1	1	1	-0.43	-0.81	-0.12	-0.50
D3Z0T0	Transcription initiation factor TFIID subunit 6 OS=Mus musculus GN=Taf6 PE=2 SV=1 - [D3Z0T0_MOUSE]	1.89	2	1	1	2	0.30	0.06	0.25	0.00
Q99P58	Ras-related protein Rab-27B OS=Mus musculus GN=Rab27b PE=1 SV=3 - [RB27B_MOUSE]	27.98	1	5	5	12	0.17	0.24	0.31	0.25
E9PY03	Protein Tstd1 OS=Mus musculus GN=Tstd1 PE=4 SV=1 - [E9PY03_MOUSE]	9.02	1	2	2	8	0.30	0.40	0.33	0.38
A2RTH5	Leucine carboxyl methyltransferase 1 OS=Mus musculus GN=Lcmt1 PE=2 SV=1 - [A2RTH5_MOUSE]	11.45	1	4	4	12	0.33	0.13	0.52	0.32
P61804	Dolichyl-diphosphooligosaccharide--protein glycosyltransferase subunit DAD1 OS=Mus musculus GN=Dad1 PE=2 SV=3 - [DAD1_MOUSE]	19.47	1	2	2	52	0.10	-0.03	0.22	0.06
Q7TPR4	Alpha-actinin-1 OS=Mus musculus GN=Actn1 PE=1 SV=1 [ACTN1_MOUSE]	59.64	1	2	51	1871	0.31	0.25	0.18	0.10
A2A5G4	WAP four-disulfide core domain protein 2 OS=Mus musculus GN=Wfdc2 PE=2 SV=1 - [A2A5G4_MOUSE]	24.32	3	2	2	4	0.23	-0.04	0.25	-0.02
Q5SV80	Unconventional myosin XIX OS=Mus musculu GN=Myo19 PE=2 SV=1 - [MYO19_MOUSE]	4.47	2	3	3	3	0.14	-0.16	0.69	0.39
P61290	Proteasome activator complex subunit 3 OS=Mus musculus GN=Psme3 PE=1 SV=1 - [PSME3_MOUSE]	33.07	3	9	9	56	0.35	0.43	0.34	0.46

D3Z6Y5	Fanconi anemia group A protein homolog OS=Mus musculus GN=Fanca PE=2 SV=1 [D3Z6Y5_MOUSE]	1.46	2	1	1	2	-0.79	-0.90	-0.62	-0.74		
E9Q0B5	Protein Fcgbp OS=Mus musculus GN=Fcgbp PE=2 SV=1 - [E9Q0B5_MOUSE]	16.96	2	31	39	552	1.14	1.13	1.13	1.10		
Q8K224	N-acetyltransferase 10 OS=Mus musculus GN=Nat10 PE=2 SV=1 [NAT10_MOUSE]	2.73	3	3	3	9	0.18	0.21	0.27	0.20		
Q8CIB5	Fermitin family homolog 2 OS=Mus musculus GN=Fermt2 PE=1 SV=1 - [FERM2_MOUSE]	54.26	2	20	30	260	0.39	0.39	0.43	0.43		
Q4FZG9	NADH dehydrogenase [ubiquinone] 1 alpha subcomplex subunit 4-like 2 OS=Mus musculus GN=Ndufa4l2 PE=3 SV=1 - [NUA4L_MOUSE]	27.59	1	1	1	1	-0.10	-0.03	-0.30	-0.23		
Q148R4	Protein Spink5 OS=Mus musculus GN=Spink5 PE=2 SV=1 - [Q148R4_MOUSE]	1.28	1	1	1	1	-0.09	0.03	-0.08	0.04		
Q62425	NADH dehydrogenase [ubiquinone] 1 alpha subcomplex subunit 4 OS=Mus musculus GN=Ndufa4 PE=1 SV=2 - [NDUA4_MOUSE]	56.10	1	6	6	133	-0.06	0.04	-0.09	0.11		
Q3UKU1	RNA polymerase II elongation factor ELL2 OS=Mus musculus GN=Ell2 PE=2 SV=2 - [ELL2_MOUSE]	1.10	1	1	1	2	1.39	1.45	1.65	1.72		
P70168	Importin subunit beta-1 OS=Mus musculus GN=Kpnb1 PE=1 SV=2 - [IMB1_MOUSE]	26.26	1	20	20	244	0.38	0.37	0.41	0.39		
A7L9Z8	Calcium-transporting ATPase type 2C member 2 OS=Mus musculus GN=Atp2c2 PE=2 SV=1 - [AT2C2_MOUSE]	5.83	1	3	4	6	0.29	-0.05	0.49	0.15		
Q9QY81	Nuclear pore membrane glycoprotein 210 OS=Mus musculus GN=Nup210 PE=1 SV=2 - [PO210_MOUSE]	7.53	2	8	8	15	0.22	0.19	0.22	0.25		
E9Q5G3	Protein Kif23 OS=Mus musculus GN=Kif23 PE=2 SV=1 - [E9Q5G3_MOUSE]	3.15	1	2	2	2	-0.29	-0.02	0.45	0.71		
Q78JE5	F-box only protein 22 OS=Mus musculus GN=Fbxo22 PE=2 SV=2 - [FBX22_MOUSE]	8.21	4	3	3	10	0.55	0.36	0.70	0.51		
Q8BM39-2	Isoform 2 of Pre-mRNA splicing factor 18 OS=Mus musculus GN=Prpf18 - [PRP18_MOUSE]	6.86	2	1	1	2	0.11	0.19	0.66	0.73		

G5E8Q0	Lysosomal trafficking regulator, isoform CRA_a OS=Mus musculus GN=Lyst PE=4 SV=1 - [G5E8Q0_MOUSE]	0.40	2	1	1	15	0.59	0.48	0.01	-0.10
Q91ZE6	Beta-spectrin 4 OS=Mus musculus GN=Sptbn4 PE=2 SV=1 - [Q91ZE6_MOUSE]	2.35	4	2	5	22	0.10	-0.09	0.44	-0.08
B7ZNG0-2	Isoform 2 of Kinesin-like protein KIF7 OS=Mus musculus GN=Kif7 - [KIF7_MOUSE]	3.93	4	2	2	5	0.33	0.16	0.50	0.33
Q3U6Q4-2	Isoform 2 of Phosphoinositide 3-kinase regulatory subunit 6 OS=Mus musculus GN=Pik3r6 - [PI3R6_MOUSE]	9.44	3	4	4	23	-0.16	-0.07	-0.06	0.15
Q99KF1	Transmembrane emp24 domain-containing protein 9 OS=Mus musculus GN=Tmed9 PE=2 SV=2 - [TMED9_MOUSE]	31.49	1	8	10	168	0.43	0.52	0.41	0.47
Q8R2R3	Alpha- and gamma-adaptin-binding protein p34 OS=Mus musculus GN=Aagab PE=2 SV=2 - [AAGAB_MOUSE]	31.01	1	3	3	5	-0.12	0.56	-0.04	0.64
P35385	Heat shock protein beta-7 OS=Mus musculus GN=Hspb7 PE=1 SV=3 - [HSPB7_MOUSE]	48.52	1	6	6	45	0.32	-0.10	0.31	-0.08
Q8VHE6	Dynein heavy chain 5, axonemal OS=Mus musculus GN=Dnah5 PE=1 SV=2 - [DYH5_MOUSE]	0.95	1	3	4	4	0.28	0.51	0.35	0.57
P07901	Heat shock protein HSP 90-alpha OS=Mus musculus GN=Hsp90aa1 PE=1 SV=4 - [HS90A_MOUSE]	46.93	8	21	38	982	0.58	0.63	0.54	0.61
Q80UJ7	Rab3 GTPase-activating protein catalytic subunit OS=Mus musculus GN=Rab3gap1 PE=2 SV=4 - [RB3GP_MOUSE]	6.83	1	6	6	12	0.16	0.27	0.18	0.30
Q7TMK6	Protein Hook homolog 2 OS=Mus musculus GN=Hook2 PE=1 SV=3 - [HOOK2_MOUSE]	6.28	1	3	5	32	0.49	0.91	0.39	0.58
D3Z460	Protein Gm1330 OS=Mus musculus GN=Gm1330 PE=2 SV=1 - [D3Z460_MOUSE]	8.70	2	1	1	1	0.37	-0.01	0.87	0.49
Q5U405	Transmembrane protease serine 13 OS=Mus musculus GN=Tmprss13 PE=2 SV=2 - [TMPSD_MOUSE]	1.47	2	1	1	85	-0.21	0.05	-0.40	-0.14
G3X9V0	MCG22048, isoform CRA_a OS=Mus musculus GN=Psme2 PE=2 SV=1 - [G3X9V0_MOUSE]	38.53	3	8	8	78	0.32	0.43	0.34	0.46
A2ALN0	C-C motif chemokine 22 OS=Mus musculus GN=Abcd1 PE=2 SV=1 - [A2ALN0_MOUSE]	10.52	2	3	3	5	-0.27	-0.43	-0.11	-0.27

E9Q6J5	Protein Bod1l OS=Mus musculus GN=Bod1l PE=2 SV=1 - [E9Q6J5_MOUSE]	3.53	1	5	5	7	0.40	0.54	0.51	0.65
P00405	Cytochrome c oxidase subunit 2 OS=Mus musculus GN=Mtco2 PE=1 SV=1 - [COX2_MOUSE]	23.79	1	5	5	73	-0.07	-0.07	-0.04	-0.01
Q9CYN2	Signal peptidase complex subunit 2 OS=Mus musculus GN=Spcs2 PE=2 SV=1 [SPCS2_MOUSE]	22.57	1	5	5	32	0.41	0.13	0.40	0.01
O70622-2	Isoform 2 of Reticulon-2 OS=Mus musculus GN=Rtn2 - [RTN2_MOUSE]	12.25	2	2	2	4	0.06	0.19	0.05	-0.09
Q91YT8	Transmembrane protein 63A OS=Mus musculus GN=Tmem63a PE=2 SV=1 - [TM63A_MOUSE]	4.48	3	3	3	10	-0.02	-0.04	-0.01	-0.07
P31245	Homeobox protein Hox-A2 OS=Mus musculus GN=Hoxa2 PE=2 SV=1 - [HXA2_MOUSE]	8.60	1	1	1	1	1.10	0.17	0.50	-0.43
Q69Z37	Sterile alpha motif domain-containing protein 9-like OS=Mus musculus GN=Samd9l PE=2 SV=2 - [SAM9L_MOUSE]	8.14	3	13	13	24	0.42	0.10	0.37	0.40
Q9EPK7-2	Isoform 2 of Exportin-7 OS=Mus musculus GN=Xpo7 - [XPO7_MOUSE]	11.73	3	8	8	18	0.46	0.30	0.29	0.20
D3Z3H4	Cingulin-like protein 1 OS=Mus musculus GN=Cgnl1 PE=2 SV=1 [D3Z3H4_MOUSE]	4.49	8	4	6	12	0.06	0.24	0.30	0.47
Q9CWF2	Tubulin beta-2B chain OS=Mus musculus GN=Tubb2b PE=1 SV=1 - [TBB2B_MOUSE]	69.44	2	1	21	992	-0.15	0.14	-0.25	-0.04
P02468	Laminin subunit gamma 1 OS=Mus musculus GN=Lamc1 PE=1 SV=2 - [LAMC1_MOUSE]	33.17	3	1	45	509	0.18	0.13	0.25	0.19
Q6PIC6	Sodium/potassium-transporting ATPase subunit alpha-3 OS=Mus musculus GN=Atp1a3 PE=1 SV=1 - [AT1A3_MOUSE]	23.30	2	2	20	468	-0.08	0.17	0.37	0.61
Q3UTJ2	Sorbin and SH3 domain containing protein 2 OS=Mus musculus GN=Sorbs2 PE=1 SV=2 - [SRBS2_MOUSE]	29.92	12	1	29	210	0.09	0.30	0.05	0.20
B7ZNL3	Tpm1 protein OS=Mus musculus GN=Tpm1 PE=2 SV=1 - [B7ZNL3_MOUSE]	75.35	1	4	36	2553	0.10	0.09	0.19	0.18
Q64151	Semaphorin-4C OS=Mus musculus GN=Sema4c PE=1 SV=1 - [SEM4C_MOUSE]	3.48	1	2	2	5	0.15	0.42	0.10	0.36

A2AGT5-2	Isoform 2 of Cytoskeleton-associated protein 5 OS=Mus musculus GN=Ckap5 - [CKAP5_MOUSE]	11.87	4	20	20	42	0.49	0.31	0.52	0.33
J3QP43	Protein 6330403A02Rik OS=Mus musculus GN=6330403A02Rik PE=4 SV=1 - [J3QP43_MOUSE]	21.28	2	2	2	16	0.14	0.13	-0.05	0.16
Q8CG71	Prolyl 3-hydroxylase 2 OS=Mus musculus GN=Leprel1 PE=2 SV=1 - [P3H2_MOUSE]	3.98	1	2	2	15	0.58	0.51	0.44	0.37
Q06335	Amyloid-like protein 2 OS=Mus musculus GN=Aplp2 PE=1 SV=4 [APLP2_MOUSE]	6.08	3	2	4	8	-0.26	-0.43	-0.07	-0.24
D3Z451	MCG54087 OS=Mus musculus GN=Serpina3j PE=3 SV=1 - [D3Z451_MOUSE]	6.19	1	1	2	2	0.10	-0.05	0.43	0.29
Q62159	Rho-related GTP-binding protein RhoC OS=Mus musculus GN=Rhoc PE=1 SV=2 - [RHOC_MOUSE]	40.93	3	1	6	136	0.51	0.68	0.72	0.88
Q8CIM8	Integrator complex subunit 4 OS=Mus musculus GN=Ints4 PE=2 SV=1 - [INT4_MOUSE]	7.05	3	4	4	8	-0.06	-0.16	0.37	0.52
Q9EPS3	D-glucuronyl C5-epimerase OS=Mus musculus GN=Glce PE=1 SV=2 - [GLCE_MOUSE]	1.29	1	1	1	4	0.41	0.77	0.46	0.70
Q8BZ20	Poly [ADP-ribose] polymerase 12 OS=Mus musculus GN=Parp12 PE=2 SV=3 - [PAR12_MOUSE]	14.77	1	7	7	14	0.58	0.38	0.81	0.53
E9Q3Y4	Lipopolysaccharide-responsive and beige-like anchor protein OS=Mus musculus GN=Lrba PE=2 SV=1 - [E9Q3Y4_MOUSE]	9.50	4	19	23	66	0.34	0.08	0.41	0.25
G3X928	SEC23-interacting protein OS=Mus musculus GN=Sec23ip PE=4 SV=1 - [G3X928_MOUSE]	21.04	2	18	19	72	0.36	0.50	0.31	0.35
Q4VAD4	Zfp92 protein OS=Mus musculus GN=Zfp92 PE=2 SV=1 - [Q4VAD4_MOUSE]	3.73	2	1	1	3	-0.48	-0.63	-0.03	-0.18
Q5DU37	Zinc finger FYVE domain-containing protein 26 OS=Mus musculus GN=Zfyve26 PE=2 SV=2 - [ZFY26_MOUSE]	1.74	1	2	2	3	-0.37	0.14	-0.40	0.10
Q6PAM0	5'-AMP-activated protein kinase subunit beta-2 OS=Mus musculus GN=Prkab2 PE=1 SV=1 - [AAKB2_MOUSE]	10.70	2	2	2	5	0.80	-0.22	0.92	0.08
Q91V89	Protein Ppp2r5d OS=Mus musculus GN=Ppp2r5d PE=2 SV=1 - [Q91V89_MOUSE]	17.51	1	5	10	20	0.59	0.45	0.40	0.61

Q9WUK4	Replication factor C subunit 2 OS=Mus musculus GN=Rfc2 PE=2 SV=1 - [RFC2_MOUSE]	8.88	1	2	2	2	0.16	0.42	0.24	0.38
Q925N0	Sideroflexin-5 OS=Mus musculus GN=Sfxn5 PE=1 SV=2 - [SFXN5_MOUSE]	4.68	2	2	2	2	-0.04	-0.26	-0.06	-0.24
B8JJ84	Syntaxin-binding protein 6 (Fragment) OS=Mus musculus GN=Stxbp6 PE=2 SV=1 - [B8JJ84_MOUSE]	19.10	4	2	2	3	0.31	0.55	0.41	0.65
Q9D6R2	Isocitrate dehydrogenase [NAD] subunit alpha, mitochondrial OS=Mus musculus GN=Idh3a PE=1 SV=1 - [IDH3A_MOUSE]	41.26	2	15	15	271	0.23	0.22	0.23	0.21
Q8BJY1	26S proteasome non-ATPase regulatory subunit 5 OS=Mus musculus GN=Psmd5 PE=1 SV=4 - [PSMD5_MOUSE]	24.21	2	10	10	30	0.09	-0.01	0.22	0.05
Q8BMG7	Rab3 GTPase-activating protein non-catalytic subunit OS=Mus musculus GN=Rab3gap2 PE=1 SV=2 - [RBGPR_MOUSE]	3.37	2	4	4	5	0.67	-0.01	0.78	-0.22
Q3TTY5	Keratin, type II cytoskeletal 2 epidermal OS=Mus musculus GN=Krt2 PE=1 SV=1 - [K22E_MOUSE]	14.57	1	4	6	49	1.18	1.13	-0.12	-0.17
Q9CY50	Translocon-associated protein subunit alpha OS=Mus musculus GN=Ssr1 PE=1 SV=1 - [SSRA_MOUSE]	19.58	1	3	3	75	0.44	0.71	0.38	0.67
P01843	Ig lambda-1 chain C region OS=Mus musculus PE=1 SV=1 - [LAC1_MOUSE]	16.19	1	1	1	11	1.90	2.12	1.86	2.13
Q9D9J3	Actin-related protein T1 OS=Mus musculus GN=Actrt1 PE=2 SV=1 - [ACTT1_MOUSE]	1.60	1	1	1	2	0.48	0.58	0.40	0.49
Q8VCL2	Protein SCO2 homolog mitochondrial OS=Mus musculus GN=Sco2 PE=2 SV=1 - [SCO2_MOUSE]	14.90	1	2	2	2	-0.32	-0.24	0.24	0.32
Q6A009	E3 ubiquitin-protein ligase listerin OS=Mus musculus GN=Ltn1 PE=1 SV=3 - [LTN1_MOUSE]	2.38	1	2	4	4	0.02	-0.08	0.12	0.03
E9PY90	cTAGE family member 5 OS=Mus musculus GN=Ctage5 PE=2 SV=1 - [E9PY90_MOUSE]	14.90	13	8	8	20	-0.05	-0.02	-0.17	0.19
Q6PA06-2	Isoform 2 of Atlastin-2 OS=Mus musculus GN=Atl2 - [ATLA2_MOUSE]	19.33	3	6	6	10	-0.18	-0.11	0.70	0.81
A2AN08-3	Isoform 3 of E3 ubiquitin-protein ligase UBR4 OS=Mus musculus GN=Ubr4 - [UBR4_MOUSE]	10.07	7	37	39	90	0.29	0.25	0.23	0.19

Q9EP69	Phosphatidylinositide phosphatase SAC1 OS=Mus musculus GN=Sacm1l PE=2 SV=1 - [SAC1_MOUSE]	23.51	1	13	13	56	0.17	0.14	0.06	0.18
E9PWR7	Ecotropic viral integration site 5 protein (Fragment) OS=Mus musculus GN=Evi5 PE=2 SV=1 - [E9PWR7_MOUSE]	2.88	1	1	1	4	-0.50	-0.15	-0.29	0.06
Q68FF0-2	Isoform 2 of Uncharacterized protein KIAA1841 OS=Mus musculus GN=Kiaa1841 - [K1841_MOUSE]	1.05	3	1	1	1	0.23	0.51	0.04	0.32
Q99PL6-2	Isoform 2 of UBX domain-containing protein 6 OS=Mus musculus GN=Ubxn6 - [UBXN6_MOUSE]	9.77	2	2	2	12	0.12	0.28	0.56	0.40
G5E8C4	MCG142017, isoform CRA_a OS=Mus musculus GN=Tmtc3 PE=4 SV=1 - [G5E8C4_MOUSE]	4.35	3	3	4	15	0.16	0.12	0.24	0.13
Q8BU14	Translocation protein SEC62 OS=Mus musculus GN=Sec62 PE=1 SV=1 - [SEC62_MOUSE]	9.55	1	4	4	11	0.53	0.80	0.38	0.66
Q8BLN5	Lanosterol synthase OS=Mus musculus GN=Lss PE=2 SV=2 - [ERG7_MOUSE]	14.46	2	8	8	35	0.49	0.58	0.46	0.42
Q9ES97-3	Isoform 3 of Reticulon-3 OS=Mus musculus GN=Rtn3 - [RTN3_MOUSE]	27.85	5	3	4	27	0.45	0.25	0.29	0.16
Q8BKC5-2	Isoform 2 of Importin-5 OS=Mus musculus GN=Ipo5 - [IPO5_MOUSE]	16.78	2	14	14	61	0.67	0.68	0.51	0.53
Q9WTI7-3	Isoform 3 of Unconventional myosin-Ic OS=Mus musculus GN=Myo1c - [MYO1C_MOUSE]	43.01	5	39	40	333	-0.03	-0.10	-0.04	-0.09
Q505D1	Serine/threonine-protein phosphatase 6 regulatory ankyrin repeat subunit A OS=Mus musculus GN=Ankrd28 PE=1 SV=1 - [ANR28_MOUSE]	4.75	1	3	3	4	0.29	0.24	0.40	0.35
Q91W18-2	Isoform 2 of Tudor domain-containing protein 3 OS=Mus musculus GN=Tdrd3 - [TDRD3_MOUSE]	3.91	4	3	3	5	0.18	0.17	0.19	0.17
Q8C754	Vacuolar protein sorting associated protein 52 homolog OS=Mus musculus GN=Vps52 PE=2 SV=1 - [VPS52_MOUSE]	15.08	3	8	8	17	0.06	-0.20	0.11	-0.18
Q9EQP2	EH domain-containing protein 4 OS=Mus musculus GN=Ehd4 PE=1 SV=1 - [EHD4_MOUSE]	40.85	1	17	21	224	0.24	0.25	0.26	0.31
Q9D3W4	GPN-loop GTPase 3 OS=Mus musculus GN=Gpn3 PE=2 SV=1 - [GPN3_MOUSE]	4.58	1	1	1	1	0.53	0.42	0.52	0.41

P98203-4	Isoform 4 of Armadillo repeat protein deleted in velo-cardio-facial syndrome homolog OS=Mus musculus GN=Arvcf - [ARVC_MOUSE]	0.90	5	1	1	1	-0.28	-0.30	-0.16	-0.17
Q7TMM9	Tubulin beta-2A chain OS=Mus musculus GN=Tubb2a PE=1 SV=1 - [TBB2A_MOUSE]	60.67	2	1	20	998	0.05	0.34	0.09	0.28
Q8CIB6	Transmembrane protein 230 OS=Mus musculus GN=Tmem230 PE=1 SV=1 - [TM230_MOUSE]	20.83	1	2	2	5	0.02	0.27	-0.04	0.21
D3YUN5	T-cell immunoglobulin and mucin domain-containing protein 2 (Fragment) OS=Mus musculus GN=Timd2 PE=2 SV=1 - [D3YUN5_MOUSE]	15.22	3	1	1	4	-0.01	-0.14	0.20	-0.04
Q5JCT0	Beta-1,3-galactosyl-O-glycosyl-glycoprotein beta-1,6-N-acetylglucosaminyltransferase 3 OS=Mus musculus GN=Gcnt3 PE=2 SV=2 - [GCNT3_MOUSE]	29.06	1	11	11	33	0.08	0.08	0.04	-0.05
Q8BMA6	Signal recognition particle subunit SRP68 OS=Mus musculus GN=Srp68 PE=2 SV=2 [SRP68_MOUSE]	22.24	2	12	12	45	0.25	0.26	0.34	0.38
Q8R2I1	GDP-fucose transporter 1 OS=Mus musculus GN=Slc35c1 PE=2 SV=1 - [Q8R2I1_MOUSE]	4.86	2	2	2	2	0.06	0.28	-0.08	0.14
Q9D020-1	Isoform 1 of Cytosolic 5'-nucleotidase 3A OS=Mus musculus GN=Nt5c3a - [5NT3A_MOUSE]	56.23	2	15	15	95	0.17	0.22	0.32	0.31
Q9Z0R9	Fatty acid desaturase 2 OS=Mus musculus GN=Fads2 PE=2 SV=1 [FADS2_MOUSE]	6.08	1	2	2	6	0.74	0.55	0.55	0.36
Q9QXG4	Acetyl-coenzyme A synthetase, cytoplasmic OS=Mus musculus GN=Acss2 PE=1 SV=2 [ACSA_MOUSE]	19.40	5	12	12	63	0.27	0.30	0.29	0.34
E9QPI5	Sister chromatid cohesion protein PDS5 homolog A OS=Mus musculus GN=Pds5a PE=4 SV=1 - [E9QPI5_MOUSE]	7.66	2	7	9	32	0.19	0.10	0.49	0.45
Q8CGF6	WD repeat-containing protein 47 OS=Mus musculus GN=Wdr47 PE=1 SV=2 - [WDR47_MOUSE]	1.09	1	1	1	1	-0.72	-0.88	0.29	0.14
A2AJ15	Endoplasmic reticulum mannosyl-oligosaccharide 1,2-alpha-mannosidase OS=Mus musculus GN=Man1b1 PE=2 SV=1 - [MA1B1_MOUSE]	6.23	2	4	4	7	0.20	0.77	0.09	0.66

J3QP71	Basigin (Fragment) OS=Mus musculus GN=Bsg PE=4 SV=1 - [J3QP71_MOUSE]	37.56	1	1	6	95	0.46	1.39	0.47	1.39
Q7TQ62	Podocan OS=Mus musculus GN=Podn PE=2 SV=1 - [PODN_MOUSE]	5.40	4	3	3	4	0.93	0.92	0.43	0.42
F6T4M4	Serine/arginine repetitive matrix protein 1 (Fragment) OS=Mus musculus GN=Srrm1 PE=2 SV=1 - [F6T4M4_MOUSE]	20.00	2	1	3	47	-0.34	-0.38	-0.27	-0.27
Q6P549	Phosphatidylinositol 3,4,5-trisphosphate 5-phosphatase 2 OS=Mus musculus GN=Inppl1 PE=1 SV=1 - [SHIP2_MOUSE]	11.30	1	10	12	24	0.15	-0.07	0.21	0.13
Q9CQJ8	NADH dehydrogenase [ubiquinone] 1 beta subcomplex subunit 9 OS=Mus musculus GN=Ndufb9 PE=1 SV=3 - [NDUB9_MOUSE]	39.66	1	4	4	246	0.22	0.41	0.23	0.43
Q8CFR5	Dystrobrevin alpha OS=Mus musculus GN=Dtna PE=2 SV=1 - [Q8CFR5_MOUSE]	31.52	9	14	18	67	-0.08	0.00	-0.08	0.12
O08863	Baculoviral IAP repeat-containing protein 3 OS=Mus musculus GN=Birc3 PE=1 SV=2 - [BIRC3_MOUSE]	1.33	2	1	1	1	-0.62	0.39	0.53	1.54
Q3U487	E3 ubiquitin-protein ligase HECTD3 OS=Mus musculus GN=Hectd3 PE=1 SV=2 - [HECD3_MOUSE]	13.59	2	8	8	17	0.17	0.05	0.29	0.21
Q8K021	Secretory carrier-associated membrane protein 1 OS=Mus musculus GN=Scamp1 PE=1 SV=1 - [SCAM1_MOUSE]	24.56	3	6	6	42	0.16	0.00	0.24	0.02
Q3UPL0	Protein transport protein Sec31A OS=Mus musculus GN=Sec31a PE=1 SV=2 - [SC31A_MOUSE]	22.36	2	15	24	173	0.44	0.27	0.29	0.36
Q9CY64	Biliverdin reductase A OS=Mus musculus GN=Blvra PE=2 SV=1 - [BIEA_MOUSE]	39.66	4	12	12	52	0.35	0.28	0.33	0.34
Q8VI93	2'-5'-oligoadenylate synthase 3 OS=Mus musculus GN=Oas3 PE=2 SV=1 - [OAS3_MOUSE]	5.80	1	4	4	10	-0.18	0.88	-0.54	0.52
Q6PB66	Leucine-rich PPR motif-containing protein, mitochondrial OS=Mus musculus GN=Lrpprc PE=1 SV=2 - [LPPRC_MOUSE]	33.12	6	43	43	238	0.21	0.20	0.21	0.23
Q9CPQ1	Cytochrome c oxidase subunit 6C OS=Mus musculus GN=Cox6c PE=1 SV=3 - [COX6C_MOUSE]	47.37	2	7	7	68	0.09	0.29	0.01	0.22

Q3TC46	Protein PAT1 homolog 1 OS=Mus musculus GN=Patl1 PE=1 SV=2 - [PATL1_MOUSE]	4.68	1	2	3	4	-0.06	0.00	0.32	0.37
Q6P1G0	HEAT repeat-containing protein 6 OS=Mus musculus GN=Heatr6 PE=2 SV=1 - [HEAT6_MOUSE]	1.18	1	1	1	1	0.77	0.85	0.75	0.84
D3YWX2	YLP motif-containing protein 1 OS=Mus musculus GN=Ylpm1 PE=2 SV=2 - [D3YWX2_MOUSE]	2.66	8	4	5	7	-0.63	-0.37	-0.33	-0.06
E9QQ25	Striated muscle-specific serine/threonine-protein kinase OS=Mus musculus GN=Speg PE=2 SV=1 - [E9QQ25_MOUSE]	3.59	5	7	8	17	-0.06	-0.19	0.20	-0.07
Q9JJN5	Carboxypeptidase N catalytic chain OS=Mus musculus GN=Cpn1 PE=2 SV=1 - [CBPN_MOUSE]	11.16	1	3	4	4	0.22	-0.25	1.27	0.80
D3YXD4	IQ domain-containing protein E (Fragment) OS=Mus musculus GN=Iqce PE=2 SV=1 - [D3YXD4_MOUSE]	8.65	2	1	1	3	-0.91	-0.67	-0.31	-0.08
Q8C547	HEAT repeat-containing protein 5B OS=Mus musculus GN=Heatr5b PE=2 SV=3 - [HTR5B_MOUSE]	3.29	4	4	4	10	0.12	0.00	0.07	-0.06
Q9DC29	ATP-binding cassette sub-family B member 6, mitochondrial OS=Mus musculus GN=Abcb6 PE=1 SV=1 - [ABCB6_MOUSE]	10.57	2	3	5	16	-0.21	-0.16	-0.28	-0.04
Q8R1F0	Leydig cell tumor 10 kDa protein homolog OS=Mus musculus GN=D8Ertd738e PE=2 SV=1 - [L10K_MOUSE]	7.45	1	1	1	1	0.12	0.39	0.57	0.84
B1AZA5	Transmembrane protein 245 OS=Mus musculus GN=Tmem245 PE=1 SV=1 - [TM245_MOUSE]	4.79	3	3	3	7	-0.45	-0.17	-0.38	-0.10
A2RT91	Ankyrin and armadillo repeat-containing protein OS=Mus musculus GN=Ankar PE=2 SV=1 - [ANKAR_MOUSE]	1.77	1	1	1	1	-0.24	0.07	-0.26	0.05
D3Z5J3	Sorbin and SH3 domain containing protein 1 OS=Mus musculus GN=Sorbs1 PE=2 SV=1 - [D3Z5J3_MOUSE]	42.96	3	7	34	455	0.09	0.18	0.10	0.26
Q9WVM8	Kynurenine/alpha-aminoadipate aminotransferase, mitochondrial OS=Mus musculus GN=Aadat PE=2 SV=1 - [AADAT_MOUSE]	3.06	1	1	2	2	0.40	0.52	0.46	0.57

B1AUP0	Serine/threonine-protein kinase A-Raf (Fragment) OS=Mus musculus GN=Araf PE=2 SV=1 - [B1AUP0_MOUSE]	40.54	1	2	2	4	0.31	0.12	0.38	0.18
Q3TYX3	SET and MYND domain-containing protein 5 OS=Mus musculus GN=Smyd5 PE=2 SV=2 - [SMYD5_MOUSE]	6.49	1	2	2	7	0.29	0.62	0.12	0.44
P68373	Tubulin alpha-1C chain OS=Mus musculus GN=Tuba1c PE=1 SV=1 - [TBA1C_MOUSE]	51.00	3	5	20	962	0.29	0.37	0.33	0.39
D3YZN4	Paraplegin OS=Mus musculus GN=Spg7 PE=2 SV=1 - [D3YZN4_MOUSE]	8.28	7	4	4	11	0.16	-0.23	0.23	-0.05
O54734	Dolichyl-diphosphooligosaccharide--protein glycosyltransferase 48 kDa subunit OS=Mus musculus GN=Ddost PE=1 SV=2 - [OST48_MOUSE]	39.91	1	13	13	110	0.32	0.41	0.29	0.35
B7ZC19	GPI transamidase component PIG-T OS=Mus musculus GN=Pigt PE=2 SV=1 - [B7ZC19_MOUSE]	16.55	3	6	6	10	0.32	0.21	0.25	0.27
Q3TJD7	PDZ and LIM domain protein 7 OS=Mus musculus GN=Pdlim7 PE=2 SV=1 - [PDLI7_MOUSE]	61.05	7	19	19	320	0.18	0.18	0.24	0.22
Q9D7J4	Cytochrome c oxidase protein 20 homolog OS=Mus musculus GN=Cox20 PE=2 SV=1 - [COX20_MOUSE]	22.22	1	2	2	15	-0.28	0.50	0.05	0.30
Q9WVP1	AP-1 complex subunit mu-2 OS=Mus musculus GN=Ap1m2 PE=1 SV=3 - [AP1M2_MOUSE]	26.48	1	2	11	43	0.80	0.77	0.97	0.94
O88665	Bromodomain-containing protein 7 OS=Mus musculus GN=Brd7 PE=1 SV=1 - [BRD7_MOUSE]	5.07	1	2	2	3	-0.32	0.07	-0.18	0.21
Q9ERR1-2	Isoform 2 of Nuclear distribution protein nudE-like 1 OS=Mus musculus GN=Ndel1 - [NDEL1_MOUSE]	11.43	2	2	3	6	0.20	0.11	0.29	0.19
Q91X83	S-adenosylmethionine synthase isoform type-1 OS=Mus musculus GN=Mat1a PE=2 SV=1 - [METK1_MOUSE]	8.84	1	1	4	13	0.30	0.35	0.16	0.21
Q8VEM8	Phosphate carrier protein, mitochondrial OS=Mus musculus GN=Slc25a3 PE=1 SV=1 - [MPCP_MOUSE]	30.81	2	13	13	249	0.14	0.12	0.11	0.15
Q3TE27	Protein Arhgap4 OS=Mus musculus GN=Arhgap4 PE=2 SV=1 - [Q3TE27_MOUSE]	1.43	5	1	1	6	-0.27	-1.05	0.48	-0.38
A2AKI5	Integrin alpha-V light chain OS=Mus musculus GN=Itgav PE=2 SV=1 - [A2AKI5_MOUSE]	28.77	4	24	24	93	0.14	0.21	0.19	0.16

Q3UMB9	WASH complex subunit 7 OS=Mus musculus GN=Kiaa1033 PE=2 SV=2 - [WASH7_MOUSE]	2.73	1	2	3	7	0.52	0.59	0.44	0.51
Q9Z0W3	Nuclear pore complex protein Nup160 OS=Mus musculus GN=Nup160 PE=1 SV=2 - [NU160_MOUSE]	3.42	2	4	4	10	-0.18	0.26	-0.19	0.25
A6H5X4-2	Isoform 2 of PHD finger protein 11 OS=Mus musculus GN=Phf11 - [PHF11_MOUSE]	33.09	4	3	3	6	0.53	0.49	0.50	0.46
Q9D853	Methyltransferase-like protein 10 OS=Mus musculus GN=Mettl10 PE=2 SV=1 - [MET10_MOUSE]	10.66	1	2	2	3	-0.12	-0.61	0.10	-0.39
Q9D4H8	Cullin-2 OS=Mus musculus GN=Cul2 PE=1 SV=2 - [CUL2_MOUSE]	11.28	4	7	7	12	0.22	0.31	0.13	0.28
Q61941	NAD(P) transhydrogenase, mitochondrial OS=Mus musculus GN=Nnt PE=1 SV=2 - [NNTM_MOUSE]	27.26	4	25	25	95	1.35	1.24	1.35	1.28
Q91WD5	NADH dehydrogenase [ubiquinone] iron-sulfur protein 2, mitochondrial OS=Mus musculus GN=Ndufs2 PE=1 SV=1 - [NDUS2_MOUSE]	57.45	3	18	18	227	0.19	0.15	0.11	0.14
Q9D1F9	Protein Slc37a4 OS=Mus musculus GN=Slc37a4 PE=2 SV=1 - [Q9D1F9_MOUSE]	3.03	1	1	1	1	-1.02	-1.50	-0.25	-0.73
Q8BFZ9	Erlin-2 OS=Mus musculus GN=Erlin2 PE=1 SV=1 - [ERLN2_MOUSE]	38.53	2	9	12	81	0.14	0.25	0.12	0.20
P97393	Rho GTPase-activating protein 5 OS=Mus musculus GN=Arhgap5 PE=2 SV=2 - [RHG05_MOUSE]	5.60	2	6	7	9	0.55	0.25	0.43	0.18
Q6GTL7	6-phosphofructo-2-kinase/fructose-2, 6-biphosphatase 2 OS=Mus musculus GN=Pfkfb2 PE=2 SV=1 - [Q6GTL7_MOUSE]	16.80	10	7	8	14	0.23	-0.03	0.36	0.08
J3QNR5	Uncharacterized protein OS=Mus musculus GN=Tubb4b-ps1 PE=3 SV=1 - [J3QNR5_MOUSE]	43.92	2	2	15	1011	0.33	0.27	0.29	0.20
O55087-2	Isoform B-2 of Myocyte specific enhancer factor 2B OS=Mus musculus GN=Mef2b - [MEF2B_MOUSE]	3.54	4	1	1	1	1.04	1.34	0.86	1.16
Q9CQY5-2	Isoform 2 of Magnesium transporter protein 1 OS=Mus musculus GN=Magt1 - [MAGT1_MOUSE]	21.24	6	6	7	28	0.26	0.20	0.27	0.31
B7FAU9	Filamin, alpha OS=Mus musculus GN=Flna PE=4 SV=1 - [B7FAU9_MOUSE]	72.22	9	145	159	7048	0.40	0.44	0.36	0.40

P36916	Guanine nucleotide-binding protein-like 1 OS=Mus musculus GN=Gnl1 PE=1 SV=4 - [GNL1_MOUSE]	11.70	2	6	6	19	0.14	-0.18	0.22	0.02
F6V9H6	Protein Muc3 (Fragment) OS=Mus musculus GN=Muc3 PE=4 SV=1 - [F6V9H6_MOUSE]	26.17	2	4	5	20	0.55	0.73	0.53	0.60
Q921L3	Transmembrane and coiled-coil domain-containing protein 1 OS=Mus musculus GN=Tmco1 PE=2 SV=1 - [TMCO1_MOUSE]	12.77	1	2	2	9	0.59	0.56	0.48	0.51
E9PV80	Nuclear receptor coactivator 2 OS=Mus musculus GN=Ncoa2 PE=4 SV=1 - [E9PV80_MOUSE]	3.37	2	3	3	3	-0.30	-0.44	-0.05	-0.20
Q61696	Heat shock 70 kDa protein 1A OS=Mus musculus GN=Hspa1a PE=1 SV=2 - [HS71A_MOUSE]	57.41	2	15	33	937	-0.05	-0.07	0.00	0.01
P04925	Major prion protein OS=Mus musculus GN=Prnp PE=1 SV=2 - [PRIO_MOUSE]	4.72	1	1	1	11	0.17	0.15	0.10	0.09
Q9D281	Protein Noxp20 OS=Mus musculus GN=Fam114a1 PE=2 SV=1 - [NXP20_MOUSE]	27.24	2	10	10	28	0.21	0.18	0.20	0.34
Q99L20	Glutathione S-transferase theta 3 OS=Mus musculus GN=Gstt3 PE=2 SV=1 - [Q99L20_MOUSE]	44.81	5	8	10	93	0.06	0.20	0.00	0.07
P70302	Stromal interaction molecule 1 OS=Mus musculus GN=Stim1 PE=1 SV=2 - [STIM1_MOUSE]	23.65	1	11	12	46	0.18	0.03	0.04	-0.05
Q9WV27	Sodium/potassium-transporting ATPase subunit alpha-4 OS=Mus musculus GN=Atp1a4 PE=1 SV=3 - [AT1A4_MOUSE]	10.76	2	1	11	281	0.06	0.06	0.22	0.21
F6V682	Protein FAM178B (Fragment) OS=Mus musculus GN=Fam178b PE=4 SV=1 - [F6V682_MOUSE]	16.33	1	1	1	1	-0.39	-0.03	0.02	0.38
D3Z495	Protein spire homolog 1 OS=Mus musculus GN=Spire1 PE=2 SV=1 - [D3Z495_MOUSE]	3.69	8	1	3	11	-0.96	-1.47	-0.70	-1.21
P56392	Cytochrome c oxidase subunit 7A1, mitochondrial OS=Mus musculus GN=Cox7a1 PE=2 SV=1 - [CX7A1_MOUSE]	16.25	1	1	1	5	0.10	0.07	0.13	0.09
Q8BMI0	F-box only protein 38 OS=Mus musculus GN=Fbxo38 PE=1 SV=1 - [FBX38_MOUSE]	2.26	1	1	1	2	0.18	-0.16	-0.26	-0.60
Q5SZA1-2	Isoform 2 of Sodium-dependent phosphate transport protein 3 OS=Mus musculus GN=Slc17a2 - [NPT3_MOUSE]	4.47	2	1	1	2	-0.18	-0.25	-0.01	-0.08

O78196	H-2 class II histocompatibility antigen, I-A beta chain OS=Mus musculus GN=H2-Eb1 PE=2 SV=1 - [O78196_MOUSE]	8.71	1	1	1	8	0.44	0.57	0.52	0.79
Q68FL0	Slc8a1 protein OS=Mus musculus GN=Slc8a1 PE=2 SV=1 - [Q68FL0_MOUSE]	7.10	4	4	4	6	0.09	0.30	-0.08	-0.04
Q8CH09	SURP and G-patch domain-containing protein 2 OS=Mus musculus GN=Sugp2 PE=2 SV=2 - [SUGP2_MOUSE]	2.34	1	2	2	2	0.39	0.48	0.48	0.56
Q9CW03	Structural maintenance of chromosomes protein 3 OS=Mus musculus GN=Smc3 PE=1 SV=2 [SMC3_MOUSE]	28.27	1	30	33	113	0.31	0.39	0.34	0.42
F8VPX6	Protein Speer4a OS=Mus musculus GN=Speer4a PE=4 SV=1 - [F8VPX6_MOUSE]	8.61	1	2	2	2	-0.87	-3.07	-1.24	-3.45
Q61475	Complement decay-accelerating factor, GPI-anchored OS=Mus musculus GN=Cd55 PE=2 SV=2 - [DAF1_MOUSE]	3.85	4	2	2	4	0.13	0.05	0.53	0.45
Q6ZPS6	Ankyrin repeat and IBR domain-containing protein 1 OS=Mus musculus GN=Ankib1 PE=1 SV=2 - [AKIB1_MOUSE]	2.95	1	2	3	4	0.48	0.35	0.53	0.40
Q9EQC8	Papillary renal cell carcinoma (Translocation-associated) OS=Mus musculus GN=Prcc PE=2 SV=1 - [Q9EQC8_MOUSE]	8.76	1	3	3	4	-0.68	-0.62	-0.28	-0.22
E9PZJ7	Protein Vmn2r107 OS=Mus musculus GN=Vmn2r107 PE=2 SV=1 - [E9PZJ7_MOUSE]	2.09	1	1	2	2	0.21	0.17	0.39	0.35
E9PXY1	Cullin-4B OS=Mus musculus GN=Cul4b PE=3 SV=2 - [E9PXY1_MOUSE]	10.49	4	3	10	22	0.34	0.16	0.27	0.12
E9PWC0	Microtubule-associated protein OS=Mus musculus GN=Map4 PE=2 SV=1 - [E9PWC0_MOUSE]	21.95	4	2	17	98	-0.12	0.28	0.07	0.47
Q62074	Protein kinase C iota type OS=Mus musculus GN=Prkci PE=1 SV=3 - [KPCI_MOUSE]	3.36	1	1	2	2	0.53	-0.10	0.37	-0.26
F2Z3Z9	3-phosphoinositide-dependent protein kinase 1 OS=Mus musculus GN=Pdpk1 PE=2 SV=1 - [F2Z3Z9_MOUSE]	9.03	8	3	3	6	0.25	-0.02	0.25	-0.10
Q9Z2Z9	Glutamine--fructose-6-phosphate aminotransferase [isomerizing] 2 OS=Mus musculus GN=Gfpt2 PE=2 SV=3 [GFPT2_MOUSE]	11.58	1	1	7	136	-0.29	-0.17	-0.10	0.02

F6U6U5	Serine/threonine-protein kinase SIK3 (Fragment) OS=Mus musculus GN=Sik3 PE=4 SV=1 - [F6U6U5_MOUSE]	0.58	4	1	1	5	0.10	-0.07	0.20	0.05
F6U4D1	Glycosyltransferase 8 domain-containing protein 2 (Fragment) OS=Mus musculus GN=Glt8d2 PE=4 SV=2 - [F6U4D1_MOUSE]	40.32	1	1	1	4	0.31	0.92	0.29	0.91
O54714-3	Isoform 3 of E3 SUMO-protein ligase PIAS3 OS=Mus musculus GN=Pias3 - [PIAS3_MOUSE]	5.06	3	1	1	2	1.01	0.85	0.78	0.62
Q8CHQ0	F-box only protein 4 OS=Mus musculus GN=Fbxo4 PE=1 SV=2 - [FBX4_MOUSE]	5.71	1	2	2	4	0.09	0.37	0.17	0.44
P68368	Tubulin alpha-4A chain OS=Mus musculus GN=Tuba4a PE=1 SV=1 - [TBA4A_MOUSE]	52.23	3	6	21	911	0.26	0.25	0.29	0.22
P35436	Glutamate receptor ionotropic, NMDA 2A OS=Mus musculus GN=Grin2a PE=1 SV=2 - [NMDE1_MOUSE]	2.53	1	2	2	2	0.93	1.45	0.82	1.34
Q91ZY2	Histamine H4 receptor OS=Mus musculus GN=Hrh4 PE=2 SV=1 - [HRH4_MOUSE]	3.32	1	1	1	1	-0.15	-0.15	-0.18	-0.18
P31938	Dual specificity mitogen-activated protein kinase kinase 1 OS=Mus musculus GN=Map2k1 PE=1 SV=2 - [MP2K1_MOUSE]	37.40	6	8	12	100	0.43	0.38	0.38	0.36
Q9Z2M7	Phosphomannomutase 2 OS=Mus musculus GN=Pmm2 PE=2 SV=1 - [PMM2_MOUSE]	54.96	1	12	14	167	0.48	0.60	0.49	0.53
Q91WJ7-2	Isoform 2 of SPATS2-like protein OS=Mus musculus GN=Spats2l - [SPS2L_MOUSE]	7.72	9	3	3	8	1.54	1.05	1.35	0.42
Q9ESU7	Neutral amino acid transporter ASCT2 OS=Mus musculus GN=Slc1a5 PE=2 SV=1 - [Q9ESU7_MOUSE]	12.61	3	5	5	55	-0.05	0.00	0.00	0.03
Q9EQZ7-3	Isoform 3 of Regulating synaptic membrane exocytosis protein 2 OS=Mus musculus GN=Rims2 - [RIMS2_MOUSE]	1.00	3	1	1	1	-0.62	-0.15	-0.16	0.31
O35452	Protein Tnxb OS=Mus musculus GN=Tnxb PE=4 SV=1 - [O35452_MOUSE]	17.90	2	47	47	196	-0.02	-0.01	0.07	0.03
Q3UQ44	Ras GTPase-activating-like protein IQGAP2 OS=Mus musculus GN=Iqgap2 PE=1 SV=2 - [IQGA2_MOUSE]	42.29	1	50	56	351	0.29	0.33	0.30	0.33
Q8CCH7-3	Isoform 3 of Zinc finger protein ZFPM2 OS=Mus musculus GN=Zfpm2 - [FOG2_MOUSE]	1.15	2	1	1	1	0.02	0.00	0.81	0.79

O70309	Integrin beta-5 OS=Mus musculus GN=Itgb5 PE=1 SV=2 - [ITB5_MOUSE]	6.52	4	3	4	6	0.00	0.67	0.13	0.80
P63011	Ras-related protein Rab-3A OS=Mus musculus GN=Rab3a PE=1 SV=1 - [RAB3A_MOUSE]	31.36	14	2	6	46	-0.06	0.15	-0.13	0.08
Q8C522	Endonuclease domain-containing 1 protein OS=Mus musculus GN=Endod1 PE=1 SV=2 - [ENDD1_MOUSE]	28.94	1	9	9	66	0.08	-0.09	0.13	-0.05
Q8VBT0	Thioredoxin-related transmembrane protein 1 OS=Mus musculus GN=Tmx1 PE=1 SV=1 [TMX1_MOUSE]	15.47	2	6	6	23	0.16	0.33	0.14	0.19
P35288	Ras-related protein Rab-23 OS=Mus musculus GN=Rab23 PE=1 SV=2 - [RAB23_MOUSE]	27.85	2	4	4	9	0.70	0.95	0.53	0.78
Q5SYD0	Unconventional myosin-Id OS=Mus musculus GN=Myo1d PE=1 SV=1 - [MYO1D_MOUSE]	35.39	2	27	30	116	0.30	0.21	0.25	0.20
P82198	Transforming growth factor-beta-induced protein ig-h3 OS=Mus musculus GN=Tgfbi PE=2 SV=1 - [BGH3_MOUSE]	36.60	1	20	20	114	0.27	0.36	0.22	0.36
Q5SWU9	Acetyl-CoA carboxylase 1 OS=Mus musculus GN=Acaca PE=1 SV=1 - [ACACA_MOUSE]	8.78	3	15	18	47	0.05	0.00	0.08	0.12
A6H5Z3-2	Isoform 2 of Exocyst complex component 6B OS=Mus musculus GN=Exoc6b - [EXC6B_MOUSE]	6.93	4	4	4	5	0.29	0.10	0.75	0.55
E9QAZ2	Ribosomal protein L15 OS=Mus musculus GN=Gm10020 PE=3 SV=1 - [E9QAZ2_MOUSE]	42.16	4	9	9	124	0.31	0.45	0.37	0.41
Q9CZJ2	Heat shock 70 kDa protein 12B OS=Mus musculus GN=Hspa12b PE=1 SV=1 - [HS12B_MOUSE]	8.47	2	5	5	7	0.03	-0.30	0.10	-0.19
Q80Z19	Mucin-2 (Fragments) OS=Mus musculus GN=Muc2 PE=1 SV=2 [MUC2_MOUSE]	32.16	5	79	80	1448	0.07	0.09	0.03	0.02
Q9DBC3	Cap-specific mRNA (nucleoside-2'-O-)-methyltransferase 1 OS=Mus musculus GN=Ftsjd2 PE=1 SV=1 - [MTR1_MOUSE]	13.26	4	4	9	30	0.59	0.48	0.50	0.38
Q8CIE4	Plec1 protein OS=Mus musculus GN=Parp10 PE=2 SV=1 - [Q8CIE4_MOUSE]	3.02	1	2	2	3	0.26	0.14	0.46	0.33
P84084	ADP-ribosylation factor 5 OS=Mus musculus GN=Arf5 PE=2 SV=2 - [ARF5_MOUSE]	63.89	4	3	10	205	0.34	0.29	0.34	0.39

O35261	Transcription factor E2F3 OS=Mus musculus GN=E2f3 PE=1 SV=2 - [E2F3_MOUSE]	3.06	1	1	1	1	0.33	0.48	0.02	0.18
B2ZRS5	DEP domain-containing mTOR-interacting protein OS=Mus musculus GN=Deptor PE=4 SV=1 - [B2ZRS5_MOUSE]	18.95	4	6	6	19	0.08	-0.06	0.36	-0.03
F8WHJ1	FTS and Hook-interacting protein OS=Mus musculus GN=Fam160a2 PE=2 SV=1 - [F8WHJ1_MOUSE]	9.68	5	5	5	13	0.21	0.24	0.16	-0.24
Q9Z2M6	Ubiquitin-like protein 3 OS=Mus musculus GN=Ubl3 PE=1 SV=1 - [UBL3_MOUSE]	24.79	1	2	2	8	0.09	0.67	-0.49	0.11
A2A4N9	ATP-dependent RNA helicase DHX8 (Fragment) OS=Mus musculus GN=Dhx8 PE=2 SV=1 - [A2A4N9_MOUSE]	0.60	2	1	1	1	-0.15	-0.41	0.35	0.08
Q05915	GTP cyclohydrolase 1 OS=Mus musculus GN=Gch1 PE=2 SV=1 - [GCH1_MOUSE]	7.47	1	2	2	3	-0.46	-0.72	0.00	-0.26
P70275	Semaphorin-3E OS=Mus musculus GN=Sema3e PE=1 SV=3 - [SEM3E_MOUSE]	4.77	1	3	3	6	0.21	-0.26	0.53	0.07
Q9WU78	Programmed cell death 6-interacting protein OS=Mus musculus GN=Pdcd6ip PE=1 SV=3 - [PDC6I_MOUSE]	41.31	3	34	34	182	0.57	0.53	0.54	0.53
O54865	Guanylate cyclase soluble subunit beta-1 OS=Mus musculus GN=Gucy1b3 PE=2 SV=1 - [GCYB1_MOUSE]	17.10	1	10	10	26	0.22	0.10	0.11	0.10
Q9CZH7	Matrix-remodeling-associated protein 7 OS=Mus musculus GN=Mxra7 PE=1 SV=2 - [MXRA7_MOUSE]	37.08	2	3	3	11	-0.08	0.17	-0.01	0.06
Q8BWM0	Prostaglandin E synthase 2 OS=Mus musculus GN=Ptges2 PE=1 SV=3 - [PGES2_MOUSE]	30.99	1	8	8	49	0.22	0.45	0.24	0.63
Q8BGV8	Mitochondrial dynamic protein MID51 OS=Mus musculus GN=Smcr7l PE=1 SV=1 - [MID51_MOUSE]	6.70	1	3	3	7	0.07	-0.08	0.00	-0.15
E9QJV4	Pre-mRNA-processing factor 39 OS=Mus musculus GN=Prpf39 PE=2 SV=1 - [E9QJV4_MOUSE]	3.16	2	2	2	4	0.32	0.18	0.36	0.22
B2RSH2	Guanine nucleotide-binding protein G(i) subunit alpha-1 OS=Mus musculus GN=Gnai1 PE=2 SV=1 [GNAI1_MOUSE]	34.75	6	4	11	74	-0.43	-0.30	-0.05	0.56

A2ARP1-7	Isoform 6 of Inositol hexakisphosphate and diphosphoinositol-pentakisphosphate kinase 1 OS=Mus musculus GN=Ppip5k1 - [VIP1_MOUSE]	7.60	7	1	4	8	-0.36	0.14	-0.22	0.28
F7AFM3	Threonine synthase-like 2 (Fragment) OS=Mus musculus GN=Thnsl2 PE=4 SV=1 - [F7AFM3_MOUSE]	23.27	2	4	4	7	0.02	0.10	-0.40	-0.24
P03911	NADH-ubiquinone oxidoreductase chain 4 OS=Mus musculus GN=Mtnd4 PE=1 SV=1 - [NU4M_MOUSE]	1.31	1	1	1	2	-0.41	-0.49	-0.62	-0.70
Q9D2R6	Cytochrome c oxidase assembly protein 3 homolog, mitochondrial OS=Mus musculus GN=Coa3 PE=2 SV=1 - [COA3_MOUSE]	17.59	2	2	2	11	0.43	0.50	0.45	0.55
Q6DVA0	LEM domain-containing protein 2 OS=Mus musculus GN=Lemd2 PE=1 SV=1 - [LEMD2_MOUSE]	29.35	1	11	11	44	0.02	-0.19	0.17	-0.11
Q8BHG3	Cell cycle control protein 50B OS=Mus musculus GN=Tmem30b PE=2 SV=1 - [CC50B_MOUSE]	9.92	1	3	3	8	0.27	0.06	0.44	0.23
F8WHT3	Protein PRRC2B OS=Mus musculus GN=Prrc2b PE=2 SV=1 - [F8WHT3_MOUSE]	2.87	6	3	4	4	0.13	0.08	0.42	0.37
F6VVW4	Actin-binding LIM protein 2 (Fragment) OS=Mus musculus GN=Ablim2 PE=4 SV=1 - [F6VVW4_MOUSE]	17.73	12	2	3	8	-0.66	-0.88	-0.46	-0.57
Q9CQS4	Solute carrier family 25 member 46 OS=Mus musculus GN=Slc25a46 PE=1 SV=1 - [S2546_MOUSE]	9.81	1	3	3	9	0.07	0.11	0.00	0.12
P30677	Guanine nucleotide-binding protein subunit alpha-14 OS=Mus musculus GN=Gna14 PE=1 SV=2 - [GNA14_MOUSE]	27.89	2	4	8	45	0.33	0.28	0.33	0.18
Q91WS2-2	Isoform 2 of NACHT, LRR and PYD domains-containing protein 6 OS=Mus musculus GN=Nlrp6 - [NALP6_MOUSE]	10.99	3	4	5	9	-0.49	-0.10	0.05	0.37
A2AQE4	COP9 signalosome complex subunit 2 OS=Mus musculus GN=Cops2 PE=2 SV=1 - [A2AQE4_MOUSE]	17.16	3	7	7	30	0.65	0.58	0.53	0.54
Q7TMF3	NADH dehydrogenase [ubiquinone] 1 alpha subcomplex subunit 12 OS=Mus musculus GN=Ndufa12 PE=1 SV=2 - [NDUAC_MOUSE]	58.62	1	6	6	75	0.01	-0.04	0.04	0.02

Q9D6J5	NADH dehydrogenase [ubiquinone] 1 beta subcomplex subunit 8, mitochondrial OS=Mus musculus GN=Ndufb8 PE=1 SV=1 - [NDUB8_MOUSE]	46.77	4	6	6	49	0.03	-0.10	0.07	-0.06
P50153	Guanine nucleotide-binding protein G(I)/G(S)/G(O) subunit gamma-4 OS=Mus musculus GN=Gng4 PE=2 SV=1 - [GBG4_MOUSE]	21.33	1	1	1	6	0.03	-0.08	0.06	-0.05
Q9WV84	Nucleoside diphosphate kinase, mitochondrial OS=Mus musculus GN=Nme4 PE=1 SV=1 [NDKM_MOUSE]	4.30	1	1	1	6	0.04	0.27	0.19	0.26
P11531	Dystrophin OS=Mus musculus GN=Dmd PE=1 SV=3 - [DMD_MOUSE]	22.76	6	65	71	224	0.09	0.07	0.08	0.05
Q60809	CCR4-NOT transcription complex subunit 7 OS=Mus musculus GN=Cnot7 PE=1 SV=1 - [CNOT7_MOUSE]	11.93	1	2	2	6	0.33	-0.28	0.12	0.16
Q6P3Y9	Podocan-like protein 1 OS=Mus musculus GN=Podnl1 PE=2 SV=2 - [PONL1_MOUSE]	3.52	1	1	2	2	0.67	0.47	1.05	0.85
Q9ERD7	Tubulin beta-3 chain OS=Mus musculus GN=Tubb3 PE=1 SV=1 - [TBB3_MOUSE]	46.00	1	4	15	879	0.21	0.23	0.25	0.20
P32037	Solute carrier family 2, facilitated glucose transporter member 3 OS=Mus musculus GN=Slc2a3 PE=1 SV=1 - [GTR3_MOUSE]	6.69	1	3	3	6	-0.27	-0.21	-0.10	-0.04
Q8JZQ2	AFG3-like protein 2 OS=Mus musculus GN=Afg3l2 PE=1 SV=1 - [AFG32_MOUSE]	24.44	1	17	19	85	0.18	0.28	0.15	0.24
Q9DC16	Endoplasmic reticulum-Golgi intermediate compartment protein 1 OS=Mus musculus GN=Ergic1 PE=1 SV=1 - [ERGI1_MOUSE]	19.66	2	4	4	39	-0.02	-0.10	0.03	0.04
Q9DC40	Telomere length regulation protein TEL2 homolog OS=Mus musculus GN=Telo2 PE=1 SV=2 - [TELO2_MOUSE]	2.98	1	2	2	2	0.46	0.17	-0.09	-0.14
Q9JIA7	Sphingosine kinase 2 OS=Mus musculus GN=Sphk2 PE=1 SV=2 - [SPHK2_MOUSE]	6.65	1	3	3	14	0.10	0.08	0.21	0.19
Q9D2M8	Ubiquitin-conjugating enzyme E2 variant 2 OS=Mus musculus GN=Ube2v2 PE=2 SV=4 - [UB2V2_MOUSE]	53.10	5	3	7	71	0.54	0.52	0.64	0.69

Q5NCE8	Magnesium transporter MRS2 homolog, mitochondrial OS=Mus musculus GN=Mrs2 PE=2 SV=2 - [MRS2_MOUSE]	5.07	1	2	2	3	0.20	0.28	0.17	0.25
Q8BU30	Isoleucine--tRNA ligase, cytoplasmic OS=Mus musculus GN=Iars PE=2 SV=2 - [SYIC_MOUSE]	22.74	3	26	26	69	0.53	0.51	0.43	0.59
O08832	Polypeptide N-acetylgalactosaminyltransferase 4 OS=Mus musculus GN=Galnt4 PE=2 SV=1 - [GALT4_MOUSE]	23.53	1	9	10	43	0.05	-0.07	0.16	-0.07
Q3U4F0	Sideroflexin-3 OS=Mus musculus GN=Sfxn3 PE=2 SV=1 - [Q3U4F0_MOUSE]	32.38	3	7	8	94	-0.07	-0.14	0.01	-0.05
Q91XI1	tRNA-dihydrouridine(47) synthase [NAD(P)(+)]-like OS=Mus musculus GN=Dus3l PE=2 SV=1 [DUS3L_MOUSE]	8.95	2	5	5	22	0.07	0.00	0.42	0.23
Q99LL3	Carbohydrate sulfotransferase 12 OS=Mus musculus GN=Chst12 PE=2 SV=2 - [CHSTC_MOUSE]	1.67	1	1	1	1	0.45	0.48	0.20	0.22
Q91WC0	Histone-lysine N-methyltransferase setd3 OS=Mus musculus GN=Setd3 PE=1 SV=1 - [SETD3_MOUSE]	15.49	7	8	8	21	0.19	0.11	0.30	0.21
A2ACC7	Ral GTPase-activating protein subunit beta (Fragment) OS=Mus musculus GN=Ralgapb PE=2 SV=2 - [A2ACC7_MOUSE]	6.65	6	4	4	8	0.51	0.47	0.54	0.49
Q07113	Cation-independent mannose-6-phosphate receptor OS=Mus musculus GN=Igf2r PE=1 SV=1 - [MPRI_MOUSE]	10.51	1	19	19	51	0.64	0.47	0.42	0.37
Q8CBQ5	Phosphatidylinositol 4-kinase type 2-beta OS=Mus musculus GN=Pi4k2b PE=2 SV=1 - [P4K2B_MOUSE]	7.89	2	3	3	5	0.55	-1.21	0.37	-1.39
E9Q8Q1	BRCA1-A complex subunit BRE OS=Mus musculus GN=Bre PE=2 SV=1 - [E9Q8Q1_MOUSE]	23.26	4	1	1	3	0.23	0.21	0.51	0.49
Q3TZZ7	Extended synaptotagmin-2 OS=Mus musculus GN=Esyt2 PE=1 SV=1 - [ESYT2_MOUSE]	25.09	2	15	15	50	0.06	0.10	-0.04	0.10
Q9CRC8	Leucine-rich repeat-containing protein 40 OS=Mus musculus GN=Lrrc40 PE=2 SV=2 - [LRC40_MOUSE]	16.45	5	10	10	26	0.26	0.40	0.25	0.33
D3YZ62	Unconventional myosin-Va OS=Mus musculus GN=Myo5a PE=2 SV=1 - [D3YZ62_MOUSE]	3.61	9	6	7	11	-0.15	-0.18	-0.37	-0.26

Q8C079-4	Isoform 4 of Striatin-interacting protein 1 OS=Mus musculus GN=Strip1 - [STRP1_MOUSE]	5.49	3	2	3	14	0.04	0.17	0.31	0.18
F6QWY7	Protein Lines homolog (Fragment) OS=Mus musculus GN=Lins PE=2 SV=1 - [F6QWY7_MOUSE]	5.24	5	1	1	1	-0.12	0.10	1.23	1.45
Q62417-7	Isoform 7 of Sorbin and SH3 domain-containing protein 1 OS=Mus musculus GN=Sorbs1 - [SRBS1_MOUSE]	43.54	1	3	30	463	0.07	0.23	0.18	0.28
E9QP59	Inner nuclear membrane protein Man1 OS=Mus musculus GN=Lemd3 PE=2 SV=1 - [E9QP59_MOUSE]	5.99	4	4	4	12	0.30	0.31	0.17	0.09
A2AQ92	Protein Duox1 OS=Mus musculus GN=Duox1 PE=4 SV=1 - [A2AQ92_MOUSE]	2.71	1	1	3	4	-0.68	-0.79	-0.19	-0.31
Q69ZF7	Metal transporter CNNM4 OS=Mus musculus GN=Cnnm4 PE=1 SV=2 - [CNNM4_MOUSE]	19.46	3	12	14	75	0.42	0.38	0.51	0.36
A2A5R2	Brefeldin A-inhibited guanine nucleotide-exchange protein 2 OS=Mus musculus GN=Arfgef2 PE=1 SV=1 - [BIG2_MOUSE]	4.63	1	6	8	17	0.22	0.38	0.16	0.47
G3X9B1	HEAT repeat containing 1 OS=Mus musculus GN=Heatr1 PE=4 SV=1 - [G3X9B1_MOUSE]	3.03	1	5	7	21	0.03	0.06	-0.17	-0.09
Q4VAE3	Transmembrane protein 65 OS=Mus musculus GN=Tmem65 PE=2 SV=1 - [TMM65_MOUSE]	5.98	1	1	1	2	0.09	0.10	0.14	0.15
O08912	Polypeptide N-acetylgalactosaminyltransferase 1 OS=Mus musculus GN=Galnt1 PE=1 SV=1 - [GALT1_MOUSE]	13.24	1	6	7	11	-0.14	0.06	-0.05	-0.01
Q9Z1N5	Spliceosome RNA helicase Ddx39b OS=Mus musculus GN=Ddx39b PE=1 SV=1 - [DX39B_MOUSE]	34.11	2	6	14	216	0.20	0.30	0.23	0.36
Q8BPM2-2	Isoform 2 of Mitogen-activated protein kinase kinase kinase kinase 5 OS=Mus musculus GN=Map4k5 - [M4K5_MOUSE]	2.78	3	2	2	4	0.31	0.49	0.39	0.57
Q9D939	Sulfotransferase 1C2 OS=Mus musculus GN=Sult1c2 PE=1 SV=1 - [ST1C2_MOUSE]	31.08	1	7	8	25	-0.29	-0.12	-0.28	-0.11
P11499	Heat shock protein HSP 90-beta OS=Mus musculus GN=Hsp90ab1 PE=1 SV=3 - [HS90B_MOUSE]	54.97	9	26	45	1387	0.38	0.39	0.39	0.40
Q9D6F9	Tubulin beta-4A chain OS=Mus musculus GN=Tubb4a PE=1 SV=3 - [TBB4A_MOUSE]	65.54	1	5	18	941	0.08	0.13	0.09	0.18

Q8CGC7	Bifunctional glutamate/proline--tRNA ligase OS=Mus musculus GN=Eprs PE=1 SV=4 - [SYEP_MOUSE]	37.96	1	51	53	289	0.43	0.44	0.37	0.41
Q7M6W1	RTN1-C OS=Mus musculus GN=Rtn1 PE=2 SV=1 - [Q7M6W1_MOUSE]	14.42	2	1	3	8	0.05	-0.16	0.04	-0.17
Q9JKF1	Ras GTPase-activating-like protein IQGAP1 OS=Mus musculus GN=Iqgap1 PE=1 SV=2 - [IQGA1_MOUSE]	56.37	1	72	77	942	0.34	0.40	0.31	0.41
Q5U430	E3 ubiquitin-protein ligase UBR3 OS=Mus musculus GN=Ubr3 PE=1 SV=3 - [UBR3_MOUSE]	2.12	6	5	5	5	0.27	0.36	0.44	0.52
J3QPB5	Nucleoporin NDC1 OS=Mus musculus GN=Tmem48 PE=4 SV=1 - [J3QPB5_MOUSE]	3.88	2	1	1	6	0.43	0.24	-0.10	-0.32
Q8K1G2	Limbin OS=Mus musculus GN=Evc2 PE=1 SV=1 - [LBN_MOUSE]	3.36	1	3	3	7	0.41	0.15	0.44	0.22
Q8K2T4	UPF0723 protein C11orf83 homolog OS=Mus musculus PE=2 SV=1 - [CK083_MOUSE]	10.11	1	1	1	1	-0.13	-0.40	0.55	0.27
Q91XC8	Death-associated protein 1 OS=Mus musculus GN=Dap PE=1 SV=3 - [DAP1_MOUSE]	24.51	1	1	1	19	0.24	0.39	0.19	0.35
Q99ME9	Nucleolar GTP-binding protein 1 OS=Mus musculus GN=Gtpbp4 PE=2 SV=3 - [NOG1_MOUSE]	3.94	1	3	3	11	0.16	0.04	0.58	0.49
Q8R173	Palmitoyltransferase ZDHHC3 OS=Mus musculus GN=Zdhhc3 PE=1 SV=1 - [ZDHC3_MOUSE]	5.35	1	1	1	3	0.51	0.48	0.41	0.38
O54774	AP-3 complex subunit delta-1 OS=Mus musculus GN=Ap3d1 PE=1 SV=1 - [AP3D1_MOUSE]	10.93	1	10	10	35	0.49	0.52	0.30	0.39
O08842	GDNF family receptor alpha-2 OS=Mus musculus GN=Gfra2 PE=2 SV=1 - [GFRA2_MOUSE]	3.24	6	2	2	4	-0.46	-0.50	-0.24	-0.29
D3Z2J6	Thioredoxin-related transmembrane protein 2 OS=Mus musculus GN=Tmx2 PE=2 SV=1 [D3Z2J6_MOUSE]	21.01	2	4	4	16	-0.26	-0.15	0.15	0.06
Q9JKA5	Cell surface A33 antigen OS=Mus musculus GN=Gpa33 PE=2 SV=2 - [GPA33_MOUSE]	16.61	1	4	4	125	0.62	0.76	0.58	0.61
P70255-5	Isoform 5 of Nuclear factor 1 C-type OS=Mus musculus GN=Nfic - [NFIC_MOUSE]	15.92	5	3	3	7	-0.06	-0.36	0.19	-0.12
Q9CQS8	Protein transport protein Sec61 subunit beta OS=Mus musculus GN=Sec61b PE=1 SV=3 - [SC61B_MOUSE]	21.88	2	2	2	34	0.24	0.12	0.14	0.01

A2AWA9	Rab GTPase-activating protein 1 OS=Mus musculus GN=Rabgap1 PE=2 SV=1 - [RBGP1_MOUSE]	1.97	4	3	3	3	0.00	-0.42	0.06	-0.37
P11928	2'-5'-oligoadenylate synthase 1A OS=Mus musculus GN=Oas1a PE=2 SV=2 - [OAS1A_MOUSE]	29.16	3	8	8	37	0.45	0.44	0.41	0.29
Q8R0G9	Nuclear pore complex protein Nup133 OS=Mus musculus GN=Nup133 PE=1 SV=2 - [NU133_MOUSE]	9.96	1	7	7	22	0.18	-0.22	0.12	-0.03
Q8R5J9	PRA1 family protein 3 OS=Mus musculus GN=Arl6ip5 PE=1 SV=2 - [PRAF3_MOUSE]	20.21	1	4	4	38	-0.09	-0.35	0.00	-0.46
Q2EMV9	Poly [ADP-ribose] polymerase 14 OS=Mus musculus GN=Parp14 PE=1 SV=3 - [PAR14_MOUSE]	6.11	2	10	11	24	0.18	-0.31	0.42	-0.05
Q9D024	Coiled-coil domain-containing protein 47 OS=Mus musculus GN=Ccdc47 PE=2 SV=2 - [CCD47_MOUSE]	21.74	4	9	10	52	0.44	0.50	0.36	0.55
Q9JI13-2	Isoform 2 of Something about silencing protein 10 OS=Mus musculus GN=Utp3 - [SAS10_MOUSE]	6.30	2	3	3	5	0.10	0.42	0.12	0.44
Q80YW5	B box and SPRY domain-containing protein OS=Mus musculus GN=Bspry PE=1 SV=1 - [BSPRY_MOUSE]	6.55	2	4	4	6	0.26	0.48	0.25	0.47
Q3TJM4	Centromere protein T OS=Mus musculus GN=Cenpt PE=2 SV=2 [CENPT_MOUSE]	4.08	1	1	1	1	0.05	0.04	-0.07	-0.07
Q9D311	Dual oxidase maturation factor 2 OS=Mus musculus GN=Duoxa2 PE=2 SV=1 - [DOXA2_MOUSE]	5.00	1	1	1	2	0.67	0.88	1.21	1.41
Q9EPK6	Nucleotide exchange factor SIL1 OS=Mus musculus GN=Sil1 PE=1 SV=2 - [SIL1_MOUSE]	8.39	1	3	4	11	0.46	0.41	0.52	0.46
D3YUJ6	Protein Gm7792 OS=Mus musculus GN=Gm7792 PE=4 SV=2 - [D3YUJ6_MOUSE]	1.25	4	1	1	2	0.13	0.07	0.54	0.48
Q91YL3	Uridine-cytidine kinase-like 1 OS=Mus musculus GN=Uckl1 PE=1 SV=1 - [UCKL1_MOUSE]	12.59	5	5	5	17	0.60	0.26	0.77	0.60
F7CBP1	Eukaryotic translation initiation factor 4 gamma 2 OS=Mus musculus GN=Eif4g2 PE=4 SV=1 - [F7CBP1_MOUSE]	19.24	6	14	15	45	0.49	0.51	0.45	0.46
Q8BRV5	Uncharacterized protein KIAA1671 OS=Mus musculus GN=Kiaa1671 PE=2 SV=1 - [K1671_MOUSE]	8.44	1	2	2	2	0.57	0.72	0.37	0.52

P10404	MLV-related proviral Env polyprotein OS=Mus musculus PE=2 SV=3 - [ENV1_MOUSE]	14.66	3	6	7	49	0.60	0.45	0.64	0.34
O70209	PDZ and LIM domain protein 3 OS=Mus musculus GN=Pdlim3 PE=1 SV=1 - [PDLI3_MOUSE]	69.62	1	16	17	472	0.09	0.10	0.10	0.13
Q91YS7	Dual-specificity mitogen-activated protein kinase kinase 2 OS=Mus musculus GN=Map2k2 PE=2 SV=1 - [Q91YS7_MOUSE]	31.50	3	6	10	66	0.38	0.35	0.18	0.39
Q922J9	Fatty acyl-CoA reductase 1 OS=Mus musculus GN=Far1 PE=1 SV=1 - [FACR1_MOUSE]	6.99	3	2	2	6	0.37	-0.23	0.88	0.28
P04441-2	Isoform Short of H-2 class II histocompatibility antigen gamma chain OS=Mus musculus GN=Cd74 - [HG2A_MOUSE]	32.09	2	5	5	30	0.57	0.59	0.47	0.52
F2Z3X1	Beta-1,4-galactosyltransferase 4 OS=Mus musculus GN=B4galt4 PE=2 SV=1 - [F2Z3X1_MOUSE]	7.32	2	1	1	3	0.27	0.44	0.00	0.16
B1AX95	Tumor protein p63-regulated gene 1-like protein OS=Mus musculus GN=Tprgl PE=2 SV=1 - [B1AX95_MOUSE]	30.43	2	3	3	7	0.00	0.00	0.16	0.09
A2CG63-2	Isoform 2 of AT-rich interactive domain-containing protein 4B OS=Mus musculus GN=Arid4b - [ARI4B_MOUSE]	2.61	2	3	3	3	-0.43	0.02	-0.54	-0.10
Q9ERK4	Exportin-2 OS=Mus musculus GN=Cse1l PE=2 SV=1 - [XPO2_MOUSE]	16.89	5	14	14	51	0.58	0.49	0.38	0.50
Q59J78	Mimitin, mitochondrial OS=Mus musculus GN=Ndufaf2 PE=2 SV=1 - [MIMIT_MOUSE]	38.69	1	7	7	26	-0.13	-0.10	-0.17	-0.18
P83887	Tubulin gamma-1 chain OS=Mus musculus GN=Tubg1 PE=1 SV=1 - [TBG1_MOUSE]	13.08	2	4	4	21	0.43	0.19	0.07	-0.29
Q9CR92	Coiled-coil domain-containing protein 96 OS=Mus musculus GN=Ccdc96 PE=2 SV=1 - [CCD96_MOUSE]	2.91	1	2	2	5	0.64	0.69	0.65	0.80
E9Q735	Protein Ube4a OS=Mus musculus GN=Ube4a PE=2 SV=1 - [E9Q735_MOUSE]	1.17	2	1	1	1	0.05	-0.26	0.42	0.11
P97742	Carnitine O-palmitoyltransferase 1, liver isoform OS=Mus musculus GN=Cpt1a PE=1 SV=4 - [CPT1A_MOUSE]	26.52	2	17	17	111	-0.15	-0.04	-0.17	-0.10

H3BLQ0	Serine/threonine-protein phosphatase 2A 65 kDa regulatory subunit A beta isoform OS=Mus musculus GN=Ppp2r1b PE=2 SV=1 - [H3BLQ0_MOUSE]	12.59	8	3	7	32	-0.02	-0.56	0.00	-0.35
Q921K8	Protein FAM115C OS=Mus musculus GN=Fam115c PE=2 SV=1 - [F115C_MOUSE]	7.18	1	7	7	10	0.11	0.28	0.00	0.18
Q9CYT6	Adenylyl cyclase-associated protein 2 OS=Mus musculus GN=Cap2 PE=1 SV=1 - [CAP2_MOUSE]	24.79	2	10	11	49	0.34	0.17	0.33	0.22
P97499	Telomerase protein component 1 OS=Mus musculus GN=Tep1 PE=1 SV=1 - [TEP1_MOUSE]	4.98	1	8	9	19	0.04	0.06	0.19	0.30
Q9Z2D0	Myotubularin-related protein 9 OS=Mus musculus GN=Mtmr9 PE=2 SV=2 - [MTMR9_MOUSE]	3.12	1	2	2	4	0.02	-0.20	0.23	0.02
P22892	AP-1 complex subunit gamma-1 OS=Mus musculus GN=Ap1g1 PE=1 SV=3 - [AP1G1_MOUSE]	18.86	2	14	17	52	0.35	0.44	0.33	0.33
Q8CFA1-4	Isoform 4 of Interleukin 1 receptor-associated kinase-like 2 OS=Mus musculus GN=Irak2 - [IRAK2_MOUSE]	2.71	4	1	1	1	-0.69	-1.38	-0.04	-0.74
F8VQ75	Kinesin-like protein KIF13A OS=Mus musculus GN=Kif13a PE=2 SV=1 - [F8VQ75_MOUSE]	8.86	1	1	12	19	-0.28	0.21	-0.37	0.13
G3X924	Plasmalemma vesicle associated protein OS=Mus musculus GN=Plvap PE=4 SV=1 - [G3X924_MOUSE]	23.74	2	8	10	34	-0.23	-0.36	-0.13	-0.24
Q99J27	Acetyl-coenzyme A transporter 1 OS=Mus musculus GN=Slc33a1 PE=1 SV=1 - [ACATN_MOUSE]	4.00	1	2	2	7	0.29	0.09	0.36	0.22
G3X9S2	Ectonucleotide pyrophosphatase/phosphodiesterase 1, isoform CRA_d OS=Mus musculus GN=Enpp1 PE=1 SV=1 - [G3X9S2_MOUSE]	18.56	4	14	14	55	0.11	0.22	0.02	0.12
Q62469	Integrin alpha-2 OS=Mus musculus GN=Itga2 PE=1 SV=2 - [ITA2_MOUSE]	19.19	1	19	20	87	0.20	0.12	0.16	0.08
Q8BMS7	Calcium-transporting ATPase type 2C member 1 OS=Mus musculus GN=Atp2c1 PE=2 SV=1 - [Q8BMS7_MOUSE]	6.28	11	4	4	7	0.15	0.13	0.24	0.22
Q9EP53-4	Isoform 4 of Hamartin OS=Mus musculus GN=Tsc1 - [TSC1_MOUSE]	6.97	4	4	4	7	-0.18	-0.47	0.26	-0.07

Q8BKX1-3	Isoform 3 of Brain-specific angiogenesis inhibitor 1-associated protein 2 OS=Mus musculus GN=Baiap2 - [BAIP2_MOUSE]	6.04	5	3	3	4	-0.58	0.02	-0.11	0.20
Q8BKE6	Cytochrome P450 20A1 OS=Mus musculus GN=Cyp20a1 PE=2 SV=1 - [CP20A_MOUSE]	23.59	2	9	9	21	0.22	0.26	0.26	0.41
Q9CXR1	Dehydrogenase/reductase SDR family member 7 OS=Mus musculus GN=Dhrs7 PE=2 SV=2 [DHRS7_MOUSE]	18.05	1	6	7	31	-0.30	-0.16	-0.04	-0.09
P58281-2	Isoform 2 of Dynamin-like 120 kDa protein, mitochondrial OS=Mus musculus GN=Opa1 - [OPA1_MOUSE]	21.77	5	19	19	62	0.21	0.18	0.29	0.19
O55229	Choline/ethanolamine kinase OS=Mus musculus GN=Chkb PE=1 SV=3 - [CHKB_MOUSE]	10.41	2	4	4	6	0.11	0.24	0.10	0.19
P70663	SPARC-like protein 1 OS=Mus musculus GN=Sparcl1 PE=1 SV=3 - [SPRL1_MOUSE]	20.00	1	9	9	20	-0.36	-0.51	-0.30	-0.38
Q9D571	MCG3887, isoform CRA_a OS=Mus musculus GN=Mtl5 PE=2 SV=1 - [Q9D571_MOUSE]	5.78	2	1	1	3	-0.33	-0.35	-0.18	-0.25
Q6TL19	Guanylate cyclase 2G OS=Mus musculus GN=Gucy2g PE=1 SV=1 - [GUC2G_MOUSE]	3.18	1	2	3	4	0.04	-0.15	0.15	-0.05
Q8VCE1	DnaJ homolog subfamily C member 28 OS=Mus musculus GN=Dnajc28 PE=2 SV=2 - [DJC28_MOUSE]	1.82	2	1	1	8	0.37	0.21	0.28	0.16
A2A863-2	Isoform 2 of Integrin beta-4 OS=Mus musculus GN=Itgb4 - [ITB4_MOUSE]	27.32	4	38	38	221	0.22	0.17	0.19	0.13
Q3ULJ0-2	Isoform 2 of Glycerol-3-phosphate dehydrogenase 1-like protein OS=Mus musculus GN=Gpd1l - [GPD1L_MOUSE]	33.13	4	11	13	85	0.16	0.12	0.20	0.22
Q9QZR5-4	Isoform 4 of Homeodomain-interacting protein kinase 2 OS=Mus musculus GN=Hipk2 - [HIPK2_MOUSE]	3.53	8	2	2	2	-0.01	0.74	0.01	0.76
F6TLV3	Fibronectin type-III domain-containing protein 3A (Fragment) OS=Mus musculus GN=Fndc3a PE=4 SV=1 - [F6TLV3_MOUSE]	9.88	3	6	6	16	0.40	0.41	0.09	0.48
Q91VD9	NADH-ubiquinone oxidoreductase 75 kDa subunit, mitochondrial OS=Mus musculus GN=Ndufs1 PE=1 SV=2 - [NDUS1_MOUSE]	50.48	1	30	30	384	0.04	-0.01	0.07	-0.04

Q8CC88	von Willebrand factor A domain-containing protein 8 OS=Mus musculus GN=Vwa8 PE=2 SV=2 - [VWA8_MOUSE]	29.87	3	43	44	120	0.04	-0.07	-0.01	-0.04
Q9Z1Q9	Valine--tRNA ligase OS=Mus musculus GN=Vars PE=2 SV=1 - [SYVC_MOUSE]	31.51	21	33	34	240	0.39	0.30	0.36	0.31
P09055	Integrin beta-1 OS=Mus musculus GN=Itgb1 PE=1 SV=1 - [ITB1_MOUSE]	35.09	1	26	26	487	0.10	0.14	0.11	0.13
Q922F4	Tubulin beta-6 chain OS=Mus musculus GN=Tubb6 PE=1 SV=1 - [TBB6_MOUSE]	58.17	1	7	16	608	0.31	0.27	0.38	0.19
Q61543	Golgi apparatus protein 1 OS=Mus musculus GN=Glg1 PE=1 SV=1 - [GSLG1_MOUSE]	20.26	4	24	24	100	0.25	0.25	0.24	0.18
P20065-2	Isoform Short of Thymosin beta-4 OS=Mus musculus GN=Tmsb4x - [TYB4_MOUSE]	45.45	4	4	5	24	0.04	0.75	-0.02	0.62
Q69ZI1-4	Isoform 4 of E3 ubiquitin-protein ligase SH3RF1 OS=Mus musculus GN=Sh3rf1 - [SH3R1_MOUSE]	4.72	5	1	1	1	0.12	0.19	-0.03	0.03
Q8VHQ9	Acyl-coenzyme A thioesterase 11 OS=Mus musculus GN=Acot11 PE=2 SV=1 - [ACO11_MOUSE]	18.86	6	7	7	26	0.30	0.36	0.16	0.15
Q8BVW3-3	Isoform 3 of Tripartite motif-containing protein 14 OS=Mus musculus GN=Trim14 - [TRI14_MOUSE]	12.36	3	2	2	4	0.13	0.03	0.26	-0.08
Q3UHD9-2	Isoform 2 of Arf-GAP with GTPase, ANK repeat and PH domain-containing protein 2 OS=Mus musculus GN=Agap2 - [AGAP2_MOUSE]	0.69	2	1	1	11	0.35	0.27	0.56	0.47
Q8R010	Aminoacyl tRNA synthase complex-interacting multifunctional protein 2 OS=Mus musculus GN=Aimp2 PE=1 SV=2 - [AIMP2_MOUSE]	24.06	2	6	6	26	0.27	0.37	0.24	0.26
Q3TBA3	Antigen peptide transporter 1 OS=Mus musculus GN=Tap1 PE=2 SV=1 - [Q3TBA3_MOUSE]	11.64	3	8	8	18	0.23	0.10	0.23	0.16
Q5RJG7-5	Isoform 5 of Isoprenoid synthase domain-containing protein OS=Mus musculus GN=Ispd - [ISPD_MOUSE]	3.34	2	1	1	1	0.69	-2.63	0.39	-2.93
Q8K119	Postmeiotic segregation increased 1 (S. cerevisiae) OS=Mus musculus GN=Pms1 PE=2 SV=1 - [Q8K119_MOUSE]	0.76	1	1	1	1	-0.02	-0.04	0.24	0.22
Q9R0M4	Podocalyxin OS=Mus musculus GN=Podxl PE=1 SV=2 - [PODXL_MOUSE]	16.70	1	5	5	22	-0.19	-0.41	-0.11	-0.28

E9PVU0	Unconventional myosin-VI OS=Mus musculus GN=Myo6 PE=2 SV=1 [E9PVU0_MOUSE]	40.68	3	1	48	218	0.55	0.16	0.68	0.29
Q9QXX8	Nuclear fragile X mental retardation-interacting protein 1 OS=Mus musculus GN=Nufip1 PE=1 SV=1 - [NUFP1_MOUSE]	2.07	1	1	1	1	0.73	0.79	0.62	0.68
Q6PAV2-2	Isoform 2 of Probable E3 ubiquitin-protein ligase HERC4 OS=Mus musculus GN=Herc4 - [HERC4_MOUSE]	8.96	2	7	7	19	0.14	0.09	0.24	0.24
D3Z108	Transmembrane and ubiquitin-like domain-containing protein 1 (Fragment) OS=Mus musculus GN=Tmub1 PE=2 SV=1 - [D3Z108_MOUSE]	5.39	3	1	1	5	0.18	0.11	0.31	0.24
P17426-2	Isoform B of AP-2 complex subunit alpha-1 OS=Mus musculus GN=Ap2a1 - [AP2A1_MOUSE]	16.34	3	7	15	53	0.24	0.41	0.03	0.23
Q2YDW2-2	Isoform 2 of Protein misato homolog 1 OS=Mus musculus GN=Msto1 - [MSTO1_MOUSE]	9.23	4	2	2	6	0.06	0.21	0.25	0.40
Q9R049	E3 ubiquitin-protein ligase AMFR OS=Mus musculus GN=Amfr PE=1 SV=2 - [AMFR_MOUSE]	11.98	2	3	3	3	-0.84	-0.79	-0.19	-0.14
Q8BTM9-4	Isoform 4 of RAS guanyl-releasing protein 4 OS=Mus musculus GN=Rasgrp4 - [GRP4_MOUSE]	1.56	6	1	1	1	-0.13	-0.04	-0.01	0.08
Q9DAA6-2	Isoform 2 of Exosome complex component CSL4 OS=Mus musculus GN=Exosc1 - [EXOS1_MOUSE]	9.09	2	1	1	1	-0.33	-0.20	0.22	0.35
Q9D2W5	GRAM domain-containing protein 3 OS=Mus musculus GN=Gramd3 PE=2 SV=1 - [Q9D2W5_MOUSE]	6.94	2	2	2	4	-0.08	-0.15	-0.09	-0.16
Q3TEK6	Dual adapter for phosphotyrosine and 3-phosphotyrosine and 3-phosphoinositide OS=Mus musculus GN=Dapp1 PE=2 SV=1 - [Q3TEK6_MOUSE]	7.53	2	1	1	1	-0.35	-0.49	-0.21	-0.35
H3BK76	Cellular tumor antigen p53 (Fragment) OS=Mus musculus GN=Trp73 PE=2 SV=1 [H3BK76_MOUSE]	4.23	6	1	1	1	0.35	0.05	0.55	0.25
E9Q586	Dynactin subunit 1 OS=Mus musculus GN=Dctn1 PE=2 SV=1 [E9Q586_MOUSE]	34.38	8	33	34	145	0.36	0.33	0.29	0.27
Q8BUE4	Apoptosis-inducing factor 2 OS=Mus musculus GN=Aifm2 PE=2 SV=1 - [AIFM2_MOUSE]	16.89	2	5	5	7	0.31	0.31	0.15	0.15

Q6Q899	Probable ATP-dependent RNA helicase DDX58 OS=Mus musculus GN=Ddx58 PE=1 SV=2 - [DDX58_MOUSE]	7.34	5	7	7	12	0.60	0.49	0.43	0.77
Q6PBC0	Cds2 protein OS=Mus musculus GN=Cds2 PE=2 SV=1 - [Q6PBC0_MOUSE]	14.73	3	1	1	8	-0.47	-0.28	-0.20	-0.02
Q9R1J0	Sterol-4-alpha-carboxylate 3-dehydrogenase, decarboxylating OS=Mus musculus GN=Nsdhl PE=2 SV=1 [NSDHL_MOUSE]	22.93	1	8	8	16	0.47	0.10	0.60	0.19
Q3TPE9-2	Isoform 2 of Ankyrin repeat and MYND domain-containing protein 2 OS=Mus musculus GN=Ankmy2 [ANKY2_MOUSE]	5.00	2	2	2	5	0.40	0.09	0.40	0.10
P00158	Cytochrome b OS=Mus musculus GN=Mt-Cyb PE=2 SV=1 - [CYB_MOUSE]	1.84	1	1	1	1	0.46	0.79	0.21	0.55
D3YZ20	DNA-directed RNA polymerase II subunit RPB11 OS=Mus musculus GN=Polr2j PE=2 SV=1 - [D3YZ20_MOUSE]	13.51	3	1	1	1	-0.11	0.47	0.48	1.05
Q8K154	Protein Ugt2b34 OS=Mus musculus GN=Ugt2b34 PE=2 SV=1 - [Q8K154_MOUSE]	21.24	5	10	11	52	-0.19	-0.05	-0.15	-0.11
Q6ZQA6	Immunoglobulin superfamily member 3 OS=Mus musculus GN=Igsf3 PE=1 SV=2 - [IGSF3_MOUSE]	1.59	1	2	2	3	-0.20	-0.34	0.06	-0.08
Q9WUK6	Zinc finger and BTB domain-containing protein 18 OS=Mus musculus GN=Zbtb18 PE=2 SV=1 - [ZBT18_MOUSE]	4.60	2	1	1	1	0.60	0.40	0.29	0.09
D3Z723	Nuclear transcription factor Y subunit beta (Fragment) OS=Mus musculus GN=Nfyb PE=2 SV=1 - [D3Z723_MOUSE]	7.55	2	1	1	1	0.36	0.16	0.63	0.43
F6VR53	Guanine nucleotide exchange factor DBS (Fragment) OS=Mus musculus GN=Mcf2l PE=4 SV=1 - [F6VR53_MOUSE]	5.33	29	2	3	3	-0.01	0.30	0.72	1.03
O70152	Dolichol-phosphate mannosyltransferase OS=Mus musculus GN=Dpm1 PE=2 SV=1 [DPM1_MOUSE]	27.69	3	8	8	45	-0.06	-0.08	0.04	-0.01
O88398	Advillin OS=Mus musculus GN=Avil PE=1 SV=2 - [AVIL_MOUSE]	5.13	1	3	5	12	0.12	-0.06	-0.05	-0.24
P26638	Serine--tRNA ligase, cytoplasmic OS=Mus musculus GN=Sars PE=2 SV=3 - [SYSC_MOUSE]	25.59	4	12	12	73	0.54	0.63	0.36	0.45

Q9CWL8	Beta-catenin-like protein 1 OS=Mus musculus GN=Ctnnbl1 PE=1 SV=1 - [CTBL1_MOUSE]	3.37	1	2	2	4	0.12	0.71	0.33	0.60
Q9Z109	V-set and immunoglobulin domain-containing protein 2 OS=Mus musculus GN=Vsig2 PE=2 SV=2 - [VSIG2_MOUSE]	9.15	2	2	2	5	0.17	0.48	0.21	0.52
Q8CES0	N-alpha-acetyltransferase 30 OS=Mus musculus GN=Naa30 PE=2 SV=2 - [NAA30_MOUSE]	9.89	2	3	3	10	-0.21	-0.17	-0.42	-0.10
Q80UM7	Mannosyl-oligosaccharide glucosidase OS=Mus musculus GN=Mogs PE=2 SV=1 - [MOGS_MOUSE]	19.30	1	12	12	40	0.05	0.16	0.04	-0.06
I7HLV2	60S ribosomal protein L10 (Fragment) OS=Mus musculus GN=Rpl10 PE=4 SV=1 [I7HLV2_MOUSE]	46.27	4	10	10	106	0.45	0.51	0.44	0.47
F7CAV3	Inositol polyphosphate 5-phosphatase OCRL-1 (Fragment) OS=Mus musculus GN=Ocrl PE=4 SV=1 - [F7CAV3_MOUSE]	4.87	3	1	1	1	-0.13	-0.54	0.22	-0.19
Q76MZ3	Serine/threonine-protein phosphatase 2A 65 kDa regulatory subunit A alpha isoform OS=Mus musculus GN=Ppp2r1a PE=1 SV=3 - [2AAA_MOUSE]	48.90	3	21	25	238	0.45	0.50	0.42	0.45
H3BLK9	Espin-like protein OS=Mus musculus GN=Espnl PE=2 SV=1 - [H3BLK9_MOUSE]	0.73	2	1	1	1	0.12	-0.07	0.03	-0.16
F7BWT7-2	Isoform 2 of Tetraspanin-15 OS=Mus musculus GN=Tspan15 - [TSN15_MOUSE]	5.83	2	1	1	2	0.17	0.38	0.27	0.48
Q3UW53	Protein Niban OS=Mus musculus GN=Fam129a PE=1 SV=2 - [NIBAN_MOUSE]	39.52	4	30	30	226	0.20	0.19	0.25	0.18
A2ANU3	Synapse differentiation-inducing gene protein 1 OS=Mus musculus GN=Syndig1 PE=1 SV=1 - [SYNG1_MOUSE]	17.05	2	2	2	2	-0.02	0.35	0.14	0.50
D6RCG2	Dolichyl-phosphate beta glucosyltransferase OS=Mus musculus GN=Alg5 PE=4 SV=1 - [D6RCG2_MOUSE]	29.76	2	2	2	10	0.13	0.44	0.19	0.20
E9Q8X5	Protein Zbtb10 OS=Mus musculus GN=Zbtb10 PE=4 SV=1 - [E9Q8X5_MOUSE]	4.41	1	2	2	5	-0.44	-0.67	-0.11	-0.34
Q6ZWU9	40S ribosomal protein S27 OS=Mus musculus GN=Rps27 PE=1 SV=3 - [RS27_MOUSE]	40.48	1	2	5	73	0.33	0.36	0.23	0.45

H3BJK2	Probable global transcription activator SNF2L2 OS=Mus musculus GN=Smarca2 PE=2 SV=1 - [H3BJK2_MOUSE]	5.31	11	3	7	22	-0.11	0.56	-0.21	0.46
A2BI14	Coiled-coil domain-containing protein 171 (Fragment) OS=Mus musculus GN=Ccdc171 PE=2 SV=1 - [A2BI14_MOUSE]	1.80	4	1	2	5	0.25	0.72	0.34	0.63
F8WIN2	AT-rich interactive domain-containing protein 3B OS=Mus musculus GN=Arid3b PE=2 SV=1 - [F8WIN2_MOUSE]	7.28	7	2	2	6	0.18	-0.87	0.22	-1.03
Q68EF0	Rab-3A-interacting protein OS=Mus musculus GN=Rab3ip PE=1 SV=1 - [RAB3I_MOUSE]	12.85	1	4	4	6	0.37	0.66	0.24	0.50
Q9D9M2-2	Isoform 2 of Ubiquitin carboxyl-terminal hydrolase 12 OS=Mus musculus GN=Usp12 - [UBP12_MOUSE]	5.20	4	1	1	2	-0.03	-0.20	0.13	-0.04
Q9R099	Transducin beta-like protein 2 OS=Mus musculus GN=Tbl2 PE=2 SV=2 - [TBL2_MOUSE]	25.34	3	9	9	15	0.09	-0.03	0.27	0.08
Q6PD26	GPI transamidase component PIG-S OS=Mus musculus GN=Pigs PE=1 SV=3 - [PIGS_MOUSE]	14.23	2	7	7	20	0.19	-0.15	0.19	0.16
Q80UK0	SEC14 domain and spectrin repeat-containing protein 1 OS=Mus musculus GN=Sestd1 PE=2 SV=1 - [SESD1_MOUSE]	12.50	1	6	7	17	-0.07	-0.12	0.06	-0.20
Q8CGK3	Lon protease homolog, mitochondrial OS=Mus musculus GN=Lonp1 PE=1 SV=2 - [LONM_MOUSE]	35.83	3	22	23	117	0.21	0.21	0.21	0.22
Q9JLN9	Serine/threonine-protein kinase mTOR OS=Mus musculus GN=Mtor PE=1 SV=2 - [MTOR_MOUSE]	5.45	5	10	11	24	-0.04	-0.05	0.13	-0.07
Q5FWK3	Rho GTPase-activating protein 1 OS=Mus musculus GN=Arhgap1 PE=1 SV=1 - [RHG01_MOUSE]	43.05	2	14	15	218	0.36	0.30	0.27	0.31
O54784	Death-associated protein kinase 3 OS=Mus musculus GN=Dapk3 PE=1 SV=1 - [DAPK3_MOUSE]	14.73	1	5	5	20	-0.05	0.02	-0.01	0.14
O88870	Bestrophin-1 OS=Mus musculus GN=Best1 PE=2 SV=3 - [BEST1_MOUSE]	5.08	1	2	2	3	0.36	0.43	0.22	0.29
K3W4T2	Uncharacterized protein OS=Mus musculus GN=Gm6632 PE=4 SV=1 - [K3W4T2_MOUSE]	4.73	1	1	1	2	-0.11	-0.06	-0.24	-0.19

O08914	Fatty-acid amide hydrolase 1 OS=Mus musculus GN=Faah PE=2 SV=1 - [FAAH1_MOUSE]	6.22	1	3	3	7	-0.21	-0.31	-0.03	0.07
P46735	Unconventional myosin-Ib OS=Mus musculus GN=Myo1b PE=2 SV=3 - [MYO1B_MOUSE]	17.80	6	13	17	62	0.00	0.01	0.05	0.07
P28843	Dipeptidyl peptidase 4 OS=Mus musculus GN=Dpp4 PE=1 SV=3 - [DPP4_MOUSE]	3.03	1	2	2	8	0.10	-0.36	0.83	0.36
Q61739	Integrin alpha-6 OS=Mus musculus GN=Itga6 PE=1 SV=3 - [ITA6_MOUSE]	26.21	3	25	25	125	-0.06	-0.08	0.03	0.02
P62849-2	Isoform 2 of 40S ribosomal protein S24 OS=Mus musculus GN=Rps24 - [RS24_MOUSE]	20.77	3	3	3	25	-0.06	0.04	-0.01	0.01
Q00558	Factor VIII intron 22 protein OS=Mus musculus GN=F8a1 PE=2 SV=1 - [F8I2_MOUSE]	3.68	2	1	1	2	0.40	-0.36	0.74	-0.03
O89103	Complement component C1q receptor OS=Mus musculus GN=Cd93 PE=1 SV=1 - [C1QR1_MOUSE]	3.57	1	2	2	5	0.00	0.10	-0.05	0.05
Q8C0C7	Phenylalanine--tRNA ligase alpha subunit OS=Mus musculus GN=Farsa PE=2 SV=1 - [SYFA_MOUSE]	37.80	3	15	15	66	0.36	0.22	0.33	0.04
Q8R1F1	Niban-like protein 1 OS=Mus musculus GN=Fam129b PE=1 SV=2 - [NIBL1_MOUSE]	32.84	2	18	18	71	0.41	0.53	0.43	0.55
Q8BH02	Torsin-4A OS=Mus musculus GN=Tor4a PE=2 SV=1 - [TOR4A_MOUSE]	4.46	1	2	2	3	0.34	-0.08	0.41	-0.01
Q8BH75-2	Isoform 2 of E3 ubiquitin-protein ligase NRDP1 OS=Mus musculus GN=Rnf41 - [RNF41_MOUSE]	6.19	2	1	1	1	0.00	0.01	0.23	0.23
Q61879	Myosin-10 OS=Mus musculus GN=Myh10 PE=1 SV=2 - [MYH10_MOUSE]	34.41	4	35	65	1302	-0.18	-0.15	-0.14	-0.15
Q9EP72	ER membrane protein complex subunit 7 OS=Mus musculus GN=Emc7 PE=2 SV=1 [EMC7_MOUSE]	24.07	1	4	4	9	0.12	0.33	0.11	0.47
O88874	Cyclin-K OS=Mus musculus GN=Ccnk PE=1 SV=3 - [CCNK_MOUSE]	4.33	2	1	1	1	0.23	0.07	0.51	0.36
O88736	3-keto-steroid reductase OS=Mus musculus GN=Hsd17b7 PE=2 SV=1 - [DHB7_MOUSE]	25.15	2	4	4	10	0.38	0.07	0.37	0.05
P83882	60S ribosomal protein L36a OS=Mus musculus GN=Rpl36a PE=2 SV=2 - [RL36A_MOUSE]	25.47	1	4	4	30	0.06	-0.01	0.05	0.00

Q9ET54-4	Isoform 4 of Palladin OS=Mus musculus GN=Palld - [PALLD_MOUSE]	35.73	7	24	25	448	0.15	0.21	0.14	0.19
Q62086	Serum paraoxonase/arylesterase 2 OS=Mus musculus GN=Pon2 PE=1 SV=2 - [PON2_MOUSE]	19.21	1	5	5	40	0.16	0.32	0.11	0.28
G5E8J4	Protein tyrosine phosphatase, non-receptor type 21, isoform CRA_a OS=Mus musculus GN=Ptpn21 PE=4 SV=1 - [G5E8J4_MOUSE]	3.23	2	2	2	2	0.84	0.57	0.69	0.42
Q8BMF5	Glutamate receptor ionotropic, kainate 4 OS=Mus musculus GN=Grik4 PE=2 SV=2 [GRIK4_MOUSE]	2.72	1	1	1	1	-1.46	0.58	-2.03	0.00
Q99J36	THUMP domain-containing protein 1 OS=Mus musculus GN=Thumpd1 PE=1 SV=1 - [THUM1_MOUSE]	28.86	1	6	6	12	0.29	-0.09	0.16	0.01
Q8VDI9	Alpha-1,2-mannosyltransferase ALG9 OS=Mus musculus GN=Alg9 PE=2 SV=1 - [ALG9_MOUSE]	6.87	4	3	3	6	0.16	-0.12	0.16	-0.08
Q8CFA2	Aminomethyltransferase, mitochondrial OS=Mus musculus GN=Amt PE=2 SV=1 - [GCST_MOUSE]	10.17	1	4	4	7	0.35	0.29	0.46	0.40
P70188-2	Isoform KAP3B of Kinesin-associated protein 3 OS=Mus musculus GN=Kifap3 - [KIFA3_MOUSE]	4.92	2	2	3	5	0.27	-0.48	0.53	-0.23
A2ASZ8	Calcium-binding mitochondrial carrier protein SCaMC-2 OS=Mus musculus GN=Slc25a25 PE=2 SV=1 - [SCMC2_MOUSE]	2.77	5	1	1	1	0.17	-0.55	-0.06	-0.79
Q9JL27	Galactoside 2-alpha-L-fucosyltransferase 2 OS=Mus musculus GN=Fut2 PE=2 SV=1 - [FUT2_MOUSE]	11.24	3	4	4	17	0.09	0.08	0.12	0.09
Q6NZR5	Protein Skiv2l OS=Mus musculus GN=Skiv2l PE=2 SV=1 - [Q6NZR5_MOUSE]	11.09	2	9	10	14	0.45	-0.10	0.31	-0.41
D3Z3F4	Metaxin-1 (Fragment) OS=Mus musculus GN=Mtx1 PE=4 SV=2 - [D3Z3F4_MOUSE]	18.06	9	6	6	14	0.42	0.56	0.18	0.40
Q9QZH6	Evolutionarily conserved signaling intermediate in Toll pathway, mitochondrial OS=Mus musculus GN=Ecsit PE=1 SV=2 - [ECSIT_MOUSE]	25.52	2	6	7	12	0.16	0.12	0.18	0.13
A2AMH5	Choline transporter-like protein 1 OS=Mus musculus GN=Slc44a1 PE=2 SV=1 - [A2AMH5_MOUSE]	5.64	6	3	3	15	0.13	-0.31	0.08	-0.28

Q9EPL8	Importin-7 OS=Mus musculus GN=Ipo7 PE=1 SV=2 - [IPO7_MOUSE]	12.81	1	12	12	41	0.36	0.14	0.26	0.16
Q8VDW0	ATP-dependent RNA helicase DDX39A OS=Mus musculus GN=Ddx39a PE=2 SV=1 - [DX39A_MOUSE]	33.49	3	5	13	139	0.31	0.70	0.51	0.75
Q8BH74	Nuclear pore complex protein Nup107 OS=Mus musculus GN=Nup107 PE=2 SV=1 - [NU107_MOUSE]	3.56	2	3	3	3	0.50	-0.04	0.81	0.27
Q9QZQ8	Core histone macro-H2A.1 OS=Mus musculus GN=H2afy PE=1 SV=3 - [H2AY_MOUSE]	51.34	2	15	16	135	-0.04	0.02	-0.06	0.02
P68404-2	Isoform Beta-II of Protein kinase C beta type OS=Mus musculus GN=Prkcb - [KPCB_MOUSE]	17.53	2	4	7	26	0.67	0.76	0.62	0.58
Q9R1E6	Ectonucleotide pyrophosphatase/phosphodiesterase family member 2 OS=Mus musculus GN=Enpp2 PE=1 SV=3 - [ENPP2_MOUSE]	1.04	4	1	1	1	0.95	1.33	0.70	1.08
P43137	Lithostathine-1 OS=Mus musculus GN=Reg1 PE=2 SV=1 - [LIT1_MOUSE]	24.24	1	3	3	16	1.47	1.94	1.45	1.75
Q8CGP2	Histone H2B type 1-P OS=Mus musculus GN=Hist1h2bp PE=1 SV=3 - [H2B1P_MOUSE]	67.46	11	4	12	1074	0.26	0.37	0.18	0.31
Q3T9X3	Dynamin-2 OS=Mus musculus GN=Dnm2 PE=2 SV=1 - [Q3T9X3_MOUSE]	30.81	15	15	25	97	0.21	0.37	0.24	0.42
Q8R323	Replication factor C subunit 3 OS=Mus musculus GN=Rfc3 PE=2 SV=1 - [RFC3_MOUSE]	12.64	1	4	4	14	0.16	0.20	0.12	-0.02
Q689Z5	Protein strawberry notch homolog 1 OS=Mus musculus GN=Sbno1 PE=1 SV=2 - [SBNO1_MOUSE]	4.03	3	1	4	5	0.30	0.60	0.14	0.44
Q7TRC9	Olfactory receptor 904 OS=Mus musculus GN=Olfr904 PE=3 SV=1 - [Q7TRC9_MOUSE]	5.48	1	1	1	1	0.10	-0.16	0.01	-0.25
O35239	Tyrosine-protein phosphatase non-receptor type 9 OS=Mus musculus GN=Ptpn9 PE=2 SV=2 [PTN9_MOUSE]	6.24	1	3	3	7	-0.14	-0.31	0.03	-0.08
Q8K3Z9	Nuclear envelope pore membrane protein POM 121 OS=Mus musculus GN=Pom121 PE=1 SV=2 - [PO121_MOUSE]	3.42	2	2	2	5	0.41	0.39	0.42	0.52
Q3TES0	IQ motif and SEC7 domain-containing protein 3 OS=Mus musculus GN=Iqsec3 PE=1 SV=1 - [IQEC3_MOUSE]	2.76	1	1	2	3	-0.39	-0.16	-0.16	0.07

Q8K4E0	Alstrom syndrome protein 1 homolog OS=Mus musculus GN=Alms1 PE=1 SV=2 - [ALMS1_MOUSE]	1.05	1	2	2	7	0.03	0.27	0.03	0.50
Q8C172	Ceramide synthase 6 OS=Mus musculus GN=Cers6 PE=1 SV=1 [CERS6_MOUSE]	3.39	2	2	2	3	-0.15	-1.11	0.01	-0.95
Q6PF93	Phosphatidylinositol 3-kinase catalytic subunit type 3 OS=Mus musculus GN=Pik3c3 PE=1 SV=1 - [PK3C3_MOUSE]	5.07	4	2	2	6	0.32	-0.11	0.29	-0.13
Q8CJ61	CKLF-like MARVEL transmembrane domain-containing protein 4 OS=Mus musculus GN=Cmtm4 PE=2 SV=1 - [CKLF4_MOUSE]	3.37	1	1	1	1	0.09	0.02	0.25	0.17
Q9QXI6	SGLT1 protein OS=Mus musculus GN=Slc5a1 PE=2 SV=1 - [Q9QXI6_MOUSE]	4.81	2	3	3	5	0.50	0.25	0.17	-0.12
Q924H9-2	Isoform 2 of ATP-dependent RNA helicase DQX1 OS=Mus musculus GN=Dqx1 - [DQX1_MOUSE]	4.65	2	1	1	2	-0.65	-0.90	-0.54	-0.79
Q61387	Cytochrome c oxidase subunit 7A-related protein, mitochondrial OS=Mus musculus GN=Cox7a2l PE=2 SV=1 - [COX7R_MOUSE]	18.92	3	2	2	3	-0.06	-0.65	0.01	-0.59
F7CFT1	AN1-type zinc finger protein 1 (Fragment) OS=Mus musculus GN=Zfand1 PE=2 SV=1 - [F7CFT1_MOUSE]	6.48	3	2	2	5	0.17	-0.07	0.21	-0.02
Q5RKZ7-2	Isoform Mocs1a of Molybdenum cofactor biosynthesis protein 1 OS=Mus musculus GN=Mocs1 - [MOCS1_MOUSE]	6.49	3	2	2	3	-0.27	-0.13	-0.14	-0.01
P51881	ADP/ATP translocase 2 OS=Mus musculus GN=Slc25a5 PE=1 SV=3 - [ADT2_MOUSE]	51.01	2	9	20	444	0.35	0.39	0.31	0.26
Q3V2J0	Protein FAM92B OS=Mus musculus GN=Fam92b PE=2 SV=2 - [FA92B_MOUSE]	6.51	2	2	2	2	0.22	0.21	0.21	0.20
Q8BL48	RING finger protein unkempt homolog OS=Mus musculus GN=Unk PE=1 SV=1 - [UNK_MOUSE]	4.20	2	2	2	5	-0.77	-1.01	-0.28	-0.53
Q3V3R4	Integrin alpha-1 OS=Mus musculus GN=Itga1 PE=2 SV=2 - [ITA1_MOUSE]	21.97	1	23	23	130	-0.21	-0.13	-0.15	-0.07
Q9Z176	Serine/threonine-protein phosphatase 2A regulatory subunit B'' subunit delta OS=Mus musculus GN=Ppp2r3d PE=2 SV=1 - [P2R3D_MOUSE]	7.33	1	2	2	3	-0.16	0.15	0.20	0.50

P48962	ADP/ATP translocase 1 OS=Mus musculus GN=Slc25a4 PE=1 SV=4 - [ADT1_MOUSE]	53.02	2	10	21	424	-0.05	0.05	-0.12	-0.02
P55096	ATP-binding cassette sub-family D member 3 OS=Mus musculus GN=Abcd3 PE=1 SV=2 - [ABCD3_MOUSE]	18.97	1	10	11	40	0.19	0.35	0.08	0.18
E0CYG3	Sperm flagellar protein 2 OS=Mus musculus GN=Spef2 PE=2 SV=1 [E0CYG3_MOUSE]	2.55	3	2	3	7	-0.44	0.02	-0.47	-0.16
P11881-8	Isoform 8 of Inositol 1,4,5-trisphosphate receptor type 1 OS=Mus musculus GN=Itpr1 - [ITPR1_MOUSE]	8.17	9	15	20	69	-0.01	-0.06	-0.03	-0.19
O88983	Syntaxin-8 OS=Mus musculus GN=Stx8 PE=1 SV=1 - [STX8_MOUSE]	22.46	2	5	5	19	-0.22	-0.18	0.32	0.29
E9Q7C3	Protein Plekhg4 OS=Mus musculus GN=Plekhg4 PE=2 SV=1 - [E9Q7C3_MOUSE]	1.35	2	1	1	3	0.26	-0.20	0.16	-0.30
Q7TRN4	Olfactory receptor 698 OS=Mus musculus GN=Olfr698 PE=3 SV=1 - [Q7TRN4_MOUSE]	2.25	1	1	1	2	0.16	0.15	0.27	0.26
Q80ZJ1	Ras-related protein Rap-2a OS=Mus musculus GN=Rap2a PE=1 SV=2 - [RAP2A_MOUSE]	25.68	3	1	3	6	-0.07	0.04	0.17	0.28
Q8BHE8	Uncharacterized protein C2orf47 homolog, mitochondrial OS=Mus musculus PE=2 SV=1 - [CB047_MOUSE]	11.68	1	3	3	8	-0.22	-0.12	-0.07	0.02
Q6NV83-3	Isoform 3 of U2 snRNP-associated SURP motif-containing protein OS=Mus musculus GN=U2surp - [SR140_MOUSE]	8.02	3	7	7	9	0.32	0.19	0.28	0.20
Q9Z110-2	Isoform Short of Delta-1-pyrroline-5-carboxylate synthase OS=Mus musculus GN=Aldh18a1 - [P5CS_MOUSE]	23.71	5	16	16	76	0.11	0.09	0.16	0.16
Q99J39-2	Isoform Cytoplasmic+peroxisomal of Malonyl-CoA decarboxylase, mitochondrial OS=Mus musculus GN=Mlycd - [DCMC_MOUSE]	14.32	2	5	5	23	-0.03	0.01	0.39	0.26
P68181-2	Isoform 2 of cAMP-dependent protein kinase catalytic subunit beta OS=Mus musculus GN=Prkacb - [KAPCB_MOUSE]	20.12	4	4	8	37	0.54	0.64	0.50	0.60
P99024	Tubulin beta-5 chain OS=Mus musculus GN=Tubb5 PE=1 SV=1 - [TBB5_MOUSE]	69.59	2	4	21	1068	0.27	0.38	0.32	0.43

D3YXP6	Phosphomevalonate kinase OS=Mus musculus GN=Pmvk PE=2 SV=1 - [D3YXP6_MOUSE]	22.15	4	3	3	4	1.01	0.40	0.95	0.34
Q8K337-3	Isoform 3 of Type II inositol 1,4,5-trisphosphate 5-phosphatase OS=Mus musculus GN=Inpp5b - [I5P2_MOUSE]	2.96	3	1	2	5	-0.64	-0.77	0.10	-0.52
Q9WVQ0	Polyamine-modulated factor 1-binding protein 1 OS=Mus musculus GN=Pmfbp1 PE=2 SV=1 - [PMFBP_MOUSE]	4.31	3	3	5	6	0.06	0.35	-0.13	0.15
Q99NB1	Acetyl-coenzyme A synthetase 2-like, mitochondrial OS=Mus musculus GN=Acss1 PE=1 SV=1 - [ACS2L_MOUSE]	18.48	1	12	12	44	0.05	-0.06	0.04	0.04
Q91YZ2	C-terminal binding protein 2, isoform CRA_b OS=Mus musculus GN=Ctbp2 PE=2 SV=1 - [Q91YZ2_MOUSE]	19.52	5	4	7	44	0.28	0.17	0.61	0.32
Q8CGY6-2	Isoform 2 of Protein unc-45 homolog B OS=Mu musculus GN=Unc45b [UN45B_MOUSE]	1.83	2	1	1	2	0.44	0.81	0.37	0.74
Q60952	Centrosome-associated protein CEP250 OS=Mus musculus GN=Cep250 PE=1 SV=4 - [CP250_MOUSE]	2.65	6	4	5	23	0.29	0.87	-0.57	0.01
Q5U464	Arf-GAP with SH3 domain, ANK repeat and PH domain-containing protein 3 OS=Mus musculus GN=Asap3 PE=2 SV=1 - [ASAP3_MOUSE]	7.96	1	2	2	14	-0.20	0.41	-0.05	0.55
Q3UMT1	Protein phosphatase 1 regulatory subunit 12C OS=Mus musculus GN=Ppp1r12c PE=1 SV=1 - [PP12C_MOUSE]	39.26	2	26	26	165	0.24	0.28	0.28	0.28
Q9Z0G0	PDZ domain-containing protein GIPC1 OS=Mu musculus GN=Gipc1 PE=1 SV=1 - [GIPC1_MOUSE]	20.72	1	4	4	8	-0.18	-0.12	0.26	0.32
Q8VE70	Programmed cell death protein 10 OS=Mus musculus GN=Pdcd10 PE=1 SV=1 - [PDC10_MOUSE]	32.55	3	7	7	33	0.61	0.47	0.48	0.58
Q8R3F5	Malonyl-CoA-acyl carrier protein transacylase, mitochondrial OS=Mus musculus GN=Mcat PE=2 SV=3 - [FABD_MOUSE]	17.32	1	5	5	10	-0.77	-0.61	-0.44	-0.28
P60762-2	Isoform 2 of Mortality factor 4-like protein 1 OS=Mus musculus GN=Morf4l1 - [MO4L1_MOUSE]	6.19	2	2	2	2	0.19	-0.54	-0.18	-0.90

Q62219-2	Isoform 2 of Transforming growth factor beta-1-induced transcript 1 protein OS=Mus musculus GN=Tgfb1i1 - [TGFI1_MOUSE]	25.23	11	6	6	199	0.03	0.18	0.07	0.15
P19783	Cytochrome c oxidase subunit 4 isoform 1, mitochondrial OS=Mus musculus GN=Cox4i1 PE=1 SV=2 - [COX41_MOUSE]	50.30	4	9	9	211	0.08	0.11	0.05	0.06
O54943	Period circadian protein homolog 2 OS=Mus musculus GN=Per2 PE=1 SV=3 - [PER2_MOUSE]	1.35	1	1	1	3	-0.69	-1.18	-0.11	-0.61
Q9CWQ2	Zinc finger SWIM domain-containing protein 7 OS=Mus musculus GN=Zswim7 PE=2 SV=1 - [ZSWM7_MOUSE]	7.89	1	1	1	1	-0.01	-0.11	-0.08	-0.18
O09111	NADH dehydrogenase [ubiquinone] 1 beta subcomplex subunit 11, mitochondrial OS=Mus musculus GN=Ndufb11 PE=1 SV=2 - [NDUBB_MOUSE]	61.59	1	5	5	72	0.07	0.17	0.00	0.12
Q99J95-2	Isoform 2 of Cyclin-dependent kinase 9 OS=Mus musculus GN=Cdk9 - [CDK9_MOUSE]	24.61	35	5	7	31	0.06	0.20	0.07	0.03
P14869	60S acidic ribosomal protein P0 OS=Mus musculus GN=Rplp0 PE=1 SV=3 - [RLA0_MOUSE]	41.96	3	12	13	200	0.56	0.62	0.54	0.55
Q8R2S1-2	Isoform 2 of Ribosomal protein S6 kinase-like 1 OS=Mus musculus GN=Rps6kl1 - [RPKL1_MOUSE]	4.99	2	1	2	3	-0.94	-0.75	-0.59	-0.40
Q9JMC8	Band 4.1-like protein 4B OS=Mus musculus GN=Epb41l4b PE=2 SV=2 - [E41LB_MOUSE]	15.94	2	4	4	6	0.03	-0.10	0.25	0.11
P57080	Ubiquitin carboxyl-terminal hydrolase 25 OS=Mus musculus GN=Usp25 PE=1 SV=2 - [UBP25_MOUSE]	1.61	1	1	2	11	0.07	-0.07	0.21	0.06
P61079	Ubiquitin-conjugating enzyme E2 D3 OS=Mus musculus GN=Ube2d3 PE=1 SV=1 - [UB2D3_MOUSE]	21.09	3	2	3	45	0.43	1.15	0.40	0.66
Q8BVE3	V-type proton ATPase subunit H OS=Mus musculus GN=Atp6v1h PE=1 SV=1 - [VATH_MOUSE]	22.36	1	7	7	27	0.21	0.37	0.12	0.25
Q8VDD8	WAS protein family homolog 1 OS=Mus musculus GN=Wash1 PE=2 SV=1 - [WASH1_MOUSE]	17.89	1	4	4	15	0.40	0.41	0.21	0.16
Q5SWT3	Solute carrier family 25 member 35 OS=Mus musculus GN=Slc25a35 PE=2 SV=2 - [S2535_MOUSE]	35.67	1	9	9	20	-0.05	-0.22	-0.07	-0.29

Q9ER64	Oxysterol-binding protein-related protein 5 OS=Mus musculus GN=Osbpl5 PE=2 SV=3 - [OSBL5_MOUSE]	2.29	3	2	2	3	-0.37	-0.16	0.07	0.28
Q8BNU0	Armadillo repeat-containing protein 6 OS=Mus musculus GN=Armc6 PE=2 SV=1 - [ARMC6_MOUSE]	4.27	2	2	2	8	0.24	0.33	0.38	0.38
Q9Z1Y4	Thyroid receptor-interacting protein 6 OS=Mus musculus GN=Trip6 PE=1 SV=1 - [TRIP6_MOUSE]	14.17	1	3	3	13	0.18	0.22	0.36	0.37
Q8BZW8	NHL repeat-containing protein 2 OS=Mus musculus GN=Nhlrc2 PE=2 SV=1 - [NHLC2_MOUSE]	14.07	1	9	10	42	0.20	0.30	0.25	0.13
Q9CR33	MANSC domain-containing protein 1 OS=Mus musculus GN=Mansc1 PE=2 SV=1 - [MANS1_MOUSE]	1.45	1	1	1	1	0.09	-0.10	0.19	0.00
Q8K3K8	Optineurin OS=Mus musculus GN=Optn PE=1 SV=1 - [OPTN_MOUSE]	5.31	1	3	3	5	-0.96	-0.78	-0.04	0.14
Q5I0W6	Methyltransferase like 7A2 OS=Mus musculus GN=Ubie PE=2 SV=1 - [Q5I0W6_MOUSE]	17.62	5	1	5	31	0.02	-0.08	-0.47	-0.57
Q9DBR7-2	Isoform 2 of Protein phosphatase 1 regulatory subunit 12A OS=Mus musculus GN=Ppp1r12a - [MYPT1_MOUSE]	41.83	1	1	44	390	-0.02	-0.17	0.63	0.55
Q8R180	ERO1-like protein alpha OS=Mus musculus GN=Ero1l PE=1 SV=2 - [ERO1A_MOUSE]	22.41	1	6	7	38	-0.02	0.03	0.11	0.17
Q9Z0S1	3'(2'),5'-bisphosphate nucleotidase 1 OS=Mus musculus GN=Bpnt1 PE=1 SV=2 - [BPNT1_MOUSE]	57.79	3	12	12	97	0.02	-0.03	0.05	-0.07
Q9R0A0	Peroxisomal membrane protein PEX14 OS=Mus musculus GN=Pex14 PE=1 SV=1 [PEX14_MOUSE]	5.59	2	2	2	5	0.26	-0.10	0.70	0.20
A2APY7	NADH dehydrogenase [ubiquinone] 1 alpha subcomplex assembly factor 5 OS=Mus musculus GN=Ndufaf5 PE=2 SV=1 - [NDUF5_MOUSE]	8.45	2	3	3	3	-0.48	-0.28	-0.36	-0.17
E9PXV7	Protein diaphanous homolog 1 OS=Mus musculus GN=Diap1 PE=2 SV=1 - [E9PXV7_MOUSE]	13.23	5	17	17	46	0.29	0.34	0.18	0.26
Q9DBG6	Dolichyl-diphosphooligosaccharide--protein glycosyltransferase subunit 2 OS=Mus musculus GN=Rpn2 PE=2 SV=1 - [RPN2_MOUSE]	34.23	2	15	15	126	0.18	0.24	0.14	0.22

P63254	Cysteine-rich protein 1 OS=Mus musculus GN=Crip1 PE=2 SV=2 - [CRIP1_MOUSE]	67.53	1	4	5	136	-0.07	-0.18	-0.10	-0.16
Q6A0A9	Constitutive coactivator of PPAR-gamma-like protein 1 OS=Mus musculus GN=FAM120A PE=1 SV=2 - [F120A_MOUSE]	23.02	1	17	19	81	0.05	0.04	0.14	0.18
E9Q8E3	Protein Abi3bp OS=Mus musculus GN=Abi3bp PE=2 SV=1 - [E9Q8E3_MOUSE]	8.69	4	6	6	17	0.18	0.27	0.11	0.28
G3UXN0	CTD small phosphatase-like protein OS=Mus musculus GN=Ctdspl PE=2 SV=1 - [G3UXN0_MOUSE]	9.81	2	1	2	5	-0.03	0.19	-0.01	0.21
Q99LC8	Translation initiation factor eIF-2B subunit alpha OS=Mus musculus GN=Eif2b1 PE=2 SV=1 - [EI2BA_MOUSE]	23.61	2	6	6	18	0.49	0.20	0.52	0.41
M0QWD1	Eukaryotic translation initiation factor 4E type 2 (Fragment) OS=Mus musculus GN=Eif4e2 PE=3 SV=1 - [M0QWD1_MOUSE]	13.28	8	2	2	2	0.78	0.74	0.77	0.73
Q91ZR1	Ras-related protein Rab-4B OS=Mus musculus GN=Rab4b PE=2 SV=2 - [RAB4B_MOUSE]	20.66	11	2	4	35	-0.05	-0.44	0.14	-0.29
P97314	Cysteine and glycine-rich protein 2 OS=Mus musculus GN=Csrp2 PE=1 SV=3 - [CSRP2_MOUSE]	61.66	1	8	9	28	0.02	0.20	0.22	0.48
Q8VCH6	Delta(24)-sterol reductase OS=Mus musculus GN=Dhcr24 PE=2 SV=1 - [DHC24_MOUSE]	6.40	1	4	4	8	0.19	0.10	0.35	-0.06
Q9DCG6	Phenazine biosynthesis-like domain-containing protein 1 OS=Mus musculus GN=Pbld1 PE=2 SV=2 - [PBLD1_MOUSE]	14.93	2	1	4	8	-1.06	-0.83	-1.13	-0.90
O55100-2	Isoform 1B of Synaptogyrin-1 OS=Mus musculus GN=Syngr1 - [SNG1_MOUSE]	6.28	2	1	1	3	-0.44	-0.90	-0.23	-0.69
P62267	40S ribosomal protein S23 OS=Mus musculus GN=Rps23 PE=2 SV=3 - [RS23_MOUSE]	46.15	1	6	7	44	0.47	0.38	0.39	0.52
Q91XC9	Peroxisomal membrane protein PEX16 OS=Mus musculus GN=Pex16 PE=2 SV=2 [PEX16_MOUSE]	10.71	1	2	2	9	-0.48	-0.19	-0.10	0.18
E9QML5	Zinc finger protein 638 OS=Mus musculus GN=Zfml PE=2 SV=1 - [E9QML5_MOUSE]	4.05	8	5	6	11	-0.07	-0.20	0.11	0.03

D6RIK1	Myotubularin-related protein 11 OS=Mus musculus GN=Mtmr11 PE=2 SV=1 - [D6RIK1_MOUSE]	7.69	2	1	2	3	-0.18	0.10	0.07	0.34
P59672	Ankyrin repeat and SAM domain-containing protein 1A OS=Mus musculus GN=Anks1a PE=1 SV=3 - [ANS1A_MOUSE]	2.78	3	2	3	12	0.07	0.35	0.28	0.37
D3YUI0	MCG130577 OS=Mus musculus GN=Vmn2r24 PE=3 SV=2 - [D3YUI0_MOUSE]	4.57	2	2	2	2	0.04	0.71	0.20	0.87
P50540-2	Isoform Short of Max-interacting protein 1 OS=Mus musculus GN=Mxi1 - [MXI1_MOUSE]	6.77	3	1	1	1	-0.20	-0.38	-0.08	-0.25
Q9D662	Protein transport protein Sec23B OS=Mus musculus GN=Sec23b PE=2 SV=1 - [SC23B_MOUSE]	23.47	2	12	15	147	0.21	0.26	0.18	0.27
Q62313	Trans-Golgi network integral membrane protein 1 OS=Mus musculus GN=Tgoln1 PE=1 SV=1 - [TGON1_MOUSE]	28.33	2	7	7	24	0.57	0.74	0.53	0.50
Q9ERN0	Secretory carrier-associated membrane protein 2 OS=Mus musculus GN=Scamp2 PE=2 SV=1 - [SCAM2_MOUSE]	10.03	1	3	3	26	-0.15	0.14	0.40	0.10
Q5SFM8-2	Isoform 2 of RNA-binding protein 27 OS=Mus musculus GN=Rbm27 - [RBM27_MOUSE]	7.96	3	5	7	14	-0.22	-0.09	0.30	0.38
Q9CXJ1	Probable glutamate--tRNA ligase, mitochondrial OS=Mus musculus GN=Ears2 PE=2 SV=1 - [SYEM_MOUSE]	13.96	1	5	6	13	0.61	0.47	0.55	0.44
Q571H0	Nucleolar pre-ribosomal associated protein 1 OS=Mus musculus GN=Urb1 PE=2 SV=2 - [NPA1P_MOUSE]	2.20	6	2	3	10	0.32	0.39	-0.11	-0.04
Q9CQW1	Synaptobrevin homolog YKT6 OS=Mus musculus GN=Ykt6 PE=2 SV=1 - [YKT6_MOUSE]	41.92	1	7	7	57	0.02	0.09	0.07	0.16
E9PUM3	Phosphorylase OS=Mus musculus GN=Pygm PE=2 SV=1 - [E9PUM3_MOUSE]	38.06	2	18	27	183	0.03	0.11	0.06	0.20
P47809	Dual specificity mitogen-activated protein kinase kinase 4 OS=Mus musculus GN=Map2k4 PE=1 SV=2 - [MP2K4_MOUSE]	16.37	3	3	3	14	0.43	0.34	0.39	0.48
E9PYI3	1-phosphatidylinositol 4,5-bisphosphate phosphodiesterase beta-2 OS=Mus musculus GN=Plcb2 PE=2 SV=1 - [E9PYI3_MOUSE]	1.21	2	1	1	1	-0.11	-0.05	0.82	0.88

D3Z7Q8	DNA-directed RNA polymerases I and III subunit RPAC1 (Fragment) OS=Mus musculus GN=Polr1c PE=2 SV=2 - [D3Z7Q8_MOUSE]	7.45	5	2	2	4	0.38	0.52	0.48	0.62
Q91UZ1	Phospholipase C beta 4 OS=Mus musculus GN=Plcb4 PE=2 SV=1 - [Q91UZ1_MOUSE]	11.49	2	9	10	16	-0.30	0.01	0.00	-0.16
Q80YQ2	Mediator of RNA polymerase II transcription subunit 23 OS=Mus musculus GN=Med23 PE=1 SV=2 - [MED23_MOUSE]	1.76	5	1	2	2	-0.12	-0.28	-0.11	-0.27
Q6ZQB6-2	Isoform 2 of Inositol hexakisphosphate and diphosphoinositol-pentakisphosphate kinase 2 OS=Mus musculus GN=Ppip5k2 - [VIP2_MOUSE]	10.11	5	4	8	14	0.22	-0.22	0.34	0.16
P61089	Ubiquitin-conjugating enzyme E2 N OS=Mus musculus GN=Ube2n PE=1 SV=1 - [UBE2N_MOUSE]	53.29	2	7	7	99	0.47	0.07	0.46	0.19
J3QN09	Arginyl aminopeptidase (Aminopeptidase B)-like 1, isoform CRA_a OS=Mus musculus GN=Rnpepl1 PE=4 SV=1 - [J3QN09_MOUSE]	4.25	3	2	2	4	0.06	-0.01	0.13	0.05
Q91ZW3	SWI/SNF-related matrix-associated actin-dependent regulator of chromatin subfamily A member 5 OS=Mus musculus GN=Smarca5 PE=1 SV=1 - [SMCA5_MOUSE]	19.98	4	21	21	40	0.43	0.30	0.44	0.31
P54822	Adenylosuccinate lyase OS=Mus musculus GN=Adsl PE=2 SV=2 - [PUR8_MOUSE]	20.87	5	8	8	44	0.36	0.25	0.27	0.27
Q920S1	Granzyme N OS=Mus musculus GN=Gzmn PE=2 SV=1 - [Q920S1_MOUSE]	9.27	1	1	1	1	-0.41	-0.55	-0.02	-0.16
Q61102	ATP-binding cassette sub-family B member 7, mitochondrial OS=Mus musculus GN=Abcb7 PE=1 SV=3 - [ABCB7_MOUSE]	8.64	1	6	6	18	-0.01	0.25	0.12	0.08
Q6P4T2	U5 small nuclear ribonucleoprotein 200 kDa helicase OS=Mus musculus GN=Snrnp200 PE=2 SV=1 - [U520_MOUSE]	26.31	1	43	43	173	0.32	0.13	0.28	0.21
Q9QXB9	Developmentally-regulated GTP-binding protein 2 OS=Mus musculus GN=Drg2 PE=1 SV=1 - [DRG2_MOUSE]	10.99	1	3	4	9	0.42	0.69	0.19	0.48

Q9WV55	Vesicle-associated membrane protein-associated protein A OS=Mus musculus GN=Vapa PE=1 SV=2 - [VAPA_MOUSE]	46.59	1	11	12	144	0.30	0.39	0.35	0.37
Q68FL6	Methionine--tRNA ligase, cytoplasmic OS=Mus musculus GN=Mars PE=2 SV=1 - [SYMC_MOUSE]	22.17	3	16	16	58	0.25	0.24	0.30	0.23
Q80UU9	Membrane-associated progesterone receptor component 2 OS=Mus musculus GN=Pgrmc2 PE=1 SV=2 - [PGRC2_MOUSE]	47.93	1	6	8	74	0.12	0.14	0.23	0.21
Q6P3E7	Histone deacetylase 10 OS=Mus musculus GN=Hdac10 PE=2 SV=2 - [HDA10_MOUSE]	2.85	1	1	1	4	-0.42	-0.13	-0.18	0.11
Q8K199	COX assembly mitochondrial protein 2 homolog OS=Mus musculus GN=Cmc2 PE=2 SV=1 - [COXM2_MOUSE]	12.66	1	1	1	2	0.22	0.38	0.40	0.56
O88738-3	Isoform 3 of Baculoviral IAP repeat-containing protein 6 OS=Mus musculus GN=Birc6 - [BIRC6_MOUSE]	5.26	4	14	16	45	0.12	-0.29	0.06	-0.06
Q6DID5	PWWP domain-containing protein MUM1 OS=Mus musculus GN=Mum1 PE=2 SV=1 - [MUM1_MOUSE]	7.92	2	3	3	3	-0.04	0.41	0.09	0.53
Q8C163	Nuclease EXOG, mitochondrial OS=Mus musculus GN=Exog PE=2 SV=1 - [EXOG_MOUSE]	26.90	6	5	6	19	-0.46	-0.66	-0.20	-0.39
Q99JB8	Protein kinase C and casein kinase II substrate protein 3 OS=Mus musculus GN=Pacsin3 PE=1 SV=1 - [PACN3_MOUSE]	5.90	7	2	2	8	-0.34	0.27	-0.09	0.51
D3YUW8	Pogo transposable element with ZNF domain OS=Mus musculus GN=Pogz PE=2 SV=1 - [D3YUW8_MOUSE]	1.52	4	1	2	3	0.25	0.00	0.05	-0.20
Q3UYV9	Nuclear cap-binding protein subunit 1 OS=Mus musculus GN=Ncbp1 PE=1 SV=2 - [NCBP1_MOUSE]	10.25	1	6	6	11	0.58	0.58	0.87	0.48
E9QP46	Nesprin-2 OS=Mus musculus GN=Syne2 PE=2 SV=1 - [E9QP46_MOUSE]	3.32	16	18	21	41	0.38	0.15	0.45	0.18
P28663	Beta-soluble NSF attachment protein OS=Mus musculus GN=Napb PE=1 SV=2 - [SNAB_MOUSE]	11.74	2	2	4	7	-0.57	-0.39	-0.08	0.10
Q9D892	Inosine triphosphate pyrophosphatase OS=Mus musculus GN=Itpa PE=1 SV=2 - [ITPA_MOUSE]	31.82	1	4	4	59	0.23	0.14	0.31	0.21

Q9CQ19	Myosin regulatory light polypeptide 9 OS=Mus musculus GN=Myl9 PE=1 SV=3 - [MYL9_MOUSE]	83.72	2	6	14	1385	-0.23	-0.21	-0.21	-0.18
Q9Z0L8-2	Isoform II of Gamma-glutamyl hydrolase OS=Mus musculus GN=Ggh - [GGH_MOUSE]	37.46	2	11	11	69	0.42	0.02	0.48	0.17
F6WZ83	Ras-specific guanine nucleotide-releasing factor RalGPS1 (Fragment) OS=Mus musculus GN=Ralgps1 PE=4 SV=1 - [F6WZ83_MOUSE]	6.19	4	1	1	1	-0.16	-0.41	0.11	-0.14
O55060	Thiopurine S-methyltransferase OS=Mus musculus GN=Tpmt PE=1 SV=1 - [TPMT_MOUSE]	22.50	6	5	5	10	-0.01	-0.23	0.21	-0.02
P52760	Ribonuclease UK114 OS=Mus musculus GN=Hrsp12 PE=1 SV=3 - [UK114_MOUSE]	55.56	1	6	7	33	0.82	0.71	0.74	0.48
E9QAQ3	Rho GTPase-activating protein 26 OS=Mus musculus GN=Arhgap26 PE=2 SV=1 - [E9QAQ3_MOUSE]	2.64	4	2	2	3	-0.03	0.01	-0.01	0.08
F8WIV2	Serpin B6 OS=Mus musculus GN=Serpinb6a PE=2 SV=1 - [F8WIV2_MOUSE]	57.89	12	20	21	502	-0.11	-0.03	-0.14	-0.08
P47212	Galanin peptides OS=Mus musculus GN=Gal PE=2 SV=1 - [GALA_MOUSE]	12.90	1	1	1	3	0.01	-0.16	0.15	-0.02
D3Z7H8	Protein Cilp2 OS=Mus musculus GN=Cilp2 PE=4 SV=1 - [D3Z7H8_MOUSE]	0.52	2	1	1	5	0.22	0.02	0.38	-0.02
P22437	Prostaglandin G/H synthase 1 OS=Mus musculus GN=Ptgs1 PE=2 SV=1 - [PGH1_MOUSE]	21.10	1	9	9	38	-0.11	-0.16	-0.02	-0.26
Q9D8W5	26S proteasome non-ATPase regulatory subunit 12 OS=Mus musculus GN=Psmd12 PE=1 SV=4 - [PSD12_MOUSE]	29.17	3	11	12	47	0.30	0.22	0.47	0.44
Q69Z38	Pseudopodium-enriched atypical kinase 1 OS=Mus musculus GN=Peak1 PE=1 SV=4 [PEAK1_MOUSE]	2.19	1	3	3	3	-0.10	-0.28	0.07	-0.11
Q9EPE9	Probable cation-transporting ATPase 13A1 OS=Mus musculus GN=Atp13a1 PE=1 SV=2 - [AT131_MOUSE]	8.58	1	6	7	29	0.39	0.09	0.85	0.53
K7N652	Protein Vmn2r50 OS=Mus musculus GN=Vmn2r50 PE=3 SV=1 - [K7N652_MOUSE]	4.38	9	2	3	5	0.75	0.71	0.44	0.39
Q8BHH8	Clarin-3 OS=Mus musculus GN=Clrn3 PE=2 SV=1 - [CLRN3_MOUSE]	3.54	1	1	1	1	0.68	0.11	0.63	0.06

Q3UF95	Large proline-rich protein BAG6 OS=Mus musculus GN=Bag6 PE=2 SV=1 - [Q3UF95_MOUSE]	15.67	7	9	12	54	0.25	0.24	0.28	0.26
Q8K4L4	Protein POF1B OS=Mus musculus GN=Pof1b PE=1 SV=3 [POF1B_MOUSE]	12.61	1	7	7	13	-0.29	-0.42	-0.33	-0.29
Q9JKJ9	24-hydroxycholesterol 7 alpha-hydroxylase OS=Mus musculus GN=Cyp39a1 PE=2 SV=1 - [CP39A_MOUSE]	6.81	1	3	3	3	-0.41	-0.27	-0.06	0.08
P21995	Embigin OS=Mus musculus GN=Emb PE=1 SV=2 - [EMB_MOUSE]	2.73	1	1	1	5	0.53	0.65	0.48	0.59
G3X920	Armadillo repeat containing 8, isoform CRA_b OS=Mus musculus GN=Armc8 PE=4 SV=1 - [G3X920_MOUSE]	1.34	2	1	1	3	0.09	0.11	0.31	0.39
G5E8C3	G protein-coupled receptor, family C, group 5, member A OS=Mus musculus GN=Gprc5a PE=4 SV=1 - [G5E8C3_MOUSE]	8.71	2	2	2	9	0.55	0.32	0.45	0.31
Q8VCX5	Calcium uptake protein 1, mitochondrial OS=Mus musculus GN=Micu1 PE=1 SV=1 - [MICU1_MOUSE]	15.51	5	7	7	26	0.29	0.37	0.26	0.33
Q3TIR3	Synembryn-A OS=Mus musculus GN=Ric8a PE=1 SV=2 - [RIC8A_MOUSE]	9.06	1	3	4	18	-0.07	0.21	0.07	0.35
A8C756	Thyroid adenoma-associated protein homolog OS=Mus musculus GN=Thada PE=2 SV=1 - [THADA_MOUSE]	2.06	3	4	4	7	0.04	0.03	-0.28	0.13
Q8R3U1	HRAS-like suppressor 3 OS=Mus musculus GN=Pla2g16 PE=1 SV=2 - [HRSL3_MOUSE]	10.49	3	2	2	5	0.58	0.59	0.52	0.49
D3YTN4	GTPase IMAP family member 4 OS=Mus musculus GN=Gimap4 PE=2 SV=1 - [D3YTN4_MOUSE]	19.21	4	5	5	14	0.27	0.50	0.16	0.43
Q7TSG2	RNA polymerase II subunit A C-terminal domain phosphatase OS=Mus musculus GN=Ctdp1 PE=1 SV=1 [CTDP1_MOUSE]	8.33	3	6	6	16	0.15	0.08	0.34	0.11
O54950	5'-AMP-activated protein kinase subunit gamma-1 OS=Mus musculus GN=Prkag1 PE=1 SV=2 - [AAKG1_MOUSE]	5.76	1	2	2	2	0.15	0.10	0.68	0.38
D3Z001	Protein 2310057J18Rik OS=Mus musculus GN=2310057J18Rik PE=4 SV=1 - [D3Z001_MOUSE]	10.16	2	1	1	3	0.66	0.76	0.47	0.81

Q91V24	ATP-binding cassette sub-family A member 7 OS=Mus musculus GN=Abca7 PE=1 SV=1 - [ABCA7_MOUSE]	5.47	2	5	7	11	-0.40	-0.25	-0.66	-0.51
Q8K0B2-3	Isoform 3 of Probable lysosomal cobalamin transporter OS=Mus musculus GN=Lmbrd1 - [LMBD1_MOUSE]	13.28	3	2	2	5	-0.09	-0.31	0.17	-0.05
D3Z166	Alpha-1,3-mannosyl-glycoprotein 4-beta-N-acetylglucosaminyltransferase A OS=Mus musculus GN=Mgat4a PE=2 SV=1 - [D3Z166_MOUSE]	1.76	3	1	1	3	-0.08	-0.21	0.04	-0.37
Q3UED7	Interferon-gamma-inducible GTPase Ifgga2 protein OS=Mus musculus GN=Gm4951 PE=2 SV=1 - [Q3UED7_MOUSE]	10.84	1	2	3	33	-0.03	-0.15	0.03	-0.08
Q9Z1G4-3	Isoform A1-III of V-type proton ATPase 116 kDa subunit a isoform 1 OS=Mus musculus GN=Atp6v0a1 - [VPP1_MOUSE]	17.91	5	12	13	37	0.00	0.12	0.08	0.17
P70671	Interferon regulatory factor 3 OS=Mus musculus GN=Irf3 PE=1 SV=1 - [IRF3_MOUSE]	14.08	1	4	4	9	0.45	0.38	0.30	0.23
Q9D8Z1	Activating signal cointegrator 1 complex subunit 1 OS=Mus musculus GN=Ascc1 PE=2 SV=1 - [ASCC1_MOUSE]	5.06	1	2	2	5	0.32	0.37	0.37	0.44
G3UYY2	Selenium-binding protein 2 OS=Mus musculus GN=Selenbp2 PE=2 SV=1 - [G3UYY2_MOUSE]	58.54	1	2	18	642	-0.44	-0.46	-0.13	-0.15
E9QQ05	Clathrin coat assembly protein AP180 OS=Mus musculus GN=Snap91 PE=4 SV=2 - [E9QQ05_MOUSE]	9.10	8	5	7	19	-0.28	0.00	-0.13	-0.01
F8VQC9	NBCn1-G OS=Mus musculus GN=Slc4a7 PE=2 SV=1 - [F8VQC9_MOUSE]	6.90	3	6	7	20	0.22	0.39	-0.08	0.16
F6XTP0	RING finger protein 121 (Fragment) OS=Mus musculus GN=Rnf121 PE=4 SV=1 - [F6XTP0_MOUSE]	10.00	2	1	1	6	-0.23	-0.34	0.28	0.17
Q91WT9-2	Isoform 2 of Cystathionine beta-synthase OS=Mus musculus GN=Cbs - [CBS_MOUSE]	22.12	5	9	9	40	0.11	-0.13	0.15	0.06
F2Z4A3	Protein Fat1 OS=Mus musculus GN=Fat1 PE=2 SV=1 - [F2Z4A3_MOUSE]	2.51	2	8	9	13	0.17	-0.01	0.12	-0.14
A6ZI47	Fructose-bisphosphate aldolase OS=Mus musculus GN=Aldoart2 PE=2 SV=1 - [A6ZI47_MOUSE]	23.90	1	1	13	312	0.13	0.11	0.20	0.17

P97315	Cysteine and glycine-rich protein 1 OS=Mus musculus GN=Csrp1 PE=1 SV=3 - [CSRP1_MOUSE]	68.39	2	13	14	647	0.41	0.58	0.36	0.46
Q8VI75	Importin-4 OS=Mus musculus GN=Ipo4 PE=1 SV=1 - [IPO4_MOUSE]	8.60	3	8	8	15	-0.02	0.14	-0.02	0.15
Q9DCR2	AP-3 complex subunit sigma-1 OS=Mus musculus GN=Ap3s1 PE=1 SV=2 - [AP3S1_MOUSE]	18.65	1	3	3	21	0.03	-0.04	0.30	0.18
Q922K9	Tyrosine-protein kinase FRK OS=Mus musculus GN=Frk PE=1 SV=3 - [FRK_MOUSE]	10.35	1	2	4	8	-0.08	-0.03	-0.08	-0.06
Q3UMR0-2	Isoform 2 of Ankyrin repeat domain-containing protein 27 OS=Mus musculus GN=Ankrd27 - [ANR27_MOUSE]	4.43	3	2	2	2	0.11	-0.03	0.25	0.10
E9Q401	Ryanodine receptor 2 OS=Mus musculus GN=Ryr2 PE=1 SV=1 - [RYR2_MOUSE]	1.91	2	6	6	7	-0.18	0.19	-0.04	0.33
H3BL23	Ubiquitin-conjugating enzyme E2 E2 (Fragment) OS=Mus musculus GN=Ube2e2 PE=2 SV=1 - [H3BL23_MOUSE]	22.60	8	1	2	7	0.26	0.44	0.36	0.54
Q5SS80-2	Isoform 2 of Dehydrogenase/reductase SDR family member 13 OS=Mus musculus GN=Dhrs13 - [DHR13_MOUSE]	6.32	3	1	1	2	0.57	0.72	0.35	0.50
Q99MS7	EH domain-binding protein 1-like protein 1 OS=Mus musculus GN=Ehbp1l1 PE=2 SV=1 - [EH1L1_MOUSE]	3.15	7	4	4	8	0.07	-0.34	0.21	-0.25
P53702	Cytochrome c-type heme lyase OS=Mus musculus GN=Hccs PE=2 SV=2 - [CCHL_MOUSE]	31.25	2	6	6	21	0.15	0.19	0.32	0.25
Q8R059	UDP-glucose 4-epimerase OS=Mus musculus GN=Gale PE=2 SV=1 - [GALE_MOUSE]	62.82	3	18	18	285	0.35	0.38	0.45	0.48
Q6DID7-2	Isoform 2 of Protein wntless homolog OS=Mus musculus GN=Wls - [WLS_MOUSE]	3.63	2	2	2	5	-0.02	-0.21	-0.02	-0.21
Q99L04	Dehydrogenase/reductase SDR family member 1 OS=Mus musculus GN=Dhrs1 PE=2 SV=1 [DHRS1_MOUSE]	23.00	1	6	6	33	0.11	0.18	0.12	0.19
O70479	BTB/POZ domain-containing adapter for CUL3-mediated RhoA degradation protein 2 OS=Mus musculus GN=Tnfaip1 PE=1 SV=1 - [BACD2_MOUSE]	5.06	1	1	1	3	0.70	0.29	0.57	0.16
Q61080	Forkhead box protein F1 OS=Mus musculus GN=Foxf1 PE=2 SV=2 [FOXF1_MOUSE]	5.29	2	1	1	4	0.16	0.30	0.15	0.29

G5E870	E3 ubiquitin-protein ligase TRIP12 OS=Mus musculus GN=Trip12 PE=1 SV=1 - [TRIPC_MOUSE]	7.56	1	10	10	21	0.10	-0.13	0.21	-0.01
J3KML6	Protein LOC100040196 OS=Mus musculus GN=Gm20830 PE=4 SV=1 - [J3KML6_MOUSE]	6.17	4	1	1	1	-0.51	-0.09	0.69	1.11
Q9WUA2	Phenylalanine--tRNA ligase beta subunit OS=Mus musculus GN=Farsb PE=2 SV=2 - [SYFB_MOUSE]	33.62	1	17	17	65	0.20	0.23	0.14	0.33
Q8BPI1-2	Isoform 2 of Protein kintoun OS=Mus musculus GN=Dnaaf2 - [KTU_MOUSE]	6.70	2	2	2	2	0.20	-0.03	0.05	-0.18
Q9D1I6	39S ribosomal protein L14, mitochondrial OS=Mus musculus GN=Mrpl14 PE=2 SV=1 - [RM14_MOUSE]	11.72	1	2	2	3	-0.14	-1.07	-0.42	-0.64
Q9Z0Y1	Dynactin subunit 3 OS=Mus musculus GN=Dctn3 PE=2 SV=2 [DCTN3_MOUSE]	42.47	2	8	8	22	0.20	0.34	0.29	0.43
F6TBK9	Supervillin (Fragment) OS=Mus musculus GN=Svil PE=4 SV=1 - [F6TBK9_MOUSE]	14.71	1	1	2	8	-0.14	-0.24	-0.14	-0.24
P31428	Dipeptidase 1 OS=Mus musculus GN=Dpep1 PE=1 SV=2 - [DPEP1_MOUSE]	15.12	1	7	7	31	-0.27	-0.45	-0.29	-0.35
Q62000	Mimecan OS=Mus musculus GN=Ogn PE=2 SV=1 - [MIME_MOUSE]	47.99	1	14	14	290	-0.27	-0.22	-0.27	-0.22
Q9JI78	Peptide-N(4)-(N-acetyl-beta-glucosaminyl)asparagine amidase OS=Mus musculus GN=Ngly1 PE=1 SV=2 - [NGLY1_MOUSE]	11.67	1	6	6	12	-0.01	-0.03	0.06	0.08
Q6PGF7	Exocyst complex component 8 OS=Mus musculus GN=Exoc8 PE=1 SV=1 - [EXOC8_MOUSE]	7.82	1	6	6	16	0.18	0.21	0.20	0.23
G3X926	MCG1407, isoform CRA_a OS=Mus musculus GN=Rpf2 PE=4 SV=1 - [G3X926_MOUSE]	18.68	2	4	4	6	0.32	-0.17	0.11	-0.21
Q9QXT0	Protein canopy homolog 2 OS=Mus musculus GN=Cnpy2 PE=2 SV=1 - [CNPY2_MOUSE]	56.59	1	10	10	138	-0.12	-0.10	-0.10	0.02
P62046	Leucine-rich repeat and calponin homology domain-containing protein 1 OS=Mus musculus GN=Lrch1 PE=1 SV=2 - [LRCH1_MOUSE]	2.68	1	2	2	2	0.00	0.19	-0.04	0.15
G3UZ01	Polypyrimidine tract-binding protein 3 OS=Mus musculus GN=Ptbp3 PE=2 SV=1 [G3UZ01_MOUSE]	20.79	7	6	9	27	0.06	-0.20	0.25	0.08

Q3U0M1-4	Isoform 4 of Trafficking protein particle complex subunit 9 OS=Mus musculus GN=Trappc9 - [TPPC9_MOUSE]	4.02	7	2	2	4	-0.09	-0.41	0.16	-0.16
Q9D0F9	Phosphoglucomutase-1 OS=Mus musculus GN=Pgm1 PE=1 SV=4 [PGM1_MOUSE]	51.25	1	3	27	345	-0.21	-0.06	-0.11	0.13
F6QM56	Acid trehalase-like protein 1 (Fragment) OS=Mus musculus GN=Athl1 PE=4 SV=1 [F6QM56_MOUSE]	8.35	6	5	5	12	-0.01	-0.06	0.07	0.02
Q9D3P8	Plasminogen receptor (KT) OS=Mus musculus GN=Plgrkt PE=1 SV=1 - [PLRKT_MOUSE]	25.17	4	4	4	19	-0.16	-0.05	-0.14	-0.14
Q9D7S7-2	Isoform 2 of 60S ribosomal protein L22-like 1 OS=Mus musculus GN=Rpl22l1 - [RL22L_MOUSE]	45.45	2	4	4	24	0.58	0.18	0.51	0.14
Q9CXY6	Interleukin enhancer-binding factor 2 OS=Mus musculus GN=Ilf2 PE=1 SV=1 - [ILF2_MOUSE]	21.54	1	6	6	14	0.48	-0.12	0.36	0.01
Q8BML9	Glutaminyl-tRNA synthetase OS=Mus musculus GN=Qars PE=2 SV=1 - [Q8BML9_MOUSE]	31.10	4	22	22	119	0.26	0.21	0.31	0.31
E9Q4Z2	Protein Acacb OS=Mus musculus GN=Acacb PE=4 SV=1 - [E9Q4Z2_MOUSE]	3.43	1	2	5	13	0.00	0.03	0.15	0.16
Q3V384	Lactation elevated protein 1 OS=Mus musculus GN=Lace1 PE=2 SV=1 - [LACE1_MOUSE]	11.46	3	4	4	8	0.41	0.40	0.26	0.25
Q9QZF2	Glypican-1 OS=Mus musculus GN=Gpc1 PE=1 SV=1 - [GPC1_MOUSE]	23.88	1	9	10	25	-0.03	-0.07	0.13	0.18
P32233	Developmentally-regulated GTP-binding protein 1 OS=Mus musculus GN=Drg1 PE=1 SV=1 - [DRG1_MOUSE]	29.16	2	7	8	30	0.30	0.15	0.25	0.29
Q9DBY8	Nuclear valosin-containing protein-like OS=Mus musculus GN=Nvl PE=1 SV=1 - [NVL_MOUSE]	6.90	1	4	4	6	-0.75	-0.60	2.64	1.92
Q8VE18	Protein SMG8 OS=Mus musculus GN=Smg8 PE=2 SV=1 - [SMG8_MOUSE]	5.55	2	4	4	5	0.07	0.42	0.11	0.76
P31001	Desmin OS=Mus musculus GN=Des PE=1 SV=3 - [DESM_MOUSE]	75.48	1	35	40	2297	-0.20	-0.16	-0.19	-0.13
Q9DC70	NADH dehydrogenase [ubiquinone] iron-sulfur protein 7, mitochondrial OS=Mus musculus GN=Ndufs7 PE=1 SV=1 - [NDUS7_MOUSE]	21.88	1	5	5	27	0.15	0.20	-0.05	0.17

B1AUE5	Peroxisome biogenesis factor 10 OS=Mus musculus GN=Pex10 PE=2 SV=1 - [PEX10_MOUSE]	3.40	1	2	2	34	0.03	0.12	-0.10	-0.03
E9PX61	Protein Gm15800 OS=Mus musculus GN=Gm15800 PE=2 SV=1 - [E9PX61_MOUSE]	2.40	3	5	7	10	0.05	-0.05	0.16	0.06
Q9D415-4	Isoform 4 of Disks large associated protein 1 OS=Mus musculus GN=Dlgap1 - [DLGP1_MOUSE]	6.96	11	2	2	8	0.05	0.08	0.00	0.03
Q91W86	Vacuolar protein sorting associated protein 11 homolog OS=Mus musculus GN=Vps11 PE=1 SV=3 - [VPS11_MOUSE]	2.44	1	2	2	4	0.40	0.08	0.52	0.19
Q8C4Y3	Negative elongation factor B OS=Mus musculus GN=Nelfb PE=2 SV=2 - [NELFB_MOUSE]	6.21	3	3	4	18	0.32	0.02	0.32	0.18
Q61753	D-3-phosphoglycerate dehydrogenase OS=Mus musculus GN=Phgdh PE=1 SV=3 - [SERA_MOUSE]	24.02	2	13	13	58	0.55	0.60	0.42	0.51
F8VQM4	Oxysterol-binding protein OS=Mus musculus GN=Osbpl7 PE=2 SV=1 - [F8VQM4_MOUSE]	3.29	2	1	2	4	0.31	0.54	0.14	0.37
P16406	Glutamyl aminopeptidase OS=Mus musculus GN=Enpep PE=1 SV=1 - [AMPE_MOUSE]	17.04	2	7	15	32	-0.08	0.11	-0.30	0.08
E9Q6Q8	TBC1 domain family member 4 OS=Mus musculus GN=Tbc1d4 PE=2 SV=1 - [E9Q6Q8_MOUSE]	4.10	10	4	4	5	0.24	0.08	0.18	0.09
Q9D2X6	Colon SVA-like protein OS=Mus musculus GN=Sval1 PE=2 SV=1 [Q9D2X6_MOUSE]	17.22	1	3	3	13	0.50	0.50	0.21	0.41
G5E8G6	Myosin Vb, isoform CRA_c OS=Mus musculus GN=Myo5b PE=4 SV=1 - [G5E8G6_MOUSE]	3.80	4	8	8	17	0.29	0.26	0.29	0.22
Q8C8T8	Pre-rRNA-processing protein TSR2 homolog OS=Mus musculus GN=Tsr2 PE=2 SV=1 - [TSR2_MOUSE]	13.61	2	2	2	11	0.13	-0.75	-0.12	-1.33
Q9D7I5-2	Isoform 2 of Phospholysine phosphohistidine inorganic pyrophosphate phosphatase OS=Mus musculus GN=Lhpp - [LHPP_MOUSE]	12.81	2	3	3	6	0.01	-0.02	0.05	-0.11
Q5SV54	Ortholog of human amyotrophic lateral sclerosis 2 (Juvenile) chromosome region, candidate 19 (ALS2CR19) OS=Mus musculus GN=Pard3b PE=4 SV=1 - [Q5SV54_MOUSE]	2.90	3	1	3	4	0.39	0.57	0.35	0.52

Q9D967	Magnesium-dependent phosphatase 1 OS=Mus musculus GN=Mdp1 PE=1 SV=1 - [MGDP1_MOUSE]	50.00	1	7	7	79	0.04	0.05	0.13	0.07
Q9WTR1	Transient receptor potential cation channel subfamily V member 2 OS=Mus musculus GN=Trpv2 PE=1 SV=2 [TRPV2_MOUSE]	2.12	1	2	2	2	-0.46	-0.42	-0.27	-0.23
Q9JIG7	Coiled-coil domain-containing protein 22 OS=Mus musculus GN=Ccdc22 PE=1 SV=1 - [CCD22_MOUSE]	23.13	1	12	12	36	0.09	0.07	0.20	0.12
Q9D394-2	Isoform 2 of Protein RUFY3 OS=Mus musculus GN=Rufy3 - [RUFY3_MOUSE]	11.28	4	7	8	14	0.16	0.14	0.15	0.07
Q9CQY1	Ubiquitin-like protein ATG12 OS=Mus musculus GN=Atg12 PE=1 SV=1 - [ATG12_MOUSE]	6.38	1	1	1	2	0.22	0.46	0.17	0.41
Q9CYI0	Protein Njmu-R1 OS=Mus musculus PE=2 SV=2 - [NJMU_MOUSE]	4.33	3	2	2	3	-0.69	0.39	-0.45	-0.06
Q99KK2	N-acylneuraminate cytidylyltransferase OS=Mus musculus GN=Cmas PE=1 SV=2 - [NEUA_MOUSE]	43.52	2	18	18	110	0.09	0.04	0.10	0.03
Q7TQD2	Tubulin polymerization-promoting protein OS=Mus musculus GN=Tppp PE=1 SV=1 - [TPPP_MOUSE]	45.41	2	7	8	47	-0.09	0.05	-0.09	0.12
Q922B2	Aspartate--tRNA ligase, cytoplasmic OS=Mus musculus GN=Dars PE=2 SV=2 - [SYDC_MOUSE]	42.91	2	20	20	113	0.20	0.20	0.25	0.23
Q3TXY2	Protein Tex264 OS=Mus musculus GN=Tex264 PE=2 SV=1 - [Q3TXY2_MOUSE]	13.65	2	3	3	17	0.21	0.04	0.20	0.05
E9Q7T4	Sorting nexin-13 OS=Mus musculus GN=Snx13 PE=2 SV=1 - [E9Q7T4_MOUSE]	7.98	3	2	2	3	0.11	-0.68	0.40	-0.30
B2RX12-3	Isoform 3 of Canalicular multispecific organic anion transporter 2 OS=Mus musculus GN=Abcc3 - [MRP3_MOUSE]	11.95	9	12	14	37	0.04	0.07	-0.12	-0.06
E9Q8U3	Protein Gcom1 OS=Mus musculus GN=Gcom1 PE=2 SV=1 - [E9Q8U3_MOUSE]	14.48	7	5	5	16	-0.28	-0.29	-0.13	-0.10
Q80YD1	ATP-dependent RNA helicase SUPV3L1, mitochondrial OS=Mus musculus GN=Supv3l1 PE=2 SV=1 - [SUV3_MOUSE]	8.99	1	5	5	14	0.09	-0.05	0.03	0.00
A2ALS5	Protein Rap1gap OS=Mus musculus GN=Rap1gap PE=2 SV=1 - [A2ALS5_MOUSE]	6.20	4	3	3	3	-0.19	-0.39	-0.25	-0.46

O35392	Forkhead box protein D2 OS=Mus musculus GN=Foxd2 PE=2 SV=1 - [FOXD2_MOUSE]	7.32	1	2	2	5	0.40	0.02	0.50	0.12
P46471	26S protease regulatory subunit 7 OS=Mus musculus GN=Psmc2 PE=1 SV=5 - [PRS7_MOUSE]	57.74	2	21	21	168	0.39	0.32	0.37	0.39
Q4PJX1-3	Isoform 3 of Protein odr 4 homolog OS=Mus musculus GN=Odr4 - [ODR4_MOUSE]	16.87	3	5	5	10	0.21	-0.04	0.25	0.01
E9Q9V3	Nuclear receptor corepressor 2 OS=Mus musculus GN=Ncor2 PE=2 SV=1 - [E9Q9V3_MOUSE]	1.37	11	1	3	3	-0.26	-1.74	-0.46	-1.93
Q9CR57	60S ribosomal protein L14 OS=Mus musculus GN=Rpl14 PE=2 SV=3 [RL14_MOUSE]	31.34	1	7	7	134	0.28	0.34	0.34	0.32
P35951	Low-density lipoprotein receptor OS=Mus musculus GN=Ldlr PE=1 SV=2 - [LDLR_MOUSE]	2.90	1	2	2	6	0.25	0.49	0.09	0.33
Q8VCF0	Mitochondrial antiviral-signaling protein OS=Mus musculus GN=Mavs PE=1 SV=1 - [MAVS_MOUSE]	21.07	1	6	6	55	-0.07	-0.03	0.14	0.00
P35979	60S ribosomal protein L12 OS=Mus musculus GN=Rpl12 PE=1 SV=2 [RL12_MOUSE]	63.64	3	9	9	245	0.33	0.23	0.38	0.28
A2AP32	NADH dehydrogenase [ubiquinone] 1 beta subcomplex subunit 6 OS=Mus musculus GN=Ndufb6 PE=2 SV=1 - [A2AP32_MOUSE]	25.77	2	2	2	57	-0.07	-0.02	-0.02	-0.05
P51175	Protoporphyrinogen oxidase OS=Mus musculus GN=Ppox PE=2 SV=1 - [PPOX_MOUSE]	5.03	1	2	2	5	-0.14	-0.71	-0.15	-0.72
Q80TJ1-4	Isoform 4 of Calcium-dependent secretion activator 1 OS=Mus musculus GN=Cadps - [CAPS1_MOUSE]	1.04	6	2	2	2	0.27	0.21	0.24	0.18
F6VTH5	Leucine zipper transcription factor-like protein 1 (Fragment) OS=Mus musculus GN=Lztfl1 PE=4 SV=1 [F6VTH5_MOUSE]	20.43	4	4	4	32	0.23	-0.06	0.06	0.20
B7ZCC2	Protein Aim1l OS=Mus musculus GN=Aim1l PE=4 SV=1 - [B7ZCC2_MOUSE]	0.73	1	1	1	2	-0.08	-0.05	0.02	0.04
Q8CBM2	Aspartyl/asparaginyl beta-hydroxylase OS=Mus musculus GN=Asph PE=2 SV=1 - [Q8CBM2_MOUSE]	28.57	15	16	16	66	0.08	0.23	-0.02	0.03

Q9CR62	Mitochondrial 2-oxoglutarate/malate carrier protein OS=Mus musculus GN=Slc25a11 PE=1 SV=3 - [M2OM_MOUSE]	20.38	3	6	6	28	0.01	-0.22	0.07	-0.20
Q9ERA6	Tuftelin-interacting protein 11 OS=Mus musculus GN=Tfip11 PE=1 SV=1 - [TFP11_MOUSE]	3.70	1	2	2	7	-0.13	-0.24	0.58	0.46
Q3TEA8-2	Isoform 2 of Heterochromatin protein 1-binding protein 3 OS=Mus musculus GN=Hp1bp3 - [HP1B3_MOUSE]	25.88	8	14	14	77	0.18	0.18	0.14	0.03
Q61881	DNA replication licensing factor MCM7 OS=Mus musculus GN=Mcm7 PE=2 SV=1 - [MCM7_MOUSE]	13.63	5	7	7	21	0.54	0.42	0.53	0.50
Q9EQY0	Serine/threonine-protein kinase/endoribonuclease IRE1 OS=Mus musculus GN=Ern1 PE=1 SV=1 - [ERN1_MOUSE]	4.20	1	2	2	6	-0.67	-0.05	-0.16	0.46
E9QAF2	Protein Abca16 OS=Mus musculus GN=Abca16 PE=2 SV=1 - [E9QAF2_MOUSE]	2.27	2	2	2	2	-0.20	-0.06	-0.37	-0.24
Q9D6Z1	Nucleolar protein 56 OS=Mus musculus GN=Nop56 PE=1 SV=2 - [NOP56_MOUSE]	29.48	8	15	15	105	0.27	0.30	0.28	0.26
P62830	60S ribosomal protein L23 OS=Mus musculus GN=Rpl23 PE=1 SV=1 [RL23_MOUSE]	45.71	1	6	6	73	0.45	0.20	0.34	0.19
G3XA21	MCG134445, isoform CRA_a OS=Mus musculus GN=Mroh1 PE=4 SV=1 - [G3XA21_MOUSE]	1.16	4	2	2	3	0.29	0.33	0.37	0.41
E9QMF0	Grainyhead-like protein 1 homolog OS=Mus musculus GN=Grhl1 PE=2 SV=1 - [E9QMF0_MOUSE]	5.04	3	2	2	2	-0.24	-0.37	0.21	0.08
P12787	Cytochrome c oxidase subunit 5A, mitochondrial OS=Mus musculus GN=Cox5a PE=1 SV=2 - [COX5A_MOUSE]	58.22	1	8	9	447	-0.10	0.02	-0.13	0.03
Q61081	Hsp90 co-chaperone Cdc37 OS=Mus musculus GN=Cdc37 PE=2 SV=1 - [CDC37_MOUSE]	37.47	1	13	13	101	0.40	0.50	0.45	0.37
Q3UFY7-4	Isoform 4 of 7-methylguanosine phosphate-specific 5'-nucleotidase OS=Mus musculus GN=Nt5c3b - [5NT3B_MOUSE]	17.81	4	5	5	13	0.43	0.33	0.26	-0.04
Q9ERZ4	Muscarinic acetylcholine receptor M2 OS=Mus musculus GN=Chrm2 PE=1 SV=2 - [ACM2_MOUSE]	3.86	1	1	1	2	0.18	0.01	0.27	0.11

Q5U5V2	Hydroxylysine kinase OS=Mus musculus GN=Agphd1 PE=2 SV=2 - [AGPD1_MOUSE]	8.78	2	3	3	7	0.48	0.43	0.47	0.43
D3YY07	Palmitoyltransferase ZDHHC13 (Fragment) OS=Mus musculus GN=Zdhhc13 PE=2 SV=1 - [D3YY07_MOUSE]	16.06	2	5	5	15	0.40	0.26	0.23	0.15
Q792Y8	MCG15081 OS=Mus musculus GN=Gm10334 PE=3 SV=1 - [Q792Y8_MOUSE]	12.20	2	1	2	134	-0.59	0.24	-0.59	0.06
O08599	Syntaxin-binding protein 1 OS=Mus musculus GN=Stxbp1 PE=1 SV=2 - [STXB1_MOUSE]	24.41	2	11	11	37	-0.09	0.01	0.11	0.03
O88544	COP9 signalosome complex subunit 4 OS=Mus musculus GN=Cops4 PE=1 SV=1 - [CSN4_MOUSE]	30.79	4	11	11	49	0.29	0.34	0.27	0.28
Q6ZWY3	40S ribosomal protein S27-like OS=Mus musculus GN=Rps27l PE=2 SV=3 - [RS27L_MOUSE]	39.29	3	1	4	69	0.61	0.90	0.47	0.68
Q9DCJ5	NADH dehydrogenase [ubiquinone] 1 alpha subcomplex subunit 8 OS=Mus musculus GN=Ndufa8 PE=1 SV=3 - [NDUA8_MOUSE]	37.79	1	7	7	38	0.30	0.34	0.24	0.13
Q922D4-2	Isoform 2 of Serine/threonine-protein phosphatase 6 regulatory subunit 3 OS=Mus musculus GN=Ppp6r3 - [PP6R3_MOUSE]	17.53	5	11	12	42	0.31	0.27	0.29	0.17
Q921G8	Gamma-tubulin complex component 2 OS=Mus musculus GN=Tubgcp2 PE=2 SV=2 - [GCP2_MOUSE]	4.09	1	2	2	3	0.21	-0.46	0.09	-0.88
P04104	Keratin, type II cytoskeletal 1 OS=Mus musculus GN=Krt1 PE=1 SV=4 - [K2C1_MOUSE]	8.48	1	2	6	78	2.05	0.26	0.30	-1.84
Q9QXK7	Cleavage and polyadenylation specificity factor subunit 3 OS=Mus musculus GN=Cpsf3 PE=1 SV=2 - [CPSF3_MOUSE]	3.36	1	2	3	5	0.15	0.08	0.33	0.27
Q8BIJ7	RUN and FYVE domain-containing protein 1 OS=Mus musculus GN=Rufy1 PE=1 SV=1 - [RUFY1_MOUSE]	11.24	1	5	8	13	-0.02	0.17	0.09	0.58
Q8BH64	EH domain-containing protein 2 OS=Mus musculus GN=Ehd2 PE=1 SV=1 - [EHD2_MOUSE]	57.46	2	27	28	411	-0.01	0.03	-0.01	0.05
Q80ZP8	Armet protein OS=Mus musculus GN=Manf PE=2 SV=1 - [Q80ZP8_MOUSE]	55.76	6	4	11	178	0.35	0.50	0.25	0.40

F6V6T4	Transmembrane emp24 domain-containing protein 2 (Fragment) OS=Mus musculus GN=Tmed2 PE=3 SV=1 - [F6V6T4_MOUSE]	34.09	3	5	5	25	-0.02	0.03	0.00	0.08
P70124	Serpin B5 OS=Mus musculus GN=Serpinb5 PE=2 SV=1 - [SPB5_MOUSE]	51.47	1	15	15	164	0.41	0.31	0.41	0.29
Q9CZW5	Mitochondrial import receptor subunit TOM70 OS=Mus musculus GN=Tomm70a PE=1 SV=2 - [TOM70_MOUSE]	37.48	1	21	22	83	0.05	0.15	0.11	0.09
E9QNL5	Sulfotransferase 1A1 OS=Mus musculus GN=Sult1a1 PE=2 SV=1 - [E9QNL5_MOUSE]	58.08	6	14	14	206	-0.14	-0.11	-0.13	-0.15
Q9R1R8	Retinol dehydrogenase 11 OS=Mus musculus GN=Rdh11 PE=2 SV=1 - [Q9R1R8_MOUSE]	15.67	2	3	3	11	0.62	0.16	0.36	0.27
Q80X60	EF-hand calcium-binding domain-containing protein 3 OS=Mus musculus GN=Efcab3 PE=2 SV=1 - [EFCB3_MOUSE]	3.70	1	1	1	1	-1.40	-1.32	-1.00	-0.92
A2AS86	Glucagon (Fragment) OS=Mus musculus GN=Gcg PE=2 SV=1 - [A2AS86_MOUSE]	38.15	2	4	4	50	-0.23	-0.36	-0.14	-0.31
Q8BRF7	Sec1 family domain-containing protein 1 OS=Mus musculus GN=Scfd1 PE=2 SV=1 [SCFD1_MOUSE]	29.73	3	15	15	67	0.37	0.28	0.31	0.31
P97311	DNA replication licensing factor MCM6 OS=Mus musculus GN=Mcm6 PE=1 SV=1 - [MCM6_MOUSE]	20.10	1	16	17	51	0.40	0.46	0.35	0.49
Q9JJR8	Transmembrane protein 9B OS=Mus musculus GN=Tmem9b PE=1 SV=1 - [TMM9B_MOUSE]	23.12	2	2	2	3	0.05	0.15	0.02	0.21
Q9CQZ6	NADH dehydrogenase [ubiquinone] 1 beta subcomplex subunit 3 OS=Mus musculus GN=Ndufb3 PE=1 SV=1 - [NDUB3_MOUSE]	17.31	1	2	2	23	-0.01	0.06	0.00	0.06
D3YUA8	F-box/WD repeat-containing protein 7 OS=Mus musculus GN=Fbxw7 PE=2 SV=1 - [D3YUA8_MOUSE]	2.38	3	1	1	1	0.23	-0.17	1.62	1.22
D3Z669	Villin-like protein OS=Mus musculus GN=Vill PE=2 SV=1 - [D3Z669_MOUSE]	6.38	6	2	2	2	-0.04	0.76	-0.20	0.60
Q8VDP3-2	Isoform 2 of Protein-methionine sulfoxide oxidase MICAL1 OS=Mus musculus GN=Mical1 - [MICA1_MOUSE]	20.51	4	17	17	48	0.20	0.21	0.26	0.22

Q3UE37	Ubiquitin-conjugating enzyme E2 Z OS=Mus musculus GN=Ube2z PE=1 SV=2 - [UBE2Z_MOUSE]	18.82	1	6	6	29	0.42	-0.02	0.38	-0.01
Q8C4U3	Secreted frizzled-related protein 1 OS=Mus musculus GN=Sfrp1 PE=1 SV=3 - [SFRP1_MOUSE]	5.10	1	1	1	7	0.80	0.58	0.93	0.63
Q9Z266	SNARE-associated protein Snapin OS=Mus musculus GN=Snapin PE=1 SV=1 - [SNAPN_MOUSE]	8.82	1	1	1	2	0.04	0.63	0.18	0.76
Q8R1B4	Eukaryotic translation initiation factor 3 subunit C OS=Mus musculus GN=Eif3c PE=1 SV=1 - [EIF3C_MOUSE]	34.47	1	25	25	130	0.15	0.20	0.25	0.22
Q8R3N6	THO complex subunit 1 OS=Mus musculus GN=Thoc1 PE=1 SV=1 - [THOC1_MOUSE]	15.07	1	8	8	25	0.44	0.51	0.18	0.38
Q99K28	ADP-ribosylation factor GTPase-activating protein 2 OS=Mus musculus GN=Arfgap2 PE=1 SV=1 - [ARFG2_MOUSE]	20.77	2	8	8	38	0.16	0.08	0.23	0.20
Q9Z1R9	MCG124046 OS=Mus musculus GN=Prss1 PE=2 SV=1 - [Q9Z1R9_MOUSE]	15.45	2	1	3	1197	-0.09	-0.06	-0.05	0.00
Q8CGB6-2	Isoform 2 of Tensin-like C1 domain-containing phosphatase OS=Mus musculus GN=Tenc1 - [TENC1_MOUSE]	10.02	4	9	11	37	-0.04	-0.01	-0.07	0.03
Q7TQ65-2	Isoform 2 of Transmembrane channel like protein 4 OS=Mus musculus GN=Tmc4 - [TMC4_MOUSE]	3.33	5	2	2	5	-0.12	-0.02	0.28	0.28
P52482	Ubiquitin-conjugating enzyme E2 E1 OS=Mus musculus GN=Ube2e1 PE=2 SV=1 - [UB2E1_MOUSE]	12.95	3	1	2	6	0.13	0.40	0.25	0.53
O88713	Killer cell lectin-like receptor subfamily G member 1 OS=Mus musculus GN=Klrg1 PE=1 SV=2 - [KLRG1_MOUSE]	4.79	1	1	1	4	-0.41	-0.59	-0.36	-0.54
Q8VHE0	Translocation protein SEC63 homolog OS=Mus musculus GN=Sec63 PE=1 SV=4 [SEC63_MOUSE]	19.61	1	11	11	52	0.25	0.19	0.17	0.21
G3UW40	MCG4620, isoform CRA_b OS=Mus musculus GN=Mcc PE=4 SV=1 - [G3UW40_MOUSE]	3.26	2	3	3	3	-0.19	-0.22	0.17	0.14
B4YDY0	Small conductance calcium-activated potassium channel protein 2 OS=Mus musculus GN=Kcnn2 PE=2 SV=1 - [B4YDY0_MOUSE]	5.30	6	2	2	2	-0.50	-0.69	-0.47	-0.68

Q8K4M5	COMM domain-containing protein 1 OS=Mus musculus GN=Commd1 PE=2 SV=2 - [COMD1_MOUSE]	12.77	4	3	3	7	-0.18	0.69	-0.19	0.30
Q91VC9	Growth hormone-inducible transmembrane protein OS=Mus musculus GN=Ghitm PE=2 SV=1 - [GHITM_MOUSE]	2.89	1	1	1	5	0.02	0.05	0.22	0.25
E9Q912	Protein Rap1gds1 OS=Mus musculus GN=Rap1gds1 PE=2 SV=1 - [E9Q912_MOUSE]	24.38	3	13	13	65	0.37	0.32	0.36	0.28
O35945	Aldehyde dehydrogenase, cytosolic 1 OS=Mus musculus GN=Aldh1a7 PE=2 SV=1 - [AL1A7_MOUSE]	37.13	1	5	16	221	-0.37	-0.30	-0.18	-0.12
P49717	DNA replication licensing factor MCM4 OS=Mus musculus GN=Mcm4 PE=2 SV=1 - [MCM4_MOUSE]	17.17	1	11	12	30	0.50	0.47	0.34	0.41
Q5SVL6-2	Isoform 2 of Rap1 GTPase-activating protein 2 OS=Mus musculus GN=Rap1gap2 - [RPGP2_MOUSE]	16.02	2	5	5	10	-0.12	-0.14	0.00	-0.12
Q9JKR6	Hypoxia up-regulated protein 1 OS=Mus musculus GN=Hyou1 PE=1 SV=1 - [HYOU1_MOUSE]	48.15	3	36	36	424	0.51	0.56	0.52	0.52
Q3TZX8-3	Isoform 3 of Polynucleotide 5'-hydroxyl-kinase NOL9 OS=Mus musculus GN=Nol9 - [NOL9_MOUSE]	1.75	3	1	1	1	0.41	0.21	-0.04	-0.24
Q05BC3-2	Isoform 2 of Echinoderm microtubule-associated protein-like 1 OS=Mus musculus GN=Eml1 - [EMAL1_MOUSE]	27.97	5	19	20	118	0.14	0.12	0.17	0.22
A3KGQ6	Actin-related protein 2/3 complex subunit 5-like protein OS=Mus musculus GN=Arpc5l PE=2 SV=1 - [A3KGQ6_MOUSE]	64.05	2	10	10	46	0.18	0.22	0.36	0.30
G3X8Y3	N-alpha-acetyltransferase 15, NatA auxiliary subunit OS=Mus musculus GN=Naa15 PE=4 SV=1 - [G3X8Y3_MOUSE]	23.82	2	18	20	73	0.38	0.30	0.36	0.26
Q7M759	Alpha/beta hydrolase domain-containing protein 17B OS=Mus musculus GN=Abhd17b PE=2 SV=1 - [AB17B_MOUSE]	7.29	1	2	2	2	0.03	-0.02	0.15	0.10
Q9DBP5	UMP-CMP kinase OS=Mus musculus GN=Cmpk1 PE=1 SV=1 - [KCY_MOUSE]	58.67	1	13	14	442	0.00	0.16	-0.03	0.17

Q9CQS2	H/ACA ribonucleoprotein complex subunit 3 OS=Mus musculus GN=Nop10 PE=3 SV=1 - [NOP10_MOUSE]	45.31	1	2	2	4	0.40	0.50	0.32	0.42
Q68FG2	Protein Sptbn2 OS=Mus musculus GN=Sptbn2 PE=2 SV=1 - [Q68FG2_MOUSE]	4.40	1	1	12	85	-2.45	-2.28	-2.20	-2.04
Q9QXL2-3	Isoform 3 of Kinesin-like protein KIF21A OS=Mus musculus GN=Kif21a - [KI21A_MOUSE]	7.15	9	6	7	15	0.12	-0.10	0.13	-0.11
Q9D7X3	Dual specificity protein phosphatase 3 OS=Mus musculus GN=Dusp3 PE=1 SV=1 - [DUS3_MOUSE]	40.54	4	3	5	42	0.62	0.60	0.53	0.57
Q9CPT0	Apoptosis facilitator Bcl-2-like protein 14 OS=Mus musculus GN=Bcl2l14 PE=2 SV=1 - [B2L14_MOUSE]	48.78	2	14	14	40	0.03	0.18	0.15	0.13
Q80VQ1	Leucine-rich repeat-containing protein 1 OS=Mus musculus GN=Lrrc1 PE=2 SV=2 - [LRRC1_MOUSE]	17.18	4	5	7	10	0.06	0.15	0.15	0.14
D3YVU1	Ubiquitin carboxyl-terminal hydrolase 16 OS=Mus musculus GN=Usp16 PE=2 SV=1 - [D3YVU1_MOUSE]	5.70	4	2	2	12	-0.51	-0.69	-0.25	-0.60
Q9Z0G7	Zinc finger and BTB domain-containing protein 22 OS=Mus musculus GN=Zbtb22 PE=2 SV=1 - [ZBT22_MOUSE]	4.23	1	1	1	1	-1.09	-2.28	-0.98	-2.17
Q6ZPI3	Ligand-dependent corepressor OS=Mus musculus GN=Lcor PE=2 SV=2 - [LCOR_MOUSE]	1.39	1	1	1	2	-0.19	0.29	0.13	0.60
Q9D5T0	ATPase family AAA domain-containing protein 1 OS=Mus musculus GN=Atad1 PE=1 SV=1 - [ATAD1_MOUSE]	17.17	1	5	5	17	0.02	-0.18	-0.07	-0.05
O55028	[3-methyl-2-oxobutanoate dehydrogenase [lipoamide]] kinase, mitochondrial OS=Mus musculus GN=Bckdk PE=1 SV=1 - [BCKD_MOUSE]	13.83	2	3	4	13	0.09	0.09	-0.01	0.02
Q9JJ28	Protein flightless-1 homolog OS=Mus musculus GN=Flii PE=1 SV=1 - [FLII_MOUSE]	17.86	1	19	19	61	0.14	0.02	0.16	0.14
E9QAI5	DNA fragmentation factor subunit beta OS=Mus musculus GN=Cad PE=2 SV=1 - [E9QAI5_MOUSE]	14.66	10	19	22	78	0.38	0.33	0.47	0.50
Q9ERU3	Zinc finger protein 22 OS=Mus musculus GN=Znf22 PE=1 SV=2 [ZNF22_MOUSE]	12.24	120	2	3	5	-0.41	-0.32	-0.40	-0.31

Q9CQ56	Vesicle transport protein USE1 OS=Mus musculus GN=Use1 PE=2 SV=1 - [USE1_MOUSE]	8.52	3	2	2	5	0.41	0.40	0.23	0.21
D3Z5U5	P2X purinoceptor (Fragment) OS=Mus musculus GN=P2rx4 PE=2 SV=1 - [D3Z5U5_MOUSE]	10.06	8	4	4	9	0.04	0.40	0.13	0.40
P62242	40S ribosomal protein S8 OS=Mus musculus GN=Rps8 PE=1 SV=2 - [RS8_MOUSE]	37.98	1	8	8	134	0.20	0.24	0.20	0.27
Q9CY27	Very-long-chain enoyl-CoA reductase OS=Mus musculus GN=Tecr PE=1 SV=1 - [TECR_MOUSE]	8.44	3	2	3	28	0.50	0.49	0.44	0.44
Q8R0W0	Epiplakin OS=Mus musculus GN=Eppk1 PE=1 SV=2 - [EPIPL_MOUSE]	34.64	1	29	34	286	0.18	0.10	0.09	0.08
P60867	40S ribosomal protein S20 OS=Mus musculus GN=Rps20 PE=1 SV=1 - [RS20_MOUSE]	41.18	2	5	6	107	0.43	0.38	0.53	0.43
D3YXZ3	Kinesin light chain 2 OS=Mus musculus GN=Klc2 PE=4 SV=1 - [D3YXZ3_MOUSE]	11.51	4	3	6	17	0.00	0.18	-0.03	0.05
Q3B7Z2	Oxysterol-binding protein 1 OS=Mus musculus GN=Osbp PE=1 SV=3 - [OSBP1_MOUSE]	25.96	2	12	13	42	0.30	0.38	0.33	0.45
Q9D8Y1	Transmembrane protein 126A OS=Mus musculus GN=Tmem126a PE=2 SV=1 - [T126A_MOUSE]	11.22	2	2	2	6	-0.04	-0.25	-0.02	-0.23
Q9Z0N1	Eukaryotic translation initiation factor 2 subunit 3, X-linked OS=Mus musculus GN=Eif2s3x PE=1 SV=2 - [IF2G_MOUSE]	33.26	2	3	12	61	0.27	0.16	0.42	0.31
P97350	Plakophilin-1 OS=Mus musculus GN=Pkp1 PE=1 SV=1 - [PKP1_MOUSE]	3.30	1	2	2	8	0.86	0.89	0.65	0.60
Q99KI3	ER membrane protein complex subunit 3 OS=Mus musculus GN=Emc3 PE=2 SV=3 [EMC3_MOUSE]	22.99	1	6	6	15	0.08	0.11	0.05	0.10
Q8BMS1	Trifunctional enzyme subunit alpha, mitochondrial OS=Mus musculus GN=Hadha PE=1 SV=1 - [ECHA_MOUSE]	54.13	2	29	31	549	0.07	0.08	0.00	0.02
E9QAS4	Chromodomain-helicase DNA-binding protein 4 OS=Mus musculus GN=Chd4 PE=2 SV=1 - [E9QAS4_MOUSE]	13.62	7	21	21	57	0.27	0.40	0.32	0.37
Q9D023	Mitochondrial pyruvate carrier 2 OS=Mus musculus GN=Mpc2 PE=1 SV=1 - [MPC2_MOUSE]	13.39	1	3	3	16	-0.14	0.40	-0.09	0.28

Q9DCU6	39S ribosomal protein L4, mitochondrial OS=Mus musculus GN=Mrpl4 PE=2 SV=1 [RM04_MOUSE]	13.61	1	4	4	15	-1.17	0.54	-1.22	0.48
Q8R1X6	Spartin OS=Mus musculus GN=Spg20 PE=2 SV=1 - [SPG20_MOUSE]	11.77	3	4	4	9	0.32	0.39	0.19	0.26
Q99MB1	Toll-like receptor 3 OS=Mus musculus GN=Tlr3 PE=1 SV=2 - [TLR3_MOUSE]	3.76	1	3	3	4	0.10	0.09	0.11	0.21
Q9DCT2	NADH dehydrogenase [ubiquinone] iron-sulfur protein 3, mitochondrial OS=Mus musculus GN=Ndufs3 PE=1 SV=2 - [NDUS3_MOUSE]	35.36	1	9	9	126	-0.07	0.08	-0.03	0.05
Q8BVL3	Sorting nexin-17 OS=Mus musculus GN=Snx17 PE=1 SV=2 - [SNX17_MOUSE]	18.09	4	6	6	15	0.32	0.66	0.22	0.58
Q5U4C3	Splicing factor, arginine/serine-rich 19 OS=Mus musculus GN=Scaf1 PE=1 SV=1 [SFR19_MOUSE]	3.03	1	3	3	7	0.03	0.40	0.00	0.31
Q8BK64	Activator of 90 kDa heat shock protein ATPase homolog 1 OS=Mus musculus GN=Ahsa1 PE=2 SV=2 - [AHSA1_MOUSE]	43.49	1	11	12	91	0.25	0.25	0.23	0.24
P52431	DNA polymerase delta catalytic subunit OS=Mus musculus GN=Pold1 PE=1 SV=2 [DPOD1_MOUSE]	4.34	2	5	5	6	0.26	0.03	0.18	0.06
P26231	Catenin alpha-1 OS=Mus musculus GN=Ctnna1 PE=1 SV=1 - [CTNA1_MOUSE]	42.72	3	29	38	383	0.34	0.36	0.37	0.39
E9Q1U2	Synaptopodin-2 OS=Mus musculus GN=Synpo2 PE=2 SV=1 - [E9Q1U2_MOUSE]	52.19	3	9	48	498	-0.07	0.08	-0.02	0.05
Q8BP47	Asparagine--tRNA ligase, cytoplasmic OS=Mus musculus GN=Nars PE=1 SV=2 - [SYNC_MOUSE]	33.45	1	17	17	233	0.50	0.60	0.48	0.48
P11798	Calcium/calmodulin-dependent protein kinase type II subunit alpha OS=Mus musculus GN=Camk2a PE=1 SV=2 - [KCC2A_MOUSE]	11.72	6	1	5	45	-0.63	-0.45	-0.19	-0.01
Q63810-2	Isoform 2 of Calcineurin subunit B type 1 OS=Mus musculus GN=Ppp3r1 - [CANB1_MOUSE]	66.88	3	8	8	52	0.13	0.32	0.16	0.33
Q99JR8-2	Isoform 2 of SWI/SNF-related matrix-associated actin-dependent regulator of chromatin subfamily D member 2 OS=Mus musculus GN=Smarcd2 - [SMRD2_MOUSE]	6.61	3	3	3	6	0.31	0.44	0.44	0.57

Q8CCJ3	E3 UFM1-protein ligase 1 OS=Mus musculus GN=Ufl1 PE=1 SV=2 - [UFL1_MOUSE]	20.68	3	15	16	42	0.30	0.22	0.27	0.06
Q8VEH5	EPM2A-interacting protein 1 OS=Mus musculus GN=Epm2aip1 PE=2 SV=1 - [EPMIP_MOUSE]	6.93	1	3	3	9	-0.24	-1.11	-0.01	-0.26
Q8K284-2	Isoform 2 of General transcription factor 3C polypeptide 1 OS=Mus musculus GN=Gtf3c1 - [TF3C1_MOUSE]	2.99	3	4	4	6	0.09	0.72	-0.40	-2.41
Q812A5-2	Isoform 2 of Proline-rich protein 5 OS=Mus musculus GN=Prr5 - [PRR5_MOUSE]	6.88	2	1	2	2	0.61	1.28	0.62	1.28
Q61024	Asparagine synthetase [glutamine-hydrolyzing] OS=Mus musculus GN=Asns PE=2 SV=3 - [ASNS_MOUSE]	28.34	2	13	14	76	0.90	0.88	0.71	0.88
Q8BYN3	Inositol-tetrakisphosphate 1-kinase OS=Mus musculus GN=Itpk1 PE=2 SV=1 - [ITPK1_MOUSE]	16.71	2	5	5	33	-0.17	-0.29	0.00	-0.12
Q8R5F7-2	Isoform 2 of Interferon-induced helicase C domain-containing protein 1 OS=Mus musculus GN=Ifih1 - [IFIH1_MOUSE]	10.76	2	10	10	45	0.34	0.22	0.33	0.32
E9PXC2	Upstream stimulatory factor 2 OS=Mus musculus GN=Usf2 PE=2 SV=1 - [E9PXC2_MOUSE]	16.74	7	3	3	4	0.36	0.09	0.49	0.22
Q9CQC7	NADH dehydrogenase [ubiquinone] 1 beta subcomplex subunit 4 OS=Mus musculus GN=Ndufb4 PE=1 SV=3 - [NDUB4_MOUSE]	50.39	1	4	5	44	-0.15	-0.09	0.00	-0.05
O08582	GTP-binding protein 1 OS=Mus musculus GN=Gtpbp1 PE=1 SV=2 - [GTPB1_MOUSE]	29.49	1	12	12	45	0.31	0.23	0.26	0.23
F7AQZ8	Uncharacterized protein OS=Mus musculus GN=Gm21857 PE=4 SV=1 - [F7AQZ8_MOUSE]	3.25	7	1	1	6	0.15	-0.20	-0.09	-0.23
Q7TQ95	Protein lunapark OS=Mus musculus GN=Lnp PE=1 SV=1 - [LNP_MOUSE]	19.29	4	5	5	9	0.17	0.00	0.35	0.10
Q9Z0U9	Sphingosine 1-phosphate receptor 3 OS=Mus musculus GN=S1pr3 PE=2 SV=1 [S1PR3_MOUSE]	10.05	1	2	2	2	0.15	-0.13	0.12	-0.16
D3YW19	Protein 9030624J02Rik (Fragment) OS=Mus musculus GN=9030624J02Rik PE=2 SV=2 - [D3YW19_MOUSE]	5.32	8	5	5	6	0.20	-0.24	0.49	0.12

E9PYG6	Protein Rasa1 OS=Mus musculus GN=Rasa1 PE=2 SV=1 - [E9PYG6_MOUSE]	2.41	1	2	2	7	0.12	-0.11	0.23	0.00
Q9D186	Apolipoprotein O OS=Mus musculus GN=Apoo PE=2 SV=1 - [Q9D186_MOUSE]	21.95	3	3	3	20	0.03	-0.01	-0.01	-0.06
F6TE23	Podocalyxin-like protein 2 (Fragment) OS=Mus musculus GN=Podxl2 PE=2 SV=1 - [F6TE23_MOUSE]	3.55	2	1	1	5	-0.17	0.22	-0.22	0.17
Q91X21	Uncharacterized protein KIAA2013 OS=Mus musculus GN=Kiaa2013 PE=2 SV=1 - [K2013_MOUSE]	11.67	3	3	3	10	-0.08	0.15	0.12	0.23
Q6GYP7-6	Isoform 1S of Ral GTPase-activating protein subunit alpha-1 OS=Mus musculus GN=Ralgapa1 - [RGPA1_MOUSE]	4.93	6	5	5	8	0.01	0.06	0.25	-0.09
Q9DA64	CMT1A duplicated region transcript 4 protei homolog OS=Mus musculus GN=Cdrt4 PE=2 SV=1 - [CDRT4_MOUSE]	5.03	1	1	1	1	0.28	0.09	0.28	0.09
G5E8J9	SCY1-like protein 2 OS=Mus musculus GN=Scyl2 PE=2 SV=1 [G5E8J9_MOUSE]	6.78	4	5	5	7	-0.15	-0.07	0.15	0.16
Q8BXA1	Golgi integral membrane protein 4 OS=Mus musculus GN=Golim4 PE=1 SV=1 - [GOLI4_MOUSE]	5.80	3	3	3	12	-0.12	-0.26	-0.12	-0.15
Q9QXH4	Integrin alpha-X OS=Mus musculus GN=Itgax PE=2 SV=1 - [ITAX_MOUSE]	3.51	1	4	4	10	0.13	0.03	0.28	0.60
Q99KR8	Plasma alpha-L-fucosidase OS=Mus musculus GN=Fuca2 PE=2 SV=1 - [FUCO2_MOUSE]	18.44	2	8	8	24	-0.33	-0.34	-0.20	-0.25
Q7TMW6	Cytosolic Fe-S cluster assembly factor NARFL OS=Mus musculus GN=Narfl PE=2 SV=2 - [NARFL_MOUSE]	15.76	3	5	5	20	0.07	-0.01	0.29	0.14
Q792Z1	MCG140784 OS=Mus musculus GN=Try10 PE=2 SV=1 - [Q792Z1_MOUSE]	10.57	1	1	3	56	-0.14	-0.03	-0.25	-0.23
Q9JHC9-3	Isoform 2 of ETS-related transcription factor Elf-2 OS=Mus musculus GN=Elf2 - [ELF2_MOUSE]	8.06	6	2	3	4	-0.30	-0.08	0.09	0.31
D3Z4I3	RNA-binding protein 24 OS=Mus musculus GN=Rbm24 PE=3 SV=1 - [RBM24_MOUSE]	4.24	2	1	1	1	0.02	-0.02	0.14	0.09
Q8VC85	U6 snRNA-associated Sm-like protein LSm1 OS=Mus musculus GN=Lsm1 PE=2 SV=1 [LSM1_MOUSE]	26.32	1	2	2	10	-0.08	-0.53	0.48	-0.24

Q8VDC1	FYVE and coiled-coil domain-containing protein 1 OS=Mus musculus GN=Fyco1 PE=1 SV=1 - [FYCO1_MOUSE]	5.71	1	4	5	9	-0.60	-0.62	-0.46	-0.48
Q05921	2-5A-dependent ribonuclease OS=Mus musculus GN=Rnasel PE=2 SV=2 - [RN5A_MOUSE]	19.18	1	15	15	36	0.01	0.08	0.16	0.14
Q9CY62-2	Isoform 2 of E3 ubiquitin-protein ligase RNF181 OS=Mus musculus GN=Rnf181 - [RN181_MOUSE]	6.78	3	1	1	1	0.71	0.13	0.90	0.32
Q0VGU4	MCG18019 OS=Mus musculus GN=Vgf PE=2 SV=1 - [Q0VGU4_MOUSE]	11.35	1	5	5	12	-0.14	-0.05	-0.13	0.00
Q4LDD4-3	Isoform 3 of Arf-GAP with Rho-GAP domain, ANK repeat and PH domain-containing protein 1 OS=Mus musculus GN=Arap1 - [ARAP1_MOUSE]	6.29	5	5	6	12	0.49	0.60	0.22	0.33
P70700	DNA-directed RNA polymerase I subunit RPA2 OS=Mus musculus GN=Polr1b PE=2 SV=2 - [RPA2_MOUSE]	0.97	1	1	1	1	0.68	0.91	0.56	0.79
D6RCX5	Mitochondrial fission regulator 1-like OS=Mus musculus GN=Mtfr1l PE=2 SV=1 - [D6RCX5_MOUSE]	3.96	4	1	1	2	-0.07	-0.18	-0.04	-0.14
Q9CQN1	Heat shock protein 75 kDa, mitochondrial OS=Mus musculus GN=Trap1 PE=1 SV=1 [TRAP1_MOUSE]	42.21	1	23	24	325	0.24	0.26	0.25	0.26
Q8BKU8-2	Isoform 2 of Transmembrane protein 87B OS=Mus musculus GN=Tmem87b - [TM87B_MOUSE]	1.22	3	1	1	2	0.18	0.36	0.18	0.36
A2AIR4	Protein Grin3a OS=Mus musculus GN=Grin3a PE=2 SV=1 - [A2AIR4_MOUSE]	1.35	4	1	2	3	-0.15	-0.20	-0.03	-0.07
P58742	Aladin OS=Mus musculus GN=Aaas PE=1 SV=1 - [AAAS_MOUSE]	7.69	1	2	2	12	0.13	0.31	-0.29	-0.11
B0QZV3	Protein Slc9a6 OS=Mus musculus GN=Slc9a6 PE=2 SV=1 - [B0QZV3_MOUSE]	5.82	2	3	3	6	-0.51	-0.29	-0.35	-0.25
Q3TPX4	Exocyst complex component 5 OS=Mus musculus GN=Exoc5 PE=1 SV=2 - [EXOC5_MOUSE]	4.80	3	3	4	4	-0.01	0.08	0.10	0.19
Q8C7H1	Methylmalonic aciduria type A homolog, mitochondrial OS=Mus musculus GN=Mmaa PE=2 SV=1 - [MMAA_MOUSE]	4.10	1	2	2	5	-0.02	-0.09	0.01	-0.06
Q8R3P8	Protein Rnf113a1 OS=Mus musculus GN=Rnf113a1 PE=2 SV=1 - [Q8R3P8_MOUSE]	5.28	1	2	2	3	0.15	0.18	0.50	0.54

Q80TC6	Zinc finger SWIM domain-containing protein 5 OS=Mus musculus GN=Zswim5 PE=2 SV=2 - [ZSWM5_MOUSE]	2.10	1	1	2	2	0.33	0.25	0.14	0.06
P61600	N-alpha-acetyltransferase 20 OS=Mus musculus GN=Naa20 PE=2 SV=1 - [NAA20_MOUSE]	9.55	6	2	2	3	1.06	0.63	1.08	0.48
Q4V9V3	Elongation of very long chain fatty acids (FEN1/Elo2, SUR4/Elo3, yeast)-like 1 OS=Mus musculus GN=Elovl1 PE=2 SV=1 - [Q4V9V3_MOUSE]	12.87	2	2	2	8	0.58	0.46	0.44	0.43
Q80TP3	E3 ubiquitin-protein ligase UBR5 OS=Mus musculus GN=Ubr5 PE=1 SV=2 - [UBR5_MOUSE]	2.47	2	4	4	10	0.19	0.22	0.75	0.47
Q9ES74	Serine/threonine-protein kinase Nek7 OS=Mus musculus GN=Nek7 PE=2 SV=1 - [NEK7_MOUSE]	21.52	3	6	6	19	0.52	0.02	0.32	0.03
Q6PDN3-3	Isoform 3 of Myosin light chain kinase, smooth muscle OS=Mus musculus GN=Mylk - [MYLK_MOUSE]	44.33	9	40	42	894	0.40	0.49	0.34	0.41
P70388-2	Isoform 2 of DNA repair protein RAD50 OS=Mus musculus GN=Rad50 - [RAD50_MOUSE]	4.72	7	5	5	9	0.31	-0.12	0.22	0.03
A2RTF1	Cation channel sperm-associated protein subunit beta OS=Mus musculus GN=Catsperb PE=1 SV=1 - [CTSRB_MOUSE]	1.98	1	1	2	2	-0.11	-0.13	0.43	0.42
Q9CXJ4	ATP-binding cassette sub-family B member 8, mitochondrial OS=Mus musculus GN=Abcb8 PE=2 SV=1 - [ABCB8_MOUSE]	8.37	3	5	5	13	-0.26	0.03	-0.16	0.14
Q5STT6	Protein FAM71B OS=Mus musculus GN=Fam71b PE=2 SV=2 - [FA71B_MOUSE]	5.62	1	1	1	1	0.29	-0.18	0.03	-0.45
Q8BSN5	PHD finger protein 23 OS=Mus musculus GN=Phf23 PE=2 SV=2 [PHF23_MOUSE]	4.74	4	2	2	2	0.05	0.06	0.19	0.20
O08573-2	Isoform Short of Galectin-9 OS=Mus musculus GN=Lgals9 - [LEG9_MOUSE]	22.36	4	5	5	41	-0.18	-0.09	-0.07	0.03
Q9CQE7	Endoplasmic reticulum-Golgi intermediate compartment protein 3 OS=Mus musculus GN=Ergic3 PE=2 SV=1 - [ERGI3_MOUSE]	5.74	4	2	2	9	0.45	0.35	0.19	0.25
P97500-4	Isoform 4 of Myelin transcription factor 1-like protein OS=Mus musculus GN=Myt1l - [MYT1L_MOUSE]	3.22	5	1	1	6	-0.63	-0.41	-0.39	-0.18

P49025-3	Isoform 3 of Citron Rho interacting kinase OS=Mus musculus GN=Cit - [CTRO_MOUSE]	4.32	9	2	8	9	-0.33	-0.50	0.10	-0.19
D3Z4R1	Protein Hfm1 OS=Mus musculus GN=Hfm1 PE=4 SV=2 - [D3Z4R1_MOUSE]	3.56	2	3	3	8	-0.26	0.13	-0.44	-0.09
Q99LB7	Sarcosine dehydrogenase, mitochondrial OS=Mus musculus GN=Sardh PE=1 SV=1 - [SARDH_MOUSE]	7.40	3	3	3	6	-0.31	-0.08	-0.22	-0.66
Q3UM45	Protein phosphatase 1 regulatory subunit 7 OS=Mus musculus GN=Ppp1r7 PE=1 SV=2 - [PP1R7_MOUSE]	33.52	2	11	11	58	0.17	0.07	0.18	0.14
P56388-2	Isoform Short of Cocaine- and amphetamine-regulated transcript protein OS=Mus musculus GN=Cartpt - [CART_MOUSE]	13.79	2	2	2	3	0.18	-0.17	0.07	-0.28
Q8BZF8	Phosphoglucomutase-like protein 5 OS=Mus musculus GN=Pgm5 PE=1 SV=2 - [PGM5_MOUSE]	51.15	1	27	29	550	-0.13	-0.10	-0.11	-0.14
Q7TRH2	MCG1048742 OS=Mus musculus GN=Olfr823 PE=3 SV=1 - [Q7TRH2_MOUSE]	8.25	2	1	1	5	0.08	-0.29	-0.09	-0.46
Q9EQH2	Endoplasmic reticulum aminopeptidase 1 OS=Mus musculus GN=Erap1 PE=2 SV=2 [ERAP1_MOUSE]	22.58	1	20	20	71	0.41	0.25	0.41	0.31
P54775	26S protease regulatory subunit 6B OS=Mus musculus GN=Psmc4 PE=1 SV=2 - [PRS6B_MOUSE]	42.11	1	13	13	56	0.63	0.73	0.55	0.59
Q02257	Junction plakoglobin OS=Mus musculus GN=Jup PE=1 SV=3 - [PLAK_MOUSE]	27.11	1	12	16	120	0.23	0.21	0.32	0.27
Q9DC69	NADH dehydrogenase [ubiquinone] 1 alpha subcomplex subunit 9, mitochondrial OS=Mus musculus GN=Ndufa9 PE=1 SV=2 - [NDUA9_MOUSE]	27.59	1	9	9	78	0.00	0.09	-0.08	0.05
Q9CSB4-2	Isoform 2 of Partitioning defective 3 homolog B OS=Mus musculus GN=Pard3b - [PAR3L_MOUSE]	2.66	3	1	3	5	-0.28	-0.21	-0.07	0.00
Q9CQ54	NADH dehydrogenase [ubiquinone] 1 subunit C2 OS=Mus musculus GN=Ndufc2 PE=1 SV=1 - [NDUC2_MOUSE]	39.17	1	7	7	48	0.01	0.00	-0.08	-0.11
F6VVY4	Protein Slc25a1 (Fragment) OS=Mus musculus GN=Slc25a1 PE=3 SV=1 - [F6VVY4_MOUSE]	15.74	2	3	3	19	-0.06	-0.17	-0.11	-0.16

A2AQA7	Intron-binding protein aquarius OS=Mus musculus GN=Aqr PE=2 SV=1 - [A2AQA7_MOUSE]	6.29	2	6	6	12	0.09	0.16	0.15	0.32
E9Q4K7	Protein Kif13b OS=Mus musculus GN=Kif13b PE=2 SV=1 - [E9Q4K7_MOUSE]	19.26	1	23	25	110	0.10	0.04	0.04	0.09
G3UY96	MutS protein homolog 5 OS=Mus musculus GN=Msh5 PE=2 SV=1 [G3UY96_MOUSE]	17.73	4	2	2	8	-0.33	0.20	-0.48	0.05
P35279	Ras-related protein Rab-6A OS=Mus musculus GN=Rab6a PE=1 SV=4 - [RAB6A_MOUSE]	53.37	14	11	12	105	0.21	0.14	0.17	0.17
Q8VDP6	CDP-diacylglycerol--inositol 3-phosphatidyltransferase OS=Mus musculus GN=Cdipt PE=1 SV=1 - [CDIPT_MOUSE]	9.86	1	2	2	12	-0.03	-0.16	-0.06	-0.07
Q05DV2	Dihydroxyacetone phosphate acyltransferase (Fragment) OS=Mus musculus GN=Gnpat PE=2 SV=1 - [Q05DV2_MOUSE]	6.84	2	2	2	2	-0.06	-0.17	-0.09	-0.19
Q6ZQI3	Malectin OS=Mus musculus GN=Mlec PE=2 SV=2 - [MLEC_MOUSE]	29.90	2	8	8	75	0.29	0.25	0.25	0.16
P70268	Serine/threonine-protein kinase N1 OS=Mus musculus GN=Pkn1 PE=1 SV=3 - [PKN1_MOUSE]	3.70	3	2	3	15	0.06	0.00	0.51	0.44
Q9CYA0	Cysteine-rich with EGF-like domain protein 2 OS=Mus musculus GN=Creld2 PE=2 SV=1 - [CREL2_MOUSE]	20.29	1	7	7	28	0.34	0.23	0.37	0.23
Q9D1C8	Vacuolar protein sorting associated protein 28 homolog OS=Mus musculus GN=Vps28 PE=2 SV=1 - [VPS28_MOUSE]	38.46	1	8	8	18	0.05	0.30	0.15	0.44
Q09324	Beta-1,3-galactosyl-O-glycosyl-glycoprotein beta-1,6-N-acetylglucosaminyltransferase OS=Mus musculus GN=Gcnt1 PE=1 SV=2 - [GCNT1_MOUSE]	3.04	2	1	1	1	0.02	1.21	-0.03	1.15
Q9EST4-3	Isoform 3 of Proteasome assembly chaperone 2 OS=Mus musculus GN=Psmg2 - [PSMG2_MOUSE]	4.02	2	1	1	1	-0.30	-0.98	0.13	-0.54
P97346-2	Isoform 2 of Nucleoredoxin OS=Mus musculus GN=Nxn - [NXN_MOUSE]	18.16	2	5	5	13	0.01	0.27	0.10	0.17
Q2M2N2	Speckle-type POZ protein-like OS=Mus musculus GN=Spopl PE=2 SV=3 - [SPOPL_MOUSE]	10.46	1	2	2	2	0.40	0.40	0.40	0.41

P54823	Probable ATP-dependent RNA helicase DDX6 OS=Mus musculus GN=Ddx6 PE=2 SV=1 - [DDX6_MOUSE]	34.37	4	12	13	80	0.34	0.11	0.34	0.21
P70335	Rho-associated protein kinase 1 OS=Mus musculus GN=Rock1 PE=1 SV=1 - [ROCK1_MOUSE]	19.65	2	21	26	68	0.18	0.14	0.24	0.18
Q9JL62	Glycolipid transfer protein OS=Mus musculus GN=Gltp PE=2 SV=3 - [GLTP_MOUSE]	31.10	3	7	7	35	0.71	0.54	0.42	0.44
O88533	Aromatic-L-amino-acid decarboxylase OS=Mus musculus GN=Ddc PE=2 SV=1 - [DDC_MOUSE]	17.71	2	9	9	33	-0.27	-0.37	-0.27	-0.43
A2A995	Uncharacterized protein C1orf168 homolog OS=Mus musculus PE=2 SV=1 - [CA168_MOUSE]	3.02	2	2	2	2	-0.01	-0.05	0.04	-0.06
A2ADR5	Monocarboxylate transporter 8 (Fragment) OS=Mus musculus GN=Slc16a2 PE=2 SV=1 - [A2ADR5_MOUSE]	4.08	2	1	1	1	-0.67	-0.86	2.99	2.80
Q9WTL7	Acyl-protein thioesterase 2 OS=Mus musculus GN=Lypla2 PE=1 SV=1 - [LYPA2_MOUSE]	33.77	1	7	7	35	0.16	0.12	0.05	0.12
Q5XJY5	Coatomer subunit delta OS=Mus musculus GN=Arcn1 PE=2 SV=2 - [COPD_MOUSE]	46.18	1	25	25	146	0.00	0.09	0.11	0.12
Q91WP6	Serine protease inhibitor A3N OS=Mus musculus GN=Serpina3n PE=1 SV=1 - [SPA3N_MOUSE]	41.15	6	1	14	91	0.88	0.91	0.67	0.70
O70503	Estradiol 17-beta-dehydrogenase 12 OS=Mus musculus GN=Hsd17b12 PE=2 SV=1 - [DHB12_MOUSE]	23.72	2	7	7	22	0.23	0.39	0.16	0.26
Q9D920	Loss of heterozygosity 12 chromosomal region 1 protein homolog OS=Mus musculus GN=Loh12cr1 PE=2 SV=1 - [L12R1_MOUSE]	13.85	2	2	2	2	0.34	0.23	0.12	0.01
Q6PEE2	CBP80/20-dependent translation initiation factor OS=Mus musculus GN=Ctif PE=1 SV=2 - [CTIF_MOUSE]	5.00	3	3	3	4	-0.38	-0.46	-0.46	-0.54
Q62351	Transferrin receptor protein 1 OS=Mus musculus GN=Tfrc PE=1 SV=1 - [TFR1_MOUSE]	28.57	2	22	22	50	0.92	0.74	0.85	0.84
Q91V12-2	Isoform A of Cytosolic acyl coenzyme A thioester hydrolase OS=Mus musculus GN=Acot7 - [BACH_MOUSE]	29.29	5	6	6	36	0.31	0.29	0.31	0.32

Q5SUA5	Unconventional myosin-Ig OS=Mus musculus GN=Myo1g PE=2 SV=1 - [MYO1G_MOUSE]	9.57	1	4	7	20	0.25	0.32	0.27	0.10
P97929	Breast cancer type 2 susceptibility protein homolog OS=Mus musculus GN=Brca2 PE=1 SV=2 - [BRCA2_MOUSE]	0.24	1	1	1	1	-0.15	-0.38	0.14	-0.09
O88291	DBIRD complex subunit ZNF326 OS=Mus musculus GN=Znf326 PE=1 SV=1 - [ZN326_MOUSE]	4.31	2	2	2	5	-0.28	-0.34	0.18	0.22
P61166	Transmembrane protein 258 OS=Mus musculus GN=Tmem258 PE=2 SV=1 - [TM258_MOUSE]	10.13	1	1	1	9	0.10	0.13	0.03	0.11
B9EKR1	Protein Ptprz1 OS=Mus musculus GN=Ptprz1 PE=2 SV=1 - [B9EKR1_MOUSE]	3.63	1	3	3	6	0.48	0.03	-0.13	-0.58
Q91ZU6	Dystonin OS=Mus musculus GN=Dst PE=1 SV=1 - [DYST_MOUSE]	2.11	8	8	12	20	-0.07	0.19	0.11	0.37
Q8K4D7-2	Isoform 2 of 1-phosphatidylinositol 4,5 bisphosphate phosphodiesterase zeta-1 OS=Mus musculus GN=Plcz1 - [PLCZ1_MOUSE]	3.17	2	1	1	4	0.09	-0.02	-0.05	-0.17
B1ARW4	NADH dehydrogenase [ubiquinone] iron-sulfur protein 5 (Fragment) OS=Mus musculus GN=Ndufs5 PE=2 SV=1 - [B1ARW4_MOUSE]	59.34	2	6	6	52	0.05	0.13	0.19	0.14
Q9EQC5	N-terminal kinase-like protein OS=Mus musculus GN=Scyl1 PE=1 SV=1 - [NTKL_MOUSE]	13.52	1	6	6	9	-0.59	0.02	-0.96	0.12
P14602-2	Isoform B of Heat shock protein beta-1 OS=Mus musculus GN=Hspb1 - [HSPB1_MOUSE]	64.97	4	11	11	223	0.38	0.46	0.33	0.38
D3YZY2	Protein Zc3h7a (Fragment) OS=Mus musculus GN=Zc3h7a PE=2 SV=1 - [D3YZY2_MOUSE]	3.88	5	1	1	1	0.31	0.34	0.40	0.42
Q61315-4	Isoform 4 of Adenomatous polyposis coli protein OS=Mus musculus GN=Apc - [APC_MOUSE]	2.47	14	4	5	9	-0.21	-0.43	0.33	0.15
Q9QZW0-2	Isoform 2 of Probable phospholipid-transporting ATPase 11C OS=Mus musculus GN=Atp11c - [AT11C_MOUSE]	2.34	5	1	2	7	-0.28	0.34	-0.13	0.18
P61021	Ras-related protein Rab-5B OS=Mus musculus GN=Rab5b PE=1 SV=1 - [RAB5B_MOUSE]	33.49	1	3	6	36	0.03	-0.11	0.11	-0.09
B2RXS4	Plexin-B2 OS=Mus musculus GN=Plxnb2 PE=1 SV=1 - [PLXB2_MOUSE]	14.60	1	21	21	77	-0.11	-0.15	-0.04	0.02

Q925E0	Gamma-2-syntrophin OS=Mus musculus GN=Sntg2 PE=2 SV=1 [SNTG2_MOUSE]	9.46	2	4	4	8	-0.45	-0.07	-0.36	-0.14
G5E866	Splicing factor 3B subunit 1 OS=Mus musculus GN=Sf3b1 PE=4 SV=1 - [G5E866_MOUSE]	30.98	2	30	30	126	0.28	0.23	0.22	0.22
Q9D7N3	28S ribosomal protein S9, mitochondrial OS=Mus musculus GN=Mrps9 PE=1 SV=3 - [RT09_MOUSE]	34.87	1	9	9	28	0.10	0.02	-0.10	-0.04
P80560	Receptor-type tyrosine-protein phosphatase N2 OS=Mus musculus GN=Ptprn2 PE=1 SV=2 - [PTPR2_MOUSE]	3.10	1	3	3	6	-0.39	-0.11	-0.36	-0.24
Q8BFW7-4	Isoform 4 of Lipoma-preferred partner homolog OS=Mus musculus GN=Lpp - [LPP_MOUSE]	56.35	1	1	17	676	0.37	0.77	0.24	0.64
Q80U35	Rho guanine nucleotide exchange factor 17 OS=Mus musculus GN=Arhgef17 PE=1 SV=2 - [ARHGH_MOUSE]	3.65	2	5	5	11	-0.17	-0.12	-0.21	-0.41
Q5F284	Phospholipid scramblase 3 (Fragment) OS=Mus musculus GN=Plscr3 PE=2 SV=1 - [Q5F284_MOUSE]	3.57	2	1	1	2	0.28	-0.10	0.14	-0.24
Q148W0	Probable phospholipid-transporting ATPase IC OS=Mus musculus GN=Atp8b1 PE=2 SV=2 - [AT8B1_MOUSE]	8.95	3	9	10	38	-0.06	-0.02	0.04	0.13
Q3UE31	Uncharacterized protein KIAA0930 homolog OS=Mus musculus PE=1 SV=2 - [K0930_MOUSE]	2.97	2	1	1	1	-1.17	-0.26	-1.32	-0.42
Q9JMD3	PCTP-like protein OS=Mus musculus GN=Stard10 PE=1 SV=1 - [PCTL_MOUSE]	46.05	9	9	9	58	0.01	0.03	0.03	0.06
Q61586	Glycerol-3-phosphate acyltransferase 1, mitochondrial OS=Mus musculus GN=Gpam PE=1 SV=2 - [GPAT1_MOUSE]	1.33	1	1	1	1	0.61	0.17	0.34	-0.11
P70271	PDZ and LIM domain protein 4 OS=Mus musculus GN=Pdlim4 PE=1 SV=3 - [PDLI4_MOUSE]	56.97	3	14	14	138	-0.25	-0.18	-0.16	-0.16
P40124	Adenylyl cyclase-associated protein 1 OS=Mus musculus GN=Cap1 PE=1 SV=4 - [CAP1_MOUSE]	51.90	2	18	19	328	0.48	0.47	0.48	0.48
P62855	40S ribosomal protein S26 OS=Mus musculus GN=Rps26 PE=2 SV=3 - [RS26_MOUSE]	20.87	1	2	2	23	-0.21	-0.06	0.11	0.10
Q8CAS9-3	Isoform 3 of Poly [ADP ribose] polymerase 9 OS=Mus musculus GN=Parp9 - [PARP9_MOUSE]	21.24	4	12	12	34	0.29	0.05	0.31	0.23

P36371	Antigen peptide transporter 2 OS=Mus musculus GN=Tap2 PE=2 SV=1 - [TAP2_MOUSE]	17.81	1	10	10	29	0.25	0.17	0.25	0.29
Q9DBH5	Vesicular integral-membrane protein VIP36 OS=Mus musculus GN=Lman2 PE=2 SV=2 - [LMAN2_MOUSE]	47.21	1	13	13	113	0.07	0.08	0.11	0.07
Q3UQ28	Peroxidasin homolog OS=Mus musculus GN=Pxdn PE=2 SV=2 - [PXDN_MOUSE]	5.69	2	6	6	14	0.17	0.05	-0.10	0.17
Q9EPR5	VPS10 domain-containing receptor SorCS2 OS=Mus musculus GN=Sorcs2 PE=1 SV=2 - [SORC2_MOUSE]	6.38	3	5	6	13	-0.28	-0.61	-0.18	-0.30
Q9ER38	Torsin-3A OS=Mus musculus GN=Tor3a PE=1 SV=2 - [TOR3A_MOUSE]	2.08	1	1	1	1	0.45	0.34	0.45	0.34
O09110-2	Isoform 1 of Dual specificity mitogen-activated protein kinase kinase 3 OS=Mus musculus GN=Map2k3 [MP2K3_MOUSE]	28.98	2	5	8	27	0.25	0.41	0.32	0.58
O08648-2	Isoform B of Mitogen-activated protein kinase kinase kinase 4 OS=Mus musculus GN=Map3k4 - [M3K4_MOUSE]	2.72	2	3	3	3	-0.04	-0.19	0.22	0.05
Q64459	Cytochrome P450 3A11 OS=Mus musculus GN=Cyp3a11 PE=1 SV=1 - [CP3AB_MOUSE]	1.39	2	1	1	2	-0.12	0.08	-0.12	0.08
Q9ES83	Blood vessel epicardial substance OS=Mus musculus GN=Bves PE=1 SV=1 - [POPD1_MOUSE]	16.48	1	5	5	13	-0.02	-0.04	-0.39	-0.15
Q8BWR8	Rhophilin-2 OS=Mus musculus GN=Rhpn2 PE=1 SV=2 - [RHPN2_MOUSE]	5.39	1	3	3	8	0.17	0.07	0.29	0.18
H3BKC5	U6 snRNA-associated Sm-like protein LSm5 (Fragment) OS=Mus musculus GN=Lsm5 PE=2 SV=1 - [H3BKC5_MOUSE]	14.29	2	1	1	2	0.01	0.00	0.18	-0.03
Q9R098	Hepatocyte growth factor activator OS=Mus musculus GN=Hgfac PE=1 SV=1 [HGFA_MOUSE]	9.04	1	5	5	11	0.19	-0.08	0.29	0.17
Q8VED2	Biogenesis of lysosome-related organelles complex 1 subunit 4 OS=Mus musculus GN=Bloc1s4 PE=1 SV=1 - [BL1S4_MOUSE]	2.79	1	1	1	1	0.15	0.21	0.11	0.17
P62908	40S ribosomal protein S3 OS=Mus musculus GN=Rps3 PE=1 SV=1 - [RS3_MOUSE]	74.49	2	18	18	336	0.32	0.35	0.28	0.28
B1AZI6	THO complex subunit 2 OS=Mus musculus GN=Thoc2 PE=2 SV=1 - [THOC2_MOUSE]	4.45	5	3	7	14	0.40	0.54	0.44	0.58

P20029	78 kDa glucose-regulated protein OS=Mus musculus GN=Hspa5 PE=1 SV=3 - [GRP78_MOUSE]	62.14	1	45	48	2008	0.17	0.27	0.14	0.25
Q61749	Translation initiation factor eIF-2B subunit delta OS=Mus musculus GN=Eif2b4 PE=2 SV=2 - [EI2BD_MOUSE]	14.69	4	6	6	28	0.46	0.32	0.40	0.41
O88708	Origin recognition complex subunit 4 OS=Mus musculus GN=Orc4 PE=1 SV=2 - [ORC4_MOUSE]	1.39	1	1	1	5	-0.01	0.25	0.00	0.26
Q9Z103	Activity-dependent neuroprotector homeobo protein OS=Mus musculus GN=Adnp PE=2 SV=1 - [ADNP_MOUSE]	10.75	2	6	6	16	-0.07	-0.09	-0.19	-0.04
G3UYJ3	Solute carrier family 25 member 51 (Fragment) OS=Mus musculus GN=Slc25a51 PE=2 SV=1 - [G3UYJ3_MOUSE]	9.18	4	2	2	2	-0.26	0.04	-0.36	-0.06
A2AGG1	Protein Ttc17 OS=Mus musculus GN=Ttc17 PE=2 SV=1 - [A2AGG1_MOUSE]	1.12	4	1	1	1	-1.09	-1.00	-0.41	-0.32
Q64261	Cyclin-dependent kinase 6 OS=Mus musculus GN=Cdk6 PE=1 SV=2 - [CDK6_MOUSE]	19.63	31	4	5	28	0.45	-0.05	0.17	-0.06
O88329	Unconventional myosin-Ia OS=Mus musculus GN=Myo1a PE=2 SV=2 - [MYO1A_MOUSE]	37.01	1	30	35	185	0.26	0.21	0.25	0.19
E9Q3L2	Protein Pi4ka OS=Mus musculus GN=Pi4ka PE=4 SV=1 - [E9Q3L2_MOUSE]	2.89	5	5	5	7	0.06	0.09	-0.04	-0.01
Q924T7-2	Isoform 2 of E3 ubiquitin-protein ligase RNF31 OS=Mus musculus GN=Rnf31 - [RNF31_MOUSE]	6.91	4	5	5	20	0.07	0.02	0.19	0.15
E9Q8R5	TraB domain-containing protein OS=Mus musculus GN=Trabd PE=2 SV=1 - [E9Q8R5_MOUSE]	9.69	3	2	2	6	0.00	0.15	0.02	0.07
Q61655	ATP-dependent RNA helicase DDX19A OS=Mus musculus GN=Ddx19a PE=2 SV=2 - [DD19A_MOUSE]	16.11	4	7	7	55	0.46	0.47	0.45	0.52
G3X8R0	Receptor accessory protein 5, isoform CRA_a OS=Mus musculus GN=Reep5 PE=4 SV=1 - [G3X8R0_MOUSE]	19.58	2	4	4	32	-0.02	0.01	-0.10	-0.12
Q9D1I2	Bcl10-interacting CARD protein OS=Mus musculus PE=2 SV=1 - [BINCA_MOUSE]	7.10	1	1	1	5	0.21	-0.17	0.23	-0.15
P63239	Neuroendocrine convertase 1 OS=Mus musculus GN=Pcsk1 PE=1 SV=1 - [NEC1_MOUSE]	6.77	1	3	3	7	-0.47	-0.86	-0.09	-0.33

Q8BPU7	Engulfment and cell motility protein 1 OS=Mus musculus GN=Elmo1 PE=1 SV=2 - [ELMO1_MOUSE]	18.16	4	10	14	32	0.08	0.10	0.21	0.20
P10107	Annexin A1 OS=Mus musculus GN=Anxa1 PE=1 SV=2 - [ANXA1_MOUSE]	65.32	1	18	20	389	0.61	0.52	0.61	0.48
Q8K363	ATP-dependent RNA helicase DDX18 OS=Mus musculus GN=Ddx18 PE=2 SV=1 - [DDX18_MOUSE]	6.06	1	4	4	9	0.37	0.31	0.36	0.37
E9PWV3	Ribosomal protein S6 kinase OS=Mus musculus GN=Rps6ka1 PE=2 SV=1 - [E9PWV3_MOUSE]	38.80	25	14	23	103	0.22	0.39	0.22	0.26
P70295	Ancient ubiquitous protein 1 OS=Mus musculus GN=Aup1 PE=2 SV=1 - [AUP1_MOUSE]	7.32	2	4	4	6	0.17	-0.15	0.09	-0.16
Q9DBZ5	Eukaryotic translation initiation factor 3 subunit K OS=Mus musculus GN=Eif3k PE=1 SV=1 - [EIF3K_MOUSE]	38.07	2	6	6	47	0.56	0.71	0.55	0.60
Q9D084-2	Isoform 2 of Centromere protein S OS=Mus musculus GN=Apitd1 - [CENPS_MOUSE]	15.79	3	1	1	1	-0.30	0.21	0.02	0.53
Q8C178	Acyl-coenzyme A oxidase OS=Mus musculus GN=Acox3 PE=2 SV=1 - [Q8C178_MOUSE]	7.17	3	3	4	10	0.75	0.32	0.72	0.29
Q6ZPR6-2	Isoform 2 of Inhibitor of Bruton tyrosine kinase OS=Mus musculus GN=Ibtk - [IBTK_MOUSE]	0.56	2	1	1	1	-0.18	-0.03	0.12	0.27
G5E8E3	Protein 2310022A10Rik OS=Mus musculus GN=2310022A10Rik PE=4 SV=1 - [G5E8E3_MOUSE]	3.15	1	1	1	3	-0.41	-0.13	0.08	0.35
P52430	Serum paraoxonase/arylesterase 1 OS=Mus musculus GN=Pon1 PE=1 SV=2 - [PON1_MOUSE]	11.27	3	2	2	8	0.05	0.03	0.23	0.26
F6SPK0	Ubiquitin-conjugating enzyme E2 J1 (Fragment) OS=Mus musculus GN=Ube2j1 PE=4 SV=1 - [F6SPK0_MOUSE]	5.73	2	1	1	2	0.00	0.60	-0.02	0.55
E9Q7L0	Protein Ogdhl OS=Mus musculus GN=Ogdhl PE=2 SV=1 - [E9Q7L0_MOUSE]	18.95	1	5	13	80	-0.86	-0.85	-0.83	-0.83
A9C445	Protein 2610015P09Rik OS=Mus musculus GN=2610015P09Rik PE=2 SV=1 - [A9C445_MOUSE]	2.80	3	1	2	2	-0.05	-0.22	0.08	-0.08
Q9D4V7	Rab-like protein 3 OS=Mus musculus GN=Rabl3 PE=2 SV=1 [RABL3_MOUSE]	4.66	1	1	1	9	0.06	-0.05	0.04	0.08

Q8BT51	Cytochrome c oxidase assembly factor 4 homolog, mitochondrial OS=Mus musculus GN=COA4 PE=3 SV=1 - [COA4_MOUSE]	17.24	1	1	1	6	-0.15	-0.12	-0.07	-0.12
Q8K4F5	Alpha/beta hydrolase domain-containing protein 11 OS=Mus musculus GN=Abhd11 PE=2 SV=1 - [ABHDB_MOUSE]	17.59	2	4	4	13	0.14	0.22	0.17	0.20
Q07139	Protein ECT2 OS=Mus musculus GN=Ect2 PE=1 SV=2 - [ECT2_MOUSE]	3.29	5	2	4	4	0.61	0.46	0.38	0.22
Q8C7X2-2	Isoform 2 of ER membrane protein complex subunit 1 OS=Mus musculus GN=Emc1 - [EMC1_MOUSE]	16.40	3	14	14	43	0.12	0.13	0.03	0.03
P49443	Protein phosphatase 1A OS=Mus musculus GN=Ppm1a PE=1 SV=1 - [PPM1A_MOUSE]	29.58	1	7	10	51	0.22	0.02	0.23	0.32
E9PZJ8	Activating signal cointegrator 1 complex subunit 3 OS=Mus musculus GN=Ascc3 PE=2 SV=1 - [ASCC3_MOUSE]	3.96	2	5	5	7	0.49	-0.10	0.08	-0.24
D3Z0Q6	Glycogen [starch] synthase, muscle OS=Mus musculus GN=Gys1 PE=2 SV=1 - [D3Z0Q6_MOUSE]	9.64	2	4	4	14	0.06	-0.10	0.23	0.29
Q8K3J1	NADH dehydrogenase [ubiquinone] iron-sulfur protein 8, mitochondrial OS=Mus musculus GN=Ndufs8 PE=1 SV=1 - [NDUS8_MOUSE]	32.55	1	5	5	89	0.21	0.21	0.08	0.21
P18828-2	Isoform 2 of Syndecan-1 OS=Mus musculus GN=Sdc1 - [SDC1_MOUSE]	19.85	2	2	2	4	0.33	0.48	0.21	0.37
Q9Z127	Large neutral amino acids transporter small subunit 1 OS=Mus musculus GN=Slc7a5 PE=1 SV=2 - [LAT1_MOUSE]	2.73	1	1	1	2	-0.24	-0.22	-0.32	-0.30
Q80Y14	Glutaredoxin-related protein 5, mitochondrial OS=Mus musculus GN=Glrx5 PE=2 SV=2 [GLRX5_MOUSE]	34.87	1	4	5	75	0.06	0.25	0.09	0.31
Q91WQ3	Tyrosine--tRNA ligase, cytoplasmic OS=Mus musculus GN=Yars PE=2 SV=3 - [SYYC_MOUSE]	62.12	3	34	35	198	0.34	0.33	0.31	0.31
Q99M31	Heat shock 70 kDa protein 14 OS=Mus musculus GN=Hspa14 PE=2 SV=2 - [HSP7E_MOUSE]	23.58	8	7	8	15	-0.22	0.06	-0.26	0.06

Q61496	Probable ATP-dependent RNA helicase DDX4 OS=Mus musculus GN=Ddx4 PE=1 SV=2 - [DDX4_MOUSE]	9.12	2	1	5	23	0.70	0.96	0.70	0.96
E9PX53	Serine/threonine-protein phosphatase 4 regulatory subunit 1 OS=Mus musculus GN=Ppp4r1 PE=2 SV=1 - [E9PX53_MOUSE]	4.07	9	4	4	9	0.19	-0.02	0.36	0.15
Q9CZP5	Mitochondrial chaperone BCS1 OS=Mus musculus GN=Bcs1l PE=1 SV=1 [BCS1_MOUSE]	10.53	1	4	4	7	0.02	-0.17	0.16	-0.03
Q3U0V2	Tumor necrosis factor receptor type 1-associated DEATH domain protein OS=Mus musculus GN=Tradd PE=1 SV=1 [TRADD_MOUSE]	30.32	3	9	9	75	0.31	0.11	0.47	0.12
P10852	4F2 cell-surface antigen heavy chain OS=Mus musculus GN=Slc3a2 PE=1 SV=1 - [4F2_MOUSE]	19.20	2	9	9	38	0.24	0.40	0.11	0.24
Q8VCF1	Soluble calcium-activated nucleotidase 1 OS=Mus musculus GN=Cant1 PE=2 SV=1 [CANT1_MOUSE]	33.25	3	12	12	28	-0.01	0.04	0.08	0.07
B9EHJ3	Tight junction protein ZO-1 OS=Mus musculus GN=Tjp1 PE=2 SV=1 - [B9EHJ3_MOUSE]	23.80	1	1	31	128	-0.17	-0.04	0.20	0.34
Q9D1Q4	Dolichol-phosphate mannosyltransferase subunit 3 OS=Mus musculus GN=Dpm3 PE=3 SV=1 - [DPM3_MOUSE]	10.87	1	1	1	23	-0.03	0.13	0.09	0.21
P54987	Cis-aconitate decarboxylase OS=Mus musculus GN=Irg1 PE=1 SV=2 - [IRG1_MOUSE]	1.64	1	1	1	1	-3.74	0.23	-4.00	-0.02
E9PXM9	Protein Fbxw24 OS=Mus musculus GN=Fbxw24 PE=4 SV=1 - [E9PXM9_MOUSE]	4.51	1	1	1	3	0.19	0.29	0.63	0.73
E9Q361	Integrator complex subunit 10 OS=Mus musculus GN=Ints10 PE=2 SV=1 - [E9Q361_MOUSE]	1.52	3	1	1	1	1.13	-0.18	1.45	0.14
O35857	Mitochondrial import inner membrane translocase subunit TIM44 OS=Mus musculus GN=Timm44 PE=2 SV=2 - [TIM44_MOUSE]	26.55	2	12	12	44	0.29	0.22	0.26	0.20
Q9R1I1	Carbohydrate sulfotransferase 4 OS=Mus musculus GN=Chst4 PE=2 SV=1 [CHST4_MOUSE]	7.99	1	3	3	8	0.87	0.89	0.83	0.85

D3Z2Q3	Diablo homolog, mitochondrial OS=Mus musculus GN=Diablo PE=2 SV=1 - [D3Z2Q3_MOUSE]	10.92	3	2	2	25	0.01	0.04	0.02	0.14
Q8C7R4	Ubiquitin-like modifier-activating enzyme 6 OS=Mus musculus GN=Uba6 PE=1 SV=1 - [UBA6_MOUSE]	17.28	2	12	12	33	0.31	0.41	0.35	0.27
Q04857	Collagen alpha-1(VI) chain OS=Mus musculus GN=Col6a1 PE=2 SV=1 - [CO6A1_MOUSE]	20.78	1	17	17	188	-0.44	-0.53	-0.44	-0.55
Q9R1Q8	Transgelin-3 OS=Mus musculus GN=Tagln3 PE=1 SV=1 - [TAGL3_MOUSE]	30.65	1	3	6	49	-0.34	-0.21	-0.14	0.09
P47857	6-phosphofructokinase, muscle type OS=Mus musculus GN=Pfkm PE=1 SV=3 - [K6PF_MOUSE]	24.74	1	12	14	49	0.09	0.19	0.20	0.00
P31750	RAC-alpha serine/threonine-protein kinase OS=Mus musculus GN=Akt1 PE=1 SV=2 - [AKT1_MOUSE]	12.50	6	2	5	13	0.44	0.09	0.30	-0.06
Q8JZK9	Hydroxymethylglutaryl-CoA synthase, cytoplasmic OS=Mus musculus GN=Hmgcs1 PE=1 SV=1 - [HMCS1_MOUSE]	36.15	1	12	14	90	0.71	0.53	0.76	0.75
Q9D7M1	Glucose-induced degradation protein 8 homolog OS=Mus musculus GN=Gid8 PE=2 SV=1 - [GID8_MOUSE]	23.25	1	3	3	23	-0.21	-0.34	0.04	0.06
H7BX02	Probable E3 ubiquitin-protein ligase Roquin OS=Mus musculus GN=Rc3h1 PE=2 SV=1 - [H7BX02_MOUSE]	2.14	4	2	2	5	0.62	0.22	0.58	0.18
Q6PAR5-2	Isoform 2 of GTPase-activating protein and VPS9 domain-containing protein 1 OS=Mus musculus GN=Gapvd1 - [GAPD1_MOUSE]	10.09	12	14	14	34	0.35	0.13	0.27	0.19
D3Z5F9	Protein YIF1B (Fragment) OS=Mus musculus GN=Yif1b PE=2 SV=1 - [D3Z5F9_MOUSE]	13.89	4	1	1	4	0.23	-0.05	0.49	0.21
Q8VI59-2	Isoform 2 of Pecanex-like protein 3 OS=Mus musculus GN=Pcnxl3 - [PCX3_MOUSE]	0.86	2	1	1	4	-0.05	0.00	-0.19	-0.14
Q9D3N8-3	Isoform 3 of Growth hormone-regulated TBC protein 1 OS=Mus musculus GN=Grtp1 - [GRTP1_MOUSE]	17.44	4	4	4	17	0.08	0.00	0.13	-0.02
Q8BMJ2	Leucine--tRNA ligase, cytoplasmic OS=Mus musculus GN=Lars PE=2 SV=2 - [SYLC_MOUSE]	29.88	1	29	29	132	0.42	0.45	0.37	0.42

Q5SQM0	Echinoderm microtubule-associated protein-like 6 OS=Mus musculus GN=Eml6 PE=2 SV=1 - [EMAL6_MOUSE]	1.74	1	1	2	6	-0.12	0.14	-0.24	0.02
E9Q1B3	Uncharacterized protein OS=Mus musculus GN=Gm7275 PE=4 SV=1 - [E9Q1B3_MOUSE]	5.67	1	1	1	1	0.34	1.28	-0.54	0.40
Q8VE10	N-alpha-acetyltransferase 40 OS=Mus musculus GN=Naa40 PE=2 SV=1 - [NAA40_MOUSE]	3.80	1	1	1	2	0.60	0.10	0.55	0.05
Q8R4C2	RUN and FYVE domain-containing protein 2 OS=Mus musculus GN=Rufy2 PE=1 SV=2 - [RUFY2_MOUSE]	3.63	4	1	3	8	0.79	0.98	0.81	1.00
Q9Z1P6	NADH dehydrogenase [ubiquinone] 1 alpha subcomplex subunit 7 OS=Mus musculus GN=Ndufa7 PE=1 SV=3 - [NDUA7_MOUSE]	66.37	1	9	9	72	0.01	0.09	-0.11	-0.02
Q8BG95	Protein phosphatase 1 regulatory subunit 12B OS=Mus musculus GN=Ppp1r12b PE=1 SV=2 - [MYPT2_MOUSE]	11.58	3	8	12	110	0.27	0.26	0.24	0.25
Q6NVF2	Phospholipase D1 OS=Mus musculus GN=Pld1 PE=2 SV=1 - [Q6NVF2_MOUSE]	5.79	7	5	5	8	0.00	-0.44	-0.12	-0.10
Q99KR3	Beta-lactamase-like protein 2 OS=Mus musculus GN=Lactb2 PE=1 SV=1 - [LACB2_MOUSE]	19.10	1	8	8	23	0.34	0.12	0.38	0.23
O35136-2	Isoform Short of Neural cell adhesion molecule 2 OS=Mus musculus GN=Ncam2 - [NCAM2_MOUSE]	12.52	2	6	6	15	-0.32	-0.44	-0.50	-0.51
Q80W68	Kin of IRRE-like protein 1 OS=Mus musculus GN=Kirrel PE=1 SV=1 - [KIRR1_MOUSE]	6.59	2	3	4	8	-0.08	-0.16	0.04	-0.03
P49769	Presenilin-1 OS=Mus musculus GN=Psen1 PE=1 SV=1 - [PSN1_MOUSE]	10.28	5	3	3	14	0.12	0.54	0.44	0.78
Q8VIM9	Immunity-related GTPase family Q protein OS=Mus musculus GN=Irgq PE=2 SV=1 - [IRGQ_MOUSE]	5.83	1	3	3	5	0.27	0.04	0.53	0.30
Q61656	Probable ATP-dependent RNA helicase DDX5 OS=Mus musculus GN=Ddx5 PE=1 SV=2 - [DDX5_MOUSE]	39.09	5	21	28	286	0.34	0.39	0.35	0.44
E9QA15	Protein Cald1 OS=Mus musculus GN=Cald1 PE=2 SV=1 - [E9QA15_MOUSE]	46.48	2	4	40	946	-0.01	-0.03	-0.26	-0.28

P52480-2	Isoform M1 of Pyruvate kinase PKM OS=Mus musculus GN=Pkm - [KPYM_MOUSE]	63.65	1	3	32	1260	0.15	0.33	0.15	0.32
E9Q6R7	Protein Utrn OS=Mus musculus GN=Utrn PE=2 SV=1 - [E9Q6R7_MOUSE]	21.31	4	56	63	181	-0.04	-0.02	-0.07	-0.05
P70404	Isocitrate dehydrogenase [NAD] subunit gamma 1, mitochondrial OS=Mus musculus GN=Idh3g PE=1 SV=1 - [IDHG1_MOUSE]	43.00	1	13	13	123	-0.22	-0.22	-0.09	-0.16
F7BWJ0	Vang-like protein 1 (Fragment) OS=Mus musculus GN=Vangl1 PE=4 SV=1 - [F7BWJ0_MOUSE]	10.67	2	2	2	3	0.15	0.13	-0.08	-0.10
O35639	Annexin A3 OS=Mus musculus GN=Anxa3 PE=1 SV=4 - [ANXA3_MOUSE]	65.63	1	22	25	438	0.51	0.44	0.48	0.45
Q91WV0	Protein Dr1 OS=Mus musculus GN=Dr1 PE=2 SV=1 - [NC2B_MOUSE]	7.39	1	1	1	5	-0.08	-0.14	-0.01	-0.07
Q8R127	Saccharopine dehydrogenase-like oxidoreductase OS=Mus musculus GN=Sccpdh PE=2 SV=1 - [SCPDL_MOUSE]	4.90	3	2	2	3	0.13	0.32	0.09	0.27
P47962	60S ribosomal protein L5 OS=Mus musculus GN=Rpl5 PE=1 SV=3 - [RL5_MOUSE]	41.08	2	14	15	150	0.20	0.12	0.21	0.19
Q8K1M6-3	Isoform 3 of Dynamin-1-like protein OS=Mus musculus GN=Dnm1l - [DNM1L_MOUSE]	33.91	6	21	21	141	0.24	0.21	0.29	0.26
Q922Q8	Leucine-rich repeat-containing protein 59 OS=Mus musculus GN=Lrrc59 PE=2 SV=1 - [LRC59_MOUSE]	34.53	1	11	11	150	0.63	0.64	0.56	0.58
Q8VI24-2	Isoform 2 of DNA-binding protein SATB2 OS=Mus musculus GN=Satb2 - [SATB2_MOUSE]	19.29	3	8	10	27	0.25	0.25	0.41	0.24
E9Q2B0	General transcription factor 3C polypeptide 4 OS=Mus musculus GN=Gtf3c4 PE=2 SV=1 - [E9Q2B0_MOUSE]	3.70	4	2	2	2	0.08	0.42	-0.07	0.27
E9Q704	Peptidyl-glycine alpha-amidating monooxygenase OS=Mus musculus GN=Pam PE=2 SV=1 - [E9Q704_MOUSE]	14.32	5	8	8	36	-0.27	-0.23	-0.28	-0.17
P14576	Signal recognition particle 54 kDa protein OS=Mus musculus GN=Srp54 PE=1 SV=2 [SRP54_MOUSE]	28.97	3	11	11	41	0.32	0.38	0.35	0.41
P10833	Ras-related protein R-Ras OS=Mus musculus GN=Rras PE=2 SV=1 - [RRAS_MOUSE]	40.37	1	7	9	218	-0.02	0.00	-0.10	-0.09
Q8BSZ2	AP-3 complex subunit sigma-2 OS=Mus musculus GN=Ap3s2 PE=1 SV=1 - [AP3S2_MOUSE]	5.18	1	1	1	2	0.00	-0.02	-0.07	-0.09

Q9D5J6	Sedoheptulokinase OS=Mus musculus GN=Shpk PE=1 SV=1 - [SHPK_MOUSE]	6.30	1	3	3	11	-0.26	-0.44	-0.25	-0.44
Q8BPB0	MOB kinase activator 1B OS=Mus musculus GN=Mob1b PE=1 SV=3 - [MOB1B_MOUSE]	17.13	4	4	4	17	0.44	0.83	0.62	0.70
O08663	Methionine aminopeptidase 2 OS=Mus musculus GN=Metap2 PE=1 SV=1 - [MAP2_MOUSE]	31.17	7	13	13	56	0.20	0.24	0.17	0.25
Q9DBG1	Sterol 26-hydroxylase, mitochondrial OS=Mus musculus GN=Cyp27a1 PE=1 SV=1 - [CP27A_MOUSE]	5.82	1	2	2	4	-0.29	-1.04	-0.41	-1.16
P62245	40S ribosomal protein S15a OS=Mus musculus GN=Rps15a PE=2 SV=2 - [RS15A_MOUSE]	56.15	4	7	7	50	0.24	0.24	0.19	0.32
Q91VV4-2	Isoform 2 of DENN domain-containing protein 2D OS=Mus musculus GN=Dennd2d - [DEN2D_MOUSE]	15.45	2	4	4	5	-0.03	0.26	0.05	0.33
E9Q933	Transmembrane protein 11, mitochondrial OS=Mus musculus GN=Tmem11 PE=2 SV=1 - [E9Q933_MOUSE]	4.55	2	1	1	2	-0.21	-0.57	-0.22	-0.59
D3YZ05	Translation initiation factor eIF-2B subunit alpha (Fragment) OS=Mus musculus GN=Eif2b1 PE=2 SV=1 - [D3YZ05_MOUSE]	4.51	1	1	1	7	0.37	0.51	0.15	0.36
B9EKI3	TATA element modulatory factor OS=Mus musculus GN=Tmf1 PE=1 SV=2 - [TMF1_MOUSE]	15.86	2	12	12	27	0.12	0.09	0.10	0.04
E9PV12	Cullin-5 OS=Mus musculus GN=Cul5 PE=2 SV=1 - [E9PV12_MOUSE]	11.23	4	8	9	27	0.24	0.28	0.19	0.13
J3QQ22	Serine/threonine-protein kinase SMG1 OS=Mus musculus GN=Smg1 PE=4 SV=1 - [J3QQ22_MOUSE]	0.89	2	2	3	7	0.00	0.34	-0.13	0.21
B2RWE3	CDGSH iron sulfur domain 3 OS=Mus musculus GN=Cisd3 PE=2 SV=1 - [B2RWE3_MOUSE]	24.63	2	3	3	10	-0.03	0.18	0.02	0.15
G8JL83	YrdC domain-containing protein, mitochondrial (Fragment) OS=Mus musculus GN=Yrdc PE=2 SV=1 - [G8JL83_MOUSE]	8.57	1	1	1	1	0.32	0.55	0.37	0.60
Q91ZJ5	UTP--glucose-1-phosphate uridylyltransferase OS=Mus musculus GN=Ugp2 PE=2 SV=3 - [UGPA_MOUSE]	55.51	2	26	26	214	0.15	0.11	0.18	0.17

Q9QXM1-3	Isoform 3 of Junction-mediating and -regulatory protein OS=Mus musculus GN=Jmy - [JMY_MOUSE]	7.78	3	5	5	11	-1.03	-0.61	-0.78	-0.48
Q99J72-3	Isoform 3 of DNA dC->dU-editing enzyme APOBEC-3 OS=Mus musculus GN=Apobec3 - [ABEC3_MOUSE]	16.01	14	5	5	9	0.13	-0.06	0.35	0.27
Q9CW79-2	Isoform 2 of Golgin subfamily A member 1 OS=Mus musculus GN=Golga1 - [GOGA1_MOUSE]	10.78	3	6	7	27	0.23	0.20	0.03	0.05
Q8R3K3	Pentatricopeptide repeat containing protein 2, mitochondrial OS=Mus musculus GN=Ptcd2 PE=2 SV=1 - [PTCD2_MOUSE]	10.76	1	4	4	4	0.58	0.60	0.36	0.39
Q9DBC7	cAMP-dependent protein kinase type I- alpha regulatory subunit OS=Mus musculus GN=Prkar1a PE=1 SV=3 - [KAP0_MOUSE]	40.42	7	13	13	94	0.39	0.39	0.34	0.30
Q8BR07-2	Isoform 2 of Protein bicaudal D homolog 1 OS=Mus musculus GN=Bicd1 - [BICD1_MOUSE]	9.05	6	2	2	9	0.08	0.19	-0.09	0.12
Q9EQW7	Kinesin-like protein KIF13A OS=Mus musculus GN=Kif13a PE=1 SV=1 - [KI13A_MOUSE]	8.86	1	1	12	24	0.17	0.19	-0.31	0.03
P82343-2	Isoform 2 of N-acylglucosamine 2-epimerase OS=Mus musculus GN=Renbp - [RENBP_MOUSE]	6.73	3	3	3	12	0.60	0.99	0.50	0.88
Q922Q9	Chitinase domain-containing protein 1 OS=Mus musculus GN=Chid1 PE=2 SV=1 [CHID1_MOUSE]	20.61	4	7	7	25	-0.25	-0.15	-0.20	-0.14
E9QPZ4	TD and POZ domain-containing protein 1 OS=Mus musculus GN=Tdpoz1 PE=4 SV=1 - [E9QPZ4_MOUSE]	7.40	17	1	2	5	0.71	0.03	0.53	-0.15
E9QPX3	NADH dehydrogenase [ubiquinone] iron-sulfur protein 4, mitochondrial OS=Mus musculus GN=Ndufs4 PE=4 SV=1 - [E9QPX3_MOUSE]	38.29	2	8	8	53	0.04	0.12	-0.04	0.05
Q8BFY9-2	Isoform 2 of Transportin-1 OS=Mus musculus GN=Tnpo1 - [TNPO1_MOUSE]	8.20	4	4	7	30	0.07	-0.01	0.08	0.05
P27784	C-C motif chemokine 6 OS=Mus musculus GN=Ccl6 PE=1 SV=1 - [CCL6_MOUSE]	10.34	1	2	2	3	0.12	-0.16	0.40	0.12
Q80YS6	Actin filament-associated protein 1 OS=Mus musculus GN=Afap1 PE=1 SV=1 [AFAP1_MOUSE]	8.21	2	5	6	20	0.12	0.21	0.23	0.32

Q9CZA6-2	Isoform 2 of Nuclear distribution protein nudE homolog 1 OS=Mus musculus GN=Nde1 - [NDE1_MOUSE]	23.03	6	5	6	11	0.11	0.16	-0.15	-0.10
B1ATW6	EF-hand calcium-binding domain-containing protein 9 OS=Mus musculus GN=Efcab9 PE=2 SV=1 - [B1ATW6_MOUSE]	5.36	2	1	1	1	-1.43	-3.42	-0.96	-2.95
E9PYD1	Protein Fam98c OS=Mus musculus GN=Fam98c PE=2 SV=1 - [E9PYD1_MOUSE]	18.60	5	4	4	19	0.07	-0.11	0.04	-0.16
Q80WE4-3	Isoform 3 of Kinesin-like protein KIF20B OS=Mus musculus GN=Kif20b - [KI20B_MOUSE]	1.04	6	2	3	5	0.75	0.76	0.12	0.12
P81117	Nucleobindin-2 OS=Mus musculus GN=Nucb2 PE=1 SV=2 - [NUCB2_MOUSE]	29.76	1	10	10	64	0.22	0.39	0.23	0.35
Q9D819	Inorganic pyrophosphatase OS=Mus musculus GN=Ppa1 PE=1 SV=1 - [IPYR_MOUSE]	75.43	1	23	23	443	0.35	0.41	0.27	0.34
O35075	Down syndrome critical region protein 3 homolog OS=Mus musculus GN=Dscr3 PE=2 SV=1 - [DSCR3_MOUSE]	5.72	1	1	1	7	-0.54	-1.02	-0.28	-0.76
Q6NXZ1-2	Isoform 2 of Protein KRBA1 OS=Mus musculus GN=Krba1 - [KRBA1_MOUSE]	2.71	3	1	2	2	-0.11	-0.06	0.44	0.49
Q8CHS8-2	Isoform 2 of Vacuolar protein sorting-associated protein 37A OS=Mus musculus GN=Vps37a - [VP37A_MOUSE]	4.91	5	1	2	3	0.26	0.84	0.22	0.79
Q01730	Ras suppressor protein 1 OS=Mus musculus GN=Rsu1 PE=2 SV=3 - [RSU1_MOUSE]	38.99	6	11	11	201	0.33	0.44	0.28	0.36
Q80TQ2	Ubiquitin carboxyl-terminal hydrolase CYLD OS=Mus musculus GN=Cyld PE=1 SV=2 - [CYLD_MOUSE]	2.42	3	2	2	3	-0.22	-0.04	-0.08	0.10
O35295	Transcriptional activator protein Pur-beta OS=Mus musculus GN=Purb PE=1 SV=3 - [PURB_MOUSE]	52.16	3	8	12	51	0.12	0.25	0.13	0.25
P10126	Elongation factor 1-alpha 1 OS=Mus musculus GN=Eef1a1 PE=1 SV=3 - [EF1A1_MOUSE]	66.88	3	13	23	1067	0.57	0.61	0.55	0.55
Q9WUP7-2	Isoform 2 of Ubiquitin carboxyl-terminal hydrolase isozyme L5 OS=Mus musculus GN=Uchl5 - [UCHL5_MOUSE]	32.32	2	7	8	37	0.57	0.43	0.18	0.17
Q5Y5T1-2	Isoform 2 of Probable palmitoyltransferase ZDHHC20 OS=Mus musculus GN=Zdhhc20 [ZDH20_MOUSE]	9.78	2	2	2	4	0.24	-0.29	0.45	-0.04

Q3TPM3	Cullin-1 (Fragment) OS=Mus musculus GN=Cul1 PE=2 SV=1 - [Q3TPM3_MOUSE]	18.17	3	9	9	25	0.33	0.31	0.29	0.28
Q8BJS4-3	Isoform 3 of SUN domain-containing protein 2 OS=Mus musculus GN=Sun2 - [SUN2_MOUSE]	16.60	4	9	9	24	0.04	-0.13	-0.08	-0.22
Q8R3H7	Heparan sulfate 2-O-sulfotransferase 1 OS=Mus musculus GN=Hs2st1 PE=1 SV=2 - [HS2ST_MOUSE]	2.25	1	1	1	1	0.28	0.11	0.37	0.19
Q60932-2	Isoform Mt-VDAC1 of Voltage-dependent anion-selective channel protein 1 OS=Mus musculus GN=Vdac1 - [VDAC1_MOUSE]	73.50	3	14	16	425	-0.32	-0.20	-0.26	-0.14
Q5DQR4-5	Isoform 5 of Syntaxin-binding protein 5-like OS=Mus musculus GN=Stxbp5l - [STB5L_MOUSE]	5.39	7	2	2	2	0.17	0.11	-0.17	0.26
Q9QUK9	MCG15083 OS=Mus musculus GN=Try5 PE=2 SV=1 - [Q9QUK9_MOUSE]	22.76	4	1	5	337	-0.03	0.01	-0.14	0.01
Q9D2R0	Acetoacetyl-CoA synthetase OS=Mus musculus GN=Aacs PE=1 SV=1 - [AACS_MOUSE]	22.92	1	16	16	89	0.48	0.42	0.45	0.45
Q8R349	Cell division cycle protein 16 homolog OS=Mus musculus GN=Cdc16 PE=2 SV=1 - [CDC16_MOUSE]	5.65	1	3	3	10	0.32	-0.28	0.40	-0.20
Q8CG48	Structural maintenance of chromosomes protein 2 OS=Mus musculus GN=Smc2 PE=1 SV=2 [SMC2_MOUSE]	10.66	2	12	13	32	0.58	0.40	0.56	0.44
Q99P31	Hsp70-binding protein 1 OS=Mus musculus GN=Hspbp1 PE=2 SV=1 - [HPBP1_MOUSE]	11.48	1	5	5	20	0.68	0.66	0.45	0.72
G3UWX1	Replication factor C subunit 1 OS=Mus musculus GN=Rfc1 PE=2 SV=1 - [G3UWX1_MOUSE]	3.19	7	4	4	6	0.03	-0.40	0.09	-0.09
E0CYE2	Inosine-5'-monophosphate dehydrogenase OS=Mus musculus GN=Impdh1 PE=2 SV=1 - [E0CYE2_MOUSE]	15.54	4	3	6	32	0.32	0.11	0.16	-0.05
A2AFR3-2	Isoform 2 of FERM and PDZ domain-containing protein 4 OS=Mus musculus GN=Frmpd4 - [FRPD4_MOUSE]	1.48	3	1	1	1	-0.17	-0.08	-0.01	0.09
P58774	Tropomyosin beta chain OS=Mus musculus GN=Tpm2 PE=1 SV=1 [TPM2_MOUSE]	62.32	5	2	34	1768	0.19	0.10	0.03	0.07
Q80W93	Hydrocephalus-inducing protein OS=Mus musculus GN=Hydin PE=2 SV=2 - [HYDIN_MOUSE]	1.47	2	4	4	5	-0.34	-1.41	0.17	-0.90

Q9CWD3	Nucleoside diphosphate-linked moiety X motif 17 OS=Mus musculus GN=Nudt17 PE=2 SV=1 - [NUD17_MOUSE]	5.41	2	1	1	1	-0.24	-0.11	-0.22	-0.09
Q61176	Arginase-1 OS=Mus musculus GN=Arg1 PE=1 SV=1 - [ARGI1_MOUSE]	8.36	1	2	3	16	0.31	0.32	0.25	0.19
Q9R053	Sodium channel protein type 11 subunit alpha OS=Mus musculus GN=Scn11a PE=1 SV=2 - [SCNBA_MOUSE]	1.08	1	1	1	20	-0.17	-0.13	0.11	0.05
Q8CGP0	Histone H2B type 3-B OS=Mus musculus GN=Hist3h2bb PE=1 SV=3 - [H2B3B_MOUSE]	55.56	3	2	9	891	-0.08	-0.17	-0.23	-0.30
D3Z497	Uncharacterized protein OS=Mus musculus GN=Gm7075 PE=4 SV=1 - [D3Z497_MOUSE]	8.04	2	1	1	4	-0.22	-0.29	0.35	0.34
Q9CQZ5	NADH dehydrogenase [ubiquinone] 1 alpha subcomplex subunit 6 OS=Mus musculus GN=Ndufa6 PE=1 SV=1 - [NDUA6_MOUSE]	23.66	1	3	3	27	0.18	0.15	-0.03	0.03
P00860	Ornithine decarboxylase OS=Mus musculus GN=Odc1 PE=1 SV=2 - [DCOR_MOUSE]	4.34	1	1	1	2	-0.09	0.22	0.05	0.35
Q6DFW4	Nucleolar protein 58 OS=Mus musculus GN=Nop58 PE=1 SV=1 - [NOP58_MOUSE]	26.68	1	10	10	41	0.40	0.46	0.39	0.51
D3YYS9	Acid-sensing ion channel 4 OS=Mus musculus GN=Asic4 PE=2 SV=1 - [D3YYS9_MOUSE]	6.35	2	1	1	1	1.06	0.95	0.86	0.75
Q8BZR6	TOM1-like protein 1 OS=Mus musculus GN=Tom1l1 PE=2 SV=1 - [Q8BZR6_MOUSE]	21.41	5	5	6	62	0.13	0.13	0.26	0.04
Q91WE4	UPF0729 protein C18orf32 homolog OS=Mus musculus PE=2 SV=1 - [CR032_MOUSE]	13.89	1	1	1	4	0.46	0.58	0.42	0.55
Q8VH51-2	Isoform 2 of RNA-binding protein 39 OS=Mus musculus GN=Rbm39 - [RBM39_MOUSE]	28.05	6	4	13	73	0.27	0.50	0.19	0.39
P61255	60S ribosomal protein L26 OS=Mus musculus GN=Rpl26 PE=2 SV=1 [RL26_MOUSE]	42.76	4	7	8	62	0.39	0.40	0.37	0.35
Q6NZQ2	DEAD/H (Asp-Glu-Ala-Asp/His) box polypeptide 31 OS=Mus musculus GN=Ddx31 PE=2 SV=2 - [Q6NZQ2_MOUSE]	1.89	1	1	1	1	0.29	-0.21	0.30	-0.19
Q6ZQA0	Neurobeachin-like protein 2 OS=Mus musculus GN=Nbeal2 PE=2 SV=2 - [NBEL2_MOUSE]	3.90	5	6	6	10	0.26	-0.66	0.37	-0.26

Q8CD10	Calcium uptake protein 2, mitochondrial OS=Mus musculus GN=Micu2 PE=1 SV=2 - [MICU2_MOUSE]	17.82	1	6	6	13	0.21	0.41	0.13	0.25
E9Q5F9	Protein Setd2 OS=Mus musculus GN=Setd2 PE=2 SV=1 - [E9Q5F9_MOUSE]	1.85	4	2	3	3	1.34	0.31	1.26	0.22
Q9QXS6-3	Isoform E2 of Drebrin OS=Mus musculus GN=Dbn1 - [DREB_MOUSE]	24.70	4	10	10	22	-0.30	-0.04	-0.05	-0.02
Q7TMY8-3	Isoform 3 of E3 ubiquitin-protein ligase HUWE1 OS=Mus musculus GN=Huwe1 - [HUWE1_MOUSE]	9.93	7	22	23	56	0.21	0.21	0.19	0.22
P00397	Cytochrome c oxidase subunit 1 OS=Mus musculus GN=Mtco1 PE=3 SV=1 - [COX1_MOUSE]	6.23	2	1	1	35	0.11	0.37	0.05	-0.04
P61264	Syntaxin-1B OS=Mus musculus GN=Stx1b PE=1 SV=1 - [STX1B_MOUSE]	16.67	1	3	4	5	-0.14	-0.21	-0.24	-0.32
Q8BTZ7	Mannose-1-phosphate guanyltransferase beta OS=Mus musculus GN=Gmppb PE=2 SV=1 - [GMPPB_MOUSE]	28.33	1	8	8	82	0.18	0.17	0.20	0.19
Q8CCK0	Core histone macro-H2A.2 OS=Mus musculus GN=H2afy2 PE=1 SV=3 - [H2AW_MOUSE]	31.99	1	9	10	44	-0.15	-0.44	-0.21	-0.30
O35098	Dihydropyrimidinase-related protein 4 OS=Mus musculus GN=Dpysl4 PE=1 SV=1 - [DPYL4_MOUSE]	4.55	3	1	2	6	0.32	0.26	0.24	0.18
Q9Z1B5	Mitotic spindle assembly checkpoint protein MAD2A OS=Mus musculus GN=Mad2l1 PE=2 SV=2 - [MD2L1_MOUSE]	13.17	1	3	3	4	0.65	0.48	0.86	0.69
B2RUR8	OTU domain-containing protein 7B OS=Mus musculus GN=Otud7b PE=1 SV=1 - [OTU7B_MOUSE]	4.05	3	2	3	4	0.20	-0.02	0.18	-0.04
Q8CG03	cGMP-specific 3',5'-cyclic phosphodiesterase OS=Mus musculus GN=Pde5a PE=1 SV=2 [PDE5A_MOUSE]	25.32	1	17	17	62	0.29	0.20	0.40	0.23
Q3UV66	Protein Slfn4 OS=Mus musculus GN=Slfn4 PE=2 SV=1 - [Q3UV66_MOUSE]	1.99	1	1	2	14	0.28	0.41	0.69	0.79
Q9CXE7	Transmembrane emp24 domain-containing protein 5 OS=Mus musculus GN=Tmed5 PE=2 SV=1 - [TMED5_MOUSE]	17.90	6	5	5	25	0.53	0.57	0.46	0.42
A2AWS5	Histone deacetylase 5 OS=Mus musculus GN=Hdac5 PE=2 SV=1 - [A2AWS5_MOUSE]	1.55	5	1	2	2	-0.12	-0.60	-0.02	-0.50

F8VPU8	Protein AU041133 OS=Mus musculus GN=AU041133 PE=4 SV=1 - [F8VPU8_MOUSE]	5.03	1	1	1	1	-0.19	-0.25	0.00	-0.06
Q9CZX9	ER membrane protein complex subunit 4 OS=Mus musculus GN=Emc4 PE=2 SV=1 [EMC4_MOUSE]	22.40	1	3	3	13	-0.05	0.01	-0.02	-0.03
Q8K3X4	Interferon regulatory factor 2-binding protein-like OS=Mus musculus GN=Irf2bpl PE=1 SV=1 - [I2BPL_MOUSE]	10.84	1	7	7	15	0.05	-0.14	0.11	0.02
P61924	Coatomer subunit zeta-1 OS=Mus musculus GN=Copz1 PE=2 SV=1 - [COPZ1_MOUSE]	19.21	1	3	3	75	0.04	0.04	0.04	0.09
Q8CAQ8	Mitochondrial inner membrane protein OS=Mus musculus GN=Immt PE=1 SV=1 - [IMMT_MOUSE]	57.86	4	3	41	239	-0.01	-0.01	-0.09	-0.04
P62334	26S protease regulatory subunit 10B OS=Mus musculus GN=Psmc6 PE=1 SV=1 - [PRS10_MOUSE]	50.64	1	17	17	156	0.26	0.27	0.28	0.27
E9PV48	Protein I830012O16Rik OS=Mus musculus GN=I830012O16Rik PE=4 SV=1 - [E9PV48_MOUSE]	6.20	2	2	2	5	0.32	0.00	0.21	0.07
Q9DB20	ATP synthase subunit O, mitochondrial OS=Mus musculus GN=Atp5o PE=1 SV=1 - [ATPO_MOUSE]	48.83	4	10	10	191	-0.02	0.09	-0.03	0.05
Q9WTN0	Geranylgeranyl pyrophosphate synthase OS=Mus musculus GN=Ggps1 PE=2 SV=1 - [GGPPS_MOUSE]	8.67	1	3	3	7	0.61	0.32	0.11	0.11
Q8BZR9	Uncharacterized protein C17orf85 homolog OS=Mus musculus PE=1 SV=1 - [CQ085_MOUSE]	6.02	3	3	3	3	0.51	0.14	0.66	0.29
Q640N1-2	Isoform 2 of Adipocyte enhancer-binding protein 1 OS=Mus musculus GN=Aebp1 - [AEBP1_MOUSE]	12.97	2	7	7	12	0.08	0.11	0.07	0.27
O55143	Sarcoplasmic/endoplasmic reticulum calcium ATPase 2 OS=Mus musculus GN=Atp2a2 PE=1 SV=2 - [AT2A2_MOUSE]	34.67	3	29	36	426	0.06	0.00	-0.03	0.04
Q8R1Q8	Cytoplasmic dynein 1 light intermediate chain 1 OS=Mus musculus GN=Dync1li1 PE=1 SV=1 - [DC1L1_MOUSE]	37.86	1	12	13	66	0.42	0.54	0.42	0.33
P49615	Cyclin-dependent kinase 5 OS=Mus musculus GN=Cdk5 PE=1 SV=1 - [CDK5_MOUSE]	18.15	31	3	4	35	0.31	0.50	0.22	0.37

K3W4Q8	Basigin OS=Mus musculus GN=Bsg PE=4 SV=1 - [K3W4Q8_MOUSE]	33.49	3	2	7	99	0.25	-0.68	-0.60	-1.53
E9Q8N5	CLIP-associating protein 2 OS=Mus musculus GN=Clasp2 PE=2 SV=1 - [E9Q8N5_MOUSE]	6.22	6	6	7	13	0.02	-0.13	0.06	-0.13
Q91VA7	Isocitrate dehydrogenase 3 (NAD+) beta OS=Mus musculus GN=Idh3b PE=2 SV=1 - [Q91VA7_MOUSE]	41.67	1	16	16	141	0.05	0.09	0.00	0.02
J3QMV3	Uncharacterized protein OS=Mus musculus GN=Gm21778 PE=4 SV=1 - [J3QMV3_MOUSE]	10.26	3	1	1	1	-0.93	-0.78	-1.32	-1.18
Q68FH0-2	Isoform 2 of Plakophilin-4 OS=Mus musculus GN=Pkp4 - [PKP4_MOUSE]	5.49	6	4	4	6	0.06	-0.25	-0.13	-0.22
Q9D0M3-2	Isoform 2 of Cytochrome c1, heme protein, mitochondrial OS=Mus musculus GN=Cyc1 - [CY1_MOUSE]	44.36	2	8	8	320	0.11	0.13	0.14	0.07
Q32KG4	Retrotransposon gag domain-containing protein 1 OS=Mus musculus GN=Rgag1 PE=2 SV=1 - [RGAG1_MOUSE]	2.84	3	3	3	4	0.02	-0.42	0.15	-0.29
Q9D7A8	Armadillo repeat-containing protein 1 OS=Mus musculus GN=Armc1 PE=2 SV=1 - [ARMC1_MOUSE]	8.87	1	3	3	8	0.17	0.31	0.04	0.05
D6RIJ8	Protein 2700029M09Rik OS=Mus musculus GN=2700029M09Rik PE=4 SV=1 - [D6RIJ8_MOUSE]	13.48	2	1	1	2	0.46	0.78	0.43	0.74
Q8CGA0	Protein phosphatase 1F OS=Mus musculus GN=Ppm1f PE=2 SV=1 - [PPM1F_MOUSE]	4.42	1	1	1	4	0.42	0.06	0.40	0.04
Q9Z1W9	STE20/SPS1-related proline-alanine-rich protein kinase OS=Mus musculus GN=Stk39 PE=1 SV=1 - [STK39_MOUSE]	8.99	1	3	3	14	0.07	0.38	0.00	0.34
O55013	Trafficking protein particle complex subunit 3 OS=Mus musculus GN=Trappc3 PE=1 SV=1 - [TPPC3_MOUSE]	12.78	1	2	2	16	0.05	0.13	0.00	0.12
Q8C4J7	Transducin beta-like protein 3 OS=Mus musculus GN=Tbl3 PE=2 SV=1 - [TBL3_MOUSE]	4.74	2	3	3	5	0.36	0.20	0.29	0.26
Q9Z1T1	AP-3 complex subunit beta-1 OS=Mus musculus GN=Ap3b1 PE=1 SV=2 - [AP3B1_MOUSE]	14.12	2	14	14	53	0.21	0.12	0.26	0.11
E9PWF0	Thrombospondin-3 OS=Mus musculus GN=Thbs3 PE=2 SV=1 - [E9PWF0_MOUSE]	4.13	3	1	2	4	-0.01	0.05	0.05	0.11

Q8R2T8-2	Isoform 2 of General transcription factor 3C polypeptide 5 OS=Mus musculus GN=Gtf3c5 - [TF3C5_MOUSE]	4.86	3	2	2	5	0.03	-0.07	-0.50	-0.18
P46660	Alpha-internexin OS=Mus musculus GN=Ina PE=1 SV=2 - [AINX_MOUSE]	19.44	1	7	8	143	-0.53	-0.60	-0.63	-0.71
Q05117	Tartrate-resistant acid phosphatase type 5 OS=Mus musculus GN=Acp5 PE=2 SV=2 - [PPA5_MOUSE]	3.67	1	1	1	18	-0.23	-0.21	-0.07	-0.23
P27641	X-ray repair cross-complementing protein 5 OS=Mus musculus GN=Xrcc5 PE=2 SV=4 [XRCC5_MOUSE]	9.56	1	5	6	20	0.35	0.36	0.24	0.22
D3Z5Q8	Synaptoporin (Fragment) OS=Mus musculus GN=Synpr PE=2 SV=1 - [D3Z5Q8_MOUSE]	15.20	1	1	1	14	-0.02	-0.39	0.15	-0.38
P48722	Heat shock 70 kDa protein 4L OS=Mus musculus GN=Hspa4l PE=1 SV=2 - [HS74L_MOUSE]	33.29	5	22	24	114	0.10	0.07	0.17	0.13
Q9QYF9	Protein NDRG3 OS=Mus musculus GN=Ndrg3 PE=1 SV=1 - [NDRG3_MOUSE]	20.00	6	4	5	32	0.19	0.24	-0.08	-0.03
Q61133	Glutathione S-transferase theta-2 OS=Mus musculus GN=Gstt2 PE=2 SV=4 - [GSTT2_MOUSE]	32.38	1	6	7	42	0.90	0.80	0.81	0.73
Q64237	Dopamine beta-hydroxylase OS=Mus musculus GN=Dbh PE=2 SV=1 - [DOPO_MOUSE]	3.70	2	2	2	11	-0.52	-0.30	-0.15	-0.02
O88456	Calpain small subunit 1 OS=Mus musculus GN=Capns1 PE=2 SV=1 - [CPNS1_MOUSE]	61.34	3	9	9	82	0.29	0.37	0.26	0.37
Q8BH44	Coronin-2B OS=Mus musculus GN=Coro2b PE=2 SV=2 - [COR2B_MOUSE]	5.21	2	1	2	11	-0.43	-0.32	-0.35	-0.26
Q8VDU4	Proto-oncogene vav OS=Mus musculus GN=Vav1 PE=2 SV=1 - [Q8VDU4_MOUSE]	0.99	3	1	1	2	0.28	0.16	0.10	-0.02
Q8VE99	Coiled-coil domain-containing protein 115 OS=Mus musculus GN=Ccdc115 PE=2 SV=1 - [CC115_MOUSE]	13.89	1	2	2	4	0.12	-0.16	0.38	0.10
Q9CYG7	Mitochondrial import receptor subunit TOM34 OS=Mus musculus GN=Tomm34 PE=2 SV=1 - [TOM34_MOUSE]	38.51	2	10	10	29	0.22	0.19	0.34	0.25
Q8CIG9	F-box/LRR-repeat protein 8 OS=Mus musculus GN=Fbxl8 PE=1 SV=1 - [FBXL8_MOUSE]	6.15	1	2	2	2	0.31	0.16	0.16	0.01
Q80TR8-4	Isoform 4 of Protein VPRBP OS=Mus musculus GN=Vprbp - [VPRBP_MOUSE]	7.89	4	7	8	20	0.10	0.40	-0.18	0.20

O35683	NADH dehydrogenase [ubiquinone] 1 alpha subcomplex subunit 1 OS=Mus musculus GN=Ndufa1 PE=2 SV=1 - [NDUA1_MOUSE]	14.29	1	1	1	13	-0.40	-0.50	-0.18	-0.34
Q7TNL7	Dual specificity protein phosphatase OS=Mus musculus GN=Dusp9 PE=2 SV=1 - [Q7TNL7_MOUSE]	6.64	1	2	2	2	0.28	-0.26	0.70	0.16
Q9D517	1-acyl-sn-glycerol-3-phosphate acyltransferase gamma OS=Mus musculus GN=Agpat3 PE=1 SV=2 - [PLCC_MOUSE]	2.66	1	1	1	2	0.11	0.16	-0.10	-0.06
E9Q0I8	Homer protein homolog 1 OS=Mus musculus GN=Homer1 PE=2 SV=1 - [E9Q0I8_MOUSE]	3.89	6	1	1	1	-0.19	-0.08	0.02	0.13
O55029	Coatomer subunit beta' OS=Mus musculus GN=Copb2 PE=2 SV=2 - [COPB2_MOUSE]	48.95	1	36	36	324	0.20	0.23	0.18	0.23
P14131	40S ribosomal protein S16 OS=Mus musculus GN=Rps16 PE=2 SV=4 - [RS16_MOUSE]	56.85	1	11	11	114	0.32	0.28	0.31	0.30
Q8CDV7	Ectonucleoside triphosphate diphosphohydrolase 1 OS=Mus musculus GN=Entpd1 PE=2 SV=1 - [Q8CDV7_MOUSE]	25.23	5	12	12	52	-0.02	0.11	-0.22	0.04
Q9R1Z8	Vinexin OS=Mus musculus GN=Sorbs3 PE=1 SV=1 - [VINEX_MOUSE]	12.96	1	8	8	34	-0.18	-0.22	-0.27	-0.33
O35071	Kinesin-like protein KIF1C OS=Mus musculus GN=Kif1c PE=1 SV=2 - [KIF1C_MOUSE]	11.73	5	4	8	24	0.54	0.16	0.41	0.18
E9Q4N7	Protein Arid1b OS=Mus musculus GN=Arid1b PE=2 SV=1 - [E9Q4N7_MOUSE]	7.49	3	7	7	16	-0.03	-0.15	0.07	0.14
Q8R404	Protein QIL1 OS=Mus musculus GN=Qil1 PE=2 SV=1 - [QIL1_MOUSE]	41.18	1	3	3	20	0.27	0.39	0.11	0.23
Q9JLV5	Cullin-3 OS=Mus musculus GN=Cul3 PE=1 SV=1 - [CUL3_MOUSE]	29.69	11	17	18	48	0.14	0.20	0.10	0.18
Q9CZY3	Ubiquitin-conjugating enzyme E2 variant 1 OS=Mus musculus GN=Ube2v1 PE=1 SV=1 - [UB2V1_MOUSE]	57.14	6	4	8	112	0.17	0.26	0.20	0.21
Q99JW5	Epithelial cell adhesion molecule OS=Mus musculus GN=Epcam PE=2 SV=1 - [EPCAM_MOUSE]	37.14	1	11	11	506	0.37	0.45	0.34	0.41
Q922U2	Keratin, type II cytoskeletal 5 OS=Mus musculus GN=Krt5 PE=1 SV=1 - [K2C5_MOUSE]	8.97	1	1	8	198	1.55	1.75	0.10	0.30

Q8CBD1	Nuclear receptor-interacting protein 1 OS=Mus musculus GN=Nrip1 PE=1 SV=1 [NRIP1_MOUSE]	3.19	1	1	1	1	0.62	-0.09	0.53	-0.18
P33174	Chromosome-associated kinesin KIF4 OS=Mus musculus GN=Kif4 PE=1 SV=3 - [KIF4_MOUSE]	3.74	1	3	4	5	-0.39	-3.86	-0.10	-3.57
Q8CJ26	Death domain-containing membrane protein NRADD OS=Mus musculus GN=Nradd PE=1 SV=1 [NRADD_MOUSE]	8.77	1	2	2	5	0.07	0.06	0.12	0.11
Q8VCH8	UBX domain-containing protein 4 OS=Mus musculus GN=Ubxn4 PE=1 SV=1 - [UBXN4_MOUSE]	6.72	1	3	3	25	0.20	0.45	0.38	0.54
P11688	Integrin alpha-5 OS=Mus musculus GN=Itga5 PE=1 SV=3 - [ITA5_MOUSE]	19.56	1	18	18	122	0.02	0.08	-0.03	0.08
Q64471	Glutathione S-transferase theta-1 OS=Mus musculus GN=Gstt1 PE=1 SV=4 - [GSTT1_MOUSE]	29.17	3	8	8	24	-0.15	0.30	-0.09	0.15
B2RW38	Coiled-coil domain containing 147 OS=Mus musculus GN=Ccdc147 PE=2 SV=1 - [B2RW38_MOUSE]	4.70	1	3	3	5	-0.05	-0.19	0.46	0.23
Q3TWV4	AP-2 complex subunit mu OS=Mus musculus GN=Ap2m1 PE=2 SV=1 - [Q3TWV4_MOUSE]	34.41	2	16	16	85	-0.05	-0.08	-0.07	-0.05
Q9EQ08	Heparan N-sulfatase OS=Mus musculus GN=Sgsh PE=2 SV=1 - [Q9EQ08_MOUSE]	8.96	2	3	3	5	-0.15	-0.62	-0.03	-0.50
Q9CPN9	Protein 2210010C04Rik OS=Mus musculus GN=2210010C04Rik PE=2 SV=1 - [Q9CPN9_MOUSE]	25.10	1	2	3	9	-0.48	-0.07	-0.18	0.23
H3BIX4	Neuroplastin (Fragment) OS=Mus musculus GN=Nptn PE=4 SV=1 - [H3BIX4_MOUSE]	35.75	6	7	7	53	-0.11	-0.24	-0.18	-0.21
Q99MR8	Methylcrotonoyl-CoA carboxylase subunit alpha, mitochondrial OS=Mus musculus GN=Mccc1 PE=2 SV=2 - [MCCA_MOUSE]	40.45	3	21	23	89	-0.13	-0.27	-0.10	-0.17
Q3TDT0	Tripartite motif-containing protein 3 OS=Mus musculus GN=Trim3 PE=2 SV=1 [Q3TDT0_MOUSE]	5.44	2	3	3	5	0.06	0.01	0.08	0.07
Q62277	Synaptophysin OS=Mus musculus GN=Syp PE=1 SV=2 - [SYPH_MOUSE]	3.18	1	1	1	4	-0.16	-0.14	-0.29	-0.27
B1AVK5	Protein Col4a6 OS=Mus musculus GN=Col4a6 PE=2 SV=1 - [B1AVK5_MOUSE]	3.73	1	2	3	4	-0.14	-0.78	-0.30	-0.94

E9PZR3	Poly(A) polymerase alpha OS=Mus musculus GN=Papola PE=2 SV=1 - [E9PZR3_MOUSE]	8.36	12	3	4	12	0.17	0.30	0.06	0.24
P35295	Ras-related protein Rab-20 OS=Mus musculus GN=Rab20 PE=2 SV=2 - [RAB20_MOUSE]	5.15	1	1	1	1	-0.01	-0.63	-0.04	-0.66
Q9D614	Syntaxin 17, isoform CRA_b OS=Mus musculus GN=Stx17 PE=2 SV=1 - [Q9D614_MOUSE]	13.41	3	3	3	4	-0.15	0.02	-0.18	-0.02
P24638	Lysosomal acid phosphatase OS=Mus musculus GN=Acp2 PE=2 SV=2 - [PPAL_MOUSE]	5.20	2	2	2	5	-0.04	-0.18	0.00	-0.22
Q3UGR5	Haloacid dehalogenase-like hydrolase domain-containing protein 2 OS=Mus musculus GN=Hdhd2 PE=1 SV=2 - [HDHD2_MOUSE]	31.27	4	5	6	20	-0.21	-0.17	-0.14	-0.02
Q9DBX6	Cytochrome P450 2S1 OS=Mus musculus GN=Cyp2s1 PE=2 SV=1 - [CP2S1_MOUSE]	12.18	2	6	6	21	-0.29	-0.17	-0.38	-0.29
E9Q4X2	Protein Uggt2 OS=Mus musculus GN=Uggt2 PE=2 SV=1 - [E9Q4X2_MOUSE]	0.93	3	1	2	6	-0.13	0.50	-0.15	0.47
E9PWK1	Epoxide hydrolase 1 OS=Mus musculus GN=Ephx1 PE=2 SV=1 - [E9PWK1_MOUSE]	37.64	4	16	16	61	-0.08	-0.03	-0.11	-0.10
Q9CX00	IST1 homolog OS=Mus musculus GN=Ist1 PE=2 SV=1 - [IST1_MOUSE]	32.32	2	10	10	42	0.54	0.39	0.43	0.38
Q3TZ89-2	Isoform 2 of Protein transport protein Sec31B OS=Mus musculus GN=Sec31b - [SC31B_MOUSE]	1.50	2	1	2	2	0.29	0.60	0.42	0.74
Q8K4K6-2	Isoform 2 of Pantothenate kinase 1 OS=Mus musculus GN=Pank1 - [PANK1_MOUSE]	5.36	3	1	2	2	0.35	0.85	0.11	0.60
Q8BHX6	Nucleolar complex protein 2 homolog OS=Mus musculus GN=Noc2l PE=2 SV=1 [Q8BHX6_MOUSE]	6.24	3	3	3	3	0.12	0.56	0.23	0.68
E9PWI6	GRIP and coiled-coil domain-containing protein 2 OS=Mus musculus GN=Gcc2 PE=2 SV=1 - [E9PWI6_MOUSE]	11.92	6	17	18	30	0.12	0.00	0.02	0.06
Q8BZA9	Fructose-2,6-bisphosphatase TIGAR OS=Mus musculus GN=Tigar PE=2 SV=1 - [TIGAR_MOUSE]	6.32	1	1	1	1	0.04	0.24	0.12	0.31
Q921J2	GTP-binding protein Rheb OS=Mus musculus GN=Rheb PE=1 SV=1 - [RHEB_MOUSE]	20.11	1	3	4	14	0.32	0.53	0.20	0.33
P13808-2	Isoform B1 of Anion exchange protein 2 OS=Mus musculus GN=Slc4a2 - [B3A2_MOUSE]	8.83	9	7	8	21	-0.63	-0.60	-0.53	-0.49

G3X8U7	Serine/threonine-protein phosphatase OS=Mus musculus GN=Ppp3cb PE=3 SV=1 - [G3X8U7_MOUSE]	19.22	6	4	9	36	-0.14	0.14	-0.29	0.30
Q3UR70	Transforming growth factor-beta receptor-associated protein 1 OS=Mus musculus GN=Tgfbrap1 PE=2 SV=1 - [TGFA1_MOUSE]	2.44	1	1	1	3	0.75	-0.26	0.85	-0.16
P62192	26S protease regulatory subunit 4 OS=Mus musculus GN=Psmc1 PE=1 SV=1 - [PRS4_MOUSE]	54.09	1	18	19	125	0.41	0.43	0.44	0.46
Q8R0X2-2	Isoform 2 of CDK2-associated and cullin domain-containing protein 1 OS=Mus musculus GN=Cacul1 - [CACL1_MOUSE]	4.28	3	1	1	3	-0.01	0.18	0.25	0.43
G5E825	DNA segment, Chr 10, ERATO Doi 610, expressed, isoform CRA_b OS=Mus musculus GN=Arhgef25 PE=4 SV=1 - [G5E825_MOUSE]	4.53	16	1	2	7	0.30	0.31	0.35	0.35
Q6P5B0	RRP12-like protein OS=Mus musculus GN=Rrp12 PE=1 SV=1 [RRP12_MOUSE]	5.56	1	4	4	8	0.31	-0.26	0.29	0.35
Q9CQ52	Chymotrypsin-like elastase family member 3B OS=Mus musculus GN=Cela3b PE=2 SV=1 - [CEL3B_MOUSE]	20.45	1	3	3	15	0.89	0.27	1.24	0.45
Q9QYE6	Golgin subfamily A member 5 OS=Mus musculus GN=Golga5 PE=1 SV=2 - [GOGA5_MOUSE]	32.92	1	18	18	65	-0.05	0.06	-0.02	0.12
P49586	Choline-phosphate cytidylyltransferase A OS=Mus musculus GN=Pcyt1a PE=1 SV=1 - [PCY1A_MOUSE]	31.34	6	10	10	34	0.29	0.24	0.27	0.09
Q8R5C5	Beta-centractin OS=Mus musculus GN=Actr1b PE=1 SV=1 - [ACTY_MOUSE]	40.43	4	4	12	114	-0.23	0.13	-0.01	0.10
G3UXX3	Sepiapterin reductase OS=Mus musculus GN=Spr PE=2 SV=1 - [G3UXX3_MOUSE]	57.53	5	10	10	176	0.14	0.18	0.08	0.17
P70662-2	Isoform 2 of LIM domain-binding protein 1 OS=Mus musculus GN=Ldb1 - [LDB1_MOUSE]	3.13	4	1	1	2	0.12	0.12	0.13	0.13
J3QJV7	Rab GTPase-binding effector protein 1 OS=Mus musculus GN=Rabep1 PE=4 SV=1 - [J3QJV7_MOUSE]	13.11	8	7	7	16	-0.03	-0.07	0.10	0.03
P70227	Inositol 1,4,5-trisphosphate receptor type 3 OS=Mus musculus GN=Itpr3 PE=1 SV=3 - [ITPR3_MOUSE]	7.60	1	11	14	38	-0.02	-0.09	0.05	-0.20

E9PV60	Protein Wdfy4 OS=Mus musculus GN=Wdfy4 PE=2 SV=1 - [E9PV60_MOUSE]	1.22	2	2	3	5	0.21	0.23	0.28	0.28
Q8C0E3	Tripartite motif-containing protein 47 OS=Mus musculus GN=Trim47 PE=2 SV=2 - [TRI47_MOUSE]	13.73	2	7	7	25	0.56	0.28	0.51	0.21
G5E843	Roundabout homolog 1 OS=Mus musculus GN=Robo1 PE=4 SV=1 - [G5E843_MOUSE]	3.72	2	3	4	7	-0.33	-0.51	-0.57	-0.26
P58774-2	Isoform 2 of Tropomyosin beta chain OS=Mus musculus GN=Tpm2 - [TPM2_MOUSE]	72.89	5	6	38	2858	0.13	0.22	0.09	0.16
D6RFQ6	Mitochondrial fission regulator 1 OS=Mus musculus GN=Mtfr1 PE=2 SV=1 - [D6RFQ6_MOUSE]	15.38	5	1	1	1	0.83	0.87	0.66	0.70
A2A6P4	Protein Fam104a OS=Mus musculus GN=Fam104a PE=2 SV=1 - [A2A6P4_MOUSE]	13.51	1	2	2	3	-0.15	0.16	-0.15	0.16
P46460	Vesicle-fusing ATPase OS=Mus musculus GN=Nsf PE=1 SV=2 - [NSF_MOUSE]	32.93	3	22	23	96	0.34	0.35	0.18	0.31
Q9Z2I0	LETM1 and EF-hand domain-containing protein 1, mitochondrial OS=Mus musculus GN=Letm1 PE=2 SV=1 - [LETM1_MOUSE]	32.93	1	21	21	167	0.20	0.34	0.16	0.30
Q9QXX4	Calcium-binding mitochondrial carrier protein Aralar2 OS=Mus musculus GN=Slc25a13 PE=1 SV=1 - [CMC2_MOUSE]	23.22	2	9	14	70	-0.06	-0.08	0.01	-0.04
P35564	Calnexin OS=Mus musculus GN=Canx PE=1 SV=1 - [CALX_MOUSE]	28.43	1	20	20	319	0.37	0.38	0.27	0.32
Q8CHW4	Translation initiation factor eIF-2B subunit epsilon OS=Mus musculus GN=Eif2b5 PE=1 SV=1 - [EI2BE_MOUSE]	6.28	1	2	2	9	-0.47	-0.59	-0.57	-0.17
P61027	Ras-related protein Rab-10 OS=Mus musculus GN=Rab10 PE=1 SV=1 - [RAB10_MOUSE]	42.00	13	5	9	99	0.20	0.15	0.12	0.20
Q9D2G9	HHIP-like protein 2 OS=Mus musculus GN=Hhipl2 PE=2 SV=2 - [HIPL2_MOUSE]	4.60	1	1	1	1	0.98	0.43	1.23	0.68
Q9Z1N9-2	Isoform 2 of Protein unc 13 homolog B OS=Mu musculus GN=Unc13b [UN13B_MOUSE]	0.82	4	1	1	1	0.17	0.60	-0.33	0.10
R4GML6	Centriolin (Fragment) OS=Mus musculus GN=Cep110 PE=4 SV=1 - [R4GML6_MOUSE]	3.02	8	4	7	8	0.37	0.58	0.85	0.81

P57746	V-type proton ATPase subunit D OS=Mus musculus GN=Atp6v1d PE=1 SV=1 - [VATD_MOUSE]	26.32	1	8	8	30	0.33	0.17	0.23	0.17
P30275	Creatine kinase U-type, mitochondrial OS=Mus musculus GN=Ckmt1 PE=1 SV=1 - [KCRU_MOUSE]	56.70	6	17	19	1191	0.00	0.05	-0.06	0.03
O88384	Vesicle transport through interaction with t-SNAREs homolog 1B OS=Mus musculus GN=Vti1b PE=1 SV=1 - [VTI1B_MOUSE]	14.22	5	3	3	14	-0.14	-0.03	-0.06	0.15
Q5SRX1-3	Isoform 3 of TOM1-like protein 2 OS=Mus musculus GN=Tom1l2 - [TM1L2_MOUSE]	21.36	9	7	7	34	-0.31	-0.59	-0.18	-0.36
Q9DAI2	Rab-like protein 5 OS=Mus musculus GN=Rabl5 PE=2 SV=1 [RABL5_MOUSE]	5.95	1	1	1	4	-0.18	-0.06	-0.25	-0.14
E9Q3N9	EGF-containing fibulin-like extracellular matrix protein 2 OS=Mus musculus GN=Efemp2 PE=2 SV=1 - [E9Q3N9_MOUSE]	10.45	8	4	4	6	-0.06	-0.60	0.15	-0.39
Q9CXI0	2-methoxy-6-polyprenyl 1,4-benzoquinol methylase, mitochondrial OS=Mus musculus GN=Coq5 PE=2 SV=2 - [COQ5_MOUSE]	24.77	2	5	5	29	0.40	0.50	0.36	0.44
J3QP10	Uncharacterized protein OS=Mus musculus GN=Gm5117 PE=4 SV=1 - [J3QP10_MOUSE]	5.36	2	3	3	3	0.01	0.03	0.76	0.30
G5E8V9	MCG18094 (Fragment) OS=Mus musculus GN=Arfip1 PE=4 SV=1 - [G5E8V9_MOUSE]	42.36	4	11	11	72	0.16	-0.01	0.13	0.07
Q91XZ4	Protein Pcdhb6 OS=Mus musculus GN=Pcdhb6 PE=2 SV=1 - [Q91XZ4_MOUSE]	5.31	10	1	2	3	0.65	0.19	0.60	0.14
Q7TSH2	Phosphorylase b kinase regulatory subunit beta OS=Mus musculus GN=Phkb PE=1 SV=1 - [KPBB_MOUSE]	3.13	1	2	2	3	-0.57	0.00	-0.41	0.17
Q9CQ75	NADH dehydrogenase [ubiquinone] 1 alpha subcomplex subunit 2 OS=Mus musculus GN=Ndufa2 PE=1 SV=3 - [NDUA2_MOUSE]	44.44	1	4	5	136	0.03	0.17	-0.02	0.05
B1AYM9	Protein CK137956 OS=Mus musculus GN=CK137956 PE=2 SV=1 - [B1AYM9_MOUSE]	2.68	1	1	1	1	0.34	0.20	0.40	0.25

E9Q421	GPI-anchor transamidase OS=Mus musculus GN=Pigk PE=2 SV=1 - [E9Q421_MOUSE]	4.70	4	2	2	4	0.09	0.04	0.12	0.07
E9Q5N7	Protein Nrxn2 OS=Mus musculus GN=Nrxn2 PE=4 SV=2 - [E9Q5N7_MOUSE]	0.60	4	1	1	1	-0.54	-0.45	-0.24	-0.15
P61205	ADP-ribosylation factor 3 OS=Mus musculus GN=Arf3 PE=2 SV=2 - [ARF3_MOUSE]	61.88	7	4	10	262	0.05	0.05	0.04	0.07
Q8JZR4	Excitatory amino acid transporter 5 OS=Mus musculus GN=Slc1a7 PE=2 SV=1 - [EAA5_MOUSE]	3.58	1	1	1	1	0.13	0.83	0.07	0.77
P01638	Ig kappa chain V-V region L6 (Fragment) OS=Mus musculus PE=4 SV=1 - [KV5A6_MOUSE]	13.04	2	1	1	10	-0.51	-0.79	-0.54	-0.40
Q6PDG8-2	Isoform 2 of Vacuolar fusion protein MON1 homolog A OS=Mus musculus GN=Mon1a - [MON1A_MOUSE]	8.24	2	2	2	5	0.17	0.04	-0.06	-0.07
Q8BYF6	Sodium-coupled monocarboxylate transporter 1 OS=Mus musculus GN=Slc5a8 PE=1 SV=1 - [SC5A8_MOUSE]	1.80	1	1	1	2	1.19	1.09	1.13	1.02
F7C9T6	Serine protease hepsin (Fragment) OS=Mus musculus GN=Hpn PE=4 SV=1 - [F7C9T6_MOUSE]	21.88	10	1	1	2	-0.39	0.12	-0.73	-0.22
Q99JT9	1,2-dihydroxy-3-keto-5-methylthiopentene dioxygenase OS=Mus musculus GN=Adi1 PE=1 SV=1 - [MTND_MOUSE]	14.53	2	2	2	6	-1.22	-1.23	-0.42	-0.43
Q8CIR4	Transient receptor potential cation channel subfamily M member 6 OS=Mus musculus GN=Trpm6 PE=2 SV=1 - [TRPM6_MOUSE]	2.22	3	1	3	4	-0.21	-0.05	-0.23	-0.07
P31324	cAMP-dependent protein kinase type II-beta regulatory subunit OS=Mus musculus GN=Prkar2b PE=1 SV=3 - [KAP3_MOUSE]	11.06	2	1	4	50	0.05	-0.03	0.17	0.09
Q9WUX5-2	Isoform 2 of Protein MRVI1 OS=Mus musculus GN=Mrvi1 - [MRVI1_MOUSE]	18.57	4	9	10	45	0.11	0.45	-0.02	0.27
Q9CZJ1	Probable U3 small nucleolar RNA-associated protein 11 OS=Mus musculus GN=Utp11l PE=2 SV=1 - [UTP11_MOUSE]	3.56	1	1	1	1	0.32	-0.06	0.40	0.03
P62281	40S ribosomal protein S11 OS=Mus musculus GN=Rps11 PE=2 SV=3 - [RS11_MOUSE]	42.41	1	8	8	127	0.37	0.44	0.38	0.46
Q91Z92	Beta-1,3-galactosyltransferase 6 OS=Mus musculus GN=B3galt6 PE=2 SV=1 - [B3GT6_MOUSE]	4.00	1	1	1	1	0.52	0.49	0.51	0.47

Q7TPS5-3	Isoform 3 of C2 domain containing protein 5 OS=Mus musculus GN=C2cd5 - [C2CD5_MOUSE]	1.22	3	1	1	4	-0.62	-0.48	-0.34	-0.20
Q8BTT6	Digestive organ expansion factor homolog OS=Mus musculus GN=Diexf PE=2 SV=2 - [DIEXF_MOUSE]	6.22	1	3	4	7	0.22	0.27	0.11	-0.08
O70589	Peripheral plasma membrane protein CASK OS=Mus musculus GN=Cask PE=1 SV=2 - [CSKP_MOUSE]	20.63	8	7	14	24	0.35	0.39	0.42	0.59
Q8BNA6-3	Isoform 3 of Protocadherin Fat 3 OS=Mus musculus GN=Fat3 - [FAT3_MOUSE]	2.54	1	1	1	1	0.30	0.14	0.56	0.39
Q61644	Protein kinase C and casein kinase substrate in neurons protein 1 OS=Mus musculus GN=Pacsin1 PE=1 SV=1 - [PACN1_MOUSE]	4.99	1	2	3	15	0.85	1.29	0.70	1.13
P07310	Creatine kinase M-type OS=Mus musculus GN=Ckm PE=1 SV=1 - [KCRM_MOUSE]	60.37	1	19	22	491	0.29	0.35	0.29	0.33
Q64435	UDP-glucuronosyltransferase 1-6 OS=Mus musculus GN=Ugt1a6 PE=1 SV=1 - [UD16_MOUSE]	19.40	14	3	8	67	-0.42	-0.55	-0.08	-0.18
A2AWI9	Endophilin-B2 OS=Mus musculus GN=Sh3glb2 PE=4 SV=1 - [A2AWI9_MOUSE]	17.91	5	5	7	25	0.36	0.09	0.33	0.54
Q9D1K2	V-type proton ATPase subunit F OS=Mus musculus GN=Atp6v1f PE=1 SV=2 - [VATF_MOUSE]	47.90	2	5	5	23	-0.13	0.09	-0.17	-0.08
P48771	Cytochrome c oxidase subunit 7A2, mitochondrial OS=Mus musculus GN=Cox7a2 PE=1 SV=2 - [CX7A2_MOUSE]	27.71	1	2	2	94	0.20	0.19	0.09	0.12
D3Z6I9	Coiled-coil domain-containing protein 114 OS=Mus musculus GN=Ccdc114 PE=2 SV=1 - [D3Z6I9_MOUSE]	17.56	4	1	2	2	0.20	-0.73	0.58	-0.35
Q80WW9	DDRGK domain-containing protein 1 OS=Mus musculus GN=Ddrgk1 PE=1 SV=2 - [DDRGK_MOUSE]	5.40	1	1	1	4	0.39	0.51	0.14	0.26
Q9WU03-2	Isoform 2 of Kunitz-type protease inhibitor 2 OS=Mus musculus GN=Spint2 - [SPIT2_MOUSE]	25.13	2	4	4	22	0.46	0.27	0.35	0.15
Q91ZW9	CD209 antigen-like protein C OS=Mus musculus GN=Cd209c PE=2 SV=1 - [C209C_MOUSE]	16.29	1	1	2	13	0.36	0.60	-1.05	-0.48

D3YTT4	Isobutyryl-CoA dehydrogenase, mitochondrial OS=Mus musculus GN=Acad8 PE=2 SV=1 - [D3YTT4_MOUSE]	38.50	2	15	15	50	-0.04	0.03	0.08	0.02
Q60770	Syntaxin-binding protein 3 OS=Mus musculus GN=Stxbp3 PE=1 SV=1 - [STXB3_MOUSE]	16.55	4	11	11	49	-0.03	-0.32	-0.03	-0.32
Q8VDQ8-2	Isoform 2 of NAD-dependent protein deacetylase sirtuin-2 OS=Mus musculus GN=Sirt2 - [SIR2_MOUSE]	20.45	3	6	6	19	0.11	0.35	0.22	0.24
G3UW34	MCG130981 OS=Mus musculus GN=Rpl9-ps6 PE=4 SV=1 - [G3UW34_MOUSE]	47.92	4	8	8	83	0.15	0.16	0.14	0.16
Q91YW3	DnaJ homolog subfamily C member 3 OS=Mus musculus GN=Dnajc3 PE=1 SV=1 - [DNJC3_MOUSE]	37.90	1	21	22	71	0.22	0.29	0.20	0.31
O35465-2	Isoform 2 of Peptidyl-prolyl cis-trans isomerase FKBP8 OS=Mus musculus GN=Fkbp8 - [FKBP8_MOUSE]	17.87	3	6	6	28	0.23	0.16	0.17	0.09
Q64191	N(4)-(beta-N-acetylglucosaminyl)-L-asparaginase OS=Mus musculus GN=Aga PE=2 SV=1 - [ASPG_MOUSE]	7.51	1	2	2	2	0.07	-0.43	0.19	-0.32
Q9DC51	Guanine nucleotide-binding protein G(k) subunit alpha OS=Mus musculus GN=Gnai3 PE=1 SV=3 - [GNAI3_MOUSE]	29.66	6	4	9	104	-0.10	-0.11	-0.02	0.03
Q3UVK0	Endoplasmic reticulum metallopeptidase 1 OS=Mus musculus GN=Ermp1 PE=1 SV=2 - [ERMP1_MOUSE]	12.25	4	8	9	18	-0.21	-0.47	-0.32	-0.47
Q6PAL7	AT-hook DNA-binding motif-containing protein 1 OS=Mus musculus GN=Ahdc1 PE=1 SV=1 - [AHDC1_MOUSE]	3.51	1	4	4	5	0.37	0.28	0.22	0.00
E9Q855	Secretory carrier-associated membrane protein 3 OS=Mus musculus GN=Scamp3 PE=2 SV=1 - [E9Q855_MOUSE]	27.62	3	4	4	13	0.15	0.39	0.02	0.18
E0CXV1	Protein Rapgef1 OS=Mus musculus GN=Rapgef1 PE=2 SV=1 - [E0CXV1_MOUSE]	1.90	4	1	1	2	0.26	-0.37	0.29	-0.34
O88543	COP9 signalosome complex subunit 3 OS=Mus musculus GN=Cops3 PE=1 SV=3 - [CSN3_MOUSE]	19.15	2	7	7	30	0.02	-0.09	0.08	0.06
P52293	Importin subunit alpha-1 OS=Mus musculus GN=Kpna2 PE=1 SV=2 - [IMA1_MOUSE]	19.85	6	7	8	17	0.53	0.59	0.42	0.53

Q91YJ2	Sorting nexin-4 OS=Mus musculus GN=Snx4 PE=2 SV=1 - [SNX4_MOUSE]	12.67	1	5	5	12	-0.02	0.19	0.27	0.30
Q3UN10	Wolframin OS=Mus musculus GN=Wfs1 PE=2 SV=1 - [Q3UN10_MOUSE]	2.46	2	2	2	10	0.29	0.32	0.28	0.35
G5E8M9	Intercellular adhesion molecule 2 (Fragment) OS=Mus musculus GN=Icam2 PE=2 SV=1 - [G5E8M9_MOUSE]	6.79	3	1	1	1	0.25	0.10	-0.05	-0.20
Q99KR7	Peptidyl-prolyl cis-trans isomerase F, mitochondrial OS=Mus musculus GN=Ppif PE= SV=1 - [PPIF_MOUSE]	52.91	1	8	11	126	-0.27	-0.08	-0.42	-0.20
Q6P8M1-2	Isoform 2 of Putative deoxyribonuclease TATDN1 OS=Mus musculus GN=Tatdn1 - [TATD1_MOUSE]	24.49	2	5	6	9	-0.15	-0.03	-0.09	0.01
Q9DBS1	Transmembrane protein 43 OS=Mus musculus GN=Tmem43 PE=1 SV=1 - [TMM43_MOUSE]	34.75	1	10	10	66	-0.05	-0.09	-0.03	-0.21
O88512	AP-1 complex subunit gamma-like 2 OS=Mus musculus GN=Ap1g2 PE=2 SV=2 - [AP1G2_MOUSE]	8.09	2	4	6	24	-0.04	0.09	-0.14	-0.01
P62071	Ras-related protein R-Ras2 OS=Mus musculus GN=Rras2 PE=1 SV=1 - [RRAS2_MOUSE]	33.82	1	4	6	123	-0.13	-0.31	0.15	-0.09
Q8VE88-2	Isoform 2 of Protein FAM114A2 OS=Mus musculus GN=Fam114a2 - [F1142_MOUSE]	14.69	2	5	5	17	0.19	0.08	0.21	0.23
Q1RME4	CAR-like soluble protein OS=Mus musculus GN=Gm1123 PE=2 SV=1 - [Q1RME4_MOUSE]	29.49	1	8	8	25	-0.70	-0.62	-0.63	-0.62
Q8CFV9	Riboflavin kinase OS=Mus musculus GN=Rfk PE=1 SV=2 - [RIFK_MOUSE]	25.81	1	3	3	17	0.27	0.26	0.09	0.05
Q8BG46	Ankyrin repeat domain-containing protein 10 OS=Mus musculus GN=Ankrd10 PE=2 SV=1 - [Q8BG46_MOUSE]	3.49	2	1	1	2	-0.01	0.21	-0.19	0.02
Q91W96	Anaphase-promoting complex subunit 4 OS=Mus musculus GN=Anapc4 PE=2 SV=1 - [APC4_MOUSE]	6.07	1	3	3	4	-0.45	-0.27	0.44	0.62
Q8R2E9	ERO1-like protein beta OS=Mus musculus GN=Ero1lb PE=2 SV=1 - [ERO1B_MOUSE]	8.57	1	3	4	8	0.37	0.06	0.67	0.22
O35621	Phosphomannomutase 1 OS=Mus musculus GN=Pmm1 PE=1 SV=1 - [PMM1_MOUSE]	27.48	2	4	6	39	-0.02	0.07	0.12	0.16
D6RFB9	Syntaxin-1A OS=Mus musculus GN=Stx1a PE=2 SV=1 - [D6RFB9_MOUSE]	13.67	2	2	3	10	-0.36	-0.61	-0.13	-0.41

Q8BJ34-4	Isoform 4 of Meiosis arrest female protein 1 OS=Mus musculus GN=Marf1 - [MARF1_MOUSE]	2.89	4	3	4	11	-0.10	-0.50	-0.07	-0.47
A2ARZ3	Fibrous sheath-interacting protein 2 OS=Mus musculus GN=Fsip2 PE=1 SV=3 - [FSIP2_MOUSE]	1.56	4	8	10	27	-0.16	0.10	-0.07	0.10
Q99L88	Beta-1-syntrophin OS=Mus musculus GN=Sntb1 PE=1 SV=4 [SNTB1_MOUSE]	11.17	4	3	5	21	-0.41	-0.56	0.21	0.00
B2RR83	Probable ATP-dependent RNA helicase YTHDC2 OS=Mus musculus GN=Ythdc2 PE=2 SV=1 - [YTDC2_MOUSE]	1.80	1	2	2	3	0.31	0.02	0.27	-0.02
Q8BU88	39S ribosomal protein L22, mitochondrial OS=Mus musculus GN=Mrpl22 PE=2 SV=1 - [RM22_MOUSE]	22.82	1	5	6	11	-0.08	-0.34	0.37	-0.16
Q8BVA2	Transmembrane protein 222 OS=Mus musculus GN=Tmem222 PE=2 SV=1 - [TM222_MOUSE]	12.50	1	1	1	7	0.09	0.05	0.10	0.06
Q69ZN7	Myoferlin OS=Mus musculus GN=Myof PE=1 SV=2 - [MYOF_MOUSE]	29.88	5	49	51	172	0.10	0.05	0.08	0.09
Q9Z0M3	Cadherin-20 OS=Mus musculus GN=Cdh20 PE=2 SV=1 - [CAD20_MOUSE]	1.37	1	1	1	1	-0.79	-1.41	-0.63	-1.26
Q61335	B-cell receptor-associated protein 31 OS=Mus musculus GN=Bcap31 PE=1 SV=4 - [BAP31_MOUSE]	28.98	1	11	11	83	0.17	0.22	0.12	0.14
Q8VBV7	COP9 signalosome complex subunit 8 OS=Mus musculus GN=Cops8 PE=1 SV=1 - [CSN8_MOUSE]	30.14	1	4	4	52	0.07	0.10	0.10	0.15
Q9JJG9	Nitric oxide-associated protein 1 OS=Mus musculus GN=Noa1 PE=1 SV=1 - [NOA1_MOUSE]	1.30	1	1	1	2	-0.49	-0.26	-0.58	-0.36
E9PWY6	2-amino-3-ketobutyrate coenzyme A ligase, mitochondrial OS=Mus musculus GN=Gcat PE=2 SV=1 - [E9PWY6_MOUSE]	8.90	2	3	4	10	0.14	-0.05	0.42	-0.12
Q9Z2B9	Ribosomal protein S6 kinase alpha-4 OS=Mus musculus GN=Rps6ka4 PE=1 SV=2 - [KS6A4_MOUSE]	5.56	1	3	3	8	0.22	-0.35	0.28	-0.25
Q62028	Secretory phospholipase A2 receptor OS=Mus musculus GN=Pla2r1 PE=1 SV=1 - [PLA2R_MOUSE]	1.75	2	3	3	4	-0.22	-0.16	-0.14	-0.09

P60879	Synaptosomal-associated protein 25 OS=Mus musculus GN=Snap25 PE=1 SV=1 - [SNP25_MOUSE]	34.95	2	5	6	48	-0.60	-0.40	-0.35	-0.52
Q8BIL5	Protein Hook homolog 1 OS=Mus musculus GN=Hook1 PE=1 SV=2 - [HOOK1_MOUSE]	16.07	3	12	12	21	0.56	0.53	0.43	0.44
D3Z3Z8	DNA-directed RNA polymerase II subunit RPB9 (Fragment) OS=Mus musculus GN=Polr2i PE=2 SV=1 [D3Z3Z8_MOUSE]	27.71	3	1	1	1	-0.20	0.33	0.13	0.66
Q7TPV4	Myb-binding protein 1A OS=Mus musculus GN=Mybbp1a PE=1 SV=2 - [MBB1A_MOUSE]	19.79	6	24	24	74	0.32	0.23	0.42	0.27
Q78XR0	Trafficking protein particle complex subunit 6A OS=Mus musculus GN=Trappc6a PE=2 SV=1 - [TPC6A_MOUSE]	5.03	2	1	1	2	-0.01	-0.14	0.27	0.15
Q8R0X7	Sphingosine-1-phosphate lyase 1 OS=Mus musculus GN=Sgpl1 PE=2 SV=1 [SGPL1_MOUSE]	21.48	3	12	12	44	0.19	-0.03	0.15	-0.08
P62754	40S ribosomal protein S6 OS=Mus musculus GN=Rps6 PE=1 SV=1 - [RS6_MOUSE]	29.32	1	9	9	70	0.03	0.24	0.06	0.23
Q505D7	Optic atrophy 3 protein homolog OS=Mus musculus GN=Opa3 PE=1 SV=1 - [OPA3_MOUSE]	11.73	1	2	2	6	0.04	0.02	0.16	0.32
Q7TPD0	Integrator complex subunit 3 OS=Mus musculus GN=Ints3 PE=1 SV=2 - [INT3_MOUSE]	8.84	2	7	7	11	0.69	0.45	0.37	0.27
G8JL40	Lysine-specific histone demethylase 1A (Fragment) OS=Mus musculus GN=Kdm1a PE=4 SV=1 - [G8JL40_MOUSE]	4.98	5	3	3	16	0.51	-0.15	0.43	-0.15
Q99MK8	Beta-adrenergic receptor kinase 1 OS=Mus musculus GN=Adrbk1 PE=2 SV=2 - [ARBK1_MOUSE]	8.27	5	5	5	11	0.04	-0.04	0.08	0.11
D3YUM1	NADH dehydrogenase [ubiquinone] flavoprotein 1, mitochondrial OS=Mus musculus GN=Ndufv1 PE=2 SV=1 - [D3YUM1_MOUSE]	34.51	6	15	15	133	0.02	0.13	-0.08	-0.03
Q6DFW0	Protein C9orf72 homolog OS=Mus musculus PE=2 SV=1 - [CI072_MOUSE]	1.90	2	1	1	1	0.02	-0.24	0.37	0.10
Q91WX5	Bak1 protein OS=Mus musculus GN=Bak1 PE=2 SV=1 - [Q91WX5_MOUSE]	66.23	4	4	4	19	-0.02	0.13	-0.05	0.10

P35293	Ras-related protein Rab-18 OS=Mus musculus GN=Rab18 PE=2 SV=2 - [RAB18_MOUSE]	37.38	1	7	7	48	0.13	0.06	0.12	0.13
Q8VCP8	Adenylate kinase isoenzyme 6 OS=Mus musculus GN=Taf9 PE=2 SV=1 - [KAD6_MOUSE]	5.23	1	1	1	1	-0.31	-0.54	-0.12	-0.34
Q9Z1X4	Interleukin enhancer-binding factor 3 OS=Mus musculus GN=Ilf3 PE=1 SV=2 - [ILF3_MOUSE]	23.39	4	13	15	63	0.10	0.26	0.09	0.11
Q80VI1	E3 ubiquitin-protein ligase TRIM56 OS=Mus musculus GN=Trim56 PE=1 SV=1 - [TRI56_MOUSE]	18.66	2	12	12	30	-0.11	-0.09	-0.04	0.02
P62717	60S ribosomal protein L18a OS=Mus musculus GN=Rpl18a PE=1 SV=1 - [RL18A_MOUSE]	26.14	2	5	5	54	0.25	0.18	0.21	0.22
Q9WTP9	Ventral anterior homeobox 2 OS=Mus musculus GN=Vax2 PE=1 SV=1 - [VAX2_MOUSE]	2.05	1	1	1	1	0.15	0.01	0.02	-0.12
Q9R008	Mevalonate kinase OS=Mus musculus GN=Mvk PE=2 SV=1 - [KIME_MOUSE]	16.46	3	4	4	18	0.21	-0.20	-0.10	-0.34
Q9ESP1	Stromal cell-derived factor 2-like protein 1 OS=Mus musculus GN=Sdf2l1 PE=2 SV=2 - [SDF2L_MOUSE]	28.51	1	3	3	34	0.43	0.51	0.39	0.48
P62196	26S protease regulatory subunit 8 OS=Mus musculus GN=Psmc5 PE=1 SV=1 - [PRS8_MOUSE]	51.97	2	18	19	142	0.43	0.35	0.44	0.33
Q8BY89-2	Isoform 2 of Choline transporter-like protein 2 OS=Mus musculus GN=Slc44a2 - [CTL2_MOUSE]	8.95	2	5	5	6	-0.03	0.21	-0.22	0.08
Q9JLZ8	Single Ig IL-1-related receptor OS=Mus musculus GN=Sigirr PE=1 SV=2 - [SIGIR_MOUSE]	10.51	2	3	3	3	-0.30	-0.19	-0.40	-0.31
P23475	X-ray repair cross-complementing protein 6 OS=Mus musculus GN=Xrcc6 PE=1 SV=5 [XRCC6_MOUSE]	20.23	5	10	11	38	0.14	0.05	0.16	0.18
Q08189	Protein-glutamine gamma-glutamyltransferase E OS=Mus musculus GN=Tgm3 PE=1 SV=2 [TGM3_MOUSE]	47.33	1	28	28	514	-0.08	-0.30	-0.07	-0.24
Q6PB44-2	Isoform 2 of Tyrosine-protein phosphatase non receptor type 23 OS=Mus musculus GN=Ptpn23 - [PTN23_MOUSE]	7.46	2	10	10	29	0.20	0.52	0.28	0.53
P03930	ATP synthase protein 8 OS=Mus musculus GN=Mtatp8 PE=1 SV=1 - [ATP8_MOUSE]	55.22	1	5	5	42	0.03	0.23	0.05	0.17

P86049	Probable RNA-binding protein 46 OS=Mus musculus GN=Rbm46 PE=4 SV=1 - [RBM46_MOUSE]	5.44	1	1	2	4	0.06	-1.05	-0.37	-1.48
Q9CXX9	CUE domain-containing protein 2 OS=Mus musculus GN=Cuedc2 PE=2 SV=1 - [CUED2_MOUSE]	3.17	1	1	1	3	0.16	0.21	0.01	0.23
Q8CI32	BAG family molecular chaperone regulator 5 OS=Mus musculus GN=Bag5 PE=1 SV=1 - [BAG5_MOUSE]	8.95	1	4	4	16	0.38	0.33	0.18	0.10
P01901	H-2 class I histocompatibility antigen, K-B alpha chain OS=Mus musculus GN=H2-K1 PE=1 SV=1 - [HA1B_MOUSE]	39.02	6	5	10	98	0.25	0.29	0.27	0.22
Q8C7U7	Polypeptide N-acetylgalactosaminyltransferase 6 OS=Mus musculus GN=Galnt6 PE=2 SV=1 - [GALT6_MOUSE]	9.81	1	4	4	12	-0.11	0.13	-0.13	0.09
B1AT03	DNA ligase OS=Mus musculus GN=Lig3 PE=3 SV=1 - [B1AT03_MOUSE]	7.97	7	6	6	12	-0.06	0.12	0.05	0.19
Q80TM6-2	Isoform 2 of R3H domain-containing protein 2 OS=Mus musculus GN=R3hdm2 [R3HD2_MOUSE]	1.31	3	1	1	1	-0.07	-0.45	-0.23	-0.61
D3Z2Y6	Protein S100a16 (Fragment) OS=Mus musculus GN=S100a16 PE=2 SV=1 - [D3Z2Y6_MOUSE]	47.95	2	3	3	24	0.22	0.16	0.22	0.02
Q8C7K6	Prenylcysteine oxidase-like OS=Mus musculus GN=Pcyox1l PE=2 SV=1 - [PCYXL_MOUSE]	3.84	1	1	1	6	-0.35	-0.54	0.08	-0.30
G3UX26	Voltage-dependent anion-selective channel protein 2 (Fragment) OS=Mus musculus GN=Vdac2 PE=4 SV=1 - [G3UX26_MOUSE]	52.30	4	12	13	226	-0.26	-0.19	-0.24	-0.15
E9QAT4	Protein Sec16a OS=Mus musculus GN=Sec16a PE=2 SV=1 - [E9QAT4_MOUSE]	12.13	3	22	22	46	0.17	-0.06	0.11	0.04
P60229	Eukaryotic translation initiation factor 3 subunit E OS=Mus musculus GN=Eif3e PE=1 SV=1 - [EIF3E_MOUSE]	35.28	1	13	13	48	0.43	0.15	0.25	0.06
D3YVA2	Neurocalcin-delta (Fragment) OS=Mus musculus GN=Ncald PE=2 SV=1 - [D3YVA2_MOUSE]	41.38	4	4	8	24	-0.38	-0.40	-0.33	-0.24
P62702	40S ribosomal protein S4, X isoform OS=Mus musculus GN=Rps4x PE=2 SV=2 - [RS4X_MOUSE]	49.81	3	15	15	131	0.17	0.26	0.17	0.17

Q8C5W0-2	Isoform 2 of Calmin OS=Mus musculus GN=Clmn - [CLMN_MOUSE]	15.64	4	10	10	28	0.44	0.27	0.36	0.37
Q9DCC4	Pyrroline-5-carboxylate reductase 3 OS=Mus musculus GN=Pycrl PE=2 SV=2 - [P5CR3_MOUSE]	31.75	1	8	8	39	-0.08	-0.15	-0.18	-0.18
Q9WVJ2	26S proteasome non-ATPase regulatory subunit 13 OS=Mus musculus GN=Psmd13 PE=1 SV=1 - [PSD13_MOUSE]	49.47	6	15	15	72	0.22	0.19	0.33	0.12
E9Q3Q6	CD166 antigen OS=Mus musculus GN=Alcam PE=2 SV=1 - [E9Q3Q6_MOUSE]	39.47	5	15	15	40	-0.18	-0.22	-0.40	-0.30
H3BJR7	WD repeat-containing protein 37 (Fragment) OS=Mus musculus GN=Wdr37 PE=2 SV=1 - [H3BJR7_MOUSE]	10.00	6	2	2	4	-0.44	-0.43	0.01	0.03
Q9DCD2	Pre-mRNA-splicing factor SYF1 OS=Mus musculus GN=Xab2 PE=2 SV=1 - [SYF1_MOUSE]	8.89	3	7	7	11	-0.43	-0.34	-0.25	-0.19
P18872-2	Isoform Alpha-2 of Guanine nucleotide-binding protein G(o) subunit alpha OS=Mus musculus GN=Gnao1 - [GNAO_MOUSE]	32.77	6	9	12	101	-0.35	-0.46	-0.24	-0.35
Q922Q1	MOSC domain-containing protein 2, mitochondrial OS=Mus musculus GN=Marc2 PE=1 SV=1 - [MOSC2_MOUSE]	41.42	6	12	12	78	-0.29	-0.22	-0.16	-0.16
Q3TUA9	Probable inactive protein kinase-like protein SgK196 OS=Mus musculus GN=Sgk196 PE=2 SV=2 - [SG196_MOUSE]	5.44	1	1	1	2	0.18	-0.05	0.31	0.09
Q61235	Beta-2-syntrophin OS=Mus musculus GN=Sntb2 PE=1 SV=2 [SNTB2_MOUSE]	44.04	3	15	17	72	-0.27	-0.38	-0.25	-0.16
A4Q9F1	Protein monoglycylase TTLL8 OS=Mus musculus GN=Ttll8 PE=1 SV=1 - [TTLL8_MOUSE]	3.61	1	2	2	3	0.08	-0.50	0.11	-0.46
Q8BY79	Solute carrier family 35 member G1 OS=Mus musculus GN=Slc35g1 PE=2 SV=1 - [S35G1_MOUSE]	1.63	1	1	1	2	0.12	0.05	0.29	0.22
G3UXK3	Protein 2410137M14Rik OS=Mus musculus GN=2410137M14Rik PE=2 SV=1 - [G3UXK3_MOUSE]	13.60	2	1	1	1	1.17	0.86	0.92	0.60
Q80ZD9	Amphoterin-induced protein 2 OS=Mus musculus GN=Amigo2 PE=1 SV=1 - [AMGO2_MOUSE]	3.66	1	1	1	2	0.18	0.53	0.09	0.43

O55186	CD59A glycoprotein OS=Mus musculus GN=Cd59a PE=2 SV=1 - [CD59A_MOUSE]	13.82	1	1	1	8	0.35	0.07	0.36	0.09
P47911	60S ribosomal protein L6 OS=Mus musculus GN=Rpl6 PE=1 SV=3 - [RL6_MOUSE]	34.12	2	12	12	109	0.12	0.19	0.09	0.16
P19096	Fatty acid synthase OS=Mus musculus GN=Fasn PE=1 SV=2 - [FAS_MOUSE]	42.89	1	80	80	542	0.14	0.16	0.17	0.14
B7ZNY3	Pard3 protein OS=Mus musculus GN=Pard3 PE=2 SV=1 - [B7ZNY3_MOUSE]	5.16	20	5	6	9	-0.19	-0.05	0.08	-0.24
D3Z286	Protein Tmppe OS=Mus musculus GN=Tmppe PE=4 SV=2 - [D3Z286_MOUSE]	3.80	1	1	1	1	0.19	-0.08	0.34	0.07
O08746-2	Isoform 2 of Matrilin-2 OS=Mus musculus GN=Matn2 - [MATN2_MOUSE]	4.91	2	4	4	11	0.13	0.15	-0.01	-0.15
Q91VE0	Long-chain fatty acid transport protein 4 OS=Mus musculus GN=Slc27a4 PE=1 SV=1 - [S27A4_MOUSE]	23.64	1	11	11	34	-0.26	-0.32	-0.23	-0.35
P49442	Inositol polyphosphate 1-phosphatase OS=Mus musculus GN=Inpp1 PE=1 SV=2 - [INPP_MOUSE]	15.66	2	6	6	19	-0.07	-0.05	0.00	-0.10
D3Z5T2	Armadillo repeat-containing protein 10 OS=Mus musculus GN=Armc10 PE=2 SV=1 - [D3Z5T2_MOUSE]	31.93	3	5	5	29	0.16	-0.39	0.31	0.06
A6H5Y3	Methionine synthase OS=Mus musculus GN=Mtr PE=2 SV=1 - [METH_MOUSE]	6.78	1	6	7	7	0.22	0.00	0.60	0.38
Q3U8K7-5	Isoform 5 of Histone-lysine N-methyltransferase SUV420H1 OS=Mus musculus GN=Suv420h1 - [SV421_MOUSE]	1.96	3	1	2	3	-0.05	0.01	0.15	0.21
Q6P1B3	PILR alpha-associated neural protein OS=Mus musculus GN=Pianp PE=1 SV=1 - [PIANP_MOUSE]	3.96	1	1	1	2	-0.39	0.09	-0.31	0.17
Q80WQ2	Protein VAC14 homolog OS=Mus musculus GN=Vac14 PE=1 SV=1 - [VAC14_MOUSE]	4.86	1	3	3	5	0.56	0.04	0.64	-0.21
P13595	Neural cell adhesion molecule 1 OS=Mus musculus GN=Ncam1 PE=1 SV=3 - [NCAM1_MOUSE]	16.41	6	15	15	63	-0.30	-0.21	-0.34	-0.29
Q9Z1F9	SUMO-activating enzyme subunit 2 OS=Mus musculus GN=Uba2 PE=2 SV=1 - [SAE2_MOUSE]	36.99	5	15	15	103	0.26	0.20	0.27	0.31
Q8VEJ9	Vacuolar protein sorting associated protein 4A OS=Mus musculus GN=Vps4a PE=1 SV=1 - [VPS4A_MOUSE]	14.87	1	4	7	23	0.31	0.24	0.45	0.40

Q6P4S8	Integrator complex subunit 1 OS=Mus musculus GN=Ints1 PE=1 SV=2 - [INT1_MOUSE]	1.09	2	2	2	8	0.24	-0.04	0.39	0.11
Q9WVE8	Protein kinase C and casein kinase substrate in neurons protein 2 OS=Mus musculus GN=Pacsin2 PE=1 SV=1 - [PACN2_MOUSE]	36.83	1	17	17	94	0.07	0.18	0.08	0.19
Q9DCT8	Cysteine-rich protein 2 OS=Mus musculus GN=Crip2 PE=1 SV=1 - [CRIP2_MOUSE]	45.67	5	6	7	118	-0.17	-0.05	-0.15	-0.03
Q91W61	F-box/LRR-repeat protein 15 OS=Mus musculus GN=Fbxl15 PE=1 SV=2 - [FXL15_MOUSE]	3.67	1	1	1	1	0.42	0.34	0.68	0.60
Q9D0K1	Peroxisomal membrane protein PEX13 OS=Mus musculus GN=Pex13 PE=1 SV=1 [PEX13_MOUSE]	4.94	1	2	2	3	0.11	-0.13	0.12	-0.13
Q8BH04	Phosphoenolpyruvate carboxykinase [GTP], mitochondrial OS=Mus musculus GN=Pck2 PE=2 SV=1 - [PCKGM_MOUSE]	35.47	1	16	16	137	0.05	-0.10	0.00	-0.08
F8VQ29	Protein Iqgap3 OS=Mus musculus GN=Iqgap3 PE=2 SV=1 - [F8VQ29_MOUSE]	2.51	1	3	5	23	0.11	0.20	0.20	0.29
Q8VCM5-3	Isoform 3 of Mitochondrial ubiquitin ligase activator of NFKB 1 OS=Mus musculus GN=Mul1 - [MUL1_MOUSE]	12.97	3	2	2	18	-0.19	1.01	-2.08	0.10
Q9JJF0	Nucleosome assembly protein 1-like 5 OS=Mus musculus GN=Nap1l5 PE=2 SV=1 - [NP1L5_MOUSE]	7.05	1	1	1	1	0.57	0.26	0.52	0.22
Q99LD9	Translation initiation factor eIF-2B subunit beta OS=Mus musculus GN=Eif2b2 PE=2 SV=1 - [EI2BB_MOUSE]	16.81	3	5	5	16	0.06	0.14	0.18	0.16
Q3U7R1	Extended synaptotagmin-1 OS=Mus musculus GN=Esyt1 PE=2 SV=2 - [ESYT1_MOUSE]	28.85	2	27	27	138	-0.13	-0.13	-0.14	-0.08
P48758	Carbonyl reductase [NADPH] 1 OS=Mus musculus GN=Cbr1 PE=1 SV=3 - [CBR1_MOUSE]	64.62	1	15	16	554	-0.06	0.01	0.00	0.07
Q91W69	Epsin-3 OS=Mus musculus GN=Epn3 PE=2 SV=1 - [EPN3_MOUSE]	8.96	2	2	4	8	-0.25	0.14	-0.32	0.07
Q8BPE4	Transmembrane protein 177 OS=Mus musculus GN=Tmem177 PE=2 SV=1 - [TM177_MOUSE]	3.22	1	1	1	1	-0.45	-0.14	-0.19	0.12
Q91VF2	Histamine N-methyltransferase OS=Mus musculus GN=Hnmt PE=1 SV=1 - [HNMT_MOUSE]	16.95	1	3	3	4	-0.27	-0.39	0.03	0.05

Q921Y4	Major facilitator superfamily domain-containing protein 5 OS=Mus musculus GN=Mfsd5 PE=2 SV=1 - [MFSD5_MOUSE]	3.11	1	1	1	2	-0.14	-0.14	-0.58	-0.59
Q3V3N7	Protein Bbs1 OS=Mus musculus GN=Bbs1 PE=2 SV=1 - [Q3V3N7_MOUSE]	1.85	1	1	1	1	1.11	1.20	1.03	1.12
Q8VDD5	Myosin-9 OS=Mus musculus GN=Myh9 PE=1 SV=4 - [MYH9_MOUSE]	60.00	3	100	134	3946	0.16	0.21	0.14	0.18
P43247	DNA mismatch repair protein Msh2 OS=Mus musculus GN=Msh2 PE=2 SV=1 - [MSH2_MOUSE]	10.05	2	8	8	25	0.14	0.20	0.22	0.29
Q91W39	Nuclear receptor coactivator 5 OS=Mus musculus GN=Ncoa5 PE=1 SV=1 - [NCOA5_MOUSE]	6.74	4	4	4	13	0.02	-0.18	-0.04	-0.37
P24369	Peptidyl-prolyl cis-trans isomerase B OS=Mus musculus GN=Ppib PE=2 SV=2 - [PPIB_MOUSE]	60.65	1	14	15	414	0.16	0.21	0.09	0.19
Q8VBT9	Tether containing UBX domain for GLUT4 OS=Mus musculus GN=Aspscr1 PE=1 SV=1 - [ASPC1_MOUSE]	14.36	13	6	6	14	-0.08	0.17	0.10	0.32
D3Z0I8	Transmembrane protein 109 (Fragment) OS=Mus musculus GN=Tmem109 PE=2 SV=1 - [D3Z0I8_MOUSE]	13.15	6	4	4	24	-0.05	0.06	-0.16	-0.04
Q8C052	Microtubule-associated protein 1S OS=Mus musculus GN=Map1s PE=1 SV=2 - [MAP1S_MOUSE]	11.41	1	9	9	38	0.08	0.19	0.15	0.23
Q9DC33	High mobility group protein 20A OS=Mus musculus GN=Hmg20a PE=2 SV=1 - [HM20A_MOUSE]	5.78	1	1	1	3	-0.24	-0.11	0.11	0.11
Q6P9N1	Hyccin OS=Mus musculus GN=Fam126a PE=1 SV=2 - [HYCCI_MOUSE]	6.72	3	3	3	34	0.08	-0.07	-0.39	-0.55
Q8BG32	26S proteasome non-ATPase regulatory subunit 11 OS=Mus musculus GN=Psmd11 PE=1 SV=3 - [PSD11_MOUSE]	53.55	12	20	20	92	0.32	0.42	0.23	0.31
O70400	PDZ and LIM domain protein 1 OS=Mus musculus GN=Pdlim1 PE=2 SV=4 - [PDLI1_MOUSE]	74.01	1	19	19	378	-0.21	-0.19	-0.22	-0.16
G5E8J3	Bromodomain and WD repeat domain containing 2, isoform CRA_a OS=Mus musculus GN=Wdr11 PE=4 SV=1 - [G5E8J3_MOUSE]	4.58	2	5	5	10	0.26	-0.03	0.40	0.16

Q8VHL0-2	Isoform 2 of Urea transporter 1 OS=Mus musculus GN=Slc14a1 - [UT1_MOUSE]	1.82	1	1	1	3	0.05	-0.19	0.08	-0.16
Q9DAW9	Calponin-3 OS=Mus musculus GN=Cnn3 PE=2 SV=1 - [CNN3_MOUSE]	40.91	1	8	12	149	0.07	-0.19	0.09	-0.25
A2ATI9	Golgi reassembly stacking protein 2, isoform CRA_d OS=Mus musculus GN=Gorasp2 PE=4 SV=1 - [A2ATI9_MOUSE]	21.11	5	6	7	52	0.08	0.15	-0.10	0.01
Q4VA93	Protein kinase C OS=Mus musculus GN=Prkca PE=2 SV=1 - [Q4VA93_MOUSE]	15.63	2	1	10	35	-0.04	0.27	-0.33	-0.02
Q9D6K9	Ceramide synthase 5 OS=Mus musculus GN=Cers5 PE=1 SV=1 [CERS5_MOUSE]	3.62	4	2	2	2	0.34	0.41	0.32	0.38
Q9EQQ9	Bifunctional protein NCOAT OS=Mus musculus GN=Mgea5 PE=1 SV=2 - [NCOAT_MOUSE]	2.84	2	2	2	3	-0.49	-2.92	0.28	-2.15
Q9WVH9	Fibulin-5 OS=Mus musculus GN=Fbln5 PE=2 SV=1 - [FBLN5_MOUSE]	21.65	1	8	8	58	-0.21	-0.19	-0.24	-0.22
O08789	Max-binding protein MNT OS=Mus musculus GN=Mnt PE=2 SV=2 - [MNT_MOUSE]	3.05	1	2	2	2	0.04	0.05	0.14	0.15
Q8CCF0-2	Isoform 2 of U4/U6 small nuclear ribonucleoprotein Prp31 OS=Mus musculus GN=Prpf31 - [PRP31_MOUSE]	17.65	5	6	7	25	0.24	0.00	0.16	0.10
P62264	40S ribosomal protein S14 OS=Mus musculus GN=Rps14 PE=2 SV=3 - [RS14_MOUSE]	43.05	3	8	8	161	0.19	0.27	0.26	0.14
O88632-2	Isoform A of Semaphorin-3F OS=Mus musculus GN=Sema3f - [SEM3F_MOUSE]	0.93	2	1	1	1	0.13	0.11	0.01	-0.01
O55225	Otogelin OS=Mus musculus GN=Otog PE=1 SV=2 - [OTOG_MOUSE]	0.48	2	1	1	2	-0.23	-1.75	-0.05	-1.57
Q9JMA9	Sodium- and chloride-dependent neutral and basic amino acid transporter B(0+) OS=Mus musculus GN=Slc6a14 PE=2 SV=1 - [S6A14_MOUSE]	7.37	1	4	4	5	0.06	0.09	0.09	0.11
G3X999	Protein A330021E22Rik (Fragment) OS=Mus musculus GN=A330021E22Rik PE=4 SV=1 - [G3X999_MOUSE]	1.22	4	1	1	1	-0.09	-0.17	-0.06	-0.14
P97930	Thymidylate kinase OS=Mus musculus GN=Dtymk PE=2 SV=2 - [KTHY_MOUSE]	25.94	3	5	5	15	-0.02	0.16	0.03	0.06

J3QP37	MCG1038270, isoform CRA_a OS=Mus musculus GN=Gm5480 PE=4 SV=1 - [J3QP37_MOUSE]	14.29	1	1	1	4	0.44	0.03	0.78	0.37
D3YU12	NmrA-like family domain-containing protein 1 OS=Mus musculus GN=Nmral1 PE=2 SV=1 - [D3YU12_MOUSE]	26.42	5	6	7	23	0.39	0.44	0.37	0.39
Q04736	Tyrosine-protein kinase Yes OS=Mus musculus GN=Yes1 PE=1 SV=3 - [YES_MOUSE]	21.81	36	4	11	47	0.15	0.25	0.00	0.04
Q9CY34	NEDD8-conjugating enzyme UBE2F OS=Mus musculus GN=Ube2f PE=1 SV=1 [UB2FA_MOUSE]	29.19	1	2	3	11	0.30	0.39	0.13	0.36
Q99P88	Nuclear pore complex protein Nup155 OS=Mus musculus GN=Nup155 PE=2 SV=1 - [NU155_MOUSE]	2.44	1	3	3	6	0.27	0.27	0.21	0.17
B2RWH8	Dimethylaniline monooxygenase [N-oxide-forming] OS=Mus musculus GN=Gm4846 PE=2 SV=1 - [B2RWH8_MOUSE]	6.69	2	1	2	5	0.10	0.58	0.23	0.71
Q91WS0	CDGSH iron-sulfur domain-containing protein 1 OS=Mus musculus GN=Cisd1 PE=1 SV=1 - [CISD1_MOUSE]	44.44	1	5	5	54	-0.07	-0.06	-0.16	-0.11
P54818	Galactocerebrosidase OS=Mus musculus GN=Galc PE=1 SV=2 - [GALC_MOUSE]	3.51	1	2	2	6	0.11	0.22	0.08	0.18
Q8VD26-3	Isoform 3 of Transmembrane protein 143 OS=Mus musculus GN=Tmem143 - [TM143_MOUSE]	10.42	4	1	1	8	-0.02	-0.38	0.40	0.04
P01648	Ig kappa chain V-V region HP 91A3 OS=Mus musculus PE=1 SV=1 - [KV5AF_MOUSE]	16.67	2	1	1	1	0.44	0.80	0.54	0.89
Q8BXV2	BRI3-binding protein OS=Mus musculus GN=Bri3bp PE=2 SV=1 - [BRI3B_MOUSE]	15.81	1	3	3	13	0.30	-0.29	0.04	-0.36
E9Q4T9	Transmembrane protease serine 4 OS=Mus musculus GN=Tmprss4 PE=2 SV=1 - [E9Q4T9_MOUSE]	6.87	2	2	2	3	0.10	-0.01	0.09	-0.02
P16858	Glyceraldehyde-3-phosphate dehydrogenase OS=Mus musculus GN=Gapdh PE=1 SV=2 - [G3P_MOUSE]	72.67	8	21	22	938	0.35	0.44	0.31	0.44
Q9CQW2	ADP-ribosylation factor like protein 8B OS=Mus musculus GN=Arl8b PE=2 SV=1 [ARL8B_MOUSE]	31.18	1	1	5	30	0.23	0.64	0.04	0.45
Q4ZJM7	Otolin-1 OS=Mus musculus GN=Otol1 PE=1 SV=2 - [OTOL1_MOUSE]	6.22	1	2	2	10	-0.28	-0.02	-0.20	0.04

E9PXU9	Dysferlin OS=Mus musculus GN=Dysf PE=2 SV=1 - [E9PXU9_MOUSE]	20.16	6	29	31	97	0.27	0.25	0.23	0.26
Q8BM96	Probable G-protein coupled receptor 128 OS=Mus musculus GN=Gpr128 PE=1 SV=2 - [GP128_MOUSE]	2.68	1	2	2	5	0.05	0.23	0.39	0.57
Q6ZWN5	40S ribosomal protein S9 OS=Mus musculus GN=Rps9 PE=2 SV=3 - [RS9_MOUSE]	41.75	6	11	12	128	0.00	0.01	0.08	0.06
G3UZU7	T-cell differentiation antigen CD6 (Fragment) OS=Mus musculus GN=Cd6 PE=2 SV=1 - [G3UZU7_MOUSE]	4.28	4	1	1	1	0.31	0.31	0.34	0.33
Q8BXZ1	Protein disulfide-isomerase TMX3 OS=Mus musculus GN=Tmx3 PE=1 SV=2 [TMX3_MOUSE]	19.96	1	7	8	33	0.17	0.29	0.13	0.35
Q922D8	C-1-tetrahydrofolate synthase, cytoplasmic OS=Mus musculus GN=Mthfd1 PE=1 SV=4 - [C1TC_MOUSE]	34.97	1	30	30	180	0.42	0.45	0.31	0.45
Q5EBG8	Uncharacterized protein C1orf50 homolog OS=Mus musculus PE=2 SV=1 - [CA050_MOUSE]	16.58	1	3	3	6	-0.49	-0.44	0.02	-0.23
Q9QUI0	Transforming protein RhoA OS=Mus musculus GN=Rhoa PE=1 SV=1 - [RHOA_MOUSE]	47.15	3	2	7	143	0.21	0.57	0.10	0.59
P23116	Eukaryotic translation initiation factor 3 subunit A OS=Mus musculus GN=Eif3a PE=1 SV=5 - [EIF3A_MOUSE]	25.30	1	31	31	218	0.20	0.17	0.17	0.07
P41105	60S ribosomal protein L28 OS=Mus musculus GN=Rpl28 PE=1 SV=2 [RL28_MOUSE]	51.09	2	10	10	77	0.07	0.14	0.05	0.13
E9Q0A3	Protein Arhgef11 (Fragment) OS=Mus musculus GN=Arhgef11 PE=2 SV=1 - [E9Q0A3_MOUSE]	2.51	3	3	3	5	-0.09	-0.28	0.19	0.01
Q3U1T9-3	Isoform 3 of DENN domain-containing protein 1B OS=Mus musculus GN=Dennd1b - [DEN1B_MOUSE]	6.90	4	1	1	1	0.19	0.24	-0.07	-0.03
F7AR15	Protein Nxf7 (Fragment) OS=Mus musculus GN=Nxf7 PE=4 SV=1 - [F7AR15_MOUSE]	9.26	2	4	4	6	-0.02	0.30	-0.09	0.23
P61458	Pterin-4-alpha-carbinolamine dehydratase OS=Mus musculus GN=Pcbd1 PE=1 SV=2 - [PHS_MOUSE]	57.69	1	5	6	29	-0.17	-0.39	-0.12	-0.21
Q8CFI7	DNA-directed RNA polymerase II subunit RPB2 OS=Mus musculus GN=Polr2b PE=2 SV=2 - [RPB2_MOUSE]	14.05	1	13	13	35	0.33	0.39	0.41	0.39

Q80SW1	Putative adenosylhomocysteinase 2 OS=Mus musculus GN=Ahcyl1 PE=1 SV=1 - [SAHH2_MOUSE]	33.40	5	5	18	141	0.07	0.29	0.04	0.37
Q9R187	Tumor necrosis factor receptor superfamily member EDAR OS=Mus musculus GN=Edar PE=1 SV=1 - [EDAR_MOUSE]	1.34	1	1	1	1	-0.40	-0.18	-0.31	-0.09
P36552	Coproporphyrinogen-III oxidase, mitochondrial OS=Mus musculus GN=Cpox PE=1 SV=2 - [HEM6_MOUSE]	32.28	1	10	10	48	-0.04	0.00	-0.01	0.05
Q9JIY8	Probable N-acetyltransferase CML3 OS=Mus musculus GN=Cml3 PE=2 SV=1 - [CMLO3_MOUSE]	3.10	2	1	1	1	1.97	1.32	1.75	1.10
Q61526	Receptor tyrosine-protein kinase erbB-3 OS=Mus musculus GN=Erbb3 PE=1 SV=2 [ERBB3_MOUSE]	2.24	1	2	2	3	0.41	-0.10	0.07	-0.43
Q69ZK0	Phosphatidylinositol 3,4,5-trisphosphate-dependent Rac exchanger 1 protein OS=Mus musculus GN=Prex1 PE=1 SV=2 [PREX1_MOUSE]	2.42	2	3	3	5	0.09	0.19	0.10	0.16
P27773	Protein disulfide-isomerase A3 OS=Mus musculus GN=Pdia3 PE=1 SV=2 - [PDIA3_MOUSE]	70.30	2	45	45	1519	0.18	0.27	0.13	0.23
A7XUZ6	Selection and upkeep of intraepithelial T-cells protein 6 OS=Mus musculus GN=Skint6 PE=2 SV=1 - [SKIT6_MOUSE]	4.84	3	3	5	9	-0.14	0.12	1.00	1.26
P62141	Serine/threonine-protein phosphatase PP1-beta catalytic subunit OS=Mus musculus GN=Ppp1cb PE=1 SV=3 - [PP1B_MOUSE]	57.19	1	6	14	196	0.15	-0.02	0.20	0.04
Q00493	Carboxypeptidase E OS=Mus musculus GN=Cpe PE=1 SV=2 - [CBPE_MOUSE]	28.15	1	10	10	36	-0.58	-0.61	-0.58	-0.71
Q8CJ00-2	Isoform 2 of NADPH oxidase activator 1 OS=Mus musculus GN=Noxa1 - [NOXA1_MOUSE]	11.24	2	4	4	8	0.72	0.61	0.77	0.55
O35704	Serine palmitoyltransferase 1 OS=Mus musculus GN=Sptlc1 PE=2 SV=2 - [SPTC1_MOUSE]	15.22	1	5	5	21	0.40	0.41	0.24	0.16
Q99N87	28S ribosomal protein S5, mitochondrial OS=Mus musculus GN=Mrps5 PE=2 SV=1 - [RT05_MOUSE]	10.19	2	5	5	12	0.03	-0.06	0.07	0.08

Q9DB73	NADH-cytochrome b5 reductase 1 OS=Mus musculus GN=Cyb5r1 PE=2 SV=1 - [NB5R1_MOUSE]	22.30	4	6	6	21	-0.07	0.01	-0.09	0.03
Q3TL44	NLR family member X1 OS=Mus musculus GN=Nlrx1 PE=2 SV=1 [NLRX1_MOUSE]	6.87	1	6	6	13	0.08	-0.05	-0.06	-0.21
Q8CHC4	Synaptojanin-1 OS=Mus musculus GN=Synj1 PE=1 SV=3 [SYNJ1_MOUSE]	6.54	8	7	7	13	-0.14	-0.28	0.10	-0.38
A2ADZ8	IQ domain-containing protein C OS=Mus musculus GN=Iqcc PE=2 SV=1 - [IQCC_MOUSE]	5.98	1	2	2	2	0.09	-0.14	-0.02	-0.25
Q64437	Alcohol dehydrogenase class 4 mu/sigma chain OS=Mus musculus GN=Adh7 PE=2 SV=2 - [ADH7_MOUSE]	16.04	1	2	3	9	0.15	0.48	0.27	0.60
E9Q740	Signal recognition particle subunit SRP72 OS=Mus musculus GN=Srp72 PE=2 SV=1 [E9Q740_MOUSE]	25.25	2	13	13	46	0.14	0.20	0.11	0.05
E9Q3M0	Mothers against decapentaplegic homolog 2 OS=Mus musculus GN=Smad2 PE=2 SV=1 - [E9Q3M0_MOUSE]	11.06	5	2	4	6	-0.01	-0.03	-0.82	-0.71
Q3U5F4	YrdC domain-containing protein, mitochondrial OS=Mus musculus GN=Yrdc PE=1 SV=1 - [YRDC_MOUSE]	5.00	1	1	1	2	0.02	0.07	-0.90	-0.85
Q9D6M3	Mitochondrial glutamate carrier 1 OS=Mus musculus GN=Slc25a22 PE=1 SV=1 - [GHC1_MOUSE]	16.72	9	6	6	9	-0.14	-0.04	-0.10	0.05
Q60841-3	Isoform 3 of Reelin OS=Mus musculus GN=Reln - [RELN_MOUSE]	2.04	3	2	2	5	-0.35	-0.53	0.77	0.58
Q9JKW0	ADP-ribosylation factor like protein 6-interacting protein 1 OS=Mus musculus GN=Arl6ip1 PE=2 SV=1 - [AR6P1_MOUSE]	7.88	1	2	2	17	0.46	0.00	0.29	0.11
Q14CH7	Alanine--tRNA ligase, mitochondrial OS=Mus musculus GN=Aars2 PE=2 SV=1 - [SYAM_MOUSE]	4.90	1	4	4	5	0.05	-0.01	-0.04	-0.10
P15105	Glutamine synthetase OS=Mus musculus GN=Glul PE=1 SV=6 - [GLNA_MOUSE]	26.27	3	9	9	83	-0.02	-0.17	-0.06	-0.16
Q8BYZ7-2	Isoform 2 of Engulfment and cell motility protein 3 OS=Mus musculus GN=Elmo3 - [ELMO3_MOUSE]	15.49	2	7	7	12	0.51	0.42	0.10	0.19

Q3TVI8	Pre-B-cell leukemia transcription factor-interacting protein 1 OS=Mus musculus GN=Pbxip1 PE=1 SV=2 - [PBIP1_MOUSE]	22.15	4	9	9	28	0.14	0.03	0.13	0.14
Q9DBD5	Proline-, glutamic acid- and leucine-rich protein 1 OS=Mus musculus GN=Pelp1 PE=1 SV=2 - [PELP1_MOUSE]	4.99	1	3	3	18	0.08	0.22	0.16	0.23
Q91VK1	Basic leucine zipper and W2 domain-containing protein 2 OS=Mus musculus GN=Bzw2 PE=1 SV=1 - [BZW2_MOUSE]	25.06	1	10	11	38	0.41	0.33	0.39	0.34
Q9Z0H3-2	Isoform B of SWI/SNF-related matrix-associated actin-dependent regulator of chromatin subfamily B member 1 OS=Mus musculus GN=Smarcb1 - [SNF5_MOUSE]	8.24	4	3	3	6	0.35	0.26	0.28	0.18
P26040	Ezrin OS=Mus musculus GN=Ezr PE=1 SV=3 - [EZRI_MOUSE]	43.00	1	19	32	419	0.28	0.34	0.27	0.28
Q8BUH8	Sentrin-specific protease 7 OS=Mus musculus GN=Senp7 PE=2 SV=1 - [SENP7_MOUSE]	7.52	3	5	5	5	-0.07	-1.01	0.23	-0.61
O08600	Endonuclease G, mitochondrial OS=Mus musculus GN=Endog PE=2 SV=1 - [NUCG_MOUSE]	28.91	1	6	6	34	-0.50	-0.27	-0.36	0.01
Q4PZA2-3	Isoform C of Endothelin converting enzyme 1 OS=Mus musculus GN=Ece1 - [ECE1_MOUSE]	11.02	4	7	7	16	-0.10	-0.35	-0.11	-0.19
K7N6T3	Protein Gm3159 OS=Mus musculus GN=Gm3159 PE=4 SV=1 - [K7N6T3_MOUSE]	16.18	7	3	4	5	0.56	0.71	0.33	0.48
Q8BGC4	Zinc-binding alcohol dehydrogenase domain-containing protein 2 OS=Mus musculus GN=Zadh2 PE=2 SV=1 - [ZADH2_MOUSE]	14.59	1	5	5	16	-0.14	-0.32	-0.54	-0.20
Q9D6Y9	1,4-alpha-glucan-branching enzyme OS=Mus musculus GN=Gbe1 PE=2 SV=1 - [GLGB_MOUSE]	17.66	3	11	11	46	-0.27	-0.52	-0.10	-0.34
Q149F1	RNA pseudouridylate synthase domain-containing protein 2 OS=Mus musculus GN=Rpusd2 PE=2 SV=2 - [RUSD2_MOUSE]	4.52	1	1	2	5	0.03	0.15	0.16	0.27
P61082	NEDD8-conjugating enzyme Ubc12 OS=Mus musculus GN=Ube2m PE=2 SV=1 - [UBC12_MOUSE]	26.78	5	5	5	29	0.28	0.30	0.24	0.28
Q8R3G9	Tetraspanin-8 OS=Mus musculus GN=Tspan8 PE=2 SV=1 - [TSN8_MOUSE]	22.98	1	5	5	57	0.23	0.22	0.24	0.29

Q8BKR5	Protein phosphatase 1 regulatory subunit 37 OS=Mus musculus GN=Ppp1r37 PE=2 SV=1 - [PPR37_MOUSE]	2.53	1	1	1	3	0.25	0.68	0.16	0.59
P32067	Lupus La protein homolog OS=Mus musculus GN=Ssb PE=2 SV=1 - [LA_MOUSE]	38.31	4	19	20	105	0.36	0.46	0.30	0.35
Q3UV17	Keratin, type II cytoskeletal 2 oral OS=Mus musculus GN=Krt76 PE=2 SV=1 [K22O_MOUSE]	7.74	2	1	6	211	1.59	1.60	0.09	0.09
Q99LS3	Phosphoserine phosphatase OS=Mus musculus GN=Psph PE=1 SV=1 - [SERB_MOUSE]	8.00	3	2	2	4	0.24	0.50	0.48	0.35
A2CG22	Butyrophilin-like 6 OS=Mus musculus GN=Btnl6 PE=4 SV=1 - [A2CG22_MOUSE]	5.57	1	1	1	1	-0.38	-0.25	0.01	0.14
Q91YE7-2	Isoform 2 of RNA-binding protein 5 OS=Mus musculus GN=Rbm5 - [RBM5_MOUSE]	5.90	2	3	4	13	0.03	0.26	0.36	0.26
E9PVA6	ARF GTPase-activating protein GIT2 OS=Mus musculus GN=Git2 PE=2 SV=1 - [E9PVA6_MOUSE]	7.51	10	4	5	8	0.01	0.29	0.11	0.42
Q9D309	Protein FAM3B OS=Mus musculus GN=Fam3b PE=1 SV=1 - [FAM3B_MOUSE]	43.83	1	9	9	56	0.11	0.15	0.01	0.05
Q6PDL0	Cytoplasmic dynein 1 light intermediate chain 2 OS=Mus musculus GN=Dync1li2 PE=1 SV=2 - [DC1L2_MOUSE]	28.46	1	10	11	52	0.11	0.08	0.08	0.07
Q8VHY0	Chondroitin sulfate proteoglycan 4 OS=Mus musculus GN=Cspg4 PE=1 SV=3 - [CSPG4_MOUSE]	24.71	3	40	40	185	-0.28	-0.36	-0.27	-0.39
G3X9C8	LIX1-like protein OS=Mus musculus GN=Lix1l PE=4 SV=1 - [G3X9C8_MOUSE]	2.97	2	1	1	4	0.19	-0.15	0.23	-0.11
Q5SW46	Protein Lpo OS=Mus musculus GN=Lpo PE=2 SV=1 - [Q5SW46_MOUSE]	8.31	1	3	4	6	-0.63	-0.74	-0.33	-0.33
G3UZX2	Ubiquitin-conjugating enzyme E2 G2 (Fragment) OS=Mus musculus GN=Ube2g2 PE=2 SV=1 - [G3UZX2_MOUSE]	64.79	2	3	3	15	0.07	0.01	0.06	0.03
Q99JI1	Musculoskeletal embryonic nuclear protein 1 OS=Mus musculus GN=Mustn1 PE=2 SV=1 - [MSTN1_MOUSE]	43.90	1	3	3	30	0.00	0.09	-0.05	0.07
Q91WG5-2	Isoform B of 5'-AMP-activated protein kinase subunit gamma-2 OS=Mus musculus GN=Prkag2 - [AAKG2_MOUSE]	8.28	4	3	3	3	-0.28	-1.06	-0.08	-0.54

D3Z7G7	Protein Zfp882 (Fragment) OS=Mus musculus GN=Zfp882 PE=2 SV=1 - [D3Z7G7_MOUSE]	10.40	1	1	1	1	-0.33	-0.43	-0.32	-0.42
Q3UZP4	Small VCP/p97-interacting protein OS=Mus musculus GN=Svip PE=2 SV=1 - [SVIP_MOUSE]	32.47	1	1	2	6	-0.23	-0.32	-0.20	-0.29
P27601	Guanine nucleotide-binding protein subunit alpha-13 OS=Mus musculus GN=Gna13 PE=1 SV=1 - [GNA13_MOUSE]	38.99	5	10	13	79	0.05	0.05	0.06	0.00
Q9D8B4	NADH dehydrogenase [ubiquinone] 1 alpha subcomplex subunit 11 OS=Mus musculus GN=Ndufa11 PE=2 SV=2 - [NDUAB_MOUSE]	26.24	2	2	2	13	-0.12	-0.03	-0.04	0.01
Q99MQ4	Asporin OS=Mus musculus GN=Aspn PE=1 SV=1 - [ASPN_MOUSE]	35.39	1	13	14	134	-0.19	-0.17	-0.14	-0.15
Q8VDP4	DBIRD complex subunit KIAA1967 homolog OS=Mus musculus PE=1 SV=2 - [K1967_MOUSE]	16.70	1	11	12	58	0.12	0.11	0.04	0.10
Q9JLI8	Squamous cell carcinoma antigen recognized by T-cells 3 OS=Mus musculus GN=Sart3 PE=2 SV=1 - [SART3_MOUSE]	3.22	1	3	3	18	0.04	0.19	0.12	0.31
E9Q1T1	Calcium/calmodulin-dependent protein kinase type II subunit beta OS=Mus musculus GN=Camk2d PE=2 SV=1 - [E9Q1T1_MOUSE]	34.90	17	11	16	142	0.22	0.31	0.19	0.24
Q8BKS9	Pumilio domain-containing protein KIAA0020 OS=Mus musculus GN=Kiaa0020 PE=2 SV=2 - [K0020_MOUSE]	11.59	1	5	5	7	0.63	0.40	0.55	0.32
Q6ZQK5	Arf-GAP with coiled-coil, ANK repeat and PH domain-containing protein 2 OS=Mus musculus GN=Acap2 PE=1 SV=2 - [ACAP2_MOUSE]	20.78	4	14	14	31	0.02	0.13	0.23	0.32
Q9CYH2	Redox-regulatory protein FAM213A OS=Mus musculus GN=Fam213a PE=1 SV=2 - [F213A_MOUSE]	19.27	8	4	4	13	-0.36	-0.25	-0.22	-0.16
Q62420	Endophilin-A1 OS=Mus musculus GN=Sh3gl2 PE=1 SV=2 - [SH3G2_MOUSE]	19.60	5	4	6	17	0.16	0.29	0.08	0.21
Q8R268	Protein Vmn1r212 OS=Mus musculus GN=Vmn1r212 PE=2 SV=1 - [Q8R268_MOUSE]	10.36	3	2	2	2	0.06	-1.99	0.02	-2.03

Q9CQ39	Mediator of RNA polymerase II transcription subunit 21 OS=Mus musculus GN=Med21 PE=1 SV=1 - [MED21_MOUSE]	22.22	1	1	2	7	-0.14	0.16	0.27	0.57
Q8BSK8-2	Isoform Alpha II of Ribosomal protein S6 kinase beta-1 OS=Mus musculus GN=Rps6kb1 - [KS6B1_MOUSE]	3.98	2	1	2	4	0.36	0.24	-0.12	-0.24
Q6F3F9	G-protein coupled receptor 126 OS=Mus musculus GN=Gpr126 PE=1 SV=1 - [GP126_MOUSE]	1.55	1	1	1	1	0.02	0.00	0.41	0.38
Q9JJY3	Sphingomyelin phosphodiesterase 3 OS=Mus musculus GN=Smpd3 PE=1 SV=1 - [NSMA2_MOUSE]	5.80	1	3	3	6	0.22	-0.09	0.26	0.03
Q9D7N9	Adipocyte plasma membrane-associated protein OS=Mus musculus GN=Apmap PE=1 SV=1 - [APMAP_MOUSE]	44.10	1	16	16	53	-0.17	-0.07	-0.20	-0.10
E9PVG8	Protein 9530053A07Rik OS=Mus musculus GN=9530053A07Rik PE=4 SV=1 - [E9PVG8_MOUSE]	4.22	1	2	10	167	-0.17	-0.13	-0.28	-0.20
Q9Z211	Peroxisomal membrane protein 11A OS=Mus musculus GN=Pex11a PE=2 SV=1 - [PX11A_MOUSE]	4.07	1	1	1	1	-0.60	-0.66	-0.14	-0.21
Q9Z1Q2	Abhydrolase domain-containing protein 16A OS=Mus musculus GN=Abhd16a PE=1 SV=3 - [ABHGA_MOUSE]	13.26	3	5	5	12	-0.10	-0.24	-0.07	-0.28
P15626	Glutathione S-transferase Mu 2 OS=Mus musculus GN=Gstm2 PE=1 SV=2 - [GSTM2_MOUSE]	75.69	4	12	21	379	0.27	0.35	0.26	0.28
Q8K1Z0	Ubiquinone biosynthesis protein COQ9, mitochondrial OS=Mus musculus GN=Coq9 PE=1 SV=1 - [COQ9_MOUSE]	22.36	2	5	5	24	-0.32	-0.27	-0.10	-0.11
O09012-2	Isoform 2 of Peroxisomal targeting signal 1 receptor OS=Mus musculus GN=Pex5 - [PEX5_MOUSE]	18.11	3	4	4	13	-0.22	0.04	0.34	0.24
Q8BLU2	Protein-lysine methyltransferase METTL21C OS=Mus musculus GN=Mettl21c PE=2 SV=1 - [MT21C_MOUSE]	8.06	1	1	1	7	0.33	0.51	0.15	0.34
Q9Z1M2	Interferon-g induced GTPase OS=Mus musculus GN=Irgm2 PE=2 SV=1 - [Q9Z1M2_MOUSE]	18.48	1	4	6	17	0.43	0.35	0.55	0.46
Q8C460-2	Isoform 2 of ERI1 exoribonuclease 3 OS=Mus musculus GN=Eri3 - [ERI3_MOUSE]	5.07	2	2	2	4	-0.11	-0.09	0.13	0.15

Q78ZM0	Sorting nexin 3 OS=Mus musculus GN=Snx3 PE=2 SV=1 - [Q78ZM0_MOUSE]	56.17	4	7	8	39	0.18	0.39	0.20	0.37
Q8BYI6-2	Isoform 2 of Lysophosphatidylcholine acyltransferase 2 OS=Mus musculus GN=Lpcat2 - [PCAT2_MOUSE]	7.54	3	3	3	5	-0.02	-0.24	0.14	-0.08
Q9DBX3	Sushi domain-containing protein 2 OS=Mus musculus GN=Susd2 PE=1 SV=1 [SUSD2_MOUSE]	18.41	3	11	11	42	-0.33	-0.45	-0.33	-0.34
Q32M07	Adenylate kinase 8 OS=Mus musculus GN=Ak8 PE=2 SV=1 - [KAD8_MOUSE]	1.46	1	1	1	1	0.09	-0.10	0.24	0.05
Q99PP9-3	Isoform 3 of Tripartite motif-containing protein 16 OS=Mus musculus GN=Trim16 - [TRI16_MOUSE]	14.29	5	5	5	8	-0.07	-0.43	0.15	-0.24
A2AFQ9	Gem-associated protein 5 OS=Mus musculus GN=Gemin5 PE=2 SV=1 - [A2AFQ9_MOUSE]	2.13	4	3	3	5	-0.52	-0.42	-0.01	0.05
Q91YM4-2	Isoform 2 of Protein TBRG4 OS=Mus musculus GN=Tbrg4 - [TBRG4_MOUSE]	9.00	5	4	4	22	-0.06	0.02	0.00	-0.04
E9PV66	Protein Myo18b OS=Mus musculus GN=Myo18b PE=4 SV=1 - [E9PV66_MOUSE]	0.54	1	1	1	1	0.18	-0.10	0.08	-0.19
E9PYL9	Uncharacterized protein OS=Mus musculus GN=Gm10036 PE=3 SV=1 - [E9PYL9_MOUSE]	32.58	6	6	6	60	0.12	0.15	0.16	0.22
E9QKT1	Ubiquitin-protein ligase E3A OS=Mus musculus GN=Ube3a PE=2 SV=1 - [E9QKT1_MOUSE]	15.75	2	11	11	24	0.28	0.35	0.08	0.25
Q5UE59	Kinesin light chain 1 OS=Mus musculus GN=Klc1 PE=2 SV=1 - [Q5UE59_MOUSE]	26.01	6	9	13	37	0.06	0.02	0.10	0.06
P25444	40S ribosomal protein S2 OS=Mus musculus GN=Rps2 PE=1 SV=3 - [RS2_MOUSE]	56.66	11	17	17	223	0.41	0.46	0.30	0.42
Q9QY33	Tetraspanin-3 OS=Mus musculus GN=Tspan3 PE=1 SV=1 - [TSN3_MOUSE]	2.77	1	1	1	1	0.07	0.11	0.11	0.16
E9PV03	Protein Nbeal1 OS=Mus musculus GN=Nbeal1 PE=4 SV=1 - [E9PV03_MOUSE]	6.51	2	7	7	9	0.42	0.44	-0.26	-0.13
Q3TXS7	26S proteasome non-ATPase regulatory subunit 1 OS=Mus musculus GN=Psmd1 PE=1 SV=1 - [PSMD1_MOUSE]	33.79	3	27	27	161	0.22	0.24	0.16	0.23
P14115	60S ribosomal protein L27a OS=Mus musculus GN=Rpl27a PE=2 SV=5 - [RL27A_MOUSE]	30.41	1	7	7	82	0.06	-0.09	0.24	0.09

Q3TMP1	General transcription factor IIIC, polypeptide 3 OS=Mus musculus GN=Gtf3c3 PE=2 SV=1 - [Q3TMP1_MOUSE]	2.61	1	1	2	5	-0.19	0.14	-0.10	-0.01
F8WHP8	ATP synthase subunit f, mitochondrial OS=Mus musculus GN=Atp5j2 PE=2 SV=1 - [F8WHP8_MOUSE]	15.79	2	1	1	18	-0.09	0.12	-0.19	0.00
P82347	Delta-sarcoglycan OS=Mus musculus GN=Sgcd PE=1 SV=1 - [SGCD_MOUSE]	29.76	2	7	7	42	-0.31	-0.45	-0.31	-0.17
Q9CQI7	U2 small nuclear ribonucleoprotein B" OS=Mus musculus GN=Snrpb2 PE=2 SV=1 - [RU2B_MOUSE]	27.56	2	4	6	31	-0.05	0.00	0.09	0.05
Q9ERI2	Ras-related protein Rab-27A OS=Mus musculus GN=Rab27a PE=1 SV=1 - [RB27A_MOUSE]	10.86	1	2	2	4	-0.07	-0.31	0.48	0.07
Q80YA8	Protein crumbs homolog 2 OS=Mus musculus GN=Crb2 PE=2 SV=3 - [CRUM2_MOUSE]	1.25	1	1	1	1	0.30	-0.68	0.60	-0.38
P59509	A disintegrin and metalloproteinase with thrombospondin motifs 19 OS=Mus musculus GN=Adamts19 PE=2 SV=1 - [ATS19_MOUSE]	3.80	1	2	3	4	0.06	-0.57	0.31	-0.33
E9PXJ0	Neuronal tyrosine-phosphorylated phosphoinositide-3-kinase adapter 1 (Fragment) OS=Mus musculus GN=Nyap1 PE=2 SV=1 - [E9PXJ0_MOUSE]	1.65	4	2	2	4	-0.19	-0.29	-0.14	-0.24
O70252	Heme oxygenase 2 OS=Mus musculus GN=Hmox2 PE=2 SV=1 - [HMOX2_MOUSE]	40.00	4	9	9	53	0.24	0.17	0.27	0.11
P97454	Mothers against decapentaplegic homolog 5 OS=Mus musculus GN=Smad5 PE=1 SV=2 - [SMAD5_MOUSE]	7.10	1	1	3	9	0.40	1.03	0.30	0.93
Q9JI91	Alpha-actinin-2 OS=Mus musculus GN=Actn2 PE=1 SV=2 [ACTN2_MOUSE]	18.01	5	3	17	351	0.14	0.26	-0.01	0.05
Q91YL2	E3 ubiquitin-protein ligase RNF126 OS=Mus musculus GN=Rnf126 PE=1 SV=1 - [RN126_MOUSE]	7.67	1	1	1	3	-0.71	-0.10	-0.96	-0.45
Q9D358	Low molecular weight phosphotyrosine protein phosphatase OS=Mus musculus GN=Acp1 PE=1 SV=3 - [PPAC_MOUSE]	39.87	1	1	5	59	-0.67	-0.13	-0.19	-0.05
Q6DI94	DNA excision repair protein ERCC-6-like 2 OS=Mus musculus GN=Ercc6l2 PE=2 SV=1 - [ER6L2_MOUSE]	4.96	3	2	2	3	0.30	0.73	0.20	0.62

Q99MU3-5	Isoform 5 of Double-stranded RNA-specific adenosine deaminase OS=Mus musculus GN=Adar - [DSRAD_MOUSE]	6.02	5	4	4	11	0.05	-0.18	0.29	0.10
Q8BHF7	CDP-diacylglycerol--glycerol-3-phosphate 3-phosphatidyltransferase, mitochondrial OS=Mus musculus GN=Pgs1 PE=2 SV=1 - [PGPS1_MOUSE]	3.98	3	2	2	5	-0.03	0.04	0.18	0.25
D3Z0V2	Rho guanine nucleotide exchange factor 7 OS=Mus musculus GN=Arhgef7 PE=2 SV=1 - [D3Z0V2_MOUSE]	20.00	10	9	10	23	0.31	0.33	0.25	0.31
Q9Z2I8-2	Isoform 2 of Succinyl-CoA ligase [GDP-forming] subunit beta, mitochondrial OS=Mus musculus GN=Suclg2 - [SUCB2_MOUSE]	63.80	2	23	24	396	-0.14	-0.05	-0.13	-0.04
G5E829	MCG13663, isoform CRA_a OS=Mus musculus GN=Atp2b1 PE=3 SV=1 - [G5E829_MOUSE]	23.77	1	12	26	165	-0.37	-0.40	-0.33	-0.45
Q2HXL6	ER degradation-enhancing alpha-mannosidase-like protein 3 OS=Mus musculus GN=Edem3 PE=1 SV=2 - [EDEM3_MOUSE]	3.33	1	3	3	4	0.08	0.18	0.07	0.32
B2RXQ9	Sorbin and SH3 domain containing protein 2 OS=Mus musculus GN=Sorbs2 PE=2 SV=1 - [B2RXQ9_MOUSE]	50.31	2	1	27	200	0.07	0.00	-0.02	-0.09
F8WIV1	Zinc finger B-box domain-containing protein 1 OS=Mus musculus GN=Zbbx PE=2 SV=1 - [F8WIV1_MOUSE]	8.86	13	3	3	4	-0.72	-1.44	-0.14	-0.86
Q8VFI7	Olfactory receptor 866 OS=Mus musculus GN=Olfr866 PE=2 SV=1 - [Q8VFI7_MOUSE]	5.45	1	1	2	2	0.15	0.33	0.12	0.30
Q8BJ71	Nuclear pore complex protein Nup93 OS=Mus musculus GN=Nup93 PE=2 SV=1 - [NUP93_MOUSE]	23.32	2	19	19	59	0.26	0.18	0.24	0.11
Q9D289	Trafficking protein particle complex subunit 6B OS=Mus musculus GN=Trappc6b PE=2 SV=1 - [TPC6B_MOUSE]	18.99	1	3	3	5	0.15	0.22	0.22	0.22
E0CYM1	Filamin A-interacting protein 1-like OS=Mus musculus GN=Filip1l PE=2 SV=1 - [E0CYM1_MOUSE]	27.10	4	22	23	76	0.04	0.12	0.05	0.20
E9QAD4	Coiled-coil domain-containing protein 93 OS=Mus musculus GN=Ccdc93 PE=2 SV=1 - [E9QAD4_MOUSE]	14.65	2	8	8	17	0.18	0.23	0.33	0.10

Q9D6E4	Tetratricopeptide repeat protein 9B OS=Mus musculus GN=Ttc9b PE=2 SV=1 - [TTC9B_MOUSE]	10.88	1	2	4	6	0.19	0.25	0.22	0.27
Q8C561-2	Isoform 2 of LMBR1 domain-containing protein 2 OS=Mus musculus GN=Lmbrd2 - [LMBD2_MOUSE]	5.70	3	2	2	3	-0.58	0.90	-0.16	1.32
Q9R233-2	Isoform Short of Tapasin OS=Mus musculus GN=Tapbp - [TPSN_MOUSE]	18.62	5	7	7	43	0.28	0.22	0.32	0.24
Q8R3Y8-2	Isoform 2 of Interferon regulatory factor 2-binding protein 1 OS=Mus musculus GN=Irf2bp1 - [I2BP1_MOUSE]	4.78	2	3	3	7	0.20	0.14	0.05	0.01
B1ARH1	Diphthamide biosynthesis protein 1 (Fragment) OS=Mus musculus GN=Dph1 PE=2 SV=1 - [B1ARH1_MOUSE]	2.54	2	1	1	1	0.30	0.65	0.44	0.79
P51863	V-type proton ATPase subunit d 1 OS=Mus musculus GN=Atp6v0d1 PE=1 SV=2 - [VA0D1_MOUSE]	21.65	1	6	6	40	0.12	-0.04	0.07	-0.01
Q3UHK1	Proton myo-inositol cotransporter OS=Mus musculus GN=Slc2a13 PE=2 SV=2 - [MYCT_MOUSE]	1.57	1	1	1	2	0.35	0.21	0.16	0.02
Q9Z0H7	B-cell lymphoma/leukemia 10 OS=Mus musculus GN=Bcl10 PE=1 SV=1 [BCL10_MOUSE]	10.73	1	2	2	3	0.21	0.26	0.37	0.42
A2AC80	Metalloendopeptidase homolog PEX (Fragment) OS=Mus musculus GN=Phex PE=2 SV=1 - [A2AC80_MOUSE]	2.55	2	1	1	2	0.48	1.27	0.48	1.27
Q8C850	Scavenger receptor class A member 3 OS=Mus musculus GN=Scara3 PE=2 SV=1 - [SCAR3_MOUSE]	2.48	1	1	1	1	0.06	0.03	-0.13	-0.17
O08529	Calpain-2 catalytic subunit OS=Mus musculus GN=Capn2 PE=2 SV=4 - [CAN2_MOUSE]	39.00	2	23	23	174	0.13	0.02	0.17	0.08
Q8R0F6	Integrin-linked kinase-associated serine/threonine phosphatase 2C OS=Mus musculus GN=Ilkap PE=2 SV=1 - [ILKAP_MOUSE]	11.99	2	5	5	13	-0.09	-0.14	0.06	0.01
O54916-4	Isoform 4 of RalBP1-associated Eps domain-containing protein 1 OS=Mus musculus GN=Reps1 - [REPS1_MOUSE]	15.07	9	6	8	20	-0.54	-0.48	-0.47	-0.44
D3YTP0	Metalloreductase STEAP3 (Fragment) OS=Mus musculus GN=Steap3 PE=2 SV=1 - [D3YTP0_MOUSE]	13.37	4	6	6	14	0.20	0.33	0.31	0.40

G3X972	Protein Sec24c OS=Mus musculus GN=Sec24c PE=4 SV=1 - [G3X972_MOUSE]	21.17	1	18	19	90	0.02	-0.10	0.00	-0.13
Q9WV91	Prostaglandin F2 receptor negative regulator OS=Mus musculus GN=Ptgfrn PE=1 SV=2 - [FPRP_MOUSE]	17.75	1	14	14	48	-0.10	-0.05	-0.10	-0.18
O35638	Cohesin subunit SA-2 OS=Mus musculus GN=Stag2 PE=1 SV=3 - [STAG2_MOUSE]	10.72	5	10	10	19	0.30	0.14	0.33	-0.05
Q5U458	DnaJ homolog subfamily C member 11 OS=Mus musculus GN=Dnajc11 PE=2 SV=2 - [DJC11_MOUSE]	10.73	3	5	5	14	-0.06	-0.11	0.02	-0.19
Q8BG51	Mitochondrial Rho GTPase 1 OS=Mus musculus GN=Rhot1 PE=2 SV=1 - [MIRO1_MOUSE]	3.17	5	2	2	7	-0.07	0.02	-0.02	-0.01
P27659	60S ribosomal protein L3 OS=Mus musculus GN=Rpl3 PE=2 SV=3 - [RL3_MOUSE]	41.94	3	20	20	220	0.33	0.33	0.24	0.26
Q9EPX2	Papilin OS=Mus musculus GN=Papln PE=2 SV=2 - [PPN_MOUSE]	2.19	2	2	2	7	-0.72	-0.91	-0.79	-0.99
B1AYL1	Protein Scn7a OS=Mus musculus GN=Scn7a PE=2 SV=1 - [B1AYL1_MOUSE]	10.11	1	13	14	36	-0.26	-0.17	-0.14	-0.10
Q9CQA1	Trafficking protein particle complex subunit 5 OS=Mus musculus GN=Trappc5 PE=1 SV=1 - [TPPC5_MOUSE]	16.49	1	3	4	13	-0.21	-0.37	0.10	-0.28
Q6XPS7	L-threonine aldolase OS=Mus musculus GN=Tha1 PE=2 SV=1 - [Q6XPS7_MOUSE]	18.75	1	6	6	20	0.13	0.07	0.18	-0.04
G3UYB5	N6-adenosine-methyltransferase 70 kDa subunit OS=Mus musculus GN=Mettl3 PE=4 SV=1 - [G3UYB5_MOUSE]	7.19	3	1	1	2	-0.13	-0.32	0.01	-0.18
Q8R332-4	Isoform 4 of Nucleoporin p58/p45 OS=Mus musculus GN=Nupl1 - [NUPL1_MOUSE]	12.03	4	5	5	8	0.65	0.42	0.57	0.72
B1AUY7	N-alpha-acetyltransferase 10 OS=Mus musculus GN=Naa10 PE=2 SV=1 - [B1AUY7_MOUSE]	22.32	6	3	4	5	-0.58	-1.16	-0.39	-0.97
Q91V35	Protein tyrosin phosphatase receptor type alpha OS=Mus musculus GN=Ptpra PE=2 SV=1 - [Q91V35_MOUSE]	8.07	6	5	5	12	-0.29	-0.05	-0.18	0.01
F6SQH7	Polymerase delta-interacting protein 2 (Fragment) OS=Mus musculus GN=Poldip2 PE=4 SV=1 - [F6SQH7_MOUSE]	17.96	2	3	3	12	0.28	0.39	0.23	0.18

Q8BFU3-5	Isoform 5 of RING finger protein 214 OS=Mus musculus GN=Rnf214 - [RN214_MOUSE]	7.99	5	3	4	9	0.27	0.29	0.37	0.39
Q9CQH3	NADH dehydrogenase [ubiquinone] 1 beta subcomplex subunit 5, mitochondrial OS=Mus musculus GN=Ndufb5 PE=1 SV=1 - [NDUB5_MOUSE]	28.04	5	5	5	34	0.04	0.05	0.04	0.07
P35123	Ubiquitin carboxyl-terminal hydrolase 4 OS=Mus musculus GN=Usp4 PE=1 SV=3 - [UBP4_MOUSE]	16.42	1	13	14	45	0.21	0.26	0.21	0.33
F8VPX2	Protein Fbxw9 OS=Mus musculus GN=Fbxw9 PE=2 SV=1 - [F8VPX2_MOUSE]	4.59	1	2	2	3	0.03	0.09	0.04	0.11
Q8BG40	Katanin p80 WD40 repeat-containing subunit B1 OS=Mus musculus GN=Katnb1 PE=1 SV=1 - [KTNB1_MOUSE]	2.28	1	1	1	1	0.30	-0.56	-0.13	-0.99
P14431	H-2 class I histocompatibility antigen, Q9 alpha chain (Fragment) OS=Mus musculus GN=H2-Q9 PE=3 SV=1 - [HA19_MOUSE]	22.50	6	1	4	49	-0.92	-1.36	-0.50	-0.94
D3YWN7	Ensconsin OS=Mus musculus GN=Map7 PE=4 SV=2 - [D3YWN7_MOUSE]	7.32	4	4	5	8	-0.45	-0.11	-0.31	-0.05
O35153	BET1-like protein OS=Mus musculus GN=Bet1l PE=3 SV=1 - [BET1L_MOUSE]	18.92	1	2	2	3	-0.05	-1.23	0.17	-1.08
E9Q769	Junctional adhesion molecule C OS=Mus musculus GN=Jam3 PE=2 SV=1 - [E9Q769_MOUSE]	10.95	2	2	2	5	-0.28	-0.45	-0.37	-0.45
Q91VH2	Sorting nexin-9 OS=Mus musculus GN=Snx9 PE=1 SV=1 - [SNX9_MOUSE]	20.50	1	11	11	63	0.15	0.02	0.21	0.16
Q921G7	Electron transfer flavoprotein-ubiquinone oxidoreductase, mitochondrial OS=Mus musculus GN=Etfdh PE=1 SV=1 - [ETFD_MOUSE]	40.42	2	22	22	166	-0.13	-0.14	-0.10	-0.10
B1AXC8	Coiled-coil-helix-coiled-coil-helix domain-containing protein 7 (Fragment) OS=Mus musculus GN=Chchd7 PE=2 SV=1 - [B1AXC8_MOUSE]	41.77	5	2	2	16	-0.17	0.44	-0.10	0.04
Q9CX80	Cytoglobin OS=Mus musculus GN=Cygb PE=2 SV=1 - [CYGB_MOUSE]	20.53	1	4	4	7	0.18	0.13	0.42	0.27
Q8C1E7	Transmembrane protein 120A OS=Mus musculus GN=Tmem120a PE=2 SV=1 - [T120A_MOUSE]	4.96	1	1	1	4	-0.53	-0.43	-0.24	-0.15

Q9Z277	Tyrosine-protein kinase BAZ1B OS=Mus musculus GN=Baz1b PE=1 SV=2 - [BAZ1B_MOUSE]	7.84	2	12	12	19	0.07	0.20	0.16	0.28
E9PY51	Protein Trappc8 OS=Mus musculus GN=Trappc8 PE=2 SV=1 - [E9PY51_MOUSE]	8.52	2	7	7	20	-0.25	0.17	0.08	0.27
Q9QWK5	Baculoviral IAP repeat-containing protein 1a OS=Mus musculus GN=Naip1 PE=2 SV=3 [BIR1A_MOUSE]	7.84	5	6	10	22	0.14	0.19	0.41	0.46
E9QAH1	Protein Golgb1 OS=Mus musculus GN=Golgb1 PE=2 SV=1 - [E9QAH1_MOUSE]	28.31	4	73	76	249	0.00	0.03	0.01	0.06
F8WI65	Adenosylhomocysteinase OS=Mus musculus GN=Ahcyl2 PE=2 SV=1 - [F8WI65_MOUSE]	41.14	8	1	20	223	-0.02	0.10	0.00	0.12
Q9CRB9	Coiled-coil-helix-coiled-coil-helix domain-containing protein 3, mitochondrial OS=Mus musculus GN=Chchd3 PE=1 SV=1 - [CHCH3_MOUSE]	39.21	4	11	11	50	-0.16	0.03	-0.27	-0.10
Q3THE2	Myosin regulatory light chain 12B OS=Mus musculus GN=Myl12b PE=1 SV=2 - [ML12B_MOUSE]	66.28	2	1	13	1109	-0.07	-0.39	0.03	-0.15
D3Z7G3	Protein 2010107G23Rik (Fragment) OS=Mus musculus GN=2010107G23Rik PE=2 SV=1 - [D3Z7G3_MOUSE]	11.11	4	1	1	2	-0.20	-0.19	-0.18	-0.17
Q8VHF2-2	Isoform 2 of Cadherin-related family member 5 OS=Mus musculus GN=Cdhr5 - [CDHR5_MOUSE]	14.95	3	7	7	26	0.08	0.00	0.15	-0.03
E9PUE7	Active breakpoint cluster region-related protein OS=Mus musculus GN=Abr PE=2 SV=1 - [E9PUE7_MOUSE]	8.00	8	5	6	23	0.13	0.23	0.00	0.00
P28653	Biglycan OS=Mus musculus GN=Bgn PE=2 SV=1 - [PGS1_MOUSE]	35.23	1	11	12	385	-0.10	-0.14	-0.14	-0.12
D3YWC9	MTSS1-like protein OS=Mus musculus GN=Mtss1l PE=2 SV=1 - [D3YWC9_MOUSE]	12.25	4	3	3	4	-0.70	-0.71	-0.36	-0.37
Q3TXN1	Prostaglandin reductase 2 OS=Mus musculus GN=Ptgr2 PE=2 SV=1 - [Q3TXN1_MOUSE]	50.16	4	10	10	57	-0.14	-0.30	-0.02	-0.23
Q9D832	DnaJ homolog subfamily B member 4 OS=Mus musculus GN=Dnajb4 PE=2 SV=1 - [DNJB4_MOUSE]	14.54	1	4	5	31	0.26	0.33	0.17	0.22
Q561M1	Acp1 protein OS=Mus musculus GN=Acp1 PE=2 SV=1 - [Q561M1_MOUSE]	43.67	2	1	5	53	-0.05	-0.04	0.19	0.20

G5E904	MCG19189 OS=Mus musculus GN=Gm4794 PE=4 SV=1 - [G5E904_MOUSE]	7.17	1	1	2	3	-0.12	-0.12	-0.53	-0.54
P16460	Argininosuccinate synthase OS=Mus musculus GN=Ass1 PE=1 SV=1 - [ASSY_MOUSE]	40.05	2	15	15	105	-0.15	-0.03	-0.14	0.01
P45700	Mannosyl-oligosaccharide 1,2-alpha-mannosidase IA OS=Mus musculus GN=Man1a1 PE=1 SV=1 - [MA1A1_MOUSE]	5.80	2	3	3	8	0.01	0.03	0.02	0.08
P62307	Small nuclear ribonucleoprotein F OS=Mus musculus GN=Snrpf PE=2 SV=1 - [RUXF_MOUSE]	16.28	1	2	2	3	-0.56	-0.62	-0.27	-0.20
Q91VW5	Golgin subfamily A member 4 OS=Mus musculus GN=Golga4 PE=1 SV=2 - [GOGA4_MOUSE]	17.96	1	35	37	88	0.07	0.06	0.05	0.08
Q8BGF6	ELMO domain-containing protein 2 OS=Mus musculus GN=Elmod2 PE=2 SV=1 - [ELMD2_MOUSE]	2.39	1	1	1	1	-0.18	-0.56	0.23	-0.16
Q60767	Lymphocyte antigen 75 OS=Mus musculus GN=Ly75 PE=1 SV=2 - [LY75_MOUSE]	4.35	2	6	6	11	-0.29	-0.40	-0.12	-0.23
Q99MR6-3	Isoform C of Serrate RNA effector molecule homolog OS=Mus musculus GN=Srrt - [SRRT_MOUSE]	20.49	5	16	16	53	0.22	0.18	0.24	0.23
Q6PGC1	ATP-dependent RNA helicase Dhx29 OS=Mus musculus GN=Dhx29 PE=2 SV=1 - [DHX29_MOUSE]	5.13	1	6	6	8	0.07	0.06	0.13	-0.09
Q9D880	Mitochondrial import inner membrane translocase subunit TIM50 OS=Mus musculus GN=Timm50 PE=1 SV=1 - [TIM50_MOUSE]	24.36	1	7	7	40	-0.05	-0.38	0.07	-0.18
Q8R4G6	Alpha-1,6-mannosylglycoprotein 6 beta-N-acetylglucosaminyltransferase A OS=Mus musculus GN=Mgat5 PE=2 SV=1 - [MGT5A_MOUSE]	9.05	1	7	7	10	-0.04	-0.15	0.04	-0.32
Q9ESU6	Bromodomain-containing protein 4 OS=Mus musculus GN=Brd4 PE=1 SV=2 - [BRD4_MOUSE]	6.00	6	5	7	22	-0.07	-0.26	0.22	0.20
Q61029	Lamina-associated polypeptide 2, isoforms beta/delta/epsilon/gamma OS=Mus musculus GN=Tmpo PE=1 SV=4 [LAP2B_MOUSE]	52.43	5	10	18	371	0.05	0.16	0.05	0.13

Q8BX70-3	Isoform 3 of Vacuolar protein sorting-associated protein 13C OS=Mus musculus GN=Vps13c - [VP13C_MOUSE]	7.44	3	22	22	51	-0.02	0.12	0.10	0.05
Q920Q6-3	Isoform 3 of RNA-binding protein Musashi homolog 2 OS=Mus musculus GN=Msi2 - [MSI2H_MOUSE]	13.12	5	3	3	10	0.14	0.21	0.05	-0.02
Q6P5D8	Structural maintenance of chromosomes flexible hinge domain-containing protein 1 OS=Mus musculus GN=Smchd1 PE=1 SV=2 - [SMHD1_MOUSE]	9.47	1	13	14	31	0.22	0.20	0.39	0.32
Q91YN5-3	Isoform 3 of UDP-N-acetylhexosamine pyrophosphorylase OS=Mus musculus GN=Uap1 - [UAP1_MOUSE]	40.12	8	20	20	147	-0.09	-0.10	-0.07	-0.11
Q99N57	RAF proto-oncogene serine/threonine-protein kinase OS=Mus musculus GN=Raf1 PE=1 SV=2 - [RAF1_MOUSE]	9.10	3	4	5	10	0.43	-0.18	0.35	-0.07
O70551	SRSF protein kinase 1 OS=Mus musculus GN=Srpk1 PE=1 SV=2 [SRPK1_MOUSE]	8.18	2	3	4	18	0.41	0.39	0.66	0.66
A2AUY4	Protein Baz2b OS=Mus musculus GN=Baz2b PE=4 SV=1 - [A2AUY4_MOUSE]	1.55	2	2	4	5	0.17	0.39	0.27	0.49
P14069	Protein S100-A6 OS=Mus musculus GN=S100a6 PE=1 SV=3 - [S10A6_MOUSE]	34.83	1	5	5	222	0.25	0.33	0.24	0.29
Q80TM9	Nischarin OS=Mus musculus GN=Nisch PE=1 SV=2 - [NISCH_MOUSE]	6.91	11	8	9	17	0.04	-0.39	0.04	-0.30
A2ALP5	FYVE, RhoGEF and PH domain-containing protein 1 OS=Mus musculus GN=Fgd1 PE=2 SV=1 - [A2ALP5_MOUSE]	0.76	2	1	1	5	0.92	1.22	0.80	1.09
Q9JI44	DNA methyltransferase 1-associated protein 1 OS=Mus musculus GN=Dmap1 PE=1 SV=1 - [DMAP1_MOUSE]	8.33	2	2	2	4	-0.11	-0.19	-0.02	-0.11
E9Q9T1	Leucine-rich repeat flightless-interacting protein 1 OS=Mus musculus GN=Lrrfip1 PE=4 SV=2 - [E9Q9T1_MOUSE]	20.86	3	1	11	75	-0.21	-0.08	-0.29	-0.17
Q9CQP0	39S ribosomal protein L33, mitochondrial OS=Mus musculus GN=Mrpl33 PE=2 SV=1 - [RM33_MOUSE]	40.00	2	2	2	6	0.47	0.60	0.46	0.59
Q921E6	Polycomb protein EED OS=Mus musculus GN=Eed PE=1 SV=1 - [EED_MOUSE]	7.48	3	3	3	4	0.32	0.52	0.56	0.77

Q8JZX9	Cdc42 effector protein 2 OS=Mus musculus GN=Cdc42ep2 PE=1 SV=1 - [BORG1_MOUSE]	11.21	1	1	1	2	-0.61	-1.06	-0.08	-0.53
E9QAE3	Protein Btaf1 OS=Mus musculus GN=Btaf1 PE=2 SV=1 - [E9QAE3_MOUSE]	2.33	1	3	3	16	-0.12	-0.16	-0.52	-0.24
O35435	Dihydroorotate dehydrogenase (quinone), mitochondrial OS=Mus musculus GN=Dhodh PE=2 SV=2 - [PYRD_MOUSE]	25.82	4	8	8	17	0.25	0.21	0.41	0.15
P70303	CTP synthase 2 OS=Mus musculus GN=Ctps2 PE=1 SV=1 [PYRG2_MOUSE]	10.92	4	4	4	16	-0.51	0.10	-0.59	-0.03
Q9DBR7	Protein phosphatase 1 regulatory subunit 12A OS=Mus musculus GN=Ppp1r12a PE=1 SV=2 - [MYPT1_MOUSE]	42.47	1	3	47	394	0.20	0.38	0.22	0.35
F2Z452	Uncharacterized protein OS=Mus musculus GN=Atp5l-ps1 PE=4 SV=1 - [F2Z452_MOUSE]	25.24	4	2	3	36	0.04	0.35	-0.01	0.28
Q9QXY6	EH domain-containing protein 3 OS=Mus musculus GN=Ehd3 PE=1 SV=2 - [EHD3_MOUSE]	22.43	1	3	11	83	-1.09	-0.16	-0.93	0.02
Q99LC3	NADH dehydrogenase [ubiquinone] 1 alpha subcomplex subunit 10, mitochondrial OS=Mus musculus GN=Ndufa10 PE=1 SV=1 - [NDUAA_MOUSE]	44.51	1	14	14	103	-0.16	-0.05	-0.15	-0.04
O55142	60S ribosomal protein L35a OS=Mus musculus GN=Rpl35a PE=2 SV=2 - [RL35A_MOUSE]	50.91	1	8	8	24	0.18	0.22	0.15	0.20
P54071	Isocitrate dehydrogenase [NADP], mitochondrial OS=Mus musculus GN=Idh2 PE=1 SV=3 - [IDHP_MOUSE]	44.25	2	22	23	353	-0.10	-0.02	-0.11	-0.06
P67778	Prohibitin OS=Mus musculus GN=Phb PE=1 SV=1 - [PHB_MOUSE]	48.16	2	13	13	206	0.05	0.04	0.04	0.04
O08804	NK13 OS=Mus musculus GN=Serpinb6b PE=2 SV=2 - [O08804_MOUSE]	7.43	2	1	3	12	0.29	0.40	-0.12	-0.02
O88325	Alpha-N-acetylglucosaminidase OS=Mus musculus GN=Naglu PE=2 SV=1 [O88325_MOUSE]	13.67	1	10	10	29	-0.44	-0.42	-0.50	-0.40
Q149L6-3	Isoform 3 of DnaJ homolog subfamily B member 14 OS=Mus musculus GN=Dnajb14 [DJB14_MOUSE]	6.96	3	1	1	4	0.44	0.40	0.25	0.20

Q8BFZ3	Beta-actin-like protein 2 OS=Mus musculus GN=Actbl2 PE=1 SV=1 - [ACTBL_MOUSE]	48.40	1	7	19	2766	0.18	-0.16	0.17	-0.25
P70206	Plexin-A1 OS=Mus musculus GN=Plxna1 PE=1 SV=1 - [PLXA1_MOUSE]	0.84	3	2	2	2	0.02	0.22	-0.33	-0.14
Q99PM9	Uridine-cytidine kinase 2 OS=Mus musculus GN=Uck2 PE=1 SV=1 - [UCK2_MOUSE]	14.56	1	2	3	8	0.20	0.16	0.25	0.21
F7C090	Beclin-1 (Fragment) OS=Mus musculus GN=Becn1 PE=2 SV=1 - [F7C090_MOUSE]	5.95	3	1	1	2	-0.22	0.07	0.04	0.33
O88811-2	Isoform 2 of Signal transducing adapter molecule 2 OS=Mus musculus GN=Stam2 - [STAM2_MOUSE]	12.53	2	3	4	11	0.70	0.49	0.70	0.53
Q91VJ4	Serine/threonine-protein kinase 38 OS=Mus musculus GN=Stk38 PE=1 SV=1 - [STK38_MOUSE]	13.12	1	4	6	8	-0.19	0.02	-0.06	0.15
Q569Z5	Probable ATP-dependent RNA helicase DDX46 OS=Mus musculus GN=Ddx46 PE=1 SV=2 - [DDX46_MOUSE]	22.87	3	22	22	82	0.05	0.30	0.20	0.27
Q9DBZ1-2	Isoform 2 of Inhibitor of nuclear factor kappa-B kinase-interacting protein OS=Mus musculus GN=Ikbip - [IKIP_MOUSE]	15.36	1	2	4	10	-1.17	-1.08	-0.99	-0.89
Q3TWL2	Transmembrane protein 55B OS=Mus musculus GN=Tmem55b PE=1 SV=1 - [TM55B_MOUSE]	40.14	5	4	4	7	0.01	0.18	0.13	0.30
Q7TPN9	Proline-rich protein 14 OS=Mus musculus GN=Prr14 PE=2 SV=1 - [PRR14_MOUSE]	1.63	1	1	1	2	-0.58	-1.12	-0.04	-0.59
Q91VJ2	Protein kinase C delta-binding protein OS=Mus musculus GN=Prkcdbp PE=1 SV=1 - [PRDBP_MOUSE]	32.69	1	6	6	64	0.00	-0.28	-0.08	-0.25
Q9QYB2-2	Isoform 2 of Dachshund homolog 1 OS=Mus musculus GN=Dach1 - [DACH1_MOUSE]	3.00	2	1	1	1	-0.22	-0.10	-0.22	-0.10
Q8K2Z4	Condensin complex subunit 1 OS=Mus musculus GN=Ncapd2 PE=1 SV=2 - [CND1_MOUSE]	3.59	2	3	3	5	0.44	-0.11	0.11	-0.25
D6RIL0	Protein polybromo-1 OS=Mus musculus GN=Pbrm1 PE=2 SV=1 - [D6RIL0_MOUSE]	1.99	13	2	2	2	-0.17	-0.56	0.02	-0.37
P19639	Glutathione S-transferase Mu 3 OS=Mus musculus GN=Gstm3 PE=1 SV=2 - [GSTM4_MOUSE]	55.96	1	7	16	221	-0.24	-0.33	-0.22	-0.14

E9Q4M4	Coiled-coil-helix-coiled-coil-helix domain-containing protein 6, mitochondrial OS=Mus musculus GN=Chchd6 PE=2 SV=1 - [E9Q4M4_MOUSE]	24.90	2	5	5	12	-0.17	-0.27	-0.25	-0.37
P62852	40S ribosomal protein S25 OS=Mus musculus GN=Rps25 PE=2 SV=1 - [RS25_MOUSE]	29.60	1	5	5	113	0.50	0.57	0.46	0.50
D3Z4T4	Sec1 family domain-containing protein 2 OS=Mus musculus GN=Scfd2 PE=2 SV=1 [D3Z4T4_MOUSE]	3.13	4	2	2	4	0.25	0.29	0.21	0.25
Q3UQ84	Threonine--tRNA ligase, mitochondrial OS=Mus musculus GN=Tars2 PE=2 SV=1 - [SYTM_MOUSE]	16.87	4	9	10	18	0.07	-0.25	0.14	0.11
Q9D9L5	Actin-related protein T2 OS=Mus musculus GN=Actrt2 PE=2 SV=1 - [ACTT2_MOUSE]	4.77	1	2	2	2	0.44	-0.05	0.42	-0.07
Q5SSH7-2	Isoform 2 of Zinc finger ZZ-type and EF-hand domain-containing protein 1 OS=Mus musculus GN=Zzef1 - [ZZEF1_MOUSE]	3.94	4	9	9	14	0.40	0.44	0.16	0.10
P0DM40	Fer-1-like protein 5 OS=Mus musculus GN=Fer1l5 PE=1 SV=1 - [FR1L5_MOUSE]	0.83	1	1	1	2	-0.16	0.00	-0.35	-0.18
Q9CPT3	N-acylneuraminate-9-phosphatase OS=Mus musculus GN=Nanp PE=1 SV=1 - [NANP_MOUSE]	2.82	1	1	1	3	0.34	0.45	0.28	0.39
O70480	Vesicle-associated membrane protein 4 OS=Mus musculus GN=Vamp4 PE=1 SV=1 - [VAMP4_MOUSE]	23.40	2	2	2	14	-0.25	0.02	-0.31	-0.17
Q60996-3	Isoform 3 of Serine/threonine-protein phosphatase 2A 56 kDa regulatory subunit gamma isoform OS=Mus musculus GN=Ppp2r5c - [2A5G_MOUSE]	16.08	4	4	8	19	0.35	-0.08	0.30	-0.12
Q8K2C7-2	Isoform 2 of Protein OS 9 OS=Mus musculus GN=Os9 - [OS9_MOUSE]	14.75	4	5	6	26	0.12	0.40	0.02	0.17
G3UWR2	PERQ amino acid-rich with GYF domain-containing protein 2 OS=Mus musculus GN=Gigyf2 PE=2 SV=1 - [G3UWR2_MOUSE]	7.04	7	5	5	16	0.00	-0.24	0.24	-0.12
Q8K4Q8	Collectin-12 OS=Mus musculus GN=Colec12 PE=1 SV=1 - [COL12_MOUSE]	4.18	1	2	2	8	-0.11	-0.04	-0.04	0.17

Q921V5	Alpha-1,6-mannosyl-glycoprotein 2-beta-N-acetylglucosaminyltransferase OS=Mus musculus GN=Mgat2 PE=2 SV=1 - [MGAT2_MOUSE]	7.92	1	3	3	7	0.60	0.23	0.26	-0.03
Q8BH95	Enoyl-CoA hydratase, mitochondrial OS=Mus musculus GN=Echs1 PE=1 SV=1 - [ECHM_MOUSE]	39.66	2	12	12	133	-0.03	-0.07	-0.06	-0.02
Q9QXK8	Neuromedin-U OS=Mus musculus GN=Nmu PE=2 SV=1 - [NMU_MOUSE]	8.62	1	1	1	3	-0.43	-0.27	-0.14	0.02
Q9CQD1	Ras-related protein Rab-5A OS=Mus musculus GN=Rab5a PE=1 SV=1 - [RAB5A_MOUSE]	51.16	1	5	8	56	0.27	0.03	0.36	0.16
E9PUA2	Terminal uridylyltransferase 7 OS=Mus musculus GN=Zcchc6 PE=4 SV=1 - [E9PUA2_MOUSE]	9.57	2	7	8	15	0.22	0.13	0.04	0.19
D3Z6H1	Fibroblast growth factor 1 OS=Mus musculus GN=Fgf1 PE=2 SV=1 - [D3Z6H1_MOUSE]	18.33	4	1	1	2	-0.20	-0.55	-0.07	-0.42
P28033	CCAAT/enhancer-binding protein beta OS=Mus musculus GN=Cebpb PE=1 SV=1 - [CEBPB_MOUSE]	20.27	1	2	2	9	-0.85	-0.47	-0.84	-0.46
Q8VDG7	Platelet-activating factor acetylhydrolase 2, cytoplasmic OS=Mus musculus GN=Pafah2 PE=2 SV=2 - [PAFA2_MOUSE]	13.08	3	4	4	7	0.12	0.19	0.15	0.05
H3BK86	Peptide YY OS=Mus musculus GN=Pyy PE=3 SV=1 - [H3BK86_MOUSE]	15.00	1	1	1	7	-0.44	-0.34	-0.61	-0.50
Q9JKK1	Syntaxin-6 OS=Mus musculus GN=Stx6 PE=1 SV=1 - [STX6_MOUSE]	10.20	2	2	2	9	0.06	0.10	0.12	0.17
Q920I9-2	Isoform 2 of WD repeat-containing protein 7 OS=Mus musculus GN=Wdr7 - [WDR7_MOUSE]	2.06	2	2	2	6	0.16	-0.31	0.11	-0.37
Q62442-2	Isoform 2 of Vesicle-associated membrane protein 1 OS=Mus musculus GN=Vamp1 - [VAMP1_MOUSE]	20.51	3	1	2	9	0.39	0.90	0.11	0.62
Q8CG72	Poly(ADP-ribose) glycohydrolase ARH3 OS=Mus musculus GN=Adprhl2 PE=1 SV=1 - [ARHL2_MOUSE]	19.73	2	5	5	11	-0.09	-0.38	-0.16	-0.34
A2AJ72	MCG130458 OS=Mus musculus GN=Fubp3 PE=4 SV=1 - [A2AJ72_MOUSE]	27.24	5	10	12	47	0.24	0.23	0.21	0.13
Q8C3X2	Coiled-coil domain-containing protein 90B, mitochondrial OS=Mus musculus GN=Ccdc90b PE=2 SV=1 - [CC90B_MOUSE]	25.78	2	7	7	20	-0.22	-0.03	-0.04	-0.03

F6ZJ37	Zinc finger protein 827 (Fragment) OS=Mus musculus GN=Zfp827 PE=4 SV=1 - [F6ZJ37_MOUSE]	4.37	1	1	1	1	0.27	0.61	0.27	0.61
F6SVV1	Protein Gm9493 OS=Mus musculus GN=Gm9493 PE=4 SV=1 - [F6SVV1_MOUSE]	46.35	3	6	6	93	0.24	0.38	0.22	0.21
P97390	Vacuolar protein sorting associated protein 45 OS=Mus musculus GN=Vps45 PE=1 SV=1 - [VPS45_MOUSE]	9.47	1	5	5	8	0.10	0.26	0.11	0.25
F8WI14	Extracellular matrix protein 1 OS=Mus musculus GN=Ecm1 PE=2 SV=1 - [F8WI14_MOUSE]	3.41	2	2	2	5	-0.70	-0.26	-0.39	-0.30
A2AJ76	Hemicentin-2 OS=Mus musculus GN=Hmcn2 PE=1 SV=1 - [HMCN2_MOUSE]	4.33	2	13	13	33	-0.24	-0.41	-0.01	-0.21
Q02788	Collagen alpha-2(VI) chain OS=Mus musculus GN=Col6a2 PE=2 SV=3 - [CO6A2_MOUSE]	19.05	9	19	19	142	-0.33	-0.28	-0.38	-0.31
Q80XU3	Nuclear ubiquitous casein and cyclin-dependent kinase substrate 1 OS=Mus musculus GN=Nucks1 PE=1 SV=1 - [NUCKS_MOUSE]	12.39	1	2	2	12	0.69	0.23	0.59	0.12
F6TCF9	BAG family molecular chaperone regulator 1 OS=Mus musculus GN=Bag1 PE=2 SV=1 - [F6TCF9_MOUSE]	23.10	3	7	7	46	0.16	0.23	0.25	0.19
O09167	60S ribosomal protein L21 OS=Mus musculus GN=Rpl21 PE=2 SV=3 [RL21_MOUSE]	26.25	4	6	6	47	0.46	0.64	0.23	0.49
Q91UZ5	Inositol monophosphatase 2 OS=Mus musculus GN=Impa2 PE=1 SV=1 - [IMPA2_MOUSE]	2.76	1	1	1	2	-0.12	-0.55	0.04	-0.39
Q9Z2W1	Serine/threonine-protein kinase 25 OS=Mus musculus GN=Stk25 PE=1 SV=2 - [STK25_MOUSE]	16.90	2	4	6	14	0.21	0.01	0.26	0.01
D3Z5G0	Protein Pydc4 OS=Mus musculus GN=Pydc4 PE=2 SV=1 - [D3Z5G0_MOUSE]	3.07	1	1	1	2	0.34	0.06	-0.41	-0.70
Q501J6	Probable ATP-dependent RNA helicase DDX17 OS=Mus musculus GN=Ddx17 PE=2 SV=1 - [DDX17_MOUSE]	35.69	3	15	22	219	0.12	0.10	0.10	0.13
Q8BGU5-2	Isoform 2 of Cyclin-Y OS=Mus musculus GN=Ccny - [CCNY_MOUSE]	5.38	2	1	1	3	-0.22	-0.77	0.31	-0.24
P35762	CD81 antigen OS=Mus musculus GN=Cd81 PE=1 SV=2 - [CD81_MOUSE]	17.80	1	2	2	15	-0.90	-0.26	-0.14	-0.09

Q924D0	Reticulon-4-interacting protein 1, mitochondrial OS=Mus musculus GN=Rtn4ip1 PE=1 SV=2 - [RT4I1_MOUSE]	9.85	2	4	4	5	0.77	0.23	0.25	0.35
Q8QZY1	Eukaryotic translation initiation factor 3 subunit L OS=Mus musculus GN=Eif3l PE=1 SV=1 - [EIF3L_MOUSE]	39.18	1	20	20	109	0.26	-0.07	0.14	-0.02
O35379	Multidrug resistance-associated protein 1 OS=Mus musculus GN=Abcc1 PE=1 SV=1 - [MRP1_MOUSE]	7.33	2	6	8	17	-0.21	-0.06	-0.08	-0.06
P07744	Keratin, type II cytoskeletal 4 OS=Mus musculus GN=Krt4 PE=1 SV=2 - [K2C4_MOUSE]	5.90	1	1	5	110	-0.08	-0.11	-0.41	-0.44
B2RUP2-2	Isoform 2 of Protein unc 13 homolog D OS=Mu musculus GN=Unc13d [UN13D_MOUSE]	3.51	2	2	3	4	0.04	-0.06	0.08	-0.03
P45878	Peptidyl-prolyl cis-trans isomerase FKBP2 OS=Mus musculus GN=Fkbp2 PE=1 SV=1 - [FKBP2_MOUSE]	69.29	1	10	10	107	0.13	0.33	0.01	0.25
L7N1Y0	Protein Dnah7b OS=Mus musculus GN=Dnah7b PE=4 SV=1 - [L7N1Y0_MOUSE]	2.34	2	6	6	29	-0.05	-0.02	-0.01	-0.23
E9Q3A0	E3 ubiquitin-protein ligase Trim36 OS=Mus musculus GN=Trim36 PE=2 SV=1 - [E9Q3A0_MOUSE]	3.07	2	2	2	2	-0.91	0.49	-0.67	0.73
Q8C0D5	Elongation factor Tu GTP-binding domain-containing protein 1 OS=Mus musculus GN=Eftud1 PE=2 SV=1 - [ETUD1_MOUSE]	10.74	2	9	10	21	0.12	0.18	0.25	0.02
Q8BYU6	Torsin-1A-interacting protein 2 OS=Mus musculus GN=Tor1aip2 PE=1 SV=1 - [TOIP2_MOUSE]	9.56	1	3	3	12	-0.20	-0.33	-0.02	-0.27
Q91VJ1	Interferon-inducible protein AIM2 OS=Mus musculus GN=Aim2 PE=1 SV=2 - [AIM2_MOUSE]	5.65	1	2	2	2	-0.25	-0.66	-0.17	-0.58
Q9WVK4	EH domain-containing protein 1 OS=Mus musculus GN=Ehd1 PE=1 SV=1 - [EHD1_MOUSE]	55.99	1	18	26	208	0.06	0.23	-0.05	0.17
D3YYN7	Sodium/potassium-transporting ATPase subunit alpha-2 OS=Mus musculus GN=Atp1a2 PE=2 SV=1 - [D3YYN7_MOUSE]	25.77	3	4	22	478	-0.57	-0.41	-0.62	-0.50
Q9CRD2	ER membrane protein complex subunit 2 OS=Mus musculus GN=Emc2 PE=2 SV=1 [EMC2_MOUSE]	20.20	1	6	6	26	0.18	0.12	0.15	0.14

P09103	Protein disulfide-isomerase OS=Mus musculus GN=P4hb PE=1 SV=2 - [PDIA1_MOUSE]	68.57	2	36	39	1077	0.16	0.24	0.12	0.22
Q91V55	40S ribosomal protein S5 OS=Mus musculus GN=Rps5 PE=2 SV=1 - [Q91V55_MOUSE]	35.78	3	7	7	142	0.46	0.65	0.48	0.64
Q8BGQ1-2	Isoform 2 of Spermatogenesis-defective protein 39 homolog OS=Mus musculus GN=Vipas39 - [SPE39_MOUSE]	9.53	2	4	4	9	0.24	0.02	0.16	-0.06
Q5S006	Leucine-rich repeat serine/threonine-protein kinase 2 OS=Mus musculus GN=Lrrk2 PE=1 SV=2 - [LRRK2_MOUSE]	3.13	1	5	5	11	-0.24	-0.07	0.30	0.38
Q8C7D2-2	Isoform 2 of Protein cereblon OS=Mus musculus GN=Crbn - [CRBN_MOUSE]	6.50	3	2	2	3	-0.73	-1.02	-0.45	-0.73
Q9QXJ2	Signal transducer and activator of transcription 2 OS=Mus musculus GN=Stat2 PE=1 SV=1 - [Q9QXJ2_MOUSE]	9.22	3	6	6	16	0.07	0.10	0.21	0.16
Q9QWR8	Alpha-N-acetylgalactosaminidase OS=Mus musculus GN=Naga PE=2 SV=2 - [NAGAB_MOUSE]	42.41	1	13	14	80	-0.18	-0.09	-0.08	-0.04
P97478	Ubiquinone biosynthesis protein COQ7 homolog OS=Mus musculus GN=Coq7 PE=2 SV=3 - [COQ7_MOUSE]	12.44	2	2	2	9	0.20	0.27	0.36	0.38
Q62167	ATP-dependent RNA helicase DDX3X OS=Mus musculus GN=Ddx3x PE=1 SV=3 - [DDX3X_MOUSE]	39.27	3	24	27	184	0.32	0.36	0.17	0.29
Q62388	Serine-protein kinase ATM OS=Mus musculus GN=Atm PE=1 SV=2 - [ATM_MOUSE]	2.38	1	4	5	7	-1.51	-1.48	-1.75	-1.72
P45591	Cofilin-2 OS=Mus musculus GN=Cfl2 PE=1 SV=1 - [COF2_MOUSE]	66.87	1	7	10	309	0.35	0.23	0.29	0.22
Q07076	Annexin A7 OS=Mus musculus GN=Anxa7 PE=2 SV=2 - [ANXA7_MOUSE]	23.97	1	10	11	126	0.23	0.24	0.22	0.21
Q80X85	28S ribosomal protein S7, mitochondrial OS=Mus musculus GN=Mrps7 PE=2 SV=1 - [RT07_MOUSE]	15.29	1	3	3	8	0.26	0.47	0.28	0.48
P24668	Cation-dependent mannose-6-phosphate receptor OS=Mus musculus GN=M6pr PE=1 SV=1 - [MPRD_MOUSE]	19.42	1	5	5	34	0.10	0.11	0.07	0.15
A2A9W3	MIF4G domain-containing protein OS=Mus musculus GN=Mif4gd PE=2 SV=1 - [A2A9W3_MOUSE]	10.84	4	2	2	4	1.18	-0.03	1.11	-0.10

P49813	Tropomodulin-1 OS=Mus musculus GN=Tmod1 PE=2 SV=2 - [TMOD1_MOUSE]	19.22	1	3	4	16	0.19	-0.68	-0.27	-0.87
Q9D0D3	Poly(A) RNA polymerase, mitochondrial OS=Mus musculus GN=Mtpap PE=2 SV=1 - [PAPD1_MOUSE]	14.36	1	5	5	20	0.49	0.22	0.36	0.14
Q99JI4	26S proteasome non-ATPase regulatory subunit 6 OS=Mus musculus GN=Psmd6 PE=1 SV=1 - [PSMD6_MOUSE]	34.70	1	14	14	117	0.24	0.19	0.33	0.36
Q91YP0	L-2-hydroxyglutarate dehydrogenase, mitochondrial OS=Mus musculus GN=L2hgdh PE=2 SV=1 - [L2HDH_MOUSE]	16.81	1	7	7	22	0.05	-0.28	-0.24	-0.31
P13020-2	Isoform 2 of Gelsolin OS=Mus musculus GN=Gsn - [GELS_MOUSE]	45.28	3	27	27	693	-0.17	-0.11	-0.15	-0.07
F8VPX1	Ubiquitin carboxyl-terminal hydrolase OS=Mus musculus GN=Usp7 PE=2 SV=1 - [F8VPX1_MOUSE]	16.41	7	15	15	31	0.38	0.37	0.28	0.33
Q9EQG9-2	Isoform 2 of Collagen type IV alpha-3-binding protein OS=Mus musculus GN=Col4a3bp - [C43BP_MOUSE]	10.03	3	6	6	17	0.12	0.08	0.15	0.24
P10922	Histone H1.0 OS=Mus musculus GN=H1f0 PE=2 SV=4 - [H10_MOUSE]	24.74	1	6	6	68	-0.37	-0.24	-0.32	-0.23
D3YXC2	Mitochondrial carrier homolog 1 (Fragment) OS=Mus musculus GN=Mtch1 PE=2 SV=1 - [D3YXC2_MOUSE]	10.19	3	1	1	3	0.21	-0.64	0.12	-0.72
P01575	Interferon beta OS=Mus musculus GN=Ifnb1 PE=1 SV=1 - [IFNB_MOUSE]	7.14	1	1	1	1	0.80	0.99	1.10	1.30
Q80YT7	Myomegalin OS=Mus musculus GN=Pde4dip PE=2 SV=2 - [MYOME_MOUSE]	5.71	6	8	9	16	0.12	-0.05	-0.04	-0.21
Q3UWA6-2	Isoform 2 of Heat-stable enterotoxin receptor OS=Mus musculus GN=Gucy2c - [GUC2C_MOUSE]	4.29	2	4	4	10	-0.10	-0.19	-0.11	-0.22
Q99LI7	Cleavage stimulation factor subunit 3 OS=Mus musculus GN=Cstf3 PE=1 SV=1 - [CSTF3_MOUSE]	7.95	1	5	5	12	0.13	0.16	0.20	0.23
O88559-2	Isoform 2 of Menin OS=Mus musculus GN=Men1 - [MEN1_MOUSE]	6.12	4	2	3	3	-0.46	-0.46	-0.25	-0.26
O35387	HCLS1-associated protein X-1 OS=Mus musculus GN=Hax1 PE=1 SV=1 - [HAX1_MOUSE]	3.93	1	1	1	1	0.05	0.03	0.25	0.23

Q9CR95	Adaptin ear-binding coat-associated protein 1 OS=Mus musculus GN=Necap1 PE=1 SV=2 - [NECP1_MOUSE]	18.55	1	3	3	6	-0.35	-0.52	-0.06	-0.24
E9QNN1	ATP-dependent RNA helicase A OS=Mus musculus GN=Dhx9 PE=2 SV=1 - [E9QNN1_MOUSE]	25.43	7	29	30	228	0.03	0.10	0.11	0.14
F8WGM8	Actin, cytoplasmic 2 (Fragment) OS=Mus musculus GN=Actg1 PE=2 SV=1 - [F8WGM8_MOUSE]	87.62	1	1	9	1428	0.07	0.13	0.16	0.22
Q7TN37	Transient receptor potential cation channel subfamily M member 4 OS=Mus musculus GN=Trpm4 PE=1 SV=1 - [TRPM4_MOUSE]	5.77	3	5	5	7	-1.63	-0.51	-2.89	-0.49
Q9CPU4	Microsomal glutathione S-transferase 3 OS=Mus musculus GN=Mgst3 PE=1 SV=1 - [MGST3_MOUSE]	24.84	1	3	3	55	-0.11	-0.11	-0.17	-0.17
Q8BUU7	N-acetylgalactosamine kinase OS=Mus musculus GN=Galk2 PE=2 SV=1 - [Q8BUU7_MOUSE]	30.43	5	10	10	26	0.22	-0.07	-0.07	-0.21
Q8BLX7	Collagen alpha-1(XVI) chain OS=Mus musculus GN=Col16a1 PE=2 SV=2 - [COGA1_MOUSE]	3.92	4	3	3	4	0.00	-0.92	-0.05	-0.85
E9Q634	Unconventional myosin-Ie OS=Mus musculus GN=Myo1e PE=1 SV=1 - [MYO1E_MOUSE]	12.38	1	10	12	32	-0.24	0.12	-0.02	0.27
F6SY41	tRNA (uracil-5-)-methyltransferase homolog A (Fragment) OS=Mus musculus GN=Trmt2a PE=2 SV=1 - [F6SY41_MOUSE]	21.43	4	1	1	5	0.10	-0.29	0.09	-0.30
Q99PT1	Rho GDP-dissociation inhibitor 1 OS=Mus musculus GN=Arhgdia PE=1 SV=3 - [GDIR1_MOUSE]	69.61	1	15	15	243	0.55	0.59	0.51	0.46
Q8VEH8	Endoplasmic reticulum lectin 1 OS=Mus musculus GN=Erlec1 PE=2 SV=1 - [ERLEC_MOUSE]	3.31	2	2	2	3	0.03	0.17	-0.02	0.11
Q80W54	CAAX prenyl protease 1 homolog OS=Mus musculus GN=Zmpste24 PE=1 SV=2 - [FACE1_MOUSE]	12.84	2	6	6	16	0.04	0.14	0.01	0.02
Q99PM3	Transcription initiation factor IIA subunit 1 OS=Mus musculus GN=Gtf2a1 PE=2 SV=2 - [TF2AA_MOUSE]	5.29	3	2	2	4	0.27	-0.39	0.47	-0.20
D3Z4D8	BRISC complex subunit Abro1 (Fragment) OS=Mus musculus GN=Fam175b PE=2 SV=2 - [D3Z4D8_MOUSE]	14.53	2	3	3	12	0.19	-0.04	0.30	0.25

Q9D6J6	NADH dehydrogenase [ubiquinone] flavoprotein 2, mitochondrial OS=Mus musculus GN=Ndufv2 PE=1 SV=2 - [NDUV2_MOUSE]	55.24	4	12	13	174	-0.19	-0.20	-0.13	-0.20
Q61329	Zinc finger homeobox protein 3 OS=Mus musculus GN=Zfhx3 PE=1 SV=1 - [ZFHX3_MOUSE]	2.17	2	3	3	8	-0.84	0.15	-0.83	0.15
Q8BTV1	Tumor suppressor candidate 3 OS=Mus musculus GN=Tusc3 PE=2 SV=1 - [TUSC3_MOUSE]	9.51	2	2	4	7	0.47	0.08	0.08	-0.31
O54791	Transcription factor MafF OS=Mus musculus GN=Maff PE=2 SV=1 - [MAFF_MOUSE]	4.49	3	1	1	1	-0.18	0.07	0.32	0.57
Q64464	Cytochrome P450 3A13 OS=Mus musculus GN=Cyp3a13 PE=2 SV=1 - [CP3AD_MOUSE]	13.32	1	5	6	18	-0.01	-0.16	0.25	-0.02
P63168	Dynein light chain 1, cytoplasmic OS=Mus musculus GN=Dynll1 PE=1 SV=1 - [DYL1_MOUSE]	37.08	2	3	3	43	0.09	0.42	0.33	0.58
Q8CI08	SLAIN motif-containing protein 2 OS=Mus musculus GN=Slain2 PE=1 SV=2 - [SLAI2_MOUSE]	14.11	2	5	5	12	-0.06	-0.26	-0.02	-0.33
F8VQE9	Arf-GAP with GTPase, ANK repeat and PH domain-containing protein 3 OS=Mus musculus GN=Agap3 PE=2 SV=1 - [F8VQE9_MOUSE]	3.08	6	3	3	4	0.10	0.20	-0.02	0.08
P67871	Casein kinase II subuni beta OS=Mus musculus GN=Csnk2b PE=1 SV=1 - [CSK2B_MOUSE]	41.40	6	2	8	32	-0.40	-0.61	0.00	-0.21
Q0VGB7	Serine/threonine-protein phosphatase 4 regulatory subunit 2 OS=Mus musculus GN=Ppp4r2 PE=1 SV=1 - [PP4R2_MOUSE]	8.87	1	3	3	7	0.17	0.58	0.14	0.41
Q9CQ69	Cytochrome b-c1 complex subunit 8 OS=Mus musculus GN=Uqcrq PE=1 SV=3 [QCR8_MOUSE]	56.10	2	6	6	103	0.00	0.07	-0.02	0.03
Q02566	Myosin-6 OS=Mus musculus GN=Myh6 PE=1 SV=2 - [MYH6_MOUSE]	1.55	2	1	5	122	-0.08	0.17	0.09	0.34
J3QMM7	ATP-dependent (S)-NAD(P)H-hydrate dehydratase OS=Mus musculus GN=Carkd PE=3 SV=1 - [J3QMM7_MOUSE]	23.85	7	6	6	67	-0.01	-0.07	-0.07	0.05
P59708	Pre-mRNA branch site protein p14 OS=Mus musculus GN=Sf3b14 PE=2 SV=1 - [PM14_MOUSE]	28.00	1	3	3	25	0.08	-0.20	0.21	-0.06

Q99JR1	Sideroflexin-1 OS=Mus musculus GN=Sfxn1 PE=1 SV=3 - [SFXN1_MOUSE]	31.37	1	7	8	44	-0.12	0.00	-0.23	-0.28
O54724	Polymerase I and transcript release factor OS=Mus musculus GN=Ptrf PE=1 SV=1 - [PTRF_MOUSE]	39.29	2	18	19	366	-0.13	-0.08	-0.21	-0.13
E9Q4U7	Protein diaphanous homolog 2 OS=Mus musculus GN=Diap2 PE=2 SV=1 - [E9Q4U7_MOUSE]	5.63	3	5	7	10	0.41	0.38	0.51	0.38
Q8BG67	Protein EFR3 homolog A OS=Mus musculus GN=Efr3a PE=1 SV=1 - [EFR3A_MOUSE]	8.67	3	5	5	11	0.23	0.18	0.06	-0.05
Q80VD1	Protein FAM98B OS=Mus musculus GN=Fam98b PE=2 SV=1 - [FA98B_MOUSE]	18.18	1	3	6	45	0.18	-0.06	0.16	0.06
Q99K99	Uncharacterized protein C4orf19 homolog OS=Mus musculus PE=1 SV=2 - [CD019_MOUSE]	11.50	1	2	2	2	-0.12	-0.06	-0.22	-0.16
Q8BYH8-2	Isoform 2 of Chromodomain-helicase-DNA-binding protein 9 OS=Mus musculus GN=Chd9 - [CHD9_MOUSE]	2.27	2	4	4	4	-0.23	-0.38	0.05	-0.10
Q61768	Kinesin-1 heavy chain OS=Mus musculus GN=Kif5b PE=1 SV=3 [KINH_MOUSE]	51.19	2	32	41	271	0.30	0.35	0.25	0.26
P49718	DNA replication licensing factor MCM5 OS=Mus musculus GN=Mcm5 PE=2 SV=1 - [MCM5_MOUSE]	19.78	2	12	12	52	0.33	0.24	0.27	0.27
Q6P9L6	Kinesin-like protein KIF15 OS=Mus musculus GN=Kif15 PE=1 SV=1 - [KIF15_MOUSE]	1.80	1	1	2	2	0.18	-0.24	0.53	0.11
Q9JI39	ATP-binding cassette sub-family B member 10, mitochondrial OS=Mus musculus GN=Abcb10 PE=2 SV=1 - [ABCBA_MOUSE]	3.50	1	2	2	5	0.07	0.36	-0.01	0.18
F6WLY0	Lymphoid-restricted membrane protein (Fragment) OS=Mus musculus GN=Lrmp PE=4 SV=1 - [F6WLY0_MOUSE]	4.37	3	2	2	3	-0.91	-3.30	-0.79	-3.18
Q8BW96-2	Isoform 2 of Calcium/calmodulin-dependent protein kinase type 1D OS=Mus musculus GN=Camk1d - [KCC1D_MOUSE]	37.02	3	7	10	48	0.55	0.49	0.45	0.44
P59016	Vacuolar protein sorting associated protein 33B OS=Mus musculus GN=Vps33b PE=1 SV=1 - [VP33B_MOUSE]	7.78	1	5	5	6	0.00	-0.02	-0.06	-0.08
Q8R4X3	RNA-binding protein 12 OS=Mus musculus GN=Rbm12 PE=1 SV=3 - [RBM12_MOUSE]	11.09	5	10	10	23	-0.24	0.12	-0.34	-0.05

P70452	Syntaxin-4 OS=Mus musculus GN=Stx4 PE=1 SV=1 - [STX4_MOUSE]	29.87	1	8	8	31	0.07	0.04	-0.07	0.06
Q9DD02-2	Isoform 2 of Protein Hikeshi OS=Mus musculus GN=L7rn6 - [HIKES_MOUSE]	10.13	2	1	1	4	0.39	0.29	0.30	0.19
P58802	TBC1 domain family member 10A OS=Mus musculus GN=Tbc1d10a PE=1 SV=1 - [TB10A_MOUSE]	2.60	2	1	1	1	0.07	-0.07	0.67	0.53
F6UCX4	Protein Sptbn5 (Fragment) OS=Mus musculus GN=Sptbn5 PE=4 SV=1 - [F6UCX4_MOUSE]	1.24	1	2	2	3	0.22	0.44	0.36	0.57
F6YR19	Splicing factor 1 (Fragment) OS=Mus musculus GN=Sf1 PE=4 SV=1 - [F6YR19_MOUSE]	12.00	1	1	1	4	0.33	0.27	0.01	-0.05
Q920A7	AFG3-like protein 1 OS=Mus musculus GN=Afg3l1 PE=2 SV=2 - [AFG31_MOUSE]	4.06	2	2	3	7	-1.23	-1.09	-1.13	-1.00
Q6P3Z3	THAP domain-containing protein 4 OS=Mus musculus GN=Thap4 PE=2 SV=1 - [THAP4_MOUSE]	8.08	2	3	3	4	-0.03	0.29	-0.24	0.07
Q921K9-2	Isoform 2 of B-cell CLL/lymphoma 7 protein family member B OS=Mus musculus GN=Bcl7b - [BCL7B_MOUSE]	18.97	5	1	1	2	0.26	0.17	0.14	0.05
P29594	Caspase-2 OS=Mus musculus GN=Casp2 PE=1 SV=5 - [CASP2_MOUSE]	1.33	1	1	1	8	0.35	0.36	0.58	0.59
E9PV38	Protein Ces2g OS=Mus musculus GN=Ces2g PE=2 SV=1 - [E9PV38_MOUSE]	21.43	7	3	9	77	0.58	-0.63	0.29	-0.92
D3Z0M9	MCG18410, isoform CRA_a OS=Mus musculus GN=Ddx23 PE=3 SV=1 - [D3Z0M9_MOUSE]	12.82	1	9	9	30	0.06	0.14	0.31	0.31
Q9D2C2	Protein SAAL1 OS=Mus musculus GN=Saal1 PE=1 SV=1 - [SAAL1_MOUSE]	9.70	2	4	4	5	-0.08	0.20	0.09	0.36
P21447	Multidrug resistance protein 1A OS=Mus musculus GN=Abcb1a PE=1 SV=3 - [MDR1A_MOUSE]	28.13	3	19	28	142	-0.40	-0.43	-0.35	-0.32
Q8C5W3	Tubulin-specific chaperone cofactor E-like protein OS=Mus musculus GN=Tbcel PE=1 SV=1 - [TBCEL_MOUSE]	13.92	3	5	5	13	-0.01	-0.31	-0.17	-0.41
Q3TH34	Protein FAM45A OS=Mus musculus GN=Fam45a PE=2 SV=1 - [Q3TH34_MOUSE]	27.54	2	3	3	14	0.08	0.28	-0.13	0.22
F8WI35	Histone H3 OS=Mus musculus GN=H3f3a PE=2 SV=1 - [F8WI35_MOUSE]	34.07	6	2	8	341	-0.13	0.06	-0.17	0.16

Q8CIH9	Amidophosphoribosyltransferase OS=Mus musculus GN=Ppat PE=2 SV=1 - [Q8CIH9_MOUSE]	14.51	1	5	5	10	0.29	0.15	0.08	0.13
Q91YK2	Ribosomal RNA processing protein 1 homolog B OS=Mus musculus GN=Rrp1b PE=2 SV=2 - [RRP1B_MOUSE]	5.39	1	3	3	4	-0.54	-0.10	-0.44	0.21
Q8CIZ8	von Willebrand factor OS=Mus musculus GN=Vwf PE=1 SV=2 - [VWF_MOUSE]	3.20	2	7	7	14	-0.53	-0.38	-0.57	-0.70
Q61771	Kinesin-like protein KIF3B OS=Mus musculus GN=Kif3b PE=1 SV=1 - [KIF3B_MOUSE]	6.43	7	3	4	8	0.06	0.25	0.09	0.12
P14685	26S proteasome non-ATPase regulatory subunit 3 OS=Mus musculus GN=Psmd3 PE=1 SV=3 - [PSMD3_MOUSE]	39.81	2	20	20	114	0.29	0.34	0.26	0.27
Q8K268	ATP-binding cassette sub-family F member 3 OS=Mus musculus GN=Abcf3 PE=1 SV=1 - [ABCF3_MOUSE]	6.77	1	4	4	6	0.41	0.33	0.25	0.16
Q9WV85	Nucleoside diphosphate kinase 3 OS=Mus musculus GN=Nme3 PE=2 SV=3 - [NDK3_MOUSE]	27.22	1	4	4	16	0.04	0.04	-0.09	-0.02
G3X9I4	Aly/REF export factor 2 OS=Mus musculus GN=Alyref2 PE=4 SV=1 - [G3X9I4_MOUSE]	19.72	2	1	3	48	0.16	-0.12	0.15	-0.13
Q9DBG5	Perilipin-3 OS=Mus musculus GN=Plin3 PE=1 SV=1 - [PLIN3_MOUSE]	39.82	1	10	10	24	0.06	-0.03	0.26	0.21
Q04447	Creatine kinase B-type OS=Mus musculus GN=Ckb PE=1 SV=1 - [KCRB_MOUSE]	74.80	1	20	22	1724	0.14	0.20	0.09	0.15
Q5EG47	5'-AMP-activated protein kinase catalytic subunit alpha-1 OS=Mus musculus GN=Prkaa1 PE=1 SV=2 - [AAPK1_MOUSE]	18.43	1	8	10	33	-0.12	0.06	-0.09	0.17
A2AQP0	Myosin-7B OS=Mus musculus GN=Myh7b PE=3 SV=1 - [MYH7B_MOUSE]	3.25	1	3	7	96	0.03	0.07	0.32	0.36
Q4U4S6	Xin actin-binding repeat containing protein 2 OS=Mus musculus GN=Xirp2 PE=1 SV=1 [XIRP2_MOUSE]	2.40	4	5	8	24	-0.06	0.17	-0.39	-0.14
P53986	Monocarboxylate transporter 1 OS=Mus musculus GN=Slc16a1 PE=1 SV=1 - [MOT1_MOUSE]	13.39	1	5	5	19	0.24	0.38	0.12	0.07
P14438	H-2 class II histocompatibility antigen, A-U alpha chain (Fragment) OS=Mus musculus GN=H2-Aa PE=1 SV=1 - [HA2U_MOUSE]	25.55	10	5	5	18	0.03	0.02	-0.09	-0.05

Q78IS1	Transmembrane emp24 domain-containing protein 3 OS=Mus musculus GN=Tmed3 PE=2 SV=1 - [TMED3_MOUSE]	16.74	1	3	3	6	1.04	-0.22	0.94	-0.18
Q91V01	Lysophospholipid acyltransferase 5 OS=Mus musculus GN=Lpcat3 PE=1 SV=1 - [MBOA5_MOUSE]	5.95	2	2	3	7	-0.12	-0.04	-0.18	-0.11
A2AQV2	Carbohydrate sulfotransferase 14 OS=Mus musculus GN=Chst14 PE=2 SV=1 - [A2AQV2_MOUSE]	3.99	2	1	1	1	-0.31	-0.16	-0.27	-0.12
Q922M3	BTB/POZ domain-containing adapter for CUL3-mediated RhoA degradation protein 3 OS=Mus musculus GN=Kctd10 PE=1 SV=1 - [BACD3_MOUSE]	11.75	2	3	3	10	-0.02	0.02	0.12	0.10
H3BKD4	Arf-GAP with SH3 domain, ANK repeat and PH domain-containing protein 1 OS=Mus musculus GN=Asap1 PE=2 SV=1 - [H3BKD4_MOUSE]	5.49	11	4	4	5	-0.77	-0.90	-0.22	-0.60
Q9DBT4-2	Isoform 2 of Ectonucleoside triphosphate diphosphohydrolase 4 OS=Mus musculus GN=Entpd4 - [ENTP4_MOUSE]	6.61	6	3	3	8	-0.16	-0.05	0.11	0.01
Q9CQT1	Methylthioribose-1-phosphate isomerase OS=Mus musculus GN=Mri1 PE=2 SV=1 - [MTNA_MOUSE]	24.12	3	6	6	15	0.16	0.22	0.18	0.30
F8WIP7	Zinc finger protein ubi-d4 (Fragment) OS=Mus musculus GN=Dpf2 PE=4 SV=1 - [F8WIP7_MOUSE]	11.68	3	2	2	6	-0.30	-0.03	-0.07	-0.08
Q8R1H0	Homeodomain-only protein OS=Mus musculus GN=Hopx PE=1 SV=1 - [HOP_MOUSE]	13.70	1	2	2	7	0.04	-0.06	-0.09	-0.20
Q8C180	Fibroblast growth factor receptor substrate 2 OS=Mus musculus GN=Frs2 PE=1 SV=3 - [FRS2_MOUSE]	10.24	1	2	2	5	-0.25	-0.06	-0.22	-0.03
Q61136	Serine/threonine-protein kinase PRP4 homolog OS=Mus musculus GN=Prpf4b PE=1 SV=3 - [PRP4B_MOUSE]	8.94	1	7	8	16	-0.13	0.19	-0.20	0.25
Q62433	Protein NDRG1 OS=Mus musculus GN=Ndrg1 PE=1 SV=1 - [NDRG1_MOUSE]	28.68	11	7	7	68	0.13	0.37	0.15	0.23
Q5ND52	RNA methyltransferase-like protein 1 OS=Mus musculus GN=Rnmtl1 PE=2 SV=1 - [RMTL1_MOUSE]	1.67	1	1	1	3	0.11	-0.44	0.12	-0.44

Q60605	Myosin light polypeptide 6 OS=Mus musculus GN=Myl6 PE=1 SV=3 - [MYL6_MOUSE]	88.08	1	2	13	1591	-0.40	-0.21	-0.34	-0.43
Q9CZH3	Proteasome assembly chaperone 3 OS=Mus musculus GN=Psmg3 PE=1 SV=1 - [PSMG3_MOUSE]	42.62	1	3	3	8	-0.33	0.00	-0.40	-0.33
Q91X78	Erlin-1 OS=Mus musculus GN=Erlin1 PE=1 SV=1 - [ERLN1_MOUSE]	16.18	2	2	5	37	0.35	0.10	0.14	-1.01
Q9CQB4	Cytochrome b-c1 complex subunit 7 OS=Mus musculus GN=Uqcrb PE=3 SV=1 [Q9CQB4_MOUSE]	47.75	2	9	9	110	0.03	0.05	0.00	0.05
Q5VCS6	Tudor domain-containing protein 5 OS=Mus musculus GN=Tdrd5 PE=1 SV=3 [TDRD5_MOUSE]	2.12	4	2	2	5	0.33	0.33	0.25	0.25
Q99MY8	Histone-lysine N-methyltransferase ASH1L OS=Mus musculus GN=Ash1l PE=1 SV=3 - [ASH1L_MOUSE]	1.05	1	3	3	4	-0.20	0.97	0.71	0.96
I3ITR1	MCG50313 OS=Mus musculus GN=AK157302 PE=4 SV=1 - [I3ITR1_MOUSE]	19.38	2	2	2	2	-0.28	-0.02	-0.47	-0.21
Q9EP73	Programmed cell death 1 ligand 1 OS=Mus musculus GN=Cd274 PE=1 SV=1 - [PD1L1_MOUSE]	13.79	1	1	2	6	0.00	-0.03	-0.32	-0.35
Q99N05	Membrane-spanning 4-domains subfamily A member 4D OS=Mus musculus GN=Ms4a4d PE=2 SV=1 - [M4A4D_MOUSE]	2.67	1	1	1	2	-0.38	-0.33	-0.34	-0.29
Q02053	Ubiquitin-like modifier-activating enzyme 1 OS=Mus musculus GN=Uba1 PE=1 SV=1 - [UBA1_MOUSE]	44.80	2	37	37	652	0.30	0.29	0.28	0.26
Q9CPW2	Adrenodoxin-like protein, mitochondrial OS=Mus musculus GN=Fdx1l PE=2 SV=1 [ADXL_MOUSE]	6.32	1	1	1	3	-0.46	-0.44	-0.26	-0.25
Q9WVL6	Exostosin-like 3 OS=Mus musculus GN=Extl3 PE=2 SV=2 - [EXTL3_MOUSE]	3.70	2	2	4	10	-0.39	-0.67	-0.21	-0.49
Q8BMP6	Golgi resident protein GCP60 OS=Mus musculus GN=Acbd3 PE=1 SV=3 - [GCP60_MOUSE]	16.95	1	8	8	28	0.02	-0.05	-0.10	-0.11
E9Q8Z4	Catenin delta-1 OS=Mus musculus GN=Ctnnd1 PE=2 SV=1 - [E9Q8Z4_MOUSE]	40.67	12	1	31	223	0.62	0.06	0.33	-0.24
Q63829	COMM domain-containing protein 3 OS=Mus musculus GN=Commd3 PE=2 SV=1 - [COMD3_MOUSE]	21.03	1	4	4	9	-0.06	0.13	0.01	0.04

Q9D3G5	Syntaxin-11 OS=Mus musculus GN=Stx11 PE=2 SV=1 - [STX11_MOUSE]	5.57	1	1	1	1	0.43	0.07	0.33	-0.03
B2RVL6	Zinc finger CCHC domain-containing protein 24 OS=Mus musculus GN=Zcchc24 PE=2 SV=1 - [ZCH24_MOUSE]	13.69	1	2	2	2	-0.61	-1.40	-0.46	-1.25
P98083-2	Isoform p52Shc of SHC transforming protein 1 OS=Mus musculus GN=Shc1 - [SHC1_MOUSE]	14.50	7	4	4	9	-0.22	-0.04	-0.09	0.10
Q921C5	Protein bicaudal D homolog 2 OS=Mus musculus GN=Bicd2 PE=1 SV=1 - [BICD2_MOUSE]	6.10	4	4	5	8	0.01	0.00	0.02	0.01
Q9D1G1	Ras-related protein Rab-1B OS=Mus musculus GN=Rab1b PE=1 SV=1 - [RAB1B_MOUSE]	77.61	14	5	13	160	0.17	0.33	0.16	0.30
Q9D4H1	Exocyst complex component 2 OS=Mus musculus GN=Exoc2 PE=1 SV=1 - [EXOC2_MOUSE]	4.22	1	4	4	11	-0.14	-0.23	0.15	0.10
Q62504-3	Isoform 3 of Msx2-interacting protein OS=Mus musculus GN=Spen - [MINT_MOUSE]	1.38	5	3	3	4	-0.29	-0.57	-0.27	-0.21
O54825	Bystin OS=Mus musculus GN=Bysl PE=1 SV=3 - [BYST_MOUSE]	13.30	1	4	5	9	0.25	0.19	0.23	0.08
P97792-2	Isoform 2 of Coxsackievirus and adenovirus receptor homolog OS=Mus musculus GN=Cxadr - [CXAR_MOUSE]	17.90	3	6	6	17	0.13	-0.02	0.30	0.17
Q3UDD3	Polymerase delta-interacting protein 3 OS=Mus musculus GN=Poldip3 PE=2 SV=1 - [Q3UDD3_MOUSE]	26.09	3	7	7	22	-0.02	-0.14	0.08	0.07
Q8C8R3-7	Isoform 7 of Ankyrin-2 OS=Mus musculus GN=Ank2 - [ANK2_MOUSE]	20.93	2	1	8	24	0.03	-0.12	-0.13	-0.28
O88967	ATP-dependent zinc metalloprotease YME1L1 OS=Mus musculus GN=Yme1l1 PE=2 SV=1 - [YMEL1_MOUSE]	14.55	1	8	8	14	-0.07	-0.12	0.22	0.12
Q8BP01	Vimentin-type intermediate filament-associated coiled-coil protein OS=Mus musculus GN=Vmac PE=2 SV=1 - [VMAC_MOUSE]	28.74	1	3	4	15	-0.37	-0.40	0.10	-0.18
P26150	3 beta-hydroxysteroid dehydrogenase/Delta 5-->4-isomerase type 3 OS=Mus musculus GN=Hsd3b3 PE=2 SV=3 - [3BHS3_MOUSE]	20.38	8	4	5	20	-0.08	-0.24	-0.22	-0.45
Q9WTX2	Interferon-inducible double stranded RNA-dependent protein kinase activator A OS=Mus musculus GN=Prkra PE=1 SV=1 - [PRKRA_MOUSE]	15.65	1	4	4	17	0.20	0.05	0.24	0.04

P46096	Synaptotagmin-1 OS=Mus musculus GN=Syt1 PE=1 SV=1 - [SYT1_MOUSE]	24.23	3	9	11	35	-0.46	-0.52	-0.40	-0.43
Q91XD2	LIM and senescent cell antigen-like-containing domain protein 2 OS=Mus musculus GN=Lims2 PE=1 SV=1 - [LIMS2_MOUSE]	23.17	1	4	8	56	0.23	0.12	0.28	0.18
Q921M4-2	Isoform 2 of Golgin subfamily A member 2 OS=Mus musculus GN=Golga2 - [GOGA2_MOUSE]	31.23	10	18	20	73	-0.04	-0.18	-0.06	-0.19
I7HPW8	Protein Myo15b (Fragment) OS=Mus musculus GN=Myo15b PE=4 SV=1 - [I7HPW8_MOUSE]	11.26	4	18	18	62	0.25	0.18	0.19	0.12
Q5SWD9-3	Isoform 3 of Pre-rRNA-processing protein TSR1 homolog OS=Mus musculus GN=Tsr1 - [TSR1_MOUSE]	3.93	2	1	1	1	0.11	0.74	-0.04	0.58
E9PUP1	Protein Syde2 OS=Mus musculus GN=Syde2 PE=4 SV=2 - [E9PUP1_MOUSE]	2.28	1	1	2	2	-0.18	-0.82	0.55	-0.10
Q8C8I3	Eukaryotic translation initiation factor 2D OS=Mus musculus GN=Eif2d PE=2 SV=1 - [Q8C8I3_MOUSE]	4.86	4	2	2	2	0.60	0.39	0.37	0.15
Q9D2P4	Ubiquitin-related modifier 1 homolog OS=Mus musculus GN=Urm1 PE=1 SV=1 [URM1_MOUSE]	14.85	1	1	1	3	-0.17	-0.26	0.02	-0.06
D3YTM0	Ceramide synthase 2 (Fragment) OS=Mus musculus GN=Cers2 PE=2 SV=1 - [D3YTM0_MOUSE]	15.45	4	3	3	12	0.25	0.30	0.19	0.24
Q02819	Nucleobindin-1 OS=Mus musculus GN=Nucb1 PE=1 SV=2 - [NUCB1_MOUSE]	64.49	4	26	26	313	-0.17	-0.12	-0.10	-0.08
O88845-3	Isoform 3 of A-kinase anchor protein 10, mitochondrial OS=Mus musculus GN=Akap10 - [AKA10_MOUSE]	5.89	3	2	3	5	-0.03	0.02	-0.21	-0.16
O88851	Putative hydrolase RBBP9 OS=Mus musculus GN=Rbbp9 PE=1 SV=2 - [RBBP9_MOUSE]	50.54	1	7	7	45	0.65	0.63	0.50	0.51
Q9JK92	Heat shock protein beta-8 OS=Mus musculus GN=Hspb8 PE=1 SV=1 - [HSPB8_MOUSE]	41.84	1	3	3	7	-0.39	0.12	-0.11	0.13
O88895	Histone deacetylase 3 OS=Mus musculus GN=Hdac3 PE=1 SV=1 - [HDAC3_MOUSE]	11.32	3	4	4	7	0.12	0.19	0.24	0.42
Q80XI3-2	Isoform 2 of Eukaryotic translation initiation factor 4 gamma 3 OS=Mus musculus GN=Eif4g3 - [IF4G3_MOUSE]	9.81	10	8	12	35	-0.15	-0.15	0.35	0.06

Q9JKC6	Cell cycle exit and neuronal differentiation protein 1 OS=Mus musculus GN=Cend1 PE=1 SV=1 - [CEND_MOUSE]	8.72	1	1	1	4	-0.60	-0.36	-0.67	-0.44
Q62470-3	Isoform 3 of Integrin alpha-3 OS=Mus musculus GN=Itga3 - [ITA3_MOUSE]	5.28	3	6	6	26	-0.02	0.08	0.01	0.05
P08553	Neurofilament medium polypeptide OS=Mus musculus GN=Nefm PE=1 SV=4 - [NFM_MOUSE]	6.60	2	5	6	101	-0.82	-0.77	-0.56	-0.45
G5E8K5-3	Isoform 3 of Ankyrin-3 OS=Mus musculus GN=Ank3 - [ANK3_MOUSE]	17.85	24	20	22	57	-0.15	-0.18	-0.11	-0.11
D3YU17	Nicalin OS=Mus musculus GN=Ncln PE=2 SV=1 - [D3YU17_MOUSE]	16.01	3	10	10	58	0.20	0.06	0.14	-0.03
F6ZCF0	Cytosolic carboxypeptidase 1 (Fragment) OS=Mus musculus GN=Agtpbp1 PE=4 SV=1 - [F6ZCF0_MOUSE]	1.02	6	1	1	2	0.15	-0.02	0.23	0.05
Q9D2Y4-2	Isoform 2 of Mixed lineage kinase domain-like protein OS=Mus musculus GN=Mlkl - [MLKL_MOUSE]	15.09	3	6	6	23	0.10	0.02	0.22	0.09
Q91WE6-3	Isoform 3 of Threonylcarbamoyladenosine tRNA methylthiotransferase OS=Mus musculus GN=Cdkal1 - [CDKAL_MOUSE]	3.99	3	1	1	1	-0.75	-1.09	-0.45	-0.80
A8XY17	Vacuolar protein-sorting associated protein 25 OS=Mus musculus GN=Vps25 PE=2 SV=1 - [A8XY17_MOUSE]	72.66	6	7	7	47	0.35	0.38	0.25	0.42
Q920A5	Retinoid-inducible serine carboxypeptidase OS=Mus musculus GN=Scpep1 PE=2 SV=2 - [RISC_MOUSE]	15.49	1	6	6	37	0.24	0.36	0.25	0.44
Q8BTY1	Kynurenine--oxoglutarate transaminase 1 OS=Mus musculus GN=Ccbl1 PE=2 SV=1 [KAT1_MOUSE]	10.85	3	3	3	12	-0.34	-0.44	-0.60	-0.47
A2AB59-2	Isoform 2 of Rho GTPase-activating protein 27 OS=Mus musculus GN=Arhgap27 - [RHG27_MOUSE]	10.90	2	6	6	16	-0.31	-0.21	0.04	0.02
Q61033	Lamina-associated polypeptide 2, isoforms alpha/zeta OS=Mus musculus GN=Tmpo PE=1 SV=4 - [LAP2A_MOUSE]	33.19	1	10	18	329	0.27	0.26	0.32	0.24
P27808	Alpha-1,3-mannosyl-glycoprotein 2-beta-N-acetylglucosaminyltransferase OS=Mus musculus GN=Mgat1 PE=2 SV=1 - [MGAT1_MOUSE]	11.41	2	4	4	11	0.41	0.22	0.46	0.20

O08807	Peroxiredoxin-4 OS=Mus musculus GN=Prdx4 PE=1 SV=1 [PRDX4_MOUSE]	48.18	2	8	10	278	-0.06	0.02	-0.03	0.06
Q5ND34-3	Isoform 3 of WD repeat-containing protein 81 OS=Mus musculus GN=Wdr81 - [WDR81_MOUSE]	1.23	4	1	1	1	0.13	0.24	0.33	0.44
Q64737	Trifunctional purine biosynthetic protein adenosine-3 OS=Mus musculus GN=Gart PE=2 SV=3 - [PUR2_MOUSE]	25.15	3	22	22	94	0.32	0.36	0.30	0.31
Q8BM88	Cathepsin O OS=Mus musculus GN=Ctso PE=2 SV=1 - [CATO_MOUSE]	3.85	1	1	1	2	-0.58	-0.12	-0.54	-0.08
Q9JM73	Serum response factor OS=Mus musculus GN=Srf PE=1 SV=1 - [SRF_MOUSE]	2.38	1	1	1	1	0.10	0.92	-0.02	0.79
Q9CQQ7	ATP synthase subunit b mitochondrial OS=Mus musculus GN=Atp5f1 PE=1 SV=1 - [AT5F1_MOUSE]	36.72	1	10	11	196	0.02	0.13	-0.03	0.10
F8VQM1	G-protein-coupled receptor 161 OS=Mus musculus GN=Gpr161 PE=2 SV=1 - [F8VQM1_MOUSE]	1.29	4	1	1	7	0.57	-0.58	0.47	-0.67
Q80X80	C2 domain-containing protein 2-like OS=Mus musculus GN=C2cd2l PE=1 SV=3 - [C2C2L_MOUSE]	7.37	1	4	4	9	-0.40	-0.13	-0.29	-0.18
D3Z5K6	Mitochondrial import inner membrane translocase subunit TIM14 OS=Mus musculus GN=Dnajc19 PE=2 SV=1 - [D3Z5K6_MOUSE]	43.64	5	5	5	7	0.29	-0.18	0.21	-0.27
E9Q5E0	Myocyte-specific enhancer factor 2D OS=Mus musculus GN=Mef2d PE=2 SV=1 - [E9Q5E0_MOUSE]	6.72	5	2	2	3	-0.09	-0.07	0.00	0.02
G5E8T0	DnaJ (Hsp40) homolog, subfamily C, member 5, isoform CRA_b OS=Mus musculus GN=Dnajc5 PE=4 SV=1 - [G5E8T0_MOUSE]	34.13	3	3	3	34	0.02	0.35	-0.16	0.20
Q61712	DnaJ homolog subfamily C member 1 OS=Mus musculus GN=Dnajc1 PE=1 SV=1 - [DNJC1_MOUSE]	9.60	3	5	5	13	0.18	0.26	0.18	0.26
Q921T2-2	Isoform 2 of Torsin-1A-interacting protein 1 OS=Mus musculus GN=Tor1aip1 - [TOIP1_MOUSE]	26.22	8	10	10	80	-0.17	-0.17	-0.12	-0.17
G3X8U9	Mitogen activated protein kinase 8, isoform CRA_b OS=Mus musculus GN=Mapk8 PE=4 SV=1 - [G3X8U9_MOUSE]	19.91	13	2	4	42	0.38	0.58	0.41	0.61

D3Z7E5	Glycogen synthase kinase-3 alpha OS=Mus musculus GN=Gsk3a PE=2 SV=1 - [D3Z7E5_MOUSE]	18.31	2	3	8	27	0.20	-0.71	0.14	-0.57
F6RBL8	Intraflagellar transport protein 52 homolog (Fragment) OS=Mus musculus GN=Ift52 PE=2 SV=1 - [F6RBL8_MOUSE]	2.56	2	1	1	1	-0.15	0.14	-0.24	0.05
D3Z6C3	40S ribosomal protein S3a OS=Mus musculus GN=Rps3a2 PE=3 SV=1 - [D3Z6C3_MOUSE]	44.70	3	14	14	277	0.36	0.42	0.30	0.36
Q8BFW7	Lipoma-preferred partner homolog OS=Mus musculus GN=Lpp PE=1 SV=1 - [LPP_MOUSE]	57.91	4	8	24	778	0.20	0.28	0.18	0.24
Q8BWU5	Probable tRNA threonylcarbamoyladenosine biosynthesis protein Osgep OS=Mus musculus GN=Osgep PE=2 SV=2 - [OSGEP_MOUSE]	18.21	5	4	5	15	0.20	0.19	0.23	0.32
F6RJ39	Apoptotic chromatin condensation inducer in the nucleus (Fragment) OS=Mus musculus GN=Acin1 PE=4 SV=1 [F6RJ39_MOUSE]	20.52	9	13	25	66	0.03	0.08	-0.07	0.06
Q3UVG3	Protein FAM91A1 OS=Mus musculus GN=Fam91a1 PE=1 SV=1 - [F91A1_MOUSE]	3.23	4	2	3	6	0.43	0.52	0.30	0.44
E9Q3M9	Protein 2010300C02Rik OS=Mus musculus GN=2010300C02Rik PE=2 SV=1 - [E9Q3M9_MOUSE]	3.83	1	1	3	85	-0.33	0.44	-0.52	0.25
Q8BQB5	Protein 6430548M08Rik OS=Mus musculus GN=6430548M08Rik PE=2 SV=1 - [Q8BQB5_MOUSE]	4.04	3	1	1	1	0.37	0.29	0.50	0.42
Q9ER88	28S ribosomal protein S29, mitochondrial OS=Mus musculus GN=Dap3 PE=2 SV=1 - [RT29_MOUSE]	18.67	9	6	6	19	-0.15	-0.48	0.10	-0.27
E9Q9E1	Eukaryotic translation initiation factor 4 gamma 1 OS=Mus musculus GN=Eif4g1 PE=2 SV=1 - [E9Q9E1_MOUSE]	29.38	10	30	35	191	0.35	0.32	0.27	0.34
Q9D7B1	tRNA-dihydrouridine(20) synthase [NAD(P)+]-like OS=Mus musculus GN=Dus2l PE=1 SV=1 [DUS2L_MOUSE]	6.69	2	2	2	6	-0.02	-0.04	-0.13	-0.06
Q9D0I9	Arginine--tRNA ligase, cytoplasmic OS=Mus musculus GN=Rars PE=2 SV=2 - [SYRC_MOUSE]	29.70	1	20	20	108	0.25	0.17	0.26	0.21
Q8K310	Matrin-3 OS=Mus musculus GN=Matr3 PE=1 SV=1 - [MATR3_MOUSE]	21.87	4	16	16	71	-0.01	-0.11	-0.14	-0.08

B2RXW8	Ppfia1 protein OS=Mus musculus GN=Ppfia1 PE=2 SV=1 - [B2RXW8_MOUSE]	5.80	3	6	8	27	-0.18	-0.15	0.08	0.00
Q3URQ4	Protein C78339 OS=Mus musculus GN=C78339 PE=2 SV=1 - [Q3URQ4_MOUSE]	5.26	1	1	1	1	-0.17	-0.30	0.19	0.05
P51807	Dynein light chain Tctex-type 1 OS=Mus musculus GN=Dynlt1 PE=1 SV=1 - [DYLT1_MOUSE]	14.16	1	1	1	2	0.39	0.35	0.32	0.28
Q9DBX5	Cytosolic phospholipase A2 OS=Mus musculus GN=Pla2g4a PE=2 SV=1 - [Q9DBX5_MOUSE]	16.49	2	12	12	31	0.30	0.34	0.33	0.32
P14483	H-2 class II histocompatibility antigen, A beta chain OS=Mus musculus GN=H2-Ab1 PE=1 SV=1 - [HB2A_MOUSE]	34.34	7	7	7	101	-0.14	-0.04	-0.12	-0.15
Q8BKI2	Trinucleotide repeat-containing gene 6B protein OS=Mus musculus GN=Tnrc6b PE=2 SV=2 - [TNR6B_MOUSE]	4.03	2	5	5	9	-0.11	-0.09	0.08	0.11
Q9D1M0	Protein SEC13 homolog OS=Mus musculus GN=Sec13 PE=2 SV=3 [SEC13_MOUSE]	42.55	1	12	12	64	-0.05	-0.10	-0.07	-0.03
F6RUV8	Protein AA386476 OS=Mus musculus GN=AA386476 PE=4 SV=2 - [F6RUV8_MOUSE]	12.84	1	1	1	1	0.21	0.19	0.36	0.34
A2A7R1	Transmembrane protein 54 OS=Mus musculus GN=Tmem54 PE=4 SV=1 - [A2A7R1_MOUSE]	5.41	3	1	1	1	-0.21	-0.40	-0.47	-0.66
Q8C9R9	EF-hand calcium-binding domain-containing protein 8 OS=Mus musculus GN=Efcab8 PE=2 SV=1 - [EFCB8_MOUSE]	8.37	1	1	1	1	-0.76	-0.47	-0.12	0.17
Q3V3R1	Monofunctional C1-tetrahydrofolate synthase, mitochondrial OS=Mus musculus GN=Mthfd1l PE=1 SV=2 - [C1TM_MOUSE]	26.20	1	20	20	73	0.33	0.31	0.25	0.22
E9Q8N8	Electrogenic sodium bicarbonate cotransporter 1 OS=Mus musculus GN=Slc4a4 PE=2 SV=1 - [E9Q8N8_MOUSE]	5.67	5	4	4	7	-0.23	-0.17	-0.23	-0.22
Q8BWQ5	Serine/threonine-protein kinase DCLK3 OS=Mus musculus GN=Dclk3 PE=2 SV=2 [DCLK3_MOUSE]	1.77	1	1	1	1	-0.40	-0.44	-0.35	-0.40
P23591	GDP-L-fucose synthase OS=Mus musculus GN=Tsta3 PE=2 SV=3 - [FCL_MOUSE]	42.06	1	9	10	71	0.14	0.09	0.11	0.11

Q9D7J9	Enoyl-CoA hydratase domain-containing protein 3, mitochondrial OS=Mus musculus GN=Echdc3 PE=2 SV=1 - [ECHD3_MOUSE]	13.67	2	3	3	12	-0.34	0.05	-0.36	0.03
E9Q7N5	Ribosome-releasing factor 2, mitochondrial OS=Mus musculus GN=Gfm2 PE=2 SV=1 [E9Q7N5_MOUSE]	12.28	6	7	7	24	0.17	0.18	0.17	0.18
Q8CAI1-2	Isoform 2 of Coiled-coil domain-containing protein 142 OS=Mus musculus GN=Ccdc142 [CC142_MOUSE]	6.34	2	1	1	3	-0.24	0.07	-0.23	0.08
Q9D273	Cob(I)yrinic acid a,c-diamide adenosyltransferase, mitochondrial OS=Mus musculus GN=Mmab PE=2 SV=1 - [MMAB_MOUSE]	13.92	3	3	3	16	-0.06	-0.39	0.03	-0.18
Q8BW94-2	Isoform 2 of Dynein heavy chain 3, axonemal OS=Mus musculus GN=Dnah3 - [DYH3_MOUSE]	1.19	3	4	4	6	0.10	0.02	-0.16	-0.18
P60710	Actin, cytoplasmic 1 OS=Mus musculus GN=Actb PE=1 SV=1 - [ACTB_MOUSE]	72.00	10	4	28	5121	0.22	0.16	0.23	0.15
P47739	Aldehyde dehydrogenase, dimeric NADP-preferring OS=Mus musculus GN=Aldh3a1 PE=2 SV=2 - [AL3A1_MOUSE]	6.40	1	2	3	10	-1.08	-1.43	0.51	0.15
Q8R395	COMM domain-containing protein 5 OS=Mus musculus GN=Commd5 PE=2 SV=1 - [COMD5_MOUSE]	7.14	1	1	1	2	0.34	0.14	0.47	0.27
Q8BFP9	[Pyruvate dehydrogenase [lipoamide]] kinase isozyme 1, mitochondrial OS=Mus musculus GN=Pdk1 PE=2 SV=2 - [PDK1_MOUSE]	8.29	1	2	3	3	0.34	0.23	0.05	-0.06
Q9JLZ6-2	Isoform 2 of Hypermethylated in cancer 2 protein OS=Mus musculus GN=Hic2 - [HIC2_MOUSE]	1.77	2	1	1	24	-0.18	-0.28	-0.10	-0.20
Q8BUH2	Nucleoside diphosphate kinase 7 OS=Mus musculus GN=Nme7 PE=2 SV=1 - [Q8BUH2_MOUSE]	3.25	2	1	1	2	-0.39	-0.45	-0.32	-0.37
Q8BYY4-2	Isoform 2 of Tetratricopeptide repeat protein 39B OS=Mus musculus GN=Ttc39b - [TT39B_MOUSE]	6.56	5	3	3	6	-0.05	-0.21	0.01	-0.15

P63328-2	Isoform 2 of Serine/threonine-protein phosphatase 2B catalytic subunit alpha isoform OS=Mus musculus GN=Ppp3ca - [PP2BA_MOUSE]	16.83	3	4	9	31	0.24	0.18	0.18	0.19
P62315	Small nuclear ribonucleoprotein Sm D1 OS=Mus musculus GN=Snrpd1 PE=1 SV=1 - [SMD1_MOUSE]	27.73	1	2	2	16	-0.29	-0.43	-0.22	-0.23
Q8VHK1	Caskin-2 OS=Mus musculus GN=Caskin2 PE=1 SV=3 - [CSKI2_MOUSE]	5.25	1	3	3	6	-0.35	-0.10	-0.25	0.00
P19536	Cytochrome c oxidase subunit 5B, mitochondrial OS=Mus musculus GN=Cox5b PE=1 SV=1 - [COX5B_MOUSE]	42.97	2	7	7	297	-0.07	0.03	-0.14	-0.07
P98191	Phosphatidate cytidylyltransferase 1 OS=Mus musculus GN=Cds1 PE=1 SV=2 - [CDS1_MOUSE]	4.99	1	2	2	6	0.06	0.37	0.40	0.23
P21279	Guanine nucleotide-binding protein G(q) subunit alpha OS=Mus musculus GN=Gnaq PE=1 SV=4 - [GNAQ_MOUSE]	37.88	2	6	11	117	-0.19	-0.23	-0.16	-0.29
Q8C0M9	Isoaspartyl peptidase/L-asparaginase OS=Mus musculus GN=Asrgl1 PE=1 SV=1 - [ASGL1_MOUSE]	14.42	1	4	4	12	-0.24	-0.08	-0.24	-0.57
Q8BHC4	Dephospho-CoA kinase domain-containing protein OS=Mus musculus GN=Dcakd PE=2 SV=1 - [DCAKD_MOUSE]	12.55	2	4	4	10	-0.01	-0.31	-0.24	-0.55
Q8VD75	Huntingtin-interacting protein 1 OS=Mus musculus GN=Hip1 PE=1 SV=2 - [HIP1_MOUSE]	3.79	1	3	4	9	0.15	0.31	0.11	0.23
Q8BM75-2	Isoform 2 of AT-rich interactive domain-containing protein 5B OS=Mus musculus GN=Arid5b - [ARI5B_MOUSE]	2.96	2	2	2	3	0.12	-0.11	0.09	-0.15
Q02248	Catenin beta-1 OS=Mus musculus GN=Ctnnb1 PE=1 SV=1 - [CTNB1_MOUSE]	40.46	8	17	21	198	0.12	0.09	0.06	0.09
Q7TPW1	Nexilin OS=Mus musculus GN=Nexn PE=1 SV=3 - [NEXN_MOUSE]	15.16	1	9	10	45	-0.04	-0.05	-0.03	0.07
P97363	Serine palmitoyltransferase 2 OS=Mus musculus GN=Sptlc2 PE=2 SV=2 - [SPTC2_MOUSE]	25.36	1	11	11	27	0.24	0.57	0.31	0.46
Q3U2A8	Valine--tRNA ligase, mitochondrial OS=Mus musculus GN=Vars2 PE=2 SV=2 - [SYVM_MOUSE]	3.40	1	4	4	7	0.11	-0.22	0.20	-0.10

P60843	Eukaryotic initiation factor 4A-I OS=Mus musculus GN=Eif4a1 PE=2 SV=1 - [IF4A1_MOUSE]	51.97	3	9	22	476	0.34	0.53	0.23	0.46
A2AJI0	MAP7 domain-containing protein 1 OS=Mus musculus GN=Map7d1 PE=1 SV=1 - [MA7D1_MOUSE]	16.67	5	9	10	16	-0.13	-0.31	-0.49	0.04
P25206	DNA replication licensing factor MCM3 OS=Mus musculus GN=Mcm3 PE=1 SV=2 - [MCM3_MOUSE]	28.57	1	16	16	68	0.46	0.30	0.38	0.46
Q61187	Tumor susceptibility gene 101 protein OS=Mus musculus GN=Tsg101 PE=1 SV=2 - [TS101_MOUSE]	24.04	3	8	8	31	0.17	0.00	0.16	-0.03
O88327	Alpha-catulin OS=Mus musculus GN=Ctnnal1 PE=2 SV=1 - [CTNL1_MOUSE]	3.28	2	3	3	8	0.13	0.13	-0.08	-0.06
Q3UN02	Lysocardiolipin acyltransferase 1 OS=Mus musculus GN=Lclat1 PE=2 SV=2 - [LCLT1_MOUSE]	17.82	2	6	6	27	0.09	0.06	0.18	-0.01
B1AUH6	Peripheral plasma membrane protein CASK OS=Mus musculus GN=Cask PE=2 SV=1 - [B1AUH6_MOUSE]	11.71	1	1	1	1	-0.18	-1.17	-0.18	-1.17
P63094-2	Isoform Gnas-2 of Guanine nucleotide-binding protein G(s) subunit alpha isoforms short OS=Mus musculus GN=Gnas - [GNAS2_MOUSE]	23.42	7	8	10	77	-0.10	-0.22	-0.27	-0.36
Q6PDY2	2-aminoethanethiol dioxygenase OS=Mus musculus GN=Ado PE=1 SV=2 - [AEDO_MOUSE]	24.22	1	4	4	47	0.16	-0.19	0.04	-0.11
Q60855	Receptor-interacting serine/threonine-protein kinase 1 OS=Mus musculus GN=Ripk1 PE=1 SV=1 - [RIPK1_MOUSE]	14.63	2	8	8	17	0.01	0.12	0.16	0.15
Q91Y97	Fructose-bisphosphate aldolase B OS=Mus musculus GN=Aldob PE=1 SV=3 - [ALDOB_MOUSE]	65.38	1	17	18	216	-0.30	-0.28	-0.33	-0.31
P56371	Ras-related protein Rab-4A OS=Mus musculus GN=Rab4a PE=1 SV=2 - [RAB4A_MOUSE]	19.72	11	2	4	35	-0.35	-0.54	-0.17	-0.39
Q8K1M3	Protein kinase, cAMP dependent regulatory, type II alpha OS=Mus musculus GN=Prkar2a PE=2 SV=1 - [Q8K1M3_MOUSE]	48.01	2	13	16	124	-0.04	-0.09	0.00	0.08
Q9JKY5	Huntingtin-interacting protein 1-related protein OS=Mus musculus GN=Hip1r PE=1 SV=2 [HIP1R_MOUSE]	9.36	3	9	11	25	0.30	0.25	0.25	0.20

F8WHV1	Mitotic spindle-associated MMXD complex subunit MIP18 OS=Mus musculus GN=Fam96b PE=2 SV=1 - [F8WHV1_MOUSE]	20.59	2	2	2	14	-0.33	0.03	-0.13	-0.04
Q9EPJ9	ADP-ribosylation factor GTPase-activating protein 1 OS=Mus musculus GN=Arfgap1 PE=1 SV=2 - [ARFG1_MOUSE]	25.36	3	7	7	25	0.06	0.01	0.17	0.19
Q9ES56	Trafficking protein particle complex subunit 4 OS=Mus musculus GN=Trappc4 PE=1 SV=1 - [TPPC4_MOUSE]	9.13	1	2	2	6	-0.28	-0.19	0.27	-0.20
Q7TQK4	Exosome complex component RRP40 OS=Mus musculus GN=Exosc3 PE=1 SV=3 - [EXOS3_MOUSE]	18.61	1	2	2	7	-0.02	0.04	0.03	0.09
Q9JHL1	Na(+)/H(+) exchange regulatory cofactor NHE-RF2 OS=Mus musculus GN=Slc9a3r2 PE=1 SV=2 - [NHRF2_MOUSE]	32.64	2	8	8	39	-0.18	-0.15	-0.31	-0.32
Q8BJU9	Peptide chain release factor 1-like, mitochondrial OS=Mus musculus GN=Mtrf1l PE=2 SV=1 - [RF1ML_MOUSE]	14.48	1	3	4	10	-0.27	-1.01	-0.24	-1.00
Q60953	Protein PML OS=Mus musculus GN=Pml PE=1 SV=3 - [PML_MOUSE]	12.54	5	7	7	9	-0.64	-0.48	-0.11	-0.45
Q8K2C9	Very-long-chain (3R)-3-hydroxyacyl-[acyl-carrier protein] dehydratase 3 OS=Mus musculus GN=ptplad1 PE=1 SV=2 - [HACD3_MOUSE]	16.30	1	4	5	13	-0.07	-0.07	0.20	-0.08
O88935-1	Isoform Ib of Synapsin-1 OS=Mus musculus GN=Syn1 - [SYN1_MOUSE]	16.87	3	7	7	21	-0.68	-0.34	-0.43	-0.28
P06537-3	Isoform 1-B of Glucocorticoid receptor OS=Mus musculus GN=Nr3c1 - [GCR_MOUSE]	4.89	8	3	3	4	-0.02	-0.33	0.16	-0.15
A2A6M1	Vacuolar-sorting protein SNF8 OS=Mus musculus GN=Snf8 PE=2 SV=1 - [A2A6M1_MOUSE]	15.02	2	3	3	19	0.25	0.28	0.34	0.29
Q01815-2	Isoform 2 of Voltage-dependent L-type calcium channel subunit alpha-1C OS=Mus musculus GN=Cacna1c [CAC1C_MOUSE]	1.83	7	2	3	3	-0.47	-0.68	-0.02	-0.22
Q8BWY3	Eukaryotic peptide chain release factor subunit 1 OS=Mus musculus GN=Etf1 PE=1 SV=4 - [ERF1_MOUSE]	34.10	1	12	12	46	0.24	0.32	0.25	0.34

B1AYU4	Pre-mRNA-splicing factor CWC22 homolog OS=Mus musculus GN=Cwc22 PE=2 SV=1 - [B1AYU4_MOUSE]	2.77	8	2	2	5	0.07	0.42	-0.13	0.22
P54729	NEDD8 ultimate buster 1 OS=Mus musculus GN=Nub1 PE=1 SV=2 - [NUB1_MOUSE]	22.64	1	12	12	56	0.14	0.23	0.12	0.21
A6H584	Collagen alpha-5(VI) chain OS=Mus musculus GN=Col6a5 PE=1 SV=2 - [CO6A5_MOUSE]	2.50	2	4	6	10	0.50	0.26	0.52	0.07
Q8R3L2	Transcription factor 25 OS=Mus musculus GN=Tcf25 PE=1 SV=2 [TCF25_MOUSE]	9.76	5	3	3	4	0.06	0.21	0.01	0.16
F6SMJ7	GTP-binding protein 8 (Fragment) OS=Mus musculus GN=Gtpbp8 PE=4 SV=1 - [F6SMJ7_MOUSE]	20.41	4	2	2	7	-0.29	-0.38	-0.03	-0.02
Q6P2K6	Serine/threonine-protein phosphatase 4 regulatory subunit 3A OS=Mus musculus GN=Smek1 PE=1 SV=1 - [P4R3A_MOUSE]	5.98	4	2	5	8	0.55	0.61	0.38	0.44
Q5IXF8	Glucagon-like peptide 2 receptor OS=Mus musculus GN=Glp2r PE=2 SV=2 - [GLP2R_MOUSE]	2.73	1	1	1	3	0.31	-2.13	0.44	-2.00
Q0V8T7	Contactin-associated protein like 5-3 OS=Mus musculus GN=Cntnap5c PE=2 SV=1 - [CTP5C_MOUSE]	1.69	1	1	1	16	-0.59	-0.46	-0.53	-0.33
P53994	Ras-related protein Rab-2A OS=Mus musculus GN=Rab2a PE=1 SV=1 - [RAB2A_MOUSE]	59.43	1	4	10	188	0.16	0.26	0.23	0.33
Q8BJD1	Inter-alpha-trypsin inhibitor heavy chain H5 OS=Mus musculus GN=Itih5 PE=2 SV=1 - [ITIH5_MOUSE]	16.81	2	15	15	63	-0.25	-0.39	-0.29	-0.37
Q99PV0	Pre-mRNA-processing-splicing factor 8 OS=Mus musculus GN=Prpf8 PE=1 SV=2 - [PRP8_MOUSE]	21.24	2	41	42	176	0.21	0.19	0.21	0.21
F8VQK5	SAM and SH3 domain-containing protein 1 OS=Mus musculus GN=Sash1 PE=2 SV=1 [F8VQK5_MOUSE]	5.20	2	5	6	8	-0.25	0.31	-0.22	0.34
Q9ET22	Dipeptidyl peptidase 2 OS=Mus musculus GN=Dpp7 PE=2 SV=2 - [DPP2_MOUSE]	5.53	1	2	2	3	-1.52	-0.44	-1.48	-0.41
Q9JLB2	MAGUK p55 subfamily member 5 OS=Mus musculus GN=Mpp5 PE=1 SV=1 - [MPP5_MOUSE]	17.48	1	8	8	15	0.11	0.13	0.14	0.18
Q9D7H3	RNA 3'-terminal phosphate cyclase OS=Mus musculus GN=RtcA PE=2 SV=2 - [RTCA_MOUSE]	23.50	4	6	6	14	-0.21	-0.13	-0.29	-0.11

Q5EBG6	Heat shock protein beta-6 OS=Mus musculus GN=Hspb6 PE=1 SV=1 - [HSPB6_MOUSE]	14.81	1	2	2	14	-0.19	0.30	0.00	0.60
A2AKH7	Leucine-rich repeat-containing protein 57 (Fragment) OS=Mus musculus GN=Lrrc57 PE=2 SV=1 - [A2AKH7_MOUSE]	12.33	4	3	3	7	0.17	0.15	0.16	0.28
Q6P5U7	Leucine-rich repeat and WD repeat-containing protein KIAA1239 OS=Mus musculus GN=Kiaa1239 PE=2 SV=2 - [K1239_MOUSE]	0.34	1	1	1	1	-0.06	0.29	-0.10	0.24
J3QK23	Uncharacterized protein OS=Mus musculus GN=Gm9825 PE=4 SV=1 - [J3QK23_MOUSE]	19.15	3	3	3	14	-0.01	-0.15	0.08	-0.03
E9PUK8	Ephrin type-A receptor 6 OS=Mus musculus GN=Epha6 PE=2 SV=1 - [E9PUK8_MOUSE]	13.18	10	1	3	4	0.64	0.06	0.64	0.06
Q8VCA8	Secernin-2 OS=Mus musculus GN=Scrn2 PE=2 SV=1 - [SCRN2_MOUSE]	26.82	2	7	7	21	-0.53	-0.49	-0.34	-0.19
Q5SQP1-2	Isoform 2 of HORMA domain-containing protein 2 OS=Mus musculus GN=Hormad2 - [HORM2_MOUSE]	7.29	2	1	1	1	-0.58	-0.78	-0.40	-0.60
E9PYK7	Contactin-5 OS=Mus musculus GN=Cntn5 PE=4 SV=1 - [E9PYK7_MOUSE]	3.36	2	2	3	4	-0.41	-0.38	0.07	0.10
Q8BY71	Histone acetyltransferase type B catalytic subunit OS=Mus musculus GN=Hat1 PE=2 SV=1 - [HAT1_MOUSE]	6.73	2	3	3	4	0.04	0.80	0.20	0.95
Q61550	Double-strand-break repair protein rad21 homolog OS=Mus musculus GN=Rad21 PE=1 SV=3 - [RAD21_MOUSE]	17.64	1	8	8	42	0.01	-0.07	-0.02	-0.05
Q07813	Apoptosis regulator BAX OS=Mus musculus GN=Bax PE=1 SV=1 - [BAX_MOUSE]	23.44	1	4	4	18	0.15	0.16	0.21	0.20
D3YZZ5	Protein Tmed7 OS=Mus musculus GN=Tmed7 PE=2 SV=1 - [D3YZZ5_MOUSE]	26.34	2	4	5	16	0.06	-0.30	0.12	-0.18
P15208	Insulin receptor OS=Mus musculus GN=Insr PE=1 SV=2 - [INSR_MOUSE]	3.28	1	5	5	13	-0.19	-0.42	-0.13	-0.36
P59759	MKL/myocardin-like protein 2 OS=Mus musculus GN=Mkl2 PE=1 SV=1 - [MKL2_MOUSE]	11.30	4	6	9	43	0.16	0.24	0.11	0.19
D3YZY3	Transcription cofactor vestigial-like protein 4 (Fragment) OS=Mus musculus GN=Vgll4 PE=2 SV=1 - [D3YZY3_MOUSE]	11.11	3	2	2	3	-0.10	-0.29	0.07	-0.12

Q9DCS3	Trans-2-enoyl-CoA reductase, mitochondrial OS=Mus musculus GN=Mecr PE=2 SV=2 - [MECR_MOUSE]	33.51	3	9	10	113	-0.08	-0.13	0.01	-0.07
Q6NSW3-4	Isoform 4 of A-kinase anchor protein SPHKAP OS=Mus musculus GN=Sphkap - [SPKAP_MOUSE]	5.49	5	3	3	4	0.01	0.32	0.11	0.41
Q8JZR0	Long-chain-fatty-acid--CoA ligase 5 OS=Mus musculus GN=Acsl5 PE=2 SV=1 - [ACSL5_MOUSE]	13.62	1	6	8	19	0.23	-0.07	0.00	-0.25
Q8BGH2	Sorting and assembly machinery component 50 homolog OS=Mus musculus GN=Samm50 PE=1 SV=1 - [SAM50_MOUSE]	29.42	1	11	11	64	-0.28	-0.29	-0.26	-0.26
Q60673	Receptor-type tyrosine-protein phosphatase-like N OS=Mus musculus GN=Ptprn PE=2 SV=2 - [PTPRN_MOUSE]	7.05	2	4	4	8	-0.81	-0.76	-0.81	-0.79
Q8R1K1	Ubiquitin-associated domain-containing protein 2 OS=Mus musculus GN=Ubac2 PE=2 SV=1 - [UBAC2_MOUSE]	6.96	1	2	3	5	-0.22	-1.06	-0.12	-0.97
F8VPR1	Leiomodin-1 OS=Mus musculus GN=Lmod1 PE=2 SV=1 - [F8VPR1_MOUSE]	32.94	2	18	18	167	0.03	0.10	0.06	0.09
O35083	1-acyl-sn-glycerol-3-phosphate acyltransferase alpha OS=Mus musculus GN=Agpat1 PE=2 SV=1 - [PLCA_MOUSE]	15.09	3	2	3	11	0.12	0.00	0.06	0.09
P49138	MAP kinase-activated protein kinase 2 OS=Mus musculus GN=Mapkapk2 PE=1 SV=2 - [MAPK2_MOUSE]	22.80	4	3	6	9	0.31	-0.28	0.45	-0.13
P22315	Ferrochelatase, mitochondrial OS=Mus musculus GN=Fech PE=1 SV=2 - [HEMH_MOUSE]	24.05	2	10	10	39	-0.38	-0.32	-0.20	-0.20
P42669	Transcriptional activator protein Pur-alpha OS=Mus musculus GN=Pura PE=1 SV=1 - [PURA_MOUSE]	51.40	3	9	11	109	0.01	0.12	-0.02	0.13
Q9CWW6	Peptidyl-prolyl cis-trans isomerase NIMA-interacting 4 OS=Mus musculus GN=Pin4 PE=2 SV=1 - [PIN4_MOUSE]	48.09	1	4	4	13	0.00	0.13	0.33	0.38
Q91W52	Transmembrane protein 19 OS=Mus musculus GN=Tmem19 PE=2 SV=1 - [TMM19_MOUSE]	5.95	2	2	2	5	0.01	0.08	0.38	0.12
Q8CE96-3	Isoform 3 of tRNA (adenine(58)-N(1))-methyltransferase non-catalytic subunit TRM6 OS=Mus musculus GN=Trmt6 - [TRM6_MOUSE]	2.23	2	1	1	1	0.27	0.11	0.33	0.16

Q7M6Y3-5	Isoform 5 of Phosphatidylinositol-binding clathrin assembly protein OS=Mus musculus GN=Picalm - [PICAL_MOUSE]	30.08	6	13	14	79	0.17	-0.06	0.09	-0.02
Q9QUJ7-2	Isoform Short of Long-chain-fatty-acid--CoA ligase 4 OS=Mus musculus GN=Acsl4 - [ACSL4_MOUSE]	4.03	2	1	2	4	-0.20	-0.68	0.18	-0.31
Q69ZK7	Protein FAM214A OS=Mus musculus GN=Fam214a PE=2 SV=3 - [F214A_MOUSE]	1.12	2	1	1	1	0.08	-0.37	0.52	0.07
O88451	Retinol dehydrogenase 7 OS=Mus musculus GN=Rdh7 PE=2 SV=1 - [RDH7_MOUSE]	5.38	10	2	2	4	0.12	-0.04	0.30	0.01
Q9QXV0	ProSAAS OS=Mus musculus GN=Pcsk1n PE=1 SV=2 - [PCSK1_MOUSE]	22.48	1	4	4	23	-0.46	-0.72	-0.35	-0.45
P12023-3	Isoform APP751 of Amyloid beta A4 protein OS=Mus musculus GN=App - [A4_MOUSE]	8.44	3	5	6	8	0.27	0.17	0.21	0.10
Q8BH65-2	Isoform 2 of Protein DENND6A OS=Mus musculus GN=Dennd6a - [DEN6A_MOUSE]	6.62	2	1	1	6	0.84	2.20	0.42	1.79
Q9QYA2	Mitochondrial import receptor subunit TOM40 homolog OS=Mus musculus GN=Tomm40 PE=1 SV=3 - [TOM40_MOUSE]	28.25	2	7	7	47	0.04	0.33	0.06	0.20
Q3UZ39	Leucine-rich repeat flightless-interacting protein 1 OS=Mus musculus GN=Lrrfip1 PE=1 SV=2 - [LRRF1_MOUSE]	27.85	2	7	15	96	0.26	0.16	0.23	0.13
D3Z069	Pleckstrin homology-like domain family B member 2 (Fragment) OS=Mus musculus GN=Phldb2 PE=2 SV=1 - [D3Z069_MOUSE]	3.45	5	3	3	4	-0.09	-0.17	0.07	-0.01
Q8R146-2	Isoform 2 of Acylamino acid-releasing enzyme OS=Mus musculus GN=Apeh - [APEH_MOUSE]	35.56	2	19	19	166	-0.16	-0.17	-0.11	-0.14
Q99MT2	MutS protein homolog 4 OS=Mus musculus GN=Msh4 PE=2 SV=1 [MSH4_MOUSE]	0.73	1	1	1	5	0.38	-0.02	0.47	0.06
P16627	Heat shock 70 kDa protein 1-like OS=Mus musculus GN=Hspa1l PE=2 SV=4 - [HS71L_MOUSE]	31.05	1	2	21	582	0.04	0.00	-0.04	-0.19
Q9DBH0	NEDD4-like E3 ubiquitin-protein ligase WWP2 OS=Mus musculus GN=Wwp2 PE=1 SV=1 - [WWP2_MOUSE]	6.67	1	5	5	9	0.08	0.29	0.01	0.23
Q9EQ61	Pescadillo homolog OS=Mus musculus GN=Pes1 PE=1 SV=1 - [PESC_MOUSE]	1.54	2	1	1	2	0.18	0.09	0.39	0.30

E9PYK3	Protein Parp4 OS=Mus musculus GN=Parp4 PE=4 SV=1 - [E9PYK3_MOUSE]	9.60	1	13	14	50	0.23	0.11	0.23	0.23
Q99JY0	Trifunctional enzyme subunit beta, mitochondrial OS=Mus musculus GN=Hadhb PE=1 SV=1 - [ECHB_MOUSE]	42.32	2	19	20	346	-0.04	0.01	-0.09	-0.03
Q9JJL8	Serine--tRNA ligase, mitochondrial OS=Mus musculus GN=Sars2 PE=2 SV=2 - [SYSM_MOUSE]	22.39	1	7	7	36	-0.12	-0.26	-0.06	-0.04
Q62348	Translin OS=Mus musculus GN=Tsn PE=1 SV=1 - [TSN_MOUSE]	39.91	1	10	10	52	0.49	0.62	0.41	0.54
A6H8H2	DENN domain-containing protein 4C OS=Mus musculus GN=Dennd4c PE=1 SV=1 - [DEN4C_MOUSE]	9.29	4	14	14	34	0.21	0.31	0.08	0.22
P51450	Nuclear receptor ROR-gamma OS=Mus musculus GN=Rorc PE=1 SV=1 - [RORG_MOUSE]	5.04	8	2	3	4	-0.57	-1.31	-0.27	-1.01
Q8R4D5	Transient receptor potential cation channel subfamily M member 8 OS=Mus musculus GN=Trpm8 PE=1 SV=3 - [TRPM8_MOUSE]	2.63	1	2	3	3	-0.67	0.73	-0.79	0.61
P97352	Protein S100-A13 OS=Mus musculus GN=S100a13 PE=1 SV=1 - [S10AD_MOUSE]	55.10	1	7	7	73	-0.20	-0.12	-0.24	-0.17
Q64511	DNA topoisomerase 2-beta OS=Mus musculus GN=Top2b PE=1 SV=2 - [TOP2B_MOUSE]	21.46	4	22	31	109	0.22	0.37	0.14	0.22
Q4VAB4-2	Isoform 2 of Protein canopy homolog 1 OS=Mus musculus GN=Cnpy1 - [CNPY1_MOUSE]	6.36	1	1	1	1	0.11	-0.24	0.06	-0.30
D3YWP3	MCG1034428 OS=Mus musculus GN=Rpl23a-ps3 PE=3 SV=1 - [D3YWP3_MOUSE]	51.92	7	12	12	80	0.10	0.24	0.13	0.23
P43025	Tetranectin OS=Mus musculus GN=Clec3b PE=1 SV=2 - [TETN_MOUSE]	37.62	2	5	5	18	-0.34	-0.45	-0.26	-0.40
Q8CIM3	D-2-hydroxyglutarate dehydrogenase, mitochondrial OS=Mus musculus GN=D2hgdh PE=2 SV=3 - [D2HDH_MOUSE]	1.68	2	1	1	1	0.95	0.61	0.81	0.47
E9QMH7	Inhibitor of nuclear factor kappa-B kinase-interacting protein OS=Mus musculus GN=Ikbip PE=2 SV=1 - [E9QMH7_MOUSE]	9.65	2	2	4	6	-0.67	-0.65	-0.84	-0.82

Q03963	Interferon-induced, double-stranded RNA-activated protein kinase OS=Mus musculus GN=Eif2ak2 PE=1 SV=2 - [E2AK2_MOUSE]	11.07	1	5	5	13	0.25	0.29	0.46	0.54
E9Q8Y7	Sarcospan OS=Mus musculus GN=Sspn PE=4 SV=1 - [E9Q8Y7_MOUSE]	6.43	3	1	1	1	0.12	0.11	-0.07	-0.09
E9Q3B5	Uncharacterized protein OS=Mus musculus GN=Gm10774 PE=3 SV=1 - [E9Q3B5_MOUSE]	29.85	2	2	2	5	0.02	-0.39	-0.05	-0.46
Q80ZX0	Protein Sec24b OS=Mus musculus GN=Sec24b PE=2 SV=1 - [Q80ZX0_MOUSE]	9.43	3	8	9	32	-0.01	-0.16	0.17	0.00
P62631	Elongation factor 1-alpha 2 OS=Mus musculus GN=Eef1a2 PE=1 SV=1 - [EF1A2_MOUSE]	43.20	1	6	16	265	0.11	0.12	-0.05	0.03
B1AXN9	Ribosomal protein S6 kinase alpha-3 OS=Mus musculus GN=Rps6ka3 PE=2 SV=1 - [B1AXN9_MOUSE]	37.36	27	14	24	107	0.23	0.06	0.20	0.12
P63154	Crooked neck-like protein 1 OS=Mus musculus GN=Crnkl1 PE=2 SV=1 - [CRNL1_MOUSE]	9.71	2	6	6	10	0.04	0.07	0.21	0.05
Q9CQL5	39S ribosomal protein L18, mitochondrial OS=Mus musculus GN=Mrpl18 PE=2 SV=1 - [RM18_MOUSE]	12.22	1	2	2	9	-0.17	-0.57	0.22	-0.16
F7B6N2	SID1 transmembrane family member 2 (Fragment) OS=Mus musculus GN=Sidt2 PE=4 SV=1 - [F7B6N2_MOUSE]	2.71	1	1	1	4	-0.35	-0.66	-0.14	-0.45
P70695	Fructose-1,6-bisphosphatase isozyme 2 OS=Mus musculus GN=Fbp2 PE=2 SV=2 - [F16P2_MOUSE]	31.86	1	9	10	137	-0.12	-0.05	-0.14	-0.03
Q8BH58	TIP41-like protein OS=Mus musculus GN=Tiprl PE=2 SV=1 - [TIPRL_MOUSE]	23.25	1	4	4	33	0.25	0.18	0.20	0.19
Q9D6K8	FUN14 domain-containing protein 2 OS=Mus musculus GN=Fundc2 PE=2 SV=1 - [FUND2_MOUSE]	15.89	1	2	2	2	0.00	0.26	0.02	0.28
Q91XE8	Transmembrane protein 205 OS=Mus musculus GN=Tmem205 PE=1 SV=1 - [TM205_MOUSE]	12.70	1	2	2	23	0.03	0.01	-0.10	0.00
Q91YM2	Rho GTPase-activating protein 35 OS=Mus musculus GN=Arhgap35 PE=1 SV=3 - [RHG35_MOUSE]	4.40	1	5	5	8	0.04	-0.03	0.17	0.05
Q64324	Syntaxin-binding protein 2 OS=Mus musculus GN=Stxbp2 PE=2 SV=1 - [STXB2_MOUSE]	13.83	2	7	7	21	0.24	0.06	0.32	0.28

Q8K2X3	CST complex subunit STN1 OS=Mus musculus GN=Obfc1 PE=1 SV=2 - [STN1_MOUSE]	7.14	1	2	2	3	-0.45	-0.71	-0.39	-0.64
Q03265	ATP synthase subunit alpha, mitochondrial OS=Mus musculus GN=Atp5a1 PE=1 SV=1 - [ATPA_MOUSE]	54.61	2	1	30	1239	-0.14	-0.29	-0.02	-0.15
Q9JM14	5'(3')-deoxyribonucleotidase, cytosolic type OS=Mus musculus GN=Nt5c PE=1 SV=1 - [NT5C_MOUSE]	56.00	1	2	9	32	-0.04	0.33	-0.14	0.19
E9Q397	Spectrin beta chain, erythrocytic OS=Mus musculus GN=Sptb PE=2 SV=1 - [E9Q397_MOUSE]	7.21	4	8	15	31	0.32	0.00	0.19	0.13
Q9CQ91	NADH dehydrogenase [ubiquinone] 1 alpha subcomplex subunit 3 OS=Mus musculus GN=Ndufa3 PE=1 SV=1 - [NDUA3_MOUSE]	48.81	2	4	4	106	0.07	-0.06	-0.06	-0.04
Q3U0J8	TBC1 domain family member 2B OS=Mus musculus GN=Tbc1d2b PE=1 SV=2 - [TBD2B_MOUSE]	2.90	1	2	3	7	-0.06	-0.60	0.33	-0.22
P13439	Uridine 5'-monophosphate synthase OS=Mus musculus GN=Umps PE=2 SV=3 - [UMPS_MOUSE]	24.95	2	11	11	31	0.23	0.13	0.23	0.19
G5E889	Mitochondrial translation optimization 1 homolog (S. cerevisiae), isoform CRA_b OS=Mus musculus GN=Mto1 PE=3 SV=1 - [G5E889_MOUSE]	6.28	3	2	2	7	-0.75	-0.93	-0.57	-0.75
Q9R0L6	Pericentriolar material 1 protein OS=Mus musculus GN=Pcm1 PE=1 SV=2 - [PCM1_MOUSE]	4.49	2	4	4	6	0.08	0.58	0.20	0.71
Q9CR26	Vacuolar protein sorting associated protein VTA1 homolog OS=Mus musculus GN=Vta1 PE=1 SV=1 - [VTA1_MOUSE]	17.48	2	4	5	12	0.11	0.25	0.26	0.17
Q61285	ATP-binding cassette sub-family D member 2 OS=Mus musculus GN=Abcd2 PE=1 SV=1 - [ABCD2_MOUSE]	2.97	1	2	2	3	-3.09	0.46	-3.19	0.36
Q99K51	Plastin-3 OS=Mus musculus GN=Pls3 PE=1 SV=3 - [PLST_MOUSE]	32.86	2	12	19	151	0.18	0.14	0.21	0.26
H3BJN8	RWD domain-containing protein 4 (Fragment) OS=Mus musculus GN=Rwdd4a PE=2 SV=1 - [H3BJN8_MOUSE]	26.52	3	2	2	5	-0.27	0.07	-0.22	0.05

B1AS29	Glutamate receptor ionotropic, kainate 3 OS=Mus musculus GN=Grik3 PE=2 SV=1 [GRIK3_MOUSE]	1.63	1	1	1	1	0.23	0.31	0.27	0.35
P00375	Dihydrofolate reductase OS=Mus musculus GN=Dhfr PE=1 SV=3 - [DYR_MOUSE]	25.67	1	5	5	25	0.33	0.54	0.45	0.63
Q61733	28S ribosomal protein S31, mitochondrial OS=Mus musculus GN=Mrps31 PE=2 SV=1 - [RT31_MOUSE]	21.61	1	7	8	27	0.15	0.12	0.04	0.02
B1ARD6	Protein Slfn9 OS=Mus musculus GN=Slfn9 PE=4 SV=1 - [B1ARD6_MOUSE]	4.07	3	3	3	5	0.31	-0.44	0.80	0.05
Q9QWG7	Sulfotransferase family cytosolic 1B member 1 OS=Mus musculus GN=Sult1b1 PE=2 SV=2 - [ST1B1_MOUSE]	42.14	2	11	12	85	0.08	-0.04	0.09	-0.14
G5E8R3	Pyruvate carboxylase OS=Mus musculus GN=Pcx PE=3 SV=1 - [G5E8R3_MOUSE]	28.78	3	29	29	178	-0.25	-0.31	-0.15	-0.28
G5E823	D-tyrosyl-tRNA(Tyr) deacylase 1 OS=Mus musculus GN=Hars2 PE=4 SV=1 - [G5E823_MOUSE]	18.87	2	6	9	24	0.11	-0.15	0.03	-0.06
Q8CH72	E3 ubiquitin-protein ligase TRIM32 OS=Mus musculus GN=Trim32 PE=1 SV=2 - [TRI32_MOUSE]	8.40	2	5	5	7	-0.05	-0.28	-0.08	-0.17
Q9WV68	Peroxisomal 2,4-dienoy CoA reductase OS=Mu musculus GN=Decr2 PE=1 SV=1 - [DECR2_MOUSE]	8.22	1	2	2	11	-0.30	-0.45	0.11	0.02
Q91WU5	Arsenite methyltransferase OS=Mus musculus GN=As3mt PE=2 SV=2 - [AS3MT_MOUSE]	55.85	1	17	17	137	0.11	0.08	0.13	0.13
P63321	Ras-related protein Ral-A OS=Mus musculus GN=Rala PE=1 SV=1 - [RALA_MOUSE]	38.83	1	3	6	59	0.10	0.26	0.13	0.21
Q9R1T2	SUMO-activating enzyme subunit 1 OS=Mus musculus GN=Sae1 PE=2 SV=1 - [SAE1_MOUSE]	24.57	2	7	7	37	0.33	0.22	0.31	0.07
Q8BU85	Methionine-R-sulfoxide reductase B3, mitochondrial OS=Mus musculus GN=Msrb3 PE=1 SV=2 - [MSRB3_MOUSE]	27.27	2	4	4	14	-0.09	0.09	-0.40	-0.15
P49698-2	Isoform Short of Hepatocyte nuclear factor 4-alpha OS=Mus musculus GN=Hnf4a - [HNF4A_MOUSE]	7.97	2	3	3	7	-0.30	0.50	-0.45	0.02
G3UXZ5	Proteasome activator complex subunit 1 (Fragment) OS=Mus musculus GN=Psme1 PE=2 SV=1 - [G3UXZ5_MOUSE]	56.30	6	16	16	249	0.34	0.36	0.31	0.36

Q80XS7	Protein FAM83E OS=Mus musculus GN=Fam83e PE=2 SV=2 - [FA83E_MOUSE]	9.15	1	3	3	7	-0.11	0.09	0.12	0.23
Q63ZW7-5	Isoform 5 of InaD-like protein OS=Mus musculus GN=Inadl - [INADL_MOUSE]	3.18	5	1	1	3	0.94	0.04	0.85	-0.05
A2BFL2	Protein zyg-11 homolog A OS=Mus musculus GN=Zyg11a PE=2 SV=1 - [ZY11A_MOUSE]	2.71	2	2	2	2	0.29	0.56	0.02	0.29
Q6PER3	Microtubule-associated protein RP/EB family member 3 OS=Mus musculus GN=Mapre3 PE=1 SV=1 - [MARE3_MOUSE]	20.28	2	1	4	23	0.54	-0.15	0.40	-0.02
Q63870	Collagen alpha-1(VII) chain OS=Mus musculus GN=Col7a1 PE=2 SV=3 - [CO7A1_MOUSE]	3.74	1	6	6	7	0.37	-0.24	-0.02	-0.34
P28867	Protein kinase C delta type OS=Mus musculus GN=Prkcd PE=1 SV=3 [KPCD_MOUSE]	26.71	9	14	16	37	0.20	0.17	0.15	0.13
Q9Z1A9	TBC1 domain family member 8 OS=Mus musculus GN=Tbc1d8 PE=2 SV=2 - [TBCD8_MOUSE]	3.26	1	2	2	2	0.56	0.16	0.13	-0.27
F8VPP8	Protein Zc3h7b OS=Mus musculus GN=Zc3h7b PE=2 SV=1 - [F8VPP8_MOUSE]	3.26	1	3	3	8	0.22	0.02	0.07	0.02
Q91WR3-2	Isoform 2 of Activating signal cointegrator 1 complex subunit 2 OS=Mus musculus GN=Ascc2 - [ASCC2_MOUSE]	2.82	3	2	2	6	0.20	-0.16	0.44	0.08
O88531	Palmitoyl-protein thioesterase 1 OS=Mus musculus GN=Ppt1 PE=2 SV=2 - [PPT1_MOUSE]	25.82	3	7	7	38	-0.35	-0.29	-0.17	-0.09
Q9CRA7	ATP synthase subunit s, mitochondrial OS=Mus musculus GN=Atp5s PE=2 SV=1 - [ATP5S_MOUSE]	13.00	1	2	2	7	0.07	0.24	-0.02	-0.10
Q8C788	Sorting nexin 18 OS=Mus musculus GN=Snx18 PE=2 SV=1 - [Q8C788_MOUSE]	10.24	3	6	6	12	-0.31	-0.41	-0.07	-0.20
E9QN31	Putative ribosomal RNA methyltransferase NOP2 OS=Mus musculus GN=Nop2 PE=4 SV=1 - [E9QN31_MOUSE]	13.85	2	9	9	27	0.10	0.06	0.09	-0.01
P41241	Tyrosine-protein kinase CSK OS=Mus musculus GN=Csk PE=1 SV=2 - [CSK_MOUSE]	29.11	1	11	12	28	0.29	0.28	0.25	0.24
Q3TCV3	Gamma-secretase-activating protein OS=Mus musculus GN=Gsap PE=2 SV=1 - [GSAP_MOUSE]	2.33	2	1	2	2	-0.18	-0.51	-0.15	-0.48

O08686	Homeobox protein BarH-like 2 OS=Mus musculus GN=Barx2 PE=2 SV=2 - [BARX2_MOUSE]	5.30	1	1	1	1	-0.05	-0.05	-0.13	-0.14
Q78T54-2	Isoform 2 of Vacuolar ATPase assembly integral membrane protein VMA21 OS=Mus musculus GN=Vma21 - [VMA21_MOUSE]	26.51	3	2	2	12	0.12	-0.20	-0.39	-0.32
Q8BUV3	Gephyrin OS=Mus musculus GN=Gphn PE=1 SV=2 - [GEPH_MOUSE]	11.57	2	7	7	24	0.16	0.36	0.11	0.27
H9KUX9	Retinoid isomerohydrolase OS=Mus musculus GN=Rpe65 PE=2 SV=1 - [H9KUX9_MOUSE]	1.69	2	1	1	1	-0.08	-0.06	-0.06	-0.03
D3YVL0	Putative helicase MOV-10 OS=Mus musculus GN=Mov10 PE=4 SV=1 - [D3YVL0_MOUSE]	16.53	4	13	13	37	0.18	0.28	0.13	0.38
Q99LD4	COP9 signalosome complex subunit 1 OS=Mus musculus GN=Gps1 PE=1 SV=1 - [CSN1_MOUSE]	23.35	5	10	11	21	0.34	0.01	0.37	0.01
Q3U3V8	X-ray radiation resistance-associated protein 1 OS=Mus musculus GN=Xrra1 PE=2 SV=1 - [XRRA1_MOUSE]	1.02	1	1	1	1	-0.01	-0.01	0.01	0.00
Q9CR61	NADH dehydrogenase [ubiquinone] 1 beta subcomplex subunit 7 OS=Mus musculus GN=Ndufb7 PE=1 SV=3 - [NDUB7_MOUSE]	58.39	1	6	6	56	0.25	-0.03	0.09	-0.07
P08752	Guanine nucleotide-binding protein G(i) subunit alpha-2 OS=Mus musculus GN=Gnai2 PE=1 SV=5 [GNAI2_MOUSE]	43.38	6	7	14	200	-0.16	-0.20	-0.10	-0.17
P13864	DNA (cytosine-5)-methyltransferase 1 OS=Mus musculus GN=Dnmt1 PE=1 SV=5 - [DNMT1_MOUSE]	5.25	3	6	6	16	0.20	0.27	0.16	0.13
A2AFX4	43 kDa receptor-associated protein of the synapse OS=Mus musculus GN=Rapsn PE=2 SV=1 - [A2AFX4_MOUSE]	3.40	2	1	1	1	-0.16	0.08	0.15	0.38
Q8VEE4	Replication protein A 70 kDa DNA-binding subunit OS=Mus musculus GN=Rpa1 PE=2 SV=1 - [RFA1_MOUSE]	16.05	2	8	8	39	0.15	0.15	0.13	0.24
P60840-2	Isoform 2 of Alpha-endosulfine OS=Mus musculus GN=Ensa - [ENSA_MOUSE]	55.56	3	4	5	29	0.38	0.42	0.06	0.15
P52503	NADH dehydrogenase [ubiquinone] iron-sulfur protein 6, mitochondrial OS=Mus musculus GN=Ndufs6 PE=1 SV=2 - [NDUS6_MOUSE]	52.59	2	6	6	68	-0.11	-0.14	-0.09	-0.13

Q9WU79	Proline dehydrogenase 1, mitochondrial OS=Mus musculus GN=Prodh PE=2 SV=2 [PROD_MOUSE]	12.02	3	5	5	11	-0.57	-0.30	-0.37	-0.23
P01899	H-2 class I histocompatibility antigen, D-B alpha chain OS=Mus musculus GN=H2-D1 PE=1 SV=2 - [HA11_MOUSE]	37.29	12	2	11	128	0.34	0.22	0.15	0.12
P22907-2	Isoform 2 of Porphobilinogen deaminase OS=Mus musculus GN=Hmbs - [HEM3_MOUSE]	15.99	2	4	4	14	0.11	0.00	0.10	0.17
Q9JHU2	Palmdelphin OS=Mus musculus GN=Palmd PE=1 SV=1 - [PALMD_MOUSE]	9.26	2	2	2	3	0.13	-0.73	0.15	-0.71
Q8BHL5-2	Isoform 2 of Engulfment and cell motility protein 2 OS=Mus musculus GN=Elmo2 - [ELMO2_MOUSE]	4.72	4	1	4	10	-0.04	-0.18	-0.09	-0.23
Q9QYJ3	DnaJ homolog subfamily B member 1 OS=Mus musculus GN=Dnajb1 PE=2 SV=3 - [DNJB1_MOUSE]	18.24	1	6	7	23	0.30	0.17	0.15	0.08
P05132-2	Isoform 2 of cAMP-dependent protein kinase catalytic subunit alpha OS=Mus musculus GN=Prkaca - [KAPCA_MOUSE]	24.49	2	2	7	43	-0.02	0.09	0.13	0.16
Q9WTR5	Cadherin-13 OS=Mus musculus GN=Cdh13 PE=1 SV=2 - [CAD13_MOUSE]	22.97	1	15	15	99	-0.19	-0.15	-0.20	-0.13
Q9R0Y5	Adenylate kinase isoenzyme 1 OS=Mus musculus GN=Ak1 PE=1 SV=1 - [KAD1_MOUSE]	56.70	2	9	9	83	0.09	0.17	0.16	0.19
Q9JME7	Trafficking protein particle complex subunit 2-like protein OS=Mus musculus GN=Trappc2l PE=2 SV=1 - [TPC2L_MOUSE]	15.83	1	2	2	5	0.15	0.27	0.27	0.39
Q9DBG3	AP-2 complex subunit beta OS=Mus musculus GN=Ap2b1 PE=1 SV=1 - [AP2B1_MOUSE]	34.47	10	13	30	183	0.06	0.11	0.07	0.13
P97452	Ribosome biogenesis protein BOP1 OS=Mus musculus GN=Bop1 PE=1 SV=1 - [BOP1_MOUSE]	3.14	1	1	1	6	0.01	-0.11	-0.15	-0.28
P23927	Alpha-crystallin B chain OS=Mus musculus GN=Cryab PE=1 SV=2 [CRYAB_MOUSE]	24.57	1	4	4	17	0.45	0.39	0.46	0.47
Q01320	DNA topoisomerase 2-alpha OS=Mus musculus GN=Top2a PE=1 SV=2 - [TOP2A_MOUSE]	9.03	1	6	13	37	-0.02	0.16	0.03	0.10
P63073	Eukaryotic translation initiation factor 4E OS=Mus musculus GN=Eif4e PE=1 SV=1 - [IF4E_MOUSE]	18.43	1	3	3	22	0.25	-0.06	0.43	0.11

Q9D1H8	39S ribosomal protein L53, mitochondrial OS=Mus musculus GN=Mrpl53 PE=2 SV=1 - [RM53_MOUSE]	33.05	1	3	3	10	0.45	0.27	0.28	0.14
Q9D964	Glycine amidinotransferase, mitochondrial OS=Mus musculus GN=Gatm PE=1 SV=1 - [GATM_MOUSE]	24.59	1	7	7	15	-0.22	0.28	-0.13	0.32
K7N6C2	Protein Cyp2c68 OS=Mus musculus GN=Cyp2c68 PE=3 SV=1 - [K7N6C2_MOUSE]	10.59	8	4	5	10	-0.11	-0.48	-0.08	-0.63
Q5PPR2	Exocyst complex component 1 OS=Mus musculus GN=Exoc1 PE=2 SV=1 - [Q5PPR2_MOUSE]	8.99	3	6	6	15	0.11	-0.10	0.24	0.03
Q920Q8-4	Isoform 4 of Influenza virus NS1A-binding protein homolog OS=Mus musculus GN=Ivns1abp - [NS1BP_MOUSE]	7.50	3	3	3	13	0.57	0.31	0.31	0.28
Q68FE6	Protein FAM65A OS=Mus musculus GN=Fam65a PE=1 SV=2 - [FA65A_MOUSE]	1.23	1	1	1	5	0.03	-0.25	0.13	-0.23
Q8C167-2	Isoform 2 of Prolyl endopeptidase-like OS=Mus musculus GN=Prepl - [PPCEL_MOUSE]	6.11	4	3	3	3	-0.14	-0.52	0.34	-0.04
Q91WQ5	TAF5-like RNA polymerase II p300/CBP-associated factor-associated factor 65 kDa subunit 5L OS=Mus musculus GN=Taf5l PE=2 SV=1 - [TAF5L_MOUSE]	2.04	1	1	1	1	-0.10	0.09	0.06	0.24
Q8BI08	Protein MAL2 OS=Mus musculus GN=Mal2 PE=1 SV=1 - [MAL2_MOUSE]	6.29	1	1	1	4	-0.05	-1.12	-0.11	-1.18
Q60520	Paired amphipathic helix protein Sin3a OS=Mus musculus GN=Sin3a PE=1 SV=3 - [SIN3A_MOUSE]	7.14	3	7	7	23	0.07	0.28	0.03	0.06
Q8K2M0	39S ribosomal protein L38, mitochondrial OS=Mus musculus GN=Mrpl38 PE=2 SV=2 - [RM38_MOUSE]	20.26	2	7	7	14	0.08	0.02	0.23	0.01
Q9D915	Uncharacterized protein C8orf4 homolog OS=Mus musculus PE=2 SV=1 - [CH004_MOUSE]	15.09	1	1	1	1	0.00	-0.19	0.12	-0.08
P48678	Prelamin-A/C OS=Mus musculus GN=Lmna PE=1 SV=2 - [LMNA_MOUSE]	68.57	4	49	51	1387	-0.23	-0.22	-0.23	-0.22
E9PZF0	Nucleoside diphosphate kinase OS=Mus musculus GN=Gm20390 PE=2 SV=1 - [E9PZF0_MOUSE]	63.30	3	5	12	265	0.51	0.69	0.41	0.55

F7BJB9	Protein Morc3 OS=Mus musculus GN=Morc3 PE=4 SV=1 - [F7BJB9_MOUSE]	0.74	1	1	1	1	-0.15	-0.32	-0.17	-0.34
E9Q512	Protein Trip11 OS=Mus musculus GN=Trip11 PE=2 SV=1 - [E9Q512_MOUSE]	8.35	5	13	18	35	0.04	-0.07	0.00	0.05
Q80YA7	Dipeptidyl peptidase 8 OS=Mus musculus GN=Dpp8 PE=1 SV=1 - [DPP8_MOUSE]	5.38	1	3	4	10	-0.37	-0.27	-0.20	-0.10
Q68FF6	ARF GTPase-activating protein GIT1 OS=Mus musculus GN=Git1 PE=1 SV=1 - [GIT1_MOUSE]	15.71	3	7	8	17	0.22	-0.14	-0.08	-0.20
Q8R5H1-5	Isoform 5 of Ubiquitin carboxyl-terminal hydrolase 15 OS=Mus musculus GN=Usp15 - [UBP15_MOUSE]	9.66	3	1	11	18	-0.20	0.40	0.06	0.66
Q3TLH4	Protein PRRC2C OS=Mus musculus GN=Prrc2c PE=1 SV=3 - [PRC2C_MOUSE]	9.66	2	22	24	66	-0.08	0.05	-0.10	0.03
Q8R502	Leucine-rich repeat-containing protein 8C OS=Mus musculus GN=Lrrc8c PE=1 SV=1 - [LRC8C_MOUSE]	6.72	1	4	4	4	-0.33	-0.51	-0.17	-0.35
Q9WUZ9	Ectonucleoside triphosphate diphosphohydrolase 5 OS=Mus musculus GN=Entpd5 PE=1 SV=1 - [ENTP5_MOUSE]	27.63	2	8	8	54	-0.09	-0.06	0.01	-0.10
Q80TY0-2	Isoform 2 of Formin-binding protein 1 OS=Mus musculus GN=Fnbp1 - [FNBP1_MOUSE]	27.09	13	13	13	35	0.16	0.23	0.11	0.11
P57759	Endoplasmic reticulum resident protein 29 OS=Mus musculus GN=Erp29 PE=1 SV=2 [ERP29_MOUSE]	47.71	4	11	11	115	0.08	0.03	0.07	0.06
Q3UDK1-2	Isoform 2 of TRAF-type zinc finger domain-containing protein 1 OS=Mus musculus GN=Trafd1 - [TRAD1_MOUSE]	4.69	2	2	2	2	-0.18	0.06	0.34	0.57
P70704	Probable phospholipid-transporting ATPase IA OS=Mus musculus GN=Atp8a1 PE=1 SV=1 - [AT8A1_MOUSE]	5.66	6	6	6	16	0.04	-0.31	-0.04	-0.22
E9PXG9	Vacuolar protein sorting associated protein 53 homolog OS=Mus musculus GN=Vps53 PE=2 SV=1 - [E9PXG9_MOUSE]	8.34	5	5	5	10	0.05	0.34	-0.23	0.06
O55125	Protein NipSnap homolog 1 OS=Mus musculus GN=Nipsnap1 PE=1 SV=1 - [NIPS1_MOUSE]	37.68	2	10	11	79	0.12	-0.05	0.10	-0.03
Q8K409	DNA polymerase beta OS=Mus musculus GN=Polb PE=2 SV=3 - [DPOLB_MOUSE]	17.91	1	5	6	13	-0.12	-0.15	0.02	-0.04

Q80ZM8	Cardiolipin synthase OS=Mus musculus GN=Crls1 PE=2 SV=1 - [CRLS1_MOUSE]	1.98	1	1	1	1	0.41	0.27	0.26	0.12
P61963	DDB1- and CUL4-associated factor 7 OS=Mus musculus GN=Dcaf7 PE=2 SV=1 [DCAF7_MOUSE]	12.28	1	3	3	18	-0.19	-0.13	-0.01	-0.01
P52196	Thiosulfate sulfurtransferase OS=Mus musculus GN=Tst PE=1 SV=3 - [THTR_MOUSE]	49.16	1	14	14	168	-0.35	-0.31	-0.38	-0.38
P70333	Heterogeneous nuclear ribonucleoprotein H2 OS=Mus musculus GN=Hnrnph2 PE=1 SV=1 - [HNRH2_MOUSE]	35.41	3	6	12	147	-0.11	0.20	-0.03	0.11
A2BH40-3	Isoform 3 of AT-rich interactive domain-containing protein 1A OS=Mus musculus GN=Arid1a - [ARI1A_MOUSE]	7.50	5	11	12	19	0.11	0.20	0.17	0.08
O70421	Frizzled-1 OS=Mus musculus GN=Fzd1 PE=2 SV=3 - [FZD1_MOUSE]	3.89	1	2	2	3	0.24	0.26	0.21	0.23
P97789-3	Isoform 3 of 5'-3' exoribonuclease 1 OS=Mus musculus GN=Xrn1 - [XRN1_MOUSE]	3.32	4	5	5	6	0.24	0.32	0.15	0.44
Q8R1N4-2	Isoform 2 of NudC domain-containing protein 3 OS=Mus musculus GN=Nudcd3 - [NUDC3_MOUSE]	12.38	5	3	3	4	-0.16	0.45	-0.15	0.39
P23506	Protein-L-isoaspartate(D-aspartate) O-methyltransferase OS=Mus musculus GN=Pcmt1 PE=1 SV=3 - [PIMT_MOUSE]	51.10	9	9	9	45	0.01	0.03	-0.08	-0.01
P97760	DNA-directed RNA polymerase II subunit RPB3 OS=Mus musculus GN=Polr2c PE=2 SV=1 - [RPB3_MOUSE]	21.82	2	4	4	7	0.15	-0.12	0.20	-0.11
P53811	Phosphatidylinositol transfer protein beta isoform OS=Mus musculus GN=Pitpnb PE=1 SV=2 - [PIPNB_MOUSE]	40.22	1	11	12	24	0.25	0.09	0.15	0.04
Q8C669	E3 ubiquitin-protein ligase pellino homolog 1 OS=Mus musculus GN=Peli1 PE=1 SV=2 - [PELI1_MOUSE]	5.02	1	1	1	1	-0.23	-0.14	-0.37	-0.28
D3Z093	Lipoyltransferase 1, mitochondrial (Fragment) OS=Mus musculus GN=Lipt1 PE=2 SV=1 - [D3Z093_MOUSE]	7.39	1	1	1	1	-0.02	0.33	-0.09	0.26
Q05AA6	Dystrophin-related protein 2 OS=Mus musculus GN=Drp2 PE=1 SV=1 - [DRP2_MOUSE]	5.02	2	2	4	13	0.45	0.64	0.10	0.29

Q8VDM4	26S proteasome non-ATPase regulatory subunit 2 OS=Mus musculus GN=Psmd2 PE=1 SV=1 - [PSMD2_MOUSE]	31.17	3	23	23	132	0.37	0.35	0.32	0.37
Q8CDG3-2	Isoform 2 of Deubiquitinating protein VCIP135 OS=Mus musculus GN=Vcpip1 - [VCIP1_MOUSE]	7.95	2	6	6	14	0.47	0.30	0.22	0.31
Q64516-1	Isoform 1 of Glycerol kinase OS=Mus musculus GN=Gk - [GLPK_MOUSE]	21.95	5	11	11	29	0.17	-0.13	-0.12	-0.10
F7A8R0	N-alpha-acetyltransferase 35, NatC auxiliary subunit (Fragment) OS=Mus musculus GN=Naa35 PE=2 SV=1 - [F7A8R0_MOUSE]	15.42	6	4	4	4	0.57	0.34	0.70	0.47
Q6URW6	Myosin-14 OS=Mus musculus GN=Myh14 PE=1 SV=1 - [MYH14_MOUSE]	42.95	5	62	78	1399	-0.27	-0.25	-0.26	-0.22
Q810Q5	Normal mucosa of esophagus-specific gene 1 protein OS=Mus musculus GN=Nmes1 PE=2 SV=1 - [NMES1_MOUSE]	48.19	2	4	4	70	0.18	0.22	0.19	0.21
Q61164	Transcriptional repressor CTCF OS=Mus musculus GN=Ctcf PE=1 SV=2 - [CTCF_MOUSE]	9.65	1	4	4	9	0.04	-0.11	0.16	-0.09
P70257-2	Isoform NFIX2 of Nuclear factor 1 X-type OS=Mus musculus GN=Nfix - [NFIX_MOUSE]	6.25	6	1	2	5	1.13	1.36	0.72	0.95
Q8VHM5	Heterogeneous nuclear ribonucleoprotein R OS=Mus musculus GN=Hnrnpr PE=2 SV=1 - [Q8VHM5_MOUSE]	32.59	7	12	17	141	0.07	0.07	0.04	0.14
Q69ZS7	HBS1-like protein OS=Mus musculus GN=Hbs1l PE=1 SV=2 [HBS1L_MOUSE]	24.19	3	7	11	28	0.28	0.00	0.58	0.09
Q9CY16	28S ribosomal protein S28, mitochondrial OS=Mus musculus GN=Mrps28 PE=2 SV=1 - [RT28_MOUSE]	18.82	1	3	3	8	-0.09	-0.13	-0.29	-0.20
Q64514-2	Isoform Short of Tripeptidyl-peptidase 2 OS=Mus musculus GN=Tpp2 - [TPP2_MOUSE]	28.26	2	28	28	95	0.31	0.17	0.46	0.27
Q61112	45 kDa calcium-binding protein OS=Mus musculus GN=Sdf4 PE=2 SV=1 - [CAB45_MOUSE]	34.90	2	10	10	39	-0.02	0.02	-0.14	0.02
E9QQ10	A-kinase anchor protein 9 OS=Mus musculus GN=Akap9 PE=2 SV=1 - [E9QQ10_MOUSE]	7.36	6	23	25	54	0.10	0.23	0.14	0.08
P80313	T-complex protein 1 subunit eta OS=Mus musculus GN=Cct7 PE=1 SV=1 - [TCPH_MOUSE]	45.77	1	23	23	201	0.22	0.19	0.22	0.16

Q80UY1	UPF0586 protein C9orf41 homolog OS=Mus musculus PE=2 SV=1 - [CI041_MOUSE]	8.50	1	3	3	6	-0.15	-0.16	-0.10	-0.19
Q9WVL3	Solute carrier family 12 member 7 OS=Mus musculus GN=Slc12a7 PE=1 SV=1 - [S12A7_MOUSE]	7.11	2	7	8	33	-0.23	-0.14	-0.18	-0.09
Q9CZU3	Superkiller viralicidic activity 2-like 2 OS=Mus musculus GN=Skiv2l2 PE=2 SV=1 - [SK2L2_MOUSE]	17.21	1	12	13	44	0.30	0.32	0.24	0.41
Q8K0C9	GDP-mannose 4,6 dehydratase OS=Mus musculus GN=Gmds PE=2 SV=1 - [GMDS_MOUSE]	71.24	3	25	25	317	-0.11	-0.06	-0.14	-0.10
Q8CC86	Nicotinate phosphoribosyltransferase OS=Mus musculus GN=Naprt1 PE=2 SV=1 - [PNCB_MOUSE]	23.79	1	10	10	35	0.02	-0.08	0.03	-0.01
Q8C5Q4	G-rich sequence factor 1 OS=Mus musculus GN=Grsf1 PE=1 SV=2 - [GRSF1_MOUSE]	19.83	4	8	8	22	-0.24	-0.04	0.01	0.09
Q9D8C4	Interferon-induced 35 kDa protein homolog OS=Mus musculus GN=Ifi35 PE=2 SV=3 - [IN35_MOUSE]	35.31	2	6	6	26	0.04	0.14	0.06	0.44
Q8BTG6	UPF0454 protein C12orf49 homolog OS=Mus musculus PE=2 SV=1 - [CL049_MOUSE]	13.66	1	1	2	3	-0.15	-0.28	-0.29	-0.43
Q9Z0J4-2	Isoform N-NOS-2 of Nitric oxide synthase, brain OS=Mus musculus GN=Nos1 - [NOS1_MOUSE]	13.67	6	15	15	37	-0.13	-0.19	-0.17	-0.14
Q9DCS2	UPF0585 protein C16orf13 homolog OS=Mus musculus PE=1 SV=1 - [CP013_MOUSE]	56.37	5	8	8	52	-0.21	-0.16	-0.29	-0.11
Q3USJ8-3	Isoform 3 of FCH and double SH3 domains protein 2 OS=Mus musculus GN=Fchsd2 - [FCSD2_MOUSE]	1.38	3	1	1	1	-0.26	-0.12	-0.18	-0.04
Q6PDG5-2	Isoform 2 of SWI/SNF complex subunit SMARCC2 OS=Mus musculus GN=Smarcc2 [SMRC2_MOUSE]	22.66	3	13	19	58	0.10	0.17	0.16	0.19
Q9D6W8	Uncharacterized protein C17orf59 homolog OS=Mus musculus PE=1 SV=1 - [CQ059_MOUSE]	12.22	1	2	2	4	-0.32	-0.36	0.05	0.01
Q8K3A0	Iron-sulfur cluster co-chaperone protein HscB, mitochondrial OS=Mus musculus GN=Hscb PE=2 SV=2 - [HSC20_MOUSE]	34.19	3	6	6	22	-0.50	-0.43	-0.20	-0.37
P50427	Steryl-sulfatase OS=Mus musculus GN=Sts PE=2 SV=1 - [STS_MOUSE]	1.60	1	1	1	2	-0.72	-0.43	-0.64	-0.35
Q9WVA4	Transgelin-2 OS=Mus musculus GN=Tagln2 PE=1 SV=4 - [TAGL2_MOUSE]	70.35	1	12	14	579	-0.05	-0.05	-0.04	0.02

P35278	Ras-related protein Rab-5C OS=Mus musculus GN=Rab5c PE=1 SV=2 - [RAB5C_MOUSE]	54.63	3	5	7	78	0.31	0.18	0.11	0.15
O08856	RNA polymerase II elongation factor ELL OS=Mus musculus GN=Ell PE=2 SV=2 - [ELL_MOUSE]	4.49	1	2	3	3	0.09	0.11	-0.07	-0.05
P24788-2	Isoform 2 of Cyclin-dependent kinase 11B OS=Mus musculus GN=Cdk11b - [CD11B_MOUSE]	14.35	3	6	6	17	0.19	-0.08	0.31	0.08
Q9D1Q6	Endoplasmic reticulum resident protein 44 OS=Mus musculus GN=Erp44 PE=1 SV=1 [ERP44_MOUSE]	53.69	1	16	17	222	0.06	0.13	0.03	0.13
Q60790	Ras GTPase-activating protein 3 OS=Mus musculus GN=Rasa3 PE=1 SV=2 - [RASA3_MOUSE]	3.36	1	3	3	6	0.13	0.00	0.15	-0.05
E9PVW4	V-set and immunoglobulin domain-containing protein 10 OS=Mus musculus GN=Vsig10 PE=2 SV=1 - [E9PVW4_MOUSE]	8.10	2	2	2	6	0.15	0.20	0.27	0.13
P63276	40S ribosomal protein S17 OS=Mus musculus GN=Rps17 PE=1 SV=2 - [RS17_MOUSE]	40.74	1	7	7	162	0.70	0.49	0.62	0.45
Q8VED9	Galectin-related protein OS=Mus musculus GN=Lgalsl PE=1 SV=1 - [LEGL_MOUSE]	27.91	2	4	4	7	-0.02	-0.12	-0.06	-0.42
Q8VCS3	Glycosaminoglycan xylosylkinase OS=Mus musculus GN=Fam20b PE=2 SV=1 - [XYLK_MOUSE]	6.85	1	2	2	3	-0.14	0.07	-0.35	-0.14
Q99N17	Cytochrome P450 CYP4F16 OS=Mus musculus GN=Cyp4f16 PE=2 SV=1 - [Q99N17_MOUSE]	3.05	8	1	2	3	0.11	-0.17	-0.01	-0.29
P18524	Ig heavy chain V region RF OS=Mus musculus PE=1 SV=1 - [HVM53_MOUSE]	5.13	1	1	1	3	0.38	0.28	0.25	0.15
Q99KV1	DnaJ homolog subfamily B member 11 OS=Mus musculus GN=Dnajb11 PE=1 SV=1 - [DJB11_MOUSE]	34.92	1	12	12	135	0.44	0.56	0.30	0.54
A2AQ17	Complex I intermediate-associated protein 30, mitochondrial OS=Mus musculus GN=Ndufaf1 PE=4 SV=1 - [A2AQ17_MOUSE]	9.15	2	3	3	9	0.00	-1.77	0.21	-1.55
Q9D0T1	NHP2-like protein 1 OS=Mus musculus GN=Nhp2l1 PE=2 SV=4 - [NH2L1_MOUSE]	17.97	2	2	2	32	0.07	-0.06	0.15	0.08
Q7TNC4-2	Isoform 2 of Putative RNA-binding protein Luc7-like 2 OS=Mus musculus GN=Luc7l2 - [LC7L2_MOUSE]	26.77	7	5	8	40	0.31	0.35	0.27	0.33

Q8K0G8	Epithelial splicing regulatory protein 2 OS=Mus musculus GN=Esrp2 PE=2 SV=1 [ESRP2_MOUSE]	6.97	2	3	5	18	-0.33	-0.67	-0.09	-0.35
O54946	DnaJ homolog subfamily B member 6 OS=Mus musculus GN=Dnajb6 PE=1 SV=4 - [DNJB6_MOUSE]	19.45	4	2	5	9	0.13	0.28	-0.04	0.11
D3YXJ0	Protein Dgkh OS=Mus musculus GN=Dgkh PE=4 SV=1 - [D3YXJ0_MOUSE]	4.76	1	2	3	11	0.27	0.28	0.04	-0.14
P51661	Corticosteroid 11-beta-dehydrogenase isozyme 2 OS=Mus musculus GN=Hsd11b2 PE=2 SV=2 - [DHI2_MOUSE]	25.13	1	7	7	61	0.43	0.41	0.46	0.39
Q6ZWX6	Eukaryotic translation initiation factor 2 subunit 1 OS=Mus musculus GN=Eif2s1 PE=1 SV=3 - [IF2A_MOUSE]	51.75	1	18	18	104	0.11	0.04	0.11	0.04
O55126	Protein NipSnap homolog 2 OS=Mus musculus GN=Gbas PE=2 SV=1 - [NIPS2_MOUSE]	37.72	2	10	11	60	0.28	0.35	0.21	0.43
O08579	Emerin OS=Mus musculus GN=Emd PE=1 SV=1 - [EMD_MOUSE]	39.38	4	9	9	49	-0.06	0.08	-0.07	-0.13
Q8C257	Il18r1 protein OS=Mus musculus GN=Il18r1 PE=2 SV=1 - [Q8C257_MOUSE]	6.45	2	1	1	1	-0.48	-0.38	-0.24	-0.15
P35585	AP-1 complex subunit mu-1 OS=Mus musculus GN=Ap1m1 PE=1 SV=3 - [AP1M1_MOUSE]	25.77	2	7	9	48	-0.09	-0.08	-0.09	-0.13
Q8C0M2	Abhydrolase domain-containing protein 4 OS=Mus musculus GN=Abhd4 PE=2 SV=1 - [Q8C0M2_MOUSE]	2.83	3	1	1	3	-0.25	-0.60	-0.09	-0.44
Q3V1M1	Immunoglobulin superfamily member 10 OS=Mus musculus GN=Igsf10 PE=2 SV=2 - [IGS10_MOUSE]	0.27	1	1	1	2	-0.34	-0.42	-0.37	-0.45
Q9Z1J3-2	Isoform Cytoplasmic of Cysteine desulfurase, mitochondrial OS=Mus musculus GN=Nfs1 - [NFS1_MOUSE]	14.86	3	6	6	16	-0.01	0.01	0.28	0.24
O08553	Dihydropyrimidinase-related protein 2 OS=Mus musculus GN=Dpysl2 PE=1 SV=2 - [DPYL2_MOUSE]	67.31	1	22	28	548	-0.02	-0.08	-0.09	-0.14
Q64337-2	Isoform 2 of Sequestosome-1 OS=Mus musculus GN=Sqstm1 - [SQSTM_MOUSE]	20.30	4	4	4	8	0.08	0.33	0.22	0.49
Q9R0Q6	Actin-related protein 2/3 complex subunit 1A OS=Mus musculus GN=Arpc1a PE=1 SV=1 - [ARC1A_MOUSE]	15.14	2	6	6	17	0.21	0.09	0.07	0.06

Q9ERU9	E3 SUMO-protein ligase RanBP2 OS=Mus musculus GN=Ranbp2 PE=1 SV=2 - [RBP2_MOUSE]	20.73	1	46	47	135	0.21	0.19	0.18	0.23
Q61578	NADPH:adrenodoxin oxidoreductase, mitochondrial OS=Mus musculus GN=Fdxr PE=2 SV=1 - [ADRO_MOUSE]	21.46	1	8	8	26	-0.34	-0.29	-0.12	-0.25
Q6RI63-2	Isoform 2 of Constitutive coactivator of peroxisome proliferator-activated receptor gamma OS=Mus musculus GN=Fam120b - [F120B_MOUSE]	2.23	2	1	1	1	0.04	-0.36	0.49	0.09
O08528	Hexokinase-2 OS=Mus musculus GN=Hk2 PE=1 SV=1 - [HXK2_MOUSE]	34.46	3	21	27	247	0.57	0.64	0.52	0.59
Q9D7Y9-2	Isoform 2 of Protein SLX4IP OS=Mus musculus GN=Slx4ip - [SLX4I_MOUSE]	3.50	2	1	1	6	0.17	0.27	0.30	0.39
O88630	Golgi SNAP receptor complex member 1 OS=Mus musculus GN=Gosr1 PE=1 SV=2 [GOSR1_MOUSE]	29.60	2	8	9	25	-0.14	0.07	-0.09	0.04
Q9CWI3	BRCA2 and CDKN1A-interacting protein OS=Mus musculus GN=Bccip PE=2 SV=1 [BCCIP_MOUSE]	22.47	1	6	6	27	0.03	0.04	0.13	0.16
P08003	Protein disulfide-isomerase A4 OS=Mus musculus GN=Pdia4 PE=1 SV=3 - [PDIA4_MOUSE]	53.76	1	38	38	692	0.18	0.22	0.10	0.16
B1AUN2	MCG14442 OS=Mus musculus GN=Eif2b3 PE=4 SV=1 - [B1AUN2_MOUSE]	10.40	3	4	4	6	0.10	0.20	0.05	0.14
E9PYM7	Alpha-mannosidase 2C1 (Fragment) OS=Mus musculus GN=Man2c1 PE=2 SV=1 - [E9PYM7_MOUSE]	15.24	9	9	9	40	0.00	-0.06	0.05	0.06
A2ABY3	Ethanolamine- phosphate cytidylyltransferase OS=Mus musculus GN=Pcyt2 PE=4 SV=1 - [A2ABY3_MOUSE]	13.47	2	7	7	15	0.05	0.03	0.00	-0.06
O88441	Metaxin-2 OS=Mus musculus GN=Mtx2 PE=1 SV=1 - [MTX2_MOUSE]	20.15	1	6	6	21	0.13	0.11	-0.07	-0.02
P14152	Malate dehydrogenase, cytoplasmic OS=Mus musculus GN=Mdh1 PE=1 SV=3 - [MDHC_MOUSE]	49.40	2	20	21	654	-0.20	-0.15	-0.24	-0.19
P53810	Phosphatidylinositol transfer protein alpha isoform OS=Mus musculus GN=Pitpna PE=1 SV=2 - [PIPNA_MOUSE]	54.98	4	13	14	73	0.34	0.38	0.31	0.29

Q8R3E3-2	Isoform 2 of WD repeat domain phosphoinositide-interacting protein 1 OS=Mus musculus GN=Wipi1 - [WIPI1_MOUSE]	8.01	2	2	2	3	0.48	0.44	0.32	0.28
Q923D4	Splicing factor 3B subunit 5 OS=Mus musculus GN=Sf3b5 PE=2 SV=1 - [SF3B5_MOUSE]	52.33	1	3	4	12	-0.20	0.10	0.02	0.45
Q8C6H0	Galectin-8 OS=Mus musculus GN=Lgals8 PE=2 SV=1 - [Q8C6H0_MOUSE]	11.66	3	2	2	2	0.13	0.17	0.34	0.38
Q922H2	[Pyruvate dehydrogenase [lipoamide]] kinase isozyme 3, mitochondrial OS=Mus musculus GN=Pdk3 PE=2 SV=1 - [PDK3_MOUSE]	25.30	1	8	8	45	-0.10	0.06	-0.04	0.04
A2AKX3-2	Isoform 2 of Probable helicase senataxin OS=Mus musculus GN=Setx - [SETX_MOUSE]	3.13	3	6	7	10	0.00	0.29	-0.03	0.17
D3YZR1	Protein TSSC4 (Fragment) OS=Mus musculus GN=Tssc4 PE=2 SV=1 - [D3YZR1_MOUSE]	25.35	5	4	4	14	-0.20	-0.57	-0.02	-0.35
E9Q7U2	Calcium-binding and coiled-coil domain-containing protein 1 OS=Mus musculus GN=Calcoco1 PE=2 SV=1 - [E9Q7U2_MOUSE]	16.27	2	5	6	15	-0.12	0.11	-0.29	-0.02
E9QKG3	tRNA (guanine(10)-N2) methyltransferase homolog OS=Mus musculus GN=Trmt11 PE=4 SV=1 - [E9QKG3_MOUSE]	8.48	3	3	3	6	0.08	-0.32	0.43	-0.05
E9PYF7	AP1AR OS=Mus musculus GN=Ap1ar PE=4 SV=1 - [E9PYF7_MOUSE]	2.35	1	1	1	2	0.26	0.18	0.30	0.23
Q3TAI4	Tumor protein D52-like 2, isoform CRA_c OS=Mus musculus GN=Tpd52l2 PE=2 SV=1 - [Q3TAI4_MOUSE]	50.97	6	12	12	54	0.14	0.16	0.09	0.16
P26618	Platelet-derived growth factor receptor alpha OS=Mus musculus GN=Pdgfra PE=1 SV=3 - [PGFRA_MOUSE]	7.90	12	5	6	26	-0.37	-0.30	-0.37	-0.31
Q6ZWZ7	60S ribosomal protein L17 OS=Mus musculus GN=Rpl17 PE=2 SV=1 [Q6ZWZ7_MOUSE]	40.76	2	4	6	117	0.48	0.74	0.51	0.62
O55091	Protein IMPACT OS=Mus musculus GN=Impact PE=1 SV=2 - [IMPCT_MOUSE]	16.35	1	3	3	5	0.15	0.11	0.12	0.08
Q9QXN3-2	Isoform 2 of Activating signal cointegrator 1 OS=Mus musculus GN=Trip4 - [TRIP4_MOUSE]	4.27	2	2	2	2	0.17	-0.09	0.27	0.01
Q8BUK6	Protein Hook homolog 3 OS=Mus musculus GN=Hook3 PE=1 SV=2 - [HOOK3_MOUSE]	17.41	2	10	11	28	0.12	-0.06	-0.03	0.07

Q9CWT6	Probable ATP-dependent RNA helicase DDX28 OS=Mus musculus GN=Ddx28 PE=2 SV=2 - [DDX28_MOUSE]	1.67	1	1	1	2	-0.03	-0.18	-0.17	-0.32
Q62179	Semaphorin-4B OS=Mus musculus GN=Sema4b PE=1 SV=2 - [SEM4B_MOUSE]	9.84	2	6	6	17	-0.06	0.01	-0.08	0.03
E0CYH4	WD repeat-containing protein 26 OS=Mus musculus GN=Wdr26 PE=2 SV=1 - [E0CYH4_MOUSE]	2.24	3	1	2	3	-0.19	0.49	-0.07	0.61
G5E8U8	28S ribosomal protein S10, mitochondrial OS=Mus musculus GN=Mrps10 PE=3 SV=1 - [G5E8U8_MOUSE]	23.50	5	4	4	8	0.11	0.17	0.01	0.07
E9Q774	Protein Akap11 OS=Mus musculus GN=Akap11 PE=2 SV=1 - [E9Q774_MOUSE]	1.85	2	2	2	3	0.53	-0.03	-0.09	0.00
H3BJ67	Protein Evi5l OS=Mus musculus GN=Evi5l PE=2 SV=1 - [H3BJ67_MOUSE]	2.25	3	1	1	1	-0.14	0.10	-0.34	-0.11
Q9CYA6	Zinc finger CCHC domain-containing protein 8 OS=Mus musculus GN=Zcchc8 PE=2 SV=3 - [ZCHC8_MOUSE]	7.62	1	4	4	15	0.01	0.12	-0.02	0.04
B1AVZ0	Uracil phosphoribosyltransferase homolog OS=Mus musculus GN=Uprt PE=2 SV=1 - [UPP_MOUSE]	19.35	1	4	4	13	0.12	0.19	-0.05	-0.04
Q9Z0H1	WD repeat-containing protein 46 OS=Mus musculus GN=Wdr46 PE=2 SV=1 - [WDR46_MOUSE]	4.50	1	1	2	4	-0.52	-2.80	-0.23	-2.51
P06330	Ig heavy chain V region AC38 205.12 OS=Mus musculus PE=1 SV=1 - [HVM51_MOUSE]	40.68	3	3	3	29	0.24	0.08	0.07	0.09
Q80W00-2	Isoform 2 of Serine/threonine-protein phosphatase 1 regulatory subunit 10 OS=Mus musculus GN=Ppp1r10 - [PP1RA_MOUSE]	0.92	2	1	1	2	0.21	0.11	0.28	0.18
Q9CQ73	Plakophilin 2 OS=Mus musculus GN=Pkp2 PE=2 SV=1 - [Q9CQ73_MOUSE]	16.98	4	11	12	34	0.16	0.02	0.11	0.09
F6QU40	Peroxisomal targeting signal 2 receptor (Fragment) OS=Mus musculus GN=Pex7 PE=4 SV=1 - [F6QU40_MOUSE]	6.22	3	1	1	2	0.27	0.21	0.38	0.32
P43274	Histone H1.4 OS=Mus musculus GN=Hist1h1e PE=1 SV=2 - [H14_MOUSE]	42.01	1	4	15	468	-0.10	0.17	-0.21	-0.03
O08796	Eukaryotic elongation factor 2 kinase OS=Mus musculus GN=Eef2k PE=1 SV=1 - [EF2K_MOUSE]	8.01	5	5	6	17	0.37	0.52	0.18	0.32

G3UZ26	Serine hydroxymethyltransferase (Fragment) OS=Mus musculus GN=Shmt1 PE=3 SV=1 - [G3UZ26_MOUSE]	36.67	4	14	15	79	-0.09	-0.04	0.07	0.09
P61087	Ubiquitin-conjugating enzyme E2 K OS=Mus musculus GN=Ube2k PE=1 SV=3 - [UBE2K_MOUSE]	50.50	3	8	9	64	0.14	0.12	0.23	0.36
Q5D525-3	Isoform 3 of Synaptonemal complex central element protein 1-like OS=Mus musculus GN=Syce1l - [SYC1L_MOUSE]	3.10	2	1	1	1	0.10	-0.18	-0.14	-0.41
Q05186	Reticulocalbin-1 OS=Mus musculus GN=Rcn1 PE=1 SV=1 - [RCN1_MOUSE]	21.23	1	7	7	27	0.01	0.18	-0.01	0.09
Q8BGD7	Neuronal PAS domain-containing protein 4 OS=Mus musculus GN=Npas4 PE=1 SV=1 - [NPAS4_MOUSE]	1.62	1	1	1	1	0.09	0.09	0.18	0.19
P18111	Homeobox protein CDX-1 OS=Mus musculus GN=Cdx1 PE=2 SV=2 - [CDX1_MOUSE]	4.85	1	1	1	1	-0.26	-1.19	-0.11	-1.04
Q8R2U0-2	Isoform 2 of Nucleoporin SEH1 OS=Mus musculus GN=Seh1l - [SEH1_MOUSE]	19.89	2	5	5	22	-0.07	-0.13	-0.04	-0.05
P62827	GTP-binding nuclear protein Ran OS=Mus musculus GN=Ran PE=1 SV=3 - [RAN_MOUSE]	24.07	3	7	7	85	-0.04	0.19	0.15	0.22
Q9WTU0	Lysine-specific demethylase PHF2 OS=Mus musculus GN=Phf2 PE=1 SV=2 - [PHF2_MOUSE]	1.00	1	1	1	4	-0.10	-0.01	-0.06	0.03
Q1HFZ0	tRNA (cytosine(34)-C(5))-methyltransferase OS=Mus musculus GN=Nsun2 PE=1 SV=2 - [NSUN2_MOUSE]	21.53	4	14	14	57	0.48	0.61	0.53	0.42
P07146	Anionic trypsin-2 OS=Mus musculus GN=Prss2 PE=2 SV=1 - [TRY2_MOUSE]	22.36	1	2	4	78	0.17	0.03	0.44	0.20
Q9WV60	Glycogen synthase kinase-3 beta OS=Mus musculus GN=Gsk3b PE=1 SV=2 - [GSK3B_MOUSE]	35.24	2	6	11	48	0.37	0.26	0.44	0.39
Q811D0-2	Isoform 2 of Disks large homolog 1 OS=Mus musculus GN=Dlg1 - [DLG1_MOUSE]	29.12	19	16	19	86	-0.13	-0.19	-0.09	-0.05
Q9JLM8	Serine/threonine-protein kinase DCLK1 OS=Mus musculus GN=Dclk1 PE=1 SV=1 [DCLK1_MOUSE]	11.77	5	6	7	21	0.07	0.01	-0.03	0.05

Q9CX34	Suppressor of G2 allele of SKP1 homolog OS=Mus musculus GN=Sugt1 PE=2 SV=3 [SUGT1_MOUSE]	47.92	1	14	14	92	0.19	0.13	0.16	0.13
Q9CQN3	Mitochondrial import receptor subunit TOM6 homolog OS=Mus musculus GN=Tomm6 PE=3 SV=1 - [TOM6_MOUSE]	18.92	1	1	1	2	0.24	-0.53	-0.44	-0.19
Q6GQT9	Nodal modulator 1 OS=Mus musculus GN=Nomo1 PE=1 SV=1 - [NOMO1_MOUSE]	23.31	2	20	20	74	0.05	-0.04	0.06	0.00
Q8BHA3	Probable D-tyrosyl-tRNA(Tyr) deacylase 2 OS=Mus musculus GN=Dtd2 PE=2 SV=1 - [DTD2_MOUSE]	17.26	2	2	2	10	-0.47	-0.84	-0.49	-0.80
P62823	Ras-related protein Rab-3C OS=Mus musculus GN=Rab3c PE=1 SV=1 - [RAB3C_MOUSE]	29.96	11	2	6	49	-0.55	-0.42	-0.42	-0.29
Q6NZM9	Histone deacetylase 4 OS=Mus musculus GN=Hdac4 PE=1 SV=1 - [HDAC4_MOUSE]	1.02	1	1	1	2	-0.73	0.03	-0.42	0.35
G3UW81	MCG125472 OS=Mus musculus GN=Cyp4f40 PE=3 SV=1 - [G3UW81_MOUSE]	6.30	7	2	3	5	-0.29	-0.20	-0.32	0.09
Q7TSV4	Phosphoglucomutase-2 OS=Mus musculus GN=Pgm2 PE=1 SV=1 [PGM2_MOUSE]	37.26	1	24	25	216	-0.21	-0.18	-0.22	-0.19
P19324	Serpin H1 OS=Mus musculus GN=Serpinh1 PE=1 SV=3 - [SERPH_MOUSE]	40.29	1	13	13	183	0.06	0.07	0.09	0.16
Q8C129	Leucyl-cystinyl aminopeptidase OS=Mus musculus GN=Lnpep PE=1 SV=1 - [LCAP_MOUSE]	13.46	1	12	12	29	-0.07	-0.26	-0.15	-0.28
Q78PY7	Staphylococcal nuclease domain-containing protein 1 OS=Mus musculus GN=Snd1 PE=1 SV=1 - [SND1_MOUSE]	47.58	3	35	35	298	0.37	0.36	0.34	0.35
Q9CZS1	Aldehyde dehydrogenase X, mitochondrial OS=Mus musculus GN=Aldh1b1 PE=2 SV=1 - [AL1B1_MOUSE]	50.29	2	23	27	658	-0.41	-0.31	-0.40	-0.35
Q9EQI8	39S ribosomal protein L46, mitochondrial OS=Mus musculus GN=Mrpl46 PE=2 SV=1 - [RM46_MOUSE]	27.92	1	6	6	29	0.08	0.18	0.16	0.18
O08688	Calpain-5 OS=Mus musculus GN=Capn5 PE=2 SV=1 - [CAN5_MOUSE]	18.28	3	9	9	18	0.18	0.46	0.24	0.63
P63030	Mitochondrial pyruvate carrier 1 OS=Mus musculus GN=Mpc1 PE=1 SV=1 - [MPC1_MOUSE]	22.94	5	3	4	10	-1.08	-1.29	-1.06	-1.27

Q8R3P6	von Willebrand factor A domain-containing protein 9 OS=Mus musculus GN=Vwa9 PE=2 SV=1 - [VWA9_MOUSE]	9.13	1	2	2	4	0.31	-0.22	0.08	-0.45
Q8BH00	Aldehyde dehydrogenase family 8 member A1 OS=Mus musculus GN=Aldh8a1 PE=1 SV=1 - [AL8A1_MOUSE]	1.64	1	1	1	3	-0.38	-0.66	-0.38	-0.66
P46061	Ran GTPase-activating protein 1 OS=Mus musculus GN=Rangap1 PE=1 SV=2 - [RAGP1_MOUSE]	21.22	1	11	11	50	0.41	0.51	0.34	0.45
Q9ERI6	Retinol dehydrogenase 14 OS=Mus musculus GN=Rdh14 PE=1 SV=1 - [RDH14_MOUSE]	13.47	1	4	4	10	-0.13	-0.04	-0.04	-0.04
Q9QZE7	Translin-associated protein X OS=Mus musculus GN=Tsnax PE=1 SV=1 - [TSNAX_MOUSE]	29.66	1	6	6	18	0.23	-0.04	0.45	0.09
Q7TRF5	Olfactory receptor Olfr859 OS=Mus musculus GN=Olfr859 PE=3 SV=1 - [Q7TRF5_MOUSE]	10.68	1	1	1	1	0.06	-0.35	-0.04	-0.45
Q9QYI4	DnaJ homolog subfamily B member 12 OS=Mus musculus GN=Dnajb12 PE=2 SV=2 - [DJB12_MOUSE]	21.54	3	5	5	11	0.12	0.41	0.12	0.35
Q99KP3	Lambda-crystallin homolog OS=Mus musculus GN=Cryl1 PE=2 SV=3 - [CRYL1_MOUSE]	65.52	1	15	15	207	-0.46	-0.53	-0.33	-0.42
P35283	Ras-related protein Rab-12 OS=Mus musculus GN=Rab12 PE=1 SV=3 - [RAB12_MOUSE]	11.93	12	2	3	36	0.28	0.00	0.20	-0.12
Q8R191	Synaptogyrin-3 OS=Mus musculus GN=Syngr3 PE=1 SV=1 - [SNG3_MOUSE]	11.79	1	2	2	6	-0.54	-0.45	-0.56	-0.46
Q6P5B5	Fragile X mental retardation syndrome-related protein 2 OS=Mus musculus GN=Fxr2 PE=2 SV=1 - [Q6P5B5_MOUSE]	13.95	2	5	7	31	0.17	0.16	0.17	0.10
F6VY64	U3 small nucleolar ribonucleoprotein protein IMP4 (Fragment) OS=Mus musculus GN=Imp4 PE=2 SV=1 - [F6VY64_MOUSE]	4.09	2	1	1	1	0.28	-0.23	0.15	-0.35
Q91X43	SH3 domain-containing protein 19 OS=Mus musculus GN=Sh3d19 PE=1 SV=2 - [SH319_MOUSE]	9.63	2	5	5	15	-0.25	0.01	0.08	0.34
P15535-2	Isoform Short of Beta-1,4-galactosyltransferase 1 OS=Mus musculus GN=B4galt1 - [B4GT1_MOUSE]	10.88	3	5	5	10	0.34	0.42	0.42	0.49

Q501J2	Protein FAM173A OS=Mus musculus GN=Fam173a PE=2 SV=1 - [F173A_MOUSE]	9.61	1	2	2	4	0.34	0.30	0.18	0.14
D3Z440	COP9 signalosome complex subunit 7a (Fragment) OS=Mus musculus GN=Cops7a PE=2 SV=1 - [D3Z440_MOUSE]	21.15	5	5	5	18	0.16	0.15	0.06	0.11
Q68EF6	Brain-enriched guanylate kinase-associated protein OS=Mus musculus GN=Begain PE=1 SV=2 - [BEGIN_MOUSE]	1.33	2	1	1	1	0.03	-0.02	-0.08	-0.14
A2AGS6	Tumor protein p53-inducible protein 11 (Fragment) OS=Mus musculus GN=Trp53i11 PE=2 SV=1 - [A2AGS6_MOUSE]	29.89	2	2	2	6	-0.64	-0.84	-0.48	-0.63
Q8K201	Keratinocyte-associated transmembrane protein 2 OS=Mus musculus GN=Kct2 PE=2 SV=2 - [KCT2_MOUSE]	3.86	1	1	1	2	-0.07	-0.08	0.13	0.12
D3Z2J3	Putative ATP-dependent RNA helicase DHX30 OS=Mus musculus GN=Dhx30 PE=2 SV=1 - [D3Z2J3_MOUSE]	4.81	4	4	4	8	-0.61	-0.34	-0.40	-0.51
D3Z7J7	Deoxyhypusine hydroxylase (Fragment) OS=Mus musculus GN=Dohh PE=2 SV=1 - [D3Z7J7_MOUSE]	24.47	5	2	2	3	-0.27	0.83	0.48	1.57
Q921M7	Protein FAM49B OS=Mus musculus GN=Fam49b PE=2 SV=1 - [FA49B_MOUSE]	28.70	1	6	8	52	0.44	0.91	0.32	0.61
Q9DBK0	Acyl-coenzyme A thioesterase 12 OS=Mus musculus GN=Acot12 PE=2 SV=1 - [ACO12_MOUSE]	2.88	1	1	1	1	-0.51	-0.71	-0.06	-0.25
G3X9J0	MCG122846 OS=Mus musculus GN=Sipa1l3 PE=4 SV=1 - [G3X9J0_MOUSE]	2.53	1	3	3	5	-0.19	-0.35	0.09	-0.14
F2Z3U3	Protein Raph1 (Fragment) OS=Mus musculus GN=Raph1 PE=2 SV=1 - [F2Z3U3_MOUSE]	1.01	4	1	1	1	-0.57	-0.10	-0.09	0.39
E9Q368	Protein Gm17296 OS=Mus musculus GN=Gm17296 PE=4 SV=1 - [E9Q368_MOUSE]	1.90	1	2	2	5	-0.08	0.69	-0.78	-0.01
O55022	Membrane-associated progesterone receptor component 1 OS=Mus musculus GN=Pgrmc1 PE=1 SV=4 - [PGRC1_MOUSE]	40.00	1	5	7	70	-0.25	0.04	-0.25	-0.20
E9PX65	Tyrosine--tRNA ligase, mitochondrial OS=Mus musculus GN=Yars2 PE=3 SV=1 - [E9PX65_MOUSE]	14.33	2	4	4	7	-0.01	-0.84	0.02	-0.82

Q6WKZ8	E3 ubiquitin-protein ligase UBR2 OS=Mus musculus GN=Ubr2 PE=1 SV=2 - [UBR2_MOUSE]	3.82	3	6	6	14	-0.07	-2.64	-0.18	-2.79
Q9JKX6	ADP-sugar pyrophosphatase OS=Mus musculus GN=Nudt5 PE=1 SV=1 [NUDT5_MOUSE]	14.22	2	3	3	18	-0.04	0.00	0.03	0.08
Q8C4G3	Probable phospholipid-transporting ATPase IIA OS=Mus musculus GN=Atp9a PE=2 SV=1 [Q8C4G3_MOUSE]	6.90	4	3	3	6	-0.30	-0.45	-0.27	-0.41
F6W043	Protein Vmn2r40 OS=Mus musculus GN=Vmn2r40 PE=3 SV=2 - [F6W043_MOUSE]	3.62	12	1	2	4	0.40	-0.04	0.02	-0.43
P54751	CMP-N-acetylneuraminate-beta-galactosamide-alpha-2,3 sialyltransferase 1 OS=Mus musculus GN=St3gal1 PE=2 SV=1 - [SIA4A_MOUSE]	3.86	1	1	1	1	0.03	-0.22	0.17	-0.07
Q5DU05-2	Isoform 2 of Centrosomal protein of 164 kDa OS=Mus musculus GN=Cep164 - [CE164_MOUSE]	4.74	7	4	5	7	-0.12	-0.40	0.10	-0.11
Q9Z1W8	Potassium-transporting ATPase alpha chain 2 OS=Mus musculus GN=Atp12a PE=1 SV=3 - [AT12A_MOUSE]	29.47	1	19	25	197	-0.05	0.05	-0.21	-0.15
Q8BKG3	Inactive tyrosine-protein kinase 7 OS=Mus musculus GN=Ptk7 PE=1 SV=1 - [PTK7_MOUSE]	15.44	1	13	13	40	-0.14	-0.33	0.02	-0.23
P62331	ADP-ribosylation factor 6 OS=Mus musculus GN=Arf6 PE=1 SV=2 - [ARF6_MOUSE]	36.57	2	4	5	14	-0.33	-0.05	0.02	0.21
Q9R226	KH domain-containing, RNA-binding, signal transduction-associated protein 3 OS=Mus musculus GN=Khdrbs3 PE=1 SV=1 - [KHDR3_MOUSE]	7.51	1	1	3	14	0.06	0.02	-0.11	-0.14
Q9D0F6	Replication factor C subunit 5 OS=Mus musculus GN=Rfc5 PE=2 SV=1 - [RFC5_MOUSE]	10.91	1	2	3	6	0.24	-0.40	0.03	-0.60
P08775	DNA-directed RNA polymerase II subunit RPB1 OS=Mus musculus GN=Polr2a PE=1 SV=3 - [RPB1_MOUSE]	15.89	1	20	21	43	0.23	0.25	0.18	0.35
Q925I1	ATPase family AAA domain-containing protein 3 OS=Mus musculus GN=Atad3 PE=1 SV=1 - [ATAD3_MOUSE]	36.21	5	16	16	76	-0.03	-0.11	-0.13	-0.05

D3Z7C8	Branched-chain-amino-acid aminotransferase OS=Mus musculus GN=Bcat2 PE=2 SV=1 [D3Z7C8_MOUSE]	34.02	4	11	11	76	-0.31	-0.09	-0.25	-0.12
Q9Z1B3-2	Isoform B of 1-phosphatidylinositol 4,5 bisphosphate phosphodiesterase beta-1 OS=Mus musculus GN=Plcb1 - [PLCB1_MOUSE]	6.22	4	5	5	10	-0.06	-0.05	0.06	0.02
P97816	Protein S100-G OS=Mus musculus GN=S100g PE=2 SV=3 - [S100G_MOUSE]	69.62	1	5	5	94	-2.67	-2.41	-2.88	-2.49
Q9DCV4	Regulator of microtubule dynamics protein 1 OS=Mus musculus GN=Rmdn1 PE=2 SV=2 - [RMD1_MOUSE]	25.90	3	6	7	16	0.03	-0.19	0.09	-0.28
Q3U5Q7	UMP-CMP kinase 2, mitochondrial OS=Mus musculus GN=Cmpk2 PE=2 SV=2 - [CMPK2_MOUSE]	15.44	1	6	6	20	-0.30	-0.32	-0.16	-0.14
Q8BGJ5	MCG13402, isoform CRA_a OS=Mus musculus GN=Ptbp1 PE=2 SV=1 - [Q8BGJ5_MOUSE]	29.49	9	7	10	228	-0.07	0.18	0.13	0.21
Q8BKZ9	Pyruvate dehydrogenase protein X component, mitochondrial OS=Mus musculus GN=Pdhx PE=2 SV=1 - [ODPX_MOUSE]	24.15	2	9	9	69	-0.06	0.00	0.00	0.05
Q99JZ4	GTP-binding protein SAR1a OS=Mus musculus GN=Sar1a PE=2 SV=1 - [Q99JZ4_MOUSE]	15.66	2	2	3	37	0.22	-0.04	0.02	-0.04
E9Q956	MCM domain-containing protein 2 OS=Mus musculus GN=Mcmdc2 PE=2 SV=1 - [MCMD2_MOUSE]	5.43	4	2	2	2	-0.11	-0.32	0.02	-0.20
A2ASS6	Titin OS=Mus musculus GN=Ttn PE=1 SV=1 - [TITIN_MOUSE]	0.74	12	18	25	53	0.04	0.16	0.14	0.15
P07934	Phosphorylase b kinase gamma catalytic chain, skeletal muscle/heart isoform OS=Mus musculus GN=Phkg1 PE=2 SV=3 - [PHKG1_MOUSE]	3.09	1	1	2	4	-0.21	-0.08	-0.69	-0.39
Q9ER00	Syntaxin-12 OS=Mus musculus GN=Stx12 PE=1 SV=1 - [STX12_MOUSE]	36.50	1	7	7	35	0.03	0.01	0.09	0.10
Q8BIW9	Chromosome transmission fidelity protein 18 homolog OS=Mus musculus GN=Chtf18 PE=2 SV=2 - [CTF18_MOUSE]	1.86	1	1	1	1	0.48	0.47	-0.08	-0.10

Q9Z0F8	Disintegrin and metalloproteinase domain-containing protein 17 OS=Mus musculus GN=Adam17 PE=1 SV=3 - [ADA17_MOUSE]	15.11	3	7	7	15	-0.05	0.06	0.17	-0.04
Q9QYI3	DnaJ homolog subfamily C member 7 OS=Mus musculus GN=Dnajc7 PE=1 SV=2 - [DNJC7_MOUSE]	27.73	3	13	13	46	0.23	0.17	0.40	0.27
Q8C3W1	Uncharacterized protein C1orf198 homolog OS=Mus musculus PE=1 SV=1 - [CA198_MOUSE]	20.19	1	4	4	15	0.12	-0.02	0.14	-0.04
Q9CPT4	UPF0556 protein C19orf10 homolog OS=Mus musculus GN=D17Wsu104e PE=2 SV=1 - [CS010_MOUSE]	28.31	1	4	4	20	0.07	0.40	-0.01	0.21
Q9Z1A1	Protein Tfg OS=Mus musculus GN=Tfg PE=2 SV=1 - [Q9Z1A1_MOUSE]	31.49	7	11	11	67	-0.04	-0.07	-0.14	-0.08
Q7TSC1	Protein PRRC2A OS=Mus musculus GN=Prrc2a PE=1 SV=1 - [PRC2A_MOUSE]	9.36	2	11	13	43	0.00	0.14	-0.15	0.00
Q8VEJ4	Notchless protein homolog 1 OS=Mus musculus GN=Nle1 PE=2 SV=4 - [NLE1_MOUSE]	6.19	1	2	2	4	-0.22	-0.20	0.66	0.68
Q9QYB1	Chloride intracellular channel protein 4 OS=Mus musculus GN=Clic4 PE=1 SV=3 - [CLIC4_MOUSE]	59.29	1	11	12	121	0.32	0.48	0.29	0.45
Q9CSH3	Exosome complex exonuclease RRP44 OS=Mus musculus GN=Dis3 PE=2 SV=4 - [RRP44_MOUSE]	5.95	1	4	6	19	0.03	0.25	0.13	0.44
Q8CGZ0	Calcium homeostasis endoplasmic reticulum protein OS=Mus musculus GN=Cherp PE=1 SV=1 - [CHERP_MOUSE]	5.66	2	5	5	13	-0.27	-0.06	0.08	0.16
J3QN79	MCG8345 OS=Mus musculus GN=Fabp3-ps1 PE=3 SV=1 - [J3QN79_MOUSE]	18.05	2	2	2	3	-0.15	-0.06	0.12	0.20
Q8VE11	Myotubularin-related protein 6 OS=Mus musculus GN=Mtmr6 PE=1 SV=1 - [MTMR6_MOUSE]	5.83	1	3	3	7	0.17	-0.01	0.29	-0.08
Q6GV12	3-ketodihydrosphingosine reductase OS=Mus musculus GN=Kdsr PE=2 SV=1 - [KDSR_MOUSE]	7.23	1	2	2	3	0.32	0.41	0.05	0.14
Q6NZA9	Transcription initiation factor TFIID subunit 9B OS=Mus musculus GN=Taf9b PE=1 SV=2 [TAF9B_MOUSE]	3.21	3	1	1	2	0.02	0.85	-0.38	0.45
P16330	2',3'-cyclic-nucleotide 3' phosphodiesterase OS=Mus musculus GN=Cnp PE=1 SV=3 - [CN37_MOUSE]	20.24	6	9	10	17	-0.07	-0.21	-0.04	-0.13

Q8BH80	Vesicle-associated membrane protein, associated protein B and C OS=Mus musculus GN=Vapb PE=2 SV=1 - [Q8BH80_MOUSE]	40.74	2	8	9	59	0.21	0.23	0.18	0.21
Q7TNC9	Inpp5a protein OS=Mus musculus GN=Inpp5a PE=2 SV=1 - [Q7TNC9_MOUSE]	9.95	3	4	4	16	-0.29	-0.17	-0.16	-0.22
Q9D125	28S ribosomal protein S25, mitochondrial OS=Mus musculus GN=Mrps25 PE=1 SV=1 - [RT25_MOUSE]	17.54	1	3	3	3	-0.32	0.06	0.03	0.41
O70299	Protein mab-21-like 1 OS=Mus musculus GN=Mab21l1 PE=2 SV=1 - [MB211_MOUSE]	2.51	2	1	1	1	0.11	-0.16	0.05	-0.23
P21440	Multidrug resistance protein 3 OS=Mus musculus GN=Abcb4 PE=2 SV=2 - [MDR3_MOUSE]	7.45	2	1	10	30	0.07	-0.08	0.09	-0.06
Q9CU62	Structural maintenance of chromosomes protein 1A OS=Mus musculus GN=Smc1a PE=1 SV=4 - [SMC1A_MOUSE]	21.57	3	29	29	89	0.28	0.20	0.16	0.21
Q8R1T1-2	Isoform 2 of Charged multivesicular body protein 7 OS=Mus musculus GN=Chmp7 - [CHMP7_MOUSE]	5.68	2	2	2	3	0.24	0.32	0.25	0.25
P54923	[Protein ADP-ribosylarginine] hydrolase OS=Mus musculus GN=Adprh PE=2 SV=1 - [ADPRH_MOUSE]	33.70	1	10	10	95	0.10	0.06	0.20	0.15
A2A839	Protein 4.1 OS=Mus musculus GN=Epb4.1 PE=2 SV=1 - [A2A839_MOUSE]	15.18	10	6	9	17	0.01	0.00	-0.01	-0.04
Q8R574	Phosphoribosyl pyrophosphate synthase-associated protein 2 OS=Mus musculus GN=Prpsap2 PE=1 SV=1 - [KPRB_MOUSE]	31.98	1	6	9	50	-0.20	-0.31	-0.12	-0.05
Q6PGH1	Protein BUD31 homolog OS=Mus musculus GN=Bud31 PE=2 SV=1 - [BUD31_MOUSE]	19.42	2	1	1	7	-0.18	0.15	-0.10	0.21
P10649	Glutathione S-transferase Mu 1 OS=Mus musculus GN=Gstm1 PE=1 SV=2 - [GSTM1_MOUSE]	71.10	4	9	18	716	-0.10	-0.03	-0.14	-0.08
P02535-3	Isoform 3 of Keratin, type I cytoskeletal 10 OS=Mus musculus GN=Krt10 - [K1C10_MOUSE]	10.39	7	3	5	35	0.86	0.47	-0.25	-0.55
Q810A3	Tetratricopeptide repeat protein 9C OS=Mus musculus GN=Ttc9c PE=2 SV=1 - [TTC9C_MOUSE]	12.28	2	2	2	6	-0.05	-0.49	0.05	-0.39
P30412	Peptidyl-prolyl cis-trans isomerase C OS=Mus musculus GN=Ppic PE=1 SV=1 - [PPIC_MOUSE]	27.83	1	5	6	46	-0.10	-0.12	-0.07	-0.05

A2A6Q8	Myosin light chain 4 (Fragment) OS=Mus musculus GN=Myl4 PE=2 SV=1 - [A2A6Q8_MOUSE]	50.52	3	9	9	53	-0.90	-0.95	-0.91	-0.87
Q8BG60-2	Isoform 2 of Thioredoxin-interacting protein OS=Mus musculus GN=Txnip - [TXNIP_MOUSE]	8.84	2	3	3	10	-0.01	0.07	-0.06	-0.03
D3YYK8	Microtubule-associated protein RP/EB family member 2 (Fragment) OS=Mus musculus GN=Mapre2 PE=2 SV=1 - [D3YYK8_MOUSE]	41.92	5	7	9	66	0.08	0.01	0.12	0.05
Q4FK66-2	Isoform 2 of Pre-mRNA splicing factor 38A OS=Mus musculus GN=Prpf38a - [PR38A_MOUSE]	18.18	2	1	1	2	0.47	0.40	0.50	0.43
P17156	Heat shock-related 70 kDa protein 2 OS=Mus musculus GN=Hspa2 PE=1 SV=2 - [HSP72_MOUSE]	45.18	1	12	30	1029	0.07	0.02	0.06	0.06
L7N202	Uncharacterized protein OS=Mus musculus GN=Gm16477 PE=4 SV=1 - [L7N202_MOUSE]	45.86	11	8	17	185	0.15	0.42	0.07	0.33
Q91ZU6-5	Isoform 5 of Dystonin OS=Mus musculus GN=Dst - [DYST_MOUSE]	1.91	1	1	4	9	-1.15	-1.13	-0.50	-0.48
P27046	Alpha-mannosidase 2 OS=Mus musculus GN=Man2a1 PE=1 SV=2 - [MA2A1_MOUSE]	21.65	2	21	22	70	0.06	0.11	0.19	0.15
Q7M6Z4-2	Isoform 2 of Kinesin-like protein KIF27 OS=Mus musculus GN=Kif27 - [KIF27_MOUSE]	4.40	2	3	3	3	-0.32	-0.25	-0.84	-0.42
Q91YR7	Pre-mRNA-processing factor 6 OS=Mus musculus GN=Prpf6 PE=2 SV=1 - [PRP6_MOUSE]	5.63	2	7	7	13	0.22	0.21	0.27	0.25
Q99LI2	Chloride channel CLIC-like protein 1 OS=Mus musculus GN=Clcc1 PE=1 SV=1 - [CLCC1_MOUSE]	5.75	2	2	2	5	0.20	0.15	0.16	0.23
Q3TDN2	FAS-associated factor 2 OS=Mus musculus GN=Faf2 PE=2 SV=2 - [FAF2_MOUSE]	31.46	5	8	8	64	0.00	0.16	-0.01	0.14
Q80X95	Ras-related GTP-binding protein A OS=Mus musculus GN=Rraga PE=2 SV=1 [RRAGA_MOUSE]	10.54	3	3	4	15	-0.15	-0.25	-0.13	-0.22
Q9D8N0	Elongation factor 1-gamma OS=Mus musculus GN=Eef1g PE=1 SV=3 - [EF1G_MOUSE]	39.36	1	20	20	250	0.30	0.41	0.27	0.39
O08739	AMP deaminase 3 OS=Mus musculus GN=Ampd3 PE=2 SV=2 - [AMPD3_MOUSE]	6.66	4	3	5	8	-0.09	-0.30	-0.17	-0.38

Q9CR27	WASH complex subunit CCDC53 OS=Mus musculus GN=Ccdc53 PE=2 SV=1 - [CCD53_MOUSE]	16.49	1	1	2	6	0.07	0.38	0.07	-0.04
Q9JJI8	60S ribosomal protein L38 OS=Mus musculus GN=Rpl38 PE=2 SV=3 [RL38_MOUSE]	50.00	5	4	5	27	0.48	0.63	0.56	0.59
Q6KAR6	Exocyst complex component 3 OS=Mus musculus GN=Exoc3 PE=1 SV=2 - [EXOC3_MOUSE]	10.60	1	6	6	14	-0.36	0.10	-0.23	0.08
P60041	Somatostatin OS=Mus musculus GN=Sst PE=2 SV=1 - [SMS_MOUSE]	6.90	1	1	1	3	-0.51	-0.31	-0.78	-0.59
Q80Y56	Rabenosyn-5 OS=Mus musculus GN=Zfyve20 PE=2 SV=1 - [RBNS5_MOUSE]	2.68	1	2	3	3	-0.16	-0.16	-0.09	-0.10
Q148V7	LisH domain and HEAT repeat-containing protein KIAA1468 OS=Mus musculus GN=Kiaa1468 PE=1 SV=1 - [K1468_MOUSE]	12.83	5	9	9	18	0.21	0.16	0.23	0.16
F6WZH1	Dipeptidase 3 (Fragment) OS=Mus musculus GN=Mbd3 PE=4 SV=1 - [F6WZH1_MOUSE]	21.08	5	2	4	10	0.13	0.19	0.14	0.08
Q60872	Eukaryotic translation initiation factor 1A OS=Mus musculus GN=Eif1a PE=2 SV=3 - [IF1A_MOUSE]	29.17	17	3	5	19	0.44	0.66	0.17	0.48
Q6ZWV7	60S ribosomal protein L35 OS=Mus musculus GN=Rpl35 PE=2 SV=1 [RL35_MOUSE]	34.15	4	5	7	39	-0.19	-0.24	-0.21	-0.19
Q76LS9-2	Isoform 2 of Protein FAM63A OS=Mus musculus GN=Fam63a - [FA63A_MOUSE]	4.79	3	2	2	5	0.97	0.98	0.48	0.49
Q8BHZ0	Protein FAM49A OS=Mus musculus GN=Fam49a PE=2 SV=1 - [FA49A_MOUSE]	21.67	1	3	5	42	-0.61	-0.64	-0.67	-0.64
Q9D6Z7	Coiled-coil domain containing 82, isoform CRA_a OS=Mus musculus GN=Ccdc82 PE=2 SV=1 - [Q9D6Z7_MOUSE]	2.17	2	1	1	2	0.00	-0.06	0.06	0.00
Q9JHE3	Neutral ceramidase OS=Mus musculus GN=Asah2 PE=1 SV=1 - [ASAH2_MOUSE]	4.50	1	2	2	5	-0.79	-0.78	-0.61	-0.66
E9PWB1	Pleckstrin homology-like domain family B member 1 OS=Mus musculus GN=Phldb1 PE=2 SV=1 - [E9PWB1_MOUSE]	11.93	10	12	14	35	-0.36	-0.25	-0.33	-0.30
Q64521	Glycerol-3-phosphate dehydrogenase, mitochondrial OS=Mus musculus GN=Gpd2 PE=1 SV=2 - [GPDM_MOUSE]	32.74	2	19	19	96	-0.13	-0.23	-0.12	-0.18

Q9JL26-2	Isoform 2 of Formin-like protein 1 OS=Mus musculus GN=Fmnl1 - [FMNL_MOUSE]	7.71	4	7	7	12	0.37	0.02	0.00	-0.20
Q9D8S4	Oligoribonuclease, mitochondrial OS=Mus musculus GN=Rexo2 PE=1 SV=2 - [ORN_MOUSE]	36.29	1	8	8	44	-0.03	0.16	0.05	0.11
Q5DTW7-2	Isoform 2 of Uncharacterized protein KIAA1551 OS=Mus musculus GN=Kiaa1551 - [K1551_MOUSE]	3.65	3	4	4	5	-0.70	-1.31	-0.94	-1.55
Q8BGA9	Mitochondrial inner membrane protein OXA1L OS=Mus musculus GN=Oxa1l PE=2 SV=1 - [OXA1L_MOUSE]	6.70	1	3	3	6	-0.39	-0.60	-0.15	-0.36
Q8BP27-2	Isoform 2 of Swi5-dependent recombination DNA repair protein 1 homolog OS=Mus musculus GN=Sfr1 - [SFR1_MOUSE]	22.44	3	3	3	4	0.07	0.17	0.08	0.18
E9Q2X6	Structural maintenance of chromosomes protein OS=Mus musculus GN=Smc4 PE=2 SV=1 - [E9Q2X6_MOUSE]	5.95	3	7	7	11	0.43	0.21	0.41	0.17
P61922	4-aminobutyrate aminotransferase, mitochondrial OS=Mus musculus GN=Abat PE=1 SV=1 - [GABT_MOUSE]	42.60	5	16	16	209	-0.34	-0.22	-0.37	-0.31
Q8CGC6	RNA-binding protein 28 OS=Mus musculus GN=Rbm28 PE=1 SV=4 - [RBM28_MOUSE]	7.20	1	5	5	7	-0.06	0.25	-0.12	0.18
Q09200	Beta-1,4 N-acetylgalactosaminyltransferase 1 OS=Mus musculus GN=B4galnt1 PE=2 SV=1 - [B4GN1_MOUSE]	13.70	1	6	6	9	0.17	0.15	0.17	-0.08
Q924A2	Protein capicua homolog OS=Mus musculus GN=Cic PE=1 SV=2 - [CIC_MOUSE]	4.98	4	6	6	8	0.21	0.11	0.20	0.26
Q8BLY2	Probable threonine--tRNA ligase 2, cytoplasmic OS=Mus musculus GN=Tarsl2 PE=2 SV=1 - [SYTC2_MOUSE]	9.11	1	4	6	19	0.16	0.29	0.18	0.31
Q91ZF4	Cathepsin Q (Precursor) OS=Mus musculus GN=Ctsq PE=2 SV=1 - [Q91ZF4_MOUSE]	6.41	1	1	1	1	-0.11	-1.00	0.34	-0.54
P70372	ELAV-like protein 1 OS=Mus musculus GN=Elavl1 PE=1 SV=2 - [ELAV1_MOUSE]	39.88	1	11	11	98	0.04	0.08	0.09	0.07
Q8R0H9	ADP-ribosylation factor binding protein GGA1 OS=Mus musculus GN=Gga1 PE=1 SV=1 - [GGA1_MOUSE]	12.44	1	6	6	19	0.13	0.28	0.28	0.27

Q9D0C4-2	Isoform 2 of tRNA (guanine(37)-N1)-methyltransferase OS=Mus musculus GN=Trmt5 - [TRM5_MOUSE]	6.78	2	2	3	5	-0.47	0.04	-0.41	0.10
F6V619	Tankyrase-2 (Fragment) OS=Mus musculus GN=Tnks2 PE=2 SV=1 - [F6V619_MOUSE]	3.98	2	1	1	1	-0.41	0.08	-0.29	0.19
E9Q132	60S ribosomal protein L24 OS=Mus musculus GN=Rpl24 PE=2 SV=1 [E9Q132_MOUSE]	42.11	3	7	7	57	0.26	0.42	0.31	0.37
Q9D8X5	CCR4-NOT transcription complex subunit 8 OS=Mus musculus GN=Cnot8 PE=2 SV=1 - [CNOT8_MOUSE]	4.79	1	1	1	3	-0.01	0.14	0.13	0.29
Q71FD7	Filamin-binding LIM protein 1 OS=Mus musculus GN=Fblim1 PE=1 SV=2 - [FBLI1_MOUSE]	28.00	4	6	6	24	0.17	-0.08	0.47	0.31
Q58A65-2	Isoform 2 of C-Jun-amino-terminal kinase-interacting protein 4 OS=Mus musculus GN=Spag9 - [JIP4_MOUSE]	13.08	20	10	12	32	0.13	0.08	0.24	0.20
P52624	Uridine phosphorylase 1 OS=Mus musculus GN=Upp1 PE=1 SV=2 - [UPP1_MOUSE]	26.69	5	6	6	30	-0.40	-0.04	0.01	0.01
P01628	Ig kappa chain V-II region MOPC 511 OS=Mus musculus PE=1 SV=1 - [KV2A3_MOUSE]	21.24	1	1	1	7	0.23	0.01	0.26	0.04
Q8VD37	SH3-containing GRB2-like protein 3-interacting protein 1 OS=Mus musculus GN=Sgip1 PE=1 SV=1 [SGIP1_MOUSE]	1.12	3	1	1	2	-0.04	-0.08	0.00	-0.04
Q8R2Y8	Peptidyl-tRNA hydrolase 2, mitochondrial OS=Mus musculus GN=Ptrh2 PE=2 SV=1 - [PTH2_MOUSE]	25.41	1	3	3	71	0.18	0.33	0.35	0.29
Q9JK38	Glucosamine 6-phosphate N-acetyltransferase OS=Mus musculus GN=Gnpnat1 PE=2 SV=1 - [GNA1_MOUSE]	63.04	1	9	9	48	-0.03	-0.07	-0.04	-0.10
Q91YP3	Putative deoxyribose-phosphate aldolase OS=Mus musculus GN=Dera PE=2 SV=1 - [DEOC_MOUSE]	30.82	1	7	7	44	-0.02	0.12	0.09	0.15
O54786	DNA fragmentation factor subunit alpha OS=Mus musculus GN=Dffa PE=1 SV=2 - [DFFA_MOUSE]	3.63	1	1	1	1	-0.18	0.10	-0.17	0.12
Q9JK48	Endophilin-B1 OS=Mus musculus GN=Sh3glb1 PE=1 SV=1 - [SHLB1_MOUSE]	21.10	2	7	8	38	0.23	0.00	0.24	0.00
P70399	Tumor suppressor p53-binding protein 1 OS=Mus musculus GN=Tp53bp1 PE=1 SV=2 - [TP53B_MOUSE]	5.67	7	6	6	20	0.08	0.27	0.17	0.14

J3QMQ6	Protein Gm3336 OS=Mus musculus GN=Gm3336 PE=4 SV=1 - [J3QMQ6_MOUSE]	9.60	1	1	1	4	0.39	0.12	0.41	0.14
Q8R3R8	Gamma-aminobutyric acid receptor-associated protein-like 1 OS=Mus musculus GN=Gabarapl1 PE=1 SV=2 - [GBRL1_MOUSE]	5.98	1	1	1	1	-0.35	-0.09	-0.16	0.10
Q7TT50	Serine/threonine-protein kinase MRCK beta OS=Mus musculus GN=Cdc42bpb PE=1 SV=2 - [MRCKB_MOUSE]	10.86	2	15	18	59	0.01	0.10	0.00	0.03
Q61398	Procollagen C-endopeptidase enhancer 1 OS=Mus musculus GN=Pcolce PE=1 SV=2 - [PCOC1_MOUSE]	15.38	5	6	6	18	0.05	0.09	-0.08	0.02
Q91YL7-2	Isoform 2 of PGAP2-interacting protein OS=Mus musculus GN=Cwh43 - [PG2IP_MOUSE]	7.99	2	4	4	8	0.16	0.13	-0.02	-0.06
P63001	Ras-related C3 botulinum toxin substrate 1 OS=Mus musculus GN=Rac1 PE=1 SV=1 - [RAC1_MOUSE]	38.02	10	6	7	86	0.30	0.24	0.27	0.33
G3UW94	MCG14259, isoform CRA_b OS=Mus musculus GN=U2af1 PE=4 SV=1 - [G3UW94_MOUSE]	28.87	11	5	5	101	0.25	0.26	0.30	0.22
Q99K23	Ufm1-specific protease 2 OS=Mus musculus GN=Ufsp2 PE=1 SV=1 [UFSP2_MOUSE]	19.74	1	5	6	37	0.53	0.02	0.30	0.03
Q9D8B6	Protein FAM210B OS=Mus musculus GN=Fam210b PE=2 SV=3 - [F210B_MOUSE]	23.68	1	2	2	3	0.13	0.39	-0.14	0.13
E9Q5F1	Protein Olfr430 OS=Mus musculus GN=Olfr430 PE=2 SV=1 - [E9Q5F1_MOUSE]	2.21	1	1	1	1	0.28	0.41	0.17	0.30
Q80U38	Protein KHNYN OS=Mus musculus GN=Khnyn PE=2 SV=2 - [KHNYN_MOUSE]	6.41	1	3	3	3	0.60	0.23	0.41	0.14
Q8R0F8	Acylpyruvase FAHD1, mitochondrial OS=Mus musculus GN=Fahd1 PE=1 SV=2 - [FAHD1_MOUSE]	29.07	1	5	5	39	-0.19	0.05	-0.26	-0.06
Q8VD62	UPF0696 protein C11orf68 homolog OS=Mus musculus GN=Bles03 PE=2 SV=1 - [CK068_MOUSE]	12.75	2	3	3	15	-0.06	-0.29	-0.12	-0.22
Q80WT5-2	Isoform 2 of Aftiphilin OS=Mus musculus GN=Aftph - [AFTIN_MOUSE]	9.62	5	8	8	17	0.10	-0.24	0.20	-0.18
P97384	Annexin A11 OS=Mus musculus GN=Anxa11 PE=1 SV=2 - [ANX11_MOUSE]	32.01	2	13	14	244	0.12	0.08	0.09	0.06

Q9DBK7	MCG18845, isoform CRA_d OS=Mus musculus GN=Uba7 PE=2 SV=1 - [Q9DBK7_MOUSE]	9.83	3	8	9	29	0.05	0.05	0.00	-0.05
Q3U4I7	Pyridine nucleotide-disulfide oxidoreductase domain-containing protein 2 OS=Mus musculus GN=Pyroxd2 PE=2 SV=2 - [PYRD2_MOUSE]	3.28	1	1	1	1	0.49	0.07	0.40	-0.02
D3YVN7	Elongation factor Tu OS=Mus musculus GN=Gm9755 PE=3 SV=1 - [D3YVN7_MOUSE]	57.74	3	24	24	320	0.08	0.10	0.05	0.07
Q9CPU2	NADH dehydrogenase [ubiquinone] 1 beta subcomplex subunit 2, mitochondrial OS=Mus musculus GN=Ndufb2 PE=3 SV=1 - [NDUB2_MOUSE]	6.67	1	1	1	5	-0.06	-0.09	-0.13	-0.15
P09411	Phosphoglycerate kinase 1 OS=Mus musculus GN=Pgk1 PE=1 SV=4 - [PGK1_MOUSE]	80.82	4	24	31	888	0.20	0.35	0.10	0.26
Q9EQF5	Dihydropyrimidinase OS=Mus musculus GN=Dpys PE=2 SV=2 - [DPYS_MOUSE]	8.09	1	2	2	3	-0.16	-0.10	-0.17	-0.10
A2A5F7	Potassium voltage-gated channel, subfamily H (Eag-related), member 4 OS=Mus musculus GN=Kcnh4 PE=2 SV=1 - [A2A5F7_MOUSE]	1.77	1	1	1	1	-1.97	-2.96	-1.52	-2.51
Q80VC9	Calmodulin-regulated spectrin-associated protein 3 OS=Mus musculus GN=Camsap3 PE=1 SV=1 - [CAMP3_MOUSE]	12.06	3	10	10	19	0.19	0.15	0.11	0.07
Q5SF07	Insulin-like growth factor 2 mRNA-binding protein 2 OS=Mus musculus GN=Igf2bp2 PE=1 SV=1 - [IF2B2_MOUSE]	23.99	3	9	10	28	-0.54	-0.38	-0.53	-0.52
P50518	V-type proton ATPase subunit E 1 OS=Mus musculus GN=Atp6v1e1 PE=1 SV=2 - [VATE1_MOUSE]	52.65	2	11	11	118	0.09	0.19	0.06	0.14
A6ZI44	Fructose-bisphosphate aldolase OS=Mus musculus GN=Aldoa PE=2 SV=1 - [A6ZI44_MOUSE]	77.51	5	5	32	1610	-0.09	-0.15	-0.12	-0.22
Q8BNY6	Neuronal calcium sensor 1 OS=Mus musculus GN=Ncs1 PE=2 SV=3 - [NCS1_MOUSE]	3.68	1	1	1	1	-0.16	-0.16	0.06	0.06
Q6P542	ATP-binding cassette sub-family F member 1 OS=Mus musculus GN=Abcf1 PE=1 SV=1 - [ABCF1_MOUSE]	17.56	2	11	11	46	0.23	0.53	0.22	0.43

Q9CQF9	Prenylcysteine oxidase OS=Mus musculus GN=Pcyox1 PE=1 SV=1 - [PCYOX_MOUSE]	23.17	3	9	9	33	-0.05	-0.14	0.06	-0.04
Q921F4	Heterogeneous nuclear ribonucleoprotein L-like OS=Mus musculus GN=Hnrnpll PE=1 SV=3 - [HNRLL_MOUSE]	35.87	3	17	17	63	-0.13	0.00	0.05	0.08
O88307	Sortilin-related receptor OS=Mus musculus GN=Sorl1 PE=2 SV=3 - [SORL_MOUSE]	3.66	1	4	4	11	0.43	0.12	0.34	0.07
E9QA63	Microtubule-actin cross-linking factor 1 OS=Mus musculus GN=Macf1 PE=4 SV=2 - [E9QA63_MOUSE]	7.79	15	6	46	98	-0.10	-0.21	-0.02	-0.10
Q80U56	Late secretory pathway protein AVL9 homolog OS=Mus musculus GN=Avl9 PE=2 SV=2 - [AVL9_MOUSE]	2.16	1	2	2	2	-0.31	-0.19	-0.35	-0.23
Q5SW88	Ras-related protein Rab-1A OS=Mus musculus GN=Rab1 PE=2 SV=1 - [Q5SW88_MOUSE]	82.18	16	5	16	246	0.02	0.13	0.00	0.15
Q3U0B3	Dehydrogenase/reductase SDR family member 11 OS=Mus musculus GN=Dhrs11 PE=2 SV=1 - [DHR11_MOUSE]	26.54	2	6	6	32	0.00	0.07	-0.07	-0.25
Q3UHC7-2	Isoform 2 of Disabled homolog 2-interacting protein OS=Mus musculus GN=Dab2ip - [DAB2P_MOUSE]	9.11	9	6	7	14	-0.08	0.32	-0.01	0.28
Q8BI72	CDKN2A-interacting protein OS=Mus musculus GN=Cdkn2aip PE=2 SV=1 - [CARF_MOUSE]	4.62	1	2	2	3	-0.26	0.12	-0.24	0.15
O88685	26S protease regulatory subunit 6A OS=Mus musculus GN=Psmc3 PE=1 SV=2 - [PRS6A_MOUSE]	53.17	4	20	20	220	0.15	0.21	0.11	0.17
A2AMC3	GDP-fucose protein O-fucosyltransferase 1 OS=Mus musculus GN=Pofut1 PE=2 SV=1 - [A2AMC3_MOUSE]	18.47	5	4	4	12	-0.15	-0.62	0.10	0.20
Q3T9A3	Serine/threonine-protein kinase MARK2 OS=Mus musculus GN=Mark2 PE=2 SV=1 - [Q3T9A3_MOUSE]	21.94	19	10	14	27	-0.19	-0.20	-0.24	-0.09
Q8K057-2	Isoform 2 of Intraflagellar transport protein 80 homolog OS=Mus musculus GN=Ift80 - [IFT80_MOUSE]	3.22	2	2	3	8	0.38	0.57	0.26	0.46
Q8VDC0	Probable leucine--tRNA ligase, mitochondrial OS=Mus musculus GN=Lars2 PE=2 SV=1 - [SYLM_MOUSE]	5.76	1	5	5	11	-0.29	-0.21	-0.06	-0.07

Q9D154	Leukocyte elastase inhibitor A OS=Mus musculus GN=Serpinb1a PE=1 SV=1 - [ILEUA_MOUSE]	58.84	4	24	25	428	0.15	0.10	0.06	0.06
Q99KW3-1	Isoform 1 of TRIO and F-actin-binding protein OS=Mus musculus GN=Triobp - [TARA_MOUSE]	33.79	8	17	19	53	-0.23	-0.20	-0.10	-0.18
Q810B6	Ankyrin repeat and FYVE domain-containing protein 1 OS=Mus musculus GN=Ankfy1 PE=2 SV=2 - [ANFY1_MOUSE]	17.79	2	13	15	58	0.37	0.18	0.26	-0.03
A0JLY1	Coiled-coil domain-containing protein 173 OS=Mus musculus GN=Ccdc173 PE=2 SV=1 - [CC173_MOUSE]	1.28	1	1	1	3	-0.08	-0.20	-0.05	-0.17
Q9R0Q1	Synaptotagmin-like protein 4 OS=Mus musculus GN=Sytl4 PE=1 SV=1 - [SYTL4_MOUSE]	5.05	3	2	2	5	-0.64	-0.58	-0.63	-0.57
A2RSY6-3	Isoform 3 of TRMT1-like protein OS=Mus musculus GN=Trmt1l - [TRM1L_MOUSE]	2.81	3	1	1	3	0.31	0.30	0.48	0.46
Q9CV60	MCG146286 (Fragment) OS=Mus musculus GN=2310015B20Rik PE=2 SV=1 - [Q9CV60_MOUSE]	8.62	1	1	1	4	-0.01	0.07	-0.09	-0.01
L7N466	Protein Gm2382 OS=Mus musculus GN=Gm2382 PE=4 SV=1 - [L7N466_MOUSE]	22.17	7	4	4	13	0.21	0.18	0.08	0.13
E9PZ58	Spermine synthase OS=Mus musculus GN=Sms PE=2 SV=1 - [E9PZ58_MOUSE]	13.95	3	3	3	7	-0.55	-0.23	-0.28	-0.01
P46467	Vacuolar protein sorting associated protein 4B OS=Mus musculus GN=Vps4b PE=1 SV=2 - [VPS4B_MOUSE]	34.68	1	11	13	90	0.32	0.49	0.26	0.38
Q6PFH3	DDB1- and CUL4-associated factor 15 OS=Mus musculus GN=Dcaf15 PE=2 SV=1 - [DCA15_MOUSE]	4.83	2	2	2	3	-0.35	-0.77	-0.45	-0.87
A8Y5N8	Pleckstrin homology domain-containing family F member 2 (Fragment) OS=Mus musculus GN=Plekhf2 PE=2 SV=1 - [A8Y5N8_MOUSE]	18.78	2	1	3	7	-0.71	-1.17	-0.43	-0.90
H7BX54	Short-chain dehydrogenase/reductase 3 OS=Mus musculus GN=Dhrs3 PE=2 SV=1 [H7BX54_MOUSE]	8.13	5	1	1	2	-0.52	-0.46	-0.14	-0.09
Q8K1R7	Serine/threonine-protein kinase Nek9 OS=Mus musculus GN=Nek9 PE=1 SV=2 - [NEK9_MOUSE]	18.19	1	10	10	20	-0.03	-0.17	-0.10	-0.04

Q8BY98	Ankyrin repeat domain-containing protein SOWAHD OS=Mus musculus GN=Sowahd PE=2 SV=1 - [SWAHD_MOUSE]	4.28	1	1	1	1	-0.02	0.11	-0.17	-0.04
P14211	Calreticulin OS=Mus musculus GN=Calr PE=1 SV=1 - [CALR_MOUSE]	65.14	1	26	26	1352	0.00	0.12	-0.04	0.07
Q9CQW0	ER membrane protein complex subunit 6 OS=Mus musculus GN=Emc6 PE=2 SV=1 [EMC6_MOUSE]	10.91	1	1	1	10	0.25	0.39	0.26	0.36
O35085	Homeobox protein ARX OS=Mus musculus GN=Arx PE=2 SV=3 - [ARX_MOUSE]	3.37	1	1	1	2	0.41	0.23	0.18	0.00
Q9WTP7	GTP:AMP phosphotransferase AK3, mitochondrial OS=Mus musculus GN=Ak3 PE=1 SV=3 - [KAD3_MOUSE]	65.64	2	14	14	194	-0.09	-0.17	-0.10	-0.16
Q62393-3	Isoform 3 of Tumor protein D52 OS=Mus musculus GN=Tpd52 - [TPD52_MOUSE]	60.80	7	1	13	153	0.07	0.24	0.04	0.21
A2AW05	FACT complex subunit SSRP1 (Fragment) OS=Mus musculus GN=Ssrp1 PE=2 SV=1 - [A2AW05_MOUSE]	20.55	3	11	11	35	0.23	0.18	0.27	0.21
P62880	Guanine nucleotide-binding protein G(I)/G(S)/G(T) subunit beta-2 OS=Mus musculus GN=Gnb2 PE=1 SV=3 - [GBB2_MOUSE]	36.47	7	4	9	88	-0.03	-0.10	-0.09	-0.31
P32648	VIP peptides OS=Mus musculus GN=Vip PE=1 SV=1 - [VIP_MOUSE]	48.24	1	8	8	40	-0.50	-0.40	-0.49	-0.46
E0CY18	AN1-type zinc finger protein 2B (Fragment) OS=Mus musculus GN=Zfand2b PE=2 SV=1 - [E0CY18_MOUSE]	10.55	2	2	2	4	-0.22	-0.14	-0.04	0.05
Q9CXU4	MCG11298 OS=Mus musculus GN=Timm23 PE=2 SV=1 - [Q9CXU4_MOUSE]	13.40	2	2	2	5	-0.07	-0.56	-0.57	0.03
J3QMC5	Midasin OS=Mus musculus GN=Mdn1 PE=3 SV=1 - [J3QMC5_MOUSE]	1.25	2	4	5	16	0.22	0.43	0.19	-0.20
B1AW21	Myotubularin OS=Mus musculus GN=Mtm1 PE=4 SV=1 - [B1AW21_MOUSE]	22.38	3	9	9	13	-0.43	-0.23	-0.18	-0.21
D3Z1X8	SWI/SNF complex subunit SMARCC1 OS=Mus musculus GN=Smarcc1 PE=2 SV=1 - [D3Z1X8_MOUSE]	16.70	5	9	15	38	0.40	0.35	0.43	0.38
P23780	Beta-galactosidase OS=Mus musculus GN=Glb1 PE=2 SV=1 - [BGAL_MOUSE]	27.51	2	12	12	78	-0.52	-0.52	-0.48	-0.47

Q3UDW8-2	Isoform 2 of Heparan-alpha-glucosaminide N-acetyltransferase OS=Mus musculus GN=Hgsnat - [HGNAT_MOUSE]	1.76	2	1	1	1	-0.14	-0.17	-0.10	-0.13
A2AS05	Protein Helz2 OS=Mus musculus GN=Helz2 PE=2 SV=2 - [A2AS05_MOUSE]	2.89	3	6	6	8	0.27	0.20	0.00	0.01
Q9CQ60	6-phosphogluconolactonase OS=Mus musculus GN=Pgls PE=2 SV=1 - [6PGL_MOUSE]	52.53	4	11	11	140	-0.09	-0.02	-0.03	-0.01
Q62087	Serum paraoxonase/lactonase 3 OS=Mus musculus GN=Pon3 PE=1 SV=2 - [PON3_MOUSE]	29.94	2	6	6	54	-0.20	-0.18	-0.25	-0.17
Q80U72	Protein scribble homolog OS=Mus musculus GN=Scrib PE=1 SV=2 - [SCRIB_MOUSE]	15.57	6	14	17	45	0.25	-0.12	0.27	-0.15
Q8C605	6-phosphofructokinase OS=Mus musculus GN=Pfkp PE=2 SV=1 - [Q8C605_MOUSE]	30.48	5	18	21	174	0.37	0.36	0.26	0.33
Q3UI43	BRISC and BRCA1-A complex member 1 OS=Mus musculus GN=Babam1 PE=2 SV=1 - [BABA1_MOUSE]	25.23	1	5	5	19	0.12	0.06	0.14	0.03
Q8QZS1	3-hydroxyisobutyryl-CoA hydrolase, mitochondrial OS=Mus musculus GN=Hibch PE=1 SV=1 - [HIBCH_MOUSE]	39.22	2	15	15	61	0.23	0.21	0.13	0.17
Q9D5H4	Ferritin, mitochondrial OS=Mus musculus GN=Ftmt PE=2 SV=2 - [FTMT_MOUSE]	2.95	1	1	1	2	0.41	0.04	0.37	0.00
Q99L45	Eukaryotic translation initiation factor 2 subunit 2 OS=Mus musculus GN=Eif2s2 PE=1 SV=1 - [IF2B_MOUSE]	43.50	2	9	15	106	0.41	0.50	0.28	0.56
P63044	Vesicle-associated membrane protein 2 OS=Mus musculus GN=Vamp2 PE=1 SV=2 - [VAMP2_MOUSE]	35.34	2	2	5	64	0.10	-0.10	0.06	-0.10
Q8R2K1-2	Isoform 2 of Fucose mutarotase OS=Mus musculus GN=Fuom - [FUCM_MOUSE]	39.85	8	4	4	11	-0.24	-0.27	-0.14	-0.25
Q6GU68	Immunoglobulin superfamily containing leucine-rich repeat protein OS=Mus musculus GN=Islr PE=1 SV=1 - [ISLR_MOUSE]	7.94	1	3	3	10	-0.37	-0.50	-0.42	-0.42
Q5SU71	E3 ubiquitin/ISG15 ligase TRIM25 OS=Mus musculus GN=Trim25 PE=2 SV=1 - [Q5SU71_MOUSE]	20.13	3	10	10	33	0.02	0.00	0.04	0.02
Q99P72	Reticulon-4 OS=Mus musculus GN=Rtn4 PE=1 SV=2 - [RTN4_MOUSE]	22.38	4	4	16	159	0.06	-0.03	-0.03	-0.11

Q8BIK4	Dedicator of cytokinesis protein 9 OS=Mus musculus GN=Dock9 PE=1 SV=2 - [DOCK9_MOUSE]	2.04	5	3	3	10	-0.14	-0.09	0.16	0.21
P53395	Lipoamide acyltransferase component of branched-chain alpha-keto acid dehydrogenase complex, mitochondrial OS=Mus musculus GN=Dbt PE=2 SV=2 - [ODB2_MOUSE]	33.40	1	17	17	62	-0.21	-0.10	-0.19	-0.13
Q5SUR0	Phosphoribosylformylglycinamidine synthase OS=Mus musculus GN=Pfas PE=2 SV=1 - [PUR4_MOUSE]	17.80	3	16	16	71	-0.01	-0.02	0.11	-0.02
Q9CYC5	Kinetochore-associated protein DSN1 homolog OS=Mus musculus GN=Dsn1 PE=2 SV=1 - [DSN1_MOUSE]	4.31	1	1	1	1	0.24	-0.18	0.10	-0.32
P21278	Guanine nucleotide-binding protein subunit alpha-11 OS=Mus musculus GN=Gna11 PE=1 SV=1 - [GNA11_MOUSE]	47.91	2	8	15	103	-0.11	-0.29	-0.15	-0.24
Q91YR1	Twinfilin-1 OS=Mus musculus GN=Twf1 PE=1 SV=2 - [TWF1_MOUSE]	40.57	2	12	14	100	0.13	0.17	0.09	0.11
Q9QZ73	DCN1-like protein 1 OS=Mus musculus GN=Dcun1d1 PE=2 SV=1 - [DCNL1_MOUSE]	18.15	10	5	5	13	-0.11	-0.29	0.00	-0.18
A3KG01	Eph receptor B2 OS=Mus musculus GN=Ephb2 PE=2 SV=2 - [A3KG01_MOUSE]	8.82	14	5	7	22	0.11	-0.09	0.10	-0.01
H3BL26	Tetraspanin-6 (Fragment) OS=Mus musculus GN=Tspan6 PE=2 SV=1 - [H3BL26_MOUSE]	7.19	3	1	1	6	0.04	0.23	-0.13	0.02
P15920	V-type proton ATPase 116 kDa subunit a isoform 2 OS=Mus musculus GN=Atp6v0a2 PE=1 SV=2 - [VPP2_MOUSE]	5.49	1	3	4	12	0.01	0.04	0.04	0.11
Q3UUI3-2	Isoform 2 of Acyl-coenzyme A thioesterase THEM4 OS=Mus musculus GN=Them4 - [THEM4_MOUSE]	11.36	2	2	2	5	-0.09	-0.78	-0.30	-1.14
Q6ZWR6-4	Isoform 4 of Nesprin-1 OS=Mus musculus GN=Syne1 - [SYNE1_MOUSE]	2.66	9	14	21	40	0.36	0.37	0.36	0.20
Q9CQV5	28S ribosomal protein S24, mitochondrial OS=Mus musculus GN=Mrps24 PE=2 SV=1 - [RT24_MOUSE]	4.79	1	1	1	2	0.11	0.19	0.06	0.14
Q8VDG3	Poly(A)-specific ribonuclease PARN OS=Mus musculus GN=Parn PE=1 SV=1 - [PARN_MOUSE]	9.13	1	5	5	18	0.27	0.10	0.19	0.07

Q91VR8	Protein BRICK1 OS=Mus musculus GN=Brk1 PE=2 SV=1 - [BRK1_MOUSE]	73.33	2	7	7	23	0.31	0.18	0.15	0.18
Q9DCL9	Multifunctional protein ADE2 OS=Mus musculus GN=Paics PE=1 SV=4 - [PUR6_MOUSE]	29.65	3	12	13	56	0.14	0.08	0.24	0.12
Q6UL01	MORN repeat-containing protein 2 OS=Mus musculus GN=Morn2 PE=2 SV=1 - [MORN2_MOUSE]	17.72	1	1	1	1	0.22	0.72	-0.13	0.36
E9Q9E4	Cyclin-dependent kinase 4 (Fragment) OS=Mus musculus GN=Cdk4 PE=2 SV=1 - [E9Q9E4_MOUSE]	12.25	35	2	3	27	0.26	0.09	0.27	0.00
Q8K5B2	Multiple coagulation factor deficiency protein 2 homolog OS=Mus musculus GN=Mcfd2 PE=2 SV=1 - [MCFD2_MOUSE]	28.28	2	2	2	8	-0.03	0.42	-0.18	0.27
Q61001	Laminin subunit alpha-5 OS=Mus musculus GN=Lama5 PE=1 SV=4 - [LAMA5_MOUSE]	12.94	4	35	36	186	-0.58	-0.60	-0.49	-0.53
Q80UF7	TIR domain-containing adapter molecule 1 OS=Mus musculus GN=Ticam1 PE=1 SV=1 - [TCAM1_MOUSE]	0.96	1	1	1	1	0.15	-0.86	-0.04	-1.05
Q3U1V6	Ubiquitin-conjugating enzyme E2 variant 3 OS=Mus musculus GN=Uevld PE=2 SV=1 [UEVLD_MOUSE]	8.92	2	3	4	17	-0.23	0.08	-0.12	0.18
Q9QUH0	Glutaredoxin-1 OS=Mus musculus GN=Glrx PE=1 SV=3 - [GLRX1_MOUSE]	17.76	1	3	3	26	-0.15	-0.19	-0.34	-0.39
C6KI89	Cation channel sperm-associated protein subunit gamma 2 OS=Mus musculus GN=Catsperg2 PE=1 SV=1 - [CTSG2_MOUSE]	1.83	1	1	2	2	0.75	0.64	0.58	0.47
Q99LF4	tRNA-splicing ligase RtcB homolog OS=Mus musculus GN=D10Wsu52e PE=2 SV=1 - [RTCB_MOUSE]	51.49	1	22	22	192	0.01	0.04	0.05	0.06
Q9CQV4	Protein FAM134C OS=Mus musculus GN=Fam134c PE=2 SV=1 - [F134C_MOUSE]	7.73	2	2	3	9	-0.16	-0.01	-0.13	0.01
Q91WM3	U3 small nucleolar RNA-interacting protein 2 OS=Mus musculus GN=Rrp9 PE=1 SV=1 - [U3IP2_MOUSE]	3.16	1	1	1	1	0.26	0.31	0.59	0.65
Q3U034	Methyltransferase-like protein 4 OS=Mus musculus GN=Mettl4 PE=2 SV=1 - [METL4_MOUSE]	6.16	1	1	2	2	0.42	-2.56	0.34	-2.64

P61219	DNA-directed RNA polymerases I, II, and III subunit RPABC2 OS=Mus musculus GN=Polr2f PE=2 SV=1 - [RPAB2_MOUSE]	6.30	1	1	1	3	0.09	0.54	-0.24	0.20
Q8VD69	Arrestin domain containing 4 OS=Mus musculus GN=Arrdc4 PE=2 SV=1 - [Q8VD69_MOUSE]	6.76	1	1	1	1	-0.04	0.06	0.05	0.15
Q8K1C9	Leucine-rich repeat-containing protein 41 OS=Mus musculus GN=Lrrc41 PE=1 SV=3 - [LRC41_MOUSE]	1.98	1	1	2	4	-0.11	0.03	-0.03	0.11
Q922H4	Mannose-1-phosphate guanyltransferase alpha OS=Mus musculus GN=Gmppa PE=2 SV=1 - [GMPPA_MOUSE]	31.67	8	9	9	41	-0.23	0.08	-0.10	-0.05
Q9CZ30	Obg-like ATPase 1 OS=Mus musculus GN=Ola1 PE=1 SV=1 - [OLA1_MOUSE]	44.44	3	14	15	115	0.46	0.45	0.31	0.48
Q8VD31	Tapasin-related protein OS=Mus musculus GN=Tapbpl PE=2 SV=2 - [TPSNR_MOUSE]	6.87	1	3	3	13	-0.03	0.30	-0.01	0.07
Q8BZX4-2	Isoform 2 of Splicing regulatory glutamine/lysine-rich protein 1 OS=Mus musculus GN=Srek1 - [SREK1_MOUSE]	4.59	2	2	2	5	0.22	0.27	0.35	0.26
P56528	ADP-ribosyl cyclase 1 OS=Mus musculus GN=Cd38 PE=1 SV=2 - [CD38_MOUSE]	18.42	1	6	6	15	0.62	0.62	0.40	0.41
Q9D338	39S ribosomal protein L19, mitochondrial OS=Mus musculus GN=Mrpl19 PE=2 SV=1 - [RM19_MOUSE]	28.08	1	6	6	17	-0.13	-0.05	-0.15	0.09
Q8CHP8	Phosphoglycolate phosphatase OS=Mus musculus GN=Pgp PE=2 SV=1 - [PGP_MOUSE]	46.42	1	14	14	100	0.16	0.19	0.03	0.13
Q9D7V1	SH2 domain-containing protein 4A OS=Mus musculus GN=Sh2d4a PE=1 SV=1 - [SH24A_MOUSE]	14.01	1	5	5	9	0.57	0.64	0.61	0.67
Q6PAC3	DDB1- and CUL4-associated factor 13 OS=Mus musculus GN=Dcaf13 PE=2 SV=2 - [DCA13_MOUSE]	2.25	1	1	1	1	0.82	-0.17	0.16	-0.82
Q8BV79-2	Isoform 2 of TPR and ankyrin repeat-containing protein 1 OS=Mus musculus GN=Trank1 - [TRNK1_MOUSE]	2.77	2	2	3	7	0.13	-0.02	-0.18	-0.08
Q8BH43	Wiskott-Aldrich syndrome protein family member 2 OS=Mus musculus GN=Wasf2 PE=1 SV=1 - [WASF2_MOUSE]	16.50	2	11	11	55	-0.05	-0.06	0.02	-0.06
Q61990-2	Isoform 2 of Poly(rC)-binding protein 2 OS=Mus musculus GN=Pcbp2 - [PCBP2_MOUSE]	38.67	7	4	10	204	-0.20	-0.13	0.03	-0.06

Q80X81	Acetyl-Coenzyme A acetyltransferase 3 OS=Mus musculus GN=Acat3 PE=2 SV=1 [Q80X81_MOUSE]	39.29	2	2	11	84	-1.09	-0.52	-0.28	-0.26
F8WJ43	Merlin (Fragment) OS=Mus musculus GN=Nf2 PE=4 SV=1 - [F8WJ43_MOUSE]	5.18	3	2	2	2	0.11	-0.27	0.24	-0.15
A2ALA0	Surfeit locus protein 6 OS=Mus musculus GN=Surf6 PE=2 SV=1 - [A2ALA0_MOUSE]	13.46	2	2	3	5	-0.05	-0.18	0.02	-0.11
P58389	Serine/threonine-protein phosphatase 2A activator OS=Mus musculus GN=Ppp2r4 PE=1 SV=1 - [PTPA_MOUSE]	32.51	6	8	8	67	0.24	0.13	0.25	0.08
Q8R4H2	Rho guanine nucleotide exchange factor 12 OS=Mus musculus GN=Arhgef12 PE=1 SV=2 - [ARHGC_MOUSE]	5.31	2	6	7	20	0.01	0.05	0.10	0.03
Q8R0L1-2	Isoform 2 of Signal-transducing adaptor protein 2 OS=Mus musculus GN=Stap2 - [STAP2_MOUSE]	5.15	2	1	1	1	-0.24	-0.35	0.03	-0.08
A2AFG8	Neural cell adhesion molecule L1 OS=Mus musculus GN=L1cam PE=2 SV=1 - [A2AFG8_MOUSE]	20.72	7	19	20	106	-0.37	-0.34	-0.37	-0.30
E9Q913	Protein G430095P16Rik OS=Mus musculus GN=G430095P16Rik PE=4 SV=1 - [E9Q913_MOUSE]	7.48	1	1	1	1	0.52	0.30	0.27	0.05
Q5SUQ9-2	Isoform 2 of CST complex subunit CTC1 OS=Mus musculus GN=Ctc1 - [CTC1_MOUSE]	1.16	4	2	2	4	-0.08	-0.02	0.00	0.07
Q80TH2-1	Isoform 1 of Protein LAP2 OS=Mus musculu GN=Erbb2ip - [LAP2_MOUSE]	11.34	4	13	13	40	-0.03	0.07	0.06	0.07
Q8CEC0-3	Isoform 3 of Nuclear pore complex protein Nup88 OS=Mus musculus GN=Nup88 - [NUP88_MOUSE]	8.63	3	6	6	10	0.33	0.57	0.13	0.38
O08919	Numb-like protein OS=Mus musculus GN=Numbl PE=1 SV=3 - [NUMBL_MOUSE]	5.63	1	1	3	8	-0.19	-0.42	-0.13	-0.36
Q80SX0	StAR-related lipid transfer protein 4 OS=Mus musculus GN=Stard4 PE=2 SV=1 - [Q80SX0_MOUSE]	7.59	2	1	1	6	0.30	0.54	-0.07	0.33
Q6IRU5-2	Isoform 2 of Clathrin light chain B OS=Mus musculus GN=Cltb - [CLCB_MOUSE]	34.60	3	1	9	79	0.11	-0.02	0.02	-0.11
Q8C436-2	Isoform 2 of Protein-lysine methyltransferase METTL21D OS=Mus musculus GN=Mettl21d - [MT21D_MOUSE]	24.72	2	1	1	1	0.38	0.58	-0.08	0.11

Q9DCX2	ATP synthase subunit d, mitochondrial OS=Mus musculus GN=Atp5h PE=1 SV=3 - [ATP5H_MOUSE]	88.82	4	16	16	266	0.05	0.16	-0.01	0.14
Q499X9	Methionine--tRNA ligase, mitochondrial OS=Mus musculus GN=Mars2 PE=2 SV=2 - [SYMM_MOUSE]	6.83	1	4	4	5	-0.34	-0.32	0.32	0.16
B1ASP2	Tyrosine-protein kinase OS=Mus musculus GN=Jak1 PE=3 SV=1 - [B1ASP2_MOUSE]	5.38	9	4	5	14	0.01	-0.09	0.01	-0.07
Q6P8X1	Sorting nexin-6 OS=Mus musculus GN=Snx6 PE=1 SV=2 - [SNX6_MOUSE]	27.83	1	12	14	70	0.11	0.30	0.17	0.32
Q9Z0G9-2	Isoform 2 of Claudin-3 OS=Mus musculus GN=Cldn3 - [CLD3_MOUSE]	16.58	2	3	3	6	0.06	-0.80	0.29	-0.56
E9QK16	Protocadherin Fat 3 OS=Mus musculus GN=Fat3 PE=2 SV=1 - [E9QK16_MOUSE]	1.12	3	2	3	3	0.38	0.47	0.19	0.27
Q9WTP6-2	Isoform 2 of Adenylate kinase 2, mitochondrial OS=Mus musculus GN=Ak2 - [KAD2_MOUSE]	56.47	2	11	14	305	0.19	0.28	0.06	0.21
Q5RL20	39S ribosomal protein L43, mitochondrial OS=Mus musculus GN=Mrpl43 PE=2 SV=1 - [Q5RL20_MOUSE]	22.64	2	4	4	13	-0.06	0.02	0.01	0.14
Q9D8L5	Coiled-coil domain-containing protein 91 OS=Mus musculus GN=Ccdc91 PE=2 SV=2 - [CCD91_MOUSE]	9.73	1	3	4	10	-0.12	-0.11	0.15	0.12
Q8C6B0	MCG20149, isoform CRA_a OS=Mus musculus GN=Mettl7a1 PE=2 SV=1 - [Q8C6B0_MOUSE]	40.98	3	4	8	66	-0.18	-0.04	-0.28	-0.16
Q3TDD9	Protein phosphatase 1 regulatory subunit 21 OS=Mus musculus GN=Ppp1r21 PE=2 SV=2 - [PPR21_MOUSE]	18.33	3	10	10	25	0.05	0.08	0.19	0.33
Q9D2Q8	Protein S100-A14 OS=Mus musculus GN=S100a14 PE=2 SV=1 - [S10AE_MOUSE]	51.92	2	6	6	46	0.64	0.82	0.66	0.73
Q8BGR9	Ubiquitin-like domain-containing CTD phosphatase 1 OS=Mus musculus GN=Ublcp1 PE=1 SV=1 - [UBCP1_MOUSE]	22.96	2	7	7	20	0.03	0.36	0.04	0.44
Q60749	KH domain-containing, RNA-binding, signal transduction-associated protein 1 OS=Mus musculus GN=Khdrbs1 PE=1 SV=2 - [KHDR1_MOUSE]	29.57	3	8	10	46	0.23	-0.02	0.19	-0.09

P97863-3	Isoform 3 of Nuclear factor 1 B-type OS=Mus musculus GN=Nfib - [NFIB_MOUSE]	8.33	8	2	3	6	-0.05	-0.09	-0.16	-0.20
P52927	High mobility group protein HMGI-C OS=Mus musculus GN=Hmga2 PE=1 SV=1 - [HMGA2_MOUSE]	40.74	2	3	3	5	0.27	0.01	0.23	-0.03
Q62189	U1 small nuclear ribonucleoprotein A OS=Mus musculus GN=Snrpa PE=2 SV=3 [SNRPA_MOUSE]	26.83	4	5	7	73	-0.08	-0.18	-0.06	-0.21
Q8R274	MCG142205 OS=Mus musculus GN=Vmn1r210 PE=4 SV=1 - [Q8R274_MOUSE]	10.13	1	1	1	1	-0.90	-1.11	-0.60	-0.81
P62274	40S ribosomal protein S29 OS=Mus musculus GN=Rps29 PE=2 SV=2 - [RS29_MOUSE]	23.21	2	2	2	12	0.35	0.40	0.17	0.27
F8WIG5	Protein diaphanous homolog 3 OS=Mus musculus GN=Diap3 PE=4 SV=2 - [F8WIG5_MOUSE]	1.62	2	1	1	3	0.87	1.43	1.56	2.12
Q6PCP5	Mitochondrial fission factor OS=Mus musculus GN=Mff PE=1 SV=1 - [MFF_MOUSE]	13.75	7	2	2	3	-0.64	-0.32	-0.28	0.04
Q7TSG1-2	Isoform 2 of Centrosomal protein of 120 kDa OS=Mus musculus GN=Cep120 - [CE120_MOUSE]	1.61	2	1	1	2	-0.53	-0.11	-0.37	0.05
Q5U3K5	Rab-like protein 6 OS=Mus musculus GN=Rabl6 PE=1 SV=2 [RABL6_MOUSE]	4.83	1	3	3	8	0.08	0.10	0.12	0.15
Q8K4T5	Dual specificity protein phosphatase 19 OS=Mus musculus GN=Dusp19 PE=2 SV=1 - [DUS19_MOUSE]	2.73	2	1	1	1	0.14	0.23	0.18	0.27
Q9CRD0	OCIA domain-containing protein 1 OS=Mus musculus GN=Ociad1 PE=1 SV=1 - [OCAD1_MOUSE]	25.91	3	5	5	9	0.02	-0.13	-0.05	-0.02
Q9CXF4	TBC1 domain family member 15 OS=Mus musculus GN=Tbc1d15 PE=1 SV=1 - [TBC15_MOUSE]	9.24	1	6	6	14	0.26	0.25	-0.24	0.12
Q9WUH1	Transmembrane protein 115 OS=Mus musculus GN=Tmem115 PE=1 SV=1 - [TM115_MOUSE]	6.86	1	2	2	2	0.00	-0.32	0.24	-0.23
Q8C7V8-2	Isoform 2 of Coiled-coil domain-containing protein 134 OS=Mus musculus GN=Ccdc134 [CC134_MOUSE]	11.46	2	2	2	6	-0.19	0.17	-0.02	0.34
Q9D2Z1	Protein Ceacam20 OS=Mus musculus GN=Ceacam20 PE=2 SV=1 - [Q9D2Z1_MOUSE]	4.16	1	2	2	3	0.71	0.52	0.63	0.43

P19253	60S ribosomal protein L13a OS=Mus musculus GN=Rpl13a PE=1 SV=4 - [RL13A_MOUSE]	37.44	3	10	10	97	0.14	0.10	0.18	0.15
Q8C3J5	Dedicator of cytokinesis protein 2 OS=Mus musculus GN=Dock2 PE=1 SV=3 - [DOCK2_MOUSE]	4.54	3	6	6	8	-0.29	0.15	-0.26	0.09
Q80XI6	Mitogen-activated protein kinase kinase kinase 11 OS=Mus musculus GN=Map3k11 PE=1 SV=1 - [M3K11_MOUSE]	6.35	1	2	2	5	0.69	0.79	0.22	-0.33
Q9DCN2-2	Isoform 2 of NADH-cytochrome b5 reductase 3 OS=Mus musculus GN=Cyb5r3 - [NB5R3_MOUSE]	50.00	4	13	13	212	-0.11	-0.07	-0.10	-0.11
Q9QXD8	LIM domain-containing protein 1 OS=Mus musculus GN=Limd1 PE=1 SV=2 - [LIMD1_MOUSE]	13.62	1	5	5	20	0.11	0.15	0.03	0.26
F7AJ01	SUN domain-containing protein 1 (Fragment) OS=Mus musculus GN=Sun1 PE=4 SV=1 - [F7AJ01_MOUSE]	5.98	1	1	1	10	-0.16	-0.19	-0.13	-0.14
P46938	Yorkie homolog OS=Mus musculus GN=Yap1 PE=1 SV=2 - [YAP1_MOUSE]	12.91	6	5	5	18	-0.15	-0.17	-0.07	-0.28
Q99KQ4	Nicotinamide phosphoribosyltransferase OS=Mus musculus GN=Nampt PE=1 SV=1 - [NAMPT_MOUSE]	42.57	1	18	18	85	0.31	0.23	0.30	0.28
P82349	Beta-sarcoglycan OS=Mus musculus GN=Sgcb PE=1 SV=1 - [SGCB_MOUSE]	20.00	1	4	4	24	-0.49	-0.89	-0.63	-0.82
Q8VD04	GRIP1-associated protein 1 OS=Mus musculus GN=Gripap1 PE=1 SV=1 - [GRAP1_MOUSE]	19.11	6	11	11	30	-0.18	-0.07	-0.19	0.06
P54731	FAS-associated factor 1 OS=Mus musculus GN=Faf1 PE=1 SV=2 - [FAF1_MOUSE]	14.79	1	6	6	37	0.08	0.04	0.27	0.18
Q9WUB0	RanBP-type and C3HC4-type zinc finger containing protein 1 OS=Mus musculus GN=Rbck1 PE=1 SV=2 - [HOIL1_MOUSE]	10.63	1	4	4	9	0.06	-0.13	-0.02	-0.20
Q9DB77	Cytochrome b-c1 complex subunit 2, mitochondrial OS=Mus musculus GN=Uqcrc2 PE=1 SV=1 - [QCR2_MOUSE]	47.68	1	20	21	576	-0.14	-0.17	-0.13	-0.15
E9QNR5	Thrombospondin type-1 domain-containing protein 7A OS=Mus musculus GN=Thsd7a PE=2 SV=1 - [E9QNR5_MOUSE]	1.34	5	2	2	3	-0.03	-0.32	-0.32	-0.24
F6SDD7	DNA topoisomerase (Fragment) OS=Mus musculus GN=Top3b PE=3 SV=1 - [F6SDD7_MOUSE]	7.78	2	3	3	8	0.09	-0.35	0.16	-0.53

P28658	Ataxin-10 OS=Mus musculus GN=Atxn10 PE=1 SV=2 - [ATX10_MOUSE]	14.95	2	7	7	27	0.17	0.16	0.05	0.02
Q8R2G4-8	Isoform 7 of Ecto-ADP-ribosyltransferase 3 OS=Mus musculus GN=Art3 - [NAR3_MOUSE]	17.17	15	3	4	19	-0.80	-0.40	-0.77	-0.63
Q8JZM6	TRAF-interacting protein with FHA domain-containing protein B OS=Mus musculus GN=Tifab PE=2 SV=1 - [TIFAB_MOUSE]	17.01	1	1	1	3	-0.06	-0.79	-0.09	-0.82
Q99NG0	Helicase ARIP4 OS=Mus musculus GN=Rad54l2 PE=1 SV=1 - [ARIP4_MOUSE]	1.09	2	1	2	3	0.19	0.23	0.18	0.22
D3Z3J6	Polyadenylate-binding protein-interacting protein 1 OS=Mus musculus GN=Paip1 PE=2 SV=1 - [D3Z3J6_MOUSE]	17.17	7	6	7	25	0.00	-0.06	0.10	-0.03
Q8BKL6	Gamma-tubulin complex component 4 OS=Mus musculus GN=Tubgcp4 PE=2 SV=1 - [Q8BKL6_MOUSE]	1.77	3	1	1	1	-0.41	-0.46	-0.38	-0.44
Q80XN0	D-beta-hydroxybutyrate dehydrogenase, mitochondrial OS=Mus musculus GN=Bdh1 PE=1 SV=2 - [BDH_MOUSE]	41.69	2	11	12	230	0.10	0.10	0.10	0.06
Q8VDL4-3	Isoform 3 of ADP-dependent glucokinase OS=Mus musculus GN=Adpgk - [ADPGK_MOUSE]	16.57	3	4	4	20	-0.12	-0.35	-0.08	-0.32
Q8BVY0	Protein Rsl1d1 OS=Mus musculus GN=Rsl1d1 PE=2 SV=1 - [Q8BVY0_MOUSE]	27.65	1	9	9	31	0.50	0.32	0.44	0.10
P26011	Integrin beta-7 OS=Mus musculus GN=Itgb7 PE=1 SV=2 - [ITB7_MOUSE]	3.47	1	2	3	5	-0.24	-0.23	0.01	0.02
A0A4W9	Neuronal growth regulator 1 OS=Mus musculus GN=Negr1 PE=2 SV=1 - [A0A4W9_MOUSE]	11.55	4	2	2	9	-1.98	-0.81	-0.52	-0.81
Q8R409	Protein HEXIM1 OS=Mus musculus GN=Hexim1 PE=1 SV=1 - [HEXI1_MOUSE]	34.83	1	7	7	33	0.10	0.05	0.20	0.06
Q8R242-2	Isoform 2 of Di-N-acetylchitobiase OS=Mus musculus GN=Ctbs - [DIAC_MOUSE]	8.97	2	2	2	6	-0.43	-0.52	-0.07	-0.16
Q8QZV4	Serine/threonine-protein kinase 32C OS=Mus musculus GN=Stk32c PE=2 SV=1 - [ST32C_MOUSE]	4.30	1	1	1	2	0.09	-0.91	0.48	-0.52
G3X8U3	MCG6895 OS=Mus musculus GN=2210016F16Rik PE=4 SV=1 - [G3X8U3_MOUSE]	17.46	1	6	6	42	0.02	0.05	0.05	0.07

D3Z4U0	Zinc finger Ran-binding domain-containing protein 2 OS=Mus musculus GN=Zranb2 PE=2 SV=1 - [D3Z4U0_MOUSE]	12.73	5	4	4	8	0.06	0.21	-0.08	0.15
O54983	Thiomorpholine-carboxylate dehydrogenase OS=Mus musculus GN=Crym PE=1 SV=1 - [CRYM_MOUSE]	5.75	1	2	2	3	-0.16	-0.48	-0.11	-0.45
P99026	Proteasome subunit beta type-4 OS=Mus musculus GN=Psmb4 PE=1 SV=1 - [PSB4_MOUSE]	37.88	1	6	6	90	0.07	-0.24	-0.02	-0.10
Q8CGC4	Protein LSM14 homolog B OS=Mus musculus GN=Lsm14b PE=2 SV=3 - [LS14B_MOUSE]	4.68	2	1	2	17	-0.26	0.17	-0.85	-0.43
Q9WU81-2	Isoform 2 of Sugar phosphate exchanger 2 OS=Mus musculus GN=Slc37a2 - [SPX2_MOUSE]	4.74	2	2	2	6	0.22	-0.21	0.23	-0.20
P52633	Signal transducer and transcription activator 6 OS=Mus musculus GN=Stat6 PE=2 SV=2 - [STAT6_MOUSE]	5.62	2	5	6	13	0.00	0.15	-0.02	0.14
Q62009-5	Isoform 5 of Periostin OS=Mus musculus GN=Postn - [POSTN_MOUSE]	46.10	3	1	27	346	0.48	0.52	0.34	0.38
Q99LE6	ATP-binding cassette sub-family F member 2 OS=Mus musculus GN=Abcf2 PE=2 SV=1 - [ABCF2_MOUSE]	17.36	1	10	10	32	0.29	0.35	0.34	0.36
Q9D8W7	OCIA domain-containing protein 2 OS=Mus musculus GN=Ociad2 PE=2 SV=1 - [OCAD2_MOUSE]	22.73	1	4	4	25	0.35	0.55	0.29	0.48
Q99NF7	Ppm1b protein OS=Mus musculus GN=Ppm1b PE=2 SV=1 - [Q99NF7_MOUSE]	26.00	7	8	11	79	-0.08	0.09	-0.03	0.10
Q9D0J8	Parathymosin OS=Mus musculus GN=Ptms PE=2 SV=3 - [PTMS_MOUSE]	10.89	1	1	1	40	-0.02	0.03	-0.16	-0.01
P05063	Fructose-bisphosphate aldolase C OS=Mus musculus GN=Aldoc PE=1 SV=4 - [ALDOC_MOUSE]	49.31	1	10	17	524	-0.30	-0.41	-0.21	-0.19
Q9JLV2-2	Isoform 2 of Short transient receptor potential channel 4-associated protein OS=Mus musculus GN=Trpc4ap - [TP4AP_MOUSE]	6.21	2	2	2	3	-0.77	-0.20	-0.41	0.16
Q9D0L8	mRNA cap guanine-N7 methyltransferase OS=Mus musculus GN=Rnmt PE=1 SV=1 - [MCES_MOUSE]	11.83	6	4	4	10	0.17	0.25	0.16	0.21
Q8CH18-3	Isoform 3 of Cell division cycle and apoptosis regulator protein 1 OS=Mus musculus GN=Ccar1 - [CCAR1_MOUSE]	13.65	4	12	13	43	0.06	0.14	0.12	0.12

D3YVV1	Cell cycle control protein 50A OS=Mus musculus GN=Tmem30a PE=2 SV=1 - [D3YVV1_MOUSE]	10.67	2	2	2	6	-0.01	0.08	-0.09	0.00
E0CY96	tRNA-splicing endonuclease subunit Sen15 OS=Mus musculus GN=Tsen15 PE=2 SV=1 - [E0CY96_MOUSE]	14.06	2	1	1	4	-0.32	-0.31	-0.24	-0.23
F8VPN4	Protein Agl OS=Mus musculus GN=Agl PE=4 SV=1 - [F8VPN4_MOUSE]	12.60	5	15	16	31	-0.52	-0.48	-0.36	-0.51
Q60766-2	Isoform 2 of Immunity-related GTPase family M protein 1 OS=Mus musculus GN=Irgm1 - [IRGM1_MOUSE]	15.52	3	6	6	18	0.00	0.04	-0.12	-0.06
Q9Z1G3	V-type proton ATPase subunit C 1 OS=Mus musculus GN=Atp6v1c1 PE=1 SV=4 - [VATC1_MOUSE]	32.98	1	13	13	30	0.33	0.20	0.23	0.13
O35250-2	Isoform 2 of Exocyst complex component 7 OS=Mus musculus GN=Exoc7 - [EXOC7_MOUSE]	15.31	5	9	9	18	0.05	0.23	0.13	0.09
H3BIY2	Protein 2510003E04Rik OS=Mus musculus GN=2510003E04Rik PE=2 SV=1 - [H3BIY2_MOUSE]	12.24	5	3	3	3	-0.23	-0.35	-0.41	-0.53
E0CYJ0	MOB-like protein phocein (Fragment) OS=Mus musculus GN=Mob4 PE=2 SV=1 [E0CYJ0_MOUSE]	24.73	4	4	4	12	-0.25	-0.20	-0.05	-0.35
Q61411	GTPase HRas OS=Mus musculus GN=Hras1 PE=1 SV=2 - [RASH_MOUSE]	44.44	5	4	6	64	0.30	-0.07	0.18	-0.14
B0R091	Protein Gm20056 OS=Mus musculus GN=Chp1 PE=2 SV=1 - [B0R091_MOUSE]	70.16	3	10	10	47	-0.61	-0.46	-0.61	-0.49
O88673	Diacylglycerol kinase alpha OS=Mus musculus GN=Dgka PE=2 SV=2 - [DGKA_MOUSE]	8.36	1	5	5	7	-0.05	-0.21	0.00	0.28
B2RXC1	Trafficking protein particle complex subunit 11 OS=Mus musculus GN=Trappc11 PE=2 SV=1 - [TPC11_MOUSE]	5.83	2	6	6	12	0.41	0.51	0.36	0.39
Q3TP05	Angiomotin-like protein 2 OS=Mus musculus GN=Amotl2 PE=2 SV=1 - [Q3TP05_MOUSE]	4.32	4	2	4	6	-0.47	-0.75	-0.05	-0.33
P97429	Annexin A4 OS=Mus musculus GN=Anxa4 PE=2 SV=4 - [ANXA4_MOUSE]	64.58	3	21	22	527	0.24	0.33	0.24	0.30
E0CY15	DNA-directed RNA polymerase, mitochondrial OS=Mus musculus GN=Polrmt PE=2 SV=1 - [E0CY15_MOUSE]	2.44	3	1	1	1	0.73	0.95	0.28	0.50

G8JL68	Myelin expression factor 2 (Fragment) OS=Mus musculus GN=Myef2 PE=4 SV=1 - [G8JL68_MOUSE]	26.23	8	9	9	21	-0.13	0.05	-0.03	0.13
P61953	Guanine nucleotide-binding protein G(I)/G(S)/G(O) subunit gamma-11 OS=Mus musculus GN=Gng11 PE=1 SV=1 - [GBG11_MOUSE]	9.59	1	1	1	2	-0.65	-0.71	-0.53	-0.59
E9PVB3	Coiled-coil domain-containing protein 175 OS=Mus musculus GN=Ccdc175 PE=2 SV=1 - [CC175_MOUSE]	0.85	1	1	1	2	0.42	0.32	0.32	0.22
P12960	Contactin-1 OS=Mus musculus GN=Cntn1 PE=1 SV=1 - [CNTN1_MOUSE]	10.78	1	9	9	16	-0.07	-0.01	-0.06	-0.18
Q9QYJ0	DnaJ homolog subfamily A member 2 OS=Mus musculus GN=Dnaja2 PE=1 SV=1 - [DNJA2_MOUSE]	49.27	1	16	16	105	0.11	0.23	0.04	0.15
Q91VC3	Eukaryotic initiation factor 4A-III OS=Mus musculus GN=Eif4a3 PE=2 SV=3 - [IF4A3_MOUSE]	45.99	3	17	20	137	-0.06	0.02	-0.03	-0.07
Q8CAQ8-3	Isoform 3 of Mitochondrial inner membrane protein OS=Mus musculus GN=Immt - [IMMT_MOUSE]	56.56	3	2	40	250	-0.14	-0.55	-0.18	-0.53
Q811G0-2	Isoform 2 of Protein PTHB1 OS=Mus musculus GN=Bbs9 - [PTHB1_MOUSE]	4.32	3	2	2	8	-0.16	0.21	0.57	0.94
P19157	Glutathione S-transferase P 1 OS=Mus musculus GN=Gstp1 PE=1 SV=2 - [GSTP1_MOUSE]	65.71	3	10	10	630	-0.30	-0.23	-0.31	-0.30
P32883-2	Isoform 2B of GTPase KRas OS=Mus musculus GN=Kras - [RASK_MOUSE]	38.30	5	3	5	69	-0.17	-0.07	-0.27	-0.09
Q8K419	Galectin-4 OS=Mus musculus GN=Lgals4 PE=1 SV=2 - [LEG4_MOUSE]	53.99	2	17	17	718	-0.41	-0.41	-0.35	-0.38
Q62084	Protein phosphatase 1 regulatory subunit 14B OS=Mus musculus GN=Ppp1r14b PE=1 SV=2 - [PP14B_MOUSE]	17.01	1	2	2	26	-0.12	-0.19	-0.15	-0.10
Q7TSJ2	Microtubule-associated protein 6 OS=Mus musculus GN=Map6 PE=1 SV=2 - [MAP6_MOUSE]	19.98	4	12	12	24	-0.11	-0.03	-0.14	-0.02
E9PWH2	Protein Card6 OS=Mus musculus GN=Card6 PE=2 SV=1 - [E9PWH2_MOUSE]	0.60	1	1	1	1	0.28	-0.32	0.38	-0.22
Q60605-2	Isoform Smooth muscle of Myosin light polypeptide 6 OS=Mus musculus GN=Myl6 - [MYL6_MOUSE]	84.77	1	1	12	2014	-0.38	-0.36	-0.40	-0.42

Q05CL8	La-related protein 7 OS=Mus musculus GN=Larp7 PE=1 SV=2 [LARP7_MOUSE]	14.21	3	6	7	12	0.19	0.07	0.02	0.14
E9PYR6	Otoferlin OS=Mus musculus GN=Otof PE=4 SV=1 - [E9PYR6_MOUSE]	4.26	5	5	6	6	0.51	0.03	0.39	0.09
P97449	Aminopeptidase N OS=Mus musculus GN=Anpep PE=1 SV=4 - [AMPN_MOUSE]	27.12	1	20	20	141	-0.48	-0.44	-0.46	-0.46
P62915	Transcription initiation factor IIB OS=Mus musculus GN=Gtf2b PE=1 SV=1 - [TF2B_MOUSE]	18.67	1	6	6	10	0.02	-0.28	-0.04	-0.34
Q8VBX6-2	Isoform 2 of Multiple PDZ domain protein OS=Mus musculus GN=Mpdz - [MPDZ_MOUSE]	2.08	9	3	3	4	-1.35	-0.40	-0.85	0.11
Q80U78-2	Isoform 2 of Pumilio homolog 1 OS=Mus musculus GN=Pum1 - [PUM1_MOUSE]	12.23	9	8	13	38	-0.10	-0.12	0.02	0.10
Q8K3G5	Inactive serine/threonine-protein kinase VRK3 OS=Mus musculus GN=Vrk3 PE=1 SV=2 - [VRK3_MOUSE]	13.47	5	4	4	14	0.15	0.18	0.14	0.20
Q5SVG5	AP-1 complex subunit beta-1 OS=Mus musculus GN=Ap1b1 PE=2 SV=1 - [Q5SVG5_MOUSE]	34.50	8	14	31	287	0.00	-0.06	-0.11	-0.14
O55026	Ectonucleoside triphosphate diphosphohydrolase 2 OS=Mus musculus GN=Entpd2 PE=1 SV=2 - [ENTP2_MOUSE]	12.32	2	4	5	17	-0.61	-0.44	-0.49	-0.44
Q8CIH5	1-phosphatidylinositol 4,5-bisphosphate phosphodiesterase gamma-2 OS=Mus musculus GN=Plcg2 PE=1 SV=1 - [PLCG2_MOUSE]	10.12	4	9	11	30	0.58	0.36	0.48	0.41
E9Q467	Protein Abcc4 OS=Mus musculus GN=Abcc4 PE=2 SV=1 - [E9Q467_MOUSE]	0.72	2	1	1	1	0.89	0.26	0.16	-0.47
P15066	Transcription factor jun-D OS=Mus musculus GN=Jund PE=1 SV=1 - [JUND_MOUSE]	17.30	3	3	3	3	-0.56	0.13	-0.45	0.24
Q8R2N2	Cirhin OS=Mus musculus GN=Cirh1a PE=2 SV=3 - [CIR1A_MOUSE]	8.60	1	5	5	12	-0.32	-0.08	-0.20	-0.07
O35737	Heterogeneous nuclear ribonucleoprotein H OS=Mus musculus GN=Hnrnph1 PE=1 SV=3 - [HNRH1_MOUSE]	44.99	3	7	14	299	-0.02	-0.03	-0.01	0.02
A2AH75	Kinesin family member 1B, isoform CRA_a OS=Mus musculus GN=Kif1b PE=3 SV=1 [A2AH75_MOUSE]	4.26	2	2	5	15	0.07	0.59	0.19	0.71

A2VDH3	Leucine-rich repeat-containing protein 38 OS=Mus musculus GN=Lrrc38 PE=2 SV=2 - [LRC38_MOUSE]	4.03	1	1	1	1	0.14	0.31	0.03	0.19
Q91VY9	Zinc finger protein 622 OS=Mus musculus GN=Znf622 PE=2 SV=1 - [ZN622_MOUSE]	5.88	1	3	3	5	0.12	-0.29	0.01	-0.44
Q9DB34	Charged multivesicular body protein 2a OS=Mus musculus GN=Chmp2a PE=1 SV=1 - [CHM2A_MOUSE]	18.92	3	3	4	21	-0.20	0.05	-0.12	0.14
Q9D0J4	ADP-ribosylation factor like protein 2 OS=Mus musculus GN=Arl2 PE=1 SV=1 - [ARL2_MOUSE]	15.22	1	2	2	6	-0.01	-0.22	0.17	-0.04
Q9CWK8	Sorting nexin-2 OS=Mus musculus GN=Snx2 PE=1 SV=2 - [SNX2_MOUSE]	43.74	1	20	22	114	0.17	0.07	0.20	0.12
Q63739	Protein tyrosine phosphatase type IVA 1 OS=Mus musculus GN=Ptp4a1 PE=1 SV=1 - [TP4A1_MOUSE]	39.88	2	6	7	47	0.31	0.07	0.13	0.08
Q8JZN1	Macrophage galactose N-acetyl-galactosamine specific lectin 2 OS=Mus musculus GN=Mgl2 PE=2 SV=1 - [Q8JZN1_MOUSE]	16.27	7	4	4	12	-0.06	-0.17	0.17	0.08
Q9D0K2	Succinyl-CoA:3-ketoacid coenzyme A transferase 1, mitochondrial OS=Mus musculus GN=Oxct1 PE=1 SV=1 - [SCOT1_MOUSE]	49.42	2	19	19	266	-0.09	-0.08	-0.05	-0.02
Q8K354	Carbonyl reductase [NADPH] 3 OS=Mus musculus GN=Cbr3 PE=2 SV=1 - [CBR3_MOUSE]	57.40	1	14	15	183	-0.01	-0.05	-0.05	-0.04
E9QMZ5	Plectin OS=Mus musculus GN=Plec PE=2 SV=1 - [E9QMZ5_MOUSE]	48.98	26	1	203	1465	0.21	-0.09	0.34	0.04
Q8C3Q9	Caspase-9 OS=Mus musculus GN=Casp9 PE=1 SV=1 - [CASP9_MOUSE]	4.19	3	2	2	4	0.12	-0.07	0.24	0.04
Q9D902	General transcription factor IIE subunit 2 OS=Mus musculus GN=Gtf2e2 PE=2 SV=2 - [T2EB_MOUSE]	7.53	1	2	2	3	0.08	0.37	0.25	0.53
Q62347	Cyclic AMP-responsive element-binding protein 1 OS=Mus musculus GN=Creb1 PE=2 SV=1 [Q62347_MOUSE]	10.80	22	4	4	16	-0.03	0.02	0.17	0.04
G3X956	FACT complex subunit SPT16 OS=Mus musculus GN=Supt16 PE=4 SV=1 - [G3X956_MOUSE]	17.19	2	18	18	78	0.30	0.28	0.24	0.28

P35486	Pyruvate dehydrogenase E1 component subunit alpha, somatic form, mitochondrial OS=Mus musculus GN=Pdha1 PE=1 SV=1 - [ODPA_MOUSE]	44.87	1	18	19	265	-0.24	-0.19	-0.23	-0.19
P97310	DNA replication licensing factor MCM2 OS=Mus musculus GN=Mcm2 PE=1 SV=3 - [MCM2_MOUSE]	16.92	1	13	13	35	0.24	-0.26	0.19	-0.19
Q99LJ6	Glutathione peroxidase 7 OS=Mus musculus GN=Gpx7 PE=2 SV=1 - [GPX7_MOUSE]	15.05	1	3	3	5	0.13	-0.10	0.16	-0.09
Q08093	Calponin-2 OS=Mus musculus GN=Cnn2 PE=2 SV=1 - [CNN2_MOUSE]	39.34	2	8	11	83	0.12	0.20	0.18	0.18
Q8BR93-2	Isoform 2 of Putative nuclease HARBI1 OS=Mus musculus GN=Harbi1 - [HARB1_MOUSE]	5.33	3	1	1	1	0.68	0.96	0.27	0.54
G3UW42	MCG49030 OS=Mus musculus GN=Gm6900 PE=4 SV=1 - [G3UW42_MOUSE]	50.44	6	6	6	82	0.27	0.25	0.28	0.37
Q8VDG5	Phosphopantothenate--cysteine ligase OS=Mus musculus GN=Ppcs PE=2 SV=1 - [PPCS_MOUSE]	26.37	2	8	8	36	-0.13	-0.15	-0.21	-0.13
D6RH74	UPF0462 protein C4orf33 homolog OS=Mus musculus GN=D3Ertd751e PE=2 SV=1 - [D6RH74_MOUSE]	13.41	4	1	1	2	0.20	-0.48	-0.06	-0.74
Q9WTM5	RuvB-like 2 OS=Mus musculus GN=Ruvbl2 PE=2 SV=3 - [RUVB2_MOUSE]	41.04	2	16	16	64	0.02	0.20	0.04	0.14
Q8VCM4	Lipoyltransferase 1, mitochondrial OS=Mus musculus GN=Lipt1 PE=2 SV=1 - [LIPT_MOUSE]	2.95	1	1	1	1	-0.65	-0.93	-0.54	-0.82
Q8BK30	NADH dehydrogenase [ubiquinone] flavoprotein 3, mitochondrial OS=Mus musculus GN=Ndufv3 PE=2 SV=1 - [NDUV3_MOUSE]	44.23	1	2	5	13	-0.22	0.05	-0.11	0.04
B2RXC2	Inositol 1,4,5-trisphosphate 3-kinase B OS=Mus musculus GN=Itpkb PE=2 SV=1 - [B2RXC2_MOUSE]	15.29	5	9	10	15	-0.34	-0.37	-0.05	-0.18
Q504M2	MCG53395 OS=Mus musculus GN=Pdp2 PE=2 SV=1 - [Q504M2_MOUSE]	5.64	1	3	3	4	0.04	-0.27	-0.02	-0.33
Q6P9J9	Anoctamin-6 OS=Mus musculus GN=Ano6 PE=1 SV=1 - [ANO6_MOUSE]	12.29	2	8	8	21	-0.19	-0.23	-0.32	-0.09
P25085-2	Isoform 2 of Interleukin 1 receptor antagonist protein OS=Mus musculus GN=Il1rn - [IL1RA_MOUSE]	22.01	4	3	3	17	0.53	0.28	0.34	0.25

P70696	Histone H2B type 1-A OS=Mus musculus GN=Hist1h2ba PE=1 SV=3 - [H2B1A_MOUSE]	52.76	1	1	8	316	0.14	0.37	-0.03	0.20
J3QQ16	Protein Col6a3 OS=Mus musculus GN=Col6a3 PE=4 SV=1 - [J3QQ16_MOUSE]	29.70	1	2	69	757	-0.55	-0.40	-0.63	-0.57
Q8VHK9	Probable ATP-dependent RNA helicase DHX36 OS=Mus musculus GN=Dhx36 PE=2 SV=2 - [DHX36_MOUSE]	13.29	1	7	8	16	-0.17	-0.11	-0.10	-0.60
Q9NYQ2	Hydroxyacid oxidase 2 OS=Mus musculus GN=Hao2 PE=2 SV=1 - [HAOX2_MOUSE]	42.49	1	12	12	138	-0.03	-0.10	-0.08	-0.11
Q9D0G0	28S ribosomal protein S30, mitochondrial OS=Mus musculus GN=Mrps30 PE=2 SV=1 - [RT30_MOUSE]	10.41	1	4	4	10	-0.10	0.04	-0.04	0.07
Q8CCP0	Nuclear export mediator factor Nemf OS=Mus musculus GN=Nemf PE=1 SV=2 - [NEMF_MOUSE]	2.73	3	2	3	4	0.05	-0.34	0.37	-0.61
Q80UW8	DNA-directed RNA polymerases I, II, and III subunit RPABC1 OS=Mus musculus GN=Polr2e PE=2 SV=1 - [RPAB1_MOUSE]	36.67	1	6	6	11	-0.13	-0.14	-0.11	-0.12
F8VQJ3	Laminin subunit gamma 1 OS=Mus musculus GN=Lamc1 PE=2 SV=1 - [F8VQJ3_MOUSE]	34.97	3	2	46	529	-0.92	-0.84	-0.83	-0.82
Q8BH69	Selenide, water dikinase 1 OS=Mus musculus GN=Sephs1 PE=2 SV=1 - [SPS1_MOUSE]	23.72	1	5	6	20	-0.16	-0.56	-0.11	-0.37
Q6P5E4	UDP-glucose:glycoprotein glucosyltransferase 1 OS=Mus musculus GN=Uggt1 PE=1 SV=4 - [UGGG1_MOUSE]	36.43	7	42	43	531	-0.02	0.00	-0.07	0.01
Q6ZQ03	Formin-binding protein 4 OS=Mus musculus GN=Fnbp4 PE=1 SV=2 - [FNBP4_MOUSE]	6.30	3	2	2	3	-0.40	-0.18	-0.25	-0.03
Q9CQU0	Thioredoxin domain-containing protein 12 OS=Mus musculus GN=Txndc12 PE=2 SV=1 - [TXD12_MOUSE]	63.53	1	7	7	124	-0.10	-0.06	-0.15	-0.04
P43024	Cytochrome c oxidase subunit 6A1, mitochondrial OS=Mus musculus GN=Cox6a1 PE=1 SV=2 - [CX6A1_MOUSE]	40.54	2	2	2	63	-0.07	-0.19	0.03	-0.17
P21661	Neuroendocrine convertase 2 OS=Mus musculus GN=Pcsk2 PE=2 SV=1 - [NEC2_MOUSE]	2.67	1	2	2	3	-0.06	0.00	0.72	0.54

Q62296-2	Isoform Short of Transcriptional enhancer factor TEF-3 OS=Mus musculus GN=Tead4 - [TEAD4_MOUSE]	4.69	2	1	1	4	-0.04	-0.07	-0.26	-0.29
Q3UPR7	Transmembrane protein 102 OS=Mus musculus GN=Tmem102 PE=1 SV=2 - [TM102_MOUSE]	8.25	1	2	2	3	0.20	0.27	0.20	0.26
P27600	Guanine nucleotide-binding protein subunit alpha-12 OS=Mus musculus GN=Gna12 PE=1 SV=3 - [GNA12_MOUSE]	13.72	4	2	4	30	-1.46	-0.96	-1.16	-0.66
G3UX44	Estradiol 17-beta-dehydrogenase 8 (Fragment) OS=Mus musculus GN=H2-Ke6 PE=2 SV=1 - [G3UX44_MOUSE]	27.35	3	4	4	27	-0.26	-0.32	-0.21	-0.26
G3UVV4	Hexokinase 1, isoform CRA_f OS=Mus musculus GN=Hk1 PE=3 SV=1 - [G3UVV4_MOUSE]	42.75	9	34	38	503	-0.28	-0.22	-0.27	-0.16
P09671	Superoxide dismutase [Mn], mitochondrial OS=Mus musculus GN=Sod2 PE=1 SV=3 - [SODM_MOUSE]	50.90	1	10	10	168	-0.06	0.02	-0.19	-0.04
Q61823	Programmed cell death protein 4 OS=Mus musculus GN=Pdcd4 PE=1 SV=1 - [PDCD4_MOUSE]	30.49	1	12	12	80	-0.02	0.04	-0.04	0.06
Q8BTV2-2	Isoform 2 of Cleavage and polyadenylation specificity factor subunit 7 OS=Mus musculus GN=Cpsf7 - [CPSF7_MOUSE]	10.39	3	4	5	14	0.10	0.12	-0.05	0.02
Q9WTL8-3	Isoform 3 of Aryl hydrocarbon receptor nuclear translocator-like protein 1 OS=Mus musculus GN=Arntl - [BMAL1_MOUSE]	2.12	4	1	1	2	-0.18	-0.33	-0.11	-0.26
P00920	Carbonic anhydrase 2 OS=Mus musculus GN=Ca2 PE=1 SV=4 - [CAH2_MOUSE]	75.00	1	17	17	1116	-0.24	-0.21	-0.26	-0.23
Q9CS42	Ribose-phosphate pyrophosphokinase 2 OS=Mus musculus GN=Prps2 PE=1 SV=4 - [PRPS2_MOUSE]	27.04	2	5	9	54	-0.15	-0.17	-0.22	-0.25
E9QP62	LIM and senescent cell antigen-like-containing domain protein 1 OS=Mus musculus GN=Lims1 PE=2 SV=1 - [E9QP62_MOUSE]	26.36	3	7	11	57	0.06	0.07	-0.01	0.05
P58871	182 kDa tankyrase-1-binding protein OS=Mus musculus GN=Tnks1bp1 PE=1 SV=2 - [TB182_MOUSE]	31.45	1	34	35	130	-0.14	-0.16	-0.01	-0.06
Q8K3K7	1-acyl-sn-glycerol-3-phosphate acyltransferase beta OS=Mus musculus GN=Agpat2 PE=1 SV=1 - [PLCB_MOUSE]	5.76	1	2	2	2	-0.46	-0.57	-0.20	-0.31

Q61908	Cx9C motif-containing protein 4 OS=Mus musculus GN=Cmc4 PE=2 SV=1 - [CMC4_MOUSE]	13.24	1	1	1	2	0.07	-0.01	0.30	0.21
Q922P9	Putative oxidoreductase GLYR1 OS=Mus musculus GN=Glyr1 PE=2 SV=1 - [GLYR1_MOUSE]	11.36	2	6	6	20	0.22	0.23	0.12	0.17
B2RUJ8	Rho GTPase activating protein 12 OS=Mus musculus GN=Arhgap12 PE=2 SV=1 - [B2RUJ8_MOUSE]	3.94	2	3	3	9	-0.06	-0.02	-0.05	-0.01
Q9JHW4	Selenocysteine-specific elongation factor OS=Mus musculus GN=Eefsec PE=2 SV=2 - [SELB_MOUSE]	9.43	1	6	6	15	-0.18	-0.26	-0.14	-0.22
P35550	rRNA 2'-O-methyltransferase fibrillarin OS=Mus musculus GN=Fbl PE=1 SV=2 - [FBRL_MOUSE]	37.00	2	11	11	109	0.18	0.26	0.11	0.21
Q6PDK2	Histone-lysine N-methyltransferase 2D OS=Mus musculus GN=Kmt2d PE=1 SV=2 - [KMT2D_MOUSE]	2.58	2	7	7	27	-0.44	0.02	-0.56	-0.07
Q8BFY6	Peflin OS=Mus musculus GN=Pef1 PE=2 SV=1 - [PEF1_MOUSE]	20.36	1	5	5	21	0.12	-0.03	0.05	0.10
P50295	Arylamine N-acetyltransferase 2 OS=Mus musculus GN=Nat2 PE=1 SV=1 - [ARY2_MOUSE]	18.28	3	4	4	13	0.23	0.04	0.35	-0.14
Q9JHI0	Matrix metalloproteinase-19 OS=Mus musculus GN=Mmp19 PE=2 SV=1 - [MMP19_MOUSE]	5.12	1	1	1	2	0.16	0.44	0.19	0.47
Q9DBS5	Kinesin light chain 4 OS=Mus musculus GN=Klc4 PE=1 SV=1 - [KLC4_MOUSE]	33.12	3	13	17	59	0.30	0.39	0.32	0.33
Q62318	Transcription intermediary factor 1-beta OS=Mus musculus GN=Trim28 PE=1 SV=3 - [TIF1B_MOUSE]	35.49	2	20	20	130	0.22	0.14	0.15	0.19
O89116	Vesicle transport through interaction with t-SNAREs homolog 1A OS=Mus musculus GN=Vti1a PE=1 SV=1 - [VTI1A_MOUSE]	7.37	1	2	2	2	0.18	0.16	0.28	0.25
Q8BMD8	Calcium-binding mitochondrial carrier protein SCaMC-1 OS=Mus musculus GN=Slc25a24 PE=2 SV=1 - [SCMC1_MOUSE]	33.26	1	18	18	109	0.04	0.00	0.00	-0.02
E9Q446	E3 ubiquitin-protein ligase RNF25 OS=Mus musculus GN=Rnf25 PE=2 SV=1 - [E9Q446_MOUSE]	4.40	4	2	2	4	0.01	-0.17	0.22	0.04

Q6P9Q4	FH1/FH2 domain-containing protein 1 OS=Mus musculus GN=Fhod1 PE=2 SV=3 - [FHOD1_MOUSE]	8.77	1	8	8	28	-0.09	-0.20	-0.08	-0.12
E2QRQ2	Rho guanine nucleotide exchange factor 38 OS=Mus musculus GN=Arhgef38 PE=2 SV=2 - [E2QRQ2_MOUSE]	3.51	4	2	3	4	0.45	0.32	0.47	0.33
Q8K1H1	Tudor domain-containing protein 7 OS=Mus musculus GN=Tdrd7 PE=1 SV=1 [TDRD7_MOUSE]	1.10	1	1	1	1	-0.40	-0.05	-0.33	0.02
Q6PGB6-3	Isoform 3 of N-alpha-acetyltransferase 50 OS=Mus musculus GN=Naa50 - [NAA50_MOUSE]	28.68	5	4	4	7	0.20	0.19	0.00	0.07
Q8VCC1	15-hydroxyprostaglandin dehydrogenase [NAD(+)] OS=Mus musculus GN=Hpgd PE=2 SV=1 - [PGDH_MOUSE]	18.96	1	4	4	9	-0.46	-1.13	-0.33	-0.92
Q8BMF4	Dihydrolipoyllysine-residue acetyltransferase component of pyruvate dehydrogenase complex, mitochondrial OS=Mus musculus GN=Dlat PE=1 SV=2 - [ODP2_MOUSE]	25.39	2	13	14	183	-0.05	0.04	-0.12	-0.02
Q8K366	Exosc10 protein OS=Mus musculus GN=Exosc10 PE=2 SV=1 - [Q8K366_MOUSE]	8.58	3	5	6	14	0.36	0.14	0.10	-0.01
Q62383	Transcription elongation factor SPT6 OS=Mus musculus GN=Supt6h PE=1 SV=2 - [SPT6H_MOUSE]	9.44	1	7	8	30	-0.14	0.42	0.14	0.54
P39053-3	Isoform 3 of Dynamin-1 OS=Mus musculus GN=Dnm1 - [DYN1_MOUSE]	11.75	10	2	12	55	-0.24	-0.49	-0.09	-0.34
E9QP57	Pecanex-like protein 2 OS=Mus musculus GN=Pcnxl2 PE=2 SV=1 - [E9QP57_MOUSE]	0.66	3	1	1	1	-0.54	-0.48	-0.40	-0.34
Q9D4Y8	Protein 4930538K18Rik OS=Mus musculus GN=4930538K18Rik PE=2 SV=1 - [Q9D4Y8_MOUSE]	9.45	1	1	1	2	0.19	0.01	0.13	-0.05
P62320	Small nuclear ribonucleoprotein Sm D3 OS=Mus musculus GN=Snrpd3 PE=1 SV=1 - [SMD3_MOUSE]	36.51	1	4	4	39	-0.31	-0.20	-0.07	-0.02
Q9D172	ES1 protein homolog, mitochondrial OS=Mus musculus GN=D10Jhu81e PE=1 SV=1 - [ES1_MOUSE]	40.98	1	9	9	116	-0.13	-0.19	-0.12	-0.16

A2AJY2	Collagen alpha-1(XV) chain OS=Mus musculus GN=Col15a1 PE=2 SV=1 - [A2AJY2_MOUSE]	6.47	4	7	7	57	-0.48	-0.42	-0.58	-0.54
Q8R4N0	Citrate lyase subunit beta-like protein, mitochondrial OS=Mus musculus GN=Clybl PE=2 SV=2 - [CLYBL_MOUSE]	38.76	1	11	11	80	-0.29	-0.23	-0.29	-0.20
P62835	Ras-related protein Rap-1A OS=Mus musculus GN=Rap1a PE=2 SV=1 - [RAP1A_MOUSE]	45.11	1	3	9	92	0.03	0.40	0.06	0.32
E9PYS0	Protein Vmn2r108 OS=Mus musculus GN=Vmn2r108 PE=3 SV=2 - [E9PYS0_MOUSE]	1.75	3	1	1	1	-0.36	0.48	-0.19	0.65
O08532-3	Isoform 2C of Voltage-dependent calcium channel subunit alpha-2/delta-1 OS=Mus musculus GN=Cacna2d1 - [CA2D1_MOUSE]	29.84	6	24	25	123	-0.70	-0.60	-0.73	-0.62
Q8C147	Dedicator of cytokinesis protein 8 OS=Mus musculus GN=Dock8 PE=1 SV=4 - [DOCK8_MOUSE]	4.33	1	5	6	7	-0.55	-0.73	-0.32	-0.50
Q9JLC8-2	Isoform 2 of Sacsin OS=Mus musculus GN=Sacs - [SACS_MOUSE]	0.55	4	2	2	2	-0.33	-0.15	0.39	0.57
Q5SUH7	Clathrin interactor 1 OS=Mus musculus GN=Clint1 PE=2 SV=1 - [Q5SUH7_MOUSE]	28.57	4	16	16	90	0.21	0.41	0.17	0.24
Q8K0Q5	Rho GTPase-activating protein 18 OS=Mus musculus GN=Arhgap18 PE=2 SV=1 - [RHG18_MOUSE]	6.79	2	3	3	3	0.07	0.08	0.17	0.19
Q7TMR0	Lysosomal Pro-X carboxypeptidase OS=Mus musculus GN=Prcp PE=2 SV=2 - [PCP_MOUSE]	20.57	2	7	7	27	-0.19	-0.12	-0.36	-0.31
Q80UW5	Serine/threonine-protein kinase MRCK gamma OS=Mus musculus GN=Cdc42bpg PE=2 SV=2 - [MRCKG_MOUSE]	6.83	1	7	9	24	-0.12	0.10	0.16	0.19
Q9CYI4-2	Isoform 2 of Putative RNA-binding protein Luc7-like 1 OS=Mus musculus GN=Luc7l - [LUC7L_MOUSE]	16.35	5	2	5	22	-0.10	-0.05	0.09	0.15
Q3TJ91	Lethal(2) giant larvae protein homolog 2 OS=Mus musculus GN=Llgl2 PE=2 SV=2 - [L2GL2_MOUSE]	17.33	8	11	11	27	-0.35	-0.24	-0.28	-0.24
B1AQ75	Keratin, type I cuticular Ha6 OS=Mus musculus GN=Krt36 PE=1 SV=1 [KRT36_MOUSE]	12.05	6	1	5	39	0.07	-0.03	0.41	0.31
Q78IK4	Apolipoprotein O-like OS=Mus musculus GN=Apool PE=2 SV=1 - [APOOL_MOUSE]	41.51	2	10	10	45	-0.18	-0.05	-0.31	-0.15

Q9JHZ2	Progressive ankylosis protein OS=Mus musculus GN=Ankh PE=2 SV=1 - [ANKH_MOUSE]	5.89	1	2	3	3	-0.58	-0.57	-0.47	-0.46
Q8CIM7	Cytochrome P450 2D26 OS=Mus musculus GN=Cyp2d26 PE=1 SV=1 - [CP2DQ_MOUSE]	3.00	1	1	1	7	-0.49	-0.57	-0.57	-0.51
Q8BW49	Tetratricopeptide repeat protein 12 OS=Mus musculus GN=Ttc12 PE=2 SV=1 - [TTC12_MOUSE]	0.99	1	1	1	2	-0.08	-0.07	-0.23	-0.23
Q9WVL0	Maleylacetoacetate isomerase OS=Mus musculus GN=Gstz1 PE=1 SV=1 - [MAAI_MOUSE]	33.80	1	7	7	32	-0.11	-0.12	-0.06	-0.13
Q08509	Epidermal growth factor receptor kinase substrate 8 OS=Mus musculus GN=Eps8 PE=1 SV=2 - [EPS8_MOUSE]	14.62	4	9	9	32	0.19	0.03	0.26	0.14
Q8CI75	DIS3-like exonuclease 2 OS=Mus musculus GN=Dis3l2 PE=1 SV=1 - [DI3L2_MOUSE]	18.16	2	10	10	15	-0.16	-0.19	0.01	-0.13
J3QMK2	MCG22959, isoform CRA_a OS=Mus musculus GN=Bbip1 PE=4 SV=1 - [J3QMK2_MOUSE]	14.93	1	1	1	4	-0.50	-0.41	-0.43	-0.34
Q9CZK6	Ankyrin repeat and SAM domain-containing protein 3 OS=Mus musculus GN=Anks3 PE=2 SV=2 - [ANKS3_MOUSE]	3.05	1	1	1	1	-0.06	0.02	0.06	0.15
G3UXL2	Ribose-phosphate pyrophosphokinase OS=Mus musculus GN=Prps1l3 PE=2 SV=1 - [G3UXL2_MOUSE]	28.62	4	6	10	56	-0.06	-0.18	0.12	-0.06
Q8BJR7	Centrosomal protein of 97 kDa OS=Mus musculus GN=Cep97 PE=2 SV=1 - [Q8BJR7_MOUSE]	14.57	8	1	2	7	0.35	0.20	0.19	0.03
O88622-2	Isoform 2 of Poly(ADP-ribose) glycohydrolase OS=Mus musculus GN=Parg - [PARG_MOUSE]	5.87	5	5	5	10	0.26	-0.23	0.17	-0.08
Q6PGF3	Mediator of RNA polymerase II transcription subunit 16 OS=Mus musculus GN=Med16 PE=2 SV=2 - [MED16_MOUSE]	8.33	3	3	4	5	0.06	0.23	-0.25	-0.08
Q9CQC6	Basic leucine zipper and W2 domain-containing protein 1 OS=Mus musculus GN=Bzw1 PE=1 SV=1 - [BZW1_MOUSE]	26.49	1	13	14	85	0.43	0.35	0.37	0.35
O55042-2	Isoform 2 of Alpha-synuclein OS=Mus musculus GN=Snca - [SYUA_MOUSE]	23.97	2	2	2	4	-0.33	-0.19	-0.26	-0.12

Q9DCH2	Ribonuclease P protein subunit p20 OS=Mus musculus GN=Pop7 PE=2 SV=1 - [POP7_MOUSE]	10.71	1	1	1	1	-0.20	0.45	-0.13	0.52
P00493	Hypoxanthine-guanine phosphoribosyltransferase OS=Mus musculus GN=Hprt1 PE=1 SV=3 [HPRT_MOUSE]	43.12	1	8	8	102	0.21	0.27	0.20	0.25
A2AUR3	Phosphotriesterase-related protein (Fragment) OS=Mus musculus GN=Pter PE=2 SV=1 - [A2AUR3_MOUSE]	19.34	3	3	3	8	0.23	0.12	0.17	0.02
Q8BGQ7	Alanine--tRNA ligase, cytoplasmic OS=Mus musculus GN=Aars PE=1 SV=1 - [SYAC_MOUSE]	38.84	1	29	29	128	0.25	0.24	0.32	0.34
Q8VHB5	Carbonic anhydrase 9 OS=Mus musculus GN=Ca9 PE=2 SV=2 - [CAH9_MOUSE]	6.41	3	2	2	4	-0.21	-0.39	-0.03	-0.22
Q8K1D3	Protein 9130221H12Rik OS=Mus musculus GN=9130221H12Rik PE=2 SV=1 - [Q8K1D3_MOUSE]	5.84	1	1	1	4	-0.54	-0.33	-0.12	0.09
Q91XD6	Vacuolar protein-sorting associated protein 36 OS=Mus musculus GN=Vps36 PE=1 SV=1 - [VPS36_MOUSE]	24.87	1	9	9	24	0.14	0.03	0.03	0.12
Q3TKR3	NACHT, LRR and PYD domains-containing protein 4C OS=Mus musculus GN=Nlrp4c PE=2 SV=1 - [NAL4C_MOUSE]	4.07	1	1	3	5	0.41	0.69	0.21	0.49
F8VQC7	Kinectin OS=Mus musculus GN=Ktn1 PE=2 SV=1 - [F8VQC7_MOUSE]	27.73	1	3	31	116	-0.22	-0.58	-0.06	-0.51
Q8K1J6	CCA tRNA nucleotidyltransferase 1, mitochondrial OS=Mus musculus GN=Trnt1 PE=2 SV=1 - [TRNT1_MOUSE]	20.28	2	8	8	36	0.34	0.41	0.16	0.31
Q61292	Laminin subunit beta-2 OS=Mus musculus GN=Lamb2 PE=2 SV=2 - [LAMB2_MOUSE]	26.46	1	42	43	267	-0.68	-0.69	-0.69	-0.70
Q9DC71	28S ribosomal protein S15, mitochondrial OS=Mus musculus GN=Mrps15 PE=2 SV=2 - [RT15_MOUSE]	10.08	1	2	2	3	0.13	0.15	0.27	0.28
P26883	Peptidyl-prolyl cis-trans isomerase FKBP1A OS=Mus musculus GN=Fkbp1a PE=1 SV=2 - [FKB1A_MOUSE]	30.56	2	4	4	81	-0.16	-0.03	-0.18	-0.03
P70699	Lysosomal alpha-glucosidase OS=Mus musculus GN=Gaa PE=1 SV=2 - [LYAG_MOUSE]	14.69	5	10	10	45	-0.12	-0.17	-0.02	-0.10
Q9QYY8	Spastin OS=Mus musculus GN=Spast PE=2 SV=3 - [SPAST_MOUSE]	12.38	1	5	5	8	0.13	-0.36	0.20	0.00

Q3V1V3	ESF1 homolog OS=Mus musculus GN=Esf1 PE=1 SV=1 - [ESF1_MOUSE]	2.96	1	2	2	2	0.12	0.10	0.33	0.30
Q3ZAS0	Sodium/hydrogen exchanger OS=Mus musculus GN=Slc9a2 PE=2 SV=1 - [Q3ZAS0_MOUSE]	1.35	1	1	1	1	-0.14	-0.32	-0.27	-0.45
Q8C196	Carbamoyl-phosphate synthase [ammonia], mitochondrial OS=Mus musculus GN=Cps1 PE=1 SV=2 - [CPSM_MOUSE]	5.67	1	6	8	13	-0.10	-0.13	0.07	0.04
D3Z598	Latent-transforming growth factor beta-binding protein 4 OS=Mus musculus GN=Ltbp4 PE=2 SV=1 [D3Z598_MOUSE]	15.66	7	20	20	69	-0.04	-0.07	-0.13	-0.19
Q80YR5	Scaffold attachment factor B2 OS=Mus musculus GN=Safb2 PE=1 SV=2 - [SAFB2_MOUSE]	11.20	6	4	10	49	0.14	-0.01	0.31	0.16
Q8BR63	Protein FAM177A1 OS=Mus musculus GN=Fam177a1 PE=2 SV=1 - [F177A_MOUSE]	33.33	1	6	6	30	0.20	0.22	0.12	0.27
E0CXW4	Histone-lysine N-methyltransferase SUV420H2 OS=Mus musculus GN=Suv420h2 PE=2 SV=1 - [E0CXW4_MOUSE]	14.48	3	1	1	1	0.09	0.12	-0.07	-0.04
Q9Z2W0	Aspartyl aminopeptidase OS=Mus musculus GN=Dnpep PE=2 SV=2 - [DNPEP_MOUSE]	40.17	1	13	13	117	-0.26	-0.42	-0.24	-0.31
Q11011	Puromycin-sensitive aminopeptidase OS=Mus musculus GN=Npepps PE=1 SV=2 - [PSA_MOUSE]	43.91	7	33	33	187	0.01	0.06	0.03	0.13
Q8BG94	COMM domain-containing protein 7 OS=Mus musculus GN=Commd7 PE=2 SV=1 - [COMD7_MOUSE]	23.00	2	4	4	6	-0.96	0.08	-0.93	0.11
E9QP99	Golgin subfamily A member 3 OS=Mus musculus GN=Golga3 PE=2 SV=1 - [E9QP99_MOUSE]	15.20	3	18	18	48	0.06	-0.01	0.13	0.08
Q62468	Villin-1 OS=Mus musculus GN=Vil1 PE=1 SV=3 - [VILI_MOUSE]	57.44	2	42	44	682	0.03	0.06	0.05	0.07
F6WNY1	Protein Tex16 (Fragment) OS=Mus musculus GN=Tex16 PE=2 SV=1 - [F6WNY1_MOUSE]	2.35	2	2	2	4	0.72	0.42	1.11	0.81
Q810A7	ATP-dependent RNA helicase DDX42 OS=Mus musculus GN=Ddx42 PE=1 SV=3 - [DDX42_MOUSE]	18.19	2	13	14	48	0.21	0.12	0.20	0.11
G5E8A7	Protein Smaug homolog 2 OS=Mus musculus GN=Samd4b PE=4 SV=1 - [G5E8A7_MOUSE]	6.40	2	2	2	4	0.18	0.14	0.31	0.56

Q99N92	39S ribosomal protein L27, mitochondrial OS=Mus musculus GN=Mrpl27 PE=2 SV=1 - [RM27_MOUSE]	29.73	1	4	4	6	-0.06	0.03	-0.02	0.03
Q149C3	Leucine-rich repeat and immunoglobulin-like domain containing-NOGO receptor-interacting protein 4 OS=Mus musculus GN=Lingo4 PE=2 SV=2 - [LIGO4_MOUSE]	1.01	1	1	1	1	-0.02	-0.08	0.03	-0.03
Q91Z49-2	Isoform 2 of UAP56-interacting factor OS=Mus musculus GN=Fyttd1 - [UIF_MOUSE]	4.59	2	1	1	2	-0.38	-0.11	0.12	0.38
Q61469	Lipid phosphate phosphohydrolase 1 OS=Mus musculus GN=Ppap2a PE=1 SV=1 - [LPP1_MOUSE]	8.83	2	1	1	4	-0.83	-0.33	-0.41	0.10
P16125	L-lactate dehydrogenase B chain OS=Mus musculus GN=Ldhb PE=1 SV=2 - [LDHB_MOUSE]	43.11	2	13	15	181	0.17	0.24	0.10	0.19
Q99P72-3	Isoform 2 of Reticulon-4 OS=Mus musculus GN=Rtn4 - [RTN4_MOUSE]	17.59	2	1	13	81	0.22	0.35	0.24	0.36
Q9R078	5'-AMP-activated protein kinase subunit beta-1 OS=Mus musculus GN=Prkab1 PE=1 SV=2 - [AAKB1_MOUSE]	17.41	1	3	3	11	0.31	0.28	0.12	0.10
Q9DBM2	Peroxisomal bifunctional enzyme OS=Mus musculus GN=Ehhadh PE=1 SV=4 - [ECHP_MOUSE]	29.94	1	18	18	73	-0.28	-0.35	-0.34	-0.34
Q9JMA2	Queuine tRNA-ribosyltransferase OS=Mus musculus GN=Qtrt1 PE=1 SV=2 - [TGT_MOUSE]	9.18	1	3	3	6	0.09	-0.12	0.01	-0.02
Q3UMR5	Calcium uniporter protein, mitochondrial OS=Mus musculus GN=Mcu PE=1 SV=2 - [MCU_MOUSE]	20.00	2	6	6	38	-0.15	-0.23	-0.07	-0.28
E9Q705	BolA-like protein 3 OS=Mus musculus GN=Bola3 PE=2 SV=1 [E9Q705_MOUSE]	21.05	3	2	2	6	0.13	0.18	-0.02	0.02
F6WZB3	Kin of IRRE-like protein 2 (Fragment) OS=Mus musculus GN=Kirrel2 PE=4 SV=1 - [F6WZB3_MOUSE]	8.00	3	1	1	1	0.17	0.26	0.22	0.30
Q9ER72	Cysteine--tRNA ligase, cytoplasmic OS=Mus musculus GN=Cars PE=1 SV=2 - [SYCC_MOUSE]	29.24	2	24	24	87	0.22	0.15	0.20	0.14
Q5XG73-2	Isoform 2 of Acyl-CoA-binding domain-containing protein 5 OS=Mus musculus GN=Acbd5 - [ACBD5_MOUSE]	5.08	4	3	3	8	0.08	-0.09	-0.05	-0.16

A2AVJ7	Ribosome-binding protein 1 OS=Mus musculus GN=Rrbp1 PE=2 SV=1 - [A2AVJ7_MOUSE]	41.80	20	50	60	1007	0.14	0.19	0.09	0.13
Q8BWW9-3	Isoform 3 of Serine/threonine-protein kinase N2 OS=Mus musculus GN=Pkn2 - [PKN2_MOUSE]	9.91	5	4	6	11	0.16	-0.31	0.28	-0.19
Q61699-2	Isoform HSP105-beta of Heat shock protein 105 kDa OS=Mus musculus GN=Hsph1 - [HS105_MOUSE]	41.89	5	26	29	190	0.04	0.09	-0.02	0.09
Q8BVL9	Janus kinase and microtubule-interacting protein 1 OS=Mus musculus GN=Jakmip1 PE=1 SV=2 - [JKIP1_MOUSE]	12.94	6	6	8	16	-0.23	-0.39	-0.15	-0.32
O35218	Cleavage and polyadenylation specificity factor subunit 2 OS=Mus musculus GN=Cpsf2 PE=1 SV=1 - [CPSF2_MOUSE]	11.38	1	7	7	10	0.24	0.33	0.27	0.35
Q3UMW7	MAP kinase-activated protein kinase 3 OS=Mus musculus GN=Mapkapk3 PE=1 SV=2 - [MAPK3_MOUSE]	12.50	6	2	5	10	-0.06	-0.03	-0.04	-0.01
Q99MN9	Propionyl-CoA carboxylase beta chain, mitochondrial OS=Mus musculus GN=Pccb PE=1 SV=2 - [PCCB_MOUSE]	48.24	3	19	19	205	-0.24	-0.10	-0.31	-0.23
F6WKE4	Epsilon-sarcoglycan (Fragment) OS=Mus musculus GN=Sgce PE=2 SV=1 - [F6WKE4_MOUSE]	17.52	8	5	5	15	-0.37	-0.58	-0.70	-0.34
F6Q269	JmjC domain-containing protein 8 (Fragment) OS=Mus musculus GN=Jmjd8 PE=4 SV=1 - [F6Q269_MOUSE]	14.97	3	2	2	2	0.10	-0.26	0.19	-0.17
Q05BC8	Nbr1 protein OS=Mus musculus GN=Nbr1 PE=2 SV=1 - [Q05BC8_MOUSE]	1.91	7	2	2	6	-0.40	-0.09	-0.42	-0.11
P28078	Class II histocompatibility antigen, M alpha chain OS=Mus musculus GN=H2-DMa PE=1 SV=1 - [DMA_MOUSE]	3.45	2	1	1	4	0.11	-0.15	0.07	-0.29
Q8JZU0	Nucleoside diphosphate-linked moiety X motif 13 OS=Mus musculus GN=Nudt13 PE=2 SV=2 - [NUD13_MOUSE]	2.56	1	1	1	2	0.14	0.42	0.04	0.31
Q3UJB9-2	Isoform 2 of Enhancer of mRNA-decapping protein 4 OS=Mus musculus GN=Edc4 - [EDC4_MOUSE]	11.94	5	11	11	39	0.23	0.08	0.09	0.00
P28651	Carbonic anhydrase-related protein OS=Mus musculus GN=Ca8 PE=1 SV=5 - [CAH8_MOUSE]	16.49	1	3	3	28	-0.84	-0.62	-0.74	-0.74

Q3UYC0	Protein phosphatase 1H OS=Mus musculus GN=Ppm1h PE=1 SV=1 - [PPM1H_MOUSE]	15.98	3	5	5	10	-0.48	-0.31	-0.35	-0.21
Q9CR56	NF-kappa-B inhibitor-interacting Ras-like protein 2 OS=Mus musculus GN=Nkiras2 PE=2 SV=1 - [KBRS2_MOUSE]	10.47	2	2	2	4	-0.17	0.02	-0.15	0.03
P80315	T-complex protein 1 subunit delta OS=Mus musculus GN=Cct4 PE=1 SV=3 - [TCPD_MOUSE]	44.90	5	21	22	237	0.03	0.09	0.10	0.14
Q9ESX5	H/ACA ribonucleoprotein complex subunit 4 OS=Mus musculus GN=Dkc1 PE=1 SV=4 - [DKC1_MOUSE]	11.98	5	6	6	19	0.14	0.15	0.09	0.00
E9QA93	Arachidonate 5-lipoxygenase OS=Mus musculus GN=Alox5 PE=2 SV=1 - [E9QA93_MOUSE]	4.86	3	3	3	4	-0.03	-0.14	0.08	-0.03
B1AVH7	TBC1 domain family member 2A OS=Mus musculus GN=Tbc1d2 PE=2 SV=1 - [TBD2A_MOUSE]	6.62	2	4	4	9	0.30	0.38	0.41	0.26
Q924W5	Structural maintenance of chromosomes protein 6 OS=Mus musculus GN=Smc6 PE=2 SV=1 [SMC6_MOUSE]	2.83	2	3	3	4	-0.16	-0.04	-0.19	-0.07
P43276	Histone H1.5 OS=Mus musculus GN=Hist1h1b PE=1 SV=2 - [H15_MOUSE]	40.81	1	9	14	228	0.15	0.30	0.00	0.21
Q91WT8-2	Isoform 2 of RNA-binding protein 47 OS=Mus musculus GN=Rbm47 - [RBM47_MOUSE]	31.03	4	10	11	38	0.25	0.13	0.16	0.15
Q6PB93-2	Isoform 2 of Polypeptide N-acetylgalactosaminyltransferase 2 OS=Mus musculus GN=Galnt2 - [GALT2_MOUSE]	13.43	2	6	7	14	-0.06	-0.08	0.12	-0.06
Q8BTS4	Nuclear pore complex protein Nup54 OS=Mus musculus GN=Nup54 PE=1 SV=1 - [NUP54_MOUSE]	24.90	1	6	6	17	0.45	0.24	0.32	0.20
Q9JHK4	Geranylgeranyl transferase type-2 subunit alpha OS=Mus musculus GN=Rabggta PE=1 SV=1 - [PGTA_MOUSE]	11.11	2	7	7	20	0.40	0.45	0.26	0.34
Q8K157	Aldose 1-epimerase OS=Mus musculus GN=Galm PE=2 SV=1 - [GALM_MOUSE]	19.30	2	7	7	32	-0.47	-0.54	-0.54	-0.47
Q8K0P3	TLD domain-containing protein 1 OS=Mus musculus GN=Tldc1 PE=2 SV=1 - [TLDC1_MOUSE]	9.01	1	3	3	5	-0.20	-0.36	-0.24	-0.25

Q3TCX3-2	Isoform 2 of UPF0469 protein KIAA0907 OS=Mus musculus GN=Kiaa0907 - [K0907_MOUSE]	7.60	3	3	3	6	0.22	0.76	0.04	0.57
E9Q066	La-related protein 4 OS=Mus musculus GN=Larp4 PE=2 SV=1 [E9Q066_MOUSE]	9.05	3	6	6	11	0.23	0.15	0.32	0.43
P28661-5	Isoform 5 of Septin-4 OS=Mus musculus GN=Sept4 - [SEPT4_MOUSE]	15.02	5	3	4	10	-0.50	-0.73	-0.23	-0.45
Q9Z2Y8	Proline synthase co-transcribed bacterial homolog protein OS=Mus musculus GN=Prosc PE=1 SV=1 - [PROSC_MOUSE]	51.09	2	10	11	47	-0.27	-0.14	-0.18	-0.21
Q9D2E2-2	Isoform 2 of Target of EGR1 protein 1 OS=Mus musculus GN=Toe1 - [TOE1_MOUSE]	2.13	2	1	1	1	0.22	0.37	0.41	0.56
H3BKM2	Nuclear-interacting partner of ALK OS=Mus musculus GN=Zc3hc1 PE=2 SV=1 - [H3BKM2_MOUSE]	4.58	3	1	1	3	0.27	0.18	0.52	0.43
O35129	Prohibitin-2 OS=Mus musculus GN=Phb2 PE=1 SV=1 - [PHB2_MOUSE]	60.54	3	17	17	151	-0.20	-0.24	-0.14	-0.17
D3YWT1	MCG11326, isoform CRA_b OS=Mus musculus GN=Hnrnph3 PE=4 SV=1 - [D3YWT1_MOUSE]	31.72	3	6	8	23	-0.12	-0.19	-0.10	-0.20
Q3U3J1	2-oxoisovalerate dehydrogenase subunit alpha, mitochondrial OS=Mus musculus GN=Bckdha PE=2 SV=1 - [Q3U3J1_MOUSE]	14.80	2	6	6	14	-0.26	-0.51	-0.23	-0.55
Q3UIR3	E3 ubiquitin-protein ligase DTX3L OS=Mus musculus GN=Dtx3l PE=2 SV=1 - [DTX3L_MOUSE]	14.84	2	9	9	26	0.22	-0.12	0.17	-0.08
P16332	Methylmalonyl-CoA mutase, mitochondrial OS=Mus musculus GN=Mut PE=2 SV=2 - [MUTA_MOUSE]	41.04	1	25	25	116	-0.07	0.01	-0.10	-0.03
Q80X90	Filamin-B OS=Mus musculus GN=Flnb PE=1 SV=3 - [FLNB_MOUSE]	62.80	1	108	120	2064	0.12	0.18	0.09	0.13
Q9ES52-3	Isoform 3 of Phosphatidylinositol 3,4,5-trisphosphate 5-phosphatase 1 OS=Mus musculus GN=Inpp5d - [SHIP1_MOUSE]	7.52	8	4	7	10	0.51	0.42	0.39	0.29
Q8R3C0	Mini-chromosome maintenance complex-binding protein OS=Mus musculus GN=Mcmbp PE=2 SV=1 - [MCMBP_MOUSE]	6.85	2	4	4	6	0.01	0.29	-0.07	0.21
Q61107	Guanylate-binding protein 4 OS=Mus musculus GN=Gbp4 PE=1 SV=1 - [GBP4_MOUSE]	13.06	8	4	8	22	0.50	0.53	0.46	0.51

P30115	Glutathione S-transferase A3 OS=Mus musculus GN=Gsta3 PE=1 SV=2 - [GSTA3_MOUSE]	34.84	1	4	8	38	-0.24	-0.26	-0.04	-0.08
A2AUK7	Band 4.1-like protein 1 OS=Mus musculus GN=Epb4.1l1 PE=2 SV=1 - [A2AUK7_MOUSE]	22.47	7	7	15	35	-0.48	-0.49	-0.39	-0.41
E9QKV6	Unconventional myosin-IXb OS=Mus musculus GN=Myo9b PE=2 SV=1 - [E9QKV6_MOUSE]	1.33	6	3	3	3	0.37	0.18	0.35	0.15
B2RY53	EG620155 protein OS=Mus musculus GN=Gm6133 PE=2 SV=1 - [B2RY53_MOUSE]	12.63	1	1	2	22	-0.26	-0.35	-0.29	-0.38
Q8C753-3	Isoform 3 of Uncharacterized protein KIAA0556 OS=Mus musculus GN=Kiaa0556 - [K0556_MOUSE]	1.22	3	1	1	1	-0.15	-0.66	-0.04	-0.56
F8VPM7	ELKS/Rab6-interacting/CAST family member 1 OS=Mus musculus GN=Erc1 PE=2 SV=1 - [F8VPM7_MOUSE]	15.80	4	10	11	35	-0.40	-0.31	-0.44	-0.19
D3Z7Q2	Uncharacterized protein OS=Mus musculus GN=Smim20 PE=2 SV=1 - [D3Z7Q2_MOUSE]	20.29	1	1	1	2	0.39	0.16	0.46	0.23
Q8VDZ4	Palmitoyltransferase ZDHHC5 OS=Mus musculus GN=Zdhhc5 PE=1 SV=1 - [ZDHC5_MOUSE]	8.25	2	4	4	8	-0.01	0.16	0.21	0.13
Q8C2Q3	RNA-binding protein 14 OS=Mus musculus GN=Rbm14 PE=1 SV=1 - [RBM14_MOUSE]	26.01	5	14	15	71	-0.06	-0.12	0.01	-0.08
Q8VEK3	Heterogeneous nuclear ribonucleoprotein U OS=Mus musculus GN=Hnrnpu PE=1 SV=1 - [HNRPU_MOUSE]	36.50	2	29	29	489	0.11	0.18	0.12	0.16
P81122	Insulin receptor substrate 2 OS=Mus musculus GN=Irs2 PE=1 SV=2 - [IRS2_MOUSE]	2.35	1	1	1	1	0.06	-0.20	0.10	-0.16
Q80US4	Actin-related protein 5 OS=Mus musculus GN=Actr5 PE=2 SV=3 [ARP5_MOUSE]	6.61	1	2	3	5	0.13	0.25	-0.14	-0.02
P18608	Non-histone chromosomal protein HMG-14 OS=Mus musculus GN=Hmgn1 PE=1 SV=2 - [HMGN1_MOUSE]	18.75	2	3	3	21	0.44	0.63	0.31	0.59
P63325	40S ribosomal protein S10 OS=Mus musculus GN=Rps10 PE=1 SV=1 - [RS10_MOUSE]	50.30	3	10	11	125	0.17	0.19	0.11	0.08
H3BJU7	Rho guanine nucleotide exchange factor 2 OS=Mus musculus GN=Arhgef2 PE=2 SV=1 - [H3BJU7_MOUSE]	8.68	17	7	8	17	-0.05	-0.13	0.16	-0.24

P97873	Lysyl oxidase homolog 1 OS=Mus musculus GN=Loxl1 PE=2 SV=3 [LOXL1_MOUSE]	2.14	1	1	1	1	0.43	-0.27	0.38	-0.32
O35343	Importin subunit alpha-3 OS=Mus musculus GN=Kpna4 PE=2 SV=1 - [IMA3_MOUSE]	9.02	2	1	4	14	-0.30	-0.17	0.65	0.77
Q8C1B7-3	Isoform 3 of Septin-11 OS=Mus musculus GN=Sept11 - [SEP11_MOUSE]	38.82	3	6	13	78	0.00	-0.05	0.05	0.03
G5E897	KDEL (Lys-Asp-Glu-Leu) containing 2, isoform CRA_b OS=Mus musculus GN=Kdelc2 PE=4 SV=1 - [G5E897_MOUSE]	14.71	1	7	7	12	-0.13	-0.28	0.01	0.07
Q8C3I8	Protein FAM203A OS=Mus musculus GN=Fam203a PE=2 SV=1 - [F203A_MOUSE]	5.34	1	2	2	3	0.38	0.22	0.35	0.18
Q9WU66	Secreted frizzled-related protein 5 OS=Mus musculus GN=Sfrp5 PE=2 SV=1 - [SFRP5_MOUSE]	1.91	1	1	1	1	-0.05	0.24	-0.10	0.19
P10639	Thioredoxin OS=Mus musculus GN=Txn PE=1 SV=3 - [THIO_MOUSE]	36.19	1	6	6	238	0.23	0.35	0.19	0.36
Q91WT7	3-alpha-hydroxysteroid dehydrogenase type 1 OS=Mus musculus GN=Akr1c14 PE=2 SV=1 - [Q91WT7_MOUSE]	44.27	2	10	13	83	-0.22	-0.20	-0.07	-0.02
G5E846	Peripherin OS=Mus musculus GN=Prph PE=3 SV=1 - [G5E846_MOUSE]	49.05	5	20	24	420	-0.64	-0.62	-0.65	-0.62
Q5DTZ0	Protein NYNRIN OS=Mus musculus GN=Nynrin PE=1 SV=2 - [NYNRI_MOUSE]	2.45	1	2	2	2	0.31	0.18	0.36	0.23
Q0VBM2	Protein FAM83B OS=Mus musculus GN=Fam83b PE=1 SV=1 - [FA83B_MOUSE]	4.94	1	3	3	3	-0.06	-0.14	-0.06	-0.15
Q8BGY3	Leucine zipper protein 2 OS=Mus musculus GN=Luzp2 PE=2 SV=1 - [LUZP2_MOUSE]	3.48	1	1	2	25	0.08	0.16	0.07	0.16
Q9ESN6	Tripartite motif-containing protein 2 OS=Mus musculus GN=Trim2 PE=1 SV=1 [TRIM2_MOUSE]	10.75	5	6	6	24	-0.15	-0.11	-0.27	-0.19
Q9D0E1-2	Isoform 2 of Heterogeneous nuclear ribonucleoprotein M OS=Mus musculus GN=Hnrnpm - [HNRPM_MOUSE]	46.96	6	27	28	210	-0.03	-0.07	0.03	0.03
Q3UJU9	Regulator of microtubule dynamics protein 3 OS=Mus musculus GN=Rmdn3 PE=1 SV=2 - [RMD3_MOUSE]	25.53	1	9	9	31	-0.15	-0.10	-0.10	-0.20

Q9EQH3	Vacuolar protein sorting associated protein 35 OS=Mus musculus GN=Vps35 PE=1 SV=1 - [VPS35_MOUSE]	26.63	1	20	21	171	-0.07	-0.14	-0.04	-0.11
Q3V0K9	Plastin-1 OS=Mus musculus GN=Pls1 PE=2 SV=1 - [PLSI_MOUSE]	49.37	2	23	26	380	0.21	0.24	0.15	0.18
P46935	E3 ubiquitin-protein ligase NEDD4 OS=Mus musculus GN=Nedd4 PE=1 SV=3 - [NEDD4_MOUSE]	34.05	1	22	24	145	0.05	-0.09	0.02	-0.06
D3Z1R2	Methenyltetrahydrofolate synthase domain-containing protein (Fragment) OS=Mus musculus GN=Mthfsd PE=2 SV=1 - [D3Z1R2_MOUSE]	3.58	3	1	1	1	0.31	0.32	0.02	0.03
Q8CD94	Protein lin-52 homolog OS=Mus musculus GN=Lin52 PE=2 SV=1 [LIN52_MOUSE]	12.93	1	1	1	2	-0.35	-0.78	-0.03	-0.45
Q8CG76	Aflatoxin B1 aldehyde reductase member 2 OS=Mus musculus GN=Akr7a2 PE=1 SV=3 - [ARK72_MOUSE]	31.88	1	11	11	57	-0.26	-0.20	-0.24	-0.21
D3Z0F5	COP9 signalosome complex subunit 6 OS=Mus musculus GN=Cops6 PE=2 SV=1 - [D3Z0F5_MOUSE]	17.17	3	5	5	11	-0.08	-1.09	-0.32	-1.39
Q80ZV0	Ribonuclease H2 subunit B OS=Mus musculus GN=Rnaseh2b PE=1 SV=2 - [RNH2B_MOUSE]	7.79	1	2	2	4	0.70	0.84	0.50	0.64
P63017	Heat shock cognate 71 kDa protein OS=Mus musculus GN=Hspa8 PE=1 SV=1 - [HSP7C_MOUSE]	70.74	4	28	45	2472	0.02	0.07	0.00	0.07
Q8K4Z5	Splicing factor 3A subunit 1 OS=Mus musculus GN=Sf3a1 PE=1 SV=1 - [SF3A1_MOUSE]	32.74	1	21	22	80	0.19	0.24	0.20	0.25
Q3KNY0	Immunoglobulin-like and fibronectin type III domain-containing protein 1 OS=Mus musculus GN=Igfn1 PE=1 SV=3 - [IGFN1_MOUSE]	1.54	4	3	3	4	0.39	0.16	0.40	0.16
D3YYP5	Non-syndromic hearing impairment protein 5 homolog OS=Mus musculus GN=Dfna5 PE=2 SV=2 - [D3YYP5_MOUSE]	4.01	3	1	1	1	0.05	0.36	-0.10	0.21
Q9CRB2	H/ACA ribonucleoprotein complex subunit 2 OS=Mus musculus GN=Nhp2 PE=2 SV=1 - [NHP2_MOUSE]	6.54	1	1	1	6	0.15	0.41	0.34	0.49
Q9CZG9	PDZ domain-containing protein 11 OS=Mus musculus GN=Pdzd11 PE=1 SV=1 - [PDZ11_MOUSE]	51.43	1	3	4	53	-0.08	-0.44	0.10	-0.34

Q91WK2	Eukaryotic translation initiation factor 3 subunit H OS=Mus musculus GN=Eif3h PE=1 SV=1 - [EIF3H_MOUSE]	34.38	1	10	10	36	0.22	0.36	0.23	0.36
Q9JIK5	Nucleolar RNA helicase 2 OS=Mus musculus GN=Ddx21 PE=1 SV=3 - [DDX21_MOUSE]	32.20	2	20	21	81	0.32	0.44	0.21	0.39
Q63844	Mitogen-activated protein kinase 3 OS=Mus musculus GN=Mapk3 PE=1 SV=5 - [MK03_MOUSE]	49.47	14	12	18	342	-0.04	-0.06	-0.02	-0.02
Q3UKW2	Calmodulin OS=Mus musculus GN=Calm1 PE=2 SV=1 - [Q3UKW2_MOUSE]	65.48	4	8	11	680	-0.33	-0.10	-0.35	-0.13
Q91ZT5-2	Isoform 2 of FYVE, RhoGEF and PH domain-containing protein 4 OS=Mus musculus GN=Fgd4 - [FGD4_MOUSE]	12.60	6	5	5	8	0.28	0.57	0.26	0.74
Q9CWG9	Biogenesis of lysosome-related organelles complex 1 subunit 2 OS=Mus musculus GN=Bloc1s2 PE=1 SV=1 - [BL1S2_MOUSE]	16.78	1	2	2	3	-0.11	-0.15	-0.24	-0.28
Q6IQY5-2	Isoform 2 of Centrosomal protein of 70 kDa OS=Mus musculus GN=Cep70 - [CEP70_MOUSE]	3.29	2	1	2	2	0.31	-0.25	0.26	-0.31
P80318	T-complex protein 1 subunit gamma OS=Mus musculus GN=Cct3 PE=1 SV=1 - [TCPG_MOUSE]	57.06	6	29	29	278	0.17	0.19	0.16	0.20
E9PZM7	Protein Scaf11 OS=Mus musculus GN=Scaf11 PE=2 SV=1 - [E9PZM7_MOUSE]	3.91	1	4	4	5	0.28	-0.04	0.26	-0.07
Q61559	IgG receptor FcRn large subunit p51 OS=Mus musculus GN=Fcgrt PE=1 SV=1 - [FCGRN_MOUSE]	16.44	1	4	4	19	-0.21	0.09	-0.13	-0.15
D3Z3A0	MCG126099, isoform CRA_b OS=Mus musculus GN=Ppp1r2 PE=4 SV=1 - [D3Z3A0_MOUSE]	47.18	2	4	4	40	-0.10	0.15	0.06	0.14
P28271	Cytoplasmic aconitate hydratase OS=Mus musculus GN=Aco1 PE=1 SV=3 - [ACOC_MOUSE]	43.98	1	33	34	333	-0.09	-0.12	-0.09	-0.14
E9QAJ9	Rho GTPase-activating protein 17 OS=Mus musculus GN=Arhgap17 PE=2 SV=1 - [E9QAJ9_MOUSE]	13.33	8	7	7	34	-0.19	-0.07	-0.08	0.16
Q32ME9	Desmocollin 1 OS=Mus musculus GN=Dsc1 PE=2 SV=1 - [Q32ME9_MOUSE]	8.05	3	3	3	4	0.15	-1.71	0.17	-1.69
H7BWY2	AP-3 complex subunit mu-1 OS=Mus musculus GN=Ap3m1 PE=2 SV=1 - [H7BWY2_MOUSE]	28.30	2	6	6	27	0.26	0.35	0.10	0.26

E9Q3Z5	Supervillin OS=Mus musculus GN=Svil PE=2 SV=1 - [E9Q3Z5_MOUSE]	37.21	3	2	59	234	0.11	0.36	0.19	0.44
Q9D4C5	ELL-associated factor 1 OS=Mus musculus GN=Eaf1 PE=1 SV=2 - [EAF1_MOUSE]	8.21	1	1	1	4	-0.04	-0.28	0.04	-0.20
Q3V009-2	Isoform 2 of Transmembrane emp24 domain-containing protein 1 OS=Mus musculus GN=Tmed1 - [TMED1_MOUSE]	6.34	2	1	1	1	0.71	0.33	-0.09	-0.47
Q61635	GTP-binding protein OS=Mus musculus GN=Ifi47 PE=2 SV=1 - [Q61635_MOUSE]	35.24	1	12	13	64	0.22	0.49	0.10	0.42
P34152-3	Isoform 3 of Focal adhesion kinase 1 OS=Mus musculus GN=Ptk2 - [FAK1_MOUSE]	9.32	9	6	7	23	0.15	0.12	0.10	-0.04
E9PUF4	RNA-binding protein 26 OS=Mus musculus GN=Rbm26 PE=2 SV=1 - [E9PUF4_MOUSE]	5.80	8	4	6	12	-0.14	-0.16	-0.11	-0.14
Q61735	Leukocyte surface antigen CD47 OS=Mus musculus GN=Cd47 PE=1 SV=2 - [CD47_MOUSE]	14.19	3	4	4	23	0.04	0.20	-0.13	-0.11
Q9D816	Cytochrome P450 2C55 OS=Mus musculus GN=Cyp2c55 PE=2 SV=1 - [CP255_MOUSE]	39.39	4	19	21	315	-1.08	-1.08	-1.14	-1.09
Q8R1A8	Ornithine carbamoyltransferase, mitochondrial OS=Mus musculus GN=Otc PE=2 SV=1 - [Q8R1A8_MOUSE]	47.29	2	13	13	61	-0.71	-0.64	-0.63	-0.58
D3YVG1	Septin-1 (Fragment) OS=Mus musculus GN=Sept1 PE=2 SV=1 - [D3YVG1_MOUSE]	5.38	2	1	1	1	-0.52	-0.39	-0.42	-0.29
O35344	Importin subunit alpha-4 OS=Mus musculus GN=Kpna3 PE=1 SV=1 - [IMA4_MOUSE]	8.45	2	1	4	17	0.37	0.36	0.34	0.33
Q571I9	Aldehyde dehydrogenase family 16 member A1 OS=Mus musculus GN=Aldh16a1 PE=2 SV=2 - [A16A1_MOUSE]	20.32	3	12	12	72	0.12	0.23	0.13	0.20
Q8VCQ8	Caldesmon 1 OS=Mus musculus GN=Cald1 PE=2 SV=1 - [Q8VCQ8_MOUSE]	57.74	4	1	35	857	0.33	0.27	0.24	0.19
Q8K0U4	Heat shock 70 kDa protein 12A OS=Mus musculus GN=Hspa12a PE=1 SV=1 - [HS12A_MOUSE]	14.07	1	7	7	23	-0.37	-0.29	-0.16	-0.12
Q9CWZ7	Gamma-soluble NSF attachment protein OS=Mus musculus GN=Napg PE=1 SV=1 - [SNAG_MOUSE]	47.44	2	10	11	46	-0.08	0.06	0.01	-0.05
Q9DC28-2	Isoform 2 of Casein kinase I isoform delta OS=Mus musculus GN=Csnk1d - [KC1D_MOUSE]	8.80	4	3	3	9	0.16	0.12	0.14	0.15

Q8CHT0	Delta-1-pyrroline-5-carboxylate dehydrogenase, mitochondrial OS=Mus musculus GN=Aldh4a1 PE=1 SV=3 - [AL4A1_MOUSE]	36.65	1	17	17	101	-0.20	-0.14	-0.21	-0.15
Q9CQE5	Regulator of G-protein signaling 10 OS=Mus musculus GN=Rgs10 PE=2 SV=1 - [RGS10_MOUSE]	24.86	2	4	4	14	0.12	0.02	0.08	0.21
Q9CRB6	Tubulin polymerization-promoting protein family member 3 OS=Mus musculus GN=Tppp3 PE=1 SV=1 - [TPPP3_MOUSE]	57.39	2	12	13	113	-0.11	-0.07	-0.23	-0.17
F6ZFT1	Acyl carrier protein (Fragment) OS=Mus musculus GN=Ndufab1 PE=2 SV=1 - [F6ZFT1_MOUSE]	30.71	3	5	5	33	0.14	0.38	-0.04	0.20
Q8CBC4	Consortin OS=Mus musculus GN=Cnst PE=1 SV=1 - [CNST_MOUSE]	9.85	1	2	3	9	-0.45	-0.72	-0.21	-0.60
Q91V08	C-type lectin domain family 2 member D OS=Mus musculus GN=Clec2d PE=1 SV=1 - [CLC2D_MOUSE]	14.49	3	2	2	5	0.22	0.17	0.17	0.12
P17427	AP-2 complex subunit alpha-2 OS=Mus musculus GN=Ap2a2 PE=1 SV=2 - [AP2A2_MOUSE]	29.74	2	20	24	101	-0.06	-0.17	-0.16	-0.24
P21841	Pulmonary surfactant-associated protein C OS=Mus musculus GN=Sftpc PE=2 SV=1 - [PSPC_MOUSE]	8.81	2	1	1	41	-0.06	-0.08	0.07	-0.08
Q9CQE8	UPF0568 protein C14orf166 homolog OS=Mus musculus PE=2 SV=1 - [CN166_MOUSE]	59.43	2	14	14	152	-0.13	-0.03	-0.07	-0.02
D3Z3D9	Beta-chimaerin OS=Mus musculus GN=Chn2 PE=2 SV=1 - [D3Z3D9_MOUSE]	8.67	2	1	1	1	-0.25	-0.32	-0.35	-0.42
P80316	T-complex protein 1 subunit epsilon OS=Mus musculus GN=Cct5 PE=1 SV=1 - [TCPE_MOUSE]	51.20	2	23	24	351	0.27	0.28	0.20	0.27
Q2WG76	Protein ripply2 OS=Mus musculus GN=Ripply2 PE=2 SV=1 - [RIPP2_MOUSE]	13.28	1	1	1	1	1.37	1.77	1.17	1.57
Q08481	Platelet endothelial cell adhesion molecule OS=Mus musculus GN=Pecam1 PE=1 SV=1 - [PECA1_MOUSE]	10.59	6	6	6	25	-0.36	-0.38	-0.33	-0.44
Q9D051	Pyruvate dehydrogenase E1 component subunit beta, mitochondrial OS=Mus musculus GN=Pdhb PE=1 SV=1 - [ODPB_MOUSE]	36.49	1	10	10	220	-0.06	-0.04	-0.14	-0.15

Q91YS8	Calcium/calmodulin-dependent protein kinase type 1 OS=Mus musculus GN=Camk1 PE=1 SV=1 - [KCC1A_MOUSE]	26.47	2	5	8	47	0.14	0.19	0.14	0.21
H3BJ37	Catechol O-methyltransferase domain-containing protein 1 OS=Mus musculus GN=Comtd1 PE=2 SV=1 - [H3BJ37_MOUSE]	10.50	2	2	2	7	0.07	0.13	0.05	0.10
Q9CQL4	39S ribosomal protein L20, mitochondrial OS=Mus musculus GN=Mrpl20 PE=1 SV=1 - [RM20_MOUSE]	32.89	1	4	4	5	0.22	0.29	0.20	0.26
H3BJE0	Granulins (Fragment) OS=Mus musculus GN=Grn PE=2 SV=1 - [H3BJE0_MOUSE]	9.31	1	1	1	1	0.36	0.07	0.26	-0.03
Q9D8S3	ADP-ribosylation factor GTPase-activating protein 3 OS=Mus musculus GN=Arfgap3 PE=2 SV=2 - [ARFG3_MOUSE]	31.55	1	13	13	73	-0.02	-0.05	0.01	-0.09
Q91Z40	Gbp6 protein OS=Mus musculus GN=Gbp7 PE=2 SV=2 - [Q91Z40_MOUSE]	14.11	1	5	8	22	0.05	-0.07	0.25	0.02
P68254-2	Isoform 2 of 14-3-3 protein theta OS=Mus musculus GN=Ywhaq - [1433T_MOUSE]	65.43	4	11	17	444	0.10	0.24	0.03	0.06
E9PZM4	Protein Chd2 OS=Mus musculus GN=Chd2 PE=2 SV=1 - [E9PZM4_MOUSE]	3.56	2	2	2	3	0.21	0.23	-0.18	-0.16
Q9CQY2	RNMT-activating mini protein OS=Mus musculus GN=Fam103a1 PE=2 SV=1 - [RAM_MOUSE]	21.01	3	2	2	8	0.04	0.13	-0.11	-0.10
P60335	Poly(rC)-binding protein 1 OS=Mus musculus GN=Pcbp1 PE=1 SV=1 - [PCBP1_MOUSE]	52.53	1	9	15	304	0.06	0.02	0.14	0.05
P58044	Isopentenyl-diphosphate Delta-isomerase 1 OS=Mus musculus GN=Idi1 PE=2 SV=1 - [IDI1_MOUSE]	42.29	5	8	9	69	0.18	0.45	0.22	0.41
O54984	ATPase Asna1 OS=Mus musculus GN=Asna1 PE=1 SV=2 - [ASNA_MOUSE]	25.00	1	7	7	22	0.08	0.14	0.14	-0.08
Q924B0	Inositol (Myo)-1(Or 4)-monophosphatase 1 OS=Mus musculus GN=Impa1 PE=2 SV=1 - [Q924B0_MOUSE]	46.21	4	12	12	123	-0.05	0.07	-0.19	0.01
P51660	Peroxisomal multifunctional enzyme type 2 OS=Mus musculus GN=Hsd17b4 PE=1 SV=3 - [DHB4_MOUSE]	36.73	1	20	20	103	-0.20	-0.17	-0.22	-0.15

Q3USB7	Inactive phospholipase C-like protein 1 OS=Mus musculus GN=Plcl1 PE=1 SV=3 - [PLCL1_MOUSE]	3.92	1	2	2	2	0.08	-0.38	-0.16	-0.62
A2A9X5	5'(3')-deoxyribonucleotidase, cytosolic type OS=Mus musculus GN=Nt5c PE=2 SV=1 - [A2A9X5_MOUSE]	50.52	1	1	8	40	-0.08	-0.49	0.05	-0.47
Q99JB2	Stomatin-like protein 2, mitochondrial OS=Mus musculus GN=Stoml2 PE=1 SV=1 - [STML2_MOUSE]	22.95	4	7	7	46	-0.08	-0.09	-0.10	-0.07
Q810N8	Protein Rhox11 OS=Mus musculus GN=Rhox11 PE=2 SV=1 - [Q810N8_MOUSE]	2.91	1	1	1	1	1.73	1.66	-0.16	-0.23
Q8R5I6	Glutathione S-transferase mu 4 OS=Mus musculus GN=Gstm4 PE=2 SV=1 - [Q8R5I6_MOUSE]	37.61	3	3	8	189	-0.37	-0.33	-0.04	-0.26
A2ALW2	Protein Zkscan16 OS=Mus musculus GN=Zkscan16 PE=4 SV=1 - [A2ALW2_MOUSE]	4.53	119	2	3	4	0.00	-0.27	-0.43	-0.70
Q6PHN1	Coiled-coil domain-containing protein 57 OS=Mus musculus GN=Ccdc57 PE=2 SV=1 - [CCD57_MOUSE]	2.76	3	2	3	7	0.05	0.31	-0.03	0.23
E9PUL3	Calcium-activated chloride channel regulator 4 OS=Mus musculus GN=Clca4 PE=4 SV=1 - [E9PUL3_MOUSE]	14.56	2	8	11	48	0.60	0.57	0.64	0.62
Q9QZM8-2	Isoform 2 of F-box only protein 17 OS=Mus musculus GN=Fbxo17 - [FBX17_MOUSE]	3.31	2	1	1	1	-0.54	-0.24	-0.70	-0.40
Q9JJY4	Probable ATP-dependent RNA helicase DDX20 OS=Mus musculus GN=Ddx20 PE=1 SV=2 - [DDX20_MOUSE]	3.03	1	2	2	3	0.89	0.39	0.19	-0.31
Q8BNJ6	Neuropilin and tolloid-like protein 2 OS=Mus musculus GN=Neto2 PE=1 SV=1 - [NETO2_MOUSE]	6.48	2	2	2	2	0.67	0.49	0.27	0.09
Q8BWG9	Calcium release-activated calcium channel protein 1 OS=Mus musculus GN=Orai1 PE=2 SV=1 [CRCM1_MOUSE]	4.28	1	1	1	2	0.28	0.16	0.10	-0.03
A2AGR1	MAP kinase-activating death domain protein OS=Mus musculus GN=Madd PE=2 SV=1 [A2AGR1_MOUSE]	1.76	27	1	2	2	0.11	-0.06	0.66	0.49
Q8BY87-2	Isoform 2 of Ubiquitin carboxyl-terminal hydrolase 47 OS=Mus musculus GN=Usp47 - [UBP47_MOUSE]	7.96	2	9	9	15	0.01	-0.31	0.09	-0.26

Q91VR5	ATP-dependent RNA helicase DDX1 OS=Mus musculus GN=Ddx1 PE=1 SV=1 - [DDX1_MOUSE]	51.08	1	31	31	217	0.00	-0.02	-0.03	0.00
Q9WV54	Acid ceramidase OS=Mus musculus GN=Asah1 PE=1 SV=1 - [ASAH1_MOUSE]	33.50	3	15	15	118	-0.33	-0.39	-0.40	-0.47
Q9R0P5	Destrin OS=Mus musculus GN=Dstn PE=1 SV=3 - [DEST_MOUSE]	83.03	1	18	19	554	0.13	0.16	0.13	0.13
P56391	Cytochrome c oxidase subunit 6B1 OS=Mus musculus GN=Cox6b1 PE=1 SV=2 - [CX6B1_MOUSE]	70.93	1	7	7	236	0.17	0.25	0.09	0.23
P15656	Fibroblast growth factor 5 OS=Mus musculus GN=Fgf5 PE=2 SV=1 - [FGF5_MOUSE]	2.27	1	1	1	1	0.10	0.25	-0.05	0.10
Q9JMH9-1	Isoform 1 of Unconventional myosin-XVIIIa OS=Mus musculus GN=Myo18a - [MY18A_MOUSE]	20.84	14	30	32	101	0.08	0.10	0.03	0.04
Q99KH8	Serine/threonine-protein kinase 24 OS=Mus musculus GN=Stk24 PE=1 SV=1 - [STK24_MOUSE]	28.07	1	6	11	48	0.00	0.00	0.07	0.03
P04919-2	Isoform Kidney of Band 3 anion transport protein OS=Mus musculus GN=Slc4a1 - [B3AT_MOUSE]	3.76	2	2	2	6	-0.14	-0.25	-0.24	-0.36
Q99JW2	Aminoacylase-1 OS=Mus musculus GN=Acy1 PE=1 SV=1 - [ACY1_MOUSE]	31.62	1	11	11	36	-0.25	-0.60	-0.27	-0.45
H3BKG8	Putative adenosylhomocysteinase 3 (Fragment) OS=Mus musculus GN=Ahcyl2 PE=2 SV=1 - [H3BKG8_MOUSE]	47.06	2	1	1	3	0.23	0.13	-0.05	-0.16
P46737	Lys-63-specific deubiquitinase BRCC36 OS=Mus musculus GN=Brcc3 PE=2 SV=1 [BRCC3_MOUSE]	19.59	5	5	5	19	-0.07	-0.11	0.01	-0.01
G5E8T9	Hydroxyacyl glutathione hydrolase OS=Mus musculus GN=Hagh PE=3 SV=1 - [G5E8T9_MOUSE]	31.39	8	9	9	24	-0.20	-0.31	-0.17	-0.43
Q9EPK2	Protein XRP2 OS=Mus musculus GN=Rp2 PE=1 SV=3 - [XRP2_MOUSE]	10.37	4	4	5	19	-0.09	-0.03	-0.12	-0.12
H3BLB5	Uncharacterized protein OS=Mus musculus GN=Vmn2r-ps128 PE=2 SV=1 - [H3BLB5_MOUSE]	1.30	2	1	1	1	-0.78	-0.18	-0.54	0.06
Q8R5H1	Ubiquitin carboxyl-terminal hydrolase 15 OS=Mus musculus GN=Usp15 PE=2 SV=1 - [UBP15_MOUSE]	11.21	3	2	12	20	0.73	0.41	0.33	0.00

Q7TMY4	THO complex subunit 7 homolog OS=Mus musculus GN=Thoc7 PE=1 SV=2 - [THOC7_MOUSE]	12.75	2	2	3	4	0.14	-0.12	-0.09	-0.35
Q9DB05	Alpha-soluble NSF attachment protein OS=Mus musculus GN=Napa PE=1 SV=1 - [SNAA_MOUSE]	45.08	1	10	12	53	-0.06	-0.09	0.10	-0.10
Q9ESW4	Acylglycerol kinase, mitochondrial OS=Mus musculus GN=Agk PE=1 SV=1 - [AGK_MOUSE]	12.35	2	3	3	6	-0.19	-0.60	-0.10	-0.46
D3Z4B0	Protein Srsf11 (Fragment) OS=Mus musculus GN=Srsf11 PE=2 SV=1 - [D3Z4B0_MOUSE]	42.71	7	4	4	12	0.02	-0.37	0.37	-0.03
Q99MP8-2	Isoform 2 of BRCA1-associated protein OS=Mus musculus GN=Brap - [BRAP_MOUSE]	5.17	3	2	3	9	0.80	0.16	0.18	-0.46
P61622	Integrin alpha-11 OS=Mus musculus GN=Itga11 PE=1 SV=1 [ITA11_MOUSE]	3.87	1	2	2	5	-0.50	0.40	-0.41	0.50
Q8K274	Ketosamine-3-kinase OS=Mus musculus GN=Fn3krp PE=2 SV=2 - [KT3K_MOUSE]	19.42	1	6	6	11	-0.27	-0.10	-0.39	-0.20
Q99JY9	Actin-related protein 3 OS=Mus musculus GN=Actr3 PE=1 SV=3 [ARP3_MOUSE]	48.33	1	18	18	419	0.13	0.15	0.07	0.15
E9Q043	Protein Fndc1 OS=Mus musculus GN=Fndc1 PE=2 SV=1 - [E9Q043_MOUSE]	0.64	1	1	1	2	0.39	0.34	0.19	0.14
Q9Z2X1-2	Isoform 2 of Heterogeneous nuclear ribonucleoprotein F OS=Mus musculus GN=Hnrnpf - [HNRPF_MOUSE]	34.43	5	9	11	330	0.01	0.14	0.03	0.18
O35127	Protein C10 OS=Mus musculus GN=Grcc10 PE=2 SV=1 - [C10_MOUSE]	10.32	1	1	1	3	0.06	-0.46	-0.08	-0.60
Q8VC91	Ikbkg protein OS=Mus musculus GN=Ikbkg PE=2 SV=1 - [Q8VC91_MOUSE]	16.06	9	5	6	15	-0.13	-0.24	0.04	-0.05
Q61160	Protein FADD OS=Mus musculus GN=Fadd PE=1 SV=1 - [FADD_MOUSE]	25.85	1	5	5	16	0.17	0.21	0.20	0.06
Q9CRB8	Mitochondrial fission process protein 1 OS=Mus musculus GN=Mtfp1 PE=1 SV=1 - [MTFP1_MOUSE]	6.63	1	1	1	1	-0.32	-0.64	-0.14	-0.47
Q8BWS5	G protein-regulated inducer of neurite outgrowth 3 OS=Mus musculus GN=Gprin3 PE=1 SV=1 - [GRIN3_MOUSE]	6.42	1	3	4	6	-0.48	-0.53	-0.27	-0.32
A2A6T4	Immature colon carcinoma transcript 1, isoform CRA_c OS=Mus musculus GN=Ict1 PE=4 SV=1 - [A2A6T4_MOUSE]	32.26	4	5	5	18	0.02	-0.12	0.04	-0.05

O35144-2	Isoform 2 of Telomeric repeat-binding factor 2 OS=Mus musculus GN=Terf2 - [TERF2_MOUSE]	15.11	5	4	4	10	-0.02	-0.03	-0.08	-0.02
H3BLK8	Zinc finger homeobox protein 4 OS=Mus musculus GN=Zfhx4 PE=2 SV=1 - [H3BLK8_MOUSE]	1.50	4	3	3	4	0.39	0.65	0.08	0.34
Q9EP89	Serine beta-lactamase-like protein LACTB, mitochondrial OS=Mus musculus GN=Lactb PE=1 SV=1 - [LACTB_MOUSE]	9.26	1	5	5	10	-0.53	-0.05	-0.60	-0.05
Q3TIR1-3	Isoform 3 of Trafficking protein particle complex subunit 13 OS=Mus musculus GN=Trappc13 - [TPC13_MOUSE]	8.25	4	3	3	4	0.25	-0.19	0.05	-0.39
Q8C9B9	Death-inducer obliterator 1 OS=Mus musculus GN=Dido1 PE=1 SV=4 - [DIDO1_MOUSE]	4.43	2	8	8	24	-0.12	0.05	0.12	0.18
A2AMV1	RIKEN cDNA 2610012O22, isoform CRA_a OS=Mus musculus GN=Mrto4 PE=3 SV=1 - [A2AMV1_MOUSE]	12.18	3	3	3	7	0.16	0.23	0.20	0.27
Q8BX09-2	Isoform 2 of Retinoblastoma-binding protein 5 OS=Mus musculus GN=Rbbp5 - [RBBP5_MOUSE]	7.24	2	2	2	13	-0.17	-0.27	-0.10	-0.12
Q9JM51	Prostaglandin E synthase OS=Mus musculus GN=Ptges PE=1 SV=1 - [PTGES_MOUSE]	6.54	1	1	1	7	-0.19	0.09	-0.26	0.01
A8DUK4	Beta-globin OS=Mus musculus GN=Hbb-b1 PE=2 SV=1 - [A8DUK4_MOUSE]	62.59	2	3	10	980	-2.24	-2.20	-2.16	-2.08
P51569	Alpha-galactosidase A OS=Mus musculus GN=Gla PE=1 SV=1 - [AGAL_MOUSE]	11.22	3	4	4	18	-0.27	-0.13	-0.04	0.08
Q62273	Sulfate transporter OS=Mus musculus GN=Slc26a2 PE=1 SV=1 - [S26A2_MOUSE]	5.14	3	3	3	49	0.10	0.16	-0.07	0.15
Q8BKT7	THO complex subunit 5 homolog OS=Mus musculus GN=Thoc5 PE=1 SV=2 - [THOC5_MOUSE]	12.30	3	9	9	14	0.36	0.48	0.03	0.37
P68037	Ubiquitin-conjugating enzyme E2 L3 OS=Mus musculus GN=Ube2l3 PE=2 SV=1 - [UB2L3_MOUSE]	51.95	2	6	6	54	0.22	0.42	0.31	0.38
Q9JIX0	Enhancer of yellow 2 transcription factor homolog OS=Mus musculus GN=Eny2 PE=2 SV=1 - [ENY2_MOUSE]	40.59	1	4	4	16	-0.17	-0.28	0.09	-0.24
Q922B1	O-acetyl-ADP-ribose deacetylase MACROD1 OS=Mus musculus GN=Macrod1 PE=2 SV=2 - [MACD1_MOUSE]	6.19	1	2	2	12	-0.59	-0.30	-0.35	-0.31

Q8VCN9	Tubulin-specific chaperone C OS=Mus musculus GN=Tbcc PE=2 SV=1 - [TBCC_MOUSE]	12.90	1	4	4	10	0.43	0.32	0.33	0.20
E9Q892	Leucine-rich repeat and calponin homology domain-containing protein 3 (Fragment) OS=Mus musculus GN=Lrch3 PE=2 SV=1 [E9Q892_MOUSE]	10.74	9	3	3	10	0.01	0.07	0.51	-0.12
G3UYL7	Protein FAM13A OS=Mus musculus GN=Fam13a PE=2 SV=1 - [G3UYL7_MOUSE]	1.20	2	1	1	1	0.18	0.65	-0.07	0.40
P13745	Glutathione S-transferase A1 OS=Mus musculus GN=Gsta1 PE=1 SV=2 - [GSTA1_MOUSE]	36.32	4	1	9	42	-0.26	0.10	-0.02	0.33
P35235	Tyrosine-protein phosphatase non-receptor type 11 OS=Mus musculus GN=Ptpn11 PE=1 SV=2 - [PTN11_MOUSE]	31.66	2	17	17	91	-0.04	0.14	-0.04	0.10
Q9QXD6	Fructose-1,6-bisphosphatase 1 OS=Mus musculus GN=Fbp1 PE=2 SV=3 - [F16P1_MOUSE]	7.99	3	2	3	4	-0.54	-0.69	-0.67	-0.82
Q9R0M6	Ras-related protein Rab-9A OS=Mus musculus GN=Rab9a PE=1 SV=1 - [RAB9A_MOUSE]	29.85	2	4	4	14	0.06	0.14	0.27	0.28
Q0KL02-3	Isoform 3 of Triple functional domain protein OS=Mus musculus GN=Trio - [TRIO_MOUSE]	1.99	18	3	4	11	0.02	-0.39	-0.17	-0.58
A6H630	UPF0364 protein C6orf211 homolog OS=Mus musculus PE=2 SV=1 - [CF211_MOUSE]	10.93	4	4	4	10	0.14	0.14	0.12	0.26
Q8K3H0	DCC-interacting protein 13-alpha OS=Mus musculus GN=Appl1 PE=1 SV=1 - [DP13A_MOUSE]	17.96	1	12	12	38	-0.04	-0.11	-0.05	-0.19
O54988-2	Isoform 2 of STE20-like serine/threonine-protein kinase OS=Mus musculus GN=Slk - [SLK_MOUSE]	21.46	4	20	21	65	0.12	0.11	0.08	0.13
Q8BM54-2	Isoform 2 of E3 ubiquitin-protein ligase MYLIP OS=Mus musculus GN=Mylip - [MYLIP_MOUSE]	1.58	2	1	1	2	0.38	0.59	0.28	0.49
Q3URN6	Pregnancy-specific glycoprotein 29 OS=Mus musculus GN=Psg29 PE=2 SV=1 [Q3URN6_MOUSE]	6.84	1	1	1	2	0.54	-0.15	0.18	-0.51
Q8R516-3	Isoform 3 of E3 ubiquitin-protein ligase MIB2 OS=Mus musculus GN=Mib2 - [MIB2_MOUSE]	3.74	3	1	1	1	-0.01	0.13	-0.22	-0.09

A2A7U2	Protein Zmym6 OS=Mus musculus GN=Zmym6 PE=2 SV=1 - [A2A7U2_MOUSE]	1.90	2	1	1	1	-0.46	-0.25	-0.64	-0.42
Q8VBZ3	Cleft lip and palate transmembrane protein 1 homolog OS=Mus musculus GN=Clptm1 PE=1 SV=1 - [CLPT1_MOUSE]	16.27	1	7	7	20	0.32	0.10	-0.18	0.08
E9Q8G5	Protein A430033K04Rik OS=Mus musculus GN=A430033K04Rik PE=2 SV=1 - [E9Q8G5_MOUSE]	2.50	1	1	1	1	-0.47	-0.36	-0.29	-0.18
D3YZP9	Coiled-coil domain-containing protein 6 OS=Mus musculus GN=Ccdc6 PE=3 SV=1 - [CCDC6_MOUSE]	25.80	1	11	11	51	0.05	0.08	0.14	0.19
P14824	Annexin A6 OS=Mus musculus GN=Anxa6 PE=1 SV=3 - [ANXA6_MOUSE]	63.60	2	42	43	990	-0.16	-0.09	-0.19	-0.09
Q6NVE9	Protein phosphatase PTC7 homolog OS=Mus musculus GN=Pptc7 PE=2 SV=1 - [PPTC7_MOUSE]	4.84	1	1	1	2	0.12	0.34	-0.03	0.19
E9Q1P8	Interferon regulatory factor 2-binding protein 2 OS=Mus musculus GN=Irf2bp2 PE=1 SV=1 - [I2BP2_MOUSE]	25.96	1	10	10	27	-0.01	-0.07	-0.13	-0.24
D3YXJ5	Protein Fam84b OS=Mus musculus GN=Fam84b PE=4 SV=1 - [D3YXJ5_MOUSE]	11.61	1	2	2	8	-0.28	-0.07	-0.45	-0.22
Q99JP0	Mitogen-activated protein kinase kinase kinase kinase 3 OS=Mus musculus GN=Map4k3 PE=1 SV=4 - [M4K3_MOUSE]	2.24	2	1	1	2	0.40	0.59	0.19	0.37
Q9CQB5	CDGSH iron-sulfur domain-containing protein 2 OS=Mus musculus GN=Cisd2 PE=1 SV=1 - [CISD2_MOUSE]	42.22	2	5	5	23	0.09	0.18	0.12	0.13
P50516	V-type proton ATPase catalytic subunit A OS=Mus musculus GN=Atp6v1a PE=1 SV=2 - [VATA_MOUSE]	39.87	4	22	22	251	-0.07	-0.15	-0.15	-0.26
Q8CFE6	Sodium-coupled neutral amino acid transporter 2 OS=Mus musculus GN=Slc38a2 PE=1 SV=1 - [S38A2_MOUSE]	4.17	1	1	1	14	-0.09	-0.16	-0.01	-0.09
Q99PG2	Opioid growth factor receptor OS=Mus musculus GN=Ogfr PE=2 SV=1 - [OGFR_MOUSE]	20.70	2	5	5	23	-0.06	-0.01	0.02	-0.12
E9Q9D8	Protein Ankrd35 OS=Mus musculus GN=Ankrd35 PE=2 SV=1 - [E9Q9D8_MOUSE]	2.21	1	1	1	1	-0.50	-0.42	-0.22	-0.13

P42932	T-complex protein 1 subunit theta OS=Mus musculus GN=Cct8 PE=1 SV=3 - [TCPQ_MOUSE]	61.50	6	30	30	295	0.09	0.17	0.13	0.21
A2AR02	Peptidyl-prolyl cis-trans isomerase G OS=Mus musculus GN=Ppig PE=1 SV=1 - [PPIG_MOUSE]	2.79	2	2	2	3	0.11	0.04	-0.04	0.09
Q8VCR4	0610038D11Rik protein OS=Mus musculus GN=Trmt112 PE=4 SV=1 - [Q8VCR4_MOUSE]	12.04	2	1	1	16	0.05	-0.04	0.17	0.21
Q9WUR9	GTP:AMP phosphotransferase AK4, mitochondrial OS=Mus musculus GN=Ak4 PE=2 SV=1 - [KAD4_MOUSE]	39.01	4	6	6	25	-0.05	-0.08	-0.17	-0.20
Q61847	Meprin A subunit beta OS=Mus musculus GN=Mep1b PE=1 SV=2 - [MEP1B_MOUSE]	6.25	2	4	4	9	0.63	0.16	0.25	0.14
Q61216-2	Isoform 2 of Double-strand break repair protein MRE11A OS=Mus musculus GN=Mre11a - [MRE11_MOUSE]	10.01	5	5	5	16	-0.16	-0.01	0.01	-0.10
Q0GNC1-3	Isoform 2 of Inverted formin-2 OS=Mus musculus GN=Inf2 - [INF2_MOUSE]	2.85	3	2	2	2	0.37	0.60	0.05	0.27
P15864	Histone H1.2 OS=Mus musculus GN=Hist1h1c PE=1 SV=2 - [H12_MOUSE]	42.45	1	7	14	490	0.08	0.25	-0.07	0.15
O55201-2	Isoform 2 of Transcription elongation factor SPT5 OS=Mus musculus GN=Supt5h - [SPT5H_MOUSE]	27.25	2	19	19	67	0.08	-0.09	0.03	-0.05
B1AXA6	Dachshund homolog 2 OS=Mus musculus GN=Dach2 PE=2 SV=1 - [B1AXA6_MOUSE]	3.13	5	2	2	3	0.33	0.03	0.43	0.13
E9PWX8	Microtubule-associated serine/threonine-protein kinase 4 OS=Mus musculus GN=Mast4 PE=2 SV=1 - [E9PWX8_MOUSE]	7.54	18	9	10	20	0.07	0.04	0.01	-0.01
Q06507	Cyclic AMP-dependent transcription factor ATF-4 OS=Mus musculus GN=Atf4 PE=1 SV=2 - [ATF4_MOUSE]	3.72	1	1	1	1	-0.81	-3.37	-1.02	-3.57
Q8C4Q6	Axin interactor, dorsalization-associated protein OS=Mus musculus GN=Aida PE=1 SV=1 - [AIDA_MOUSE]	9.84	2	2	2	4	0.33	0.16	0.22	0.04
Q9DAR7	m7GpppX diphosphatase OS=Mus musculus GN=Dcps PE=1 SV=1 - [DCPS_MOUSE]	18.34	2	5	5	32	0.26	0.35	0.14	0.04
Q9DC42	CD97 antigen OS=Mus musculus GN=Cd97 PE=2 SV=1 - [Q9DC42_MOUSE]	9.83	6	6	6	13	-0.42	-0.14	-0.20	-0.08

Q61087	Laminin subunit beta-3 OS=Mus musculus GN=Lamb3 PE=2 SV=2 - [LAMB3_MOUSE]	5.31	1	4	6	15	-0.01	0.01	0.00	0.06
Q8K4P0	pre-mRNA 3' end processing protein WDR33 OS=Mus musculus GN=Wdr33 PE=2 SV=1 - [WDR33_MOUSE]	11.58	2	7	7	12	0.13	0.04	0.12	-0.02
Q63918	Serum deprivation-response protein OS=Mus musculus GN=Sdpr PE=1 SV=3 - [SDPR_MOUSE]	53.59	1	16	16	136	-0.36	-0.24	-0.36	-0.33
Q6P3A8	2-oxoisovalerate dehydrogenase subunit beta, mitochondrial OS=Mus musculus GN=Bckdhb PE=2 SV=2 - [ODBB_MOUSE]	5.13	1	1	1	2	0.13	-0.04	-0.05	-0.22
Q8VCQ3	Nuclear receptor-binding factor 2 OS=Mus musculus GN=Nrbf2 PE=1 SV=1 [NRBF2_MOUSE]	12.20	2	3	3	7	0.23	0.12	0.21	0.11
P29387	Guanine nucleotide-binding protein subunit beta-4 OS=Mus musculus GN=Gnb4 PE=2 SV=4 - [GBB4_MOUSE]	13.53	2	1	4	41	-0.54	-0.43	-0.44	-0.33
Q3LS21	Potassium channel subfamily K member 9 OS=Mus musculus GN=Kcnk9 PE=2 SV=1 - [KCNK9_MOUSE]	1.49	1	1	1	2	0.86	-0.22	1.00	-0.09
O70310	Glycylpeptide N-tetradecanoyltransferase 1 OS=Mus musculus GN=Nmt1 PE=1 SV=1 [NMT1_MOUSE]	23.39	1	8	10	55	0.31	0.21	0.31	0.20
Q3TFK5-2	Isoform 2 of G patch domain-containing protein 4 OS=Mus musculus GN=Gpatch4 [GPTC4_MOUSE]	2.74	2	1	1	2	0.08	0.01	-0.13	0.08
P01630	Ig kappa chain V-II region 7S34.1 OS=Mus musculus PE=1 SV=1 - [KV2A6_MOUSE]	9.73	1	1	1	2	-0.44	-0.78	-0.41	-0.75
Q9CQX2	Cytochrome b5 type B OS=Mus musculus GN=Cyb5b PE=1 SV=1 - [CYB5B_MOUSE]	59.59	1	7	7	81	-1.01	-1.23	-1.05	-1.03
D3YX27	Serine protease HTRA2, mitochondrial OS=Mus musculus GN=Htra2 PE=2 SV=1 - [D3YX27_MOUSE]	17.84	6	6	6	28	-0.44	-0.44	-0.32	-0.25
P97370	Sodium/potassium-transporting ATPase subunit beta-3 OS=Mus musculus GN=Atp1b3 PE=1 SV=1 - [AT1B3_MOUSE]	32.73	1	6	6	31	-0.07	-0.17	-0.30	-0.33
Q3U8Y1	28S ribosomal protein S11, mitochondrial OS=Mus musculus GN=Mrps11 PE=2 SV=1 - [Q3U8Y1_MOUSE]	15.18	2	2	2	6	-0.25	-0.32	0.02	-0.14

Q6P4T1	Protein Snx19 OS=Mus musculus GN=Snx19 PE=2 SV=1 - [Q6P4T1_MOUSE]	3.61	1	2	2	2	-0.66	-1.46	-0.61	-1.41
Q5SW19-3	Isoform 3 of Clustered mitochondria protein homolog OS=Mus musculus GN=Cluh - [CLU_MOUSE]	22.07	3	22	23	72	0.12	-0.07	0.06	-0.09
B1AY10	Transcriptional repressor NF-X1 OS=Mus musculus GN=Nfx1 PE=2 SV=1 - [NFX1_MOUSE]	0.81	1	1	1	2	0.26	0.13	0.26	0.13
Q8BG15-6	Isoform 6 of CTD small phosphatase-like protein 2 OS=Mus musculus GN=Ctdspl2 - [CTSL2_MOUSE]	1.78	3	1	1	2	0.14	0.06	-0.08	-0.16
G3UW75	Kelch-like 7 (Drosophila), isoform CRA_a OS=Mus musculus GN=Klhl7 PE=4 SV=1 - [G3UW75_MOUSE]	2.96	2	1	1	1	0.34	0.15	0.69	0.50
Q5SUT0	RNA-binding protein EWS OS=Mus musculus GN=Ewsr1 PE=2 SV=1 - [Q5SUT0_MOUSE]	13.92	4	8	8	57	0.32	0.22	0.31	0.20
Q64442	Sorbitol dehydrogenase OS=Mus musculus GN=Sord PE=1 SV=3 - [DHSO_MOUSE]	38.94	1	13	13	85	-0.49	-0.40	-0.49	-0.42
Q8CFX3	Protein Pcdh1 OS=Mus musculus GN=Pcdh1 PE=2 SV=1 - [Q8CFX3_MOUSE]	10.02	3	8	9	28	-0.22	-0.35	-0.05	-0.16
Q8BZ33	Tensin-4 OS=Mus musculus GN=Tns4 PE=2 SV=2 - [TENS4_MOUSE]	1.44	1	1	1	6	-0.42	-0.29	-0.25	-0.13
F7AMS7	Misshapen-like kinase 1 (Fragment) OS=Mus musculus GN=Mink1 PE=4 SV=1 - [F7AMS7_MOUSE]	3.84	16	3	3	3	0.67	-0.47	0.60	-0.54
Q8K2I3	Dimethylaniline monooxygenase [N-oxide-forming] 2 OS=Mus musculus GN=Fmo2 PE=1 SV=3 [FMO2_MOUSE]	23.93	2	13	13	68	-0.91	-0.95	-0.75	-0.79
D3Z6I3	Lipoyl synthase, mitochondrial OS=Mus musculus GN=Lias PE=2 SV=1 - [D3Z6I3_MOUSE]	7.27	2	1	1	3	-0.16	-0.15	-0.27	-0.26
Q9CZ13	Cytochrome b-c1 complex subunit 1, mitochondrial OS=Mus musculus GN=Uqcrc1 PE=1 SV=2 - [QCR1_MOUSE]	50.63	1	18	19	615	-0.28	-0.27	-0.26	-0.24
B0R058	Glycerophosphocholine phosphodiesterase GPCPD1 OS=Mus musculus GN=Gpcpd1 PE=2 SV=1 - [B0R058_MOUSE]	2.25	3	1	1	1	0.21	0.00	0.19	-0.02
O35350	Calpain-1 catalytic subunit OS=Mus musculus GN=Capn1 PE=2 SV=1 - [CAN1_MOUSE]	34.64	1	24	24	157	0.00	0.06	0.00	0.01

D3YVR5	DDB1- and CUL4-associated factor 11 (Fragment) OS=Mus musculus GN=Dcaf11 PE=2 SV=1 - [D3YVR5_MOUSE]	6.37	4	1	1	1	-0.07	-0.10	0.14	0.11
E9PWE8	Dihydropyrimidinase-related protein 3 OS=Mus musculus GN=Dpysl3 PE=2 SV=1 - [E9PWE8_MOUSE]	49.34	5	19	24	313	-0.03	0.05	-0.06	-0.01
D3Z7X0	MCG142036 OS=Mus musculus GN=Acad12 PE=3 SV=1 - [D3Z7X0_MOUSE]	12.79	3	1	6	28	-0.29	-0.45	-0.31	-0.46
H3BLR7	Atrial natriuretic peptide converting enzyme (Fragment) OS=Mus musculus GN=Corin PE=2 SV=1 - [H3BLR7_MOUSE]	1.13	5	1	1	2	1.31	1.51	1.46	1.67
P70296	Phosphatidylethanolamine-binding protein 1 OS=Mus musculus GN=Pebp1 PE=1 SV=3 [PEBP1_MOUSE]	66.84	6	9	9	295	-0.03	-0.06	-0.03	-0.03
Q8R048	C-Myc-binding protein OS=Mus musculus GN=Mycbp PE=4 SV=1 - [Q8R048_MOUSE]	62.14	3	5	5	16	0.13	0.18	0.02	0.05
E9Q1M6	Protein Maskbp3 OS=Mus musculus GN=Ankhd1 PE=4 SV=1 - [E9Q1M6_MOUSE]	6.56	6	5	9	23	-0.73	-0.34	-0.37	-0.10
Q8R3B1	1-phosphatidylinositol 4,5-bisphosphate phosphodiesterase delta-1 OS=Mus musculus GN=Plcd1 PE=2 SV=2 - [PLCD1_MOUSE]	26.19	1	14	15	98	-0.22	-0.15	-0.21	-0.12
Q8CBY8-2	Isoform 2 of Dynactin subunit 4 OS=Mus musculus GN=Dctn4 - [DCTN4_MOUSE]	29.13	2	8	8	23	0.00	-0.19	-0.10	-0.22
P00520-2	Isoform II of Tyrosine-protein kinase ABL1 OS=Mus musculus GN=Abl1 - [ABL1_MOUSE]	3.22	4	2	3	4	0.06	0.42	-0.04	0.31
P63037	DnaJ homolog subfamily A member 1 OS=Mus musculus GN=Dnaja1 PE=1 SV=1 - [DNJA1_MOUSE]	42.57	5	12	15	106	0.03	0.05	0.10	0.22
P80314	T-complex protein 1 subunit beta OS=Mus musculus GN=Cct2 PE=1 SV=4 - [TCPB_MOUSE]	60.19	1	28	28	477	0.17	0.27	0.16	0.21
Q91W53	Golgin subfamily A member 7 OS=Mus musculus GN=Golga7 PE=2 SV=1 - [GOGA7_MOUSE]	16.06	2	2	2	3	-0.11	-0.11	-0.25	-0.25
O35601-2	Isoform FYB-120 of FYN-binding protein OS=Mus musculus GN=Fyb - [FYB_MOUSE]	4.53	2	3	3	3	0.06	-0.52	0.19	-0.09

Q9D1H6	NADH dehydrogenase [ubiquinone] 1 alpha subcomplex assembly factor 4 OS=Mus musculus GN=Ndufaf4 PE=2 SV=1 - [NDUF4_MOUSE]	19.08	2	3	3	3	0.21	-0.08	0.17	-0.12
Q91XX4	MCG133388, isoform CRA_n OS=Mus musculus GN=Pcdhgb6 PE=2 SV=1 - [Q91XX4_MOUSE]	2.69	10	1	2	5	-0.42	-0.10	-0.28	0.03
Q3TNA1	Xylulose kinase OS=Mus musculus GN=Xylb PE=2 SV=1 - [XYLB_MOUSE]	6.17	1	2	2	4	-0.57	-0.59	-0.27	-0.29
Q3UB74	Transforming growth factor beta regulator 1 OS=Mus musculus GN=Tbrg1 PE=1 SV=2 [TBRG1_MOUSE]	1.97	1	1	1	3	0.07	0.46	0.00	0.39
Q8BIH0-2	Isoform 2 of Histone deacetylase complex subunit SAP130 OS=Mus musculus GN=Sap130 - [SP130_MOUSE]	2.62	3	2	2	4	-0.25	-0.24	-0.17	-0.16
A2APM2	CD44 antigen OS=Mus musculus GN=Cd44 PE=2 SV=1 - [A2APM2_MOUSE]	11.67	21	7	7	44	-0.38	-0.35	-0.44	-0.34
O88746	Target of Myb protein 1 OS=Mus musculus GN=Tom1 PE=1 SV=1 [TOM1_MOUSE]	30.08	3	9	9	55	-0.19	-0.18	-0.17	-0.15
P57716	Nicastrin OS=Mus musculus GN=Ncstn PE=1 SV=3 - [NICA_MOUSE]	7.34	1	5	5	29	-0.37	0.02	-0.37	0.02
Q9JKY7	Cytochrome P450 CYP2D22 OS=Mus musculus GN=Cyp2d22 PE=2 SV=1 - [Q9JKY7_MOUSE]	9.00	1	3	3	6	0.21	0.11	0.06	-0.03
G5E8S8	MAGUK p55 subfamily member 7 OS=Mus musculus GN=Mpp7 PE=4 SV=1 - [G5E8S8_MOUSE]	13.89	6	7	7	18	-0.12	-0.38	-0.17	-0.25
J3QPE8	MCG16555 OS=Mus musculus GN=Vdac3-ps1 PE=4 SV=1 - [J3QPE8_MOUSE]	53.36	3	10	12	195	-0.51	-0.36	-0.54	-0.38
P60670	Nuclear protein localization protein 4 homolog OS=Mus musculus GN=Nploc4 PE=1 SV=3 - [NPL4_MOUSE]	25.49	2	12	12	45	-0.03	0.12	-0.12	0.06
O54833	Casein kinase II subunit alpha' OS=Mus musculus GN=Csnk2a2 PE=2 SV=1 - [CSK22_MOUSE]	27.43	1	7	7	26	-0.21	-0.18	-0.29	-0.29
Q5U4C2	Dipeptidyl aminopeptidase-like protein 6 OS=Mus musculus GN=Dpp6 PE=2 SV=1 - [Q5U4C2_MOUSE]	2.50	4	2	2	2	-0.48	-0.32	-0.64	-0.48
Q06185	ATP synthase subunit e, mitochondrial OS=Mus musculus GN=Atp5i PE=1 SV=2 - [ATP5I_MOUSE]	43.66	2	3	3	21	-0.08	-0.05	-0.22	0.10

Q8C0L6	Peroxisomal N(1)-acetyl-spermine/spermidine oxidase OS=Mus musculus GN=Paox PE=1 SV=3 - [PAOX_MOUSE]	15.87	3	8	8	18	-0.05	0.15	-0.01	-0.07
F8VPU6	Ubiquitin carboxyl-terminal hydrolase OS=Mus musculus GN=Usp9y PE=3 SV=1 - [F8VPU6_MOUSE]	3.01	1	3	6	11	-1.74	-4.09	-1.32	-3.67
O70378	ER membrane protein complex subunit 8 OS=Mus musculus GN=Emc8 PE=1 SV=1 [EMC8_MOUSE]	37.20	7	7	7	16	0.24	0.23	-0.02	0.21
Q64092-3	Isoform 3 of Transcription factor E3 OS=Mus musculus GN=Tfe3 - [TFE3_MOUSE]	2.31	6	1	1	1	-0.65	-0.67	-0.58	-0.61
Q8R2Y0-2	Isoform 2 of Monoacylglycerol lipase ABHD6 OS=Mus musculus GN=Abhd6 - [ABHD6_MOUSE]	13.84	2	3	3	7	0.09	0.04	-0.32	-0.27
Q6P8N8	ATP-dependent Clp protease ATP-binding subunit clpX-like, mitochondrial OS=Mus musculus GN=Clpx PE=2 SV=1 - [Q6P8N8_MOUSE]	13.06	3	7	7	24	0.13	0.08	0.18	0.16
E9Q7P9	Protein Cdhr2 OS=Mus musculus GN=Cdhr2 PE=2 SV=1 - [E9Q7P9_MOUSE]	14.68	1	14	14	56	-0.09	-0.27	-0.05	-0.31
D3YX85	Arf-GAP with SH3 domain, ANK repeat and PH domain-containing protein 2 OS=Mus musculus GN=Asap2 PE=2 SV=2 - [D3YX85_MOUSE]	9.01	4	4	6	39	-0.56	-0.34	-0.22	-0.54
P14901	Heme oxygenase 1 OS=Mus musculus GN=Hmox1 PE=1 SV=1 - [HMOX1_MOUSE]	10.03	1	1	2	3	-0.53	-0.76	0.16	-0.08
P70436	Homeobox protein DLX 4 OS=Mus musculus GN=Dlx4 PE=2 SV=2 - [DLX4_MOUSE]	5.83	1	1	1	1	-0.19	-0.27	0.09	0.01
O35678	Monoglyceride lipase OS=Mus musculus GN=Mgll PE=1 SV=1 - [MGLL_MOUSE]	25.08	5	6	6	20	-0.58	-0.51	-0.64	-0.54
E9Q6A7	Protein Bptf OS=Mus musculus GN=Bptf PE=2 SV=1 - [E9Q6A7_MOUSE]	4.28	3	8	8	9	-0.06	0.11	0.08	0.00
P60766	Cell division control protein 42 homolog OS=Mus musculus GN=Cdc42 PE=1 SV=2 - [CDC42_MOUSE]	31.41	6	2	5	131	0.09	0.16	0.18	0.27
Q8R0Y6	Cytosolic 10-formyltetrahydrofolate dehydrogenase OS=Mus musculus GN=Aldh1l1 PE=2 SV=1 - [AL1L1_MOUSE]	41.24	1	29	31	236	0.05	0.08	0.02	0.07

Q91VT4	Carbonyl reductase family member 4 OS=Mus musculus GN=Cbr4 PE=2 SV=2 - [CBR4_MOUSE]	17.80	1	2	4	11	-0.24	0.09	-0.33	-0.01
D3YTW9	Protein Wnt OS=Mus musculus GN=Wnt5b PE=2 SV=1 - [D3YTW9_MOUSE]	6.85	2	1	1	1	-0.23	0.52	-0.53	0.22
Q8R3H9	Tetratricopeptide repeat protein 4 OS=Mus musculus GN=Ttc4 PE=2 SV=1 - [TTC4_MOUSE]	11.66	3	4	4	11	0.12	-0.07	0.32	0.03
Q9QX60-2	Isoform 2 of Deoxyguanosine kinase, mitochondrial OS=Mus musculus GN=Dguok - [DGUOK_MOUSE]	17.48	3	3	3	13	0.44	-0.09	0.26	-0.27
Q62148	Retinal dehydrogenase 2 OS=Mus musculus GN=Aldh1a2 PE=1 SV=2 - [AL1A2_MOUSE]	22.20	1	6	12	87	-0.05	-0.01	0.26	0.11
Q99KY4-2	Isoform 2 of Cyclin-G-associated kinase OS=Mus musculus GN=Gak - [GAK_MOUSE]	14.49	6	14	15	24	0.20	0.05	0.06	-0.03
A2A547	60S ribosomal protein L19 OS=Mus musculus GN=Rpl19 PE=2 SV=1 [A2A547_MOUSE]	32.99	2	7	9	193	0.19	0.07	0.11	-0.03
A2AA86	SUZ domain-containing protein 1 OS=Mus musculus GN=Szrd1 PE=2 SV=1 - [A2AA86_MOUSE]	42.42	4	4	4	11	-0.25	0.00	-0.06	0.03
Q8CA87	Protein A330017A19Rik OS=Mus musculus GN=A330017A19Rik PE=2 SV=1 - [Q8CA87_MOUSE]	5.15	1	1	1	2	0.35	-0.18	0.42	-0.11
Q9D727	Uncharacterized protein C6orf226 homolog OS=Mus musculus PE=4 SV=1 - [CF226_MOUSE]	64.91	1	4	4	9	-0.15	-0.36	-0.07	0.01
Q8CAA7	Glucose 1,6-bisphosphate synthase OS=Mus musculus GN=Pgm2l1 PE=1 SV=1 - [PGM2L_MOUSE]	7.09	2	4	4	6	-0.59	-0.95	-0.42	-0.78
E0CZ04	Gamma-glutamylaminecyclotransferase (Fragment) OS=Mus musculus GN=Ggact PE=2 SV=1 [E0CZ04_MOUSE]	41.90	4	3	3	27	-0.29	-0.31	-0.24	-0.15
Q6ZPE2	Myotubularin-related protein 5 OS=Mus musculus GN=Sbf1 PE=1 SV=2 - [MTMR5_MOUSE]	3.54	6	4	5	13	-0.13	-0.09	-0.23	0.15
O70305-3	Isoform 3 of Ataxin-2 OS=Mus musculus GN=Atxn2 - [ATX2_MOUSE]	8.93	7	9	12	33	-0.08	-0.04	-0.23	-0.20
Q8BHG9	CGG triplet repeat-binding protein 1 OS=Mus musculus GN=Cggbp1 PE=2 SV=1 - [CGBP1_MOUSE]	28.14	1	4	4	9	0.18	0.18	0.12	0.16
Q9DA97	Septin-14 OS=Mus musculus GN=Sept14 PE=2 SV=3 - [SEP14_MOUSE]	1.40	1	1	1	5	0.30	0.38	0.18	0.26

Q5SUF2	Luc7-like protein 3 OS=Mus musculus GN=Luc7l3 PE=1 SV=1 - [LC7L3_MOUSE]	13.66	3	5	5	13	0.35	0.35	0.24	0.28
A2AWF8	Receptor-type tyrosine-protein phosphatase eta OS=Mus musculus GN=Ptprj PE=2 SV=1 - [A2AWF8_MOUSE]	1.03	4	1	1	1	-0.66	-2.05	-0.18	-1.56
G3UYD0	General transcription factor II-I OS=Mus musculus GN=Gtf2i PE=2 SV=1 - [G3UYD0_MOUSE]	18.95	13	13	15	32	-0.20	-0.09	-0.14	-0.08
E9Q7N9	Protein Dnah11 OS=Mus musculus GN=Dnah11 PE=4 SV=2 - [E9Q7N9_MOUSE]	2.25	1	5	7	9	0.10	-0.29	-0.24	-0.55
P25911-2	Isoform 2 of Tyrosine-protein kinase Lyn OS=Mus musculus GN=Lyn - [LYN_MOUSE]	23.83	32	6	10	50	0.12	-0.16	0.61	0.04
Q9D8U8	Sorting nexin-5 OS=Mus musculus GN=Snx5 PE=1 SV=1 - [SNX5_MOUSE]	45.30	1	19	20	73	0.15	0.31	0.17	0.30
Q6PFY1	FCH and double SH3 domains protein 1 OS=Mus musculus GN=Fchsd1 PE=2 SV=1 - [FCSD1_MOUSE]	8.43	1	3	3	4	0.90	0.54	0.58	0.22
E0CYH0	MCG16685, isoform CRA_d OS=Mus musculus GN=Wtap PE=4 SV=1 - [E0CYH0_MOUSE]	14.39	5	5	5	12	0.08	-0.06	0.28	0.00
F7CJY7	Syntaxin-16 (Fragment) OS=Mus musculus GN=Stx16 PE=4 SV=1 [F7CJY7_MOUSE]	12.79	5	3	3	6	0.06	0.19	-0.13	0.01
Q8C0E2	Vacuolar protein sorting associated protein 26B OS=Mus musculus GN=Vps26b PE=1 SV=1 - [VP26B_MOUSE]	30.95	2	9	10	33	-0.17	-0.11	-0.13	-0.04
Q05860-4	Isoform 4 of Formin-1 OS=Mus musculus GN=Fmn1 - [FMN1_MOUSE]	1.12	5	1	1	2	-0.13	-0.20	-0.32	-0.39
Q8BTW9	Serine/threonine-protein kinase PAK 4 OS=Mus musculus GN=Pak4 PE=1 SV=1 - [PAK4_MOUSE]	9.44	3	3	3	23	-0.43	-0.60	-0.50	-0.66
Q9D5Y1	Coiled-coil domain-containing protein 39 OS=Mus musculus GN=Ccdc39 PE=2 SV=1 - [CCD39_MOUSE]	2.03	1	2	2	2	-0.56	-0.43	-0.39	-0.27
Q8K003	Translation machinery-associated protein 7 OS=Mus musculus GN=Tma7 PE=2 SV=1 [TMA7_MOUSE]	39.06	5	2	3	8	0.25	0.57	0.40	0.52
Q8BVF2	Phosducin-like protein 3 OS=Mus musculus GN=Pdcl3 PE=1 SV=1 - [PDCL3_MOUSE]	22.08	1	6	6	24	0.16	0.19	0.25	0.22
Q8BV13	COP9 signalosome complex subunit 7b OS=Mus musculus GN=Cops7b PE=1 SV=1 - [CSN7B_MOUSE]	16.29	1	3	3	11	-0.35	-0.11	-0.42	-0.21

A2AEV2	WD repeat domain phosphoinositide-interacting protein 4 OS=Mus musculus GN=Wdr45 PE=2 SV=1 - [A2AEV2_MOUSE]	2.73	4	1	1	1	0.09	-0.23	-0.02	-0.33
P00416	Cytochrome c oxidase subunit 3 OS=Mus musculus GN=Mtco3 PE=3 SV=1 - [COX3_MOUSE]	5.36	2	1	1	8	-0.41	-0.10	-0.45	-0.41
Q9JHI7	Exosome complex component RRP45 OS=Mus musculus GN=Exosc9 PE=2 SV=1 - [EXOS9_MOUSE]	12.56	2	2	2	3	-0.24	-0.10	-0.24	-0.10
Q3UHJ0-2	Isoform 2 of AP2-associated protein kinase 1 OS=Mus musculus GN=Aak1 - [AAK1_MOUSE]	14.58	2	7	7	31	-0.19	-0.14	-0.17	-0.26
B1AUC4	Protein Ube2u OS=Mus musculus GN=Ube2u PE=2 SV=1 - [B1AUC4_MOUSE]	3.69	1	2	2	2	0.08	0.12	0.19	0.22
O35607	Bone morphogenetic protein receptor type-2 OS=Mus musculus GN=Bmpr2 PE=2 SV=1 - [BMPR2_MOUSE]	5.49	1	3	4	5	-0.15	0.14	-0.35	-0.06
Q99N28	Cell adhesion molecule 3 OS=Mus musculus GN=Cadm3 PE=1 SV=1 - [CADM3_MOUSE]	10.61	2	3	3	9	-0.45	-0.20	-0.52	-0.27
F6QKK2	ADP-ribosylation factor like protein 8A (Fragment) OS=Mus musculus GN=Arl8a PE=4 SV=1 - [F6QKK2_MOUSE]	55.76	2	3	7	35	-0.37	-0.29	-0.27	-0.19
Q91VB8	Alpha globin 1 OS=Mus musculus GN=Hba-a1 PE=2 SV=1 - [Q91VB8_MOUSE]	61.27	4	8	8	617	0.07	0.02	0.07	0.04
P11983	T-complex protein 1 subunit alpha OS=Mus musculus GN=Tcp1 PE=1 SV=3 - [TCPA_MOUSE]	44.42	3	23	23	189	-0.03	-0.02	0.01	0.04
P97798-5	Isoform 5 of Neogenin OS=Mus musculus GN=Neo1 - [NEO1_MOUSE]	8.26	6	8	8	39	-0.14	-0.03	-0.02	-0.14
Q8BM72	Heat shock 70 kDa protein 13 OS=Mus musculus GN=Hspa13 PE=2 SV=1 - [HSP13_MOUSE]	15.92	3	6	6	32	0.03	-0.08	0.09	-0.08
P70349	Histidine triad nucleotide-binding protein 1 OS=Mus musculus GN=Hint1 PE=1 SV=3 - [HINT1_MOUSE]	62.70	2	7	7	136	-0.06	0.02	-0.07	0.02
Q9CWV4	Melanoma-associated antigen B16 OS=Mus musculus GN=Mageb16 PE=2 SV=1 - [MAGBG_MOUSE]	3.03	1	1	1	1	0.64	0.20	0.72	0.28
P50544	Very long-chain specific acyl-CoA dehydrogenase, mitochondrial OS=Mus musculus GN=Acadvl PE=1 SV=3 - [ACADV_MOUSE]	38.26	2	26	26	242	-0.23	-0.23	-0.22	-0.21

Q9D2X5-2	Isoform 2 of MAU2 chromatid cohesion factor homolog OS=Mus musculus GN=Mau2 - [SCC4_MOUSE]	1.13	2	1	1	2	0.32	0.03	0.30	0.01
Q9DCI9	39S ribosomal protein L32, mitochondrial OS=Mus musculus GN=Mrpl32 PE=2 SV=1 - [RM32_MOUSE]	6.42	1	1	1	6	0.68	-0.36	0.75	0.34
A2AAE1-4	Isoform 4 of Uncharacterized protein KIAA1109 OS=Mus musculus GN=Kiaa1109 - [K1109_MOUSE]	0.67	5	1	2	2	0.19	-0.01	0.09	-0.11
Q8BLF1	Neutral cholesterol ester hydrolase 1 OS=Mus musculus GN=Nceh1 PE=1 SV=1 - [NCEH1_MOUSE]	17.65	1	4	6	31	-0.41	-0.37	-0.47	-0.30
Q8R3D1	TBC1 domain family member 13 OS=Mus musculus GN=Tbc1d13 PE=2 SV=1 - [TBC13_MOUSE]	11.75	1	4	4	6	0.09	-0.43	-0.02	-0.38
Q9WUN2	Serine/threonine-protein kinase TBK1 OS=Mus musculus GN=Tbk1 PE=1 SV=1 - [TBK1_MOUSE]	7.27	1	3	3	7	-0.09	-0.30	-0.10	-0.32
Q8R317-2	Isoform 2 of Ubiquilin-1 OS=Mus musculus GN=Ubqln1 - [UBQL1_MOUSE]	18.59	2	5	8	66	-0.13	0.22	-0.28	0.03
P20060	Beta-hexosaminidase subunit beta OS=Mus musculus GN=Hexb PE=2 SV=2 - [HEXB_MOUSE]	29.85	1	15	15	75	0.01	-0.04	-0.01	-0.01
Q922J3	CAP-Gly domain-containing linker protein 1 OS=Mus musculus GN=Clip1 PE=1 SV=1 - [CLIP1_MOUSE]	27.61	8	29	35	98	0.01	-0.03	-0.07	-0.07
E9QA05	Nuclear autoantigen Sp-100 OS=Mus musculus GN=Sp100 PE=2 SV=1 - [E9QA05_MOUSE]	19.03	9	6	6	13	-0.17	0.70	-0.01	0.50
O88939-2	Isoform 2 of Zinc finger and BTB domain-containing protein 7A OS=Mus musculus GN=Zbtb7a - [ZBT7A_MOUSE]	7.96	4	3	3	9	0.36	-0.24	0.22	-0.27
Q9R1A8	E3 ubiquitin-protein ligase RFWD2 OS=Mus musculus GN=Rfwd2 PE=1 SV=2 - [RFWD2_MOUSE]	3.55	1	2	2	2	0.43	0.16	0.56	0.29
O55128	Histone deacetylase complex subunit SAP18 OS=Mus musculus GN=Sap18 PE=1 SV=1 [SAP18_MOUSE]	43.14	5	7	7	25	-0.11	-0.12	0.03	0.03
Q9D1M4	Eukaryotic translation elongation factor 1 epsilon-1 OS=Mus musculus GN=Eef1e1 PE=2 SV=1 - [MCA3_MOUSE]	10.34	1	2	2	3	-0.06	0.71	-0.34	0.43

P38060	Hydroxymethylglutaryl-CoA lyase, mitochondria OS=Mus musculus GN=Hmgcl PE=1 SV=2 - [HMGCL_MOUSE]	31.08	4	8	8	59	-0.32	-0.22	-0.37	-0.18
Q9JII6	Alcohol dehydrogenase [NADP(+)] OS=Mus musculus GN=Akr1a1 PE=1 SV=3 - [AK1A1_MOUSE]	47.08	2	14	14	323	-0.21	-0.21	-0.13	-0.10
O08795-2	Isoform 2 of Glucosidase 2 subunit beta OS=Mus musculus GN=Prkcsh - [GLU2B_MOUSE]	29.92	2	15	15	216	-0.12	-0.12	-0.14	-0.09
A2BGI8	Peptidyl-prolyl cis-trans isomerase H (Fragment) OS=Mus musculus GN=Ppih PE=2 SV=1 - [A2BGI8_MOUSE]	34.81	4	4	6	43	-0.04	0.07	-0.17	-0.06
B2RY56	RNA-binding protein 25 OS=Mus musculus GN=Rbm25 PE=1 SV=2 - [RBM25_MOUSE]	10.86	1	6	7	29	0.54	0.37	0.41	0.48
Q8K0Z7	Translational activator of cytochrome c oxidase 1 OS=Mus musculus GN=Taco1 PE=2 SV=1 [TACO1_MOUSE]	13.95	1	4	4	12	0.07	0.12	-0.23	0.03
Q91YD9	Neural Wiskott-Aldrich syndrome protein OS=Mus musculus GN=Wasl PE=1 SV=1 - [WASL_MOUSE]	23.55	2	8	9	44	0.00	0.02	0.10	0.13
Q62077	1-phosphatidylinositol 4,5-bisphosphate phosphodiesterase gamma-1 OS=Mus musculus GN=Plcg1 PE=1 SV=2 - [PLCG1_MOUSE]	5.84	3	6	6	10	-0.10	-0.18	-0.18	-0.14
Q99LX0	Protein DJ-1 OS=Mus musculus GN=Park7 PE=1 SV=1 - [PARK7_MOUSE]	62.43	5	11	11	179	-0.06	0.02	-0.07	0.00
P62317	Small nuclear ribonucleoprotein Sm D2 OS=Mus musculus GN=Snrpd2 PE=1 SV=1 - [SMD2_MOUSE]	61.86	2	7	7	105	0.04	0.13	-0.08	0.03
Q8C0R9	Leucine-rich repeat and death domain-containing protein 1 OS=Mus musculus GN=Lrrd1 PE=2 SV=2 - [LRRD1_MOUSE]	7.03	2	5	5	8	-0.05	-0.33	-0.23	-0.05
Q9JHI5	Isovaleryl-CoA dehydrogenase, mitochondrial OS=Mus musculus GN=Ivd PE=1 SV=1 - [IVD_MOUSE]	34.20	1	15	15	109	-0.40	-0.40	-0.44	-0.39
P15947	Kallikrein-1 OS=Mus musculus GN=Klk1 PE=1 SV=3 - [KLK1_MOUSE]	48.66	5	7	7	78	-0.40	-0.41	-0.48	-0.46
P21550	Beta-enolase OS=Mus musculus GN=Eno3 PE=1 SV=3 - [ENOB_MOUSE]	54.38	9	13	22	332	-0.12	-0.16	-0.17	-0.14

Q9CXC3	Mitochondrial genome maintenance exonuclease 1 OS=Mus musculus GN=Mgme1 PE=2 SV=1 - [MGME1_MOUSE]	10.36	1	2	2	5	-0.13	-0.20	0.01	-0.07
Q9DBE9	pre-rRNA processing protein FTSJ3 OS=Mus musculus GN=Ftsj3 PE=1 SV=1 - [SPB1_MOUSE]	1.19	1	1	1	1	0.81	1.10	0.53	0.81
D3Z030	Leucine-rich repeat-containing protein 16A OS=Mus musculus GN=Lrrc16a PE=2 SV=2 - [D3Z030_MOUSE]	8.63	5	9	9	16	-0.23	0.07	-0.12	-0.10
H7BWZ1	Ubiquitin-fold modifier 1 OS=Mus musculus GN=Ufm1 PE=2 SV=1 [H7BWZ1_MOUSE]	26.32	4	2	2	3	0.15	0.08	0.16	0.11
Q9D952	Envoplakin OS=Mus musculus GN=Evpl PE=2 SV=3 - [EVPL_MOUSE]	2.26	1	4	4	5	0.14	-0.14	0.26	-0.03
Q3TMH2	Secernin-3 OS=Mus musculus GN=Scrn3 PE=1 SV=1 - [SCRN3_MOUSE]	6.70	2	3	3	9	-0.15	0.09	-0.17	0.04
P42227-2	Isoform Stat3B of Signal transducer and activator of transcription 3 OS=Mus musculus GN=Stat3 - [STAT3_MOUSE]	28.95	4	15	16	74	0.19	0.10	0.32	0.27
Q99M71-2	Isoform 2 of Mammalian ependymin-related protein 1 OS=Mus musculus GN=Epdr1 - [EPDR1_MOUSE]	12.22	2	1	1	4	-0.27	-0.21	-0.44	-0.38
F6RJV6	LanC-like protein 2 (Fragment) OS=Mus musculus GN=Lancl2 PE=2 SV=1 - [F6RJV6_MOUSE]	17.69	2	5	5	14	-0.27	-0.04	-0.04	-0.06
Q8CGY8-2	Isoform 2 of UDP-N-acetylglucosamine--peptide N-acetylglucosaminyltransferase 110 kDa subunit OS=Mus musculus GN=Ogt - [OGT1_MOUSE]	23.36	2	17	17	32	0.16	0.17	0.14	0.13
P10711	Transcription elongation factor A protein 1 OS=Mus musculus GN=Tcea1 PE=1 SV=2 [TCEA1_MOUSE]	34.88	6	11	11	53	-0.23	-0.38	-0.08	-0.20
G3V018	Heterogeneous nuclear ribonucleoprotein Q OS=Mus musculus GN=Syncrip PE=2 SV=1 - [G3V018_MOUSE]	41.84	9	17	21	278	0.29	0.38	0.25	0.41
Q9D7I9	Protein-glutamine gamma-glutamyltransferase 5 OS=Mus musculus GN=Tgm5 PE=2 SV=3 [TGM5_MOUSE]	0.83	1	1	1	1	-0.38	-0.42	-0.48	-0.52
Q91ZA3	Propionyl-CoA carboxylase alpha chain, mitochondrial OS=Mus musculus GN=Pcca PE=2 SV=2 - [PCCA_MOUSE]	41.85	5	25	26	214	-0.35	-0.32	-0.39	-0.33

B1AZP2-2	Isoform 2 of Disks large associated protein 4 OS=Mus musculus GN=Dlgap4 - [DLGP4_MOUSE]	6.07	10	5	5	10	0.25	0.24	0.19	0.39
Q3UPF5	Zinc finger CCCH-type antiviral protein 1 OS=Mus musculus GN=Zc3hav1 PE=1 SV=1 - [ZCCHV_MOUSE]	16.07	4	12	12	43	-0.09	-0.07	-0.04	0.05
D3Z7P3-2	Isoform 2 of Glutaminase kidney isoform, mitochondrial OS=Mus musculus GN=Gls - [GLSK_MOUSE]	32.67	3	3	14	58	0.10	0.15	-0.21	-0.07
Q3UC65-3	Isoform 3 of UPF0471 protein C1orf63 homolog OS=Mus musculus GN=D4Wsu53e - [CA063_MOUSE]	11.97	3	2	2	2	-0.35	0.48	-0.43	0.40
Q7TPL9	Regulating synaptic membrane exocytosis protein 1 OS=Mus musculus GN=Rims1 PE=2 SV=1 - [Q7TPL9_MOUSE]	19.63	11	4	4	8	-0.12	0.04	-0.21	-0.05
P97377-2	Isoform CDK2-alpha of Cyclin-dependent kinase 2 OS=Mus musculus GN=Cdk2 - [CDK2_MOUSE]	16.44	36	2	4	24	-0.12	-0.24	0.05	-0.07
Q60634-3	Isoform 3 of Flotillin-2 OS=Mus musculus GN=Flot2 - [FLOT2_MOUSE]	30.08	4	11	11	40	-0.13	-0.13	-0.15	-0.20
Q9EQN3	TSC22 domain family protein 4 OS=Mus musculus GN=Tsc22d4 PE=2 SV=2 - [T22D4_MOUSE]	36.69	8	9	10	23	-0.13	0.04	0.00	0.11
Q3USX5	Interleukin-1 receptor-associated kinase 4 OS=Mus musculus GN=Irak4 PE=2 SV=1 - [Q3USX5_MOUSE]	11.70	2	5	6	14	0.00	-0.10	0.11	-0.07
F6YLP9	Pleckstrin homology domain-containing family A member 1 (Fragment) OS=Mus musculus GN=Plekha1 PE=4 SV=1 - [F6YLP9_MOUSE]	10.97	4	1	1	2	0.20	-0.47	-0.09	-0.76
O35286	Putative pre-mRNA-splicing factor ATP-dependent RNA helicase DHX15 OS=Mus musculus GN=Dhx15 PE=2 SV=2 - [DHX15_MOUSE]	26.54	1	20	20	124	0.00	-0.16	0.02	-0.02
Q6P9Q6	FK506-binding protein 15 OS=Mus musculus GN=Fkbp15 PE=1 SV=2 - [FKB15_MOUSE]	9.38	5	10	10	29	-0.05	-0.19	-0.02	-0.07
Q9DCV7	Keratin, type II cytoskeletal 7 OS=Mus musculus GN=Krt7 PE=1 SV=1 - [K2C7_MOUSE]	63.89	2	19	27	438	-0.61	-0.56	-0.55	-0.57
Q9CPX7	28S ribosomal protein S16, mitochondrial OS=Mus musculus GN=Mrps16 PE=2 SV=1 - [RT16_MOUSE]	19.26	1	2	2	11	0.69	0.54	0.52	0.38

Q9WU28	Prefoldin subunit 5 OS=Mus musculus GN=Pfdn5 PE=1 SV=1 [PFD5_MOUSE]	43.51	6	6	6	35	0.13	0.29	-0.08	0.09
G3UVU2	Splicing factor 3A subunit 2 OS=Mus musculus GN=Sf3a2 PE=4 SV=1 - [G3UVU2_MOUSE]	15.05	4	5	5	18	0.27	0.35	0.26	0.27
Q8BH49	Sesquipedalian-1 OS=Mus musculus GN=Fam109a PE=2 SV=1 - [SESQ1_MOUSE]	7.14	1	1	1	1	-0.56	-1.06	-0.33	-0.83
E9Q1S3	Protein transport protein Sec23A OS=Mus musculus GN=Sec23a PE=2 SV=1 - [E9Q1S3_MOUSE]	21.74	2	12	15	149	-0.10	-0.07	-0.11	-0.10
Q9QZD8	Mitochondrial dicarboxylate carrier OS=Mus musculus GN=Slc25a10 PE=2 SV=2 - [DIC_MOUSE]	24.39	1	8	8	33	-0.22	-1.15	-0.35	-1.29
Q9WVC3	Caveolin-2 OS=Mus musculus GN=Cav2 PE=1 SV=1 - [CAV2_MOUSE]	33.95	3	3	3	9	-0.47	-0.97	-0.60	-1.09
Q91VA1	Choline transporter-like protein 4 OS=Mus musculus GN=Slc44a4 PE=1 SV=1 - [CTL4_MOUSE]	2.26	2	2	2	2	0.60	-0.23	0.61	-0.22
Q91WM1-2	Isoform 2 of Spermatid perinuclear RNA-binding protein OS=Mus musculus GN=Strbp - [STRBP_MOUSE]	9.72	4	4	6	18	-0.04	0.02	0.06	0.01
Q9JKB3-2	Isoform 2 of Y-box-binding protein 3 OS=Mus musculus GN=Ybx3 - [YBOX3_MOUSE]	35.96	3	4	10	63	-0.04	-0.12	-0.11	-0.19
Q8R2L5	28S ribosomal protein S18c, mitochondrial OS=Mus musculus GN=Mrps18c PE=2 SV=1 - [RT18C_MOUSE]	10.49	1	1	1	2	1.14	1.40	1.01	1.27
Q3UV70	[Pyruvate dehydrogenase [acetyl-transferring]]-phosphatase 1, mitochondrial OS=Mus musculus GN=Pdp1 PE=2 SV=1 - [PDP1_MOUSE]	13.94	4	6	6	9	-0.34	-0.32	-0.21	-0.27
A2RRY8	Spermatogenesis-associated serine-rich protein 1 OS=Mus musculus GN=Spats1 PE=2 SV=1 - [SPAS1_MOUSE]	6.32	2	1	1	1	-0.03	0.03	0.00	0.06
H3BJ97	Tubulointerstitial nephritis antigen-like OS=Mus musculus GN=Tinagl1 PE=2 SV=1 - [H3BJ97_MOUSE]	17.70	2	5	5	34	-0.37	-0.58	-0.44	-0.54
Q61035	Histidine--tRNA ligase, cytoplasmic OS=Mus musculus GN=Hars PE=2 SV=2 - [SYHC_MOUSE]	31.24	1	13	16	102	-0.01	-0.03	-0.02	-0.01

Q62465	Synaptic vesicle membrane protein VAT-1 homolog OS=Mus musculus GN=Vat1 PE=1 SV=3 - [VAT1_MOUSE]	39.66	1	12	12	91	-0.05	-0.04	-0.16	-0.13
F8WHY8	Metastasis-associated protein MTA1 OS=Mus musculus GN=Mta1 PE=2 SV=1 - [F8WHY8_MOUSE]	14.33	4	3	8	25	-0.17	0.01	-0.20	-0.04
F7BHM8	Monoacylglycerol lipase ABHD12 (Fragment) OS=Mus musculus GN=Abhd12 PE=4 SV=1 - [F7BHM8_MOUSE]	30.36	3	1	1	2	0.18	-0.09	0.12	-0.15
Q9D871	Carcinoembryonic antigen-related cell adhesion molecule 18 OS=Mus musculus GN=Ceacam18 PE=2 SV=1 - [CEA18_MOUSE]	4.52	1	1	1	7	0.12	0.23	-0.29	-0.18
P09925	Surfeit locus protein 1 OS=Mus musculus GN=Surf1 PE=2 SV=3 - [SURF1_MOUSE]	24.84	2	6	6	17	-0.17	-0.29	-0.11	-0.21
Q9DAX2	Lipid phosphate phosphohydrolase 2 OS=Mus musculus GN=Ppap2c PE=1 SV=1 - [LPP2_MOUSE]	8.70	3	2	2	8	0.31	-0.21	0.06	-0.28
Q9EPU0	Regulator of nonsense transcripts 1 OS=Mus musculus GN=Upf1 PE=1 SV=2 - [RENT1_MOUSE]	31.67	2	27	27	165	0.04	-0.01	0.12	-0.03
Q9D1L0	Coiled-coil-helix-coiled-coil-helix domain-containing protein 2, mitochondrial OS=Mus musculus GN=Chchd2 PE=1 SV=1 - [CHCH2_MOUSE]	30.07	3	3	3	21	0.11	0.11	0.09	0.05
Q505F5	Leucine-rich repeat-containing protein 47 OS=Mus musculus GN=Lrrc47 PE=1 SV=1 - [LRC47_MOUSE]	23.06	4	11	11	44	0.06	0.11	0.02	0.00
Q9DBJ3	Brain-specific angiogenesis inhibitor 1-associated protein 2-like protein 1 OS=Mus musculus GN=Baiap2l1 PE=1 SV=1 - [BI2L1_MOUSE]	34.44	1	14	14	56	-0.04	-0.22	-0.08	-0.20
O70318	Band 4.1-like protein 2 OS=Mus musculus GN=Epb41l2 PE=1 SV=2 - [E41L2_MOUSE]	26.11	1	19	23	95	-0.35	-0.35	-0.26	-0.30
P42232	Signal transducer and activator of transcription 5B OS=Mus musculus GN=Stat5b PE=1 SV=1 - [STA5B_MOUSE]	4.71	3	3	3	9	-0.11	0.12	-0.05	0.18
Q6P7W2	SH3KBP1-binding protein 1 OS=Mus musculus GN=Shkbp1 PE=1 SV=1 - [SHKB1_MOUSE]	3.13	1	1	1	1	0.06	-0.74	0.09	-0.72

Q9JIK9	28S ribosomal protein S34, mitochondrial OS=Mus musculus GN=Mrps34 PE=2 SV=1 - [RT34_MOUSE]	17.89	1	4	4	13	-0.13	0.09	-0.03	0.10
G3X9K3	Brefeldin A-inhibited guanine nucleotide-exchange protein 1 OS=Mus musculus GN=Arfgef1 PE=2 SV=1 - [BIG1_MOUSE]	5.20	2	6	8	15	0.43	0.58	0.34	0.40
P48774	Glutathione S-transferase Mu 5 OS=Mus musculus GN=Gstm5 PE=1 SV=1 - [GSTM5_MOUSE]	58.04	6	14	15	110	-0.03	-0.07	0.04	-0.03
Q9JK42	[Pyruvate dehydrogenase [lipoamide]] kinase isozyme 2, mitochondrial OS=Mus musculus GN=Pdk2 PE=1 SV=2 - [PDK2_MOUSE]	8.85	1	2	3	7	0.10	-0.08	-0.01	-0.14
P27048	Small nuclear ribonucleoprotein-associated protein B OS=Mus musculus GN=Snrpb PE=1 SV=1 [RSMB_MOUSE]	18.18	2	4	4	34	-0.13	-0.13	-0.11	-0.15
Q8BGS2	BolA-like protein 2 OS=Mus musculus GN=Bola2 PE=1 SV=1 [BOLA2_MOUSE]	43.02	2	3	3	12	-0.15	-0.20	-0.31	-0.32
P61161	Actin-related protein 2 OS=Mus musculus GN=Actr2 PE=1 SV=1 [ARP2_MOUSE]	37.56	1	14	15	312	0.08	0.04	0.07	0.06
Q5SNZ0-3	Isoform 3 of Girdin OS=Mus musculus GN=Ccdc88a - [GRDN_MOUSE]	1.28	4	2	2	3	0.08	-0.05	0.08	-0.04
P10076	Zinc finger protein 26 OS=Mus musculus GN=Zfp26 PE=2 SV=2 [ZFP26_MOUSE]	1.63	2	1	1	2	0.19	0.09	-0.09	-0.29
Q3TL72	NEDD8-activating enzyme E1 catalytic subunit OS=Mus musculus GN=Uba3 PE=2 SV=1 - [Q3TL72_MOUSE]	18.97	3	6	6	32	0.25	0.43	0.02	0.23
O70194	Eukaryotic translation initiation factor 3 subunit D OS=Mus musculus GN=Eif3d PE=1 SV=2 - [EIF3D_MOUSE]	26.64	1	9	9	53	-0.04	-0.08	-0.02	0.14
Q5RKN9	Capping protein (Actin filament) muscle Z-line, alpha 1 OS=Mus musculus GN=Capza1 PE=2 SV=1 - [Q5RKN9_MOUSE]	68.18	2	10	13	342	-0.18	-0.19	-0.22	-0.27
Q3V1T4-3	Isoform 3 of Prolyl 3-hydroxylase 1 OS=Mus musculus GN=Lepre1 - [P3H1_MOUSE]	9.11	5	4	4	5	0.00	0.03	0.05	0.08
Q9QYK4	Heparan-sulfate 6-O-sulfotransferase 3 OS=Mus musculus GN=Hs6st3 PE=1 SV=2 - [H6ST3_MOUSE]	3.83	2	2	2	4	-0.49	-0.67	-0.40	-0.58

H7BXB6	Protein CLEC16A OS=Mus musculus GN=Clec16a PE=2 SV=1 - [H7BXB6_MOUSE]	1.20	5	1	1	2	-0.20	-0.05	-0.23	-0.08
P56382	ATP synthase subunit epsilon, mitochondrial OS=Mus musculus GN=Atp5e PE=2 SV=2 [ATP5E_MOUSE]	28.85	1	2	2	24	-0.45	-0.55	-0.37	-0.43
Q8BHX1	HAUS augmin-like complex subunit 1 OS=Mus musculus GN=Haus1 PE=2 SV=2 - [HAUS1_MOUSE]	8.99	1	2	2	2	0.34	0.31	0.20	0.20
Q9D8E6	60S ribosomal protein L4 OS=Mus musculus GN=Rpl4 PE=1 SV=3 - [RL4_MOUSE]	37.23	1	17	17	241	0.20	0.28	0.19	0.23
E9QK48	Echinoderm microtubule-associated protein-like 2 OS=Mus musculus GN=Eml2 PE=2 SV=1 - [E9QK48_MOUSE]	20.10	5	11	13	83	-0.06	0.19	-0.05	0.14
Q8VDM6-2	Isoform 2 of Heterogeneous nuclear ribonucleoprotein U-like protein 1 OS=Mus musculus GN=Hnrnpul1 - [HNRL1_MOUSE]	22.40	3	12	12	57	-0.20	-0.20	-0.08	-0.05
G5E920	MCG114640 OS=Mus musculus GN=Gm6104 PE=4 SV=1 - [G5E920_MOUSE]	34.50	3	8	9	42	0.41	0.50	0.37	0.36
A2ADY9	Protein DDI1 homolog 2 OS=Mus musculus GN=Ddi2 PE=1 SV=1 - [DDI2_MOUSE]	40.85	3	11	11	42	-0.15	0.02	-0.11	0.01
Q9JHF5	A3 subunit of vacuolar-adenosine triphosphatase OS=Mus musculus GN=Tcirg1 PE=2 SV=1 - [Q9JHF5_MOUSE]	6.35	1	3	3	6	-0.45	-0.43	-0.47	-0.46
Q8C048	Protein Atp2b3 OS=Mus musculus GN=Atp2b3 PE=2 SV=1 - [Q8C048_MOUSE]	9.70	3	1	11	64	-0.61	-0.52	-0.76	-0.68
Q8K010	5-oxoprolinase OS=Mus musculus GN=Oplah PE=2 SV=1 [OPLA_MOUSE]	16.61	7	16	17	51	-0.34	-0.33	-0.28	-0.30
Q9WTL2	Ras-related protein Rab-25 OS=Mus musculus GN=Rab25 PE=1 SV=2 - [RAB25_MOUSE]	28.17	2	4	5	20	-0.05	-0.18	-0.07	-0.09
E9Q4Q2	Splicing factor 1 OS=Mus musculus GN=Sf1 PE=2 SV=1 - [E9Q4Q2_MOUSE]	19.16	7	6	7	70	-0.02	0.03	0.11	0.07
Q6NZD2	Sorting nexin 1 OS=Mus musculus GN=Snx1 PE=2 SV=1 - [Q6NZD2_MOUSE]	34.55	3	14	16	95	0.01	0.04	-0.04	0.01
Q60829	Protein phosphatase 1 regulatory subunit 1B OS=Mus musculus GN=Ppp1r1b PE=1 SV=2 - [PPR1B_MOUSE]	50.00	2	6	6	234	-0.75	-0.51	-0.61	-0.49

Q920H4	Muscarinic acetylcholine receptor M5 OS=Mus musculus GN=Chrm5 PE=2 SV=2 - [ACM5_MOUSE]	2.82	1	1	1	1	-0.10	-0.24	-0.05	-0.19
Q9DB29	Isoamyl acetate-hydrolyzing esterase 1 homolog OS=Mus musculus GN=Iah1 PE=2 SV=1 - [IAH1_MOUSE]	21.69	1	4	4	16	-0.03	0.45	0.11	0.40
Q9CVB6	Actin-related protein 2/3 complex subunit 2 OS=Mus musculus GN=Arpc2 PE=1 SV=3 - [ARPC2_MOUSE]	57.00	2	15	15	198	0.12	0.18	0.14	0.23
Q62018-3	Isoform 3 of RNA polymerase-associated protein CTR9 homolog OS=Mus musculus GN=Ctr9 - [CTR9_MOUSE]	6.33	13	6	6	9	0.35	0.45	0.29	0.34
Q9R0M5	Thiamin pyrophosphokinase 1 OS=Mus musculus GN=Tpk1 PE=1 SV=1 - [TPK1_MOUSE]	27.16	2	4	4	13	-0.26	-0.23	-0.17	-0.01
P70122	Ribosome maturation protein SBDS OS=Mus musculus GN=Sbds PE=1 SV=4 - [SBDS_MOUSE]	37.20	3	10	10	34	0.38	0.22	0.20	0.18
E9PUN5	Tyrosine-protein kinase transmembrane receptor OS=Mus musculus GN=Ror2 PE=2 SV=1 - [E9PUN5_MOUSE]	9.44	2	4	4	9	-0.56	-0.29	-0.42	-0.22
Q3UHB1	5'-nucleotidase domain-containing protein 3 OS=Mus musculus GN=Nt5dc3 PE=2 SV=1 - [NT5D3_MOUSE]	5.31	1	2	2	3	-0.28	-0.65	-0.10	-0.47
O08917	Flotillin-1 OS=Mus musculus GN=Flot1 PE=1 SV=1 - [FLOT1_MOUSE]	33.18	4	11	11	65	-0.26	-0.39	-0.22	-0.26
Q5GLZ0	Cytochrome P450 2C66 OS=Mus musculus GN=Cyp2c66 PE=2 SV=1 - [Q5GLZ0_MOUSE]	18.16	4	1	10	75	0.09	0.01	0.24	0.15
Q9CY97-2	Isoform 2 of RNA polymerase II subunit A C-terminal domain phosphatase SSU72 OS=Mus musculus GN=Ssu72 - [SSU72_MOUSE]	19.23	2	3	3	17	0.05	-0.10	0.12	-0.05
Q99LJ1	Tissue alpha-L-fucosidase OS=Mus musculus GN=Fuca1 PE=2 SV=1 - [FUCO_MOUSE]	33.19	1	11	11	53	-0.52	-0.56	-0.53	-0.43
Q14DL0	Ubiquilin-like protein OS=Mus musculus GN=Ubqlnl PE=1 SV=2 - [UBQLN_MOUSE]	2.62	1	1	1	1	0.47	0.01	0.22	-0.23
E9PZ36	Protein Pkhd1 OS=Mus musculus GN=Pkhd1 PE=4 SV=1 - [E9PZ36_MOUSE]	1.53	1	2	2	2	-0.24	-0.68	-0.43	-0.87
Q8BG30	Negative elongation factor A OS=Mus musculus GN=Nelfa PE=1 SV=1 - [NELFA_MOUSE]	13.21	2	5	6	15	0.03	0.19	-0.02	0.32

Q7TQF7	Amphiphysin OS=Mus musculus GN=Amph PE=1 SV=1 - [AMPH_MOUSE]	7.00	1	3	5	8	0.31	-0.22	-0.16	-0.14
P51150	Ras-related protein Rab-7a OS=Mus musculus GN=Rab7a PE=1 SV=2 - [RAB7A_MOUSE]	69.08	1	16	16	228	0.00	-0.05	0.05	0.09
O70433	Four and a half LIM domains protein 2 OS=Mus musculus GN=Fhl2 PE=1 SV=1 - [FHL2_MOUSE]	12.19	1	4	4	18	-0.09	-0.41	-0.14	-0.28
Q9JLV1	BAG family molecular chaperone regulator 3 OS=Mus musculus GN=Bag3 PE=1 SV=2 - [BAG3_MOUSE]	38.65	1	16	16	123	-0.08	-0.12	-0.10	-0.12
E9PYK8	Rho guanine nucleotide exchange factor 4 OS=Mus musculus GN=Arhgef4 PE=2 SV=2 - [E9PYK8_MOUSE]	2.16	3	3	3	6	-0.09	-0.80	0.64	-0.07
Q9CR67	Transmembrane protein 33 OS=Mus musculus GN=Tmem33 PE=2 SV=1 - [TMM33_MOUSE]	4.05	1	1	1	3	-0.20	-0.37	-0.09	-0.45
P62806	Histone H4 OS=Mus musculus GN=Hist1h4a PE=1 SV=2 - [H4_MOUSE]	61.17	1	10	10	853	-0.44	-0.39	-0.50	-0.42
Q9QZB7	Actin-related protein 10 OS=Mus musculus GN=Actr10 PE=1 SV=2 - [ARP10_MOUSE]	27.34	1	7	8	36	0.10	0.25	-0.10	0.32
Q91VH6	Protein MEMO1 OS=Mus musculus GN=Memo1 PE=1 SV=1 - [MEMO1_MOUSE]	30.64	1	6	6	18	-0.11	0.01	-0.12	-0.16
Q6P5D3-2	Isoform 2 of Putative ATP-dependent RNA helicase DHX57 OS=Mus musculus GN=Dhx57 - [DHX57_MOUSE]	4.19	5	3	4	7	-0.82	-0.68	-0.73	-0.59
D3YVJ8	Retinol dehydrogenase 13 (Fragment) OS=Mus musculus GN=Rdh13 PE=2 SV=1 - [D3YVJ8_MOUSE]	27.83	2	3	3	5	0.25	0.26	0.21	0.20
Q9Z0F0	Beta-1,3-galactosyltransferase 4 OS=Mus musculus GN=B3galt4 PE=2 SV=1 - [B3GT4_MOUSE]	3.23	1	1	1	3	-0.43	-0.40	-0.12	-0.08
H3BKQ7	Protein Ppp1r9a OS=Mus musculus GN=Ppp1r9a PE=2 SV=1 - [H3BKQ7_MOUSE]	9.11	9	3	5	20	-0.68	-0.14	-0.11	0.43
Q7TS72	Inositol-trisphosphate 3-kinase C OS=Mus musculus GN=Itpkc PE=1 SV=1 - [IP3KC_MOUSE]	4.72	1	2	2	5	-0.07	0.37	-0.14	0.11
Q3UDE2	Tubulin--tyrosine ligase like protein 12 OS=Mu musculus GN=Ttll12 PE=1 SV=1 - [TTL12_MOUSE]	14.24	2	7	7	28	-0.13	-0.30	0.02	-0.12

F7AC58	Piezo-type mechanosensitive ion channel component 1 (Fragment) OS=Mus musculus GN=Piezo1 PE=4 SV=1 - [F7AC58_MOUSE]	2.09	4	3	3	3	0.43	0.15	0.48	0.21
Q8CIN4	Serine/threonine-protein kinase PAK 2 OS=Mus musculus GN=Pak2 PE=1 SV=1 - [PAK2_MOUSE]	45.42	3	13	17	145	0.13	0.17	0.10	0.22
Q9WUH7	Semaphorin-4G OS=Mus musculus GN=Sema4g PE=1 SV=1 - [SEM4G_MOUSE]	2.99	1	2	2	8	0.33	-0.55	-0.21	-1.10
A2CF80	Protein 0610010K14Rik OS=Mus musculus GN=0610010K14Rik PE=2 SV=1 - [A2CF80_MOUSE]	29.41	11	2	2	5	0.15	0.11	0.04	0.14
Q6ZQM8	UDP-glucuronosyltransferase 1-7C OS=Mus musculus GN=Ugt1a7c PE=2 SV=1 - [UD17C_MOUSE]	28.06	15	6	13	140	-0.45	-0.71	-0.54	-0.60
Q99L13	3-hydroxyisobutyrate dehydrogenase, mitochondrial OS=Mus musculus GN=Hibadh PE=2 SV=1 - [3HIDH_MOUSE]	26.27	1	7	7	42	-0.29	-0.11	-0.48	-0.22
Q9CR16	Peptidyl-prolyl cis-trans isomerase D OS=Mus musculus GN=Ppid PE=1 SV=3 - [PPID_MOUSE]	59.46	1	21	23	164	0.07	0.16	0.12	0.16
Q9D0R9	WD repeat-containing protein 89 OS=Mus musculus GN=Wdr89 PE=2 SV=1 - [WDR89_MOUSE]	2.85	1	1	1	1	-0.12	-0.16	0.16	0.12
Q8R2Y2-2	Isoform 2 of Cell surface glycoprotein MUC18 OS=Mus musculus GN=Mcam - [MUC18_MOUSE]	30.36	3	14	14	94	-0.46	-0.41	-0.43	-0.35
H7BWZ3	Actin-related protein 2/3 complex subunit 3 OS=Mus musculus GN=Arpc3 PE=2 SV=1 - [H7BWZ3_MOUSE]	23.53	4	5	5	58	0.11	0.31	0.15	0.16
F7AMG0	Large subunit GTPase 1 homolog (Fragment) OS=Mus musculus GN=Lsg1 PE=2 SV=1 - [F7AMG0_MOUSE]	9.75	3	2	2	3	0.09	-0.33	-0.02	-0.45
E9PZ87	Protein Pappa2 OS=Mus musculus GN=Pappa2 PE=4 SV=1 - [E9PZ87_MOUSE]	2.74	1	2	2	4	0.75	0.84	0.42	0.50
P35980	60S ribosomal protein L18 OS=Mus musculus GN=Rpl18 PE=2 SV=3 [RL18_MOUSE]	27.13	5	4	4	100	-0.28	-0.35	-0.14	-0.23
Q91WL0	Epidermal growth factor receptor kinase substrate 8-like protein 3 OS=Mus musculus GN=Eps8l3 PE=2 SV=1 - [ES8L3_MOUSE]	17.33	1	8	8	41	-0.09	0.13	-0.02	0.16
O35927-2	Isoform 2 of Catenin delta-2 OS=Mus musculus GN=Ctnnd2 - [CTND2_MOUSE]	2.62	3	2	2	4	0.40	-0.19	0.19	-0.41

O55074	A-kinase anchor protein 7 isoform alpha OS=Mus musculus GN=Akap7 PE=1 SV=4 - [AKA7A_MOUSE]	14.81	2	1	1	1	-0.56	-0.45	-0.57	-0.46
Q80WJ7	Protein LYRIC OS=Mus musculus GN=Mtdh PE=1 SV=1 - [LYRIC_MOUSE]	30.40	6	14	14	45	0.03	0.09	0.01	0.10
Q04750	DNA topoisomerase 1 OS=Mus musculus GN=Top1 PE=1 SV=2 - [TOP1_MOUSE]	29.20	2	22	22	77	0.29	0.37	0.26	0.31
Q8R2H9	Phosphoethanolamine/phosphocholine phosphatase OS=Mus musculus GN=Phospho1 PE=2 SV=1 - [PHOP1_MOUSE]	10.86	3	3	3	4	-0.64	-0.70	-0.58	-0.64
O09172	Glutamate--cysteine ligase regulatory subunit OS=Mus musculus GN=Gclm PE=2 SV=1 - [GSH0_MOUSE]	40.15	3	9	10	40	-0.05	0.04	0.02	0.05
Q9EQ06	Estradiol 17-beta-dehydrogenase 11 OS=Mus musculus GN=Hsd17b11 PE=2 SV=1 - [DHB11_MOUSE]	25.84	2	5	6	46	-0.28	-0.25	-0.25	-0.17
Q8R4R6	Nucleoporin NUP53 OS=Mus musculus GN=Nup35 PE=1 SV=2 - [NUP53_MOUSE]	23.08	2	5	5	13	-0.24	-0.26	-0.01	0.06
Q8BYW1-2	Isoform 2 of Rho GTPase-activating protein 25 OS=Mus musculus GN=Arhgap25 - [RHG25_MOUSE]	12.16	3	3	4	5	-0.60	-0.81	-0.04	-0.24
Q91VL8	Telomeric repeat-binding factor 2-interacting protein 1 OS=Mus musculus GN=Terf2ip PE=1 SV=1 - [TE2IP_MOUSE]	23.66	1	6	6	10	-0.05	-0.40	-0.04	-0.17
Q3THK7	GMP synthase [glutamine-hydrolyzing] OS=Mus musculus GN=Gmps PE=1 SV=2 [GUAA_MOUSE]	29.00	1	16	16	87	0.04	0.01	0.16	0.02
A1L341	Dual-specificity tyrosine-phosphorylation-regulated kinase 1A OS=Mus musculus GN=Dyrk1a PE=2 SV=1 - [A1L341_MOUSE]	4.51	2	2	2	6	0.36	0.16	0.34	0.19
P55258	Ras-related protein Rab-8A OS=Mus musculus GN=Rab8a PE=1 SV=2 - [RAB8A_MOUSE]	54.11	13	5	12	91	-0.24	-0.05	-0.31	-0.10
Q69ZQ2	Pre-mRNA-splicing factor ISY1 homolog OS=Mus musculus GN=Isy1 PE=1 SV=2 - [ISY1_MOUSE]	23.16	1	6	6	31	0.11	0.07	-0.11	-0.10
Q05D44	Eukaryotic translation initiation factor 5B OS=Mus musculus GN=Eif5b PE=1 SV=2 - [IF2P_MOUSE]	20.23	1	19	21	98	0.24	0.38	0.22	0.31

Q6PFX8	N-acetylaspartyl-glutamate synthetase A OS=Mus musculus GN=Rimkla PE=1 SV=1 - [RIMKA_MOUSE]	6.84	1	2	2	8	-0.13	-0.05	-0.46	-0.03
G3X940	Histone acetyltransferase KAT6A OS=Mus musculus GN=Kat6a PE=4 SV=1 - [G3X940_MOUSE]	0.85	2	1	1	2	-1.10	-2.96	-0.71	-2.57
O70475	UDP-glucose 6-dehydrogenase OS=Mus musculus GN=Ugdh PE=1 SV=1 - [UGDH_MOUSE]	60.24	4	30	30	594	-0.76	-0.64	-0.76	-0.64
Q5SSW2	Proteasome activator complex subunit 4 OS=Mus musculus GN=Psme4 PE=1 SV=1 - [PSME4_MOUSE]	3.96	1	4	5	15	-0.18	-0.28	-0.30	-0.19
Q8BYQ0	Arylacetamide deacetylase-like 1, isoform CRA_c OS=Mus musculus GN=Nceh1 PE=2 SV=1 - [Q8BYQ0_MOUSE]	27.14	1	1	3	9	0.49	0.75	0.38	0.64
Q8K023-2	Isoform 2 of Aldo-keto reductase family 1 member C18 OS=Mus musculus GN=Akr1c18 [AKC1H_MOUSE]	18.18	5	2	6	9	-0.38	0.46	-0.64	0.20
H3BKJ1	Protein Gm20683 (Fragment) OS=Mus musculus GN=Gm20683 PE=4 SV=1 - [H3BKJ1_MOUSE]	6.34	2	2	2	5	0.55	0.03	0.09	-0.37
Q8CJ53-4	Isoform 4 of Cdc42-interacting protein 4 OS=Mus musculus GN=Trip10 - [CIP4_MOUSE]	30.04	4	13	13	52	-0.01	-0.29	0.06	-0.17
Q99LB2	Dehydrogenase/reductase SDR family member 4 OS=Mus musculus GN=Dhrs4 PE=2 SV=3 [DHRS4_MOUSE]	20.07	1	7	7	37	-0.17	-0.35	-0.13	-0.40
Q6P069-2	Isoform 2 of Sorcin OS=Mus musculus GN=Sri - [SORCN_MOUSE]	55.19	3	10	10	230	0.07	0.11	-0.01	-0.01
Q9CZ96-2	Isoform 2 of Zinc finger CCHC-type and RNA-binding motif-containing protein 1 OS=Mus musculus GN=Zcrb1 - [ZCRB1_MOUSE]	6.94	2	1	1	2	-0.17	-0.22	-0.18	-0.24
E9QPX6	Protein AW554918 OS=Mus musculus GN=AW554918 PE=2 SV=1 - [E9QPX6_MOUSE]	4.49	6	3	3	12	-0.14	0.04	0.02	0.16
Q9R190	Metastasis-associated protein MTA2 OS=Mus musculus GN=Mta2 PE=1 SV=1 - [MTA2_MOUSE]	32.63	2	16	20	71	0.06	0.08	0.22	0.17
Q9D1P4	Cysteine and histidine-rich domain-containing protein 1 OS=Mus musculus GN=Chordc1 PE=1 SV=1 - [CHRD1_MOUSE]	23.87	1	5	5	21	0.25	0.39	0.11	0.33

Q8VC57	BTB/POZ domain-containing protein KCTD5 OS=Mus musculus GN=Kctd5 PE=2 SV=1 - [KCTD5_MOUSE]	5.56	1	1	1	2	0.00	0.14	0.11	0.25
Q99J62	Replication factor C subunit 4 OS=Mus musculus GN=Rfc4 PE=1 SV=1 - [RFC4_MOUSE]	9.34	2	3	3	5	-0.14	-0.41	0.05	-0.22
P97807-2	Isoform Cytoplasmic of Fumarate hydratase, mitochondrial OS=Mus musculus GN=Fh - [FUMH_MOUSE]	57.17	3	20	21	470	-0.11	-0.09	-0.14	-0.10
Q8VE97	Serine/arginine-rich splicing factor 4 OS=Mus musculus GN=Srsf4 PE=2 SV=1 - [SRSF4_MOUSE]	16.97	3	5	8	51	0.15	0.02	-0.02	0.09
P58137	Acyl-coenzyme A thioesterase 8 OS=Mus musculus GN=Acot8 PE=1 SV=1 - [ACOT8_MOUSE]	21.25	3	5	5	15	0.42	-0.40	0.26	-0.48
Q8BK72	28S ribosomal protein S27, mitochondrial OS=Mus musculus GN=Mrps27 PE=2 SV=2 - [RT27_MOUSE]	26.99	1	9	9	31	0.03	0.05	0.06	0.12
Q6NS46	Protein RRP5 homolog OS=Mus musculus GN=Pdcd11 PE=2 SV=2 - [RRP5_MOUSE]	2.31	1	2	2	5	0.18	0.60	-0.07	0.36
Q14C51	Pentatricopeptide repeat domain-containing protein 3, mitochondrial OS=Mus musculus GN=Ptcd3 PE=2 SV=2 [PTCD3_MOUSE]	16.35	1	8	10	29	-0.02	0.08	0.06	0.01
Q924T2	28S ribosomal protein S2, mitochondrial OS=Mus musculus GN=Mrps2 PE=2 SV=1 - [RT02_MOUSE]	20.96	1	4	5	12	-0.16	-0.16	-0.05	0.09
H7BXB9	Protein 1700037H04Rik (Fragment) OS=Mus musculus GN=1700037H04Rik PE=2 SV=1 - [H7BXB9_MOUSE]	22.70	3	3	3	7	-0.44	-0.31	-0.07	-0.26
Q9DCT5	Stromal cell-derived factor 2 OS=Mus musculus GN=Sdf2 PE=2 SV=1 - [SDF2_MOUSE]	32.70	1	4	4	46	-0.22	-0.19	-0.09	-0.09
Q9D1I5	Methylmalonyl-CoA epimerase, mitochondrial OS=Mus musculus GN=Mcee PE=2 SV=1 - [MCEE_MOUSE]	17.98	1	2	2	18	-0.73	-0.66	-0.44	-0.58
Q08501-3	Isoform PRL-R2 of Prolactin receptor OS=Mus musculus GN=Prlr - [PRLR_MOUSE]	2.05	4	1	1	1	-0.34	-0.46	-0.49	-0.61
Q9WUR0	Protein Tinag OS=Mus musculus GN=Tinag PE=2 SV=1 - [Q9WUR0_MOUSE]	5.26	1	2	2	4	-0.51	-0.41	-0.61	-0.40

Q6PGH2	Hematological and neurological expressed 1-like protein OS=Mus musculus GN=Hn1l PE=2 SV=1 - [HN1L_MOUSE]	43.68	2	6	6	24	-0.03	-0.22	-0.05	-0.13
P31230	Aminoacyl tRNA synthase complex-interacting multifunctional protein 1 OS=Mus musculus GN=Aimp1 PE=1 SV=2 - [AIMP1_MOUSE]	54.84	7	11	12	115	0.25	0.28	0.19	0.31
G3X973	Stabilin 1, isoform CRA_a OS=Mus musculus GN=Stab1 PE=4 SV=1 - [G3X973_MOUSE]	3.73	3	8	8	19	-0.25	0.02	-0.28	-0.01
P84104-2	Isoform Short of Serine/arginine-rich splicing factor 3 OS=Mus musculus GN=Srsf3 - [SRSF3_MOUSE]	49.19	3	6	7	147	0.29	0.42	0.27	0.40
Q8R0S1	Cyclic AMP-dependent transcription factor ATF-7 OS=Mus musculus GN=Atf7 PE=1 SV=1 - [ATF7_MOUSE]	8.47	1	2	2	4	0.02	0.12	-0.05	-0.14
Q75UR0-2	Isoform 2 of Anoctamin 5 OS=Mus musculus GN=Ano5 - [ANO5_MOUSE]	3.63	3	1	1	1	-0.31	-0.94	0.12	-0.52
Q68FG3	Protein SPT2 homolog OS=Mus musculus GN=Spty2d1 PE=2 SV=1 - [SPT2_MOUSE]	4.40	1	3	3	5	0.08	0.49	-0.10	0.22
Q9CZD3	Glycine--tRNA ligase OS=Mus musculus GN=Gars PE=1 SV=1 - [SYG_MOUSE]	29.08	1	19	19	167	0.26	0.24	0.16	0.16
Q8BWZ3	N-alpha-acetyltransferase 25, NatB auxiliary subunit OS=Mus musculus GN=Naa25 PE=1 SV=1 - [NAA25_MOUSE]	17.59	4	13	14	32	0.21	0.35	-0.01	0.12
Q6ZPZ3	Zinc finger CCCH domain-containing protein 4 OS=Mus musculus GN=Zc3h4 PE=1 SV=2 - [ZC3H4_MOUSE]	8.97	3	6	6	15	-0.04	0.18	-0.06	0.10
Q8BU04	Putative E3 ubiquitin-protein ligase UBR7 OS=Mus musculus GN=Ubr7 PE=2 SV=1 - [UBR7_MOUSE]	9.18	1	4	4	4	-0.29	-0.52	-0.01	-0.22
D6RIQ7	Transcription initiation factor IIA subunit 2 OS=Mus musculus GN=Gtf2a2 PE=2 SV=1 - [D6RIQ7_MOUSE]	14.52	6	1	1	3	-0.05	0.26	-0.13	0.03
Q8BVU5	ADP-ribose pyrophosphatase, mitochondrial OS=Mus musculus GN=Nudt9 PE=2 SV=1 - [NUDT9_MOUSE]	16.29	5	5	5	12	-0.16	0.01	0.13	0.22

Q9D0Q7	39S ribosomal protein L45, mitochondrial OS=Mus musculus GN=Mrpl45 PE=2 SV=1 - [RM45_MOUSE]	17.97	2	5	5	33	-0.03	0.13	-0.08	0.04
Q6PFX9	Tankyrase-1 OS=Mus musculus GN=Tnks PE=1 SV=1 - [TNKS1_MOUSE]	3.48	2	2	3	6	0.29	0.15	0.33	0.19
D3YUF9	UDP-glucuronic acid decarboxylase 1 (Fragment) OS=Mus musculus GN=Uxs1 PE=2 SV=1 - [D3YUF9_MOUSE]	7.09	2	1	1	2	0.26	0.20	0.32	0.26
Q9WUB7	Chloride channel protein ClC-Ka OS=Mus musculus GN=Clcnka PE=1 SV=2 - [CLCKA_MOUSE]	14.41	2	4	5	7	-0.68	-0.57	-0.80	-0.69
P80317	T-complex protein 1 subunit zeta OS=Mus musculus GN=Cct6a PE=1 SV=3 - [TCPZ_MOUSE]	48.96	4	24	24	222	0.22	0.22	0.16	0.14
Q8R5F3	O-acetyl-ADP-ribose deacetylase 1 OS=Mus musculus GN=Oard1 PE=2 SV=2 - [OARD1_MOUSE]	13.16	1	2	2	2	0.49	0.46	0.30	0.26
A2AJK8	Tetratricopeptide repeat protein 1 OS=Mus musculus GN=Ttc1 PE=2 SV=1 - [A2AJK8_MOUSE]	10.00	2	2	2	7	0.15	0.39	0.16	0.26
O89050	Muskelin OS=Mus musculus GN=Mkln1 PE=2 SV=1 - [MKLN1_MOUSE]	6.39	1	3	3	6	0.22	0.25	0.26	0.24
P54754	Ephrin type-B receptor 3 OS=Mus musculus GN=Ephb3 PE=1 SV=2 - [EPHB3_MOUSE]	9.26	8	4	7	21	0.45	-0.20	0.35	-0.14
Q99N94	39S ribosomal protein L9, mitochondrial OS=Mus musculus GN=Mrpl9 PE=2 SV=2 [RM09_MOUSE]	18.11	2	5	5	11	-0.10	-0.45	-0.04	-0.39
Q99LB6-2	Isoform 2 of Methionine adenosyltransferase 2 subunit beta OS=Mus musculus GN=Mat2b - [MAT2B_MOUSE]	41.49	3	12	12	50	-0.07	-0.20	-0.23	-0.20
P03966	N-myc proto-oncogene protein OS=Mus musculus GN=Mycn PE=2 SV=2 - [MYCN_MOUSE]	1.73	1	1	1	5	0.57	0.79	0.47	0.68
Q9D0R2	Threonine--tRNA ligase, cytoplasmic OS=Mus musculus GN=Tars PE=1 SV=2 - [SYTC_MOUSE]	41.97	2	27	28	256	0.08	0.08	0.08	0.12
Q6AXE3	ATP-binding cassette sub-family A member 3 OS=Mus musculus GN=Abca3 PE=2 SV=1 - [Q6AXE3_MOUSE]	2.69	2	3	3	3	0.35	0.49	-0.26	0.50
E9PZD2	Protein Micall2 OS=Mus musculus GN=Micall2 PE=2 SV=1 - [E9PZD2_MOUSE]	7.99	2	6	6	9	-0.35	-0.20	-0.39	-0.25

Q9Z204-2	Isoform C1 of Heterogeneous nuclear ribonucleoproteins C1/C2 OS=Mus musculus GN=Hnrnpc - [HNRPC_MOUSE]	44.33	7	1	14	152	-0.06	-0.19	0.24	-0.21
D3Z3C3	Heparanase OS=Mus musculus GN=Hpse PE=2 SV=1 - [D3Z3C3_MOUSE]	8.28	2	3	3	3	-1.15	-0.90	-0.42	-0.17
Q9EP52	Twisted gastrulation protein homolog 1 OS=Mus musculus GN=Twsg1 PE=1 SV=1 - [TWSG1_MOUSE]	4.05	1	1	1	2	-0.15	-0.08	-0.01	0.06
Q921K2	Poly (ADP-ribose) polymerase family, member 1 OS=Mus musculus GN=Parp1 PE=2 SV=1 - [Q921K2_MOUSE]	22.98	3	21	21	83	0.19	0.15	0.06	0.13
Q8BGX2	Uncharacterized protein C19orf52 homolog OS=Mus musculus PE=2 SV=1 - [CS052_MOUSE]	6.02	1	1	1	3	-0.20	-0.57	-0.05	-0.57
E0CY91	Ubiquitin-like protein 5 OS=Mus musculus GN=Ubl5 PE=2 SV=1 - [E0CY91_MOUSE]	61.70	7	3	3	18	0.21	0.01	0.11	-0.06
Q8BQ47	Protein canopy homolog 4 OS=Mus musculus GN=Cnpy4 PE=1 SV=1 - [CNPY4_MOUSE]	34.29	2	8	8	41	-0.49	-0.45	-0.30	-0.29
Q9D7B7	Probable glutathione peroxidase 8 OS=Mus musculus GN=Gpx8 PE=2 SV=1 - [GPX8_MOUSE]	9.09	1	2	2	3	-0.01	-0.51	-0.04	-0.60
P52875	Transmembrane protein 165 OS=Mus musculus GN=Tmem165 PE=2 SV=2 - [TM165_MOUSE]	7.74	1	1	1	8	-0.87	-1.84	1.00	-0.36
Q8BIP0	Aspartate--tRNA ligase mitochondrial OS=Mus musculus GN=Dars2 PE=2 SV=1 - [SYDM_MOUSE]	8.73	1	4	4	18	0.07	-0.40	-0.06	-0.61
Q9DBR0	A-kinase anchor protein 8 OS=Mus musculus GN=Akap8 PE=1 SV=1 - [AKAP8_MOUSE]	9.61	1	6	7	16	0.09	0.18	0.04	0.16
Q8R016	Bleomycin hydrolase OS=Mus musculus GN=Blmh PE=2 SV=1 - [BLMH_MOUSE]	38.02	4	6	14	110	0.16	0.16	0.09	0.18
E9Q0I4	SCO-spondin OS=Mus musculus GN=Sspo PE=4 SV=2 - [E9Q0I4_MOUSE]	0.52	2	2	2	2	1.01	0.94	0.54	-0.21
Q9Z2G6	Protein sel-1 homolog 1 OS=Mus musculus GN=Sel1l PE=2 SV=2 - [SE1L1_MOUSE]	12.41	3	6	6	16	0.23	0.04	0.18	0.14
Q6GQV7	Erythroid differentiation related factor 1 OS=Mus musculus GN=Edrf1 PE=2 SV=1 - [EDRF1_MOUSE]	5.08	3	3	3	6	-2.06	-0.99	-1.20	-0.14
Q3UJB0	Protein Sf3b2 OS=Mus musculus GN=Sf3b2 PE=2 SV=1 - [Q3UJB0_MOUSE]	27.45	1	21	21	103	0.23	0.28	0.24	0.34

Q3TVF4	Geranylgeranyl transferase type-2 subunit beta OS=Mus musculus GN=Rabggtb PE=2 SV=1 - [Q3TVF4_MOUSE]	6.65	2	2	2	5	0.06	0.15	0.09	0.18
Q9D8U2	Transmembrane protein 41A OS=Mus musculus GN=Tmem41a PE=2 SV=1 - [TM41A_MOUSE]	2.65	1	1	1	1	0.48	-0.26	0.52	-0.22
Q9JLJ0	Lipopolysaccharide-induced tumor necrosis factor-alpha factor homolog OS=Mus musculus GN=Litaf PE=1 SV=1 - [LITAF_MOUSE]	4.35	1	1	1	9	0.10	0.34	0.12	0.28
P62814	V-type proton ATPase subunit B, brain isoform OS=Mus musculus GN=Atp6v1b2 PE=1 SV=1 - [VATB2_MOUSE]	38.94	2	16	16	132	-0.20	-0.19	-0.19	-0.15
Q9DBJ1	Phosphoglycerate mutase 1 OS=Mus musculus GN=Pgam1 PE=1 SV=3 - [PGAM1_MOUSE]	70.47	2	15	15	763	0.07	0.14	0.04	0.07
Q6PGG2-2	Isoform 2 of GEM-interacting protein OS=Mus musculus GN=Gmip - [GMIP_MOUSE]	4.17	2	2	2	6	-0.30	-0.43	-0.22	-0.38
Q78ZA7	Nucleosome assembly protein 1-like 4 OS=Mus musculus GN=Nap1l4 PE=1 SV=1 - [NP1L4_MOUSE]	40.27	1	11	12	96	0.32	0.26	0.24	0.21
Q8VE65	Transcription initiation factor TFIID subunit 12 OS=Mus musculus GN=Taf12 PE=1 SV=1 [TAF12_MOUSE]	11.80	2	2	2	4	-0.22	0.42	-0.33	0.31
Q9D7M8	DNA-directed RNA polymerase II subunit RPB4 OS=Mus musculus GN=Polr2d PE=2 SV=2 - [RPB4_MOUSE]	28.17	1	3	3	7	-0.47	-0.28	-0.46	-0.25
O70570	Polymeric immunoglobulin receptor OS=Mus musculus GN=Pigr PE=1 SV=1 - [PIGR_MOUSE]	44.88	3	23	23	290	0.18	0.19	0.15	0.18
D3YVX2	Coiled-coil domain-containing protein 7 (Fragment) OS=Mus musculus GN=Ccdc7 PE=2 SV=1 - [D3YVX2_MOUSE]	4.89	3	2	2	4	0.52	0.58	0.29	0.42
J3QNY6	Bile salt export pump OS=Mus musculus GN=Abcb11 PE=3 SV=1 - [J3QNY6_MOUSE]	0.53	2	1	1	2	0.43	0.48	0.02	0.06
O35841	Apoptosis inhibitor 5 OS=Mus musculus GN=Api5 PE=2 SV=2 - [API5_MOUSE]	28.77	1	13	13	108	0.04	-0.02	0.06	-0.02
Q99MR3	Solute carrier family 12 member 9 OS=Mus musculus GN=Slc12a9 PE=1 SV=2 - [S12A9_MOUSE]	1.42	1	2	2	2	0.33	0.45	0.42	0.54

A2ARZ7	RAB22A, member RAS oncogene family, isoform CRA_c OS=Mus musculus GN=Rab22a PE=4 SV=1 - [A2ARZ7_MOUSE]	19.25	3	1	3	9	-0.04	-0.96	-0.12	-1.04
Q9CZX0	Elongator complex protein 3 OS=Mus musculus GN=Elp3 PE=2 SV=1 - [ELP3_MOUSE]	10.42	2	4	4	8	-0.05	0.10	0.03	0.09
D3YUU6	Protein Crnn OS=Mus musculus GN=Crnn PE=4 SV=1 - [D3YUU6_MOUSE]	1.15	1	1	1	1	0.25	0.65	0.16	0.56
F6XC25	Coiled-coil and C2 domain-containing protein 1B (Fragment) OS=Mus musculus GN=Cc2d1b PE=4 SV=1 - [F6XC25_MOUSE]	13.25	2	8	9	21	-0.09	0.11	0.06	0.22
P43241	Homeobox protein CDX-2 OS=Mus musculus GN=Cdx2 PE=1 SV=1 - [CDX2_MOUSE]	20.90	1	5	5	26	0.14	0.15	0.15	0.18
P62743	AP-2 complex subunit sigma OS=Mus musculus GN=Ap2s1 PE=1 SV=1 - [AP2S1_MOUSE]	26.76	1	3	4	9	-0.14	-0.05	-0.16	-0.15
Q9CPQ3	Mitochondrial import receptor subunit TOM22 homolog OS=Mus musculus GN=Tomm22 PE=2 SV=3 - [TOM22_MOUSE]	8.45	1	1	1	20	0.07	-0.06	0.09	0.03
Q8C181-3	Isoform 3 of Muscleblind-like protein 2 OS=Mus musculus GN=Mbnl2 - [MBNL2_MOUSE]	15.32	7	4	4	40	-0.05	-0.14	-0.02	-0.08
D3YX13	Inhibitor of growth protein 3 (Fragment) OS=Mus musculus GN=Ing3 PE=2 SV=1 - [D3YX13_MOUSE]	5.30	3	1	1	4	0.31	0.27	0.32	0.28
A2AF31	Protein Tmsb15b2 OS=Mus musculus GN=Tmsb15b2 PE=2 SV=1 - [A2AF31_MOUSE]	48.89	3	2	2	10	0.19	0.16	0.02	0.01
Q9CQA3	Succinate dehydrogenase [ubiquinone] iron-sulfur subunit, mitochondrial OS=Mus musculus GN=Sdhb PE=1 SV=1 - [DHSB_MOUSE]	40.43	1	14	14	229	-0.25	-0.22	-0.30	-0.29
P60122	RuvB-like 1 OS=Mus musculus GN=Ruvbl1 PE=1 SV=1 - [RUVB1_MOUSE]	24.78	2	8	8	61	0.27	0.26	0.00	0.13
Q8C2Q8	ATP synthase subunit gamma OS=Mus musculus GN=Atp5c1 PE=2 SV=1 - [Q8C2Q8_MOUSE]	43.80	7	13	14	161	-0.23	-0.17	-0.29	-0.18
Q9CQU1	Microfibrillar-associated protein 1 OS=Mus musculus GN=Mfap1 PE=1 SV=1 - [MFAP1_MOUSE]	13.90	1	4	4	16	0.12	0.18	0.38	0.38

Q9CWK3	CD2 antigen cytoplasmic tail-binding protein 2 OS=Mus musculus GN=Cd2bp2 PE=1 SV=1 - [CD2B2_MOUSE]	34.80	1	7	7	22	-0.05	0.00	-0.06	-0.02
P03958	Adenosine deaminase OS=Mus musculus GN=Ada PE=1 SV=3 - [ADA_MOUSE]	29.83	1	7	7	38	0.19	0.09	0.08	0.14
Q923G2	DNA-directed RNA polymerases I, II, and III subunit RPABC3 OS=Mus musculus GN=Polr2h PE=2 SV=3 - [RPAB3_MOUSE]	44.67	1	4	4	25	-0.17	-0.22	-0.05	-0.05
O08784	Treacle protein OS=Mus musculus GN=Tcof1 PE=1 SV=1 [TCOF_MOUSE]	16.14	5	19	19	64	0.15	0.21	0.15	0.22
P62075	Mitochondrial import inner membrane translocase subunit Tim13 OS=Mus musculus GN=Timm13 PE=1 SV=1 - [TIM13_MOUSE]	52.63	1	4	4	44	0.14	0.16	0.03	0.14
Q8JZN5	Acyl-CoA dehydrogenase family member 9, mitochondrial OS=Mus musculus GN=Acad9 PE=2 SV=2 - [ACAD9_MOUSE]	35.04	3	18	18	81	-0.19	-0.16	-0.33	-0.23
Q8BH73-2	Isoform 2 of Glutaminyl peptide cyclotransferase like protein OS=Mus musculus GN=Qpctl - [QPCTL_MOUSE]	11.78	2	2	2	4	0.14	-0.24	0.35	-0.03
Q80WU2	Rhophilin-1 OS=Mus musculus GN=Rhpn1 PE=2 SV=1 - [Q80WU2_MOUSE]	2.00	3	1	1	1	-0.28	-3.76	-0.21	-3.70
Q64674	Spermidine synthase OS=Mus musculus GN=Srm PE=2 SV=1 - [SPEE_MOUSE]	22.19	1	7	7	34	0.22	0.46	0.27	0.39
Q8BFS6-4	Isoform 4 of Calcineurin like phosphoesterase domain containing protein 1 OS=Mus musculus GN=Cpped1 - [CPPED_MOUSE]	20.30	6	4	4	28	-0.01	-0.05	-0.07	-0.08
Q8VHI3	GDP-fucose protein O-fucosyltransferase 2 OS=Mus musculus GN=Pofut2 PE=1 SV=1 - [OFUT2_MOUSE]	13.75	1	5	5	17	-0.07	-0.27	-0.10	-0.21
B7ZC85	Serine/threonine-protein kinase tousled-like 2 OS=Mus musculus GN=Tlk2 PE=2 SV=1 - [B7ZC85_MOUSE]	5.21	18	2	3	4	0.15	0.29	0.05	0.18
P55284	Cadherin-5 OS=Mus musculus GN=Cdh5 PE=1 SV=2 - [CADH5_MOUSE]	10.97	1	7	7	13	-0.48	-0.46	-0.27	-0.23
Q61584-2	Isoform A of Fragile X mental retardation syndrome-related protein 1 OS=Mus musculus GN=Fxr1 - [FXR1_MOUSE]	22.63	7	8	10	30	-0.21	-0.61	-0.09	-0.50

O35640	Annexin A8 OS=Mus musculus GN=Anxa8 PE=2 SV=2 - [ANXA8_MOUSE]	25.99	2	4	5	24	0.83	0.92	0.66	1.11
Q9CZX8	40S ribosomal protein S19 OS=Mus musculus GN=Rps19 PE=1 SV=3 - [RS19_MOUSE]	50.34	5	10	10	208	0.20	0.19	0.22	0.21
D6RGL4	Ankyrin repeat domain-containing protein 16 OS=Mus musculus GN=Ankrd16 PE=2 SV=1 - [D6RGL4_MOUSE]	4.82	3	1	1	1	0.08	0.09	0.02	0.03
Q8BJL1	F-box only protein 30 OS=Mus musculus GN=Fbxo30 PE=2 SV=2 - [FBX30_MOUSE]	1.88	1	1	2	4	-0.09	0.04	-0.07	0.06
P43275	Histone H1.1 OS=Mus musculus GN=Hist1h1a PE=1 SV=2 - [H11_MOUSE]	30.52	1	5	7	137	0.04	0.18	0.04	0.17
P40336	Vacuolar protein sorting associated protein 26A OS=Mus musculus GN=Vps26a PE=2 SV=1 - [VP26A_MOUSE]	45.87	2	13	14	91	-0.28	-0.22	-0.26	-0.22
A2BGN7	Protein MANBAL OS=Mus musculus GN=Manbal PE=4 SV=1 - [A2BGN7_MOUSE]	9.41	1	1	1	1	0.30	-0.32	0.31	-0.31
A8WHU2	Coiled-coil domain-containing protein 30 (Fragment) OS=Mus musculus GN=Ccdc30 PE=2 SV=1 - [A8WHU2_MOUSE]	10.06	4	1	2	9	-0.04	0.25	-0.03	0.26
Q9CQM5	Thioredoxin domain-containing protein 17 OS=Mus musculus GN=Txndc17 PE=1 SV=1 - [TXD17_MOUSE]	7.32	1	1	1	8	-0.01	-0.09	0.10	0.04
Q99KJ8	Dynactin subunit 2 OS=Mus musculus GN=Dctn2 PE=1 SV=3 [DCTN2_MOUSE]	55.47	1	21	21	227	-0.06	-0.10	-0.09	-0.13
P51949	CDK-activating kinase assembly factor MAT1 OS=Mus musculus GN=Mnat1 PE=2 SV=2 - [MAT1_MOUSE]	5.18	1	1	1	5	-0.03	-0.23	0.14	-0.06
Q8BTX9	Inactive hydroxysteroid dehydrogenase-like protein 1 OS=Mus musculus GN=Hsdl1 PE=2 SV=1 - [HSDL1_MOUSE]	14.24	1	3	4	6	-0.67	-0.64	-0.68	-0.65
Q9Z2N8	Actin-like protein 6A OS=Mus musculus GN=Actl6a PE=1 SV=2 - [ACL6A_MOUSE]	41.72	5	12	12	60	-0.09	-0.14	-0.14	-0.18
P48025	Tyrosine-protein kinase SYK OS=Mus musculus GN=Syk PE=1 SV=2 - [KSYK_MOUSE]	13.35	2	5	5	21	0.20	0.22	0.13	0.09
Q61249	Immunoglobulin-binding protein 1 OS=Mus musculus GN=Igbp1 PE=1 SV=1 [IGBP1_MOUSE]	17.65	1	7	7	21	0.11	-0.09	0.09	0.10

Q91V41	Ras-related protein Rab-14 OS=Mus musculus GN=Rab14 PE=1 SV=3 - [RAB14_MOUSE]	67.44	12	11	12	120	0.02	0.14	0.04	0.09
Q8C176	Transcription initiation factor TFIID subunit 2 OS=Mus musculus GN=Taf2 PE=2 SV=2 - [TAF2_MOUSE]	1.63	2	2	2	2	-0.05	-0.26	0.09	-0.12
B8ZXI1-2	Isoform 2 of Queuine tRNA-ribosyltransferase subunit QTRTD1 OS=Mus musculus GN=Qtrtd1 - [QTRD1_MOUSE]	9.30	2	2	2	5	-0.53	-0.30	0.27	0.07
Q9D1P0	39S ribosomal protein L13, mitochondrial OS=Mus musculus GN=Mrpl13 PE=2 SV=1 - [RM13_MOUSE]	36.52	3	6	6	34	0.19	0.05	0.08	0.11
Q8BJ03-2	Isoform 2 of Cytochrome c oxidase assembly protein COX15 homolog OS=Mus musculus GN=Cox15 - [COX15_MOUSE]	8.33	2	3	3	8	-0.23	-0.09	-0.22	-0.04
E9Q6P5	Protein Ttc7b OS=Mus musculus GN=Ttc7b PE=2 SV=1 - [E9Q6P5_MOUSE]	1.54	1	1	1	1	0.00	-0.56	0.11	-0.45
Q3V156	Oxysterol-binding protein OS=Mus musculus GN=Osbpl1a PE=2 SV=1 - [Q3V156_MOUSE]	11.11	6	4	4	9	0.03	-0.16	0.15	-0.04
P57780	Alpha-actinin-4 OS=Mus musculus GN=Actn4 PE=1 SV=1 [ACTN4_MOUSE]	65.46	4	25	59	1999	-0.01	0.09	-0.01	0.09
Q7TT37	Elongator complex protein 1 OS=Mus musculus GN=Ikbkap PE=2 SV=2 - [ELP1_MOUSE]	6.38	1	7	7	16	0.09	0.13	-0.12	0.13
D3YUJ0	Ellis-van Creveld syndrome protein homolog OS=Mus musculus GN=Evc PE=2 SV=1 - [D3YUJ0_MOUSE]	2.33	4	2	2	3	-1.07	-0.65	-0.18	0.23
Q8BWG8-2	Isoform 1B of Beta-arrestin-1 OS=Mus musculus GN=Arrb1 - [ARRB1_MOUSE]	20.98	6	10	10	33	-0.07	-0.15	-0.41	-0.46
Q60597	2-oxoglutarate dehydrogenase, mitochondrial OS=Mus musculus GN=Ogdh PE=1 SV=3 - [ODO1_MOUSE]	54.15	5	38	46	556	-0.25	-0.21	-0.26	-0.24
Q8C1Y8	Vacuolar fusion protein CCZ1 homolog OS=Mus musculus GN=Ccz1 PE=1 SV=1 - [CCZ1_MOUSE]	6.46	1	3	3	7	-0.51	-0.58	-0.41	-0.48
P22366	Myeloid differentiation primary response protein MyD88 OS=Mus musculus GN=Myd88 PE=1 SV=3 - [MYD88_MOUSE]	7.77	3	2	2	3	-0.05	-0.08	-0.15	-0.17

Q8BZ97	PR domain zinc finger protein 8 OS=Mus musculus GN=Prdm8 PE=2 SV=1 - [PRDM8_MOUSE]	3.49	2	1	1	1	-0.34	-0.80	-0.13	-0.59
P60853	Leucine zipper putative tumor suppressor 1 OS=Mus musculus GN=Lzts1 PE=2 SV=3 - [LZTS1_MOUSE]	3.84	1	1	1	1	0.29	0.10	0.21	0.01
Q3U0V1	Far upstream element-binding protein 2 OS=Mus musculus GN=Khsrp PE=1 SV=2 [FUBP2_MOUSE]	39.84	1	20	22	228	-0.04	0.04	-0.05	-0.03
Q62181	Semaphorin-3C OS=Mus musculus GN=Sema3c PE=1 SV=2 - [SEM3C_MOUSE]	0.80	1	1	1	1	-0.42	-0.31	-0.34	-0.23
Q9R0N5	Synaptotagmin-5 OS=Mus musculus GN=Syt5 PE=1 SV=1 - [SYT5_MOUSE]	5.96	1	1	3	5	0.75	0.31	0.13	-0.32
Q91WH2	MCG131371 OS=Mus musculus GN=Ugt2b38 PE=2 SV=1 - [Q91WH2_MOUSE]	6.60	10	2	3	24	-0.84	-0.89	-0.78	-0.84
Q8CIF4	Biotinidase OS=Mus musculus GN=Btd PE=1 SV=2 - [BTD_MOUSE]	4.62	1	2	2	3	-0.14	-0.44	-0.30	-0.09
Q62376	U1 small nuclear ribonucleoprotein 70 kDa OS=Mus musculus GN=Snrnp70 PE=1 SV=2 - [RU17_MOUSE]	20.98	2	11	11	46	-0.09	-0.18	-0.15	-0.22
Q7TNU7	LETM1 domain-containing protein LETM2, mitochondrial OS=Mus musculus GN=Letm2 PE=2 SV=1 - [LETM2_MOUSE]	1.25	1	1	1	1	0.26	-0.03	-0.02	-0.31
Q9D1J1	Adaptin ear-binding coat-associated protein 2 OS=Mus musculus GN=Necap2 PE=1 SV=1 - [NECP2_MOUSE]	23.68	2	4	4	13	-0.13	-0.08	-0.06	-0.03
Q8CJ19	Protein-methionine sulfoxide oxidase MICAL3 OS=Mus musculus GN=Mical3 PE=1 SV=2 - [MICA3_MOUSE]	4.16	7	5	7	18	-0.44	0.48	-0.62	0.23
Q9JKS4-3	Isoform 3 of LIM domain-binding protein 3 OS=Mus musculus GN=Ldb3 - [LDB3_MOUSE]	26.32	7	11	12	51	0.07	-0.01	0.09	0.08
A1Y9I9	Transmembrane O-methyltransferase OS=Mus musculus GN=Tomt PE=1 SV=1 - [TOMT_MOUSE]	2.33	1	1	1	4	-0.71	-0.66	-0.43	-0.39
Q14BA6-2	Isoform 2 of Mesenteric estrogen-dependent adipogenesis protein OS=Mus musculus GN=Medag - [MEDAG_MOUSE]	14.29	2	1	1	2	-0.10	-0.19	-0.58	-0.68

Q9EQ20	Methylmalonate-semialdehyde dehydrogenase [acylating], mitochondrial OS=Mus musculus GN=Aldh6a1 PE=1 SV=1 - [MMSA_MOUSE]	43.93	1	22	22	130	-0.51	-0.51	-0.48	-0.58
Q9DBR1-2	Isoform 2 of 5'-3' exoribonuclease 2 OS=Mus musculus GN=Xrn2 - [XRN2_MOUSE]	27.46	2	23	23	75	0.10	-0.16	0.10	-0.08
Q9D8T7-2	Isoform 2 of SRA stem-loop-interacting RNA-binding protein, mitochondrial OS=Mus musculus GN=Slirp - [SLIRP_MOUSE]	38.38	4	2	2	24	0.09	0.31	-0.09	0.05
Q3UH60	Disco-interacting protein 2 homolog B OS=Mus musculus GN=Dip2b PE=1 SV=1 [DIP2B_MOUSE]	5.34	4	5	5	8	-0.06	0.22	-0.10	0.05
G3UX50	Death domain-associated protein 6 OS=Mus musculus GN=Daxx PE=2 SV=1 - [G3UX50_MOUSE]	2.56	3	1	1	1	-0.10	-0.02	-0.37	-0.29
Q99MN1	Lysine--tRNA ligase OS=Mus musculus GN=Kars PE=1 SV=1 - [SYK_MOUSE]	37.82	2	25	25	212	0.04	0.02	0.03	-0.04
Q8CJG1	Protein argonaute-1 OS=Mus musculus GN=Ago1 PE=1 SV=2 - [AGO1_MOUSE]	11.55	6	5	8	27	-0.27	-0.20	0.11	-0.04
E9QN99	Alpha/beta hydrolase domain-containing protein 14B OS=Mus musculus GN=Abhd14b PE=4 SV=1 - [E9QN99_MOUSE]	47.14	2	7	7	117	-0.08	-0.08	-0.15	-0.11
D3Z4C9	Uncharacterized protein OS=Mus musculus GN=Mnf1 PE=2 SV=1 - [D3Z4C9_MOUSE]	38.58	3	4	4	15	0.44	0.48	0.08	0.19
Q9CWN7	CCR4-NOT transcription complex subunit 11 OS=Mus musculus GN=Cnot11 PE=2 SV=1 - [CNO11_MOUSE]	12.67	1	3	3	12	0.22	0.08	-0.23	-0.34
P70195	Proteasome subunit beta type-7 OS=Mus musculus GN=Psmb7 PE=1 SV=1 - [PSB7_MOUSE]	14.80	1	4	4	39	0.05	0.17	0.12	0.19
Q9JM62-2	Isoform 2 of Receptor expression-enhancing protein 6 OS=Mus musculus GN=Reep6 - [REEP6_MOUSE]	14.37	2	2	2	3	-0.42	-0.36	-0.42	-0.37
Q9JKP7	DNA polymerase epsilon subunit 3 OS=Mus musculus GN=Pole3 PE=2 SV=1 [DPOE3_MOUSE]	24.83	2	3	3	6	0.10	0.01	-0.22	-0.30
Q9CQF0	39S ribosomal protein L11, mitochondrial OS=Mus musculus GN=Mrpl11 PE=2 SV=1 - [RM11_MOUSE]	39.06	1	7	7	12	-0.13	0.02	-0.09	-0.08

E0CZE0	NEDD8-activating enzyme E1 regulatory subunit OS=Mus musculus GN=Nae1 PE=2 SV=1 - [E0CZE0_MOUSE]	13.53	2	7	7	24	0.09	0.24	0.17	0.40
Q9WVB0	RNA-binding protein with multiple splicing OS=Mus musculus GN=Rbpms PE=2 SV=2 - [RBPMS_MOUSE]	8.63	4	2	2	5	-0.08	0.05	-0.13	-0.12
Q99M87-2	Isoform 2 of DnaJ homolog subfamily A member 3, mitochondrial OS=Mus musculus GN=Dnaja3 - [DNJA3_MOUSE]	23.62	3	10	10	44	-0.02	-0.07	-0.03	-0.04
F7DAY5	Regulation of nuclear pre-mRNA domain-containing protein 1B (Fragment) OS=Mus musculus GN=Rprd1b PE=4 SV=1 - [F7DAY5_MOUSE]	45.45	5	5	5	42	0.09	-0.13	0.00	-0.20
Q9CR68	Cytochrome b-c1 complex subunit Rieske, mitochondrial OS=Mus musculus GN=Uqcrfs1 PE=1 SV=1 - [UCRI_MOUSE]	17.52	1	7	7	102	-0.30	-0.16	-0.28	-0.22
Q8CAK3-2	Isoform 2 of UPF0515 protein C19orf66 homolog OS=Mus musculus - [CS066_MOUSE]	11.04	2	2	2	4	-0.31	-0.34	-0.17	-0.20
Q811P8-2	Isoform 2 of Rho GTPase-activating protein 32 OS=Mus musculus GN=Arhgap32 - [RHG32_MOUSE]	4.43	5	5	5	14	-0.37	-0.20	-0.24	-0.18
E9Q641	Nebulin-related-anchoring protein OS=Mus musculus GN=Nrap PE=2 SV=1 - [E9Q641_MOUSE]	2.49	7	3	3	3	0.12	-0.32	0.16	-0.27
P63024	Vesicle-associated membrane protein 3 OS=Mus musculus GN=Vamp3 PE=1 SV=1 - [VAMP3_MOUSE]	39.81	1	2	5	85	-0.03	-0.05	-0.02	-0.15
Q3TAS6-2	Isoform 2 of ER membrane protein complex subunit 10 OS=Mus musculus GN=Emc10 - [EMC10_MOUSE]	7.09	2	2	2	4	-0.07	-0.16	0.08	-0.01
Q8CIC2-2	Isoform 2 of Nucleoporin-like protein 2 OS=Mus musculus GN=Nupl2 - [NUPL2_MOUSE]	11.25	3	1	1	2	0.06	0.17	-0.10	0.01
Q9CQ22	Ragulator complex protein LAMTOR1 OS=Mus musculus GN=Lamtor1 PE=1 SV=1 - [LTOR1_MOUSE]	31.68	1	5	5	19	0.23	0.00	0.20	-0.06
Q9QZK2-2	Isoform 2 of Breast cancer anti-estrogen resistance protein 3 OS=Mus musculus GN=Bcar3 - [BCAR3_MOUSE]	5.14	2	2	2	4	0.22	0.32	0.13	0.23
Q80VP1	Epsin-1 OS=Mus musculus GN=Epn1 PE=1 SV=3 - [EPN1_MOUSE]	12.35	4	5	6	45	-0.16	-0.03	-0.08	0.11

Q9D0A3	UPF0552 protein C15orf38 homolog OS=Mus musculus PE=2 SV=1 - [CO038_MOUSE]	62.83	1	10	10	55	-0.21	-0.07	-0.18	-0.02
Q62261	Spectrin beta chain, non erythrocytic 1 OS=Mus musculus GN=Sptbn1 PE=1 SV=2 - [SPTB2_MOUSE]	60.47	5	9	126	1397	-0.08	-0.03	-0.13	-0.02
Q3V3P1	DNA topoisomerase OS=Mus musculus GN=Top3a PE=2 SV=1 [Q3V3P1_MOUSE]	1.68	2	1	1	1	-0.49	-0.34	-0.17	-0.03
P15532	Nucleoside diphosphate kinase A OS=Mus musculus GN=Nme1 PE=1 SV=1 - [NDKA_MOUSE]	66.45	3	3	10	213	0.03	0.25	0.10	0.24
I6L974	TBC1 domain family member 17 OS=Mus musculus GN=Tbc1d17 PE=2 SV=1 - [I6L974_MOUSE]	1.47	2	1	1	1	-0.07	-0.23	-0.12	-0.28
Q9QZN4	F-box only protein 6 OS=Mus musculus GN=Fbxo6 PE=1 SV=1 - [FBX6_MOUSE]	9.15	3	2	2	7	-0.16	-0.24	0.17	-0.65
E9PVU9	Bis(5'-adenosyl)-triphosphatase (Fragment) OS=Mus musculus GN=Fhit PE=2 SV=1 - [E9PVU9_MOUSE]	12.90	4	1	1	5	-0.47	-0.62	-0.20	-0.34
O08915	AH receptor-interacting protein OS=Mus musculus GN=Aip PE=1 SV=1 - [AIP_MOUSE]	21.21	2	6	6	18	0.26	0.46	0.27	0.22
Q8BGD8	Cytochrome c oxidase assembly factor 6 homolog OS=Mus musculus GN=Coa6 PE=1 SV=1 - [COA6_MOUSE]	35.44	1	2	2	10	-0.23	0.21	0.13	0.24
Q9Z2I9	Succinyl-CoA ligase [ADP-forming] subunit beta, mitochondrial OS=Mus musculus GN=Sucla2 PE=1 SV=2 - [SUCB1_MOUSE]	42.98	1	20	20	226	-0.19	-0.16	-0.18	-0.16
Q61189	Methylosome subunit pICln OS=Mus musculus GN=Clns1a PE=1 SV=1 - [ICLN_MOUSE]	16.10	2	3	3	26	-0.11	-0.15	-0.06	-0.09
Q3TLS3	GDP-D-glucose phosphorylase 1 OS=Mus musculus GN=Gdpgp1 PE=2 SV=2 - [GDPP1_MOUSE]	11.14	1	3	4	12	-0.38	-0.08	-0.45	-0.35
P47754	F-actin-capping protein subunit alpha-2 OS=Mus musculus GN=Capza2 PE=1 SV=3 - [CAZA2_MOUSE]	60.14	2	9	12	377	-0.16	-0.14	-0.18	-0.16
Q91VM5	RNA binding motif protein, X-linked-like-1 OS=Mus musculus GN=Rbmxl1 PE=1 SV=1 - [RMXL1_MOUSE]	19.85	1	7	7	70	-0.02	-0.12	-0.07	-0.19

Q9CX56	26S proteasome non-ATPase regulatory subunit 8 OS=Mus musculus GN=Psmd8 PE=1 SV=2 - [PSMD8_MOUSE]	27.20	1	10	10	52	0.03	-0.15	0.04	-0.05
Q9CQ86	Migration and invasion enhancer 1 OS=Mus musculus GN=Mien1 PE=1 SV=1 - [MIEN1_MOUSE]	14.78	1	2	2	8	-0.41	-0.26	-0.33	-0.17
Q6Y5D8-2	Isoform 2 of Rho GTPase-activating protein 10 OS=Mus musculus GN=Arhgap10 - [RHG10_MOUSE]	3.40	4	2	2	3	-0.60	0.12	0.01	0.07
Q9DBQ7	Protein-associating with the carboxyl-terminal domain of ezrin OS=Mus musculus GN=Scyl3 PE=1 SV=3 [PACE1_MOUSE]	8.44	2	3	3	9	0.08	0.26	-0.18	-0.07
F8WHU9	Zinc finger protein ZPR1 (Fragment) OS=Mus musculus GN=Zfp259 PE=4 SV=1 - [F8WHU9_MOUSE]	43.52	3	13	13	37	-0.10	0.11	-0.05	0.17
O35685	Nuclear migration protein nudC OS=Mus musculus GN=Nudc PE=1 SV=1 - [NUDC_MOUSE]	52.71	1	17	18	134	0.22	0.32	0.09	0.24
Q64430	Copper-transporting ATPase 1 OS=Mus musculus GN=Atp7a PE=1 SV=3 - [ATP7A_MOUSE]	7.85	2	10	10	23	0.00	-0.04	0.03	0.02
Q9CPR5	39S ribosomal protein L15, mitochondrial OS=Mus musculus GN=Mrpl15 PE=1 SV=1 - [RM15_MOUSE]	19.32	2	5	5	17	-0.27	-0.28	-0.18	-0.09
Q9CY73	39S ribosomal protein L44, mitochondrial OS=Mus musculus GN=Mrpl44 PE=1 SV=3 - [RM44_MOUSE]	13.21	2	3	3	11	-0.13	0.10	-0.08	-0.11
Q8CI51	PDZ and LIM domain protein 5 OS=Mus musculus GN=Pdlim5 PE=1 SV=4 - [PDLI5_MOUSE]	41.96	3	11	16	109	-0.11	-0.33	-0.18	-0.35
Q80SY4	E3 ubiquitin-protein ligase MIB1 OS=Mus musculus GN=Mib1 PE=1 SV=1 - [MIB1_MOUSE]	7.16	3	5	7	13	0.47	-0.27	0.38	0.03
P11440	Cyclin-dependent kinase 1 OS=Mus musculus GN=Cdk1 PE=1 SV=3 - [CDK1_MOUSE]	20.20	36	4	6	29	-0.28	0.01	-0.13	0.00
P57784	U2 small nuclear ribonucleoprotein A' OS=Mus musculus GN=Snrpa1 PE=1 SV=2 - [RU2A_MOUSE]	25.10	2	6	6	47	0.12	0.01	-0.06	0.01
D3YU80	Cold-inducible RNA-binding protein (Fragment) OS=Mus musculus GN=Cirbp PE=2 SV=1 - [D3YU80_MOUSE]	23.15	3	2	2	9	-0.18	-0.52	-0.40	-0.83
Q80X82	Symplekin OS=Mus musculus GN=Sympk PE=1 SV=1 - [SYMPK_MOUSE]	2.96	2	2	2	2	0.13	0.46	-0.18	0.15

Q8BIW1	Protein prune homolog OS=Mus musculus GN=Prune PE=2 SV=1 [PRUNE_MOUSE]	11.67	1	4	4	12	0.25	0.04	0.21	-0.14
F6QUA9	TBC1 domain family member 1 OS=Mus musculus GN=Tbc1d1 PE=2 SV=2 - [F6QUA9_MOUSE]	8.94	4	6	6	13	0.06	0.12	-0.09	-0.02
F6SPX7	Protein-L-isoaspartate O-methyltransferase domain-containing protein 2 (Fragment) OS=Mus musculus GN=Pcmtd2 PE=4 SV=1 - [F6SPX7_MOUSE]	5.81	4	1	1	1	-0.51	-0.19	-0.38	-0.06
A6H611	Mitochondrial intermediate peptidase OS=Mus musculus GN=Mipep PE=2 SV=1 - [MIPEP_MOUSE]	12.66	1	9	10	17	-0.10	-0.40	-0.09	0.12
P39447	Tight junction protein ZO-1 OS=Mus musculus GN=Tjp1 PE=1 SV=2 - [ZO1_MOUSE]	22.87	1	1	31	129	0.23	0.27	0.20	0.23
Q9DBX2	Phosducin-like protein OS=Mus musculus GN=Pdcl PE=1 SV=1 - [PHLP_MOUSE]	3.32	1	1	1	1	0.31	0.79	-0.35	0.12
A2APF7	Z-DNA-binding protein 1 OS=Mus musculus GN=Zbp1 PE=1 SV=1 - [A2APF7_MOUSE]	12.65	3	4	4	12	0.44	0.39	0.15	0.15
Q8VBT6	Apolipoprotein B receptor OS=Mus musculus GN=Apobr PE=1 SV=1 - [APOBR_MOUSE]	2.34	1	2	2	4	-0.04	-0.13	0.01	-0.08
Q80V94	AP-4 complex subunit epsilon-1 OS=Mus musculus GN=Ap4e1 PE=2 SV=3 - [AP4E1_MOUSE]	0.62	1	1	1	1	-0.23	-0.46	-0.25	-0.48
A2AVR9	Dynein light chain roadblock-type 1 OS=Mus musculus GN=Dynlrb1 PE=2 SV=1 - [A2AVR9_MOUSE]	84.62	2	6	6	109	-0.14	-0.04	-0.17	-0.05
P63087-2	Isoform Gamma-2 of Serine/threonine-protein phosphatase PP1-gamma catalytic subunit OS=Mus musculus GN=Ppp1cc - [PP1G_MOUSE]	36.50	2	3	12	154	-0.01	-0.25	-0.02	-0.23
Q9D7S0	Ly6/PLAUR domain-containing protein 8 OS=Mus musculus GN=Lypd8 PE=2 SV=1 - [LYPD8_MOUSE]	3.53	1	1	1	15	0.24	0.47	0.04	0.19
B1ATI9	Growth arrest-specific protein 7 OS=Mus musculus GN=Gas7 PE=4 SV=1 - [B1ATI9_MOUSE]	13.59	4	4	4	5	-0.42	-0.33	-0.26	-0.41
O70218	Homeobox protein HMX1 OS=Mus musculus GN=Hmx1 PE=2 SV=1 - [HMX1_MOUSE]	9.64	1	1	1	1	-0.08	-0.52	-0.14	-0.57
Q9JJA4	Ribosome biogenesis protein WDR12 OS=Mus musculus GN=Wdr12 PE=2 SV=1 - [WDR12_MOUSE]	8.75	1	2	3	5	0.09	0.26	-0.07	0.10

Q8CC12-3	Isoform 3 of Codanin-1 OS=Mus musculus GN=Cdan1 - [CDAN1_MOUSE]	0.78	3	1	1	3	-0.14	-0.07	-0.16	-0.10
Q9DBC0	Selenoprotein O OS=Mus musculus GN=Selo PE=1 SV=4 - [SELO_MOUSE]	6.30	1	3	3	8	-0.25	-0.41	-0.22	-0.41
Q8CFE3	REST corepressor 1 OS=Mus musculus GN=Rcor1 PE=1 SV=2 [RCOR1_MOUSE]	18.03	4	4	4	9	0.10	0.32	0.00	0.21
D3YYT0	Cadherin-2 OS=Mus musculus GN=Cdh2 PE=2 SV=1 - [D3YYT0_MOUSE]	5.42	2	2	2	5	-0.44	-0.25	-0.50	-0.31
Q3UEG6	Alanine--glyoxylate aminotransferase 2, mitochondrial OS=Mus musculus GN=Agxt2 PE=2 SV=1 - [AGT2_MOUSE]	2.34	2	1	1	2	-0.07	-0.13	-0.11	-0.18
Q5SVQ0-4	Isoform 4 of Histone acetyltransferase KAT7 OS=Mus musculus GN=Kat7 - [KAT7_MOUSE]	8.61	5	3	3	15	-0.27	-0.12	-0.10	0.19
Q921S7	39S ribosomal protein L37, mitochondrial OS=Mus musculus GN=Mrpl37 PE=2 SV=1 - [RM37_MOUSE]	24.82	1	10	10	36	-0.16	0.01	-0.27	-0.03
P53996-2	Isoform 2 of Cellular nucleic acid-binding protein OS=Mus musculus GN=Cnbp - [CNBP_MOUSE]	24.12	5	4	4	16	-0.11	-0.22	0.03	-0.11
Q8BXC6	COMM domain-containing protein 2 OS=Mus musculus GN=Commd2 PE=1 SV=1 - [COMD2_MOUSE]	41.21	2	6	6	12	-0.06	-0.13	0.01	-0.05
G5E924	Heterogeneous nuclear ribonucleoprotein L (Fragment) OS=Mus musculus GN=Hnrnpl PE=4 SV=1 - [G5E924_MOUSE]	44.55	5	19	19	501	0.02	0.07	-0.02	0.02
O35711-2	Isoform 2 of Liprin-beta 2 OS=Mus musculus GN=Ppfibp2 - [LIPB2_MOUSE]	4.02	4	2	3	11	-0.22	-0.18	-0.10	-0.19
D3YZY4	Retinoic acid receptor gamma (Fragment) OS=Mus musculus GN=Rarg PE=2 SV=1 - [D3YZY4_MOUSE]	6.17	8	1	1	1	0.25	0.25	0.19	0.19
P61327	Protein mago nashi homolog OS=Mus musculus GN=Magoh PE=2 SV=1 - [MGN_MOUSE]	34.93	4	4	4	27	0.11	0.25	-0.02	0.13
Q6ZQL4	WD repeat-containing protein 43 OS=Mus musculus GN=Wdr43 PE=1 SV=2 - [WDR43_MOUSE]	4.28	1	4	4	5	-0.16	-0.50	-0.12	-0.47
O88532	Zinc finger RNA-binding protein OS=Mus musculus GN=Zfr PE=1 SV=2 - [ZFR_MOUSE]	8.94	4	7	7	15	0.13	0.10	0.08	0.05
Q8C6Y6-2	Isoform 2 of Ankyrin repeat and SOCS box protein 14 OS=Mus musculus GN=Asb14 - [ASB14_MOUSE]	6.47	3	2	2	2	0.86	0.94	1.15	1.23

Q61166	Microtubule-associated protein RP/EB family member 1 OS=Mus musculus GN=Mapre1 PE=1 SV=3 - [MARE1_MOUSE]	49.63	2	9	12	147	0.21	0.17	0.14	0.18
Q9D706	RNA polymerase II-associated protein 3 OS=Mus musculus GN=Rpap3 PE=1 SV=1 - [RPAP3_MOUSE]	11.36	1	6	6	20	0.21	0.03	0.26	0.06
Q9CPX6	Ubiquitin-like-conjugating enzyme ATG3 OS=Mus musculus GN=Atg3 PE=1 SV=1 - [ATG3_MOUSE]	22.29	1	6	6	24	0.14	0.41	0.24	0.42
Q9Z1K5	E3 ubiquitin-protein ligase ARIH1 OS=Mus musculus GN=Arih1 PE=2 SV=3 - [ARI1_MOUSE]	11.71	3	6	6	32	0.03	-0.11	0.03	-0.07
P53808	Phosphatidylcholine transfer protein OS=Mus musculus GN=Pctp PE=1 SV=2 - [PPCT_MOUSE]	19.63	2	4	4	8	-0.03	0.04	-0.10	0.09
Q8R007-2	Isoform 2 of Poliovirus receptor-related protein 4 OS=Mus musculus GN=Pvrl4 - [PVRL4_MOUSE]	1.24	3	1	1	1	0.47	-0.01	0.33	-0.15
Q91X84	CREB-regulated transcription coactivator 3 OS=Mus musculus GN=Crtc3 PE=2 SV=2 - [CRTC3_MOUSE]	1.62	1	1	1	2	-0.03	-0.13	-0.22	-0.33
F8VPK5	Rho-associated protein kinase 2 OS=Mus musculus GN=Rock2 PE=2 SV=1 - [F8VPK5_MOUSE]	28.31	3	26	34	143	0.09	0.26	0.03	0.08
Q9WUM4	Coronin-1C OS=Mus musculus GN=Coro1c PE=1 SV=2 - [COR1C_MOUSE]	44.51	9	23	25	311	0.17	0.30	0.09	0.22
A2AWT6	Nucleolar transcription factor 1 OS=Mus musculus GN=Ubtf PE=4 SV=1 - [A2AWT6_MOUSE]	23.80	7	16	17	60	0.17	0.04	0.23	0.00
Q0KK59	Protein unc-79 homolog OS=Mus musculus GN=Unc79 PE=1 SV=1 - [UNC79_MOUSE]	0.58	1	1	1	2	-0.70	-2.39	-0.51	-2.21
P22682	E3 ubiquitin-protein ligase CBL OS=Mus musculus GN=Cbl PE=1 SV=3 - [CBL_MOUSE]	17.42	1	9	11	29	-0.17	-0.21	-0.27	-0.27
Q9DBF1-2	Isoform 2 of Alpha-aminoadipic semialdehyde dehydrogenase OS=Mus musculus GN=Aldh7a1 - [AL7A1_MOUSE]	36.99	6	11	11	48	-0.02	-0.03	-0.09	0.00
B2FDF6	Synaptosomal-associated protein 47 OS=Mus musculus GN=Snap47 PE=2 SV=1 - [B2FDF6_MOUSE]	4.56	3	1	1	1	-0.12	0.36	-0.48	0.00

Q4KMM3-2	Isoform 2 of Oxidation resistance protein 1 OS=Mus musculus GN=Oxr1 - [OXR1_MOUSE]	13.62	6	6	6	20	0.14	0.02	0.24	0.03
Q6P3D0	U8 snoRNA-decapping enzyme OS=Mus musculus GN=Nudt16 PE=1 SV=1 - [NUD16_MOUSE]	41.54	4	7	7	18	-0.14	-0.08	-0.19	-0.16
Q9WTK3	Glycosylphosphatidylinositol anchor attachment 1 protein OS=Mus musculus GN=Gpaa1 PE=1 SV=3 - [GPAA1_MOUSE]	5.64	4	2	2	3	0.12	-0.54	-0.26	-0.92
P97814	Proline-serine-threonine phosphatase-interacting protein 1 OS=Mus musculus GN=Pstpip1 PE=1 SV=1 - [PPIP1_MOUSE]	7.47	1	2	2	2	-0.02	0.32	0.06	0.40
F7BP55	Adenylate kinase 2, mitochondrial (Fragment) OS=Mus musculus GN=Ak2 PE=2 SV=1 - [F7BP55_MOUSE]	55.56	1	1	4	76	0.14	0.15	0.17	0.18
Q9WTU6-3	Isoform Beta-1 of Mitogen-activated protein kinase 9 OS=Mus musculus GN=Mapk9 - [MK09_MOUSE]	24.41	13	5	7	66	-0.14	-0.14	-0.11	-0.14
E9Q9E8	SLAM family member 5 OS=Mus musculus GN=Cd84 PE=2 SV=1 - [E9Q9E8_MOUSE]	10.98	4	2	2	2	0.23	0.26	0.44	0.47
P56480	ATP synthase subunit beta, mitochondrial OS=Mus musculus GN=Atp5b PE=1 SV=2 [ATPB_MOUSE]	68.05	1	26	26	2285	-0.26	-0.23	-0.26	-0.27
Q5SVD0	Protein FAM101B OS=Mus musculus GN=Fam101b PE=2 SV=1 - [F101B_MOUSE]	12.04	1	2	2	3	0.25	0.26	0.03	0.04
O35691	Pinin OS=Mus musculus GN=Pnn PE=1 SV=4 - [PININ_MOUSE]	9.38	2	6	6	12	0.08	0.13	0.00	0.10
G3UZ34	116 kDa U5 small nuclear ribonucleoprotein component OS=Mus musculus GN=Eftud2 PE=2 SV=1 - [G3UZ34_MOUSE]	35.14	5	26	27	186	-0.03	-0.15	-0.04	-0.17
Q6NV99	Protein Haus6 OS=Mus musculus GN=Haus6 PE=2 SV=1 - [Q6NV99_MOUSE]	4.82	1	2	4	7	-0.29	0.85	-0.34	0.80
P17809	Solute carrier family 2, facilitated glucose transporter member 1 OS=Mus musculus GN=Slc2a1 PE=1 SV=4 - [GTR1_MOUSE]	6.91	1	2	2	4	0.55	0.54	0.09	0.12
Q9DAW6	U4/U6 small nuclear ribonucleoprotein Prp4 OS=Mus musculus GN=Prpf4 PE=2 SV=1 - [PRP4_MOUSE]	19.77	1	7	7	31	-0.03	-0.09	-0.08	-0.09

Q9QUR6	Prolyl endopeptidase OS=Mus musculus GN=Prep PE=2 SV=1 - [PPCE_MOUSE]	45.63	1	26	26	137	0.13	0.17	0.03	0.13
Q80T06	Elongation factor 1-delta OS=Mus musculus GN=Eef1d PE=2 SV=1 - [Q80T06_MOUSE]	60.85	4	1	16	344	-0.16	-0.04	-0.05	-0.17
O89112	LanC-like protein 1 OS=Mus musculus GN=Lancl1 PE=1 SV=1 - [LANC1_MOUSE]	19.05	2	7	7	47	-0.15	-0.19	-0.15	-0.22
P27612	Phospholipase A-2-activating protein OS=Mus musculus GN=Plaa PE=2 SV=4 - [PLAP_MOUSE]	23.55	1	9	16	71	0.16	0.18	0.02	-0.04
P46664	Adenylosuccinate synthetase isozyme 2 OS=Mus musculus GN=Adss PE=1 SV=2 - [PURA2_MOUSE]	39.91	1	14	15	177	0.20	-0.05	0.15	0.06
D3YTK6	NAD-dependent protein deacetylase sirtuin-3 (Fragment) OS=Mus musculus GN=Sirt3 PE=2 SV=2 - [D3YTK6_MOUSE]	9.69	1	2	2	2	-0.70	-0.72	-0.28	-0.30
Q924M7	Mannose-6-phosphate isomerase OS=Mus musculus GN=Mpi PE=2 SV=1 - [MPI_MOUSE]	29.08	2	11	11	77	-0.11	-0.11	-0.20	-0.19
Q925B0-2	Isoform 2 of PRKC apoptosis WT1 regulator protein OS=Mus musculus GN=Pawr - [PAWR_MOUSE]	44.64	2	7	8	27	0.02	-0.04	-0.05	-0.05
Q8VDT9	39S ribosomal protein L50, mitochondrial OS=Mus musculus GN=Mrpl50 PE=2 SV=2 - [RM50_MOUSE]	44.65	1	6	6	27	-0.05	-0.29	-0.07	-0.10
Q9Z2A5-2	Isoform ATE1-2 of Arginyl-tRNA--protein transferase 1 OS=Mus musculus GN=Ate1 - [ATE1_MOUSE]	16.67	4	5	5	10	-0.13	0.14	-0.23	-0.03
P50396	Rab GDP dissociation inhibitor alpha OS=Mus musculus GN=Gdi1 PE=1 SV=3 - [GDIA_MOUSE]	38.93	3	9	14	232	-0.07	-0.03	-0.11	-0.03
P17742	Peptidyl-prolyl cis-trans isomerase A OS=Mus musculus GN=Ppia PE=1 SV=2 - [PPIA_MOUSE]	71.34	2	13	15	858	-0.06	0.08	-0.09	0.05
Q8VHL1	Histone-lysine N-methyltransferase SETD7 OS=Mus musculus GN=Setd7 PE=2 SV=2 - [SETD7_MOUSE]	38.80	1	9	10	34	-0.05	-0.08	-0.05	-0.01
B1ATZ0	Hepatocyte growth factor-regulated tyrosine kinase substrate OS=Mu musculus GN=Hgs PE=2 SV=1 - [B1ATZ0_MOUSE]	16.58	10	11	11	35	-0.14	0.18	0.06	0.05
Q9CQB2	Protein FAM195A OS=Mus musculus GN=Fam195a PE=1 SV=1 - [F195A_MOUSE]	26.25	1	2	3	6	-0.20	0.30	-0.33	0.17
H7BX05	Obscurin OS=Mus musculus GN=Obscn PE=2 SV=2 - [H7BX05_MOUSE]	1.53	8	6	6	23	-0.17	-0.24	-0.13	-0.20

D3YUM6	MCG2960, isoform CRA_a OS=Mus musculus GN=Steap2 PE=4 SV=1 - [D3YUM6_MOUSE]	3.98	3	1	1	1	-0.49	-0.07	-0.69	-0.27
Q91Z96-2	Isoform 2 of BMP-2-inducible protein kinase OS=Mus musculus GN=Bmp2k - [BMP2K_MOUSE]	2.31	2	1	1	3	-0.29	-1.10	-0.31	-1.13
P17182	Alpha-enolase OS=Mus musculus GN=Eno1 PE=1 SV=3 - [ENOA_MOUSE]	76.50	5	22	30	1180	0.19	0.31	0.11	0.24
Q925F2	Endothelial cell-selective adhesion molecule OS=Mus musculus GN=Esam PE=1 SV=1 - [ESAM_MOUSE]	11.93	2	3	3	6	-0.53	-0.61	-0.62	-0.69
Q99N96	39S ribosomal protein L1, mitochondrial OS=Mus musculus GN=Mrpl1 PE=1 SV=2 [RM01_MOUSE]	20.24	2	5	6	17	0.08	0.15	-0.02	0.11
Q9JJ66	Cell division cycle protein 20 homolog OS=Mus musculus GN=Cdc20 PE=1 SV=2 - [CDC20_MOUSE]	2.40	1	1	1	1	0.82	0.65	0.58	0.41
G3UZE8	Paired box protein Pax-4 OS=Mus musculus GN=Pax4 PE=2 SV=1 - [G3UZE8_MOUSE]	14.01	5	2	2	2	-0.42	0.08	-0.46	0.04
Q9EP71	Ankycorbin OS=Mus musculus GN=Rai14 PE=1 SV=1 - [RAI14_MOUSE]	7.76	1	6	7	16	-0.16	-0.20	-0.11	-0.18
P01887	Beta-2-microglobulin OS=Mus musculus GN=B2m PE=1 SV=2 - [B2MG_MOUSE]	40.34	1	4	4	119	-0.27	-0.18	-0.14	-0.09
Q5FWX7	Oxysterol-binding protein OS=Mus musculus GN=Osbpl9 PE=2 SV=1 - [Q5FWX7_MOUSE]	8.31	6	4	5	7	0.03	-0.04	0.11	-0.02
P14148	60S ribosomal protein L7 OS=Mus musculus GN=Rpl7 PE=2 SV=2 - [RL7_MOUSE]	55.93	2	20	20	321	0.07	0.09	0.07	0.04
Q9Z2Z6	Mitochondrial carnitine/acylcarnitine carrier protein OS=Mus musculus GN=Slc25a20 PE=1 SV=1 - [MCAT_MOUSE]	23.26	1	10	10	31	-0.45	-0.50	-0.44	-0.59
P0C0A3	Charged multivesicular body protein 6 OS=Mus musculus GN=Chmp6 PE=2 SV=2 - [CHMP6_MOUSE]	22.50	3	3	4	13	-0.25	-0.45	-0.12	-0.31
Q8JZV7	Putative N-acetylglucosamine-6-phosphate deacetylase OS=Mus musculus GN=Amdhd2 PE=2 SV=1 - [NAGA_MOUSE]	23.96	3	6	6	13	-0.28	-0.28	-0.25	-0.32
Q8VCW8	Acyl-CoA synthetase family member 2, mitochondrial OS=Mus musculus GN=Acsf2 PE=2 SV=1 - [ACSF2_MOUSE]	43.58	1	23	24	330	-0.73	-0.69	-0.71	-0.67

Q3UHX2	28 kDa heat- and acid-stable phosphoprotein OS=Mus musculus GN=Pdap1 PE=1 SV=1 - [HAP28_MOUSE]	28.18	1	6	6	41	0.28	0.39	0.32	0.40
E0CXW2	Uncharacterized aarF domain-containing protein kinase 5 OS=Mus musculus GN=Adck5 PE=2 SV=1 - [E0CXW2_MOUSE]	6.00	5	3	3	9	-0.36	-0.54	-0.38	-0.55
Q9QY96	Extracellular calcium-sensing receptor OS=Mus musculus GN=Casr PE=2 SV=2 - [CASR_MOUSE]	5.56	2	2	2	7	-0.04	-0.30	-0.03	-0.29
Q6LC96	RXR alpha 2 OS=Mus musculus GN=Rxra PE=2 SV=1 - [Q6LC96_MOUSE]	2.28	2	1	1	1	-0.11	-0.23	-0.15	-0.27
O08583	THO complex subunit 4 OS=Mus musculus GN=Alyref PE=1 SV=3 - [THOC4_MOUSE]	35.29	4	4	6	108	-0.43	-0.46	-0.40	-0.25
P61971	Nuclear transport factor 2 OS=Mus musculus GN=Nutf2 PE=2 SV=1 [NTF2_MOUSE]	56.69	1	5	5	56	0.05	0.14	0.07	0.15
Q9D4B2	Tetratricopeptide repeat protein 25 OS=Mus musculus GN=Ttc25 PE=2 SV=3 - [TTC25_MOUSE]	4.97	1	2	2	6	-1.29	-0.34	-1.19	-0.25
Q00PI9	Heterogeneous nuclear ribonucleoprotein U-like protein 2 OS=Mus musculus GN=Hnrnpul2 PE=1 SV=2 - [HNRL2_MOUSE]	35.03	1	24	24	178	-0.06	-0.02	-0.09	-0.01
F8VPN2	Protein Tex15 OS=Mus musculus GN=Tex15 PE=2 SV=1 - [F8VPN2_MOUSE]	1.87	2	2	2	4	-0.48	-1.04	-0.10	-0.66
Q52KF7	Disks large homolog 3 OS=Mus musculus GN=Dlg3 PE=2 SV=1 - [Q52KF7_MOUSE]	11.51	3	6	7	22	0.05	-0.26	-0.04	-0.23
Q8R2K3	Single-stranded DNA-binding protein OS=Mus musculus GN=Ssbp1 PE=2 SV=1 [Q8R2K3_MOUSE]	31.08	3	5	5	34	-0.32	-0.13	-0.30	-0.20
Q9QX47-3	Isoform 3 of Protein SON OS=Mus musculus GN=Son - [SON_MOUSE]	13.77	9	22	22	76	0.09	0.07	0.05	0.06
Q921B4	Neurotrophin receptor-interacting factor 2 OS=Mus musculus GN=Nrif2 PE=1 SV=1 - [NRIF2_MOUSE]	1.21	2	1	1	1	-0.63	-0.27	-0.65	-0.29
Q8BP48	Methionine aminopeptidase 1 OS=Mus musculus GN=Metap1 PE=2 SV=1 - [MAP11_MOUSE]	42.23	1	11	11	59	0.14	0.24	0.09	0.26
P16546-2	Isoform 2 of Spectrin alpha chain, non-erythrocytic 1 OS=Mus musculus GN=Sptan1 - [SPTN1_MOUSE]	68.92	3	2	153	2310	-0.11	0.26	-0.26	0.15

Q8R000	Organic solute transporter subunit alpha OS=Mus musculus GN=Slc51a PE=1 SV=1 - [OSTA_MOUSE]	9.12	1	1	1	2	0.11	0.36	-0.07	0.18
Q8VHR5	Transcriptional repressor p66-beta OS=Mus musculus GN=Gatad2b PE=1 SV=1 - [P66B_MOUSE]	7.58	2	2	3	16	0.04	0.12	-0.22	-0.14
Q8CFK1	Glycylpeptide N-tetradecanoyltransferase OS=Mus musculus GN=Nmt2 PE=2 SV=1 - [Q8CFK1_MOUSE]	16.08	3	3	5	26	0.00	-0.04	0.22	0.22
P70665-3	Isoform 3 of Sialate O-acetylesterase OS=Mus musculus GN=Siae - [SIAE_MOUSE]	3.85	3	1	1	7	-0.05	-0.29	-0.27	-0.36
Q9Z0V1-2	Isoform 2 of Potassium voltage-gated channel subfamily D member 3 OS=Mus musculus GN=Kcnd3 - [KCND3_MOUSE]	3.46	2	1	1	1	-0.32	-0.35	0.16	0.12
Q60759	Glutaryl-CoA dehydrogenase, mitochondrial OS=Mus musculus GN=Gcdh PE=2 SV=2 - [GCDH_MOUSE]	30.14	3	7	7	83	-0.14	-0.27	-0.17	-0.12
Q9R013	Cathepsin F OS=Mus musculus GN=Ctsf PE=2 SV=1 - [CATF_MOUSE]	1.52	1	1	1	4	0.09	0.15	0.30	0.34
F6RG68	FCH domain only protein 2 OS=Mus musculus GN=Fcho2 PE=4 SV=2 - [F6RG68_MOUSE]	10.71	13	7	8	11	0.06	-0.19	0.07	0.00
Q80U93	Nuclear pore complex protein Nup214 OS=Mus musculus GN=Nup214 PE=1 SV=2 - [NU214_MOUSE]	9.02	6	14	15	36	0.04	0.12	0.03	0.22
Q8BGN5	NIPA-like protein 3 OS=Mus musculus GN=Nipal3 PE=2 SV=1 - [NPAL3_MOUSE]	5.37	1	1	1	2	0.13	0.14	-0.07	-0.06
Q9DCM0	Persulfide dioxygenase ETHE1, mitochondrial OS=Mus musculus GN=Ethe1 PE=1 SV=2 [ETHE1_MOUSE]	66.14	1	12	12	319	-0.24	-0.20	-0.16	-0.18
Q8BHJ9	Pre-mRNA-splicing factor SLU7 OS=Mus musculus GN=Slu7 PE=1 SV=1 - [SLU7_MOUSE]	6.67	2	3	3	5	0.17	-0.03	0.13	-0.07
P52825	Carnitine O-palmitoyltransferase 2, mitochondrial OS=Mus musculus GN=Cpt2 PE=1 SV=2 - [CPT2_MOUSE]	39.06	4	22	22	168	-0.35	-0.38	-0.24	-0.23
Q9D6Y7-2	Isoform 2 of Mitochondrial peptide methionine sulfoxide reductase OS=Mus musculus GN=Msra - [MSRA_MOUSE]	43.19	4	7	7	73	-0.43	-0.42	-0.40	-0.32

P19137	Laminin subunit alpha-1 OS=Mus musculus GN=Lama1 PE=1 SV=1 - [LAMA1_MOUSE]	2.30	2	5	6	9	-0.09	-0.32	-0.03	-0.12
Q9CZU6	Citrate synthase, mitochondrial OS=Mus musculus GN=Cs PE=1 SV=1 - [CISY_MOUSE]	33.41	1	9	16	506	-0.24	-0.16	-0.26	-0.21
P83093	Stromal interaction molecule 2 OS=Mus musculus GN=Stim2 PE=1 SV=2 - [STIM2_MOUSE]	14.21	3	5	7	18	-0.25	-0.04	-0.64	-0.52
P61222	ATP-binding cassette sub-family E member 1 OS=Mus musculus GN=Abce1 PE=2 SV=1 - [ABCE1_MOUSE]	35.39	1	16	16	78	0.18	0.24	0.06	0.14
Q9CS72	Filamin-A-interacting protein 1 OS=Mus musculus GN=Filip1 PE=1 SV=2 - [FLIP1_MOUSE]	2.97	2	2	3	4	-0.56	-0.71	-0.25	-0.41
P37913	DNA ligase 1 OS=Mus musculus GN=Lig1 PE=1 SV=2 - [DNLI1_MOUSE]	16.05	6	12	13	32	0.40	0.35	0.34	0.13
A2CI98	Dystrotelin OS=Mus musculus GN=Dytn PE=2 SV=1 - [DYTN_MOUSE]	1.38	1	1	1	1	-0.12	0.74	-0.48	0.37
Q8CF89	TGF-beta-activated kinase 1 and MAP3K7-binding protein 1 OS=Mus musculus GN=Tab1 PE=1 SV=2 - [TAB1_MOUSE]	26.49	1	7	7	17	0.16	0.33	0.21	0.19
Q9QY93	dCTP pyrophosphatase 1 OS=Mus musculus GN=Dctpp1 PE=1 SV=1 - [DCTP1_MOUSE]	27.06	1	4	4	10	-0.40	-0.47	-0.25	-0.54
E9Q2C4	Type II inositol 3,4-bisphosphate 4-phosphatase (Fragment) OS=Mus musculus GN=Inpp4b PE=2 SV=1 - [E9Q2C4_MOUSE]	8.85	6	3	3	4	0.32	0.72	-0.02	0.37
Q9CYW4	Haloacid dehalogenase-like hydrolase domain-containing protein 3 OS=Mus musculus GN=Hdhd3 PE=2 SV=1 - [HDHD3_MOUSE]	34.66	1	7	7	23	-0.36	-0.48	-0.22	-0.45
Q6ZWZ6	40S ribosomal protein S12 OS=Mus musculus GN=Rps12 PE=2 SV=1 - [Q6ZWZ6_MOUSE]	46.21	2	5	5	117	0.19	0.26	0.06	0.24
Q6TXD4	Dynamin-binding protein OS=Mus musculus GN=Dnmbp PE=1 SV=2 - [DNMBP_MOUSE]	3.80	2	4	5	16	-0.04	-0.18	-0.23	-0.12
Q9CQ10	Charged multivesicular body protein 3 OS=Mus musculus GN=Chmp3 PE=1 SV=3 - [CHMP3_MOUSE]	10.27	1	3	3	8	-0.05	-0.24	0.00	-0.06
Q91ZX7	Prolow-density lipoprotein receptor-related protein 1 OS=Mus musculus GN=Lrp1 PE=1 SV=1 - [LRP1_MOUSE]	12.15	3	45	45	180	-0.20	-0.20	-0.20	-0.22

Q9CPT5	Nucleolar protein 16 OS=Mus musculus GN=Nop16 PE=2 SV=1 - [NOP16_MOUSE]	8.43	2	2	2	3	0.12	0.07	0.06	0.01
E9Q1G8	Septin-7 OS=Mus musculus GN=Sept7 PE=3 SV=2 - [E9Q1G8_MOUSE]	52.86	3	18	20	194	0.04	0.09	-0.05	0.03
P28666	Murinoglobulin-2 OS=Mus musculus GN=Mug2 PE=2 SV=2 [MUG2_MOUSE]	13.03	1	3	17	65	0.10	-0.49	0.53	-0.06
Q8BW75	Amine oxidase [flavin-containing] B OS=Mus musculus GN=Maob PE=1 SV=4 - [AOFB_MOUSE]	49.62	3	17	21	142	-0.89	-0.76	-0.84	-0.74
Q9JI19	Acidic fibroblast growth factor intracellular-binding protein OS=Mus musculus GN=Fibp PE=2 SV=1 - [FIBP_MOUSE]	4.20	2	1	1	2	-0.58	-0.42	-0.54	-0.38
Q91WM2	Cat eye syndrome critical region protein 5 homolog OS=Mus musculus GN=Cecr5 PE=2 SV=1 - [CECR5_MOUSE]	25.30	1	8	8	21	-0.39	-0.41	-0.35	-0.25
E9Q2I4	Uncharacterized protein OS=Mus musculus GN=Elmsan1 PE=4 SV=1 - [E9Q2I4_MOUSE]	3.03	1	2	2	2	0.09	0.17	0.08	0.16
G5E884	Serine/threonine-protein kinase PAK 1 OS=Mus musculus GN=Pak1 PE=4 SV=1 - [G5E884_MOUSE]	38.97	2	10	14	102	-0.14	0.06	-0.19	0.00
Q8JZX4	Splicing factor 45 OS=Mus musculus GN=Rbm17 PE=1 SV=1 - [SPF45_MOUSE]	15.56	2	6	6	16	-0.05	0.07	-0.09	0.12
Q9WTX8	Mitotic spindle assembly checkpoint protein MAD1 OS=Mus musculus GN=Mad1l1 PE=2 SV=1 - [MD1L1_MOUSE]	9.07	2	6	6	15	0.10	-0.01	-0.15	0.01
Q9JHC0	Glutathione peroxidase 2 OS=Mus musculus GN=Gpx2 PE=2 SV=3 - [GPX2_MOUSE]	67.89	1	12	12	182	0.23	0.19	0.28	0.25
Q9CZC8	Secernin-1 OS=Mus musculus GN=Scrn1 PE=1 SV=1 - [SCRN1_MOUSE]	17.15	2	5	5	11	-0.56	-0.44	-0.28	-0.15
Q3ULD5	Methylcrotonoyl-CoA carboxylase beta chain, mitochondrial OS=Mus musculus GN=Mccc2 PE=2 SV=1 - [MCCB_MOUSE]	28.24	1	12	12	56	-0.48	-0.38	-0.56	-0.53
Q3U569	Protein Ankrd12 (Fragment) OS=Mus musculus GN=Ankrd12 PE=2 SV=1 - [Q3U569_MOUSE]	6.80	2	1	1	1	-0.06	-0.40	-0.20	-0.54
O88273	Gremlin-2 OS=Mus musculus GN=Grem2 PE=1 SV=1 - [GREM2_MOUSE]	16.07	1	2	2	3	-0.15	-0.34	0.12	-0.07

P61759	Prefoldin subunit 3 OS=Mus musculus GN=Vbp1 PE=2 SV=2 - [PFD3_MOUSE]	47.45	2	9	10	53	-0.02	0.08	-0.02	0.05
Q9DD18-2	Isoform 2 of D-tyrosyl-tRNA(Tyr) deacylase 1 OS=Mus musculus GN=Dtd1 - [DTD1_MOUSE]	24.38	2	3	3	5	-1.08	-0.09	-1.07	-0.20
Q9ET78	Junctophilin-2 OS=Mus musculus GN=Jph2 PE=1 SV=2 - [JPH2_MOUSE]	24.86	1	10	10	39	-0.40	-0.42	-0.38	-0.39
Q9QYC7	Vitamin K-dependent gamma-carboxylase OS=Mus musculus GN=Ggcx PE=2 SV=1 - [VKGC_MOUSE]	3.30	1	2	2	5	-0.02	-0.53	-0.17	-0.26
Q9JMA1	Ubiquitin carboxyl-terminal hydrolase 14 OS=Mus musculus GN=Usp14 PE=1 SV=3 - [UBP14_MOUSE]	30.83	2	16	16	95	0.23	0.40	0.12	0.27
Q8BJH1-2	Isoform 2 of Zinc finger C2HC domain-containing protein 1A OS=Mus musculus GN=Zc2hc1a - [ZC21A_MOUSE]	15.28	2	5	5	10	0.31	0.35	0.24	0.25
Q8BX80	Cytosolic endo-beta-N-acetylglucosaminidase OS=Mus musculus GN=Engase PE=2 SV=1 - [ENASE_MOUSE]	12.94	3	4	4	9	0.15	0.00	0.23	0.08
Q9QZM0	Ubiquilin-2 OS=Mus musculus GN=Ubqln2 PE=1 SV=2 - [UBQL2_MOUSE]	15.83	1	4	7	70	0.13	-0.07	-0.30	-0.15
P20352	Tissue factor OS=Mus musculus GN=F3 PE=1 SV=2 - [TF_MOUSE]	13.95	1	4	4	11	0.07	0.13	0.16	0.28
Q99KI0	Aconitate hydratase, mitochondrial OS=Mus musculus GN=Aco2 PE=1 SV=1 - [ACON_MOUSE]	54.87	1	42	42	1063	-0.47	-0.44	-0.47	-0.44
Q8BP71-6	Isoform 6 of RNA binding protein fox-1 homolog 2 OS=Mus musculus GN=Rbfox2 - [RFOX2_MOUSE]	6.53	21	2	2	8	-0.40	-0.40	-0.20	-0.31
Q9CQ48	NudC domain-containing protein 2 OS=Mus musculus GN=Nudcd2 PE=1 SV=1 - [NUDC2_MOUSE]	17.83	2	2	2	19	-0.15	0.01	0.04	-0.21
P26516	26S proteasome non-ATPase regulatory subunit 7 OS=Mus musculus GN=Psmd7 PE=1 SV=2 - [PSMD7_MOUSE]	24.92	1	6	6	34	0.19	0.21	0.18	0.09
Q4FZH1	Protein FAM189A2 OS=Mus musculus GN=Fam189a2 PE=1 SV=2 - [F1892_MOUSE]	5.17	2	1	1	2	0.73	0.54	0.50	0.31
E9PX60	Fibroblast growth factor receptor (Fragment) OS=Mus musculus GN=Fgfr2 PE=2 SV=1 - [E9PX60_MOUSE]	4.46	46	2	3	24	-0.40	-0.80	-0.57	-0.86

Q8BJ05-2	Isoform 2 of Zinc finger CCCH domain-containing protein 14 OS=Mus musculus GN=Zc3h14 - [ZC3HE_MOUSE]	11.05	3	5	5	8	0.02	0.35	-0.04	0.33
Q64133	Amine oxidase [flavin-containing] A OS=Mus musculus GN=Maoa PE=1 SV=3 - [AOFA_MOUSE]	46.58	5	19	24	317	-0.18	-0.16	-0.19	-0.19
P03921	NADH-ubiquinone oxidoreductase chain 5 OS=Mus musculus GN=Mtnd5 PE=3 SV=2 - [NU5M_MOUSE]	3.95	2	2	2	13	-0.21	-0.18	-0.44	-0.51
Q9CZN8	Glutamyl-tRNA(Gln) amidotransferase subunit A, mitochondrial OS=Mus musculus GN=Qrsl1 PE=2 SV=1 - [GATA_MOUSE]	14.48	1	6	6	15	0.10	0.32	-0.01	0.18
P49722	Proteasome subunit alpha type-2 OS=Mus musculus GN=Psma2 PE=1 SV=3 - [PSA2_MOUSE]	54.70	1	10	10	103	-0.05	-0.03	-0.10	-0.13
E9QNP0	KxDL motif-containing protein 1 OS=Mus musculus GN=Kxd1 PE=2 SV=1 - [E9QNP0_MOUSE]	33.62	9	2	12	406	1.17	1.69	1.18	1.39
H3BJP2	S-formylglutathione hydrolase (Fragment) OS=Mus musculus GN=Esd PE=2 SV=1 - [H3BJP2_MOUSE]	42.51	8	8	8	110	-0.20	-0.21	-0.22	-0.27
F7AA26	Protein Gm20459 (Fragment) OS=Mus musculus GN=Gm20459 PE=4 SV=1 - [F7AA26_MOUSE]	15.22	10	11	12	65	-0.11	0.01	0.02	-0.01
Q9Z0M1	Enamel matrix serine proteinase 1 (Precursor) OS=Mus musculus GN=Klk4 PE=2 SV=1 - [Q9Z0M1_MOUSE]	9.41	1	1	1	3	0.09	0.09	-0.15	-0.16
Q8QZT1	Acetyl-CoA acetyltransferase, mitochondrial OS=Mus musculus GN=Acat1 PE=1 SV=1 - [THIL_MOUSE]	43.87	1	16	16	442	-0.35	-0.23	-0.33	-0.23
Q8BGU2	Cerebellin-2 OS=Mus musculus GN=Cbln2 PE=1 SV=1 - [CBLN2_MOUSE]	5.80	1	1	1	1	0.06	0.00	0.12	0.07
Q3UZA1-2	Isoform 2 of CapZ-interacting protein OS=Mus musculus GN=Rcsd1 - [CPZIP_MOUSE]	7.59	2	2	2	5	-0.33	-0.45	0.18	0.06
P12265	Beta-glucuronidase OS=Mus musculus GN=Gusb PE=2 SV=2 - [BGLR_MOUSE]	27.16	3	14	14	66	-0.18	-0.23	-0.09	-0.09
Q9R0X2	ADAM DEC1 OS=Mus musculus GN=Adamdec1 PE=2 SV=1 - [ADEC1_MOUSE]	3.21	1	1	1	2	0.17	0.06	0.17	0.06

J3KMG9	TATA box-binding protein-associated factor RNA polymerase I subunit A OS=Mus musculus GN=Taf1a PE=4 SV=1 - [J3KMG9_MOUSE]	2.43	2	1	1	4	0.01	0.03	-0.34	-0.32
Q3U5C8	Rho guanine nucleotide exchange factor 16 OS=Mus musculus GN=Arhgef16 PE=1 SV=3 - [ARHGG_MOUSE]	16.83	3	9	9	32	0.06	0.02	-0.04	0.02
Q61191	Host cell factor 1 OS=Mus musculus GN=Hcfc1 PE=1 SV=2 [HCFC1_MOUSE]	10.07	3	15	16	61	-0.03	0.08	-0.09	0.10
Q9DBL1	Short/branched chain specific acyl-CoA dehydrogenase, mitochondrial OS=Mus musculus GN=Acadsb PE=1 SV=1 - [ACDSB_MOUSE]	24.77	2	9	9	21	-0.21	-0.49	-0.32	-0.56
P21784	V(D)J recombination-activating protein 2 OS=Mus musculus GN=Rag2 PE=1 SV=2 - [RAG2_MOUSE]	3.98	1	1	1	1	-0.10	-0.25	-0.09	-0.25
A4UUI2	Guanylate binding protein 1 OS=Mus musculus GN=Gbp2b PE=2 SV=1 - [A4UUI2_MOUSE]	2.21	1	1	1	1	0.50	0.28	-0.01	-0.24
P97376	Protein FRG1 OS=Mus musculus GN=Frg1 PE=1 SV=2 - [FRG1_MOUSE]	15.50	1	3	3	12	0.37	0.15	0.29	0.17
O35066	Kinesin-like protein KIF3C OS=Mus musculus GN=Kif3c PE=2 SV=3 - [KIF3C_MOUSE]	3.02	7	2	3	4	-0.14	-0.25	-0.17	-0.27
P51859	Hepatoma-derived growth factor OS=Mus musculus GN=Hdgf PE=1 SV=2 - [HDGF_MOUSE]	50.21	3	11	12	90	0.10	0.11	0.07	0.09
Q8CHX7	Raftlin-2 OS=Mus musculus GN=Rftn2 PE=2 SV=3 - [RFTN2_MOUSE]	7.20	1	2	2	2	-0.35	-0.50	-0.02	-0.17
P25425-10	Isoform 10 of POU domain, class 2, transcription factor 1 OS=Mus musculus GN=Pou2f1 - [PO2F1_MOUSE]	6.51	30	2	2	2	0.01	0.29	0.18	0.47
O35386	Phytanoyl-CoA dioxygenase, peroxisomal OS=Mus musculus GN=Phyh PE=1 SV=1 - [PAHX_MOUSE]	8.58	1	2	2	2	-0.53	-0.36	-0.06	0.11
Q8VDY9	Caspase activity and apoptosis inhibitor 1 OS=Mus musculus GN=Caap1 PE=1 SV=2 - [CAAP1_MOUSE]	23.03	1	3	3	6	0.13	-0.36	0.85	0.15
Q9D061	Acyl-CoA-binding domain-containing protein 6 OS=Mus musculus GN=Acbd6 PE=1 SV=2 - [ACBD6_MOUSE]	7.45	3	2	2	5	0.02	-0.03	0.19	0.14

Q02614	SAP30-binding protein OS=Mus musculus GN=Sap30bp PE=2 SV=2 - [S30BP_MOUSE]	22.40	1	5	5	11	0.00	-0.01	0.06	0.04
Q9EPQ7	StAR-related lipid transfer protein 5 OS=Mus musculus GN=Stard5 PE=2 SV=2 - [STAR5_MOUSE]	23.47	2	4	4	17	-0.22	-0.05	-0.06	0.11
Q3U332	Ras and Rab interactor 3 OS=Mus musculus GN=Rin3 PE=2 SV=1 - [Q3U332_MOUSE]	2.00	2	2	2	4	-0.02	-0.01	-0.23	-0.22
E9Q1T5	MARVEL domain-containing protein 2 OS=Mus musculus GN=Marveld2 PE=2 SV=1 - [E9Q1T5_MOUSE]	3.96	2	1	1	4	0.32	-0.05	-0.07	-0.43
Q8BJZ4	28S ribosomal protein S35, mitochondrial OS=Mus musculus GN=Mrps35 PE=2 SV=2 - [RT35_MOUSE]	33.44	1	8	8	39	0.04	0.08	0.10	0.19
P26443	Glutamate dehydrogenase 1, mitochondrial OS=Mus musculus GN=Glud1 PE=1 SV=1 - [DHE3_MOUSE]	48.75	3	29	29	453	-0.24	-0.15	-0.27	-0.19
O35074	Prostacyclin synthase OS=Mus musculus GN=Ptgis PE=2 SV=1 - [PTGIS_MOUSE]	18.16	2	7	7	40	-0.55	-0.65	-0.54	-0.73
D3Z3D2	Nuclear cap-binding protein subunit 2 OS=Mus musculus GN=Ncbp2 PE=2 SV=1 - [D3Z3D2_MOUSE]	18.45	2	2	2	10	0.06	0.08	0.01	0.05
A2AKV7	Alpha-ketoglutarate-dependent dioxygenase alkB homolog 3 (Fragment) OS=Mus musculus GN=Alkbh3 PE=2 SV=1 - [A2AKV7_MOUSE]	14.29	3	2	2	3	0.36	0.42	0.43	0.49
A2APB1	Ras association domain-containing protein 2 (Fragment) OS=Mus musculus GN=Rassf2 PE=2 SV=1 - [A2APB1_MOUSE]	6.93	2	1	1	2	0.50	0.38	0.61	0.49
Q9QYB8	Beta-adducin OS=Mus musculus GN=Add2 PE=1 SV=4 - [ADDB_MOUSE]	2.21	3	1	2	11	-0.29	0.08	0.00	0.37
E9Q4G7	Casein kinase I isoform alpha OS=Mus musculus GN=Csnk1a1 PE=2 SV=1 - [E9Q4G7_MOUSE]	37.50	8	10	10	26	0.17	-0.17	0.06	-0.10
O09044	Synaptosomal-associated protein 23 OS=Mus musculus GN=Snap23 PE=1 SV=1 - [SNP23_MOUSE]	64.29	5	9	10	50	-0.18	-0.12	-0.24	-0.05
Q8BIJ6	Isoleucine--tRNA ligase, mitochondrial OS=Mus musculus GN=Iars2 PE=2 SV=1 - [SYIM_MOUSE]	26.38	3	23	23	115	-0.14	-0.17	-0.14	-0.19

D3YXK2	Scaffold attachment factor B1 OS=Mus musculus GN=Safb PE=1 SV=2 - [SAFB1_MOUSE]	25.93	2	9	15	82	0.35	0.21	0.19	0.33
Q9CPY1	39S ribosomal protein L51, mitochondrial OS=Mus musculus GN=Mrpl51 PE=2 SV=1 - [RM51_MOUSE]	17.97	1	2	2	2	0.30	0.19	0.53	0.42
Q9JLI6	Selenocysteine lyase OS=Mus musculus GN=Scly PE=1 SV=1 - [SCLY_MOUSE]	20.60	7	6	6	34	-0.43	-0.17	-0.30	-0.37
P42128	Forkhead box protein K1 OS=Mus musculus GN=Foxk1 PE=1 SV=2 - [FOXK1_MOUSE]	9.46	1	2	3	14	0.23	0.22	0.03	-0.09
Q8VFZ8	MCG1048721 OS=Mus musculus GN=Olfr781 PE=3 SV=1 - [Q8VFZ8_MOUSE]	2.57	2	1	1	1	-0.39	-0.74	-0.24	-0.60
P10637	Microtubule-associated protein tau OS=Mus musculus GN=Mapt PE=1 SV=3 - [TAU_MOUSE]	11.87	11	6	6	13	-0.16	-0.38	-0.28	-0.37
Q9WUM3	Coronin-1B OS=Mus musculus GN=Coro1b PE=1 SV=1 - [COR1B_MOUSE]	31.61	2	15	15	226	-0.10	-0.06	-0.10	0.00
P17183	Gamma-enolase OS=Mus musculus GN=Eno2 PE=1 SV=2 - [ENOG_MOUSE]	43.55	8	7	13	222	-0.36	-0.37	-0.34	-0.29
Q5ND29	Rab-interacting lysosomal protein OS=Mus musculus GN=Rilp PE=1 SV=1 - [RILP_MOUSE]	5.15	1	1	2	4	0.04	-0.11	-0.08	-0.23
Q9D554	Splicing factor 3A subunit 3 OS=Mus musculus GN=Sf3a3 PE=2 SV=2 - [SF3A3_MOUSE]	30.54	1	14	14	66	0.14	0.22	0.09	0.09
A2AI08	Taperin OS=Mus musculus GN=Tprn PE=1 SV=1 - [TPRN_MOUSE]	4.81	1	3	3	8	0.07	-0.22	-0.05	-0.25
F6ZYX6	Lariat debranching enzyme (Fragment) OS=Mus musculus GN=Dbr1 PE=4 SV=1 - [F6ZYX6_MOUSE]	19.87	4	2	2	6	-0.02	-0.05	0.17	0.14
O09159	Lysosomal alpha-mannosidase OS=Mus musculus GN=Man2b1 PE=2 SV=4 - [MA2B1_MOUSE]	13.72	1	12	12	58	-0.41	-0.55	-0.34	-0.58
E9Q1Z0	Protein 4732456N10Rik OS=Mus musculus GN=4732456N10Rik PE=3 SV=1 - [E9Q1Z0_MOUSE]	24.54	2	3	9	291	2.40	2.05	-0.23	-0.46
O55106	Striatin OS=Mus musculus GN=Strn PE=1 SV=2 - [STRN_MOUSE]	17.69	2	2	10	37	0.06	-0.37	-0.01	-0.27
A2ALR9	Ubiquitin carboxyl-terminal hydrolase 48 OS=Mus musculus GN=Usp48 PE=2 SV=1 - [A2ALR9_MOUSE]	3.47	11	3	4	4	0.22	0.28	-0.13	-0.08

Q60649	Caseinolytic peptidase B protein homolog OS=Mus musculus GN=Clpb PE=1 SV=1 - [CLPB_MOUSE]	12.85	3	7	7	12	0.07	0.33	-0.21	0.10
P02088	Hemoglobin subunit beta-1 OS=Mus musculus GN=Hbb-b1 PE=1 SV=2 - [HBB1_MOUSE]	56.46	1	1	8	937	0.60	0.65	0.63	0.67
Q9D753	Exosome complex component RRP43 OS=Mus musculus GN=Exosc8 PE=2 SV=1 - [EXOS8_MOUSE]	12.32	3	3	3	8	-0.05	-0.33	0.21	-0.24
E9Q357	Protein Gm17673 OS=Mus musculus GN=Gm17673 PE=4 SV=2 - [E9Q357_MOUSE]	4.61	1	2	2	3	0.67	0.88	0.26	0.47
Q8CHW5	Bicaudal D-related protein 2 OS=Mus musculus GN=Ccdc64b PE=1 SV=1 - [BICR2_MOUSE]	10.65	1	2	3	7	0.07	-0.21	0.02	-0.24
P54276	DNA mismatch repair protein Msh6 OS=Mus musculus GN=Msh6 PE=1 SV=3 - [MSH6_MOUSE]	7.14	1	8	8	14	0.29	0.27	0.34	0.36
D3Z564	TGF-beta-activated kinase 1 and MAP3K7-binding protein 2 (Fragment) OS=Mus musculus GN=Tab2 PE=2 SV=1 - [D3Z564_MOUSE]	1.56	3	1	1	1	-0.32	-1.03	0.00	-0.71
Q9Z1Q5	Chloride intracellular channel protein 1 OS=Mus musculus GN=Clic1 PE=1 SV=3 - [CLIC1_MOUSE]	60.17	1	11	12	218	0.18	0.32	0.26	0.28
Q9DD20	Methyltransferase-like protein 7B OS=Mus musculus GN=Mettl7b PE=2 SV=2 - [MET7B_MOUSE]	21.72	1	6	6	29	-0.53	-0.73	-0.51	-0.67
Q9CVD2	Ataxin-3 OS=Mus musculus GN=Atxn3 PE=1 SV=2 - [ATX3_MOUSE]	5.07	1	1	1	2	-0.18	-0.31	-0.09	-0.22
Q9CW46	Ribonucleoprotein PTB-binding 1 OS=Mus musculus GN=Raver1 PE=1 SV=2 - [RAVR1_MOUSE]	14.30	2	6	6	39	-0.13	0.08	0.04	0.08
Q8BUG5-2	Isoform 2 of Calcyphosin-2 OS=Mus musculus GN=Caps2 - [CAYP2_MOUSE]	2.82	4	1	1	2	0.36	-0.36	2.30	1.58
E9PZI9	OX-2 membrane glycoprotein OS=Mus musculus GN=Cd200 PE=2 SV=1 - [E9PZI9_MOUSE]	17.83	5	2	2	3	-0.19	-0.80	-0.26	-0.88
Q8JZY2	COMM domain-containing protein 10 OS=Mus musculus GN=Commd10 PE=2 SV=1 - [COMDA_MOUSE]	9.90	1	2	2	4	-0.25	-0.09	0.14	0.29
G3UZ30	Protein phosphatase 1 regulatory subunit 11 (Fragment) OS=Mus musculus GN=Ppp1r11 PE=2 SV=2 - [G3UZ30_MOUSE]	36.67	3	3	3	13	0.01	-0.30	-0.09	-0.25

Q9DBT5	AMP deaminase 2 OS=Mus musculus GN=Ampd2 PE=1 SV=1 - [AMPD2_MOUSE]	11.53	5	9	9	24	-0.16	-0.38	-0.11	-0.16
Q8VE37	Regulator of chromosome condensation OS=Mus musculus GN=Rcc1 PE=1 SV=1 - [RCC1_MOUSE]	32.07	3	9	9	55	-0.18	-0.23	0.06	0.09
Q8K2Q0	COMM domain-containing protein 9 OS=Mus musculus GN=Commd9 PE=2 SV=3 - [COMD9_MOUSE]	29.29	1	5	5	23	0.15	0.03	0.08	-0.17
D6RIN4	Dynein light chain 2, cytoplasmic OS=Mus musculus GN=Dynll2 PE=2 SV=1 - [D6RIN4_MOUSE]	62.26	2	3	3	76	-0.40	-0.18	-0.45	-0.26
Q8BW84	Glutamyl-tRNA(Gln) amidotransferase subunit B, mitochondrial OS=Mus musculus GN=Pet112 PE=2 SV=1 - [Q8BW84_MOUSE]	11.39	3	5	5	6	0.04	0.43	-0.14	0.44
P34914	Bifunctional epoxide hydrolase 2 OS=Mus musculus GN=Ephx2 PE=1 SV=2 - [HYES_MOUSE]	24.73	2	8	8	20	-0.53	-0.34	-0.43	-0.11
Q8C8R3-2	Isoform 2 of Ankyrin-2 OS=Mus musculus GN=Ank2 - [ANK2_MOUSE]	11.92	7	25	34	93	-0.42	-0.38	-0.36	-0.42
P28659-2	Isoform 2 of CUGBP Elav-like family member 1 OS=Mus musculus GN=Celf1 - [CELF1_MOUSE]	14.32	4	5	5	12	-0.06	-0.21	-0.09	-0.17
Q8VCN5	Cystathionine gamma-lyase OS=Mus musculus GN=Cth PE=1 SV=1 - [CGL_MOUSE]	37.19	1	12	12	126	-0.78	-0.76	-0.64	-0.68
Q9QXD1	Peroxisomal acyl-coenzyme A oxidase 2 OS=Mus musculus GN=Acox2 PE=1 SV=2 - [ACOX2_MOUSE]	3.38	1	2	2	4	-0.17	-0.15	-0.09	-0.06
A2AMZ4	Uncharacterized protein C17orf89 homolog OS=Mus musculus PE=4 SV=1 - [CQ089_MOUSE]	10.81	1	1	1	1	-0.20	0.33	-0.55	-0.03
Q9R1T4-2	Isoform I of Septin-6 OS=Mus musculus GN=Sept6 - [SEPT6_MOUSE]	36.77	4	6	13	76	-0.05	-0.16	-0.04	-0.11
P40630-2	Isoform Nuclear of Transcription factor A, mitochondrial OS=Mus musculus GN=Tfam - [TFAM_MOUSE]	33.02	3	8	8	51	-0.14	-0.04	-0.15	0.09
Q8K2C6	NAD-dependent protein deacylase sirtuin-5, mitochondrial OS=Mus musculus GN=Sirt5 PE=1 SV=1 - [SIR5_MOUSE]	13.23	1	3	3	7	-0.29	-0.24	-0.23	-0.27
Q7TNV0	Protein DEK OS=Mus musculus GN=Dek PE=1 SV=1 - [DEK_MOUSE]	21.58	3	10	10	63	0.27	0.17	0.22	0.19

G3UXR7	Coiled-coil domain-containing protein 9 (Fragment) OS=Mus musculus GN=Ccdc9 PE=2 SV=1 - [G3UXR7_MOUSE]	12.12	4	1	1	2	-0.03	0.10	-0.18	-0.05
A2A8L5	Receptor-type tyrosine-protein phosphatase F OS=Mus musculus GN=Ptprf PE=1 SV=1 - [PTPRF_MOUSE]	11.22	4	12	13	42	-0.47	-0.63	-0.25	-0.33
Q9CPW3	39S ribosomal protein L54, mitochondrial OS=Mus musculus GN=Mrpl54 PE=2 SV=1 - [RM54_MOUSE]	28.89	1	2	2	3	0.75	1.50	0.48	1.24
Q9CQ62	2,4-dienoyl-CoA reductase, mitochondrial OS=Mus musculus GN=Decr1 PE=1 SV=1 - [DECR_MOUSE]	29.55	1	11	11	243	-0.37	-0.35	-0.36	-0.34
Q64010	Adapter molecule crk OS=Mus musculus GN=Crk PE=1 SV=1 - [CRK_MOUSE]	48.68	6	12	12	71	-0.28	-0.23	-0.16	-0.26
Q91WA6-2	Isoform 2 of Sharpin OS=Mus musculus GN=Sharpin - [SHRPN_MOUSE]	13.11	3	3	3	3	-0.02	0.16	-0.19	-0.02
Q3UMF0-3	Isoform 3 of Cordon-bleu protein-like 1 OS=Mus musculus GN=Cobll1 - [COBL1_MOUSE]	19.55	9	15	15	43	-0.02	0.02	0.01	0.05
P61164	Alpha-centractin OS=Mus musculus GN=Actr1a PE=2 SV=1 - [ACTZ_MOUSE]	41.76	2	4	13	223	-0.11	-0.12	-0.23	-0.21
P56375	Acylphosphatase-2 OS=Mus musculus GN=Acyp2 PE=2 SV=2 - [ACYP2_MOUSE]	33.96	1	3	3	15	-0.44	-0.29	-0.42	-0.21
P11930	Nucleoside diphosphate-linked moiety X motif 19, mitochondrial OS=Mus musculus GN=Nudt19 PE=1 SV=2 - [NUD19_MOUSE]	33.05	1	6	6	59	-0.02	-0.14	-0.01	-0.11
Q9DCB8	Iron-sulfur cluster assembly 2 homolog, mitochondrial OS=Mus musculus GN=Isca2 PE=2 SV=2 - [ISCA2_MOUSE]	33.77	1	5	5	18	-0.59	-0.69	-0.60	-0.64
Q91VZ6	Stromal membrane-associated protein 1 OS=Mus musculus GN=Smap1 PE=1 SV=1 - [SMAP1_MOUSE]	15.45	2	6	6	36	0.02	0.01	0.05	0.16
E9Q9X4	Nitric oxide synthase OS=Mus musculus GN=Nos3 PE=2 SV=1 - [E9Q9X4_MOUSE]	1.54	2	2	2	3	-0.21	-0.61	-0.31	-0.72
Q7M739	Nuclear pore complex-associated intranuclear coiled-coil protein TPR OS=Mus musculus GN=Tpr PE=2 SV=1 - [Q7M739_MOUSE]	24.73	8	50	52	217	-0.16	-0.10	-0.14	-0.09

Q9JL35	High mobility group nucleosome-binding domain-containing protein 5 OS=Mus musculus GN=Hmgn5 PE=1 SV=2 - [HMGN5_MOUSE]	30.05	1	9	9	36	-0.08	0.14	-0.17	0.14
A7DTG3	SERTA domain-containing protein 4 OS=Mus musculus GN=Sertad4 PE=2 SV=1 - [SRTD4_MOUSE]	1.86	1	1	1	1	-1.02	-0.64	-0.74	-0.36
P62746	Rho-related GTP-binding protein RhoB OS=Mus musculus GN=Rhob PE=1 SV=1 - [RHOB_MOUSE]	34.18	1	3	4	74	-0.31	-0.67	-0.30	-0.50
Q9QZ41-2	Isoform 2 of Protein DBF4 homolog A OS=Mus musculus GN=Dbf4 - [DBF4A_MOUSE]	2.27	2	1	1	2	-0.41	-0.43	-0.60	-0.68
Q8BT35	Uncharacterized protein LINC00116 homolog OS=Mus musculus PE=4 SV=1 - [YB039_MOUSE]	10.71	1	1	1	1	-0.54	-0.38	-0.53	-0.38
F6TQW2	Protein Ighg2c OS=Mus musculus GN=Ighg2c PE=4 SV=1 - [F6TQW2_MOUSE]	17.12	4	5	5	22	-1.35	-1.40	-0.98	-1.23
Q61686	Chromobox protein homolog 5 OS=Mus musculus GN=Cbx5 PE=1 SV=1 - [CBX5_MOUSE]	44.50	2	7	8	41	0.06	0.04	-0.11	-0.02
Q6A0A2	La-related protein 4B OS=Mus musculus GN=Larp4b PE=1 SV=2 - [LAR4B_MOUSE]	15.79	3	9	10	22	0.00	-0.03	-0.14	0.12
P12815	Programmed cell death protein 6 OS=Mus musculus GN=Pdcd6 PE=1 SV=2 - [PDCD6_MOUSE]	30.89	1	6	6	40	-0.18	-0.10	-0.17	-0.12
Q8BH39	MCG1032870 OS=Mus musculus GN=4930579K19Rik PE=4 SV=1 - [Q8BH39_MOUSE]	13.27	1	1	1	1	-0.22	-0.25	-0.24	-0.27
Q6WVG3	BTB/POZ domain-containing protein KCTD12 OS=Mus musculus GN=Kctd12 PE=1 SV=1 - [KCD12_MOUSE]	39.76	1	7	8	64	-0.46	-0.44	-0.33	-0.32
E9Q852	Afadin OS=Mus musculus GN=Mllt4 PE=4 SV=2 - [E9Q852_MOUSE]	22.49	6	25	32	75	-0.04	0.07	0.01	0.05
Q61687	Transcriptional regulator ATRX OS=Mus musculus GN=Atrx PE=1 SV=3 - [ATRX_MOUSE]	2.02	2	4	4	7	0.03	0.12	-0.12	-0.06
E9Q7Z8	Protein Ythdc1 OS=Mus musculus GN=Ythdc1 PE=2 SV=1 - [E9Q7Z8_MOUSE]	1.83	4	1	2	4	-0.08	-0.16	-0.07	-0.16
Q9D1J3	SAP domain-containing ribonucleoprotein OS=Mus musculus GN=Sarnp PE=1 SV=3 [SARNP_MOUSE]	36.19	1	7	7	49	0.04	0.01	0.09	0.04

Q9CS84-4	Isoform 4a of Neurexin-1 OS=Mus musculus GN=Nrxn1 - [NRX1A_MOUSE]	3.57	10	2	3	6	-0.22	-0.72	-0.21	-0.71
Q8CB77	Transcription elongation factor B polypeptide 3 OS=Mus musculus GN=Tceb3 PE=1 SV=3 [ELOA1_MOUSE]	7.76	1	6	7	16	0.03	-0.06	0.21	0.09
Q5RI75-2	Isoform 2 of Ras and EF-hand domain-containing protein homolog OS=Mus musculus GN=Rasef - [RASEF_MOUSE]	3.24	2	2	2	3	-0.06	-0.41	0.02	-0.33
Q6RHR9-2	Isoform 2 of Membrane-associated guanylate kinase, WW and PDZ domain-containing protein 1 OS=Mus musculus GN=Magi1 - [MAGI1_MOUSE]	4.95	4	3	4	16	0.28	-0.08	0.22	0.04
Q8R5A3	Amyloid beta A4 precursor protein-binding family B member 1-interacting protein OS=Mus musculus GN=Apbb1ip PE=1 SV=2 - [AB1IP_MOUSE]	9.10	1	4	4	24	0.09	0.01	0.05	0.12
Q00560	Interleukin-6 receptor subunit beta OS=Mus musculus GN=Il6st PE=1 SV=2 - [IL6RB_MOUSE]	0.65	1	1	1	1	0.02	-0.11	-0.06	-0.20
O89110	Caspase-8 OS=Mus musculus GN=Casp8 PE=1 SV=1 - [CASP8_MOUSE]	43.33	1	19	19	95	0.22	0.19	0.17	0.29
Q91VM9	Inorganic pyrophosphatase 2, mitochondrial OS=Mus musculus GN=Ppa2 PE=2 SV=1 - [IPYR2_MOUSE]	47.27	5	15	15	175	-0.30	-0.19	-0.42	-0.21
Q9CQ40	39S ribosomal protein L49, mitochondrial OS=Mus musculus GN=Mrpl49 PE=2 SV=1 - [RM49_MOUSE]	12.05	1	2	2	11	-0.03	-0.27	0.18	-0.12
E9Q5I7	Endoglin OS=Mus musculus GN=Eng PE=2 SV=1 - [E9Q5I7_MOUSE]	10.14	3	5	5	9	-0.60	-0.42	-0.25	-0.12
Q6QI06-2	Isoform 2 of Rapamycin insensitive companion of mTOR OS=Mus musculus GN=Rictor - [RICTR_MOUSE]	1.41	2	1	2	2	-0.92	-0.53	-0.19	0.20
Q9D711	Pirin OS=Mus musculus GN=Pir PE=2 SV=1 - [PIR_MOUSE]	12.07	2	2	2	10	-0.77	-0.64	-0.42	-0.29
Q6P9S1	ATM interactor OS=Mus musculus GN=Atmin PE=2 SV=2 - [ATMIN_MOUSE]	1.83	1	1	1	3	0.04	0.67	-0.08	0.54
Q6PAM1	Alpha-taxilin OS=Mus musculus GN=Txlna PE=2 SV=1 - [TXLNA_MOUSE]	22.74	12	10	10	39	0.01	0.30	0.03	0.29
Q8JZQ9	Eukaryotic translation initiation factor 3 subunit B OS=Mus musculus GN=Eif3b PE=1 SV=1 - [EIF3B_MOUSE]	35.62	1	24	24	140	0.22	0.11	0.26	0.26

Q9QZ08	N-acetyl-D- glucosamine kinase OS=Mus musculus GN=Nagk PE=2 SV=3 - [NAGK_MOUSE]	30.61	3	9	9	54	-0.16	-0.34	-0.02	-0.19
Q3UGN9	Signal transducing adapter molecule 1 OS=Mus musculus GN=Stam PE=2 SV=1 - [Q3UGN9_MOUSE]	12.34	3	3	4	16	-0.14	0.13	0.01	0.22
Q62245	Son of sevenless homolog 1 OS=Mus musculus GN=Sos1 PE=1 SV=2 - [SOS1_MOUSE]	5.38	1	3	4	4	0.07	-0.48	0.50	0.14
D3YWF6	Ubiquitin thioesterase OTUB1 OS=Mus musculus GN=Otub1 PE=2 SV=1 - [D3YWF6_MOUSE]	32.37	3	6	6	38	0.19	0.23	0.19	0.15
Q9Z0X1	Apoptosis-inducing factor 1, mitochondrial OS=Mus musculus GN=Aifm1 PE=1 SV=1 - [AIFM1_MOUSE]	27.78	2	14	14	152	-0.22	-0.15	-0.20	-0.10
Q69ZW3-2	Isoform 2 of EH domain binding protein 1 OS=Mus musculus GN=Ehbp1 - [EHBP1_MOUSE]	1.66	2	1	1	1	-0.04	0.00	-0.21	-0.17
Q3SXD3	HD domain-containing protein 2 OS=Mus musculus GN=Hddc2 PE=2 SV=1 - [HDDC2_MOUSE]	19.60	2	3	3	17	0.08	0.33	0.11	0.37
Q9D6U8	Protein FAM162A OS=Mus musculus GN=Fam162a PE=2 SV=1 - [F162A_MOUSE]	27.74	1	5	5	24	-0.23	-0.38	-0.13	-0.43
Q9D6L8	Peptidyl-prolyl cis-trans isomerase-like 3 OS=Mus musculus GN=Ppil3 PE=2 SV=1 - [PPIL3_MOUSE]	15.53	2	2	2	3	0.06	-0.33	0.01	-0.38
Q8BWR2	PITH domain-containing protein 1 OS=Mus musculus GN=Pithd1 PE=2 SV=1 - [PITH1_MOUSE]	18.48	4	4	4	25	-0.50	-0.43	-0.48	-0.43
Q920D3	Mediator of RNA polymerase II transcription subunit 28 OS=Mus musculus GN=Med28 PE=2 SV=2 - [MED28_MOUSE]	10.67	1	2	2	4	-0.25	0.04	-0.12	-0.46
P97762	Retinitis pigmentosa 9 protein homolog OS=Mus musculus GN=rp9 PE=1 SV=1 - [RP9_MOUSE]	5.16	1	1	1	1	0.20	-0.62	0.40	-0.43
Q9Z2E1	Methyl-CpG-binding domain protein 2 OS=Mus musculus GN=Mbd2 PE=2 SV=2 [MBD2_MOUSE]	17.87	3	5	7	13	-0.25	-0.17	-0.01	0.02
P26450	Phosphatidylinositol 3-kinase regulatory subunit alpha OS=Mus musculus GN=Pik3r1 PE=1 SV=2 - [P85A_MOUSE]	17.27	5	6	10	21	-0.05	-0.02	0.19	0.40

O70404	Vesicle-associated membrane protein 8 OS=Mus musculus GN=Vamp8 PE=1 SV=1 - [VAMP8_MOUSE]	18.81	1	2	2	4	-0.04	-0.07	0.33	0.29
Q9QYR6	Microtubule-associated protein 1A OS=Mus musculus GN=Map1a PE=1 SV=2 - [MAP1A_MOUSE]	5.87	3	9	10	29	-0.09	-0.14	-0.20	-0.27
O70439	Syntaxin-7 OS=Mus musculus GN=Stx7 PE=1 SV=3 - [STX7_MOUSE]	27.20	2	7	7	59	-0.07	-0.13	0.00	-0.11
Q5F2E8	Serine/threonine-protein kinase TAO1 OS=Mus musculus GN=Taok1 PE=1 SV=1 - [TAOK1_MOUSE]	5.00	3	3	5	5	0.44	0.58	0.04	-0.01
Q9QZZ4-2	Isoform 2 of Unconventional myosin-XV OS=Mus musculus GN=Myo15a - [MYO15_MOUSE]	1.37	4	3	4	19	-0.36	-0.03	-0.42	0.00
Q9CQD4	Charged multivesicular body protein 1b-2 OS=Mus musculus GN=Chmp1b2 PE=2 SV=2 - [CH1B2_MOUSE]	14.07	1	1	4	8	-0.16	-0.51	-0.13	-0.48
Q8BMS4	Hexaprenyldihydroxybenzoate methyltransferase, mitochondrial OS=Mus musculus GN=Coq3 PE=2 SV=1 - [COQ3_MOUSE]	17.84	1	4	4	10	0.03	0.10	-0.01	0.06
Q91V64	Isochorismatase domain containing protein 1 OS=Mus musculus GN=Isoc1 PE=2 SV=1 - [ISOC1_MOUSE]	47.81	1	12	12	57	-0.23	-0.26	-0.23	-0.25
Q8CG73	Protein fantom OS=Mus musculus GN=Rpgrip1l PE=1 SV=2 - [FTM_MOUSE]	5.46	5	3	5	26	0.14	0.15	0.01	0.02
P10810	Monocyte differentiation antigen CD14 OS=Mus musculus GN=Cd14 PE=1 SV=1 - [CD14_MOUSE]	7.65	1	2	2	3	-0.58	-1.23	-0.63	-1.28
Q9D824-4	Isoform 4 of Pre-mRNA 3'-end-processing factor FIP1 OS=Mus musculus GN=Fip1l1 - [FIP1_MOUSE]	11.81	7	5	5	14	-0.39	-0.16	-0.48	-0.15
G5E819	Serine/threonine-protein phosphatase OS=Mus musculus GN=Ppp5c PE=3 SV=1 - [G5E819_MOUSE]	37.27	3	17	17	68	-0.08	0.01	0.00	-0.08
P70698	CTP synthase 1 OS=Mus musculus GN=Ctps1 PE=1 SV=2 [PYRG1_MOUSE]	15.74	1	8	8	22	0.33	0.28	0.15	0.06
P33175	Kinesin heavy chain isoform 5A OS=Mus musculus GN=Kif5a PE=1 SV=3 - [KIF5A_MOUSE]	7.69	1	1	7	52	0.12	-0.15	0.19	-0.08

Q921F2	TAR DNA-binding protein 43 OS=Mus musculus GN=Tardbp PE=1 SV=1 - [TADBP_MOUSE]	17.87	5	8	8	91	0.16	0.02	0.18	0.07
Q3V1H1	Cytoskeleton-associated protein 2 OS=Mus musculus GN=Ckap2 PE=2 SV=1 - [CKAP2_MOUSE]	1.81	1	1	1	1	-0.20	-0.53	0.15	-0.18
P70697	Uroporphyrinogen decarboxylase OS=Mus musculus GN=Urod PE=1 SV=2 - [DCUP_MOUSE]	28.88	1	9	9	25	-0.20	-0.39	-0.15	-0.20
O54991	Contactin-associated protein 1 OS=Mus musculus GN=Cntnap1 PE=1 SV=2 - [CNTP1_MOUSE]	0.43	1	1	1	1	-0.13	0.17	-0.28	0.03
Q8K012-2	Isoform 2 of Formin-binding protein 1-like OS=Mus musculus GN=Fnbp1l - [FBP1L_MOUSE]	11.09	4	4	4	20	0.06	-0.23	-0.13	-0.42
Q7TQG1	Pleckstrin homology domain-containing family A member 6 OS=Mus musculus GN=Plekha6 PE=1 SV=1 - [PKHA6_MOUSE]	26.94	2	23	24	91	0.08	0.10	0.00	0.12
Q8BIV3	Ran-binding protein 6 OS=Mus musculus GN=Ranbp6 PE=2 SV=3 - [RNBP6_MOUSE]	6.88	1	3	3	6	-0.32	-0.12	-0.36	-0.19
Q01853	Transitional endoplasmic reticulum ATPase OS=Mus musculus GN=Vcp PE=1 SV=4 - [TERA_MOUSE]	52.23	8	38	39	819	0.07	0.08	0.00	0.04
E9QKG6	Ankyrin repeat domain-containing protein 17 OS=Mus musculus GN=Ankrd17 PE=2 SV=1 - [E9QKG6_MOUSE]	5.82	5	4	8	25	-1.08	-0.01	-1.18	0.08
Q9QZS3-4	Isoform 4 of Protein numb homolog OS=Mus musculus GN=Numb - [NUMB_MOUSE]	16.36	10	6	8	11	-0.16	0.34	-0.11	-0.22
P48036	Annexin A5 OS=Mus musculus GN=Anxa5 PE=1 SV=1 - [ANXA5_MOUSE]	68.34	1	22	22	452	-0.35	-0.34	-0.41	-0.36
Q61012	Guanine nucleotide-binding protein G(T) subunit gamma-T1 OS=Mus musculus GN=Gngt1 PE=1 SV=3 [GBG1_MOUSE]	17.57	1	1	2	2	-0.17	-0.06	-0.10	0.00
A2AMS2	Galactose-1-phosphate uridylyltransferase OS=Mus musculus GN=Galt PE=3 SV=1 - [A2AMS2_MOUSE]	28.65	3	5	5	19	-0.64	-0.41	-0.57	-0.23
Q9CZN7	Serine hydroxymethyltransferase OS=Mus musculus GN=Shmt2 PE=2 SV=1 - [Q9CZN7_MOUSE]	21.43	1	9	10	67	0.10	0.07	0.06	0.02

P32921-2	Isoform 2 of Tryptophan--tRNA ligase, cytoplasmic OS=Mus musculus GN=Wars - [SYWC_MOUSE]	52.00	1	1	21	256	0.13	0.02	0.36	0.25
Q9Z1B7	Mitogen-activated protein kinase 13 OS=Mus musculus GN=Mapk13 PE=1 SV=2 - [MK13_MOUSE]	50.82	7	13	14	98	-0.05	0.09	-0.03	0.04
F7D4U1	Protein Vmn2r88 (Fragment) OS=Mus musculus GN=Vmn2r88 PE=2 SV=1 - [F7D4U1_MOUSE]	4.11	5	1	3	3	-0.06	0.05	-0.18	-0.08
P54728	UV excision repair protein RAD23 homolog B OS=Mus musculus GN=Rad23b PE=1 SV=2 - [RD23B_MOUSE]	17.79	1	6	9	159	-0.08	-0.08	-0.07	-0.05
F6RPD4	Phosphorylated adapter RNA export protein (Fragment) OS=Mus musculus GN=Phax PE=4 SV=1 - [F6RPD4_MOUSE]	3.19	3	1	1	2	0.04	0.12	0.04	0.12
Q3TE40	DNA-directed RNA polymerase I subunit RPA2 OS=Mus musculus GN=Rpa2 PE=2 SV=1 - [Q3TE40_MOUSE]	7.41	2	2	2	9	-0.03	-0.18	-0.02	-0.05
Q9JM13	Rab5 GDP/GTP exchange factor OS=Mus musculus GN=Rabgef1 PE=1 SV=1 - [RABX5_MOUSE]	14.46	3	5	6	17	-0.23	0.39	-0.23	0.27
E9QAS9	Fragile X mental retardation protein 1 homolog OS=Mus musculus GN=Fmr1 PE=2 SV=1 - [E9QAS9_MOUSE]	11.45	18	4	5	13	0.15	0.34	0.22	0.41
E9Q947	Nucleolar protein 4 OS=Mus musculus GN=Nol4 PE=2 SV=1 - [E9Q947_MOUSE]	4.55	5	1	2	4	-0.54	-0.39	-0.60	-0.45
Q9Z0Z4-2	Isoform 2 of Hephaestin OS=Mus musculus GN=Heph - [HEPH_MOUSE]	20.42	3	15	16	63	-0.17	-0.31	-0.14	-0.27
Q8VEE0	Ribulose-phosphate 3-epimerase OS=Mus musculus GN=Rpe PE=2 SV=1 - [RPE_MOUSE]	28.07	4	4	5	53	0.05	0.04	0.13	0.10
A2ASQ1-3	Isoform 3 of Agrin OS=Mus musculus GN=Agrn - [AGRIN_MOUSE]	10.71	4	16	17	52	-0.98	-0.93	-0.99	-0.82
P97379-2	Isoform B of Ras GTPase-activating protein-binding protein 2 OS=Mus musculus GN=G3bp2 - [G3BP2_MOUSE]	22.72	1	1	9	42	-0.26	-0.28	-0.25	-0.28
Q99LL5	Periodic tryptophan protein 1 homolog OS=Mus musculus GN=Pwp1 PE=1 SV=1 - [PWP1_MOUSE]	5.79	2	3	3	9	0.07	0.19	0.15	0.05
Q99K30	Epidermal growth factor receptor kinase substrate 8-like protein 2 OS=Mus musculus GN=Eps8l2 PE=1 SV=1 - [ES8L2_MOUSE]	17.70	3	10	10	51	-0.34	-0.43	-0.41	-0.45

P30416	Peptidyl-prolyl cis-trans isomerase FKBP4 OS=Mus musculus GN=Fkbp4 PE=1 SV=5 - [FKBP4_MOUSE]	45.20	4	20	20	151	0.05	-0.10	0.07	-0.05
Q91XU3	Phosphatidylinositol 5-phosphate 4-kinase type 2 gamma OS=Mus musculus GN=Pip4k2c PE=2 SV=1 - [PI42C_MOUSE]	17.81	1	5	5	15	0.03	0.06	-0.28	-0.23
Q9Z2C4	Myotubularin-related protein 1 OS=Mus musculus GN=Mtmr1 PE=1 SV=1 - [MTMR1_MOUSE]	2.24	2	2	2	4	-0.67	-0.17	-0.66	-0.17
Q8K4L0	ATP-dependent RNA helicase DDX54 OS=Mus musculus GN=Ddx54 PE=1 SV=1 - [DDX54_MOUSE]	3.55	1	3	4	5	0.20	0.43	0.26	0.48
Q99L47	Hsc70-interacting protein OS=Mus musculus GN=St13 PE=2 SV=1 - [F10A1_MOUSE]	32.08	4	12	12	119	0.08	0.15	-0.02	0.03
Q6NSR8	Probable aminopeptidase NPEPL1 OS=Mus musculus GN=Npepl1 PE=2 SV=1 - [PEPL1_MOUSE]	20.23	2	8	8	23	-0.15	-0.11	-0.15	-0.14
A2APX7	Protein Scn1a OS=Mus musculus GN=Scn1a PE=2 SV=1 - [A2APX7_MOUSE]	0.40	3	1	1	1	-0.43	-0.54	-0.37	-0.48
Q99JI6	Ras-related protein Rap-1b OS=Mus musculus GN=Rap1b PE=2 SV=2 - [RAP1B_MOUSE]	63.59	1	4	10	114	0.03	0.20	-0.07	0.06
Q99JF8	PC4 and SFRS1-interacting protein OS=Mus musculus GN=Psip1 PE=1 SV=1 - [PSIP1_MOUSE]	19.70	4	11	12	65	0.19	0.38	0.26	0.48
Q60764	Probable E3 ubiquitin-protein ligase makorin-3 OS=Mus musculus GN=Mkrn3 PE=2 SV=2 - [MKRN3_MOUSE]	1.29	1	1	1	4	0.12	0.35	-0.06	0.16
Q60825	Sodium-dependent phosphate transport protein 2A OS=Mus musculus GN=Slc34a1 PE=1 SV=2 - [NPT2A_MOUSE]	2.20	4	1	2	2	0.22	0.71	-0.10	0.40
Q7TSI3	Serine/threonine-protein phosphatase 6 regulatory subunit 1 OS=Mus musculus GN=Ppp6r1 PE=1 SV=1 - [PP6R1_MOUSE]	10.75	1	6	6	14	-0.16	-0.43	0.04	-0.29
P55264-2	Isoform Short of Adenosine kinase OS=Mus musculus GN=Adk - [ADK_MOUSE]	40.00	2	15	15	226	-0.35	-0.28	-0.42	-0.35
Q8K2I1	Protein farnesyltransferase subunit beta OS=Mus musculus GN=Fntb PE=1 SV=1 - [FNTB_MOUSE]	7.55	2	3	3	11	0.14	0.55	0.10	0.31

F8WJI3	Coiled-coil domain-containing protein 58 OS=Mus musculus GN=Ccdc58 PE=2 SV=1 - [F8WJI3_MOUSE]	48.51	3	7	7	31	-0.22	-0.22	-0.25	-0.37
Q62440-3	Isoform 3 of Transducin like enhancer protein 1 OS=Mus musculus GN=Tle1 - [TLE1_MOUSE]	6.00	29	4	4	8	-0.32	-0.52	-0.05	-0.32
E9QME5	E3 ubiquitin-protein ligase TRIM33 OS=Mus musculus GN=Trim33 PE=2 SV=1 - [E9QME5_MOUSE]	3.29	4	3	4	9	-0.04	-0.22	0.01	0.00
Q8R105	Vacuolar protein sorting associated protein 37C OS=Mus musculus GN=Vps37c PE=2 SV=1 - [VP37C_MOUSE]	22.44	2	5	5	21	-0.06	0.19	-0.18	0.03
Q7TNL9	Coiled-coil-helix-coiled-coil-helix domain containing 10 OS=Mus musculus GN=Chchd10 PE=2 SV=1 - [Q7TNL9_MOUSE]	6.52	1	1	1	1	-0.46	0.03	-0.39	0.10
P79621-3	Isoform 3 of MHC class II transactivator OS=Mus musculus GN=Ciita - [C2TA_MOUSE]	1.23	3	1	1	1	-0.11	0.62	-0.46	0.27
P63038	60 kDa heat shock protein, mitochondrial OS=Mus musculus GN=Hspd1 PE=1 SV=1 - [CH60_MOUSE]	74.52	4	41	41	1519	0.10	0.15	0.06	0.09
Q9D0B0	Serine/arginine-rich splicing factor 9 OS=Mus musculus GN=Srsf9 PE=1 SV=1 - [SRSF9_MOUSE]	13.51	2	3	3	18	-0.36	-0.45	-0.24	-0.27
O09053	Werner syndrome ATP-dependent helicase homolog OS=Mus musculus GN=Wrn PE=1 SV=3 - [WRN_MOUSE]	4.93	1	3	3	3	-0.35	0.73	-0.55	0.41
Q3UTH8-2	Isoform 2 of Rho guanine nucleotide exchange factor 9 OS=Mus musculus GN=Arhgef9 - [ARHG9_MOUSE]	2.63	6	2	2	2	0.04	0.32	-0.02	0.25
Q8C2T6	DNA polymerase alpha subunit B OS=Mus musculus GN=Pola2 PE=2 SV=1 - [Q8C2T6_MOUSE]	11.31	2	4	5	16	0.31	0.28	0.17	0.22
B2RQG2	PHD finger protein 3 OS=Mus musculus GN=Phf3 PE=2 SV=1 - [B2RQG2_MOUSE]	3.11	1	2	3	9	0.01	-0.14	-0.11	0.02
Q9CQJ6	Density-regulated protein OS=Mus musculus GN=Denr PE=2 SV=1 - [DENR_MOUSE]	20.20	2	4	4	25	-0.04	-0.04	-0.13	-0.08
B1AYG7	Integrin beta-6 (Fragment) OS=Mus musculus GN=Itgb6 PE=2 SV=1 - [B1AYG7_MOUSE]	1.19	3	1	1	2	0.21	0.39	0.17	0.35
Q61555	Fibrillin-2 OS=Mus musculus GN=Fbn2 PE=1 SV=2 - [FBN2_MOUSE]	1.58	1	2	3	8	0.03	0.06	-0.28	-0.26

Q8K2F0	Bromodomain-containing protein 3 OS=Mus musculus GN=Brd3 PE=1 SV=2 - [BRD3_MOUSE]	8.40	3	3	5	14	0.22	0.27	0.19	0.29
J3QP81	CLIP-associating protein 1 OS=Mus musculus GN=Clasp1 PE=4 SV=1 - [J3QP81_MOUSE]	8.02	6	10	10	17	0.10	-0.11	0.06	-0.15
Q9DCL2	MIP18 family protein FAM96A OS=Mus musculus GN=Fam96a PE=2 SV=1 - [FA96A_MOUSE]	12.50	1	2	2	8	-0.07	-0.09	-0.17	-0.18
Q9QXN0-4	Isoform 4 of Protein Shroom3 OS=Mus musculus GN=Shroom3 - [SHRM3_MOUSE]	8.09	7	10	11	33	0.15	0.11	-0.15	0.15
Q9R061	Cytosolic Fe-S cluster assembly factor NUBP2 OS=Mus musculus GN=Nubp2 PE=1 SV=1 - [NUBP2_MOUSE]	13.09	1	2	3	6	0.02	-0.27	0.00	-0.04
Q8BGC0	HIV Tat-specific factor 1 homolog OS=Mus musculus GN=Htatsf1 PE=1 SV=1 - [HTSF1_MOUSE]	15.46	2	6	7	24	0.12	0.01	0.09	0.22
P35441	Thrombospondin-1 OS=Mus musculus GN=Thbs1 PE=1 SV=1 - [TSP1_MOUSE]	5.47	2	7	7	17	0.24	-0.08	0.20	-0.03
Q3UH85	Protein 8430427H17Rik OS=Mus musculus GN=8430427H17Rik PE=2 SV=1 - [Q3UH85_MOUSE]	12.37	5	5	6	12	-0.01	-0.45	0.02	-0.42
Q99JF5	Diphosphomevalonate decarboxylase OS=Mus musculus GN=Mvd PE=1 SV=2 - [MVD1_MOUSE]	30.42	1	8	8	37	0.30	0.44	0.39	0.39
Q8R344	Coiled-coil domain-containing protein 12 OS=Mus musculus GN=Ccdc12 PE=1 SV=2 - [CCD12_MOUSE]	19.28	1	3	3	12	0.38	0.45	0.36	0.29
Q8K348	Activin receptor type-1C OS=Mus musculus GN=Acvr1c PE=2 SV=3 - [ACV1C_MOUSE]	3.45	1	1	1	2	-0.72	-0.98	0.30	0.04
P28740-2	Isoform 2 of Kinesin-like protein KIF2A OS=Mus musculus GN=Kif2a - [KIF2A_MOUSE]	16.39	7	8	9	29	-0.17	0.20	-0.06	0.10
F8WGT1	Adenosylhomocysteinase OS=Mus musculus GN=Ahcyl2 PE=2 SV=1 - [F8WGT1_MOUSE]	32.84	10	2	21	223	0.07	-0.28	0.19	-0.19
Q8BH15-2	Isoform 2 of CCR4-NOT transcription complex subunit 10 OS=Mus musculus GN=Cnot10 - [CNO10_MOUSE]	3.77	6	3	3	8	-0.48	-0.48	-0.23	-0.22
P47955	60S acidic ribosomal protein P1 OS=Mus musculus GN=Rplp1 PE=1 SV=1 - [RLA1_MOUSE]	42.98	3	2	2	27	0.10	0.19	0.17	0.23

Q80X41-2	Isoform 2 of Serine/threonine-protein kinase VRK1 OS=Mus musculus GN=Vrk1 - [VRK1_MOUSE]	25.76	4	9	10	24	0.55	0.38	0.62	0.47
Q69ZR9-2	Isoform 2 of Protein FAM208A OS=Mus musculus GN=Fam208a - [F208A_MOUSE]	2.54	2	2	3	6	0.04	0.12	-0.26	-0.18
Q9CXT7-2	Isoform 2 of Transmembrane protein 192 OS=Mus musculus GN=Tmem192 - [TM192_MOUSE]	4.52	2	1	1	1	0.38	1.04	-0.11	0.55
G3UZF0	Ubiquitin carboxyl-terminal hydrolase OS=Mus musculus GN=Usp3 PE=2 SV=1 - [G3UZF0_MOUSE]	8.82	3	3	3	5	0.12	-0.10	0.23	-0.01
Q921W2	Nucleolysin TIAR OS=Mus musculus GN=Tial1 PE=2 SV=1 - [Q921W2_MOUSE]	10.93	6	3	4	13	0.15	0.20	0.04	0.00
Q9QYC0	Alpha-adducin OS=Mus musculus GN=Add1 PE=1 SV=2 - [ADDA_MOUSE]	29.80	9	17	18	106	-0.21	-0.29	-0.11	-0.26
Q6NZQ4	PAX-interacting protein 1 OS=Mus musculus GN=Paxip1 PE=1 SV=1 - [PAXI1_MOUSE]	2.37	1	1	1	4	-0.13	-0.09	-0.11	-0.07
P63147	Ubiquitin-conjugating enzyme E2 B OS=Mus musculus GN=Ube2b PE=1 SV=1 - [UBE2B_MOUSE]	19.08	1	2	2	7	-0.08	-0.22	-0.01	-0.16
P08032	Spectrin alpha chain, erythrocytic 1 OS=Mus musculus GN=Spta1 PE=2 SV=3 - [SPTA1_MOUSE]	3.52	4	5	7	29	0.13	0.47	-0.15	0.24
Q61598	Rab GDP dissociation inhibitor beta OS=Mus musculus GN=Gdi2 PE=1 SV=1 - [GDIB_MOUSE]	63.60	2	21	26	594	0.09	0.17	0.04	0.09
Q9CPY7	Cytosol aminopeptidase OS=Mus musculus GN=Lap3 PE=1 SV=3 - [AMPL_MOUSE]	52.41	2	23	23	226	0.04	0.03	0.03	0.10
Q8K3X6	Ankyrin repeat and SAM domain-containing protein 4B OS=Mus musculus GN=Anks4b PE=1 SV=1 - [ANS4B_MOUSE]	19.62	1	9	9	29	0.07	0.08	0.12	0.12
O09106	Histone deacetylase 1 OS=Mus musculus GN=Hdac1 PE=1 SV=1 - [HDAC1_MOUSE]	25.93	2	6	9	175	-0.01	0.00	-0.16	-0.07
P01849	T-cell receptor alpha chain C region OS=Mus musculus GN=Tcra PE=1 SV=1 - [TCA_MOUSE]	7.97	1	1	1	1	0.10	0.42	0.14	0.45

Q9WUM5	Succinyl-CoA ligase [ADP/GDP-forming] subunit alpha, mitochondrial OS=Mus musculus GN=Suclg1 PE=1 SV=4 - [SUCA_MOUSE]	23.41	1	9	9	137	-0.47	-0.36	-0.50	-0.36
Q4KL52	Zfyve19 protein OS=Mus musculus GN=Zfyve19 PE=2 SV=1 - [Q4KL52_MOUSE]	16.67	2	2	3	6	0.22	-0.20	-0.36	0.02
Q62446	Peptidyl-prolyl cis-trans isomerase FKBP3 OS=Mus musculus GN=Fkbp3 PE=1 SV=2 - [FKBP3_MOUSE]	44.64	1	8	9	40	-0.04	-0.36	-0.21	-0.09
P08730	Keratin, type I cytoskeletal 13 OS=Mus musculus GN=Krt13 PE=1 SV=2 [K1C13_MOUSE]	13.73	6	1	8	327	0.05	-0.40	0.15	-0.30
Q9QY40	Plexin-B3 OS=Mus musculus GN=Plxnb3 PE=1 SV=2 - [PLXB3_MOUSE]	1.16	1	2	2	2	0.08	0.33	0.09	0.33
Q2MHE5	Docking protein 6 OS=Mus musculus GN=Dok6 PE=2 SV=1 - [DOK6_MOUSE]	3.93	1	1	1	1	-0.26	-0.18	-0.07	0.01
Q8K135	Dyslexia-associated protein KIAA0319-like protein OS=Mus musculus GN=Kiaa0319l PE=1 SV=1 - [K319L_MOUSE]	2.86	2	2	2	2	0.05	0.53	0.05	0.54
P45376	Aldose reductase OS=Mus musculus GN=Akr1b1 PE=1 SV=3 - [ALDR_MOUSE]	51.58	2	15	16	223	0.00	-0.04	0.03	-0.01
Q9DCH4	Eukaryotic translation initiation factor 3 subunit F OS=Mus musculus GN=Eif3f PE=1 SV=2 - [EIF3F_MOUSE]	13.02	1	4	4	37	0.13	0.09	0.12	0.15
Q8K215	LYR motif-containing protein 4 OS=Mus musculus GN=Lyrm4 PE=2 SV=1 - [LYRM4_MOUSE]	19.78	1	2	2	6	0.10	-0.11	0.01	-0.22
O08997	Copper transport protein ATOX1 OS=Mus musculus GN=Atox1 PE=2 SV=1 - [ATOX1_MOUSE]	58.82	1	4	4	63	0.24	0.35	0.14	0.27
P63166	Small ubiquitin-related modifier 1 OS=Mus musculus GN=Sumo1 PE=1 SV=1 - [SUMO1_MOUSE]	41.58	1	5	5	27	-0.28	-0.12	-0.38	-0.16
A2A8E2	Protein 0610037L13Rik OS=Mus musculus GN=0610037L13Rik PE=2 SV=1 - [A2A8E2_MOUSE]	54.35	8	7	7	44	0.03	0.03	-0.03	0.01
Q61210-2	Isoform 2 of Rho guanine nucleotide exchange factor 1 OS=Mus musculus GN=Arhgef1 - [ARHG1_MOUSE]	15.89	7	11	11	38	0.22	0.12	0.05	0.03

P62874	Guanine nucleotide-binding protein G(I)/G(S)/G(T) subunit beta-1 OS=Mus musculus GN=Gnb1 PE=1 SV=3 - [GBB1_MOUSE]	36.47	4	5	9	128	-0.09	-0.27	-0.14	-0.27
D3YVZ9	28S ribosomal protein S21, mitochondrial (Fragment) OS=Mus musculus GN=Mrps21 PE=2 SV=1 - [D3YVZ9_MOUSE]	61.36	2	2	2	7	-0.04	-0.52	0.04	-0.40
P59114	Phosphorylated CTD-interacting factor 1 OS=Mus musculus GN=Pcif1 PE=1 SV=1 - [PCIF1_MOUSE]	4.53	1	3	3	3	-0.27	-0.03	0.09	0.04
Q9D241	RING finger protein 186 OS=Mus musculus GN=Rnf186 PE=2 SV=3 - [RN186_MOUSE]	11.95	2	1	1	2	-0.67	-0.52	-0.58	-0.43
Q61792	LIM and SH3 domain protein 1 OS=Mus musculus GN=Lasp1 PE=1 SV=1 - [LASP1_MOUSE]	70.34	13	14	15	148	-0.32	-0.30	-0.29	-0.27
P58252	Elongation factor 2 OS=Mus musculus GN=Eef2 PE=1 SV=2 - [EF2_MOUSE]	59.32	1	50	52	1100	0.04	0.08	0.02	0.08
F6RCZ2	Protein Wbscr27 (Fragment) OS=Mus musculus GN=Wbscr27 PE=2 SV=1 - [F6RCZ2_MOUSE]	27.63	3	2	2	10	-0.36	-0.22	-0.06	-0.09
Q61091	Frizzled-8 OS=Mus musculus GN=Fzd8 PE=1 SV=2 - [FZD8_MOUSE]	8.61	1	2	2	2	-0.18	-0.44	-0.08	-0.34
Q6PCX9	E3 ubiquitin-protein ligase TRIM37 OS=Mus musculus GN=Trim37 PE=2 SV=1 - [TRI37_MOUSE]	1.77	2	2	2	6	0.27	0.09	0.18	0.00
E9Q8P5	PDZ and LIM domain protein 5 OS=Mus musculus GN=Pdlim5 PE=2 SV=2 - [E9Q8P5_MOUSE]	42.26	3	1	6	48	-0.07	0.25	0.04	0.19
Q9D7S9	Charged multivesicular body protein 5 OS=Mus musculus GN=Chmp5 PE=2 SV=1 - [CHMP5_MOUSE]	31.51	1	5	5	14	0.40	0.47	0.15	0.49
Q9CV28-2	Isoform 2 of Protein FAM188A OS=Mus musculus GN=Fam188a - [F188A_MOUSE]	9.68	7	1	2	2	-0.45	-0.91	-0.25	-0.71
Q9Z0P5-2	Isoform 2 of Twinfilin-2 OS=Mus musculus GN=Twf2 - [TWF2_MOUSE]	31.41	4	6	9	43	0.11	0.03	-0.01	-0.11
Q8BSL0	Protein Zkscan8 OS=Mus musculus GN=Zkscan8 PE=2 SV=1 - [Q8BSL0_MOUSE]	2.55	1	1	1	1	-0.30	0.19	0.07	0.56
D3Z795	Proteasome assembly chaperone 1 OS=Mus musculus GN=Psmg1 PE=2 SV=1 - [D3Z795_MOUSE]	3.73	2	1	1	2	0.19	0.13	0.05	0.00

Q91YR9	Prostaglandin reductase 1 OS=Mus musculus GN=Ptgr1 PE=2 SV=2 - [PTGR1_MOUSE]	33.13	1	10	10	66	-0.14	-0.12	-0.18	-0.20
O55135	Eukaryotic translation initiation factor 6 OS=Mus musculus GN=Eif6 PE=1 SV=2 - [IF6_MOUSE]	26.94	2	4	4	37	-0.14	-0.20	-0.19	-0.20
P33766	fMet-Leu-Phe receptor OS=Mus musculus GN=Fpr1 PE=2 SV=1 - [FPR1_MOUSE]	3.02	1	1	1	8	-0.04	-0.33	0.11	-0.08
P16110	Galectin-3 OS=Mus musculus GN=Lgals3 PE=1 SV=3 - [LEG3_MOUSE]	34.47	2	9	9	319	-0.04	0.02	-0.01	-0.01
O54990-6	Isoform 6 of Prominin-1 OS=Mus musculus GN=Prom1 - [PROM1_MOUSE]	8.75	10	5	5	21	0.31	0.22	0.42	0.33
Q8BTW3	Exosome complex component MTR3 OS=Mus musculus GN=Exosc6 PE=1 SV=1 - [EXOS6_MOUSE]	18.68	1	3	3	16	0.07	0.10	-0.03	0.12
P07759	Serine protease inhibito A3K OS=Mus musculu GN=Serpina3k PE=1 SV=2 - [SPA3K_MOUSE]	43.06	1	16	17	294	-1.25	-1.11	-1.04	-0.94
Q9JKS5	Intracellular hyaluronan binding protein 4 OS=Mus musculus GN=Habp4 PE=2 SV=2 - [HABP4_MOUSE]	2.19	2	1	1	3	0.08	0.40	-0.07	0.24
Q64311	Protein N-terminal asparagine amidohydrolase OS=Mus musculus GN=Ntan1 PE=2 SV=3 [NTAN1_MOUSE]	13.87	2	3	3	7	0.03	0.21	0.02	0.19
Q80Y81-2	Isoform 2 of Zinc phosphodiesterase ELAC protein 2 OS=Mus musculus GN=Elac2 - [RNZ2_MOUSE]	12.14	4	9	9	23	0.02	0.19	0.02	0.24
H3BJK8	Protein 9930021J03Rik (Fragment) OS=Mus musculus GN=9930021J03Rik PE=4 SV=1 - [H3BJK8_MOUSE]	10.77	1	1	2	4	0.12	0.22	0.02	0.12
Q8CGB3	Uveal autoantigen with coiled-coil domains and ankyrin repeats OS=Mus musculus GN=Uaca PE=1 SV=2 - [UACA_MOUSE]	7.94	3	9	10	20	-0.47	-0.28	-0.56	-0.39
Q91YI0	Argininosuccinate lyase OS=Mus musculus GN=Asl PE=2 SV=1 - [ARLY_MOUSE]	46.55	5	17	18	54	-0.20	-0.32	-0.16	-0.28
Q91WL8	WW domain-containing oxidoreductase OS=Mus musculus GN=Wwox PE=1 SV=1 - [WWOX_MOUSE]	5.56	4	2	2	2	-0.10	0.00	-0.10	0.00

Q9JLZ3	Methylglutaconyl-CoA hydratase, mitochondrial OS=Mus musculus GN=Auh PE=2 SV=1 - [AUHM_MOUSE]	22.29	4	6	6	19	-0.25	-0.31	-0.40	-0.31
Q8VCZ7	Zinc finger and BTB domain-containing protein 7C OS=Mus musculus GN=Zbtb7c PE=2 SV=1 - [ZBT7C_MOUSE]	0.97	1	1	1	1	0.75	0.74	0.79	0.78
Q9D287	Pre-mRNA-splicing factor SPF27 OS=Mus musculus GN=Bcas2 PE=2 SV=1 - [SPF27_MOUSE]	20.89	2	6	6	15	0.22	0.22	0.28	0.17
A2AX52	Collagen alpha-4(VI) chain OS=Mus musculus GN=Col6a4 PE=1 SV=2 - [CO6A4_MOUSE]	4.16	1	7	7	10	-0.83	-0.77	-0.67	-0.62
Q05C51	Ribonuclease OS=Mus musculus GN=Rnaseh2a PE=2 SV=1 - [Q05C51_MOUSE]	17.32	4	4	4	22	-0.01	-0.20	0.20	0.01
Q3TZ86	C5a anaphylatoxin chemotactic receptor 1 OS=Mus musculus GN=C5ar1 PE=2 SV=1 [Q3TZ86_MOUSE]	3.42	1	1	1	2	-0.83	-0.65	-0.62	-0.44
O54941	SWI/SNF-related matrix-associated actin-dependent regulator of chromatin subfamily E member 1 OS=Mus musculus GN=Smarce1 PE=1 SV=1 - [SMCE1_MOUSE]	19.22	1	5	6	26	-0.04	0.00	0.07	0.20
Q8BU25	Inactive serine protease PAMR1 OS=Mus musculus GN=Pamr1 PE=2 SV=3 - [PAMR1_MOUSE]	1.11	1	1	1	1	-0.93	-0.77	-0.42	-0.26
P47811	Mitogen-activated protein kinase 14 OS=Mus musculus GN=Mapk14 PE=1 SV=3 - [MK14_MOUSE]	37.78	10	9	10	113	-0.08	-0.09	-0.02	-0.02
Q9Z0P4-2	Isoform 2 of Paralemmin-1 OS=Mus musculus GN=Palm - [PALM_MOUSE]	41.30	2	10	11	42	-0.44	-0.42	-0.48	-0.42
Q64339	Ubiquitin-like protein ISG15 OS=Mus musculus GN=Isg15 PE=1 SV=4 - [ISG15_MOUSE]	21.12	1	2	2	8	-0.62	-1.79	-0.12	-1.29
Q9D7E3	Ovarian cancer-associated gene 2 protein homolog OS=Mus musculus GN=Ovca2 PE=2 SV=1 - [OVCA2_MOUSE]	5.78	1	1	1	1	-0.03	0.47	-0.42	0.09
E9Q3K0	Protein Gm6588 OS=Mus musculus GN=Gm6588 PE=4 SV=1 - [E9Q3K0_MOUSE]	1.30	1	1	1	1	0.12	0.35	0.11	0.35
Q9WTV7	E3 ubiquitin-protein ligase RLIM OS=Mus musculus GN=Rlim PE=1 SV=2 - [RNF12_MOUSE]	3.33	1	1	1	2	0.62	-0.10	-0.40	-0.45

F8WI30	Sorting nexin-7 OS=Mus musculus GN=Snx7 PE=2 SV=1 - [F8WI30_MOUSE]	10.34	4	3	3	13	0.08	0.18	0.12	0.08
P47738	Aldehyde dehydrogenase, mitochondrial OS=Mus musculus GN=Aldh2 PE=1 SV=1 - [ALDH2_MOUSE]	47.98	2	23	27	928	-0.26	-0.23	-0.28	-0.27
Q9D7E4	UPF0449 protein C19orf25 homolog OS=Mus musculus PE=1 SV=1 - [CS025_MOUSE]	25.69	1	2	2	13	0.22	0.04	0.13	0.11
Q9D1A2	Cytosolic non-specific dipeptidase OS=Mus musculus GN=Cndp2 PE=1 SV=1 - [CNDP2_MOUSE]	60.42	1	20	21	250	-0.21	-0.25	-0.26	-0.30
F7B6K4	Pantothenate kinase 4 (Fragment) OS=Mus musculus GN=Pank4 PE=4 SV=1 - [F7B6K4_MOUSE]	12.47	4	5	5	13	-0.16	-0.07	-0.22	-0.14
P56212-2	Isoform ARPP-16 of cAMP-regulated phosphoprotein 19 OS=Mus musculus GN=Arpp19 - [ARP19_MOUSE]	73.96	5	4	5	39	0.27	0.30	0.35	0.20
Q9Z0Z3-2	Isoform 2 of S-phase kinase-associated protein 2 OS=Mus musculus GN=Skp2 - [SKP2_MOUSE]	8.76	3	1	2	5	0.05	-0.23	0.26	-0.02
Q9D4H7	LON peptidase N-terminal domain and RING finger protein 3 OS=Mus musculus GN=Lonrf3 PE=2 SV=1 - [LONF3_MOUSE]	3.45	1	2	2	2	0.02	0.05	-0.16	-0.13
Q8CHY6	Transcriptional repressor p66 alpha OS=Mus musculus GN=Gatad2a PE=1 SV=2 - [P66A_MOUSE]	11.13	2	4	5	21	-0.09	0.12	-0.05	0.00
A7XUY5-2	Isoform 2 of Selection and upkeep of intraepithelial T-cells protein 5 OS=Mus musculus GN=Skint5 - [SKIT5_MOUSE]	3.54	5	2	2	6	-0.51	-0.25	-0.48	-0.21
Q9Z247	Peptidyl-prolyl cis-trans isomerase FKBP9 OS=Mus musculus GN=Fkbp9 PE=1 SV=1 - [FKBP9_MOUSE]	18.95	1	10	10	31	-0.20	-0.16	-0.17	-0.30
P62900	60S ribosomal protein L31 OS=Mus musculus GN=Rpl31 PE=2 SV=1 [RL31_MOUSE]	25.60	1	4	4	42	-0.35	-0.28	-0.19	-0.25
Q0KK56-2	Isoform 2 of Protein FAM184B OS=Mus musculus GN=Fam184b - [F184B_MOUSE]	9.06	2	3	4	8	-0.31	-1.18	-0.57	-1.44
Q8R164	Valacyclovir hydrolase OS=Mus musculus GN=Bphl PE=2 SV=1 - [BPHL_MOUSE]	53.26	1	14	14	73	-0.47	-0.49	-0.54	-0.55
Q91X51	Golgi reassembly-stacking protein 1 OS=Mus musculus GN=Gorasp1 PE=2 SV=3 - [GORS1_MOUSE]	15.47	1	3	4	28	-0.03	-0.25	-0.45	-0.60

Q0P678	Zinc finger CCCH domain-containing protein 18 OS=Mus musculus GN=Zc3h18 PE=1 SV=1 - [ZCH18_MOUSE]	10.55	5	7	8	30	0.20	0.27	0.08	0.09
P0CG14	Chromosome transmission fidelity protein 8 homolog isoform 2 OS=Mus musculus GN=Chtf8 PE=2 SV=1 - [CTF8A_MOUSE]	2.63	1	1	1	1	-0.24	0.96	-1.22	-0.03
E9PY16	Protein Adap1 OS=Mus musculus GN=Adap1 PE=2 SV=1 - [E9PY16_MOUSE]	3.21	1	1	1	1	0.33	0.27	0.32	0.26
Q80X50	Ubiquitin-associated protein 2-like OS=Mus musculus GN=Ubap2l PE=1 SV=1 - [UBP2L_MOUSE]	14.81	5	13	13	92	0.07	0.11	0.03	0.14
Q9JHW2	Omega-amidase NIT2 OS=Mus musculus GN=Nit2 PE=1 SV=1 - [NIT2_MOUSE]	47.83	1	12	12	63	-0.32	-0.24	-0.30	-0.27
O35177	Enhancer of filamentation 1 OS=Mus musculus GN=Nedd9 PE=1 SV=2 - [CASL_MOUSE]	1.44	1	1	1	1	-0.51	0.20	-1.04	-0.34
Q8CFB8	NAD+ ADP-ribosyltransferase 3 PARP-3 OS=Mus musculus GN=Parp3 PE=2 SV=1 - [Q8CFB8_MOUSE]	18.75	4	8	8	34	0.46	0.42	0.46	0.25
F8WGL3	Cofilin-1 OS=Mus musculus GN=Cfl1 PE=2 SV=1 - [F8WGL3_MOUSE]	56.39	1	1	22	670	0.20	0.25	-0.01	0.03
Q9JII5-2	Isoform 2 of DAZ-associated protein 1 OS=Mus musculus GN=Dazap1 - [DAZP1_MOUSE]	13.09	4	4	4	36	-0.35	-0.35	-0.10	-0.30
Q5XJF6	Ribosomal protein OS=Mus musculus GN=Rpl10a PE=2 SV=1 - [Q5XJF6_MOUSE]	41.01	4	11	11	268	0.11	0.13	0.05	0.12
Q8R4U7	Leucine zipper protein 1 OS=Mus musculus GN=Luzp1 PE=1 SV=2 - [LUZP1_MOUSE]	18.35	2	16	16	46	-0.23	-0.18	-0.18	-0.18
Q9CQH7	Transcription factor BTF3 homolog 4 OS=Mus musculus GN=Btf3l4 PE=2 SV=1 - [BT3L4_MOUSE]	16.46	1	3	4	15	0.29	0.13	0.08	0.02
Q8C4B4	Protein unc-119 homolog B OS=Mus musculus GN=Unc119b PE=2 SV=1 - [U119B_MOUSE]	25.90	2	4	4	15	0.25	-0.24	0.18	-0.42
P62342	Selenoprotein T OS=Mus musculus GN=Selt PE=2 SV=2 - [SELT_MOUSE]	15.38	1	3	3	14	-0.02	-0.10	-0.12	-0.36
Q8BZH1	Protein-glutamine gamma-glutamyltransferase 4 OS=Mus musculus GN=Tgm4 PE=1 SV=2 [TGM4_MOUSE]	4.03	1	1	1	3	0.13	-0.17	0.25	-0.04

Q8BPB5	EGF-containing fibulin-like extracellular matrix protein 1 OS=Mus musculus GN=Efemp1 PE=2 SV=1 - [FBLN3_MOUSE]	22.52	2	7	7	44	-0.56	-0.54	-0.43	-0.67
O35709	Ectoderm-neural cortex protein 1 OS=Mus musculus GN=Enc1 PE=2 SV=2 - [ENC1_MOUSE]	7.64	1	3	3	3	0.05	-0.44	0.18	-0.31
Q61553	Fascin OS=Mus musculus GN=Fscn1 PE=1 SV=4 - [FSCN1_MOUSE]	35.70	5	13	13	44	-0.33	-0.26	-0.23	-0.13
Q6PFF0	Protein Scaf4 OS=Mus musculus GN=Scaf4 PE=2 SV=1 - [Q6PFF0_MOUSE]	3.64	2	3	3	8	-0.11	0.07	-0.24	0.05
P62918	60S ribosomal protein L8 OS=Mus musculus GN=Rpl8 PE=2 SV=2 - [RL8_MOUSE]	28.02	1	7	7	75	-0.05	-0.08	-0.10	-0.12
P70670	Nascent polypeptide-associated complex subunit alpha, muscle-specific form OS=Mus musculus GN=Naca PE=1 SV=2 - [NACAM_MOUSE]	3.25	2	5	5	62	0.12	0.17	0.16	0.15
Q5NC83	Uncharacterized protein C7orf72 homolog OS=Mus musculus PE=2 SV=2 - [CG072_MOUSE]	2.04	1	1	1	2	-0.50	-0.45	-0.41	-0.36
Q3THG9	Alanyl-tRNA editing protein Aarsd1 OS=Mus musculus GN=Aarsd1 PE=1 SV=2 - [AASD1_MOUSE]	25.49	3	8	8	23	-0.05	-0.08	-0.27	-0.29
Q3TC93	HCLS1-binding protein 3 OS=Mus musculus GN=Hs1bp3 PE=1 SV=2 - [H1BP3_MOUSE]	14.18	1	4	4	11	-0.26	-0.26	-0.17	-0.08
Q8R422	CD109 antigen OS=Mus musculus GN=Cd109 PE=2 SV=1 - [CD109_MOUSE]	14.63	1	21	21	52	-0.77	-0.65	-0.74	-0.57
Q9DA03	Complex III assembly factor LYRM7 OS=Mus musculus GN=Lyrm7 PE=2 SV=2 - [LYRM7_MOUSE]	16.35	1	1	1	8	0.17	-0.40	0.25	-0.25
Q922Y1	UBX domain-containing protein 1 OS=Mus musculus GN=Ubxn1 PE=1 SV=1 - [UBXN1_MOUSE]	35.02	1	8	8	38	-0.06	-0.11	-0.08	-0.06
Q9CZG3	COMM domain-containing protein 8 OS=Mus musculus GN=Commd8 PE=2 SV=1 - [COMD8_MOUSE]	40.44	1	6	6	21	0.07	0.25	-0.07	0.20
P70362	Ubiquitin fusion degradation protein 1 homolog OS=Mus musculus GN=Ufd1l PE=1 SV=2 - [UFD1_MOUSE]	17.59	5	5	5	21	0.10	-0.01	0.07	0.12

Q8CFQ9	Fusion, derived from t(1216) malignant liposarcoma (Human) OS=Mus musculus GN=Fus PE=2 SV=1 - [Q8CFQ9_MOUSE]	20.89	5	6	9	165	-0.01	0.02	0.03	0.08
Q56A08	G patch domain and KOW motifs-containing protein OS=Mus musculus GN=Gpkow PE=2 SV=2 - [GPKOW_MOUSE]	8.81	2	4	4	8	0.20	0.14	0.07	0.00
O09061	Proteasome subunit beta type-1 OS=Mus musculus GN=Psmb1 PE=1 SV=1 - [PSB1_MOUSE]	57.92	1	11	11	167	-0.22	-0.18	-0.20	-0.17
Q91XF0	Pyridoxine-5'-phosphate oxidase OS=Mus musculus GN=Pnpo PE=1 SV=1 - [PNPO_MOUSE]	29.89	2	7	7	31	-0.65	-0.64	-0.87	-0.84
O88653	Ragulator complex protein LAMTOR3 OS=Mus musculus GN=Lamtor3 PE=1 SV=1 - [LTOR3_MOUSE]	28.23	1	2	2	4	0.03	0.13	-0.05	0.05
Q91YE3	Egl nine homolog 1 OS=Mus musculus GN=Egln1 PE=2 SV=2 [EGLN1_MOUSE]	3.25	1	1	1	4	0.23	0.41	0.01	0.19
Q6A068	Cell division cycle 5-like protein OS=Mus musculus GN=Cdc5l PE=1 SV=2 - [CDC5L_MOUSE]	23.44	1	11	12	40	0.06	0.05	-0.08	0.06
A2A7S8-2	Isoform 2 of Uncharacterized protein KIAA1522 OS=Mus musculus GN=Kiaa1522 - [K1522_MOUSE]	15.51	4	11	11	21	-0.26	-0.29	-0.26	-0.22
Q8K0C5	Zymogen granule membrane protein 16 OS=Mus musculus GN=Zg16 PE=2 SV=1 - [ZG16_MOUSE]	69.46	1	8	8	155	0.11	0.15	0.09	0.07
F8VQH0	Abelson tyrosine-protein kinase 2 OS=Mus musculus GN=Abl2 PE=2 SV=1 - [F8VQH0_MOUSE]	3.72	3	2	3	7	0.30	0.16	0.01	-0.13
Q6PGF5	BMS1 homolog, ribosome assembly protein (Yeast) OS=Mus musculus GN=Bms1 PE=2 SV=1 - [Q6PGF5_MOUSE]	0.93	1	1	1	1	-0.56	-0.41	-0.23	-0.09
A2AD97	Matrix-remodeling-associated protein 8 (Fragment) OS=Mus musculus GN=Mxra8 PE=2 SV=1 - [A2AD97_MOUSE]	21.15	2	4	4	7	0.09	-0.31	-0.25	-0.27
Q8BP74	L-seryl-tRNA(Sec) kinase OS=Mus musculus GN=Pstk PE=1 SV=2 - [PSTK_MOUSE]	1.67	1	1	1	6	0.05	0.05	0.07	0.00
Q8VE95	UPF0598 protein C8orf82 homolog OS=Mus musculus PE=2 SV=1 - [CH082_MOUSE]	26.15	1	4	4	17	-0.52	-0.97	-0.80	-0.94
E0CZ73	Occludin OS=Mus musculus GN=Ocln PE=2 SV=1 - [E0CZ73_MOUSE]	24.26	3	5	5	15	-0.21	-0.07	-0.24	-0.14

Q99J47-2	Isoform 2 of Dehydrogenase/reductase SDR family member 7B OS=Mus musculus GN=Dhrs7b - [DRS7B_MOUSE]	23.25	2	6	6	17	-0.15	0.11	-0.18	-0.02
Q9D1R9	60S ribosomal protein L34 OS=Mus musculus GN=Rpl34 PE=3 SV=2 [RL34_MOUSE]	30.77	2	6	6	117	-0.09	0.04	-0.08	0.07
Q03517	Secretogranin-2 OS=Mus musculus GN=Scg2 PE=1 SV=1 - [SCG2_MOUSE]	28.69	1	14	15	142	-0.82	-0.74	-0.81	-0.71
P42208	Septin-2 OS=Mus musculus GN=Sept2 PE=1 SV=2 - [SEPT2_MOUSE]	44.60	9	11	12	130	0.07	0.17	-0.04	0.03
Q9CZ44	NSFL1 cofactor p47 OS=Mus musculus GN=Nsfl1c PE=1 SV=1 - [NSF1C_MOUSE]	53.24	4	15	15	131	-0.07	-0.09	-0.10	-0.07
E9Q450	Tropomyosin alpha-1 chain OS=Mus musculus GN=Tpm1 PE=2 SV=1 - [E9Q450_MOUSE]	76.41	6	1	36	2037	0.19	-0.83	-0.11	-0.17
Q9ERS5	Pleckstrin homology domain-containing family A member 2 OS=Mus musculus GN=Plekha2 PE=1 SV=1 - [PKHA2_MOUSE]	3.29	1	1	1	5	-0.05	0.02	0.22	0.28
Q7TQH0-2	Isoform 2 of Ataxin-2-like protein OS=Mus musculus GN=Atxn2l - [ATX2L_MOUSE]	15.08	6	12	14	40	0.06	0.03	-0.01	0.01
Q9Z0V7	Mitochondrial import inner membrane translocase subunit Tim17-B OS=Mus musculus GN=Timm17b PE=2 SV=1 - [TI17B_MOUSE]	21.51	2	2	2	3	-0.19	-0.48	0.17	-0.49
P40237	CD82 antigen OS=Mus musculus GN=Cd82 PE=1 SV=1 - [CD82_MOUSE]	12.78	3	3	3	12	0.07	0.36	-0.18	0.36
P97794	ATP-sensitive inward rectifier potassium channel 8 OS=Mus musculus GN=Kcnj8 PE=2 SV=1 - [IRK8_MOUSE]	3.07	1	1	1	2	0.08	0.19	-0.07	0.03
P70202	Latexin OS=Mus musculus GN=Lxn PE=1 SV=2 - [LXN_MOUSE]	12.16	1	2	2	13	0.52	0.51	0.18	0.45
Q6ZWM4	N-alpha-acetyltransferase 38, NatC auxiliary subunit OS=Mus musculus GN=Naa38 PE=3 SV=3 - [NAA38_MOUSE]	43.75	1	4	4	46	-0.36	-0.41	-0.20	-0.24
Q9R069	Basal cell adhesion molecule OS=Mus musculus GN=Bcam PE=2 SV=1 - [BCAM_MOUSE]	34.08	2	15	15	87	-0.56	-0.53	-0.54	-0.53
Q8BJW5-2	Isoform 2 of Nucleolar protein 11 OS=Mus musculus GN=Nol11 - [NOL11_MOUSE]	1.85	2	1	1	2	0.25	0.97	-0.07	0.64

Q99N95	39S ribosomal protein L3, mitochondrial OS=Mus musculus GN=Mrpl3 PE=2 SV=1 [RM03_MOUSE]	17.53	3	7	7	12	-0.49	0.08	-0.38	0.12
Q9D8P4	39S ribosomal protein L17, mitochondrial OS=Mus musculus GN=Mrpl17 PE=1 SV=1 - [RM17_MOUSE]	14.77	1	3	3	27	-0.09	-0.65	-0.25	-0.82
Q3U4W8	Ubiquitin carboxyl-terminal hydrolase OS=Mus musculus GN=Usp5 PE=2 SV=1 - [Q3U4W8_MOUSE]	34.97	4	21	21	163	0.06	0.05	0.05	0.05
Q61880	Meiotic recombination protein DMC1/LIM15 homolog OS=Mus musculus GN=Dmc1 PE=1 SV=1 - [DMC1_MOUSE]	6.47	1	1	1	5	-0.19	0.25	-0.18	0.16
Q9Z2U1	Proteasome subunit alpha type-5 OS=Mus musculus GN=Psma5 PE=1 SV=1 - [PSA5_MOUSE]	43.98	2	10	10	153	-0.04	0.00	-0.11	-0.10
Q3UEB3-2	Isoform 2 of Poly(U)-binding-splicing factor PUF60 OS=Mus musculus GN=Puf60 - [PUF60_MOUSE]	27.24	3	12	12	78	0.07	0.13	0.06	0.00
Q8R050-2	Isoform 2 of Eukaryotic peptide chain release factor GTP-binding subunit ERF3A OS=Mus musculus GN=Gspt1 - [ERF3A_MOUSE]	37.32	4	19	20	104	0.10	0.14	0.04	0.02
Q9R1P3	Proteasome subunit beta type-2 OS=Mus musculus GN=Psmb2 PE=1 SV=1 - [PSB2_MOUSE]	57.21	1	11	12	73	-0.17	-0.15	-0.13	-0.15
P46414	Cyclin-dependent kinase inhibitor 1B OS=Mus musculus GN=Cdkn1b PE=1 SV=2 - [CDN1B_MOUSE]	25.38	1	2	3	8	-0.49	-0.91	-0.33	-0.75
Q9DCE9	Protein Igtp OS=Mus musculus GN=Igtp PE=2 SV=1 - [Q9DCE9_MOUSE]	22.93	1	8	10	33	0.20	0.24	0.17	0.24
Q8BTI8-3	Isoform 3 of Serine/arginine repetitive matrix protein 2 OS=Mus musculus GN=Srrm2 - [SRRM2_MOUSE]	15.44	3	27	27	136	-0.07	-0.03	-0.09	-0.05
Q9R0X4	Acyl-coenzyme A thioesterase 9, mitochondrial OS=Mus musculus GN=Acot9 PE=1 SV=1 - [ACOT9_MOUSE]	35.31	2	15	15	76	-0.17	-0.05	-0.21	-0.18
Q9R1C7	Pre-mRNA-processing factor 40 homolog A OS=Mus musculus GN=Prpf40a PE=1 SV=1 - [PR40A_MOUSE]	17.63	9	18	18	76	0.10	0.10	0.07	0.12
Q8BH48-2	Isoform 2 of Ubiquitin-associated protein 1 OS=Mus musculus GN=Ubap1 - [UBAP1_MOUSE]	8.39	2	3	3	13	-0.12	-0.21	-0.21	-0.16

Q9EQF6	Dihydropyrimidinase-related protein 5 OS=Mus musculus GN=Dpysl5 PE=1 SV=1 - [DPYL5_MOUSE]	17.91	2	7	7	17	-0.35	-0.28	-0.31	-0.30
O35405	Phospholipase D3 OS=Mus musculus GN=Pld3 PE=2 SV=1 - [PLD3_MOUSE]	6.15	1	3	3	14	-0.12	-0.20	-0.28	-0.17
Q8CBW3-5	Isoform 5 of Abl interactor 1 OS=Mus musculus GN=Abi1 - [ABI1_MOUSE]	37.63	12	13	13	72	0.05	0.03	-0.10	0.00
P83870	PHD finger-like domain containing protein 5A OS=Mus musculus GN=Phf5a PE=1 SV=1 [PHF5A_MOUSE]	44.55	1	6	6	17	-0.12	-0.06	-0.04	-0.12
Q2VPQ9	Chromatin modification related protein MEAF6 OS=Mus musculus GN=Meaf6 PE=2 SV=1 - [EAF6_MOUSE]	5.24	2	1	1	4	0.52	-0.12	0.53	-0.01
Q8BHK9	DNA excision repair protein ERCC-6-like OS=Mus musculus GN=Ercc6l PE=1 SV=1 - [ERC6L_MOUSE]	6.21	1	4	5	5	0.35	0.67	0.72	1.04
O35595	Protein patched homolog 2 OS=Mus musculus GN=Ptch2 PE=2 SV=2 - [PTC2_MOUSE]	1.52	1	1	1	1	-0.36	-0.70	-0.38	-0.72
Q9DC53	Copine-8 OS=Mus musculus GN=Cpne8 PE=2 SV=2 - [CPNE8_MOUSE]	7.62	7	2	3	7	0.04	0.00	0.23	0.19
Q99KK7	Dipeptidyl peptidase 3 OS=Mus musculus GN=Dpp3 PE=2 SV=2 - [DPP3_MOUSE]	48.37	1	28	28	198	-0.01	-0.04	-0.05	-0.03
O55033	Cytoplasmic protein NCK2 OS=Mus musculus GN=Nck2 PE=1 SV=1 - [NCK2_MOUSE]	19.74	1	5	6	21	0.16	0.03	0.11	-0.05
F8WJ93	Echinoderm microtubule-associated protein-like 4 OS=Mus musculus GN=Eml4 PE=2 SV=1 - [F8WJ93_MOUSE]	20.45	5	17	17	55	-0.23	-0.20	-0.20	-0.20
Q9DAV6	Protein Serpinb9b OS=Mus musculus GN=Serpinb9b PE=2 SV=1 - [Q9DAV6_MOUSE]	11.67	2	1	4	18	0.09	-0.25	-0.44	-0.77
Q8VE80	THO complex subunit 3 OS=Mus musculus GN=Thoc3 PE=2 SV=1 - [THOC3_MOUSE]	16.24	1	5	5	10	-0.22	-0.02	-0.26	-0.07
P05622	Platelet-derived growth factor receptor beta OS=Mus musculus GN=Pdgfrb PE=1 SV=1 - [PGFRB_MOUSE]	2.09	13	1	2	16	-0.97	-0.70	-0.88	-0.60
Q9ET01	Glycogen phosphorylase, liver form OS=Mus musculus GN=Pygl PE=1 SV=4 - [PYGL_MOUSE]	17.88	2	7	14	97	0.15	0.14	0.09	0.22

Q8JZM2	Proline-rich protein 15-like protein OS=Mus musculus GN=Prr15l PE=2 SV=1 - [PR15L_MOUSE]	46.00	1	2	2	4	-0.17	0.08	-0.21	-0.10
O35598	Disintegrin and metalloproteinase domain-containing protein 10 OS=Mus musculus GN=Adam10 PE=1 SV=2 - [ADA10_MOUSE]	15.09	3	9	9	28	-0.05	0.02	0.11	-0.06
E9Q8F1	Lysine-specific demethylase 5A OS=Mus musculus GN=Kdm5a PE=2 SV=1 - [E9Q8F1_MOUSE]	2.65	2	2	2	2	0.23	0.87	0.05	0.69
P35991	Tyrosine-protein kinase BTK OS=Mus musculus GN=Btk PE=1 SV=4 - [BTK_MOUSE]	5.31	2	3	4	10	0.06	-0.12	-0.07	-0.15
P49817	Caveolin-1 OS=Mus musculus GN=Cav1 PE=1 SV=1 - [CAV1_MOUSE]	41.01	6	1	7	186	-0.70	-0.66	-0.90	-0.72
Q7TMI3	E3 ubiquitin-protein ligase UHRF2 OS=Mus musculus GN=Uhrf2 PE=1 SV=1 - [UHRF2_MOUSE]	3.36	2	2	2	2	-0.11	-0.17	-0.25	-0.31
B1AXP6	Mitochondrial import receptor subunit TOM5 homolog OS=Mus musculus GN=Tomm5 PE=3 SV=1 - [TOM5_MOUSE]	62.75	4	5	5	25	-0.21	-0.41	-0.18	-0.49
O08749	Dihydrolipoyl dehydrogenase, mitochondrial OS=Mus musculus GN=Dld PE=1 SV=2 - [DLDH_MOUSE]	50.88	1	17	17	329	-0.30	-0.23	-0.37	-0.28
Q8CCH2	NHL repeat-containing protein 3 OS=Mus musculus GN=Nhlrc3 PE=2 SV=1 - [NHLC3_MOUSE]	17.29	2	4	4	10	-0.25	-0.39	-0.29	-0.37
Q91Z53	Glyoxylate reductase/hydroxypyruvate reductase OS=Mus musculus GN=Grhpr PE=1 SV=1 - [GRHPR_MOUSE]	32.32	3	9	9	99	-0.35	-0.33	-0.11	-0.16
Q8VC30	Bifunctional ATP-dependent dihydroxyacetone kinase/FAD-AMP lyase (cyclizing) OS=Mus musculus GN=Dak PE=2 SV=1 - [DHAK_MOUSE]	12.46	1	5	5	11	-0.59	-0.30	-0.37	-0.20
Q8BGH4	Receptor expression-enhancing protein 1 OS=Mus musculus GN=Reep1 PE=1 SV=1 - [REEP1_MOUSE]	8.46	1	1	1	1	-0.16	0.09	-0.37	-0.12
E9Q7G0	Protein Numa1 OS=Mus musculus GN=Numa1 PE=2 SV=1 - [E9Q7G0_MOUSE]	37.49	4	58	59	259	-0.08	-0.05	-0.11	-0.02

F6R8S6	Proline-rich AKT1 substrate 1 (Fragment) OS=Mus musculus GN=Akt1s1 PE=4 SV=1 - [F6R8S6_MOUSE]	29.08	8	4	4	6	0.05	-2.35	0.06	-2.34
G3UYZ1	Immunoglobulin superfamily member 8 OS=Mus musculus GN=Igsf8 PE=2 SV=1 - [G3UYZ1_MOUSE]	3.65	2	1	1	2	0.05	0.19	-0.37	-0.22
Q9CWZ3-2	Isoform 2 of RNA-binding protein 8A OS=Mus musculus GN=Rbm8a - [RBM8A_MOUSE]	15.61	2	3	3	24	0.43	0.50	0.63	0.57
Q78IQ7	Zinc transporter ZIP4 OS=Mus musculus GN=Slc39a4 PE=1 SV=1 - [S39A4_MOUSE]	6.67	2	2	2	8	0.13	-0.17	0.36	-0.14
F7CZ64	Voltage-dependent calcium channel gamma 8 subunit OS=Mus musculus GN=Cacng8 PE=4 SV=2 - [F7CZ64_MOUSE]	8.98	2	1	1	4	0.22	0.32	0.28	0.38
Q02013	Aquaporin-1 OS=Mus musculus GN=Aqp1 PE=1 SV=3 - [AQP1_MOUSE]	11.15	1	2	2	16	-0.20	0.14	-0.59	0.08
P97431	Interferon regulatory factor 6 OS=Mus musculus GN=Irf6 PE=1 SV=2 - [IRF6_MOUSE]	10.28	1	4	4	9	0.28	0.40	0.11	0.20
E9Q504	Histone demethylase UTY OS=Mus musculus GN=Uty PE=2 SV=1 - [E9Q504_MOUSE]	2.52	5	1	1	1	-0.20	-0.09	-0.30	-0.19
P02089	Hemoglobin subunit beta-2 OS=Mus musculus GN=Hbb-b2 PE=1 SV=2 - [HBB2_MOUSE]	62.59	3	5	10	472	1.43	1.23	1.31	1.13
Q9D906	Ubiquitin-like modifier-activating enzyme ATG7 OS=Mus musculus GN=Atg7 PE=1 SV=1 - [ATG7_MOUSE]	13.32	1	5	6	18	0.51	0.44	0.33	0.26
Q8C1A5	Thimet oligopeptidase OS=Mus musculus GN=Thop1 PE=1 SV=1 - [THOP1_MOUSE]	35.95	1	21	22	99	-0.11	-0.18	-0.04	0.00
Q9D7N6	39S ribosomal protein L30, mitochondrial OS=Mus musculus GN=Mrpl30 PE=2 SV=1 - [RM30_MOUSE]	6.88	1	1	1	1	0.08	0.32	0.01	0.24
Q9D1E6	Tubulin-folding cofactor B OS=Mus musculus GN=Tbcb PE=1 SV=2 - [TBCB_MOUSE]	31.56	1	8	8	44	-0.03	-0.05	-0.06	0.02
Q9CZL5	Pterin-4-alpha-carbinolamine dehydratase 2 OS=Mus musculus GN=Pcbd2 PE=1 SV=2 - [PHS2_MOUSE]	36.76	1	4	5	17	-0.48	-0.25	-0.32	-0.23
Q62266	Cornifin-A OS=Mus musculus GN=Sprr1a PE=2 SV=1 - [SPR1A_MOUSE]	28.47	2	2	2	3	0.36	0.40	0.59	0.63

Q6P6L6	Gem (Nuclear organelle) associated protein 4 OS=Mus musculus GN=Gemin4 PE=2 SV=1 - [Q6P6L6_MOUSE]	3.21	1	3	3	7	-0.07	-0.26	0.19	-0.03
F6YU83	Serine/threonine-protein kinase PDIK1L (Fragment) OS=Mus musculus GN=Pdik1l PE=2 SV=1 - [F6YU83_MOUSE]	4.52	3	1	1	1	-0.18	-0.09	-0.24	-0.16
Q6ZWQ9	MCG5400 OS=Mus musculus GN=Myl12a PE=2 SV=1 - [Q6ZWQ9_MOUSE]	83.72	3	3	12	1032	-0.08	-0.14	0.02	-0.03
Q61072	Disintegrin and metalloproteinase domain-containing protein 9 OS=Mus musculus GN=Adam9 PE=1 SV=2 - [ADAM9_MOUSE]	4.50	2	2	2	7	0.30	0.43	-0.02	0.46
Q5NCJ0	Protein Rapgef6 OS=Mus musculus GN=Rapgef6 PE=2 SV=1 - [Q5NCJ0_MOUSE]	0.94	6	1	2	2	0.30	-0.22	0.37	-0.15
Q9Z2X2	26S proteasome non-ATPase regulatory subunit 10 OS=Mus musculus GN=Psmd10 PE=1 SV=3 - [PSD10_MOUSE]	12.99	1	2	2	4	-0.12	-0.13	-0.27	-0.28
Q91YJ3	Thymocyte nuclear protein 1 OS=Mus musculus GN=Thyn1 PE=1 SV=1 - [THYN1_MOUSE]	31.86	1	7	7	18	0.12	0.07	-0.05	0.01
P42567	Epidermal growth factor receptor substrate 15 OS=Mus musculus GN=Eps15 PE=1 SV=1 [EPS15_MOUSE]	23.52	6	14	14	47	-0.27	-0.15	-0.32	-0.21
O35627-2	Isoform CAR2 of Nuclear receptor subfamily 1 group I member 3 OS=Mus musculus GN=Nr1i3 - [NR1I3_MOUSE]	3.50	3	1	1	1	-0.68	0.69	-0.91	0.46
O88342	WD repeat-containing protein 1 OS=Mus musculus GN=Wdr1 PE=1 SV=3 - [WDR1_MOUSE]	68.15	1	32	32	533	0.01	0.00	0.00	0.01
Q2KN98	Cytospin-A OS=Mus musculus GN=Specc1l PE=1 SV=1 - [CYTSA_MOUSE]	16.73	6	12	13	44	-0.25	-0.14	-0.31	-0.23
P99028	Cytochrome b-c1 complex subunit 6, mitochondrial OS=Mus musculus GN=Uqcrh PE=1 SV=2 - [QCR6_MOUSE]	66.29	1	5	5	136	-0.15	-0.11	-0.14	-0.16
Q60989	E3 ubiquitin-protein ligase XIAP OS=Mus musculus GN=Xiap PE=1 SV=2 - [XIAP_MOUSE]	13.51	2	3	3	12	0.17	0.15	0.08	0.08
E9QMV2	Protein Gm6314 OS=Mus musculus GN=Abracl PE=4 SV=1 - [E9QMV2_MOUSE]	11.11	2	1	1	11	-0.06	0.10	0.03	0.20

Q3U6N9-2	Isoform 2 of UPF0488 protein C8orf33 homolog OS=Mus musculus - [CH033_MOUSE]	10.06	3	1	1	2	-0.13	0.22	-0.10	0.26
Q9ER39	Torsin-1A OS=Mus musculus GN=Tor1a PE=1 SV=1 - [TOR1A_MOUSE]	13.51	1	4	4	9	-0.30	-0.05	-0.23	-0.03
Q3U319	E3 ubiquitin-protein ligase BRE1B OS=Mus musculus GN=Rnf40 PE=2 SV=2 - [BRE1B_MOUSE]	8.89	3	7	7	16	-0.12	0.10	0.06	0.21
P46638	Ras-related protein Rab-11B OS=Mus musculus GN=Rab11b PE=1 SV=3 - [RB11B_MOUSE]	61.01	8	12	13	168	-0.11	-0.20	-0.08	-0.11
E9Q575	Caspase OS=Mus musculus GN=Casp12 PE=2 SV=1 - [E9Q575_MOUSE]	8.88	3	2	2	2	0.55	0.24	0.51	0.20
P56376	Acylphosphatase-1 OS=Mus musculus GN=Acyp1 PE=2 SV=2 - [ACYP1_MOUSE]	35.35	2	3	3	20	-0.14	-0.01	-0.38	-0.19
P59644	Phosphatidylinositol 4,5 bisphosphate 5-phosphatase A OS=Mus musculus GN=Inpp5j PE=1 SV=2 - [PI5PA_MOUSE]	5.08	2	3	3	5	0.19	-0.18	-0.07	-0.44
Q3TCN2	Putative phospholipase B-like 2 OS=Mus musculus GN=Plbd2 PE=1 SV=2 - [PLBL2_MOUSE]	6.73	2	3	3	12	-0.31	-0.30	-0.37	-0.32
Q09199	Beta-1,4 N-acetylgalactosaminyltransferase 2 OS=Mus musculus GN=B4galnt2 PE=2 SV=1 - [B4GN2_MOUSE]	31.96	1	15	17	143	-0.36	-0.33	-0.28	-0.33
Q5SWZ5	Myosin phosphatase Rho-interacting protein OS=Mus musculus GN=Mprip PE=2 SV=1 [Q5SWZ5_MOUSE]	15.47	9	24	25	64	-0.31	-0.28	-0.37	-0.38
Q9CPU0	Lactoylglutathione lyase OS=Mus musculus GN=Glo1 PE=1 SV=3 - [LGUL_MOUSE]	49.46	1	8	8	118	0.05	0.07	0.03	0.06
P17751	Triosephosphate isomerase OS=Mus musculus GN=Tpi1 PE=1 SV=4 - [TPIS_MOUSE]	59.53	2	16	16	513	-0.10	0.01	-0.14	-0.05
Q80XA6	RalBP1-associated Eps domain-containing protein 2 OS=Mus musculus GN=Reps2 PE=1 SV=1 - [REPS2_MOUSE]	8.25	4	2	3	4	-0.04	0.14	0.08	0.26
Q3U9G9	Lamin-B receptor OS=Mus musculus GN=Lbr PE=1 SV=2 - [LBR_MOUSE]	18.69	1	13	13	51	0.16	0.08	0.26	0.06
Q99LA2	Protein Zfp207 OS=Mus musculus GN=Zfp207 PE=2 SV=1 - [Q99LA2_MOUSE]	11.64	6	4	4	27	0.11	-0.22	-0.07	-0.20

E9PYQ0	Protein Rsph10b OS=Mus musculus GN=Rsph10b PE=2 SV=1 - [E9PYQ0_MOUSE]	3.42	1	2	2	3	-0.31	-0.51	0.64	0.43
B1AUQ7	Centrin-2 (Fragment) OS=Mus musculus GN=Cetn2 PE=2 SV=1 [B1AUQ7_MOUSE]	14.41	3	2	2	6	-0.29	-0.20	-0.10	-0.01
Q9Z255	Ubiquitin-conjugating enzyme E2 A OS=Mus musculus GN=Ube2a PE=2 SV=1 - [UBE2A_MOUSE]	11.18	1	1	1	8	-0.02	-0.05	0.07	0.00
D3Z4D9	C2 domain-containing protein 3 (Fragment) OS=Mus musculus GN=C2cd3 PE=2 SV=1 - [D3Z4D9_MOUSE]	1.39	7	2	2	6	0.27	0.37	0.14	0.24
P97864	Caspase-7 OS=Mus musculus GN=Casp7 PE=1 SV=2 - [CASP7_MOUSE]	34.65	1	12	12	26	0.09	0.23	0.13	0.14
Q922H9-2	Isoform 2 of Zinc finger protein 330 OS=Mus musculus GN=Znf330 - [ZN330_MOUSE]	4.50	2	2	2	4	0.05	0.11	-0.04	0.01
Q8BQB4	Four-jointed box protein 1 OS=Mus musculus GN=Fjx1 PE=1 SV=1 - [FJX1_MOUSE]	4.00	1	1	2	4	-0.94	-1.04	-0.54	-0.65
Q63886	UDP-glucuronosyltransferase 1-1 OS=Mus musculus GN=Ugt1a1 PE=2 SV=2 - [UD11_MOUSE]	19.25	13	4	9	74	-1.40	-1.32	-1.40	-1.40
Q8VEN2-2	Isoform 2 of Placenta-expressed transcript 1 protein OS=Mus musculus GN=Plet1 - [PLET1_MOUSE]	9.28	2	1	1	4	0.59	0.88	0.36	0.65
D3YWV8	Protein Sycp2l (Fragment) OS=Mus musculus GN=Sycp2l PE=2 SV=1 - [D3YWV8_MOUSE]	2.45	1	1	1	1	-0.10	0.32	-0.34	0.08
P60487	Pyridoxal phosphate phosphatase OS=Mus musculus GN=Pdxp PE=1 SV=1 - [PLPP_MOUSE]	8.56	1	2	2	3	-0.29	-0.22	-0.29	-0.23
Q8R0K2	E3 ubiquitin-protein ligase TRIM31 OS=Mus musculus GN=Trim31 PE=1 SV=1 - [TRI31_MOUSE]	19.92	1	10	10	34	0.29	0.30	0.21	0.25
Q9R1P1	Proteasome subunit beta type-3 OS=Mus musculus GN=Psmb3 PE=1 SV=1 - [PSB3_MOUSE]	23.41	3	6	6	43	-0.19	-0.08	-0.10	-0.15
Q99N15	17beta-hydroxysteroid dehydrogenase type 10/short chain L-3-hydroxyacyl-CoA dehydrogenase OS=Mus musculus GN=Hsd17b10 PE=2 SV=1 - [Q99N15_MOUSE]	59.00	3	12	12	161	-0.27	-0.17	-0.24	-0.26
O88792	Junctional adhesion molecule A OS=Mus musculus GN=F11r PE=1 SV=2 - [JAM1_MOUSE]	13.33	1	3	3	22	0.15	0.27	0.17	0.11

D3Z285	NFU1 iron-sulfur cluster scaffold homolog, mitochondrial (Fragment) OS=Mus musculus GN=Nfu1 PE=2 SV=1 - [D3Z285_MOUSE]	16.08	1	1	3	8	-0.56	-0.10	-0.48	0.01
Q9QZ23	NFU1 iron-sulfur cluster scaffold homolog, mitochondrial OS=Mus musculus GN=Nfu1 PE=1 SV=2 - [NFU1_MOUSE]	21.96	1	3	5	27	-0.11	-0.13	-0.15	-0.17
P63085	Mitogen-activated protein kinase 1 OS=Mus musculus GN=Mapk1 PE=1 SV=3 - [MK01_MOUSE]	52.51	12	11	18	291	-0.21	-0.24	-0.18	-0.18
Q9JHJ0	Tropomodulin-3 OS=Mus musculus GN=Tmod3 PE=1 SV=1 - [TMOD3_MOUSE]	40.06	1	12	13	138	-0.27	-0.10	-0.26	-0.15
Q923S6-3	Isoform 3 of Neuralized like protein 1A OS=Mus musculus GN=Neurl - [NEU1A_MOUSE]	7.34	3	1	1	2	0.16	-0.98	0.39	-0.75
Q6IRU2	Tropomyosin alpha-4 chain OS=Mus musculus GN=Tpm4 PE=2 SV=3 - [TPM4_MOUSE]	64.11	1	14	19	551	-0.21	-0.22	-0.24	-0.23
Q8CJG0	Protein argonaute-2 OS=Mus musculus GN=Ago2 PE=1 SV=3 - [AGO2_MOUSE]	19.53	2	11	14	58	-0.26	-0.08	-0.25	-0.20
P83940	Transcription elongation factor B polypeptide 1 OS=Mus musculus GN=Tceb1 PE=1 SV=1 [ELOC_MOUSE]	58.04	1	5	5	66	-0.20	-0.01	-0.17	0.06
J3QNB1	La-related protein 1 OS=Mus musculus GN=Larp1 PE=4 SV=1 [J3QNB1_MOUSE]	14.55	2	12	12	34	0.21	0.16	0.09	0.14
Q9Z2G9	Oxidoreductase HTATIP2 OS=Mus musculus GN=Htatip2 PE=1 SV=3 - [HTAI2_MOUSE]	16.53	2	4	4	6	-0.18	-0.33	-0.13	-0.28
Q6P5C5	Single-strand selective monofunctional uracil DNA glycosylase OS=Mus musculus GN=Smug1 PE=2 SV=1 - [SMUG1_MOUSE]	6.09	1	1	1	4	-0.06	-1.20	0.10	-1.04
A6H6A9-3	Isoform 3 of Rab GTPase-activating protein 1-like OS=Mus musculus GN=Rabgap1l - [RBG1L_MOUSE]	2.97	1	1	1	10	0.01	0.16	-0.13	0.11
Q62073	Mitogen-activated protein kinase kinase kinase 7 OS=Mus musculus GN=Map3k7 PE=1 SV=1 - [M3K7_MOUSE]	8.81	4	3	4	6	0.19	-0.28	0.14	0.15
J3QNK1	Synphilin-1 OS=Mus musculus GN=Sncaip PE=4 SV=1 - [J3QNK1_MOUSE]	5.26	6	3	3	6	-0.06	-0.24	-0.25	0.00

Q6NVF9	Cleavage and polyadenylation specificity factor subunit 6 OS=Mus musculus GN=Cpsf6 PE=1 SV=1 - [CPSF6_MOUSE]	20.33	5	9	9	65	0.04	-0.02	-0.04	-0.04
P45377	Aldose reductase-related protein 2 OS=Mus musculus GN=Akr1b8 PE=1 SV=2 - [ALD2_MOUSE]	45.57	1	6	12	116	0.21	0.33	0.31	0.27
P29416	Beta-hexosaminidase subunit alpha OS=Mus musculus GN=Hexa PE=2 SV=2 - [HEXA_MOUSE]	21.40	1	9	10	42	-0.01	-0.13	0.01	-0.06
Q9CRA8	Exosome complex component RRP46 OS=Mus musculus GN=Exosc5 PE=1 SV=1 - [EXOS5_MOUSE]	14.04	1	3	3	6	-0.51	-0.59	-0.09	-0.17
J3QQ26	Proliferating cell nuclear antigen OS=Mus musculus GN=Pcna-ps2 PE=3 SV=1 - [J3QQ26_MOUSE]	44.06	2	9	9	87	0.24	0.08	0.16	0.04
Q52KR3-3	Isoform 3 of Protein prune homolog 2 OS=Mus musculus GN=Prune2 - [PRUN2_MOUSE]	4.95	5	1	2	4	0.10	0.53	-0.02	0.41
Q8BYB9	Protein O-glucosyltransferase 1 OS=Mus musculus GN=Poglut1 PE=2 SV=2 - [PGLT1_MOUSE]	11.73	1	5	5	23	-0.11	-0.30	-0.05	-0.31
Q8CH25-2	Isoform 2 of SAFB-like transcription modulator OS=Mus musculus GN=Sltm - [SLTM_MOUSE]	11.94	2	9	10	25	-0.02	-0.11	0.02	-0.01
Q60715	Prolyl 4-hydroxylase subunit alpha-1 OS=Mus musculus GN=P4ha1 PE=2 SV=2 - [P4HA1_MOUSE]	25.47	3	10	10	32	-0.17	-0.23	-0.01	-0.24
P63158	High mobility group protein B1 OS=Mus musculus GN=Hmgb1 PE=1 SV=2 - [HMGB1_MOUSE]	42.33	3	15	17	483	0.67	0.84	0.52	0.73
Q9JJV2-3	Isoform 3 of Profilin-2 OS=Mus musculus GN=Pfn2 - [PROF2_MOUSE]	41.76	4	5	5	15	-0.24	-0.26	-0.31	-0.29
Q91VC7	Protein phosphatase 1 regulatory subunit 14A OS=Mus musculus GN=Ppp1r14a PE=2 SV=1 - [PP14A_MOUSE]	49.66	1	6	6	71	-0.16	-0.02	-0.15	0.06
P61358	60S ribosomal protein L27 OS=Mus musculus GN=Rpl27 PE=2 SV=2 [RL27_MOUSE]	40.44	3	6	6	70	-0.18	-0.08	-0.15	-0.03
Q9CR02-2	Isoform 2 of Translation machinery-associated protein 16 OS=Mus musculus GN=Tma16 - [TMA16_MOUSE]	11.39	2	1	1	1	0.03	0.17	0.07	0.21

D3Z3R1	60S ribosomal protein L36 OS=Mus musculus GN=Gm5745 PE=3 SV=1 - [D3Z3R1_MOUSE]	36.54	5	5	6	108	0.09	0.12	0.13	0.18
Q3TA68	Protein Wdr36 OS=Mus musculus GN=Wdr36 PE=2 SV=1 - [Q3TA68_MOUSE]	3.40	2	2	3	5	-0.10	-0.40	0.39	0.09
E9Q3X0	V-type proton ATPase 16 kDa proteolipid subunit OS=Mus musculus GN=Mvp PE=2 SV=1 - [E9Q3X0_MOUSE]	61.26	3	41	41	630	0.02	0.05	0.00	0.04
O88696	Putative ATP-dependent Clp protease proteolytic subunit, mitochondrial OS=Mus musculus GN=Clpp PE=2 SV=1 - [CLPP_MOUSE]	31.99	1	8	8	91	-0.16	-0.06	-0.17	-0.06
P56677	Suppressor of tumorigenicity 14 protein homolog OS=Mus musculus GN=St14 PE=1 SV=2 - [ST14_MOUSE]	8.42	2	6	6	26	-0.39	0.10	-0.20	0.15
P61965	WD repeat-containing protein 5 OS=Mus musculus GN=Wdr5 PE=1 SV=1 - [WDR5_MOUSE]	21.26	2	5	5	19	-0.57	-0.17	-0.34	-0.13
Q8R326-2	Isoform 2 of Paraspeckle component 1 OS=Mus musculus GN=Pspc1 - [PSPC1_MOUSE]	24.03	3	10	11	43	-0.20	-0.12	-0.10	-0.18
O88466	Zinc finger protein 106 OS=Mus musculus GN=Znf106 PE=1 SV=3 - [ZN106_MOUSE]	1.43	5	2	2	15	0.06	-0.13	-0.01	-0.21
Q8BH57-2	Isoform 2 of WD repeat-containing protein 48 OS=Mus musculus GN=Wdr48 - [WDR48_MOUSE]	15.56	3	8	8	16	0.00	-0.01	-0.19	-0.20
Q9D3U0	Putative tRNA pseudouridine synthase Pus10 OS=Mus musculus GN=Pus10 PE=1 SV=1 - [PUS10_MOUSE]	3.04	2	2	2	6	-0.07	0.27	-0.03	0.31
Q8VE38-2	Isoform 2 of Oxidoreductase NAD-binding domain-containing protein 1 OS=Mus musculus GN=Oxnad1 - [OXND1_MOUSE]	19.20	7	4	4	12	0.01	-0.01	-0.07	-0.05
Q3U422	NADH dehydrogenase [ubiquinone] flavoprotein 3, mitochondrial OS=Mus musculus GN=Ndufv3 PE=2 SV=1 - [Q3U422_MOUSE]	49.57	1	10	13	67	-0.42	-0.06	-0.50	-0.27
E9Q2A6	Protein-tyrosine kinase 2-beta OS=Mus musculus GN=Ptk2b PE=2 SV=1 - [E9Q2A6_MOUSE]	8.69	4	6	7	24	-0.06	-0.07	0.03	0.04

P14428	H-2 class I histocompatibility antigen, K-Q alpha chain (Fragment) OS=Mus musculus GN=H2-K1 PE=2 SV=1 - [HA1Q_MOUSE]	24.39	2	2	5	16	-0.18	-0.45	-0.44	-0.71
Q8BP92	Reticulocalbin-2 OS=Mus musculus GN=Rcn2 PE=2 SV=1 - [RCN2_MOUSE]	39.38	1	9	10	148	-0.31	-0.23	-0.47	-0.28
F6R8U9	Sulfatase-modifying factor 2 (Fragment) OS=Mus musculus GN=Sumf2 PE=4 SV=1 - [F6R8U9_MOUSE]	9.73	2	2	2	4	-0.33	-0.45	-0.23	-0.35
Q9DCM2	Glutathione S-transferase kappa 1 OS=Mus musculus GN=Gstk1 PE=1 SV=3 [GSTK1_MOUSE]	57.08	1	11	12	91	-0.28	-0.47	-0.28	-0.33
Q61140	Breast cancer anti-estrogen resistance protein 1 OS=Mus musculus GN=Bcar1 PE=1 SV=2 - [BCAR1_MOUSE]	6.86	2	4	4	8	0.18	-0.76	0.19	-0.78
Q3TQI7	Uncharacterized protein C9orf78 homolog OS=Mus musculus PE=1 SV=2 - [CI078_MOUSE]	16.96	1	3	3	5	-0.34	-0.14	-0.38	-0.18
Q6ZPF4-2	Isoform 2 of Formin-like protein 3 OS=Mus musculus GN=Fmnl3 - [FMNL3_MOUSE]	1.95	4	2	2	2	0.04	-0.38	-0.29	-0.71
Q7TNE3	Sperm-associated antigen 7 OS=Mus musculus GN=Spag7 PE=1 SV=1 - [SPAG7_MOUSE]	15.86	2	3	3	17	0.00	-0.33	-0.06	-0.49
J3QNB4	Uncharacterized protein OS=Mus musculus GN=Gm20091 PE=4 SV=1 - [J3QNB4_MOUSE]	14.29	2	1	1	4	-0.29	-0.31	-0.53	-0.54
Q8BVA5	UPF0554 protein C2orf43 homolog OS=Mus musculus PE=2 SV=1 - [CB043_MOUSE]	11.96	3	3	3	12	-0.07	0.19	-0.04	0.52
E9Q3I4	Intersectin-1 OS=Mus musculus GN=Itsn1 PE=2 SV=1 - [E9Q3I4_MOUSE]	21.17	12	20	21	56	-0.15	-0.14	-0.18	-0.13
D3Z141	Galectin-7 (Fragment) OS=Mus musculus GN=Lgals7 PE=2 SV=1 - [D3Z141_MOUSE]	25.58	3	1	1	1	-0.09	-0.08	-0.07	-0.07
Q8C0J2-2	Isoform 2 of Autophagy related protein 16-1 OS=Mus musculus GN=Atg16l1 - [A16L1_MOUSE]	15.48	5	7	9	17	-0.49	-0.55	-0.31	-0.38
Q9CPV4	Glyoxalase domain-containing protein 4 OS=Mus musculus GN=Glod4 PE=2 SV=1 [GLOD4_MOUSE]	67.45	8	16	16	189	0.05	0.08	0.01	0.07
Q9CQC9	GTP-binding protein SAR1b OS=Mus musculus GN=Sar1b PE=1 SV=1 - [SAR1B_MOUSE]	17.17	1	2	3	34	-0.53	-0.32	-0.31	-0.23

Q9EPU4	Cleavage and polyadenylation specificity factor subunit 1 OS=Mus musculus GN=Cpsf1 PE=1 SV=1 - [CPSF1_MOUSE]	6.18	1	8	8	22	-0.16	-0.10	-0.10	0.00
Q9CPP0	Nucleoplasmin-3 OS=Mus musculus GN=Npm3 PE=2 SV=3 [NPM3_MOUSE]	8.57	1	1	1	11	-0.47	-0.18	-0.43	-0.15
Q9QYS9-4	Isoform 4 of Protein quaking OS=Mus musculus GN=Qki - [QKI_MOUSE]	16.93	8	4	5	8	-0.04	-0.27	-0.22	-0.17
Q3TFP0	Serine/arginine-rich-splicing factor 10 OS=Mus musculus GN=Srsf10 PE=2 SV=1 - [Q3TFP0_MOUSE]	26.92	5	4	4	25	0.08	-0.07	-0.06	-0.11
P62748	Hippocalcin-like protein 1 OS=Mus musculus GN=Hpcal1 PE=2 SV=2 - [HPCL1_MOUSE]	64.77	2	8	11	64	-0.30	-0.32	-0.25	-0.30
Q8R480	Nuclear pore complex protein Nup85 OS=Mus musculus GN=Nup85 PE=1 SV=1 - [NUP85_MOUSE]	3.66	1	2	2	19	-0.69	-0.45	-0.52	-0.25
G3UWG1	MCG115977 OS=Mus musculus GN=Gm10108 PE=3 SV=1 - [G3UWG1_MOUSE]	67.62	3	10	10	263	-0.41	-0.25	-0.47	-0.37
Q60864	Stress-induced-phosphoprotein 1 OS=Mus musculus GN=Stip1 PE=1 SV=1 - [STIP1_MOUSE]	58.75	1	35	35	388	0.03	0.08	-0.06	0.04
O54782	Epididymis-specific alpha-mannosidase OS=Mus musculus GN=Man2b2 PE=2 SV=2 - [MA2B2_MOUSE]	5.80	2	4	4	8	-0.49	-0.33	-0.68	-0.50
A2RS43	Protein Pcdh7 OS=Mus musculus GN=Pcdh7 PE=2 SV=1 - [A2RS43_MOUSE]	4.86	2	3	3	8	-0.20	-0.35	-0.47	-0.36
E9QAB6	Protein Ptar1 OS=Mus musculus GN=Ptar1 PE=4 SV=1 - [E9QAB6_MOUSE]	2.36	1	1	1	5	0.07	-0.08	0.04	-0.11
A2RSX7	tRNA wybutosine-synthesizing protein 5 OS=Mus musculus GN=Tyw5 PE=2 SV=2 - [TYW5_MOUSE]	2.22	1	1	1	3	-0.47	-0.37	-0.33	-0.23
P68433	Histone H3.1 OS=Mus musculus GN=Hist1h3a PE=1 SV=2 - [H31_MOUSE]	33.82	2	2	8	380	0.28	0.43	0.12	0.19
E9QLX1	Antigen-presenting glycoprotein CD1d2 OS=Mus musculus GN=Cd1d2 PE=4 SV=2 - [E9QLX1_MOUSE]	2.39	3	1	1	2	-0.62	-0.37	-0.68	-0.44
F8VQ43	Laminin subunit alpha-2 OS=Mus musculus GN=Lama2 PE=2 SV=1 - [F8VQ43_MOUSE]	13.02	2	36	36	101	-0.79	-0.89	-0.94	-0.89

Q571E4	N-acetylgalactosamine-6-sulfatase OS=Mus musculus GN=Galns PE=2 SV=2 - [GALNS_MOUSE]	8.27	1	4	4	9	-0.67	-0.45	-0.72	-0.50
Q8VCI0	Phospholipase B-like 1 OS=Mus musculus GN=Plbd1 PE=1 SV=1 [PLBL1_MOUSE]	23.82	1	10	10	35	0.01	-0.07	0.19	0.14
Q6PHS9-4	Isoform 4 of Voltage-dependent calcium channel subunit alpha-2/delta-2 OS=Mus musculus GN=Cacna2d2 - [CA2D2_MOUSE]	2.18	7	1	2	5	-0.31	0.15	-0.43	0.02
G3X9K8	TP53RK-binding protein OS=Mus musculus GN=Tprkb PE=3 SV=1 - [G3X9K8_MOUSE]	16.46	2	1	1	7	-0.63	-1.27	-0.58	-1.21
Q8BN21-2	Isoform 2 of Serine/threonine-protein kinase VRK2 OS=Mus musculus GN=Vrk2 - [VRK2_MOUSE]	2.86	2	1	1	3	-0.09	0.47	0.02	0.57
Q3UHD6-2	Isoform 2 of Sorting nexin-27 OS=Mus musculus GN=Snx27 - [SNX27_MOUSE]	10.08	5	4	4	9	0.21	0.09	-0.06	-0.18
P62878	E3 ubiquitin-protein ligase RBX1 OS=Mus musculus GN=Rbx1 PE=1 SV=1 - [RBX1_MOUSE]	30.56	1	3	3	6	0.07	0.04	-0.17	-0.08
Q8BHG1	Nardilysin OS=Mus musculus GN=Nrd1 PE=1 SV=1 - [NRDC_MOUSE]	20.50	4	22	22	86	-0.07	-0.06	-0.05	-0.05
Q71RI9-2	Isoform 2 of Kynurenine--oxoglutarate transaminase 3 OS=Mus musculus GN=Ccbl2 - [KAT3_MOUSE]	12.62	3	5	5	12	-0.14	0.02	-0.23	0.03
Q8CHR6	Dihydropyrimidine dehydrogenase [NADP(+)] OS=Mus musculus GN=Dpyd PE=2 SV=1 - [DPYD_MOUSE]	6.34	1	4	4	16	-0.88	-0.88	-0.96	-1.04
B2KF50	Protein Uhrf1bp1 OS=Mus musculus GN=Uhrf1bp1 PE=2 SV=1 - [B2KF50_MOUSE]	1.68	1	2	2	4	-0.07	0.11	0.21	0.39
K3W4Q5	Protein Fam186a OS=Mus musculus GN=Fam186a PE=4 SV=1 - [K3W4Q5_MOUSE]	0.86	2	2	2	2	-0.26	-0.53	-0.45	-0.73
G3X925	Pyruvate kinase OS=Mus musculus GN=Pklr PE=3 SV=1 - [G3X925_MOUSE]	39.20	4	17	20	134	-0.51	-0.38	-0.53	-0.43
P35290	Ras-related protein Rab-24 OS=Mus musculus GN=Rab24 PE=1 SV=2 - [RAB24_MOUSE]	30.05	1	5	5	8	-0.13	0.07	-0.34	-0.17
D3YWR1	Glucosamine-6-phosphate isomerase 2 OS=Mus musculus GN=Gnpda2 PE=2 SV=1 - [D3YWR1_MOUSE]	27.04	4	3	4	17	-0.26	0.03	-0.65	-0.22
O54851	Gap junction delta-2 protein OS=Mus musculus GN=Gjd2 PE=2 SV=2 - [CXD2_MOUSE]	6.54	1	1	1	2	-0.11	-0.03	-0.13	-0.05

Q80ZS3	28S ribosomal protein S26, mitochondrial OS=Mus musculus GN=Mrps26 PE=2 SV=1 - [RT26_MOUSE]	20.00	1	4	4	17	-0.02	0.06	0.04	0.00
O35593	26S proteasome non-ATPase regulatory subunit 14 OS=Mus musculus GN=Psmd14 PE=1 SV=2 - [PSDE_MOUSE]	18.06	1	7	7	31	0.03	0.21	0.04	0.13
Q3THS6	S-adenosylmethionine synthase isoform type-2 OS=Mus musculus GN=Mat2a PE=2 SV=2 - [METK2_MOUSE]	54.94	1	15	18	189	-0.25	-0.36	-0.27	-0.36
A3KMP2	Tetratricopeptide repeat protein 38 OS=Mus musculus GN=Ttc38 PE=2 SV=2 - [TTC38_MOUSE]	47.10	3	18	18	170	-0.68	-0.69	-0.58	-0.58
Q9ERR7	15 kDa selenoprotein OS=Mus musculus GN=Sep15 PE=1 SV=3 [SEP15_MOUSE]	29.63	2	4	4	88	-0.18	0.09	-0.29	-0.03
Q91Z31	Polypyrimidine tract-binding protein 2 OS=Mus musculus GN=Ptbp2 PE=1 SV=2 [PTBP2_MOUSE]	9.04	2	3	4	4	0.41	0.50	0.17	0.26
Q8BGD9	Eukaryotic translation initiation factor 4B OS=Mus musculus GN=Eif4b PE=1 SV=1 - [IF4B_MOUSE]	23.90	1	13	13	75	0.01	0.21	-0.12	0.01
Q91VE6-2	Isoform 2 of MKI67 FHA domain-interacting nucleolar phosphoprotein OS=Mus musculus GN=Mki67ip - [MK67I_MOUSE]	11.90	2	3	4	7	0.20	-0.33	0.39	-0.14
Q8K0C1	Importin-13 OS=Mus musculus GN=Ipo13 PE=2 SV=1 - [IPO13_MOUSE]	1.45	2	1	1	1	0.13	-0.12	0.29	0.04
Q9D8T2	Gasdermin-D OS=Mus musculus GN=Gsdmdc1 PE=2 SV=1 - [GSDMD_MOUSE]	29.16	1	11	11	76	0.26	0.26	0.21	0.27
Q9CYK1	Tryptophan--tRNA ligase, mitochondrial OS=Mus musculus GN=Wars2 PE=2 SV=2 - [SYWM_MOUSE]	15.56	1	5	5	9	-0.32	-0.31	-0.24	-0.31
Q99NB8	Ubiquilin-4 OS=Mus musculus GN=Ubqln4 PE=1 SV=1 - [UBQL4_MOUSE]	13.42	1	4	6	48	0.03	0.02	0.11	0.06
Q8HWE6	MHC I like leukocyte 2 OS=Mus musculus GN=Mill2 PE=2 SV=1 - [Q8HWE6_MOUSE]	4.41	2	1	1	1	-0.29	-0.84	-0.79	-1.35
Q9QXA5	U6 snRNA-associated Sm-like protein LSm4 OS=Mus musculus GN=Lsm4 PE=2 SV=1 [LSM4_MOUSE]	26.28	3	4	4	13	0.23	-0.08	0.20	-0.07
Q99JX7	Nuclear RNA export factor 1 OS=Mus musculus GN=Nxf1 PE=1 SV=3 - [NXF1_MOUSE]	10.68	1	5	5	11	0.08	0.02	-0.03	-0.10

Q9D6V8	Polyadenylate-binding protein-interacting protein 2 OS=Mus musculus GN=Paip2 PE=2 SV=1 - [PAIP2_MOUSE]	6.45	1	1	1	4	-0.13	0.17	-0.10	0.20
P50428	Arylsulfatase A OS=Mus musculus GN=Arsa PE=2 SV=2 - [ARSA_MOUSE]	7.71	1	4	4	14	-0.27	-0.26	-0.43	-0.18
Q148V8	Protein FAM83H OS=Mus musculus GN=Fam83h PE=1 SV=1 - [FA83H_MOUSE]	7.69	1	6	6	10	0.09	-0.46	-0.01	-0.15
Q9CSN1	SNW domain-containing protein 1 OS=Mus musculus GN=Snw1 PE=1 SV=3 - [SNW1_MOUSE]	25.56	1	11	12	54	0.17	0.21	0.19	0.35
Q08024	Core-binding factor subunit beta OS=Mus musculus GN=Cbfb PE=1 SV=1 - [PEBB_MOUSE]	30.48	4	5	5	11	0.15	0.40	0.18	0.37
Q8BJF9	Charged multivesicular body protein 2b OS=Mus musculus GN=Chmp2b PE=2 SV=1 - [CHM2B_MOUSE]	27.70	1	6	7	19	-0.08	0.01	-0.19	0.04
Q8BTJ4-2	Isoform 2 of Bis(5'-adenosyl)-triphosphatase enpp4 OS=Mus musculus GN=Enpp4 - [ENPP4_MOUSE]	5.20	2	2	2	4	-0.12	-0.20	-0.45	-0.53
P58064	28S ribosomal protein S6, mitochondrial OS=Mus musculus GN=Mrps6 PE=2 SV=3 - [RT06_MOUSE]	20.00	1	3	3	11	-0.07	-0.02	0.00	-0.10
B1AWT4	Ras-related GTP-binding protein D OS=Mus musculus GN=Rragd PE=2 SV=1 [B1AWT4_MOUSE]	4.30	5	1	1	2	-0.11	0.05	-0.11	0.05
P37040	NADPH--cytochrome P450 reductase OS=Mus musculus GN=Por PE=1 SV=2 - [NCPR_MOUSE]	32.01	5	16	16	158	-0.60	-0.62	-0.53	-0.61
Q8BJU0-2	Isoform 2 of Small glutamine-rich tetratricopeptide repeat-containing protein alpha OS=Mus musculus GN=Sgta - [SGTA_MOUSE]	29.30	2	8	8	37	-0.10	-0.03	-0.13	0.04
P56213	FAD-linked sulfhydryl oxidase ALR OS=Mus musculus GN=Gfer PE=2 SV=2 - [ALR_MOUSE]	16.16	1	2	2	6	0.08	0.22	0.03	0.17
Q811S7-2	Isoform 2 of Upstream-binding protein 1 OS=Mus musculus GN=Ubp1 - [UBIP1_MOUSE]	8.33	3	3	4	7	0.32	0.58	-0.07	0.18
Q9ERB0	Synaptosomal-associated protein 29 OS=Mus musculus GN=Snap29 PE=1 SV=1 - [SNP29_MOUSE]	20.00	1	5	5	11	-0.06	-0.41	-0.18	-0.45

Q8VC70	RNA-binding motif, single-stranded-interacting protein 2 OS=Mus musculus GN=Rbms2 PE=2 SV=1 - [RBMS2_MOUSE]	7.31	2	2	2	4	-0.02	0.12	-0.32	-0.18
Q9Z0U1	Tight junction protein ZO-2 OS=Mus musculus GN=Tjp2 PE=1 SV=2 - [ZO2_MOUSE]	28.79	1	25	26	122	0.11	0.23	0.14	0.22
Q3TYD6	Serine/threonine-protein kinase LMTK2 OS=Mus musculus GN=Lmtk2 PE=1 SV=3 - [LMTK2_MOUSE]	4.69	1	5	5	6	-0.04	-0.64	-0.39	-0.61
F6ZV59	Heterogeneous nuclear ribonucleoprotein D0 (Fragment) OS=Mus musculus GN=Hnrnpd PE=4 SV=1 - [F6ZV59_MOUSE]	57.87	10	12	13	181	0.03	0.13	-0.08	0.05
Q91W97	Putative hexokinase HKDC1 OS=Mus musculus GN=Hkdc1 PE=2 SV=1 - [HKDC1_MOUSE]	7.87	1	2	6	48	-0.15	-0.19	0.03	-0.01
O89023	Tripeptidyl-peptidase 1 OS=Mus musculus GN=Tpp1 PE=1 SV=2 - [TPP1_MOUSE]	1.25	1	1	1	1	-0.44	-0.27	-0.62	-0.45
Q9CQ02	COMM domain-containing protein 4 OS=Mus musculus GN=Commd4 PE=2 SV=1 - [COMD4_MOUSE]	14.07	1	2	2	4	0.09	0.22	-0.01	0.11
Q3UH53-2	Isoform 2 of Protein sidekick-1 OS=Mus musculus GN=Sdk1 - [SDK1_MOUSE]	4.35	2	4	5	8	-0.38	-0.16	0.01	-0.15
Q8BGB7	Enolase-phosphatase E1 OS=Mus musculus GN=Enoph1 PE=2 SV=1 - [ENOPH_MOUSE]	25.68	2	5	5	32	-0.12	-0.27	-0.11	-0.22
P35821	Tyrosine-protein phosphatase non-receptor type 1 OS=Mus musculus GN=Ptpn1 PE=1 SV=2 [PTN1_MOUSE]	13.19	1	4	4	10	0.00	-0.48	-0.05	-0.31
Q6PNC0	DmX-like protein 1 OS=Mus musculus GN=Dmxl1 PE=1 SV=1 - [DMXL1_MOUSE]	1.86	2	4	5	5	-1.06	-0.85	-0.57	-0.36
D3YZX8	Signal recognition particle 9 kDa protein OS=Mus musculus GN=Srp9 PE=2 SV=1 - [D3YZX8_MOUSE]	12.70	2	1	1	4	-0.29	-0.25	-0.23	-0.18
P62301	40S ribosomal protein S13 OS=Mus musculus GN=Rps13 PE=1 SV=2 - [RS13_MOUSE]	39.07	1	7	7	90	0.07	0.11	-0.02	-0.06
Q9EQ80	NIF3-like protein 1 OS=Mus musculus GN=Nif3l1 PE=1 SV=4 - [NIF3L_MOUSE]	18.35	3	5	5	18	-0.10	-0.04	-0.13	0.01
P62482	Voltage-gated potassium channel subunit beta-2 OS=Mus musculus GN=Kcnab2 PE=1 SV=1 - [KCAB2_MOUSE]	4.90	5	1	3	10	0.25	-0.05	-0.07	-0.37

Q3TC72	Fumarylacetoacetate hydrolase domain-containing protein 2A OS=Mus musculus GN=Fahd2 PE=1 SV=1 - [FAHD2_MOUSE]	24.92	1	6	6	23	-0.45	-0.42	-0.54	-0.55
Q99K48	Non-POU domain-containing octamer-binding protein OS=Mus musculus GN=Nono PE=1 SV=3 - [NONO_MOUSE]	31.29	3	12	14	194	-0.08	-0.01	-0.05	-0.02
O35130	Ribosomal RNA small subunit methyltransferase NEP1 OS=Mus musculus GN=Emg1 PE=1 SV=1 [NEP1_MOUSE]	21.72	1	4	4	9	0.12	0.16	-0.10	-0.04
E9Q3G8	Protein Nup153 OS=Mus musculus GN=Nup153 PE=2 SV=1 - [E9Q3G8_MOUSE]	10.88	1	13	13	27	0.00	0.04	-0.17	-0.02
Q9D404	3-oxoacyl-[acyl-carrier-protein] synthase, mitochondrial OS=Mus musculus GN=Oxsm PE=2 SV=1 - [OXSM_MOUSE]	14.16	2	4	4	15	-0.25	-0.04	-0.30	-0.13
E9Q3I3	Spermatogenesis-associated protein 13 OS=Mus musculus GN=Spata13 PE=4 SV=1 - [E9Q3I3_MOUSE]	1.85	4	2	3	4	-0.39	-0.34	-0.15	-0.11
A2ATL1	MCG17280 OS=Mus musculus GN=Fam171a1 PE=4 SV=1 - [A2ATL1_MOUSE]	3.13	3	1	1	1	-0.14	-0.21	-0.31	-0.39
Q9CQR6	Serine/threonine-protein phosphatase 6 catalytic subunit OS=Mus musculus GN=Ppp6c PE=2 SV=1 - [PPP6_MOUSE]	25.57	1	7	7	15	-0.19	-0.11	-0.05	0.22
Q8BIQ5-2	Isoform 2 of Cleavage stimulation factor subunit 2 OS=Mus musculus GN=Cstf2 - [CSTF2_MOUSE]	26.86	7	11	11	48	-0.02	0.00	-0.13	-0.10
G3UYF9	Prefoldin subunit 6 OS=Mus musculus GN=H2-Ke2 PE=2 SV=1 - [G3UYF9_MOUSE]	57.43	1	1	6	35	-0.11	-0.10	-0.18	-0.17
Q9DCT1	1,5-anhydro-D-fructose reductase OS=Mus musculus GN=Akr1e2 PE=1 SV=1 - [AKCL2_MOUSE]	30.23	2	8	8	59	-0.46	-0.33	-0.60	-0.42
Q9R097	Kunitz-type protease inhibitor 1 OS=Mus musculus GN=Spint1 PE=1 SV=2 - [SPIT1_MOUSE]	23.87	1	7	7	17	0.32	0.34	0.40	0.39
Q8K4Z3	NAD(P)H-hydrate epimerase OS=Mus musculus GN=Apoa1bp PE=1 SV=1 - [NNRE_MOUSE]	25.18	1	7	7	65	-0.15	-0.22	-0.26	-0.25
Q3TML4	Gene model 773, (NCBI) OS=Mus musculus GN=Gm773 PE=2 SV=1 - [Q3TML4_MOUSE]	9.22	1	1	1	1	-0.16	-0.47	-0.22	-0.53

A2AAY5	SH3 and PX domain-containing protein 2B OS=Mus musculus GN=Sh3pxd2b PE=1 SV=1 - [SPD2B_MOUSE]	10.79	1	8	9	16	-0.05	0.01	-0.16	-0.07
Q6PIP5	NudC domain-containing protein 1 OS=Mus musculus GN=Nudcd1 PE=2 SV=2 - [NUDC1_MOUSE]	15.81	1	7	7	27	0.01	-0.16	-0.09	-0.26
D3YZD6	Quinone oxidoreductase like protein 1 OS=Mus musculus GN=Cryzl1 PE=2 SV=1 - [D3YZD6_MOUSE]	14.67	5	3	3	7	-0.14	-0.02	-0.29	-0.19
Q9Z315	U4/U6.U5 tri-snRNP-associated protein 1 OS=Mus musculus GN=Sart1 PE=2 SV=1 - [SNUT1_MOUSE]	14.14	1	8	8	25	0.09	0.22	0.06	0.09
Q921E2	Ras-related protein Rab-31 OS=Mus musculus GN=Rab31 PE=1 SV=1 - [RAB31_MOUSE]	46.91	3	5	7	28	-0.14	-0.24	-0.39	-0.03
B0QZS8	Mitochondrial import inner membrane translocase subunit Tim22 OS=Mus musculus GN=Timm22 PE=2 SV=1 - [B0QZS8_MOUSE]	4.26	2	1	1	1	-0.10	0.35	-0.13	0.32
P28474	Alcohol dehydrogenase class-3 OS=Mus musculus GN=Adh5 PE=1 SV=3 - [ADHX_MOUSE]	37.97	1	12	13	353	-0.39	-0.39	-0.47	-0.43
P97298	Pigment epithelium-derived factor OS=Mus musculus GN=Serpinf1 PE=1 SV=2 - [PEDF_MOUSE]	16.55	4	5	5	9	-0.36	-0.39	-0.26	-0.41
O54824	Pro-interleukin-16 OS=Mus musculus GN=Il16 PE=1 SV=3 - [IL16_MOUSE]	7.19	2	8	8	27	-0.04	-0.31	-0.04	-0.15
B0V2N1-2	Isoform 2 of Receptor-type tyrosine-protein phosphatase S OS=Mus musculus GN=Ptprs - [PTPRS_MOUSE]	3.78	6	4	5	13	-0.25	0.07	-0.60	-0.40
O35216	Histone H3-like centromeric protein A OS=Mus musculus GN=Cenpa PE=2 SV=1 - [CENPA_MOUSE]	5.22	1	1	1	2	-0.95	-1.15	-0.65	-0.85
Q80X98	DEAH (Asp-Glu-Ala-His) box polypeptide 38 OS=Mus musculus GN=Dhx38 PE=2 SV=1 - [Q80X98_MOUSE]	2.04	1	1	1	1	0.87	-0.10	0.58	-0.38
P16045	Galectin-1 OS=Mus musculus GN=Lgals1 PE=1 SV=3 - [LEG1_MOUSE]	80.74	1	9	9	490	-0.13	0.01	-0.20	-0.03
Q91VI7	Ribonuclease inhibitor OS=Mus musculus GN=Rnh1 PE=1 SV=1 - [RINI_MOUSE]	41.89	1	16	16	205	0.14	0.17	0.15	0.18
Q9CQJ4	E3 ubiquitin-protein ligase RING2 OS=Mus musculus GN=Rnf2 PE=1 SV=1 - [RING2_MOUSE]	12.50	1	3	4	8	-0.34	-0.46	-0.07	-0.19

Q9D306	Alpha-1,3-mannosyl-glycoprotein 4-beta-N-acetylglucosaminyltransferase C OS=Mus musculus GN=Mgat4c PE=2 SV=1 - [MGT4C_MOUSE]	13.81	5	6	6	29	0.24	0.48	0.12	0.28
F8VQD3	Protein Vmn2r37 OS=Mus musculus GN=Vmn2r37 PE=3 SV=1 - [F8VQD3_MOUSE]	7.61	11	2	3	6	-0.25	0.21	-0.72	-0.26
Q9WTX5	S-phase kinase-associated protein 1 OS=Mus musculus GN=Skp1 PE=1 SV=3 - [SKP1_MOUSE]	49.08	4	8	8	133	-0.10	-0.01	-0.15	-0.08
Q8K1T1	Leucine-rich repeat-containing protein 25 OS=Mus musculus GN=Lrrc25 PE=1 SV=1 - [LRC25_MOUSE]	10.44	1	2	2	8	-0.55	-0.56	-0.13	-0.14
Q8CCA5	Protein Apol10a OS=Mus musculus GN=Apol10a PE=2 SV=1 - [Q8CCA5_MOUSE]	18.87	1	5	5	26	0.06	-0.04	0.26	-0.02
Q9EPL2	Calsyntenin-1 OS=Mus musculus GN=Clstn1 PE=1 SV=1 - [CSTN1_MOUSE]	5.21	2	3	3	6	0.19	0.20	0.05	-0.01
Q8K298	Actin-binding protein anillin OS=Mus musculus GN=Anln PE=1 SV=2 - [ANLN_MOUSE]	3.03	1	2	2	2	0.34	0.21	0.08	-0.06
Q8VDK1-2	Isoform 2 of Nitrilase homolog 1 OS=Mus musculus GN=Nit1 - [NIT1_MOUSE]	27.93	5	6	6	25	-0.08	-0.36	-0.14	-0.26
P97868-2	Isoform 2 of E3 ubiquitin-protein ligase RBBP6 OS=Mus musculus GN=Rbbp6 - [RBBP6_MOUSE]	2.33	2	4	4	14	0.14	0.18	0.15	0.16
Q3TIU4	2',5'-phosphodiesterase 12 OS=Mus musculus GN=Pde12 PE=2 SV=2 - [PDE12_MOUSE]	14.64	1	6	6	39	0.00	-0.14	0.03	-0.14
Q6YI28	EP1 OS=Mus musculus GN=2210407C18Rik PE=2 SV=1 - [Q6YI28_MOUSE]	20.00	1	4	4	10	-2.46	-2.38	-2.12	-2.04
Q19AB2	ROBO2 isoform b OS=Mus musculus GN=Robo2 PE=2 SV=1 - [Q19AB2_MOUSE]	2.92	2	3	3	3	0.23	0.01	-0.33	-0.55
Q99MR1	PERQ amino acid-rich with GYF domain-containing protein 1 OS=Mus musculus GN=Gigyf1 PE=1 SV=2 - [PERQ1_MOUSE]	5.75	1	2	2	4	-0.60	1.08	-2.42	-0.21
F2Z4B3	Cyclin-dependent kinase 2-associated protein 1 OS=Mus musculus GN=Cdk2ap1 PE=2 SV=1 - [F2Z4B3_MOUSE]	41.38	4	2	2	2	0.02	0.36	0.06	0.40
Q922U1	U4/U6 small nuclear ribonucleoprotein Prp3 OS=Mus musculus GN=Prpf3 PE=1 SV=1 - [PRPF3_MOUSE]	13.32	2	8	9	35	-0.06	-0.09	-0.14	-0.08

G3UXW3	Protein Ccdc28a (Fragment) OS=Mus musculus GN=Ccdc28a PE=4 SV=1 - [G3UXW3_MOUSE]	32.35	1	1	1	1	-1.52	-0.72	-1.40	-0.60
Q8CIG0	DEP domain-containing protein 1A OS=Mus musculus GN=Depdc1a PE=1 SV=1 - [DEP1A_MOUSE]	2.36	3	2	2	2	-0.35	-0.37	-0.48	-0.51
Q8BQ30	Phostensin OS=Mus musculus GN=Ppp1r18 PE=1 SV=1 - [PPR18_MOUSE]	9.60	2	5	5	10	0.12	-0.05	0.05	0.14
Q8CFZ0	SUMO-conjugating enzyme UBC9 OS=Mus musculus GN=Ube2i PE=4 SV=1 - [Q8CFZ0_MOUSE]	54.46	3	7	7	50	0.44	0.47	0.33	0.41
Q9CR86	Calcium-regulated heat stable protein 1 OS=Mus musculus GN=Carhsp1 PE=1 SV=1 - [CHSP1_MOUSE]	54.05	2	6	6	35	-0.03	0.00	-0.07	-0.09
P39749	Flap endonuclease 1 OS=Mus musculus GN=Fen1 PE=1 SV=1 - [FEN1_MOUSE]	15.08	2	6	6	23	0.33	0.44	0.33	0.36
P05202	Aspartate aminotransferase, mitochondrial OS=Mus musculus GN=Got2 PE=1 SV=1 - [AATM_MOUSE]	41.16	1	19	20	394	-0.36	-0.31	-0.36	-0.32
Q93092	Transaldolase OS=Mus musculus GN=Taldo1 PE=1 SV=2 - [TALDO_MOUSE]	43.62	1	20	20	343	-0.31	-0.19	-0.35	-0.22
A8Y5Q6	Mediator of RNA polymerase II transcription subunit 18 (Fragment) OS=Mus musculus GN=Med18 PE=2 SV=1 - [A8Y5Q6_MOUSE]	8.20	2	1	1	1	0.60	0.54	0.16	0.10
Q5NCU4	SPARC OS=Mus musculus GN=Sparc PE=2 SV=1 - [Q5NCU4_MOUSE]	15.61	3	4	4	9	-0.17	-0.35	-0.16	-0.34
P35822	Receptor-type tyrosine-protein phosphatase kappa OS=Mus musculus GN=Ptprk PE=1 SV=1 - [PTPRK_MOUSE]	10.84	1	10	12	36	-0.12	-0.17	-0.36	-0.24
Q3U2G2	Heat shock 70 kDa protein 4 OS=Mus musculus GN=Hspa4 PE=2 SV=1 - [Q3U2G2_MOUSE]	60.57	2	44	47	539	-0.15	-0.09	-0.20	-0.11
P09803	Cadherin-1 OS=Mus musculus GN=Cdh1 PE=1 SV=1 - [CADH1_MOUSE]	31.00	1	21	21	199	-0.03	-0.10	-0.04	-0.08
Q9D219	B-cell CLL/lymphoma 9 protein OS=Mus musculus GN=Bcl9 PE=1 SV=3 - [BCL9_MOUSE]	2.39	1	1	2	2	0.00	-0.03	-0.02	-0.06
B2RTM1	Transmembrane protein 158 OS=Mus musculus GN=Tmem158 PE=2 SV=1 - [B2RTM1_MOUSE]	5.59	2	1	1	1	0.14	0.29	-0.02	0.13

Q9R059	Four and a half LIM domains protein 3 OS=Mus musculus GN=Fhl3 PE=2 SV=2 - [FHL3_MOUSE]	19.03	3	5	5	17	-0.50	-0.37	-0.28	-0.43
O88572	Low-density lipoprotein receptor-related protein 6 OS=Mus musculus GN=Lrp6 PE=1 SV=1 - [LRP6_MOUSE]	1.67	1	1	1	1	-0.20	0.16	-0.36	0.00
F6RPJ9	Insulin-degrading enzyme (Fragment) OS=Mus musculus GN=Ide PE=3 SV=1 - [F6RPJ9_MOUSE]	27.96	2	27	27	101	-0.55	-0.50	-0.50	-0.45
Q9D820-2	Isoform 2 of Prolyl-tRNA synthetase associated domain-containing protein 1 OS=Mus musculus GN=Prorsd1 - [PRXD1_MOUSE]	14.07	2	2	2	7	0.07	0.22	0.04	0.18
Q9ERG0	LIM domain and actin-binding protein 1 OS=Mus musculus GN=Lima1 PE=1 SV=3 - [LIMA1_MOUSE]	40.11	2	26	28	145	-0.10	-0.08	-0.11	-0.12
P10493	Nidogen-1 OS=Mus musculus GN=Nid1 PE=1 SV=2 - [NID1_MOUSE]	37.35	1	36	37	338	-0.67	-0.74	-0.61	-0.68
Q78PG9	Coiled-coil domain-containing protein 25 OS=Mus musculus GN=Ccdc25 PE=1 SV=1 - [CCD25_MOUSE]	24.04	1	6	7	18	-0.06	0.10	-0.10	0.07
Q8BS28	Protein Gm10032 OS=Mus musculus GN=Gm10032 PE=2 SV=1 - [Q8BS28_MOUSE]	11.90	1	1	1	2	-0.58	-0.14	-0.82	-0.38
Q3UND0-2	Isoform 2 of Src kinase-associated phosphoprotein 2 OS=Mus musculus GN=Skap2 - [SKAP2_MOUSE]	7.41	2	3	3	9	0.09	0.17	-0.13	0.19
P33609	DNA polymerase alpha catalytic subunit OS=Mus musculus GN=Pola1 PE=1 SV=2 - [DPOLA_MOUSE]	1.09	1	1	1	3	0.18	0.00	-0.01	-0.19
P62869	Transcription elongation factor B polypeptide 2 OS=Mus musculus GN=Tceb2 PE=1 SV=1 [ELOB_MOUSE]	55.93	1	5	5	70	-0.14	-0.10	-0.23	-0.19
Q9Z2A7	Diacylglycerol O-acyltransferase 1 OS=Mus musculus GN=Dgat1 PE=1 SV=1 [DGAT1_MOUSE]	11.04	3	3	3	8	-0.28	-0.09	-0.51	-0.39
B2RQS1	Striatin-3 OS=Mus musculus GN=Strn3 PE=2 SV=1 - [B2RQS1_MOUSE]	13.62	1	1	8	24	-0.26	0.03	-0.95	-0.66
Q7TPE5	Probable RNA polymerase II nuclear localization protein SLC7A6OS OS=Mus musculus GN=Slc7a6os PE=1 SV=1 - [S7A6O_MOUSE]	3.59	1	1	1	2	-0.03	0.10	-0.20	-0.07

Q8C341	SUN domain-containing ossification factor OS=Mus musculus GN=Suco PE=1 SV=3 - [SUCO_MOUSE]	5.12	3	3	4	6	0.00	0.04	0.00	0.05
Q80UG5	Septin-9 OS=Mus musculus GN=Sept9 PE=1 SV=1 - [SEPT9_MOUSE]	48.54	5	21	21	132	-0.09	-0.08	-0.08	-0.18
H3BLR8	Diphosphoinositol polyphosphate phosphohydrolase 1 OS=Mus musculus GN=Nudt3 PE=2 SV=1 [H3BLR8_MOUSE]	31.97	5	3	3	12	-0.27	-0.27	-0.09	-0.09
E9Q409	Protein Prss32 OS=Mus musculus GN=Prss32 PE=2 SV=1 - [E9Q409_MOUSE]	8.98	2	3	3	13	0.19	0.08	0.08	-0.17
O09127	Ephrin type-A receptor 8 OS=Mus musculus GN=Epha8 PE=1 SV=2 - [EPHA8_MOUSE]	1.79	1	1	2	3	-0.47	-0.27	-0.18	0.03
F6QA74	DNA-(apurinic or apyrimidinic site) lyase (Fragment) OS=Mus musculus GN=Apex1 PE=2 SV=1 - [F6QA74_MOUSE]	41.87	3	10	10	62	0.24	0.50	0.20	0.45
Q61425	Hydroxyacyl-coenzyme A dehydrogenase, mitochondrial OS=Mus musculus GN=Hadh PE=1 SV=2 - [HCDH_MOUSE]	65.29	2	21	22	441	-0.56	-0.47	-0.58	-0.45
Q6P6J4	Alpha-ketoglutarate-dependent dioxygenase alkB homolog 2 OS=Mus musculus GN=Alkbh2 PE=1 SV=1 - [ALKB2_MOUSE]	8.37	1	1	1	1	0.50	-0.28	0.51	-0.27
Q8CAK1	Putative transferase CAF17 homolog, mitochondrial OS=Mus musculus GN=Iba57 PE=2 SV=1 - [CAF17_MOUSE]	12.29	1	4	4	12	0.01	-0.23	-0.06	-0.30
P06745	Glucose-6-phosphate isomerase OS=Mus musculus GN=Gpi PE=1 SV=4 - [G6PI_MOUSE]	51.79	2	27	27	548	-0.15	-0.08	-0.18	-0.10
P32507-2	Isoform Alpha of Poliovirus receptor-related protein 2 OS=Mus musculus GN=Pvrl2 - [PVRL2_MOUSE]	30.41	4	10	10	29	-0.12	-0.02	-0.16	0.06
Q9Z2U0	Proteasome subunit alpha type-7 OS=Mus musculus GN=Psma7 PE=1 SV=1 - [PSA7_MOUSE]	43.55	2	9	9	142	-0.19	-0.14	-0.27	-0.18
Q9D5V6	Synapse-associated protein 1 OS=Mus musculus GN=Syap1 PE=1 SV=1 - [SYAP1_MOUSE]	43.29	1	11	11	47	-0.42	-0.20	-0.32	-0.10
Q923D2	Flavin reductase (NADPH) OS=Mus musculus GN=Blvrb PE=2 SV=3 - [BLVRB_MOUSE]	46.12	3	7	7	129	-0.24	-0.27	-0.30	-0.22

Q5M8N4-2	Isoform 2 of Epimerase family protein SDR39U1 OS=Mus musculus GN=Sdr39u1 [D39U1_MOUSE]	12.63	2	4	4	5	-0.26	-0.66	-0.18	-0.57
Q60596	DNA repair protein XRCC1 OS=Mus musculus GN=Xrcc1 PE=1 SV=2 - [XRCC1_MOUSE]	11.41	1	5	5	8	-0.04	0.30	-0.06	0.22
Q9D9W1	Protein pitchfork OS=Mus musculus GN=Pifo PE=1 SV=1 - [PIFO_MOUSE]	10.14	2	1	2	3	0.29	-0.38	0.08	-0.59
Q52KR6	Acin1 protein OS=Mus musculus GN=Acin1 PE=2 SV=1 - [Q52KR6_MOUSE]	21.20	3	1	13	30	0.03	-0.06	-0.05	-0.15
Q9D8I3	Glyoxalase domain-containing protein 5 OS=Mus musculus GN=Glod5 PE=2 SV=1 [GLOD5_MOUSE]	31.08	1	3	3	10	0.07	-0.09	-0.03	-0.26
D3Z3A7	Low-density lipoprotein receptor-related protein 11 (Fragment) OS=Mus musculus GN=Lrp11 PE=2 SV=1 - [D3Z3A7_MOUSE]	5.57	5	1	2	2	0.14	-0.98	-0.07	-1.20
Q562E2	BTB/POZ domain-containing protein KCTD19 OS=Mus musculus GN=Kctd19 PE=2 SV=2 - [KCD19_MOUSE]	2.70	3	2	2	2	-1.00	-0.56	-0.39	0.05
P35700	Peroxiredoxin-1 OS=Mus musculus GN=Prdx1 PE=1 SV=1 [PRDX1_MOUSE]	67.34	4	14	16	866	-0.25	-0.23	-0.26	-0.25
G3X9U9	Fission 1 (Mitochondrial outer membrane) homolog (Yeast), isoform CRA_c OS=Mus musculus GN=Fis1 PE=4 SV=1 - [G3X9U9_MOUSE]	25.52	3	4	4	73	-0.22	-0.08	-0.14	-0.12
Q8QZY9	Splicing factor 3B subunit 4 OS=Mus musculus GN=Sf3b4 PE=2 SV=1 - [SF3B4_MOUSE]	11.56	1	3	3	46	-0.14	-0.14	-0.07	-0.22
Q6DFV7	Nuclear receptor coactivator 7 OS=Mus musculus GN=Ncoa7 PE=2 SV=2 - [NCOA7_MOUSE]	1.06	1	1	1	1	-0.10	-0.38	-0.02	-0.30
Q8CGF7-2	Isoform 2 of Transcription elongation regulator 1 OS=Mus musculus GN=Tcerg1 - [TCRG1_MOUSE]	17.05	6	21	21	63	-0.11	0.00	-0.05	-0.03
Q8K183	Pyridoxal kinase OS=Mus musculus GN=Pdxk PE=1 SV=1 - [PDXK_MOUSE]	33.97	2	9	9	129	-0.46	-0.41	-0.57	-0.51
D3YTU3	Bifunctional lysine-specific demethylase and histidyl-hydroxylase MINA OS=Mus musculus GN=Mina PE=2 SV=1 - [D3YTU3_MOUSE]	7.51	2	2	2	12	-0.12	-0.46	-0.06	-0.40

Q9D1E8	1-acyl-sn-glycerol-3-phosphate acyltransferase epsilon OS=Mus musculus GN=Agpat5 PE=2 SV=2 - [PLCE_MOUSE]	7.67	2	3	3	6	0.11	-0.19	-0.26	-0.56
Q5SQY2	Biorientation of chromosomes in cell division protein 1 OS=Mus musculus GN=Bod1 PE=2 SV=1 - [BOD1_MOUSE]	12.72	1	2	2	3	-0.23	-0.32	0.21	-0.19
Q5XJE5	RNA polymerase-associated protein LEO1 OS=Mus musculus GN=Leo1 PE=1 SV=2 - [LEO1_MOUSE]	6.15	1	2	2	5	0.14	0.10	0.16	0.12
E9QLB8	Uncharacterized aarF domain-containing protein kinase 4 OS=Mus musculus GN=Adck4 PE=4 SV=1 - [E9QLB8_MOUSE]	11.82	2	2	2	3	-0.29	-0.69	-0.39	-0.78
P29758	Ornithine aminotransferase, mitochondrial OS=Mus musculus GN=Oat PE=1 SV=1 - [OAT_MOUSE]	50.34	2	20	21	246	-0.74	-0.66	-0.80	-0.73
Q9Z0Z7	Krueppel-like factor 5 OS=Mus musculus GN=Klf5 PE=2 SV=2 - [KLF5_MOUSE]	4.71	1	2	2	4	-0.17	-0.07	-0.28	-0.18
D3Z2K2	28S ribosomal protein S14, mitochondrial OS=Mus musculus GN=Mrps14 PE=2 SV=1 - [D3Z2K2_MOUSE]	12.30	2	1	1	12	-0.10	-0.50	-0.13	-0.49
D3YXV8	Leukocyte receptor cluster member 8 homolog (Fragment) OS=Mus musculus GN=Leng8 PE=2 SV=1 - [D3YXV8_MOUSE]	2.74	3	2	2	2	-0.33	0.56	-0.64	0.25
Q9JJG0-4	Isoform 4 of Transforming acidic coiled-coil-containing protein 2 OS=Mus musculus GN=Tacc2 - [TACC2_MOUSE]	14.88	6	1	6	28	-0.32	-0.07	-0.32	-0.08
D3Z442	Uncharacterized protein OS=Mus musculus GN=Aamdc PE=2 SV=1 - [D3Z442_MOUSE]	51.08	9	7	7	20	-0.63	-0.46	-0.60	-0.61
Q9QYG0	Protein NDRG2 OS=Mus musculus GN=Ndrg2 PE=1 SV=1 - [NDRG2_MOUSE]	52.83	5	10	11	192	-0.05	-0.14	-0.19	-0.32
Q80WC7-2	Isoform 2 of Arf-GAP domain and FG repeat-containing protein 2 OS=Mus musculus GN=Agfg2 - [AGFG2_MOUSE]	15.22	4	3	4	4	0.02	0.05	-0.12	-0.09
A6H6E2	Multimerin-2 OS=Mus musculus GN=Mmrn2 PE=2 SV=1 - [MMRN2_MOUSE]	2.65	2	2	3	3	-0.79	-0.59	-0.82	-0.63
Q3UZZ6	Sulfotransferase 1 family member D1 OS=Mus musculus GN=Sult1d1 PE=1 SV=1 - [ST1D1_MOUSE]	50.51	1	14	16	163	-0.39	-0.37	-0.53	-0.51

Q00899	Transcriptional repressor protein YY1 OS=Mus musculus GN=Yy1 PE=1 SV=1 - [TYY1_MOUSE]	10.14	2	3	3	10	0.16	0.21	-0.05	0.10
Q11136	Xaa-Pro dipeptidase OS=Mus musculus GN=Pepd PE=2 SV=3 - [PEPD_MOUSE]	29.01	2	12	12	64	-0.36	-0.24	-0.36	-0.32
Q80TJ7-2	Isoform 2 of Histone lysine demethylase PHF8 OS=Mus musculus GN=Phf8 - [PHF8_MOUSE]	4.40	2	2	2	3	0.27	0.25	-0.19	-0.22
Q61165	Sodium/hydrogen exchanger 1 OS=Mus musculus GN=Slc9a1 PE=1 SV=1 - [SL9A1_MOUSE]	14.76	1	8	8	32	-0.68	-0.48	-0.50	-0.51
P16675	Lysosomal protective protein OS=Mus musculus GN=Ctsa PE=1 SV=1 - [PPGB_MOUSE]	25.53	4	8	9	29	-0.35	-0.49	-0.22	-0.47
Q6ZQ73	Cullin-associated NEDD8-dissociated protein 2 OS=Mus musculus GN=Cand2 PE=1 SV=2 - [CAND2_MOUSE]	2.02	1	1	3	25	0.27	0.12	0.27	0.11
Q9D142	Uridine diphosphate glucose pyrophosphatase OS=Mus musculus GN=Nudt14 PE=2 SV=1 - [NUD14_MOUSE]	16.22	1	3	3	6	-0.13	-0.48	-0.18	-0.53
Q3USN8	Protein Gm10654 OS=Mus musculus GN=Gm10654 PE=2 SV=1 - [Q3USN8_MOUSE]	4.88	1	1	1	1	0.01	0.21	-0.05	0.15
Q8BKL1	Protein SSXT OS=Mus musculus GN=Ss18 PE=2 SV=1 - [Q8BKL1_MOUSE]	8.72	3	2	2	3	-0.07	0.25	-0.26	0.06
Q8R0S2	IQ motif and SEC7 domain-containing protein 1 OS=Mus musculus GN=Iqsec1 PE=1 SV=2 - [IQEC1_MOUSE]	4.06	3	2	4	6	-0.34	-0.52	-0.20	-0.38
E9Q9I2	Protein Dlg5 OS=Mus musculus GN=Dlg5 PE=2 SV=1 - [E9Q9I2_MOUSE]	2.48	4	3	3	5	-0.58	-0.31	-0.53	-0.19
Q9DCH6	AN1-type zinc finger protein 6 OS=Mus musculus GN=Zfand6 PE=1 SV=1 - [ZFAN6_MOUSE]	8.07	1	2	2	3	-0.15	0.04	0.12	0.31
D3Z6H3	Dynactin 6, isoform CRA_b OS=Mus musculus GN=Dctn6 PE=4 SV=1 - [D3Z6H3_MOUSE]	26.49	5	4	4	33	-0.11	-0.10	-0.18	-0.09
P35505	Fumarylacetoacetase OS=Mus musculus GN=Fah PE=1 SV=2 - [FAAA_MOUSE]	10.74	2	3	3	16	-0.41	-0.42	-0.48	-0.22
E9QNR7	Probable ribonuclease ZC3H12D OS=Mus musculus GN=Zc3h12d PE=4 SV=1 - [E9QNR7_MOUSE]	3.56	2	1	1	1	0.17	0.29	-0.31	-0.20
Q99020	Heterogeneous nuclear ribonucleoprotein A/B OS=Mus musculus GN=Hnrnpab PE=1 SV=1 - [ROAA_MOUSE]	41.05	3	11	12	204	0.05	0.17	0.04	0.16

Q505F1-2	Isoform 2 of Nuclear receptor subfamily 2 group C member 1 OS=Mus musculus GN=Nr2c1 - [NR2C1_MOUSE]	5.47	2	1	1	3	-0.89	-0.76	-1.17	-1.04
Q9DBL7	Bifunctional coenzyme A synthase OS=Mus musculus GN=Coasy PE=1 SV=2 - [COASY_MOUSE]	12.97	1	5	5	14	-0.30	-0.25	-0.33	-0.34
Q9DD03	Ras-related protein Rab-13 OS=Mus musculus GN=Rab13 PE=1 SV=1 - [RAB13_MOUSE]	31.19	2	3	6	31	-0.10	0.08	-0.06	0.10
Q3U1J4	DNA damage-binding protein 1 OS=Mus musculus GN=Ddb1 PE=1 SV=2 - [DDB1_MOUSE]	33.86	1	33	33	197	-0.20	-0.25	-0.15	-0.16
B1B0C7	Basement membrane-specific heparan sulfate proteoglycan core protein OS=Mus musculus GN=Hspg2 PE=2 SV=1 - [B1B0C7_MOUSE]	37.71	3	102	102	1206	-0.63	-0.60	-0.63	-0.60
Q8R1I1	Cytochrome b-c1 complex subunit 9 OS=Mus musculus GN=Uqcr10 PE=1 SV=1 - [QCR9_MOUSE]	26.56	1	1	1	10	-0.12	-0.02	-0.18	-0.09
P47791-2	Isoform Cytoplasmic of Glutathione reductase, mitochondrial OS=Mus musculus GN=Gsr - [GSHR_MOUSE]	33.54	2	13	13	113	0.02	0.09	-0.01	-0.01
Q923D5	WW domain-binding protein 11 OS=Mus musculus GN=Wbp11 PE=1 SV=2 - [WBP11_MOUSE]	12.79	1	7	7	28	0.20	0.19	0.18	0.20
Q9R1P4	Proteasome subunit alpha type-1 OS=Mus musculus GN=Psma1 PE=1 SV=1 - [PSA1_MOUSE]	58.56	1	12	12	282	-0.19	-0.43	-0.18	-0.30
P06801	NADP-dependent malic enzyme OS=Mus musculus GN=Me1 PE=1 SV=2 - [MAOX_MOUSE]	33.39	1	17	17	95	-0.31	-0.31	-0.31	-0.27
E9QMK6	Uncharacterized protein OS=Mus musculus GN=Ptchd4 PE=4 SV=1 - [E9QMK6_MOUSE]	4.77	2	2	2	2	-1.04	-1.78	-0.40	-1.15
Q69ZS0-2	Isoform 2 of E3 ubiquitin-protein ligase PDZRN3 OS=Mus musculus GN=Pdzrn3 - [PZRN3_MOUSE]	0.99	3	1	1	1	-0.55	-0.56	-0.75	-0.77
H7BX26	Centrosomal protein of 170 kDa OS=Mus musculus GN=Cep170 PE=2 SV=1 - [H7BX26_MOUSE]	1.14	3	2	2	3	-0.45	-0.06	-0.43	-0.05
Q61239	Protein farnesyltransferase/geranylgeranyltransferase type-1 subunit alpha OS=Mus musculus GN=Fnta PE=1 SV=1 - [FNTA_MOUSE]	19.10	1	7	7	24	0.01	-0.12	0.00	-0.09

B2RY04	Dedicator of cytokinesis protein 5 OS=Mus musculus GN=Dock5 PE=1 SV=2 - [DOCK5_MOUSE]	0.70	1	1	1	2	0.05	0.14	-0.09	-0.01
P59325	Eukaryotic translation initiation factor 5 OS=Mus musculus GN=Eif5 PE=1 SV=1 - [IF5_MOUSE]	26.11	2	12	13	77	0.34	0.49	0.32	0.32
Q9R0U9	Protein Tex21 OS=Mus musculus GN=Tex21 PE=2 SV=1 - [Q9R0U9_MOUSE]	8.17	1	2	3	5	-0.44	-0.26	-0.28	-0.10
Q5DTM8	E3 ubiquitin-protein ligase BRE1A OS=Mus musculus GN=Rnf20 PE=1 SV=2 - [BRE1A_MOUSE]	5.34	5	5	5	5	0.33	0.51	0.33	0.51
Q8BU11	TOX high mobility group box family member 4 OS=Mus musculus GN=Tox4 PE=1 SV=3 - [TOX4_MOUSE]	8.24	8	4	4	13	0.23	0.27	0.04	0.08
Q3TDX8-5	Isoform 2 of Cytochrome b5 reductase 4 OS=Mus musculus GN=Cyb5r4 - [NB5R4_MOUSE]	5.45	3	2	2	6	-0.19	0.07	-0.14	-0.06
P84096	Rho-related GTP-binding protein RhoG OS=Mus musculus GN=Rhog PE=2 SV=1 - [RHOG_MOUSE]	50.26	6	7	8	79	0.01	-0.10	-0.04	-0.06
Q3US41-4	Isoform 4 of Epithelial splicing regulatory protein 1 OS=Mus musculus GN=Esrp1 - [ESRP1_MOUSE]	21.21	6	7	10	60	0.04	0.03	0.02	-0.01
P83741-4	Isoform 4 of Serine/threonine-protein kinase WNK1 OS=Mus musculus GN=Wnk1 - [WNK1_MOUSE]	8.36	12	9	11	21	-0.15	-0.53	0.02	-0.37
O35114	Lysosome membrane protein 2 OS=Mus musculus GN=Scarb2 PE=1 SV=3 - [SCRB2_MOUSE]	19.87	1	7	7	36	-0.01	-0.05	-0.11	-0.04
Q921M3	Splicing factor 3B subunit 3 OS=Mus musculus GN=Sf3b3 PE=2 SV=1 - [SF3B3_MOUSE]	25.39	2	26	26	132	-0.17	-0.25	-0.16	-0.19
Q9DAA4	Probable inactive serine protease 37 OS=Mus musculus GN=Prss37 PE=2 SV=1 - [PRS37_MOUSE]	4.64	1	1	1	1	-0.61	-0.38	-0.62	-0.39
P62270	40S ribosomal protein S18 OS=Mus musculus GN=Rps18 PE=1 SV=3 - [RS18_MOUSE]	38.82	4	8	8	91	-0.04	-0.07	-0.11	-0.09
Q9D7P6	Iron-sulfur cluster assembly enzyme ISCU, mitochondrial OS=Mus musculus GN=Iscu PE=1 SV=1 - [ISCU_MOUSE]	30.36	3	5	5	15	-0.43	-0.45	-0.34	-0.44
Q6DID3	Protein SCAF8 OS=Mus musculus GN=Scaf8 PE=1 SV=1 - [SCAF8_MOUSE]	4.50	1	4	4	5	0.05	0.12	-0.06	0.03

Q8K2B3	Succinate dehydrogenase [ubiquinone] flavoprotein subunit, mitochondrial OS=Mus musculus GN=Sdha PE=1 SV=1 - [DHSA_MOUSE]	56.33	1	29	29	712	-0.46	-0.46	-0.50	-0.49
P37889-2	Isoform 2 of Fibulin-2 OS=Mus musculus GN=Fbln2 - [FBLN2_MOUSE]	3.32	2	4	4	11	-0.35	-0.27	-0.30	-0.13
Q91V09	WD repeat-containing protein 13 OS=Mus musculus GN=Wdr13 PE=1 SV=1 - [WDR13_MOUSE]	17.94	2	6	6	9	-0.41	-0.26	-0.49	-0.26
Q8BJW6	Eukaryotic translation initiation factor 2A OS=Mus musculus GN=Eif2a PE=2 SV=2 - [EIF2A_MOUSE]	33.56	5	14	15	66	0.01	0.02	0.00	0.10
O35309	N-myc-interactor OS=Mus musculus GN=Nmi PE=2 SV=1 - [NMI_MOUSE]	30.89	3	7	7	17	-0.18	-0.03	-0.32	-0.11
P32921	Tryptophan--tRNA ligase, cytoplasmic OS=Mus musculus GN=Wars PE=1 SV=2 - [SYWC_MOUSE]	50.10	1	1	21	258	0.33	0.39	0.17	0.23
H3BLA9	Sentrin-specific protease 6 (Fragment) OS=Mus musculus GN=Senp6 PE=2 SV=1 [H3BLA9_MOUSE]	3.43	1	1	1	3	-2.24	-2.14	-2.34	-2.39
E9Q5L7	PHD finger protein 10 OS=Mus musculus GN=Phf10 PE=2 SV=2 [E9Q5L7_MOUSE]	2.56	4	1	1	1	-0.05	0.06	-0.03	0.07
Q8K0D5	Elongation factor G, mitochondrial OS=Mus musculus GN=Gfm1 PE=2 SV=1 - [EFGM_MOUSE]	22.50	2	13	13	46	-0.13	-0.12	-0.14	-0.22
F7BCD5	Tetratricopeptide repeat protein 19, mitochondrial (Fragment) OS=Mus musculus GN=Ttc19 PE=4 SV=1 - [F7BCD5_MOUSE]	11.88	3	2	2	2	-0.25	-0.03	-0.39	-0.17
E9Q3A7	Protein Cdh19 OS=Mus musculus GN=Cdh19 PE=3 SV=1 - [E9Q3A7_MOUSE]	4.42	1	3	3	8	-0.73	-0.80	-0.48	-0.55
Q61371	Intraflagellar transport protein 88 homolog OS=Mus musculus GN=Ift88 PE=1 SV=2 - [IFT88_MOUSE]	1.09	2	1	1	1	-0.74	-0.36	-0.51	-0.13
Q3TNU4	Putative uncharacterized protein ENSP00000382790 homolog OS=Mus musculus GN=Gm3414 PE=5 SV=1 - [YD021_MOUSE]	1.71	1	1	1	1	-0.06	-1.13	0.20	-0.88
Q91W50	Cold shock domain-containing protein E1 OS=Mus musculus GN=Csde1 PE=2 SV=1 - [CSDE1_MOUSE]	35.71	1	26	27	105	0.09	0.06	0.11	0.07

A2ADR8	Nuclear inhibitor of protein phosphatase 1 OS=Mus musculus GN=Ppp1r8 PE=2 SV=1 - [A2ADR8_MOUSE]	31.43	2	6	6	43	0.12	0.03	0.07	-0.16
Q8BFQ4	WD repeat-containing protein 82 OS=Mus musculus GN=Wdr82 PE=1 SV=1 - [WDR82_MOUSE]	14.38	1	4	5	7	-0.05	-0.21	0.11	-0.17
Q2TBE6	Phosphatidylinositol 4-kinase type 2-alpha OS=Mus musculus GN=Pi4k2a PE=1 SV=1 - [P4K2A_MOUSE]	13.78	1	3	4	9	-0.03	-0.35	0.28	-0.05
Q9D8B3	Charged multivesicular body protein 4b OS=Mus musculus GN=Chmp4b PE=2 SV=2 - [CHM4B_MOUSE]	52.23	1	11	12	124	0.12	0.19	0.21	0.19
Q9QZ06	Toll-interacting protein OS=Mus musculus GN=Tollip PE=1 SV=1 [TOLIP_MOUSE]	25.18	4	5	5	18	-0.14	-0.29	-0.21	-0.27
P11031	Activated RNA polymerase II transcriptional coactivator p15 OS=Mus musculus GN=Sub1 PE=1 SV=3 - [TCP4_MOUSE]	50.39	1	7	8	86	0.00	-0.07	-0.14	-0.11
J3QP68	Uncharacterized protein OS=Mus musculus GN=Gm4204 PE=3 SV=1 - [J3QP68_MOUSE]	26.84	3	6	7	78	0.20	0.23	0.28	0.39
Q3V3W4	Protein Zbtb2 OS=Mus musculus GN=Zbtb2 PE=2 SV=1 - [Q3V3W4_MOUSE]	1.17	1	1	1	2	-0.77	-0.63	-0.69	-0.55
Q62165	Dystroglycan OS=Mus musculus GN=Dag1 PE=1 SV=4 - [DAG1_MOUSE]	12.99	1	10	11	54	-0.41	-0.48	-0.59	-0.46
Q61206	Platelet-activating factor acetylhydrolase IB subunit beta OS=Mus musculus GN=Pafah1b2 PE=1 SV=2 - [PA1B2_MOUSE]	18.78	5	4	4	11	-0.03	0.39	-0.15	0.23
O08691	Arginase-2, mitochondrial OS=Mus musculus GN=Arg2 PE=1 SV=1 - [ARGI2_MOUSE]	7.34	1	2	2	6	-1.00	-0.89	-0.94	-0.83
P60521	Gamma-aminobutyric acid receptor-associated protein-like 2 OS=Mus musculus GN=Gabarapl2 PE=1 SV=1 - [GBRL2_MOUSE]	41.03	1	5	6	18	-0.27	-0.18	-0.39	-0.18
Q8BY05	Poly(A) polymerase gamma OS=Mus musculus GN=Papolg PE=2 SV=1 - [Q8BY05_MOUSE]	3.26	4	1	2	4	-0.36	-0.19	-0.18	-0.01
P62962	Profilin-1 OS=Mus musculus GN=Pfn1 PE=1 SV=2 - [PROF1_MOUSE]	73.57	3	12	12	773	-0.13	-0.09	-0.13	-0.06

P68510	14-3-3 protein eta OS=Mus musculus GN=Ywhah PE=1 SV=2 - [1433F_MOUSE]	57.72	1	10	15	281	-0.04	0.05	-0.08	-0.01
Q8R1Q9	Ribokinase OS=Mus musculus GN=Rbks PE=1 SV=1 - [RBSK_MOUSE]	10.22	1	3	3	8	-0.60	-0.37	-0.35	-0.21
P97364	Selenide, water dikinase 2 OS=Mus musculus GN=Sephs2 PE=1 SV=3 - [SPS2_MOUSE]	11.50	1	3	4	10	-0.53	-0.62	-0.37	-0.47
Q9D0W5	Peptidyl-prolyl cis-trans isomerase-like 1 OS=Mus musculus GN=Ppil1 PE=2 SV=1 - [PPIL1_MOUSE]	31.93	1	5	5	51	-0.34	-0.09	-0.34	0.04
Q9CPW4	Actin-related protein 2/3 complex subunit 5 OS=Mus musculus GN=Arpc5 PE=2 SV=3 - [ARPC5_MOUSE]	58.94	3	8	8	106	0.15	0.16	0.13	0.05
Q3URE1	Acyl-CoA synthetase family member 3, mitochondrial OS=Mus musculus GN=Acsf3 PE=2 SV=2 - [ACSF3_MOUSE]	16.30	2	9	9	24	-0.16	-0.30	-0.30	-0.30
E9Q1Q9	Ketohexokinase OS=Mus musculus GN=Khk PE=2 SV=1 - [E9Q1Q9_MOUSE]	6.12	3	2	2	3	-0.83	-0.86	-0.64	-0.81
Q62422	Osteoclast-stimulating factor 1 OS=Mus musculus GN=Ostf1 PE=1 SV=2 - [OSTF1_MOUSE]	33.49	1	5	5	79	-0.21	-0.15	-0.14	-0.01
D3YTQ9	40S ribosomal protein S15 OS=Mus musculus GN=Rps15 PE=2 SV=1 - [D3YTQ9_MOUSE]	55.93	3	5	5	49	0.29	0.48	0.00	0.30
Q80U49	Centrosomal protein of 170 kDa protein B OS=Mus musculus GN=Cep170b PE=1 SV=2 - [C170B_MOUSE]	3.88	2	5	5	8	-0.11	-0.04	0.00	0.20
Q99KG3-2	Isoform 2 of RNA-binding protein 10 OS=Mus musculus GN=Rbm10 - [RBM10_MOUSE]	11.02	3	8	9	20	0.13	-0.20	0.21	-0.04
P14206	40S ribosomal protein SA OS=Mus musculus GN=Rpsa PE=1 SV=4 - [RSSA_MOUSE]	51.53	2	11	11	350	-0.03	-0.11	-0.12	-0.20
Q8BYR2	Serine/threonine-protein kinase LATS1 OS=Mu musculus GN=Lats1 PE=1 SV=3 - [LATS1_MOUSE]	3.90	5	2	4	6	0.23	0.41	0.03	0.21
G5E8T6	Membrane associated guanylate kinase, WW and PDZ domain containing 3, isoform CRA_a OS=Mus musculus GN=Magi3 PE=4 SV=1 - [G5E8T6_MOUSE]	2.24	3	3	4	5	0.12	-0.18	-0.17	-0.61
Q8K2Y7	39S ribosomal protein L47, mitochondrial OS=Mus musculus GN=Mrpl47 PE=2 SV=2 - [RM47_MOUSE]	14.68	1	4	4	9	-0.52	-0.84	-0.10	-0.45

Q9DC63-3	Isoform 3 of F-box only protein 3 OS=Mus musculus GN=Fbxo3 - [FBX3_MOUSE]	13.17	4	3	3	11	-0.10	-0.11	-0.08	-0.04
Q6ZWZ2	Ubiquitin-conjugating enzyme E2 R2 OS=Mus musculus GN=Ube2r2 PE=2 SV=1 - [UB2R2_MOUSE]	10.08	1	2	2	7	0.08	-0.27	0.01	0.00
Q9D8X2	Coiled-coil domain-containing protein 124 OS=Mus musculus GN=Ccdc124 PE=2 SV=1 - [CC124_MOUSE]	33.18	1	7	7	30	0.00	-0.02	0.04	0.13
Q91WG2-1	Isoform 2 of Rab GTPase-binding effector protein 2 OS=Mus musculus GN=Rabep2 - [RABE2_MOUSE]	25.44	5	11	11	33	-0.01	-0.09	-0.06	-0.11
D3YZB3	Synaptojanin-2 OS=Mus musculus GN=Synj2 PE=2 SV=1 [D3YZB3_MOUSE]	5.12	16	3	3	4	-0.18	-0.13	-0.05	-0.17
Q9DAT5	Mitochondrial tRNA-specific 2-thiouridylase 1 OS=Mus musculus GN=Trmu PE=2 SV=1 - [MTU1_MOUSE]	6.00	1	2	2	5	-0.19	-0.28	-0.38	-0.48
P83917	Chromobox protein homolog 1 OS=Mus musculus GN=Cbx1 PE=1 SV=1 - [CBX1_MOUSE]	25.41	3	3	4	78	-0.25	-0.41	-0.27	-0.36
Q08ED5	Protein Ces2f OS=Mus musculus GN=Ces2f PE=2 SV=1 - [Q08ED5_MOUSE]	6.06	3	1	3	50	-0.34	-0.40	-0.48	-0.54
P47199	Quinone oxidoreductase OS=Mus musculus GN=Cryz PE=2 SV=1 - [QOR_MOUSE]	35.65	5	9	9	34	-0.35	-0.42	-0.36	-0.36
P48428	Tubulin-specific chaperone A OS=Mus musculus GN=Tbca PE=2 SV=3 - [TBCA_MOUSE]	58.33	1	9	9	61	-0.02	-0.04	-0.04	-0.12
Q9QUR7	Peptidyl-prolyl cis-trans isomerase NIMA-interacting 1 OS=Mus musculus GN=Pin1 PE=1 SV=1 - [PIN1_MOUSE]	50.91	1	6	6	36	-0.32	-0.28	-0.37	-0.53
P20108	Thioredoxin-dependent peroxide reductase, mitochondrial OS=Mus musculus GN=Prdx3 PE=1 SV=1 - [PRDX3_MOUSE]	43.97	1	10	10	255	-0.42	-0.30	-0.49	-0.43
Q921G9	Tight junction protein 3 OS=Mus musculus GN=Tjp3 PE=2 SV=1 - [Q921G9_MOUSE]	17.37	2	10	11	34	0.15	0.03	-0.04	-0.03
Q9WTK5	Nuclear factor NF-kappa-B p100 subunit OS=Mus musculus GN=Nfkb2 PE=1 SV=1 - [NFKB2_MOUSE]	9.57	1	6	7	12	0.05	-0.52	0.19	-0.76
H7BX88	Carnitine O-acetyltransferase OS=Mus musculus GN=Crat PE=2 SV=1 - [H7BX88_MOUSE]	22.64	5	13	13	54	-0.33	-0.26	-0.35	-0.29

P02463	Collagen alpha-1(IV) chain OS=Mus musculus GN=Col4a1 PE=2 SV=4 - [CO4A1_MOUSE]	10.13	3	9	9	24	-0.73	-0.84	-0.89	-1.02
Q9JJJ7-4	Isoform 4 of Protein-cysteine N-palmitoyltransferase porcupine OS=Mus musculus GN=Porcn - [PORCN_MOUSE]	8.67	4	2	2	6	-0.43	-0.60	-0.74	-0.95
D6RG99	Mitochondrial import inner membrane translocase subunit Tim10 B OS=Mus musculus GN=Timm10b PE=2 SV=1 - [D6RG99_MOUSE]	27.94	2	1	1	3	0.06	-0.11	-0.15	-0.31
Q6PDD0	UDP-glucuronosyltransferase 2A2 OS=Mus musculus GN=Ugt2a2 PE=2 SV=1 - [UD2A2_MOUSE]	6.25	3	1	2	4	-0.45	-1.78	-0.65	-1.98
P42125	Enoyl-CoA delta isomerase 1, mitochondrial OS=Mus musculus GN=Eci1 PE=2 SV=2 - [ECI1_MOUSE]	31.14	1	9	9	120	-0.32	-0.24	-0.39	-0.32
Q9DCC1	RNA guanylyltransferase and 5'-phosphatase, isoform CRA_a OS=Mus musculus GN=Rngtt PE=2 SV=1 - [Q9DCC1_MOUSE]	5.51	2	3	3	3	0.51	-0.01	0.39	-0.14
P40240	CD9 antigen OS=Mus musculus GN=Cd9 PE=1 SV=2 - [CD9_MOUSE]	5.31	1	2	2	11	-0.16	-0.03	-0.21	-0.07
Q8BP40	Lysophosphatidic acid phosphatase type 6 OS=Mus musculus GN=Acp6 PE=2 SV=1 - [PPA6_MOUSE]	44.26	1	13	14	97	-0.62	-0.54	-0.58	-0.46
Q80WK2	Organic solute transporter subunit beta OS=Mus musculus GN=Slc51b PE=1 SV=1 - [OSTB_MOUSE]	14.06	1	1	1	2	-1.47	-0.93	-1.40	-0.85
P62311	U6 snRNA-associated Sm-like protein LSm3 OS=Mus musculus GN=Lsm3 PE=3 SV=2 [LSM3_MOUSE]	17.65	1	2	2	18	-0.24	-0.20	-0.25	-0.18
Q91VW3	SH3 domain-binding glutamic acid-rich-like protein 3 OS=Mus musculus GN=Sh3bgrl3 PE=1 SV=1 - [SH3L3_MOUSE]	39.78	2	5	5	187	-0.24	-0.24	-0.14	-0.24
P08030	Adenine phosphoribosyltransferase OS=Mus musculus GN=Aprt PE=2 SV=2 - [APT_MOUSE]	21.67	1	4	4	28	0.03	-0.07	0.05	0.03
O08966	Solute carrier family 22 member 1 OS=Mus musculus GN=Slc22a1 PE=2 SV=2 - [S22A1_MOUSE]	5.04	1	2	2	6	-0.65	-0.68	-0.30	-0.34
P15327	Bisphosphoglycerate mutase OS=Mus musculus GN=Bpgm PE=2 SV=2 - [PMGE_MOUSE]	35.91	2	5	5	20	-0.08	-0.70	-0.04	-0.63

Q8K382	DENN domain-containing protein 1A OS=Mus musculus GN=Dennd1a PE=1 SV=2 - [DEN1A_MOUSE]	4.43	2	4	4	7	0.01	0.10	0.06	0.32
Q8BND5-2	Isoform 2 of Sulfhydryl oxidase 1 OS=Mus musculus GN=Qsox1 - [QSOX1_MOUSE]	12.56	4	7	7	27	-0.22	-0.42	-0.11	-0.27
Q4VAA2-2	Isoform 2 of Protein CDV3 OS=Mus musculus GN=Cdv3 - [CDV3_MOUSE]	53.81	3	8	8	91	0.02	0.14	-0.10	0.01
P97823	Acyl-protein thioesterase 1 OS=Mus musculus GN=Lypla1 PE=1 SV=1 - [LYPA1_MOUSE]	31.30	7	5	5	72	-0.04	-0.25	-0.02	-0.15
P63242	Eukaryotic translation initiation factor 5A-1 OS=Mus musculus GN=Eif5a PE=1 SV=2 - [IF5A1_MOUSE]	53.25	3	10	10	459	-0.06	0.06	-0.08	0.03
Q3UMU9-4	Isoform 4 of Hepatoma-derived growth factor-related protein 2 OS=Mus musculus GN=Hdgfrp2 - [HDGR2_MOUSE]	9.59	4	5	7	23	0.15	0.27	0.33	0.30
O55057	Retinal rod rhodopsin-sensitive cGMP 3',5'-cyclic phosphodiesterase subunit delta OS=Mus musculus GN=Pde6d PE=1 SV=1 - [PDE6D_MOUSE]	20.67	2	2	2	6	-0.67	-0.81	-0.40	-0.53
O88207	Collagen alpha-1(V) chain OS=Mus musculus GN=Col5a1 PE=2 SV=2 - [CO5A1_MOUSE]	8.11	4	8	8	19	-0.33	-0.21	-0.34	-0.22
Q9JI75	Ribosyldihydronicotinamide dehydrogenase [quinone] OS=Mus musculus GN=Nqo2 PE=2 SV=3 - [NQO2_MOUSE]	41.13	1	9	9	78	-0.24	-0.15	-0.21	-0.23
F7BGR7	RNA-binding protein 4 OS=Mus musculus GN=Rbm4 PE=4 SV=1 [F7BGR7_MOUSE]	26.21	10	6	7	27	-0.10	-0.29	-0.03	-0.29
D3Z5F0	Cdc42 effector protein 5 (Fragment) OS=Mus musculus GN=Cdc42ep5 PE=2 SV=1 - [D3Z5F0_MOUSE]	32.91	2	2	2	4	-0.47	-0.60	-0.34	-0.48
Q69ZT1-2	Isoform 2 of Fanconi-associated nuclease 1 OS=Mus musculus GN=Fan1 - [FAN1_MOUSE]	1.48	2	2	2	3	-0.23	-0.13	-0.41	-0.31
P13516	Acyl-CoA desaturase 1 OS=Mus musculus GN=Scd1 PE=1 SV=2 - [ACOD1_MOUSE]	1.97	3	1	1	1	-0.27	-0.13	-0.22	-0.09
P26369	Splicing factor U2AF 65 kDa subunit OS=Mus musculus GN=U2af2 PE=1 SV=3 [U2AF2_MOUSE]	37.05	2	11	11	91	0.14	0.18	0.10	0.10
D3YWR2	B-cell linker protein OS=Mus musculus GN=Blnk PE=2 SV=1 - [D3YWR2_MOUSE]	1.54	2	1	1	1	0.43	0.52	0.45	0.54

Q9WV69-2	Isoform 2 of Dematin OS=Mus musculus GN=Dmtn - [DEMA_MOUSE]	10.44	4	3	4	6	-0.57	-0.45	-0.48	-0.43
Q922B9-3	Isoform 3 of Sperm-specific antigen 2 homolog OS=Mus musculus GN=Ssfa2 - [SSFA2_MOUSE]	6.81	5	5	6	8	-0.26	-0.26	-0.41	-0.43
Q8K1A6	Coiled-coil and C2 domain-containing protein 1A OS=Mus musculus GN=Cc2d1a PE=1 SV=2 - [C2D1A_MOUSE]	13.47	2	9	9	24	-0.06	0.06	-0.13	-0.06
Q61205	Platelet-activating factor acetylhydrolase IB subunit gamma OS=Mus musculus GN=Pafah1b3 PE=1 SV=1 - [PA1B3_MOUSE]	34.91	4	7	7	43	0.15	0.00	-0.01	0.07
G3X969	Endonuclease 8-like 2 OS=Mus musculus GN=Neil2 PE=4 SV=1 - [G3X969_MOUSE]	2.74	2	1	1	1	-0.12	-0.20	0.04	-0.04
Q8CGM2	Retinitis pigmentosa 1-like 1 protein OS=Mus musculus GN=Rp1l1 PE=1 SV=1 - [RP1L1_MOUSE]	2.04	1	2	2	4	-0.33	-0.19	-0.39	-0.25
Q8VBX4	C-type lectin domain family 4 member K OS=Mus musculus GN=Cd207 PE=2 SV=1 - [CLC4K_MOUSE]	3.93	1	1	1	2	-0.32	-0.09	-0.17	0.06
O08797	Protein Serpinb9 OS=Mus musculus GN=Serpinb9 PE=2 SV=1 - [O08797_MOUSE]	41.98	6	12	14	69	-0.13	-0.16	-0.25	-0.27
E9Q557	Desmoplakin OS=Mus musculus GN=Dsp PE=2 SV=1 - [DESP_MOUSE]	11.31	2	28	31	101	0.10	-0.05	0.16	0.06
Q8R550-5	Isoform 5 of SH3 domain-containing kinase-binding protein 1 OS=Mus musculus GN=Sh3kbp1 - [SH3K1_MOUSE]	20.14	9	4	7	14	-0.67	-0.14	-0.91	-0.37
Q8R5L3-2	Isoform 2 of Vam6/Vps39-like protein OS=Mus musculus GN=Vps39 - [VPS39_MOUSE]	4.57	2	2	2	2	0.13	0.40	-0.28	-0.01
Q61576	Peptidyl-prolyl cis-trans isomerase FKBP10 OS=Mus musculus GN=Fkbp10 PE=1 SV=2 - [FKB10_MOUSE]	16.01	2	11	11	41	0.03	0.03	0.07	0.05
E9PWI8	Protein C-ets-1 OS=Mus musculus GN=Ets1 PE=2 SV=1 - [E9PWI8_MOUSE]	9.92	2	2	2	3	-0.17	-0.16	-0.60	-0.60
A2AV19	Exosome complex component RRP4 (Fragment) OS=Mus musculus GN=Exosc2 PE=2 SV=1 - [A2AV19_MOUSE]	10.53	2	2	2	5	-0.28	-0.51	-0.28	-0.28
Q3UFK1	Coiled-coil domain containing 28A OS=Mus musculus GN=Ccdc28a PE=2 SV=1 - [Q3UFK1_MOUSE]	9.24	2	1	1	6	-0.08	-0.20	-0.01	-0.19

Q9WVG6	Histone-arginine methyltransferase CARM1 OS=Mus musculus GN=Carm1 PE=1 SV=2 - [CARM1_MOUSE]	14.31	4	4	4	16	-0.27	-0.03	-0.39	-0.16
D3Z0Y2	Peroxiredoxin-6 OS=Mus musculus GN=Prdx6 PE=2 SV=1 [D3Z0Y2_MOUSE]	74.50	3	16	16	791	-0.15	-0.03	-0.15	-0.08
Q9D8S9	BolA-like protein 1 OS=Mus musculus GN=Bola1 PE=1 SV=1 [BOLA1_MOUSE]	62.04	1	7	7	29	-0.31	-0.26	-0.30	-0.32
A2AM05-4	Isoform 4 of Centlein OS=Mus musculus GN=Cntln - [CNTLN_MOUSE]	2.87	6	2	4	9	-0.56	-0.79	-0.46	-0.70
Q9R100	Cadherin-17 OS=Mus musculus GN=Cdh17 PE=1 SV=1 - [CAD17_MOUSE]	48.00	2	29	29	653	-0.01	0.01	-0.05	-0.02
Q9JIW9	Ras-related protein Ral-B OS=Mus musculus GN=Ralb PE=2 SV=1 - [RALB_MOUSE]	39.32	2	4	7	45	0.06	-0.15	-0.06	-0.17
Q61043-2	Isoform 2 of Ninein OS=Mus musculus GN=Nin - [NIN_MOUSE]	1.33	3	1	3	3	-0.11	0.65	-0.41	0.36
P63101	14-3-3 protein zeta/delta OS=Mus musculus GN=Ywhaz PE=1 SV=1 - [1433Z_MOUSE]	66.12	4	11	18	696	-0.09	0.04	-0.15	0.00
P38647	Stress-70 protein, mitochondrial OS=Mus musculus GN=Hspa9 PE=1 SV=3 - [GRP75_MOUSE]	54.64	1	37	38	903	-0.12	-0.04	-0.19	-0.09
Q8BLR5	PH and SEC7 domain-containing protein 4 OS=Mus musculus GN=Psd4 PE=2 SV=1 - [PSD4_MOUSE]	4.18	1	1	3	3	-0.92	-0.71	-0.79	-0.58
Q6DFW5	ATPase, Class VI, type 11B OS=Mus musculus GN=Atp11b PE=2 SV=1 - [Q6DFW5_MOUSE]	1.96	4	2	3	7	0.22	0.34	0.05	0.17
Q8BL97-3	Isoform 3 of Serine/arginine-rich splicing factor 7 OS=Mus musculus GN=Srsf7 - [SRSF7_MOUSE]	31.21	4	4	5	59	-0.32	-0.17	-0.34	-0.20
Q9DB27	Malignant T-cell-amplified sequence 1 OS=Mus musculus GN=Mcts1 PE=2 SV=1 [MCTS1_MOUSE]	36.46	3	5	5	14	0.04	0.01	-0.05	-0.08
Q6KCD5-2	Isoform 2 of Nipped-B-like protein OS=Mus musculus GN=Nipbl - [NIPBL_MOUSE]	3.31	3	6	6	8	0.23	0.20	0.32	0.16
Q9JJF3	Bifunctional lysine-specific demethylase and histidyl-hydroxylase NO66 OS=Mus musculus GN=No66 PE=1 SV=2 - [NO66_MOUSE]	2.16	1	2	2	3	-0.62	-0.63	-0.40	-0.41
Q9DBE0	Cysteine sulfinic acid decarboxylase OS=Mus musculus GN=Csad PE=2 SV=1 - [CSAD_MOUSE]	15.01	1	8	8	25	-0.55	-0.29	-0.68	-0.49

P45952	Medium-chain specific acyl-CoA dehydrogenase, mitochondrial OS=Mus musculus GN=Acadm PE=1 SV=1 - [ACADM_MOUSE]	42.99	2	15	16	173	-0.58	-0.57	-0.53	-0.58
O88286-2	Isoform S of Protein Wiz OS=Mus musculus GN=Wiz - [WIZ_MOUSE]	4.81	5	3	3	7	0.03	0.16	-0.08	-0.03
P35282	Ras-related protein Rab-21 OS=Mus musculus GN=Rab21 PE=1 SV=4 - [RAB21_MOUSE]	34.23	4	6	6	25	0.06	0.02	-0.08	0.17
P97450	ATP synthase-coupling factor 6, mitochondrial OS=Mus musculus GN=Atp5j PE=1 SV=1 - [ATP5J_MOUSE]	50.93	2	7	7	150	-0.44	-0.40	-0.49	-0.40
Q8VI40	Tripartite motif-containing protein 60 OS=Mus musculus GN=Trim60 PE=2 SV=1 - [TRI60_MOUSE]	1.50	1	1	1	1	-0.11	-0.11	-0.31	-0.32
Q6P1B1	Xaa-Pro aminopeptidase 1 OS=Mus musculus GN=Xpnpep1 PE=2 SV=1 - [XPP1_MOUSE]	40.13	1	20	20	142	-0.23	-0.25	-0.16	-0.25
Q8N7N5-2	Isoform 2 of DDB1- and CUL4-associated factor 8 OS=Mus musculus GN=Dcaf8 - [DCAF8_MOUSE]	8.27	2	3	3	4	-0.01	0.00	0.04	0.04
F6VZ99	Non-structural maintenance of chromosomes element 1 homolog (Fragment) OS=Mus musculus GN=Nsmce1 PE=4 SV=1 - [F6VZ99_MOUSE]	15.50	2	1	2	2	-0.35	-0.59	0.20	-0.05
Q99KP6	Pre-mRNA-processing factor 19 OS=Mus musculus GN=Prpf19 PE=2 SV=1 - [PRP19_MOUSE]	32.14	4	12	12	44	0.04	-0.16	0.06	0.02
Q9Z2D6	Methyl-CpG-binding protein 2 OS=Mus musculus GN=Mecp2 PE=1 SV=1 - [MECP2_MOUSE]	28.93	4	10	10	45	-0.04	-0.09	-0.05	-0.12
Q9ERE7	LDLR chaperone MESD OS=Mus musculus GN=Mesdc2 PE=1 SV=1 - [MESD_MOUSE]	23.21	2	5	5	16	-0.03	-0.08	0.03	-0.08
Q9D8Z2	TP53-regulated inhibitor of apoptosis 1 OS=Mus musculus GN=Triap1 PE=2 SV=1 - [TRIA1_MOUSE]	55.26	1	4	4	11	0.09	-0.19	0.05	-0.24
Q3V125	Coiled-coil domain-containing protein 110 OS=Mus musculus GN=Ccdc110 PE=2 SV=1 - [CC110_MOUSE]	3.07	1	2	2	2	0.11	0.02	0.04	-0.05
Q8K394	Inactive phospholipase C-like protein 2 OS=Mus musculus GN=Plcl2 PE=1 SV=2 - [PLCL2_MOUSE]	5.50	1	3	4	5	-0.10	0.05	-0.25	-0.10

Q61301-2	Isoform 2 of Catenin alpha-2 OS=Mus musculus GN=Ctnna2 - [CTNA2_MOUSE]	17.79	5	5	15	112	-0.38	-0.60	-0.24	-0.38
Q9CRC3	UPF0235 protein C15orf40 homolog OS=Mus musculus PE=2 SV=1 - [CO040_MOUSE]	10.32	1	1	1	2	0.01	-0.75	-0.25	-1.01
Q9JK81	UPF0160 protein MYG1, mitochondrial OS=Mus musculus GN=Myg1 PE=2 SV=1 [MYG1_MOUSE]	30.26	3	9	9	49	-0.11	-0.15	-0.10	-0.06
P70444	BH3-interacting domain death agonist OS=Mus musculus GN=Bid PE=1 SV=2 - [BID_MOUSE]	12.82	2	2	2	4	-0.10	-0.22	-0.04	-0.16
P41731	CD63 antigen OS=Mus musculus GN=Cd63 PE=1 SV=2 - [CD63_MOUSE]	4.62	1	1	1	19	0.23	-0.26	0.33	-0.26
Q8CIG8	Protein arginine N-methyltransferase 5 OS=Mus musculus GN=Prmt5 PE=1 SV=3 [ANM5_MOUSE]	13.81	2	9	9	23	-0.01	0.14	0.04	0.25
Q8BVQ5	Protein phosphatase methylesterase 1 OS=Mus musculus GN=Ppme1 PE=1 SV=5 - [PPME1_MOUSE]	16.32	1	7	7	15	-0.03	0.04	-0.14	0.00
Q921H9	Sel1 repeat-containing protein 1 OS=Mus musculus GN=Selrc1 PE=1 SV=1 - [SELR1_MOUSE]	14.72	1	4	4	19	-0.10	0.00	-0.19	0.03
Q9CQ65	S-methyl-5'-thioadenosine phosphorylase OS=Mus musculus GN=Mtap PE=2 SV=1 - [MTAP_MOUSE]	23.32	1	4	4	79	-0.14	-0.22	-0.10	-0.18
Q6A4L0	Solute carrier family 22 member 13 OS=Mus musculus GN=Slc22a13 PE=2 SV=3 - [S22AD_MOUSE]	6.35	1	1	1	2	0.53	0.42	0.41	0.29
Q8BW70	Ubiquitin carboxyl-terminal hydrolase 38 OS=Mus musculus GN=Usp38 PE=2 SV=2 - [UBP38_MOUSE]	0.77	1	1	1	1	-0.21	-0.44	-0.24	-0.48
D0QMC3	Myeloid cell nuclear differentiation antigen-like protein OS=Mus musculus GN=Mndal PE=2 SV=1 - [MNDAL_MOUSE]	11.34	4	3	5	8	0.00	0.10	-0.14	0.14
Q61171	Peroxiredoxin-2 OS=Mus musculus GN=Prdx2 PE=1 SV=3 [PRDX2_MOUSE]	59.09	2	10	10	250	-0.25	-0.22	-0.29	-0.30
Q6P9R2	Serine/threonine-protein kinase OSR1 OS=Mus musculus GN=Oxsr1 PE=1 SV=1 - [OXSR1_MOUSE]	19.54	2	8	9	62	-0.12	-0.14	-0.15	-0.07
Q8C845	EF-hand domain-containing protein D2 OS=Mus musculus GN=Efhd2 PE=2 SV=1 [Q8C845_MOUSE]	46.67	3	13	13	140	-0.29	-0.22	-0.23	-0.14

Q8BJK1	BTB/POZ domain-containing protein KCTD7 OS=Mus musculus GN=Kctd7 PE=1 SV=1 - [KCTD7_MOUSE]	2.08	1	1	1	2	-0.28	-0.11	-0.11	0.05
O88587-2	Isoform Soluble of Catechol O-methyltransferase OS=Mus musculus GN=Comt - [COMT_MOUSE]	40.54	3	7	7	58	-0.24	-0.06	-0.30	-0.16
P20152	Vimentin OS=Mus musculus GN=Vim PE=1 SV=3 - [VIME_MOUSE]	67.60	2	29	37	2205	-0.58	-0.52	-0.61	-0.54
Q9CQR4	Acyl-coenzyme A thioesterase 13 OS=Mus musculus GN=Acot13 PE=1 SV=1 - [ACO13_MOUSE]	42.14	1	5	5	21	-0.73	-0.35	-0.91	-0.41
Q6PCS8	Protein Zbtb40 OS=Mus musculus GN=Zbtb40 PE=2 SV=1 - [Q6PCS8_MOUSE]	4.05	1	2	3	9	-0.10	-0.03	-0.28	-0.21
A2AMW0	Capping protein (Actin filament) muscle Z-line, beta, isoform CRA_a OS=Mus musculus GN=Capzb PE=4 SV=1 - [A2AMW0_MOUSE]	60.38	7	21	22	415	-0.26	-0.16	-0.29	-0.24
E9PVX6	Protein Mki67 OS=Mus musculus GN=Mki67 PE=2 SV=1 - [E9PVX6_MOUSE]	5.57	1	14	14	31	0.32	0.05	0.12	0.05
O08715-2	Isoform 1 of A-kinase anchor protein 1, mitochondrial OS=Mus musculus GN=Akap1 - [AKAP1_MOUSE]	32.54	6	9	9	37	-0.04	0.20	-0.13	0.09
Q99N93	39S ribosomal protein L16, mitochondrial OS=Mus musculus GN=Mrpl16 PE=2 SV=1 - [RM16_MOUSE]	18.73	1	3	3	19	0.05	-0.14	-0.15	-0.26
Q61418	H(+)/Cl(-) exchange transporter 4 OS=Mus musculus GN=Clcn4 PE=2 SV=2 - [CLCN4_MOUSE]	0.80	1	1	1	5	0.95	0.21	0.67	-0.07
Q64378	Peptidyl-prolyl cis-trans isomerase FKBP5 OS=Mus musculus GN=Fkbp5 PE=1 SV=1 - [FKBP5_MOUSE]	28.07	2	12	12	36	-0.43	-0.31	-0.50	-0.37
Q60716-2	Isoform IIa of Prolyl 4-hydroxylase subunit alpha-2 OS=Mus musculus GN=P4ha2 - [P4HA2_MOUSE]	2.99	3	1	2	2	-0.52	-0.98	-0.66	-1.11
Q9JMG1	Endothelial differentiation-related factor 1 OS=Mus musculus GN=Edf1 PE=1 SV=1 - [EDF1_MOUSE]	27.03	1	4	4	19	0.26	0.00	0.21	0.04
Q62093	Serine/arginine-rich splicing factor 2 OS=Mus musculus GN=Srsf2 PE=1 SV=4 - [SRSF2_MOUSE]	14.48	1	3	3	39	-0.31	-0.31	-0.24	-0.18

Q8BH60-2	Isoform 2 of Golgi-associated PDZ and coiled-coil motif-containing protein OS=Mus musculus GN=Gopc - [GOPC_MOUSE]	22.86	3	8	9	24	-0.17	-0.36	-0.31	-0.42
Q9D8Y7	Tumor necrosis factor alpha-induced protein 8-like protein 2 OS=Mus musculus GN=Tnfaip8l2 PE=1 SV=1 - [TP8L2_MOUSE]	22.83	1	3	3	9	0.05	0.72	0.02	0.87
Q78JW9	Ubiquitin domain-containing protein UBFD1 OS=Mus musculus GN=Ubfd1 PE=1 SV=2 - [UBFD1_MOUSE]	12.23	1	4	4	9	0.01	0.30	-0.15	0.14
P85094	Isochorismatase domain containing protein 2A, mitochondrial OS=Mus musculus GN=Isoc2a PE=2 SV=1 - [ISC2A_MOUSE]	27.67	1	3	3	38	-0.33	-0.61	-0.38	-0.65
O35943	Frataxin, mitochondrial OS=Mus musculus GN=Fxn PE=1 SV=1 - [FRDA_MOUSE]	16.91	1	3	3	8	-0.16	0.09	-0.30	-0.16
E9Q6B2	Coiled-coil domain-containing protein 85C OS=Mus musculus GN=Ccdc85c PE=2 SV=1 - [CC85C_MOUSE]	17.86	1	5	5	15	-0.12	-0.18	-0.31	-0.46
O88569	Heterogeneous nuclear ribonucleoproteins A2/B1 OS=Mus musculus GN=Hnrnpa2b1 PE=1 SV=2 - [ROA2_MOUSE]	74.50	3	22	26	920	-0.27	-0.22	-0.30	-0.23
Q9JHS9	Spliceosome-associated protein CWC15 homolog OS=Mus musculus GN=Cwc15 PE=1 SV=1 - [CWC15_MOUSE]	6.99	1	2	2	5	-0.25	-0.28	-0.19	-0.23
F6VS49	Protein Apol8 OS=Mus musculus GN=Apol8 PE=4 SV=2 - [F6VS49_MOUSE]	3.26	2	1	1	1	-0.07	-0.30	-0.30	-0.53
O70591	Prefoldin subunit 2 OS=Mus musculus GN=Pfdn2 PE=2 SV=2 [PFD2_MOUSE]	38.31	2	4	4	15	-0.16	-0.09	-0.13	-0.25
P05201	Aspartate aminotransferase, cytoplasmic OS=Mus musculus GN=Got1 PE=1 SV=3 - [AATC_MOUSE]	51.57	2	19	19	145	-0.34	-0.40	-0.30	-0.36
Q8R0A0	General transcription factor IIF subunit 2 OS=Mus musculus GN=Gtf2f2 PE=1 SV=1 - [T2FB_MOUSE]	18.47	1	4	5	10	-0.03	0.02	-0.06	0.10
Q9JK53	Prolargin OS=Mus musculus GN=Prelp PE=2 SV=2 - [PRELP_MOUSE]	30.95	1	12	12	197	-0.57	-0.54	-0.52	-0.57

Q3ZT31	Sorting nexin-25 OS=Mus musculus GN=Snx25 PE=2 SV=3 - [SNX25_MOUSE]	1.43	2	1	1	1	0.01	0.32	0.09	0.40
Q8BMD5-3	Isoform 3 of Cytidine and dCMP deaminase domain-containing protein 1 OS=Mus musculus GN=Cdadc1 - [CDAC1_MOUSE]	7.04	6	1	2	6	-0.78	-0.68	-0.65	-0.55
Q8VEA4	Mitochondrial intermembrane space import and assembly protein 40 OS=Mus musculus GN=Chchd4 PE=1 SV=1 - [MIA40_MOUSE]	12.23	1	2	2	14	-0.21	-0.24	-0.34	-0.38
Q6A028	Switch-associated protein 70 OS=Mus musculus GN=Swap70 PE=1 SV=2 - [SWP70_MOUSE]	17.95	1	10	10	27	0.04	-0.10	0.07	-0.23
Q80YR4-2	Isoform 2 of Zinc finger protein 598 OS=Mus musculus GN=Znf598 - [ZN598_MOUSE]	3.75	3	3	3	5	-0.38	-0.30	-0.41	-0.28
P08228	Superoxide dismutase [Cu-Zn] OS=Mus musculus GN=Sod1 PE=1 SV=2 - [SODC_MOUSE]	79.87	1	9	9	262	-0.31	-0.18	-0.36	-0.24
P27546-2	Isoform 2 of Microtubule-associated protein 4 OS=Mus musculus GN=Map4 - [MAP4_MOUSE]	48.75	7	24	39	349	-0.03	0.13	-0.09	0.03
H3BKT0	Homeobox protein cut-like 1 OS=Mus musculus GN=Cux1 PE=2 SV=1 - [H3BKT0_MOUSE]	19.67	7	1	10	29	-0.20	-0.36	-0.31	-0.45
Q5SXY1-3	Isoform 4 of Cytospin-B OS=Mus musculus GN=Specc1 - [CYTSB_MOUSE]	8.61	3	9	9	20	-0.38	-0.49	-0.20	-0.31
Q5Q9H7	DNA polymerase kappa OS=Mus musculus GN=Polk PE=2 SV=1 - [Q5Q9H7_MOUSE]	5.42	3	2	3	16	-0.04	-0.54	-0.14	-0.42
Q9JIH2	Nuclear pore complex protein Nup50 OS=Mus musculus GN=Nup50 PE=1 SV=3 - [NUP50_MOUSE]	22.53	1	8	8	26	0.20	0.41	-0.02	-0.06
P08249	Malate dehydrogenase, mitochondrial OS=Mus musculus GN=Mdh2 PE=1 SV=3 - [MDHM_MOUSE]	55.92	1	16	16	583	-0.16	-0.16	-0.29	-0.25
Q04207-2	Isoform p65 delta of Transcription factor p65 OS=Mus musculus GN=Rela - [TF65_MOUSE]	6.49	2	3	3	12	-0.06	-0.03	0.02	-0.02
Q8BIV1	Protein Zfp719 OS=Mus musculus GN=Zfp719 PE=2 SV=1 - [Q8BIV1_MOUSE]	1.22	1	1	1	1	-0.13	-0.79	0.81	0.15
P29452	Caspase-1 OS=Mus musculus GN=Casp1 PE=1 SV=1 - [CASP1_MOUSE]	37.56	1	14	14	66	0.24	0.35	0.23	0.25

Q9CR51	V-type proton ATPase subunit G 1 OS=Mus musculus GN=Atp6v1g1 PE=2 SV=3 - [VATG1_MOUSE]	23.73	1	2	2	3	0.12	-0.13	0.08	-0.17
O35326	Serine/arginine-rich splicing factor 5 OS=Mus musculus GN=Srsf5 PE=1 SV=2 - [SRSF5_MOUSE]	25.28	2	5	6	61	0.20	0.24	0.13	0.25
Q9D1T5	Proline-rich protein 15 OS=Mus musculus GN=Prr15 PE=2 SV=1 - [PRR15_MOUSE]	17.21	1	1	1	9	-1.24	-1.02	-1.33	-1.12
Q8CEE6	PAS domain-containing serine/threonine-protein kinase OS=Mus musculus GN=Pask PE=2 SV=3 - [PASK_MOUSE]	1.23	1	1	1	1	-0.11	-0.09	-0.12	-0.11
E9Q7H5	Uncharacterized protein OS=Mus musculus GN=Gm8991 PE=4 SV=1 - [E9Q7H5_MOUSE]	40.00	7	11	13	442	-0.07	0.02	-0.10	-0.02
Q8CCS6-2	Isoform 2 of Polyadenylate-binding protein 2 OS=Mus musculus GN=Pabpn1 - [PABP2_MOUSE]	34.93	9	5	5	15	-0.04	-0.01	-0.07	-0.03
E9Q394	Protein Akap13 OS=Mus musculus GN=Akap13 PE=2 SV=1 - [E9Q394_MOUSE]	7.42	6	13	13	24	0.06	0.27	-0.11	0.05
Q3UT76	Protein Zfp957 OS=Mus musculus GN=Zfp957 PE=2 SV=1 - [Q3UT76_MOUSE]	8.64	1	1	2	2	-1.48	-0.95	-1.34	-0.81
A2CG77	Histone-lysine N-methyltransferase EHMT2 (Fragment) OS=Mus musculus GN=Ehmt2 PE=2 SV=1 - [A2CG77_MOUSE]	4.30	1	1	1	1	-0.58	-0.95	-0.92	-1.30
P24604-2	Isoform 2 of Tyrosine-protein kinase Tec OS=Mus musculus GN=Tec - [TEC_MOUSE]	1.14	7	1	1	1	-0.07	0.05	-0.80	-0.69
Q3UGS4	Protein FAM195B OS=Mus musculus GN=Fam195b PE=1 SV=1 - [F195B_MOUSE]	11.34	1	1	1	3	0.01	0.19	0.06	0.25
Q9CQF3	Cleavage and polyadenylation specificity factor subunit 5 OS=Mus musculus GN=Nudt21 PE=2 SV=1 - [CPSF5_MOUSE]	37.00	1	9	9	46	-0.22	-0.15	-0.13	-0.16
Q8C1D8	Protein IWS1 homolog OS=Mus musculus GN=Iws1 PE=1 SV=1 - [IWS1_MOUSE]	6.53	2	4	5	8	-0.05	0.14	-0.09	0.09
P59235	Nucleoporin Nup43 OS=Mus musculus GN=Nup43 PE=2 SV=2 - [NUP43_MOUSE]	14.74	1	3	3	6	-0.15	-0.18	-0.13	-0.22
Q8BI29	Specifically androgen-regulated gene protein OS=Mus musculus GN=Sarg PE=1 SV=2 - [SARG_MOUSE]	10.40	2	4	4	7	-0.58	-0.39	-0.46	-0.28

Q6PFD9	Nuclear pore complex protein Nup98-Nup96 OS=Mus musculus GN=Nup98 PE=1 SV=2 - [NUP98_MOUSE]	16.46	2	21	22	97	0.01	0.09	-0.05	-0.03
Q9DBB8	Trans-1,2-dihydrobenzene-1,2-diol dehydrogenase OS=Mus musculus GN=Dhdh PE=2 SV=1 - [DHDH_MOUSE]	27.33	1	7	7	26	-0.06	-0.08	-0.14	-0.18
Q9DBB4	N-alpha-acetyltransferase 16, NatA auxiliary subunit OS=Mus musculus GN=Naa16 PE=2 SV=1 - [NAA16_MOUSE]	5.79	2	2	4	8	0.03	-0.25	-0.23	-0.52
Q9Z0H8-2	Isoform 2 of CAP-Gly domain-containing linker protein 2 OS=Mus musculus GN=Clip2 - [CLIP2_MOUSE]	32.41	15	21	29	105	-0.16	-0.21	-0.16	-0.10
Q60737	Casein kinase II subunit alpha OS=Mus musculus GN=Csnk2a1 PE=1 SV=2 - [CSK21_MOUSE]	51.92	3	16	16	148	-0.41	-0.38	-0.29	-0.37
Q9DAK9	14 kDa phosphohistidine phosphatase OS=Mus musculus GN=Phpt1 PE=1 SV=1 - [PHP14_MOUSE]	29.03	1	3	3	22	-0.06	-0.05	-0.02	0.08
Q6NVE8	WD repeat-containing protein 44 OS=Mus musculus GN=Wdr44 PE=1 SV=1 - [WDR44_MOUSE]	5.68	2	3	3	4	0.33	0.31	0.28	0.09
A2AER8	Polyglutamine-binding protein 1 (Fragment) OS=Mus musculus GN=Pqbp1 PE=2 SV=1 - [A2AER8_MOUSE]	15.68	3	3	3	11	0.32	0.28	-0.07	0.03
Q8BFQ8	Parkinson disease 7 domain-containing protein 1 OS=Mus musculus GN=Pddc1 PE=1 SV=1 - [PDDC1_MOUSE]	22.73	1	4	4	18	-0.41	-0.15	-0.33	-0.14
Q9EPM5-3	Isoform 3 of Syncoilin OS=Mus musculus GN=Sync - [SYNCI_MOUSE]	5.99	3	2	2	2	-0.18	-0.36	-0.19	-0.37
Q9WUL7	ADP-ribosylation factor like protein 3 OS=Mus musculus GN=Arl3 PE=1 SV=1 - [ARL3_MOUSE]	47.80	1	6	6	22	-0.08	-0.35	-0.07	-0.18
Q9QY23	Plakophilin-3 OS=Mus musculus GN=Pkp3 PE=1 SV=2 - [PKP3_MOUSE]	16.81	4	11	11	36	-0.21	-0.18	0.01	-0.02
Q8R1J9-2	Isoform 2 of Torsin-2A OS=Mus musculus GN=Tor2a - [TOR2A_MOUSE]	10.06	3	1	1	2	0.07	-0.01	0.22	0.13
Q61321-3	Isoform 3 of Homeobox protein SIX4 OS=Mus musculus GN=Six4 - [SIX4_MOUSE]	4.59	1	1	1	1	-0.26	0.09	-0.64	-0.30
Q9CPZ8	COX assembly mitochondrial protein homolog OS=Mus musculus GN=Cmc1 PE=3 SV=1 - [COXM1_MOUSE]	23.58	1	3	3	8	0.07	0.04	0.06	0.02

E9QAF9	Protein TANC1 OS=Mus musculus GN=Tanc1 PE=2 SV=1 [E9QAF9_MOUSE]	4.60	3	5	5	12	-0.10	0.18	-0.15	0.04
Q9D0B6	Protein PBDC1 OS=Mus musculus GN=Pbdc1 PE=2 SV=1 - [PBDC1_MOUSE]	32.83	2	6	6	18	0.35	0.05	0.29	-0.06
P09470	Angiotensin-converting enzyme OS=Mus musculus GN=Ace PE=1 SV=3 - [ACE_MOUSE]	4.50	3	4	4	4	-0.55	-0.53	-0.60	-0.59
Q80XU2	Neuronal proto-oncogene tyrosine-protein kinase Src OS=Mus musculus GN=Src PE=2 SV=1 - [Q80XU2_MOUSE]	19.25	2	4	9	31	-0.25	-0.18	-0.42	-0.17
P70460	Vasodilator-stimulated phosphoprotein OS=Mus musculus GN=Vasp PE=1 SV=4 - [VASP_MOUSE]	39.73	1	15	15	173	0.13	0.25	0.06	0.21
Q8CG70	Prolyl 3-hydroxylase 3 OS=Mus musculus GN=Leprel2 PE=2 SV=1 - [P3H3_MOUSE]	2.60	1	1	1	5	-0.26	-0.15	-0.54	-0.43
Q9WV38	Solute carrier family 2, facilitated glucose transporter member 5 OS=Mus musculus GN=Slc2a5 PE=2 SV=2 - [GTR5_MOUSE]	1.20	1	1	1	1	0.77	-0.04	0.59	-0.21
Q810U4-2	Isoform 2 of Neuronal cell adhesion molecule OS=Mus musculus GN=Nrcam - [NRCAM_MOUSE]	3.96	3	2	2	2	0.15	0.28	-0.20	-0.07
Q9D1B9	39S ribosomal protein L28, mitochondrial OS=Mus musculus GN=Mrpl28 PE=2 SV=3 - [RM28_MOUSE]	17.90	2	3	3	6	-0.02	0.32	-0.03	0.25
Q9QZD9	Eukaryotic translation initiation factor 3 subunit I OS=Mus musculus GN=Eif3i PE=1 SV=1 - [EIF3I_MOUSE]	58.77	2	13	14	97	-0.27	-0.29	-0.25	-0.26
Q5NCB9	Tripartite motif-containing protein 7 (Fragment) OS=Mus musculus GN=Trim7 PE=2 SV=2 - [Q5NCB9_MOUSE]	25.93	1	1	1	8	-0.60	-0.56	-0.70	-0.67
Q8K2H2	OTU domain-containing protein 6B OS=Mus musculus GN=Otud6b PE=2 SV=1 - [OTU6B_MOUSE]	30.61	3	5	5	12	-0.08	-0.02	0.07	-0.09
Q60714	Long-chain fatty acid transport protein 1 OS=Mus musculus GN=Slc27a1 PE=1 SV=1 - [S27A1_MOUSE]	10.84	1	3	3	5	-1.16	-1.23	-0.79	-0.89
Q6P1F6	Serine/threonine-protein phosphatase 2A 55 kDa regulatory subunit B alpha isoform OS=Mus musculus GN=Ppp2r2a PE=1 SV=1 - [2ABA_MOUSE]	31.54	7	12	12	94	-0.03	-0.07	-0.03	-0.09

Q3UGC7	Eukaryotic translation initiation factor 3 subunit J-A OS=Mus musculus GN=Eif3j1 PE=2 SV=1 - [EI3JA_MOUSE]	32.95	2	10	10	59	0.15	0.26	0.12	0.18
G5E8R8	MCG129950, isoform CRA_a OS=Mus musculus GN=Ubxn7 PE=4 SV=1 - [G5E8R8_MOUSE]	8.38	2	4	4	6	-0.19	-0.14	-0.18	-0.12
Q91XQ0	Dynein heavy chain 8, axonemal OS=Mus musculus GN=Dnahc8 PE=2 SV=2 - [DYH8_MOUSE]	2.43	3	7	9	15	-0.11	0.01	-0.19	-0.03
P70677	Caspase-3 OS=Mus musculus GN=Casp3 PE=1 SV=1 - [CASP3_MOUSE]	24.55	1	6	6	58	-0.10	-0.11	-0.24	-0.23
Q6PDM2	Serine/arginine-rich splicing factor 1 OS=Mus musculus GN=Srsf1 PE=1 SV=3 - [SRSF1_MOUSE]	42.34	6	11	11	292	-0.31	-0.35	-0.34	-0.37
E9Q9M5	Ubiquitin carboxyl-terminal hydrolase OS=Mus musculus GN=Usp19 PE=2 SV=1 - [E9Q9M5_MOUSE]	7.16	4	4	4	12	0.16	0.12	-0.01	0.03
Q80U12	Small G protein signaling modulator 2 OS=Mus musculus GN=Sgsm2 PE=2 SV=2 - [SGSM2_MOUSE]	4.28	9	2	3	5	-0.51	-0.41	-0.34	-0.24
F6QKX8	Protein Mmp27 (Fragment) OS=Mus musculus GN=Mmp27 PE=4 SV=1 - [F6QKX8_MOUSE]	1.70	1	1	1	1	0.10	-0.04	-0.13	-0.27
P03975	IgE-binding protein OS=Mus musculus GN=Iap PE=2 SV=1 - [IGEB_MOUSE]	1.97	1	1	1	2	-0.09	-0.50	-0.25	-0.66
D6RIM8	Harmonin OS=Mus musculus GN=Ush1c PE=2 SV=1 - [D6RIM8_MOUSE]	17.58	8	11	11	52	-0.18	0.03	-0.16	0.04
Q60902-4	Isoform 4 of Epidermal growth factor receptor substrate 15-like 1 OS=Mus musculus GN=Eps15l1 - [EP15R_MOUSE]	31.85	4	19	19	82	-0.26	-0.14	-0.24	-0.19
F6X4A2	Regulator of G-protein-signaling 3 (Fragment) OS=Mus musculus GN=Rgs3 PE=4 SV=1 - [F6X4A2_MOUSE]	6.35	5	1	1	2	-0.05	-0.13	-0.22	-0.30
O70325-2	Isoform Cytoplasmic of Phospholipid hydroperoxide glutathione peroxidase, mitochondrial OS=Mus musculus GN=Gpx4 - [GPX41_MOUSE]	33.53	4	6	6	14	-0.21	-0.07	-0.33	-0.04
K7N6R9	Protein Vmn2r66 OS=Mus musculus GN=Vmn2r66 PE=3 SV=1 - [K7N6R9_MOUSE]	2.94	1	1	1	2	-0.33	-0.46	-0.02	-0.15
Q91X52	L-xylulose reductase OS=Mus musculus GN=Dcxr PE=2 SV=2 - [DCXR_MOUSE]	24.18	2	5	6	23	0.04	-0.08	-0.02	0.08

Q2TPA8	Hydroxysteroid dehydrogenase-like protein 2 OS=Mus musculus GN=Hsdl2 PE=2 SV=1 - [HSDL2_MOUSE]	36.73	3	11	11	58	-0.65	-0.71	-0.77	-0.73
O70582	Arachidonate 12-lipoxygenase, 12R-type OS=Mus musculus GN=Alox12b PE=2 SV=1 - [LX12B_MOUSE]	1.57	1	1	1	4	-0.02	-0.06	-0.08	-0.12
P35582	Hepatocyte nuclear factor 3-alpha OS=Mus musculus GN=Foxa1 PE=1 SV=2 - [FOXA1_MOUSE]	3.63	1	1	1	3	-0.18	0.08	-0.32	-0.05
Q3TW96	UDP-N-acetylhexosamine pyrophosphorylase-like protein 1 OS=Mus musculus GN=Uap1l1 PE=2 SV=1 - [UAP1L_MOUSE]	31.95	2	14	15	80	-0.12	-0.13	-0.12	-0.24
G3X9T1	Protein Zfp715 OS=Mus musculus GN=Zfp715 PE=4 SV=1 - [G3X9T1_MOUSE]	0.68	1	1	1	1	0.83	-0.33	0.63	-0.54
Q9WVA2	Mitochondrial import inner membrane translocase subunit Tim8 A OS=Mus musculus GN=Timm8a1 PE=1 SV=1 - [TIM8A_MOUSE]	37.11	4	4	4	8	-0.39	-0.15	-0.48	-0.26
Q9Z0E6	Interferon-induced guanylate-binding protein 2 OS=Mus musculus GN=Gbp2 PE=1 SV=1 - [GBP2_MOUSE]	19.69	3	11	11	92	0.75	0.59	0.63	0.43
Q8K2Q9	Shootin-1 OS=Mus musculus GN=Kiaa1598 PE=1 SV=1 - [SHOT1_MOUSE]	27.89	2	15	15	56	-0.07	-0.07	-0.09	-0.25
Q9CWU6-2	Isoform 2 of Ubiquinol-cytochrome c reductase complex chaperone CBP3 homolog OS=Mus musculus GN=Uqcc - [UQCC_MOUSE]	17.72	7	2	2	3	-0.34	-0.24	-0.65	-0.56
Q99KN2	Probable cytosolic iron-sulfur protein assembly protein CIAO1 OS=Mus musculus GN=Ciao1 PE=2 SV=1 [CIAO1_MOUSE]	8.85	4	2	2	5	-0.03	0.26	-0.07	0.24
Q99PJ2	Probable E3 ubiquitin-protein ligase TRIM8 OS=Mus musculus GN=Trim8 PE=1 SV=3 [TRIM8_MOUSE]	1.45	1	1	1	1	-0.14	0.11	-0.32	-0.08
Q9CXN7	Phenazine biosynthesis-like domain-containing protein 2 OS=Mus musculus GN=Pbld2 PE=2 SV=1 - [PBLD2_MOUSE]	23.96	2	4	7	29	-0.82	-0.82	-0.98	-0.82
A2AB83	E3 ubiquitin-protein ligase TRIM11 OS=Mus musculus GN=Trim11 PE=2 SV=1 - [A2AB83_MOUSE]	5.93	3	1	1	1	-0.65	-0.82	-0.90	-1.07

Q62523	Zyxin OS=Mus musculus GN=Zyx PE=1 SV=2 - [ZYX_MOUSE]	29.43	1	12	12	90	0.08	-0.07	0.01	-0.13
Q3U1Z5-2	Isoform 2 of G-protein-signaling modulator 3 OS=Mus musculus GN=Gpsm3 - [GPSM3_MOUSE]	14.88	2	1	1	8	-0.29	-0.46	-0.36	-0.45
Q99MZ3-4	Isoform 4 of Carbohydrate-responsive element-binding protein OS=Mus musculus GN=Mlxipl - [MLXPL_MOUSE]	5.60	7	2	2	3	-0.26	0.47	-0.47	0.25
Q9CR98	Protein FAM136A OS=Mus musculus GN=Fam136a PE=1 SV=1 - [F136A_MOUSE]	36.23	2	4	4	65	-0.22	-0.04	-0.25	-0.06
Q8K0V4	CCR4-NOT transcription complex subunit 3 OS=Mus musculus GN=Cnot3 PE=1 SV=1 - [CNOT3_MOUSE]	23.17	2	7	7	12	0.20	0.04	-0.10	-0.15
Q3THK3	General transcription factor IIF subunit 1 OS=Mus musculus GN=Gtf2f1 PE=1 SV=2 - [T2FA_MOUSE]	16.73	1	7	7	17	0.25	0.70	0.31	0.62
Q9WVA3	Mitotic checkpoint protein BUB3 OS=Mus musculus GN=Bub3 PE=2 SV=2 - [BUB3_MOUSE]	29.75	1	8	8	32	-0.14	-0.03	-0.13	-0.22
K7N759	Protein Gm8356 OS=Mus musculus GN=Gm8356 PE=4 SV=1 - [K7N759_MOUSE]	4.90	5	1	1	2	0.20	0.31	0.14	0.24
Q9R1P0	Proteasome subunit alpha type-4 OS=Mus musculus GN=Psma4 PE=1 SV=1 - [PSA4_MOUSE]	35.63	3	8	9	81	-0.42	-0.48	-0.37	-0.41
P29341	Polyadenylate-binding protein 1 OS=Mus musculus GN=Pabpc1 PE=1 SV=2 - [PABP1_MOUSE]	40.72	4	15	25	203	0.03	0.04	0.01	0.05
Q9QYB5-2	Isoform 1 of Gamma-adducin OS=Mus musculus GN=Add3 - [ADDG_MOUSE]	19.58	2	11	11	69	-0.42	-0.36	-0.40	-0.41
D3YVR9	MCG7316, isoform CRA_b OS=Mus musculus GN=1110004F10Rik PE=4 SV=1 - [D3YVR9_MOUSE]	17.52	3	3	3	19	-0.04	-0.03	-0.01	0.00
E9QN52	Leucine-rich repeat flightless-interacting protein 2 OS=Mus musculus GN=Lrrfip2 PE=2 SV=1 - [E9QN52_MOUSE]	29.64	3	9	11	49	-0.45	-0.32	-0.44	-0.33
E9Q9A5	Bifunctional polynucleotide phosphatase/kinase OS=Mus musculus GN=Pnkp PE=2 SV=1 - [E9Q9A5_MOUSE]	19.34	4	8	8	38	-0.01	-0.16	0.01	-0.22
Q9CQ45	Neudesin OS=Mus musculus GN=Nenf PE=1 SV=1 - [NENF_MOUSE]	32.75	1	4	4	20	-0.37	-0.21	-0.45	-0.39

D3Z5X8	Cell growth-regulating nucleolar protein (Fragment) OS=Mus musculus GN=Lyar PE=2 SV=1 - [D3Z5X8_MOUSE]	31.30	5	4	5	36	0.20	-0.13	0.22	-0.05
A2AQ25	Sickle tail protein OS=Mus musculus GN=Skt PE=1 SV=1 - [SKT_MOUSE]	15.26	14	22	22	60	-0.06	-0.23	-0.09	-0.25
Q9CYL5	Golgi-associated plant pathogenesis-related protein 1 OS=Mus musculus GN=Glipr2 PE=2 SV=3 - [GAPR1_MOUSE]	48.05	2	6	6	40	-0.29	-0.45	-0.42	-0.37
Q9Z2L6	Multiple inositol polyphosphate phosphatase 1 OS=Mus musculus GN=Minpp1 PE=1 SV=3 - [MINP1_MOUSE]	12.27	1	5	5	16	-0.28	-0.06	-0.39	-0.25
P50247	Adenosylhomocysteinase OS=Mus musculus GN=Ahcy PE=1 SV=3 - [SAHH_MOUSE]	54.40	2	22	24	377	-0.33	-0.28	-0.37	-0.31
Q80TB8	Synaptic vesicle membrane protein VAT-1 homolog-like OS=Mus musculus GN=Vat1l PE=2 SV=2 - [VAT1L_MOUSE]	5.28	1	2	2	11	-0.36	-0.23	-0.37	-0.34
P62137	Serine/threonine-protein phosphatase PP1-alpha catalytic subunit OS=Mus musculus GN=Ppp1ca PE=1 SV=1 - [PP1A_MOUSE]	36.67	1	3	11	159	-0.18	-0.24	-0.18	-0.19
Q9DCE5	p21-activated protein kinase-interacting protein 1 OS=Mus musculus GN=Pak1ip1 PE=2 SV=2 - [PK1IP_MOUSE]	6.28	1	3	3	6	0.06	0.29	-0.03	0.20
Q9CZE3	Ras-related protein Rab-32 OS=Mus musculus GN=Rab32 PE=2 SV=3 - [RAB32_MOUSE]	23.32	4	4	4	9	0.13	0.11	0.10	0.06
Q9CQ26	STAM-binding protein OS=Mus musculus GN=Stambp PE=2 SV=1 - [STABP_MOUSE]	15.80	1	5	5	12	0.14	0.06	0.12	0.01
Q9CQE3	28S ribosomal protein S17, mitochondrial OS=Mus musculus GN=Mrps17 PE=2 SV=1 - [RT17_MOUSE]	20.00	1	1	1	8	-0.07	0.21	-0.11	-0.23
A2AMY5	Ubiquitin-associated protein 2 OS=Mus musculus GN=Ubap2 PE=2 SV=1 - [A2AMY5_MOUSE]	7.16	5	7	7	14	0.03	0.02	-0.05	-0.08
D3YU96	Cytohesin-2 OS=Mus musculus GN=Cyth2 PE=2 SV=1 - [D3YU96_MOUSE]	4.44	8	2	2	3	-0.08	-0.12	-0.10	-0.25
Q921W0	Charged multivesicular body protein 1a OS=Mus musculus GN=Chmp1a PE=1 SV=1 - [CHM1A_MOUSE]	19.39	1	5	5	27	0.11	0.02	0.05	0.01

Q8BGF3	WD repeat-containing protein 92 OS=Mus musculus GN=Wdr92 PE=2 SV=1 - [WDR92_MOUSE]	4.20	1	1	1	1	-0.75	-0.25	-0.72	-0.23
P51855	Glutathione synthetase OS=Mus musculus GN=Gss PE=2 SV=1 - [GSHB_MOUSE]	51.27	5	20	20	217	-0.41	-0.36	-0.36	-0.31
Q8R2L3	MKL/myocardin-like 2 OS=Mus musculus GN=Mkl2 PE=4 SV=1 - [Q8R2L3_MOUSE]	25.49	1	1	1	1	-0.08	-0.28	-0.02	-0.22
Q9D2G2	Dihydrolipoyllysine-residue succinyltransferase component of 2-oxoglutarate dehydrogenase complex, mitochondrial OS=Mus musculus GN=Dlst PE=1 SV=1 - [ODO2_MOUSE]	32.60	2	11	11	225	-0.39	-0.35	-0.38	-0.33
Q8CDN6	Thioredoxin-like protein 1 OS=Mus musculus GN=Txnl1 PE=1 SV=3 - [TXNL1_MOUSE]	38.75	1	7	7	86	-0.09	-0.01	-0.08	-0.06
Q5NCU0	Glycine receptor subunit alpha-1 OS=Mus musculus GN=Glra1 PE=2 SV=1 - [Q5NCU0_MOUSE]	1.91	3	1	1	2	-0.70	-0.78	-0.84	-0.91
Q9JI90-2	Isoform 2 of E3 ubiquitin-protein ligase RNF14 OS=Mus musculus GN=Rnf14 - [RNF14_MOUSE]	12.66	2	1	1	2	-0.14	0.06	-0.01	0.19
E9Q453	Tropomyosin alpha-1 chain OS=Mus musculus GN=Tpm1 PE=2 SV=1 - [E9Q453_MOUSE]	62.86	2	1	23	1302	-0.85	-0.67	-0.75	-0.56
O09131	Glutathione S-transferase omega-1 OS=Mus musculus GN=Gsto1 PE=2 SV=2 [GSTO1_MOUSE]	57.92	3	16	16	326	-0.29	-0.13	-0.31	-0.22
Q9D1L9	Ragulator complex protein LAMTOR5 OS=Mus musculus GN=Lamtor5 PE=2 SV=1 - [LTOR5_MOUSE]	21.98	2	1	1	21	-0.19	-0.46	-0.21	-0.41
Q6P8I4	PEST proteolytic signal-containing nuclear protein OS=Mus musculus GN=Pcnp PE=1 SV=1 - [PCNP_MOUSE]	31.46	6	5	5	22	-0.15	0.01	-0.27	-0.08
Q8C7B4	Gamma-glutamyltransferase 5 OS=Mus musculus GN=Ggt5 PE=2 SV=1 - [Q8C7B4_MOUSE]	10.12	3	6	6	15	-0.74	-0.67	-0.96	-0.86
Q68FE8-2	Isoform 2 of Zinc finger protein 280D OS=Mus musculus GN=Znf280d [Z280D_MOUSE]	1.65	2	1	1	1	-0.44	-0.12	-0.43	-0.11
B2RY71	Protein Wdr63 OS=Mus musculus GN=Wdr63 PE=2 SV=1 - [B2RY71_MOUSE]	0.65	1	1	1	3	-0.62	-0.34	-0.72	-0.44

Q8K1B8	Fermitin family homolog 3 OS=Mus musculus GN=Fermt3 PE=1 SV=1 - [URP2_MOUSE]	19.55	1	8	9	20	0.15	0.03	0.09	-0.01
H3BIX9	Leucine-rich repeat and calponin homology domain-containing protein 4 OS=Mus musculus GN=Lrch4 PE=2 SV=1 - [H3BIX9_MOUSE]	9.74	4	4	4	8	0.08	0.52	-0.15	0.29
D3YYW8	RNA-binding protein 42 OS=Mus musculus GN=Rbm42 PE=2 SV=1 - [D3YYW8_MOUSE]	1.78	3	1	1	4	0.08	0.13	0.03	0.08
Q505B7-2	Isoform 2 of Protein archease OS=Mus musculus GN=Zbtb8os - [ARCH_MOUSE]	31.51	5	5	5	11	-0.21	0.07	-0.44	0.04
Q99LP6	GrpE protein homolog 1, mitochondrial OS=Mus musculus GN=Grpel1 PE=1 SV=1 - [GRPE1_MOUSE]	38.71	1	9	9	67	-0.17	-0.06	-0.08	-0.05
Q8C6K9-2	Isoform 2 of Collagen alpha-6(VI) chain OS=Mus musculus GN=Col6a6 - [CO6A6_MOUSE]	3.72	3	1	3	6	-0.42	-0.52	0.98	0.88
P55292	Desmocollin-2 OS=Mus musculus GN=Dsc2 PE=2 SV=1 - [DSC2_MOUSE]	36.03	18	19	21	118	-0.02	-0.04	0.04	-0.04
P40142	Transketolase OS=Mus musculus GN=Tkt PE=1 SV=1 - [TKT_MOUSE]	59.23	1	34	34	934	-0.16	-0.10	-0.21	-0.12
P56389	Cytidine deaminase OS=Mus musculus GN=Cda PE=1 SV=2 - [CDD_MOUSE]	25.34	1	2	2	10	-0.16	-0.38	-0.04	-0.25
Q8BGC1-2	Isoform 2 of UPF0489 protein C5orf22 homolog OS=Mus musculus - [CE022_MOUSE]	5.28	2	1	1	7	0.20	0.51	0.04	0.35
A2A860	WW domain binding protein 2, isoform CRA_a OS=Mus musculus GN=Wbp2 PE=4 SV=1 - [A2A860_MOUSE]	13.89	2	3	3	11	0.04	0.08	-0.04	0.02
D3Z433	NXPE family member 2 (Fragment) OS=Mus musculus GN=Nxpe2 PE=2 SV=1 - [D3Z433_MOUSE]	4.81	1	1	1	2	-0.13	0.03	-0.05	0.10
Q9CR80	Protein FAM32A OS=Mus musculus GN=Fam32a PE=2 SV=1 - [FA32A_MOUSE]	8.04	1	1	1	1	0.96	0.74	0.87	0.65
Q8BHC9	Alpha-(1,3)-fucosyltransferase 11 OS=Mus musculus GN=Fut11 PE=2 SV=1 [FUT11_MOUSE]	5.52	1	2	2	4	-0.32	-0.08	-0.18	-0.27
Q9CQV8-2	Isoform Short of 14-3-3 protein beta/alpha OS=Mus musculus GN=Ywhab - [1433B_MOUSE]	53.69	3	6	14	345	-0.20	0.05	-0.25	-0.06
P99027	60S acidic ribosomal protein P2 OS=Mus musculus GN=Rplp2 PE=1 SV=3 - [RLA2_MOUSE]	60.87	1	8	8	200	-0.03	0.05	-0.08	0.02

Q9DBL9-2	Isoform 2 of 1-acylglycerol-3-phosphate O-acyltransferase ABHD5 OS=Mus musculus GN=Abhd5 - [ABHD5_MOUSE]	11.11	2	1	1	1	-0.97	-0.65	-0.98	-0.66
L7N1X8	Protein Gbp6 OS=Mus musculus GN=Gbp6 PE=4 SV=1 - [L7N1X8_MOUSE]	7.86	2	2	5	13	0.33	0.20	-0.15	-0.28
Q9Z1D1	Eukaryotic translation initiation factor 3 subunit G OS=Mus musculus GN=Eif3g PE=1 SV=2 - [EIF3G_MOUSE]	23.13	1	8	8	59	0.28	0.35	0.16	0.22
F6UB40	Ras-responsive element-binding protein 1 (Fragment) OS=Mus musculus GN=Rreb1 PE=4 SV=1 - [F6UB40_MOUSE]	2.81	4	1	1	1	0.45	0.58	0.33	0.46
Q8CHH9	Septin-8 OS=Mus musculus GN=Sept8 PE=1 SV=4 - [SEPT8_MOUSE]	33.57	6	9	12	57	-0.25	-0.20	-0.39	-0.40
M0QWQ9	Uncharacterized protein OS=Mus musculus GN=Gm17174 PE=4 SV=1 - [M0QWQ9_MOUSE]	10.85	3	1	2	3	-0.70	-0.52	-0.66	-0.48
E9Q5D5	WW domain-containing adapter protein with coiled-coil OS=Mus musculus GN=Wac PE=2 SV=1 - [E9Q5D5_MOUSE]	8.04	11	3	3	10	0.05	0.28	-0.30	-0.11
Q5F2E7	Nuclear fragile X mental retardation-interacting protein 2 OS=Mus musculus GN=Nufip2 PE=1 SV=1 - [NUFP2_MOUSE]	19.80	2	9	9	15	-0.07	0.01	0.01	-0.02
F6U238	Protein Maml2 (Fragment) OS=Mus musculus GN=Maml2 PE=2 SV=1 - [F6U238_MOUSE]	3.49	1	1	1	2	0.06	-1.01	1.27	0.20
Q3UKZ7	ARL14 effector protein-like OS=Mus musculus GN=Arl14epl PE=2 SV=1 - [A14EL_MOUSE]	4.61	1	1	1	1	-0.18	-0.04	-0.19	-0.06
E9QNG1	Intersectin-2 OS=Mus musculus GN=Itsn2 PE=2 SV=1 - [E9QNG1_MOUSE]	6.51	3	9	10	18	-0.13	-0.22	-0.13	-0.13
E9QLB2	Lysophospholipase-like protein 1 OS=Mus musculus GN=Lyplal1 PE=4 SV=1 - [E9QLB2_MOUSE]	4.18	2	1	1	2	-0.44	-0.44	-0.34	-0.34
P56380	Bis(5'-nucleosyl)-tetraphosphatase [asymmetrical] OS=Mus musculus GN=Nudt2 PE=1 SV=3 - [AP4A_MOUSE]	21.77	1	2	2	19	0.02	0.27	-0.18	0.10
E9QLU4	Tetratricopeptide repeat protein 29 OS=Mus musculus GN=Ttc29 PE=4 SV=1 - [E9QLU4_MOUSE]	2.12	2	1	1	2	0.01	-0.02	0.16	0.13

Q99LD8	N(G),N(G)-dimethylarginine dimethylaminohydrolase 2 OS=Mus musculus GN=Ddah2 PE=1 SV=1 - [DDAH2_MOUSE]	34.39	3	7	8	50	-0.62	-0.37	-0.61	-0.41
Q9Z0F7	Gamma-synuclein OS=Mus musculus GN=Sncg PE=1 SV=1 - [SYUG_MOUSE]	73.98	1	9	9	57	-0.20	-0.20	-0.21	-0.26
Q8BQZ5-3	Isoform 3 of Cleavage and polyadenylation specificity factor subunit 4 OS=Mus musculus GN=Cpsf4 - [CPSF4_MOUSE]	21.70	8	3	3	5	-0.28	-0.09	0.05	0.25
Q9WTP2	Protein sprouty homolog 4 OS=Mus musculus GN=Spry4 PE=2 SV=1 - [SPY4_MOUSE]	4.00	1	1	1	1	-0.01	-0.05	-0.35	-0.40
O70251	Elongation factor 1-beta OS=Mus musculus GN=Eef1b PE=1 SV=5 [EF1B_MOUSE]	66.67	5	11	13	308	-0.05	0.02	-0.14	-0.07
E9QMC1	Cingulin OS=Mus musculus GN=Cgn PE=2 SV=1 - [E9QMC1_MOUSE]	29.11	4	32	33	138	-0.11	-0.11	-0.14	-0.07
Q922V4	Pleiotropic regulator 1 OS=Mus musculus GN=Plrg1 PE=2 SV=1 - [PLRG1_MOUSE]	23.39	3	7	7	31	-0.12	-0.11	-0.04	-0.10
Q8K1R3	Polyribonucleotide nucleotidyltransferase 1, mitochondrial OS=Mus musculus GN=Pnpt1 PE=1 SV=1 - [PNPT1_MOUSE]	24.90	2	19	19	52	-0.15	-0.18	-0.15	-0.14
Q8R5A6	TBC1 domain family member 22A OS=Mus musculus GN=Tbc1d22a PE=2 SV=3 - [TB22A_MOUSE]	9.30	2	3	3	4	-0.95	-2.24	-0.01	-1.30
P10518	Delta-aminolevulinic acid dehydratase OS=Mus musculus GN=Alad PE=1 SV=1 - [HEM2_MOUSE]	30.00	1	7	7	78	-0.25	-0.14	-0.31	-0.17
Q7TNI2	Transmembrane protein 59-like OS=Mus musculus GN=Tmem59l PE=2 SV=1 - [TM59L_MOUSE]	6.82	1	1	1	1	-0.15	-0.83	-0.45	-1.14
P67984	60S ribosomal protein L22 OS=Mus musculus GN=Rpl22 PE=2 SV=2 [RL22_MOUSE]	44.53	1	4	5	56	-0.07	0.02	-0.03	-0.09
Q5DTI6-4	Isoform 4 of KAT8 regulatory NSL complex subunit 1-like protein OS=Mus musculus GN=Kansl1l - [KAL1L_MOUSE]	2.17	6	1	1	1	-0.84	-3.58	-0.63	-3.37
Q9D103	Interferon-induced transmembrane protein 1 OS=Mus musculus GN=Ifitm1 PE=2 SV=1 [IFM1_MOUSE]	15.09	1	1	1	3	0.41	-0.10	0.27	-0.23
E9Q9H2	DnaJ homolog subfamily C member 2 OS=Mus musculus GN=Dnajc2 PE=2 SV=1 - [E9Q9H2_MOUSE]	12.25	3	8	8	28	0.07	0.12	-0.09	-0.04

Q8VC03	Echinoderm microtubule-associated protein-like 3 OS=Mus musculus GN=Eml3 PE=2 SV=1 - [EMAL3_MOUSE]	7.25	1	5	5	6	-0.04	-0.22	-0.27	-0.28
P56812	Programmed cell death protein 5 OS=Mus musculus GN=Pdcd5 PE=1 SV=3 - [PDCD5_MOUSE]	48.41	2	6	6	55	-0.13	-0.15	-0.14	-0.18
P63005	Platelet-activating factor acetylhydrolase IB subunit alpha OS=Mus musculus GN=Pafah1b1 PE=1 SV=2 - [LIS1_MOUSE]	44.15	3	14	15	113	-0.10	0.00	-0.09	-0.04
Q03958	Prefoldin subunit 6 OS=Mus musculus GN=Pfdn6 PE=2 SV=1 [PFD6_MOUSE]	55.91	1	1	7	37	0.18	0.19	0.01	0.02
Q8R5M8-5	Isoform 5 of Cell adhesion molecule 1 OS=Mus musculus GN=Cadm1 - [CADM1_MOUSE]	10.71	9	3	4	12	-0.56	-0.72	-0.63	-0.74
Q8BHL8	Proteasome inhibitor PI31 subunit OS=Mus musculus GN=Psmf1 PE=1 SV=1 - [PSMF1_MOUSE]	14.76	1	4	4	13	-0.02	-0.35	-0.07	-0.20
Q9WUK2-2	Isoform Short of Eukaryotic translation initiation factor 4H OS=Mus musculus GN=Eif4h - [IF4H_MOUSE]	37.72	2	7	7	98	0.00	0.07	-0.10	-0.02
Q91ZE0	Trimethyllysine dioxygenase, mitochondrial OS=Mus musculus GN=Tmlhe PE=2 SV=2 - [TMLH_MOUSE]	11.64	1	6	6	10	-0.64	-0.43	-0.61	-0.71
O35381	Acidic leucine-rich nuclear phosphoprotein 32 family member A OS=Mus musculus GN=Anp32a PE=1 SV=1 - [AN32A_MOUSE]	36.44	5	8	12	255	-0.51	-0.50	-0.53	-0.49
Q6Y685	Transforming acidic coiled-coil-containing protein 1 OS=Mus musculus GN=Tacc1 PE=1 SV=1 - [TACC1_MOUSE]	7.88	3	5	7	23	-0.36	-0.46	-0.41	-0.56
Q8BWF0	Succinate-semialdehyde dehydrogenase, mitochondrial OS=Mus musculus GN=Aldh5a1 PE=1 SV=1 - [SSDH_MOUSE]	23.71	1	10	10	53	-0.55	-0.53	-0.59	-0.48
Q9CY58	Plasminogen activator inhibitor 1 RNA-binding protein OS=Mus musculus GN=Serbp1 PE=1 SV=2 - [PAIRB_MOUSE]	41.52	4	17	17	173	0.27	0.31	0.24	0.31
Q8CGI1	Protein FAM193A OS=Mus musculus GN=Fam193a PE=1 SV=2 - [F193A_MOUSE]	3.90	3	3	3	7	0.05	0.15	-0.25	-0.16

E9Q309	Protein Cep350 OS=Mus musculus GN=Cep350 PE=2 SV=1 - [E9Q309_MOUSE]	2.42	2	5	7	15	0.08	0.24	-0.05	0.03
Q711T7	Glutamine-dependent NAD(+) synthetase OS=Mus musculus GN=Nadsyn1 PE=2 SV=1 - [NADE_MOUSE]	5.52	3	3	3	4	-0.28	-0.41	-0.16	-0.21
Q9WUR2-2	Isoform 2 of Enoyl-CoA delta isomerase 2, mitochondrial OS=Mus musculus GN=Eci2 - [ECI2_MOUSE]	20.95	11	6	6	20	-0.10	-0.18	-0.18	-0.03
P12382	6-phosphofructokinase, liver type OS=Mus musculus GN=Pfkl PE=1 SV=4 - [K6PL_MOUSE]	33.21	1	17	24	104	0.11	-0.03	0.19	0.04
Q9D787	Peptidyl-prolyl cis-trans isomerase-like 2 OS=Mus musculus GN=Ppil2 PE=2 SV=2 - [PPIL2_MOUSE]	12.09	2	5	5	16	0.02	0.22	0.05	0.19
Q8R1G2	Carboxymethylenebutenolidase homolog OS=Mus musculus GN=Cmbl PE=2 SV=1 - [CMBL_MOUSE]	26.94	3	6	6	21	-0.44	-0.49	-0.48	-0.53
P12961	Neuroendocrine protein 7B2 OS=Mus musculu GN=Scg5 PE=1 SV=1 [7B2_MOUSE]	9.43	1	1	1	4	-0.42	-0.39	-0.75	-0.72
B2RXR6	Serine/threonine-protein phosphatase 6 regulatory ankyrin repeat subunit B OS=Mus musculus GN=Ankrd44 PE=2 SV=1 - [ANR44_MOUSE]	3.22	3	2	2	3	0.18	0.18	0.24	0.24
B1AY13	Ubiquitin carboxyl-terminal hydrolase 24 OS=Mus musculus GN=Usp24 PE=1 SV=1 - [UBP24_MOUSE]	2.03	4	3	5	18	-0.87	-1.06	-1.21	-0.94
Q01279	Epidermal growth factor receptor OS=Mus musculus GN=Egfr PE= SV=1 - [EGFR_MOUSE]	11.07	2	10	10	30	-0.24	-0.16	-0.28	-0.22
Q3URQ7-2	Isoform 2 of Methenyltetrahydrofolate synthase domain-containing protein OS=Mus musculus GN=Mthfsd - [MTHSD_MOUSE]	1.98	2	1	1	1	-0.31	-0.43	0.07	-0.05
Q91YD3	mRNA-decapping enzyme 1A OS=Mus musculus GN=Dcp1a PE=1 SV=1 - [DCP1A_MOUSE]	5.32	1	2	2	6	-0.16	-0.40	-0.14	-0.38
P46656	Adrenodoxin, mitochondrial OS=Mus musculus GN=Fdx1 PE=2 SV=1 - [ADX_MOUSE]	16.49	1	4	4	8	-0.32	-0.33	-0.42	-0.35
Q14AR7	MCG6726, isoform CRA_f OS=Mus musculus GN=Mrgbp PE=2 SV=1 - [Q14AR7_MOUSE]	6.37	3	1	1	2	0.48	0.26	-0.11	-0.33

Q8VCT3	Aminopeptidase B OS=Mus musculus GN=Rnpep PE=2 SV=2 - [AMPB_MOUSE]	33.69	2	20	20	184	0.20	0.12	0.17	0.14
A2AKK5-2	Isoform 2 of Acyl-coenzyme A amino acid N-acyltransferase 1 OS=Mus musculus GN=Acnat1 - [ACNT1_MOUSE]	12.06	6	3	3	15	-0.34	-0.08	-0.23	-0.06
Q3UN00	P2X purinoceptor OS=Mus musculus GN=P2rx7 PE=2 SV=1 [Q3UN00_MOUSE]	3.25	3	1	1	1	-0.90	-0.40	-1.11	-0.60
P63213	Guanine nucleotide-binding protein G(I)/G(S)/G(O) subunit gamma-2 OS=Mus musculus GN=Gng2 PE=1 SV=2 - [GBG2_MOUSE]	52.11	1	3	3	13	-0.21	0.03	-0.01	-0.12
Q8BWT1	3-ketoacyl-CoA thiolase mitochondrial OS=Mus musculus GN=Acaa2 PE=1 SV=3 - [THIM_MOUSE]	61.71	1	17	17	233	-0.38	-0.32	-0.45	-0.40
Q9CY00	Tetratricopeptide repeat protein 30B OS=Mus musculus GN=Ttc30b PE=2 SV=1 - [TT30B_MOUSE]	8.13	3	3	3	3	0.35	0.58	0.36	0.60
A1YIY0	Fc receptor-like protein 6 OS=Mus musculus GN=Fcrl6 PE=2 SV=1 - [FCRL6_MOUSE]	10.66	3	1	2	3	-0.35	0.05	-0.19	0.20
P62715	Serine/threonine-protein phosphatase 2A catalytic subunit beta isoform OS=Mus musculus GN=Ppp2cb PE=1 SV=1 - [PP2AB_MOUSE]	47.25	1	1	11	322	-0.04	-0.36	-0.07	-0.26
Q9CZR8	Elongation factor Ts, mitochondrial OS=Mus musculus GN=Tsfm PE=2 SV=1 - [EFTS_MOUSE]	26.23	3	7	7	69	-0.10	0.00	-0.29	-0.13
Q9JLJ2	4-trimethylaminobutyraldehyde dehydrogenase OS=Mus musculus GN=Aldh9a1 PE=1 SV=1 - [AL9A1_MOUSE]	34.62	2	15	16	95	-0.37	-0.28	-0.30	-0.17
F6QL70	Protein Gm17669 OS=Mus musculus GN=Gm17669 PE=4 SV=1 - [F6QL70_MOUSE]	25.32	5	4	4	54	-0.07	-0.03	-0.24	-0.17
Q9JJ89	Coiled-coil domain-containing protein 86 OS=Mus musculus GN=Ccdc86 PE=1 SV=2 - [CCD86_MOUSE]	2.58	1	1	1	2	-0.03	-0.01	0.13	0.15
E9Q8C5	Protein FAM3C OS=Mu musculus GN=Fam3c PE=2 SV=1 - [E9Q8C5_MOUSE]	20.20	6	2	2	3	-0.13	0.11	-0.30	-0.06
P55050	Fatty acid-binding protein, intestinal OS=Mus musculus GN=Fabp2 PE=2 SV=2 [FABPI_MOUSE]	41.67	1	7	7	127	-0.70	-0.48	-0.71	-0.51

Q9JJ26	Pyrin OS=Mus musculus GN=Mefv PE=2 SV=2 - [MEFV_MOUSE]	0.91	2	1	1	2	0.07	-0.01	0.05	-0.03
B1AT92	Growth factor receptor-bound protein 2 OS=Mus musculus GN=Grb2 PE=2 SV=1 - [B1AT92_MOUSE]	57.64	4	12	12	62	-0.25	-0.09	-0.31	-0.24
Q60598	Src substrate cortactin OS=Mus musculus GN=Cttn PE=1 SV=2 - [SRC8_MOUSE]	44.32	2	23	23	141	-0.21	-0.15	-0.20	-0.15
G3UYV7	40S ribosomal protein S28 (Fragment) OS=Mus musculus GN=Rps28 PE=2 SV=1 - [G3UYV7_MOUSE]	57.14	3	3	3	107	0.10	-0.03	-0.01	-0.03
Q8BG73	SH3 domain-binding glutamic acid-rich-like protein 2 OS=Mus musculus GN=Sh3bgrl2 PE=1 SV=1 - [SH3L2_MOUSE]	52.34	1	5	5	122	-0.13	-0.10	-0.13	-0.06
O89109	Intermediate conductance calcium-activated potassium channel protein 4 OS=Mus musculus GN=Kcnn4 PE=2 SV=1 - [KCNN4_MOUSE]	1.65	1	1	1	2	-0.02	0.21	-0.13	0.10
Q9D9F1	Uncharacterized protein OS=Mus musculus GN=1700084J12Rik PE=2 SV=1 - [Q9D9F1_MOUSE]	13.01	2	1	1	2	-0.18	-0.25	0.05	-0.02
Q91WG0	Carboxylesterase 2 OS=Mus musculus GN=Ces2c PE=2 SV=1 [Q91WG0_MOUSE]	41.89	3	9	15	229	-0.92	-0.98	-0.91	-1.00
Q3TIV5	Zinc finger CCCH domain-containing protein 15 OS=Mus musculus GN=Zc3h15 PE=1 SV=2 - [ZC3HF_MOUSE]	17.84	3	7	7	20	0.30	0.37	0.09	0.48
O08716	Fatty acid-binding protein 9 OS=Mus musculus GN=Fabp9 PE=2 SV=2 - [FABP9_MOUSE]	9.85	2	1	2	27	0.53	0.50	0.30	0.28
Q8K2D3	Enhancer of mRNA-decapping protein 3 OS=Mus musculus GN=Edc3 PE=2 SV=1 - [EDC3_MOUSE]	4.53	1	2	2	2	-0.04	0.31	-0.09	0.26
Q9D9V3-2	Isoform 2 of Ethylmalonyl-CoA decarboxylase OS=Mus musculus GN=Echdc1 - [ECHD1_MOUSE]	17.73	3	4	4	26	-0.08	-0.11	-0.19	-0.16
A2A7B0	SPRY domain-containing SOCS box protein 1 (Fragment) OS=Mus musculus GN=Spsb1 PE=2 SV=1 [A2A7B0_MOUSE]	30.00	6	1	1	1	-0.16	0.05	-0.04	0.17
Q9JIY2-4	Isoform 4 of E3 ubiquitin-protein ligase Hakai OS=Mus musculus GN=Cbll1 - [HAKAI_MOUSE]	10.24	5	2	2	3	-0.04	-0.06	-0.06	-0.08

G3XA35	MCG116562, isoform CRA_a OS=Mus musculus GN=Vcan PE=4 SV=1 - [G3XA35_MOUSE]	6.85	12	12	12	80	-0.18	-0.18	-0.21	-0.25
Q6R891	Neurabin-2 OS=Mus musculus GN=Ppp1r9b PE=1 SV=1 - [NEB2_MOUSE]	17.99	3	8	11	51	-0.28	-0.84	-0.14	-0.53
Q9CXW3	Calcyclin-binding protein OS=Mus musculus GN=Cacybp PE=1 SV=1 - [CYBP_MOUSE]	42.79	1	8	9	45	0.22	0.09	0.21	0.24
Q9ERF3	WD repeat-containing protein 61 OS=Mus musculus GN=Wdr61 PE=2 SV=1 - [WDR61_MOUSE]	40.00	4	7	7	27	-0.48	-0.19	-0.31	-0.30
A3KGU7	Spectrin alpha chain, non-erythrocytic 1 OS=Mus musculus GN=Sptan1 PE=2 SV=1 - [A3KGU7_MOUSE]	68.19	3	2	153	2283	-0.64	-0.30	-0.73	-0.42
O55111	Desmoglein-2 OS=Mus musculus GN=Dsg2 PE=1 SV=3 - [DSG2_MOUSE]	36.99	3	27	27	269	-0.05	0.02	-0.09	-0.02
Q91WN1	DnaJ homolog subfamily C member 9 OS=Mus musculus GN=Dnajc9 PE=2 SV=2 - [DNJC9_MOUSE]	38.61	1	7	7	17	0.09	-0.10	0.09	-0.17
A2APX3	Cystatin-C (Fragment) OS=Mus musculus GN=Cst3 PE=2 SV=1 - [A2APX3_MOUSE]	10.78	2	1	1	10	-0.18	0.33	-0.45	-0.12
O35988	Syndecan-4 OS=Mus musculus GN=Sdc4 PE=1 SV=1 - [SDC4_MOUSE]	26.26	1	4	4	15	-0.38	-0.18	-0.54	-0.37
P14106	Complement C1q subcomponent subunit B OS=Mus musculus GN=C1qb PE=1 SV=2 - [C1QB_MOUSE]	4.74	1	1	1	3	0.04	0.09	0.13	-0.05
P58466	Carboxy-terminal domain RNA polymerase II polypeptide A small phosphatase 1 OS=Mus musculus GN=Ctdsp1 PE=1 SV=1 - [CTDS1_MOUSE]	7.28	1	1	2	6	-0.52	0.26	-0.31	0.47
P52480	Pyruvate kinase PKM OS=Mus musculus GN=Pkm PE=1 SV=4 - [KPYM_MOUSE]	63.65	1	4	33	1314	-0.11	-0.13	-0.08	-0.21
O08539-2	Isoform 2 of Myc box-dependent-interacting protein 1 OS=Mus musculus GN=Bin1 - [BIN1_MOUSE]	26.73	3	7	9	32	-0.14	-0.13	-0.14	-0.34
P50580	Proliferation-associated protein 2G4 OS=Mus musculus GN=Pa2g4 PE=1 SV=3 - [PA2G4_MOUSE]	50.76	2	17	18	352	0.11	0.19	0.06	0.15
Q8BGS7	Choline/ethanolaminephosphotransferase 1 OS=Mus musculus GN=Cept1 PE=2 SV=1 [CEPT1_MOUSE]	2.16	2	1	1	1	-0.04	-0.36	-0.32	-0.64

Q9DCW4	Electron transfer flavoprotein subunit beta OS=Mus musculus GN=Etfb PE=1 SV=3 - [ETFB_MOUSE]	58.04	1	14	14	313	-0.43	-0.52	-0.43	-0.42
E9PZP3	Receptor-interacting serine/threonine-protein kinase 3 OS=Mus musculus GN=Ripk3 PE=4 SV=1 - [E9PZP3_MOUSE]	27.96	3	6	6	16	-0.04	-0.06	0.13	0.18
Q14BI7	Putative ATP-dependent RNA helicase TDRD9 OS=Mus musculus GN=Tdrd9 PE=1 SV=3 [TDRD9_MOUSE]	0.94	1	1	1	1	-0.30	-0.66	0.11	-0.25
P18760	Cofilin-1 OS=Mus musculus GN=Cfl1 PE=1 SV=3 - [COF1_MOUSE]	85.54	1	1	22	702	0.17	0.03	0.24	0.02
Q9D115	Zinc finger protein 706 OS=Mus musculus GN=Znf706 PE=2 SV=1 - [ZN706_MOUSE]	25.00	1	2	2	6	-0.03	-0.63	0.32	0.18
Q543M9	MCG14605, isoform CRA_c OS=Mus musculus GN=Trp53rk PE=2 SV=1 - [Q543M9_MOUSE]	11.07	3	2	2	8	0.28	0.29	0.26	0.15
O88844	Isocitrate dehydrogenase [NADP] cytoplasmic OS=Mus musculus GN=Idh1 PE=1 SV=2 - [IDHC_MOUSE]	64.01	2	30	31	853	-0.08	0.02	-0.17	-0.06
P46718	Programmed cell death protein 2 OS=Mus musculus GN=Pdcd2 PE=2 SV=2 - [PDCD2_MOUSE]	4.66	1	1	1	4	0.37	0.50	0.27	0.40
Q9CR00	26S proteasome non-ATPase regulatory subunit 9 OS=Mus musculus GN=Psmd9 PE=1 SV=1 - [PSMD9_MOUSE]	26.58	1	5	5	34	-0.28	-0.29	-0.25	-0.30
F8VQ28	Paxillin OS=Mus musculus GN=Pxn PE=2 SV=1 - [F8VQ28_MOUSE]	26.06	3	11	11	40	-0.05	0.04	-0.12	-0.03
Q9CXW2	28S ribosomal protein S22, mitochondrial OS=Mus musculus GN=Mrps22 PE=2 SV=1 - [RT22_MOUSE]	19.50	1	6	6	16	-0.08	-0.07	-0.17	-0.18
Q8JZM7	Parafibromin OS=Mus musculus GN=Cdc73 PE=2 SV=1 - [CDC73_MOUSE]	14.31	1	9	9	39	-0.13	-0.06	-0.32	-0.13
Q6IFX2	Keratin, type I cytoskeletal 42 OS=Mus musculus GN=Krt42 PE=1 SV=1 [K1C42_MOUSE]	10.62	5	1	8	203	-0.04	0.07	0.00	0.11
O35679	Serine protease inhibitor Kazal-type 4 OS=Mus musculus GN=Spink4 PE=2 SV=2 - [ISK4_MOUSE]	6.98	1	1	1	7	0.85	0.96	0.79	0.84

Q8BX10-2	Isoform 2 of Serine/threonine-protein phosphatase PGAM5, mitochondrial OS=Mus musculus GN=Pgam5 - [PGAM5_MOUSE]	30.66	3	8	10	24	-0.29	-0.42	-0.36	-0.41
Q9Z1M8	Protein Red OS=Mus musculus GN=Ik PE=2 SV=2 - [RED_MOUSE]	17.59	1	9	9	26	-0.01	0.09	-0.03	0.03
F8VQD7	Receptor-type tyrosine-protein phosphatase gamma OS=Mus musculus GN=Ptprg PE=2 SV=1 - [F8VQD7_MOUSE]	2.36	3	2	2	5	-0.84	-0.60	-0.73	-0.50
P56565	Protein S100-A1 OS=Mus musculus GN=S100a1 PE=1 SV=2 - [S10A1_MOUSE]	15.96	2	1	1	17	-0.28	-0.26	-0.37	-0.34
Q99K41	EMILIN-1 OS=Mus musculus GN=Emilin1 PE=1 SV=1 - [EMIL1_MOUSE]	20.94	1	18	18	110	-0.49	-0.55	-0.43	-0.48
O88487	Cytoplasmic dynein 1 intermediate chain 2 OS=Mus musculus GN=Dync1i2 PE=2 SV=1 - [DC1I2_MOUSE]	17.81	5	9	9	100	-0.22	-0.22	-0.13	-0.03
F6TZU3	Gigaxonin (Fragment) OS=Mus musculus GN=Gan PE=4 SV=1 - [F6TZU3_MOUSE]	10.72	2	4	4	9	-0.11	-0.33	-0.36	-0.35
P51885	Lumican OS=Mus musculus GN=Lum PE=1 SV=2 - [LUM_MOUSE]	40.53	1	12	12	721	-0.52	-0.43	-0.57	-0.47
Q9Z0N2	Eukaryotic translation initiation factor 2 subunit 3, Y-linked OS=Mus musculus GN=Eif2s3y PE=2 SV=2 - [IF2H_MOUSE]	30.93	1	2	11	49	-0.81	-0.43	-0.36	0.04
Q9D0M0	Exosome complex exonuclease RRP42 OS=Mus musculus GN=Exosc7 PE=2 SV=2 - [EXOS7_MOUSE]	18.56	1	3	3	18	-0.37	-0.17	-0.27	-0.59
P62911	60S ribosomal protein L32 OS=Mus musculus GN=Rpl32 PE=2 SV=2 [RL32_MOUSE]	39.26	4	7	7	78	-0.19	-0.10	-0.09	-0.04
P09041	Phosphoglycerate kinase 2 OS=Mus musculus GN=Pgk2 PE=1 SV=4 - [PGK2_MOUSE]	21.58	4	1	9	177	0.08	-0.05	-0.01	-0.14
P24527	Leukotriene A-4 hydrolase OS=Mus musculus GN=Lta4h PE=1 SV=4 - [LKHA4_MOUSE]	40.75	1	24	24	253	-0.39	-0.43	-0.45	-0.49
Q9JKB1	Ubiquitin carboxyl-terminal hydrolase isozyme L3 OS=Mus musculus GN=Uchl3 PE=1 SV=2 - [UCHL3_MOUSE]	42.61	2	8	8	63	-0.12	-0.20	-0.15	-0.17
O35245-2	Isoform 2 of Polycystin-2 OS=Mus musculus GN=Pkd2 - [PKD2_MOUSE]	8.52	5	3	3	5	-0.30	-0.37	-0.46	-0.73

Q9WV32	Actin-related protein 2/3 complex subunit 1B OS=Mus musculus GN=Arpc1b PE=1 SV=4 - [ARC1B_MOUSE]	39.25	3	10	14	164	-0.01	-0.10	-0.01	-0.04
Q922E6	FAST kinase domain-containing protein 2 OS=Mus musculus GN=Fastkd2 PE=2 SV=2 - [FAKD2_MOUSE]	5.66	1	3	3	5	-0.29	0.00	-0.33	-0.13
Q9CQM9	Glutaredoxin-3 OS=Mus musculus GN=Glrx3 PE=1 SV=1 [GLRX3_MOUSE]	32.05	1	9	9	111	-0.17	-0.27	-0.21	-0.27
Q9D903	Probable rRNA-processing protein EBP2 OS=Mus musculus GN=Ebna1bp2 PE=2 SV=1 - [EBP2_MOUSE]	8.82	2	4	4	8	0.31	0.37	0.20	0.25
Q9R112	Sulfide:quinone oxidoreductase, mitochondrial OS=Mus musculus GN=Sqrdl PE=2 SV=3 - [SQRD_MOUSE]	62.00	4	28	28	397	-0.18	-0.17	-0.21	-0.15
E9Q2C0	Protein 9330101J02Rik (Fragment) OS=Mus musculus GN=9330101J02Rik PE=2 SV=1 - [E9Q2C0_MOUSE]	0.98	1	1	1	1	-0.15	-0.90	-0.01	-0.76
O89100	GRB2-related adaptor protein 2 OS=Mus musculus GN=Grap2 PE=1 SV=1 - [GRAP2_MOUSE]	4.35	1	1	1	2	-0.51	-0.51	-0.40	-0.40
Q8CI66	Stomatin-like protein 1 OS=Mus musculus GN=Stoml1 PE=2 SV=1 - [STML1_MOUSE]	1.50	1	1	1	3	-0.26	-0.03	-0.29	-0.06
Q8K019-3	Isoform 3 of Bcl-2-associated transcription factor 1 OS=Mus musculus GN=Bclaf1 - [BCLF1_MOUSE]	14.57	4	9	9	32	0.00	-0.04	-0.01	-0.08
J3QM75	MCG1039283 OS=Mus musculus GN=Scgb2b19 PE=4 SV=1 - [J3QM75_MOUSE]	10.71	1	1	1	1	0.11	0.01	-0.01	-0.11
P23198	Chromobox protein homolog 3 OS=Mus musculus GN=Cbx3 PE=1 SV=2 - [CBX3_MOUSE]	39.89	4	5	7	117	0.11	0.27	0.06	0.17
E9Q3P4	Protein Cenpf OS=Mus musculus GN=Cenpf PE=2 SV=1 - [E9Q3P4_MOUSE]	1.84	2	2	5	8	-0.68	-0.68	-0.92	-0.93
O88958	Glucosamine-6-phosphate isomerase 1 OS=Mus musculus GN=Gnpda1 PE=2 SV=3 - [GNPI1_MOUSE]	26.99	2	5	6	23	-0.44	-0.12	-0.56	-0.34
P34022	Ran-specific GTPase-activating protein OS=Mus musculus GN=Ranbp1 PE=1 SV=2 - [RANG_MOUSE]	51.72	2	6	6	113	-0.01	-0.08	0.02	-0.06

Q148W8	Inactive dual specificity phosphatase 27 OS=Mus musculus GN=Dusp27 PE=2 SV=1 - [DUS27_MOUSE]	4.31	1	3	3	5	-0.35	-0.66	-0.11	-0.42
Q9CQE1	Protein NipSnap homolog 3B OS=Mus musculus GN=Nipsnap3b PE=1 SV=1 - [NPS3B_MOUSE]	24.29	2	5	5	16	-0.32	-0.16	-0.58	-0.36
Q80T11	Usher syndrome type-1G protein homolog OS=Mus musculus GN=Ush1g PE=1 SV=1 - [USH1G_MOUSE]	4.77	1	2	2	3	-0.18	-0.04	-0.01	0.13
D3Z7C0	39S ribosomal protein L40, mitochondrial OS=Mus musculus GN=Mrpl40 PE=2 SV=1 - [D3Z7C0_MOUSE]	27.16	2	4	4	16	0.60	0.49	0.30	0.46
Q80VJ2	Steroid receptor RNA activator 1 OS=Mus musculus GN=Sra1 PE=1 SV=3 - [SRA1_MOUSE]	21.55	2	5	5	10	0.00	0.27	-0.12	0.15
Q99LU0	Charged multivesicular body protein 1b-1 OS=Mus musculus GN=Chmp1b1 PE=1 SV=1 - [CH1B1_MOUSE]	18.09	1	2	5	13	0.08	-0.30	-0.55	-0.96
P97369	Neutrophil cytosol factor 4 OS=Mus musculus GN=Ncf4 PE=2 SV=2 - [NCF4_MOUSE]	13.27	2	3	3	10	0.59	0.41	0.53	0.35
Q8BML1-3	Isoform 3 of Protein-methionine sulfoxide oxidase MICAL2 OS=Mus musculus GN=Mical2 - [MICA2_MOUSE]	1.47	3	1	1	1	-0.11	0.30	-0.33	0.07
Q8BYC6	Serine/threonine-protein kinase TAO3 OS=Mus musculus GN=Taok3 PE=1 SV=2 - [TAOK3_MOUSE]	11.25	5	8	10	22	-0.26	-0.46	-0.25	-0.46
Q9D2F7	Nuclear pore membrane glycoprotein 210-like OS=Mus musculus GN=Nup210l PE=2 SV=2 - [P210L_MOUSE]	3.03	1	3	3	4	-0.20	0.09	-0.22	0.07
Q91YZ8	Poly(A) binding protein, cytoplasmic 4 OS=Mus musculus GN=Pabpc4 PE=2 SV=1 - [Q91YZ8_MOUSE]	22.93	5	7	13	82	0.11	0.24	-0.02	0.06
P62077	Mitochondrial import inner membrane translocase subunit Tim8 B OS=Mus musculus GN=Timm8b PE=3 SV=1 - [TIM8B_MOUSE]	31.33	1	3	3	10	-0.44	-0.32	-0.37	-0.16
D3YWD5	Major facilitator superfamily domain-containing protein 10 (Fragment) OS=Mus musculus GN=Mfsd10 PE=2 SV=1 - [D3YWD5_MOUSE]	9.32	4	2	2	15	-0.05	-0.17	-0.05	-0.16

Q8BT07	Centrosomal protein of 55 kDa OS=Mus musculus GN=Cep55 PE=1 SV=2 - [CEP55_MOUSE]	3.03	1	1	1	2	-0.22	-0.24	-0.13	-0.15
Q9D3D9	ATP synthase subunit delta, mitochondrial OS=Mus musculus GN=Atp5d PE=1 SV=1 [ATPD_MOUSE]	17.26	2	2	3	58	-0.38	-0.26	-0.50	-0.34
P11087-2	Isoform 2 of Collagen alpha-1(I) chain OS=Mus musculus GN=Col1a1 - [CO1A1_MOUSE]	33.39	2	18	19	126	-0.52	-0.44	-0.56	-0.42
Q91Z58	Uncharacterized protein C6orf132 homolog OS=Mus musculus PE=1 SV=2 - [CF132_MOUSE]	1.99	1	2	2	4	-0.16	-0.65	-0.19	-0.51
Q62419	Endophilin-A2 OS=Mus musculus GN=Sh3gl1 PE=1 SV=1 - [SH3G1_MOUSE]	39.13	1	9	11	50	-0.03	-0.21	-0.21	-0.05
P51432	1-phosphatidylinositol 4,5-bisphosphate phosphodiesterase beta-3 OS=Mus musculus GN=Plcb3 PE=2 SV=2 - [PLCB3_MOUSE]	32.09	1	28	30	248	-0.26	-0.34	-0.24	-0.28
P33587	Vitamin K-dependent protein C OS=Mus musculus GN=Proc PE=1 SV=2 - [PROC_MOUSE]	5.65	1	2	2	5	-0.46	-0.82	-0.03	-0.39
Q9QXE7	F-box-like/WD repeat-containing protein TBL1X OS=Mus musculus GN=Tbl1x PE=2 SV=2 - [TBL1X_MOUSE]	8.35	1	1	3	7	-0.32	-0.36	-0.51	-0.63
P63330	Serine/threonine-protein phosphatase 2A catalytic subunit alpha isoform OS=Mus musculus GN=Ppp2ca PE=1 SV=1 - [PP2AA_MOUSE]	49.84	1	2	12	334	-0.10	-0.17	-0.28	-0.33
Q8VIJ6	Splicing factor, proline- and glutamine-rich OS=Mus musculus GN=Sfpq PE=1 SV=1 - [SFPQ_MOUSE]	27.61	1	18	19	247	-0.17	-0.15	-0.19	-0.15
O70423	Membrane primary amine oxidase OS=Mus musculus GN=Aoc3 PE=2 SV=3 - [AOC3_MOUSE]	24.44	5	16	16	366	-0.78	-0.69	-0.77	-0.66
Q9Z0J1	Reversion-inducing cysteine-rich protein with Kazal motifs OS=Mus musculus GN=Reck PE=2 SV=2 - [RECK_MOUSE]	1.03	1	1	1	1	-0.24	-0.22	-0.58	-0.57
Q8JZP9	GAS2-like protein 1 OS=Mus musculus GN=Gas2l1 PE=2 SV=1 - [GA2L1_MOUSE]	1.62	1	1	1	2	-0.24	0.13	-0.69	-0.32
Q8R2V5	Arf-GAP with dual PH domain-containing protein 2 OS=Mus musculus GN=Adap2 PE=2 SV=1 - [ADAP2_MOUSE]	1.57	1	1	1	6	0.10	0.17	0.03	0.10

D3Z7C6	Prostaglandin E synthase 3 OS=Mus musculus GN=Ptges3 PE=2 SV=1 - [D3Z7C6_MOUSE]	46.15	2	6	6	126	0.11	0.10	0.04	0.05
E9Q166	Protein Atad2b OS=Mus musculus GN=Atad2b PE=2 SV=1 - [E9Q166_MOUSE]	1.23	1	2	2	7	-0.19	-0.20	-0.14	-0.15
Q8C166	Copine-1 OS=Mus musculus GN=Cpne1 PE=1 SV=1 - [CPNE1_MOUSE]	21.08	8	6	10	48	-0.41	-0.70	-0.43	-0.72
Q923P0-2	Isoform 2 of Collagen alpha-1(XX) chain OS=Mus musculus GN=Col20a1 - [COKA1_MOUSE]	1.11	3	1	1	9	-0.29	-0.50	-0.23	-0.36
B7ZNP3	COMM domain-containing protein 6 OS=Mus musculus GN=Commd6 PE=4 SV=1 - [B7ZNP3_MOUSE]	16.87	2	1	1	11	0.10	0.16	-0.06	-0.01
Q9WUD1	STIP1 homology and U box-containing protein 1 OS=Mus musculus GN=Stub1 PE=1 SV=1 [CHIP_MOUSE]	36.51	1	9	10	29	0.01	0.15	-0.08	0.06
P08551	Neurofilament light polypeptide OS=Mus musculus GN=Nefl PE=1 SV=5 - [NFL_MOUSE]	4.60	1	2	3	95	-0.91	-0.88	-1.02	-0.99
Q9JI11	Serine/threonine-protein kinase 4 OS=Mus musculus GN=Stk4 PE=1 SV=1 - [STK4_MOUSE]	27.31	5	8	12	31	-0.06	0.12	-0.02	0.18
D3YUD8	Uncharacterized protein OS=Mus musculus GN=Gm9774 PE=4 SV=3 - [D3YUD8_MOUSE]	10.68	2	3	3	9	-0.38	-0.14	-0.26	0.08
Q9CX60	Protein LBH OS=Mus musculus GN=Lbh PE=1 SV=1 - [LBH_MOUSE]	36.19	1	3	3	19	0.26	0.08	0.16	-0.04
Q8BS30	Protein Svs2 OS=Mus musculus GN=Svs2 PE=2 SV=1 - [Q8BS30_MOUSE]	2.93	1	1	1	1	-0.60	-0.52	-0.11	-0.03
Q9Z2X8	Kelch-like ECH-associated protein 1 OS=Mus musculus GN=Keap1 PE=1 SV=1 - [KEAP1_MOUSE]	5.93	1	3	3	5	0.04	-0.35	0.30	-0.09
Q9CY66-3	Isoform 3 of H/ACA ribonucleoprotein complex subunit 1 OS=Mus musculus GN=Gar1 - [GAR1_MOUSE]	26.27	2	1	6	13	-0.33	-0.24	-0.58	-0.49
P47941	Crk-like protein OS=Mus musculus GN=Crkl PE=1 SV=2 - [CRKL_MOUSE]	30.03	1	7	7	42	-0.30	-0.22	-0.44	-0.05
Q9CPN8	Insulin-like growth factor 2 mRNA-binding protein 3 OS=Mus musculus GN=Igf2bp3 PE=1 SV=1 - [IF2B3_MOUSE]	3.63	1	1	2	3	0.47	0.38	-0.05	-0.13
H9H9T1	Protein FAM107B OS=Mus musculus GN=Fam107b PE=2 SV=1 - [H9H9T1_MOUSE]	50.98	5	7	7	17	0.07	-0.10	-0.07	-0.21

P33610	DNA primase large subunit OS=Mus musculus GN=Prim2 PE=1 SV=1 - [PRI2_MOUSE]	5.54	1	3	3	7	0.27	0.07	0.11	-0.09
Q91YA2	Serine/threonine-protein kinase H1 OS=Mus musculus GN=Pskh1 PE=2 SV=3 - [KPSH1_MOUSE]	1.89	1	1	1	1	0.08	-0.81	0.06	-0.82
E9PV26	Protein BC068157 OS=Mus musculus GN=BC068157 PE=2 SV=1 - [E9PV26_MOUSE]	1.28	1	1	1	1	-1.47	-2.04	-0.39	-0.96
Q61493	DNA polymerase zeta catalytic subunit OS=Mus musculus GN=Rev3l PE=1 SV=3 [DPOLZ_MOUSE]	2.15	3	3	4	7	0.59	0.44	0.28	0.25
Q9Z0J0	Epididymal secretory protein E1 OS=Mus musculus GN=Npc2 PE=2 SV=1 - [NPC2_MOUSE]	42.28	1	8	8	87	-0.06	0.03	-0.11	0.00
P62889	60S ribosomal protein L30 OS=Mus musculus GN=Rpl30 PE=2 SV=2 [RL30_MOUSE]	47.83	1	8	8	71	0.00	-0.04	-0.13	-0.08
Q99JP4	Anaphase-promoting complex subunit CDC26 OS=Mus musculus GN=Cdc26 PE=2 SV=1 - [CDC26_MOUSE]	41.18	1	2	2	8	0.04	-0.26	-0.23	-0.52
Q9JLQ0	CD2-associated protein OS=Mus musculus GN=Cd2ap PE=1 SV=3 - [CD2AP_MOUSE]	45.37	1	23	23	89	-0.22	-0.16	-0.25	-0.16
P29533	Vascular cell adhesion protein 1 OS=Mus musculus GN=Vcam1 PE=1 SV=1 - [VCAM1_MOUSE]	2.44	2	2	2	2	0.12	-0.18	0.20	-0.10
E9PXB7	E3 ubiquitin-protein ligase NEDD4-like OS=Mus musculus GN=Nedd4l PE=2 SV=1 - [E9PXB7_MOUSE]	15.27	5	9	12	36	-0.18	-0.04	-0.31	-0.11
O55234	Proteasome subunit beta type-5 OS=Mus musculus GN=Psmb5 PE=1 SV=3 - [PSB5_MOUSE]	41.67	1	10	10	39	-0.30	-0.31	-0.15	-0.25
B1ASB6	Protein Spocd1 OS=Mus musculus GN=Spocd1 PE=2 SV=1 - [B1ASB6_MOUSE]	2.96	1	1	1	1	-0.39	0.28	-0.65	0.02
Q91VN6	Probable ATP-dependent RNA helicase DDX41 OS=Mus musculus GN=Ddx41 PE=1 SV=2 - [DDX41_MOUSE]	1.93	1	1	1	2	0.42	0.71	0.15	0.44
Q99KC8	von Willebrand factor A domain-containing protein 5A OS=Mus musculus GN=Vwa5a PE=1 SV=2 - [VMA5A_MOUSE]	39.85	2	23	26	237	-0.10	-0.10	-0.11	-0.09
O88492	Perilipin-4 OS=Mus musculus GN=Plin4 PE=1 SV=2 - [PLIN4_MOUSE]	60.94	2	29	29	156	-0.30	-0.19	-0.32	-0.23

Q8CHP5-2	Isoform 2 of Partner of Y14 and mago OS=Mus musculus GN=Wibg - [WIBG_MOUSE]	38.12	2	3	3	11	0.07	0.14	-0.03	0.03
F6QRE9	Protein BC007180 (Fragment) OS=Mus musculus GN=BC007180 PE=4 SV=2 - [F6QRE9_MOUSE]	1.12	1	1	1	2	-0.33	0.11	-0.20	0.25
Q8R4P9-2	Isoform 2 of Multidrug resistance-associated protein 7 OS=Mus musculus GN=Abcc10 - [MRP7_MOUSE]	1.71	3	2	2	2	-0.41	-0.20	-0.68	-0.47
O09174	Alpha-methylacyl-CoA racemase OS=Mus musculus GN=Amacr PE=1 SV=4 - [AMACR_MOUSE]	8.40	1	2	2	8	-0.25	-0.49	-0.15	-0.39
G3UYA1	DENN domain-containing protein 3 OS=Mus musculus GN=Dennd3 PE=2 SV=1 - [G3UYA1_MOUSE]	1.49	3	1	2	4	-1.39	-4.39	-0.95	-3.95
Q99LC5	Electron transfer flavoprotein subunit alpha, mitochondrial OS=Mus musculus GN=Etfa PE=1 SV=2 - [ETFA_MOUSE]	57.06	1	18	18	293	-0.26	-0.16	-0.40	-0.26
A2AGL3-2	Isoform 2 of Ryanodine receptor 3 OS=Mus musculus GN=Ryr3 - [RYR3_MOUSE]	1.47	3	4	5	8	-0.10	-0.57	-0.07	-0.48
Q99JY8	Lipid phosphate phosphohydrolase 3 OS=Mus musculus GN=Ppap2b PE=1 SV=1 - [LPP3_MOUSE]	3.53	1	1	1	11	-0.82	-0.88	-0.88	-0.94
Q8CII2	Cell division cycle protein 123 homolog OS=Mus musculus GN=Cdc123 PE=2 SV=2 - [CD123_MOUSE]	15.18	2	3	4	62	-0.05	-0.20	0.08	0.21
Q9ET26	RING finger protein 114 OS=Mus musculus GN=Rnf114 PE=2 SV=2 - [RN114_MOUSE]	15.72	1	4	4	8	-0.21	-0.68	-0.02	-0.48
Q8K0B3	Leucine-rich repeat-containing protein 66 OS=Mus musculus GN=Lrrc66 PE=2 SV=1 - [LRC66_MOUSE]	1.95	1	1	1	1	-0.04	0.25	-0.08	0.20
Q810D6	Glutamate-rich WD repeat-containing protein 1 OS=Mus musculus GN=Grwd1 PE=2 SV=2 - [GRWD1_MOUSE]	15.92	1	4	4	18	0.40	0.35	0.44	0.20
Q9R0P9	Ubiquitin carboxyl-terminal hydrolase isozyme L1 OS=Mus musculus GN=Uchl1 PE=1 SV=1 - [UCHL1_MOUSE]	30.49	1	4	5	94	-0.22	-0.11	-0.41	-0.28
H7BWX9	Small ubiquitin-related modifier 2 OS=Mus musculus GN=Sumo2 PE=2 SV=1 - [H7BWX9_MOUSE]	41.51	4	1	2	28	-0.03	0.06	-0.15	-0.07

Q8K0G5	Protein TSSC1 OS=Mus musculus GN=Tssc1 PE=1 SV=2 - [TSSC1_MOUSE]	3.37	1	1	1	10	-0.01	-0.19	-0.22	-0.38
Q3V1L4	Cytosolic purine 5'-nucleotidase OS=Mus musculus GN=Nt5c2 PE=1 SV=2 - [5NTC_MOUSE]	20.89	3	7	7	16	-0.44	0.03	-0.27	-0.21
Q63871	DNA-directed RNA polymerases I, II, and III subunit RPABC4 OS=Mus musculus GN=Polr2k PE=2 SV=2 - [RPAB4_MOUSE]	12.07	2	1	1	3	-0.14	-0.04	-0.17	-0.04
P16014	Secretogranin-1 OS=Mus musculus GN=Chgb PE=1 SV=2 - [SCG1_MOUSE]	23.49	1	15	15	71	-1.00	-0.77	-1.09	-0.76
P61028	Ras-related protein Rab-8B OS=Mus musculus GN=Rab8b PE=1 SV=1 - [RAB8B_MOUSE]	48.31	13	4	11	80	-0.19	-0.15	-0.19	-0.28
Q9CR39	WD repeat domain phosphoinositide-interacting protein 3 OS=Mus musculus GN=Wdr45b PE=2 SV=2 - [WIPI3_MOUSE]	5.23	1	2	2	2	-0.20	0.05	-0.31	-0.06
Q8CI61	BAG family molecular chaperone regulator 4 OS=Mus musculus GN=Bag4 PE=1 SV=2 - [BAG4_MOUSE]	11.38	1	4	4	7	-0.41	0.34	-0.47	0.28
Q9D2V7	Coronin-7 OS=Mus musculus GN=Coro7 PE=2 SV=2 - [CORO7_MOUSE]	40.56	3	25	25	120	-0.34	-0.28	-0.24	-0.25
F6TFN2	Protein Lmo7 (Fragment) OS=Mus musculus GN=Lmo7 PE=4 SV=1 - [F6TFN2_MOUSE]	33.14	6	30	30	130	-0.01	0.00	-0.02	0.02
P26043	Radixin OS=Mus musculus GN=Rdx PE=1 SV=3 - [RADI_MOUSE]	41.85	2	14	26	243	-0.32	-0.25	-0.29	-0.22
B1AXZ0	ELAV-like protein 2 OS=Mus musculus GN=Elavl2 PE=2 SV=1 - [B1AXZ0_MOUSE]	5.20	18	2	2	8	-0.45	-0.51	-0.52	-0.52
Q8C5H8-2	Isoform 2 of NAD kinase 2, mitochondrial OS=Mus musculus GN=Nadk2 - [NAKD2_MOUSE]	12.73	4	4	4	9	-0.18	-0.60	-0.04	-0.47
Q91V76	Ester hydrolase C11orf54 homolog OS=Mus musculus PE=2 SV=1 - [CK054_MOUSE]	37.46	1	10	10	85	-1.06	-1.02	-1.08	-1.04
Q91YY4	ATP synthase mitochondrial F1 complex assembly factor 2 OS=Mus musculus GN=Atpaf2 PE=2 SV=1 - [ATPF2_MOUSE]	7.61	1	2	2	6	0.12	-0.01	0.17	-0.04
Q9CQR2	40S ribosomal protein S21 OS=Mus musculus GN=Rps21 PE=2 SV=1 - [RS21_MOUSE]	45.78	1	5	5	95	-0.05	-0.03	-0.10	-0.04
O70456	14-3-3 protein sigma OS=Mus musculus GN=Sfn PE=1 SV=2 - [1433S_MOUSE]	59.27	1	10	14	317	0.02	0.04	-0.05	-0.02

P30681	High mobility group protein B2 OS=Mus musculus GN=Hmgb2 PE=1 SV=3 - [HMGB2_MOUSE]	38.57	1	13	15	209	0.54	0.51	0.46	0.50
Q9R0E1	Procollagen-lysine,2-oxoglutarate 5-dioxygenase 3 OS=Mus musculus GN=Plod3 PE=1 SV=1 - [PLOD3_MOUSE]	14.30	2	9	9	26	-0.46	-0.53	-0.22	-0.35
P51658	Estradiol 17-beta-dehydrogenase 2 OS=Mus musculus GN=Hsd17b2 PE=2 SV=2 - [DHB2_MOUSE]	19.42	1	7	7	27	-0.38	-0.39	-0.47	-0.27
Q8BGL5	Protein C730034F03Rik OS=Mus musculus GN=C730034F03Rik PE=4 SV=1 - [Q8BGL5_MOUSE]	9.91	1	1	1	1	-0.74	-0.87	-0.61	-0.75
Q8CEC6	Peptidylprolyl isomerase domain and WD repeat-containing protein 1 OS=Mus musculus GN=Ppwd1 PE=2 SV=2 - [PPWD1_MOUSE]	9.60	1	6	6	8	-0.16	0.12	0.09	0.08
Q62230	Sialoadhesin OS=Mus musculus GN=Siglec1 PE=1 SV=2 - [SN_MOUSE]	7.79	6	10	10	31	-0.36	-0.47	-0.04	-0.32
D3YYD5	Vacuolar protein sorting associated protein 29 (Fragment) OS=Mus musculus GN=Vps29 PE=2 SV=1 - [D3YYD5_MOUSE]	41.13	5	6	6	31	-0.25	-0.10	-0.34	-0.17
P01867-2	Isoform 2 of Ig gamma-2B chain C region OS=Mus musculus GN=Igh-3 - [IGG2B_MOUSE]	15.52	2	4	4	27	-0.27	-0.43	-0.58	-0.57
Q9D975	Sulfiredoxin-1 OS=Mus musculus GN=Srxn1 PE=2 SV=1 - [SRXN1_MOUSE]	14.71	2	2	2	2	-0.35	-0.87	-0.47	-0.99
Q9CQQ8	U6 snRNA-associated Sm-like protein LSm7 OS=Mus musculus GN=Lsm7 PE=3 SV=1 [LSM7_MOUSE]	32.04	1	3	3	26	-0.20	-0.07	-0.18	-0.05
Q61074	Protein phosphatase 1G OS=Mus musculus GN=Ppm1g PE=2 SV=3 - [PPM1G_MOUSE]	27.49	1	13	13	48	-0.07	-0.01	-0.08	-0.11
A2A4L0	Pleckstrin homology domain-containing family H member 3 (Fragment) OS=Mus musculus GN=Plekhh3 PE=2 SV=1 - [A2A4L0_MOUSE]	6.88	5	1	1	5	-0.36	-0.28	-0.33	-0.25
Q8BHB0	Nucleotide-binding oligomerization domain-containing protein 1 OS=Mus musculus GN=Nod1 PE=2 SV=1 - [NOD1_MOUSE]	3.25	1	2	2	2	-0.50	-0.67	-0.49	-0.66
Q99N69	Leupaxin OS=Mus musculus GN=Lpxn PE=1 SV=2 - [LPXN_MOUSE]	1.81	1	1	1	2	0.07	0.09	-0.12	-0.11

P28063	Proteasome subunit beta type-8 OS=Mus musculus GN=Psmb8 PE=1 SV=2 - [PSB8_MOUSE]	37.68	2	8	8	80	-0.27	-0.29	-0.29	-0.26
P11438	Lysosome-associated membrane glycoprotein 1 OS=Mus musculus GN=Lamp1 PE=1 SV=2 - [LAMP1_MOUSE]	21.43	1	5	5	44	-0.29	-0.17	-0.17	-0.07
Q60692	Proteasome subunit beta type-6 OS=Mus musculus GN=Psmb6 PE=1 SV=3 - [PSB6_MOUSE]	32.35	1	4	4	44	-0.32	-0.30	-0.20	-0.27
Q5RIM6	Nuclear receptor co-repressor 1, isoform CRA_a OS=Mus musculus GN=Ncor1 PE=4 SV=1 - [Q5RIM6_MOUSE]	3.71	11	5	8	13	-0.08	0.05	0.01	0.14
Q9CQW9	Interferon-induced transmembrane protein 3 OS=Mus musculus GN=Ifitm3 PE=1 SV=1 [IFM3_MOUSE]	32.12	3	3	3	14	-0.36	-0.45	-0.19	-0.16
A2A5R8	Double-stranded RNA-binding protein Staufen homolog 1 OS=Mus musculus GN=Stau1 PE=2 SV=1 - [A2A5R8_MOUSE]	20.62	4	9	9	26	0.08	-0.10	-0.10	-0.22
Q9JJU8	SH3 domain-binding glutamic acid-rich-like protein OS=Mus musculus GN=Sh3bgrl PE=3 SV=1 - [SH3L1_MOUSE]	75.44	1	7	7	251	-0.04	0.01	-0.18	-0.11
J3QP80	MCG15559 OS=Mus musculus GN=Gm5814 PE=4 SV=1 - [J3QP80_MOUSE]	24.80	2	2	2	11	0.01	0.09	-0.23	-0.14
Q9DCJ9	N-acetylneuraminate lyase OS=Mus musculus GN=Npl PE=1 SV=1 - [NPL_MOUSE]	41.56	1	10	10	26	0.23	0.34	0.26	0.19
Q9ER73	Elongator complex protein 4 OS=Mus musculus GN=Elp4 PE=2 SV=2 - [ELP4_MOUSE]	12.32	2	4	4	12	0.33	0.30	0.33	0.11
Q811J3	Iron-responsive element binding protein 2 OS=Mus musculus GN=Ireb2 PE=2 SV=2 - [IREB2_MOUSE]	7.68	1	4	5	12	0.25	0.11	0.53	0.39
B1AR09	Protein Mllt6 OS=Mus musculus GN=Mllt6 PE=2 SV=1 - [B1AR09_MOUSE]	5.38	2	2	2	8	-0.65	-0.55	-0.86	-0.68
P56183-2	Isoform 2 of Ribosomal RNA processing protein 1 homolog A OS=Mus musculus GN=Rrp1 - [RRP1_MOUSE]	11.74	3	3	3	4	-0.35	-0.20	0.13	0.28
Q9D4D5	Protein 4933414I15Rik OS=Mus musculus GN=4933414I15Rik PE=2 SV=1 - [Q9D4D5_MOUSE]	13.53	1	1	1	2	-0.79	-0.69	-0.93	-0.84

P59999	Actin-related protein 2/3 complex subunit 4 OS=Mus musculus GN=Arpc4 PE=1 SV=3 - [ARPC4_MOUSE]	51.19	2	8	8	151	-0.17	-0.08	-0.15	-0.07
E9Q6H8	Protein Plekha5 OS=Mus musculus GN=Plekha5 PE=2 SV=1 - [E9Q6H8_MOUSE]	7.09	1	8	8	22	0.03	-0.10	-0.16	-0.17
E9Q743	Protein Wdr66 OS=Mus musculus GN=Wdr66 PE=2 SV=1 - [E9Q743_MOUSE]	4.16	4	2	2	9	-0.26	-0.50	-0.19	-0.43
P61514	60S ribosomal protein L37a OS=Mus musculus GN=Rpl37a PE=2 SV=2 - [RL37A_MOUSE]	36.96	1	5	5	12	0.06	0.39	-0.13	-0.02
Q3UFM5	Nucleolar MIF4G domain-containing protein 1 OS=Mus musculus GN=Nom1 PE=2 SV=2 - [NOM1_MOUSE]	1.64	1	1	1	2	0.16	-0.07	-0.10	-0.33
Q1ERP8-4	Isoform 4 of CMRF35-like molecule 9 OS=Mus musculus GN=Cd300lg - [CLM9_MOUSE]	5.49	5	1	1	2	-0.39	-0.46	-0.87	-0.95
Q8C9S4	Uncharacterized protein C10orf118 homolog OS=Mus musculus GN=Otg1 PE=2 SV=2 - [CJ118_MOUSE]	6.43	1	5	5	15	-0.26	-0.11	-0.40	-0.31
P19001	Keratin, type I cytoskeletal 19 OS=Mus musculus GN=Krt19 PE=1 SV=1 [K1C19_MOUSE]	80.40	5	27	39	1525	-0.30	-0.26	-0.29	-0.24
E9QMK9	Protein 9030617O03Rik OS=Mus musculus GN=9030617O03Rik PE=2 SV=1 - [E9QMK9_MOUSE]	15.46	3	8	8	13	-1.04	-0.93	-0.88	-0.75
P47968	Ribose-5-phosphate isomerase OS=Mus musculus GN=Rpia PE=2 SV=2 - [RPIA_MOUSE]	41.91	1	11	11	50	-0.13	-0.20	-0.15	-0.28
Q501J7-2	Isoform 2 of Phosphatase and actin regulator 4 OS=Mus musculus GN=Phactr4 - [PHAR4_MOUSE]	11.84	4	4	6	16	-0.07	-0.44	-0.06	-0.19
Q8R086	Sulfite oxidase, mitochondrial OS=Mus musculus GN=Suox PE=1 SV=2 - [SUOX_MOUSE]	10.07	1	4	4	7	-0.13	-0.12	-0.46	-0.45
O35972	39S ribosomal protein L23, mitochondrial OS=Mus musculus GN=Mrpl23 PE=2 SV=1 - [RM23_MOUSE]	8.90	1	1	1	3	-0.84	-0.25	-1.08	-0.48
Q9D113	DNL-type zinc finger protein OS=Mus musculus GN=Dnlz PE=2 SV=1 - [DNLZ_MOUSE]	7.34	1	1	1	2	-0.29	-0.33	-0.53	-0.57
P97855	Ras GTPase-activating protein-binding protein 1 OS=Mus musculus GN=G3bp1 PE=1 SV=1 - [G3BP1_MOUSE]	29.89	1	10	11	95	0.23	0.10	0.02	0.02

Q8CBY0	Glutamyl-tRNA(Gln) amidotransferase subunit C, mitochondrial OS=Mus musculus GN=Gatc PE=2 SV=1 - [GATC_MOUSE]	24.52	1	2	2	13	0.38	-0.21	0.11	-0.49
P19467	Mucin-13 OS=Mus musculus GN=Muc13 PE=2 SV=1 - [MUC13_MOUSE]	17.28	1	8	8	166	0.24	0.24	0.20	0.21
Q8VEB4	Group XV phospholipase A2 OS=Mus musculus GN=Pla2g15 PE=1 SV=1 - [PAG15_MOUSE]	9.71	1	3	3	6	-0.53	-0.45	-0.30	-0.39
A2AQ19	RNA polymerase-associated protein RTF1 homolog OS=Mus musculus GN=Rtf1 PE=2 SV=1 - [RTF1_MOUSE]	8.95	1	6	7	12	-0.03	0.03	-0.12	-0.02
P08122	Collagen alpha-2(IV) chain OS=Mus musculus GN=Col4a2 PE=2 SV=4 - [CO4A2_MOUSE]	10.54	2	10	11	89	-1.41	-1.43	-1.36	-1.11
Q6DFV3	Rho GTPase-activating protein 21 OS=Mus musculus GN=Arhgap21 PE=1 SV=1 - [RHG21_MOUSE]	2.47	4	3	4	7	-0.08	-0.38	-0.30	-0.37
Q9CQT5	Proteasome maturation protein OS=Mus musculus GN=Pomp PE=2 SV=1 - [POMP_MOUSE]	15.60	1	2	2	5	0.04	-0.54	0.08	-0.49
P11679	Keratin, type II cytoskeletal 8 OS=Mus musculus GN=Krt8 PE=1 SV=4 - [K2C8_MOUSE]	66.12	2	34	44	2299	-0.09	-0.05	-0.10	-0.06
Q3TIX9	U4/U6.U5 tri-snRNP-associated protein 2 OS=Mus musculus GN=Usp39 PE=2 SV=2 - [SNUT2_MOUSE]	11.17	1	6	6	11	-0.06	0.08	-0.07	-0.11
P68040	Guanine nucleotide-binding protein subunit beta-2-like 1 OS=Mus musculus GN=Gnb2l1 PE=1 SV=3 - [GBLP_MOUSE]	60.57	1	17	17	490	-0.15	-0.05	-0.13	-0.13
Q9CQF7	Prefoldin 1 OS=Mus musculus GN=Pfdn1 PE=2 SV=1 - [Q9CQF7_MOUSE]	42.62	2	4	4	25	-0.05	-0.01	-0.14	-0.11
P17563	Selenium-binding protein 1 OS=Mus musculus GN=Selenbp1 PE=1 SV=2 - [SBP1_MOUSE]	67.58	4	10	26	1066	-0.84	-0.78	-0.76	-0.72
Q61672	Equilibrative nucleoside transporter 2 OS=Mus musculus GN=Slc29a2 PE=1 SV=2 - [S29A2_MOUSE]	2.63	1	1	1	1	0.00	0.53	-0.04	0.48
Q9CQN7	39S ribosomal protein L41, mitochondrial OS=Mus musculus GN=Mrpl41 PE=2 SV=1 - [RM41_MOUSE]	34.81	2	4	4	15	0.09	-0.11	-0.12	0.02
P49935	Pro-cathepsin H OS=Mus musculus GN=Ctsh PE=2 SV=2 - [CATH_MOUSE]	32.73	2	6	6	48	-0.04	-0.07	-0.01	0.11

P07356	Annexin A2 OS=Mus musculus GN=Anxa2 PE=1 SV=2 - [ANXA2_MOUSE]	64.60	4	24	24	900	-0.08	-0.02	-0.13	-0.09
Q9D1H9	Microfibril-associated glycoprotein 4 OS=Mus musculus GN=Mfap4 PE=1 SV=1 - [MFAP4_MOUSE]	2.72	2	1	1	2	-0.55	-0.51	-0.65	-0.62
Q8C570	mRNA export factor OS=Mus musculus GN=Rae1 PE=1 SV=1 - [RAE1L_MOUSE]	35.33	2	12	12	58	-0.16	-0.10	-0.13	-0.07
P99029-2	Isoform Cytoplasmic+peroxisomal of Peroxiredoxin-5, mitochondrial OS=Mus musculus GN=Prdx5 - [PRDX5_MOUSE]	49.38	4	9	9	224	-0.29	-0.16	-0.33	-0.22
Q60973	Histone-binding protein RBBP7 OS=Mus musculus GN=Rbbp7 PE=1 SV=1 - [RBBP7_MOUSE]	54.82	5	9	15	72	0.14	0.15	-0.04	0.03
B0QZL2	Nicotinamide mononucleotide adenylyltransferase 1 OS=Mus musculus GN=Nmnat1 PE=2 SV=1 - [B0QZL2_MOUSE]	7.38	2	1	1	1	-0.83	-0.56	-0.51	-0.24
D5MCW4	Protein CutA OS=Mus musculus GN=Cuta PE=2 SV=1 - [D5MCW4_MOUSE]	7.79	2	1	1	1	-0.12	-0.02	-0.28	-0.18
Q9D8C9	Purine nucleoside phosphorylase OS=Mus musculus GN=Pnp2 PE=2 SV=1 - [Q9D8C9_MOUSE]	37.38	1	5	8	97	-0.25	-0.27	-0.24	-0.29
Q80W47	WD repeat domain phosphoinositide-interacting protein 2 OS=Mus musculus GN=Wipi2 PE=1 SV=1 [WIPI2_MOUSE]	7.87	2	3	3	5	0.06	0.15	-0.60	-0.51
Q9CWS0	N(G),N(G)-dimethylarginine dimethylaminohydrolase 1 OS=Mus musculus GN=Ddah1 PE=1 SV=3 - [DDAH1_MOUSE]	45.96	2	10	11	105	-0.38	-0.42	-0.44	-0.36
Q80U87	Ubiquitin carboxyl-terminal hydrolase 8 OS=Mus musculus GN=Usp8 PE=1 SV=2 - [UBP8_MOUSE]	6.39	2	5	5	19	-0.20	-0.09	0.02	-0.10
B2RS91	RNA polymerase I-specific transcription initiation factor RRN3 OS=Mus musculus GN=Rrn3 PE=1 SV=1 - [RRN3_MOUSE]	1.37	1	1	1	2	-0.16	-0.19	-0.24	-0.27
P27661	Histone H2A.x OS=Mus musculus GN=H2afx PE=1 SV=2 - [H2AX_MOUSE]	26.57	3	3	7	50	-0.30	-0.08	-0.47	-0.24
E9Q7C4	Protein Prr14l OS=Mus musculus GN=Prr14l PE=2 SV=1 - [E9Q7C4_MOUSE]	2.18	2	1	2	3	0.05	-0.28	-0.02	-0.35

Q6P2L6	Histone-lysine N-methyltransferase NSD3 OS=Mus musculus GN=Whsc1l1 PE=1 SV=2 - [NSD3_MOUSE]	4.17	12	4	6	7	-0.33	-0.43	-0.13	-0.37
Q9DAU1	Protein canopy homolog 3 OS=Mus musculus GN=Cnpy3 PE=1 SV=1 - [CNPY3_MOUSE]	23.91	3	6	6	10	-0.04	-0.06	-0.20	-0.19
P28076	Proteasome subunit beta type-9 OS=Mus musculus GN=Psmb9 PE=1 SV=1 - [PSB9_MOUSE]	19.18	1	3	3	18	-0.49	-0.54	-0.61	-0.68
Q9QYR9	Acyl-coenzyme A thioesterase 2, mitochondrial OS=Mus musculus GN=Acot2 PE=1 SV=2 - [ACOT2_MOUSE]	24.94	5	3	8	31	-0.32	-0.64	-0.26	-0.45
Q9Z0F4	Calcium and integrin-binding protein 1 OS=Mus musculus GN=Cib1 PE=1 SV=3 - [CIB1_MOUSE]	25.13	3	4	4	14	-0.16	-0.33	-0.11	-0.12
Q8R2M2	Deoxynucleotidyltransferase terminal-interacting protein 2 OS=Mus musculus GN=Dnttip2 PE=1 SV=1 - [TDIF2_MOUSE]	3.43	1	2	3	6	-0.18	-0.56	-0.18	-0.55
Q9CQV1	Mitochondrial import inner membrane translocase subunit TIM16 OS=Mus musculus GN=Pam16 PE=2 SV=1 - [TIM16_MOUSE]	35.20	1	2	3	8	0.11	-0.12	-0.12	0.41
E9Q1Q0	Protein Slc4a1ap OS=Mus musculus GN=Slc4a1ap PE=2 SV=1 - [E9Q1Q0_MOUSE]	5.06	3	2	2	3	1.76	1.46	0.82	0.52
Q3ZK22	Vezatin OS=Mus musculus GN=Vezt PE=1 SV=2 - [VEZA_MOUSE]	2.05	2	1	1	2	-0.25	-0.29	-0.03	-0.06
E9Q9C7	Actin-binding LIM protein 1 OS=Mus musculus GN=Ablim1 PE=2 SV=1 - [E9Q9C7_MOUSE]	14.07	16	6	7	24	-0.49	-0.55	-0.34	-0.32
P84089	Enhancer of rudimentary homolog OS=Mus musculus GN=Erh PE=1 SV=1 - [ERH_MOUSE]	34.62	3	4	4	45	-0.14	-0.06	-0.25	-0.15
P21619	Lamin-B2 OS=Mus musculus GN=Lmnb2 PE=1 SV=2 - [LMNB2_MOUSE]	51.51	2	23	28	185	-0.51	-0.46	-0.50	-0.46
Q8BUY9	Geranylgeranyl transferase type-1 subunit beta OS=Mus musculus GN=Pggt1b PE=2 SV=1 - [PGTB1_MOUSE]	4.24	1	2	2	4	-0.74	-0.96	-0.56	-0.78
Q6PEV3	WAS/WASL-interacting protein family member 2 OS=Mus musculus GN=Wipf2 PE=2 SV=1 - [WIPF2_MOUSE]	21.14	2	7	7	23	-0.23	-0.17	-0.51	-0.32
O70326	Gremlin-1 OS=Mus musculus GN=Grem1 PE=2 SV=1 - [GREM1_MOUSE]	6.52	1	1	1	1	0.41	0.38	0.55	0.51

Q8C4Y1	Max protein OS=Mus musculus GN=Max PE=2 SV=1 - [Q8C4Y1_MOUSE]	19.21	3	3	3	10	-0.19	-0.07	-0.16	-0.19
G5E850	Cytochrome b-5, isoform CRA_a OS=Mus musculus GN=Cyb5 PE=3 SV=1 - [G5E850_MOUSE]	40.82	3	4	4	83	-0.34	-0.30	-0.34	-0.33
Q9WVJ3-2	Isoform 2 of Carboxypeptidase Q OS=Mus musculus GN=Cpq - [CBPQ_MOUSE]	17.47	2	6	6	32	-0.79	-0.53	-0.73	-0.87
Q8K297	Procollagen galactosyltransferase 1 OS=Mus musculus GN=Colgalt1 PE=1 SV=2 - [GT251_MOUSE]	16.69	1	9	9	52	-0.43	-0.35	-0.23	-0.37
Q6PI17	Metalloproteinase inhibitor 2 OS=Mus musculus GN=Timp2 PE=2 SV=1 - [Q6PI17_MOUSE]	20.91	3	2	2	3	-0.79	-0.45	-1.09	-0.75
P35831	Tyrosine-protein phosphatase non-receptor type 12 OS=Mus musculus GN=Ptpn12 PE=1 SV=3 - [PTN12_MOUSE]	5.81	1	3	3	6	0.25	0.36	0.11	0.22
P26041	Moesin OS=Mus musculus GN=Msn PE=1 SV=3 - [MOES_MOUSE]	50.09	1	26	37	603	0.19	0.17	0.11	0.09
Q6PIU9	Uncharacterized protein FLJ45252 homolog OS=Mus musculus PE=1 SV=2 - [YJ005_MOUSE]	11.86	1	3	3	7	-0.39	-0.33	-0.43	-0.32
Q9QUM9	Proteasome subunit alpha type-6 OS=Mus musculus GN=Psma6 PE=1 SV=1 - [PSA6_MOUSE]	50.81	3	13	13	121	-0.29	-0.34	-0.30	-0.25
Q9CWJ9	Bifunctional purine biosynthesis protein PURH OS=Mus musculus GN=Atic PE=1 SV=2 - [PUR9_MOUSE]	55.91	1	23	24	289	-0.12	-0.20	-0.12	-0.19
P97825	Hematological and neurological expressed 1 protein OS=Mus musculus GN=Hn1 PE=1 SV=3 - [HN1_MOUSE]	52.60	1	3	3	27	-0.20	-0.15	-0.37	-0.15
F6V5H4	Sushi, nidogen and EGF like domain-containing protein 1 (Fragment) OS=Mus musculus GN=Sned1 PE=4 SV=1 [F6V5H4_MOUSE]	6.46	3	1	1	1	-1.11	-0.71	-0.87	-0.48
Q91ZU6-8	Isoform 7 of Dystonin OS=Mus musculus GN=Dst - [DYST_MOUSE]	2.06	1	1	1	1	0.20	0.03	0.27	0.10
Q9R0E2	Procollagen-lysine,2-oxoglutarate 5-dioxygenase 1 OS=Mus musculus GN=Plod1 PE=1 SV=1 - [PLOD1_MOUSE]	5.22	3	3	3	3	-0.42	-0.46	-0.47	-0.51
Q8C0I1	Alkyldihydroxyacetone phosphate synthase, peroxisomal OS=Mus musculus GN=Agps PE=1 SV=1 - [ADAS_MOUSE]	22.02	5	9	10	33	-0.27	-0.28	-0.09	-0.32

Q8K1J5	Protein SDE2 homolog OS=Mus musculus GN=Sde2 PE=1 SV=1 - [SDE2_MOUSE]	4.02	1	2	2	4	-0.19	-0.09	-0.13	-0.03
Q62426	Cystatin-B OS=Mus musculus GN=Cstb PE=1 SV=1 - [CYTB_MOUSE]	48.98	1	4	4	45	-0.10	0.22	-0.18	0.17
D3Z041	Long-chain-fatty-acid--CoA ligase 1 OS=Mus musculus GN=Acsl1 PE=2 SV=1 - [D3Z041_MOUSE]	43.92	11	24	26	134	-0.87	-0.83	-0.92	-0.82
Q9CX86	Heterogeneous nuclear ribonucleoprotein A0 OS=Mus musculus GN=Hnrnpa0 PE=1 SV=1 - [ROA0_MOUSE]	38.69	1	6	8	61	0.38	0.36	0.32	0.15
Q9DBU5	E3 ubiquitin-protein ligase RNF6 OS=Mus musculus GN=Rnf6 PE=1 SV=1 - [RNF6_MOUSE]	10.04	4	2	2	2	-1.29	-1.52	-0.99	-1.22
E0CXN5	Glycerol-3-phosphate dehydrogenase [NAD(+)], cytoplasmic OS=Mus musculus GN=Gpd1 PE=2 SV=1 - [E0CXN5_MOUSE]	40.80	2	11	12	61	-1.15	-1.19	-1.11	-1.16
P21126	Ubiquitin-like protein 4A OS=Mus musculus GN=Ubl4a PE=2 SV=1 [UBL4A_MOUSE]	36.94	1	6	6	20	-0.11	-0.16	-0.05	-0.14
Q8C4X2	Casein kinase I isoform gamma-3 OS=Mus musculus GN=Csnk1g3 PE=1 SV=2 - [KC1G3_MOUSE]	5.42	3	2	2	3	0.23	0.32	0.18	0.27
Q91YP2	Neurolysin, mitochondrial OS=Mus musculus GN=Nln PE=2 SV=1 - [NEUL_MOUSE]	26.56	1	16	16	48	-0.21	-0.23	-0.21	-0.27
B8JK50	HMG box transcription factor BBX OS=Mus musculus GN=Bbx PE=2 SV=1 - [B8JK50_MOUSE]	1.91	5	1	1	1	-0.12	-0.40	-0.21	-0.49
O35226-2	Isoform Rpn10B of 26S proteasome non-ATPase regulatory subunit 4 OS=Mus musculus GN=Psmd4 - [PSMD4_MOUSE]	26.91	4	1	9	59	0.03	0.04	0.04	0.05
Q9WV98	Mitochondrial import inner membrane translocase subunit Tim9 OS=Mus musculus GN=Timm9 PE=1 SV=1 - [TIM9_MOUSE]	34.83	1	3	3	52	-0.58	-0.42	-0.42	-0.36
F8WHT2	Protein phosphatase Slingshot homolog 1 OS=Mus musculus GN=Ssh1 PE=2 SV=1 - [F8WHT2_MOUSE]	4.50	3	2	3	4	-0.43	-0.03	-0.78	-0.38
D3Z2X2	Protein Dnhd1 OS=Mus musculus GN=Dnhd1 PE=2 SV=2 - [D3Z2X2_MOUSE]	1.68	3	3	7	11	0.04	-0.38	-0.10	-0.52
Q60772	Cyclin-dependent kinase 4 inhibitor C OS=Mus musculus GN=Cdkn2c PE=2 SV=1 - [CDN2C_MOUSE]	21.43	1	4	4	9	-0.41	-0.46	-0.43	-0.48

P54869	Hydroxymethylglutaryl-CoA synthase, mitochondrial OS=Mus musculus GN=Hmgcs2 PE=1 SV=2 - [HMCS2_MOUSE]	50.98	1	26	27	663	-1.25	-1.08	-1.27	-1.14
Q5PRE5	Proline and serine-rich protein 1 OS=Mus musculus GN=Proser1 PE=2 SV=1 - [PRSR1_MOUSE]	1.10	1	1	1	1	-0.13	-0.79	-0.26	-0.93
Q8BQX0	RAB37, member of RAS oncogene family, isoform CRA_a OS=Mus musculus GN=Rab37 PE=2 SV=1 - [Q8BQX0_MOUSE]	9.72	13	1	2	28	-0.38	-0.62	-0.18	-0.42
Q5SSZ5	Tensin-3 OS=Mus musculus GN=Tns3 PE=1 SV=1 - [TENS3_MOUSE]	9.24	2	7	10	42	-0.04	0.04	-0.20	-0.30
G3X939	Sodium/hydrogen exchanger OS=Mus musculus GN=Slc9a3 PE=3 SV=1 - [G3X939_MOUSE]	5.19	1	3	3	5	-0.28	0.51	-0.29	0.27
Q3UD01	Putative ataxin-7-like protein 3B OS=Mus musculus GN=Atxn7l3b PE=3 SV=1 - [A7L3B_MOUSE]	60.82	1	3	3	8	-0.02	-0.02	-0.13	-0.08
Q91WQ9	Calmodulin-like protein 4 OS=Mus musculus GN=Calml4 PE=2 SV=2 - [CALL4_MOUSE]	49.02	4	8	8	31	-0.19	-0.01	-0.13	-0.06
Q8CFI2	Ubiquitin-conjugating enzyme E2 R1 OS=Mus musculus GN=Cdc34 PE=1 SV=1 - [UB2R1_MOUSE]	19.57	1	2	2	2	-0.38	-0.17	0.27	0.48
Q9CQX8	28S ribosomal protein S36, mitochondrial OS=Mus musculus GN=Mrps36 PE=1 SV=1 - [RT36_MOUSE]	70.59	4	5	5	87	-0.32	-0.20	-0.44	-0.29
B0R105	Proline-rich protein 5-like (Fragment) OS=Mus musculus GN=Prr5l PE=2 SV=1 - [B0R105_MOUSE]	17.89	3	1	1	3	-0.12	-0.37	-0.15	-0.40
Q6AXC6	Probable ATP-dependent RNA helicase DDX11 OS=Mus musculus GN=Ddx11 PE=2 SV=1 - [DDX11_MOUSE]	3.86	1	3	3	3	0.11	-0.70	-0.86	-1.23
Q8R2Z5	von Willebrand factor A domain-containing protein 1 OS=Mus musculus GN=Vwa1 PE=1 SV=1 - [VWA1_MOUSE]	16.63	1	3	3	13	-0.60	-0.74	-0.55	-0.64
Q6XLQ8	Calumenin OS=Mus musculus GN=Calu PE=2 SV=1 - [Q6XLQ8_MOUSE]	68.89	5	5	19	438	-0.03	0.07	-0.16	-0.06
P06151	L-lactate dehydrogenase A chain OS=Mus musculus GN=Ldha PE=1 SV=3 - [LDHA_MOUSE]	50.90	4	20	22	492	-0.11	-0.02	-0.15	-0.08
P62774	Myotrophin OS=Mus musculus GN=Mtpn PE=1 SV=2 - [MTPN_MOUSE]	55.08	1	6	6	56	0.04	0.12	-0.10	-0.07

P14873	Microtubule-associated protein 1B OS=Mus musculus GN=Map1b PE=1 SV=2 - [MAP1B_MOUSE]	14.33	1	25	26	68	-0.26	-0.22	-0.36	-0.24
Q8K411-2	Isoform 2 of Presequence protease, mitochondrial OS=Mus musculus GN=Pitrm1 - [PREP_MOUSE]	31.98	3	24	25	101	-0.31	-0.29	-0.36	-0.41
Q8K1I7	WAS/WASL-interacting protein family member 1 OS=Mus musculus GN=Wipf1 PE=1 SV=1 - [WIPF1_MOUSE]	13.18	1	5	5	29	-0.09	-0.02	-0.16	-0.20
Q9CWU9	Nucleoporin Nup37 OS=Mus musculus GN=Nup37 PE=2 SV=2 - [NUP37_MOUSE]	10.43	1	2	3	27	-0.12	0.14	-0.36	-0.09
P11032-2	Isoform HF2 of Granzyme A OS=Mus musculus GN=Gzma - [GRAA_MOUSE]	3.50	2	1	1	1	-0.37	-0.17	-0.71	-0.51
Q9ERV1	Probable E3 ubiquitin-protein ligase makorin-2 OS=Mus musculus GN=Mkrn2 PE=2 SV=2 - [MKRN2_MOUSE]	6.25	1	1	1	1	-0.18	0.18	-0.51	-0.16
P62257	Ubiquitin-conjugating enzyme E2 H OS=Mus musculus GN=Ube2h PE=2 SV=1 - [UBE2H_MOUSE]	26.78	4	4	4	10	0.18	0.07	0.08	0.02
Q9EPB4	Apoptosis-associated speck-like protein containing a CARD OS=Mus musculus GN=Pycard PE=1 SV=1 - [ASC_MOUSE]	60.62	1	9	9	327	-0.23	-0.19	-0.26	-0.23
Q9CQG1-2	Isoform 2 of Cation transport regulator-like protein 2 OS=Mus musculus GN=Chac2 - [CHAC2_MOUSE]	13.25	2	2	2	5	0.06	0.14	-0.11	-0.04
Q9ESY9	Gamma-interferon-inducible lysosomal thiol reductase OS=Mus musculus GN=Ifi30 PE=1 SV=3 - [GILT_MOUSE]	4.44	1	1	1	13	0.28	0.21	0.31	0.11
Q2KHI9	DNA helicase MCM9 OS=Mus musculus GN=Mcm9 PE=1 SV=2 - [MCM9_MOUSE]	3.09	3	2	2	5	-0.01	0.31	-0.07	0.19
Q80SZ7	Guanine nucleotide-binding protein G(I)/G(S)/G(O) subunit gamma-5 OS=Mus musculus GN=Gng5 PE=2 SV=2 - [GBG5_MOUSE]	32.35	2	3	3	21	-0.29	-0.22	-0.36	-0.34
D3Z3U6	Activated CDC42 kinase 1 OS=Mus musculus GN=Tnk2 PE=2 SV=1 - [D3Z3U6_MOUSE]	2.21	7	3	3	3	-0.70	-0.29	-0.57	-0.26
Q9EQ09	Oxidized low-density lipoprotein receptor 1 OS=Mus musculus GN=Olr1 PE=2 SV=2 - [OLR1_MOUSE]	8.26	1	1	3	3	0.19	0.32	0.06	0.19

E9PUD6	Protein Zscan18 OS=Mus musculus GN=Zscan18 PE=4 SV=1 - [E9PUD6_MOUSE]	2.22	1	1	1	1	0.17	0.69	-0.55	-0.03
P11276	Fibronectin OS=Mus musculus GN=Fn1 PE=1 SV=4 - [FINC_MOUSE]	33.02	1	59	59	533	-0.31	-0.23	-0.28	-0.21
Q91XV3	Brain acid soluble protein 1 OS=Mus musculus GN=Basp1 PE=1 SV=3 - [BASP1_MOUSE]	31.86	1	5	5	27	-0.48	-0.29	-0.75	-0.49
O88428	Bifunctional 3'-phosphoadenosine 5'-phosphosulfate synthase 2 OS=Mus musculus GN=Papss2 PE=1 SV=2 - [PAPS2_MOUSE]	46.05	1	22	24	214	-0.15	-0.13	-0.15	-0.07
Q9JMG7	Hepatoma-derived growth factor-related protein 3 OS=Mus musculus GN=Hdgfrp3 PE=1 SV=2 - [HDGR3_MOUSE]	23.76	2	4	5	41	0.39	0.47	0.37	0.40
P62488	DNA-directed RNA polymerase II subunit RPB7 OS=Mus musculus GN=Polr2g PE=2 SV=1 - [RPB7_MOUSE]	11.05	1	1	1	7	-0.52	-0.62	-0.42	-0.53
Q3UL36	Arginine and glutamate-rich protein 1 OS=Mus musculus GN=Arglu1 PE=1 SV=2 - [ARGL1_MOUSE]	16.61	3	5	7	16	0.23	0.09	0.20	0.31
P57016	Ladinin-1 OS=Mus musculus GN=Lad1 PE=1 SV=1 - [LAD1_MOUSE]	41.67	1	20	20	109	-0.40	-0.30	-0.41	-0.37
Q6NZB0	DnaJ homolog subfamily C member 8 OS=Mus musculus GN=Dnajc8 PE=2 SV=2 - [DNJC8_MOUSE]	43.08	8	9	9	54	-0.10	0.03	-0.09	0.02
Q9QZ85	Interferon-inducible GTPase 1 OS=Mus musculus GN=Iigp1 PE=1 SV=2 - [IIGP1_MOUSE]	33.90	1	9	10	64	0.26	0.14	0.27	0.23
Q9CQZ1	Heat shock factor-binding protein 1 OS=Mus musculus GN=Hsbp1 PE=2 SV=1 - [HSBP1_MOUSE]	40.79	1	2	3	15	-0.20	0.04	-0.45	-0.23
Q3UIL6-2	Isoform 2 of Pleckstrin homology domain-containing family A member 7 OS=Mus musculus GN=Plekha7 - [PKHA7_MOUSE]	15.40	6	14	14	30	-0.09	0.17	-0.11	-0.20
E9PYH3	Ethanolamine-phosphate phospho-lyase OS=Mus musculus GN=Agxt2l1 PE=2 SV=1 - [E9PYH3_MOUSE]	2.64	2	1	1	1	0.19	-0.30	-0.46	-0.96
P39061-2	Isoform 3 of Collagen alpha-1(XVIII) chain OS=Mus musculus GN=Col18a1 - [COIA1_MOUSE]	26.54	5	20	20	139	-0.26	-0.27	-0.27	-0.22
Q9DB96	Neuroguidin OS=Mus musculus GN=Ngdn PE=1 SV=1 - [NGDN_MOUSE]	6.98	1	2	2	4	-0.02	-0.07	0.00	-0.51

P23492	Purine nucleoside phosphorylase OS=Mus musculus GN=Pnp PE=1 SV=2 - [PNPH_MOUSE]	41.18	2	6	9	139	-0.45	-0.51	-0.16	-0.44
Q8BLV3-2	Isoform 2 of Sodium/hydrogen exchanger 7 OS=Mus musculus GN=Slc9a7 - [SL9A7_MOUSE]	2.01	2	1	1	1	-0.47	-0.57	-0.57	-0.67
F8WIX8	Histone H2A OS=Mus musculus GN=Hist1h2al PE=2 SV=1 - [F8WIX8_MOUSE]	39.20	12	2	6	106	-0.91	-0.86	-0.66	-0.47
Q8R1S0	Ubiquinone biosynthesis monooxygenase COQ6 OS=Mus musculus GN=Coq6 PE=2 SV=3 - [COQ6_MOUSE]	10.71	4	5	5	23	-0.16	-0.02	-0.21	-0.10
Q99L27	GMP reductase 2 OS=Mus musculus GN=Gmpr2 PE=2 SV=2 - [GMPR2_MOUSE]	11.78	1	2	5	9	-0.14	0.17	-0.27	-0.02
Q8R464	Cell adhesion molecule 4 OS=Mus musculus GN=Cadm4 PE=1 SV=1 - [CADM4_MOUSE]	4.64	1	2	2	3	-0.32	-0.53	-0.42	-0.40
P07607	Thymidylate synthase OS=Mus musculus GN=Tyms PE=1 SV=1 - [TYSY_MOUSE]	12.38	2	4	4	8	0.93	0.66	0.85	0.51
Q61937	Nucleophosmin OS=Mus musculus GN=Npm1 PE=1 SV=1 - [NPM_MOUSE]	56.51	5	18	18	305	0.04	0.10	0.00	0.05
Q9DC50	Peroxisomal carnitine O octanoyltransferase OS=Mus musculus GN=Crot PE=1 SV=1 - [OCTC_MOUSE]	22.22	1	12	12	51	-0.16	-0.18	-0.25	-0.24
Q8JZS0	Protein lin-7 homolog A OS=Mus musculus GN=Lin7a PE=1 SV=2 [LIN7A_MOUSE]	23.18	2	1	6	35	-0.12	-0.11	-0.14	-0.13
Q8JZM0	Dimethyladenosine transferase 1, mitochondrial OS=Mus musculus GN=Tfb1m PE=2 SV=1 - [TFB1M_MOUSE]	5.22	1	1	1	1	0.78	0.13	0.10	-0.54
E9QAP7	Protein Taf4a OS=Mus musculus GN=Taf4a PE=2 SV=1 - [E9QAP7_MOUSE]	3.07	2	3	3	4	-0.32	-0.39	-0.33	-0.39
Q542I8	Integrin beta OS=Mus musculus GN=Itgb2 PE=2 SV=1 - [Q542I8_MOUSE]	22.60	5	15	16	57	0.11	0.13	0.07	0.14
E9Q6U4	Protein Pfdn4 OS=Mus musculus GN=Pfdn4 PE=2 SV=1 - [E9Q6U4_MOUSE]	35.38	5	6	6	41	-0.11	-0.14	-0.15	-0.24
Q9CRA9-2	Isoform 2 of FGFR1 oncogene partner 2 homolog OS=Mus musculus GN=Fgfr1op2 - [FGOP2_MOUSE]	31.63	4	5	6	21	0.00	-0.28	-0.55	-0.83
Q8BL66	Early endosome antigen 1 OS=Mus musculus GN=Eea1 PE=1 SV=2 - [EEA1_MOUSE]	45.15	1	59	62	219	-0.22	-0.26	-0.21	-0.30

Q8JZQ5	Amiloride-sensitive amine oxidase [copper-containing] OS=Mus musculus GN=Abp1 PE=2 SV=1 - [ABP1_MOUSE]	14.51	2	9	9	27	-0.57	-0.73	-0.49	-0.58
Q3UFB2-2	Isoform 2 of Box C/D snoRNA protein 1 OS=Mus musculus GN=Znhit6 - [BCD1_MOUSE]	13.36	2	2	3	4	-0.48	-0.30	-0.62	-0.43
Q6P5H2-2	Isoform 2 of Nestin OS=Mus musculus GN=Nes - [NEST_MOUSE]	4.95	2	6	6	7	0.86	0.60	-0.67	-0.93
Q5SQX6	Cytoplasmic FMR1-interacting protein 2 OS=Mus musculus GN=Cyfip2 PE=1 SV=2 - [CYFP2_MOUSE]	5.11	2	1	7	20	-0.02	0.11	-0.13	-0.05
E9PXI8	Corticotropin-releasing factor receptor 2 OS=Mus musculus GN=Crhr2 PE=2 SV=1 [E9PXI8_MOUSE]	3.82	1	1	1	5	0.24	0.32	0.42	0.31
Q62241	U1 small nuclear ribonucleoprotein C OS=Mus musculus GN=Snrpc PE=2 SV=1 [RU1C_MOUSE]	13.21	1	2	2	27	-0.33	-0.25	-0.16	-0.07
P01868	Ig gamma-1 chain C region secreted form OS=Mus musculus GN=Ighg1 PE=1 SV=1 [IGHG1_MOUSE]	11.42	2	3	3	24	-0.66	-0.80	-0.75	-0.88
D3Z0A2	Protein arginine N-methyltransferase 1 OS=Mus musculus GN=Prmt1 PE=2 SV=1 [D3Z0A2_MOUSE]	36.79	7	10	10	40	-0.05	-0.23	-0.15	-0.35
P31809-3	Isoform 3 of Carcinoembryonic antigen-related cell adhesion molecule 1 OS=Mus musculus GN=Ceacam1 - [CEAM1_MOUSE]	12.61	8	4	4	135	0.20	0.25	0.11	0.14
Q9CXT8	Mitochondrial-processing peptidase subunit beta OS=Mus musculus GN=Pmpcb PE=2 SV=1 - [MPPB_MOUSE]	32.72	2	12	13	46	-0.14	-0.23	-0.13	-0.30
O35864	COP9 signalosome complex subunit 5 OS=Mus musculus GN=Cops5 PE=1 SV=3 - [CSN5_MOUSE]	12.28	1	3	3	17	0.12	0.41	0.10	0.29
Q8BU31	Ras-related protein Rap-2c OS=Mus musculus GN=Rap2c PE=1 SV=1 - [RAP2C_MOUSE]	26.23	2	1	3	9	-0.09	0.14	0.03	0.26
Q3UKC1	Tax1-binding protein 1 homolog OS=Mus musculus GN=Tax1bp1 PE=1 SV=2 - [TAXB1_MOUSE]	7.74	1	6	6	19	-0.04	0.15	-0.09	-0.06
Q922S8	Kinesin-like protein KIF2C OS=Mus musculus GN=Kif2c PE=1 SV=1 - [KIF2C_MOUSE]	3.19	2	2	2	3	-4.80	0.06	-4.07	0.80

Q64669	NAD(P)H dehydrogenase [quinone] 1 OS=Mus musculus GN=Nqo1 PE=1 SV=3 - [NQO1_MOUSE]	38.32	1	10	10	132	-0.44	-0.33	-0.45	-0.32
F7CXR1	Protein Gm9857 OS=Mus musculus GN=Gm9857 PE=2 SV=1 - [F7CXR1_MOUSE]	13.08	1	1	1	1	-0.30	-0.52	-0.35	-0.57
P08103-2	Isoform 2 of Tyrosine-protein kinase HCK OS=Mus musculus GN=Hck - [HCK_MOUSE]	10.74	35	2	6	35	-0.31	0.09	-0.50	0.02
Q9CXL3	Uncharacterized protein C7orf50 homolog OS=Mus musculus PE=2 SV=3 - [CG050_MOUSE]	13.85	1	1	1	3	0.47	0.63	0.00	0.17
A2AN84	55 kDa erythrocyte membrane protein OS=Mus musculus GN=Mpp1 PE=2 SV=1 [A2AN84_MOUSE]	17.26	7	6	6	14	0.00	0.18	-0.20	-0.02
Q8VHC3	Selenoprotein M OS=Mus musculus GN=Selm PE=1 SV=3 - [SELM_MOUSE]	49.66	1	4	4	63	-1.04	-1.15	-1.01	-1.13
P63028	Translationally-controlled tumor protein OS=Mus musculus GN=Tpt1 PE=1 SV=1 - [TCTP_MOUSE]	71.51	2	8	8	378	-0.08	-0.09	-0.08	-0.20
B1AU76	Nuclear autoantigenic sperm protein OS=Mus musculus GN=Nasp PE=2 SV=1 - [B1AU76_MOUSE]	43.97	9	14	16	98	-0.06	-0.04	-0.04	-0.02
P50543	Protein S100-A11 OS=Mus musculus GN=S100a11 PE=2 SV=1 - [S10AB_MOUSE]	39.80	1	4	4	116	-0.09	-0.15	-0.02	-0.11
P61982	14-3-3 protein gamma OS=Mus musculus GN=Ywhag PE=1 SV=2 - [1433G_MOUSE]	57.09	1	8	13	335	-0.27	-0.26	-0.29	-0.34
Q9CXS4	Centromere protein V OS=Mus musculus GN=Cenpv PE=2 SV=2 - [CENPV_MOUSE]	25.00	2	4	4	13	-0.68	-0.76	-0.59	-0.59
Q8BHN3	Neutral alpha-glucosidase AB OS=Mus musculus GN=Ganab PE=1 SV=1 - [GANAB_MOUSE]	42.48	2	1	28	265	0.17	0.93	-0.33	0.43
Q99LJ0	CTTNBP2 N-terminal-like protein OS=Mus musculus GN=Cttnbp2nl PE=1 SV=1 - [CT2NL_MOUSE]	11.44	1	6	6	11	0.25	0.38	0.20	0.28
Q3TB04	FERM domain-containing protein 4A OS=Mus musculus GN=Frmd4a PE=2 SV=1 - [Q3TB04_MOUSE]	8.41	4	2	2	4	-0.17	-0.15	-0.08	-0.43
Q64433	10 kDa heat shock protein, mitochondrial OS=Mus musculus GN=Hspe1 PE=1 SV=2 - [CH10_MOUSE]	53.92	1	6	6	252	-0.19	-0.07	-0.27	-0.15

D3Z5N2	Stathmin OS=Mus musculus GN=Stmn1 PE=2 SV=1 - [D3Z5N2_MOUSE]	52.38	8	9	9	77	-0.06	0.08	-0.10	0.00
Q6PGL7	WASH complex subunit FAM21 OS=Mus musculus GN=Fam21 PE=1 SV=1 - [FAM21_MOUSE]	19.87	2	13	14	43	0.05	0.20	-0.10	0.04
D3Z3A9	Reticulocalbin-3 (Fragment) OS=Mus musculus GN=Rcn3 PE=2 SV=1 - [D3Z3A9_MOUSE]	35.77	4	7	7	50	-0.09	-0.16	-0.16	-0.22
Q9EP64	Tenomodulin OS=Mus musculus GN=Tnmd PE=2 SV=1 - [TNMD_MOUSE]	9.15	1	2	2	4	-0.03	-0.38	0.09	-0.19
B1AR50	Gamma-aminobutyric acid receptor-associated protein OS=Mus musculus GN=Gabarap PE=2 SV=1 - [B1AR50_MOUSE]	16.04	2	2	2	6	-0.10	0.05	-0.34	-0.04
E9Q8F9	Protein 6030445D17Rik OS=Mus musculus GN=6030445D17Rik PE=4 SV=1 - [E9Q8F9_MOUSE]	12.16	1	2	2	2	-0.29	-0.46	-0.09	-0.26
Q07417	Short-chain specific acyl-CoA dehydrogenase, mitochondrial OS=Mus musculus GN=Acads PE=2 SV=2 - [ACADS_MOUSE]	52.18	2	18	18	436	-0.76	-0.68	-0.71	-0.66
Q9CZD0	Methylmalonic aciduria and homocystinuria type C protein homolog OS=Mus musculus GN=Mmachc PE=2 SV=2 - [MMAC_MOUSE]	6.45	1	1	1	1	-0.11	-0.52	-0.39	-0.80
Q812E0	Cytoplasmic polyadenylation element-binding protein 2 OS=Mus musculus GN=Cpeb2 PE=1 SV=1 - [CPEB2_MOUSE]	1.54	4	1	1	2	-0.27	-0.13	-0.10	0.03
D3YVI7	Protein Trim30b OS=Mus musculus GN=Trim30b PE=2 SV=1 - [D3YVI7_MOUSE]	3.81	1	1	1	1	-1.21	-3.27	-0.53	-2.59
O09160	Galactoside 2-alpha-L-fucosyltransferase 1 OS=Mus musculus GN=Fut1 PE=2 SV=1 - [FUT1_MOUSE]	5.05	2	1	1	2	-0.99	-1.21	-0.51	-0.95
Q99M07	Cytochrome c oxidase assembly factor 5 OS=Mus musculus GN=Coa5 PE=2 SV=1 - [COA5_MOUSE]	21.62	1	2	2	3	0.43	0.59	0.11	0.59
P84309	Adenylate cyclase type 5 OS=Mus musculus GN=Adcy5 PE=1 SV=2 - [ADCY5_MOUSE]	2.77	2	1	3	3	0.28	0.17	-0.05	-0.16
P62309	Small nuclear ribonucleoprotein G OS=Mus musculus GN=Snrpg PE=1 SV=1 [RUXG_MOUSE]	25.00	1	2	2	41	-0.48	-0.35	-0.54	-0.32

E9QP00	Transformer-2 protein homolog alpha OS=Mus musculus GN=Tra2a PE=2 SV=1 - [E9QP00_MOUSE]	19.86	2	3	3	8	-0.47	-0.38	-0.50	-0.39
G5E881	Mitochondrial translocator assembly and maintenance protein 41 homolog OS=Mus musculus GN=Tamm41 PE=4 SV=1 - [G5E881_MOUSE]	15.43	5	4	4	7	-0.09	-0.43	0.09	-0.19
P30678	Guanine nucleotide-binding protein subunit alpha-15 OS=Mus musculus GN=Gna15 PE=2 SV=1 - [GNA15_MOUSE]	3.74	1	1	2	3	-0.20	-0.42	0.12	-0.10
E9Q5C9	Protein Nolc1 OS=Mus musculus GN=Nolc1 PE=2 SV=1 - [E9Q5C9_MOUSE]	12.39	1	10	10	32	0.27	0.26	0.21	0.04
Q9CQI3	Glia maturation factor beta OS=Mus musculus GN=Gmfb PE=1 SV=3 [GMFB_MOUSE]	38.03	2	4	5	49	-0.18	0.04	-0.41	-0.02
Q9CR41	Huntingtin-interacting protein K OS=Mus musculus GN=Hypk PE=2 SV=2 - [HYPK_MOUSE]	34.88	2	5	5	43	-0.17	-0.19	-0.20	-0.11
P17095	High mobility group protein HMG-I/HMG-Y OS=Mus musculus GN=Hmga1 PE=1 SV=4 - [HMGA1_MOUSE]	30.84	1	1	3	37	0.39	0.25	0.09	0.17
G3X9Q3	Histocompatibility (Minor) HA-1, isoform CRA_a OS=Mus musculus GN=Hmha1 PE=4 SV=1 - [G3X9Q3_MOUSE]	5.11	6	4	4	7	-0.15	-0.01	-0.04	0.04
P62313	U6 snRNA-associated Sm-like protein LSm6 OS=Mus musculus GN=Lsm6 PE=2 SV=1 [LSM6_MOUSE]	45.00	1	5	5	12	-0.19	0.06	-0.19	0.10
Q14CH1	Molybdenum cofactor sulfurase OS=Mus musculus GN=Mocos PE=2 SV=1 - [MOCOS_MOUSE]	2.55	1	1	1	1	-0.78	-0.66	-0.60	-0.49
Q61194-2	Isoform 2 of Phosphatidylinositol 4-phosphate 3-kinase C2 domain-containing subunit alpha OS=Mus musculus GN=Pik3c2a - [P3C2A_MOUSE]	2.17	3	3	3	7	0.04	0.17	-0.01	0.10
Q9R244-2	Isoform 2 of Short transient receptor potential channel 2 OS=Mus musculus GN=Trpc2 - [TRPC2_MOUSE]	3.92	7	1	4	9	0.02	-0.65	-0.14	-0.82
Q9D7Z3-2	Isoform 2 of Nucleolar protein 7 OS=Mus musculus GN=Nol7 - [NOL7_MOUSE]	5.76	2	1	1	3	-0.58	-0.27	-0.22	0.08

Q91VR7	Microtubule-associated proteins 1A/1B light chain 3A OS=Mus musculus GN=Map1lc3a PE=1 SV=1 - [MLP3A_MOUSE]	17.36	3	2	2	5	-0.33	-0.26	-0.26	-0.10
Q9DD06	Retinoic acid receptor responder protein 2 OS=Mus musculus GN=Rarres2 PE=2 SV=1 - [RARR2_MOUSE]	9.26	1	1	1	2	-0.16	-0.55	-0.39	-0.78
A2A977	Cytochrome P450 4A10 OS=Mus musculus GN=Cyp4a10 PE=2 SV=1 - [A2A977_MOUSE]	2.20	2	1	1	3	-0.10	-0.13	-0.01	-0.04
Q78HU3	Multivesicular body subunit 12A OS=Mus musculus GN=Mvb12a PE=1 SV=1 - [MB12A_MOUSE]	3.69	1	1	1	4	-0.06	0.16	0.01	0.22
D3YVV7	Protein Nova2 OS=Mus musculus GN=Nova2 PE=4 SV=2 - [D3YVV7_MOUSE]	1.63	2	1	1	14	-0.19	0.03	-0.08	0.13
Q8BT60	Copine-3 OS=Mus musculus GN=Cpne3 PE=1 SV=2 - [CPNE3_MOUSE]	33.21	6	15	17	109	-0.07	-0.16	-0.03	-0.12
Q9WUU7	Cathepsin Z OS=Mus musculus GN=Ctsz PE=2 SV=1 - [CATZ_MOUSE]	26.14	1	7	7	88	-0.23	-0.22	-0.26	-0.22
B7ZDD9	Carnitine O-acetyltransferase OS=Mus musculus GN=Crat PE=2 SV=1 - [B7ZDD9_MOUSE]	10.34	1	1	1	4	-0.25	-0.32	-0.44	-0.56
Q07797	Galectin-3-binding protein OS=Mus musculus GN=Lgals3bp PE=1 SV=1 - [LG3BP_MOUSE]	27.04	2	12	12	74	-0.32	-0.36	-0.33	-0.43
Q9DB15	39S ribosomal protein L12, mitochondrial OS=Mus musculus GN=Mrpl12 PE=1 SV=2 - [RM12_MOUSE]	42.29	1	7	7	157	-0.19	-0.09	-0.28	-0.17
D3Z6F5	ATP synthase subunit alpha OS=Mus musculus GN=Atp5a1 PE=3 SV=1 - [D3Z6F5_MOUSE]	59.05	1	1	30	1192	-0.20	-0.38	-0.61	-0.80
P20033-2	Isoform Short of Platelet-derived growth factor subunit A OS=Mus musculus GN=Pdgfa - [PDGFA_MOUSE]	11.22	4	1	1	1	-1.44	-0.55	-1.14	-0.25
Q60972	Histone-binding protein RBBP4 OS=Mus musculus GN=Rbbp4 PE=1 SV=5 - [RBBP4_MOUSE]	42.12	1	6	12	84	-0.24	-0.17	-0.61	-0.46
Q9Z1Z2	Serine-threonine kinase receptor-associated protein OS=Mus musculus GN=Strap PE=1 SV=2 - [STRAP_MOUSE]	52.86	1	16	16	121	-0.09	0.10	-0.13	0.00
F6VIY4	Crossover junction endonuclease MUS81 (Fragment) OS=Mus musculus GN=Mus81 PE=4 SV=1 - [F6VIY4_MOUSE]	3.34	3	1	1	1	-0.26	0.00	0.02	0.28

M0QWZ4	Protein Zfp142 OS=Mus musculus GN=Zfp142 PE=4 SV=1 - [M0QWZ4_MOUSE]	5.48	1	1	1	2	0.21	0.38	0.24	0.41
Q9DC61	Mitochondrial-processing peptidase subunit alpha OS=Mus musculus GN=Pmpca PE=1 SV=1 - [MPPA_MOUSE]	30.92	2	14	14	69	-0.27	-0.20	-0.32	-0.31
Q80ZJ6-3	Isoform 3 of Protein zer-1 homolog OS=Mus musculus GN=Zer1 - [ZER1_MOUSE]	6.71	3	2	2	4	-0.05	-0.36	-0.02	-0.34
Q8BKN5-3	Isoform 3 of Gamma-tubulin complex component 5 OS=Mus musculus GN=Tubgcp5 [GCP5_MOUSE]	3.89	3	3	3	3	-0.22	-0.23	-0.59	-0.60
G3UW90	Trichorhinophalangeal syndrome I (Human), isoform CRA_b OS=Mus musculus GN=Trps1 PE=4 SV=1 [G3UW90_MOUSE]	1.87	1	1	1	1	-1.02	-0.60	-0.81	-0.40
Q80WV7-2	Isoform 2 of Serine/arginine repetitive matrix protein 3 OS=Mus musculus GN=Srrm3 - [SRRM3_MOUSE]	9.82	4	1	1	1	0.27	0.45	0.29	0.46
Q99N91	39S ribosomal protein L34, mitochondrial OS=Mus musculus GN=Mrpl34 PE=2 SV=1 - [RM34_MOUSE]	10.87	1	1	1	2	-0.24	-0.38	-0.39	-0.53
Q91ZM2-6	Isoform 6 of SH2B adapter protein 1 OS=Mus musculus GN=Sh2b1 - [SH2B1_MOUSE]	15.86	6	4	4	7	-0.26	-0.60	-0.47	-0.81
Q8BZ00	Sodium/hydrogen exchanger 9 OS=Mus musculus GN=Slc9a9 PE=2 SV=1 - [SL9A9_MOUSE]	3.73	1	1	1	4	-0.28	0.06	-0.47	-0.18
Q569Z6	Thyroid hormone receptor-associated protein 3 OS=Mus musculus GN=Thrap3 PE=1 SV=1 - [TR150_MOUSE]	16.61	4	13	15	53	0.07	0.23	-0.01	0.10
Q9QY61	Iroquois-class homeodomain protein IRX-4 OS=Mus musculus GN=Irx4 PE=2 SV=1 - [IRX4_MOUSE]	6.21	1	1	1	1	-0.72	-1.02	-0.80	-1.10
Q60936	Chaperone activity of bc1 complex-like, mitochondrial OS=Mus musculus GN=Adck3 PE=2 SV=2 - [ADCK3_MOUSE]	4.65	3	2	2	9	-0.31	-0.47	-0.50	-0.65
A2ATE0	Protein Olfr1029 OS=Mus musculus GN=Olfr1029 PE=4 SV=1 - [A2ATE0_MOUSE]	4.01	2	1	1	1	-0.04	-0.34	-0.14	-0.44
P08101-2	Isoform IIB2 of Low affinity immunoglobulin gamma Fc region receptor II OS=Mus musculus GN=Fcgr2 - [FCGR2_MOUSE]	11.66	5	3	3	10	-0.16	-0.01	-0.16	-0.08

P41539	Protachykinin-1 OS=Mus musculus GN=Tac1 PE=2 SV=1 - [TKN1_MOUSE]	14.62	2	2	2	9	-0.43	-0.38	-0.90	-1.19
P62073	Mitochondrial import inner membrane translocase subunit Tim10 OS=Mus musculus GN=Timm10 PE=1 SV=1 - [TIM10_MOUSE]	61.11	1	6	6	46	-0.38	-0.43	-0.36	-0.26
F8WJG3	Transformer-2 protein homolog beta OS=Mus musculus GN=Tra2b PE=2 SV=1 - [F8WJG3_MOUSE]	19.68	2	3	3	16	-0.32	-0.07	-0.39	-0.16
Q8C0T5-2	Isoform 2 of Signal-induced proliferation-associated 1-like protein 1 OS=Mus musculus GN=Sipa1l1 - [SI1L1_MOUSE]	4.00	2	3	4	6	-0.40	-0.09	-0.74	-0.42
P62254	Ubiquitin-conjugating enzyme E2 G1 OS=Mus musculus GN=Ube2g1 PE=2 SV=3 - [UB2G1_MOUSE]	18.82	1	3	3	24	0.01	-0.21	-0.12	-0.25
G3UYB7	Myotubularin-related protein 7 (Fragment) OS=Mus musculus GN=Mtmr7 PE=2 SV=1 - [G3UYB7_MOUSE]	8.85	5	2	2	2	-0.17	1.02	-1.41	-0.23
P24472	Glutathione S-transferase A4 OS=Mus musculus GN=Gsta4 PE=1 SV=3 - [GSTA4_MOUSE]	42.34	1	7	8	102	-0.71	-0.64	-0.74	-0.68
Q8CHI8-3	Isoform 3 of E1A-binding protein p400 OS=Mus musculus GN=Ep400 - [EP400_MOUSE]	1.33	4	1	3	4	-0.20	-0.06	-0.05	0.09
Q61324-2	Isoform 2 of Aryl hydrocarbon receptor nuclear translocator 2 OS=Mus musculus GN=Arnt2 - [ARNT2_MOUSE]	7.99	2	2	2	2	-0.08	-0.25	-0.37	-0.55
P97470	Serine/threonine-protein phosphatase 4 catalytic subunit OS=Mus musculus GN=Ppp4c PE=1 SV=2 - [PP4C_MOUSE]	18.24	1	2	3	25	-0.24	-0.10	-0.40	-0.26
D3YTV3	Probable ATP-dependent RNA helicase DHX34 OS=Mus musculus GN=Dhx34 PE=4 SV=1 - [D3YTV3_MOUSE]	1.83	2	2	2	3	0.54	-0.49	0.07	-0.96
Q9CQG2	Methyltransferase-like protein 16 OS=Mus musculus GN=Mettl16 PE=1 SV=1 - [MET16_MOUSE]	1.81	2	1	1	1	0.10	0.53	1.02	1.45
E9QKA4	Serine/arginine repetitive matrix protein 1 OS=Mus musculus GN=Srrm1 PE=2 SV=1 - [E9QKA4_MOUSE]	11.82	9	7	9	26	0.25	0.30	0.20	0.17
Q8R1N0	Zinc finger protein 830 OS=Mus musculus GN=Znf830 PE=1 SV=1 - [ZN830_MOUSE]	12.40	1	2	2	11	0.20	0.32	0.15	0.26

Q6ZWY8	Thymosin beta-10 OS=Mus musculus GN=Tmsb10 PE=2 SV=3 - [TYB10_MOUSE]	43.18	3	2	3	6	0.00	0.10	0.07	0.17
P56394	Cytochrome c oxidase copper chaperone OS=Mus musculus GN=Cox17 PE=2 SV=2 - [COX17_MOUSE]	31.75	1	2	2	19	-0.17	0.10	-0.22	0.04
Q6W8Q3	Purkinje cell protein 4-like protein 1 OS=Mus musculus GN=Pcp4l1 PE=2 SV=1 - [PC4L1_MOUSE]	70.59	1	4	4	29	-0.05	0.24	-0.18	0.23
Q3UG37	Histone deacetylase 6 (Fragment) OS=Mus musculus GN=Hdac6 PE=2 SV=1 - [Q3UG37_MOUSE]	8.62	3	4	4	9	-0.15	0.54	-0.24	0.08
Q9QZH3	Peptidyl-prolyl cis-trans isomerase E OS=Mus musculus GN=Ppie PE=2 SV=2 - [PPIE_MOUSE]	13.95	1	3	4	18	-0.17	0.17	-0.38	-0.20
E9PV44	ATPase inhibitor, mitochondrial OS=Mus musculus GN=Atpif1 PE=2 SV=1 - [E9PV44_MOUSE]	47.30	2	7	7	91	-0.37	-0.23	-0.51	-0.33
Q80ZW2	Protein THEM6 OS=Mus musculus GN=Them6 PE=2 SV=1 - [THEM6_MOUSE]	3.38	1	1	1	1	0.20	0.12	-0.32	-0.40
Q8BV49	Pyrin and HIN domain-containing protein 1 OS=Mus musculus GN=Pyhin1 PE=2 SV=1 - [IFIX_MOUSE]	14.29	3	2	5	6	-0.16	0.23	-0.11	0.27
Q9Z204	Heterogeneous nuclear ribonucleoproteins C1/C2 OS=Mus musculus GN=Hnrnpc PE=1 SV=1 - [HNRPC_MOUSE]	46.65	6	1	14	115	0.24	-0.04	0.04	-0.24
Q05816	Fatty acid-binding protein, epidermal OS=Mus musculus GN=Fabp5 PE=1 SV=3 [FABP5_MOUSE]	65.93	1	7	7	76	0.00	0.02	-0.02	-0.04
Q8K2F8	Protein LSM14 homolog A OS=Mus musculus GN=Lsm14a PE=1 SV=1 - [LS14A_MOUSE]	18.61	3	6	7	24	0.12	0.17	-0.17	-0.26
Q3TWW8	Protein Srsf6 OS=Mus musculus GN=Srsf6 PE=2 SV=1 - [Q3TWW8_MOUSE]	28.02	1	8	11	75	0.00	0.01	-0.05	-0.10
Q5UAK0-4	Isoform 4 of Mesoderm induction early response protein 1 OS=Mus musculus GN=Mier1 - [MIER1_MOUSE]	10.39	5	3	4	16	-0.76	-0.33	-1.08	-0.66
Q8R3W2	MCG6979, isoform CRA_a OS=Mus musculus GN=0610009B22Rik PE=2 SV=1 - [Q8R3W2_MOUSE]	10.00	2	1	1	7	-0.99	-1.11	-0.39	-0.47
O54962	Barrier-to-autointegration factor OS=Mus musculus GN=Banf1 PE=1 SV=1 [BAF_MOUSE]	43.82	1	3	3	66	-0.16	0.22	-0.24	-0.04

Q9D6S7	Ribosome-recycling factor, mitochondrial OS=Mus musculus GN=Mrrf PE=1 SV=1 - [RRFM_MOUSE]	6.11	1	1	1	21	-0.26	-0.20	-0.31	-0.22
Q9DAC9	POU domain, class 5, transcription factor 2 OS=Mus musculus GN=Pou5f2 PE=2 SV=1 - [PO5F2_MOUSE]	12.16	1	2	2	2	-0.29	0.36	-0.89	-0.25
Q69ZP3-2	Isoform 2 of Probable hydrolase PNKD OS=Mus musculus GN=Pnkd - [PNKD_MOUSE]	10.56	3	1	1	3	0.02	0.15	-0.22	-0.09
Q3UHC2	Leucine-rich repeat serine/threonine-protein kinase 1 OS=Mus musculus GN=Lrrk1 PE=2 SV=1 - [LRRK1_MOUSE]	4.07	3	3	3	3	-0.05	-0.10	-0.67	-0.72
O88271	Craniofacial development protein 1 OS=Mus musculus GN=Cfdp1 PE=1 SV=1 - [CFDP1_MOUSE]	19.32	1	4	4	15	0.26	0.17	-0.06	-0.05
Q9CPX8	Cytochrome b-c1 complex subunit 10 OS=Mus musculus GN=Uqcr11 PE=2 SV=1 - [QCR10_MOUSE]	21.43	1	1	1	4	-0.46	-0.54	-0.40	-0.48
P06683	Complement component C9 OS=Mus musculus GN=C9 PE=1 SV=2 - [CO9_MOUSE]	13.32	2	6	7	12	-0.15	0.07	-0.24	-0.02
Q9Z0M5	Lysosomal acid lipase/cholesteryl ester hydrolase OS=Mus musculus GN=Lipa PE=2 SV=2 - [LICH_MOUSE]	4.53	1	2	2	3	-0.39	-0.55	-0.35	-0.51
Q9QXE2	DNA polymerase lambda OS=Mus musculus GN=Poll PE=2 SV=1 - [DPOLL_MOUSE]	3.49	1	3	3	3	-0.29	0.11	-0.37	0.02
G3X9V2	Catenin (Cadherin associated protein), delta 1, isoform CRA_a OS=Mus musculus GN=Ctnnd1 PE=4 SV=1 - [G3X9V2_MOUSE]	38.30	18	1	31	226	-0.17	-0.14	-0.21	-0.18
Q8C0N2	Glycerol-3-phosphate acyltransferase 3 OS=Mus musculus GN=Agpat9 PE=1 SV=1 - [GPAT3_MOUSE]	5.02	1	1	2	2	0.24	-0.01	-0.06	-0.31
G5E8Q4	Cytohesin-3 OS=Mus musculus GN=Cyth3 PE=4 SV=1 - [G5E8Q4_MOUSE]	3.99	2	1	1	3	-0.39	-0.47	-0.43	-0.51
Q0VAV2	Exophilin-5 OS=Mus musculus GN=Exph5 PE=1 SV=1 - [EXPH5_MOUSE]	0.36	1	1	1	1	-0.69	-1.10	0.26	-0.15
Q61830	Macrophage mannose receptor 1 OS=Mus musculus GN=Mrc1 PE=1 SV=2 - [MRC1_MOUSE]	20.40	1	26	26	62	-0.26	-0.28	-0.18	-0.26
F6ZDT4	Ribonuclease H2 subunit C (Fragment) OS=Mus musculus GN=Rnaseh2c PE=4 SV=1 - [F6ZDT4_MOUSE]	22.73	2	1	1	5	0.13	-0.36	0.01	-0.54

Q8CH77-4	Isoform 4 of Neuron navigator 1 OS=Mus musculus GN=Nav1 - [NAV1_MOUSE]	5.44	4	2	2	2	-0.15	-0.06	-0.33	-0.37
Q9CQI6	Coactosin-like protein OS=Mus musculus GN=Cotl1 PE=1 SV=3 - [COTL1_MOUSE]	60.56	1	9	9	99	-0.27	-0.37	-0.27	-0.27
Q9ER41	Torsin-1B OS=Mus musculus GN=Tor1b PE=2 SV=2 - [TOR1B_MOUSE]	14.58	4	4	4	12	-0.23	-0.16	-0.32	-0.47
O54785	LIM domain kinase 2 OS=Mus musculus GN=Limk2 PE=1 SV=2 - [LIMK2_MOUSE]	11.13	4	5	5	8	0.01	-0.67	-0.56	-0.71
Q91WD9	Secretagogin OS=Mus musculus GN=Scgn PE=2 SV=1 - [SEGN_MOUSE]	46.74	1	11	11	55	-1.05	-1.03	-1.05	-0.94
Q7JJ13-2	Isoform 2 of Bromodomain-containing protein 2 OS=Mus musculus GN=Brd2 - [BRD2_MOUSE]	8.11	5	4	5	9	-0.26	-0.44	-0.30	-0.48
P70158	Acid sphingomyelinase-like phosphodiesterase 3a OS=Mus musculus GN=Smpdl3a PE=2 SV=2 - [ASM3A_MOUSE]	14.61	2	5	5	6	0.01	0.17	0.10	0.25
F7C0F2	Breast carcinoma-amplified sequence 3 homolog (Fragment) OS=Mus musculus GN=Bcas3 PE=4 SV=1 [F7C0F2_MOUSE]	4.91	6	3	3	3	-0.07	0.13	-0.26	-0.07
Q6NZN1-3	Isoform 3 of Peroxisome proliferator-activated receptor gamm coactivator- related protein 1 OS=Mus musculus GN=Pprc1 - [PPRC1_MOUSE]	4.98	4	2	2	2	0.05	0.39	-0.17	0.17
Q921H8	3-ketoacyl-CoA thiolase A, peroxisomal OS=Mus musculus GN=Acaa1a PE=2 SV=1 - [THIKA_MOUSE]	40.09	7	15	15	210	-0.24	-0.15	-0.27	-0.16
Q8CF93	Polypeptide N-acetylgalactosaminyltransferase 13 OS=Mus musculus GN=Galnt13 PE=2 SV=1 - [GLT13_MOUSE]	2.88	3	1	2	5	-0.53	-0.26	-0.72	-0.45
Q9WV52	Pleckstrin-2 OS=Mus musculus GN=Plek2 PE=1 SV=1 - [PLEK2_MOUSE]	4.82	1	2	2	4	0.54	0.68	0.38	0.51
Q8BHL3	TBC1 domain family member 10B OS=Mus musculus GN=Tbc1d10b PE=1 SV=2 - [TB10B_MOUSE]	3.13	1	2	2	4	-0.31	-0.34	0.05	-0.07
Q9D279	Mitotic interactor and substrate of PLK1 OS=Mus musculus GN=Misp PE=2 SV=1 - [MISP_MOUSE]	47.69	1	23	23	210	0.01	0.02	0.07	0.09
Q8BG54	Serine palmitoyltransferase 3 OS=Mus musculus GN=Sptlc3 PE=2 SV=1 - [SPTC3_MOUSE]	1.24	1	1	1	1	-0.31	0.20	-0.46	0.05

O89086	Putative RNA-binding protein 3 OS=Mus musculus GN=Rbm3 PE=1 SV=1 - [RBM3_MOUSE]	41.83	2	3	3	116	-0.21	-0.28	-0.13	-0.21
P51174	Long-chain specific acyl-CoA dehydrogenase, mitochondrial OS=Mus musculus GN=Acadl PE=2 SV=2 - [ACADL_MOUSE]	44.88	2	18	18	297	-0.58	-0.65	-0.61	-0.70
Q9Z130	Heterogeneous nuclear ribonucleoprotein D-like OS=Mus musculus GN=Hnrpdl PE=1 SV=1 - [HNRDL_MOUSE]	29.57	11	8	9	74	0.00	-0.04	-0.15	-0.14
Q9JKK7	Tropomodulin-2 OS=Mus musculus GN=Tmod2 PE=1 SV=2 - [TMOD2_MOUSE]	29.06	3	7	8	23	-0.76	-0.51	-0.94	-0.66
P97822-2	Isoform 2 of Acidic leucine-rich nuclear phosphoprotein 32 family member E OS=Mus musculus GN=Anp32e - [AN32E_MOUSE]	23.79	8	4	6	75	-0.32	-0.38	-0.36	-0.24
Q45VK7	Cytoplasmic dynein 2 heavy chain 1 OS=Mus musculus GN=Dync2h1 PE=1 SV=1 - [DYHC2_MOUSE]	0.84	3	3	4	6	-0.13	-0.24	-0.60	-0.72
Q9JHU9	Inositol-3-phosphate synthase 1 OS=Mus musculus GN=Isyna1 PE=2 SV=1 - [INO1_MOUSE]	17.59	1	8	8	38	-0.16	-0.07	-0.23	-0.12
Q9CQP3	Coiled-coil-helix-coiled-coil-helix domain-containing protein 5 OS=Mus musculus GN=Chchd5 PE=2 SV=1 - [CHCH5_MOUSE]	19.09	1	2	2	3	-0.08	0.14	-0.15	0.08
Q9JM96	Cdc42 effector protein 4 OS=Mus musculus GN=Cdc42ep4 PE=1 SV=1 - [BORG4_MOUSE]	22.92	3	5	5	16	-0.23	-0.44	-0.24	-0.42
Q99MJ9	ATP-dependent RNA helicase DDX50 OS=Mus musculus GN=Ddx50 PE=2 SV=1 - [DDX50_MOUSE]	7.08	2	3	4	12	-0.51	0.22	-0.96	-0.23
O35900	U6 snRNA-associated Sm-like protein LSm2 OS=Mus musculus GN=Lsm2 PE=2 SV=1 [LSM2_MOUSE]	38.95	2	3	3	46	-0.07	0.09	-0.16	-0.06
Q8VE22	28S ribosomal protein S23, mitochondrial OS=Mus musculus GN=Mrps23 PE=2 SV=1 - [RT23_MOUSE]	28.81	5	5	5	25	0.02	-0.16	-0.14	-0.14
G3UZA0	KRR1 small subunit processome component homolog OS=Mus musculus GN=Krr1 PE=2 SV=1 - [G3UZA0_MOUSE]	2.79	2	1	1	1	-3.61	-0.13	-4.21	-0.74
O35887	Calumenin OS=Mus musculus GN=Calu PE=1 SV=1 - [CALU_MOUSE]	67.94	7	3	17	424	-0.29	-0.27	-0.44	-0.54

Q99LT0	Protein dpy-30 homolog OS=Mus musculus GN=Dpy30 PE=1 SV=1 - [DPY30_MOUSE]	9.09	1	1	1	4	-0.19	-0.17	-0.20	-0.18
Q8BGT7	Survival of motor neuron-related-splicing factor 30 OS=Mus musculus GN=Smndc1 PE=2 SV=1 - [SPF30_MOUSE]	20.59	1	4	4	7	-0.19	0.13	-0.31	0.08
P25799	Nuclear factor NF-kappa-B p105 subunit OS=Mus musculus GN=Nfkb1 PE=1 SV=2 - [NFKB1_MOUSE]	12.46	5	7	7	34	-0.03	-0.28	-0.10	-0.34
Q9WUU8-2	Isoform 2 of TNFAIP3-interacting protein 1 OS=Mus musculus GN=Tnip1 - [TNIP1_MOUSE]	9.60	7	4	5	10	0.06	-0.02	0.08	0.00
Q9WV92-2	Isoform 2 of Band 4.1-like protein 3 OS=Mus musculus GN=Epb41l3 [E41L3_MOUSE]	23.00	9	14	19	67	-0.35	-0.26	-0.38	-0.29
O70172	Phosphatidylinositol 5-phosphate 4-kinase type 2 alpha OS=Mus musculus GN=Pip4k2a PE=1 SV=1 - [PI42A_MOUSE]	16.30	2	3	5	20	-0.18	-0.20	-0.26	-0.21
Q99K85	Phosphoserine aminotransferase OS=Mus musculus GN=Psat1 PE=1 SV=1 - [SERC_MOUSE]	20.00	3	6	7	16	0.41	0.54	0.50	0.32
Q6PHN9	Ras-related protein Rab-35 OS=Mus musculus GN=Rab35 PE=1 SV=1 - [RAB35_MOUSE]	31.84	14	3	6	60	-0.16	-0.03	-0.16	0.04
P56873	Sjoegren syndrome/scleroderma autoantigen 1 homolog OS=Mus musculus GN=Sssca1 PE=2 SV=1 - [SSA27_MOUSE]	5.03	1	1	1	4	-0.13	-0.09	-0.26	-0.23
P62259	14-3-3 protein epsilon OS=Mus musculus GN=Ywhae PE=1 SV=1 - [1433E_MOUSE]	76.08	3	18	21	509	-0.34	-0.32	-0.40	-0.38
Q9JHW9	Aldehyde dehydrogenase family 1 member A3 OS=Mus musculus GN=Aldh1a3 PE=2 SV=1 - [AL1A3_MOUSE]	18.75	2	4	8	41	-0.39	-0.04	-0.55	-0.57
E9QN01	5'-nucleotidase domain-containing protein 1 OS=Mus musculus GN=Nt5dc1 PE=2 SV=1 - [E9QN01_MOUSE]	6.47	4	1	1	1	0.45	0.02	0.51	0.07
Q9WVQ5	Methylthioribulose-1-phosphate dehydratase OS=Mus musculus GN=Apip PE=1 SV=1 - [MTNB_MOUSE]	27.39	1	4	4	7	-0.17	-0.12	-0.13	-0.09
Q0VBL3	Protein Rbm15 OS=Mus musculus GN=Rbm15 PE=2 SV=1 - [Q0VBL3_MOUSE]	5.61	1	3	4	8	-0.63	0.02	-0.58	0.06

E9Q944	Protein Kank1 (Fragment) OS=Mus musculus GN=Kank1 PE=2 SV=1 - [E9Q944_MOUSE]	8.63	2	3	5	8	-1.06	-5.71	-0.58	-5.24
Q99KE1	NAD-dependent malic enzyme, mitochondrial OS=Mus musculus GN=Me2 PE=2 SV=1 - [MAOM_MOUSE]	38.54	1	18	18	237	-0.21	-0.21	-0.19	-0.28
P26048	Gamma-aminobutyric acid receptor subunit alpha-2 OS=Mus musculus GN=Gabra2 PE=1 SV=1 - [GBRA2_MOUSE]	1.77	2	1	1	2	0.21	0.07	0.13	-0.01
Q5PSV9	Mediator of DNA damage checkpoint protein 1 OS=Mus musculus GN=Mdc1 PE=1 SV=1 - [MDC1_MOUSE]	1.41	2	1	1	1	0.00	-0.57	-0.14	-0.71
Q8K2T8	RNA polymerase II-associated factor 1 homolog OS=Mus musculus GN=Paf1 PE=2 SV=1 - [PAF1_MOUSE]	8.04	1	4	4	5	-0.34	-0.27	-0.27	-0.13
Q3TYL0	Putative IQ motif and ankyrin repeat domain-containing protein LOC642574 homolog OS=Mus musculus PE=5 SV=2 - [YH010_MOUSE]	7.29	1	2	2	3	0.41	0.37	-0.26	-0.30
H3BL68	PAB-dependent poly(A) specific ribonuclease subunit 3 (Fragment) OS=Mus musculus GN=Pan3 PE=4 SV=1 - [H3BL68_MOUSE]	10.93	4	1	1	4	-0.20	0.12	-0.43	-0.11
P06837	Neuromodulin OS=Mus musculus GN=Gap43 PE=1 SV=1 - [NEUM_MOUSE]	30.84	1	5	5	15	-0.57	-0.37	-0.73	-0.57
Q148B1	Cytochrome P450, family 2, subfamily c, polypeptide 65 OS=Mus musculus GN=Cyp2c65 PE=2 SV=1 - [Q148B1_MOUSE]	34.69	4	8	17	127	-0.73	-0.63	-0.76	-0.68
P06728	Apolipoprotein A-IV OS=Mus musculus GN=Apoa4 PE=2 SV=3 - [APOA4_MOUSE]	51.90	1	17	17	78	-0.21	-0.23	-0.15	-0.16
D3YUG5	MKL/myocardin-like protein 1 (Fragment) OS=Mus musculus GN=Mkl1 PE=2 SV=1 - [D3YUG5_MOUSE]	4.68	4	2	3	4	-0.70	-0.54	-0.36	-0.20
Q640M1	U3 small nucleolar RNA-associated protein 14 homolog A OS=Mus musculus GN=Utp14a PE=2 SV=1 - [UT14A_MOUSE]	7.30	1	3	3	12	-0.15	0.12	-0.21	0.15
P97872	Dimethylaniline monooxygenase [N-oxide-forming] 5 OS=Mus musculus GN=Fmo5 PE=2 SV=4 [FMO5_MOUSE]	7.69	1	3	3	3	-1.65	-1.70	-1.64	-1.70
E9QLD5	Serine protease 29 OS=Mus musculus GN=Prss29 PE=3 SV=1 - [E9QLD5_MOUSE]	3.58	2	1	1	1	0.43	0.50	0.35	0.42

F7ABZ6	Protein Dnah10 (Fragment) OS=Mus musculus GN=Dnah10 PE=4 SV=1 - [F7ABZ6_MOUSE]	1.08	2	4	6	10	-0.42	-2.14	-0.15	-1.87
A2A9K3	Centrosomal protein of 85 kDa OS=Mus musculus GN=Cep85 PE=2 SV=1 - [A2A9K3_MOUSE]	2.37	2	2	2	3	-0.74	-0.66	-0.76	-0.68
P24547	Inosine-5'-monophosphate dehydrogenase 2 OS=Mus musculus GN=Impdh2 PE=1 SV=2 - [IMDH2_MOUSE]	28.40	1	12	14	90	-0.04	0.14	0.06	0.17
E9Q9W1	Protein 4930432E11Rik OS=Mus musculus GN=4930432E11Rik PE=4 SV=1 - [E9Q9W1_MOUSE]	5.81	1	4	4	8	-0.15	0.00	-0.71	-0.52
P58242	Acid sphingomyelinase-like phosphodiesterase 3b OS=Mus musculus GN=Smpdl3b PE=1 SV=1 - [ASM3B_MOUSE]	3.51	1	1	1	1	-1.07	-1.04	-1.34	-1.31
D6RIQ3	Phytanoyl-CoA dioxygenase domain-containing protein 1 OS=Mus musculus GN=Phyhd1 PE=2 SV=1 - [D6RIQ3_MOUSE]	6.29	6	1	1	3	-0.18	-0.20	-0.22	-0.25
P10605	Cathepsin B OS=Mus musculus GN=Ctsb PE=1 SV=2 - [CATB_MOUSE]	33.33	1	11	11	117	0.07	-0.01	0.00	-0.15
Q9CRB3	5-hydroxyisourate hydrolase OS=Mus musculus GN=Urah PE=1 SV=1 - [HIUH_MOUSE]	13.56	1	2	2	5	-0.27	0.19	-0.49	0.06
Q765H6	Alpha-1,6-mannosylglycoprotein 6 beta-N-acetylglucosaminyltransferase B OS=Mus musculus GN=Mgat5b PE=1 SV=1 - [MGT5B_MOUSE]	0.76	1	1	1	1	-0.76	-0.55	-0.74	-0.53
Q571F5	SPRY domain-containing SOCS box protein 3 OS=Mus musculus GN=Spsb3 PE=2 SV=2 - [SPSB3_MOUSE]	3.11	5	1	1	1	-0.43	-0.20	-0.42	-0.20
Q03311	Cholinesterase OS=Mus musculus GN=Bche PE=2 SV=2 - [CHLE_MOUSE]	12.94	1	8	8	18	-0.77	-0.81	-0.88	-0.98
E9PX14	Protein Ccdc152 OS=Mus musculus GN=Ccdc152 PE=4 SV=1 - [E9PX14_MOUSE]	4.33	1	1	1	1	-0.13	0.07	-0.91	-0.71
Q8VBU8-3	Isoform 3 of Protein BANP OS=Mus musculus GN=Banp - [BANP_MOUSE]	3.56	8	1	2	3	-0.21	-0.70	-0.04	-0.54
P52795	Ephrin-B1 OS=Mus musculus GN=Efnb1 PE=1 SV=1 - [EFNB1_MOUSE]	18.26	1	5	5	13	-0.30	-0.24	-0.34	-0.21

P06800-3	Isoform 3 of Receptor-type tyrosine-protein phosphatase C OS=Mus musculus GN=Ptprc - [PTPRC_MOUSE]	10.94	3	10	10	28	-0.03	0.04	-0.06	-0.19
Q9D0M1	Phosphoribosyl pyrophosphate synthase-associated protein 1 OS=Mus musculus GN=Prpsap1 PE=1 SV=1 - [KPRA_MOUSE]	38.48	4	7	10	59	-0.23	-0.24	-0.31	-0.34
P97427	Dihydropyrimidinase-related protein 1 OS=Mus musculus GN=Crmp1 PE=1 SV=1 - [DPYL1_MOUSE]	29.02	2	6	10	116	-0.23	-0.30	-0.48	-0.59
Q8VEH6	COBW domain-containing protein 1 OS=Mus musculus GN=Cbwd1 PE=2 SV=1 - [CBWD1_MOUSE]	9.41	2	3	4	6	0.12	0.09	0.15	0.02
Q80Y61	Brain-specific angiogenesis inhibitor 1-associated protein 2-like protein 2 OS=Mus musculus GN=Baiap2l2 PE=1 SV=1 - [BI2L2_MOUSE]	14.56	3	6	6	9	-0.82	-1.03	-1.05	-1.23
Q05421	Cytochrome P450 2E1 OS=Mus musculus GN=Cyp2e1 PE=1 SV=1 - [CP2E1_MOUSE]	1.62	1	1	1	2	-1.11	-1.18	-0.97	-1.04
Q80U95	Ubiquitin-protein ligase E3C OS=Mus musculus GN=Ube3c PE=2 SV=2 - [UBE3C_MOUSE]	6.28	1	5	5	6	0.56	0.53	0.23	0.19
Q91WU0	Expressed sequence AU018778 OS=Mus musculus GN=Ces1f PE=2 SV=1 - [Q91WU0_MOUSE]	41.53	4	15	17	199	-0.88	-0.80	-1.01	-0.86
Q8C0P5	Coronin-2A OS=Mus musculus GN=Coro2a PE=2 SV=1 - [COR2A_MOUSE]	26.72	2	10	12	41	-0.22	-0.40	-0.23	-0.40
E9Q7D5	Protein Arhgef5 OS=Mus musculus GN=Arhgef5 PE=2 SV=1 - [E9Q7D5_MOUSE]	3.73	1	4	4	13	-0.28	-0.33	-0.21	-0.24
P59438-3	Isoform 3 of Hermansky-Pudlak syndrome 5 protein homolog OS=Mus musculus GN=Hps5 - [HPS5_MOUSE]	3.61	4	1	1	3	-0.15	0.15	-0.32	-0.02
G3UZ60	Small ubiquitin-related modifier 3 OS=Mus musculus GN=Sumo3 PE=2 SV=1 - [G3UZ60_MOUSE]	28.00	7	1	2	26	-0.09	-0.17	-0.09	-0.17
Q08331	Calretinin OS=Mus musculus GN=Calb2 PE=1 SV=3 - [CALB2_MOUSE]	57.20	2	15	15	178	-1.01	-0.88	-1.00	-0.91
O35955	Proteasome subunit beta type-10 OS=Mus musculus GN=Psmb10 PE=1 SV=1 - [PSB10_MOUSE]	35.90	1	7	7	69	-0.13	-0.10	-0.15	-0.13

D3Z091	Protein Pik3c2b (Fragment) OS=Mus musculus GN=Pik3c2b PE=2 SV=1 - [D3Z091_MOUSE]	5.48	2	1	1	1	-0.54	-0.24	-0.33	-0.04
Q9DCD0	6-phosphogluconate dehydrogenase, decarboxylating OS=Mus musculus GN=Pgd PE=2 SV=3 - [6PGD_MOUSE]	60.46	2	26	27	620	-0.13	-0.11	-0.07	-0.11
F7DBB3	Protein Ahnak2 (Fragment) OS=Mus musculus GN=Ahnak2 PE=2 SV=1 - [F7DBB3_MOUSE]	30.39	2	7	17	73	-0.57	-0.48	-1.07	-1.02
P31786	Acyl-CoA-binding protein OS=Mus musculus GN=Dbi PE=1 SV=2 - [ACBP_MOUSE]	58.62	5	5	5	207	-0.16	-0.12	-0.29	-0.22
Q8VC28	Aldo-keto reductase family 1 member C13 OS=Mus musculus GN=Akr1c13 PE=1 SV=2 - [AK1CD_MOUSE]	68.11	2	7	19	258	-0.41	-0.34	-0.41	-0.40
Q8BFR4	N-acetylglucosamine-6-sulfatase OS=Mus musculus GN=Gns PE=2 SV=1 - [GNS_MOUSE]	14.34	1	7	7	17	-0.17	-0.47	-0.47	-0.72
Q01149	Collagen alpha-2(I) chain OS=Mus musculus GN=Col1a2 PE=2 SV=2 - [CO1A2_MOUSE]	14.07	1	12	12	43	-0.33	-0.33	-0.38	-0.37
P97471	Mothers against decapentaplegic homolog 4 OS=Mus musculus GN=Smad4 PE=1 SV=2 - [SMAD4_MOUSE]	6.53	1	3	3	6	0.96	0.63	0.74	0.41
Q8CG16	Complement C1r-A subcomponent OS=Mus musculus GN=C1ra PE=1 SV=1 - [C1RA_MOUSE]	6.36	2	3	3	14	-0.13	-1.03	-0.20	-0.70
O88322	Nidogen-2 OS=Mus musculus GN=Nid2 PE=1 SV=2 - [NID2_MOUSE]	18.03	1	19	20	105	-0.92	-0.79	-0.82	-0.75
P97347	Repetin OS=Mus musculus GN=Rptn PE=1 SV=2 - [RPTN_MOUSE]	1.07	1	1	1	1	-0.83	-0.90	-0.69	-0.75
Q8C6I2-3	Isoform 3 of Succinate dehydrogenase assembly factor 2, mitochondrial OS=Mus musculus GN=Sdhaf2 - [SDHF2_MOUSE]	17.14	2	1	1	2	-0.44	-0.08	-0.02	0.33
Q9WVS4	MAPK/MAK/MRK overlapping kinase OS=Mus musculus GN=Mok PE=1 SV=1 - [MOK_MOUSE]	3.10	5	1	2	5	-0.28	-0.05	-0.75	-0.53
Q8K2K6-3	Isoform 3 of Arf-GAP domain and FG repeat-containing protein 1 OS=Mus musculus GN=Agfg1 - [AGFG1_MOUSE]	11.32	4	4	5	14	-0.27	-0.09	-0.59	-0.32
O88952	Protein lin-7 homolog C OS=Mus musculus GN=Lin7c PE=1 SV=2 [LIN7C_MOUSE]	60.41	5	7	12	60	-0.15	-0.06	-0.16	-0.09

O70435	Proteasome subunit alpha type-3 OS=Mus musculus GN=Psma3 PE=1 SV=3 - [PSA3_MOUSE]	29.80	5	11	11	64	-0.40	-0.37	-0.33	-0.31
E9Q616	Protein Ahnak OS=Mus musculus GN=Ahnak PE=2 SV=1 - [E9Q616_MOUSE]	76.03	4	251	255	3185	-0.16	-0.06	-0.23	-0.12
Q9JJF9	Signal peptide peptidase like 2A OS=Mus musculus GN=Sppl2a PE=1 SV=2 - [SPP2A_MOUSE]	1.91	1	1	1	6	-0.02	-0.37	0.14	-0.21
B2RW07	Ataxin 7-like 1 OS=Mus musculus GN=Atxn7l1 PE=2 SV=1 - [B2RW07_MOUSE]	5.55	5	2	2	2	-0.45	0.14	-0.67	-0.08
Q7TSQ8	Pyruvate dehydrogenase phosphatase regulatory subunit, mitochondrial OS=Mus musculus GN=Pdpr PE=2 SV=1 - [PDPR_MOUSE]	10.36	3	8	8	19	-0.43	-0.60	-0.36	-0.56
P02469	Laminin subunit beta-1 OS=Mus musculus GN=Lamb1 PE=1 SV=3 - [LAMB1_MOUSE]	25.98	3	40	43	306	-0.99	-0.91	-0.92	-0.88
P21107-2	Isoform 2 of Tropomyosin alpha-3 chain OS=Mus musculus GN=Tpm3 - [TPM3_MOUSE]	62.10	2	3	22	1261	-0.35	-0.27	-0.33	-0.30
P17095-1	Isoform HMG-Y of High mobility group protein HMG-I/HMG-Y OS=Mus musculus GN=Hmga1 - [HMGA1_MOUSE]	22.92	1	1	3	19	0.55	0.57	0.51	0.42
P14733	Lamin-B1 OS=Mus musculus GN=Lmnb1 PE=1 SV=3 - [LMNB1_MOUSE]	57.65	1	33	38	723	-0.47	-0.39	-0.48	-0.46
Q5NCM6	Epsin-2 OS=Mus musculus GN=Epn2 PE=2 SV=1 - [Q5NCM6_MOUSE]	13.00	7	4	4	20	-0.28	-0.25	-0.51	-0.50
Q5NCR9	Nuclear speckle splicing regulatory protein 1 OS=Mus musculus GN=Nsrp1 PE=1 SV=1 [NSRP1_MOUSE]	2.40	1	1	1	3	0.14	0.63	-0.07	0.41
Q9DBG9	Tax1-binding protein 3 OS=Mus musculus GN=Tax1bp3 PE=1 SV=1 - [TX1B3_MOUSE]	53.23	2	3	3	31	-0.34	-0.25	-0.32	-0.36
P55194	SH3 domain-binding protein 1 OS=Mus musculus GN=Sh3bp1 PE=1 SV=2 - [3BP1_MOUSE]	13.14	4	5	5	11	-0.11	-0.27	-0.08	-0.55
F2Z429	Tudor domain-containing protein 6 OS=Mus musculus GN=Tdrd6 PE=2 SV=1 [F2Z429_MOUSE]	0.66	3	1	1	1	-0.39	-0.50	-0.34	-0.45
Q9DAS9	Guanine nucleotide-binding protein G(I)/G(S)/G(O) subunit gamma-12 OS=Mus musculus GN=Gng12 PE=1 SV=3 - [GBG12_MOUSE]	59.72	1	4	4	48	-0.36	-0.23	-0.40	-0.28

O35226	26S proteasome non-ATPase regulatory subunit 4 OS=Mus musculus GN=Psmd4 PE=1 SV=1 - [PSMD4_MOUSE]	28.46	2	1	9	58	-0.81	-0.04	-0.37	-0.41
P19426-2	Isoform 2 of Negative elongation factor E OS=Mus musculus GN=Nelfe - [NELFE_MOUSE]	23.98	3	6	6	17	0.16	0.20	0.15	0.04
Q62418-3	Isoform 3 of Drebrin-like protein OS=Mus musculus GN=Dbnl - [DBNL_MOUSE]	37.27	3	12	12	186	-0.19	-0.18	-0.15	-0.05
Q8BHJ5	F-box-like/WD repeat-containing protein TBL1XR1 OS=Mus musculus GN=Tbl1xr1 PE=2 SV=1 - [TBL1R_MOUSE]	24.51	1	4	6	14	-0.45	-0.22	-0.82	-0.59
Q8BLY7	Hermansky-Pudlak syndrome 6 protein homolog OS=Mus musculus GN=Hps6 PE=1 SV=3 - [HPS6_MOUSE]	5.22	1	3	3	7	0.36	0.21	0.09	-0.07
Q69ZC8	Uncharacterized protein KIAA1704 OS=Mus musculus GN=Kiaa1704 PE=1 SV=2 - [K1704_MOUSE]	6.36	1	1	1	3	-0.02	-0.30	-0.21	-0.49
Q9CQK7	RWD domain-containing protein 1 OS=Mus musculus GN=Rwdd1 PE=1 SV=1 - [RWDD1_MOUSE]	8.23	1	3	3	9	-0.05	-0.07	-0.15	-0.17
D3Z3B0	Nicotinamide mononucleotide adenylyltransferase 3 OS=Mus musculus GN=Nmnat3 PE=2 SV=1 - [D3Z3B0_MOUSE]	8.89	3	1	1	2	-1.07	-0.58	-1.17	-0.69
G3X9N3	MCG16539 OS=Mus musculus GN=Pnmal2 PE=4 SV=1 - [G3X9N3_MOUSE]	1.51	1	1	1	1	0.26	0.34	-0.81	-0.74
Q62395	Trefoil factor 3 OS=Mus musculus GN=Tff3 PE=1 SV=2 - [TFF3_MOUSE]	19.75	1	1	1	122	0.05	0.12	-0.05	0.02
Q61079	Single-minded homolog 2 OS=Mus musculus GN=Sim2 PE=1 SV=1 - [SIM2_MOUSE]	1.52	1	1	1	1	-0.05	0.01	-0.11	-0.05
Q61233	Plastin-2 OS=Mus musculus GN=Lcp1 PE=1 SV=4 - [PLSL_MOUSE]	62.84	5	27	32	532	0.16	0.15	0.14	0.15
Q8WTY4	Anamorsin OS=Mus musculus GN=Ciapin1 PE=1 SV=1 - [CPIN1_MOUSE]	21.36	4	5	5	19	-0.13	-0.18	-0.13	-0.29
Q9EPB5	Serine hydrolase-like protein OS=Mus musculus GN=Serhl PE=2 SV=1 - [SERHL_MOUSE]	13.18	3	4	4	14	-0.82	-0.94	-0.55	-0.82
Q8VIG0-2	Isoform 2 of Zinc finger CCHC domain-containing protein 14 OS=Mus musculus GN=Zcchc14 - [ZCH14_MOUSE]	5.72	3	2	2	6	-0.96	-0.52	-1.00	-0.56
P15261	Interferon gamma receptor 1 OS=Mus musculus GN=Ifngr1 PE=1 SV=3 - [INGR1_MOUSE]	11.95	1	5	5	7	-0.09	-0.04	-0.22	-0.17

Q80VP0-2	Isoform 2 of Tectonin beta-propeller repeat-containing protein 1 OS=Mus musculus GN=Tecpr1 - [TCPR1_MOUSE]	3.51	2	2	2	3	-0.01	0.04	-0.19	-0.13
Q9CQA6	Coiled-coil-helix-coiled-coil-helix domain-containing protein 1 OS=Mus musculus GN=Chchd1 PE=2 SV=1 - [CHCH1_MOUSE]	9.32	1	1	1	2	-0.70	-0.62	-0.02	0.05
A2ALW5-2	Isoform 2 of DnaJ homolog subfamily C member 25 OS=Mus musculus GN=Dnajc25 [DJC25_MOUSE]	7.56	2	2	2	4	-0.07	0.19	-0.48	0.17
P09813	Apolipoprotein A-II OS=Mus musculus GN=Apoa2 PE=1 SV=2 - [APOA2_MOUSE]	18.63	1	2	2	20	-0.18	-0.26	-0.24	-0.21
P01642	Ig kappa chain V-V region L7 (Fragment) OS=Mus musculus GN=Gm10881 PE=1 SV=1 - [KV5A9_MOUSE]	10.43	1	1	1	3	-0.77	-1.01	-0.62	-0.84
A2AKW1	SH2 domain-containing adapter protein B (Fragment) OS=Mus musculus GN=Shb PE=2 SV=1 - [A2AKW1_MOUSE]	13.70	3	1	1	1	-0.40	-0.44	-0.52	-0.56
Q05A45	Olfr725 protein OS=Mus musculus GN=Olfr725 PE=2 SV=1 - [Q05A45_MOUSE]	2.18	1	1	1	5	-0.85	-1.15	-0.88	-1.19
Q9QZZ6	Dermatopontin OS=Mus musculus GN=Dpt PE=2 SV=1 - [DERM_MOUSE]	21.39	1	4	4	31	-1.15	-1.00	-1.11	-1.08
P55302	Alpha-2-macroglobulin receptor-associated protein OS=Mus musculus GN=Lrpap1 PE=1 SV=1 - [AMRP_MOUSE]	44.17	2	15	15	52	-0.64	-0.50	-0.60	-0.50
D3YYU8	Protein Obsl1 OS=Mus musculus GN=Obsl1 PE=2 SV=1 - [D3YYU8_MOUSE]	0.39	1	1	1	1	-0.13	-0.26	-0.32	-0.45
P70288	Histone deacetylase 2 OS=Mus musculus GN=Hdac2 PE=1 SV=1 - [HDAC2_MOUSE]	20.49	2	3	6	59	-0.38	-0.15	-0.38	-0.08
P18242	Cathepsin D OS=Mus musculus GN=Ctsd PE=1 SV=1 - [CATD_MOUSE]	30.00	3	11	11	111	-0.43	-0.43	-0.30	-0.35
D3Z5N5	1-phosphatidylinositol 3 phosphate 5-kinase OS=Mus musculus GN=Pikfyve PE=2 SV=2 - [D3Z5N5_MOUSE]	3.56	4	4	4	5	0.11	0.61	-0.58	-0.09
Q9D0S9	Histidine triad nucleotide-binding protein 2, mitochondrial OS=Mus musculus GN=Hint2 PE=2 SV=1 - [HINT2_MOUSE]	28.22	1	3	3	19	-0.58	-0.44	-0.92	-0.70

P97304	DNA-directed RNA polymerases I and III subunit RPAC2 OS=Mus musculus GN=Polr1d PE=2 SV=1 - [RPAC2_MOUSE]	10.53	1	1	1	1	-0.21	0.06	-0.81	-0.54
Q6P6M7	O-phosphoseryl-tRNA(Sec) selenium transferase OS=Mus musculus GN=Sepsecs PE=1 SV=2 - [SPCS_MOUSE]	5.75	4	2	2	17	-0.16	-0.25	-0.39	-0.39
P30204-2	Isoform II of Macrophage scavenger receptor types I and II OS=Mus musculus GN=Msr1 - [MSRE_MOUSE]	2.26	2	1	1	2	-0.47	-0.34	-0.43	-0.30
Q99MV1	Tudor domain-containing protein 1 OS=Mus musculus GN=Tdrd1 PE=1 SV=2 [TDRD1_MOUSE]	1.37	1	1	2	17	-0.33	-0.56	-0.33	-0.50
G3X8Z1	Calcium-activated chloride channel regulator 4 OS=Mus musculus GN=Clca6 PE=4 SV=1 - [G3X8Z1_MOUSE]	23.78	5	6	17	62	0.68	0.85	0.65	1.24
Q64012-2	Isoform 1 of RNA-binding protein Raly OS=Mus musculus GN=Raly - [RALY_MOUSE]	57.09	5	15	15	62	-0.34	-0.21	-0.23	-0.27
D3Z0K8	Thioredoxin reductase 2, mitochondrial OS=Mus musculus GN=Txnrd2 PE=2 SV=1 - [D3Z0K8_MOUSE]	15.89	7	5	5	12	-0.48	-0.35	-0.51	-0.38
Q8BVI4	Dihydropteridine reductase OS=Mus musculus GN=Qdpr PE=1 SV=2 - [DHPR_MOUSE]	38.59	4	7	7	84	-0.69	-0.53	-0.83	-0.70
Q6DFY2	Opioid binding protein/cell adhesion molecule-like OS=Mus musculus GN=Opcml PE=2 SV=1 - [Q6DFY2_MOUSE]	5.34	2	2	2	2	-0.50	-0.86	-0.52	-0.89
Q00612	Glucose-6-phosphate 1-dehydrogenase X OS=Mus musculus GN=G6pdx PE=1 SV=3 - [G6PD1_MOUSE]	42.52	3	17	21	233	0.09	0.11	-0.01	0.03
Q9WTQ5-2	Isoform 2 of A-kinase anchor protein 12 OS=Mus musculus GN=Akap12 - [AKA12_MOUSE]	34.07	2	29	29	170	-0.43	-0.34	-0.45	-0.38
Q8K2I4	Beta-mannosidase OS=Mus musculus GN=Manba PE=2 SV=1 - [MANBA_MOUSE]	8.65	2	7	7	13	-0.28	-0.14	-0.48	-0.49
P62983	Ubiquitin-40S ribosomal protein S27a OS=Mus musculus GN=Rps27a PE=1 SV=2 - [RS27A_MOUSE]	50.64	7	3	13	431	-0.11	0.01	-0.23	-0.03
P70441	Na(+)/H(+) exchange regulatory cofactor NHE-RF1 OS=Mus musculus GN=Slc9a3r1 PE=1 SV=3 - [NHRF1_MOUSE]	51.83	2	19	19	148	-0.29	-0.21	-0.39	-0.19

D3YXY5	DET1- and DDB1-associated protein 1 OS=Mus musculus GN=Dda1 PE=2 SV=1 - [D3YXY5_MOUSE]	7.00	2	1	1	1	-0.15	-0.45	-0.24	-0.54
Q99M15	Proline-serine-threonine phosphatase-interacting protein 2 OS=Mus musculus GN=Pstpip2 PE=1 SV=4 - [PPIP2_MOUSE]	13.47	1	4	4	9	-0.45	-0.11	-0.56	-0.05
P08121	Collagen alpha-1(III) chain OS=Mus musculus GN=Col3a1 PE=1 SV=4 - [CO3A1_MOUSE]	12.70	1	11	11	61	-0.41	-0.15	-0.45	-0.10
P70315	Wiskott-Aldrich syndrome protein homolog OS=Mus musculus GN=Was PE=1 SV=1 - [WASP_MOUSE]	12.50	1	4	5	19	0.07	-0.01	0.03	-0.06
Q62356	Follistatin-related protein 1 OS=Mus musculus GN=Fstl1 PE=1 SV=2 - [FSTL1_MOUSE]	5.56	1	2	2	2	-0.21	0.43	-0.36	0.28
Q8VCI5	Peroxisomal biogenesis factor 19 OS=Mus musculus GN=Pex19 PE=1 SV=1 - [PEX19_MOUSE]	41.47	2	9	9	65	-0.21	-0.27	-0.28	-0.41
P52479-2	Isoform 2 of Ubiquitin carboxyl-terminal hydrolase 10 OS=Mus musculus GN=Usp10 - [UBP10_MOUSE]	10.84	4	1	7	25	-0.65	-0.43	-0.42	-0.13
A2BE93	Protein SET (Fragment) OS=Mus musculus GN=Set PE=2 SV=1 - [A2BE93_MOUSE]	40.93	5	8	8	166	-0.15	-0.08	-0.20	-0.15
P46062	Signal-induced proliferation-associated protein 1 OS=Mus musculus GN=Sipa1 PE=2 SV=2 - [SIPA1_MOUSE]	1.06	2	1	1	1	-0.44	-0.98	-0.69	-1.23
P17047	Lysosome-associated membrane glycoprotein 2 OS=Mus musculus GN=Lamp2 PE=2 SV=2 - [LAMP2_MOUSE]	10.12	3	4	4	12	0.07	0.10	-0.17	-0.13
Q3TMX0	MCG4375, isoform CRA_b OS=Mus musculus GN=Sdcbp PE=2 SV=1 - [Q3TMX0_MOUSE]	12.08	5	3	3	7	-0.07	-0.56	0.04	-0.38
O89020	Afamin OS=Mus musculus GN=Afm PE=1 SV=2 - [AFAM_MOUSE]	10.03	3	5	5	9	0.03	-0.21	0.27	-0.53
Q60823	RAC-beta serine/threonine-protein kinase OS=Mus musculus GN=Akt2 PE=1 SV=1 - [AKT2_MOUSE]	9.36	4	1	3	8	-0.38	0.17	-0.37	0.18
P07742	Ribonucleoside-diphosphate reductase large subunit OS=Mus musculus GN=Rrm1 PE=1 SV=2 - [RIR1_MOUSE]	13.01	1	9	9	27	0.03	0.17	0.04	0.11
Q9D735	Uncharacterized protein C19orf43 homolog OS=Mus musculus PE=2 SV=1 - [CS043_MOUSE]	19.65	1	2	2	12	0.08	0.03	-0.06	-0.10

O35459	Delta(3,5)-Delta(2,4)-dienoyl-CoA isomerase, mitochondrial OS=Mus musculus GN=Ech1 PE=2 SV=1 - [ECH1_MOUSE]	41.90	2	14	14	114	-0.80	-0.65	-0.79	-0.70
D3Z479	Sorting nexin-15 OS=Mus musculus GN=Snx15 PE=2 SV=1 - [D3Z479_MOUSE]	4.78	3	1	2	2	-0.56	-0.33	-0.56	-0.33
Q31094	Class II histocompatibility antigen, M beta 1 chain OS=Mus musculus GN=H2-DMb1 PE=2 SV=2 - [Q31094_MOUSE]	12.64	4	3	3	5	-0.33	-0.20	-0.11	-0.10
O55137	Acyl-coenzyme A thioesterase 1 OS=Mus musculus GN=Acot1 PE=1 SV=1 - [ACOT1_MOUSE]	18.14	7	1	6	17	-0.56	-0.42	-0.66	-0.53
Q9Z2K1	Keratin, type I cytoskeletal 16 OS=Mus musculus GN=Krt16 PE=1 SV=3 [K1C16_MOUSE]	12.15	6	1	5	124	0.06	-0.05	-0.05	-0.16
Q91WE2	Protein FAM192A OS=Mus musculus GN=Fam192a PE=2 SV=1 - [F192A_MOUSE]	8.66	1	2	2	5	-0.15	-0.17	-0.05	-0.07
Q9D9D9	Uncharacterized protein C1orf189 homolog OS=Mus musculus PE=2 SV=2 - [CA189_MOUSE]	15.84	1	1	2	3	-0.06	-0.04	-0.04	-0.01
A2RSQ0	DENN domain-containing protein 5B OS=Mus musculus GN=Dennd5b PE=1 SV=2 - [DEN5B_MOUSE]	10.68	1	5	6	17	-0.14	-0.18	-0.42	-0.46
P09405	Nucleolin OS=Mus musculus GN=Ncl PE=1 SV=2 - [NUCL_MOUSE]	43.71	1	39	39	631	-0.02	0.08	-0.06	0.03
P97927	Laminin subunit alpha-4 OS=Mus musculus GN=Lama4 PE=1 SV=2 - [LAMA4_MOUSE]	23.13	6	32	35	237	-0.89	-0.82	-0.82	-0.84
Q6NZF1	Zinc finger CCCH domain-containing protein 11A OS=Mus musculus GN=Zc3h11a PE=1 SV=1 - [ZC11A_MOUSE]	17.93	1	10	10	19	0.00	0.02	-0.19	-0.06
Q9D823	60S ribosomal protein L37 OS=Mus musculus GN=Rpl37 PE=2 SV=3 [RL37_MOUSE]	26.80	1	5	5	8	0.13	-0.10	-0.36	-0.26
F6VUN6	MICAL C-terminal-like protein (Fragment) OS=Mus musculus GN=Micalcl PE=4 SV=1 - [F6VUN6_MOUSE]	1.57	2	1	1	1	-0.10	-0.15	-0.22	-0.26
P26645	Myristoylated alanine-rich C-kinase substrate OS=Mus musculus GN=Marcks PE=1 SV=2 - [MARCS_MOUSE]	49.84	1	6	7	43	-0.07	0.06	-0.11	0.13

F6YTG0	Enoyl-CoA hydratase domain-containing protein 2, mitochondrial (Fragment) OS=Mus musculus GN=Echdc2 PE=4 SV=1 - [F6YTG0_MOUSE]	11.35	5	2	2	11	-0.21	-0.43	-0.33	-0.58
Q6P9K8-2	Isoform 2 of Caskin-1 OS=Mus musculus GN=Caskin1 - [CSKI1_MOUSE]	1.76	3	2	2	2	-0.86	-0.93	-0.93	-1.01
Q810V0	U3 small nucleolar ribonucleoprotein protein MPP10 OS=Mus musculus GN=Mphosph10 PE=1 SV=2 - [MPP10_MOUSE]	2.35	1	2	2	3	-0.17	-0.02	-0.03	0.41
Q9CQ71	Replication protein A 14 kDa subunit OS=Mus musculus GN=Rpa3 PE=2 SV=1 - [RFA3_MOUSE]	15.70	1	1	1	4	-0.67	0.66	-0.94	0.41
G5E898	Periplakin OS=Mus musculus GN=Ppl PE=4 SV=1 - [G5E898_MOUSE]	5.25	2	8	9	16	-0.41	-0.40	-0.41	-0.35
Q8R2U6	Diphosphoinositol polyphosphate phosphohydrolase 2 OS=Mus musculus GN=Nudt4 PE=1 SV=1 [NUDT4_MOUSE]	27.37	2	4	4	20	-0.30	-0.26	-0.53	-0.41
Q3UHC0	Trinucleotide repeat-containing gene 6C protein OS=Mus musculus GN=Tnrc6c PE=1 SV=2 - [TNR6C_MOUSE]	4.67	3	3	4	5	-1.37	-0.97	-1.46	-1.06
D3Z6Q9	Bridging integrator 2 OS=Mus musculus GN=Bin2 PE=2 SV=1 - [BIN2_MOUSE]	17.18	1	5	7	20	-0.01	0.20	0.08	0.04
Q76N33	AMSH-like protease OS=Mus musculus GN=Stambpl1 PE=2 SV=1 - [STALP_MOUSE]	6.88	2	2	2	3	0.09	0.07	-0.25	-0.27
P21107	Tropomyosin alpha-3 chain OS=Mus musculus GN=Tpm3 PE=1 SV=3 - [TPM3_MOUSE]	63.16	6	4	26	1182	-0.75	-0.73	-0.43	-0.38
Q00422	GA-binding protein alpha chain OS=Mus musculus GN=Gabpa PE=1 SV=2 - [GABPA_MOUSE]	3.30	1	2	2	4	-0.12	0.12	-0.19	0.05
Q60994	Adiponectin OS=Mus musculus GN=Adipoq PE=1 SV=2 - [ADIPO_MOUSE]	15.79	2	4	4	48	-0.87	-0.86	-0.94	-0.82
D3Z131	THO complex subunit 6 homolog OS=Mus musculus GN=Thoc6 PE=2 SV=1 - [D3Z131_MOUSE]	11.82	4	2	2	3	-0.30	-0.84	-0.34	-0.88
Q8BYK6	YTH domain family protein 3 OS=Mus musculus GN=Ythdf3 PE=1 SV=2 - [YTHD3_MOUSE]	23.59	5	8	10	21	0.14	0.18	-0.17	0.00
P17439	Glucosylceramidase OS=Mus musculus GN=Gba PE=1 SV=1 - [GLCM_MOUSE]	15.34	1	6	6	20	-0.59	-0.53	-0.55	-0.41
Q63850	Nuclear pore glycoprotein p62 OS=Mus musculus GN=Nup62 PE=1 SV=2 - [NUP62_MOUSE]	11.79	1	4	4	44	-0.34	-0.16	-0.04	0.00

P14231	Sodium/potassium-transporting ATPase subunit beta-2 OS=Mus musculus GN=Atp1b2 PE=1 SV=2 - [AT1B2_MOUSE]	9.31	1	1	1	1	0.09	0.41	-0.29	0.03
Q8BHS3	Pre-mRNA-splicing factor RBM22 OS=Mus musculus GN=Rbm22 PE=2 SV=1 - [RBM22_MOUSE]	11.67	1	2	4	6	-0.22	-0.41	-0.15	-0.35
Q8C3V4	Signal transducer and activator of transcription 1 OS=Mus musculus GN=Stat1 PE=2 SV=1 - [Q8C3V4_MOUSE]	39.79	2	27	27	224	0.22	0.13	0.23	0.16
Q91XT6	Tandem C2 domains nuclear protein OS=Mus musculus GN=Tc2n PE=1 SV=2 - [TAC2N_MOUSE]	1.43	1	1	1	1	0.22	0.40	0.03	0.20
Q9JKF7	39S ribosomal protein L39, mitochondrial OS=Mus musculus GN=Mrpl39 PE=2 SV=4 - [RM39_MOUSE]	16.67	1	6	6	10	-0.16	-0.29	-0.31	-0.43
Q8R1G6	PDZ and LIM domain protein 2 OS=Mus musculus GN=Pdlim2 PE=1 SV=1 - [PDLI2_MOUSE]	22.64	3	6	6	20	-0.76	-0.75	-0.59	-0.55
Q8BYK5-4	Isoform 4 of Phosphatase and actin regulator 3 OS=Mus musculus GN=Phactr3 - [PHAR3_MOUSE]	1.16	5	1	1	1	-0.62	-0.02	-0.46	0.13
Q3TLD5-2	Isoform 2 of Unconventional prefoldin RPB5 interactor OS=Mus musculus GN=Uri1 - [RMP_MOUSE]	2.41	2	1	1	2	-0.11	0.05	-0.35	-0.19
Q8VCG1	Deoxyuridine triphosphatase, isoform CRA_b OS=Mus musculus GN=Dut PE=2 SV=1 - [Q8VCG1_MOUSE]	37.00	2	6	6	27	0.04	0.20	0.08	-0.04
Q9WUS4-2	Isoform 2 of Gap junction alpha-10 protein OS=Mus musculus GN=Gja10 - [CXA10_MOUSE]	1.83	2	1	1	2	-0.32	-0.38	-0.48	-0.53
P15092-2	Isoform 2 of Interferon-activable protein 204 OS=Mus musculus GN=Ifi204 - [IFI4_MOUSE]	8.63	3	6	6	11	-0.10	-0.05	-0.05	-0.02
D3YYA0	Set1/Ash2 histone methyltransferase complex subunit ASH2 OS=Mus musculus GN=Ash2l PE=2 SV=1 [D3YYA0_MOUSE]	11.98	5	5	5	7	-0.26	-0.72	-0.23	-0.92
D3YY16	Glia maturation factor gamma OS=Mus musculus GN=Gmfg PE=2 SV=1 - [D3YY16_MOUSE]	26.73	3	2	3	12	-0.03	0.02	-0.29	-0.33
P29595	NEDD8 OS=Mus musculus GN=Nedd8 PE=1 SV=2 - [NEDD8_MOUSE]	32.10	1	3	4	32	-0.15	-0.14	-0.32	-0.30

Q8R5L1	Complement component 1 Q subcomponent-binding protein, mitochondrial OS=Mus musculus GN=C1qbp PE=2 SV=1 - [Q8R5L1_MOUSE]	31.90	2	6	6	197	-0.31	-0.16	-0.36	-0.31
A2AAA9	C-type mannose receptor 2 OS=Mus musculus GN=Mrc2 PE=2 SV=1 - [A2AAA9_MOUSE]	5.13	2	3	3	7	-0.25	-0.15	-0.09	-0.06
O09164	Extracellular superoxide dismutase [Cu-Zn] OS=Mus musculus GN=Sod3 PE=1 SV=1 - [SODE_MOUSE]	13.55	1	4	4	13	-0.06	-0.20	0.02	-0.10
Q8BIQ6	MCG23335 OS=Mus musculus GN=Zfp947 PE=2 SV=1 - [Q8BIQ6_MOUSE]	8.70	119	1	2	6	-0.71	-0.83	-0.47	-0.59
Q8JZZ0	UDP-glucuronosyltransferase 3A2 OS=Mus musculus GN=Ugt3a2 PE=2 SV=2 - [UD3A2_MOUSE]	9.94	1	2	2	2	-0.85	-0.92	-0.84	-0.91
P47867-2	Isoform 2 of Secretogranin-3 OS=Mus musculus GN=Scg3 - [SCG3_MOUSE]	7.73	2	3	4	11	-1.51	-1.38	-1.36	-1.37
Q8BK67	Protein RCC2 OS=Mus musculus GN=Rcc2 PE=2 SV=1 - [RCC2_MOUSE]	26.15	2	12	12	43	-0.06	-0.11	-0.05	-0.19
D3YU60	Microsomal glutathione S-transferase 1 OS=Mus musculus GN=Mgst1 PE=2 SV=1 - [D3YU60_MOUSE]	12.75	3	1	1	7	-0.24	-0.33	-0.52	-0.53
Q9D6N1	Carbonic anhydrase 13 OS=Mus musculus GN=Ca13 PE=1 SV=1 - [CAH13_MOUSE]	20.99	1	5	5	14	-0.42	-0.41	-0.35	-0.38
E9QPG8	Deleted in malignant brain tumors 1 protein OS=Mus musculus GN=Dmbt1 PE=2 SV=1 - [E9QPG8_MOUSE]	6.94	5	9	9	35	-0.02	0.24	-0.01	0.10
Q8QZR3	Carboxylesterase 6 OS=Mus musculus GN=Ces2a PE=2 SV=1 [Q8QZR3_MOUSE]	36.56	5	11	13	140	-0.64	-0.49	-0.64	-0.46
Q3UXD9	Peroxisomal trans-2-enoyl-CoA reductase OS=Mus musculus GN=Pecr PE=2 SV=1 - [Q3UXD9_MOUSE]	10.38	3	2	2	5	-0.16	-0.24	-0.27	-0.17
Q3UW98	Chloride channel calcium activated 7 OS=Mus musculus GN=AI747448 PE=2 SV=1 - [Q3UW98_MOUSE]	16.43	2	3	14	42	-0.46	-0.78	-0.30	-0.62
Q8CHY3	Dymeclin OS=Mus musculus GN=Dym PE=2 SV=1 - [DYM_MOUSE]	2.09	2	2	2	3	-0.41	0.31	-0.76	-0.04
Q8BXR9-2	Isoform 2 of Oxysterol-binding protein-related protein 6 OS=Mus musculus GN=Osbpl6 - [OSBL6_MOUSE]	3.23	7	2	2	4	-0.01	-0.47	-0.74	-1.20

O89053	Coronin-1A OS=Mus musculus GN=Coro1a PE=1 SV=5 - [COR1A_MOUSE]	42.08	5	17	18	172	0.08	0.06	0.14	0.12
P07309	Transthyretin OS=Mus musculus GN=Ttr PE=1 SV=1 - [TTHY_MOUSE]	6.80	1	1	1	4	-0.54	-0.65	-0.10	-0.22
Q9CZH8-2	Isoform 2 of Coiled-coil domain-containing protein 77 OS=Mus musculus GN=Ccdc77 - [CCD77_MOUSE]	3.18	10	1	2	9	-0.70	-0.30	-0.83	-0.43
Q499E4-2	Isoform 2 of Zinc finger protein DZIP1L OS=Mus musculus GN=Dzip1l - [DZI1L_MOUSE]	6.34	3	2	3	3	-0.12	-0.18	0.04	-0.02
Q8CAB2	Protein AI504432 OS=Mus musculus GN=AI504432 PE=2 SV=1 - [Q8CAB2_MOUSE]	5.71	1	1	1	1	-0.40	-0.28	0.11	0.23
Q60865	Caprin-1 OS=Mus musculus GN=Caprin1 PE=1 SV=2 - [CAPR1_MOUSE]	19.09	4	9	9	80	0.14	0.22	0.05	0.12
Q8K2J1	Car12 protein OS=Mus musculus GN=Car12 PE=2 SV=1 - [Q8K2J1_MOUSE]	5.52	2	2	2	7	0.01	0.04	0.05	0.13
P49312	Heterogeneous nuclear ribonucleoprotein A1 OS=Mus musculus GN=Hnrnpa1 PE=1 SV=2 - [ROA1_MOUSE]	53.44	4	13	17	339	-0.17	-0.08	-0.24	-0.15
Q64152-2	Isoform 2 of Transcription factor BTF3 OS=Mus musculus GN=Btf3 - [BTF3_MOUSE]	38.89	2	7	7	41	0.33	0.21	0.10	-0.15
Q61462-2	Isoform 2 of Cytochrome b-245 light chain OS=Mus musculus GN=Cyba - [CY24A_MOUSE]	32.32	2	3	3	6	0.16	0.09	0.12	0.15
P58058	NAD kinase OS=Mus musculus GN=Nadk PE=1 SV=2 - [NADK_MOUSE]	10.25	4	4	4	6	-0.36	-0.42	-0.19	-0.41
D3Z375	Astrocytic phosphoprotein PEA-15 (Fragment) OS=Mus musculus GN=Pea15a PE=2 SV=1 - [D3Z375_MOUSE]	39.13	2	2	2	17	-0.32	-0.24	-0.22	-0.30
Q61152	Tyrosine-protein phosphatase non-receptor type 18 OS=Mus musculus GN=Ptpn18 PE=1 SV=1 - [PTN18_MOUSE]	6.40	1	2	2	5	-0.18	-0.11	-0.22	-0.18
Q6ZPY7-2	Isoform 2 of Lysine-specific demethylase 3B OS=Mus musculus GN=Kdm3b - [KDM3B_MOUSE]	1.69	3	2	2	2	-0.51	-0.32	-0.26	-0.07
Q9CQ01	Ribonuclease T2 OS=Mus musculus GN=Rnaset2 PE=2 SV=1 - [RNT2_MOUSE]	15.06	1	4	4	23	-0.16	-0.03	-0.24	-0.18
Q99J09	Methylosome protein 50 OS=Mus musculus GN=Wdr77 PE=1 SV=1 - [MEP50_MOUSE]	14.04	3	3	3	9	-0.48	0.21	-0.51	-0.48

P51125-3	Isoform 3 of Calpastatin OS=Mus musculus GN=Cast - [ICAL_MOUSE]	49.46	8	26	26	288	-0.17	-0.13	-0.23	-0.13
Q7TN29	Stromal membrane-associated protein 2 OS=Mus musculus GN=Smap2 PE=1 SV=1 - [SMAP2_MOUSE]	17.76	2	5	5	10	-0.65	-0.63	-0.74	-0.73
Q99LB4	Capping protein (Actin filament), gelsolin-like OS=Mus musculus GN=Capg PE=1 SV=1 - [Q99LB4_MOUSE]	39.26	6	10	10	185	0.00	-0.08	-0.01	-0.03
P13597-2	Isoform 2 of Intercellular adhesion molecule 1 OS=Mus musculus GN=Icam1 - [ICAM1_MOUSE]	4.52	2	2	2	5	-0.36	0.12	-0.20	0.28
Q5DID3-3	Isoform 3 of Uromodulin-like 1 OS=Mus musculus GN=Umodl1 - [UROL1_MOUSE]	3.24	3	2	2	2	-0.57	-0.53	-1.02	-0.97
Q3U1U4	Integrin alpha-M OS=Mus musculus GN=Itgam PE=2 SV=1 [Q3U1U4_MOUSE]	7.31	7	1	8	14	0.23	0.61	-0.02	0.36
Q91WG4	Elongator complex protein 2 OS=Mus musculus GN=Elp2 PE=1 SV=1 - [ELP2_MOUSE]	2.41	2	2	2	6	-0.86	-1.71	-1.09	-2.04
M0QW65	Asparagine synthetase domain-containing protein 1 OS=Mus musculus GN=Asnsd1 PE=4 SV=1 - [M0QW65_MOUSE]	45.24	1	1	1	2	-0.40	-0.14	-0.64	-0.38
Q8BP00-2	Isoform 2 of IQ calmodulin-binding motif-containing protein 1 OS=Mus musculus GN=Iqcb1 - [IQCB1_MOUSE]	2.35	2	1	1	1	-0.91	-0.48	-0.62	-0.20
P59997-2	Isoform 2 of Lysine-specific demethylase 2A OS=Mus musculus GN=Kdm2a - [KDM2A_MOUSE]	5.87	5	2	2	4	0.13	0.06	0.04	-0.28
A8Y5J3	Integrator complex subunit 11 OS=Mus musculus GN=Cpsf3l PE=2 SV=1 - [A8Y5J3_MOUSE]	3.63	2	2	2	4	0.30	0.06	0.21	-0.02
Q9CZR2	N-acetylated-alpha-linked acidic dipeptidase 2 OS=Mus musculus GN=Naalad2 PE=1 SV=2 - [NALD2_MOUSE]	9.46	2	5	5	10	-0.68	-0.87	-0.80	-0.98
P54116	Erythrocyte band 7 integral membrane protein OS=Mus musculus GN=Stom PE=1 SV=3 - [STOM_MOUSE]	33.80	1	6	6	14	-0.72	-0.73	-0.50	-0.51
G5E895	MCG142264, isoform CRA_b OS=Mus musculus GN=Akr1b10 PE=4 SV=1 - [G5E895_MOUSE]	38.29	2	4	12	89	0.09	0.28	-0.07	0.11
Q9CQF8	Ribosomal protein 63, mitochondrial OS=Mus musculus GN=Mrp63 PE=2 SV=1 - [RT63_MOUSE]	27.45	1	3	3	12	0.03	0.06	-0.23	-0.20

Q8CJF7	Protein ELYS OS=Mus musculus GN=Ahctf1 PE=1 SV=1 - [ELYS_MOUSE]	7.09	2	7	9	12	-0.79	-0.86	-1.26	-1.34
H3BL88	Protein 9930021J03Rik (Fragment) OS=Mus musculus GN=9930021J03Rik PE=4 SV=1 - [H3BL88_MOUSE]	1.12	3	2	3	7	-0.29	-1.13	-0.83	-1.67
Q7TT16	Inositol polyphosphate multikinase OS=Mus musculus GN=Ipmk PE=2 SV=1 - [IPMK_MOUSE]	4.29	2	1	1	1	-0.07	-0.18	-0.03	-0.14
O54998	Peptidyl-prolyl cis-trans isomerase FKBP7 OS=Mus musculus GN=Fkbp7 PE=1 SV=1 - [FKBP7_MOUSE]	3.67	1	1	1	2	-1.05	-0.55	-0.64	-0.14
Q9BCZ4	Selenoprotein S OS=Mu musculus GN=Vimp PE=2 SV=3 - [SELS_MOUSE]	8.42	1	1	1	1	0.35	-0.23	-0.35	-0.93
Q08731	Lithostathine-2 OS=Mus musculus GN=Reg2 PE=2 SV=1 - [LIT2_MOUSE]	24.28	1	3	3	13	0.61	0.29	1.14	0.61
E9PVU7	cAMP-specific 3',5'-cyclic phosphodiesterase 4D OS=Mus musculus GN=Pde4d PE=2 SV=1 [E9PVU7_MOUSE]	5.98	13	3	3	12	-0.75	-0.91	-0.86	-1.02
P29351	Tyrosine-protein phosphatase non-receptor type 6 OS=Mus musculus GN=Ptpn6 PE=1 SV=2 [PTN6_MOUSE]	37.82	5	19	20	88	0.09	-0.02	0.06	-0.01
Q921W7	Putative uncharacterized protein Tes OS=Mus musculus GN=Tes PE=2 SV=2 - [Q921W7_MOUSE]	50.36	6	17	21	115	-0.39	-0.45	-0.45	-0.42
Q8BJN4	Fibronectin type III domain-containing protein 9 OS=Mus musculus GN=Fndc9 PE=2 SV=1 - [FNDC9_MOUSE]	6.19	1	1	1	4	-0.95	-0.72	-1.06	-0.83
B1AYL0	Sodium channel protein type 9 subunit alpha OS=Mus musculus GN=Scn9a PE=2 SV=1 [B1AYL0_MOUSE]	0.66	4	1	1	1	0.12	-0.23	-0.14	-0.50
Q08AU7	Myeloid-associated differentiation marker-like protein 2 OS=Mus musculus GN=Myadml2 PE=2 SV=2 - [MADL2_MOUSE]	6.19	1	1	1	1	-0.39	-0.41	0.02	0.00
D3Z4C0	FERM, RhoGEF and pleckstrin domain-containing protein 2 OS=Mus musculus GN=Farp2 PE=2 SV=1 [D3Z4C0_MOUSE]	4.15	1	1	3	4	-0.20	0.21	-0.81	-0.41
Q923A2	Protein Spindly OS=Mus musculus GN=Spdl1 PE=1 SV=2 [SPDLY_MOUSE]	0.99	1	1	1	1	0.00	0.12	0.18	0.29

Q8QZR5	Alanine aminotransferase 1 OS=Mus musculus GN=Gpt PE=2 SV=3 - [ALAT1_MOUSE]	21.57	1	9	10	30	-1.46	-1.32	-1.65	-1.45
Q5SWP3	NAC-alpha domain-containing protein 1 OS=Mus musculus GN=Nacad PE=1 SV=1 - [NACAD_MOUSE]	0.86	1	1	1	2	-0.23	-0.06	-0.47	-0.30
D3Z7B5	Protein C330027C09Rik OS=Mus musculus GN=C330027C09Rik PE=2 SV=1 - [D3Z7B5_MOUSE]	1.43	2	1	1	2	-1.12	-1.40	-0.48	-0.76
O54951	Semaphorin-6B OS=Mus musculus GN=Sema6b PE=2 SV=1 - [SEM6B_MOUSE]	1.58	1	1	1	1	-0.20	-0.17	-0.09	-0.06
Q9Z2Q6	Septin-5 OS=Mus musculus GN=Sept5 PE=1 SV=2 - [SEPT5_MOUSE]	32.79	1	9	9	52	-0.23	-0.12	-0.17	-0.12
P52800	Ephrin-B2 OS=Mus musculus GN=Efnb2 PE=1 SV=1 - [EFNB2_MOUSE]	5.95	1	2	2	5	-0.37	-0.19	-0.39	-0.18
D3Z5I9	RNA-binding protein 33 OS=Mus musculus GN=Rbm33 PE=2 SV=1 - [D3Z5I9_MOUSE]	1.34	2	1	2	6	0.00	-0.10	-0.07	-0.18
P24270	Catalase OS=Mus musculus GN=Cat PE=1 SV=4 - [CATA_MOUSE]	51.80	2	22	22	187	-0.53	-0.51	-0.59	-0.54
Q6PE01	U5 small nuclear ribonucleoprotein 40 kDa protein OS=Mus musculus GN=Snrnp40 PE=2 SV=1 - [SNR40_MOUSE]	21.51	1	5	5	14	-0.37	-0.41	-0.38	-0.62
Q7TMF2	3'-5' exoribonuclease 1 OS=Mus musculus GN=Eri1 PE=1 SV=2 - [ERI1_MOUSE]	8.12	1	1	2	3	0.12	0.56	-0.56	-0.13
Q9JMH6	Thioredoxin reductase 1, cytoplasmic OS=Mus musculus GN=Txnrd1 PE=1 SV=3 - [TRXR1_MOUSE]	23.65	2	13	13	174	-0.49	-0.49	-0.35	-0.43
Q9Z2H7	PDZ domain-containing protein GIPC2 OS=Mus musculus GN=Gipc2 PE=1 SV=1 - [GIPC2_MOUSE]	32.17	1	9	9	48	-0.38	-0.21	-0.44	-0.42
E9Q4Y4	Protein Cep192 OS=Mus musculus GN=Cep192 PE=2 SV=1 - [E9Q4Y4_MOUSE]	0.91	1	2	2	2	-0.58	-0.55	-0.85	-0.82
P39039	Mannose-binding protein A OS=Mus musculus GN=Mbl1 PE=2 SV=1 - [MBL1_MOUSE]	5.86	1	2	2	3	-0.67	-0.76	-0.40	-0.49
Q8K262	Placenta-specific protein 9 OS=Mus musculus GN=Plac9 PE=2 SV=1 - [PLAC9_MOUSE]	10.19	1	1	2	5	-0.77	-0.54	-0.78	-0.55
E0CX48	Gap junction protein (Fragment) OS=Mus musculus GN=Gjb6 PE=2 SV=1 - [E0CX48_MOUSE]	5.78	2	1	1	1	-0.41	-0.62	-0.44	-0.66

Q9QUT0	Ammonium transporter Rh type A OS=Mus musculus GN=Rhag PE=2 SV=1 - [RHAG_MOUSE]	4.11	1	1	1	1	0.85	0.33	0.41	-0.10
P50285	Dimethylaniline monooxygenase [N-oxide-forming] 1 OS=Mus musculus GN=Fmo1 PE=1 SV=1 [FMO1_MOUSE]	4.89	1	2	2	3	-1.49	-1.37	-1.33	-1.22
E9QNP1	Microtubule-actin cross-linking factor 1 OS=Mus musculus GN=Macf1 PE=4 SV=2 - [E9QNP1_MOUSE]	9.78	8	2	40	93	-0.73	-0.41	-0.19	0.13
P54310	Hormone-sensitive lipase OS=Mus musculus GN=Lipe PE=1 SV=2 - [LIPS_MOUSE]	10.01	3	7	7	15	-1.19	-1.20	-1.12	-1.24
P48356-5	Isoform E of Leptin receptor OS=Mus musculus GN=Lepr - [LEPR_MOUSE]	2.24	5	1	1	2	-0.57	-0.46	-0.72	-0.61
P16054	Protein kinase C epsilon type OS=Mus musculus GN=Prkce PE=1 SV=1 [KPCE_MOUSE]	3.26	1	1	2	2	-0.02	0.27	-0.51	-0.22
Q9CPW7	Zinc finger matrin-type protein 2 OS=Mus musculus GN=Zmat2 PE=2 SV=1 - [ZMAT2_MOUSE]	4.52	1	1	1	3	-0.47	0.29	-0.68	0.07
Q8BVG4	Dipeptidyl peptidase 9 OS=Mus musculus GN=Dpp9 PE=2 SV=2 - [DPP9_MOUSE]	14.27	1	2	9	25	0.02	0.12	-0.25	-0.15
Q9D0R8	Protein LSM12 homolog OS=Mus musculus GN=Lsm12 PE=1 SV=1 - [LSM12_MOUSE]	13.85	1	2	2	9	-0.15	-0.07	-0.38	-0.20
Q8BTE0	UPF0369 protein C6orf57 homolog OS=Mus musculus PE=2 SV=2 - [CF057_MOUSE]	25.00	1	2	2	5	-0.32	-0.22	-0.50	-0.40
Q9JJ00	Phospholipid scramblase 1 OS=Mus musculus GN=Plscr1 PE=2 SV=1 - [PLS1_MOUSE]	6.10	1	2	3	6	0.15	0.07	-0.04	0.05
Q9EST5	Acidic leucine-rich nuclear phosphoprotein 32 family member B OS=Mus musculus GN=Anp32b PE=1 SV=1 - [AN32B_MOUSE]	30.51	2	6	10	220	-0.20	-0.13	-0.32	-0.24
Q9CQU5	ZW10 interactor OS=Mus musculus GN=Zwint PE=2 SV=1 [ZWINT_MOUSE]	2.38	1	1	1	4	0.69	0.18	0.16	-0.36
Q921L8	Polypeptide N-acetylgalactosaminyltransferase 11 OS=Mus musculus GN=Galnt11 PE=2 SV=1 - [GLT11_MOUSE]	2.47	1	1	1	2	-0.04	-0.34	-0.51	-0.81
Q3UFS4	G patch domain-containing protein 11 OS=Mus musculus GN=Gpatch11 PE=2 SV=2 - [GPT11_MOUSE]	9.16	1	2	2	2	-0.11	-0.09	-0.29	-0.27

A2A6J4	Lymphocyte-specific protein 1 OS=Mus musculus GN=Lsp1 PE=2 SV=1 - [A2A6J4_MOUSE]	31.37	4	8	8	51	-0.20	-0.15	-0.16	-0.13
P26339	Chromogranin-A OS=Mus musculus GN=Chga PE=1 SV=1 - [CMGA_MOUSE]	16.41	1	5	5	19	-1.56	-1.52	-1.60	-1.55
Q8R015-2	Isoform 2 of Biogenesis of lysosome-related organelles complex 1 subunit 5 OS=Mus musculus GN=Bloc1s5 - [BL1S5_MOUSE]	17.16	2	2	2	4	0.05	0.56	-0.66	-0.15
Q5SUC9	Protein SCO1 homolog mitochondrial OS=Mus musculus GN=Sco1 PE=2 SV=1 - [SCO1_MOUSE]	10.21	2	3	3	6	-0.30	-0.08	-0.73	-0.52
D3YZD3	Actin filament-associated protein 1-like 2 (Fragment) OS=Mus musculus GN=Afap1l2 PE=2 SV=1 - [D3YZD3_MOUSE]	5.22	4	1	1	1	0.31	0.36	0.39	0.44
E9Q3E1	Aldehyde dehydrogenase OS=Mus musculus GN=Aldh3b2 PE=2 SV=1 - [E9Q3E1_MOUSE]	14.82	3	4	4	6	-0.91	-1.06	-1.32	-1.03
P0C0S6	Histone H2A.Z OS=Mus musculus GN=H2afz PE=1 SV=2 [H2AZ_MOUSE]	31.25	5	2	4	54	-0.61	-0.40	-0.80	-0.78
Q61878	Bone marrow proteoglycan OS=Mus musculus GN=Prg2 PE=1 SV=1 - [PRG2_MOUSE]	8.07	1	2	2	2	0.59	0.50	0.23	0.14
E9PYP1	Protein Ces1a OS=Mus musculus GN=Ces1a PE=4 SV=1 - [E9PYP1_MOUSE]	13.14	1	2	3	28	-1.04	-0.98	-1.13	-1.07
D3YZ21	Protein D430041D05Rik OS=Mus musculus GN=D430041D05Rik PE=2 SV=1 - [D3YZ21_MOUSE]	0.78	2	1	1	1	-0.82	-0.80	-1.11	-1.09
P32261	Antithrombin-III OS=Mus musculus GN=Serpinc1 PE=1 SV=1 - [ANT3_MOUSE]	8.60	1	3	3	24	-0.35	-0.49	-0.53	-0.43
Q9CWW7	CpG-binding protein OS=Mus musculus GN=Cxxc1 PE=2 SV=1 - [CXXC1_MOUSE]	16.21	1	4	4	11	0.11	-0.77	0.88	0.10
Q9JLI0	Aldo-keto reductase a OS=Mus musculus GN=Akr1c12 PE=2 SV=1 - [Q9JLI0_MOUSE]	67.49	2	4	17	326	-0.51	-0.57	-0.64	-0.55
A1A546	Intestine-specific homeobox OS=Mus musculus GN=Isx PE=2 SV=2 - [ISX_MOUSE]	5.42	2	1	1	1	-0.22	0.06	-1.07	-0.79
Q8VEE1	LIM and cysteine-rich domains protein 1 OS=Mus musculus GN=Lmcd1 PE=1 SV=1 - [LMCD1_MOUSE]	22.19	1	7	7	11	0.12	0.19	0.11	0.07

Q9JI10	Serine/threonine-protein kinase 3 OS=Mus musculus GN=Stk3 PE=1 SV=1 - [STK3_MOUSE]	16.90	3	3	7	23	0.01	0.13	-0.06	-0.13
Q8R310-2	Isoform 2 of Transmembrane and coiled-coil domains protein 3 OS=Mus musculus GN=Tmcc3 - [TMCC3_MOUSE]	6.05	2	2	2	2	0.09	0.41	-0.55	-0.24
Q6P4U0	Thrombospondin type-1 domain-containing protein 7B OS=Mus musculus GN=Thsd7b PE=1 SV=1 - [THS7B_MOUSE]	1.99	2	2	2	7	-0.57	-0.47	-0.77	-0.68
Q80XJ3	Tetratricopeptide repeat protein 28 OS=Mus musculus GN=Ttc28 PE=2 SV=3 - [TTC28_MOUSE]	4.12	1	5	6	8	-1.13	-1.15	-1.15	-1.17
P20181	Neurotrophin-3 OS=Mus musculus GN=Ntf3 PE=1 SV=1 - [NTF3_MOUSE]	3.10	2	1	1	9	-0.27	-0.17	-0.34	-0.19
Q04899	Cyclin-dependent kinase 18 OS=Mus musculus GN=Cdk18 PE=1 SV=1 - [CDK18_MOUSE]	8.87	31	3	4	24	-2.14	-2.15	-1.95	-1.96
Q8VCU2	Glycosylphosphatidylinositol specific phospholipase D1 OS=Mus musculus GN=Gpld1 PE=2 SV=1 - [Q8VCU2_MOUSE]	6.89	3	5	5	8	-0.65	-1.02	-0.52	-0.72
Q8K212	Phosphofurin acidic cluster sorting protein 1 OS=Mus musculus GN=Pacs1 PE=1 SV=2 [PACS1_MOUSE]	9.57	1	5	6	10	-0.78	-1.12	-1.01	-0.77
B7ZNH7	Collagen alpha-1(XIV) chain OS=Mus musculus GN=Col14a1 PE=2 SV=1 - [B7ZNH7_MOUSE]	26.92	5	37	37	216	-0.83	-0.79	-0.80	-0.76
Q9JJZ6	Krueppel-like factor 13 OS=Mus musculus GN=Klf13 PE=2 SV=2 [KLF13_MOUSE]	6.57	1	1	1	2	-0.29	-0.14	-0.26	-0.10
F6VIG0	Protein RMD5 homolog B (Fragment) OS=Mus musculus GN=Rmnd5b PE=4 SV=1 - [F6VIG0_MOUSE]	5.95	2	1	1	3	-0.04	-0.32	-0.18	-0.32
B5THE2	Maltase-glucoamylase OS=Mus musculus GN=Mgam PE=2 SV=1 - [B5THE2_MOUSE]	4.27	1	5	5	12	-0.27	-0.30	-0.26	-0.33
P16301	Phosphatidylcholine-sterol acyltransferase OS=Mus musculus GN=Lcat PE=1 SV=2 - [LCAT_MOUSE]	6.16	1	2	2	2	-0.11	0.05	0.17	0.33
Q3UII8	Metastasis-associated protein MTA3 OS=Mus musculus GN=Mta3 PE=2 SV=1 - [Q3UII8_MOUSE]	9.56	7	2	6	18	-0.11	0.16	-0.63	-0.47
Q3V1H3	Hephaestin-like protein 1 OS=Mus musculus GN=Hephl1 PE=2 SV=2 - [HPHL1_MOUSE]	4.75	1	2	3	4	-0.42	-0.31	-0.73	-0.62

H3BLI9	TSC22 domain family protein 1 OS=Mus musculus GN=Tsc22d1 PE=4 SV=1 - [H3BLI9_MOUSE]	31.40	10	1	3	16	-0.65	-0.72	-0.66	-0.65
Q925J9-4	Isoform 4 of Mediator of RNA polymerase II transcription subunit 1 OS=Mus musculus GN=Med1 - [MED1_MOUSE]	8.72	5	9	9	12	0.27	0.07	-0.30	-0.18
Q8BH35	Complement component C8 beta chain OS=Mus musculus GN=C8b PE=1 SV=1 - [CO8B_MOUSE]	13.07	2	6	6	26	-0.64	-0.63	-0.55	-0.52
P29788	Vitronectin OS=Mus musculus GN=Vtn PE=1 SV=2 - [VTNC_MOUSE]	12.55	1	5	5	13	-0.40	-0.37	-0.35	-0.46
Q3UY93	Melanin-concentrating hormone receptor 1 OS=Mus musculus GN=Mchr1 PE=2 SV=1 - [Q3UY93_MOUSE]	4.53	2	1	1	1	-0.14	-0.10	-0.46	-0.43
P01831	Thy-1 membrane glycoprotein OS=Mus musculus GN=Thy1 PE=1 SV=1 - [THY1_MOUSE]	22.84	1	3	3	19	-1.13	-0.88	-1.26	-1.01
Q61503	5'-nucleotidase OS=Mus musculus GN=Nt5e PE=1 SV=2 - [5NTD_MOUSE]	27.60	3	12	12	52	-1.48	-1.41	-1.43	-1.31
Q9R060	Cytosolic Fe-S cluster assembly factor NUBP1 OS=Mus musculus GN=Nubp1 PE=1 SV=1 - [NUBP1_MOUSE]	5.63	1	1	1	5	0.65	0.48	0.39	0.19
P32020	Non-specific lipid-transfer protein OS=Mus musculus GN=Scp2 PE=1 SV=3 - [NLTP_MOUSE]	30.71	3	17	17	133	-0.40	-0.29	-0.44	-0.34
Q7TT52	Protein phosphatase 1 regulatory subunit 14D OS=Mus musculus GN=Ppp1r14d PE=2 SV=2 - [PP14D_MOUSE]	27.40	2	4	4	9	0.17	0.07	0.25	0.12
B1AY85	Plexin domain-containing protein 2 OS=Mus musculus GN=Plxdc2 PE=2 SV=1 - [B1AY85_MOUSE]	1.89	2	1	1	2	-0.15	-0.31	-0.40	-0.56
G3UZY2	Thioredoxin (Fragment) OS=Mus musculus GN=Txn2 PE=3 SV=1 - [G3UZY2_MOUSE]	36.15	2	3	3	21	-0.08	-0.05	-0.34	-0.29
P51827-2	Isoform 2 of AF4/FMR2 family member 3 OS=Mus musculus GN=Aff3 - [AFF3_MOUSE]	2.31	5	1	1	1	0.54	0.08	0.19	-0.28
Q9D809	MCG22941 OS=Mus musculus GN=2200002D01Rik PE=4 SV=1 - [Q9D809_MOUSE]	28.95	1	3	3	11	-0.07	-0.46	-0.77	-0.47
Q9DAM1	Leucine-rich repeat-containing protein 34 OS=Mus musculus GN=Lrrc34 PE=2 SV=1 - [LRC34_MOUSE]	7.95	1	2	2	2	-0.53	-0.12	-0.33	0.08

F6RGK4	MMS19 nucleotide excision repair protein homolog (Fragment) OS=Mus musculus GN=Mms19 PE=2 SV=1 - [F6RGK4_MOUSE]	5.59	6	2	2	6	-0.22	-0.12	-0.38	-0.19
Q99LC2	Cleavage stimulation factor subunit 1 OS=Mus musculus GN=Cstf1 PE=2 SV=1 - [CSTF1_MOUSE]	9.05	2	3	3	9	-0.31	-0.15	-0.55	-0.39
Q9QZC2	Plexin-C1 OS=Mus musculus GN=Plxnc1 PE=1 SV=1 - [PLXC1_MOUSE]	1.52	1	2	2	2	-0.18	-0.20	0.04	0.03
Q4LDF6	Protein Cfhr2 (Precursor) OS=Mus musculus GN=Cfhr2 PE=2 SV=1 - [Q4LDF6_MOUSE]	22.59	1	1	5	25	-0.68	-0.54	-0.58	-0.44
Q8BQ46	Protein Taf15 OS=Mus musculus GN=Taf15 PE=2 SV=1 - [Q8BQ46_MOUSE]	20.47	2	4	7	70	0.07	-0.31	-0.13	-0.53
F6TYB7	Myelin basic protein (Fragment) OS=Mus musculus GN=Mbp PE=4 SV=1 - [F6TYB7_MOUSE]	15.71	3	1	1	2	-0.71	-0.75	-0.79	-0.83
B1AS14	Uncharacterized protein (Fragment) OS=Mus musculus GN=Fam213b PE=2 SV=1 - [B1AS14_MOUSE]	7.89	2	1	1	3	-0.33	-0.59	-0.30	-0.56
Q91YT7	Protein Ythdf2 OS=Mus musculus GN=Ythdf2 PE=2 SV=1 - [Q91YT7_MOUSE]	6.22	1	2	4	7	-0.31	-0.23	-0.51	-0.43
P56393	Cytochrome c oxidase subunit 7B, mitochondrial OS=Mus musculus GN=Cox7b PE=2 SV=1 - [COX7B_MOUSE]	8.75	1	1	1	2	-0.34	-0.36	-0.74	-0.77
P20491	High affinity immunoglobulin epsilon receptor subunit gamma OS=Mus musculus GN=Fcer1g PE=1 SV=1 - [FCERG_MOUSE]	23.26	1	2	2	6	-0.14	-0.09	-0.13	-0.08
J3QN23	DNA repair and recombination protein RAD54B (Fragment) OS=Mus musculus GN=Rad54b PE=4 SV=1 - [J3QN23_MOUSE]	5.13	1	1	1	1	0.02	0.24	-0.08	0.15
P54320	Elastin OS=Mus musculus GN=Eln PE=2 SV=2 - [ELN_MOUSE]	8.02	1	3	3	7	-1.17	-1.18	-0.99	-1.00
P28293	Cathepsin G OS=Mus musculus GN=Ctsg PE=1 SV=2 - [CATG_MOUSE]	4.21	1	1	1	1	0.81	-0.16	1.01	0.04
E9Q8L9	Rab11 family-interacting protein 1 OS=Mus musculus GN=Rab11fip1 PE=2 SV=1 - [E9Q8L9_MOUSE]	4.55	2	4	4	12	-0.55	-0.73	-0.35	-0.67
Q9QX96	Sal-like protein 2 OS=Mus musculus GN=Sall2 PE=2 SV=2 - [SALL2_MOUSE]	2.99	2	1	1	2	-0.20	-1.31	-0.08	-1.18

E0CYF6	Protein AW554918 OS=Mus musculus GN=AW554918 PE=4 SV=1 - [E0CYF6_MOUSE]	13.75	1	1	1	4	-0.45	-0.58	-0.42	-0.64
P06797	Cathepsin L1 OS=Mus musculus GN=Ctsl1 PE=1 SV=2 - [CATL1_MOUSE]	17.66	2	4	4	23	-0.47	-0.41	-0.52	-0.47
F6R4Z5	Protein Zfp709 OS=Mus musculus GN=Zfp709 PE=2 SV=1 - [F6R4Z5_MOUSE]	5.37	119	1	3	4	-0.12	0.05	-0.94	-0.77
P24549	Retinal dehydrogenase 1 OS=Mus musculus GN=Aldh1a1 PE=1 SV=5 - [AL1A1_MOUSE]	54.89	1	17	27	483	-0.79	-0.73	-0.88	-0.80
Q8C7E4	Ribonuclease 4 OS=Mus musculus GN=Rnase4 PE=2 SV=1 - [Q8C7E4_MOUSE]	25.68	2	2	2	10	-0.12	-0.63	-0.29	-0.81
O55187-2	Isoform 2 of E3 SUMO-protein ligase CBX4 OS=Mus musculus GN=Cbx4 - [CBX4_MOUSE]	7.01	2	2	2	3	-0.56	0.04	-0.90	-0.31
Q00915	Retinol-binding protein 1 OS=Mus musculus GN=Rbp1 PE=2 SV=2 - [RET1_MOUSE]	42.96	1	5	5	10	-0.45	-0.04	-0.39	-0.04
Q64726	Zinc-alpha-2-glycoprotein OS=Mus musculus GN=Azgp1 PE=1 SV=2 - [ZA2G_MOUSE]	16.29	1	4	4	5	-0.38	-0.07	-0.47	-0.16
P33434-2	Isoform 2 of 72 kDa type IV collagenase OS=Mus musculus GN=Mmp2 - [MMP2_MOUSE]	10.75	2	4	5	10	-0.62	-0.70	-0.22	-0.47
F8WJ99	Microfibrillar-associated protein 5 OS=Mus musculus GN=Mfap5 PE=2 SV=2 - [F8WJ99_MOUSE]	12.50	4	2	2	5	-0.88	-0.81	-0.79	-0.72
Q8C156	Condensin complex subunit 2 OS=Mus musculus GN=Ncaph PE=2 SV=1 - [CND2_MOUSE]	3.42	1	2	2	5	0.60	0.58	0.22	0.20
D3Z5V3	Uncharacterized protein (Fragment) OS=Mus musculus GN=Knop1 PE=2 SV=1 - [D3Z5V3_MOUSE]	10.42	8	1	1	1	-0.74	-0.85	-0.41	-0.52
P49710	Hematopoietic lineage cell-specific protein OS=Mus musculus GN=Hcls1 PE=1 SV=2 [HCLS1_MOUSE]	26.95	2	9	9	31	-0.08	0.06	-0.12	-0.17
Q62312-2	Isoform RII-1 of TGF-beta receptor type-2 OS=Mus musculus GN=Tgfbr2 - [TGFR2_MOUSE]	1.59	2	1	1	2	-0.64	-0.52	-0.45	-0.33
P63054	Purkinje cell protein 4 OS=Mus musculus GN=Pcp4 PE=2 SV=2 - [PCP4_MOUSE]	27.42	2	1	1	27	-0.66	-0.39	-0.72	-0.57
Q8R0N6	Hydroxyacid-oxoacid transhydrogenase, mitochondrial OS=Mus musculus GN=Adhfe1 PE=2 SV=2 - [HOT_MOUSE]	13.33	2	4	4	6	-1.07	-1.29	-1.11	-1.34

Q6UKZ0	MCG8992 OS=Mus musculus GN=Serpinb3d PE=2 SV=1 - [Q6UKZ0_MOUSE]	2.07	1	1	1	1	-0.60	-0.57	-0.37	-0.34
P08207	Protein S100-A10 OS=Mus musculus GN=S100a10 PE=2 SV=2 - [S10AA_MOUSE]	34.02	1	5	5	239	-0.20	-0.08	-0.41	-0.21
D3YY50	Copper homeostasis protein cutC homolog (Fragment) OS=Mus musculus GN=Cutc PE=2 SV=1 - [D3YY50_MOUSE]	5.91	3	2	2	3	-0.13	-0.19	-0.14	-0.21
P59270-2	Isoform 2 of Galactosylgalactosylxylosylprotein 3-beta-glucuronosyltransferase 2 OS=Mus musculus GN=B3gat2 - [B3GA2_MOUSE]	4.30	2	1	1	4	-0.11	0.12	-0.38	-0.16
Q71FD5	E3 ubiquitin-protein ligase ZNRF2 OS=Mus musculus GN=Znrf2 PE=1 SV=1 - [ZNRF2_MOUSE]	5.46	1	1	1	2	-0.72	-0.85	-0.48	-0.61
P08074	Carbonyl reductase [NADPH] 2 OS=Mus musculus GN=Cbr2 PE=1 SV=1 - [CBR2_MOUSE]	39.75	2	6	7	41	-0.58	-0.57	-0.62	-0.64
P97494	Glutamate--cysteine ligase catalytic subunit OS=Mus musculus GN=Gclc PE=2 SV=4 - [GSH1_MOUSE]	29.98	1	13	13	69	-0.46	-0.33	-0.42	-0.32
P21981	Protein-glutamine gamma-glutamyltransferase 2 OS=Mus musculus GN=Tgm2 PE=1 SV=4 [TGM2_MOUSE]	37.46	2	22	22	272	-0.91	-0.92	-0.90	-0.90
Q60648	Ganglioside GM2 activator OS=Mus musculus GN=Gm2a PE=1 SV=2 - [SAP3_MOUSE]	12.95	1	3	3	9	-0.20	-0.37	-0.26	-0.35
Q91W59-2	Isoform 2 of RNA-binding motif, single-stranded-interacting protein 1 OS=Mus musculus GN=Rbms1 - [RBMS1_MOUSE]	8.11	11	2	2	11	-0.18	-0.10	-0.05	-0.16
Q9D6T0-2	Isoform 2 of Nitric oxide synthase-interacting protein OS=Mus musculus GN=Nosip - [NOSIP_MOUSE]	6.52	2	2	2	2	0.10	0.22	-0.07	0.04
O70200	Allograft inflammatory factor 1 OS=Mus musculus GN=Aif1 PE=1 SV=1 - [AIF1_MOUSE]	44.22	4	8	8	57	-0.34	-0.20	-0.38	-0.32
Q8BMK1	Methyltransferase-like protein 2 OS=Mus musculus GN=Mettl2 PE=2 SV=2 - [METL2_MOUSE]	4.88	1	1	1	2	-0.55	-0.53	-0.51	-0.49
D3Z0S5	Acid-sensing ion channel 5 OS=Mus musculus GN=Asic5 PE=2 SV=1 - [D3Z0S5_MOUSE]	2.65	2	1	1	1	-1.25	-0.58	-0.47	0.19
Q3U0S6	Ras-interacting protein 1 OS=Mus musculus GN=Rasip1 PE=1 SV=3 - [RAIN_MOUSE]	5.20	1	4	4	5	0.48	0.54	-0.45	-0.39

Q64314-2	Isoform Short of Hematopoietic progenitor cell antigen CD34 OS=Mus musculus GN=Cd34 - [CD34_MOUSE]	8.62	2	2	3	6	-0.48	-0.20	-0.80	-0.53
Q9WV03	Protein FAM50A OS=Mus musculus GN=Fam50a PE=2 SV=1 - [FA50A_MOUSE]	9.14	4	2	4	5	0.36	0.49	-0.17	-0.03
E9PVP1	Aurora kinase B OS=Mus musculus GN=Aim1 PE=2 SV=1 - [E9PVP1_MOUSE]	3.84	1	4	4	10	-0.32	-0.05	-0.40	0.03
Q9ESK4	Inhibitor of growth protein 2 OS=Mus musculus GN=Ing2 PE=1 SV=3 - [ING2_MOUSE]	4.27	1	1	1	3	0.61	0.49	0.62	0.50
Q62293	Interferon-gamma-inducible GTPase Ifggb5 protein OS=Mus musculus GN=Tgtp PE=2 SV=1 - [Q62293_MOUSE]	11.33	2	3	3	8	0.61	0.40	0.59	0.35
B1AR48	Elongator complex protein 5 (Fragment) OS=Mus musculus GN=Elp5 PE=2 SV=1 - [B1AR48_MOUSE]	15.65	3	1	1	1	0.90	1.20	0.65	0.95
E9Q3Z4	Hexokinase-3 OS=Mus musculus GN=Hk3 PE=2 SV=1 - [E9Q3Z4_MOUSE]	10.38	3	5	7	46	-0.01	0.03	0.11	0.00
Q8K5B1-5	Isoform 5 of Interleukin 31 receptor subunit alpha OS=Mus musculus GN=Il31ra - [IL31R_MOUSE]	15.32	2	2	2	2	-0.66	-0.11	-0.87	-0.31
P35492	Histidine ammonia-lyase OS=Mus musculus GN=Hal PE=1 SV=1 - [HUTH_MOUSE]	18.42	2	8	9	26	0.77	0.60	0.70	0.62
O88593	Peptidoglycan recognition protein 1 OS=Mus musculus GN=Pglyrp1 PE=2 SV=1 - [PGRP1_MOUSE]	30.77	1	3	3	34	-0.57	-1.05	-0.63	-1.03
Q61581	Insulin-like growth factor-binding protein 7 OS=Mus musculus GN=Igfbp7 PE=2 SV=3 - [IBP7_MOUSE]	35.59	3	8	8	28	0.03	-0.01	-0.08	-0.11
E9PVL3	Protein BC094916 OS=Mus musculus GN=BC094916 PE=2 SV=1 - [E9PVL3_MOUSE]	16.03	4	1	4	10	-0.79	-0.72	-0.99	-0.93
P47963	60S ribosomal protein L13 OS=Mus musculus GN=Rpl13 PE=2 SV=3 [RL13_MOUSE]	37.91	2	9	10	115	-0.80	-0.78	-0.70	-0.67
Q1RLL5	Predicted gene, EG331529 OS=Mus musculus GN=Gm5128 PE=2 SV=1 - [Q1RLL5_MOUSE]	5.63	7	2	2	4	-1.17	-0.50	-0.77	-0.21
Q91XE4	Aspartoacylase-2 OS=Mus musculus GN=Acy3 PE=1 SV=1 - [ACY3_MOUSE]	26.10	1	5	5	8	-1.04	-1.53	-1.06	-1.42

Q9CY57-3	Isoform 3 of Chromatin target of PRMT1 protein OS=Mus musculus GN=Chtop - [CHTOP_MOUSE]	34.65	8	5	5	20	-0.85	-0.93	-0.97	-0.74
Q8CID0	Cysteine-rich protein 2-binding protein OS=Mus musculus GN=Csrp2bp PE=2 SV=2 - [CSR2B_MOUSE]	3.21	3	2	2	4	-0.34	-0.37	-0.14	-0.18
P21570	Angiogenin OS=Mus musculus GN=Ang PE=1 SV=1 - [ANGI_MOUSE]	13.79	1	2	2	4	-0.07	-0.02	-0.42	0.01
D3Z734	E3 ubiquitin-protein ligase RAD18 OS=Mus musculus GN=Rad18 PE=2 SV=1 - [D3Z734_MOUSE]	3.27	4	2	2	3	-0.41	-0.30	-0.60	-0.49
D3Z420	MCG18394 OS=Mus musculus GN=Fam186b PE=4 SV=1 - [D3Z420_MOUSE]	0.64	1	1	1	1	-0.31	-0.09	-0.59	-0.38
Q6NSV5	Kynureninase OS=Mus musculus GN=Kynu PE=2 SV=1 - [Q6NSV5_MOUSE]	2.57	3	1	1	1	0.68	-0.29	0.71	-0.26
P07091	Protein S100-A4 OS=Mus musculus GN=S100a4 PE=1 SV=1 - [S10A4_MOUSE]	24.75	2	3	3	21	-0.18	-0.22	-0.22	-0.20
Q80T14	Extracellular matrix protein FRAS1 OS=Mus musculus GN=Fras1 PE=2 SV=2 - [FRAS1_MOUSE]	1.60	1	3	4	11	-0.90	-0.83	-1.00	-0.94
Q8BQM8	Echinoderm microtubule-associated protein-like 5 OS=Mus musculus GN=Eml5 PE=2 SV=2 - [EMAL5_MOUSE]	0.40	1	1	1	1	0.12	-0.41	0.03	-0.51
Q91YJ5	Translation initiation factor IF-2, mitochondrial OS=Mus musculus GN=Mtif2 PE=1 SV=2 - [IF2M_MOUSE]	4.95	6	3	4	6	-0.05	-0.16	0.05	-0.26
E9Q4P1	Protein Wdfy1 OS=Mus musculus GN=Wdfy1 PE=4 SV=1 - [E9Q4P1_MOUSE]	5.61	1	2	2	4	0.34	0.27	0.32	0.17
P28654	Decorin OS=Mus musculus GN=Dcn PE=2 SV=1 - [PGS2_MOUSE]	54.80	1	15	18	286	-0.74	-0.65	-0.77	-0.64
P16294	Coagulation factor IX OS=Mus musculus GN=F9 PE=2 SV=3 - [FA9_MOUSE]	4.25	1	2	2	3	-0.35	-0.54	-0.41	-0.60
Q8R1M0	UPF0361 protein C3orf37 homolog OS=Mus musculus PE=2 SV=1 - [CC037_MOUSE]	3.40	1	1	1	1	0.02	-0.06	-0.62	-0.71
A2AIW0-6	Isoform 6 of Serologically defined colon cancer antigen 3 homolog OS=Mus musculus GN=Sdccag3 [SDCG3_MOUSE]	15.05	2	1	1	1	-0.90	-2.32	-1.13	-2.55

A1L317	Keratin, type I cytoskeletal 24 OS=Mus musculus GN=Krt24 PE=2 SV=2 [K1C24_MOUSE]	6.84	3	2	3	26	-1.02	-0.50	-0.90	-0.37
P09528	Ferritin heavy chain OS=Mus musculus GN=Fth1 PE=1 SV=2 - [FRIH_MOUSE]	24.73	1	4	4	12	-1.20	-1.25	-1.33	-1.38
Q9DBD0	Inhibitor of carbonic anhydrase OS=Mus musculus GN=Ica PE=1 SV=1 - [ICA_MOUSE]	17.57	3	12	13	46	-0.21	-0.24	-0.07	-0.20
P70429-2	Isoform 1 of Ena/VASP-like protein OS=Mus musculus GN=Evl - [EVL_MOUSE]	11.96	4	3	3	16	-0.31	-0.01	-0.44	-0.12
Q9DCZ1	GMP reductase 1 OS=Mus musculus GN=Gmpr PE=2 SV=1 [GMPR1_MOUSE]	11.01	1	1	4	7	-0.27	-0.44	-0.29	-0.45
P98086	Complement C1q subcomponent subunit A OS=Mus musculus GN=C1qa PE=1 SV=2 - [C1QA_MOUSE]	10.20	1	2	2	7	0.08	-0.04	-0.31	-0.44
P97821	Dipeptidyl peptidase 1 OS=Mus musculus GN=Ctsc PE=2 SV=1 - [CATC_MOUSE]	17.75	2	7	7	78	-0.12	-0.03	-0.13	-0.23
Q7TQA6	Taste receptor type 2 member 38 OS=Mus musculus GN=Tas2r38 PE=2 SV=1 - [T2R38_MOUSE]	3.63	1	1	1	2	0.08	0.27	-0.32	-0.13
P0CW02	Lymphocyte antigen 6C1 OS=Mus musculus GN=Ly6c1 PE=2 SV=1 - [LY6C1_MOUSE]	14.50	2	1	1	10	-0.43	-0.34	-0.71	-0.54
P70232-2	Isoform 2 of Neural cell adhesion molecule L1-like protein OS=Mus musculus GN=Chl1 - [CHL1_MOUSE]	15.48	2	13	13	40	-0.70	-0.49	-0.65	-0.64
O89017	Legumain OS=Mus musculus GN=Lgmn PE=1 SV=1 - [LGMN_MOUSE]	16.32	1	6	6	75	-0.42	-0.54	-0.27	-0.43
E0CYX7	FXYD domain-containing ion transport regulator 5 (Fragment) OS=Mus musculus GN=Fxyd5 PE=2 SV=1 - [E0CYX7_MOUSE]	14.41	5	1	1	1	0.07	-0.87	0.03	-0.91
Q80ZI6	E3 ubiquitin-protein ligase LRSAM1 OS=Mus musculus GN=Lrsam1 PE=2 SV=1 - [LRSM1_MOUSE]	4.68	4	3	3	4	-0.65	-0.88	-1.03	-1.27
Q8CFX1	GDH/6PGL endoplasmic bifunctional protein OS=Mus musculus GN=H6pd PE=2 SV=2 - [G6PE_MOUSE]	15.08	3	11	12	70	-0.76	-0.58	-0.86	-0.63
Q5QNQ9	Collagen alpha-1(XXVII) chain OS=Mus musculus GN=Col27a1 PE=2 SV=1 - [CORA1_MOUSE]	2.60	1	2	2	2	0.29	-0.33	-0.30	-0.92

Q3UW66	Sulfurtransferase OS=Mus musculus GN=Mpst PE=2 SV=1 - [Q3UW66_MOUSE]	55.22	2	13	13	148	0.16	0.26	0.22	0.19
Q6PB89	Adarb2 protein OS=Mus musculus GN=Adarb2 PE=2 SV=1 - [Q6PB89_MOUSE]	2.57	2	1	1	1	-0.06	-0.58	-0.39	-0.91
E9Q9Q2	Protein R3hdm1 OS=Mus musculus GN=R3hdm1 PE=2 SV=1 - [E9Q9Q2_MOUSE]	1.15	1	1	2	8	-0.52	-0.56	-0.23	-0.27
E9PYB0	Protein Ahnak2 (Fragment) OS=Mus musculus GN=Ahnak2 PE=2 SV=1 - [E9PYB0_MOUSE]	46.41	2	3	13	66	-0.07	-0.38	-0.34	-0.66
Q8K385	Ferric-chelate reductase 1 OS=Mus musculus GN=FRRS1 PE=1 SV=1 - [FRRS1_MOUSE]	1.18	1	1	1	2	-0.39	-0.23	-0.63	-0.48
Q9DCS1	Transmembrane protein 176A OS=Mus musculus GN=Tmem176a PE=2 SV=2 - [T176A_MOUSE]	5.74	1	1	1	3	-1.19	-1.21	-0.78	-0.80
Q8R4P4	Transmembrane channel like protein 2 OS=Mus musculus GN=Tmc2 PE=2 SV=1 - [TMC2_MOUSE]	2.25	1	1	1	1	-0.09	-0.09	-0.04	-0.04
Q8BZN6	Dedicator of cytokinesis protein 10 OS=Mus musculus GN=Dock10 PE=1 SV=3 - [DOC10_MOUSE]	2.09	4	4	4	8	-0.04	-0.28	-0.25	-0.26
Q8BY78	Eyes absent 4 homolog (Drosophila) OS=Mus musculus GN=Eya4 PE=2 SV=1 - [Q8BY78_MOUSE]	2.11	2	1	1	3	-0.01	-0.10	-0.64	-0.73
F8VPM0	Bromodomain adjacent to zinc finger domain protein 2A OS=Mus musculus GN=Baz2a PE=2 SV=1 - [F8VPM0_MOUSE]	1.01	5	1	1	3	0.76	0.81	0.19	0.24
B1AWQ3	Espin OS=Mus musculus GN=Espn PE=2 SV=1 - [B1AWQ3_MOUSE]	6.49	16	4	4	8	-0.82	-0.49	-0.86	-0.57
A2A4A1	DnaJ homolog subfamily C member 24 OS=Mus musculus GN=Dnajc24 PE=2 SV=1 - [A2A4A1_MOUSE]	12.84	3	2	2	5	-0.19	-0.48	-0.31	-0.26
Q3UKJ7	WD40 repeat-containing protein SMU1 OS=Mus musculus GN=Smu1 PE=2 SV=2 - [SMU1_MOUSE]	12.67	2	5	5	23	-0.48	-0.45	-0.45	-0.44
A2BIN0	Major urinary proteins 11 and 8 OS=Mus musculus GN=Mup11 PE=2 SV=1 - [A2BIN0_MOUSE]	82.98	18	1	6	32	-0.71	-0.85	-0.52	-0.67

Q9CR59	Growth arrest and DNA damage-inducible proteins-interacting protein 1 OS=Mus musculus GN=Gadd45gip1 PE=2 SV=1 - [G45IP_MOUSE]	4.50	1	1	1	4	0.82	0.63	-0.05	-0.24
Q3TBT3-3	Isoform 3 of Stimulator of interferon genes protein OS=Mus musculus GN=Tmem173 - [STING_MOUSE]	15.13	3	3	3	12	0.17	-0.06	0.04	-0.19
G3X9Y6	Aldo-keto reductase family 1, member C19 OS=Mus musculus GN=Akr1c19 PE=4 SV=1 - [G3X9Y6_MOUSE]	54.18	1	12	16	350	-1.01	-0.90	-0.88	-0.82
Q6NXI6-2	Isoform 2 of Regulation of nuclear pre-mRNA domain-containing protein 2 OS=Mus musculus GN=Rprd2 - [RPRD2_MOUSE]	10.25	2	5	6	10	0.18	0.26	-0.57	-0.50
P59384	A disintegrin and metalloproteinase with thrombospondin motifs 15 OS=Mus musculus GN=Adamts15 PE=2 SV=2 - [ATS15_MOUSE]	1.79	1	1	1	1	-1.11	-0.22	-0.96	-0.07
Q8R0K4	Coiled-coil domain-containing protein 137 OS=Mus musculus GN=Ccdc137 PE=2 SV=1 - [CC137_MOUSE]	7.59	2	2	2	3	0.12	-0.34	0.13	0.24
D3Z5G7	Protein Ces1b OS=Mus musculus GN=Ces1b PE=4 SV=1 - [D3Z5G7_MOUSE]	16.75	1	1	9	75	-0.63	-1.03	-0.69	-0.98
O70370	Cathepsin S OS=Mus musculus GN=Ctss PE=2 SV=2 - [CATS_MOUSE]	32.65	2	8	8	51	-0.34	-0.22	-0.21	-0.29
Q9D7F7	Charged multivesicular body protein 4c OS=Mus musculus GN=Chmp4c PE=2 SV=1 - [CHM4C_MOUSE]	26.29	1	4	6	22	-0.52	-0.31	0.01	-0.29
Q8BUN5	Mothers against decapentaplegic homolog 3 OS=Mus musculus GN=Smad3 PE=1 SV=2 - [SMAD3_MOUSE]	4.00	1	1	2	3	0.06	0.35	-0.16	0.13
E9Q6J6	Protein Psg20 OS=Mus musculus GN=Psg20 PE=2 SV=1 - [E9Q6J6_MOUSE]	3.81	2	1	1	2	-0.94	-1.00	-1.21	-1.27
P28650	Adenylosuccinate synthetase isozyme 1 OS=Mus musculus GN=Adssl1 PE=1 SV=2 - [PURA1_MOUSE]	15.10	3	4	5	11	0.14	0.51	0.16	0.30
P08226	Apolipoprotein E OS=Mus musculus GN=Apoe PE=1 SV=2 - [APOE_MOUSE]	36.98	8	12	12	78	-0.22	-0.26	-0.17	-0.19
P50429	Arylsulfatase B OS=Mus musculus GN=Arsb PE=2 SV=3 - [ARSB_MOUSE]	7.68	3	3	3	21	-0.42	-0.43	-0.16	-0.17

Q8BGT1	Fibronectin leucine rich transmembrane protein 3 OS=Mus musculus GN=Flrt3 PE=2 SV=1 - [Q8BGT1_MOUSE]	7.70	1	4	4	4	-0.06	-0.98	-0.59	-1.51
Q9D312	Keratin, type I cytoskeletal 20 OS=Mus musculus GN=Krt20 PE=1 SV=1 [K1C20_MOUSE]	61.95	9	21	27	359	-0.36	-0.31	-0.33	-0.28
B1ATR5	Glypican-3 OS=Mus musculus GN=Gpc3 PE=2 SV=1 - [B1ATR5_MOUSE]	1.63	2	1	1	2	-0.25	-0.23	-0.34	-0.32
F6S522	Claspin (Fragment) OS=Mus musculus GN=Clspn PE=4 SV=1 [F6S522_MOUSE]	12.14	2	2	3	7	-0.37	-0.34	-0.76	-0.73
Q8WUR0	Protein C19orf12 homolog OS=Mus musculus PE=2 SV=1 - [CS012_MOUSE]	11.35	2	1	2	8	-0.79	0.09	-1.74	0.02
Q9R0H0	Peroxisomal acyl-coenzyme A oxidase 1 OS=Mus musculus GN=Acox1 PE=1 SV=5 - [ACOX1_MOUSE]	39.03	3	19	19	178	-0.67	-0.66	-0.67	-0.75
P46412	Glutathione peroxidase 3 OS=Mus musculus GN=Gpx3 PE=2 SV=2 - [GPX3_MOUSE]	34.51	5	7	7	48	-0.91	-0.91	-0.90	-0.89
Q00519	Xanthine dehydrogenase/oxidase OS=Mus musculus GN=Xdh PE=1 SV=5 - [XDH_MOUSE]	20.00	2	20	22	124	-0.50	-0.37	-0.47	-0.38
Q61179	Interferon regulatory factor 9 OS=Mus musculus GN=Irf9 PE=1 SV=1 - [IRF9_MOUSE]	2.26	3	1	1	2	-0.13	-0.10	-0.06	-0.03
Q9JI18	Low-density lipoprotein receptor-related protein 1B OS=Mus musculus GN=Lrp1b PE=2 SV=1 [LRP1B_MOUSE]	1.04	4	4	5	16	-0.56	-0.51	-0.70	-0.65
P62960	Nuclease-sensitive element-binding protein 1 OS=Mus musculus GN=Ybx1 PE=1 SV=3 - [YBOX1_MOUSE]	51.24	3	4	10	98	-0.35	-0.12	-0.60	-0.49
Q91X91	Nicotinate-nucleotide pyrophosphorylase [carboxylating] OS=Mus musculus GN=Qprt PE=2 SV=1 - [NADC_MOUSE]	8.03	1	2	2	2	-1.10	-0.79	-0.96	-0.65
Q8CGN5	Perilipin-1 OS=Mus musculus GN=Plin1 PE=1 SV=2 - [PLIN1_MOUSE]	38.49	3	7	16	96	-2.29	-2.09	-1.86	-1.63
O70145	Neutrophil cytosol factor 2 OS=Mus musculus GN=Ncf2 PE=2 SV=1 - [NCF2_MOUSE]	10.29	1	5	5	10	-0.04	-0.54	-0.05	-0.46
A2AQ53	Fibrillin-1 OS=Mus musculus GN=Fbn1 PE=4 SV=1 - [A2AQ53_MOUSE]	22.97	2	54	55	378	-0.90	-0.91	-0.91	-0.93
Q8K124	Pleckstrin homology domain-containing family O member 2 OS=Mus musculus GN=Plekho2 PE=1 SV=1 - [PKHO2_MOUSE]	26.26	1	8	8	26	-0.36	-0.34	-0.48	-0.56

E9Q1T4	Sphingomyelin phosphodiesterase 4 (Fragment) OS=Mus musculus GN=Smpd4 PE=2 SV=1 - [E9Q1T4_MOUSE]	13.60	7	1	1	1	-0.05	-0.74	-0.69	-1.38
Q9DAC2	Complement component 8, gamma subunit, isoform CRA_b OS=Mus musculus GN=C8g PE=2 SV=1 - [Q9DAC2_MOUSE]	29.76	2	3	3	13	-1.09	-1.06	-0.91	-0.90
Q9CYF5	Williams-Beuren syndrome chromosomal region 16 protein homolog OS=Mus musculus GN=Wbscr16 PE=2 SV=1 - [WBS16_MOUSE]	4.77	1	1	1	2	0.37	0.22	-0.25	-0.40
Q9QZB9	Dynactin subunit 5 OS=Mus musculus GN=Dctn5 PE=1 SV=1 [DCTN5_MOUSE]	5.49	1	1	1	2	-0.19	-1.28	-0.33	-1.43
Q8BG07-2	Isoform 2 of Phospholipase D4 OS=Mus musculus GN=Pld4 - [PLD4_MOUSE]	20.61	2	7	7	19	-0.07	-0.04	-0.10	-0.25
O54839-2	Isoform 2 of Eomesodermin homolog OS=Mus musculus GN=Eomes - [EOMES_MOUSE]	1.89	2	1	1	1	-0.03	0.03	-0.02	0.04
B7ZMZ7	Dip2c protein OS=Mus musculus GN=Dip2c PE=2 SV=1 - [B7ZMZ7_MOUSE]	5.76	3	3	6	9	0.39	0.27	-0.26	-0.37
Q8BK48	Carboxylesterase 5, isoform CRA_b OS=Mus musculus GN=Ces2e PE=2 SV=1 [Q8BK48_MOUSE]	38.82	4	11	15	248	-0.54	-0.55	-0.58	-0.59
Q8C5L3-3	Isoform 3 of CCR4-NOT transcription complex subunit 2 OS=Mus musculus GN=Cnot2 - [CNOT2_MOUSE]	5.27	5	2	2	2	-0.56	-0.79	-0.71	-0.93
Q6ZQ29	Serine/threonine-protein kinase TAO2 OS=Mus musculus GN=Taok2 PE=1 SV=3 - [TAOK2_MOUSE]	2.74	3	1	3	5	-0.24	0.02	-0.87	-0.61
Q5U462	CUB domain-containing protein 1 OS=Mus musculus GN=Cdcp1 PE=2 SV=1 - [CDCP1_MOUSE]	3.36	1	2	2	4	-0.52	-0.05	-1.01	-0.55
Q9CQW5	Galectin-2 OS=Mus musculus GN=Lgals2 PE=2 SV=1 - [LEG2_MOUSE]	46.15	1	5	5	13	-0.60	-0.82	-0.29	-0.48
Q99JZ0	Syntenin-2 OS=Mus musculus GN=Sdcbp2 PE=2 SV=1 - [SDCB2_MOUSE]	7.19	1	2	2	7	0.21	-0.12	-0.23	-0.57
P31532	Serum amyloid A-4 protein OS=Mus musculus GN=Saa4 PE=1 SV=2 - [SAA4_MOUSE]	37.69	1	4	4	12	-0.01	0.12	-0.07	-0.08
Q8C5W4-2	Isoform 2 of MORC family CW-type zinc finger protein 2B OS=Mus musculus GN=Morc2b - [MOR2B_MOUSE]	4.61	2	2	2	13	-0.76	-0.76	-0.83	-0.84

P49182	Heparin cofactor 2 OS=Mus musculus GN=Serpind1 PE=1 SV=1 - [HEP2_MOUSE]	8.16	1	4	4	8	-0.52	-0.78	-0.65	-0.72
Q9CQT0	MCG22296, isoform CRA_b OS=Mus musculus GN=Thg1l PE=2 SV=1 - [Q9CQT0_MOUSE]	4.62	2	1	1	4	0.39	0.50	-0.16	-0.05
Q6VN19	Ran-binding protein 10 OS=Mus musculus GN=Ranbp10 PE=1 SV=2 - [RBP10_MOUSE]	3.55	1	2	2	2	-0.55	-0.52	0.04	0.07
P05366	Serum amyloid A-1 protein OS=Mus musculus GN=Saa1 PE=2 SV=2 - [SAA1_MOUSE]	29.51	2	3	3	17	0.76	0.57	0.90	0.71
Q6PCM2-2	Isoform 2 of Integrator complex subunit 6 OS=Mus musculus GN=Ints6 - [INT6_MOUSE]	1.72	2	1	1	1	-0.47	-4.17	-1.21	-4.91
E9PZ00	Sulfated glycoprotein 1 OS=Mus musculus GN=Psap PE=2 SV=1 - [E9PZ00_MOUSE]	35.21	5	20	20	273	-0.38	-0.34	-0.47	-0.43
Q6P6M5	Peroxisomal membrane protein 11C OS=Mus musculus GN=Pex11g PE=2 SV=2 - [PX11C_MOUSE]	7.88	3	2	2	3	-0.62	-0.72	-0.60	-0.70
Q9CQN6	Transmembrane protein 14C OS=Mus musculus GN=Tmem14c PE=1 SV=1 - [TM14C_MOUSE]	14.91	1	2	2	4	-0.62	0.00	-1.40	-0.78
Q00897	Alpha-1-antitrypsin 1-4 OS=Mus musculus GN=Serpina1d PE=2 SV=1 - [A1AT4_MOUSE]	50.61	1	6	18	432	-0.83	-0.57	-0.87	-0.58
P09242	Alkaline phosphatase, tissue-nonspecific isozyme OS=Mus musculus GN=Alpl PE=1 SV=2 - [PPBT_MOUSE]	8.78	4	4	4	12	-0.43	-0.19	-0.33	-0.05
Q9R111	Guanine deaminase OS=Mus musculus GN=Gda PE=1 SV=1 - [GUAD_MOUSE]	29.07	2	12	13	77	-0.41	-0.46	-0.39	-0.46
P39654	Arachidonate 12-lipoxygenase, leukocyte type OS=Mus musculus GN=Alox12l PE=1 SV=4 - [LX12L_MOUSE]	9.50	1	4	4	10	-0.04	-0.26	0.29	-0.11
Q8CGE8	Interferon-activable protein 205-A OS=Mus musculus GN=Ifi205a PE=2 SV=1 - [IFI5A_MOUSE]	13.37	1	3	5	8	-0.34	-0.55	0.30	0.09
Q99NF3	Centrosomal protein of 41 kDa OS=Mus musculus GN=Cep41 PE=1 SV=1 - [CEP41_MOUSE]	6.70	1	1	1	1	-1.31	-0.51	-1.13	-0.34
Q9DAK8-2	Isoform 2 of Leucine-rich repeat-containing protein 51 OS=Mus musculus GN=Lrrc51 - [LRC51_MOUSE]	4.11	5	1	1	1	0.29	0.12	0.41	0.24
Q08857	Platelet glycoprotein 4 OS=Mus musculus GN=Cd36 PE=1 SV=2 - [CD36_MOUSE]	19.49	1	7	8	35	-1.45	-1.44	-1.42	-1.37

Q2VLH6	Scavenger receptor cysteine-rich type 1 protein M130 OS=Mus musculus GN=Cd163 PE=1 SV=2 - [C163A_MOUSE]	7.14	2	6	6	14	-1.06	-1.09	-0.94	-1.35
A2AEC2	Transcription elongation factor A protein-like 3 (Fragment) OS=Mus musculus GN=Tceal3 PE=2 SV=1 - [A2AEC2_MOUSE]	9.09	4	2	2	3	-0.16	-0.23	-0.48	-0.55
Q99P91	Transmembrane glycoprotein NMB OS=Mus musculus GN=Gpnmb PE=1 SV=2 - [GPNMB_MOUSE]	6.27	1	3	3	4	-1.40	-1.08	-1.02	-0.71
Q9CX97	WD repeat-containing protein 55 OS=Mus musculus GN=Wdr55 PE=2 SV=2 - [WDR55_MOUSE]	9.54	1	2	2	4	0.63	0.28	0.10	-0.45
Q5SSG2	Protein Mmp28 OS=Mus musculus GN=Mmp28 PE=2 SV=2 - [Q5SSG2_MOUSE]	1.72	4	1	1	1	-1.31	-1.33	-1.57	-1.59
P23953	Carboxylesterase 1C OS=Mus musculus GN=Ces1c PE=1 SV=4 [EST1C_MOUSE]	27.26	2	6	14	114	-0.22	-0.06	-0.23	-0.10
F6Z700	Striatin (Fragment) OS=Mus musculus GN=Strn PE=4 SV=1 - [F6Z700_MOUSE]	37.08	1	1	7	30	0.00	-0.16	-0.51	-0.67
P97290	Plasma protease C1 inhibitor OS=Mus musculus GN=Serping1 PE=1 SV=3 - [IC1_MOUSE]	8.33	2	4	4	8	0.04	0.07	-0.06	0.02
Q9JLB0-2	Isoform Alpha of MAGUK p55 subfamily member 6 OS=Mus musculus GN=Mpp6 - [MPP6_MOUSE]	27.64	3	13	13	41	-0.12	-0.15	0.07	-0.03
P03995-2	Isoform 2 of Glial fibrillary acidic protein OS=Mus musculus GN=Gfap - [GFAP_MOUSE]	49.07	2	16	19	210	-0.59	-0.56	-0.48	-0.55
E9Q6P4	Protein Tubgcp6 OS=Mus musculus GN=Tubgcp6 PE=2 SV=1 - [E9Q6P4_MOUSE]	2.33	3	3	4	5	-0.65	-0.55	-1.07	-0.97
Q8CE50	Sorting nexin-30 OS=Mus musculus GN=Snx30 PE=2 SV=1 - [SNX30_MOUSE]	6.18	1	2	2	3	-0.17	-0.52	-1.08	-1.43
E9QL31	Disabled homolog 2 OS=Mus musculus GN=Dab2 PE=2 SV=1 - [E9QL31_MOUSE]	23.49	10	13	13	40	-0.34	-0.50	-0.38	-0.38
G3X982	Aldehyde oxidase 3, isoform CRA_a OS=Mus musculus GN=Aox3 PE=4 SV=1 - [G3X982_MOUSE]	3.52	2	2	3	4	0.10	0.17	-0.54	-0.46
A2A998	Complement component C8 alpha chain OS=Mus musculus GN=C8a PE=2 SV=1 - [A2A998_MOUSE]	18.97	3	7	8	16	-0.71	-0.52	-0.65	-0.45

Q3USH5	Splicing factor, suppressor of white-apricot homolog OS=Mus musculus GN=Sfswap PE=1 SV=2 - [SFSWA_MOUSE]	1.90	1	2	2	3	-0.52	-0.67	-0.75	-0.90
E9Q993	Aryl hydrocarbon receptor nuclear translocator-like protein 2 OS=Mus musculus GN=Arntl2 PE=2 SV=1 - [E9Q993_MOUSE]	6.85	4	1	2	4	-0.62	-1.07	-1.43	-1.88
Q61129	Complement factor I OS=Mus musculus GN=Cfi PE=1 SV=3 - [CFAI_MOUSE]	9.29	1	6	6	10	-0.19	-0.22	-0.18	-0.29
Q9D3T1	Protein 4933436I01Rik OS=Mus musculus GN=4933436I01Rik PE=2 SV=1 - [Q9D3T1_MOUSE]	2.80	1	1	1	1	-0.87	-1.20	-0.33	-0.66
P11352	Glutathione peroxidase 1 OS=Mus musculus GN=Gpx1 PE=1 SV=2 - [GPX1_MOUSE]	49.75	1	9	9	125	-0.66	-0.52	-0.64	-0.53
Q9QXC1	Fetuin-B OS=Mus musculus GN=Fetub PE=1 SV=1 - [FETUB_MOUSE]	19.33	2	5	5	17	-0.24	-0.17	-0.31	-0.13
Q8VCC2	Liver carboxylesterase 1 OS=Mus musculus GN=Ces1 PE=2 SV=1 - [EST1_MOUSE]	26.02	1	11	15	153	-0.91	-0.86	-1.00	-0.96
Q8CFB4	Guanylate-binding protein 5 OS=Mus musculus GN=Gbp5 PE=1 SV=2 - [GBP5_MOUSE]	4.92	1	3	3	4	0.16	-0.26	0.22	-0.22
P07724	Serum albumin OS=Mus musculus GN=Alb PE=1 SV=3 - [ALBU_MOUSE]	73.68	1	48	48	3010	-0.66	-0.57	-0.71	-0.64
P28665	Murinoglobulin-1 OS=Mus musculus GN=Mug1 PE=1 SV=3 [MUG1_MOUSE]	25.34	1	16	29	138	-0.67	-0.60	-0.64	-0.41
P70261	Paladin OS=Mus musculus GN=Pald1 PE=1 SV=1 - [PALD_MOUSE]	2.10	1	1	1	1	-0.52	-0.24	-0.83	-0.54
Q9JHH6	Carboxypeptidase B2 OS=Mus musculus GN=Cpb2 PE=1 SV=1 - [CBPB2_MOUSE]	9.48	1	2	2	2	-0.90	-0.73	-0.61	-0.44
P97326	Cadherin-6 OS=Mus musculus GN=Cdh6 PE=1 SV=2 - [CADH6_MOUSE]	1.65	1	1	1	1	0.11	0.51	-0.77	-0.37
Q80WJ1	Gametogenetin OS=Mus musculus GN=Ggn PE=1 SV=2 - [GGN_MOUSE]	2.53	2	1	1	1	-0.26	-0.27	-0.30	-0.31
P28481-7	Isoform 7 of Collagen alpha-1(II) chain OS=Mus musculus GN=Col2a1 - [CO2A1_MOUSE]	5.07	8	4	4	12	-1.21	-0.99	-1.32	-1.21
F6TP58	Protein Dchs1 (Fragment) OS=Mus musculus GN=Dchs1 PE=4 SV=1 - [F6TP58_MOUSE]	1.25	3	2	2	4	-0.72	-0.91	-0.85	-0.92
P28667	MARCKS-related protein OS=Mus musculus GN=Marcksl1 PE=1 SV=2 - [MRP_MOUSE]	14.00	1	2	2	5	-0.50	-0.42	-0.45	-0.37

Q7TPM9	Myotubularin-related protein 10 OS=Mus musculus GN=Mtmr10 PE=2 SV=2 - [MTMRA_MOUSE]	6.74	2	4	4	4	0.15	0.33	-0.56	0.37
Q80YC5	Coagulation factor XII OS=Mus musculus GN=F12 PE=2 SV=2 - [FA12_MOUSE]	3.69	2	2	2	4	-0.72	-0.48	-0.67	-0.42
P11672	Neutrophil gelatinase-associated lipocalin OS=Mus musculus GN=Lcn2 PE=1 SV=1 - [NGAL_MOUSE]	14.00	1	2	2	3	-0.13	-0.09	0.07	-0.31
P12804	Fibroleukin OS=Mus musculus GN=Fgl2 PE=1 SV=1 - [FGL2_MOUSE]	12.96	1	5	5	15	-0.81	-0.66	-0.78	-0.55
Q00896	Alpha-1-antitrypsin 1-3 OS=Mus musculus GN=Serpina1c PE=1 SV=2 - [A1AT3_MOUSE]	48.54	2	3	15	503	0.04	0.05	-0.15	0.00
B2RQC2	Ubiquitin carboxyl-terminal hydrolase 42 OS=Mus musculus GN=Usp42 PE=2 SV=1 - [UBP42_MOUSE]	0.98	1	1	1	1	0.16	-0.28	-1.10	-1.54
Q3TJI8	Corticosteroid 11-beta-dehydrogenase isozyme 1 OS=Mus musculus GN=Hsd11b1 PE=2 SV=1 - [Q3TJI8_MOUSE]	13.36	3	3	3	9	-1.21	-1.06	-1.29	-1.25
Q6S9I0	Kng2 protein OS=Mus musculus GN=Kng2 PE=2 SV=1 - [Q6S9I0_MOUSE]	8.08	4	2	3	14	-0.92	0.32	-1.11	0.08
Q8BWP8-2	Isoform 2 of N-acetyllactosaminide beta-1,3-N-acetylglucosaminyltransferase OS=Mus musculus GN=B3gnt1 - [B3GN1_MOUSE]	8.18	2	1	1	3	-0.76	-0.06	-0.97	-0.27
P22599	Alpha-1-antitrypsin 1-2 OS=Mus musculus GN=Serpina1b PE=1 SV=2 - [A1AT2_MOUSE]	47.22	2	3	15	511	-0.40	-0.33	-0.63	-0.56
A8Y5N4	17-beta-hydroxysteroid dehydrogenase 13 OS=Mus musculus GN=Hsd17b13 PE=2 SV=1 - [A8Y5N4_MOUSE]	7.58	3	1	2	5	-0.32	-0.37	-0.18	-0.24
P22005	Proenkephalin-A OS=Mus musculus GN=Penk PE=2 SV=2 - [PENK_MOUSE]	17.54	2	3	3	20	-1.11	-1.10	-1.22	-1.17
Q7TSH9	Zinc finger protein 184 OS=Mus musculus GN=Zfp184 PE=2 SV=1 - [ZN184_MOUSE]	7.60	119	1	2	3	-0.80	0.25	-1.47	-0.42
P01029	Complement C4-B OS=Mus musculus GN=C4b PE=1 SV=3 - [CO4B_MOUSE]	21.75	1	30	30	93	-0.48	-0.45	-0.30	-0.36
Q3U0Y2	Uncharacterized protein ZMYM6NB OS=Mus musculus GN=Zmym6nb PE=2 SV=2 - [ZMYNB_MOUSE]	8.67	1	1	1	2	-0.28	-0.30	-0.42	-0.45

O88393	Transforming growth factor beta receptor type 3 OS=Mus musculus GN=Tgfbr3 PE=1 SV=1 - [TGBR3_MOUSE]	8.94	1	3	3	5	-0.79	-0.50	-0.84	-0.55
Q64332	Synapsin-2 OS=Mus musculus GN=Syn2 PE=1 SV=2 - [SYN2_MOUSE]	20.99	2	5	5	15	-0.33	-0.41	-0.82	-0.91
Q8VCE2	GPN-loop GTPase 1 OS=Mus musculus GN=Gpn1 PE=1 SV=1 - [GPN1_MOUSE]	2.96	1	1	1	1	-0.04	0.08	-1.53	-1.41
F7CIB0	Centrosomal protein of 128 kDa (Fragment) OS=Mus musculus GN=Cep128 PE=2 SV=1 - [F7CIB0_MOUSE]	5.76	3	1	3	7	0.56	0.18	-0.24	-0.62
F6T8K1	Izumo sperm-egg fusion protein 3 (Fragment) OS=Mus musculus GN=Izumo3 PE=2 SV=1 - [F6T8K1_MOUSE]	8.29	4	1	2	39	-0.18	-0.13	0.13	0.18
O35744	Chitinase-3-like protein 3 OS=Mus musculus GN=Chi3l3 PE=1 SV=2 - [CH3L3_MOUSE]	11.06	1	3	3	13	-0.05	0.21	0.60	0.86
D3YXV1	Indoleamine 2,3-dioxygenase 1 OS=Mus musculus GN=Ido1 PE=2 SV=1 - [D3YXV1_MOUSE]	19.30	2	4	5	9	-0.02	-0.36	-0.09	0.05
P16872	Interleukin-7 receptor subunit alpha OS=Mus musculus GN=Il7r PE=2 SV=2 - [IL7RA_MOUSE]	5.23	1	1	1	2	-0.69	-1.45	-1.40	-2.17
P29391	Ferritin light chain 1 OS=Mus musculus GN=Ftl1 PE=1 SV=2 - [FRIL1_MOUSE]	33.33	4	5	5	33	-1.56	-1.25	-1.79	-1.57
Q5NC41	Protein 1810065E05Rik OS=Mus musculus GN=1810065E05Rik PE=4 SV=1 - [Q5NC41_MOUSE]	22.13	1	5	5	26	-0.62	-0.41	-0.79	-0.64
Q8C2L6	Transmembrane protein 161B OS=Mus musculus GN=Tmem161b PE=2 SV=1 - [T161B_MOUSE]	7.80	1	1	1	1	-1.19	-0.44	-1.48	-0.74
B2RQR5	Small G protein signaling modulator 1 OS=Mus musculus GN=Sgsm1 PE=2 SV=1 - [B2RQR5_MOUSE]	5.58	5	2	2	2	0.11	-0.65	-0.49	-1.25
Q99KK1	Receptor expression-enhancing protein 3 OS=Mus musculus GN=Reep3 PE=1 SV=1 - [REEP3_MOUSE]	9.06	1	2	2	2	-0.39	-0.34	-0.77	-0.72
Q9CR13	UPF0562 protein C7orf55 homolog OS=Mus musculus PE=3 SV=1 - [CG055_MOUSE]	13.27	1	1	1	2	-0.46	-0.76	-0.60	-0.90
P01897	H-2 class I histocompatibility antigen, L-D alpha chain OS=Mus musculus GN=H2-L PE=1 SV=2 - [HA1L_MOUSE]	30.11	4	1	8	56	-0.70	-0.64	-0.54	-0.47

Q8K352	SAM and SH3 domain-containing protein 3 OS=Mus musculus GN=Sash3 PE=2 SV=2 [SASH3_MOUSE]	7.89	1	2	2	6	0.43	0.72	0.30	0.40
Q6PB70	Anoctamin-8 OS=Mus musculus GN=Ano8 PE=2 SV=3 - [ANO8_MOUSE]	0.57	1	1	1	1	-1.13	-1.39	-1.46	-1.71
Q8R121-2	Isoform 2 of Protein Z-dependent protease inhibitor OS=Mus musculus GN=Serpina10 - [ZPI_MOUSE]	7.11	2	2	2	6	0.03	0.02	-0.02	-0.02
Q62261-2	Isoform 2 of Spectrin beta chain, non-erythrocytic 1 OS=Mus musculus GN=Sptbn1 - [SPTB2_MOUSE]	61.75	5	1	118	1236	-0.96	-1.10	-0.80	-0.95
F7CFC7	Protein 2010015L04Rik (Fragment) OS=Mus musculus GN=2010015L04Rik PE=2 SV=1 - [F7CFC7_MOUSE]	8.14	3	1	1	1	-0.72	-0.29	-1.03	-0.61
Q8BHC0	Lymphatic vessel endothelial hyaluronic acid receptor 1 OS=Mus musculus GN=Lyve1 PE=1 SV=1 - [LYVE1_MOUSE]	6.60	1	2	2	12	-1.04	-0.69	-1.51	-1.16
G3UZL7	H-2 class I histocompatibility antigen, TLA(B) alpha chain OS=Mus musculus GN=H2-T3 PE=2 SV=1 - [G3UZL7_MOUSE]	17.73	5	2	3	19	-0.53	-0.37	-1.22	-1.35
F6ZGN3	Unconventional myosin-XVIIIa (Fragment) OS=Mus musculus GN=Myo18a PE=4 SV=1 - [F6ZGN3_MOUSE]	10.56	1	1	1	3	-0.10	-0.97	0.04	-0.84
Q9Z2U2-2	Isoform 2 of Zinc finger protein 292 OS=Mus musculus GN=Zfp292 - [ZN292_MOUSE]	1.26	2	2	3	7	-2.51	-2.57	-0.91	-0.97
Q91XZ7	Protein Pcdhb3 OS=Mus musculus GN=Pcdhb3 PE=2 SV=1 - [Q91XZ7_MOUSE]	2.17	1	1	1	1	-0.81	-1.05	-1.12	-1.37
P01872	Ig mu chain C region secreted form OS=Mus musculus GN=Igh-6 PE=1 SV=2 - [IGHM_MOUSE]	8.37	2	3	3	13	-0.71	-0.77	-0.85	-1.00
Q9R257	Heme-binding protein 1 OS=Mus musculus GN=Hebp1 PE=1 SV=2 - [HEBP1_MOUSE]	41.05	1	4	4	30	0.13	0.99	0.62	0.57
Q9CYX7	RRP15-like protein OS=Mus musculus GN=Rrp15 PE=2 SV=2 [RRP15_MOUSE]	6.05	1	1	1	3	-1.56	-1.54	-1.59	-1.56
A2AUG1	F-box/WD repeat-containing protein 2 OS=Mus musculus GN=Fbxw2 PE=2 SV=1 - [A2AUG1_MOUSE]	7.46	1	1	1	1	-0.32	-0.31	-0.64	-0.64

O70494-3	Isoform 3 of Transcription factor Sp3 OS=Mus musculus GN=Sp3 - [SP3_MOUSE]	2.36	3	2	2	3	-1.00	-0.93	-1.17	-1.09
F6ZK01	Myeloblastin (Fragment) OS=Mus musculus GN=Prtn3 PE=3 SV=1 - [F6ZK01_MOUSE]	7.14	2	1	1	1	1.61	0.58	1.43	0.40
Q9CXP9	Exonuclease V OS=Mus musculus GN=Exo5 PE=2 SV=1 - [EXO5_MOUSE]	8.31	1	1	1	1	-1.66	-1.87	-1.49	-1.71
P19221	Prothrombin OS=Mus musculus GN=F2 PE=1 SV=1 - [THRB_MOUSE]	33.82	3	17	18	93	-0.45	-0.47	-0.41	-0.38
Q8K2Q7	BRO1 domain-containing protein BROX OS=Mus musculus GN=Brox PE=2 SV=1 - [BROX_MOUSE]	12.65	3	2	3	5	0.20	0.30	-0.05	0.05
Q02105	Complement C1q subcomponent subunit C OS=Mus musculus GN=C1qc PE=2 SV=2 - [C1QC_MOUSE]	16.67	1	3	3	13	-0.55	-0.85	-0.39	-0.51
P04117	Fatty acid-binding protein, adipocyte OS=Mus musculus GN=Fabp4 PE=1 SV=3 [FABP4_MOUSE]	73.48	2	10	11	386	-1.29	-1.23	-1.32	-1.20
Q61599	Rho GDP-dissociation inhibitor 2 OS=Mus musculus GN=Arhgdib PE=1 SV=3 - [GDIR2_MOUSE]	49.50	2	7	7	73	0.09	0.20	0.03	0.17
Q9CQJ2	PIH1 domain-containing protein 1 OS=Mus musculus GN=Pih1d1 PE=2 SV=1 - [PIHD1_MOUSE]	13.45	4	2	2	6	-1.42	-1.11	-1.51	-1.19
E9Q4T2	Protein Gm17511 OS=Mus musculus GN=Gm17511 PE=3 SV=1 - [E9Q4T2_MOUSE]	37.72	1	1	1	1	-0.72	-1.33	-0.36	-0.97
Q01339	Beta-2-glycoprotein 1 OS=Mus musculus GN=Apoh PE=1 SV=1 - [APOH_MOUSE]	34.78	3	11	11	69	-0.62	-0.52	-0.51	-0.45
Q06890	Clusterin OS=Mus musculus GN=Clu PE=1 SV=1 - [CLUS_MOUSE]	20.98	6	9	9	86	-0.41	-0.39	-0.30	-0.21
P49290	Eosinophil peroxidase OS=Mus musculus GN=Epx PE=1 SV=2 - [PERE_MOUSE]	10.89	1	6	8	35	-0.06	0.07	-0.09	-0.05
H3BJT2	Insulin-like growth factor-binding protein 4 (Fragment) OS=Mus musculus GN=Igfbp4 PE=4 SV=1 - [H3BJT2_MOUSE]	11.48	3	1	1	5	0.00	-0.54	-0.04	-0.42
P03953-2	Isoform 2 of Complement factor D OS=Mus musculus GN=Cfd - [CFAD_MOUSE]	25.58	2	3	3	17	-1.24	-1.29	-0.94	-0.90
F6ZH76	Hepatocyte nuclear factor 1-beta (Fragment) OS=Mus musculus GN=Hnf1b PE=2 SV=1 - [F6ZH76_MOUSE]	16.18	6	1	1	2	-0.53	-2.51	-1.58	-3.56

Q8BNQ3	Integral membrane protein GPR137B OS=Mus musculus GN=Gpr137b PE=1 SV=2 - [G137B_MOUSE]	5.45	1	1	1	4	-1.23	-1.12	-0.76	-0.65
P42703	Leukemia inhibitory factor receptor OS=Mus musculus GN=Lifr PE=1 SV=1 - [LIFR_MOUSE]	4.40	2	3	3	10	-0.49	-0.41	-0.73	-0.79
Q3UJ22	Gene model 648, (NCBI) OS=Mus musculus GN=Gm648 PE=2 SV=1 - [Q3UJ22_MOUSE]	3.29	1	1	1	6	-2.01	-1.54	-2.04	-1.35
Q91WK1	SPRY domain-containing protein 4 OS=Mus musculus GN=Spryd4 PE=2 SV=1 - [SPRY4_MOUSE]	12.56	1	4	4	8	-0.81	-0.61	-1.29	-1.09
Q8VCT4	Carboxylesterase 1D OS=Mus musculus GN=Ces1d PE=1 SV=1 [CES1D_MOUSE]	34.16	1	13	16	231	-1.42	-1.22	-1.45	-1.32
D3Z7F9	Putative sodium-coupled neutral amino acid transporter 8 (Fragment) OS=Mus musculus GN=Slc38a8 PE=2 SV=1 - [D3Z7F9_MOUSE]	10.68	4	1	2	2	0.18	0.28	-0.24	-0.15
Q8K448	ATP-binding cassette sub-family A member 5 OS=Mus musculus GN=Abca5 PE=1 SV=2 - [ABCA5_MOUSE]	0.67	1	1	1	1	-0.59	-0.56	-0.05	-0.03
P01753	Ig heavy chain V region 186-1 OS=Mus musculus PE=4 SV=2 - [HVM09_MOUSE]	5.98	4	1	1	2	-1.42	-1.34	-1.71	-1.63
D3YU18	Ankyrin repeat and SOCS box protein 10 OS=Mus musculus GN=Asb10 PE=2 SV=1 - [D3YU18_MOUSE]	4.65	1	1	1	2	0.92	-0.32	0.79	-0.46
P62965	Cellular retinoic acid-binding protein 1 OS=Mus musculus GN=Crabp1 PE=1 SV=2 - [RABP1_MOUSE]	7.30	1	1	1	4	-1.34	-0.92	-1.19	-0.80
P21614	Vitamin D-binding protein OS=Mus musculus GN=Gc PE=1 SV=2 - [VTDB_MOUSE]	39.50	1	15	15	138	-0.38	-0.36	-0.41	-0.41
Q0P5V2	Sine oculis-binding protein homolog OS=Mus musculus GN=Sobp PE=2 SV=1 - [SOBP_MOUSE]	1.62	1	2	2	2	-0.88	0.00	-0.93	-0.05
G3X9T8	Ceruloplasmin OS=Mus musculus GN=Cp PE=4 SV=1 - [G3X9T8_MOUSE]	36.89	7	29	30	159	-0.67	-0.71	-0.54	-0.56
P40936	Indolethylamine N-methyltransferase OS=Mus musculus GN=Inmt PE=1 SV=1 - [INMT_MOUSE]	15.91	1	3	3	17	-1.26	-1.27	-1.14	-1.22
P16015	Carbonic anhydrase 3 OS=Mus musculus GN=Ca3 PE=1 SV=3 - [CAH3_MOUSE]	71.92	1	16	16	364	-1.66	-1.66	-1.64	-1.56

E9PUZ8	C4b-binding protein (Fragment) OS=Mus musculus GN=C4bp PE=2 SV=1 - [E9PUZ8_MOUSE]	8.98	3	2	2	6	-0.42	-0.51	-0.55	-0.56
Q7TQI7	Ankyrin repeat and BTB/POZ domain-containing protein 2 OS=Mus musculus GN=Abtb2 PE=2 SV=1 - [ABTB2_MOUSE]	2.15	1	1	1	2	-0.72	-1.45	-0.91	-1.65
Q8BH61	Coagulation factor XIII A chain OS=Mus musculus GN=F13a1 PE=2 SV=3 - [F13A_MOUSE]	22.40	1	16	16	108	-1.14	-1.09	-1.18	-1.14
B1AY15	Protein Usp51 OS=Mus musculus GN=Usp51 PE=4 SV=1 - [B1AY15_MOUSE]	8.93	1	2	2	16	0.44	-0.49	0.57	-0.65
P14847	C-reactive protein OS=Mus musculus GN=Crp PE=2 SV=2 - [CRP_MOUSE]	2.67	1	1	1	1	-0.39	-0.32	-0.09	-0.02
Q61702	Inter-alpha-trypsin inhibitor heavy chain H1 OS=Mus musculus GN=Itih1 PE=1 SV=2 - [ITIH1_MOUSE]	24.92	2	15	15	174	-0.16	-0.22	-0.22	-0.28
Q00623	Apolipoprotein A-I OS=Mus musculus GN=Apoa1 PE=1 SV=2 - [APOA1_MOUSE]	44.70	1	15	16	173	-0.58	-0.60	-0.64	-0.57
E9Q1Y3	Apolipoprotein B-100 (Fragment) OS=Mus musculus GN=Apob PE=2 SV=1 - [E9Q1Y3_MOUSE]	1.30	2	4	4	8	-0.28	-0.14	-0.53	-0.10
P41317	Mannose-binding protein C OS=Mus musculus GN=Mbl2 PE=2 SV=2 - [MBL2_MOUSE]	19.67	1	4	4	15	-0.13	-0.54	0.11	-0.15
G3UYZ0	Large proline-rich protein BAG6 (Fragment) OS=Mus musculus GN=Bag6 PE=2 SV=1 - [G3UYZ0_MOUSE]	34.76	2	1	4	28	-0.28	0.10	-0.74	-0.36
P06909	Complement factor H OS=Mus musculus GN=Cfh PE=1 SV=2 - [CFAH_MOUSE]	32.74	4	26	30	128	-0.32	-0.40	-0.34	-0.32
P04186	Complement factor B OS=Mus musculus GN=Cfb PE=1 SV=2 - [CFAB_MOUSE]	22.21	9	16	16	68	-0.42	-0.40	-0.39	-0.44
P29699	Alpha-2-HS-glycoprotein OS=Mus musculus GN=Ahsg PE=1 SV=1 - [FETUA_MOUSE]	42.32	1	10	10	144	-0.04	-0.06	-0.09	-0.14
F6ZMJ4	Trinucleotide repeat-containing gene 6A protein OS=Mus musculus GN=Tnrc6a PE=2 SV=2 - [F6ZMJ4_MOUSE]	1.16	2	1	2	2	-0.06	-0.05	-0.48	-0.47
Q1W617-2	Isoform 2 of Protein Shroom4 OS=Mus musculus GN=Shroom4 - [SHRM4_MOUSE]	3.24	2	2	3	3	-0.24	-0.41	-0.27	-0.44
E9Q035	Protein Gm20425 OS=Mus musculus GN=Gm20425 PE=4 SV=1 - [E9Q035_MOUSE]	43.05	8	46	47	1036	-0.37	-0.32	-0.41	-0.33

Q61703	Inter-alpha-trypsin inhibitor heavy chain H2 OS=Mus musculus GN=Itih2 PE=1 SV=1 - [ITIH2_MOUSE]	15.64	3	12	12	47	-0.56	-0.36	-0.19	-0.32
Q06770	Corticosteroid-binding globulin OS=Mus musculus GN=Serpina6 PE=1 SV=1 - [CBG_MOUSE]	7.81	1	3	3	6	0.44	0.28	0.18	0.12
F8VQB6	Unconventional myosin-X OS=Mus musculus GN=Myo10 PE=1 SV=1 - [MYO10_MOUSE]	4.27	3	3	4	4	-0.48	-0.68	-0.39	-0.60
P06684	Complement C5 OS=Mus musculus GN=C5 PE=1 SV=2 - [CO5_MOUSE]	1.55	1	3	3	3	0.04	-0.07	-1.09	-1.21
Q61625	Glutamate receptor ionotropic, delta-2 OS=Mus musculus GN=Grid2 PE=1 SV=1 [GRID2_MOUSE]	2.58	1	2	2	3	-1.09	-1.19	-1.03	-1.13
E9Q2S9	Coiled-coil domain-containing protein 43 OS=Mus musculus GN=Ccdc43 PE=2 SV=1 - [E9Q2S9_MOUSE]	21.17	3	4	4	9	-0.67	-0.48	-1.06	-0.73
Q61247	Alpha-2-antiplasmin OS=Mus musculus GN=Serpinf2 PE=1 SV=1 - [A2AP_MOUSE]	5.70	2	2	2	15	-0.19	0.08	-0.25	0.03
Q9QUK0-2	Isoform 2 of Cyclin-dependent kinase-like 2 OS=Mus musculus GN=Cdkl2 - [CDKL2_MOUSE]	9.04	2	3	3	3	0.15	0.20	-0.94	-0.89
P35487	Pyruvate dehydrogenase E1 component subunit alpha, testis-specific form, mitochondrial OS=Mus musculus GN=Pdha2 PE=2 SV=1 - [ODPAT_MOUSE]	12.02	1	2	4	12	-0.77	-1.74	-0.82	-1.98
Q8BL99-3	Isoform 3 of Protein dopey-1 OS=Mus musculus GN=Dopey1 - [DOP1_MOUSE]	7.61	5	2	2	2	0.10	0.37	0.10	0.36
Q9ESB3	Histidine-rich glycoprotein OS=Mus musculus GN=Hrg PE=1 SV=2 - [HRG_MOUSE]	17.71	1	8	8	42	-0.72	-0.56	-1.04	-0.83
B8QI34	Liprin-alpha 2 OS=Mus musculus GN=Ppfia2 PE=2 SV=1 - [B8QI34_MOUSE]	3.50	2	2	4	12	-1.02	-0.88	-1.42	-1.28
F8VQI8	Sodium/hydrogen exchanger 10 OS=Mus musculus GN=Slc9c1 PE=2 SV=1 - [F8VQI8_MOUSE]	4.73	3	3	3	3	-1.40	-1.27	-1.38	-1.24
Q8K3I6	Interleukin-27 subunit alpha OS=Mus musculus GN=Il27 PE=1 SV=1 - [IL27A_MOUSE]	7.26	1	1	1	1	-0.56	-1.03	-1.18	-1.65
E9QNL4	Inhibitor of nuclear factor kappa-B kinase subunit alpha OS=Mus musculus GN=Chuk PE=2 SV=1 - [E9QNL4_MOUSE]	5.91	3	2	2	8	-2.36	-2.66	-2.21	-2.11

Q5DTY9	BTB/POZ domain-containing protein KCTD16 OS=Mus musculus GN=Kctd16 PE=1 SV=2 - [KCD16_MOUSE]	3.04	4	1	2	5	-1.68	-1.38	-1.50	-1.20
P20918	Plasminogen OS=Mus musculus GN=Plg PE=1 SV=3 - [PLMN_MOUSE]	32.27	2	19	20	106	-0.70	-0.62	-0.68	-0.67
E9PV24	Protein Fga OS=Mus musculus GN=Fga PE=2 SV=1 - [E9PV24_MOUSE]	40.94	2	22	23	192	-0.26	-0.25	-0.26	-0.26
Q8K0E8	Fibrinogen beta chain OS=Mus musculus GN=Fgb PE=2 SV=1 - [FIBB_MOUSE]	45.95	3	24	24	230	-0.34	-0.35	-0.25	-0.28
Q8CG14	Complement C1s-A subcomponent OS=Mus musculus GN=C1sa PE=2 SV=2 - [CS1A_MOUSE]	4.51	3	2	2	5	-0.32	-0.92	-0.43	-1.03
Q8BK58	HSPB1-associated protein 1 OS=Mus musculus GN=Hspbap1 PE=1 SV=2 - [HBAP1_MOUSE]	4.35	1	1	1	1	0.28	-0.18	-0.60	-1.06
Q00898	Alpha-1-antitrypsin 1-5 OS=Mus musculus GN=Serpina1e PE=1 SV=1 - [A1AT5_MOUSE]	28.57	2	2	12	347	-1.58	-2.01	-2.22	-2.12
E9Q714	Putative polypeptide N-acetylgalactosaminyltransferase-like protein 3 OS=Mus musculus GN=Wbscr17 PE=2 SV=1 - [E9Q714_MOUSE]	3.77	3	1	1	1	-0.62	-0.77	-1.53	-1.68
A2A9W7	ADP-ribosylation factor binding protein GGA3 OS=Mus musculus GN=Gga3 PE=2 SV=1 - [A2A9W7_MOUSE]	3.75	2	1	1	1	-2.04	-2.11	-1.63	-1.70
Q91X72	Hemopexin OS=Mus musculus GN=Hpx PE=1 SV=2 - [HEMO_MOUSE]	45.22	1	21	21	485	-0.22	-0.32	-0.21	-0.29
Q61704	Inter-alpha-trypsin inhibitor heavy chain H3 OS=Mus musculus GN=Itih3 PE=1 SV=3 - [ITIH3_MOUSE]	22.83	2	14	14	150	-0.60	-0.57	-0.66	-0.67
P97504	Cytoplasmic tyrosine-protein kinase BMX OS=Mus musculus GN=Bmx PE=2 SV=1 - [BMX_MOUSE]	3.84	2	2	2	5	-0.25	0.03	-0.50	-0.22
P01580	Interferon gamma OS=Mus musculus GN=Ifng PE=2 SV=1 - [IFNG_MOUSE]	3.87	1	1	1	1	-0.33	-0.53	-0.24	-0.45
P08905	Lysozyme C-2 OS=Mus musculus GN=Lyz2 PE=1 SV=2 - [LYZ2_MOUSE]	14.86	2	2	2	84	-0.38	-0.62	-0.33	-0.57
Q91XL1	Leucine-rich HEV glycoprotein (Precursor) OS=Mus musculus GN=Lrg1 PE=2 SV=1 - [Q91XL1_MOUSE]	14.04	1	4	5	34	-0.56	-0.39	-0.55	-0.55
P11247	Myeloperoxidase OS=Mus musculus GN=Mpo PE=2 SV=2 - [PERM_MOUSE]	19.78	5	10	13	33	-0.03	-0.25	-0.07	-0.21

A6X935-2	Isoform 2 of Inter alpha-trypsin inhibitor, heavy chain 4 OS=Mus musculus GN=Itih4 - [ITIH4_MOUSE]	31.56	4	22	22	179	-0.08	-0.27	-0.04	-0.14
P11859	Angiotensinogen OS=Mus musculus GN=Agt PE=1 SV=1 - [ANGT_MOUSE]	6.29	2	3	3	5	0.59	-0.75	0.94	-0.41
Q9JKK8	Serine/threonine-protein kinase ATR OS=Mus musculus GN=Atr PE=1 SV=2 - [ATR_MOUSE]	2.50	2	3	3	7	-0.91	-0.34	-1.66	-1.09
P13634	Carbonic anhydrase 1 OS=Mus musculus GN=Ca1 PE=2 SV=4 - [CAH1_MOUSE]	72.41	2	12	12	2427	-1.80	-1.73	-1.82	-1.74
O35954	Membrane-associated phosphatidylinositol transfer protein 1 OS=Mus musculus GN=Pitpnm1 PE=1 SV=1 - [PITM1_MOUSE]	3.86	1	2	3	4	-1.32	-1.33	-1.40	-1.41
D3YW52	Alpha-2-macroglobulin OS=Mus musculus GN=Pzp PE=2 SV=1 - [D3YW52_MOUSE]	33.84	4	41	41	401	-0.29	-0.32	-0.32	-0.44
P34928	Apolipoprotein C-I OS=Mus musculus GN=Apoc1 PE=2 SV=1 - [APOC1_MOUSE]	18.18	1	2	2	2	-0.66	-0.31	-0.63	-0.39
D3YTY9	Kininogen-1 OS=Mus musculus GN=Kng1 PE=2 SV=1 - [D3YTY9_MOUSE]	34.58	5	15	16	155	-0.43	-0.38	-0.40	-0.35
F8WJL9	BCL-6 corepressor-like protein 1 (Fragment) OS=Mus musculus GN=Bcorl1 PE=4 SV=1 - [F8WJL9_MOUSE]	3.31	3	2	2	2	0.14	0.28	-1.08	-0.94
Q07456	Protein AMBP OS=Mus musculus GN=Ambp PE=1 SV=2 - [AMBP_MOUSE]	17.77	1	5	5	19	-0.12	-0.59	-0.19	-0.72
E9Q7P1	Protein Col22a1 OS=Mus musculus GN=Col22a1 PE=2 SV=1 - [E9Q7P1_MOUSE]	2.79	1	2	2	3	-0.68	-1.63	-0.05	-1.00
Q80V85	Tripartite motif-containing protein 62 OS=Mus musculus GN=Trim62 PE=2 SV=1 - [TRI62_MOUSE]	4.63	2	1	2	36	-1.27	-0.61	-1.69	-1.03
P55002	Microfibrillar-associated protein 2 OS=Mus musculus GN=Mfap2 PE=2 SV=1 - [MFAP2_MOUSE]	5.46	2	1	1	1	-1.29	-1.57	-0.86	-1.14
P01027	Complement C3 OS=Mus musculus GN=C3 PE=1 SV=3 - [CO3_MOUSE]	39.45	4	63	63	551	-0.39	-0.42	-0.34	-0.32
Q9D8G5	Regenerating islet-derived protein 4 OS=Mus musculus GN=Reg4 PE=2 SV=1 - [REG4_MOUSE]	40.13	1	7	7	47	-0.34	-0.41	-0.31	-0.41
P97425	Eosinophil cationic protein 2 OS=Mus musculus GN=Ear2 PE=2 SV=1 - [ECP2_MOUSE]	24.36	1	3	3	43	-0.03	-0.22	0.05	-0.04

Q99K95	Protein RTF2 homolog OS=Mus musculus GN=Rtfdc1 PE=2 SV=1 - [RTF2_MOUSE]	3.26	1	1	1	1	-0.31	-0.09	-0.52	-0.30
P58801	Receptor-interacting serine/threonine-protein kinase 2 OS=Mus musculus GN=Ripk2 PE=2 SV=1 - [RIPK2_MOUSE]	5.94	1	2	2	5	0.07	-0.23	-1.09	-1.53
Q60590	Alpha-1-acid glycoprotein 1 OS=Mus musculus GN=Orm1 PE=1 SV=1 - [A1AG1_MOUSE]	19.32	3	1	4	25	-0.20	-0.11	-0.29	-0.20
P26262	Plasma kallikrein OS=Mus musculus GN=Klkb1 PE=1 SV=2 [KLKB1_MOUSE]	3.29	1	1	1	3	-1.45	-1.20	-1.51	-1.27
Q923L7	Ear6 protein OS=Mus musculus GN=Ear6 PE=2 SV=1 - [Q923L7_MOUSE]	8.39	1	1	1	4	0.07	0.42	0.06	0.41
P61939	Thyroxine-binding globulin OS=Mus musculus GN=Serpina7 PE=1 SV=1 - [THBG_MOUSE]	8.13	3	3	3	5	-0.57	-0.73	-0.35	-0.50
F2Z461	E3 ISG15--protein ligase Herc6 OS=Mus musculus GN=Herc6 PE=2 SV=1 - [HERC6_MOUSE]	2.89	1	2	2	2	-1.32	-1.07	-1.92	-1.67
P41245	Matrix metalloproteinase-9 OS=Mus musculus GN=Mmp9 PE=2 SV=2 - [MMP9_MOUSE]	1.10	1	1	1	1	-0.15	-0.51	-0.09	-0.46
P27005	Protein S100-A8 OS=Mus musculus GN=S100a8 PE=1 SV=3 - [S10A8_MOUSE]	26.97	1	1	1	5	0.56	-0.17	0.53	-0.21
Q8CI78	Required for meiotic nuclear division protein 1 homolog OS=Mus musculus GN=Rmnd1 PE=2 SV=1 - [RMND1_MOUSE]	3.33	1	1	1	1	-0.37	-0.17	-0.40	-0.20
D3Z5N4	Glycogenin-1 (Fragment) OS=Mus musculus GN=Gyg PE=2 SV=2 - [D3Z5N4_MOUSE]	17.74	1	1	4	18	-0.39	-0.34	-0.30	-0.25
Q9D3S3	Sorting nexin-29 OS=Mus musculus GN=Snx29 PE=1 SV=2 - [SNX29_MOUSE]	2.32	3	1	2	15	-1.50	-1.36	-1.32	-1.18
Q8VCM7	Fibrinogen gamma chain OS=Mus musculus GN=Fgg PE=2 SV=1 - [FIBG_MOUSE]	39.91	1	15	15	131	-0.31	-0.29	-0.36	-0.30
D3YXQ4	Beta-chimaerin (Fragment) OS=Mus musculus GN=Chn2 PE=2 SV=1 - [D3YXQ4_MOUSE]	21.36	1	1	1	2	-1.54	-1.14	-2.24	-1.84
Q8K2A1-2	Isoform 2 of PTB domain-containing engulfment adapter protein 1 OS=Mus musculus GN=Gulp1 - [GULP1_MOUSE]	10.68	3	3	3	8	-0.83	-0.07	-0.55	0.21

O08692	Myeloid bactenecin (F1) OS=Mus musculus GN=Ngp PE=2 SV=1 - [O08692_MOUSE]	32.93	1	6	6	33	-0.01	-0.26	-0.07	-0.16
P51437	Cathelin-related antimicrobial peptide OS=Mus musculus GN=Camp PE=2 SV=1 [CRAMP_MOUSE]	14.45	1	2	2	9	-0.04	-0.23	-0.40	-0.56
O09049	Regenerating islet-derived protein 3-gamma OS=Mus musculus GN=Reg3g PE=1 SV=1 - [REG3G_MOUSE]	31.03	1	3	4	39	-0.71	-0.47	-0.35	-0.13
G3X8T9	Serine (Or cysteine) peptidase inhibitor, clade A, member 3N, isoform CRA_a OS=Mus musculus GN=Serpina3n PE=3 SV=1 - [G3X8T9_MOUSE]	38.28	2	1	13	92	0.24	0.12	-0.20	-0.32
P31725	Protein S100-A9 OS=Mus musculus GN=S100a9 PE=1 SV=3 - [S10A9_MOUSE]	26.55	1	3	3	24	0.04	0.05	0.09	0.11
F6SMY7	Probable E3 ubiquitin-protein ligase MYCBP2 (Fragment) OS=Mus musculus GN=Mycbp2 PE=2 SV=1 - [F6SMY7_MOUSE]	2.91	6	7	7	7	-0.25	0.25	0.17	0.35
P35230	Regenerating islet-derived protein 3-beta OS=Mus musculus GN=Reg3b PE=1 SV=1 - [REG3B_MOUSE]	20.57	1	2	3	5	-0.14	-0.86	-0.34	-0.95
Q61646	Haptoglobin OS=Mus musculus GN=Hp PE=1 SV=1 - [HPT_MOUSE]	39.19	1	13	13	170	-0.07	-0.34	-0.12	-0.38
P08071	Lactotransferrin OS=Mus musculus GN=Ltf PE=2 SV=4 - [TRFL_MOUSE]	41.58	1	24	24	89	-0.04	-0.13	-0.17	-0.27
P12246	Serum amyloid P-component OS=Mus musculus GN=Apcs PE=1 SV=2 - [SAMP_MOUSE]	18.75	1	3	3	15	0.24	-0.42	0.96	0.30
P07361	Alpha-1-acid glycoprotein 2 OS=Mus musculus GN=Orm2 PE=1 SV=1 - [A1AG2_MOUSE]	24.15	3	3	5	25	-0.30	-0.73	-0.12	-0.56
Q3UP87	Neutrophil elastase OS=Mus musculus GN=Elane PE=2 SV=1 - [ELNE_MOUSE]	2.64	1	1	1	1	0.16	-0.06	0.33	0.11
Q4V9Z5-3	Isoform 3 of Seizure 6-like protein 2 OS=Mus musculus GN=Sez6l2 - [SE6L2_MOUSE]	3.13	3	1	1	2	-2.35	-2.38	-3.31	-3.35

Supplementary Table 2. Significantly modulated proteins in the intestine of *Crh -/-* vs. wt mice (T-Test p-value ≤ 0.05)

Accession	Description	Crh-/- (1) vs. WT (1)	Crh-/- (1) vs. WT (2)	Crh-/-(2) vs. WT (1)	Crh-/- (2) vs. WT (2)	T-Test p-value
Q2M2N2	Speckle-type POZ protein-like OS=Mus musculus GN=Spopl PE=2 SV=3 - [SPOPL_MOUSE]	0.40	0.40	0.40	0.41	0.00
P40124	Adenylyl cyclase-associated protein 1 OS=Mus musculus GN=Cap1 PE=1 SV=4 - [CAP1_MOUSE]	0.48	0.47	0.48	0.48	0.00
P00688	Pancreatic alpha-amylase OS=Mus musculus GN=Amy2 PE=1 SV=2 - [AMYP_MOUSE]	1.93	1.97	1.93	1.95	0.00
A2AQ53	Fibrillin-1 OS=Mus musculus GN=Fbn1 PE=4 SV=1 - [A2AQ53_MOUSE]	-0.90	-0.91	-0.91	-0.93	0.00
Q9CYX7	RRP15-like protein OS=Mus musculus GN=Rrp15 PE=2 SV=2 - [RRP15_MOUSE]	-1.56	-1.54	-1.59	-1.56	0.00
P21981	Protein-glutamine gamma-glutamyltransferase 2 OS=Mus musculus GN=Tgm2 PE=1 SV=4 [TGM2_MOUSE]	-0.91	-0.92	-0.90	-0.90	0.00
Q61292	Laminin subunit beta-2 OS=Mus musculus GN=Lamb2 PE=2 SV=2 - [LAMB2_MOUSE]	-0.68	-0.69	-0.69	-0.70	0.00
E9Q0B5	Protein Fcgbp OS=Mus musculus GN=Fcgbp PE=2 SV=1 - [E9Q0B5_MOUSE]	1.14	1.13	1.13	1.10	0.00
P46412	Glutathione peroxidase 3 OS=Mus musculus GN=Gpx3 PE=2 SV=2 - [GPX3_MOUSE]	-0.91	-0.91	-0.90	-0.89	0.00
E9Q7N5	Ribosome-releasing factor 2, mitochondrial OS=Mus musculus GN=Gfm2 PE=2 SV=1 - [E9Q7N5_MOUSE]	0.17	0.18	0.17	0.18	0.00
Q80XJ3	Tetratricopeptide repeat protein 28 OS=Mus musculus GN=Ttc28 PE=2 SV=3 - [TTC28_MOUSE]	-1.13	-1.15	-1.15	-1.17	0.00
P26039	Talin-1 OS=Mus musculus GN=Tln1 PE=1 SV=2 - [TLN1_MOUSE]	0.61	0.63	0.61	0.62	0.00
P05784	Keratin, type I cytoskeletal 18 OS=Mus musculus GN=Krt18 PE=1 SV=5 - [K1C18_MOUSE]	0.63	0.61	0.61	0.62	0.00
E9Q7L0	Protein Ogdhl OS=Mus musculus GN=Ogdhl PE=2 SV=1 - [E9Q7L0_MOUSE]	-0.86	-0.85	-0.83	-0.83	0.00

Q8CGK3	Lon protease homolog, mitochondrial OS=Mus musculus GN=Lonp1 PE=1 SV=2 - [LONM_MOUSE]	0.21	0.21	0.21	0.22	0.00
P97872	Dimethylaniline monooxygenase [N-oxide-forming] 5 OS=Mus musculus GN=Fmo5 PE=2 SV=4 [FMO5_MOUSE]	-1.65	-1.70	-1.64	-1.70	0.00
P26339	Chromogranin-A OS=Mus musculus GN=Chga PE=1 SV=1 - [CMGA_MOUSE]	-1.56	-1.52	-1.60	-1.55	0.00
E9PZP8	Protein Herc1 OS=Mus musculus GN=Herc1 PE=2 SV=1 - [E9PZP8_MOUSE]	-0.43	-0.43	-0.41	-0.42	0.00
Q8VDN2	Sodium/potassium-transporting ATPase subunit alpha-1 OS=Mus musculus GN=Atp1a1 PE=1 SV=1 - [AT1A1_MOUSE]	0.27	0.27	0.26	0.28	0.00
G5E846	Peripherin OS=Mus musculus GN=Prph PE=3 SV=1 - [G5E846_MOUSE]	-0.64	-0.62	-0.65	-0.62	0.00
P13634	Carbonic anhydrase 1 OS=Mus musculus GN=Ca1 PE=2 SV=4 - [CAH1_MOUSE]	-1.80	-1.73	-1.82	-1.74	0.00
Q8VI78	Phospholipase A1 member A OS=Mus musculus GN=Pla1a PE=2 SV=3 - [PLA1A_MOUSE]	0.31	0.32	0.31	0.32	0.00
Q08857	Platelet glycoprotein 4 OS=Mus musculus GN=Cd36 PE=1 SV=2 - [CD36_MOUSE]	-1.45	-1.44	-1.42	-1.37	0.00
B1B0C7	Basement membrane-specific heparan sulfate proteoglycan core protein OS=Mus musculus GN=Hspg2 PE=2 SV=1 - [B1B0C7_MOUSE]	-0.63	-0.60	-0.63	-0.60	0.00
Q9D816	Cytochrome P450 2C55 OS=Mus musculus GN=Cyp2c55 PE=2 SV=1 - [CP255_MOUSE]	-1.08	-1.08	-1.14	-1.09	0.00
Q63886	UDP-glucuronosyltransferase 1-1 OS=Mus musculus GN=Ugt1a1 PE=2 SV=2 - [UD11_MOUSE]	-1.40	-1.32	-1.40	-1.40	0.00
Q91V76	Ester hydrolase C11orf54 homolog OS=Mus musculus PE=2 SV=1 - [CK054_MOUSE]	-1.06	-1.02	-1.08	-1.04	0.00

Q8BTX9	Inactive hydroxysteroid dehydrogenase-like protein 1 OS=Mus musculus GN=Hsdl1 PE=2 SV=1 - [HSDL1_MOUSE]	-0.67	-0.64	-0.68	-0.65	0.00
Q9R1I1	Carbohydrate sulfotransferase 4 OS=Mus musculus GN=Chst4 PE=2 SV=1 [CHST4_MOUSE]	0.87	0.89	0.83	0.85	0.00
P11499	Heat shock protein HSP 90-beta OS=Mus musculus GN=Hsp90ab1 PE=1 SV=3 - [HS90B_MOUSE]	0.38	0.39	0.39	0.40	0.00
M0QWD1	Eukaryotic translation initiation factor 4E type 2 (Fragment) OS=Mus musculus GN=Eif4e2 PE=3 SV=1 - [M0QWD1_MOUSE]	0.78	0.74	0.77	0.73	0.00
O35381	Acidic leucine-rich nuclear phosphoprotein 32 family member A OS=Mus musculus GN=Anp32a PE=1 SV=1 - [AN32A_MOUSE]	-0.51	-0.50	-0.53	-0.49	0.00
P16015	Carbonic anhydrase 3 OS=Mus musculus GN=Ca3 PE=1 SV=3 - [CAH3_MOUSE]	-1.66	-1.66	-1.64	-1.56	0.00
Q9R069	Basal cell adhesion molecule OS=Mus musculus GN=Bcam PE=2 SV=1 - [BCAM_MOUSE]	-0.56	-0.53	-0.54	-0.53	0.00
E0CXN5	Glycerol-3-phosphate dehydrogenase [NAD(+)], cytoplasmic OS=Mus musculus GN=Gpd1 PE=2 SV=1 - [E0CXN5_MOUSE]	-1.15	-1.19	-1.11	-1.16	0.00
Q80VA0	N-acetylgalactosaminyltransferase 7 OS=Mus musculus GN=Galnt7 PE=2 SV=2 - [GALT7_MOUSE]	0.26	0.27	0.25	0.25	0.00
Q8VCZ7	Zinc finger and BTB domain-containing protein 7C OS=Mus musculus GN=Zbtb7c PE=2 SV=1 - [ZBT7C_MOUSE]	0.75	0.74	0.79	0.78	0.00
A1BN54	Alpha actinin 1a OS=Mus musculus GN=Actn1 PE=2 SV=1 [A1BN54_MOUSE]	0.55	0.58	0.55	0.55	0.00
P48678	Prelamin-A/C OS=Mus musculus GN=Lmna PE=1 SV=2 - [LMNA_MOUSE]	-0.23	-0.22	-0.23	-0.22	0.00
A8DUK4	Beta-globin OS=Mus musculus GN=Hbb-b1 PE=2 SV=1 - [A8DUK4_MOUSE]	-2.24	-2.20	-2.16	-2.08	0.00

Q08EC7	Pla2g4c protein OS=Mus musculus GN=Pla2g4c PE=2 SV=1 - [Q08EC7_MOUSE]	2.24	2.13	2.17	2.08	0.00
Q9WU78	Programmed cell death 6-interacting protein OS=Mus musculus GN=Pdcd6ip PE=1 SV=3 - [PDC6I_MOUSE]	0.57	0.53	0.54	0.53	0.00
Q60673	Receptor-type tyrosine-protein phosphatase-like N OS=Mus musculus GN=Ptprn PE=2 SV=2 - [PTPRN_MOUSE]	-0.81	-0.76	-0.81	-0.79	0.00
Q9JHF5	A3 subunit of vacuolar-adenosine triphosphatase OS=Mus musculus GN=Tcirg1 PE=2 SV=1 - [Q9JHF5_MOUSE]	-0.45	-0.43	-0.47	-0.46	0.00
E9PV24	Protein Fga OS=Mus musculus GN=Fga PE=2 SV=1 - [E9PV24_MOUSE]	-0.26	-0.25	-0.26	-0.26	0.00
Q8VHX6	Filamin-C OS=Mus musculus GN=Flnc PE=1 SV=3 - [FLNC_MOUSE]	0.52	0.53	0.49	0.50	0.00
Q8BH61	Coagulation factor XIII A chain OS=Mus musculus GN=F13a1 PE=2 SV=3 - [F13A_MOUSE]	-1.14	-1.09	-1.18	-1.14	0.00
P97449	Aminopeptidase N OS=Mus musculus GN=Anpep PE=1 SV=4 - [AMPN_MOUSE]	-0.48	-0.44	-0.46	-0.46	0.00
Q8VIB3	Type 2 lactosamine alpha-2,3-sialyltransferase OS=Mus musculus GN=St3gal6 PE=2 SV=3 - [SIA10_MOUSE]	1.09	1.04	1.13	1.09	0.00
A2A6Q8	Myosin light chain 4 (Fragment) OS=Mus musculus GN=Myl4 PE=2 SV=1 - [A2A6Q8_MOUSE]	-0.90	-0.95	-0.91	-0.87	0.00
O88987	A-kinase anchor protein 3 OS=Mus musculus GN=Akap3 PE=2 SV=2 - [AKAP3_MOUSE]	1.03	1.03	1.09	1.09	0.00
Q8BHZ0	Protein FAM49A OS=Mus musculus GN=Fam49a PE=2 SV=1 - [FA49A_MOUSE]	-0.61	-0.64	-0.67	-0.64	0.00
P62737	Actin, aortic smooth muscle OS=Mus musculus GN=Acta2 PE=1 SV=1 - [ACTA_MOUSE]	0.49	0.46	0.47	0.45	0.00
P62334	26S protease regulatory subunit 10B OS=Mus musculus GN=Psmc6 PE=1 SV=1 - [PRS10_MOUSE]	0.26	0.27	0.28	0.27	0.00

Q8VCW8	Acyl-CoA synthetase family member 2, mitochondrial OS=Mus musculus GN=Acsf2 PE=2 SV=1 - [ACSF2_MOUSE]	-0.73	-0.69	-0.71	-0.67	0.00
P08113	Endoplasmin OS=Mus musculus GN=Hsp90b1 PE=1 SV=2 - [ENPL_MOUSE]	0.73	0.76	0.69	0.72	0.00
P09036	Serine protease inhibitor Kazal-type 3 OS=Mus musculus GN=Spink3 PE=1 SV=1 - [ISK3_MOUSE]	1.50	1.51	1.44	1.57	0.00
O35954	Membrane-associated phosphatidylinositol transfer protein 1 OS=Mus musculus GN=Pitpnm1 PE=1 SV=1 - [PITM1_MOUSE]	-1.32	-1.33	-1.40	-1.41	0.00
Q99KI0	Aconitate hydratase, mitochondrial OS=Mus musculus GN=Aco2 PE=1 SV=1 - [ACON_MOUSE]	-0.47	-0.44	-0.47	-0.44	0.00
Q9CQ62	2,4-dienoyl-CoA reductase, mitochondrial OS=Mus musculus GN=Decr1 PE=1 SV=1 - [DECR_MOUSE]	-0.37	-0.35	-0.36	-0.34	0.00
Q78PY7	Staphylococcal nuclease domain-containing protein 1 OS=Mus musculus GN=Snd1 PE=1 SV=1 - [SND1_MOUSE]	0.37	0.36	0.34	0.35	0.00
P97927	Laminin subunit alpha-4 OS=Mus musculus GN=Lama4 PE=1 SV=2 - [LAMA4_MOUSE]	-0.89	-0.82	-0.82	-0.84	0.00
E9PX70	Collagen alpha-1(XII) chain OS=Mus musculus GN=Col12a1 PE=2 SV=1 - [E9PX70_MOUSE]	0.54	0.51	0.49	0.49	0.00
Q9ET78	Junctophilin-2 OS=Mus musculus GN=Jph2 PE=1 SV=2 - [JPH2_MOUSE]	-0.40	-0.42	-0.38	-0.39	0.00
B7ZNH7	Collagen alpha-1(XIV) chain OS=Mus musculus GN=Col14a1 PE=2 SV=1 - [B7ZNH7_MOUSE]	-0.83	-0.79	-0.80	-0.76	0.00
Q3UKJ7	WD40 repeat-containing protein SMU1 OS=Mus musculus GN=Smu1 PE=2 SV=2 - [SMU1_MOUSE]	-0.48	-0.45	-0.45	-0.44	0.00
O35344	Importin subunit alpha-4 OS=Mus musculus GN=Kpna3 PE=1 SV=1 - [IMA4_MOUSE]	0.37	0.36	0.34	0.33	0.00

Q60604	Adseverin OS=Mus musculus GN=Scin PE=1 SV=3 - [ADSV_MOUSE]	0.76	0.80	0.81	0.83	0.00
Q08091	Calponin-1 OS=Mus musculus GN=Cnn1 PE=2 SV=1 - [CNN1_MOUSE]	0.92	0.94	0.85	0.90	0.00
Q8BK64	Activator of 90 kDa heat shock protein ATPase homolog 1 OS=Mus musculus GN=Ahsa1 PE=2 SV=2 - [AHSA1_MOUSE]	0.25	0.25	0.23	0.24	0.00
P54310	Hormone-sensitive lipase OS=Mus musculus GN=Lipe PE=1 SV=2 - [LIPS_MOUSE]	-1.19	-1.20	-1.12	-1.24	0.00
Q8K2B3	Succinate dehydrogenase [ubiquinone] flavoprotein subunit, mitochondrial OS=Mus musculus GN=Sdha PE=1 SV=1 - [DHSA_MOUSE]	-0.46	-0.46	-0.50	-0.49	0.00
Q61941	NAD(P) transhydrogenase, mitochondrial OS=Mus musculus GN=Nnt PE=1 SV=2 - [NNTM_MOUSE]	1.35	1.24	1.35	1.28	0.00
Q91ZX7	Prolow-density lipoprotein receptor-related protein 1 OS=Mus musculus GN=Lrp1 PE=1 SV=1 - [LRP1_MOUSE]	-0.20	-0.20	-0.20	-0.22	0.00
Q9D6R2	Isocitrate dehydrogenase [NAD] subunit alpha, mitochondrial OS=Mus musculus GN=Idh3a PE=1 SV=1 - [IDH3A_MOUSE]	0.23	0.22	0.23	0.21	0.00
P45952	Medium-chain specific acyl-CoA dehydrogenase, mitochondrial OS=Mus musculus GN=Acadm PE=1 SV=1 - [ACADM_MOUSE]	-0.58	-0.57	-0.53	-0.58	0.00
Q9JK53	Prolargin OS=Mus musculus GN=Prelp PE=2 SV=2 - [PRELP_MOUSE]	-0.57	-0.54	-0.52	-0.57	0.00
P04117	Fatty acid-binding protein, adipocyte OS=Mus musculus GN=Fabp4 PE=1 SV=3 - [FABP4_MOUSE]	-1.29	-1.23	-1.32	-1.20	0.00
Q9JKR6	Hypoxia up-regulated protein 1 OS=Mus musculus GN=Hyou1 PE=1 SV=1 - [HYOU1_MOUSE]	0.51	0.56	0.52	0.52	0.00
Q9D8U3	Endoplasmic reticulum resident protein 27 OS=Mus musculus GN=Erp27 PE=2 SV=1 [ERP27_MOUSE]	2.38	2.28	2.32	2.15	0.00

F6XZQ5	CMP-N-acetylneuraminate-beta 1,4-galactoside alpha-2,3-sialyltransferase (Fragment) OS=Mus musculus GN=St3gal3 PE=4 SV=1 - [F6XZQ5_MOUSE]	1.26	1.21	1.35	1.30	0.00
Q3UW64	Bifunctional UDP-N-acetylglucosamine 2-epimerase/N-acetylmannosamine kinase OS=Mus musculus GN=Gne PE=2 SV=1 - [Q3UW64_MOUSE]	0.69	0.65	0.71	0.65	0.00
Q9CR35	Chymotrypsinogen B OS=Mus musculus GN=Ctrb1 PE=2 SV=1 - [CTRB1_MOUSE]	2.69	2.69	2.48	2.51	0.00
P97805	Protein FAM3D OS=Mus musculus GN=Fam3d PE=2 SV=2 - [FAM3D_MOUSE]	0.65	0.59	0.64	0.65	0.00
Q8BMK1	Methyltransferase-like protein 2 OS=Mus musculus GN=Mettl2 PE=2 SV=2 - [METL2_MOUSE]	-0.55	-0.53	-0.51	-0.49	0.00
P35700	Peroxiredoxin-1 OS=Mus musculus GN=Prdx1 PE=1 SV=1 [PRDX1_MOUSE]	-0.25	-0.23	-0.26	-0.25	0.00
Q8C1Q4	Meteorin OS=Mus musculus GN=Metrn PE=2 SV=2 - [METRN_MOUSE]	0.63	0.65	0.59	0.60	0.00
Q9CQN1	Heat shock protein 75 kDa, mitochondrial OS=Mus musculus GN=Trap1 PE=1 SV=1 [TRAP1_MOUSE]	0.24	0.26	0.25	0.26	0.00
Q91WG0	Carboxylesterase 2 OS=Mus musculus GN=Ces2c PE=2 SV=1 - [Q91WG0_MOUSE]	-0.92	-0.98	-0.91	-1.00	0.00
Q91V12-2	Isoform A of Cytosolic acyl coenzyme A thioester hydrolase OS=Mus musculus GN=Acot7 - [BACH_MOUSE]	0.31	0.29	0.31	0.32	0.00
A3KGF9	Uncharacterized protein C15orf52 homolog OS=Mus musculus PE=1 SV=1 - [CO052_MOUSE]	1.01	0.91	1.00	0.96	0.00
Q8BK48	Carboxylesterase 5, isoform CRA_b OS=Mus musculus GN=Ces2e PE=2 SV=1 - [Q8BK48_MOUSE]	-0.54	-0.55	-0.58	-0.59	0.00
P02088	Hemoglobin subunit beta-1 OS=Mus musculus GN=Hbb-b1 PE=1 SV=2 - [HBB1_MOUSE]	0.60	0.65	0.63	0.67	0.00

P70662-2	Isoform 2 of LIM domain-binding protein 1 OS=Mus musculus GN=Ldb1 - [LDB1_MOUSE]	0.12	0.12	0.13	0.13	0.00
Q91Z92	Beta-1,3-galactosyltransferase 6 OS=Mus musculus GN=B3galt6 PE=2 SV=1 - [B3GT6_MOUSE]	0.52	0.49	0.51	0.47	0.00
Q8BH24	Transmembrane 9 superfamily member 4 OS=Mus musculus GN=Tm9sf4 PE=2 SV=1 - [TM9S4_MOUSE]	0.39	0.40	0.41	0.37	0.00
Q8JZZ0	UDP-glucuronosyltransferase 3A2 OS=Mus musculus GN=Ugt3a2 PE=2 SV=2 - [UD3A2_MOUSE]	-0.85	-0.92	-0.84	-0.91	0.00
Q9DCV7	Keratin, type II cytoskeletal 7 OS=Mus musculus GN=Krt7 PE=1 SV=1 - [K2C7_MOUSE]	-0.61	-0.56	-0.55	-0.57	0.00
P50544	Very long-chain specific acyl-CoA dehydrogenase, mitochondrial OS=Mus musculus GN=Acadvl PE=1 SV=3 - [ACADV_MOUSE]	-0.23	-0.23	-0.22	-0.21	0.00
Q61503	5'-nucleotidase OS=Mus musculus GN=Nt5e PE=1 SV=2 - [5NTD_MOUSE]	-1.48	-1.41	-1.43	-1.31	0.00
H3BLI9	TSC22 domain family protein 1 OS=Mus musculus GN=Tsc22d1 PE=4 SV=1 - [H3BLI9_MOUSE]	-0.65	-0.72	-0.66	-0.65	0.00
P62192	26S protease regulatory subunit 4 OS=Mus musculus GN=Psmc1 PE=1 SV=1 - [PRS4_MOUSE]	0.41	0.43	0.44	0.46	0.00
H3BLA9	Sentrin-specific protease 6 (Fragment) OS=Mus musculus GN=Senp6 PE=2 SV=1 - [H3BLA9_MOUSE]	-2.24	-2.14	-2.34	-2.39	0.00
Q9JHI5	Isovaleryl-CoA dehydrogenase, mitochondrial OS=Mus musculus GN=Ivd PE=1 SV=1 - [IVD_MOUSE]	-0.40	-0.40	-0.44	-0.39	0.00
Q91WQ3	Tyrosine--tRNA ligase, cytoplasmic OS=Mus musculus GN=Yars PE=2 SV=3 - [SYYC_MOUSE]	0.34	0.33	0.31	0.31	0.00
Q3V2J0	Protein FAM92B OS=Mus musculus GN=Fam92b PE=2 SV=2 - [FA92B_MOUSE]	0.22	0.21	0.21	0.20	0.00

P40936	Indolethylamine N-methyltransferase OS=Mus musculus GN=Inmt PE=1 SV=1 - [INMT_MOUSE]	-1.26	-1.27	-1.14	-1.22	0.00
E9PUL3	Calcium-activated chloride channel regulator 4 OS=Mus musculus GN=Clca4 PE=4 SV=1 - [E9PUL3_MOUSE]	0.60	0.57	0.64	0.62	0.00
P97379-2	Isoform B of Ras GTPase-activating protein-binding protein 2 OS=Mus musculus GN=G3bp2 - [G3BP2_MOUSE]	-0.26	-0.28	-0.25	-0.28	0.00
P10126	Elongation factor 1-alpha 1 OS=Mus musculus GN=Eef1a1 PE=1 SV=3 - [EF1A1_MOUSE]	0.57	0.61	0.55	0.55	0.00
P22361	Hepatocyte nuclear factor 1-alpha OS=Mus musculus GN=Hnf1a PE=1 SV=2 - [HNF1A_MOUSE]	0.91	0.94	0.84	0.87	0.00
P22005	Proenkephalin-A OS=Mus musculus GN=Penk PE=2 SV=2 - [PENK_MOUSE]	-1.11	-1.10	-1.22	-1.17	0.00
Q64727	Vinculin OS=Mus musculus GN=Vcl PE=1 SV=4 - [VINC_MOUSE]	0.52	0.57	0.52	0.56	0.00
P23780	Beta-galactosidase OS=Mus musculus GN=Glb1 PE=2 SV=1 - [BGAL_MOUSE]	-0.52	-0.52	-0.48	-0.47	0.00
Q3TML0	Protein disulfide-isomerase A6 OS=Mus musculus GN=Pdia6 PE=2 SV=1 - [Q3TML0_MOUSE]	0.74	0.70	0.66	0.73	0.00
Q920E5	Farnesyl pyrophosphate synthase OS=Mus musculus GN=Fdps PE=2 SV=1 - [FPPS_MOUSE]	1.15	1.12	1.02	1.09	0.00
P02469	Laminin subunit beta-1 OS=Mus musculus GN=Lamb1 PE=1 SV=3 - [LAMB1_MOUSE]	-0.99	-0.91	-0.92	-0.88	0.00
P70168	Importin subunit beta-1 OS=Mus musculus GN=Kpnb1 PE=1 SV=2 - [IMB1_MOUSE]	0.38	0.37	0.41	0.39	0.00
Q9R0Q1	Synaptotagmin-like protein 4 OS=Mus musculus GN=Sytl4 PE=1 SV=1 - [SYTL4_MOUSE]	-0.64	-0.58	-0.63	-0.57	0.00
P47867-2	Isoform 2 of Secretogranin-3 OS=Mus musculus GN=Scg3 - [SCG3_MOUSE]	-1.51	-1.38	-1.36	-1.37	0.00
Q7TMY8-3	Isoform 3 of E3 ubiquitin-protein ligase HUWE1 OS=Mus musculus GN=Huwe1 - [HUWE1_MOUSE]	0.21	0.21	0.19	0.22	0.00

D3Z6P0-2	Isoform 2 of Protein disulfide-isomerase A2 OS=Mus musculus GN=Pdia2 - [PDIA2_MOUSE]	1.95	1.81	2.05	1.96	0.00
P08074	Carbonyl reductase [NADPH] 2 OS=Mus musculus GN=Cbr2 PE=1 SV=1 - [CBR2_MOUSE]	-0.58	-0.57	-0.62	-0.64	0.00
P21619	Lamin-B2 OS=Mus musculus GN=Lmnb2 PE=1 SV=2 - [LMNB2_MOUSE]	-0.51	-0.46	-0.50	-0.46	0.00
J3QK03	Uncharacterized protein OS=Mus musculus GN=Ighv6-5 PE=4 SV=1 - [J3QK03_MOUSE]	0.49	0.45	0.49	0.45	0.00
Q91WH2	MCG131371 OS=Mus musculus GN=Ugt2b38 PE=2 SV=1 - [Q91WH2_MOUSE]	-0.84	-0.89	-0.78	-0.84	0.00
Q69Z69	N-acetyltransferase ESCO1 OS=Mus musculus GN=Esco1 PE=2 SV=2 - [ESCO1_MOUSE]	1.00	1.06	1.05	1.13	0.00
Q04899	Cyclin-dependent kinase 18 OS=Mus musculus GN=Cdk18 PE=1 SV=1 - [CDK18_MOUSE]	-2.14	-2.15	-1.95	-1.96	0.00
Q91WD9	Secretagogin OS=Mus musculus GN=Scgn PE=2 SV=1 - [SEGN_MOUSE]	-1.05	-1.03	-1.05	-0.94	0.00
P01723	Ig lambda-1 chain V region OS=Mus musculus PE=1 SV=2 - [LV1A_MOUSE]	1.44	1.34	1.37	1.27	0.00
Q99JY8	Lipid phosphate phosphohydrolase 3 OS=Mus musculus GN=Ppap2b PE=1 SV=1 - [LPP3_MOUSE]	-0.82	-0.88	-0.88	-0.94	0.00
Q91W18-2	Isoform 2 of Tudor domain-containing protein 3 OS=Mus musculus GN=Tdrd3 - [TDRD3_MOUSE]	0.18	0.17	0.19	0.17	0.00
P04186	Complement factor B OS=Mus musculus GN=Cfb PE=1 SV=2 - [CFAB_MOUSE]	-0.42	-0.40	-0.39	-0.44	0.00
Q80YX1-2	Isoform 2 of Tenascin OS=Mus musculus GN=Tnc - [TENA_MOUSE]	0.49	0.48	0.53	0.47	0.00
Q02053	Ubiquitin-like modifier-activating enzyme 1 OS=Mus musculus GN=Uba1 PE=1 SV=1 - [UBA1_MOUSE]	0.30	0.29	0.28	0.26	0.00
P20918	Plasminogen OS=Mus musculus GN=Plg PE=1 SV=3 - [PLMN_MOUSE]	-0.70	-0.62	-0.68	-0.67	0.00
Q9CZX8	40S ribosomal protein S19 OS=Mus musculus GN=Rps19 PE=1 SV=3 - [RS19_MOUSE]	0.20	0.19	0.22	0.21	0.00

B9EJX2	Nance-Horan syndrome (Human) OS=Mus musculus GN=Nhs PE=2 SV=1 - [B9EJX2_MOUSE]	0.71	0.64	0.70	0.64	0.00
Q8CIB5	Fermitin family homolog 2 OS=Mus musculus GN=Fermt2 PE=1 SV=1 - [FERM2_MOUSE]	0.39	0.39	0.43	0.43	0.00
G3UZU7	T-cell differentiation antigen CD6 (Fragment) OS=Mus musculus GN=Cd6 PE=2 SV=1 - [G3UZU7_MOUSE]	0.31	0.31	0.34	0.33	0.00
F7CBP1	Eukaryotic translation initiation factor 4 gamma 2 OS=Mus musculus GN=Eif4g2 PE=4 SV=1 - [F7CBP1_MOUSE]	0.49	0.51	0.45	0.46	0.00
Q9D2R0	Acetoacetyl-CoA synthetase OS=Mus musculus GN=Aacs PE=1 SV=1 - [AACS_MOUSE]	0.48	0.42	0.45	0.45	0.00
P61255	60S ribosomal protein L26 OS=Mus musculus GN=Rpl26 PE=2 SV=1 [RL26_MOUSE]	0.39	0.40	0.37	0.35	0.00
D3Z041	Long-chain-fatty-acid--CoA ligase 1 OS=Mus musculus GN=Acsl1 PE=2 SV=1 - [D3Z041_MOUSE]	-0.87	-0.83	-0.92	-0.82	0.00
Q60994	Adiponectin OS=Mus musculus GN=Adipoq PE=1 SV=2 - [ADIPO_MOUSE]	-0.87	-0.86	-0.94	-0.82	0.00
Q921U8-2	Isoform L2 of Smoothelin OS=Mus musculus GN=Smtn - [SMTN_MOUSE]	0.39	0.42	0.38	0.38	0.00
Q8BG95	Protein phosphatase 1 regulatory subunit 12B OS=Mus musculus GN=Ppp1r12b PE=1 SV=2 - [MYPT2_MOUSE]	0.27	0.26	0.24	0.25	0.00
Q00623	Apolipoprotein A-I OS=Mus musculus GN=Apoa1 PE=1 SV=2 - [APOA1_MOUSE]	-0.58	-0.60	-0.64	-0.57	0.00
F8VQJ3	Laminin subunit gamma-1 OS=Mus musculus GN=Lamc1 PE=2 SV=1 - [F8VQJ3_MOUSE]	-0.92	-0.84	-0.83	-0.82	0.00
Q8C5W4-2	Isoform 2 of MORC family CW-type zinc finger protein 2B OS=Mus musculus GN=Morc2b - [MOR2B_MOUSE]	-0.76	-0.76	-0.83	-0.84	0.00
Q61233	Plastin-2 OS=Mus musculus GN=Lcp1 PE=1 SV=4 - [PLSL_MOUSE]	0.16	0.15	0.14	0.15	0.00
P00329	Alcohol dehydrogenase 1 OS=Mus musculus GN=Adh1 PE=2 SV=2 - [ADH1_MOUSE]	0.54	0.59	0.57	0.52	0.00

Q9DBX5	Cytosolic phospholipase A2 OS=Mus musculus GN=Pla2g4a PE=2 SV=1 - [Q9DBX5_MOUSE]	0.30	0.34	0.33	0.32	0.00
Q5U5V2	Hydroxylysine kinase OS=Mus musculus GN=Agphd1 PE=2 SV=2 - [AGPD1_MOUSE]	0.48	0.43	0.47	0.43	0.00
Q07076	Annexin A7 OS=Mus musculus GN=Anxa7 PE=2 SV=2 - [ANXA7_MOUSE]	0.23	0.24	0.22	0.21	0.00
Q68FD5	Clathrin heavy chain 1 OS=Mus musculus GN=Cltc PE=1 SV=3 - [CLH1_MOUSE]	0.27	0.31	0.31	0.30	0.00
Q9R0H0	Peroxisomal acyl-coenzyme A oxidase 1 OS=Mus musculus GN=Acox1 PE=1 SV=5 - [ACOX1_MOUSE]	-0.67	-0.66	-0.67	-0.75	0.00
P55088-2	Isoform 1 of Aquaporin-4 OS=Mus musculus GN=Aqp4 - [AQP4_MOUSE]	1.38	1.42	1.25	1.29	0.00
Q64285	Bile salt-activated lipase OS=Mus musculus GN=Cel PE=1 SV=1 - [CEL_MOUSE]	1.82	1.66	1.76	1.60	0.00
P28063	Proteasome subunit beta type-8 OS=Mus musculus GN=Psmb8 PE=1 SV=2 - [PSB8_MOUSE]	-0.27	-0.29	-0.29	-0.26	0.00
Q07417	Short-chain specific acyl-CoA dehydrogenase, mitochondrial OS=Mus musculus GN=Acads PE=2 SV=2 - [ACADS_MOUSE]	-0.76	-0.68	-0.71	-0.66	0.00
Q3V3N7	Protein Bbs1 OS=Mus musculus GN=Bbs1 PE=2 SV=1 - [Q3V3N7_MOUSE]	1.11	1.20	1.03	1.12	0.00
Q6IRU2	Tropomyosin alpha-4 chain OS=Mus musculus GN=Tpm4 PE=2 SV=3 - [TPM4_MOUSE]	-0.21	-0.22	-0.24	-0.23	0.00
Q9Z0P4-2	Isoform 2 of Paralemmin-1 OS=Mus musculus GN=Palm - [PALM_MOUSE]	-0.44	-0.42	-0.48	-0.42	0.00
Q3UF95	Large proline-rich protein BAG6 OS=Mus musculus GN=Bag6 PE=2 SV=1 - [Q3UF95_MOUSE]	0.25	0.24	0.28	0.26	0.00
F8VQI8	Sodium/hydrogen exchanger 10 OS=Mus musculus GN=Slc9c1 PE=2 SV=1 - [F8VQI8_MOUSE]	-1.40	-1.27	-1.38	-1.24	0.00
A6H5X4-2	Isoform 2 of PHD finger protein 11 OS=Mus musculus GN=Phf11 - [PHF11_MOUSE]	0.53	0.49	0.50	0.46	0.00

E9PYP1	Protein Ces1a OS=Mus musculus GN=Ces1a PE=4 SV=1 - [E9PYP1_MOUSE]	-1.04	-0.98	-1.13	-1.07	0.00
P09470	Angiotensin-converting enzyme OS=Mus musculus GN=Ace PE=1 SV=3 - [ACE_MOUSE]	-0.55	-0.53	-0.60	-0.59	0.00
Q61107	Guanylate-binding protein 4 OS=Mus musculus GN=Gbp4 PE=1 SV=1 - [GBP4_MOUSE]	0.50	0.53	0.46	0.51	0.00
P14131	40S ribosomal protein S16 OS=Mus musculus GN=Rps16 PE=2 SV=4 - [RS16_MOUSE]	0.32	0.28	0.31	0.30	0.00
Q9QZZ6	Dermatopontin OS=Mus musculus GN=Dpt PE=2 SV=1 - [DERM_MOUSE]	-1.15	-1.00	-1.11	-1.08	0.00
Q9QZD9	Eukaryotic translation initiation factor 3 subunit I OS=Mus musculus GN=Eif3i PE=1 SV=1 - [EIF3I_MOUSE]	-0.27	-0.29	-0.25	-0.26	0.00
D3Z7G3	Protein 2010107G23Rik (Fragment) OS=Mus musculus GN=2010107G23Rik PE=2 SV=1 - [D3Z7G3_MOUSE]	-0.20	-0.19	-0.18	-0.17	0.00
P21614	Vitamin D-binding protein OS=Mus musculus GN=Gc PE=1 SV=2 - [VTDB_MOUSE]	-0.38	-0.36	-0.41	-0.41	0.00
P17563	Selenium-binding protein 1 OS=Mus musculus GN=Selenbp1 PE=1 SV=2 - [SBP1_MOUSE]	-0.84	-0.78	-0.76	-0.72	0.00
Q8BGH2	Sorting and assembly machinery component 50 homolog OS=Mus musculus GN=Samm50 PE=1 SV=1 - [SAM50_MOUSE]	-0.28	-0.29	-0.26	-0.26	0.00
Q70IV5-2	Isoform 2 of Synemin OS=Mus musculus GN=Synm - [SYNEM_MOUSE]	0.67	0.77	0.72	0.73	0.00
P56480	ATP synthase subunit beta, mitochondrial OS=Mus musculus GN=Atp5b PE=1 SV=2 [ATPB_MOUSE]	-0.26	-0.23	-0.26	-0.27	0.00
G5E850	Cytochrome b-5, isoform CRA_a OS=Mus musculus GN=Cyb5 PE=3 SV=1 - [G5E850_MOUSE]	-0.34	-0.30	-0.34	-0.33	0.00
G3UXZ5	Proteasome activator complex subunit 1 (Fragment) OS=Mus musculus GN=Psme1 PE=2 SV=1 - [G3UXZ5_MOUSE]	0.34	0.36	0.31	0.36	0.00

I7HPY1	Novel protein OS=Mus musculus GN=Fam3a PE=4 SV=1 - [I7HPY1_MOUSE]	0.68	0.68	0.61	0.61	0.00
Q1RME4	CAR-like soluble protein OS=Mus musculus GN=Gm1123 PE=2 SV=1 - [Q1RME4_MOUSE]	-0.70	-0.62	-0.63	-0.62	0.00
Q61625	Glutamate receptor ionotropic, delta-2 OS=Mus musculus GN=Grid2 PE=1 SV=1 - [GRID2_MOUSE]	-1.09	-1.19	-1.03	-1.13	0.00
Q8VDM4	26S proteasome non-ATPase regulatory subunit 2 OS=Mus musculus GN=Psmd2 PE=1 SV=1 - [PSMD2_MOUSE]	0.37	0.35	0.32	0.37	0.00
Q8VDJ3	Vigilin OS=Mus musculus GN=Hdlbp PE=1 SV=1 - [VIGLN_MOUSE]	0.52	0.57	0.49	0.54	0.00
F8WJ93	Echinoderm microtubule-associated protein-like 4 OS=Mus musculus GN=Eml4 PE=2 SV=1 - [F8WJ93_MOUSE]	-0.23	-0.20	-0.20	-0.20	0.00
P47856-2	Isoform 2 of Glutamine-fructose-6-phosphate aminotransferase [isomerizing] 1 OS=Mus musculus GN=Gfpt1 - [GFPT1_MOUSE]	0.57	0.61	0.54	0.63	0.00
Q64092-3	Isoform 3 of Transcription factor E3 OS=Mus musculus GN=Tfe3 - [TFE3_MOUSE]	-0.65	-0.67	-0.58	-0.61	0.00
P09528	Ferritin heavy chain OS=Mus musculus GN=Fth1 PE=1 SV=2 - [FRIH_MOUSE]	-1.20	-1.25	-1.33	-1.38	0.00
Q6P1G0	HEAT repeat-containing protein 6 OS=Mus musculus GN=Heatr6 PE=2 SV=1 - [HEAT6_MOUSE]	0.77	0.85	0.75	0.84	0.00
Q61655	ATP-dependent RNA helicase DDX19A OS=Mus musculus GN=Ddx19a PE=2 SV=2 - [DD19A_MOUSE]	0.46	0.47	0.45	0.52	0.00
Q8VHC3	Selenoprotein M OS=Mus musculus GN=Selm PE=1 SV=3 - [SELM_MOUSE]	-1.04	-1.15	-1.01	-1.13	0.00
P24472	Glutathione S-transferase A4 OS=Mus musculus GN=Gsta4 PE=1 SV=3 - [GSTA4_MOUSE]	-0.71	-0.64	-0.74	-0.68	0.00
Q99PV0	Pre-mRNA-processing-splicing factor 8 OS=Mus musculus GN=Prpf8 PE=1 SV=2 - [PRP8_MOUSE]	0.21	0.19	0.21	0.21	0.00

Q9D6Z1	Nucleolar protein 56 OS=Mus musculus GN=Nop56 PE=1 SV=2 - [NOP56_MOUSE]	0.27	0.30	0.28	0.26	0.00
A2ACC7	Ral GTPase-activating protein subunit beta (Fragment) OS=Mus musculus GN=Ralgapb PE=2 SV=2 - [A2ACC7_MOUSE]	0.51	0.47	0.54	0.49	0.00
P26231	Catenin alpha-1 OS=Mus musculus GN=Ctnna1 PE=1 SV=1 - [CTNA1_MOUSE]	0.34	0.36	0.37	0.39	0.00
P24270	Catalase OS=Mus musculus GN=Cat PE=1 SV=4 - [CATA_MOUSE]	-0.53	-0.51	-0.59	-0.54	0.00
G3UW34	MCG130981 OS=Mus musculus GN=Rpl9-ps6 PE=4 SV=1 - [G3UW34_MOUSE]	0.15	0.16	0.14	0.16	0.00
Q9D7X3	Dual specificity protein phosphatase 3 OS=Mus musculus GN=Dusp3 PE=1 SV=1 - [DUS3_MOUSE]	0.62	0.60	0.53	0.57	0.00
Q6P9K8-2	Isoform 2 of Caskin-1 OS=Mus musculus GN=Caskin1 - [CSKI1_MOUSE]	-0.86	-0.93	-0.93	-1.01	0.00
O35639	Annexin A3 OS=Mus musculus GN=Anxa3 PE=1 SV=4 - [ANXA3_MOUSE]	0.51	0.44	0.48	0.45	0.00
P06801	NADP-dependent malic enzyme OS=Mus musculus GN=Me1 PE=1 SV=2 - [MAOX_MOUSE]	-0.31	-0.31	-0.31	-0.27	0.00
Q923D5	WW domain-binding protein 11 OS=Mus musculus GN=Wbp11 PE=1 SV=2 - [WBP11_MOUSE]	0.20	0.19	0.18	0.20	0.00
Q9CZ13	Cytochrome b-c1 complex subunit 1, mitochondrial OS=Mus musculus GN=Uqcrc1 PE=1 SV=2 - [QCR1_MOUSE]	-0.28	-0.27	-0.26	-0.24	0.00
Q8VCC2	Liver carboxylesterase 1 OS=Mus musculus GN=Ces1 PE=2 SV=1 - [EST1_MOUSE]	-0.91	-0.86	-1.00	-0.96	0.00
P30681	High mobility group protein B2 OS=Mus musculus GN=Hmgb2 PE=1 SV=3 - [HMGB2_MOUSE]	0.54	0.51	0.46	0.50	0.00
I7HLV2	60S ribosomal protein L10 (Fragment) OS=Mus musculus GN=Rpl10 PE=4 SV=1 [I7HLV2_MOUSE]	0.45	0.51	0.44	0.47	0.00
Q2TPA8	Hydroxysteroid dehydrogenase-like protein 2 OS=Mus musculus GN=Hsdl2 PE=2 SV=1 - [HSDL2_MOUSE]	-0.65	-0.71	-0.77	-0.73	0.00

Q8BYF6	Sodium-coupled monocarboxylate transporter 1 OS=Mus musculus GN=Slc5a8 PE=1 SV=1 - [SC5A8_MOUSE]	1.19	1.09	1.13	1.02	0.00
Q6PB66	Leucine-rich PPR motif-containing protein, mitochondrial OS=Mus musculus GN=Lrpprc PE=1 SV=2 - [LPPRC_MOUSE]	0.21	0.20	0.21	0.23	0.00
P07901	Heat shock protein HSP 90-alpha OS=Mus musculus GN=Hsp90aa1 PE=1 SV=4 - [HS90A_MOUSE]	0.58	0.63	0.54	0.61	0.00
Q8BTZ7	Mannose-1-phosphate guanyltransferase beta OS=Mus musculus GN=Gmppb PE=2 SV=1 - [GMPPB_MOUSE]	0.18	0.17	0.20	0.19	0.00
Q60772	Cyclin-dependent kinase 4 inhibitor C OS=Mus musculus GN=Cdkn2c PE=2 SV=1 - [CDN2C_MOUSE]	-0.41	-0.46	-0.43	-0.48	0.00
Q921W7	Putative uncharacterized protein Tes OS=Mus musculus GN=Tes PE=2 SV=2 - [Q921W7_MOUSE]	-0.39	-0.45	-0.45	-0.42	0.00
P14869	60S acidic ribosomal protein P0 OS=Mus musculus GN=Rplp0 PE=1 SV=3 - [RLA0_MOUSE]	0.56	0.62	0.54	0.55	0.00
Q08331	Calretinin OS=Mus musculus GN=Calb2 PE=1 SV=3 - [CALB2_MOUSE]	-1.01	-0.88	-1.00	-0.91	0.00
Q9Z1Z0	General vesicular transport factor p115 OS=Mus musculus GN=Uso1 PE=1 SV=2 - [USO1_MOUSE]	0.37	0.38	0.43	0.41	0.00
Q8R088	Golgi phosphoprotein 3-like OS=Mus musculus GN=Golph3l PE=2 SV=1 - [GLP3L_MOUSE]	0.47	0.50	0.43	0.49	0.00
Q922Q8	Leucine-rich repeat-containing protein 59 OS=Mus musculus GN=Lrrc59 PE=2 SV=1 - [LRC59_MOUSE]	0.63	0.64	0.56	0.58	0.00
Q9QYB5-2	Isoform 1 of Gamma-adducin OS=Mus musculus GN=Add3 - [ADDG_MOUSE]	-0.42	-0.36	-0.40	-0.41	0.00
Q8BMK4	Cytoskeleton-associated protein 4 OS=Mus musculus GN=Ckap4 PE=2 SV=2 - [CKAP4_MOUSE]	0.31	0.36	0.33	0.31	0.00

F6TYB7	Myelin basic protein (Fragment) OS=Mus musculus GN=Mbp PE=4 SV=1 - [F6TYB7_MOUSE]	-0.71	-0.75	-0.79	-0.83	0.00
D3YXN9	MCG59255 OS=Mus musculus GN=5830403L16Rik PE=4 SV=1 - [D3YXN9_MOUSE]	2.24	2.53	2.29	2.57	0.00
Q91V64	Isochorismatase domain-containing protein 1 OS=Mus musculus GN=Isoc1 PE=2 SV=1 - [ISOC1_MOUSE]	-0.23	-0.26	-0.23	-0.25	0.00
G5E898	Periplakin OS=Mus musculus GN=Ppl PE=4 SV=1 - [G5E898_MOUSE]	-0.41	-0.40	-0.41	-0.35	0.00
A2A9K3	Centrosomal protein of 85 kDa OS=Mus musculus GN=Cep85 PE=2 SV=1 - [A2A9K3_MOUSE]	-0.74	-0.66	-0.76	-0.68	0.00
Q9DCB8	Iron-sulfur cluster assembly 2 homolog, mitochondrial OS=Mus musculus GN=Isca2 PE=2 SV=2 - [ISCA2_MOUSE]	-0.59	-0.69	-0.60	-0.64	0.00
P37040	NADPH--cytochrome P450 reductase OS=Mus musculus GN=Por PE=1 SV=2 - [NCPR_MOUSE]	-0.60	-0.62	-0.53	-0.61	0.00
Q9CY27	Very-long-chain enoyl-CoA reductase OS=Mus musculus GN=Tecr PE=1 SV=1 - [TECR_MOUSE]	0.50	0.49	0.44	0.44	0.00
Q9D7V1	SH2 domain-containing protein 4A OS=Mus musculus GN=Sh2d4a PE=1 SV=1 - [SH24A_MOUSE]	0.57	0.64	0.61	0.67	0.00
Q60605-2	Isoform Smooth muscle of Myosin light polypeptide 6 OS=Mus musculus GN=Myl6 - [MYL6_MOUSE]	-0.38	-0.36	-0.40	-0.42	0.00
Q03517	Secretogranin-2 OS=Mus musculus GN=Scg2 PE=1 SV=1 - [SCG2_MOUSE]	-0.82	-0.74	-0.81	-0.71	0.00
P08551	Neurofilament light polypeptide OS=Mus musculus GN=Nefl PE=1 SV=5 - [NFL_MOUSE]	-0.91	-0.88	-1.02	-0.99	0.00
D3Z6F0	Protein Gm14446 OS=Mus musculus GN=Gm14446 PE=4 SV=1 - [D3Z6F0_MOUSE]	0.67	0.70	0.60	0.63	0.00
Q8CIE6	Coatomer subunit alpha OS=Mus musculus GN=Copa PE=1 SV=2 - [COPA_MOUSE]	0.52	0.54	0.47	0.55	0.00

Q9CR00	26S proteasome non-ATPase regulatory subunit 9 OS=Mus musculus GN=Psmd9 PE=1 SV=1 - [PSMD9_MOUSE]	-0.28	-0.29	-0.25	-0.30	0.00
Q08879	Fibulin-1 OS=Mus musculus GN=Fbln1 PE=1 SV=2 - [FBLN1_MOUSE]	0.53	0.55	0.47	0.55	0.00
Q91Y97	Fructose-bisphosphate aldolase B OS=Mus musculus GN=Aldob PE=1 SV=3 - [ALDOB_MOUSE]	-0.30	-0.28	-0.33	-0.31	0.00
Q9CQC2	Colipase OS=Mus musculus GN=Clps PE=2 SV=1 - [COL_MOUSE]	1.24	1.13	1.17	1.05	0.00
P51661	Corticosteroid 11-beta-dehydrogenase isozyme 2 OS=Mus musculus GN=Hsd11b2 PE=2 SV=2 - [DHI2_MOUSE]	0.43	0.41	0.46	0.39	0.00
Q3UQ44	Ras GTPase-activating-like protein IQGAP2 OS=Mus musculus GN=Iqgap2 PE=1 SV=2 - [IQGA2_MOUSE]	0.29	0.33	0.30	0.33	0.00
Q9D7Z6	Calcium-activated chloride channel regulator 1 OS=Mus musculus GN=Clca1 PE=1 SV=2 - [CLCA1_MOUSE]	1.94	1.73	1.87	1.65	0.00
Q9Z1T2	Thrombospondin-4 OS=Mus musculus GN=Thbs4 PE=1 SV=1 - [TSP4_MOUSE]	1.24	1.33	1.31	1.13	0.00
L7N451	Interferon-induced very large GTPase 1 OS=Mus musculus GN=Gvin1 PE=4 SV=1 [L7N451_MOUSE]	-0.47	-0.54	-0.52	-0.47	0.00
Q8CGC7	Bifunctional glutamate/proline--tRNA ligase OS=Mus musculus GN=Eprs PE=1 SV=4 - [SYEP_MOUSE]	0.43	0.44	0.37	0.41	0.00
P01843	Ig lambda-1 chain C region OS=Mus musculus PE=1 SV=1 - [LAC1_MOUSE]	1.90	2.12	1.86	2.13	0.00
Q9R0E2	Procollagen-lysine,2-oxoglutarate 5-dioxygenase 1 OS=Mus musculus GN=Plod1 PE=1 SV=1 [PLOD1_MOUSE]	-0.42	-0.46	-0.47	-0.51	0.00
P20152	Vimentin OS=Mus musculus GN=Vim PE=1 SV=3 - [VIME_MOUSE]	-0.58	-0.52	-0.61	-0.54	0.00
A2AFS3	UPF0577 protein KIAA1324 OS=Mus musculus GN=Kiaa1324 PE=2 SV=1 - [K1324_MOUSE]	0.64	0.67	0.56	0.61	0.00

B1AXZ0	ELAV-like protein 2 OS=Mus musculus GN=Elavl2 PE=2 SV=1 - [B1AXZ0_MOUSE]	-0.45	-0.51	-0.52	-0.52	0.00
Q3TCH7	Cullin-4A OS=Mus musculus GN=Cul4a PE=1 SV=1 - [CUL4A_MOUSE]	0.59	0.59	0.50	0.55	0.00
Q3TPM3	Cullin-1 (Fragment) OS=Mus musculus GN=Cul1 PE=2 SV=1 - [Q3TPM3_MOUSE]	0.33	0.31	0.29	0.28	0.00
Q8CIM7	Cytochrome P450 2D26 OS=Mus musculus GN=Cyp2d26 PE=1 SV=1 - [CP2DQ_MOUSE]	-0.49	-0.57	-0.57	-0.51	0.00
F8VQ43	Laminin subunit alpha-2 OS=Mus musculus GN=Lama2 PE=2 SV=1 - [F8VQ43_MOUSE]	-0.79	-0.89	-0.94	-0.89	0.00
P54869	Hydroxymethylglutaryl-CoA synthase, mitochondrial OS=Mus musculus GN=Hmgcs2 PE=1 SV=2 - [HMCS2_MOUSE]	-1.25	-1.08	-1.27	-1.14	0.00
Q8R1G2	Carboxymethylenebutenolidase homolog OS=Mus musculus GN=Cmbl PE=2 SV=1 - [CMBL_MOUSE]	-0.44	-0.49	-0.48	-0.53	0.00
P23927	Alpha-crystallin B chain OS=Mus musculus GN=Cryab PE=1 SV=2 - [CRYAB_MOUSE]	0.45	0.39	0.46	0.47	0.00
D3YY41	Phosphatidylinositol 3-kinase, catalytic, alpha polypeptide, isoform CRA_a OS=Mus musculus GN=Pik3ca PE=4 SV=1 - [D3YY41_MOUSE]	0.58	0.66	0.57	0.64	0.00
Q8R2H9	Phosphoethanolamine/phosphocholine phosphatase OS=Mus musculus GN=Phospho1 PE=2 SV=1 - [PHOP1_MOUSE]	-0.64	-0.70	-0.58	-0.64	0.00
Q8R2S8	CD177 antigen OS=Mus musculus GN=Cd177 PE=2 SV=1 - [CD177_MOUSE]	1.30	1.11	1.25	1.31	0.00
D3YX13	Inhibitor of growth protein 3 (Fragment) OS=Mus musculus GN=Ing3 PE=2 SV=1 - [D3YX13_MOUSE]	0.31	0.27	0.32	0.28	0.00
D3YWJ5	Tumor necrosis factor alpha-induced protein 8 OS=Mus musculus GN=Tnfaip8 PE=2 SV=1 - [D3YWJ5_MOUSE]	0.60	0.52	0.62	0.59	0.00
Q61704	Inter-alpha-trypsin inhibitor heavy chain H3 OS=Mus musculus GN=Itih3 PE=1 SV=3 - [ITIH3_MOUSE]	-0.60	-0.57	-0.66	-0.67	0.00

D3YU12	NmrA-like family domain-containing protein 1 OS=Mus musculus GN=Nmral1 PE=2 SV=1 - [D3YU12_MOUSE]	0.39	0.44	0.37	0.39	0.00
P01675	Ig kappa chain V-VI region XRPC 44 OS=Mus musculus PE=1 SV=1 - [KV6A1_MOUSE]	0.55	0.56	0.60	0.65	0.00
E9PXU9	Dysferlin OS=Mus musculus GN=Dysf PE=2 SV=1 - [E9PXU9_MOUSE]	0.27	0.25	0.23	0.26	0.00
P47738	Aldehyde dehydrogenase, mitochondrial OS=Mus musculus GN=Aldh2 PE=1 SV=1 - [ALDH2_MOUSE]	-0.26	-0.23	-0.28	-0.27	0.00
Q8K3G9	DCC-interacting protein 13-beta OS=Mus musculus GN=Appl2 PE=2 SV=1 - [DP13B_MOUSE]	0.67	0.59	0.62	0.56	0.00
Q68FG2	Protein Sptbn2 OS=Mus musculus GN=Sptbn2 PE=2 SV=1 - [Q68FG2_MOUSE]	-2.45	-2.28	-2.20	-2.04	0.00
P37804	Transgelin OS=Mus musculus GN=Tagln PE=1 SV=3 - [TAGL_MOUSE]	0.59	0.66	0.55	0.62	0.00
Q8VCT4	Carboxylesterase 1D OS=Mus musculus GN=Ces1d PE=1 SV=1 - [CES1D_MOUSE]	-1.42	-1.22	-1.45	-1.32	0.00
O89050	Muskelin OS=Mus musculus GN=Mkln1 PE=2 SV=1 - [MKLN1_MOUSE]	0.22	0.25	0.26	0.24	0.00
Q3UMT1	Protein phosphatase 1 regulatory subunit 12C OS=Mus musculus GN=Ppp1r12c PE=1 SV=1 - [PP12C_MOUSE]	0.24	0.28	0.28	0.28	0.00
Q6P8U6	Pancreatic triacylglycerol lipase OS=Mus musculus GN=Pnlip PE=1 SV=1 - [LIPP_MOUSE]	2.43	2.72	2.52	2.88	0.00
Q8CI94	Glycogen phosphorylase, brain form OS=Mus musculus GN=Pygb PE=1 SV=3 - [PYGB_MOUSE]	0.37	0.34	0.37	0.41	0.00
O08691	Arginase-2, mitochondrial OS=Mus musculus GN=Arg2 PE=1 SV=1 - [ARGI2_MOUSE]	-1.00	-0.89	-0.94	-0.83	0.00
Q64737	Trifunctional purine biosynthetic protein adenosine-3 OS=Mus musculus GN=Gart PE=2 SV=3 - [PUR2_MOUSE]	0.32	0.36	0.30	0.31	0.00

P24549	Retinal dehydrogenase 1 OS=Mus musculus GN=Aldh1a1 PE=1 SV=5 - [AL1A1_MOUSE]	-0.79	-0.73	-0.88	-0.80	0.00
P29758	Ornithine aminotransferase, mitochondrial OS=Mus musculus GN=Oat PE=1 SV=1 - [OAT_MOUSE]	-0.74	-0.66	-0.80	-0.73	0.00
O70421	Frizzled-1 OS=Mus musculus GN=Fzd1 PE=2 SV=3 - [FZD1_MOUSE]	0.24	0.26	0.21	0.23	0.00
Q61792	LIM and SH3 domain protein 1 OS=Mus musculus GN=Lasp1 PE=1 SV=1 - [LASP1_MOUSE]	-0.32	-0.30	-0.29	-0.27	0.00
Q8R164	Valacyclovir hydrolase OS=Mus musculus GN=Bphl PE=2 SV=1 - [BPHL_MOUSE]	-0.47	-0.49	-0.54	-0.55	0.00
P10493	Nidogen-1 OS=Mus musculus GN=Nid1 PE=1 SV=2 - [NID1_MOUSE]	-0.67	-0.74	-0.61	-0.68	0.00
Q76MZ3	Serine/threonine-protein phosphatase 2A 65 kDa regulatory subunit A alpha isoform OS=Mus musculus GN=Ppp2r1a PE=1 SV=3 - [2AAA_MOUSE]	0.45	0.50	0.42	0.45	0.00
O88310	Intelectin-1a OS=Mus musculus GN=Itln1 PE=1 SV=1 - [ITL1A_MOUSE]	2.35	2.67	2.27	2.59	0.00
Q9EQ20	Methylmalonate-semialdehyde dehydrogenase [acylating], mitochondrial OS=Mus musculus GN=Aldh6a1 PE=1 SV=1 - [MMSA_MOUSE]	-0.51	-0.51	-0.48	-0.58	0.00
G5E825	DNA segment, Chr 10, ERATO Doi 610, expressed, isoform CRA_b OS=Mus musculus GN=Arhgef25 PE=4 SV=1 - [G5E825_MOUSE]	0.30	0.31	0.35	0.35	0.00
Q6PDM2	Serine/arginine-rich splicing factor 1 OS=Mus musculus GN=Srsf1 PE=1 SV=3 - [SRSF1_MOUSE]	-0.31	-0.35	-0.34	-0.37	0.00
Q8K363	ATP-dependent RNA helicase DDX18 OS=Mus musculus GN=Ddx18 PE=2 SV=1 - [DDX18_MOUSE]	0.37	0.31	0.36	0.37	0.00
Q99KC8	von Willebrand factor A domain-containing protein 5A OS=Mus musculus GN=Vwa5a PE=1 SV=2 - [VMA5A_MOUSE]	-0.10	-0.10	-0.11	-0.09	0.00

O09105	Neurogenic differentiation factor 4 OS=Mus musculus GN=Neurod4 PE=1 SV=1 - [NDF4_MOUSE]	0.45	0.46	0.39	0.45	0.00
P05202	Aspartate aminotransferase, mitochondrial OS=Mus musculus GN=Got2 PE=1 SV=1 - [AATM_MOUSE]	-0.36	-0.31	-0.36	-0.32	0.00
Q9D6N1	Carbonic anhydrase 13 OS=Mus musculus GN=Ca13 PE=1 SV=1 - [CAH13_MOUSE]	-0.42	-0.41	-0.35	-0.38	0.00
Q810Q5	Normal mucosa of esophagus-specific gene 1 protein OS=Mus musculus GN=Nmes1 PE=2 SV=1 - [NMES1_MOUSE]	0.18	0.22	0.19	0.21	0.00
P97816	Protein S100-G OS=Mus musculus GN=S100g PE=2 SV=3 - [S100G_MOUSE]	-2.67	-2.41	-2.88	-2.49	0.00
Q01149	Collagen alpha-2(I) chain OS=Mus musculus GN=Col1a2 PE=2 SV=2 - [CO1A2_MOUSE]	-0.33	-0.33	-0.38	-0.37	0.00
Q60597	2-oxoglutarate dehydrogenase, mitochondrial OS=Mus musculus GN=Ogdh PE=1 SV=3 - [ODO1_MOUSE]	-0.25	-0.21	-0.26	-0.24	0.00
Q8BWR2	PITH domain-containing protein 1 OS=Mus musculus GN=Pithd1 PE=2 SV=1 - [PITH1_MOUSE]	-0.50	-0.43	-0.48	-0.43	0.00
P31938	Dual specificity mitogen activated protein kinase kinase 1 OS=Mus musculus GN=Map2k1 PE=1 SV=2 - [MP2K1_MOUSE]	0.43	0.38	0.38	0.36	0.00
P48036	Annexin A5 OS=Mus musculus GN=Anxa5 PE=1 SV=1 - [ANXA5_MOUSE]	-0.35	-0.34	-0.41	-0.36	0.00
Q9D2G2	Dihydrolipoyllysine-residue succinyltransferase component of 2-oxoglutarate dehydrogenase complex, mitochondrial OS=Mus musculus GN=Dlst PE=1 SV=1 - [ODO2_MOUSE]	-0.39	-0.35	-0.38	-0.33	0.00
Q80T14	Extracellular matrix protein FRAS1 OS=Mus musculus GN=Fras1 PE=2 SV=2 [FRAS1_MOUSE]	-0.90	-0.83	-1.00	-0.94	0.00

P03995-2	Isoform 2 of Glial fibrillary acidic protein OS=Mus musculus GN=Gfap - [GFAP_MOUSE]	-0.59	-0.56	-0.48	-0.55	0.00
F6RPJ9	Insulin-degrading enzyme (Fragment) OS=Mus musculus GN=Ide PE=3 SV=1 - [F6RPJ9_MOUSE]	-0.55	-0.50	-0.50	-0.45	0.00
P51174	Long-chain specific acyl-CoA dehydrogenase, mitochondrial OS=Mus musculus GN=Acadl PE=2 SV=2 - [ACADL_MOUSE]	-0.58	-0.65	-0.61	-0.70	0.00
Q8K157	Aldose 1-epimerase OS=Mus musculus GN=Galm PE=2 SV=1 - [GALM_MOUSE]	-0.47	-0.54	-0.54	-0.47	0.00
Q7TN29	Stromal membrane-associated protein 2 OS=Mus musculus GN=Smap2 PE=1 SV=1 - [SMAP2_MOUSE]	-0.65	-0.63	-0.74	-0.73	0.00
A2ASQ1-3	Isoform 3 of Agrin OS=Mus musculus GN=Agrn - [AGRIN_MOUSE]	-0.98	-0.93	-0.99	-0.82	0.00
P50285	Dimethylaniline monooxygenase [N-oxide-forming] 1 OS=Mus musculus GN=Fmo1 PE=1 SV=1 [FMO1_MOUSE]	-1.49	-1.37	-1.33	-1.22	0.00
O70423	Membrane primary amine oxidase OS=Mus musculus GN=Aoc3 PE=2 SV=3 - [AOC3_MOUSE]	-0.78	-0.69	-0.77	-0.66	0.00
Q8BMJ2	Leucine--tRNA ligase, cytoplasmic OS=Mus musculus GN=Lars PE=2 SV=2 - [SYLC_MOUSE]	0.42	0.45	0.37	0.42	0.00
P00920	Carbonic anhydrase 2 OS=Mus musculus GN=Ca2 PE=1 SV=4 - [CAH2_MOUSE]	-0.24	-0.21	-0.26	-0.23	0.00
Q64133	Amine oxidase [flavin-containing] A OS=Mus musculus GN=Maoa PE=1 SV=3 - [AOFA_MOUSE]	-0.18	-0.16	-0.19	-0.19	0.00
Q8BGT9	Polypeptide N-acetylgalactosaminyltransferase 12 OS=Mus musculus GN=Galnt12 PE=2 SV=1 - [GLT12_MOUSE]	0.31	0.36	0.34	0.38	0.00
Q8C8R3-2	Isoform 2 of Ankyrin-2 OS=Mus musculus GN=Ank2 - [ANK2_MOUSE]	-0.42	-0.38	-0.36	-0.42	0.00
Q99LT0	Protein dpy-30 homolog OS=Mus musculus GN=Dpy30 PE=1 SV=1 - [DPY30_MOUSE]	-0.19	-0.17	-0.20	-0.18	0.00

Q8BXA5	Cleft lip and palate transmembrane protein 1-like protein OS=Mus musculus GN=Clptm1l PE=2 SV=1 - [CLP1L_MOUSE]	0.15	0.17	0.16	0.18	0.00
Q9R111	Guanine deaminase OS=Mus musculus GN=Gda PE=1 SV=1 - [GUAD_MOUSE]	-0.41	-0.46	-0.39	-0.46	0.00
Q3TJI8	Corticosteroid 11-beta-dehydrogenase isozyme 1 OS=Mus musculus GN=Hsd11b1 PE=2 SV=1 - [Q3TJI8_MOUSE]	-1.21	-1.06	-1.29	-1.25	0.00
Q9WUU7	Cathepsin Z OS=Mus musculus GN=Ctsz PE=2 SV=1 - [CATZ_MOUSE]	-0.23	-0.22	-0.26	-0.22	0.00
Q91Y74	CMP-N-acetylneuraminate-beta galactosamide-alpha-2,3-sialyltransferase 4 OS=Mus musculus GN=St3gal4 PE=2 SV=1 - [SIA4C_MOUSE]	1.07	1.00	1.21	1.14	0.00
Q8CHR6	Dihydropyrimidine dehydrogenase [NADP(+)] OS=Mus musculus GN=Dpyd PE=2 SV=1 - [DPYD_MOUSE]	-0.88	-0.88	-0.96	-1.04	0.00
D3YTY9	Kininogen-1 OS=Mus musculus GN=Kng1 PE=2 SV=1 - [D3YTY9_MOUSE]	-0.43	-0.38	-0.40	-0.35	0.00
B7FAU9	Filamin, alpha OS=Mus musculus GN=Flna PE=4 SV=1 - [B7FAU9_MOUSE]	0.40	0.44	0.36	0.40	0.00
Q8R1A8	Ornithine carbamoyltransferase, mitochondrial OS=Mus musculus GN=Otc PE=2 SV=1 - [Q8R1A8_MOUSE]	-0.71	-0.64	-0.63	-0.58	0.00
P47963	60S ribosomal protein L13 OS=Mus musculus GN=Rpl13 PE=2 SV=3 [RL13_MOUSE]	-0.80	-0.78	-0.70	-0.67	0.00
Q9D8C9	Purine nucleoside phosphorylase OS=Mus musculus GN=Pnp2 PE=2 SV=1 - [Q9D8C9_MOUSE]	-0.25	-0.27	-0.24	-0.29	0.00
P52196	Thiosulfate sulfurtransferase OS=Mus musculus GN=Tst PE=1 SV=3 - [THTR_MOUSE]	-0.35	-0.31	-0.38	-0.38	0.00
Q148B1	Cytochrome P450, family 2, subfamily c, polypeptide 65 OS=Mus musculus GN=Cyp2c65 PE=2 SV=1 - [Q148B1_MOUSE]	-0.73	-0.63	-0.76	-0.68	0.00

O35679	Serine protease inhibitor Kazal-type 4 OS=Mus musculus GN=Spink4 PE=2 SV=2 - [ISK4_MOUSE]	0.85	0.96	0.79	0.84	0.00
D3Z1D3	Protein 3425401B19Rik OS=Mus musculus GN=3425401B19Rik PE=4 SV=1 - [D3Z1D3_MOUSE]	-0.38	-0.44	-0.41	-0.47	0.00
Q05421	Cytochrome P450 2E1 OS=Mus musculus GN=Cyp2e1 PE=1 SV=1 - [CP2E1_MOUSE]	-1.11	-1.18	-0.97	-1.04	0.00
Q80UW8	DNA-directed RNA polymerases I, II, and III subunit RPABC1 OS=Mus musculus GN=Polr2e PE=2 SV=1 - [RPAB1_MOUSE]	-0.13	-0.14	-0.11	-0.12	0.00
Q9ERD7	Tubulin beta-3 chain OS=Mus musculus GN=Tubb3 PE=1 SV=1 - [TBB3_MOUSE]	0.21	0.23	0.25	0.20	0.00
P46471	26S protease regulatory subunit 7 OS=Mus musculus GN=Psmc2 PE=1 SV=5 - [PRS7_MOUSE]	0.39	0.32	0.37	0.39	0.00
Q9CWS0	N(G),N(G)-dimethylarginine dimethylaminohydrolase 1 OS=Mus musculus GN=Ddah1 PE=1 SV=3 - [DDAH1_MOUSE]	-0.38	-0.42	-0.44	-0.36	0.00
P15947	Kallikrein-1 OS=Mus musculus GN=Klk1 PE=1 SV=3 - [KLK1_MOUSE]	-0.40	-0.41	-0.48	-0.46	0.00
Q8K419	Galectin-4 OS=Mus musculus GN=Lgals4 PE=1 SV=2 - [LEG4_MOUSE]	-0.41	-0.41	-0.35	-0.38	0.00
Q9D8V7	Signal peptidase complex catalytic subunit SEC11C OS=Mus musculus GN=Sec11c PE=2 SV=3 - [SC11C_MOUSE]	0.82	0.71	0.83	0.72	0.00
Q62388	Serine-protein kinase ATM OS=Mus musculus GN=Atm PE=1 SV=2 - [ATM_MOUSE]	-1.51	-1.48	-1.75	-1.72	0.00
F7BP55	Adenylate kinase 2, mitochondrial (Fragment) OS=Mus musculus GN=Ak2 PE=2 SV=1 - [F7BP55_MOUSE]	0.14	0.15	0.17	0.18	0.00
Q8BKL6	Gamma-tubulin complex component 4 OS=Mus musculus GN=Tubgcp4 PE=2 SV=1 - [Q8BKL6_MOUSE]	-0.41	-0.46	-0.38	-0.44	0.00

D3Z1X8	SWI/SNF complex subunit SMARCC1 OS=Mus musculus GN=Smarcc1 PE=2 SV=1 - [D3Z1X8_MOUSE]	0.40	0.35	0.43	0.38	0.00
Q8BWF0	Succinate-semialdehyde dehydrogenase, mitochondrial OS=Mus musculus GN=Aldh5a1 PE=1 SV=1 - [SSDH_MOUSE]	-0.55	-0.53	-0.59	-0.48	0.00
Q920A7	AFG3-like protein 1 OS=Mus musculus GN=Afg3l1 PE=2 SV=2 - [AFG31_MOUSE]	-1.23	-1.09	-1.13	-1.00	0.00
Q8BH39	MCG1032870 OS=Mus musculus GN=4930579K19Rik PE=4 SV=1 - [Q8BH39_MOUSE]	-0.22	-0.25	-0.24	-0.27	0.00
F8WJ99	Microfibrillar-associated protein 5 OS=Mus musculus GN=Mfap5 PE=2 SV=2 - [F8WJ99_MOUSE]	-0.88	-0.81	-0.79	-0.72	0.00
Q6URW6	Myosin-14 OS=Mus musculus GN=Myh14 PE=1 SV=1 - [MYH14_MOUSE]	-0.27	-0.25	-0.26	-0.22	0.00
Q9R1P3	Proteasome subunit beta type-2 OS=Mus musculus GN=Psmb2 PE=1 SV=1 - [PSB2_MOUSE]	-0.17	-0.15	-0.13	-0.15	0.00
G3X9Y6	Aldo-keto reductase family 1, member C19 OS=Mus musculus GN=Akr1c19 PE=4 SV=1 - [G3X9Y6_MOUSE]	-1.01	-0.90	-0.88	-0.82	0.00
Q9JM62-2	Isoform 2 of Receptor expression-enhancing protein 6 OS=Mus musculus GN=Reep6 - [REEP6_MOUSE]	-0.42	-0.36	-0.42	-0.37	0.00
G3X956	FACT complex subunit SPT16 OS=Mus musculus GN=Supt16 PE=4 SV=1 - [G3X956_MOUSE]	0.30	0.28	0.24	0.28	0.00
Q9CRA5	Golgi phosphoprotein 3 OS=Mus musculus GN=Golph3 PE=2 SV=1 - [GOLP3_MOUSE]	0.61	0.74	0.64	0.70	0.00
Q8BW75	Amine oxidase [flavin-containing] B OS=Mus musculus GN=Maob PE=1 SV=4 - [AOFB_MOUSE]	-0.89	-0.76	-0.84	-0.74	0.00
Q80WJ1	Gametogenetin OS=Mus musculus GN=Ggn PE=1 SV=2 - [GGN_MOUSE]	-0.26	-0.27	-0.30	-0.31	0.00
Q8K010	5-oxoprolinase OS=Mus musculus GN=Oplah PE=2 SV=1 - [OPLA_MOUSE]	-0.34	-0.33	-0.28	-0.30	0.00

Q7M6Y6	Maestro heat-like repeat-containing protein family member 2B OS=Mus musculus GN=Mroh2b PE=1 SV=2 - [MRO2B_MOUSE]	0.70	0.63	0.64	0.57	0.00
P61082	NEDD8-conjugating enzyme Ubc12 OS=Mus musculus GN=Ube2m PE=2 SV=1 - [UBC12_MOUSE]	0.28	0.30	0.24	0.28	0.00
Q5BKQ4	Inactive pancreatic lipase-related protein 1 OS=Mus musculus GN=Pnliprp1 PE=2 SV=2 - [LIPR1_MOUSE]	2.35	2.01	2.22	1.94	0.00
P47199	Quinone oxidoreductase OS=Mus musculus GN=Cryz PE=2 SV=1 - [QOR_MOUSE]	-0.35	-0.42	-0.36	-0.36	0.00
Q61001	Laminin subunit alpha-5 OS=Mus musculus GN=Lama5 PE=1 SV=4 - [LAMA5_MOUSE]	-0.58	-0.60	-0.49	-0.53	0.00
Q6ZQH8	Nucleoporin NUP188 homolog OS=Mus musculus GN=Nup188 PE=1 SV=2 - [NU188_MOUSE]	0.55	0.62	0.57	0.67	0.00
P62852	40S ribosomal protein S25 OS=Mus musculus GN=Rps25 PE=2 SV=1 - [RS25_MOUSE]	0.50	0.57	0.46	0.50	0.00
Q99LM2	CDK5 regulatory subunit-associated protein 3 OS=Mus musculus GN=Cdk5rap3 PE=2 SV=1 - [CK5P3_MOUSE]	0.58	0.49	0.60	0.57	0.00
Q921L3	Transmembrane and coiled-coil domain-containing protein 1 OS=Mus musculus GN=Tmco1 PE=2 SV=1 - [TMCO1_MOUSE]	0.59	0.56	0.48	0.51	0.00
O54916-4	Isoform 4 of RalBP1-associated Eps domain-containing protein 1 OS=Mus musculus GN=Reps1 - [REPS1_MOUSE]	-0.54	-0.48	-0.47	-0.44	0.00
Q8VCN5	Cystathionine gamma-lyase OS=Mus musculus GN=Cth PE=1 SV=1 - [CGL_MOUSE]	-0.78	-0.76	-0.64	-0.68	0.00
Q6P6M5	Peroxisomal membrane protein 11C OS=Mus musculus GN=Pex11g PE=2 SV=2 - [PX11C_MOUSE]	-0.62	-0.72	-0.60	-0.70	0.00
P28654	Decorin OS=Mus musculus GN=Dcn PE=2 SV=1 - [PGS2_MOUSE]	-0.74	-0.65	-0.77	-0.64	0.00

Q61133	Glutathione S-transferase theta-2 OS=Mus musculus GN=Gstt2 PE=2 SV=4 - [GSTT2_MOUSE]	0.90	0.80	0.81	0.73	0.00
Q3UPH1	Protein PRRC1 OS=Mus musculus GN=Prrc1 PE=2 SV=1 - [PRRC1_MOUSE]	0.56	0.46	0.49	0.48	0.00
P14094	Sodium/potassium-transporting ATPase subunit beta-1 OS=Mus musculus GN=Atp1b1 PE=1 SV=1 - [AT1B1_MOUSE]	0.38	0.38	0.31	0.36	0.00
O08532-3	Isoform 2C of Voltage-dependent calcium channel subunit alpha-2/delta-1 OS=Mus musculus GN=Cacna2d1 - [CA2D1_MOUSE]	-0.70	-0.60	-0.73	-0.62	0.00
Q62351	Transferrin receptor protein 1 OS=Mus musculus GN=Tfrc PE=1 SV=1 - [TFR1_MOUSE]	0.92	0.74	0.85	0.84	0.00
Q3TMQ6	Angiogenin-4 OS=Mus musculus GN=Ang4 PE=1 SV=1 - [ANG4_MOUSE]	2.73	2.34	2.72	2.33	0.00
P28474	Alcohol dehydrogenase class-3 OS=Mus musculus GN=Adh5 PE=1 SV=3 - [ADHX_MOUSE]	-0.39	-0.39	-0.47	-0.43	0.00
P62748	Hippocalcin-like protein 1 OS=Mus musculus GN=Hpcal1 PE=2 SV=2 - [HPCL1_MOUSE]	-0.30	-0.32	-0.25	-0.30	0.00
P14733	Lamin-B1 OS=Mus musculus GN=Lmnb1 PE=1 SV=3 - [LMNB1_MOUSE]	-0.47	-0.39	-0.48	-0.46	0.00
O88322	Nidogen-2 OS=Mus musculus GN=Nid2 PE=1 SV=2 - [NID2_MOUSE]	-0.92	-0.79	-0.82	-0.75	0.00
D3YU58	Anaphase-promoting complex subunit 7 OS=Mus musculus GN=Anapc7 PE=2 SV=1 - [D3YU58_MOUSE]	0.47	0.55	0.51	0.58	0.00
Q6YI28	EP1 OS=Mus musculus GN=2210407C18Rik PE=2 SV=1 - [Q6YI28_MOUSE]	-2.46	-2.38	-2.12	-2.04	0.00
Q9ER71-2	Isoform 2 of Rho-related GTP-binding protein RhoJ OS=Mus musculus GN=Rhoj - [RHOJ_MOUSE]	0.82	0.90	0.72	0.81	0.00
O08528	Hexokinase-2 OS=Mus musculus GN=Hk2 PE=1 SV=1 - [HXK2_MOUSE]	0.57	0.64	0.52	0.59	0.00
P06797	Cathepsin L1 OS=Mus musculus GN=Ctsl1 PE=1 SV=2 - [CATL1_MOUSE]	-0.47	-0.41	-0.52	-0.47	0.00

P07724	Serum albumin OS=Mus musculus GN=Alb PE=1 SV=3 - [ALBU_MOUSE]	-0.66	-0.57	-0.71	-0.64	0.00
Q9CY64	Biliverdin reductase A OS=Mus musculus GN=Blvra PE=2 SV=1 - [BIEA_MOUSE]	0.35	0.28	0.33	0.34	0.00
Q91ZA3	Propionyl-CoA carboxylase alpha chain, mitochondrial OS=Mus musculus GN=Pcca PE=2 SV=2 - [PCCA_MOUSE]	-0.35	-0.32	-0.39	-0.33	0.00
Q9D8S9	BolA-like protein 1 OS=Mus musculus GN=Bola1 PE=1 SV=1 [BOLA1_MOUSE]	-0.31	-0.26	-0.30	-0.32	0.00
Q9EQF6	Dihydropyrimidinase-related protein 5 OS=Mus musculus GN=Dpysl5 PE=1 SV=1 - [DPYL5_MOUSE]	-0.35	-0.28	-0.31	-0.30	0.00
P24527	Leukotriene A-4 hydrolase OS=Mus musculus GN=Lta4h PE=1 SV=4 - [LKHA4_MOUSE]	-0.39	-0.43	-0.45	-0.49	0.00
B2RS76	Carboxypeptidase B1 (Tissue) OS=Mus musculus GN=Cpb1 PE=2 SV=1 - [B2RS76_MOUSE]	0.64	0.72	0.57	0.63	0.00
Q61263	Sterol O-acyltransferase 1 OS=Mus musculus GN=Soat1 PE=2 SV=2 [SOAT1_MOUSE]	0.60	0.67	0.54	0.61	0.00
P32648	VIP peptides OS=Mus musculus GN=Vip PE=1 SV=1 - [VIP_MOUSE]	-0.50	-0.40	-0.49	-0.46	0.00
Q9CXP9	Exonuclease V OS=Mus musculus GN=Exo5 PE=2 SV=1 - [EXO5_MOUSE]	-1.66	-1.87	-1.49	-1.71	0.00
Q8VC28	Aldo-keto reductase family 1 member C13 OS=Mus musculus GN=Akr1c13 PE=1 SV=2 - [AK1CD_MOUSE]	-0.41	-0.34	-0.41	-0.40	0.00
P39061-2	Isoform 3 of Collagen alpha-1(XVIII) chain OS=Mus musculus GN=Col18a1 - [COIA1_MOUSE]	-0.26	-0.27	-0.27	-0.22	0.00
Q09199	Beta-1,4 N-acetylgalactosaminyltransferase 2 OS=Mus musculus GN=B4galnt2 PE=2 SV=1 - [B4GN2_MOUSE]	-0.36	-0.33	-0.28	-0.33	0.00
Q9ER05	Chymopasin OS=Mus musculus GN=Ctrl PE=2 SV=1 - [Q9ER05_MOUSE]	1.56	1.25	1.47	1.50	0.00
E9PVA8	Protein Gcn1l1 OS=Mus musculus GN=Gcn1l1 PE=2 SV=1 - [E9PVA8_MOUSE]	0.55	0.45	0.55	0.56	0.00

P70351	Histone-lysine N-methyltransferase EZH1 OS=Mus musculus GN=Ezh1 PE=1 SV=1 - [EZH1_MOUSE]	0.17	0.20	0.20	0.21	0.00
Q4FK66-2	Isoform 2 of Pre-mRNA splicing factor 38A OS=Mus musculus GN=Prpf38a - [PR38A_MOUSE]	0.47	0.40	0.50	0.43	0.00
Q8QZR5	Alanine aminotransferase 1 OS=Mus musculus GN=Gpt PE=2 SV=3 - [ALAT1_MOUSE]	-1.46	-1.32	-1.65	-1.45	0.00
Q9QY81	Nuclear pore membrane glycoprotein 210 OS=Mus musculus GN=Nup210 PE=1 SV=2 - [PO210_MOUSE]	0.22	0.19	0.22	0.25	0.00
P07310	Creatine kinase M-type OS=Mus musculus GN=Ckm PE=1 SV=1 - [KCRM_MOUSE]	0.29	0.35	0.29	0.33	0.00
Q9Z2I9	Succinyl-CoA ligase [ADP-forming] subunit beta, mitochondrial OS=Mus musculus GN=Sucla2 PE=1 SV=2 - [SUCB1_MOUSE]	-0.19	-0.16	-0.18	-0.16	0.00
Q9EPK6	Nucleotide exchange factor SIL1 OS=Mus musculus GN=Sil1 PE=1 SV=2 - [SIL1_MOUSE]	0.46	0.41	0.52	0.46	0.00
Q8R3U1	HRAS-like suppressor 3 OS=Mus musculus GN=Pla2g16 PE=1 SV=2 - [HRSL3_MOUSE]	0.58	0.59	0.52	0.49	0.00
Q9R1X5	Multidrug resistance-associated protein 5 OS=Mus musculus GN=Abcc5 PE=1 SV=2 - [MRP5_MOUSE]	1.07	0.92	1.15	1.00	0.00
P62259	14-3-3 protein epsilon OS=Mus musculus GN=Ywhae PE=1 SV=1 - [1433E_MOUSE]	-0.34	-0.32	-0.40	-0.38	0.00
E9Q0S6	Protein Tns1 OS=Mus musculus GN=Tns1 PE=2 SV=1 - [E9Q0S6_MOUSE]	0.36	0.32	0.38	0.31	0.00
Q9CXN7	Phenazine biosynthesis like domain-containing protein 2 OS=Mus musculus GN=Pbld2 PE=2 SV=1 - [PBLD2_MOUSE]	-0.82	-0.82	-0.98	-0.82	0.00
Q8VCM7	Fibrinogen gamma chain OS=Mus musculus GN=Fgg PE=2 SV=1 - [FIBG_MOUSE]	-0.31	-0.29	-0.36	-0.30	0.00

Q9JLI0	Aldo-keto reductase a OS=Mus musculus GN=Akr1c12 PE=2 SV=1 - [Q9JLI0_MOUSE]	-0.51	-0.57	-0.64	-0.55	0.00
P54320	Elastin OS=Mus musculus GN=Eln PE=2 SV=2 - [ELN_MOUSE]	-1.17	-1.18	-0.99	-1.00	0.00
P41241	Tyrosine-protein kinase CSK OS=Mus musculus GN=Csk PE=1 SV=2 - [CSK_MOUSE]	0.29	0.28	0.25	0.24	0.00
Q9CQX2	Cytochrome b5 type B OS=Mus musculus GN=Cyb5b PE=1 SV=1 - [CYB5B_MOUSE]	-1.01	-1.23	-1.05	-1.03	0.00
Q7TSV4	Phosphoglucomutase-2 OS=Mus musculus GN=Pgm2 PE=1 SV=1 [PGM2_MOUSE]	-0.21	-0.18	-0.22	-0.19	0.00
Q62472	Vomeronasal secretory protein 2 OS=Mus musculus GN=Lcn4 PE=2 SV=1 - [VNS2_MOUSE]	1.78	2.11	1.78	2.10	0.00
Q00493	Carboxypeptidase E OS=Mus musculus GN=Cpe PE=1 SV=2 - [CBPE_MOUSE]	-0.58	-0.61	-0.58	-0.71	0.00
Q99LE6	ATP-binding cassette sub-family F member 2 OS=Mus musculus GN=Abcf2 PE=2 SV=1 [ABCF2_MOUSE]	0.29	0.35	0.34	0.36	0.00
B1ATS4	Sarcoplasmic/endoplasmic reticulum calcium ATPase 3 OS=Mus musculus GN=Atp2a3 PE=2 SV=1 - [B1ATS4_MOUSE]	0.59	0.60	0.56	0.48	0.00
O88746	Target of Myb protein 1 OS=Mus musculus GN=Tom1 PE=1 SV=1 - [TOM1_MOUSE]	-0.19	-0.18	-0.17	-0.15	0.00
Q9QXG4	Acetyl-coenzyme A synthetase, cytoplasmic OS=Mus musculus GN=Acss2 PE=1 SV=2 - [ACSA_MOUSE]	0.27	0.30	0.29	0.34	0.00
Q8BW96-2	Isoform 2 of Calcium/calmodulin-dependent protein kinase type 1D OS=Mus musculus GN=Camk1d - [KCC1D_MOUSE]	0.55	0.49	0.45	0.44	0.00
P97450	ATP synthase-coupling factor 6, mitochondrial OS=Mus musculus GN=Atp5j PE=1 SV=1 - [ATP5J_MOUSE]	-0.44	-0.40	-0.49	-0.40	0.00
P04441-2	Isoform Short of H-2 class II histocompatibility antigen gamma chain OS=Mus musculus GN=Cd74 - [HG2A_MOUSE]	0.57	0.59	0.47	0.52	0.00

Q2KHK6	Gasdermin-C2 OS=Mus musculus GN=Gsdmc2 PE=2 SV=2 - [GSDC2_MOUSE]	1.69	1.41	1.65	1.41	0.00
Q9QZE5	Coatomer subunit gamma-1 OS=Mus musculus GN=Copg1 PE=2 SV=1 - [COPG1_MOUSE]	0.47	0.57	0.46	0.51	0.00
Q9DAC2	Complement component 8, gamma subunit, isoform CRA_b OS=Mus musculus GN=C8g PE=2 SV=1 - [Q9DAC2_MOUSE]	-1.09	-1.06	-0.91	-0.90	0.00
Q3TCN2	Putative phospholipase B-like 2 OS=Mus musculus GN=Plbd2 PE=1 SV=2 - [PLBL2_MOUSE]	-0.31	-0.30	-0.37	-0.32	0.00
Q9CR57	60S ribosomal protein L14 OS=Mus musculus GN=Rpl14 PE=2 SV=3 [RL14_MOUSE]	0.28	0.34	0.34	0.32	0.00
Q8BWQ5	Serine/threonine-protein kinase DCLK3 OS=Mus musculus GN=Dclk3 PE=2 SV=2 [DCLK3_MOUSE]	-0.40	-0.44	-0.35	-0.40	0.00
Q8CFI7	DNA-directed RNA polymerase II subunit RPB2 OS=Mus musculus GN=Polr2b PE=2 SV=2 - [RPB2_MOUSE]	0.33	0.39	0.41	0.39	0.00
P17183	Gamma-enolase OS=Mus musculus GN=Eno2 PE=1 SV=2 - [ENOG_MOUSE]	-0.36	-0.37	-0.34	-0.29	0.00
Q61879	Myosin-10 OS=Mus musculus GN=Myh10 PE=1 SV=2 - [MYH10_MOUSE]	-0.18	-0.15	-0.14	-0.15	0.00
A2AQE4	COP9 signalosome complex subunit 2 OS=Mus musculus GN=Cops2 PE=2 SV=1 - [A2AQE4_MOUSE]	0.65	0.58	0.53	0.54	0.00
Q91WU0	Expressed sequence AU018778 OS=Mus musculus GN=Ces1f PE=2 SV=1 - [Q91WU0_MOUSE]	-0.88	-0.80	-1.01	-0.86	0.00
P19096	Fatty acid synthase OS=Mus musculus GN=Fasn PE=1 SV=2 - [FAS_MOUSE]	0.14	0.16	0.17	0.14	0.00
Q8JZV7	Putative N-acetylglucosamine-6-phosphate deacetylase OS=Mus musculus GN=Amdhd2 PE=2 SV=1 - [NAGA_MOUSE]	-0.28	-0.28	-0.25	-0.32	0.00
O35678	Monoglyceride lipase OS=Mus musculus GN=Mgll PE=1 SV=1 - [MGLL_MOUSE]	-0.58	-0.51	-0.64	-0.54	0.00

P50608	Fibromodulin OS=Mus musculus GN=Fmod PE=2 SV=1 - [FMOD_MOUSE]	0.90	0.78	0.99	0.87	0.00
Q91ZY2	Histamine H4 receptor OS=Mus musculus GN=Hrh4 PE=2 SV=1 - [HRH4_MOUSE]	-0.15	-0.15	-0.18	-0.18	0.00
Q9D3S3	Sorting nexin-29 OS=Mus musculus GN=Snx29 PE=1 SV=2 - [SNX29_MOUSE]	-1.50	-1.36	-1.32	-1.18	0.00
P19221	Prothrombin OS=Mus musculus GN=F2 PE=1 SV=1 - [THRB_MOUSE]	-0.45	-0.47	-0.41	-0.38	0.00
Q5NCB9	Tripartite motif-containing protein 7 (Fragment) OS=Mus musculus GN=Trim7 PE=2 SV=2 - [Q5NCB9_MOUSE]	-0.60	-0.56	-0.70	-0.67	0.00
P02089	Hemoglobin subunit beta-2 OS=Mus musculus GN=Hbb-b2 PE=1 SV=2 - [HBB2_MOUSE]	1.43	1.23	1.31	1.13	0.00
H3BLR7	Atrial natriuretic peptide converting enzyme (Fragment) OS=Mus musculus GN=Corin PE=2 SV=1 - [H3BLR7_MOUSE]	1.31	1.51	1.46	1.67	0.00
O88325	Alpha-N-acetylglucosaminidase OS=Mus musculus GN=Naglu PE=2 SV=1 [O88325_MOUSE]	-0.44	-0.42	-0.50	-0.40	0.00
O70494-3	Isoform 3 of Transcription factor Sp3 OS=Mus musculus GN=Sp3 - [SP3_MOUSE]	-1.00	-0.93	-1.17	-1.09	0.00
A2AFG8	Neural cell adhesion molecule L1 OS=Mus musculus GN=L1cam PE=2 SV=1 - [A2AFG8_MOUSE]	-0.37	-0.34	-0.37	-0.30	0.00
Q9WTU6-3	Isoform Beta-1 of Mitogen-activated protein kinase 9 OS=Mus musculus GN=Mapk9 - [MK09_MOUSE]	-0.14	-0.14	-0.11	-0.14	0.00
A3KMP2	Tetratricopeptide repeat protein 38 OS=Mus musculus GN=Ttc38 PE=2 SV=2 - [TTC38_MOUSE]	-0.68	-0.69	-0.58	-0.58	0.00
O35459	Delta(3,5)-Delta(2,4)-dienoyl-CoA isomerase, mitochondrial OS=Mus musculus GN=Ech1 PE=2 SV=1 - [ECH1_MOUSE]	-0.80	-0.65	-0.79	-0.70	0.00
P19001	Keratin, type I cytoskeletal 19 OS=Mus musculus GN=Krt19 PE=1 SV=1 - [K1C19_MOUSE]	-0.30	-0.26	-0.29	-0.24	0.00

Q99K41	EMILIN-1 OS=Mus musculus GN=Emilin1 PE=1 SV=1 - [EMIL1_MOUSE]	-0.49	-0.55	-0.43	-0.48	0.00
O70475	UDP-glucose 6-dehydrogenase OS=Mus musculus GN=Ugdh PE=1 SV=1 - [UGDH_MOUSE]	-0.76	-0.64	-0.76	-0.64	0.00
Q91YQ5	Dolichyl-diphosphooligosaccharide--protein glycosyltransferase subunit 1 OS=Mus musculus GN=Rpn1 PE=2 SV=1 - [RPN1_MOUSE]	0.39	0.44	0.36	0.44	0.00
Q8BH59	Calcium-binding mitochondrial carrier protein Aralar1 OS=Mus musculus GN=Slc25a12 PE=1 SV=1 - [CMC1_MOUSE]	0.13	0.13	0.12	0.15	0.00
D3Z7V3	Sarcolemmal membrane-associated protein OS=Mus musculus GN=Slmap PE=2 SV=1 - [D3Z7V3_MOUSE]	0.23	0.26	0.24	0.21	0.00
Q3UKU1	RNA polymerase II elongation factor ELL2 OS=Mus musculus GN=Ell2 PE=2 SV=2 - [ELL2_MOUSE]	1.39	1.45	1.65	1.72	0.00
P97868-2	Isoform 2 of E3 ubiquitin-protein ligase RBBP6 OS=Mus musculus GN=Rbbp6 - [RBBP6_MOUSE]	0.14	0.18	0.15	0.16	0.00
P26040	Ezrin OS=Mus musculus GN=Ezr PE=1 SV=3 - [EZRI_MOUSE]	0.28	0.34	0.27	0.28	0.00
P47754	F-actin-capping protein subunit alpha-2 OS=Mus musculus GN=Capza2 PE=1 SV=3 - [CAZA2_MOUSE]	-0.16	-0.14	-0.18	-0.16	0.00
E9QNL4	Inhibitor of nuclear factor kappa-B kinase subunit alpha OS=Mus musculus GN=Chuk PE=2 SV=1 - [E9QNL4_MOUSE]	-2.36	-2.66	-2.21	-2.11	0.00
Q9Z2M7	Phosphomannomutase 2 OS=Mus musculus GN=Pmm2 PE=2 SV=1 - [PMM2_MOUSE]	0.48	0.60	0.49	0.53	0.00
Q9DBM2	Peroxisomal bifunctional enzyme OS=Mus musculus GN=Ehhadh PE=1 SV=4 - [ECHP_MOUSE]	-0.28	-0.35	-0.34	-0.34	0.00
P19467	Mucin-13 OS=Mus musculus GN=Muc13 PE=2 SV=1 - [MUC13_MOUSE]	0.24	0.24	0.20	0.21	0.00
Q3U4W8	Ubiquitin carboxyl-terminal hydrolase OS=Mus musculus GN=Usp5 PE=2 SV=1 - [Q3U4W8_MOUSE]	0.06	0.05	0.05	0.05	0.00

Q5SSG2	Protein Mmp28 OS=Mus musculus GN=Mmp28 PE=2 SV=2 - [Q5SSG2_MOUSE]	-1.31	-1.33	-1.57	-1.59	0.00
Q9CR92	Coiled-coil domain-containing protein 96 OS=Mus musculus GN=Ccdc96 PE=2 SV=1 - [CCD96_MOUSE]	0.64	0.69	0.65	0.80	0.00
Q9CQC6	Basic leucine zipper and W2 domain-containing protein 1 OS=Mus musculus GN=Bzw1 PE=1 SV=1 - [BZW1_MOUSE]	0.43	0.35	0.37	0.35	0.00
Q9QXX8	Nuclear fragile X mental retardation-interacting protein 1 OS=Mus musculus GN=Nufip1 PE=1 SV=1 - [NUFP1_MOUSE]	0.73	0.79	0.62	0.68	0.00
P63030	Mitochondrial pyruvate carrier 1 OS=Mus musculus GN=Mpc1 PE=1 SV=1 - [MPC1_MOUSE]	-1.08	-1.29	-1.06	-1.27	0.00
Q64442	Sorbitol dehydrogenase OS=Mus musculus GN=Sord PE=1 SV=3 - [DHSO_MOUSE]	-0.49	-0.40	-0.49	-0.42	0.00
Q60967	Bifunctional 3'-phosphoadenosine 5'-phosphosulfate synthase 1 OS=Mus musculus GN=Papss1 PE=2 SV=1 - [PAPS1_MOUSE]	0.31	0.34	0.37	0.30	0.00
F6TP58	Protein Dchs1 (Fragment) OS=Mus musculus GN=Dchs1 PE=4 SV=1 - [F6TP58_MOUSE]	-0.72	-0.91	-0.85	-0.92	0.00
Q9R097	Kunitz-type protease inhibitor 1 OS=Mus musculus GN=Spint1 PE=1 SV=2 - [SPIT1_MOUSE]	0.32	0.34	0.40	0.39	0.00
Q8BM55	Transmembrane protein 214 OS=Mus musculus GN=Tmem214 PE=2 SV=1 - [TM214_MOUSE]	0.53	0.47	0.60	0.51	0.00
P80318	T-complex protein 1 subunit gamma OS=Mus musculus GN=Cct3 PE=1 SV=1 - [TCPG_MOUSE]	0.17	0.19	0.16	0.20	0.00
Q99KF1	Transmembrane emp24 domain-containing protein 9 OS=Mus musculus GN=Tmed9 PE=2 SV=2 - [TMED9_MOUSE]	0.43	0.52	0.41	0.47	0.00
P06909	Complement factor H OS=Mus musculus GN=Cfh PE=1 SV=2 - [CFAH_MOUSE]	-0.32	-0.40	-0.34	-0.32	0.00

F7A6H4	E3 ubiquitin-protein ligase RNF213 OS=Mus musculus GN=Rnf213 PE=4 SV=2 - [F7A6H4_MOUSE]	0.53	0.51	0.64	0.55	0.00
O70570	Polymeric immunoglobulin receptor OS=Mus musculus GN=Pigr PE=1 SV=1 - [PIGR_MOUSE]	0.18	0.19	0.15	0.18	0.00
Q8BH35	Complement component C8 beta chain OS=Mus musculus GN=C8b PE=1 SV=1 - [CO8B_MOUSE]	-0.64	-0.63	-0.55	-0.52	0.00
Q9D312	Keratin, type I cytoskeletal 20 OS=Mus musculus GN=Krt20 PE=1 SV=1 - [K1C20_MOUSE]	-0.36	-0.31	-0.33	-0.28	0.00
P43241	Homeobox protein CDX-2 OS=Mus musculus GN=Cdx2 PE=1 SV=1 - [CDX2_MOUSE]	0.14	0.15	0.15	0.18	0.00
O70435	Proteasome subunit alpha type-3 OS=Mus musculus GN=Psma3 PE=1 SV=3 - [PSA3_MOUSE]	-0.40	-0.37	-0.33	-0.31	0.00
Q9DCW4	Electron transfer flavoprotein subunit beta OS=Mus musculus GN=Etfb PE=1 SV=3 - [ETFB_MOUSE]	-0.43	-0.52	-0.43	-0.42	0.00
G5E8Q4	Cytohesin-3 OS=Mus musculus GN=Cyth3 PE=4 SV=1 - [G5E8Q4_MOUSE]	-0.39	-0.47	-0.43	-0.51	0.00
P07091	Protein S100-A4 OS=Mus musculus GN=S100a4 PE=1 SV=1 - [S10A4_MOUSE]	-0.18	-0.22	-0.22	-0.20	0.00
Q8JZS0	Protein lin-7 homolog A OS=Mus musculus GN=Lin7a PE=1 SV=2 - [LIN7A_MOUSE]	-0.12	-0.11	-0.14	-0.13	0.00
P46978	Dolichyl-diphosphooligosaccharide--protein glycosyltransferase subunit STT3A OS=Mus musculus GN=Stt3a PE=1 SV=1 - [STT3A_MOUSE]	0.42	0.51	0.44	0.52	0.00
P46096	Synaptotagmin-1 OS=Mus musculus GN=Syt1 PE=1 SV=1 - [SYT1_MOUSE]	-0.46	-0.52	-0.40	-0.43	0.00
Q9WVH9	Fibulin-5 OS=Mus musculus GN=Fbln5 PE=2 SV=1 - [FBLN5_MOUSE]	-0.21	-0.19	-0.24	-0.22	0.00
Q99LJ1	Tissue alpha-L-fucosidase OS=Mus musculus GN=Fuca1 PE=2 SV=1 - [FUCO_MOUSE]	-0.52	-0.56	-0.53	-0.43	0.00

Q9R1P0	Proteasome subunit alpha type-4 OS=Mus musculus GN=Psma4 PE=1 SV=1 - [PSA4_MOUSE]	-0.42	-0.48	-0.37	-0.41	0.00
D3YWR2	B-cell linker protein OS=Mus musculus GN=Blnk PE=2 SV=1 - [D3YWR2_MOUSE]	0.43	0.52	0.45	0.54	0.00
Q8BH80	Vesicle-associated membrane protein, associated protein B and C OS=Mus musculus GN=Vapb PE=2 SV=1 - [Q8BH80_MOUSE]	0.21	0.23	0.18	0.21	0.00
Q99PT1	Rho GDP-dissociation inhibitor 1 OS=Mus musculus GN=Arhgdia PE=1 SV=3 - [GDIR1_MOUSE]	0.55	0.59	0.51	0.46	0.00
O88544	COP9 signalosome complex subunit 4 OS=Mus musculus GN=Cops4 PE=1 SV=1 - [CSN4_MOUSE]	0.29	0.34	0.27	0.28	0.00
Q923T9-3	Isoform 3 of Calcium/calmodulin-dependent protein kinase type II subunit gamma OS=Mus musculus GN=Camk2g - [KCC2G_MOUSE]	0.60	0.47	0.57	0.56	0.00
B5THE2	Maltase-glucoamylase OS=Mus musculus GN=Mgam PE=2 SV=1 - [B5THE2_MOUSE]	-0.27	-0.30	-0.26	-0.33	0.00
P26262	Plasma kallikrein OS=Mus musculus GN=Klkb1 PE=1 SV=2 - [KLKB1_MOUSE]	-1.45	-1.20	-1.51	-1.27	0.00
Q8VI94	2'-5'-oligoadenylate synthase-like protein 1 OS=Mus musculus GN=Oasl1 PE=2 SV=1 [OASL1_MOUSE]	0.46	0.47	0.38	0.39	0.00
Q925F2	Endothelial cell-selective adhesion molecule OS=Mus musculus GN=Esam PE=1 SV=1 - [ESAM_MOUSE]	-0.53	-0.61	-0.62	-0.69	0.00
O09061	Proteasome subunit beta type-1 OS=Mus musculus GN=Psmb1 PE=1 SV=1 - [PSB1_MOUSE]	-0.22	-0.18	-0.20	-0.17	0.00
P62806	Histone H4 OS=Mus musculus GN=Hist1h4a PE=1 SV=2 - [H4_MOUSE]	-0.44	-0.39	-0.50	-0.42	0.00
Q8R191	Synaptogyrin-3 OS=Mus musculus GN=Syngr3 PE=1 SV=1 - [SNG3_MOUSE]	-0.54	-0.45	-0.56	-0.46	0.00
Q03311	Cholinesterase OS=Mus musculus GN=Bche PE=2 SV=2 - [CHLE_MOUSE]	-0.77	-0.81	-0.88	-0.98	0.00

Q8VIJ6	Splicing factor, proline- and glutamine-rich OS=Mus musculus GN=Sfpq PE=1 SV=1 - [SFPQ_MOUSE]	-0.17	-0.15	-0.19	-0.15	0.00
F6RJC1	Teneurin-3 (Fragment) OS=Mus musculus GN=Tenm3 PE=4 SV=1 - [F6RJC1_MOUSE]	0.71	0.68	0.60	0.56	0.00
Q9ERU9	E3 SUMO-protein ligase RanBP2 OS=Mus musculus GN=Ranbp2 PE=1 SV=2 - [RBP2_MOUSE]	0.21	0.19	0.18	0.23	0.00
A2AEX8	Four and a half LIM domains 1, isoform CRA_b OS=Mus musculus GN=Fhl1 PE=4 SV=1 - [A2AEX8_MOUSE]	0.55	0.63	0.48	0.58	0.00
Q61024	Asparagine synthetase [glutamine-hydrolyzing] OS=Mus musculus GN=Asns PE=2 SV=3 - [ASNS_MOUSE]	0.90	0.88	0.71	0.88	0.00
P62281	40S ribosomal protein S11 OS=Mus musculus GN=Rps11 PE=2 SV=3 - [RS11_MOUSE]	0.37	0.44	0.38	0.46	0.00
E9QAP7	Protein Taf4a OS=Mus musculus GN=Taf4a PE=2 SV=1 - [E9QAP7_MOUSE]	-0.32	-0.39	-0.33	-0.39	0.00
Q8K1R3	Polyribonucleotide nucleotidyltransferase 1, mitochondrial OS=Mus musculus GN=Pnpt1 PE=1 SV=1 [PNPT1_MOUSE]	-0.15	-0.18	-0.15	-0.14	0.00
Q99N05	Membrane-spanning 4-domains subfamily A member 4D OS=Mus musculus GN=Ms4a4d PE=2 SV=1 - [M4A4D_MOUSE]	-0.38	-0.33	-0.34	-0.29	0.00
E0CZE8	Bax inhibitor 1 (Fragment) OS=Mus musculus GN=Tmbim6 PE=2 SV=1 - [E0CZE8_MOUSE]	0.81	0.97	0.77	0.93	0.00
Q921L5	Conserved oligomeric Golgi complex subunit 2 OS=Mus musculus GN=Cog2 PE=2 SV=2 - [COG2_MOUSE]	0.57	0.49	0.64	0.55	0.00
Q8R0N6	Hydroxyacid-oxoacid transhydrogenase, mitochondrial OS=Mus musculus GN=Adhfe1 PE=2 SV=2 - [HOT_MOUSE]	-1.07	-1.29	-1.11	-1.34	0.00
Q9JIF7	Coatomer subunit beta OS=Mus musculus GN=Copb1 PE=1 SV=1 - [COPB_MOUSE]	0.54	0.57	0.45	0.48	0.00

Q9JJV2-3	Isoform 3 of Profilin-2 OS=Mus musculus GN=Pfn2 - [PROF2_MOUSE]	-0.24	-0.26	-0.31	-0.29	0.00
Q8BP47	Asparagine--tRNA ligase, cytoplasmic OS=Mus musculus GN=Nars PE=1 SV=2 - [SYNC_MOUSE]	0.50	0.60	0.48	0.48	0.00
Q9WV55	Vesicle-associated membrane protein-associated protein A OS=Mus musculus GN=Vapa PE=1 SV=2 - [VAPA_MOUSE]	0.30	0.39	0.35	0.37	0.00
Q91VK1	Basic leucine zipper and W2 domain-containing protein 2 OS=Mus musculus GN=Bzw2 PE=1 SV=1 - [BZW2_MOUSE]	0.41	0.33	0.39	0.34	0.00
Q61881	DNA replication licensing factor MCM7 OS=Mus musculus GN=Mcm7 PE=2 SV=1 - [MCM7_MOUSE]	0.54	0.42	0.53	0.50	0.00
Q7TNC4-2	Isoform 2 of Putative RNA-binding protein Luc7-like 2 OS=Mus musculus GN=Luc7l2 - [LC7L2_MOUSE]	0.31	0.35	0.27	0.33	0.00
P14576	Signal recognition particle 54 kDa protein OS=Mus musculus GN=Srp54 PE=1 SV=2 - [SRP54_MOUSE]	0.32	0.38	0.35	0.41	0.00
A2APM2	CD44 antigen OS=Mus musculus GN=Cd44 PE=2 SV=1 - [A2APM2_MOUSE]	-0.38	-0.35	-0.44	-0.34	0.00
Q8CEE6	PAS domain-containing serine/threonine-protein kinase OS=Mus musculus GN=Pask PE=2 SV=3 - [PASK_MOUSE]	-0.11	-0.09	-0.12	-0.11	0.00
Q8K2I3	Dimethylaniline monooxygenase [N-oxide-forming] 2 OS=Mus musculus GN=Fmo2 PE=1 SV=3 [FMO2_MOUSE]	-0.91	-0.95	-0.75	-0.79	0.00
Q8R2Y2-2	Isoform 2 of Cell surface glycoprotein MUC18 OS=Mus musculus GN=Mcam - [MUC18_MOUSE]	-0.46	-0.41	-0.43	-0.35	0.00
Q9D1H9	Microfibril-associated glycoprotein 4 OS=Mus musculus GN=Mfap4 PE=1 SV=1 - [MFAP4_MOUSE]	-0.55	-0.51	-0.65	-0.62	0.00
Q6ZWV7	60S ribosomal protein L35 OS=Mus musculus GN=Rpl35 PE=2 SV=1 [RL35_MOUSE]	-0.19	-0.24	-0.21	-0.19	0.00

Q5NCU0	Glycine receptor subunit alpha-1 OS=Mus musculus GN=Glra1 PE=2 SV=1 - [Q5NCU0_MOUSE]	-0.70	-0.78	-0.84	-0.91	0.00
P08122	Collagen alpha-2(IV) chain OS=Mus musculus GN=Col4a2 PE=2 SV=4 - [CO4A2_MOUSE]	-1.41	-1.43	-1.36	-1.11	0.00
Q9EQG7	Ectonucleotide pyrophosphatase/phosphodiesterase family member 5 OS=Mus musculus GN=Enpp5 PE=2 SV=3 - [ENPP5_MOUSE]	-0.65	-0.53	-0.67	-0.58	0.00
Q6PDI5	Proteasome-associated protein ECM29 homolog OS=Mus musculus GN=Ecm29 PE=1 SV=3 - [ECM29_MOUSE]	0.29	0.37	0.33	0.37	0.00
A2AUK7	Band 4.1-like protein 1 OS=Mus musculus GN=Epb4.1l1 PE=2 SV=1 - [A2AUK7_MOUSE]	-0.48	-0.49	-0.39	-0.41	0.00
P62908	40S ribosomal protein S3 OS=Mus musculus GN=Rps3 PE=1 SV=1 - [RS3_MOUSE]	0.32	0.35	0.28	0.28	0.00
Q9EQP2	EH domain-containing protein 4 OS=Mus musculus GN=Ehd4 PE=1 SV=1 - [EHD4_MOUSE]	0.24	0.25	0.26	0.31	0.00
P21107-2	Isoform 2 of Tropomyosin alpha-3 chain OS=Mus musculus GN=Tpm3 - [TPM3_MOUSE]	-0.35	-0.27	-0.33	-0.30	0.00
P68181-2	Isoform 2 of cAMP-dependent protein kinase catalytic subunit beta OS=Mus musculus GN=Prkacb - [KAPCB_MOUSE]	0.54	0.64	0.50	0.60	0.00
D3YWQ8	Protein kinase C eta type OS=Mus musculus GN=Prkch PE=2 SV=1 - [D3YWQ8_MOUSE]	0.47	0.53	0.56	0.62	0.00
P53810	Phosphatidylinositol transfer protein alpha isoform OS=Mus musculus GN=Pitpna PE=1 SV=2 - [PIPNA_MOUSE]	0.34	0.38	0.31	0.29	0.00
Q3UN10	Wolframin OS=Mus musculus GN=Wfs1 PE=2 SV=1 - [Q3UN10_MOUSE]	0.29	0.32	0.28	0.35	0.00
Q6PGF7	Exocyst complex component 8 OS=Mus musculus GN=Exoc8 PE=1 SV=1 - [EXOC8_MOUSE]	0.18	0.21	0.20	0.23	0.00
Q5FWK3	Rho GTPase-activating protein 1 OS=Mus musculus GN=Arhgap1 PE=1 SV=1 - [RHG01_MOUSE]	0.36	0.30	0.27	0.31	0.00

P01753	Ig heavy chain V region 186-1 OS=Mus musculus PE=4 SV=2 - [HVM09_MOUSE]	-1.42	-1.34	-1.71	-1.63	0.00
P13808-2	Isoform B1 of Anion exchange protein 2 OS=Mus musculus GN=Slc4a2 - [B3A2_MOUSE]	-0.63	-0.60	-0.53	-0.49	0.00
Q91YR7	Pre-mRNA-processing factor 6 OS=Mus musculus GN=Prpf6 PE=2 SV=1 - [PRP6_MOUSE]	0.22	0.21	0.27	0.25	0.00
Q9D2R6	Cytochrome c oxidase assembly protein 3 homolog, mitochondrial OS=Mus musculus GN=Coa3 PE=2 SV=1 - [COA3_MOUSE]	0.43	0.50	0.45	0.55	0.00
Q9CY57-3	Isoform 3 of Chromatin target of PRMT1 protein OS=Mus musculus GN=Chtop - [CHTOP_MOUSE]	-0.85	-0.93	-0.97	-0.74	0.00
E9Q035	Protein Gm20425 OS=Mus musculus GN=Gm20425 PE=4 SV=1 - [E9Q035_MOUSE]	-0.37	-0.32	-0.41	-0.33	0.00
M0QW74	MCG9626, isoform CRA_c OS=Mus musculus GN=Mtmr3 PE=4 SV=1 - [M0QW74_MOUSE]	0.71	0.61	0.80	0.70	0.00
Q8K2C6	NAD-dependent protein deacylase sirtuin-5, mitochondrial OS=Mus musculus GN=Sirt5 PE=1 SV=1 - [SIR5_MOUSE]	-0.29	-0.24	-0.23	-0.27	0.00
Q70IV5	Synemin OS=Mus musculus GN=Synm PE=1 SV=2 - [SYNEM_MOUSE]	0.69	0.67	0.70	0.54	0.00
P10107	Annexin A1 OS=Mus musculus GN=Anxa1 PE=1 SV=2 - [ANXA1_MOUSE]	0.61	0.52	0.61	0.48	0.00
Q9QUM9	Proteasome subunit alpha type-6 OS=Mus musculus GN=Psma6 PE=1 SV=1 - [PSA6_MOUSE]	-0.29	-0.34	-0.30	-0.25	0.00
Q9CQ19	Myosin regulatory light polypeptide 9 OS=Mus musculus GN=Myl9 PE=1 SV=3 - [MYL9_MOUSE]	-0.23	-0.21	-0.21	-0.18	0.00
Q9D6K9	Ceramide synthase 5 OS=Mus musculus GN=Cers5 PE=1 SV=1 [CERS5_MOUSE]	0.34	0.41	0.32	0.38	0.00
Q9D2Q8	Protein S100-A14 OS=Mus musculus GN=S100a14 PE=2 SV=1 - [S10AE_MOUSE]	0.64	0.82	0.66	0.73	0.00

E9Q9E1	Eukaryotic translation initiation factor 4 gamma 1 OS=Mus musculus GN=Eif4g1 PE=2 SV=1 - [E9Q9E1_MOUSE]	0.35	0.32	0.27	0.34	0.00
P29788	Vitronectin OS=Mus musculus GN=Vtn PE=1 SV=2 - [VTNC_MOUSE]	-0.40	-0.37	-0.35	-0.46	0.00
P68368	Tubulin alpha-4A chain OS=Mus musculus GN=Tuba4a PE=1 SV=1 - [TBA4A_MOUSE]	0.26	0.25	0.29	0.22	0.00
P05201	Aspartate aminotransferase, cytoplasmic OS=Mus musculus GN=Got1 PE=1 SV=3 - [AATC_MOUSE]	-0.34	-0.40	-0.30	-0.36	0.00
P97347	Repetin OS=Mus musculus GN=Rptn PE=1 SV=2 - [RPTN_MOUSE]	-0.83	-0.90	-0.69	-0.75	0.00
P61750	ADP-ribosylation factor 4 OS=Mus musculus GN=Arf4 PE=2 SV=2 - [ARF4_MOUSE]	0.39	0.30	0.38	0.36	0.00
P51855	Glutathione synthetase OS=Mus musculus GN=Gss PE=2 SV=1 - [GSHB_MOUSE]	-0.41	-0.36	-0.36	-0.31	0.00
Q9JJI8	60S ribosomal protein L38 OS=Mus musculus GN=Rpl38 PE=2 SV=3 [RL38_MOUSE]	0.48	0.63	0.56	0.59	0.00
P28481-7	Isoform 7 of Collagen alpha-1(II) chain OS=Mus musculus GN=Col2a1 - [CO2A1_MOUSE]	-1.21	-0.99	-1.32	-1.21	0.00
D3YVP5	Glutathione S-transferase Mu 7 OS=Mus musculus GN=Gstm7 PE=2 SV=1 - [D3YVP5_MOUSE]	0.13	0.16	0.13	0.16	0.00
P53995	Anaphase-promoting complex subunit 1 OS=Mus musculus GN=Anapc1 PE=1 SV=2 - [APC1_MOUSE]	0.33	0.34	0.39	0.42	0.00
F6R7Y0	CMP-sialic acid transporter (Fragment) OS=Mus musculus GN=Slc35a1 PE=4 SV=1 - [F6R7Y0_MOUSE]	0.44	0.49	0.50	0.58	0.00
Q9D1T5	Proline-rich protein 15 OS=Mus musculus GN=Prr15 PE=2 SV=1 - [PRR15_MOUSE]	-1.24	-1.02	-1.33	-1.12	0.00
Q9DBZ1-2	Isoform 2 of Inhibitor of nuclear factor kappa-B kinase-interacting protein OS=Mus musculus GN=Ikbip - [IKIP_MOUSE]	-1.17	-1.08	-0.99	-0.89	0.00

D3Z0V2	Rho guanine nucleotide exchange factor 7 OS=Mus musculus GN=Arhgef7 PE=2 SV=1 - [D3Z0V2_MOUSE]	0.31	0.33	0.25	0.31	0.00
G3X922	MCG115602 OS=Mus musculus GN=Dnajc13 PE=4 SV=1 - [G3X922_MOUSE]	0.19	0.22	0.25	0.23	0.00
Q9ESP1	Stromal cell-derived factor 2-like protein 1 OS=Mus musculus GN=Sdf2l1 PE=2 SV=2 - [SDF2L_MOUSE]	0.43	0.51	0.39	0.48	0.00
Q9JMG7	Hepatoma-derived growth factor-related protein 3 OS=Mus musculus GN=Hdgfrp3 PE=1 SV=2 - [HDGR3_MOUSE]	0.39	0.47	0.37	0.40	0.00
P40336	Vacuolar protein sorting associated protein 26A OS=Mus musculus GN=Vps26a PE=2 SV=1 - [VP26A_MOUSE]	-0.28	-0.22	-0.26	-0.22	0.00
Q9D8T2	Gasdermin-D OS=Mus musculus GN=Gsdmdc1 PE=2 SV=1 - [GSDMD_MOUSE]	0.26	0.26	0.21	0.27	0.00
Q04857	Collagen alpha-1(VI) chain OS=Mus musculus GN=Col6a1 PE=2 SV=1 - [CO6A1_MOUSE]	-0.44	-0.53	-0.44	-0.55	0.00
Q3UMB9	WASH complex subunit 7 OS=Mus musculus GN=Kiaa1033 PE=2 SV=2 - [WASH7_MOUSE]	0.52	0.59	0.44	0.51	0.00
Q9WVP1	AP-1 complex subunit mu-2 OS=Mus musculus GN=Ap1m2 PE=1 SV=3 - [AP1M2_MOUSE]	0.80	0.77	0.97	0.94	0.00
A2A7S8-2	Isoform 2 of Uncharacterized protein KIAA1522 OS=Mus musculus GN=Kiaa1522 - [K1522_MOUSE]	-0.26	-0.29	-0.26	-0.22	0.00
P46660	Alpha-internexin OS=Mus musculus GN=Ina PE=1 SV=2 - [AINX_MOUSE]	-0.53	-0.60	-0.63	-0.71	0.00
Q9R0P5	Destrin OS=Mus musculus GN=Dstn PE=1 SV=3 - [DEST_MOUSE]	0.13	0.16	0.13	0.13	0.00
P35492	Histidine ammonia-lyase OS=Mus musculus GN=Hal PE=1 SV=1 - [HUTH_MOUSE]	0.77	0.60	0.70	0.62	0.00
P68404-2	Isoform Beta-II of Protein kinase C beta type OS=Mus musculus GN=Prkcb - [KPCB_MOUSE]	0.67	0.76	0.62	0.58	0.00

P07759	Serine protease inhibitor A3K OS=Mus musculus GN=Serpina3k PE=1 SV=2 - [SPA3K_MOUSE]	-1.25	-1.11	-1.04	-0.94	0.00
Q9DBC7	cAMP-dependent protein kinase type I-alpha regulatory subunit OS=Mus musculus GN=Prkar1a PE=1 SV=3 - [KAP0_MOUSE]	0.39	0.39	0.34	0.30	0.00
Q99K30	Epidermal growth factor receptor kinase substrate 8-like protein 2 OS=Mus musculus GN=Eps8l2 PE=1 SV=1 - [ES8L2_MOUSE]	-0.34	-0.43	-0.41	-0.45	0.00
Q91YP2	Neurolysin, mitochondrial OS=Mus musculus GN=Nln PE=2 SV=1 - [NEUL_MOUSE]	-0.21	-0.23	-0.21	-0.27	0.00
P50247	Adenosylhomocysteinase OS=Mus musculus GN=Ahcy PE=1 SV=3 - [SAHH_MOUSE]	-0.33	-0.28	-0.37	-0.31	0.00
Q68FL6	Methionine--tRNA ligase, cytoplasmic OS=Mus musculus GN=Mars PE=2 SV=1 - [SYMC_MOUSE]	0.25	0.24	0.30	0.23	0.00
Q3UG93	Heat shock factor protein 4 OS=Mus musculus GN=Hsf4 PE=2 SV=1 - [Q3UG93_MOUSE]	0.59	0.59	0.48	0.48	0.00
P47746	Cannabinoid receptor 1 OS=Mus musculus GN=Cnr1 PE=2 SV=1 - [CNR1_MOUSE]	-0.97	-0.87	-0.82	-0.73	0.00
Q8VDP3-2	Isoform 2 of Protein-methionine sulfoxide oxidase MICAL1 OS=Mus musculus GN=Mical1 - [MICA1_MOUSE]	0.20	0.21	0.26	0.22	0.00
Q99NG0	Helicase ARIP4 OS=Mus musculus GN=Rad54l2 PE=1 SV=1 - [ARIP4_MOUSE]	0.19	0.23	0.18	0.22	0.00
Q99JW5	Epithelial cell adhesion molecule OS=Mus musculus GN=Epcam PE=2 SV=1 - [EPCAM_MOUSE]	0.37	0.45	0.34	0.41	0.00
Q99PF4-2	Isoform 2 of Cadherin-23 OS=Mus musculus GN=Cdh23 - [CAD23_MOUSE]	1.01	1.07	0.80	0.96	0.00
Q9CZS1	Aldehyde dehydrogenase X, mitochondrial OS=Mus musculus GN=Aldh1b1 PE=2 SV=1 - [AL1B1_MOUSE]	-0.41	-0.31	-0.40	-0.35	0.00

Q9DB77	Cytochrome b-c1 complex subunit 2, mitochondrial OS=Mus musculus GN=Uqcrc2 PE=1 SV=1 - [QCR2_MOUSE]	-0.14	-0.17	-0.13	-0.15	0.00
P14685	26S proteasome non-ATPase regulatory subunit 3 OS=Mus musculus GN=Psmd3 PE=1 SV=3 - [PSMD3_MOUSE]	0.29	0.34	0.26	0.27	0.00
E9QNL5	Sulfotransferase 1A1 OS=Mus musculus GN=Sult1a1 PE=2 SV=1 - [E9QNL5_MOUSE]	-0.14	-0.11	-0.13	-0.15	0.00
P01901	H-2 class I histocompatibility antigen, K-B alpha chain OS=Mus musculus GN=H2-K1 PE=1 SV=1 - [HA1B_MOUSE]	0.25	0.29	0.27	0.22	0.00
P54775	26S protease regulatory subunit 6B OS=Mus musculus GN=Psmc4 PE=1 SV=2 - [PRS6B_MOUSE]	0.63	0.73	0.55	0.59	0.00
Q61656	Probable ATP-dependent RNA helicase DDX5 OS=Mus musculus GN=Ddx5 PE=1 SV=2 - [DDX5_MOUSE]	0.34	0.39	0.35	0.44	0.00
Q8R4U7	Leucine zipper protein 1 OS=Mus musculus GN=Luzp1 PE=1 SV=2 - [LUZP1_MOUSE]	-0.23	-0.18	-0.18	-0.18	0.00
F8VPX1	Ubiquitin carboxyl-terminal hydrolase OS=Mus musculus GN=Usp7 PE=2 SV=1 - [F8VPX1_MOUSE]	0.38	0.37	0.28	0.33	0.00
P26618	Platelet-derived growth factor receptor alpha OS=Mus musculus GN=Pdgfra PE=1 SV=3 - [PGFRA_MOUSE]	-0.37	-0.30	-0.37	-0.31	0.00
Q922B2	Aspartate--tRNA ligase, cytoplasmic OS=Mus musculus GN=Dars PE=2 SV=2 - [SYDC_MOUSE]	0.20	0.20	0.25	0.23	0.00
P39447	Tight junction protein ZO-1 OS=Mus musculus GN=Tjp1 PE=1 SV=2 - [ZO1_MOUSE]	0.23	0.27	0.20	0.23	0.00
Q9DC23	DnaJ homolog subfamily C member 10 OS=Mus musculus GN=Dnajc10 PE=1 SV=2 - [DJC10_MOUSE]	0.84	0.66	0.79	0.67	0.00
Q61425	Hydroxyacyl-coenzyme A dehydrogenase, mitochondrial OS=Mus musculus GN=Hadh PE=1 SV=2 - [HCDH_MOUSE]	-0.56	-0.47	-0.58	-0.45	0.00

Q9D7P6	Iron-sulfur cluster assembly enzyme ISCU, mitochondrial OS=Mus musculus GN=Iscu PE=1 SV=1 - [ISCU_MOUSE]	-0.43	-0.45	-0.34	-0.44	0.00
O55074	A-kinase anchor protein 7 isoform alpha OS=Mus musculus GN=Akap7 PE=1 SV=4 - [AKA7A_MOUSE]	-0.56	-0.45	-0.57	-0.46	0.00
Q99PG0	Arylacetamide deacetylase OS=Mus musculus GN=Aadac PE=1 SV=3 - [AAAD_MOUSE]	1.01	0.78	0.95	1.03	0.00
Q62000	Mimecan OS=Mus musculus GN=Ogn PE=2 SV=1 - [MIME_MOUSE]	-0.27	-0.22	-0.27	-0.22	0.00
P61953	Guanine nucleotide-binding protein G(I)/G(S)/G(O) subunit gamma-11 OS=Mus musculus GN=Gng11 PE=1 SV=1 - [GBG11_MOUSE]	-0.65	-0.71	-0.53	-0.59	0.00
Q8VE47	Ubiquitin-like modifier-activating enzyme 5 OS=Mus musculus GN=Uba5 PE=2 SV=2 - [UBA5_MOUSE]	0.64	0.83	0.66	0.72	0.00
Q8BRF7	Sec1 family domain-containing protein 1 OS=Mus musculus GN=Scfd1 PE=2 SV=1 [SCFD1_MOUSE]	0.37	0.28	0.31	0.31	0.00
P51885	Lumican OS=Mus musculus GN=Lum PE=1 SV=2 - [LUM_MOUSE]	-0.52	-0.43	-0.57	-0.47	0.00
D3YVJ8	Retinol dehydrogenase 13 (Fragment) OS=Mus musculus GN=Rdh13 PE=2 SV=1 - [D3YVJ8_MOUSE]	0.25	0.26	0.21	0.20	0.00
O89001	Carboxypeptidase D OS=Mus musculus GN=Cpd PE=1 SV=2 - [CBPD_MOUSE]	0.22	0.20	0.26	0.22	0.00
P01868	Ig gamma-1 chain C region secreted form OS=Mus musculus GN=Ighg1 PE=1 SV=1 - [IGHG1_MOUSE]	-0.66	-0.80	-0.75	-0.88	0.00
Q9WTQ5-2	Isoform 2 of A-kinase anchor protein 12 OS=Mus musculus GN=Akap12 - [AKA12_MOUSE]	-0.43	-0.34	-0.45	-0.38	0.00
Q9JHZ2	Progressive ankylosis protein OS=Mus musculus GN=Ankh PE=2 SV=1 - [ANKH_MOUSE]	-0.58	-0.57	-0.47	-0.46	0.00
P28651	Carbonic anhydrase-related protein OS=Mus musculus GN=Ca8 PE=1 SV=5 - [CAH8_MOUSE]	-0.84	-0.62	-0.74	-0.74	0.00

Q9JHE3	Neutral ceramidase OS=Mus musculus GN=Asah2 PE=1 SV=1 - [ASAH2_MOUSE]	-0.79	-0.78	-0.61	-0.66	0.00
Q6DFW4	Nucleolar protein 58 OS=Mus musculus GN=Nop58 PE=1 SV=1 - [NOP58_MOUSE]	0.40	0.46	0.39	0.51	0.00
Q9D1M7	Peptidyl-prolyl cis-trans isomerase FKBP11 OS=Mus musculus GN=Fkbp11 PE=2 SV=1 - [FKB11_MOUSE]	0.78	0.70	0.63	0.58	0.00
Q9DBZ5	Eukaryotic translation initiation factor 3 subunit K OS=Mus musculus GN=Eif3k PE=1 SV=1 - [EIF3K_MOUSE]	0.56	0.71	0.55	0.60	0.00
E9PVU7	cAMP-specific 3',5'-cyclic phosphodiesterase 4D OS=Mus musculus GN=Pde4d PE=2 SV=1 - [E9PVU7_MOUSE]	-0.75	-0.91	-0.86	-1.02	0.00
E9PUZ8	C4b-binding protein (Fragment) OS=Mus musculus GN=C4bp PE=2 SV=1 - [E9PUZ8_MOUSE]	-0.42	-0.51	-0.55	-0.56	0.00
Q9EPB4	Apoptosis-associated speck-like protein containing a CARD OS=Mus musculus GN=Pycard PE=1 SV=1 - [ASC_MOUSE]	-0.23	-0.19	-0.26	-0.23	0.00
P61982	14-3-3 protein gamma OS=Mus musculus GN=Ywhag PE=1 SV=2 - [1433G_MOUSE]	-0.27	-0.26	-0.29	-0.34	0.00
P28667	MARCKS-related protein OS=Mus musculus GN=Marcksl1 PE=1 SV=2 - [MRP_MOUSE]	-0.50	-0.42	-0.45	-0.37	0.00
Q8VE70	Programmed cell death protein 10 OS=Mus musculus GN=Pdcd10 PE=1 SV=1 - [PDC10_MOUSE]	0.61	0.47	0.48	0.58	0.00
P84084	ADP-ribosylation factor 5 OS=Mus musculus GN=Arf5 PE=2 SV=2 - [ARF5_MOUSE]	0.34	0.29	0.34	0.39	0.00
P68373	Tubulin alpha-1C chain OS=Mus musculus GN=Tuba1c PE=1 SV=1 - [TBA1C_MOUSE]	0.29	0.37	0.33	0.39	0.00
Q9D4D5	Protein 4933414I15Rik OS=Mus musculus GN=4933414I15Rik PE=2 SV=1 - [Q9D4D5_MOUSE]	-0.79	-0.69	-0.93	-0.84	0.00

Q9R112	Sulfide:quinone oxidoreductase, mitochondrial OS=Mus musculus GN=Sqrdl PE=2 SV=3 - [SQRD_MOUSE]	-0.18	-0.17	-0.21	-0.15	0.00
Q8K4S1	1-phosphatidylinositol 4,5-bisphosphate phosphodiesterase epsilon-1 OS=Mus musculus GN=Plce1 PE=1 SV=3 - [PLCE1_MOUSE]	0.57	0.58	0.74	0.65	0.00
Q9ESK4	Inhibitor of growth protein 2 OS=Mus musculus GN=Ing2 PE=1 SV=3 - [ING2_MOUSE]	0.61	0.49	0.62	0.50	0.00
Q9CXS4	Centromere protein V OS=Mus musculus GN=Cenpv PE=2 SV=2 - [CENPV_MOUSE]	-0.68	-0.76	-0.59	-0.59	0.00
P46656	Adrenodoxin, mitochondrial OS=Mus musculus GN=Fdx1 PE=2 SV=1 - [ADX_MOUSE]	-0.32	-0.33	-0.42	-0.35	0.00
Q9Z1Q9	Valine--tRNA ligase OS=Mus musculus GN=Vars PE=2 SV=1 - [SYVC_MOUSE]	0.39	0.30	0.36	0.31	0.00
Q8BP40	Lysophosphatidic acid phosphatase type 6 OS=Mus musculus GN=Acp6 PE=2 SV=1 - [PPA6_MOUSE]	-0.62	-0.54	-0.58	-0.46	0.00
Q8R4C2	RUN and FYVE domain-containing protein 2 OS=Mus musculus GN=Rufy2 PE=1 SV=2 - [RUFY2_MOUSE]	0.79	0.98	0.81	1.00	0.00
G5E866	Splicing factor 3B subunit 1 OS=Mus musculus GN=Sf3b1 PE=4 SV=1 - [G5E866_MOUSE]	0.28	0.23	0.22	0.22	0.00
O55222	Integrin-linked protein kinase OS=Mus musculus GN=Ilk PE=1 SV=2 - [ILK_MOUSE]	0.57	0.61	0.49	0.66	0.00
Q6A099	MKIAA0248 protein (Fragment) OS=Mus musculus GN=Gbf1 PE=2 SV=1 - [Q6A099_MOUSE]	0.39	0.32	0.39	0.31	0.00
Q91VI7	Ribonuclease inhibitor OS=Mus musculus GN=Rnh1 PE=1 SV=1 - [RINI_MOUSE]	0.14	0.17	0.15	0.18	0.00
Q9D6Y7-2	Isoform 2 of Mitochondrial peptide methionine sulfoxide reductase OS=Mus musculus GN=Msra - [MSRA_MOUSE]	-0.43	-0.42	-0.40	-0.32	0.00
E9Q1Q9	Ketohexokinase OS=Mus musculus GN=Khk PE=2 SV=1 - [E9Q1Q9_MOUSE]	-0.83	-0.86	-0.64	-0.81	0.00
E9PY03	Protein Tstd1 OS=Mus musculus GN=Tstd1 PE=4 SV=1 - [E9PY03_MOUSE]	0.30	0.40	0.33	0.38	0.00

O08547	Vesicle-trafficking protein SEC22b OS=Mus musculus GN=Sec22b PE=1 SV=3 - [SC22B_MOUSE]	0.26	0.21	0.22	0.19	0.00
Q8K3E5-2	Isoform 2 of Jouberin OS=Mus musculus GN=Ahi1 - [AHI1_MOUSE]	0.22	0.18	0.23	0.19	0.00
F6YIY0	Protein YIPF3 (Fragment) OS=Mus musculus GN=Yipf3 PE=4 SV=1 - [F6YIY0_MOUSE]	0.31	0.39	0.31	0.30	0.00
Q9DBS5	Kinesin light chain 4 OS=Mus musculus GN=Klc4 PE=1 SV=1 - [KLC4_MOUSE]	0.30	0.39	0.32	0.33	0.00
P62717	60S ribosomal protein L18a OS=Mus musculus GN=Rpl18a PE=1 SV=1 - [RL18A_MOUSE]	0.25	0.18	0.21	0.22	0.00
Q6GU68	Immunoglobulin superfamily containing leucine-rich repeat protein OS=Mus musculus GN=Islr PE=1 SV=1 - [ISLR_MOUSE]	-0.37	-0.50	-0.42	-0.42	0.00
Q9CYK1	Tryptophan--tRNA ligase, mitochondrial OS=Mus musculus GN=Wars2 PE=2 SV=2 - [SYWM_MOUSE]	-0.32	-0.31	-0.24	-0.31	0.00
Q9CPX8	Cytochrome b-c1 complex subunit 10 OS=Mus musculus GN=Uqcr11 PE=2 SV=1 - [QCR10_MOUSE]	-0.46	-0.54	-0.40	-0.48	0.00
Q9JKF1	Ras GTPase-activating-like protein IQGAP1 OS=Mus musculus GN=Iqgap1 PE=1 SV=2 - [IQGA1_MOUSE]	0.34	0.40	0.31	0.41	0.00
Q91XA2	Golgi membrane protein 1 OS=Mus musculus GN=Golm1 PE=1 SV=2 - [GOLM1_MOUSE]	0.36	0.45	0.38	0.48	0.00
E9Q8N8	Electrogenic sodium bicarbonate cotransporter 1 OS=Mus musculus GN=Slc4a4 PE=2 SV=1 - [E9Q8N8_MOUSE]	-0.23	-0.17	-0.23	-0.22	0.00
P55302	Alpha-2-macroglobulin receptor-associated protein OS=Mus musculus GN=Lrpap1 PE=1 SV=1 - [AMRP_MOUSE]	-0.64	-0.50	-0.60	-0.50	0.00
P51655	Glypican-4 OS=Mus musculus GN=Gpc4 PE=2 SV=2 - [GPC4_MOUSE]	0.52	0.69	0.66	0.68	0.00

P62814	V-type proton ATPase subunit B, brain isoform OS=Mus musculus GN=Atp6v1b2 PE=1 SV=1 - [VATB2_MOUSE]	-0.20	-0.19	-0.19	-0.15	0.00
Q61033	Lamina-associated polypeptide 2, isoforms alpha/zeta OS=Mus musculus GN=Tmpo PE=1 SV=4 - [LAP2A_MOUSE]	0.27	0.26	0.32	0.24	0.00
Q8R5M8-5	Isoform 5 of Cell adhesion molecule 1 OS=Mus musculus GN=Cadm1 - [CADM1_MOUSE]	-0.56	-0.72	-0.63	-0.74	0.00
Q8R3G9	Tetraspanin-8 OS=Mus musculus GN=Tspan8 PE=2 SV=1 - [TSN8_MOUSE]	0.23	0.22	0.24	0.29	0.00
G3UW94	MCG14259, isoform CRA_b OS=Mus musculus GN=U2af1 PE=4 SV=1 - [G3UW94_MOUSE]	0.25	0.26	0.30	0.22	0.00
Q8R1T1-2	Isoform 2 of Charged multivesicular body protein 7 OS=Mus musculus GN=Chmp7 - [CHMP7_MOUSE]	0.24	0.32	0.25	0.25	0.00
P17095-1	Isoform HMG-Y of High mobility group protein HMG-I/HMG-Y OS=Mus musculus GN=Hmga1 - [HMGA1_MOUSE]	0.55	0.57	0.51	0.42	0.00
A2AKV7	Alpha-ketoglutarate-dependent dioxygenase alkB homolog 3 (Fragment) OS=Mus musculus GN=Alkbh3 PE=2 SV=1 - [A2AKV7_MOUSE]	0.36	0.42	0.43	0.49	0.00
Q62261-2	Isoform 2 of Spectrin beta chain, non-erythrocytic 1 OS=Mus musculus GN=Sptbn1 - [SPTB2_MOUSE]	-0.96	-1.10	-0.80	-0.95	0.00
Q8R2Z5	von Willebrand factor A domain-containing protein 1 OS=Mus musculus GN=Vwa1 PE=1 SV=1 - [VWA1_MOUSE]	-0.60	-0.74	-0.55	-0.64	0.00
O55029	Coatomer subunit beta' OS=Mus musculus GN=Copb2 PE=2 SV=2 - [COPB2_MOUSE]	0.20	0.23	0.18	0.23	0.00
Q99MR6-3	Isoform C of Serrate RNA effector molecule homolog OS=Mus musculus GN=Srrt - [SRRT_MOUSE]	0.22	0.18	0.24	0.23	0.00
A2A9W7	ADP-ribosylation factor-binding protein GGA3 OS=Mus musculus GN=Gga3 PE=2 SV=1 - [A2A9W7_MOUSE]	-2.04	-2.11	-1.63	-1.70	0.00

Q9JKA5	Cell surface A33 antigen OS=Mus musculus GN=Gpa33 PE=2 SV=2 - [GPA33_MOUSE]	0.62	0.76	0.58	0.61	0.00
Q3TJD7	PDZ and LIM domain protein 7 OS=Mus musculus GN=Pdlim7 PE=2 SV=1 - [PDLI7_MOUSE]	0.18	0.18	0.24	0.22	0.00
Q9JHW2	Omega-amidase NIT2 OS=Mus musculus GN=Nit2 PE=1 SV=1 - [NIT2_MOUSE]	-0.32	-0.24	-0.30	-0.27	0.00
P11352	Glutathione peroxidase 1 OS=Mus musculus GN=Gpx1 PE=1 SV=2 - [GPX1_MOUSE]	-0.66	-0.52	-0.64	-0.53	0.00
Q8K4Z5	Splicing factor 3A subunit 1 OS=Mus musculus GN=Sf3a1 PE=1 SV=1 - [SF3A1_MOUSE]	0.19	0.24	0.20	0.25	0.00
Q3V1M1	Immunoglobulin superfamily member 10 OS=Mus musculus GN=Igsf10 PE=2 SV=2 - [IGS10_MOUSE]	-0.34	-0.42	-0.37	-0.45	0.00
Q8BU30	Isoleucine--tRNA ligase, cytoplasmic OS=Mus musculus GN=Iars PE=2 SV=2 - [SYIC_MOUSE]	0.53	0.51	0.43	0.59	0.00
P19157	Glutathione S-transferase P 1 OS=Mus musculus GN=Gstp1 PE=1 SV=2 - [GSTP1_MOUSE]	-0.30	-0.23	-0.31	-0.30	0.00
Q9CY58	Plasminogen activator inhibitor 1 RNA-binding protein OS=Mus musculus GN=Serbp1 PE=1 SV=2 - [PAIRB_MOUSE]	0.27	0.31	0.24	0.31	0.00
Q99MQ4	Asporin OS=Mus musculus GN=Aspn PE=1 SV=1 - [ASPN_MOUSE]	-0.19	-0.17	-0.14	-0.15	0.00
Q9CQA3	Succinate dehydrogenase [ubiquinone] iron-sulfur subunit, mitochondrial OS=Mus musculus GN=Sdhb PE=1 SV=1 - [DHSB_MOUSE]	-0.25	-0.22	-0.30	-0.29	0.00
Q8K1M6-3	Isoform 3 of Dynamin-1 like protein OS=Mus musculus GN=Dnm1l - [DNM1L_MOUSE]	0.24	0.21	0.29	0.26	0.00
P27048	Small nuclear ribonucleoprotein-associated protein B OS=Mus musculus GN=Snrpb PE=1 SV=1 - [RSMB_MOUSE]	-0.13	-0.13	-0.11	-0.15	0.00
E9PY16	Protein Adap1 OS=Mus musculus GN=Adap1 PE=2 SV=1 - [E9PY16_MOUSE]	0.33	0.27	0.32	0.26	0.00

E9Q912	Protein Rap1gds1 OS=Mus musculus GN=Rap1gds1 PE=2 SV=1 - [E9Q912_MOUSE]	0.37	0.32	0.36	0.28	0.00
Q8R422	CD109 antigen OS=Mus musculus GN=Cd109 PE=2 SV=1 - [CD109_MOUSE]	-0.77	-0.65	-0.74	-0.57	0.00
D3Z6C3	40S ribosomal protein S3a OS=Mus musculus GN=Rps3a2 PE=3 SV=1 - [D3Z6C3_MOUSE]	0.36	0.42	0.30	0.36	0.00
G5E8G6	Myosin Vb, isoform CRA_c OS=Mus musculus GN=Myo5b PE=4 SV=1 - [G5E8G6_MOUSE]	0.29	0.26	0.29	0.22	0.00
Q60737	Casein kinase II subunit alpha OS=Mus musculus GN=Csnk2a1 PE=1 SV=2 - [CSK21_MOUSE]	-0.41	-0.38	-0.29	-0.37	0.00
Q9D8G5	Regenerating islet-derived protein 4 OS=Mus musculus GN=Reg4 PE=2 SV=1 - [REG4_MOUSE]	-0.34	-0.41	-0.31	-0.41	0.00
P01027	Complement C3 OS=Mus musculus GN=C3 PE=1 SV=3 - [CO3_MOUSE]	-0.39	-0.42	-0.34	-0.32	0.00
P21995	Embigin OS=Mus musculus GN=Emb PE=1 SV=2 - [EMB_MOUSE]	0.53	0.65	0.48	0.59	0.00
E9PZ00	Sulfated glycoprotein 1 OS=Mus musculus GN=Psap PE=2 SV=1 - [E9PZ00_MOUSE]	-0.38	-0.34	-0.47	-0.43	0.00
O70318	Band 4.1-like protein 2 OS=Mus musculus GN=Epb41l2 PE=1 SV=2 - [E41L2_MOUSE]	-0.35	-0.35	-0.26	-0.30	0.00
G5E829	MCG13663, isoform CRA_a OS=Mus musculus GN=Atp2b1 PE=3 SV=1 - [G5E829_MOUSE]	-0.37	-0.40	-0.33	-0.45	0.00
E9QMH7	Inhibitor of nuclear factor kappa-B kinase-interacting protein OS=Mus musculus GN=Ikbip PE=2 SV=1 - [E9QMH7_MOUSE]	-0.67	-0.65	-0.84	-0.82	0.00
P58242	Acid sphingomyelinase-like phosphodiesterase 3b OS=Mus musculus GN=Smpdl3b PE=1 SV=1 - [ASM3B_MOUSE]	-1.07	-1.04	-1.34	-1.31	0.00
Q9CW03	Structural maintenance of chromosomes protein 3 OS=Mus musculus GN=Smc3 PE=1 SV=2 - [SMC3_MOUSE]	0.31	0.39	0.34	0.42	0.00

P21447	Multidrug resistance protein 1A OS=Mus musculus GN=Abcb1a PE=1 SV=3 - [MDR1A_MOUSE]	-0.40	-0.43	-0.35	-0.32	0.00
Q6P5D3-2	Isoform 2 of Putative ATP-dependent RNA helicase DHX57 OS=Mus musculus GN=Dhx57 - [DHX57_MOUSE]	-0.82	-0.68	-0.73	-0.59	0.00
A6X940	Fermitin family homolog 2 (Fragment) OS=Mus musculus GN=Fermt2 PE=2 SV=1 - [A6X940_MOUSE]	0.98	1.24	0.97	0.95	0.00
Q8K3Z9	Nuclear envelope pore membrane protein POM 121 OS=Mus musculus GN=Pom121 PE=1 SV=2 - [PO121_MOUSE]	0.41	0.39	0.42	0.52	0.00
E9QP00	Transformer-2 protein homolog alpha OS=Mus musculus GN=Tra2a PE=2 SV=1 [E9QP00_MOUSE]	-0.47	-0.38	-0.50	-0.39	0.00
D6RFQ6	Mitochondrial fission regulator 1 OS=Mus musculus GN=Mtfr1 PE=2 SV=1 - [D6RFQ6_MOUSE]	0.83	0.87	0.66	0.70	0.00
O70400	PDZ and LIM domain protein 1 OS=Mus musculus GN=Pdlim1 PE=2 SV=4 - [PDLI1_MOUSE]	-0.21	-0.19	-0.22	-0.16	0.00
P99028	Cytochrome b-c1 complex subunit 6, mitochondrial OS=Mus musculus GN=Uqcrh PE=1 SV=2 - [QCR6_MOUSE]	-0.15	-0.11	-0.14	-0.16	0.00
Q08481	Platelet endothelial cell adhesion molecule OS=Mus musculus GN=Pecam1 PE=1 SV=1 - [PECA1_MOUSE]	-0.36	-0.38	-0.33	-0.44	0.00
Q9WV69-2	Isoform 2 of Dematin OS=Mus musculus GN=Dmtn - [DEMA_MOUSE]	-0.57	-0.45	-0.48	-0.43	0.00
P52293	Importin subunit alpha-1 OS=Mus musculus GN=Kpna2 PE=1 SV=2 - [IMA1_MOUSE]	0.53	0.59	0.42	0.53	0.00
F8VQ05	Protein Fryl OS=Mus musculus GN=Fryl PE=4 SV=1 - [F8VQ05_MOUSE]	0.54	0.64	0.46	0.55	0.00
Q9WVC8	Chloride anion exchanger OS=Mus musculus GN=Slc26a3 PE=2 SV=1 - [S26A3_MOUSE]	1.17	1.01	0.91	0.87	0.00

Q9D3W4	GPN-loop GTPase 3 OS=Mus musculus GN=Gpn3 PE=2 SV=1 - [GPN3_MOUSE]	0.53	0.42	0.52	0.41	0.00
Q8R173	Palmitoyltransferase ZDHHC3 OS=Mus musculus GN=Zdhhc3 PE=1 SV=1 - [ZDHC3_MOUSE]	0.51	0.48	0.41	0.38	0.00
D3YZY2	Protein Zc3h7a (Fragment) OS=Mus musculus GN=Zc3h7a PE=2 SV=1 - [D3YZY2_MOUSE]	0.31	0.34	0.40	0.42	0.00
G3X9T8	Ceruloplasmin OS=Mus musculus GN=Cp PE=4 SV=1 - [G3X9T8_MOUSE]	-0.67	-0.71	-0.54	-0.56	0.00
Q8BIL5	Protein Hook homolog 1 OS=Mus musculus GN=Hook1 PE=1 SV=2 - [HOOK1_MOUSE]	0.56	0.53	0.43	0.44	0.00
D3Z442	Uncharacterized protein OS=Mus musculus GN=Aamdc PE=2 SV=1 - [D3Z442_MOUSE]	-0.63	-0.46	-0.60	-0.61	0.00
A2A6M1	Vacuolar-sorting protein SNF8 OS=Mus musculus GN=Snf8 PE=2 SV=1 - [A2A6M1_MOUSE]	0.25	0.28	0.34	0.29	0.00
P57016	Ladinin-1 OS=Mus musculus GN=Lad1 PE=1 SV=1 - [LAD1_MOUSE]	-0.40	-0.30	-0.41	-0.37	0.00
A2AX52	Collagen alpha-4(VI) chain OS=Mus musculus GN=Col6a4 PE=1 SV=2 - [CO6A4_MOUSE]	-0.83	-0.77	-0.67	-0.62	0.00
Q99KQ4	Nicotinamide phosphoribosyltransferase OS=Mus musculus GN=Nampt PE=1 SV=1 - [NAMPT_MOUSE]	0.31	0.23	0.30	0.28	0.00
H7BX88	Carnitine O-acetyltransferase OS=Mus musculus GN=Crat PE=2 SV=1 - [H7BX88_MOUSE]	-0.33	-0.26	-0.35	-0.29	0.00
E9QMK9	Protein 9030617O03Rik OS=Mus musculus GN=9030617O03Rik PE=2 SV=1 - [E9QMK9_MOUSE]	-1.04	-0.93	-0.88	-0.75	0.00
Q01339	Beta-2-glycoprotein 1 OS=Mus musculus GN=Apoh PE=1 SV=1 - [APOH_MOUSE]	-0.62	-0.52	-0.51	-0.45	0.00
E9Q586	Dynactin subunit 1 OS=Mus musculus GN=Dctn1 PE=2 SV=1 [E9Q586_MOUSE]	0.36	0.33	0.29	0.27	0.00
Q9D8Z1	Activating signal cointegrator 1 complex subunit 1 OS=Mus musculus GN=Ascc1 PE=2 SV=1 - [ASCC1_MOUSE]	0.32	0.37	0.37	0.44	0.00

P62137	Serine/threonine-protein phosphatase PP1-alpha catalytic subunit OS=Mus musculus GN=Ppp1ca PE=1 SV=1 - [PP1A_MOUSE]	-0.18	-0.24	-0.18	-0.19	0.00
Q9DBL7	Bifunctional coenzyme A synthase OS=Mus musculus GN=Coasy PE=1 SV=2 - [COASY_MOUSE]	-0.30	-0.25	-0.33	-0.34	0.00
O89100	GRB2-related adaptor protein 2 OS=Mus musculus GN=Grap2 PE=1 SV=1 - [GRAP2_MOUSE]	-0.51	-0.51	-0.40	-0.40	0.00
P14602-2	Isoform B of Heat shock protein beta-1 OS=Mus musculus GN=Hspb1 - [HSPB1_MOUSE]	0.38	0.46	0.33	0.38	0.00
Q6PEE2	CBP80/20-dependent translation initiation factor OS=Mus musculus GN=Ctif PE=1 SV=2 - [CTIF_MOUSE]	-0.38	-0.46	-0.46	-0.54	0.00
Q9Z2Z6	Mitochondrial carnitine/acylcarnitine carrier protein OS=Mus musculus GN=Slc25a20 PE=1 SV=1 - [MCAT_MOUSE]	-0.45	-0.50	-0.44	-0.59	0.00
A2AJY2	Collagen alpha-1(XV) chain OS=Mus musculus GN=Col15a1 PE=2 SV=1 - [A2AJY2_MOUSE]	-0.48	-0.42	-0.58	-0.54	0.00
A2ACP1-2	Isoform 2 of Tetratricopeptide repeat protein 39A OS=Mus musculus GN=Ttc39a - [TT39A_MOUSE]	0.22	0.18	0.17	0.17	0.00
P51807	Dynein light chain Tctex type 1 OS=Mus musculus GN=Dynlt1 PE=1 SV=1 - [DYLT1_MOUSE]	0.39	0.35	0.32	0.28	0.00
Q8K183	Pyridoxal kinase OS=Mus musculus GN=Pdxk PE=1 SV=1 - [PDXK_MOUSE]	-0.46	-0.41	-0.57	-0.51	0.00
P15626	Glutathione S-transferase Mu 2 OS=Mus musculus GN=Gstm2 PE=1 SV=2 - [GSTM2_MOUSE]	0.27	0.35	0.26	0.28	0.00
Q61081	Hsp90 co-chaperone Cdc37 OS=Mus musculus GN=Cdc37 PE=2 SV=1 - [CDC37_MOUSE]	0.40	0.50	0.45	0.37	0.00
Q923D2	Flavin reductase (NADPH) OS=Mus musculus GN=Blvrb PE=2 SV=3 - [BLVRB_MOUSE]	-0.24	-0.27	-0.30	-0.22	0.00

Q501J6	Probable ATP-dependent RNA helicase DDX17 OS=Mus musculus GN=Ddx17 PE=2 SV=1 - [DDX17_MOUSE]	0.12	0.10	0.10	0.13	0.00
Q9D2M8	Ubiquitin-conjugating enzyme E2 variant 2 OS=Mus musculus GN=Ube2v2 PE=2 SV=4 - [UB2V2_MOUSE]	0.54	0.52	0.64	0.69	0.00
Q91VN0	Low-density lipoprotein receptor-related protein 5 OS=Mus musculus GN=Lrp5 PE=1 SV=3 - [LRP5_MOUSE]	1.10	0.92	0.97	0.79	0.00
Q6PIU9	Uncharacterized protein FLJ45252 homolog OS=Mus musculus PE=1 SV=2 - [YJ005_MOUSE]	-0.39	-0.33	-0.43	-0.32	0.00
Q8VEM8	Phosphate carrier protein, mitochondrial OS=Mus musculus GN=Slc25a3 PE=1 SV=1 - [MPCP_MOUSE]	0.14	0.12	0.11	0.15	0.00
Q07797	Galectin-3-binding protein OS=Mus musculus GN=Lgals3bp PE=1 SV=1 - [LG3BP_MOUSE]	-0.32	-0.36	-0.33	-0.43	0.00
Q8CG76	Aflatoxin B1 aldehyde reductase member 2 OS=Mus musculus GN=Akr7a2 PE=1 SV=3 - [ARK72_MOUSE]	-0.26	-0.20	-0.24	-0.21	0.00
Q9DC28-2	Isoform 2 of Casein kinase I isoform delta OS=Mus musculus GN=Csnk1d - [KC1D_MOUSE]	0.16	0.12	0.14	0.15	0.00
D3Z4T4	Sec1 family domain-containing protein 2 OS=Mus musculus GN=Scfd2 PE=2 SV=1 [D3Z4T4_MOUSE]	0.25	0.29	0.21	0.25	0.00
P70670	Nascent polypeptide-associated complex subunit alpha, muscle-specific form OS=Mus musculus GN=Naca PE=1 SV=2 - [NACAM_MOUSE]	0.12	0.17	0.16	0.15	0.00
P35486	Pyruvate dehydrogenase E1 component subunit alpha, somatic form, mitochondrial OS=Mus musculus GN=Pdha1 PE=1 SV=1 - [ODPA_MOUSE]	-0.24	-0.19	-0.23	-0.19	0.00
P00493	Hypoxanthine-guanine phosphoribosyltransferase OS=Mus musculus GN=Hprt1 PE=1 SV=3 - [HPRT_MOUSE]	0.21	0.27	0.20	0.25	0.00

O88851	Putative hydrolase RBBP9 OS=Mus musculus GN=Rbbp9 PE=1 SV=2 - [RBBP9_MOUSE]	0.65	0.63	0.50	0.51	0.00
D3YZY4	Retinoic acid receptor gamma (Fragment) OS=Mus musculus GN=Rarg PE=2 SV=1 - [D3YZY4_MOUSE]	0.25	0.25	0.19	0.19	0.00
D3Z141	Galectin-7 (Fragment) OS=Mus musculus GN=Lgals7 PE=2 SV=1 - [D3Z141_MOUSE]	-0.09	-0.08	-0.07	-0.07	0.00
Q9CXI0	2-methoxy-6-polyprenyl 1,4-benzoquinol methylase, mitochondrial OS=Mus musculus GN=Coq5 PE=2 SV=2 - [COQ5_MOUSE]	0.40	0.50	0.36	0.44	0.00
Q8C790	Protein FAM221A OS=Mus musculus GN=Fam221a PE=2 SV=1 - [F221A_MOUSE]	1.22	1.13	1.46	1.50	0.00
Q8BXN9	Transmembrane protein 87A OS=Mus musculus GN=Tmem87a PE=1 SV=1 - [TM87A_MOUSE]	0.59	0.58	0.67	0.77	0.00
Q02788	Collagen alpha-2(VI) chain OS=Mus musculus GN=Col6a2 PE=2 SV=3 - [CO6A2_MOUSE]	-0.33	-0.28	-0.38	-0.31	0.00
Q5SWZ5	Myosin phosphatase Rho-interacting protein OS=Mus musculus GN=Mprip PE=2 SV=1 - [Q5SWZ5_MOUSE]	-0.31	-0.28	-0.37	-0.38	0.00
P63001	Ras-related C3 botulinum toxin substrate 1 OS=Mus musculus GN=Rac1 PE=1 SV=1 - [RAC1_MOUSE]	0.30	0.24	0.27	0.33	0.00
Q9D7I9	Protein-glutamine gamma-glutamyltransferase 5 OS=Mus musculus GN=Tgm5 PE=2 SV=3 [TGM5_MOUSE]	-0.38	-0.42	-0.48	-0.52	0.00
Q6ZWQ5	Sorting nexin 12, isoform CRA_c OS=Mus musculus GN=Snx12 PE=2 SV=1 - [Q6ZWQ5_MOUSE]	0.39	0.48	0.43	0.54	0.00
P02463	Collagen alpha-1(IV) chain OS=Mus musculus GN=Col4a1 PE=2 SV=4 - [CO4A1_MOUSE]	-0.73	-0.84	-0.89	-1.02	0.00
Q8BLN5	Lanosterol synthase OS=Mus musculus GN=Lss PE=2 SV=2 - [ERG7_MOUSE]	0.49	0.58	0.46	0.42	0.00
Q9EPX2	Papilin OS=Mus musculus GN=Papln PE=2 SV=2 - [PPN_MOUSE]	-0.72	-0.91	-0.79	-0.99	0.00

G3UVU2	Splicing factor 3A subunit 2 OS=Mus musculus GN=Sf3a2 PE=4 SV=1 - [G3UVU2_MOUSE]	0.27	0.35	0.26	0.27	0.00
E9PX48	Dedicator of cytokinesis protein 7 OS=Mus musculus GN=Dock7 PE=2 SV=1 - [E9PX48_MOUSE]	0.56	0.64	0.46	0.54	0.00
Q8R1M8	Mucosal pentraxin OS=Mus musculus GN=Mptx1 PE=2 SV=1 - [MPTX_MOUSE]	2.81	2.27	2.67	2.08	0.00
Q3UW68	Calpain-13 OS=Mus musculus GN=Capn13 PE=2 SV=1 - [CAN13_MOUSE]	1.20	1.18	1.25	0.90	0.00
Q9DCG6	Phenazine biosynthesis like domain-containing protein 1 OS=Mus musculus GN=Pbld1 PE=2 SV=2 - [PBLD1_MOUSE]	-1.06	-0.83	-1.13	-0.90	0.00
P80313	T-complex protein 1 subunit eta OS=Mus musculus GN=Cct7 PE=1 SV=1 - [TCPH_MOUSE]	0.22	0.19	0.22	0.16	0.00
Q8BMD5-3	Isoform 3 of Cytidine and dCMP deaminase domain-containing protein 1 OS=Mus musculus GN=Cdadc1 - [CDAC1_MOUSE]	-0.78	-0.68	-0.65	-0.55	0.00
P62242	40S ribosomal protein S8 OS=Mus musculus GN=Rps8 PE=1 SV=2 - [RS8_MOUSE]	0.20	0.24	0.20	0.27	0.00
H3BJP2	S-formylglutathione hydrolase (Fragment) OS=Mus musculus GN=Esd PE=2 SV=1 - [H3BJP2_MOUSE]	-0.20	-0.21	-0.22	-0.27	0.00
Q9Z0F7	Gamma-synuclein OS=Mus musculus GN=Sncg PE=1 SV=1 - [SYUG_MOUSE]	-0.20	-0.20	-0.21	-0.26	0.00
Q3V3W4	Protein Zbtb2 OS=Mus musculus GN=Zbtb2 PE=2 SV=1 - [Q3V3W4_MOUSE]	-0.77	-0.63	-0.69	-0.55	0.00
P28076	Proteasome subunit beta type-9 OS=Mus musculus GN=Psmb9 PE=1 SV=1 - [PSB9_MOUSE]	-0.49	-0.54	-0.61	-0.68	0.00
Q3TC72	Fumarylacetoacetate hydrolase domain-containing protein 2A OS=Mus musculus GN=Fahd2 PE=1 SV=1 - [FAHD2_MOUSE]	-0.45	-0.42	-0.54	-0.55	0.00
P47809	Dual specificity mitogen activated protein kinase kinase 4 OS=Mus musculus GN=Map2k4 PE=1 SV=2 - [MP2K4_MOUSE]	0.43	0.34	0.39	0.48	0.00

P54276	DNA mismatch repair protein Msh6 OS=Mus musculus GN=Msh6 PE=1 SV=3 - [MSH6_MOUSE]	0.29	0.27	0.34	0.36	0.00
P11087-2	Isoform 2 of Collagen alpha-1(I) chain OS=Mus musculus GN=Col1a1 - [CO1A1_MOUSE]	-0.52	-0.44	-0.56	-0.42	0.00
Q9CPW5	Translocon-associated protein subunit beta OS=Mus musculus GN=Ssr2 PE=1 SV=1 - [SSRB_MOUSE]	1.86	2.22	1.57	1.93	0.00
Q9WUB7	Chloride channel protein ClC-Ka OS=Mus musculus GN=Clcnka PE=1 SV=2 - [CLCKA_MOUSE]	-0.68	-0.57	-0.80	-0.69	0.00
Q04750	DNA topoisomerase 1 OS=Mus musculus GN=Top1 PE=1 SV=2 - [TOP1_MOUSE]	0.29	0.37	0.26	0.31	0.00
E9PV60	Protein Wdfy4 OS=Mus musculus GN=Wdfy4 PE=2 SV=1 - [E9PV60_MOUSE]	0.21	0.23	0.28	0.28	0.00
O88329	Unconventional myosin-Ia OS=Mus musculus GN=Myo1a PE=2 SV=2 - [MYO1A_MOUSE]	0.26	0.21	0.25	0.19	0.00
E9PWK4	Immunoglobulin J chain (Fragment) OS=Mus musculus GN=Igj PE=2 SV=1 - [E9PWK4_MOUSE]	0.43	0.31	0.43	0.39	0.00
O89051	Integral membrane protein 2B OS=Mus musculus GN=Itm2b PE=2 SV=1 - [ITM2B_MOUSE]	0.48	0.55	0.41	0.57	0.00
Q8R2L5	28S ribosomal protein S18c, mitochondrial OS=Mus musculus GN=Mrps18c PE=2 SV=1 - [RT18C_MOUSE]	1.14	1.40	1.01	1.27	0.00
Q9CXE7	Transmembrane emp24 domain-containing protein 5 OS=Mus musculus GN=Tmed5 PE=2 SV=1 - [TMED5_MOUSE]	0.53	0.57	0.46	0.42	0.00
Q91YE8	Synaptopodin-2 OS=Mus musculus GN=Synpo2 PE=1 SV=2 - [SYNP2_MOUSE]	2.25	1.77	2.17	1.69	0.00
P43137	Lithostathine-1 OS=Mus musculus GN=Reg1 PE=2 SV=1 - [LIT1_MOUSE]	1.47	1.94	1.45	1.75	0.00
Q61171	Peroxiredoxin-2 OS=Mus musculus GN=Prdx2 PE=1 SV=3 [PRDX2_MOUSE]	-0.25	-0.22	-0.29	-0.30	0.00

E9Q4U7	Protein diaphanous homolog 2 OS=Mus musculus GN=Diap2 PE=2 SV=1 - [E9Q4U7_MOUSE]	0.41	0.38	0.51	0.38	0.00
P39749	Flap endonuclease 1 OS=Mus musculus GN=Fen1 PE=1 SV=1 - [FEN1_MOUSE]	0.33	0.44	0.33	0.36	0.00
O88569	Heterogeneous nuclear ribonucleoproteins A2/B1 OS=Mus musculus GN=Hnrnpa2b1 PE=1 SV=2 - [ROA2_MOUSE]	-0.27	-0.22	-0.30	-0.23	0.00
P29391	Ferritin light chain 1 OS=Mus musculus GN=Ftl1 PE=1 SV=2 - [FRIL1_MOUSE]	-1.56	-1.25	-1.79	-1.57	0.00
Q8BUH2	Nucleoside diphosphate kinase 7 OS=Mus musculus GN=Nme7 PE=2 SV=1 - [Q8BUH2_MOUSE]	-0.39	-0.45	-0.32	-0.37	0.00
Q00898	Alpha-1-antitrypsin 1-5 OS=Mus musculus GN=Serpina1e PE=1 SV=1 - [A1AT5_MOUSE]	-1.58	-2.01	-2.22	-2.12	0.00
P62196	26S protease regulatory subunit 8 OS=Mus musculus GN=Psmc5 PE=1 SV=1 - [PRS8_MOUSE]	0.43	0.35	0.44	0.33	0.00
P49718	DNA replication licensing factor MCM5 OS=Mus musculus GN=Mcm5 PE=2 SV=1 - [MCM5_MOUSE]	0.33	0.24	0.27	0.27	0.00
Q8CGY8-2	Isoform 2 of UDP-N-acetylglucosamine--peptide N-acetylglucosaminyltransferase 110 kDa subunit OS=Mus musculus GN=Ogt - [OGT1_MOUSE]	0.16	0.17	0.14	0.13	0.00
Q5DTY9	BTB/POZ domain-containing protein KCTD16 OS=Mus musculus GN=Kctd16 PE=1 SV=2 - [KCD16_MOUSE]	-1.68	-1.38	-1.50	-1.20	0.00
D3YYS9	Acid-sensing ion channel 4 OS=Mus musculus GN=Asic4 PE=2 SV=1 - [D3YYS9_MOUSE]	1.06	0.95	0.86	0.75	0.00
Q8BWT1	3-ketoacyl-CoA thiolase, mitochondrial OS=Mus musculus GN=Acaa2 PE=1 SV=3 - [THIM_MOUSE]	-0.38	-0.32	-0.45	-0.40	0.00
O89114	DnaJ homolog subfamily B member 5 OS=Mus musculus GN=Dnajb5 PE=2 SV=1 - [DNJB5_MOUSE]	0.47	0.54	0.38	0.47	0.00

Q8BJ64	Choline dehydrogenase, mitochondrial OS=Mus musculus GN=Chdh PE=1 SV=1 - [CHDH_MOUSE]	0.24	0.20	0.24	0.28	0.00
P09055	Integrin beta-1 OS=Mus musculus GN=Itgb1 PE=1 SV=1 - [ITB1_MOUSE]	0.10	0.14	0.11	0.13	0.00
Q5NC83	Uncharacterized protein C7orf72 homolog OS=Mus musculus PE=2 SV=2 - [CG072_MOUSE]	-0.50	-0.45	-0.41	-0.36	0.00
Q9JHU4	Cytoplasmic dynein 1 heavy chain 1 OS=Mus musculus GN=Dync1h1 PE=1 SV=2 - [DYHC1_MOUSE]	0.33	0.25	0.34	0.28	0.00
P63085	Mitogen-activated protein kinase 1 OS=Mus musculus GN=Mapk1 PE=1 SV=3 - [MK01_MOUSE]	-0.21	-0.24	-0.18	-0.18	0.00
P49817	Caveolin-1 OS=Mus musculus GN=Cav1 PE=1 SV=1 - [CAV1_MOUSE]	-0.70	-0.66	-0.90	-0.72	0.00
Q8BLV3-2	Isoform 2 of Sodium/hydrogen exchanger 7 OS=Mus musculus GN=Slc9a7 - [SL9A7_MOUSE]	-0.47	-0.57	-0.57	-0.67	0.00
Q61753	D-3-phosphoglycerate dehydrogenase OS=Mus musculus GN=Phgdh PE=1 SV=3 - [SERA_MOUSE]	0.55	0.60	0.42	0.51	0.00
Q9WV54	Acid ceramidase OS=Mus musculus GN=Asah1 PE=1 SV=1 - [ASAH1_MOUSE]	-0.33	-0.39	-0.40	-0.47	0.00
P97311	DNA replication licensing factor MCM6 OS=Mus musculus GN=Mcm6 PE=1 SV=1 - [MCM6_MOUSE]	0.40	0.46	0.35	0.49	0.00
Q8BGL5	Protein C730034F03Rik OS=Mus musculus GN=C730034F03Rik PE=4 SV=1 - [Q8BGL5_MOUSE]	-0.74	-0.87	-0.61	-0.75	0.00
P62267	40S ribosomal protein S23 OS=Mus musculus GN=Rps23 PE=2 SV=3 - [RS23_MOUSE]	0.47	0.38	0.39	0.52	0.00
Q03173-6	Isoform 6 of Protein enabled homolog OS=Mus musculus GN=Enah - [ENAH_MOUSE]	0.74	0.89	0.62	0.77	0.00
P67778	Prohibitin OS=Mus musculus GN=Phb PE=1 SV=1 - [PHB_MOUSE]	0.05	0.04	0.04	0.04	0.00

Q9CQJ2	PIH1 domain-containing protein 1 OS=Mus musculus GN=Pih1d1 PE=2 SV=1 - [PIHD1_MOUSE]	-1.42	-1.11	-1.51	-1.19	0.00
D3YZD3	Actin filament-associated protein 1-like 2 (Fragment) OS=Mus musculus GN=Afap1l2 PE=2 SV=1 - [D3YZD3_MOUSE]	0.31	0.36	0.39	0.44	0.00
B0R091	Protein Gm20056 OS=Mus musculus GN=Chp1 PE=2 SV=1 - [B0R091_MOUSE]	-0.61	-0.46	-0.61	-0.49	0.00
F7CVL9	Beclin-1 (Fragment) OS=Mus musculus GN=Becn1 PE=2 SV=1 - [F7CVL9_MOUSE]	3.04	3.45	2.43	2.83	0.00
Q8CGN5	Perilipin-1 OS=Mus musculus GN=Plin1 PE=1 SV=2 - [PLIN1_MOUSE]	-2.29	-2.09	-1.86	-1.63	0.00
P60867	40S ribosomal protein S20 OS=Mus musculus GN=Rps20 PE=1 SV=1 - [RS20_MOUSE]	0.43	0.38	0.53	0.43	0.00
E9Q6J6	Protein Psg20 OS=Mus musculus GN=Psg20 PE=2 SV=1 - [E9Q6J6_MOUSE]	-0.94	-1.00	-1.21	-1.27	0.00
Q8BHB9	Chloride intracellular channel protein 6 OS=Mus musculus GN=Clic6 PE=2 SV=1 - [CLIC6_MOUSE]	0.54	0.40	0.42	0.50	0.00
Q61749	Translation initiation factor eIF-2B subunit delta OS=Mus musculus GN=Eif2b4 PE=2 SV=2 - [EI2BD_MOUSE]	0.46	0.32	0.40	0.41	0.00
Q8BZF8	Phosphoglucomutase-like protein 5 OS=Mus musculus GN=Pgm5 PE=1 SV=2 - [PGM5_MOUSE]	-0.13	-0.10	-0.11	-0.14	0.00
B1AY13	Ubiquitin carboxyl-terminal hydrolase 24 OS=Mus musculus GN=Usp24 PE=1 SV=1 - [UBP24_MOUSE]	-0.87	-1.06	-1.21	-0.94	0.00
Q8R1F1	Niban-like protein 1 OS=Mus musculus GN=Fam129b PE=1 SV=2 - [NIBL1_MOUSE]	0.41	0.53	0.43	0.55	0.00
Q8K3S0	MCG55901 OS=Mus musculus GN=4930519F16Rik PE=3 SV=1 - [Q8K3S0_MOUSE]	1.18	1.09	0.94	0.85	0.00
E9PUF4	RNA-binding protein 26 OS=Mus musculus GN=Rbm26 PE=2 SV=1 - [E9PUF4_MOUSE]	-0.14	-0.16	-0.11	-0.14	0.00

Q9ERU3	Zinc finger protein 22 OS=Mus musculus GN=Znf22 PE=1 SV=2 [ZNF22_MOUSE]	-0.41	-0.32	-0.40	-0.31	0.00
Q9JI18	Low-density lipoprotein receptor-related protein 1B OS=Mus musculus GN=Lrp1b PE=2 SV=1 [LRP1B_MOUSE]	-0.56	-0.51	-0.70	-0.65	0.00
E9QLD5	Serine protease 29 OS=Mus musculus GN=Prss29 PE=3 SV=1 - [E9QLD5_MOUSE]	0.43	0.50	0.35	0.42	0.00
P15535-2	Isoform Short of Beta-1,4-galactosyltransferase 1 OS=Mus musculus GN=B4galt1 - [B4GT1_MOUSE]	0.34	0.42	0.42	0.49	0.00
Q6NSR8	Probable aminopeptidase NPEPL1 OS=Mus musculus GN=Npepl1 PE=2 SV=1 - [PEPL1_MOUSE]	-0.15	-0.11	-0.15	-0.14	0.00
Q91YN9	BAG family molecular chaperone regulator 2 OS=Mus musculus GN=Bag2 PE=1 SV=1 - [BAG2_MOUSE]	0.28	0.26	0.35	0.34	0.00
Q8BIJ6	Isoleucine--tRNA ligase, mitochondrial OS=Mus musculus GN=Iars2 PE=2 SV=1 - [SYIM_MOUSE]	-0.14	-0.17	-0.14	-0.19	0.00
P61290	Proteasome activator complex subunit 3 OS=Mus musculus GN=Psme3 PE=1 SV=1 - [PSME3_MOUSE]	0.35	0.43	0.34	0.46	0.00
Q8C1Y8	Vacuolar fusion protein CCZ1 homolog OS=Mus musculus GN=Ccz1 PE=1 SV=1 - [CCZ1_MOUSE]	-0.51	-0.58	-0.41	-0.48	0.00
Q8CH09	SURP and G-patch domain-containing protein 2 OS=Mus musculus GN=Sugp2 PE=2 SV=2 - [SUGP2_MOUSE]	0.39	0.48	0.48	0.56	0.00
Q91WE4	UPF0729 protein C18orf32 homolog OS=Mus musculus PE=2 SV=1 - [CR032_MOUSE]	0.46	0.58	0.42	0.55	0.00
O08582	GTP-binding protein 1 OS=Mus musculus GN=Gtpbp1 PE=1 SV=2 - [GTPB1_MOUSE]	0.31	0.23	0.26	0.23	0.00
O35074	Prostacyclin synthase OS=Mus musculus GN=Ptgis PE=2 SV=1 - [PTGIS_MOUSE]	-0.55	-0.65	-0.54	-0.73	0.00
O09117-2	Isoform 2 of Synaptophysin-like protein 1 OS=Mus musculus GN=Sypl1 - [SYPL1_MOUSE]	0.47	0.63	0.48	0.60	0.00

Q9WVM8	Kynurenine/alpha-aminoadipate aminotransferase, mitochondrial OS=Mus musculus GN=Aadat PE=2 SV=1 - [AADAT_MOUSE]	0.40	0.52	0.46	0.57	0.00
E9QLB2	Lysophospholipase-like protein 1 OS=Mus musculus GN=Lyplal1 PE=4 SV=1 - [E9QLB2_MOUSE]	-0.44	-0.44	-0.34	-0.34	0.00
Q8K1E0	Syntaxin-5 OS=Mus musculus GN=Stx5 PE=1 SV=3 - [STX5_MOUSE]	0.46	0.45	0.61	0.56	0.00
Q9CXJ1	Probable glutamate--tRNA ligase, mitochondrial OS=Mus musculus GN=Ears2 PE=2 SV=1 - [SYEM_MOUSE]	0.61	0.47	0.55	0.44	0.00
Q9CZ96-2	Isoform 2 of Zinc finger CCHC-type and RNA-binding motif-containing protein 1 OS=Mus musculus GN=Zcrb1 - [ZCRB1_MOUSE]	-0.17	-0.22	-0.18	-0.24	0.00
Q8VCX5	Calcium uptake protein 1, mitochondrial OS=Mus musculus GN=Micu1 PE=1 SV=1 - [MICU1_MOUSE]	0.29	0.37	0.26	0.33	0.00
O70172	Phosphatidylinositol 5-phosphate 4-kinase type-2 alpha OS=Mus musculus GN=Pip4k2a PE=1 SV=1 - [PI42A_MOUSE]	-0.18	-0.20	-0.26	-0.21	0.00
E9PVL3	Protein BC094916 OS=Mus musculus GN=BC094916 PE=2 SV=1 - [E9PVL3_MOUSE]	-0.79	-0.72	-0.99	-0.93	0.00
G3XA21	MCG134445, isoform CRA_a OS=Mus musculus GN=Mroh1 PE=4 SV=1 - [G3XA21_MOUSE]	0.29	0.33	0.37	0.41	0.00
F6V9H6	Protein Muc3 (Fragment) OS=Mus musculus GN=Muc3 PE=4 SV=1 - [F6V9H6_MOUSE]	0.55	0.73	0.53	0.60	0.00
P01878	Ig alpha chain C region OS=Mus musculus PE=1 SV=1 - [IGHA_MOUSE]	0.59	0.76	0.57	0.74	0.00
Q4V9V3	Elongation of very long chain fatty acids (FEN1/Elo2, SUR4/Elo3, yeast)-like 1 OS=Mus musculus GN=Elovl1 PE=2 SV=1 - [Q4V9V3_MOUSE]	0.58	0.46	0.44	0.43	0.00

P70232-2	Isoform 2 of Neural cell adhesion molecule L1-like protein OS=Mus musculus GN=Chl1 - [CHL1_MOUSE]	-0.70	-0.49	-0.65	-0.64	0.00
Q8R0K2	E3 ubiquitin-protein ligase TRIM31 OS=Mus musculus GN=Trim31 PE=1 SV=1 - [TRI31_MOUSE]	0.29	0.30	0.21	0.25	0.00
Q921X9	Protein disulfide-isomerase A5 OS=Mus musculus GN=Pdia5 PE=2 SV=1 - [PDIA5_MOUSE]	0.45	0.63	0.51	0.49	0.00
Q8R059	UDP-glucose 4-epimerase OS=Mus musculus GN=Gale PE=2 SV=1 - [GALE_MOUSE]	0.35	0.38	0.45	0.48	0.00
P58021	Transmembrane 9 superfamily member 2 OS=Mus musculus GN=Tm9sf2 PE=2 SV=1 - [TM9S2_MOUSE]	0.25	0.27	0.31	0.22	0.00
Q9CQP0	39S ribosomal protein L33, mitochondrial OS=Mus musculus GN=Mrpl33 PE=2 SV=1 - [RM33_MOUSE]	0.47	0.60	0.46	0.59	0.00
Q9D6E4	Tetratricopeptide repeat protein 9B OS=Mus musculus GN=Ttc9b PE=2 SV=1 - [TTC9B_MOUSE]	0.19	0.25	0.22	0.27	0.00
Q99JF5	Diphosphomevalonate decarboxylase OS=Mus musculus GN=Mvd PE=1 SV=2 - [MVD1_MOUSE]	0.30	0.44	0.39	0.39	0.00
Q8BH58	TIP41-like protein OS=Mus musculus GN=Tiprl PE=2 SV=1 - [TIPRL_MOUSE]	0.25	0.18	0.20	0.19	0.00
O55142	60S ribosomal protein L35a OS=Mus musculus GN=Rpl35a PE=2 SV=2 - [RL35A_MOUSE]	0.18	0.22	0.15	0.20	0.00
Q8CFZ0	SUMO-conjugating enzyme UBC9 OS=Mus musculus GN=Ube2i PE=4 SV=1 [Q8CFZ0_MOUSE]	0.44	0.47	0.33	0.41	0.00
Q9EPT5	Solute carrier organic anion transporter family member 2A1 OS=Mus musculus GN=Slco2a1 PE=1 SV=2 - [SO2A1_MOUSE]	0.59	0.57	0.46	0.44	0.00
P60487	Pyridoxal phosphate phosphatase OS=Mus musculus GN=Pdxp PE=1 SV=1 - [PLPP_MOUSE]	-0.29	-0.22	-0.29	-0.23	0.00

Q9D1A2	Cytosolic non-specific dipeptidase OS=Mus musculus GN=Cndp2 PE=1 SV=1 - [CNDP2_MOUSE]	-0.21	-0.25	-0.26	-0.30	0.00
P62311	U6 snRNA-associated Sm-like protein LSm3 OS=Mus musculus GN=Lsm3 PE=3 SV=2 - [LSM3_MOUSE]	-0.24	-0.20	-0.25	-0.18	0.00
Q8BKC5-2	Isoform 2 of Importin-5 OS=Mus musculus GN=Ipo5 - [IPO5_MOUSE]	0.67	0.68	0.51	0.53	0.00
Q6PDN3-3	Isoform 3 of Myosin light chain kinase, smooth muscle OS=Mus musculus GN=Mylk - [MYLK_MOUSE]	0.40	0.49	0.34	0.41	0.00
O54734	Dolichyl-diphosphooligosaccharide--protein glycosyltransferase 48 kDa subunit OS=Mus musculus GN=Ddost PE=1 SV=2 - [OST48_MOUSE]	0.32	0.41	0.29	0.35	0.00
P22892	AP-1 complex subunit gamma-1 OS=Mus musculus GN=Ap1g1 PE=1 SV=3 - [AP1G1_MOUSE]	0.35	0.44	0.33	0.33	0.00
P35564	Calnexin OS=Mus musculus GN=Canx PE=1 SV=1 - [CALX_MOUSE]	0.37	0.38	0.27	0.32	0.00
J3QMK2	MCG22959, isoform CRA_a OS=Mus musculus GN=Bbip1 PE=4 SV=1 - [J3QMK2_MOUSE]	-0.50	-0.41	-0.43	-0.34	0.00
P01872	Ig mu chain C region secreted form OS=Mus musculus GN=Igh-6 PE=1 SV=2 - [IGHM_MOUSE]	-0.71	-0.77	-0.85	-1.00	0.00
A2A4L0	Pleckstrin homology domain-containing family H member 3 (Fragment) OS=Mus musculus GN=Plekhh3 PE=2 SV=1 - [A2A4L0_MOUSE]	-0.36	-0.28	-0.33	-0.25	0.00
Q8BGE6	Cysteine protease ATG4B OS=Mus musculus GN=Atg4b PE=1 SV=2 - [ATG4B_MOUSE]	0.93	1.15	0.80	1.01	0.00
P09813	Apolipoprotein A-II OS=Mus musculus GN=Apoa2 PE=1 SV=2 - [APOA2_MOUSE]	-0.18	-0.26	-0.24	-0.21	0.00
Q80V62	Fanconi anemia group D2 protein homolog OS=Mus musculus GN=Fancd2 PE=1 SV=2 - [FACD2_MOUSE]	0.79	0.84	0.79	1.06	0.00
Q8CJ00-2	Isoform 2 of NADPH oxidase activator 1 OS=Mus musculus GN=Noxa1 - [NOXA1_MOUSE]	0.72	0.61	0.77	0.55	0.00

E9PWB1	Pleckstrin homology-like domain family B member 1 OS=Mus musculus GN=Phldb1 PE=2 SV=1 - [E9PWB1_MOUSE]	-0.36	-0.25	-0.33	-0.30	0.00
Q8C078	Calcium/calmodulin-dependent protein kinase kinase 2 OS=Mus musculus GN=Camkk2 PE=1 SV=2 - [KKCC2_MOUSE]	0.84	1.10	0.84	1.10	0.00
Q91WP6	Serine protease inhibitor A3N OS=Mus musculus GN=Serpina3n PE=1 SV=1 - [SPA3N_MOUSE]	0.88	0.91	0.67	0.70	0.00
F6YRQ2	DnaJ homolog subfamily B member 6 (Fragment) OS=Mus musculus GN=Dnajb6 PE=4 SV=1 - [F6YRQ2_MOUSE]	0.81	0.64	0.74	0.57	0.00
Q9DBG9	Tax1-binding protein 3 OS=Mus musculus GN=Tax1bp3 PE=1 SV=1 - [TX1B3_MOUSE]	-0.34	-0.25	-0.32	-0.36	0.00
P14069	Protein S100-A6 OS=Mus musculus GN=S100a6 PE=1 SV=3 - [S10A6_MOUSE]	0.25	0.33	0.24	0.29	0.00
D6RIQ3	Phytanoyl-CoA dioxygenase domain-containing protein 1 OS=Mus musculus GN=Phyhd1 PE=2 SV=1 - [D6RIQ3_MOUSE]	-0.18	-0.20	-0.22	-0.25	0.00
B2RQY6	BC035947 protein OS=Mus musculus GN=BC035947 PE=2 SV=1 - [B2RQY6_MOUSE]	0.73	0.58	0.85	0.69	0.00
Q00519	Xanthine dehydrogenase/oxidase OS=Mus musculus GN=Xdh PE=1 SV=5 - [XDH_MOUSE]	-0.50	-0.37	-0.47	-0.38	0.00
Q99K73	Nrf1 protein OS=Mus musculus GN=Nrf1 PE=2 SV=1 - [Q99K73_MOUSE]	0.40	0.31	0.44	0.35	0.00
F6TQW2	Protein Ighg2c OS=Mus musculus GN=Ighg2c PE=4 SV=1 - [F6TQW2_MOUSE]	-1.35	-1.40	-0.98	-1.23	0.00
Q9CZR2	N-acetylated-alpha-linked acidic dipeptidase 2 OS=Mus musculus GN=Naalad2 PE=1 SV=2 - [NALD2_MOUSE]	-0.68	-0.87	-0.80	-0.98	0.00
Q9D9J3	Actin-related protein T1 OS=Mus musculus GN=Actrt1 PE=2 SV=1 [ACTT1_MOUSE]	0.48	0.58	0.40	0.49	0.00

Q3UPR7	Transmembrane protein 102 OS=Mus musculus GN=Tmem102 PE=1 SV=2 - [TM102_MOUSE]	0.20	0.27	0.20	0.26	0.00
P28660-2	Isoform 2 of Nck-associated protein 1 OS=Mus musculus GN=Nckap1 - [NCKP1_MOUSE]	0.30	0.38	0.36	0.44	0.00
P01831	Thy-1 membrane glycoprotein OS=Mus musculus GN=Thy1 PE=1 SV=1 - [THY1_MOUSE]	-1.13	-0.88	-1.26	-1.01	0.00
Q8C048	Protein Atp2b3 OS=Mus musculus GN=Atp2b3 PE=2 SV=1 - [Q8C048_MOUSE]	-0.61	-0.52	-0.76	-0.68	0.00
Q8JZK9	Hydroxymethylglutaryl-CoA synthase, cytoplasmic OS=Mus musculus GN=Hmgcs1 PE=1 SV=1 - [HMCS1_MOUSE]	0.71	0.53	0.76	0.75	0.00
Q8VHE0	Translocation protein SEC63 homolog OS=Mus musculus GN=Sec63 PE=1 SV=4 - [SEC63_MOUSE]	0.25	0.19	0.17	0.21	0.00
Q9ERG0	LIM domain and actin-binding protein 1 OS=Mus musculus GN=Lima1 PE=1 SV=3 - [LIMA1_MOUSE]	-0.10	-0.08	-0.11	-0.12	0.00
Q9JMH6	Thioredoxin reductase 1, cytoplasmic OS=Mus musculus GN=Txnrd1 PE=1 SV=3 - [TRXR1_MOUSE]	-0.49	-0.49	-0.35	-0.43	0.00
P51432	1-phosphatidylinositol 4,5-bisphosphate phosphodiesterase beta-3 OS=Mus musculus GN=Plcb3 PE=2 SV=2 - [PLCB3_MOUSE]	-0.26	-0.34	-0.24	-0.28	0.00
Q6ZWQ7	Signal peptidase complex subunit 3 OS=Mus musculus GN=Spcs3 PE=2 SV=1 - [Q6ZWQ7_MOUSE]	0.34	0.25	0.33	0.26	0.00
G3X925	Pyruvate kinase OS=Mus musculus GN=Pklr PE=3 SV=1 - [G3X925_MOUSE]	-0.51	-0.38	-0.53	-0.43	0.00
P17439	Glucosylceramidase OS=Mus musculus GN=Gba PE=1 SV=1 - [GLCM_MOUSE]	-0.59	-0.53	-0.55	-0.41	0.00
Q8K0E8	Fibrinogen beta chain OS=Mus musculus GN=Fgb PE=2 SV=1 - [FIBB_MOUSE]	-0.34	-0.35	-0.25	-0.28	0.00
P62488	DNA-directed RNA polymerase II subunit RPB7 OS=Mus musculus GN=Polr2g PE=2 SV=1 - [RPB7_MOUSE]	-0.52	-0.62	-0.42	-0.53	0.00

F8VPN4	Protein Agl OS=Mus musculus GN=Agl PE=4 SV=1 - [F8VPN4_MOUSE]	-0.52	-0.48	-0.36	-0.51	0.00
P28653	Biglycan OS=Mus musculus GN=Bgn PE=2 SV=1 - [PGS1_MOUSE]	-0.10	-0.14	-0.14	-0.12	0.00
Q3UW53	Protein Niban OS=Mus musculus GN=Fam129a PE=1 SV=2 - [NIBAN_MOUSE]	0.20	0.19	0.25	0.18	0.00
A2APX7	Protein Scn1a OS=Mus musculus GN=Scn1a PE=2 SV=1 - [A2APX7_MOUSE]	-0.43	-0.54	-0.37	-0.48	0.00
Q5SF07	Insulin-like growth factor 2 mRNA-binding protein 2 OS=Mus musculus GN=Igf2bp2 PE=1 SV=1 - [IF2B2_MOUSE]	-0.54	-0.38	-0.53	-0.52	0.00
G5E8L8	Mitogen activated protein kinase kinase kinase 2 OS=Mus musculus GN=Map3k2 PE=4 SV=1 - [G5E8L8_MOUSE]	0.69	0.76	0.53	0.60	0.00
Q8VDC1	FYVE and coiled-coil domain-containing protein 1 OS=Mus musculus GN=Fyco1 PE=1 SV=1 - [FYCO1_MOUSE]	-0.60	-0.62	-0.46	-0.48	0.00
Q9DCE9	Protein Igtp OS=Mus musculus GN=Igtp PE=2 SV=1 - [Q9DCE9_MOUSE]	0.20	0.24	0.17	0.24	0.00
E9Q3I4	Intersectin-1 OS=Mus musculus GN=Itsn1 PE=2 SV=1 - [E9Q3I4_MOUSE]	-0.15	-0.14	-0.18	-0.13	0.00
Q9CU24	Protein THEMIS3 OS=Mus musculus GN=Themis3 PE=2 SV=1 - [THMS3_MOUSE]	1.04	1.23	0.84	1.02	0.00
P27659	60S ribosomal protein L3 OS=Mus musculus GN=Rpl3 PE=2 SV=3 - [RL3_MOUSE]	0.33	0.33	0.24	0.26	0.00
A2AKW1	SH2 domain-containing adapter protein B (Fragment) OS=Mus musculus GN=Shb PE=2 SV=1 - [A2AKW1_MOUSE]	-0.40	-0.44	-0.52	-0.56	0.00
P63328-2	Isoform 2 of Serine/threonine-protein phosphatase 2B catalytic subunit alpha isoform OS=Mus musculus GN=Ppp3ca - [PP2BA_MOUSE]	0.24	0.18	0.18	0.19	0.00
Q62165	Dystroglycan OS=Mus musculus GN=Dag1 PE=1 SV=4 - [DAG1_MOUSE]	-0.41	-0.48	-0.59	-0.46	0.00

Q1HFZ0	tRNA (cytosine(34)-C(5))-methyltransferase OS=Mus musculus GN=Nsun2 PE=1 SV=2 - [NSUN2_MOUSE]	0.48	0.61	0.53	0.42	0.00
Q9CRD2	ER membrane protein complex subunit 2 OS=Mus musculus GN=Emc2 PE=2 SV=1 [EMC2_MOUSE]	0.18	0.12	0.15	0.14	0.00
Q9Z0J4-2	Isoform N-NOS-2 of Nitric oxide synthase, brain OS=Mus musculus GN=Nos1 - [NOS1_MOUSE]	-0.13	-0.19	-0.17	-0.14	0.00
Q8C7B4	Gamma-glutamyltransferase 5 OS=Mus musculus GN=Ggt5 PE=2 SV=1 - [Q8C7B4_MOUSE]	-0.74	-0.67	-0.96	-0.86	0.00
D3Z6Y5	Fanconi anemia group A protein homolog OS=Mus musculus GN=Fanca PE=2 SV=1 - [D3Z6Y5_MOUSE]	-0.79	-0.90	-0.62	-0.74	0.00
E9QAZ2	Ribosomal protein L15 OS=Mus musculus GN=Gm10020 PE=3 SV=1 - [E9QAZ2_MOUSE]	0.31	0.45	0.37	0.41	0.00
Q61543	Golgi apparatus protein 1 OS=Mus musculus GN=Glg1 PE=1 SV=1 - [GSLG1_MOUSE]	0.25	0.25	0.24	0.18	0.00
A2AQ99	Protein Duox2 OS=Mus musculus GN=Duox2 PE=4 SV=1 - [A2AQ99_MOUSE]	0.45	0.65	0.55	0.63	0.00
Q9EPC1	Alpha-parvin OS=Mus musculus GN=Parva PE=1 SV=1 - [PARVA_MOUSE]	0.39	0.32	0.46	0.45	0.00
P56812	Programmed cell death protein 5 OS=Mus musculus GN=Pdcd5 PE=1 SV=3 - [PDCD5_MOUSE]	-0.13	-0.15	-0.14	-0.18	0.00
G5E920	MCG114640 OS=Mus musculus GN=Gm6104 PE=4 SV=1 - [G5E920_MOUSE]	0.41	0.50	0.37	0.36	0.00
Q8K0C4	Lanosterol 14-alpha demethylase OS=Mus musculus GN=Cyp51a1 PE=2 SV=1 - [CP51A_MOUSE]	0.58	0.77	0.67	0.83	0.00
Q2VLH6	Scavenger receptor cysteine-rich type 1 protein M130 OS=Mus musculus GN=Cd163 PE=1 SV=2 - [C163A_MOUSE]	-1.06	-1.09	-0.94	-1.35	0.00
Q9R233-2	Isoform Short of Tapasin OS=Mus musculus GN=Tapbp - [TPSN_MOUSE]	0.28	0.22	0.32	0.24	0.00

Q8K411-2	Isoform 2 of Presequence protease, mitochondrial OS=Mus musculus GN=Pitrm1 - [PREP_MOUSE]	-0.31	-0.29	-0.36	-0.41	0.00
O55135	Eukaryotic translation initiation factor 6 OS=Mus musculus GN=Eif6 PE=1 SV=2 - [IF6_MOUSE]	-0.14	-0.20	-0.19	-0.20	0.00
A6H6E2	Multimerin-2 OS=Mus musculus GN=Mmrn2 PE=2 SV=1 - [MMRN2_MOUSE]	-0.79	-0.59	-0.82	-0.63	0.00
P80316	T-complex protein 1 subunit epsilon OS=Mus musculus GN=Cct5 PE=1 SV=1 - [TCPE_MOUSE]	0.27	0.28	0.20	0.27	0.00
O35955	Proteasome subunit beta type-10 OS=Mus musculus GN=Psmb10 PE=1 SV=1 - [PSB10_MOUSE]	-0.13	-0.10	-0.15	-0.13	0.00
Q9JKY5	Huntingtin-interacting protein 1-related protein OS=Mus musculus GN=Hip1r PE=1 SV=2 - [HIP1R_MOUSE]	0.30	0.25	0.25	0.20	0.00
Q9D2V7	Coronin-7 OS=Mus musculus GN=Coro7 PE=2 SV=2 - [CORO7_MOUSE]	-0.34	-0.28	-0.24	-0.25	0.00
Q922D8	C-1-tetrahydrofolate synthase, cytoplasmic OS=Mus musculus GN=Mthfd1 PE=1 SV=4 - [C1TC_MOUSE]	0.42	0.45	0.31	0.45	0.00
Q80Y61	Brain-specific angiogenesis inhibitor 1 associated protein 2-like protein 2 OS=Mus musculus GN=Baiap2l2 PE=1 SV=1 - [BI2L2_MOUSE]	-0.82	-1.03	-1.05	-1.23	0.00
O35857	Mitochondrial import inner membrane translocase subunit TIM44 OS=Mus musculus GN=Timm44 PE=2 SV=2 - [TIM44_MOUSE]	0.29	0.22	0.26	0.20	0.00
D3YWH4	Transmembrane 9 superfamily member 1 (Fragment) OS=Mus musculus GN=Tm9sf1 PE=2 SV=1 - [D3YWH4_MOUSE]	0.23	0.23	0.29	0.21	0.00
B2RXC1	Trafficking protein particle complex subunit 11 OS=Mus musculus GN=Trappc11 PE=2 SV=1 - [TPC11_MOUSE]	0.41	0.51	0.36	0.39	0.00
Q8C605	6-phosphofructokinase OS=Mus musculus GN=Pfkp PE=2 SV=1 - [Q8C605_MOUSE]	0.37	0.36	0.26	0.33	0.00

Q3ULD5	Methylcrotonoyl-CoA carboxylase beta chain, mitochondrial OS=Mus musculus GN=Mccc2 PE=2 SV=1 - [MCCB_MOUSE]	-0.48	-0.38	-0.56	-0.53	0.00
Q8BH79	Anoctamin-10 OS=Mus musculus GN=Ano10 PE=2 SV=1 - [ANO10_MOUSE]	0.32	0.29	0.22	0.31	0.00
Q61768	Kinesin-1 heavy chain OS=Mus musculus GN=Kif5b PE=1 SV=3 - [KINH_MOUSE]	0.30	0.35	0.25	0.26	0.00
Q91XL1	Leucine-rich HEV glycoprotein (Precursor) OS=Mus musculus GN=Lrg1 PE=2 SV=1 - [Q91XL1_MOUSE]	-0.56	-0.39	-0.55	-0.55	0.00
Q9CWZ3-2	Isoform 2 of RNA-binding protein 8A OS=Mus musculus GN=Rbm8a - [RBM8A_MOUSE]	0.43	0.50	0.63	0.57	0.00
Q9ER38	Torsin-3A OS=Mus musculus GN=Tor3a PE=1 SV=2 - [TOR3A_MOUSE]	0.45	0.34	0.45	0.34	0.00
E9Q3E1	Aldehyde dehydrogenase OS=Mus musculus GN=Aldh3b2 PE=2 SV=1 - [E9Q3E1_MOUSE]	-0.91	-1.06	-1.32	-1.03	0.00
Q8BL66	Early endosome antigen 1 OS=Mus musculus GN=Eea1 PE=1 SV=2 - [EEA1_MOUSE]	-0.22	-0.26	-0.21	-0.30	0.00
Q8BI84	Melanoma inhibitory activity protein 3 OS=Mus musculus GN=Mia3 PE=1 SV=2 - [MIA3_MOUSE]	0.39	0.38	0.28	0.30	0.00
Q3UHX2	28 kDa heat- and acid-stable phosphoprotein OS=Mus musculus GN=Pdap1 PE=1 SV=1 - [HAP28_MOUSE]	0.28	0.39	0.32	0.40	0.00
Q69ZF7	Metal transporter CNNM4 OS=Mus musculus GN=Cnnm4 PE=1 SV=2 - [CNNM4_MOUSE]	0.42	0.38	0.51	0.36	0.00
Q9DBX3	Sushi domain-containing protein 2 OS=Mus musculus GN=Susd2 PE=1 SV=1 - [SUSD2_MOUSE]	-0.33	-0.45	-0.33	-0.34	0.00
Q8R1G6	PDZ and LIM domain protein 2 OS=Mus musculus GN=Pdlim2 PE=1 SV=1 - [PDLI2_MOUSE]	-0.76	-0.75	-0.59	-0.55	0.00
P56382	ATP synthase subunit epsilon, mitochondrial OS=Mus musculus GN=Atp5e PE=2 SV=2 [ATP5E_MOUSE]	-0.45	-0.55	-0.37	-0.43	0.00

Q9CX00	IST1 homolog OS=Mus musculus GN=Ist1 PE=2 SV=1 - [IST1_MOUSE]	0.54	0.39	0.43	0.38	0.00
Q9JHC0	Glutathione peroxidase 2 OS=Mus musculus GN=Gpx2 PE=2 SV=3 - [GPX2_MOUSE]	0.23	0.19	0.28	0.25	0.00
A2AN08-3	Isoform 3 of E3 ubiquitin-protein ligase UBR4 OS=Mus musculus GN=Ubr4 - [UBR4_MOUSE]	0.29	0.25	0.23	0.19	0.00
Q91XF0	Pyridoxine-5'-phosphate oxidase OS=Mus musculus GN=Pnpo PE=1 SV=1 - [PNPO_MOUSE]	-0.65	-0.64	-0.87	-0.84	0.00
O55026	Ectonucleoside triphosphate diphosphohydrolase 2 OS=Mus musculus GN=Entpd2 PE=1 SV=2 - [ENTP2_MOUSE]	-0.61	-0.44	-0.49	-0.44	0.00
D3Z7G7	Protein Zfp882 (Fragment) OS=Mus musculus GN=Zfp882 PE=2 SV=1 - [D3Z7G7_MOUSE]	-0.33	-0.43	-0.32	-0.42	0.00
B2RY56	RNA-binding protein 25 OS=Mus musculus GN=Rbm25 PE=1 SV=2 - [RBM25_MOUSE]	0.54	0.37	0.41	0.48	0.00
P01734	T-cell receptor beta chain V region 3H.25 OS=Mus musculus PE=4 SV=1 - [TVB1_MOUSE]	0.70	0.54	0.66	0.50	0.00
O08663	Methionine aminopeptidase 2 OS=Mus musculus GN=Metap2 PE=1 SV=1 - [MAP2_MOUSE]	0.20	0.24	0.17	0.25	0.00
Q99K01-2	Isoform 2 of Pyridoxal-dependent decarboxylase domain-containing protein 1 OS=Mus musculus GN=Pdxdc1 - [PDXD1_MOUSE]	0.23	0.30	0.21	0.28	0.00
P51881	ADP/ATP translocase 2 OS=Mus musculus GN=Slc25a5 PE=1 SV=3 - [ADT2_MOUSE]	0.35	0.39	0.31	0.26	0.00
Q5VCS6	Tudor domain-containing protein 5 OS=Mus musculus GN=Tdrd5 PE=1 SV=3 [TDRD5_MOUSE]	0.33	0.33	0.25	0.25	0.00
P46061	Ran GTPase-activating protein 1 OS=Mus musculus GN=Rangap1 PE=1 SV=2 - [RAGP1_MOUSE]	0.41	0.51	0.34	0.45	0.00
Q6ZQ38	Cullin-associated NEDD8-dissociated protein 1 OS=Mus musculus GN=Cand1 PE=2 SV=2 - [CAND1_MOUSE]	0.39	0.45	0.30	0.37	0.00

Q9CQI6	Coactosin-like protein OS=Mus musculus GN=Cotl1 PE=1 SV=3 - [COTL1_MOUSE]	-0.27	-0.37	-0.27	-0.27	0.00
B7ZC19	GPI transamidase component PIG-T OS=Mus musculus GN=Pigt PE=2 SV=1 - [B7ZC19_MOUSE]	0.32	0.21	0.25	0.27	0.00
Q62186	Translocon-associated protein subunit delta OS=Mus musculus GN=Ssr4 PE=2 SV=1 - [SSRD_MOUSE]	0.32	0.28	0.40	0.38	0.00
Q8BX10-2	Isoform 2 of Serine/threonine-protein phosphatase PGAM5, mitochondrial OS=Mus musculus GN=Pgam5 - [PGAM5_MOUSE]	-0.29	-0.42	-0.36	-0.41	0.00
P55264-2	Isoform Short of Adenosine kinase OS=Mus musculus GN=Adk - [ADK_MOUSE]	-0.35	-0.28	-0.42	-0.35	0.00
Q8BWY3	Eukaryotic peptide chain release factor subunit 1 OS=Mus musculus GN=Etf1 PE=1 SV=4 - [ERF1_MOUSE]	0.24	0.32	0.25	0.34	0.00
P23591	GDP-L-fucose synthase OS=Mus musculus GN=Tsta3 PE=2 SV=3 - [FCL_MOUSE]	0.14	0.09	0.11	0.11	0.00
Q4FE56	Ubiquitin carboxyl-terminal hydrolase OS=Mus musculus GN=Usp9x PE=2 SV=1 - [Q4FE56_MOUSE]	0.35	0.44	0.31	0.42	0.00
P35279	Ras-related protein Rab-6A OS=Mus musculus GN=Rab6a PE=1 SV=4 - [RAB6A_MOUSE]	0.21	0.14	0.17	0.17	0.00
Q8K0J2	UDP-GlcNAc:betaGal beta-1,3-N-acetylglucosaminyltransferase 7 OS=Mus musculus GN=B3gnt7 PE=2 SV=2 - [B3GN7_MOUSE]	0.98	0.78	0.94	0.69	0.00
Q99JB2	Stomatin-like protein 2, mitochondrial OS=Mus musculus GN=Stoml2 PE=1 SV=1 - [STML2_MOUSE]	-0.08	-0.09	-0.10	-0.07	0.00
Q8C6Y6-2	Isoform 2 of Ankyrin repeat and SOCS box protein 14 OS=Mus musculus GN=Asb14 - [ASB14_MOUSE]	0.86	0.94	1.15	1.23	0.00
Q6PD03	Serine/threonine-protein phosphatase 2A 56 kDa regulatory subunit alpha isoform OS=Mus musculus GN=Ppp2r5a PE=1 SV=1 - [2A5A_MOUSE]	0.33	0.47	0.35	0.37	0.00

G3XA35	MCG116562, isoform CRA_a OS=Mus musculus GN=Vcan PE=4 SV=1 - [G3XA35_MOUSE]	-0.18	-0.18	-0.21	-0.25	0.00
P16858	Glyceraldehyde-3-phosphate dehydrogenase OS=Mus musculus GN=Gapdh PE=1 SV=2 - [G3P_MOUSE]	0.35	0.44	0.31	0.44	0.00
Q9D8E6	60S ribosomal protein L4 OS=Mus musculus GN=Rpl4 PE=1 SV=3 - [RL4_MOUSE]	0.20	0.28	0.19	0.23	0.00
Q64735-2	Isoform 2 of Complement component receptor 1-like protein OS=Mus musculus GN=Cr1l - [CR1L_MOUSE]	0.44	0.55	0.40	0.56	0.00
Q9CR80	Protein FAM32A OS=Mus musculus GN=Fam32a PE=2 SV=1 - [FA32A_MOUSE]	0.96	0.74	0.87	0.65	0.00
Q9CQL4	39S ribosomal protein L20, mitochondrial OS=Mus musculus GN=Mrpl20 PE=1 SV=1 - [RM20_MOUSE]	0.22	0.29	0.20	0.26	0.00
O88456	Calpain small subunit 1 OS=Mus musculus GN=Capns1 PE=2 SV=1 - [CPNS1_MOUSE]	0.29	0.37	0.26	0.37	0.00
P97429	Annexin A4 OS=Mus musculus GN=Anxa4 PE=2 SV=4 - [ANXA4_MOUSE]	0.24	0.33	0.24	0.30	0.00
Q9JHS9	Spliceosome-associated protein CWC15 homolog OS=Mus musculus GN=Cwc15 PE=1 SV=1 - [CWC15_MOUSE]	-0.25	-0.28	-0.19	-0.23	0.00
P49717	DNA replication licensing factor MCM4 OS=Mus musculus GN=Mcm4 PE=2 SV=1 - [MCM4_MOUSE]	0.50	0.47	0.34	0.41	0.00
P26043	Radixin OS=Mus musculus GN=Rdx PE=1 SV=3 - [RADI_MOUSE]	-0.32	-0.25	-0.29	-0.22	0.00
Q8VDD5	Myosin-9 OS=Mus musculus GN=Myh9 PE=1 SV=4 - [MYH9_MOUSE]	0.16	0.21	0.14	0.18	0.00
Q8BJN4	Fibronectin type III domain-containing protein 9 OS=Mus musculus GN=Fndc9 PE=2 SV=1 - [FNDC9_MOUSE]	-0.95	-0.72	-1.06	-0.83	0.00
E9Q453	Tropomyosin alpha-1 chain OS=Mus musculus GN=Tpm1 PE=2 SV=1 - [E9Q453_MOUSE]	-0.85	-0.67	-0.75	-0.56	0.00

P21550	Beta-enolase OS=Mus musculus GN=Eno3 PE=1 SV=3 - [ENOB_MOUSE]	-0.12	-0.16	-0.17	-0.14	0.00
P49182	Heparin cofactor 2 OS=Mus musculus GN=Serpind1 PE=1 SV=1 - [HEP2_MOUSE]	-0.52	-0.78	-0.65	-0.72	0.00
Q9QXD6	Fructose-1,6-bisphosphatase 1 OS=Mus musculus GN=Fbp1 PE=2 SV=3 - [F16P1_MOUSE]	-0.54	-0.69	-0.67	-0.82	0.00
Q61165	Sodium/hydrogen exchanger 1 OS=Mus musculus GN=Slc9a1 PE=1 SV=1 - [SL9A1_MOUSE]	-0.68	-0.48	-0.50	-0.51	0.00
P18242	Cathepsin D OS=Mus musculus GN=Ctsd PE=1 SV=1 - [CATD_MOUSE]	-0.43	-0.43	-0.30	-0.35	0.00
O70209	PDZ and LIM domain protein 3 OS=Mus musculus GN=Pdlim3 PE=1 SV=1 - [PDLI3_MOUSE]	0.09	0.10	0.10	0.13	0.00
Q6PB70	Anoctamin-8 OS=Mus musculus GN=Ano8 PE=2 SV=3 - [ANO8_MOUSE]	-1.13	-1.39	-1.46	-1.71	0.00
G3X9V0	MCG22048, isoform CRA_a OS=Mus musculus GN=Psme2 PE=2 SV=1 - [G3X9V0_MOUSE]	0.32	0.43	0.34	0.46	0.00
Q9CZY3	Ubiquitin-conjugating enzyme E2 variant 1 OS=Mus musculus GN=Ube2v1 PE=1 SV=1 - [UB2V1_MOUSE]	0.17	0.26	0.20	0.21	0.00
P56565	Protein S100-A1 OS=Mus musculus GN=S100a1 PE=1 SV=2 - [S10A1_MOUSE]	-0.28	-0.26	-0.37	-0.34	0.00
G3X9V2	Catenin (Cadherin associated protein), delta 1, isoform CRA_a OS=Mus musculus GN=Ctnnd1 PE=4 SV=1 - [G3X9V2_MOUSE]	-0.17	-0.14	-0.21	-0.18	0.00
Q9ERK4	Exportin-2 OS=Mus musculus GN=Cse1l PE=2 SV=1 - [XPO2_MOUSE]	0.58	0.49	0.38	0.50	0.00
F6T4M4	Serine/arginine repetitive matrix protein 1 (Fragment) OS=Mus musculus GN=Srrm1 PE=2 SV=1 - [F6T4M4_MOUSE]	-0.34	-0.38	-0.27	-0.27	0.00
B2RY53	EG620155 protein OS=Mus musculus GN=Gm6133 PE=2 SV=1 - [B2RY53_MOUSE]	-0.26	-0.35	-0.29	-0.38	0.00
Q99PM9	Uridine-cytidine kinase 2 OS=Mus musculus GN=Uck2 PE=1 SV=1 - [UCK2_MOUSE]	0.20	0.16	0.25	0.21	0.00

O70326	Gremlin-1 OS=Mus musculus GN=Grem1 PE=2 SV=1 - [GREM1_MOUSE]	0.41	0.38	0.55	0.51	0.00
E9QL31	Disabled homolog 2 OS=Mus musculus GN=Dab2 PE=2 SV=1 - [E9QL31_MOUSE]	-0.34	-0.50	-0.38	-0.38	0.00
F2Z429	Tudor domain-containing protein 6 OS=Mus musculus GN=Tdrd6 PE=2 SV=1 [F2Z429_MOUSE]	-0.39	-0.50	-0.34	-0.45	0.00
Q6XQH0	Galactose-3-O-sulfotransferase 2 OS=Mus musculus GN=Gal3st2 PE=2 SV=1 - [G3ST2_MOUSE]	0.71	0.53	0.71	0.52	0.00
Q62348	Translin OS=Mus musculus GN=Tsn PE=1 SV=1 - [TSN_MOUSE]	0.49	0.62	0.41	0.54	0.00
P12804	Fibroleukin OS=Mus musculus GN=Fgl2 PE=1 SV=1 - [FGL2_MOUSE]	-0.81	-0.66	-0.78	-0.55	0.00
Q8QZR3	Carboxylesterase 6 OS=Mus musculus GN=Ces2a PE=2 SV=1 - [Q8QZR3_MOUSE]	-0.64	-0.49	-0.64	-0.46	0.00
O35218	Cleavage and polyadenylation specificity factor subunit 2 OS=Mus musculus GN=Cpsf2 PE=1 SV=1 - [CPSF2_MOUSE]	0.24	0.33	0.27	0.35	0.00
Q8JZQ5	Amiloride-sensitive amine oxidase [copper-containing] OS=Mus musculus GN=Abp1 PE=2 SV=1 - [ABP1_MOUSE]	-0.57	-0.73	-0.49	-0.58	0.00
E0CY96	tRNA-splicing endonuclease subunit Sen15 OS=Mus musculus GN=Tsen15 PE=2 SV=1 - [E0CY96_MOUSE]	-0.32	-0.31	-0.24	-0.23	0.00
E9Q8T1	Transforming acidic coiled-coil-containing protein 2 OS=Mus musculus GN=Tacc2 PE=2 SV=1 - [E9Q8T1_MOUSE]	0.60	0.40	0.59	0.54	0.00
P28867	Protein kinase C delta type OS=Mus musculus GN=Prkcd PE=1 SV=3 - [KPCD_MOUSE]	0.20	0.17	0.15	0.13	0.00
Q8R344	Coiled-coil domain-containing protein 12 OS=Mus musculus GN=Ccdc12 PE=1 SV=2 - [CCD12_MOUSE]	0.38	0.45	0.36	0.29	0.00
P25444	40S ribosomal protein S2 OS=Mus musculus GN=Rps2 PE=1 SV=3 - [RS2_MOUSE]	0.41	0.46	0.30	0.42	0.00

B1AZI6	THO complex subunit 2 OS=Mus musculus GN=Thoc2 PE=2 SV=1 - [THOC2_MOUSE]	0.40	0.54	0.44	0.58	0.00
Q9CZ44	NSFL1 cofactor p47 OS=Mus musculus GN=Nsfl1c PE=1 SV=1 - [NSF1C_MOUSE]	-0.07	-0.09	-0.10	-0.07	0.00
Q9DD20	Methyltransferase-like protein 7B OS=Mus musculus GN=Mettl7b PE=2 SV=2 - [MET7B_MOUSE]	-0.53	-0.73	-0.51	-0.67	0.00
Q62087	Serum paraoxonase/lactonase 3 OS=Mus musculus GN=Pon3 PE=1 SV=2 - [PON3_MOUSE]	-0.20	-0.18	-0.25	-0.17	0.00
Q8BML9	Glutaminyl-tRNA synthetase OS=Mus musculus GN=Qars PE=2 SV=1 - [Q8BML9_MOUSE]	0.26	0.21	0.31	0.31	0.00
Q9D819	Inorganic pyrophosphatase OS=Mus musculus GN=Ppa1 PE=1 SV=1 - [IPYR_MOUSE]	0.35	0.41	0.27	0.34	0.00
Q9DCN2-2	Isoform 2 of NADH-cytochrome b5 reductase 3 OS=Mus musculus GN=Cyb5r3 - [NB5R3_MOUSE]	-0.11	-0.07	-0.10	-0.11	0.00
Q8K3G5	Inactive serine/threonine-protein kinase VRK3 OS=Mus musculus GN=Vrk3 PE=1 SV=2 - [VRK3_MOUSE]	0.15	0.18	0.14	0.20	0.00
Q921T2-2	Isoform 2 of Torsin-1A-interacting protein 1 OS=Mus musculus GN=Tor1aip1 - [TOIP1_MOUSE]	-0.17	-0.17	-0.12	-0.17	0.00
Q8BGQ7	Alanine--tRNA ligase, cytoplasmic OS=Mus musculus GN=Aars PE=1 SV=1 - [SYAC_MOUSE]	0.25	0.24	0.32	0.34	0.00
Q07813	Apoptosis regulator BAX OS=Mus musculus GN=Bax PE=1 SV=1 - [BAX_MOUSE]	0.15	0.16	0.21	0.20	0.00
Q8BHB0	Nucleotide-binding oligomerization domain-containing protein 1 OS=Mus musculus GN=Nod1 PE=2 SV=1 - [NOD1_MOUSE]	-0.50	-0.67	-0.49	-0.66	0.00
Q9JLV1	BAG family molecular chaperone regulator 3 OS=Mus musculus GN=Bag3 PE=1 SV=2 - [BAG3_MOUSE]	-0.08	-0.12	-0.10	-0.12	0.00
P31001	Desmin OS=Mus musculus GN=Des PE=1 SV=3 - [DESM_MOUSE]	-0.20	-0.16	-0.19	-0.13	0.00

P61620	Protein transport protein Sec61 subunit alpha isoform 1 OS=Mus musculus GN=Sec61a1 PE=2 SV=2 - [S61A1_MOUSE]	0.53	0.36	0.40	0.48	0.00
D3YYN7	Sodium/potassium-transporting ATPase subunit alpha-2 OS=Mus musculus GN=Atp1a2 PE=2 SV=1 - [D3YYN7_MOUSE]	-0.57	-0.41	-0.62	-0.50	0.00
F7AJ01	SUN domain-containing protein 1 (Fragment) OS=Mus musculus GN=Sun1 PE=4 SV=1 - [F7AJ01_MOUSE]	-0.16	-0.19	-0.13	-0.14	0.00
P54822	Adenylosuccinate lyase OS=Mus musculus GN=Adsl PE=2 SV=2 - [PUR8_MOUSE]	0.36	0.25	0.27	0.27	0.00
P01897	H-2 class I histocompatibility antigen, L-D alpha chain OS=Mus musculus GN=H2-L PE=1 SV=2 - [HA1L_MOUSE]	-0.70	-0.64	-0.54	-0.47	0.00
D3Z0K8	Thioredoxin reductase 2, mitochondrial OS=Mus musculus GN=Txnrd2 PE=2 SV=1 - [D3Z0K8_MOUSE]	-0.48	-0.35	-0.51	-0.38	0.00
G3X8Y3	N-alpha-acetyltransferase 15, NatA auxiliary subunit OS=Mus musculus GN=Naa15 PE=4 SV=1 - [G3X8Y3_MOUSE]	0.38	0.30	0.36	0.26	0.00
Q8BLF1	Neutral cholesterol ester hydrolase 1 OS=Mus musculus GN=Nceh1 PE=1 SV=1 - [NCEH1_MOUSE]	-0.41	-0.37	-0.47	-0.30	0.00
E9QN52	Leucine-rich repeat flightless-interacting protein 2 OS=Mus musculus GN=Lrrfip2 PE=2 SV=1 - [E9QN52_MOUSE]	-0.45	-0.32	-0.44	-0.33	0.00
Q05A45	Olfr725 protein OS=Mus musculus GN=Olfr725 PE=2 SV=1 - [Q05A45_MOUSE]	-0.85	-1.15	-0.88	-1.19	0.00
Q8K221	Arfaptin-2 OS=Mus musculus GN=Arfip2 PE=2 SV=2 - [ARFP2_MOUSE]	0.41	0.28	0.31	0.33	0.00
Q2WG76	Protein ripply2 OS=Mus musculus GN=Ripply2 PE=2 SV=1 - [RIPP2_MOUSE]	1.37	1.77	1.17	1.57	0.00

Q2LKU9	NACHT-, LRR-, and PYD-containing protein 1 paralog a OS=Mus musculus GN=Nlrp1a PE=2 SV=1 - [Q2LKU9_MOUSE]	1.06	0.87	0.88	0.69	0.00
M0QWQ9	Uncharacterized protein OS=Mus musculus GN=Gm17174 PE=4 SV=1 - [M0QWQ9_MOUSE]	-0.70	-0.52	-0.66	-0.48	0.00
Q9DBC3	Cap-specific mRNA (nucleoside-2'-O-)-methyltransferase 1 OS=Mus musculus GN=Ftsjd2 PE=1 SV=1 - [MTR1_MOUSE]	0.59	0.48	0.50	0.38	0.00
G3UX44	Estradiol 17-beta-dehydrogenase 8 (Fragment) OS=Mus musculus GN=H2-Ke6 PE=2 SV=1 - [G3UX44_MOUSE]	-0.26	-0.32	-0.21	-0.26	0.00
Q9DBU5	E3 ubiquitin-protein ligase RNF6 OS=Mus musculus GN=Rnf6 PE=1 SV=1 - [RNF6_MOUSE]	-1.29	-1.52	-0.99	-1.22	0.00
P97807-2	Isoform Cytoplasmic of Fumarate hydratase, mitochondrial OS=Mus musculus GN=Fh - [FUMH_MOUSE]	-0.11	-0.09	-0.14	-0.10	0.00
Q9D024	Coiled-coil domain-containing protein 47 OS=Mus musculus GN=Ccdc47 PE=2 SV=2 - [CCD47_MOUSE]	0.44	0.50	0.36	0.55	0.00
D3YUR8	Protein 0610007P14Rik (Fragment) OS=Mus musculus GN=0610007P14Rik PE=2 SV=1 - [D3YUR8_MOUSE]	0.58	0.78	0.65	0.86	0.00
P47740	Fatty aldehyde dehydrogenase OS=Mus musculus GN=Aldh3a2 PE=2 SV=2 - [AL3A2_MOUSE]	0.19	0.23	0.17	0.25	0.00
Q9DCS9	NADH dehydrogenase [ubiquinone] 1 beta subcomplex subunit 10 OS=Mus musculus GN=Ndufb10 PE=1 SV=3 - [NDUBA_MOUSE]	0.36	0.31	0.26	0.24	0.00
P45377	Aldose reductase-related protein 2 OS=Mus musculus GN=Akr1b8 PE=1 SV=2 - [ALD2_MOUSE]	0.21	0.33	0.31	0.27	0.00
Q8VHY0	Chondroitin sulfate proteoglycan 4 OS=Mus musculus GN=Cspg4 PE=1 SV=3 - [CSPG4_MOUSE]	-0.28	-0.36	-0.27	-0.39	0.00

A2A6J4	Lymphocyte-specific protein 1 OS=Mus musculus GN=Lsp1 PE=2 SV=1 - [A2A6J4_MOUSE]	-0.20	-0.15	-0.16	-0.13	0.00
E9Q3P4	Protein Cenpf OS=Mus musculus GN=Cenpf PE=2 SV=1 - [E9Q3P4_MOUSE]	-0.68	-0.68	-0.92	-0.93	0.00
Q8R4N0	Citrate lyase subunit beta-like protein, mitochondrial OS=Mus musculus GN=Clybl PE=2 SV=2 - [CLYBL_MOUSE]	-0.29	-0.23	-0.29	-0.20	0.00
Q8CFX1	GDH/6PGL endoplasmic bifunctional protein OS=Mus musculus GN=H6pd PE=2 SV=2 - [G6PE_MOUSE]	-0.76	-0.58	-0.86	-0.63	0.00
E9QAS4	Chromodomain-helicase-DNA-binding protein 4 OS=Mus musculus GN=Chd4 PE=2 SV=1 - [E9QAS4_MOUSE]	0.27	0.40	0.32	0.37	0.00
Q8K224	N-acetyltransferase 10 OS=Mus musculus GN=Nat10 PE=2 SV=1 [NAT10_MOUSE]	0.18	0.21	0.27	0.20	0.00
Q4LDF6	Protein Cfhr2 (Precursor) OS=Mus musculus GN=Cfhr2 PE=2 SV=1 - [Q4LDF6_MOUSE]	-0.68	-0.54	-0.58	-0.44	0.00
P32261	Antithrombin-III OS=Mus musculus GN=Serpinc1 PE=1 SV=1 - [ANT3_MOUSE]	-0.35	-0.49	-0.53	-0.43	0.00
P97494	Glutamate--cysteine ligase catalytic subunit OS=Mus musculus GN=Gclc PE=2 SV=4 - [GSH1_MOUSE]	-0.46	-0.33	-0.42	-0.32	0.00
D3Z375	Astrocytic phosphoprotein PEA-15 (Fragment) OS=Mus musculus GN=Pea15a PE=2 SV=1 - [D3Z375_MOUSE]	-0.32	-0.24	-0.22	-0.30	0.00
D3YZ21	Protein D430041D05Rik OS=Mus musculus GN=D430041D05Rik PE=2 SV=1 - [D3YZ21_MOUSE]	-0.82	-0.80	-1.11	-1.09	0.00
Q9WUM5	Succinyl-CoA ligase [ADP/GDP-forming] subunit alpha, mitochondrial OS=Mus musculus GN=Suclg1 PE=1 SV=4 - [SUCA_MOUSE]	-0.47	-0.36	-0.50	-0.36	0.00
Q3TXS7	26S proteasome non-ATPase regulatory subunit 1 OS=Mus musculus GN=Psmd1 PE=1 SV=1 - [PSMD1_MOUSE]	0.22	0.24	0.16	0.23	0.00

Q9WV92-2	Isoform 2 of Band 4.1-like protein 3 OS=Mus musculus GN=Epb41l3 [E41L3_MOUSE]	-0.35	-0.26	-0.38	-0.29	0.00
Q8BJH1-2	Isoform 2 of Zinc finger C2HC domain-containing protein 1A OS=Mus musculus GN=Zc2hc1a - [ZC21A_MOUSE]	0.31	0.35	0.24	0.25	0.00
Q9QZH6	Evolutionarily conserved signaling intermediate in Toll pathway, mitochondrial OS=Mus musculus GN=Ecsit PE=1 SV=2 - [ECSIT_MOUSE]	0.16	0.12	0.18	0.13	0.00
Q9D0I9	Arginine--tRNA ligase, cytoplasmic OS=Mus musculus GN=Rars PE=2 SV=2 - [SYRC_MOUSE]	0.25	0.17	0.26	0.21	0.00
Q64669	NAD(P)H dehydrogenase [quinone] 1 OS=Mus musculus GN=Nqo1 PE=1 SV=3 - [NQO1_MOUSE]	-0.44	-0.33	-0.45	-0.32	0.00
Q8C0Z1	Protein ITFG3 OS=Mus musculus GN=Itfg3 PE=1 SV=1 - [ITFG3_MOUSE]	0.41	0.35	0.33	0.27	0.00
Q11136	Xaa-Pro dipeptidase OS=Mus musculus GN=Pepd PE=2 SV=3 - [PEPD_MOUSE]	-0.36	-0.24	-0.36	-0.32	0.00
Q61496	Probable ATP-dependent RNA helicase DDX4 OS=Mus musculus GN=Ddx4 PE=1 SV=2 - [DDX4_MOUSE]	0.70	0.96	0.70	0.96	0.00
Q8R146-2	Isoform 2 of Acylamino-acid-releasing enzyme OS=Mus musculus GN=Apeh - [APEH_MOUSE]	-0.16	-0.17	-0.11	-0.14	0.00
E9QNP0	KxDL motif-containing protein 1 OS=Mus musculus GN=Kxd1 PE=2 SV=1 - [E9QNP0_MOUSE]	1.17	1.69	1.18	1.39	0.00
Q8BVI4	Dihydropteridine reductase OS=Mus musculus GN=Qdpr PE=1 SV=2 - [DHPR_MOUSE]	-0.69	-0.53	-0.83	-0.70	0.00
Q9CYL5	Golgi-associated plant pathogenesis-related protein 1 OS=Mus musculus GN=Glipr2 PE=2 SV=3 - [GAPR1_MOUSE]	-0.29	-0.45	-0.42	-0.37	0.00
Q9Z1F9	SUMO-activating enzyme subunit 2 OS=Mus musculus GN=Uba2 PE=2 SV=1 - [SAE2_MOUSE]	0.26	0.20	0.27	0.31	0.00
Q5SYD0	Unconventional myosin-Id OS=Mus musculus GN=Myo1d PE=1 SV=1 - [MYO1D_MOUSE]	0.30	0.21	0.25	0.20	0.00

D3Z5N4	Glycogenin-1 (Fragment) OS=Mus musculus GN=Gyg PE=2 SV=2 - [D3Z5N4_MOUSE]	-0.39	-0.34	-0.30	-0.25	0.00
J3QQ16	Protein Col6a3 OS=Mus musculus GN=Col6a3 PE=4 SV=1 - [J3QQ16_MOUSE]	-0.55	-0.40	-0.63	-0.57	0.00
Q5SUF2	Luc7-like protein 3 OS=Mus musculus GN=Luc7l3 PE=1 SV=1 - [LC7L3_MOUSE]	0.35	0.35	0.24	0.28	0.00
Q80TJ1-4	Isoform 4 of Calcium-dependent secretion activator 1 OS=Mus musculus GN=Cadps - [CAPS1_MOUSE]	0.27	0.21	0.24	0.18	0.00
Q9D662	Protein transport protein Sec23B OS=Mus musculus GN=Sec23b PE=2 SV=1 - [SC23B_MOUSE]	0.21	0.26	0.18	0.27	0.00
Q8BPB5	EGF-containing fibulin-like extracellular matrix protein 1 OS=Mus musculus GN=Efemp1 PE=2 SV=1 - [FBLN3_MOUSE]	-0.56	-0.54	-0.43	-0.67	0.00
Q8CDI6	Coiled-coil domain-containing protein 158 OS=Mus musculus GN=Ccdc158 PE=2 SV=1 - [CD158_MOUSE]	0.66	0.54	0.54	0.42	0.00
Q62313	Trans-Golgi network integral membrane protein 1 OS=Mus musculus GN=Tgoln1 PE=1 SV=1 - [TGON1_MOUSE]	0.57	0.74	0.53	0.50	0.00
P48356-5	Isoform E of Leptin receptor OS=Mus musculus GN=Lepr - [LEPR_MOUSE]	-0.57	-0.46	-0.72	-0.61	0.00
P51660	Peroxisomal multifunctional enzyme type 2 OS=Mus musculus GN=Hsd17b4 PE=1 SV=3 - [DHB4_MOUSE]	-0.20	-0.17	-0.22	-0.15	0.00
Q9CQS2	H/ACA ribonucleoprotein complex subunit 3 OS=Mus musculus GN=Nop10 PE=3 SV=1 - [NOP10_MOUSE]	0.40	0.50	0.32	0.42	0.00
Q69ZS0-2	Isoform 2 of E3 ubiquitin-protein ligase PDZRN3 OS=Mus musculus GN=Pdzrn3 - [PZRN3_MOUSE]	-0.55	-0.56	-0.75	-0.77	0.00
B1AS29	Glutamate receptor ionotropic, kainate 3 OS=Mus musculus GN=Grik3 PE=2 SV=1 - [GRIK3_MOUSE]	0.23	0.31	0.27	0.35	0.00

A2AKI5	Integrin alpha-V light chain OS=Mus musculus GN=Itgav PE=2 SV=1 - [A2AKI5_MOUSE]	0.14	0.21	0.19	0.16	0.00
P70124	Serpin B5 OS=Mus musculus GN=Serpinb5 PE=2 SV=1 - [SPB5_MOUSE]	0.41	0.31	0.41	0.29	0.00
Q9JL27	Galactoside 2-alpha-L-fucosyltransferase 2 OS=Mus musculus GN=Fut2 PE=2 SV=1 - [FUT2_MOUSE]	0.09	0.08	0.12	0.09	0.00
Q9CZ30	Obg-like ATPase 1 OS=Mus musculus GN=Ola1 PE=1 SV=1 - [OLA1_MOUSE]	0.46	0.45	0.31	0.48	0.00
Q8BHG1	Nardilysin OS=Mus musculus GN=Nrd1 PE=1 SV=1 - [NRDC_MOUSE]	-0.07	-0.06	-0.05	-0.05	0.00
D3YWF6	Ubiquitin thioesterase OTUB1 OS=Mus musculus GN=Otub1 PE=2 SV=1 - [D3YWF6_MOUSE]	0.19	0.23	0.19	0.15	0.00
Q3THS6	S-adenosylmethionine synthase isoform type-2 OS=Mus musculus GN=Mat2a PE=2 SV=2 - [METK2_MOUSE]	-0.25	-0.36	-0.27	-0.36	0.00
Q9DCM0	Persulfide dioxygenase ETHE1, mitochondrial OS=Mus musculus GN=Ethe1 PE=1 SV=2 [ETHE1_MOUSE]	-0.24	-0.20	-0.16	-0.18	0.00
Q9D241	RING finger protein 186 OS=Mus musculus GN=Rnf186 PE=2 SV=3 - [RN186_MOUSE]	-0.67	-0.52	-0.58	-0.43	0.00
Q9DCF9-2	Isoform 2 of Translocon associated protein subunit gamma OS=Mus musculus GN=Ssr3 - [SSRG_MOUSE]	0.29	0.27	0.34	0.40	0.00
Q3UHH1-5	Isoform 5 of Zinc finger SWIM domain-containing protein 8 OS=Mus musculus GN=Zswim8 - [ZSWM8_MOUSE]	0.40	0.48	0.55	0.62	0.00
Q3UZZ6	Sulfotransferase 1 family member D1 OS=Mus musculus GN=Sult1d1 PE=1 SV=1 - [ST1D1_MOUSE]	-0.39	-0.37	-0.53	-0.51	0.00
A2A3Z2	Transcriptional regulator Kaiso (Fragment) OS=Mus musculus GN=Zbtb33 PE=2 SV=1 - [A2A3Z2_MOUSE]	0.82	0.65	0.93	0.64	0.00
P32020	Non-specific lipid-transfer protein OS=Mus musculus GN=Scp2 PE=1 SV=3 - [NLTP_MOUSE]	-0.40	-0.29	-0.44	-0.34	0.00

P08226	Apolipoprotein E OS=Mus musculus GN=Apoe PE=1 SV=2 - [APOE_MOUSE]	-0.22	-0.26	-0.17	-0.19	0.00
Q8BFU3-5	Isoform 5 of RING finger protein 214 OS=Mus musculus GN=Rnf214 - [RN214_MOUSE]	0.27	0.29	0.37	0.39	0.00
Q8R5F7-2	Isoform 2 of Interferon-induced helicase C domain-containing protein 1 OS=Mus musculus GN=Ifih1 - [IFIH1_MOUSE]	0.34	0.22	0.33	0.32	0.00
Q8C7R4	Ubiquitin-like modifier-activating enzyme 6 OS=Mus musculus GN=Uba6 PE=1 SV=1 - [UBA6_MOUSE]	0.31	0.41	0.35	0.27	0.00
Q99JH8	ER lumen protein retaining receptor 1 OS=Mus musculus GN=Kdelr1 PE=2 SV=1 - [ERD21_MOUSE]	0.76	0.79	0.55	0.58	0.00
Q91YN5-3	Isoform 3 of UDP-N-acetylhexosamine pyrophosphorylase OS=Mus musculus GN=Uap1 - [UAP1_MOUSE]	-0.09	-0.10	-0.07	-0.11	0.00
Q61166	Microtubule-associated protein RP/EB family member 1 OS=Mus musculus GN=Mapre1 PE=1 SV=3 - [MARE1_MOUSE]	0.21	0.17	0.14	0.18	0.00
O55137	Acyl-coenzyme A thioesterase 1 OS=Mus musculus GN=Acot1 PE=1 SV=1 - [ACOT1_MOUSE]	-0.56	-0.42	-0.66	-0.53	0.00
A2AW05	FACT complex subunit SSRP1 (Fragment) OS=Mus musculus GN=Ssrp1 PE=2 SV=1 [A2AW05_MOUSE]	0.23	0.18	0.27	0.21	0.00
Q9R1C7	Pre-mRNA-processing factor 40 homolog A OS=Mus musculus GN=Prpf40a PE=1 SV=1 - [PR40A_MOUSE]	0.10	0.10	0.07	0.12	0.00
Q8CG48	Structural maintenance of chromosomes protein 2 OS=Mus musculus GN=Smc2 PE=1 SV=2 - [SMC2_MOUSE]	0.58	0.40	0.56	0.44	0.00
A2APB1	Ras association domain-containing protein 2 (Fragment) OS=Mus musculus GN=Rassf2 PE=2 SV=1 - [A2APB1_MOUSE]	0.50	0.38	0.61	0.49	0.00
Q6P1B1	Xaa-Pro aminopeptidase 1 OS=Mus musculus GN=Xpnpep1 PE=2 SV=1 - [XPP1_MOUSE]	-0.23	-0.25	-0.16	-0.25	0.00

Q9DBH5	Vesicular integral-membrane protein VIP36 OS=Mus musculus GN=Lman2 PE=2 SV=2 - [LMAN2_MOUSE]	0.07	0.08	0.11	0.07	0.00
Q921G7	Electron transfer flavoprotein-ubiquinone oxidoreductase, mitochondrial OS=Mus musculus GN=Etfdh PE=1 SV=1 - [ETFD_MOUSE]	-0.13	-0.14	-0.10	-0.10	0.00
D3Z080	Sorbin and SH3 domain-containing protein 2 OS=Mus musculus GN=Sorbs2 PE=2 SV=1 - [D3Z080_MOUSE]	0.67	0.60	0.51	0.44	0.00
P03953-2	Isoform 2 of Complement factor D OS=Mus musculus GN=Cfd - [CFAD_MOUSE]	-1.24	-1.29	-0.94	-0.90	0.00
Q61830	Macrophage mannose receptor 1 OS=Mus musculus GN=Mrc1 PE=1 SV=2 - [MRC1_MOUSE]	-0.26	-0.28	-0.18	-0.26	0.00
P11276	Fibronectin OS=Mus musculus GN=Fn1 PE=1 SV=4 - [FINC_MOUSE]	-0.31	-0.23	-0.28	-0.21	0.00
B2RXR6	Serine/threonine-protein phosphatase 6 regulatory ankyrin repeat subunit B OS=Mus musculus GN=Ankrd44 PE=2 SV=1 - [ANR44_MOUSE]	0.18	0.18	0.24	0.24	0.00
E9Q947	Nucleolar protein 4 OS=Mus musculus GN=Nol4 PE=2 SV=1 - [E9Q947_MOUSE]	-0.54	-0.39	-0.60	-0.45	0.00
Q8BX02	KN motif and ankyrin repeat domain-containing protein 2 OS=Mus musculus GN=Kank2 PE=1 SV=1 - [KANK2_MOUSE]	0.17	0.21	0.15	0.22	0.00
Q9Z0S9	Prenylated Rab acceptor protein 1 OS=Mus musculus GN=Rabac1 PE=1 SV=1 - [PRAF1_MOUSE]	0.35	0.30	0.34	0.45	0.00
Q63918	Serum deprivation-response protein OS=Mus musculus GN=Sdpr PE=1 SV=3 - [SDPR_MOUSE]	-0.36	-0.24	-0.36	-0.33	0.00
O55087-2	Isoform B-2 of Myocyte-specific enhancer factor 2B OS=Mus musculus GN=Mef2b - [MEF2B_MOUSE]	1.04	1.34	0.86	1.16	0.00
P11928	2'-5'-oligoadenylate synthase 1A OS=Mus musculus GN=Oas1a PE=2 SV=2 - [OAS1A_MOUSE]	0.45	0.44	0.41	0.29	0.00

Q80YR4-2	Isoform 2 of Zinc finger protein 598 OS=Mus musculus GN=Znf598 - [ZN598_MOUSE]	-0.38	-0.30	-0.41	-0.28	0.00
E9QAI5	DNA fragmentation factor subunit beta OS=Mus musculus GN=Cad PE=2 SV=1 - [E9QAI5_MOUSE]	0.38	0.33	0.47	0.50	0.00
P16014	Secretogranin-1 OS=Mus musculus GN=Chgb PE=1 SV=2 - [SCG1_MOUSE]	-1.00	-0.77	-1.09	-0.76	0.00
Q91V55	40S ribosomal protein S5 OS=Mus musculus GN=Rps5 PE=2 SV=1 - [Q91V55_MOUSE]	0.46	0.65	0.48	0.64	0.00
Q78ZA7	Nucleosome assembly protein 1-like 4 OS=Mus musculus GN=Nap1l4 PE=1 SV=1 - [NP1L4_MOUSE]	0.32	0.26	0.24	0.21	0.00
Q9D289	Trafficking protein particle complex subunit 6B OS=Mus musculus GN=Trappc6b PE=2 SV=1 - [TPC6B_MOUSE]	0.15	0.22	0.22	0.22	0.00
Q99KE1	NAD-dependent malic enzyme, mitochondrial OS=Mus musculus GN=Me2 PE=2 SV=1 - [MAOM_MOUSE]	-0.21	-0.21	-0.19	-0.28	0.00
A2ALP5	FYVE, RhoGEF and PH domain-containing protein 1 OS=Mus musculus GN=Fgd1 PE=2 SV=1 - [A2ALP5_MOUSE]	0.92	1.22	0.80	1.09	0.00
Q9Z1M2	Interferon-g induced GTPase OS=Mus musculus GN=Irgm2 PE=2 SV=1 - [Q9Z1M2_MOUSE]	0.43	0.35	0.55	0.46	0.00
Q505F1-2	Isoform 2 of Nuclear receptor subfamily 2 group C member 1 OS=Mus musculus GN=Nr2c1 - [NR2C1_MOUSE]	-0.89	-0.76	-1.17	-1.04	0.00
E9Q166	Protein Atad2b OS=Mus musculus GN=Atad2b PE=2 SV=1 - [E9Q166_MOUSE]	-0.19	-0.20	-0.14	-0.15	0.00
Q8K212	Phosphofurin acidic cluster sorting protein 1 OS=Mus musculus GN=Pacs1 PE=1 SV=2 - [PACS1_MOUSE]	-0.78	-1.12	-1.01	-0.77	0.00
P01629	Ig kappa chain V-II region 2S1.3 OS=Mus musculus PE=1 SV=1 - [KV2A4_MOUSE]	0.47	0.35	0.54	0.41	0.00
P42125	Enoyl-CoA delta isomerase 1, mitochondrial OS=Mus musculus GN=Eci1 PE=2 SV=2 - [ECI1_MOUSE]	-0.32	-0.24	-0.39	-0.32	0.00

O88811-2	Isoform 2 of Signal transducing adapter molecule 2 OS=Mus musculus GN=Stam2 - [STAM2_MOUSE]	0.70	0.49	0.70	0.53	0.00
Q9JI75	Ribosyldihydronicotinamide dehydrogenase [quinone] OS=Mus musculus GN=Nqo2 PE=2 SV=3 - [NQO2_MOUSE]	-0.24	-0.15	-0.21	-0.23	0.00
P97298	Pigment epithelium-derived factor OS=Mus musculus GN=Serpinf1 PE=1 SV=2 - [PEDF_MOUSE]	-0.36	-0.39	-0.26	-0.41	0.00
Q8CIG0	DEP domain-containing protein 1A OS=Mus musculus GN=Depdc1a PE=1 SV=1 - [DEP1A_MOUSE]	-0.35	-0.37	-0.48	-0.51	0.00
Q765H6	Alpha-1,6-mannosylglycoprotein 6 beta-N-acetylglucosaminyltransferase B OS=Mus musculus GN=Mgat5b PE=1 SV=1 - [MGT5B_MOUSE]	-0.76	-0.55	-0.74	-0.53	0.00
Q6ZPS6	Ankyrin repeat and IBR domain-containing protein 1 OS=Mus musculus GN=Ankib1 PE=1 SV=2 - [AKIB1_MOUSE]	0.48	0.35	0.53	0.40	0.00
Q9CQY5-2	Isoform 2 of Magnesium transporter protein 1 OS=Mus musculus GN=Magt1 - [MAGT1_MOUSE]	0.26	0.20	0.27	0.31	0.00
B1ATR5	Glypican-3 OS=Mus musculus GN=Gpc3 PE=2 SV=1 - [B1ATR5_MOUSE]	-0.25	-0.23	-0.34	-0.32	0.00
Q9Z127	Large neutral amino acids transporter small subunit 1 OS=Mus musculus GN=Slc7a5 PE=1 SV=2 - [LAT1_MOUSE]	-0.24	-0.22	-0.32	-0.30	0.00
Q148V7	LisH domain and HEAT repeat-containing protein KIAA1468 OS=Mus musculus GN=Kiaa1468 PE=1 SV=1 - [K1468_MOUSE]	0.21	0.16	0.23	0.16	0.00
G3UW42	MCG49030 OS=Mus musculus GN=Gm6900 PE=4 SV=1 - [G3UW42_MOUSE]	0.27	0.25	0.28	0.37	0.00
Q9QZ23	NFU1 iron-sulfur cluster scaffold homolog, mitochondrial OS=Mus musculus GN=Nfu1 PE=1 SV=2 - [NFU1_MOUSE]	-0.11	-0.13	-0.15	-0.17	0.00

Q9D0M1	Phosphoribosyl pyrophosphate synthase-associated protein 1 OS=Mus musculus GN=Prpsap1 PE=1 SV=1 - [KPRA_MOUSE]	-0.23	-0.24	-0.31	-0.34	0.00
B1AR09	Protein Mllt6 OS=Mus musculus GN=Mllt6 PE=2 SV=1 - [B1AR09_MOUSE]	-0.65	-0.55	-0.86	-0.68	0.00
Q3B7Z2	Oxysterol-binding protein 1 OS=Mus musculus GN=Osbp PE=1 SV=3 - [OSBP1_MOUSE]	0.30	0.38	0.33	0.45	0.00
Q99KP3	Lambda-crystallin homolog OS=Mus musculus GN=Cryl1 PE=2 SV=3 - [CRYL1_MOUSE]	-0.46	-0.53	-0.33	-0.42	0.00
Q8K4L4	Protein POF1B OS=Mus musculus GN=Pof1b PE=1 SV=3 [POF1B_MOUSE]	-0.29	-0.42	-0.33	-0.29	0.00
Q8R2Q8	Bone marrow stromal antigen 2 OS=Mus musculus GN=Bst2 PE=1 SV=1 - [BST2_MOUSE]	0.50	0.48	0.68	0.46	0.00
Q8CFA2	Aminomethyltransferase, mitochondrial OS=Mus musculus GN=Amt PE=2 SV=1 - [GCST_MOUSE]	0.35	0.29	0.46	0.40	0.00
Q8BHG9	CGG triplet repeat-binding protein 1 OS=Mus musculus GN=Cggbp1 PE=2 SV=1 - [CGBP1_MOUSE]	0.18	0.18	0.12	0.16	0.00
Q8C5W0-2	Isoform 2 of Calmin OS=Mus musculus GN=Clmn - [CLMN_MOUSE]	0.44	0.27	0.36	0.37	0.00
P05366	Serum amyloid A-1 protein OS=Mus musculus GN=Saa1 PE=2 SV=2 - [SAA1_MOUSE]	0.76	0.57	0.90	0.71	0.00
Q68FM6	Protein phosphatase 1 regulatory subunit 29 OS=Mus musculus GN=Elfn2 PE=1 SV=1 - [PPR29_MOUSE]	0.95	0.77	0.72	0.60	0.00
Q8K4F5	Alpha/beta hydrolase domain-containing protein 11 OS=Mus musculus GN=Abhd11 PE=2 SV=1 - [ABHDB_MOUSE]	0.14	0.22	0.17	0.20	0.00
Q02257	Junction plakoglobin OS=Mus musculus GN=Jup PE=1 SV=3 - [PLAK_MOUSE]	0.23	0.21	0.32	0.27	0.00
Q3UJB0	Protein Sf3b2 OS=Mus musculus GN=Sf3b2 PE=2 SV=1 - [Q3UJB0_MOUSE]	0.23	0.28	0.24	0.34	0.00

Q6ZQM8	UDP-glucuronosyltransferase 1-7C OS=Mus musculus GN=Ugt1a7c PE=2 SV=1 - [UD17C_MOUSE]	-0.45	-0.71	-0.54	-0.60	0.00
E9PUB9	Chondroitin sulfate synthase 2 OS=Mus musculus GN=Chpf PE=2 SV=1 - [E9PUB9_MOUSE]	-0.16	-0.11	-0.16	-0.12	0.00
P32067	Lupus La protein homolog OS=Mus musculus GN=Ssb PE=2 SV=1 - [LA_MOUSE]	0.36	0.46	0.30	0.35	0.00
Q14CH1	Molybdenum cofactor sulfurase OS=Mus musculus GN=Mocos PE=2 SV=1 - [MOCOS_MOUSE]	-0.78	-0.66	-0.60	-0.49	0.00
Q6Y685	Transforming acidic coiled-coil-containing protein 1 OS=Mus musculus GN=Tacc1 PE=1 SV=1 - [TACC1_MOUSE]	-0.36	-0.46	-0.41	-0.56	0.00
D3YW52	Alpha-2-macroglobulin OS=Mus musculus GN=Pzp PE=2 SV=1 - [D3YW52_MOUSE]	-0.29	-0.32	-0.32	-0.44	0.00
P31230	Aminoacyl tRNA synthase complex-interacting multifunctional protein 1 OS=Mus musculus GN=Aimp1 PE=1 SV=2 - [AIMP1_MOUSE]	0.25	0.28	0.19	0.31	0.00
D3YTM0	Ceramide synthase 2 (Fragment) OS=Mus musculus GN=Cers2 PE=2 SV=1 - [D3YTM0_MOUSE]	0.25	0.30	0.19	0.24	0.00
Q3V3R1	Monofunctional C1-tetrahydrofolate synthase, mitochondrial OS=Mus musculus GN=Mthfd1l PE=1 SV=2 - [C1TM_MOUSE]	0.33	0.31	0.25	0.22	0.00
Q8CHT0	Delta-1-pyrroline-5-carboxylate dehydrogenase, mitochondrial OS=Mus musculus GN=Aldh4a1 PE=1 SV=3 - [AL4A1_MOUSE]	-0.20	-0.14	-0.21	-0.15	0.00
P52503	NADH dehydrogenase [ubiquinone] iron-sulfur protein 6, mitochondrial OS=Mus musculus GN=Ndufs6 PE=1 SV=2 - [NDUS6_MOUSE]	-0.11	-0.14	-0.09	-0.13	0.00
Q8BTW9	Serine/threonine-protein kinase PAK 4 OS=Mus musculus GN=Pak4 PE=1 SV=1 - [PAK4_MOUSE]	-0.43	-0.60	-0.50	-0.66	0.00

Q8CG71	Prolyl 3-hydroxylase 2 OS=Mus musculus GN=Leprel1 PE=2 SV=1 - [P3H2_MOUSE]	0.58	0.51	0.44	0.37	0.00
H3BJ97	Tubulointerstitial nephritis antigen-like OS=Mus musculus GN=Tinagl1 PE=2 SV=1 - [H3BJ97_MOUSE]	-0.37	-0.58	-0.44	-0.54	0.00
E9Q6J5	Protein Bod1l OS=Mus musculus GN=Bod1l PE=2 SV=1 - [E9Q6J5_MOUSE]	0.40	0.54	0.51	0.65	0.00
Q9JLZ3	Methylglutaconyl-CoA hydratase, mitochondrial OS=Mus musculus GN=Auh PE=2 SV=1 - [AUHM_MOUSE]	-0.25	-0.31	-0.40	-0.31	0.00
C6KI89	Cation channel sperm-associated protein subunit gamma 2 OS=Mus musculus GN=Catsperg2 PE=1 SV=1 - [CTSG2_MOUSE]	0.75	0.64	0.58	0.47	0.00
P70298	Homeobox protein cut-like 2 OS=Mus musculus GN=Cux2 PE=2 SV=2 - [CUX2_MOUSE]	1.36	0.99	1.28	0.91	0.00
Q9CPT3	N-acylneuraminate-9-phosphatase OS=Mus musculus GN=Nanp PE=1 SV=1 - [NANP_MOUSE]	0.34	0.45	0.28	0.39	0.00
Q9D8N0	Elongation factor 1-gamma OS=Mus musculus GN=Eef1g PE=1 SV=3 - [EF1G_MOUSE]	0.30	0.41	0.27	0.39	0.00
Q8BLR5	PH and SEC7 domain-containing protein 4 OS=Mus musculus GN=Psd4 PE=2 SV=1 - [PSD4_MOUSE]	-0.92	-0.71	-0.79	-0.58	0.00
Q80XN0	D-beta-hydroxybutyrate dehydrogenase, mitochondrial OS=Mus musculus GN=Bdh1 PE=1 SV=2 - [BDH_MOUSE]	0.10	0.10	0.10	0.06	0.00
Q6PDL0	Cytoplasmic dynein 1 light intermediate chain 2 OS=Mus musculus GN=Dync1li2 PE=1 SV=2 - [DC1L2_MOUSE]	0.11	0.08	0.08	0.07	0.00
P25206	DNA replication licensing factor MCM3 OS=Mus musculus GN=Mcm3 PE=1 SV=2 - [MCM3_MOUSE]	0.46	0.30	0.38	0.46	0.00
Q3UHC0	Trinucleotide repeat-containing gene 6C protein OS=Mus musculus GN=Tnrc6c PE=1 SV=2 - [TNR6C_MOUSE]	-1.37	-0.97	-1.46	-1.06	0.00

Q8BNU0	Armadillo repeat-containing protein 6 OS=Mus musculus GN=Armc6 PE=2 SV=1 - [ARMC6_MOUSE]	0.24	0.33	0.38	0.38	0.00
E9Q1S3	Protein transport protein Sec23A OS=Mus musculus GN=Sec23a PE=2 SV=1 - [E9Q1S3_MOUSE]	-0.10	-0.07	-0.11	-0.10	0.00
Q8BT35	Uncharacterized protein LINC00116 homolog OS=Mus musculus PE=4 SV=1 - [YB039_MOUSE]	-0.54	-0.38	-0.53	-0.38	0.00
P20108	Thioredoxin-dependent peroxide reductase, mitochondrial OS=Mus musculus GN=Prdx3 PE=1 SV=1 - [PRDX3_MOUSE]	-0.42	-0.30	-0.49	-0.43	0.00
A2AG06-2	Isoform 2 of Uncharacterized protein C17orf104 homolog OS=Mus musculus GN=Gm1564 - [CQ104_MOUSE]	0.62	0.44	0.63	0.45	0.00
Q99LI2	Chloride channel CLIC-like protein 1 OS=Mus musculus GN=Clcc1 PE=1 SV=1 - [CLCC1_MOUSE]	0.20	0.15	0.16	0.23	0.00
Q60598	Src substrate cortactin OS=Mus musculus GN=Cttn PE=1 SV=2 - [SRC8_MOUSE]	-0.21	-0.15	-0.20	-0.15	0.00
Q9WVJ3-2	Isoform 2 of Carboxypeptidase Q OS=Mus musculus GN=Cpq - [CBPQ_MOUSE]	-0.79	-0.53	-0.73	-0.87	0.00
P13595	Neural cell adhesion molecule 1 OS=Mus musculus GN=Ncam1 PE=1 SV=3 - [NCAM1_MOUSE]	-0.30	-0.21	-0.34	-0.29	0.00
A2BIN0	Major urinary proteins 11 and 8 OS=Mus musculus GN=Mup11 PE=2 SV=1 - [A2BIN0_MOUSE]	-0.71	-0.85	-0.52	-0.67	0.00
E9Q9A0	Type I inositol 3,4-bisphosphate 4-phosphatase OS=Mus musculus GN=Inpp4a PE=2 SV=1 - [E9Q9A0_MOUSE]	0.63	0.60	0.91	0.71	0.00
Q91VE0	Long-chain fatty acid transport protein 4 OS=Mus musculus GN=Slc27a4 PE=1 SV=1 - [S27A4_MOUSE]	-0.26	-0.32	-0.23	-0.35	0.00
Q99P31	Hsp70-binding protein 1 OS=Mus musculus GN=Hspbp1 PE=2 SV=1 - [HPBP1_MOUSE]	0.68	0.66	0.45	0.72	0.00

Q9QY61	Iroquois-class homeodomain protein IRX-4 OS=Mus musculus GN=Irx4 PE=2 SV=1 - [IRX4_MOUSE]	-0.72	-1.02	-0.80	-1.10	0.00
Q4V9Z5-3	Isoform 3 of Seizure 6-like protein 2 OS=Mus musculus GN=Sez6l2 - [SE6L2_MOUSE]	-2.35	-2.38	-3.31	-3.35	0.00
E9Q7D5	Protein Arhgef5 OS=Mus musculus GN=Arhgef5 PE=2 SV=1 - [E9Q7D5_MOUSE]	-0.28	-0.33	-0.21	-0.24	0.00
Q3TJ91	Lethal(2) giant larvae protein homolog 2 OS=Mus musculus GN=Llgl2 PE=2 SV=2 - [L2GL2_MOUSE]	-0.35	-0.24	-0.28	-0.24	0.00
P63158	High mobility group protein B1 OS=Mus musculus GN=Hmgb1 PE=1 SV=2 - [HMGB1_MOUSE]	0.67	0.84	0.52	0.73	0.00
P24610	Paired box protein Pax-3 OS=Mus musculus GN=Pax3 PE=1 SV=2 - [PAX3_MOUSE]	-0.84	-1.00	-0.62	-0.78	0.00
Q9D6J6	NADH dehydrogenase [ubiquinone] flavoprotein 2, mitochondrial OS=Mus musculus GN=Ndufv2 PE=1 SV=2 - [NDUV2_MOUSE]	-0.19	-0.20	-0.13	-0.20	0.00
P30204-2	Isoform II of Macrophage scavenger receptor types I and II OS=Mus musculus GN=Msr1 - [MSRE_MOUSE]	-0.47	-0.34	-0.43	-0.30	0.00
D3YUF9	UDP-glucuronic acid decarboxylase 1 (Fragment) OS=Mus musculus GN=Uxs1 PE=2 SV=1 - [D3YUF9_MOUSE]	0.26	0.20	0.32	0.26	0.00
O08795-2	Isoform 2 of Glucosidase 2 subunit beta OS=Mus musculus GN=Prkcsh - [GLU2B_MOUSE]	-0.12	-0.12	-0.14	-0.09	0.00
Q62018-3	Isoform 3 of RNA polymerase-associated protein CTR9 homolog OS=Mus musculus GN=Ctr9 - [CTR9_MOUSE]	0.35	0.45	0.29	0.34	0.00
P97822-2	Isoform 2 of Acidic leucine-rich nuclear phosphoprotein 32 family member E OS=Mus musculus GN=Anp32e - [AN32E_MOUSE]	-0.32	-0.38	-0.36	-0.24	0.00
O08749	Dihydrolipoyl dehydrogenase, mitochondrial OS=Mus musculus GN=Dld PE=1 SV=2 - [DLDH_MOUSE]	-0.30	-0.23	-0.37	-0.28	0.00

Q6WVG3	BTB/POZ domain-containing protein KCTD12 OS=Mus musculus GN=Kctd12 PE=1 SV=1 - [KCD12_MOUSE]	-0.46	-0.44	-0.33	-0.32	0.00
Q9CT10	Ran-binding protein 3 OS=Mus musculus GN=Ranbp3 PE=1 SV=2 - [RANB3_MOUSE]	0.35	0.58	0.48	0.52	0.00
Q08ED5	Protein Ces2f OS=Mus musculus GN=Ces2f PE=2 SV=1 - [Q08ED5_MOUSE]	-0.34	-0.40	-0.48	-0.54	0.00
P62073	Mitochondrial import inner membrane translocase subunit Tim10 OS=Mus musculus GN=Timm10 PE=1 SV=1 - [TIM10_MOUSE]	-0.38	-0.43	-0.36	-0.26	0.00
Q91XE4	Aspartoacylase-2 OS=Mus musculus GN=Acy3 PE=1 SV=1 - [ACY3_MOUSE]	-1.04	-1.53	-1.06	-1.42	0.00
Q9CRY7	Glycerophosphodiester phosphodiesterase domain-containing protein 1 OS=Mus musculus GN=Gdpd1 PE=2 SV=1 - [GDPD1_MOUSE]	0.47	0.59	0.37	0.55	0.00
G5E889	Mitochondrial translation optimization 1 homolog (S. cerevisiae), isoform CRA_b OS=Mus musculus GN=Mto1 PE=3 SV=1 - [G5E889_MOUSE]	-0.75	-0.93	-0.57	-0.75	0.00
Q3UJ22	Gene model 648, (NCBI) OS=Mus musculus GN=Gm648 PE=2 SV=1 - [Q3UJ22_MOUSE]	-2.01	-1.54	-2.04	-1.35	0.00
Q01730	Ras suppressor protein 1 OS=Mus musculus GN=Rsu1 PE=2 SV=3 - [RSU1_MOUSE]	0.33	0.44	0.28	0.36	0.00
P97350	Plakophilin-1 OS=Mus musculus GN=Pkp1 PE=1 SV=1 - [PKP1_MOUSE]	0.86	0.89	0.65	0.60	0.00
Q91V89	Protein Ppp2r5d OS=Mus musculus GN=Ppp2r5d PE=2 SV=1 - [Q91V89_MOUSE]	0.59	0.45	0.40	0.61	0.00
Q91X72	Hemopexin OS=Mus musculus GN=Hpx PE=1 SV=2 - [HEMO_MOUSE]	-0.22	-0.32	-0.21	-0.29	0.00
A2AD83	FERM domain-containing protein 7 OS=Mus musculus GN=Frmd7 PE=1 SV=1 - [FRMD7_MOUSE]	1.85	1.99	2.63	2.77	0.00

B4YDY0	Small conductance calcium-activated potassium channel protein 2 OS=Mus musculus GN=Kcnn2 PE=2 SV=1 - [B4YDY0_MOUSE]	-0.50	-0.69	-0.47	-0.68	0.00
O35136-2	Isoform Short of Neural cell adhesion molecule 2 OS=Mus musculus GN=Ncam2 - [NCAM2_MOUSE]	-0.32	-0.44	-0.50	-0.51	0.00
P06728	Apolipoprotein A-IV OS=Mus musculus GN=Apoa4 PE=2 SV=3 - [APOA4_MOUSE]	-0.21	-0.23	-0.15	-0.16	0.00
D3Z7C5	Ski-like protein OS=Mus musculus GN=Skil PE=2 SV=1 - [D3Z7C5_MOUSE]	0.40	0.32	0.39	0.51	0.00
Q61712	DnaJ homolog subfamily C member 1 OS=Mus musculus GN=Dnajc1 PE=1 SV=1 - [DNJC1_MOUSE]	0.18	0.26	0.18	0.26	0.00
P01642	Ig kappa chain V-V region L7 (Fragment) OS=Mus musculus GN=Gm10881 PE=1 SV=1 - [KV5A9_MOUSE]	-0.77	-1.01	-0.62	-0.84	0.00
Q9JI19	Acidic fibroblast growth factor intracellular-binding protein OS=Mus musculus GN=Fibp PE=2 SV=1 - [FIBP_MOUSE]	-0.58	-0.42	-0.54	-0.38	0.00
Q8K2F0	Bromodomain-containing protein 3 OS=Mus musculus GN=Brd3 PE=1 SV=2 - [BRD3_MOUSE]	0.22	0.27	0.19	0.29	0.00
Q9EQ06	Estradiol 17-beta-dehydrogenase 11 OS=Mus musculus GN=Hsd17b11 PE=2 SV=1 - [DHB11_MOUSE]	-0.28	-0.25	-0.25	-0.17	0.00
Q91ZE0	Trimethyllysine dioxygenase, mitochondrial OS=Mus musculus GN=Tmlhe PE=2 SV=2 - [TMLH_MOUSE]	-0.64	-0.43	-0.61	-0.71	0.00
Q9WV60	Glycogen synthase kinase-3 beta OS=Mus musculus GN=Gsk3b PE=1 SV=2 - [GSK3B_MOUSE]	0.37	0.26	0.44	0.39	0.00
P01575	Interferon beta OS=Mus musculus GN=Ifnb1 PE=1 SV=1 - [IFNB_MOUSE]	0.80	0.99	1.10	1.30	0.00
Q62009-5	Isoform 5 of Periostin OS=Mus musculus GN=Postn - [POSTN_MOUSE]	0.48	0.52	0.34	0.38	0.00

Q5RKN9	Capping protein (Actin filament) muscle Z-line, alpha 1 OS=Mus musculus GN=Capza1 PE=2 SV=1 - [Q5RKN9_MOUSE]	-0.18	-0.19	-0.22	-0.27	0.00
Q9JJ06	Glycoprotein-N-acetylgalactosamine 3-beta-galactosyltransferase 1 OS=Mus musculus GN=C1galt1 PE=1 SV=1 - [C1GLT_MOUSE]	0.18	0.29	0.25	0.28	0.00
Q60692	Proteasome subunit beta type-6 OS=Mus musculus GN=Psmb6 PE=1 SV=3 - [PSB6_MOUSE]	-0.32	-0.30	-0.20	-0.27	0.00
Q3UV70	[Pyruvate dehydrogenase [acetyl-transferring]]-phosphatase 1, mitochondrial OS=Mus musculus GN=Pdp1 PE=2 SV=1 - [PDP1_MOUSE]	-0.34	-0.32	-0.21	-0.27	0.00
Q9D287	Pre-mRNA-splicing factor SPF27 OS=Mus musculus GN=Bcas2 PE=2 SV=1 - [SPF27_MOUSE]	0.22	0.22	0.28	0.17	0.00
P55050	Fatty acid-binding protein, intestinal OS=Mus musculus GN=Fabp2 PE=2 SV=2 - [FABPI_MOUSE]	-0.70	-0.48	-0.71	-0.51	0.00
Q8BZX4-2	Isoform 2 of Splicing regulatory glutamine/lysine-rich protein 1 OS=Mus musculus GN=Srek1 - [SREK1_MOUSE]	0.22	0.27	0.35	0.26	0.00
P70700	DNA-directed RNA polymerase I subunit RPA2 OS=Mus musculus GN=Polr1b PE=2 SV=2 - [RPA2_MOUSE]	0.68	0.91	0.56	0.79	0.00
P29387	Guanine nucleotide-binding protein subunit beta-4 OS=Mus musculus GN=Gnb4 PE=2 SV=4 - [GBB4_MOUSE]	-0.54	-0.43	-0.44	-0.33	0.00
D3Z7Q8	DNA-directed RNA polymerases I and III subunit RPAC1 (Fragment) OS=Mus musculus GN=Polr1c PE=2 SV=2 - [D3Z7Q8_MOUSE]	0.38	0.52	0.48	0.62	0.00
O54714-3	Isoform 3 of E3 SUMO-protein ligase PIAS3 OS=Mus musculus GN=Pias3 - [PIAS3_MOUSE]	1.01	0.85	0.78	0.62	0.00
Q9D0L8	mRNA cap guanine-N7 methyltransferase OS=Mus musculus GN=Rnmt PE=1 SV=1 - [MCES_MOUSE]	0.17	0.25	0.16	0.21	0.00

Q8R1B4	Eukaryotic translation initiation factor 3 subunit C OS=Mus musculus GN=Eif3c PE=1 SV=1 - [EIF3C_MOUSE]	0.15	0.20	0.25	0.22	0.00
Q9CZU6	Citrate synthase, mitochondrial OS=Mus musculus GN=Cs PE=1 SV=1 - [CISY_MOUSE]	-0.24	-0.16	-0.26	-0.21	0.00
Q80X60	EF-hand calcium-binding domain-containing protein 3 OS=Mus musculus GN=Efcab3 PE=2 SV=1 - [EFCB3_MOUSE]	-1.40	-1.32	-1.00	-0.92	0.00
Q99N15	17beta-hydroxysteroid dehydrogenase type 10/short chain L-3-hydroxyacyl-CoA dehydrogenase OS=Mus musculus GN=Hsd17b10 PE=2 SV=1 - [Q99N15_MOUSE]	-0.27	-0.17	-0.24	-0.26	0.00
A8XY17	Vacuolar protein-sorting-associated protein 25 OS=Mus musculus GN=Vps25 PE=2 SV=1 - [A8XY17_MOUSE]	0.35	0.38	0.25	0.42	0.00
P29452	Caspase-1 OS=Mus musculus GN=Casp1 PE=1 SV=1 - [CASP1_MOUSE]	0.24	0.35	0.23	0.25	0.00
Q8BFW7	Lipoma-preferred partner homolog OS=Mus musculus GN=Lpp PE=1 SV=1 - [LPP_MOUSE]	0.20	0.28	0.18	0.24	0.00
J3QPE8	MCG16555 OS=Mus musculus GN=Vdac3-ps1 PE=4 SV=1 - [J3QPE8_MOUSE]	-0.51	-0.36	-0.54	-0.38	0.00
Q8K0T7	Protein unc-13 homolog C OS=Mus musculus GN=Unc13c PE=1 SV=3 - [UN13C_MOUSE]	1.09	0.83	0.98	0.67	0.00
Q3UW66	Sulfurtransferase OS=Mus musculus GN=Mpst PE=2 SV=1 - [Q3UW66_MOUSE]	0.16	0.26	0.22	0.19	0.00
Q80X41-2	Isoform 2 of Serine/threonine-protein kinase VRK1 OS=Mus musculus GN=Vrk1 - [VRK1_MOUSE]	0.55	0.38	0.62	0.47	0.00
P01029	Complement C4-B OS=Mus musculus GN=C4b PE=1 SV=3 - [CO4B_MOUSE]	-0.48	-0.45	-0.30	-0.36	0.00
P14152	Malate dehydrogenase, cytoplasmic OS=Mus musculus GN=Mdh1 PE=1 SV=3 - [MDHC_MOUSE]	-0.20	-0.15	-0.24	-0.19	0.00
Q91WM2	Cat eye syndrome critical region protein 5 homolog OS=Mus musculus GN=Cecr5 PE=2 SV=1 - [CECR5_MOUSE]	-0.39	-0.41	-0.35	-0.25	0.00

Q99MR3	Solute carrier family 12 member 9 OS=Mus musculus GN=Slc12a9 PE=1 SV=2 - [S12A9_MOUSE]	0.33	0.45	0.42	0.54	0.00
Q80TL7-2	Isoform 2 of Protein MON2 homolog OS=Mus musculus GN=Mon2 - [MON2_MOUSE]	0.28	0.25	0.38	0.36	0.00
Q8BVF2	Phosducin-like protein 3 OS=Mus musculus GN=Pdcl3 PE=1 SV=1 [PDCL3_MOUSE]	0.16	0.19	0.25	0.22	0.00
Q9WTR5	Cadherin-13 OS=Mus musculus GN=Cdh13 PE=1 SV=2 - [CAD13_MOUSE]	-0.19	-0.15	-0.20	-0.13	0.00
P28665	Murinoglobulin-1 OS=Mus musculus GN=Mug1 PE=1 SV=3 [MUG1_MOUSE]	-0.67	-0.60	-0.64	-0.41	0.00
A2AB83	E3 ubiquitin-protein ligase TRIM11 OS=Mus musculus GN=Trim11 PE=2 SV=1 - [A2AB83_MOUSE]	-0.65	-0.82	-0.90	-1.07	0.00
E9QP57	Pecanex-like protein 2 OS=Mus musculus GN=Pcnxl2 PE=2 SV=1 - [E9QP57_MOUSE]	-0.54	-0.48	-0.40	-0.34	0.00
Q9JLB2	MAGUK p55 subfamily member 5 OS=Mus musculus GN=Mpp5 PE=1 SV=1 - [MPP5_MOUSE]	0.11	0.13	0.14	0.18	0.00
Q9ET54-4	Isoform 4 of Palladin OS=Mus musculus GN=Palld - [PALLD_MOUSE]	0.15	0.21	0.14	0.19	0.00
Q8R123	FAD synthase OS=Mus musculus GN=Flad1 PE=1 SV=1 - [FAD1_MOUSE]	0.41	0.32	0.52	0.39	0.00
O54988-2	Isoform 2 of STE20-like serine/threonine-protein kinase OS=Mus musculus GN=Slk - [SLK_MOUSE]	0.12	0.11	0.08	0.13	0.00
P63276	40S ribosomal protein S17 OS=Mus musculus GN=Rps17 PE=1 SV=2 - [RS17_MOUSE]	0.70	0.49	0.62	0.45	0.00
Q91ZJ5	UTP--glucose-1-phosphate uridylyltransferase OS=Mus musculus GN=Ugp2 PE=2 SV=3 - [UGPA_MOUSE]	0.15	0.11	0.18	0.17	0.00
F6ZED4	Protein Gm9970 OS=Mus musculus GN=Gm9970 PE=4 SV=1 - [F6ZED4_MOUSE]	0.62	0.44	0.60	0.42	0.00

Q60829	Protein phosphatase 1 regulatory subunit 1B OS=Mus musculus GN=Ppp1r1b PE=1 SV=2 - [PPR1B_MOUSE]	-0.75	-0.51	-0.61	-0.49	0.00
Q9DC61	Mitochondrial-processing peptidase subunit alpha OS=Mus musculus GN=Pmpca PE=1 SV=1 - [MPPA_MOUSE]	-0.27	-0.20	-0.32	-0.31	0.00
Q8R010	Aminoacyl tRNA synthase complex-interacting multifunctional protein 2 OS=Mus musculus GN=Aimp2 PE=1 SV=2 - [AIMP2_MOUSE]	0.27	0.37	0.24	0.26	0.00
Q8CCH2	NHL repeat-containing protein 3 OS=Mus musculus GN=Nhlrc3 PE=2 SV=1 - [NHLC3_MOUSE]	-0.25	-0.39	-0.29	-0.37	0.00
Q80SZ7	Guanine nucleotide-binding protein G(I)/G(S)/G(O) subunit gamma-5 OS=Mus musculus GN=Gng5 PE=2 SV=2 - [GBG5_MOUSE]	-0.29	-0.22	-0.36	-0.34	0.00
D3YZX8	Signal recognition particle 9 kDa protein OS=Mus musculus GN=Srp9 PE=2 SV=1 - [D3YZX8_MOUSE]	-0.29	-0.25	-0.23	-0.18	0.00
B1AVH7	TBC1 domain family member 2A OS=Mus musculus GN=Tbc1d2 PE=2 SV=1 - [TBD2A_MOUSE]	0.30	0.38	0.41	0.26	0.00
Q8R464	Cell adhesion molecule 4 OS=Mus musculus GN=Cadm4 PE=1 SV=1 - [CADM4_MOUSE]	-0.32	-0.53	-0.42	-0.40	0.00
Q9D6S7	Ribosome-recycling factor, mitochondrial OS=Mus musculus GN=Mrrf PE=1 SV=1 - [RRFM_MOUSE]	-0.26	-0.20	-0.31	-0.22	0.00
Q8K262	Placenta-specific protein 9 OS=Mus musculus GN=Plac9 PE=2 SV=1 - [PLAC9_MOUSE]	-0.77	-0.54	-0.78	-0.55	0.00
E0CYF6	Protein AW554918 OS=Mus musculus GN=AW554918 PE=4 SV=1 - [E0CYF6_MOUSE]	-0.45	-0.58	-0.42	-0.64	0.00
Q9CX34	Suppressor of G2 allele of SKP1 homolog OS=Mus musculus GN=Sugt1 PE=2 SV=3 [SUGT1_MOUSE]	0.19	0.13	0.16	0.13	0.00
Q9JIB3	Baculoviral IAP repeat-containing protein 1g OS=Mus musculus GN=Naip7 PE=3 SV=1 [BIR1G_MOUSE]	0.77	1.10	0.89	1.22	0.00

Q64378	Peptidyl-prolyl cis-trans isomerase FKBP5 OS=Mus musculus GN=Fkbp5 PE=1 SV=1 - [FKBP5_MOUSE]	-0.43	-0.31	-0.50	-0.37	0.00
Q80TB8	Synaptic vesicle membrane protein VAT-1 homolog-like OS=Mus musculus GN=Vat1l PE=2 SV=2 - [VAT1L_MOUSE]	-0.36	-0.23	-0.37	-0.34	0.00
J3QNR5	Uncharacterized protein OS=Mus musculus GN=Tubb4b- ps1 PE=3 SV=1 - [J3QNR5_MOUSE]	0.33	0.27	0.29	0.20	0.00
Q91ZW3	SWI/SNF-related matrix-associated actin-dependent regulator of chromatin subfamily A member 5 OS=Mus musculus GN=Smarca5 PE=1 SV=1 - [SMCA5_MOUSE]	0.43	0.30	0.44	0.31	0.00
O08784	Treacle protein OS=Mus musculus GN=Tcof1 PE=1 SV=1 - [TCOF_MOUSE]	0.15	0.21	0.15	0.22	0.00
Q06138	Calcium-binding protein 39 OS=Mus musculus GN=Cab39 PE=1 SV=2 - [CAB39_MOUSE]	0.98	0.64	0.86	0.67	0.00
Q3U7R1	Extended synaptotagmin-1 OS=Mus musculus GN=Esyt1 PE=2 SV=2 - [ESYT1_MOUSE]	-0.13	-0.13	-0.14	-0.08	0.00
Q6ZWZ7	60S ribosomal protein L17 OS=Mus musculus GN=Rpl17 PE=2 SV=1 [Q6ZWZ7_MOUSE]	0.48	0.74	0.51	0.62	0.00
Q9ER72	Cysteine--tRNA ligase, cytoplasmic OS=Mus musculus GN=Cars PE=1 SV=2 - [SYCC_MOUSE]	0.22	0.15	0.20	0.14	0.00
Q3V0K9	Plastin-1 OS=Mus musculus GN=Pls1 PE=2 SV=1 - [PLSI_MOUSE]	0.21	0.24	0.15	0.18	0.00
Q8C5L3-3	Isoform 3 of CCR4-NOT transcription complex subunit 2 OS=Mus musculus GN=Cnot2 - [CNOT2_MOUSE]	-0.56	-0.79	-0.71	-0.93	0.00
P61922	4-aminobutyrate aminotransferase, mitochondrial OS=Mus musculus GN=Abat PE=1 SV=1 - [GABT_MOUSE]	-0.34	-0.22	-0.37	-0.31	0.00
Q8R1Q8	Cytoplasmic dynein 1 light intermediate chain 1 OS=Mus musculus GN=Dync1li1 PE=1 SV=1 - [DC1L1_MOUSE]	0.42	0.54	0.42	0.33	0.00

Q08093	Calponin-2 OS=Mus musculus GN=Cnn2 PE=2 SV=1 - [CNN2_MOUSE]	0.12	0.20	0.18	0.18	0.00
E2QRQ2	Rho guanine nucleotide exchange factor 38 OS=Mus musculus GN=Arhgef38 PE=2 SV=2 - [E2QRQ2_MOUSE]	0.45	0.32	0.47	0.33	0.00
P99024	Tubulin beta-5 chain OS=Mus musculus GN=Tubb5 PE=1 SV=1 - [TBB5_MOUSE]	0.27	0.38	0.32	0.43	0.00
Q8BMA6	Signal recognition particle subunit SRP68 OS=Mus musculus GN=Srp68 PE=2 SV=2 - [SRP68_MOUSE]	0.25	0.26	0.34	0.38	0.00
F6TCF9	BAG family molecular chaperone regulator 1 OS=Mus musculus GN=Bag1 PE=2 SV=1 - [F6TCF9_MOUSE]	0.16	0.23	0.25	0.19	0.00
Q8BH44	Coronin-2B OS=Mus musculus GN=Coro2b PE=2 SV=2 - [COR2B_MOUSE]	-0.43	-0.32	-0.35	-0.26	0.00
P54728	UV excision repair protein RAD23 homolog B OS=Mus musculus GN=Rad23b PE=1 SV=2 - [RD23B_MOUSE]	-0.08	-0.08	-0.07	-0.05	0.00
A2A998	Complement component C8 alpha chain OS=Mus musculus GN=C8a PE=2 SV=1 - [A2A998_MOUSE]	-0.71	-0.52	-0.65	-0.45	0.00
Q921M3	Splicing factor 3B subunit 3 OS=Mus musculus GN=Sf3b3 PE=2 SV=1 - [SF3B3_MOUSE]	-0.17	-0.25	-0.16	-0.19	0.00
Q9Z0M5	Lysosomal acid lipase/cholesteryl ester hydrolase OS=Mus musculus GN=Lipa PE=2 SV=2 - [LICH_MOUSE]	-0.39	-0.55	-0.35	-0.51	0.00
Q8BJD1	Inter-alpha-trypsin inhibitor heavy chain H5 OS=Mus musculus GN=Itih5 PE=2 SV=1 - [ITIH5_MOUSE]	-0.25	-0.39	-0.29	-0.37	0.00
E9Q132	60S ribosomal protein L24 OS=Mus musculus GN=Rpl24 PE=2 SV=1 [E9Q132_MOUSE]	0.26	0.42	0.31	0.37	0.00
E9Q769	Junctional adhesion molecule C OS=Mus musculus GN=Jam3 PE=2 SV=1 - [E9Q769_MOUSE]	-0.28	-0.45	-0.37	-0.45	0.00
P51859	Hepatoma-derived growth factor OS=Mus musculus GN=Hdgf PE=1 SV=2 - [HDGF_MOUSE]	0.10	0.11	0.07	0.09	0.00

Q80ZV0	Ribonuclease H2 subunit B OS=Mus musculus GN=Rnaseh2b PE=1 SV=2 - [RNH2B_MOUSE]	0.70	0.84	0.50	0.64	0.00
P54116	Erythrocyte band 7 integral membrane protein OS=Mus musculus GN=Stom PE=1 SV=3 - [STOM_MOUSE]	-0.72	-0.73	-0.50	-0.51	0.00
Q5XJE5	RNA polymerase-associated protein LEO1 OS=Mus musculus GN=Leo1 PE=1 SV=2 - [LEO1_MOUSE]	0.14	0.10	0.16	0.12	0.00
Q9WUR0	Protein Tinag OS=Mus musculus GN=Tinag PE=2 SV=1 - [Q9WUR0_MOUSE]	-0.51	-0.41	-0.61	-0.40	0.00
P36371	Antigen peptide transporter 2 OS=Mus musculus GN=Tap2 PE=2 SV=1 - [TAP2_MOUSE]	0.25	0.17	0.25	0.29	0.00
E9Q174	Unconventional myosin-VI OS=Mus musculus GN=Myo6 PE=2 SV=1 - [E9Q174_MOUSE]	0.83	0.86	0.58	0.60	0.00
Q91YW3	DnaJ homolog subfamily C member 3 OS=Mus musculus GN=Dnajc3 PE=1 SV=1 - [DNJC3_MOUSE]	0.22	0.29	0.20	0.31	0.00
Q7TNV0	Protein DEK OS=Mus musculus GN=Dek PE=1 SV=1 - [DEK_MOUSE]	0.27	0.17	0.22	0.19	0.00
O89112	LanC-like protein 1 OS=Mus musculus GN=Lancl1 PE=1 SV=1 - [LANC1_MOUSE]	-0.15	-0.19	-0.15	-0.22	0.00
Q3U1Z5-2	Isoform 2 of G-protein-signaling modulator 3 OS=Mus musculus GN=Gpsm3 - [GPSM3_MOUSE]	-0.29	-0.46	-0.36	-0.45	0.00
E9PYF7	AP1AR OS=Mus musculus GN=Ap1ar PE=4 SV=1 - [E9PYF7_MOUSE]	0.26	0.18	0.30	0.23	0.00
Q60714	Long-chain fatty acid transport protein 1 OS=Mus musculus GN=Slc27a1 PE=1 SV=1 - [S27A1_MOUSE]	-1.16	-1.23	-0.79	-0.89	0.00
B1ATI9	Growth arrest-specific protein 7 OS=Mus musculus GN=Gas7 PE=4 SV=1 - [B1ATI9_MOUSE]	-0.42	-0.33	-0.26	-0.41	0.00
P35979	60S ribosomal protein L12 OS=Mus musculus GN=Rpl12 PE=1 SV=2 [RL12_MOUSE]	0.33	0.23	0.38	0.28	0.00

Q9D1I5	Methylmalonyl-CoA epimerase, mitochondrial OS=Mus musculus GN=Mcee PE=2 SV=1 - [MCEE_MOUSE]	-0.73	-0.66	-0.44	-0.58	0.00
G5E8R8	MCG129950, isoform CRA_a OS=Mus musculus GN=Ubxn7 PE=4 SV=1 - [G5E8R8_MOUSE]	-0.19	-0.14	-0.18	-0.12	0.00
Q6P4U0	Thrombospondin type-1 domain-containing protein 7B OS=Mus musculus GN=Thsd7b PE=1 SV=1 - [THS7B_MOUSE]	-0.57	-0.47	-0.77	-0.68	0.00
Q91WU5	Arsenite methyltransferase OS=Mus musculus GN=As3mt PE=2 SV=2 - [AS3MT_MOUSE]	0.11	0.08	0.13	0.13	0.00
Q8CIH5	1-phosphatidylinositol 4,5-bisphosphate phosphodiesterase gamma-2 OS=Mus musculus GN=Plcg2 PE=1 SV=1 - [PLCG2_MOUSE]	0.58	0.36	0.48	0.41	0.00
P52795	Ephrin-B1 OS=Mus musculus GN=Efnb1 PE=1 SV=1 - [EFNB1_MOUSE]	-0.30	-0.24	-0.34	-0.21	0.00
Q9JIP7	Solute carrier family 15 member 1 OS=Mus musculus GN=Slc15a1 PE=1 SV=2 - [S15A1_MOUSE]	0.88	0.61	0.95	0.68	0.00
Q9EPB5	Serine hydrolase-like protein OS=Mus musculus GN=Serhl PE=2 SV=1 - [SERHL_MOUSE]	-0.82	-0.94	-0.55	-0.82	0.00
B2RS91	RNA polymerase I-specific transcription initiation factor RRN3 OS=Mus musculus GN=Rrn3 PE=1 SV=1 - [RRN3_MOUSE]	-0.16	-0.19	-0.24	-0.27	0.00
Q9DB10	Single-pass membrane protein with aspartate-rich tail 1, mitochondrial OS=Mus musculus GN=Smdt1 PE=2 SV=1 - [SMDT1_MOUSE]	0.74	0.71	0.52	0.49	0.00
Q7TSE6	Serine/threonine-protein kinase 38-like OS=Mus musculus GN=Stk38l PE=1 SV=2 - [ST38L_MOUSE]	0.59	0.57	0.41	0.39	0.00
Q9D2Z1	Protein Ceacam20 OS=Mus musculus GN=Ceacam20 PE=2 SV=1 - [Q9D2Z1_MOUSE]	0.71	0.52	0.63	0.43	0.00

Q5JCS9	UDP-GlcNAc:betaGal beta-1,3-N-acetylglucosaminyltransferase 3 OS=Mus musculus GN=B3gnt3 PE=2 SV=1 - [B3GN3_MOUSE]	0.89	0.62	0.93	0.64	0.00
D3Z3D9	Beta-chimaerin OS=Mus musculus GN=Chn2 PE=2 SV=1 - [D3Z3D9_MOUSE]	-0.25	-0.32	-0.35	-0.42	0.00
Q99P88	Nuclear pore complex protein Nup155 OS=Mus musculus GN=Nup155 PE=2 SV=1 - [NU155_MOUSE]	0.27	0.27	0.21	0.17	0.00
P52760	Ribonuclease UK114 OS=Mus musculus GN=Hrsp12 PE=1 SV=3 - [UK114_MOUSE]	0.82	0.71	0.74	0.48	0.00
Q62318	Transcription intermediary factor 1-beta OS=Mus musculus GN=Trim28 PE=1 SV=3 - [TIF1B_MOUSE]	0.22	0.14	0.15	0.19	0.00
Q91XZ7	Protein Pcdhb3 OS=Mus musculus GN=Pcdhb3 PE=2 SV=1 - [Q91XZ7_MOUSE]	-0.81	-1.05	-1.12	-1.37	0.00
B8QI34	Liprin-alpha 2 OS=Mus musculus GN=Ppfia2 PE=2 SV=1 - [B8QI34_MOUSE]	-1.02	-0.88	-1.42	-1.28	0.00
Q3UDW8-2	Isoform 2 of Heparan-alpha-glucosaminide N-acetyltransferase OS=Mus musculus GN=Hgsnat - [HGNAT_MOUSE]	-0.14	-0.17	-0.10	-0.13	0.00
E0CXV3	Alcohol dehydrogenase 1 OS=Mus musculus GN=Adh1 PE=2 SV=1 - [E0CXV3_MOUSE]	0.55	0.79	0.51	0.75	0.00
A2A863-2	Isoform 2 of Integrin beta-4 OS=Mus musculus GN=Itgb4 - [ITB4_MOUSE]	0.22	0.17	0.19	0.13	0.00
Q64012-2	Isoform 1 of RNA-binding protein Raly OS=Mus musculus GN=Raly - [RALY_MOUSE]	-0.34	-0.21	-0.23	-0.27	0.00
P05622	Platelet-derived growth factor receptor beta OS=Mus musculus GN=Pdgfrb PE=1 SV=1 - [PGFRB_MOUSE]	-0.97	-0.70	-0.88	-0.60	0.00
Q9DBG6	Dolichyl-diphosphooligosaccharide--protein glycosyltransferase subunit 2 OS=Mus musculus GN=Rpn2 PE=2 SV=1 - [RPN2_MOUSE]	0.18	0.24	0.14	0.22	0.00
Q3UZP4	Small VCP/p97-interacting protein OS=Mus musculus GN=Svip PE=2 SV=1 - [SVIP_MOUSE]	-0.23	-0.32	-0.20	-0.29	0.00

P60710	Actin, cytoplasmic 1 OS=Mus musculus GN=Actb PE=1 SV=1 - [ACTB_MOUSE]	0.22	0.16	0.23	0.15	0.00
P45591	Cofilin-2 OS=Mus musculus GN=Cfl2 PE=1 SV=1 - [COF2_MOUSE]	0.35	0.23	0.29	0.22	0.00
E9PZF0	Nucleoside diphosphate kinase OS=Mus musculus GN=Gm20390 PE=2 SV=1 - [E9PZF0_MOUSE]	0.51	0.69	0.41	0.55	0.00
Q91YR9	Prostaglandin reductase 1 OS=Mus musculus GN=Ptgr1 PE=2 SV=2 - [PTGR1_MOUSE]	-0.14	-0.12	-0.18	-0.20	0.00
P22682	E3 ubiquitin-protein ligase CBL OS=Mus musculus GN=Cbl PE=1 SV=3 - [CBL_MOUSE]	-0.17	-0.21	-0.27	-0.27	0.00
Q61129	Complement factor I OS=Mus musculus GN=Cfi PE=1 SV=3 - [CFAI_MOUSE]	-0.19	-0.22	-0.18	-0.29	0.00
O35640	Annexin A8 OS=Mus musculus GN=Anxa8 PE=2 SV=2 - [ANXA8_MOUSE]	0.83	0.92	0.66	1.11	0.00
D3YVA2	Neurocalcin-delta (Fragment) OS=Mus musculus GN=Ncald PE=2 SV=1 - [D3YVA2_MOUSE]	-0.38	-0.40	-0.33	-0.24	0.00
P56212-2	Isoform ARPP-16 of cAMP-regulated phosphoprotein 19 OS=Mus musculus GN=Arpp19 - [ARP19_MOUSE]	0.27	0.30	0.35	0.20	0.00
Q9DCH4	Eukaryotic translation initiation factor 3 subunit F OS=Mus musculus GN=Eif3f PE=1 SV=2 - [EIF3F_MOUSE]	0.13	0.09	0.12	0.15	0.00
P18528	Ig heavy chain V region 6.96 OS=Mus musculus PE=4 SV=1 - [HVM57_MOUSE]	0.71	0.55	0.94	0.78	0.00
Q9CQM9	Glutaredoxin-3 OS=Mus musculus GN=Glrx3 PE=1 SV=1 - [GLRX3_MOUSE]	-0.17	-0.27	-0.21	-0.27	0.00
P97364	Selenide, water dikinase 2 OS=Mus musculus GN=Sephs2 PE=1 SV=3 - [SPS2_MOUSE]	-0.53	-0.62	-0.37	-0.47	0.00
Q01279	Epidermal growth factor receptor OS=Mus musculus GN=Egfr PE=1 SV=1 - [EGFR_MOUSE]	-0.24	-0.16	-0.28	-0.22	0.00
P35123	Ubiquitin carboxyl-terminal hydrolase 4 OS=Mus musculus GN=Usp4 PE=1 SV=3 - [UBP4_MOUSE]	0.21	0.26	0.21	0.33	0.00

Q505D1	Serine/threonine-protein phosphatase 6 regulatory ankyrin repeat subunit A OS=Mus musculus GN=Ankrd28 PE=1 SV=1 - [ANR28_MOUSE]	0.29	0.24	0.40	0.35	0.00
E9Q1T1	Calcium/calmodulin-dependent protein kinase type II subunit beta OS=Mus musculus GN=Camk2d PE=2 SV=1 - [E9Q1T1_MOUSE]	0.22	0.31	0.19	0.24	0.00
P61027	Ras-related protein Rab-10 OS=Mus musculus GN=Rab10 PE=1 SV=1 - [RAB10_MOUSE]	0.20	0.15	0.12	0.20	0.00
P11531	Dystrophin OS=Mus musculus GN=Dmd PE=1 SV=3 - [DMD_MOUSE]	0.09	0.07	0.08	0.05	0.00
E9PUI0	Alpha-N-acetylgalactosaminide alpha-2,6-sialyltransferase 6 OS=Mus musculus GN=St6galnac6 PE=2 SV=1 - [E9PUI0_MOUSE]	0.29	0.27	0.38	0.22	0.00
Q8BUY9	Geranylgeranyl transferase type-1 subunit beta OS=Mus musculus GN=Pggt1b PE=2 SV=1 - [PGTB1_MOUSE]	-0.74	-0.96	-0.56	-0.78	0.00
Q8K377	Leucine-rich repeat transmembrane neuronal protein 1 OS=Mus musculus GN=Lrrtm1 PE=2 SV=1 - [LRRT1_MOUSE]	0.28	0.41	0.43	0.48	0.00
Q8C4U3	Secreted frizzled-related protein 1 OS=Mus musculus GN=Sfrp1 PE=1 SV=3 - [SFRP1_MOUSE]	0.80	0.58	0.93	0.63	0.00
P51658	Estradiol 17-beta-dehydrogenase 2 OS=Mus musculus GN=Hsd17b2 PE=2 SV=2 - [DHB2_MOUSE]	-0.38	-0.39	-0.47	-0.27	0.00
Q9WV98	Mitochondrial import inner membrane translocase subunit Tim9 OS=Mus musculus GN=Timm9 PE=1 SV=1 - [TIM9_MOUSE]	-0.58	-0.42	-0.42	-0.36	0.00
P84104-2	Isoform Short of Serine/arginine-rich splicing factor 3 OS=Mus musculus GN=Srsf3 - [SRSF3_MOUSE]	0.29	0.42	0.27	0.40	0.00
P97315	Cysteine and glycine-rich protein 1 OS=Mus musculus GN=Csrp1 PE=1 SV=3 - [CSRP1_MOUSE]	0.41	0.58	0.36	0.46	0.00

Q9Z0E6	Interferon-induced guanylate-binding protein 2 OS=Mus musculus GN=Gbp2 PE=1 SV=1 - [GBP2_MOUSE]	0.75	0.59	0.63	0.43	0.00
O54833	Casein kinase II subunit alpha' OS=Mus musculus GN=Csnk2a2 PE=2 SV=1 - [CSK22_MOUSE]	-0.21	-0.18	-0.29	-0.29	0.00
Q9DCD0	6-phosphogluconate dehydrogenase, decarboxylating OS=Mus musculus GN=Pgd PE=2 SV=3 - [6PGD_MOUSE]	-0.13	-0.11	-0.07	-0.11	0.00
P03966	N-myc proto-oncogene protein OS=Mus musculus GN=Mycn PE=2 SV=2 - [MYCN_MOUSE]	0.57	0.79	0.47	0.68	0.00
Q9QX47-3	Isoform 3 of Protein SON OS=Mus musculus GN=Son - [SON_MOUSE]	0.09	0.07	0.05	0.06	0.00
A2A8S2	Protein YIPF1 (Fragment) OS=Mus musculus GN=Yipf1 PE=2 SV=1 - [A2A8S2_MOUSE]	0.43	0.45	0.29	0.31	0.00
Q924H9-2	Isoform 2 of ATP-dependent RNA helicase DQX1 OS=Mus musculus GN=Dqx1 - [DQX1_MOUSE]	-0.65	-0.90	-0.54	-0.79	0.00
Q9ES52-3	Isoform 3 of Phosphatidylinositol 3,4,5-trisphosphate 5-phosphatase 1 OS=Mus musculus GN=Inpp5d - [SHIP1_MOUSE]	0.51	0.42	0.39	0.29	0.00
Q80V26	Inositol monophosphatase 3 OS=Mus musculus GN=Impad1 PE=1 SV=1 - [IMPA3_MOUSE]	0.42	0.37	0.61	0.48	0.00
Q9CQW0	ER membrane protein complex subunit 6 OS=Mus musculus GN=Emc6 PE=2 SV=1 [EMC6_MOUSE]	0.25	0.39	0.26	0.36	0.00
Q9CZU3	Superkiller viralicidic activity 2-like 2 OS=Mus musculus GN=Skiv2l2 PE=2 SV=1 - [SK2L2_MOUSE]	0.30	0.32	0.24	0.41	0.00
Q9QYK4	Heparan-sulfate 6-O-sulfotransferase 3 OS=Mus musculus GN=Hs6st3 PE=1 SV=2 - [H6ST3_MOUSE]	-0.49	-0.67	-0.40	-0.58	0.00
Q3USW5	FAD-dependent oxidoreductase domain-containing protein 2 OS=Mus musculus GN=Foxred2 PE=2 SV=1 - [FXRD2_MOUSE]	0.96	1.28	0.74	1.06	0.00

Q6P2K6	Serine/threonine-protein phosphatase 4 regulatory subunit 3A OS=Mus musculus GN=Smek1 PE=1 SV=1 - [P4R3A_MOUSE]	0.55	0.61	0.38	0.44	0.00
Q9WUS4-2	Isoform 2 of Gap junction alpha-10 protein OS=Mus musculus GN=Gja10 - [CXA10_MOUSE]	-0.32	-0.38	-0.48	-0.53	0.00
Q99K85	Phosphoserine aminotransferase OS=Mus musculus GN=Psat1 PE=1 SV=1 [SERC_MOUSE]	0.41	0.54	0.50	0.32	0.00
Q91YS8	Calcium/calmodulin-dependent protein kinase type 1 OS=Mus musculus GN=Camk1 PE=1 SV=1 - [KCC1A_MOUSE]	0.14	0.19	0.14	0.21	0.00
Q8R332-4	Isoform 4 of Nucleoporin p58/p45 OS=Mus musculus GN=Nupl1 - [NUPL1_MOUSE]	0.65	0.42	0.57	0.72	0.00
Q9JKB1	Ubiquitin carboxyl-terminal hydrolase isozyme L3 OS=Mus musculus GN=Uchl3 PE=1 SV=2 - [UCHL3_MOUSE]	-0.12	-0.20	-0.15	-0.17	0.00
P59325	Eukaryotic translation initiation factor 5 OS=Mus musculus GN=Eif5 PE=1 SV=1 - [IF5_MOUSE]	0.34	0.49	0.32	0.32	0.00
Q61176	Arginase-1 OS=Mus musculus GN=Arg1 PE=1 SV=1 - [ARGI1_MOUSE]	0.31	0.32	0.25	0.19	0.00
Q91VW3	SH3 domain-binding glutamic acid-rich-like protein 3 OS=Mus musculus GN=Sh3bgrl3 PE=1 SV=1 - [SH3L3_MOUSE]	-0.24	-0.24	-0.14	-0.24	0.00
P62245	40S ribosomal protein S15a OS=Mus musculus GN=Rps15a PE=2 SV=2 - [RS15A_MOUSE]	0.24	0.24	0.19	0.32	0.00
E9Q704	Peptidyl-glycine alpha-amidating monooxygenase OS=Mus musculus GN=Pam PE=2 SV=1 - [E9Q704_MOUSE]	-0.27	-0.23	-0.28	-0.17	0.00
Q64010	Adapter molecule crk OS=Mus musculus GN=Crk PE=1 SV=1 - [CRK_MOUSE]	-0.28	-0.23	-0.16	-0.26	0.00
Q00897	Alpha-1-antitrypsin 1-4 OS=Mus musculus GN=Serpina1d PE=2 SV=1 - [A1AT4_MOUSE]	-0.83	-0.57	-0.87	-0.58	0.00
O35129	Prohibitin-2 OS=Mus musculus GN=Phb2 PE=1 SV=1 - [PHB2_MOUSE]	-0.20	-0.24	-0.14	-0.17	0.00

Q60634-3	Isoform 3 of Flotillin-2 OS=Mus musculus GN=Flot2 - [FLOT2_MOUSE]	-0.13	-0.13	-0.15	-0.20	0.00
Q3UVG3	Protein FAM91A1 OS=Mus musculus GN=Fam91a1 PE=1 SV=1 - [F91A1_MOUSE]	0.43	0.52	0.30	0.44	0.00
P47962	60S ribosomal protein L5 OS=Mus musculus GN=Rpl5 PE=1 SV=3 - [RL5_MOUSE]	0.20	0.12	0.21	0.19	0.00
Q3USH5	Splicing factor, suppressor of white-apricot homolog OS=Mus musculus GN=Sfswap PE=1 SV=2 - [SFSWA_MOUSE]	-0.52	-0.67	-0.75	-0.90	0.00
Q8K4Z3	NAD(P)H-hydrate epimerase OS=Mus musculus GN=Apoa1bp PE=1 SV=1 - [NNRE_MOUSE]	-0.15	-0.22	-0.26	-0.25	0.00
G3X928	SEC23-interacting protein OS=Mus musculus GN=Sec23ip PE=4 SV=1 - [G3X928_MOUSE]	0.36	0.50	0.31	0.35	0.00
Q9JHK4	Geranylgeranyl transferase type-2 subunit alpha OS=Mus musculus GN=Rabggta PE=1 SV=1 - [PGTA_MOUSE]	0.40	0.45	0.26	0.34	0.00
Q9ESL4-2	Isoform 2 of Mitogen-activated protein kinase kinase kinase MLT OS=Mus musculus GN=Mltk - [MLTK_MOUSE]	1.48	1.01	1.46	0.99	0.00
Q05922	Dual specificity protein phosphatase 2 OS=Mus musculus GN=Dusp2 PE=2 SV=2 - [DUS2_MOUSE]	0.65	0.85	0.48	0.68	0.00
A2AD84	Protein 2610018G03Rik OS=Mus musculus GN=2610018G03Rik PE=2 SV=1 - [A2AD84_MOUSE]	0.42	0.38	0.60	0.56	0.00
Q99L59	X-linked lymphocyte-regulated protein 3A OS=Mus musculus GN=Xlr3a PE=2 SV=1 - [Q99L59_MOUSE]	0.56	0.82	0.54	0.80	0.00
D3Z001	Protein 2310057J18Rik OS=Mus musculus GN=2310057J18Rik PE=4 SV=1 - [D3Z001_MOUSE]	0.66	0.76	0.47	0.81	0.00
Q62159	Rho-related GTP-binding protein RhoC OS=Mus musculus GN=Rhoc PE=1 SV=2 - [RHOC_MOUSE]	0.51	0.68	0.72	0.88	0.00

Q91V08	C-type lectin domain family 2 member D OS=Mus musculus GN=Clec2d PE=1 SV=1 - [CLC2D_MOUSE]	0.22	0.17	0.17	0.12	0.00
P70335	Rho-associated protein kinase 1 OS=Mus musculus GN=Rock1 PE=1 SV=1 - [ROCK1_MOUSE]	0.18	0.14	0.24	0.18	0.00
E9Q4Y4	Protein Cep192 OS=Mus musculus GN=Cep192 PE=2 SV=1 - [E9Q4Y4_MOUSE]	-0.58	-0.55	-0.85	-0.82	0.00
Q8BIH0-2	Isoform 2 of Histone deacetylase complex subunit SAP130 OS=Mus musculus GN=Sap130 - [SP130_MOUSE]	-0.25	-0.24	-0.17	-0.16	0.00
Q9D0R2	Threonine--tRNA ligase, cytoplasmic OS=Mus musculus GN=Tars PE=1 SV=2 - [SYTC_MOUSE]	0.08	0.08	0.08	0.12	0.00
P43025	Tetranectin OS=Mus musculus GN=Clec3b PE=1 SV=2 - [TETN_MOUSE]	-0.34	-0.45	-0.26	-0.40	0.00
Q6P5B5	Fragile X mental retardation syndrome-related protein 2 OS=Mus musculus GN=Fxr2 PE=2 SV=1 - [Q6P5B5_MOUSE]	0.17	0.16	0.17	0.10	0.00
Q7TSQ8	Pyruvate dehydrogenase phosphatase regulatory subunit, mitochondrial OS=Mus musculus GN=Pdpr PE=2 SV=1 - [PDPR_MOUSE]	-0.43	-0.60	-0.36	-0.56	0.00
Q91X91	Nicotinate-nucleotide pyrophosphorylase [carboxylating] OS=Mus musculus GN=Qprt PE=2 SV=1 - [NADC_MOUSE]	-1.10	-0.79	-0.96	-0.65	0.00
E0CXW2	Uncharacterized aarF domain-containing protein kinase 5 OS=Mus musculus GN=Adck5 PE=2 SV=1 - [E0CXW2_MOUSE]	-0.36	-0.54	-0.38	-0.55	0.00
Q8BQB5	Protein 6430548M08Rik OS=Mus musculus GN=6430548M08Rik PE=2 SV=1 - [Q8BQB5_MOUSE]	0.37	0.29	0.50	0.42	0.00
F8VQD7	Receptor-type tyrosine-protein phosphatase gamma OS=Mus musculus GN=Ptprg PE=2 SV=1 - [F8VQD7_MOUSE]	-0.84	-0.60	-0.73	-0.50	0.00
Q9JLQ0	CD2-associated protein OS=Mus musculus GN=Cd2ap PE=1 SV=3 - [CD2AP_MOUSE]	-0.22	-0.16	-0.25	-0.16	0.00

Q8VEB4	Group XV phospholipase A2 OS=Mus musculus GN=Pla2g15 PE=1 SV=1 - [PAG15_MOUSE]	-0.53	-0.45	-0.30	-0.39	0.00
Q9D1D4	Transmembrane emp24 domain-containing protein 10 OS=Mus musculus GN=Tmed10 PE=2 SV=1 - [TMEDA_MOUSE]	0.41	0.45	0.25	0.38	0.00
D3YY50	Copper homeostasis protein cutC homolog (Fragment) OS=Mus musculus GN=Cutc PE=2 SV=1 - [D3YY50_MOUSE]	-0.13	-0.19	-0.14	-0.21	0.00
D3Z5F0	Cdc42 effector protein 5 (Fragment) OS=Mus musculus GN=Cdc42ep5 PE=2 SV=1 - [D3Z5F0_MOUSE]	-0.47	-0.60	-0.34	-0.48	0.00
P28271	Cytoplasmic aconitate hydratase OS=Mus musculus GN=Aco1 PE=1 SV=3 - [ACOC_MOUSE]	-0.09	-0.12	-0.09	-0.14	0.00
Q5SV54	Ortholog of human amyotrophic lateral sclerosis 2 (Juvenile) chromosome region, candidate 19 (ALS2CR19) OS=Mus musculus GN=Pard3b PE=4 SV=1 - [Q5SV54_MOUSE]	0.39	0.57	0.35	0.52	0.00
G5E8K5-3	Isoform 3 of Ankyrin-3 OS=Mus musculus GN=Ank3 - [ANK3_MOUSE]	-0.15	-0.18	-0.11	-0.11	0.00
O35098	Dihydropyrimidinase-related protein 4 OS=Mus musculus GN=Dpysl4 PE=1 SV=1 - [DPYL4_MOUSE]	0.32	0.26	0.24	0.18	0.00
Q61702	Inter-alpha-trypsin inhibitor heavy chain H1 OS=Mus musculus GN=Itih1 PE=1 SV=2 - [ITIH1_MOUSE]	-0.16	-0.22	-0.22	-0.28	0.00
Q571E4	N-acetylgalactosamine-6-sulfatase OS=Mus musculus GN=Galns PE=2 SV=2 - [GALNS_MOUSE]	-0.67	-0.45	-0.72	-0.50	0.00
O88713	Killer cell lectin-like receptor subfamily G member 1 OS=Mus musculus GN=Klrg1 PE=1 SV=2 - [KLRG1_MOUSE]	-0.41	-0.59	-0.36	-0.54	0.00
Q9Z1K6	E3 ubiquitin-protein ligase ARIH2 OS=Mus musculus GN=Arih2 PE=2 SV=1 - [ARI2_MOUSE]	0.44	0.35	0.34	0.25	0.00
P55086	Proteinase-activated receptor 2 OS=Mus musculus GN=F2rl1 PE=1 SV=1 - [PAR2_MOUSE]	0.76	0.95	0.54	0.73	0.00

Q0VDN7	RUN domain-containing protein 1 OS=Mus musculus GN=Rundc1 PE=2 SV=1 - [RUND1_MOUSE]	0.73	0.51	0.91	0.68	0.00
P58281-2	Isoform 2 of Dynamin-like 120 kDa protein, mitochondrial OS=Mus musculus GN=Opa1 - [OPA1_MOUSE]	0.21	0.18	0.29	0.19	0.00
A2AMV1	RIKEN cDNA 2610012O22, isoform CRA_a OS=Mus musculus GN=Mrto4 PE=3 SV=1 - [A2AMV1_MOUSE]	0.16	0.23	0.20	0.27	0.00
O88520	Leucine-rich repeat protein SHOC-2 OS=Mus musculus GN=Shoc2 PE=2 SV=2 - [SHOC2_MOUSE]	0.61	0.49	0.48	0.35	0.00
E9PUM4	Talin-2 OS=Mus musculus GN=Tln2 PE=4 SV=1 - [E9PUM4_MOUSE]	0.26	0.19	0.26	0.16	0.00
Q9EQH2	Endoplasmic reticulum aminopeptidase 1 OS=Mus musculus GN=Erap1 PE=2 SV=2 - [ERAP1_MOUSE]	0.41	0.25	0.41	0.31	0.00
F6UFZ5	Protein Pcf11 (Fragment) OS=Mus musculus GN=Pcf11 PE=4 SV=1 - [F6UFZ5_MOUSE]	0.57	0.81	0.48	0.72	0.00
Q8BW66	Solute carrier family 25 member 48 OS=Mus musculus GN=Slc25a48 PE=2 SV=2 - [S2548_MOUSE]	0.71	0.50	0.89	0.67	0.00
Q99J77	N-acetylneuraminic acid synthase (Sialic acid synthase) OS=Mus musculus GN=Nans PE=2 SV=1 - [Q99J77_MOUSE]	0.27	0.32	0.18	0.23	0.00
Q9D8B3	Charged multivesicular body protein 4b OS=Mus musculus GN=Chmp4b PE=2 SV=2 - [CHM4B_MOUSE]	0.12	0.19	0.21	0.19	0.00
E9PXI8	Corticotropin-releasing factor receptor 2 OS=Mus musculus GN=Crhr2 PE=2 SV=1 - [E9PXI8_MOUSE]	0.24	0.32	0.42	0.31	0.00
Q0VEK2	MCG3056, isoform CRA_b OS=Mus musculus GN=Chp2 PE=2 SV=1 - [Q0VEK2_MOUSE]	0.77	0.57	1.01	0.80	0.00
Q8BW10	RNA-binding protein NOB1 OS=Mus musculus GN=Nob1 PE=1 SV=1 - [NOB1_MOUSE]	0.53	0.79	0.58	0.85	0.00

P61205	ADP-ribosylation factor 3 OS=Mus musculus GN=Arf3 PE=2 SV=2 - [ARF3_MOUSE]	0.05	0.05	0.04	0.07	0.00
A2AMW0	Capping protein (Actin filament) muscle Z-line, beta, isoform CRA_a OS=Mus musculus GN=Capzb PE=4 SV=1 - [A2AMW0_MOUSE]	-0.26	-0.16	-0.29	-0.24	0.00
Q5SUT0	RNA-binding protein EWS OS=Mus musculus GN=Ewsr1 PE=2 SV=1 - [Q5SUT0_MOUSE]	0.32	0.22	0.31	0.20	0.00
F6UB40	Ras-responsive element-binding protein 1 (Fragment) OS=Mus musculus GN=Rreb1 PE=4 SV=1 - [F6UB40_MOUSE]	0.45	0.58	0.33	0.46	0.00
Q9DBL9-2	Isoform 2 of 1-acylglycerol-3-phosphate O-acyltransferase ABHD5 OS=Mus musculus GN=Abhd5 - [ABHD5_MOUSE]	-0.97	-0.65	-0.98	-0.66	0.00
Q5NCE8	Magnesium transporter MRS2 homolog, mitochondrial OS=Mus musculus GN=Mrs2 PE=2 SV=2 - [MRS2_MOUSE]	0.20	0.28	0.17	0.25	0.00
Q9JMG2	C1GALT1-specific chaperone 1 OS=Mus musculus GN=C1galt1c1 PE=2 SV=1 - [C1GLC_MOUSE]	0.33	0.49	0.29	0.38	0.00
G3V018	Heterogeneous nuclear ribonucleoprotein Q OS=Mus musculus GN=Syncrip PE=2 SV=1 - [G3V018_MOUSE]	0.29	0.38	0.25	0.41	0.00
Q8QZT1	Acetyl-CoA acetyltransferase, mitochondrial OS=Mus musculus GN=Acat1 PE=1 SV=1 - [THIL_MOUSE]	-0.35	-0.23	-0.33	-0.23	0.00
P80317	T-complex protein 1 subunit zeta OS=Mus musculus GN=Cct6a PE=1 SV=3 - [TCPZ_MOUSE]	0.22	0.22	0.16	0.14	0.00
O35216	Histone H3-like centromeric protein A OS=Mus musculus GN=Cenpa PE=2 SV=1 - [CENPA_MOUSE]	-0.95	-1.15	-0.65	-0.85	0.00
O70310	Glycylpeptide N-tetradecanoyltransferase 1 OS=Mus musculus GN=Nmt1 PE=1 SV=1 - [NMT1_MOUSE]	0.31	0.21	0.31	0.20	0.00

Q9Z1Q5	Chloride intracellular channel protein 1 OS=Mus musculus GN=Clic1 PE=1 SV=3 - [CLIC1_MOUSE]	0.18	0.32	0.26	0.28	0.00
Q9DC50	Peroxisomal carnitine O-octanoyltransferase OS=Mus musculus GN=Crot PE=1 SV=1 - [OCTC_MOUSE]	-0.16	-0.18	-0.25	-0.24	0.00
D3YVG1	Septin-1 (Fragment) OS=Mus musculus GN=Sept1 PE=2 SV=1 [D3YVG1_MOUSE]	-0.52	-0.39	-0.42	-0.29	0.00
J3QMV3	Uncharacterized protein OS=Mus musculus GN=Gm21778 PE=4 SV=1 - [J3QMV3_MOUSE]	-0.93	-0.78	-1.32	-1.18	0.00
A2AGS6	Tumor protein p53-inducible protein 11 (Fragment) OS=Mus musculus GN=Trp53i11 PE=2 SV=1 - [A2AGS6_MOUSE]	-0.64	-0.84	-0.48	-0.63	0.00
Q6ZQI3	Malectin OS=Mus musculus GN=Mlec PE=2 SV=2 - [MLEC_MOUSE]	0.29	0.25	0.25	0.16	0.00
Q3UPL0	Protein transport protein Sec31A OS=Mus musculus GN=Sec31a PE=1 SV=2 - [SC31A_MOUSE]	0.44	0.27	0.29	0.36	0.00
F7CZ64	Voltage-dependent calcium channel gamma-8 subunit OS=Mus musculus GN=Cacng8 PE=4 SV=2 - [F7CZ64_MOUSE]	0.22	0.32	0.28	0.38	0.00
Q922D4-2	Isoform 2 of Serine/threonine-protein phosphatase 6 regulatory subunit 3 OS=Mus musculus GN=Ppp6r3 - [PP6R3_MOUSE]	0.31	0.27	0.29	0.17	0.00
P01644	Ig kappa chain V-V region HP R16.7 OS=Mus musculus PE=1 SV=1 - [KV5AB_MOUSE]	1.01	1.09	0.63	0.82	0.00
P83917	Chromobox protein homolog 1 OS=Mus musculus GN=Cbx1 PE=1 SV=1 - [CBX1_MOUSE]	-0.25	-0.41	-0.27	-0.36	0.00
Q8VEK3	Heterogeneous nuclear ribonucleoprotein U OS=Mus musculus GN=Hnrnpu PE=1 SV=1 - [HNRPU_MOUSE]	0.11	0.18	0.12	0.16	0.00
P35585	AP-1 complex subunit mu-1 OS=Mus musculus GN=Ap1m1 PE=1 SV=3 - [AP1M1_MOUSE]	-0.09	-0.08	-0.09	-0.13	0.00

P14873	Microtubule-associated protein 1B OS=Mus musculus GN=Map1b PE=1 SV=2 - [MAP1B_MOUSE]	-0.26	-0.22	-0.36	-0.24	0.00
Q91WD5	NADH dehydrogenase [ubiquinone] iron-sulfur protein 2, mitochondrial OS=Mus musculus GN=Ndufs2 PE=1 SV=1 - [NDUS2_MOUSE]	0.19	0.15	0.11	0.14	0.00
Q9CRC8	Leucine-rich repeat-containing protein 40 OS=Mus musculus GN=Lrrc40 PE=2 SV=2 - [LRC40_MOUSE]	0.26	0.40	0.25	0.33	0.00
Q9R059	Four and a half LIM domains protein 3 OS=Mus musculus GN=Fhl3 PE=2 SV=2 - [FHL3_MOUSE]	-0.50	-0.37	-0.28	-0.43	0.00
Q0V8T7	Contactin-associated protein like 5-3 OS=Mus musculus GN=Cntnap5c PE=2 SV=1 - [CTP5C_MOUSE]	-0.59	-0.46	-0.53	-0.33	0.00
Q8VCQ8	Caldesmon 1 OS=Mus musculus GN=Cald1 PE=2 SV=1 - [Q8VCQ8_MOUSE]	0.33	0.27	0.24	0.19	0.00
P26638	Serine--tRNA ligase, cytoplasmic OS=Mus musculus GN=Sars PE=2 SV=3 - [SYSC_MOUSE]	0.54	0.63	0.36	0.45	0.00
P62965	Cellular retinoic acid-binding protein 1 OS=Mus musculus GN=Crabp1 PE=1 SV=2 - [RABP1_MOUSE]	-1.34	-0.92	-1.19	-0.80	0.00
P82198	Transforming growth factor-beta-induced protein ig-h3 OS=Mus musculus GN=Tgfbi PE=2 SV=1 - [BGH3_MOUSE]	0.27	0.36	0.22	0.36	0.00
Q8BTY1	Kynurenine--oxoglutarate transaminase 1 OS=Mus musculus GN=Ccbl1 PE=2 SV=1 [KAT1_MOUSE]	-0.34	-0.44	-0.60	-0.47	0.00
E0CX48	Gap junction protein (Fragment) OS=Mus musculus GN=Gjb6 PE=2 SV=1 - [E0CX48_MOUSE]	-0.41	-0.62	-0.44	-0.66	0.00
E9Q3A7	Protein Cdh19 OS=Mus musculus GN=Cdh19 PE=3 SV=1 - [E9Q3A7_MOUSE]	-0.73	-0.80	-0.48	-0.55	0.00
P70663	SPARC-like protein 1 OS=Mus musculus GN=Sparcl1 PE=1 SV=3 - [SPRL1_MOUSE]	-0.36	-0.51	-0.30	-0.38	0.00

A2AM05-4	Isoform 4 of Centlein OS=Mus musculus GN=Cntln - [CNTLN_MOUSE]	-0.56	-0.79	-0.46	-0.70	0.00
P58404-2	Isoform 2 of Striatin-4 OS=Mus musculus GN=Strn4 - [STRN4_MOUSE]	1.12	0.73	1.20	0.81	0.00
Q9D172	ES1 protein homolog, mitochondrial OS=Mus musculus GN=D10Jhu81e PE=1 SV=1 - [ES1_MOUSE]	-0.13	-0.19	-0.12	-0.16	0.00
Q3U1J4	DNA damage-binding protein 1 OS=Mus musculus GN=Ddb1 PE=1 SV=2 - [DDB1_MOUSE]	-0.20	-0.25	-0.15	-0.16	0.00
Q91VW5	Golgin subfamily A member 4 OS=Mus musculus GN=Golga4 PE=1 SV=2 - [GOGA4_MOUSE]	0.07	0.06	0.05	0.08	0.00
Q71FD5	E3 ubiquitin-protein ligase ZNRF2 OS=Mus musculus GN=Znrf2 PE=1 SV=1 - [ZNRF2_MOUSE]	-0.72	-0.85	-0.48	-0.61	0.00
P35288	Ras-related protein Rab-23 OS=Mus musculus GN=Rab23 PE=1 SV=2 - [RAB23_MOUSE]	0.70	0.95	0.53	0.78	0.00
Q6A4L0	Solute carrier family 22 member 13 OS=Mus musculus GN=Slc22a13 PE=2 SV=3 - [S22AD_MOUSE]	0.53	0.42	0.41	0.29	0.00
G3UW36	MCG117244, isoform CRA_a OS=Mus musculus GN=4932443I19Rik PE=4 SV=1 - [G3UW36_MOUSE]	0.47	0.68	0.65	0.85	0.00
D3Z7X0	MCG142036 OS=Mus musculus GN=Acad12 PE=3 SV=1 - [D3Z7X0_MOUSE]	-0.29	-0.45	-0.31	-0.46	0.00
P97369	Neutrophil cytosol factor 4 OS=Mus musculus GN=Ncf4 PE=2 SV=2 - [NCF4_MOUSE]	0.59	0.41	0.53	0.35	0.00
Q9R0M8	UDP-galactose translocator OS=Mus musculus GN=Slc35a2 PE=2 SV=1 - [S35A2_MOUSE]	0.35	0.46	0.26	0.33	0.00
Q8R071	Inositol-trisphosphate 3-kinase A OS=Mus musculus GN=Itpka PE=2 SV=1 - [IP3KA_MOUSE]	0.96	1.32	0.74	1.10	0.00
G3X9K3	Brefeldin A-inhibited guanine nucleotide-exchange protein 1 OS=Mus musculus GN=Arfgef1 PE=2 SV=1 - [BIG1_MOUSE]	0.43	0.58	0.34	0.40	0.00

Q99P58	Ras-related protein Rab-27B OS=Mus musculus GN=Rab27b PE=1 SV=3 - [RB27B_MOUSE]	0.17	0.24	0.31	0.25	0.00
Q9CR41	Huntingtin-interacting protein K OS=Mus musculus GN=Hypk PE=2 SV=2 - [HYPK_MOUSE]	-0.17	-0.19	-0.20	-0.11	0.00
Q61462-2	Isoform 2 of Cytochrome b-245 light chain OS=Mus musculus GN=Cyba - [CY24A_MOUSE]	0.16	0.09	0.12	0.15	0.00
Q9CPX7	28S ribosomal protein S16, mitochondrial OS=Mus musculus GN=Mrps16 PE=2 SV=1 - [RT16_MOUSE]	0.69	0.54	0.52	0.38	0.00
O88492	Perilipin-4 OS=Mus musculus GN=Plin4 PE=1 SV=2 - [PLIN4_MOUSE]	-0.30	-0.19	-0.32	-0.23	0.00
P62702	40S ribosomal protein S4, X isoform OS=Mus musculus GN=Rps4x PE=2 SV=2 - [RS4X_MOUSE]	0.17	0.26	0.17	0.17	0.00
Q8VCM4	Lipoyltransferase 1, mitochondrial OS=Mus musculus GN=Lipt1 PE=2 SV=1 - [LIPT_MOUSE]	-0.65	-0.93	-0.54	-0.82	0.00
Q9Z1B5	Mitotic spindle assembly checkpoint protein MAD2A OS=Mus musculus GN=Mad2l1 PE=2 SV=2 - [MD2L1_MOUSE]	0.65	0.48	0.86	0.69	0.00
Q8C4J7	Transducin beta-like protein 3 OS=Mus musculus GN=Tbl3 PE=2 SV=1 - [TBL3_MOUSE]	0.36	0.20	0.29	0.26	0.00
Q08501-3	Isoform PRL-R2 of Prolactin receptor OS=Mus musculus GN=Prlr - [PRLR_MOUSE]	-0.34	-0.46	-0.49	-0.61	0.00
P13439	Uridine 5'-monophosphate synthase OS=Mus musculus GN=Umps PE=2 SV=3 - [UMPS_MOUSE]	0.23	0.13	0.23	0.19	0.00
Q8K124	Pleckstrin homology domain-containing family O member 2 OS=Mus musculus GN=Plekho2 PE=1 SV=1 - [PKHO2_MOUSE]	-0.36	-0.34	-0.48	-0.56	0.00
Q9WV52	Pleckstrin-2 OS=Mus musculus GN=Plek2 PE=1 SV=1 - [PLEK2_MOUSE]	0.54	0.68	0.38	0.51	0.00
P03911	NADH-ubiquinone oxidoreductase chain 4 OS=Mus musculus GN=Mtnd4 PE=1 SV=1 - [NU4M_MOUSE]	-0.41	-0.49	-0.62	-0.70	0.00

Q3ULJ0-2	Isoform 2 of Glycerol-3-phosphate dehydrogenase 1-like protein OS=Mus musculus GN=Gpd1l - [GPD1L_MOUSE]	0.16	0.12	0.20	0.22	0.00
O89110	Caspase-8 OS=Mus musculus GN=Casp8 PE=1 SV=1 - [CASP8_MOUSE]	0.22	0.19	0.17	0.29	0.00
Q9CYR6	Phosphoacetylglucosamine mutase OS=Mus musculus GN=Pgm3 PE=1 SV=1 - [AGM1_MOUSE]	0.28	0.31	0.17	0.31	0.00
Q3TAI4	Tumor protein D52-like 2, isoform CRA_c OS=Mus musculus GN=Tpd52l2 PE=2 SV=1 - [Q3TAI4_MOUSE]	0.14	0.16	0.09	0.16	0.00
Q8R3C6	Probable RNA-binding protein 19 OS=Mus musculus GN=Rbm19 PE=1 SV=1 - [RBM19_MOUSE]	1.57	1.95	1.07	1.45	0.00
Q9DCS1	Transmembrane protein 176A OS=Mus musculus GN=Tmem176a PE=2 SV=2 - [T176A_MOUSE]	-1.19	-1.21	-0.78	-0.80	0.00
G3X8U9	Mitogen activated protein kinase 8, isoform CRA_b OS=Mus musculus GN=Mapk8 PE=4 SV=1 - [G3X8U9_MOUSE]	0.38	0.58	0.41	0.61	0.00
G3UVV4	Hexokinase 1, isoform CRA_f OS=Mus musculus GN=Hk1 PE=3 SV=1 - [G3UVV4_MOUSE]	-0.28	-0.22	-0.27	-0.16	0.00
P70271	PDZ and LIM domain protein 4 OS=Mus musculus GN=Pdlim4 PE=1 SV=3 - [PDLI4_MOUSE]	-0.25	-0.18	-0.16	-0.16	0.00
F8VQB6	Unconventional myosin-X OS=Mus musculus GN=Myo10 PE=1 SV=1 - [MYO10_MOUSE]	-0.48	-0.68	-0.39	-0.60	0.00
Q8CIZ8	von Willebrand factor OS=Mus musculus GN=Vwf PE=1 SV=2 - [VWF_MOUSE]	-0.53	-0.38	-0.57	-0.70	0.00
Q8C4X2	Casein kinase I isoform gamma-3 OS=Mus musculus GN=Csnk1g3 PE=1 SV=2 - [KC1G3_MOUSE]	0.23	0.32	0.18	0.27	0.00
Q3U0Y2	Uncharacterized protein ZMYM6NB OS=Mus musculus GN=Zmym6nb PE=2 SV=2 - [ZMYNB_MOUSE]	-0.28	-0.30	-0.42	-0.45	0.00
P10922	Histone H1.0 OS=Mus musculus GN=H1f0 PE=2 SV=4 - [H10_MOUSE]	-0.37	-0.24	-0.32	-0.23	0.00

Q9CQF3	Cleavage and polyadenylation specificity factor subunit 5 OS=Mus musculus GN=Nudt21 PE=2 SV=1 - [CPSF5_MOUSE]	-0.22	-0.15	-0.13	-0.16	0.00
Q8K1S3-2	Isoform 2 of Netrin receptor UNC5B OS=Mus musculus GN=Unc5b - [UNC5B_MOUSE]	0.43	0.62	0.37	0.60	0.00
Q8C4G3	Probable phospholipid-transporting ATPase IIA OS=Mus musculus GN=Atp9a PE=2 SV=1 [Q8C4G3_MOUSE]	-0.30	-0.45	-0.27	-0.41	0.00
Q99JR8-2	Isoform 2 of SWI/SNF-related matrix-associated actin-dependent regulator of chromatin subfamily D member 2 OS=Mus musculus GN=Smarcd2 - [SMRD2_MOUSE]	0.31	0.44	0.44	0.57	0.00
A2AJ72	MCG130458 OS=Mus musculus GN=Fubp3 PE=4 SV=1 - [A2AJ72_MOUSE]	0.24	0.23	0.21	0.13	0.00
Q62077	1-phosphatidylinositol 4,5-bisphosphate phosphodiesterase gamma-1 OS=Mus musculus GN=Plcg1 PE=1 SV=2 - [PLCG1_MOUSE]	-0.10	-0.18	-0.18	-0.14	0.00
F6WZB3	Kin of IRRE-like protein 2 (Fragment) OS=Mus musculus GN=Kirrel2 PE=4 SV=1 - [F6WZB3_MOUSE]	0.17	0.26	0.22	0.30	0.00
Q9DCJ9	N-acetylneuraminate lyase OS=Mus musculus GN=Npl PE=1 SV=1 - [NPL_MOUSE]	0.23	0.34	0.26	0.19	0.00
Q59J78	Mimitin, mitochondrial OS=Mus musculus GN=Ndufaf2 PE=2 SV=1 - [MIMIT_MOUSE]	-0.13	-0.10	-0.17	-0.18	0.00
P55002	Microfibrillar-associated protein 2 OS=Mus musculus GN=Mfap2 PE=2 SV=1 - [MFAP2_MOUSE]	-1.29	-1.57	-0.86	-1.14	0.00
O54774	AP-3 complex subunit delta-1 OS=Mus musculus GN=Ap3d1 PE=1 SV=1 - [AP3D1_MOUSE]	0.49	0.52	0.30	0.39	0.00
H3BK86	Peptide YY OS=Mus musculus GN=Pyy PE=3 SV=1 - [H3BK86_MOUSE]	-0.44	-0.34	-0.61	-0.50	0.00
Q8BG32	26S proteasome non-ATPase regulatory subunit 11 OS=Mus musculus GN=Psmd11 PE=1 SV=3 - [PSD11_MOUSE]	0.32	0.42	0.23	0.31	0.00

P19253	60S ribosomal protein L13a OS=Mus musculus GN=Rpl13a PE=1 SV=4 - [RL13A_MOUSE]	0.14	0.10	0.18	0.15	0.00
Q6NZQ4	PAX-interacting protein 1 OS=Mus musculus GN=Paxip1 PE=1 SV=1 - [PAXI1_MOUSE]	-0.13	-0.09	-0.11	-0.07	0.00
D3Z5G7	Protein Ces1b OS=Mus musculus GN=Ces1b PE=4 SV=1 - [D3Z5G7_MOUSE]	-0.63	-1.03	-0.69	-0.98	0.00
H7BX05	Obscurin OS=Mus musculus GN=Obscn PE=2 SV=2 - [H7BX05_MOUSE]	-0.17	-0.24	-0.13	-0.20	0.00
Q4VAE3	Transmembrane protein 65 OS=Mus musculus GN=Tmem65 PE=2 SV=1 - [TMM65_MOUSE]	0.09	0.10	0.14	0.15	0.00
O88533	Aromatic-L-amino-acid decarboxylase OS=Mus musculus GN=Ddc PE=2 SV=1 - [DDC_MOUSE]	-0.27	-0.37	-0.27	-0.43	0.00
Q9CS42	Ribose-phosphate pyrophosphokinase 2 OS=Mus musculus GN=Prps2 PE=1 SV=4 [PRPS2_MOUSE]	-0.15	-0.17	-0.22	-0.25	0.00
Q8BIQ6	MCG23335 OS=Mus musculus GN=Zfp947 PE=2 SV=1 - [Q8BIQ6_MOUSE]	-0.71	-0.83	-0.47	-0.59	0.00
Q9CU62	Structural maintenance of chromosomes protein 1A OS=Mus musculus GN=Smc1a PE=1 SV=4 - [SMC1A_MOUSE]	0.28	0.20	0.16	0.21	0.00
E9Q9Z6	Protein Olfr235 OS=Mus musculus GN=Olfr235 PE=2 SV=1 - [E9Q9Z6_MOUSE]	2.36	2.12	1.62	1.38	0.00
Q8VCT3	Aminopeptidase B OS=Mus musculus GN=Rnpep PE=2 SV=2 - [AMPB_MOUSE]	0.20	0.12	0.17	0.14	0.00
P59235	Nucleoporin Nup43 OS=Mus musculus GN=Nup43 PE=2 SV=2 - [NUP43_MOUSE]	-0.15	-0.18	-0.13	-0.22	0.00
O09159	Lysosomal alpha-mannosidase OS=Mus musculus GN=Man2b1 PE=2 SV=4 - [MA2B1_MOUSE]	-0.41	-0.55	-0.34	-0.58	0.00
Q62093	Serine/arginine-rich splicing factor 2 OS=Mus musculus GN=Srsf2 PE=1 SV=4 - [SRSF2_MOUSE]	-0.31	-0.31	-0.24	-0.18	0.00

E9PXV7	Protein diaphanous homolog 1 OS=Mus musculus GN=Diap1 PE=2 SV=1 - [E9PXV7_MOUSE]	0.29	0.34	0.18	0.26	0.00
O70370	Cathepsin S OS=Mus musculus GN=Ctss PE=2 SV=2 - [CATS_MOUSE]	-0.34	-0.22	-0.21	-0.29	0.00
Q99KR8	Plasma alpha-L-fucosidase OS=Mus musculus GN=Fuca2 PE=2 SV=1 - [FUCO2_MOUSE]	-0.33	-0.34	-0.20	-0.25	0.00
Q8K297	Procollagen galactosyltransferase 1 OS=Mus musculus GN=Colgalt1 PE=1 SV=2 - [GT251_MOUSE]	-0.43	-0.35	-0.23	-0.37	0.00
P60879	Synaptosomal-associated protein 25 OS=Mus musculus GN=Snap25 PE=1 SV=1 - [SNP25_MOUSE]	-0.60	-0.40	-0.35	-0.52	0.00
P62309	Small nuclear ribonucleoprotein G OS=Mus musculus GN=Snrpg PE=1 SV=1 [RUXG_MOUSE]	-0.48	-0.35	-0.54	-0.32	0.00
P31428	Dipeptidase 1 OS=Mus musculus GN=Dpep1 PE=1 SV=2 - [DPEP1_MOUSE]	-0.27	-0.45	-0.29	-0.35	0.00
O70589	Peripheral plasma membrane protein CASK OS=Mus musculus GN=Cask PE=1 SV=2 - [CSKP_MOUSE]	0.35	0.39	0.42	0.59	0.00
B1AR48	Elongator complex protein 5 (Fragment) OS=Mus musculus GN=Elp5 PE=2 SV=1 - [B1AR48_MOUSE]	0.90	1.20	0.65	0.95	0.00
Q8QZS1	3-hydroxyisobutyryl-CoA hydrolase, mitochondrial OS=Mus musculus GN=Hibch PE=1 SV=1 - [HIBCH_MOUSE]	0.23	0.21	0.13	0.17	0.00
O35226-2	Isoform Rpn10B of 26S proteasome non-ATPase regulatory subunit 4 OS=Mus musculus GN=Psmd4 - [PSMD4_MOUSE]	0.03	0.04	0.04	0.05	0.00
Q9QYB1	Chloride intracellular channel protein 4 OS=Mus musculus GN=Clic4 PE=1 SV=3 - [CLIC4_MOUSE]	0.32	0.48	0.29	0.45	0.00
Q8CFB8	NAD+ ADP-ribosyltransferase 3 PARP-3 OS=Mus musculus GN=Parp3 PE=2 SV=1 - [Q8CFB8_MOUSE]	0.46	0.42	0.46	0.25	0.00

P46718	Programmed cell death protein 2 OS=Mus musculus GN=Pdcd2 PE=2 SV=2 - [PDCD2_MOUSE]	0.37	0.50	0.27	0.40	0.00
Q07113	Cation-independent mannose-6-phosphate receptor OS=Mus musculus GN=Igf2r PE=1 SV=1 - [MPRI_MOUSE]	0.64	0.47	0.42	0.37	0.00
A2RSY6-3	Isoform 3 of TRMT1-like protein OS=Mus musculus GN=Trmt1l - [TRM1L_MOUSE]	0.31	0.30	0.48	0.46	0.00
Q5DTM8	E3 ubiquitin-protein ligase BRE1A OS=Mus musculus GN=Rnf20 PE=1 SV=2 - [BRE1A_MOUSE]	0.33	0.51	0.33	0.51	0.00
Q91X79	Chymotrypsin-like elastase family member 1 OS=Mus musculus GN=Cela1 PE=2 SV=1 - [CELA1_MOUSE]	1.52	0.93	1.58	1.11	0.00
O88207	Collagen alpha-1(V) chain OS=Mus musculus GN=Col5a1 PE=2 SV=2 - [CO5A1_MOUSE]	-0.33	-0.21	-0.34	-0.22	0.00
P52825	Carnitine O-palmitoyltransferase 2, mitochondrial OS=Mus musculus GN=Cpt2 PE=1 SV=2 - [CPT2_MOUSE]	-0.35	-0.38	-0.24	-0.23	0.00
Q62181	Semaphorin-3C OS=Mus musculus GN=Sema3c PE=1 SV=2 - [SEM3C_MOUSE]	-0.42	-0.31	-0.34	-0.23	0.00
Q8C2Q8	ATP synthase subunit gamma OS=Mus musculus GN=Atp5c1 PE=2 SV=1 - [Q8C2Q8_MOUSE]	-0.23	-0.17	-0.29	-0.18	0.00
Q3TZ86	C5a anaphylatoxin chemotactic receptor 1 OS=Mus musculus GN=C5ar1 PE=2 SV=1 - [Q3TZ86_MOUSE]	-0.83	-0.65	-0.62	-0.44	0.00
Q9EP71	Ankycorbin OS=Mus musculus GN=Rai14 PE=1 SV=1 - [RAI14_MOUSE]	-0.16	-0.20	-0.11	-0.18	0.00
P80314	T-complex protein 1 subunit beta OS=Mus musculus GN=Cct2 PE=1 SV=4 - [TCPB_MOUSE]	0.17	0.27	0.16	0.21	0.00
G3UX26	Voltage-dependent anion-selective channel protein 2 (Fragment) OS=Mus musculus GN=Vdac2 PE=4 SV=1 - [G3UX26_MOUSE]	-0.26	-0.19	-0.24	-0.15	0.00
E9QKA4	Serine/arginine repetitive matrix protein 1 OS=Mus musculus GN=Srrm1 PE=2 SV=1 - [E9QKA4_MOUSE]	0.25	0.30	0.20	0.17	0.00

Q3TWV4	AP-2 complex subunit mu OS=Mus musculus GN=Ap2m1 PE=2 SV=1 - [Q3TWV4_MOUSE]	-0.05	-0.08	-0.07	-0.05	0.00
Q9DAS9	Guanine nucleotide-binding protein G(I)/G(S)/G(O) subunit gamma-12 OS=Mus musculus GN=Gng12 PE=1 SV=3 - [GBG12_MOUSE]	-0.36	-0.23	-0.40	-0.28	0.00
Q6PDG5-2	Isoform 2 of SWI/SNF complex subunit SMARCC2 OS=Mus musculus GN=Smarcc2 - [SMRC2_MOUSE]	0.10	0.17	0.16	0.19	0.00
Q05D44	Eukaryotic translation initiation factor 5B OS=Mus musculus GN=Eif5b PE=1 SV=2 - [IF2P_MOUSE]	0.24	0.38	0.22	0.31	0.00
P56528	ADP-ribosyl cyclase 1 OS=Mus musculus GN=Cd38 PE=1 SV=2 - [CD38_MOUSE]	0.62	0.62	0.40	0.41	0.00
P08752	Guanine nucleotide-binding protein G(i) subunit alpha-2 OS=Mus musculus GN=Gnai2 PE=1 SV=5 [GNAI2_MOUSE]	-0.16	-0.20	-0.10	-0.17	0.00
P63054	Purkinje cell protein 4 OS=Mus musculus GN=Pcp4 PE=2 SV=2 - [PCP4_MOUSE]	-0.66	-0.39	-0.72	-0.57	0.00
Q8VDG5	Phosphopantothenate--cysteine ligase OS=Mus musculus GN=Ppcs PE=2 SV=1 - [PPCS_MOUSE]	-0.13	-0.15	-0.21	-0.13	0.00
F6QU40	Peroxisomal targeting signal 2 receptor (Fragment) OS=Mus musculus GN=Pex7 PE=4 SV=1 - [F6QU40_MOUSE]	0.27	0.21	0.38	0.32	0.00
O08583	THO complex subunit 4 OS=Mus musculus GN=Alyref PE=1 SV=3 [THOC4_MOUSE]	-0.43	-0.46	-0.40	-0.25	0.00
P20491	High affinity immunoglobulin epsilon receptor subunit gamma OS=Mus musculus GN=Fcer1g PE=1 SV=1 - [FCERG_MOUSE]	-0.14	-0.09	-0.13	-0.08	0.00
Q9CYA0	Cysteine-rich with EGF-like domain protein 2 OS=Mus musculus GN=Creld2 PE=2 SV=1 - [CREL2_MOUSE]	0.34	0.23	0.37	0.23	0.00
Q6NV83-3	Isoform 3 of U2 snRNP-associated SURP motif-containing protein OS=Mus musculus GN=U2surp - [SR140_MOUSE]	0.32	0.19	0.28	0.20	0.00

Q8C0J2-2	Isoform 2 of Autophagy-related protein 16-1 OS=Mus musculus GN=Atg16l1 - [A16L1_MOUSE]	-0.49	-0.55	-0.31	-0.38	0.00
Q9JJF3	Bifunctional lysine-specific demethylase and histidyl-hydroxylase NO66 OS=Mus musculus GN=No66 PE=1 SV=2 - [NO66_MOUSE]	-0.62	-0.63	-0.40	-0.41	0.00
Q9D0F3	Protein ERGIC-53 OS=Mus musculus GN=Lman1 PE=2 SV=1 - [LMAN1_MOUSE]	0.43	0.24	0.41	0.31	0.00
O54990-6	Isoform 6 of Prominin-1 OS=Mus musculus GN=Prom1 - [PROM1_MOUSE]	0.31	0.22	0.42	0.33	0.00
Q8R274	MCG142205 OS=Mus musculus GN=Vmn1r210 PE=4 SV=1 - [Q8R274_MOUSE]	-0.90	-1.11	-0.60	-0.81	0.00
Q99L04	Dehydrogenase/reductase SDR family member 1 OS=Mus musculus GN=Dhrs1 PE=2 SV=1 - [DHRS1_MOUSE]	0.11	0.18	0.12	0.19	0.00
Q3V384	Lactation elevated protein 1 OS=Mus musculus GN=Lace1 PE=2 SV=1 - [LACE1_MOUSE]	0.41	0.40	0.26	0.25	0.00
P18872-2	Isoform Alpha-2 of Guanine nucleotide-binding protein G(o) subunit alpha OS=Mus musculus GN=Gnao1 - [GNAO_MOUSE]	-0.35	-0.46	-0.24	-0.35	0.00
Q9CRB0	Sorting nexin-24 OS=Mus musculus GN=Snx24 PE=2 SV=1 - [SNX24_MOUSE]	0.62	0.40	0.62	0.40	0.00
P30677	Guanine nucleotide-binding protein subunit alpha-14 OS=Mus musculus GN=Gna14 PE=1 SV=2 - [GNA14_MOUSE]	0.33	0.28	0.33	0.18	0.00
P16294	Coagulation factor IX OS=Mus musculus GN=F9 PE=2 SV=3 - [FA9_MOUSE]	-0.35	-0.54	-0.41	-0.60	0.00
P26443	Glutamate dehydrogenase 1, mitochondrial OS=Mus musculus GN=Glud1 PE=1 SV=1 - [DHE3_MOUSE]	-0.24	-0.15	-0.27	-0.19	0.00
Q9JL62	Glycolipid transfer protein OS=Mus musculus GN=Gltp PE=2 SV=3 - [GLTP_MOUSE]	0.71	0.54	0.42	0.44	0.00
Q02248	Catenin beta-1 OS=Mus musculus GN=Ctnnb1 PE=1 SV=1 - [CTNB1_MOUSE]	0.12	0.09	0.06	0.09	0.00

Q5NC41	Protein 1810065E05Rik OS=Mus musculus GN=1810065E05Rik PE=4 SV=1 - [Q5NC41_MOUSE]	-0.62	-0.41	-0.79	-0.64	0.00
Q9JKK7	Tropomodulin-2 OS=Mus musculus GN=Tmod2 PE=1 SV=2 - [TMOD2_MOUSE]	-0.76	-0.51	-0.94	-0.66	0.00
Q80YC5	Coagulation factor XII OS=Mus musculus GN=F12 PE=2 SV=2 - [FA12_MOUSE]	-0.72	-0.48	-0.67	-0.42	0.00
Q9DCT1	1,5-anhydro-D-fructose reductase OS=Mus musculus GN=Akr1e2 PE=1 SV=1 - [AKCL2_MOUSE]	-0.46	-0.33	-0.60	-0.42	0.00
E9QPE7	Myosin-11 OS=Mus musculus GN=Myh11 PE=4 SV=1 - [E9QPE7_MOUSE]	0.32	0.25	0.45	0.42	0.00
Q9D8I1	Marginal zone B- and B1-cell-specific protein OS=Mus musculus GN=Mzb1 PE=1 SV=2 - [MZB1_MOUSE]	0.51	0.40	0.48	0.71	0.00
D3Z6B9	Mitochondrial 10-formyltetrahydrofolate dehydrogenase OS=Mus musculus GN=Aldh1l2 PE=2 SV=1 - [D3Z6B9_MOUSE]	0.53	0.33	0.64	0.54	0.00
Q6ZWR6-4	Isoform 4 of Nesprin-1 OS=Mus musculus GN=Syne1 - [SYNE1_MOUSE]	0.36	0.37	0.36	0.20	0.00
Q99K51	Plastin-3 OS=Mus musculus GN=Pls3 PE=1 SV=3 - [PLST_MOUSE]	0.18	0.14	0.21	0.26	0.00
Q9CYN9	Renin receptor OS=Mus musculus GN=Atp6ap2 PE=2 SV=2 - [RENR_MOUSE]	0.28	0.20	0.28	0.17	0.00
Q7TMB8	Cytoplasmic FMR1-interacting protein 1 OS=Mus musculus GN=Cyfip1 PE=1 SV=1 - [CYFP1_MOUSE]	0.24	0.21	0.30	0.17	0.00
Q7M739	Nuclear pore complex-associated intranuclear coiled-coil protein TPR OS=Mus musculus GN=Tpr PE=2 SV=1 - [Q7M739_MOUSE]	-0.16	-0.10	-0.14	-0.09	0.00
F2Z461	E3 ISG15--protein ligase Herc6 OS=Mus musculus GN=Herc6 PE=2 SV=1 - [HERC6_MOUSE]	-1.32	-1.07	-1.92	-1.67	0.00
Q5SSW2	Proteasome activator complex subunit 4 OS=Mus musculus GN=Psme4 PE=1 SV=1 - [PSME4_MOUSE]	-0.18	-0.28	-0.30	-0.19	0.00

P56392	Cytochrome c oxidase subunit 7A1, mitochondrial OS=Mus musculus GN=Cox7a1 PE=2 SV=1 - [CX7A1_MOUSE]	0.10	0.07	0.13	0.09	0.00
D3Z2J3	Putative ATP-dependent RNA helicase DHX30 OS=Mus musculus GN=Dhx30 PE=2 SV=1 - [D3Z2J3_MOUSE]	-0.61	-0.34	-0.40	-0.51	0.00
P07607	Thymidylate synthase OS=Mus musculus GN=Tyms PE=1 SV=1 - [TYSY_MOUSE]	0.93	0.66	0.85	0.51	0.00
Q9QZ41-2	Isoform 2 of Protein DBF4 homolog A OS=Mus musculus GN=Dbf4 - [DBF4A_MOUSE]	-0.41	-0.43	-0.60	-0.68	0.00
P27641	X-ray repair cross-complementing protein 5 OS=Mus musculus GN=Xrcc5 PE=2 SV=4 [XRCC5_MOUSE]	0.35	0.36	0.24	0.22	0.00
Q543M9	MCG14605, isoform CRA_c OS=Mus musculus GN=Trp53rk PE=2 SV=1 - [Q543M9_MOUSE]	0.28	0.29	0.26	0.15	0.00
G3UWG1	MCG115977 OS=Mus musculus GN=Gm10108 PE=3 SV=1 - [G3UWG1_MOUSE]	-0.41	-0.25	-0.47	-0.37	0.00
Q8BPM2-2	Isoform 2 of Mitogen-activated protein kinase kinase kinase kinase 5 OS=Mus musculus GN=Map4k5 - [M4K5_MOUSE]	0.31	0.49	0.39	0.57	0.00
Q99LI7	Cleavage stimulation factor subunit 3 OS=Mus musculus GN=Cstf3 PE=1 SV=1 - [CSTF3_MOUSE]	0.13	0.16	0.20	0.23	0.00
Q9DCM2	Glutathione S-transferase kappa 1 OS=Mus musculus GN=Gstk1 PE=1 SV=3 [GSTK1_MOUSE]	-0.28	-0.47	-0.28	-0.33	0.00
Q9ESB3	Histidine-rich glycoprotein OS=Mus musculus GN=Hrg PE=1 SV=2 - [HRG_MOUSE]	-0.72	-0.56	-1.04	-0.83	0.00
Q8BPB0	MOB kinase activator 1B OS=Mus musculus GN=Mob1b PE=1 SV=3 - [MOB1B_MOUSE]	0.44	0.83	0.62	0.70	0.00
Q8R3E3-2	Isoform 2 of WD repeat domain phosphoinositide-interacting protein 1 OS=Mus musculus GN=Wipi1 - [WIPI1_MOUSE]	0.48	0.44	0.32	0.28	0.00

Q8VE95	UPF0598 protein C8orf82 homolog OS=Mus musculus PE=2 SV=1 - [CH082_MOUSE]	-0.52	-0.97	-0.80	-0.94	0.00
G3UX45	Protein Urb2 OS=Mus musculus GN=Urb2 PE=2 SV=1 - [G3UX45_MOUSE]	0.41	0.36	0.63	0.55	0.00
Q9Z2W0	Aspartyl aminopeptidase OS=Mus musculus GN=Dnpep PE=2 SV=2 - [DNPEP_MOUSE]	-0.26	-0.42	-0.24	-0.31	0.00
Q99P72-3	Isoform 2 of Reticulon-4 OS=Mus musculus GN=Rtn4 - [RTN4_MOUSE]	0.22	0.35	0.24	0.36	0.00
O88393	Transforming growth factor beta receptor type 3 OS=Mus musculus GN=Tgfbr3 PE=1 SV=1 - [TGBR3_MOUSE]	-0.79	-0.50	-0.84	-0.55	0.00
Q8R2K1-2	Isoform 2 of Fucose mutarotase OS=Mus musculus GN=Fuom - [FUCM_MOUSE]	-0.24	-0.27	-0.14	-0.25	0.00
Q05BC3-2	Isoform 2 of Echinoderm microtubule-associated protein-like 1 OS=Mus musculus GN=Eml1 - [EMAL1_MOUSE]	0.14	0.12	0.17	0.22	0.00
Q9R1Z8	Vinexin OS=Mus musculus GN=Sorbs3 PE=1 SV=1 - [VINEX_MOUSE]	-0.18	-0.22	-0.27	-0.33	0.00
P82349	Beta-sarcoglycan OS=Mus musculus GN=Sgcb PE=1 SV=1 - [SGCB_MOUSE]	-0.49	-0.89	-0.63	-0.82	0.00
Q99LD8	N(G),N(G)-dimethylarginine dimethylaminohydrolase 2 OS=Mus musculus GN=Ddah2 PE=1 SV=1 - [DDAH2_MOUSE]	-0.62	-0.37	-0.61	-0.41	0.00
P12815	Programmed cell death protein 6 OS=Mus musculus GN=Pdcd6 PE=1 SV=2 - [PDCD6_MOUSE]	-0.18	-0.10	-0.17	-0.12	0.00
P62823	Ras-related protein Rab-3C OS=Mus musculus GN=Rab3c PE=1 SV=1 - [RAB3C_MOUSE]	-0.55	-0.42	-0.42	-0.29	0.00
Q9QZI9	Serine incorporator 3 OS=Mus musculus GN=Serinc3 PE=1 SV=2 - [SERC3_MOUSE]	0.61	0.53	0.35	0.39	0.00
Q8BH48-2	Isoform 2 of Ubiquitin-associated protein 1 OS=Mus musculus GN=Ubap1 - [UBAP1_MOUSE]	-0.12	-0.21	-0.21	-0.16	0.00

Q7TS75	APC membrane recruitment protein 1 OS=Mus musculus GN=Amer1 PE=2 SV=3 - [AMER1_MOUSE]	0.22	0.35	0.23	0.35	0.00
O78196	H-2 class II histocompatibility antigen, I-A beta chain OS=Mus musculus GN=H2-Eb1 PE=2 SV=1 - [O78196_MOUSE]	0.44	0.57	0.52	0.79	0.00
Q8C2T6	DNA polymerase alpha subunit B OS=Mus musculus GN=Pola2 PE=2 SV=1 - [Q8C2T6_MOUSE]	0.31	0.28	0.17	0.22	0.00
F6R8U9	Sulfatase-modifying factor 2 (Fragment) OS=Mus musculus GN=Sumf2 PE=4 SV=1 - [F6R8U9_MOUSE]	-0.33	-0.45	-0.23	-0.35	0.00
Q60902-4	Isoform 4 of Epidermal growth factor receptor substrate 15-like 1 OS=Mus musculus GN=Eps15l1 - [EP15R_MOUSE]	-0.26	-0.14	-0.24	-0.19	0.00
O70200	Allograft inflammatory factor 1 OS=Mus musculus GN=Aif1 PE=1 SV=1 - [AIF1_MOUSE]	-0.34	-0.20	-0.38	-0.32	0.00
Q9JMA9	Sodium- and chloride-dependent neutral and basic amino acid transporter B(0+) OS=Mus musculus GN=Slc6a14 PE=2 SV=1 - [S6A14_MOUSE]	0.06	0.09	0.09	0.11	0.00
Q9R1E6	Ectonucleotide pyrophosphatase/phosphodiesterase family member 2 OS=Mus musculus GN=Enpp2 PE=1 SV=3 - [ENPP2_MOUSE]	0.95	1.33	0.70	1.08	0.00
Q9D903	Probable rRNA-processing protein EBP2 OS=Mus musculus GN=Ebna1bp2 PE=2 SV=1 - [EBP2_MOUSE]	0.31	0.37	0.20	0.25	0.00
P68037	Ubiquitin-conjugating enzyme E2 L3 OS=Mus musculus GN=Ube2l3 PE=2 SV=1 - [UB2L3_MOUSE]	0.22	0.42	0.31	0.38	0.00
Q3URE1	Acyl-CoA synthetase family member 3, mitochondrial OS=Mus musculus GN=Acsf3 PE=2 SV=2 - [ACSF3_MOUSE]	-0.16	-0.30	-0.30	-0.30	0.00
Q3UXD9	Peroxisomal trans-2-enoyl-CoA reductase OS=Mus musculus GN=Pecr PE=2 SV=1 - [Q3UXD9_MOUSE]	-0.16	-0.24	-0.27	-0.17	0.00

Q71M36-3	Isoform 3 of Chondroitin sulfate proteoglycan 5 OS=Mus musculus GN=Cspg5 - [CSPG5_MOUSE]	1.21	1.92	1.30	2.01	0.00
E0CY52	Uncharacterized protein OS=Mus musculus GN=Slc25a54 PE=2 SV=1 - [E0CY52_MOUSE]	1.23	1.08	1.87	1.72	0.00
P02468	Laminin subunit gamma-1 OS=Mus musculus GN=Lamc1 PE=1 SV=2 - [LAMC1_MOUSE]	0.18	0.13	0.25	0.19	0.00
Q9JIY8	Probable N-acetyltransferase CML3 OS=Mus musculus GN=Cml3 PE=2 SV=1 - [CMLO3_MOUSE]	1.97	1.32	1.75	1.10	0.00
P62900	60S ribosomal protein L31 OS=Mus musculus GN=Rpl31 PE=2 SV=1 [RL31_MOUSE]	-0.35	-0.28	-0.19	-0.25	0.00
Q8BRV5	Uncharacterized protein KIAA1671 OS=Mus musculus GN=Kiaa1671 PE=2 SV=1 - [K1671_MOUSE]	0.57	0.72	0.37	0.52	0.00
Q8CJF7	Protein ELYS OS=Mus musculus GN=Ahctf1 PE=1 SV=1 - [ELYS_MOUSE]	-0.79	-0.86	-1.26	-1.34	0.00
Q9CR68	Cytochrome b-c1 complex subunit Rieske, mitochondrial OS=Mus musculus GN=Uqcrfs1 PE=1 SV=1 - [UCRI_MOUSE]	-0.30	-0.16	-0.28	-0.22	0.00
Q9CPU4	Microsomal glutathione S-transferase 3 OS=Mus musculus GN=Mgst3 PE=1 SV=1 - [MGST3_MOUSE]	-0.11	-0.11	-0.17	-0.17	0.00
O08917	Flotillin-1 OS=Mus musculus GN=Flot1 PE=1 SV=1 - [FLOT1_MOUSE]	-0.26	-0.39	-0.22	-0.26	0.00
Q9DCZ1	GMP reductase 1 OS=Mus musculus GN=Gmpr PE=2 SV=1 [GMPR1_MOUSE]	-0.27	-0.44	-0.29	-0.45	0.00
Q99KV1	DnaJ homolog subfamily B member 11 OS=Mus musculus GN=Dnajb11 PE=1 SV=1 - [DJB11_MOUSE]	0.44	0.56	0.30	0.54	0.00
Q9Z2N8	Actin-like protein 6A OS=Mus musculus GN=Actl6a PE=1 SV=2 - [ACL6A_MOUSE]	-0.09	-0.14	-0.14	-0.18	0.00

Q9Z0H3-2	Isoform B of SWI/SNF-related matrix-associated actin-dependent regulator of chromatin subfamily B member 1 OS=Mus musculus GN=Smarcb1 - [SNF5_MOUSE]	0.35	0.26	0.28	0.18	0.00
O89017	Legumain OS=Mus musculus GN=Lgmn PE=1 SV=1 - [LGMN_MOUSE]	-0.42	-0.54	-0.27	-0.43	0.00
P70257-2	Isoform NFIX2 of Nuclear factor 1 X-type OS=Mus musculus GN=Nfix - [NFIX_MOUSE]	1.13	1.36	0.72	0.95	0.00
Q3UHJ0-2	Isoform 2 of AP2-associated protein kinase 1 OS=Mus musculus GN=Aak1 - [AAK1_MOUSE]	-0.19	-0.14	-0.17	-0.26	0.00
Q8R3K3	Pentatricopeptide repeat-containing protein 2, mitochondrial OS=Mus musculus GN=Ptcd2 PE=2 SV=1 [PTCD2_MOUSE]	0.58	0.60	0.36	0.39	0.00
Q5SQP1-2	Isoform 2 of HORMA domain-containing protein 2 OS=Mus musculus GN=Hormad2 - [HORM2_MOUSE]	-0.58	-0.78	-0.40	-0.60	0.00
Q02780-6	Isoform 6 of Nuclear factor 1 A-type OS=Mus musculus GN=Nfia - [NFIA_MOUSE]	0.54	0.87	0.59	0.92	0.00
P00375	Dihydrofolate reductase OS=Mus musculus GN=Dhfr PE=1 SV=3 - [DYR_MOUSE]	0.33	0.54	0.45	0.63	0.00
O35566	CD151 antigen OS=Mus musculus GN=Cd151 PE=2 SV=2 - [CD151_MOUSE]	0.40	0.58	0.31	0.49	0.00
G3UXK3	Protein 2410137M14Rik OS=Mus musculus GN=2410137M14Rik PE=2 SV=1 - [G3UXK3_MOUSE]	1.17	0.86	0.92	0.60	0.00
Q62312-2	Isoform RII-1 of TGF-beta receptor type-2 OS=Mus musculus GN=Tgfbr2 - [TGFR2_MOUSE]	-0.64	-0.52	-0.45	-0.33	0.00
A2AGT5-2	Isoform 2 of Cytoskeleton-associated protein 5 OS=Mus musculus GN=Ckap5 - [CKAP5_MOUSE]	0.49	0.31	0.52	0.33	0.00
Q7TPV4	Myb-binding protein 1A OS=Mus musculus GN=Mybbp1a PE=1 SV=2 - [MBB1A_MOUSE]	0.32	0.23	0.42	0.27	0.00

O88312	Anterior gradient protein 2 homolog OS=Mus musculus GN=Agr2 PE=2 SV=1 - [AGR2_MOUSE]	0.17	0.29	0.18	0.26	0.00
Q8VED2	Biogenesis of lysosome related organelles complex 1 subunit 4 OS=Mus musculus GN=Bloc1s4 PE=1 SV=1 - [BL1S4_MOUSE]	0.15	0.21	0.11	0.17	0.00
Q5U430	E3 ubiquitin-protein ligase UBR3 OS=Mus musculus GN=Ubr3 PE=1 SV=3 - [UBR3_MOUSE]	0.27	0.36	0.44	0.52	0.00
Q62293	Interferon-gamma-inducible GTPase Ifggb5 protein OS=Mus musculus GN=Tgtp PE=2 SV=1 - [Q62293_MOUSE]	0.61	0.40	0.59	0.35	0.00
E9PYL9	Uncharacterized protein OS=Mus musculus GN=Gm10036 PE=3 SV=1 - [E9PYL9_MOUSE]	0.12	0.15	0.16	0.22	0.00
Q6Q899	Probable ATP-dependent RNA helicase DDX58 OS=Mus musculus GN=Ddx58 PE=1 SV=2 - [DDX58_MOUSE]	0.60	0.49	0.43	0.77	0.00
F6QKK2	ADP-ribosylation factor-like protein 8A (Fragment) OS=Mus musculus GN=Arl8a PE=4 SV=1 - [F6QKK2_MOUSE]	-0.37	-0.29	-0.27	-0.19	0.00
Q8R3B1	1-phosphatidylinositol 4,5-bisphosphate phosphodiesterase delta-1 OS=Mus musculus GN=Plcd1 PE=2 SV=2 - [PLCD1_MOUSE]	-0.22	-0.15	-0.21	-0.12	0.00
O35326	Serine/arginine-rich splicing factor 5 OS=Mus musculus GN=Srsf5 PE=1 SV=2 - [SRSF5_MOUSE]	0.20	0.24	0.13	0.25	0.00
E9PVB3	Coiled-coil domain-containing protein 175 OS=Mus musculus GN=Ccdc175 PE=2 SV=1 - [CC175_MOUSE]	0.42	0.32	0.32	0.22	0.00
P61028	Ras-related protein Rab-8B OS=Mus musculus GN=Rab8b PE=1 SV=1 - [RAB8B_MOUSE]	-0.19	-0.15	-0.19	-0.28	0.00
E9Q9C7	Actin-binding LIM protein 1 OS=Mus musculus GN=Ablim1 PE=2 SV=1 - [E9Q9C7_MOUSE]	-0.49	-0.55	-0.34	-0.32	0.00
Q6ZWY3	40S ribosomal protein S27-like OS=Mus musculus GN=Rps27l PE=2 SV=3 - [RS27L_MOUSE]	0.61	0.90	0.47	0.68	0.00

Q6PE01	U5 small nuclear ribonucleoprotein 40 kDa protein OS=Mus musculus GN=Snrnp40 PE=2 SV=1 - [SNR40_MOUSE]	-0.37	-0.41	-0.38	-0.62	0.00
Q542I8	Integrin beta OS=Mus musculus GN=Itgb2 PE=2 SV=1 - [Q542I8_MOUSE]	0.11	0.13	0.07	0.14	0.00
P08553	Neurofilament medium polypeptide OS=Mus musculus GN=Nefm PE=1 SV=4 - [NFM_MOUSE]	-0.82	-0.77	-0.56	-0.45	0.00
Q60648	Ganglioside GM2 activator OS=Mus musculus GN=Gm2a PE=1 SV=2 - [SAP3_MOUSE]	-0.20	-0.37	-0.26	-0.35	0.00
Q80UY1	UPF0586 protein C9orf41 homolog OS=Mus musculus PE=2 SV=1 - [CI041_MOUSE]	-0.15	-0.16	-0.10	-0.19	0.00
Q9Z2F2	2'-5'-oligoadenylate synthase-like protein 2 OS=Mus musculus GN=Oasl2 PE=1 SV=2 [OASL2_MOUSE]	0.58	0.82	0.43	0.64	0.00
Q8K3X6	Ankyrin repeat and SAM domain-containing protein 4B OS=Mus musculus GN=Anks4b PE=1 SV=1 - [ANS4B_MOUSE]	0.07	0.08	0.12	0.12	0.00
Q9JHW4	Selenocysteine-specific elongation factor OS=Mus musculus GN=Eefsec PE=2 SV=2 - [SELB_MOUSE]	-0.18	-0.26	-0.14	-0.22	0.00
A6ZI47	Fructose-bisphosphate aldolase OS=Mus musculus GN=Aldoart2 PE=2 SV=1 - [A6ZI47_MOUSE]	0.13	0.11	0.20	0.17	0.00
D3YX27	Serine protease HTRA2 mitochondrial OS=Mus musculus GN=Htra2 PE=2 SV=1 - [D3YX27_MOUSE]	-0.44	-0.44	-0.32	-0.25	0.00
Q8K0P3	TLD domain-containing protein 1 OS=Mus musculus GN=Tldc1 PE=2 SV=1 - [TLDC1_MOUSE]	-0.20	-0.36	-0.24	-0.25	0.00
Q8BWU5	Probable tRNA threonylcarbamoyladenosine biosynthesis protein Osgep OS=Mus musculus GN=Osgep PE=2 SV=2 - [OSGEP_MOUSE]	0.20	0.19	0.23	0.32	0.00
Q9Z1N5	Spliceosome RNA helicase Ddx39b OS=Mus musculus GN=Ddx39b PE=1 SV=1 - [DX39B_MOUSE]	0.20	0.30	0.23	0.36	0.00

Q9CZD3	Glycine--tRNA ligase OS=Mus musculus GN=Gars PE=1 SV=1 - [SYG_MOUSE]	0.26	0.24	0.16	0.16	0.00
Q9QZ85	Interferon-inducible GTPase 1 OS=Mus musculus GN=Iigp1 PE=1 SV=2 - [IIGP1_MOUSE]	0.26	0.14	0.27	0.23	0.00
Q64282	Interferon-induced protein with tetratricopeptide repeats 1 OS=Mus musculus GN=Ifit1 PE=1 SV=2 - [IFIT1_MOUSE]	0.33	0.42	0.21	0.37	0.00
Q9CYG7	Mitochondrial import receptor subunit TOM34 OS=Mus musculus GN=Tomm34 PE=2 SV=1 - [TOM34_MOUSE]	0.22	0.19	0.34	0.25	0.00
P21279	Guanine nucleotide-binding protein G(q) subunit alpha OS=Mus musculus GN=Gnaq PE=1 SV=4 - [GNAQ_MOUSE]	-0.19	-0.23	-0.16	-0.29	0.00
Q8BWY7-3	Isoform 3 of Zinc transporter ZIP11 OS=Mus musculus GN=Slc39a11 - [S39AB_MOUSE]	0.33	0.22	0.43	0.32	0.00
P32233	Developmentally-regulated GTP-binding protein 1 OS=Mus musculus GN=Drg1 PE=1 SV=1 - [DRG1_MOUSE]	0.30	0.15	0.25	0.29	0.00
P06837	Neuromodulin OS=Mus musculus GN=Gap43 PE=1 SV=1 - [NEUM_MOUSE]	-0.57	-0.37	-0.73	-0.57	0.00
Q9D958	Signal peptidase complex subunit 1 OS=Mus musculus GN=Spcs1 PE=2 SV=3 - [SPCS1_MOUSE]	0.79	1.10	0.56	0.84	0.00
P03976	Ig kappa chain V-II region 17S29.1 OS=Mus musculus PE=1 SV=1 - [KV2A5_MOUSE]	-0.65	-0.90	-0.46	-0.71	0.00
Q6ZWU9	40S ribosomal protein S27 OS=Mus musculus GN=Rps27 PE=1 SV=3 - [RS27_MOUSE]	0.33	0.36	0.23	0.45	0.00
Q99LJ0	CTTNBP2 N-terminal-like protein OS=Mus musculus GN=Cttnbp2nl PE=1 SV=1 - [CT2NL_MOUSE]	0.25	0.38	0.20	0.28	0.01
Q8VEJ9	Vacuolar protein sorting associated protein 4A OS=Mus musculus GN=Vps4a PE=1 SV=1 - [VPS4A_MOUSE]	0.31	0.24	0.45	0.40	0.01

Q91WK2	Eukaryotic translation initiation factor 3 subunit H OS=Mus musculus GN=Eif3h PE=1 SV=1 - [EIF3H_MOUSE]	0.22	0.36	0.23	0.36	0.01
Q8BQ47	Protein canopy homolog 4 OS=Mus musculus GN=Cnpy4 PE=1 SV=1 - [CNPY4_MOUSE]	-0.49	-0.45	-0.30	-0.29	0.01
O89116	Vesicle transport through interaction with t-SNAREs homolog 1A OS=Mus musculus GN=Vti1a PE=1 SV=1 - [VTI1A_MOUSE]	0.18	0.16	0.28	0.25	0.01
P35436	Glutamate receptor ionotropic, NMDA 2A OS=Mus musculus GN=Grin2a PE=1 SV=2 - [NMDE1_MOUSE]	0.93	1.45	0.82	1.34	0.01
Q99P91	Transmembrane glycoprotein NMB OS=Mus musculus GN=Gpnmb PE=1 SV=2 - [GPNMB_MOUSE]	-1.40	-1.08	-1.02	-0.71	0.01
P46467	Vacuolar protein sorting associated protein 4B OS=Mus musculus GN=Vps4b PE=1 SV=2 - [VPS4B_MOUSE]	0.32	0.49	0.26	0.38	0.01
Q78JE5	F-box only protein 22 OS=Mus musculus GN=Fbxo22 PE=2 SV=2 - [FBX22_MOUSE]	0.55	0.36	0.70	0.51	0.01
Q80U56	Late secretory pathway protein AVL9 homolog OS=Mus musculus GN=Avl9 PE=2 SV=2 - [AVL9_MOUSE]	-0.31	-0.19	-0.35	-0.23	0.01
Q8JZQ2	AFG3-like protein 2 OS=Mus musculus GN=Afg3l2 PE=1 SV=1 - [AFG32_MOUSE]	0.18	0.28	0.15	0.24	0.01
Q61644	Protein kinase C and casein kinase substrate in neurons protein 1 OS=Mus musculus GN=Pacsin1 PE=1 SV=1 - [PACN1_MOUSE]	0.85	1.29	0.70	1.13	0.01
B1AWQ3	Espin OS=Mus musculus GN=Espn PE=2 SV=1 - [B1AWQ3_MOUSE]	-0.82	-0.49	-0.86	-0.57	0.01
D3YTP0	Metalloreductase STEAP3 (Fragment) OS=Mus musculus GN=Steap3 PE=2 SV=1 - [D3YTP0_MOUSE]	0.20	0.33	0.31	0.40	0.01
Q99J83	Autophagy protein 5 OS=Mus musculus GN=Atg5 PE=1 SV=1 - [ATG5_MOUSE]	0.71	1.07	0.58	0.94	0.01

Q69ZN7	Myoferlin OS=Mus musculus GN=Myof PE=1 SV=2 - [MYOF_MOUSE]	0.10	0.05	0.08	0.09	0.01
Q8R2Y8	Peptidyl-tRNA hydrolase 2, mitochondrial OS=Mus musculus GN=Ptrh2 PE=2 SV=1 - [PTH2_MOUSE]	0.18	0.33	0.35	0.29	0.01
Q9DAA4	Probable inactive serine protease 37 OS=Mus musculus GN=Prss37 PE=2 SV=1 - [PRS37_MOUSE]	-0.61	-0.38	-0.62	-0.39	0.01
Q9CVB6	Actin-related protein 2/3 complex subunit 2 OS=Mus musculus GN=Arpc2 PE=1 SV=3 [ARPC2_MOUSE]	0.12	0.18	0.14	0.23	0.01
Q922B9-3	Isoform 3 of Sperm-specific antigen 2 homolog OS=Mus musculus GN=Ssfa2 - [SSFA2_MOUSE]	-0.26	-0.26	-0.41	-0.43	0.01
Q9CWJ9	Bifunctional purine biosynthesis protein PURH OS=Mus musculus GN=Atic PE=1 SV=2 - [PUR9_MOUSE]	-0.12	-0.20	-0.12	-0.19	0.01
Q99L45	Eukaryotic translation initiation factor 2 subunit 2 OS=Mus musculus GN=Eif2s2 PE=1 SV=1 - [IF2B_MOUSE]	0.41	0.50	0.28	0.56	0.01
Q922F4	Tubulin beta-6 chain OS=Mus musculus GN=Tubb6 PE=1 SV=1 - [TBB6_MOUSE]	0.31	0.27	0.38	0.19	0.01
Q80ZI6	E3 ubiquitin-protein ligase LRSAM1 OS=Mus musculus GN=Lrsam1 PE=2 SV=1 - [LRSM1_MOUSE]	-0.65	-0.88	-1.03	-1.27	0.01
Q80WK2	Organic solute transporter subunit beta OS=Mus musculus GN=Slc51b PE=1 SV=1 - [OSTB_MOUSE]	-1.47	-0.93	-1.40	-0.85	0.01
O88870	Bestrophin-1 OS=Mus musculus GN=Best1 PE=2 SV=3 - [BEST1_MOUSE]	0.36	0.43	0.22	0.29	0.01
Q8C3V4	Signal transducer and activator of transcription 1 OS=Mus musculus GN=Stat1 PE=2 SV=1 - [Q8C3V4_MOUSE]	0.22	0.13	0.23	0.16	0.01
Q62266	Cornifin-A OS=Mus musculus GN=Sprr1a PE=2 SV=1 - [SPR1A_MOUSE]	0.36	0.40	0.59	0.63	0.01
D3Z7C0	39S ribosomal protein L40, mitochondrial OS=Mus musculus GN=Mrpl40 PE=2 SV=1 - [D3Z7C0_MOUSE]	0.60	0.49	0.30	0.46	0.01

E9QLX1	Antigen-presenting glycoprotein CD1d2 OS=Mus musculus GN=Cd1d2 PE=4 SV=2 - [E9QLX1_MOUSE]	-0.62	-0.37	-0.68	-0.44	0.01
Q3UYV9	Nuclear cap-binding protein subunit 1 OS=Mus musculus GN=Ncbp1 PE=1 SV=2 - [NCBP1_MOUSE]	0.58	0.58	0.87	0.48	0.01
Q61335	B-cell receptor-associated protein 31 OS=Mus musculus GN=Bcap31 PE=1 SV=4 - [BAP31_MOUSE]	0.17	0.22	0.12	0.14	0.01
Q9D832	DnaJ homolog subfamily B member 4 OS=Mus musculus GN=Dnajb4 PE=2 SV=1 - [DNJB4_MOUSE]	0.26	0.33	0.17	0.22	0.01
Q91YR1	Twinfilin-1 OS=Mus musculus GN=Twf1 PE=1 SV=2 - [TWF1_MOUSE]	0.13	0.17	0.09	0.11	0.01
Q9CR89-2	Isoform 2 of Endoplasmic reticulum-Golgi intermediate compartment protein 2 OS=Mus musculus GN=Ergic2 - [ERGI2_MOUSE]	0.71	0.44	0.87	0.61	0.01
Q7TNC9	Inpp5a protein OS=Mus musculus GN=Inpp5a PE=2 SV=1 - [Q7TNC9_MOUSE]	-0.29	-0.17	-0.16	-0.22	0.01
D3YVX2	Coiled-coil domain-containing protein 7 (Fragment) OS=Mus musculus GN=Ccdc7 PE=2 SV=1 - [D3YVX2_MOUSE]	0.52	0.58	0.29	0.42	0.01
Q9EQF5	Dihydropyrimidinase OS=Mus musculus GN=Dpys PE=2 SV=2 - [DPYS_MOUSE]	-0.16	-0.10	-0.17	-0.10	0.01
P46460	Vesicle-fusing ATPase OS=Mus musculus GN=Nsf PE=1 SV=2 - [NSF_MOUSE]	0.34	0.35	0.18	0.31	0.01
Q5U3K5	Rab-like protein 6 OS=Mus musculus GN=Rabl6 PE=1 SV=2 [RABL6_MOUSE]	0.08	0.10	0.12	0.15	0.01
Q8BHX1	HAUS augmin-like complex subunit 1 OS=Mus musculus GN=Haus1 PE=2 SV=2 - [HAUS1_MOUSE]	0.34	0.31	0.20	0.20	0.01
O55091	Protein IMPACT OS=Mus musculus GN=Impact PE=1 SV=2 - [IMPCT_MOUSE]	0.15	0.11	0.12	0.08	0.01
Q80YA3-2	Isoform 2 of Phospholipase DDHD1 OS=Mus musculus GN=Ddhd1 - [DDHD1_MOUSE]	1.25	0.82	1.12	0.68	0.01

Q8K2X3	CST complex subunit STN1 OS=Mus musculus GN=Obfc1 PE=1 SV=2 - [STN1_MOUSE]	-0.45	-0.71	-0.39	-0.64	0.01
Q9D3D9	ATP synthase subunit delta, mitochondrial OS=Mus musculus GN=Atp5d PE=1 SV=1 [ATPD_MOUSE]	-0.38	-0.26	-0.50	-0.34	0.01
Q80ZP8	Armet protein OS=Mus musculus GN=Manf PE=2 SV=1 - [Q80ZP8_MOUSE]	0.35	0.50	0.25	0.40	0.01
Q9WVQ5	Methylthioribulose-1-phosphate dehydratase OS=Mus musculus GN=Apip PE=1 SV=1 - [MTNB_MOUSE]	-0.17	-0.12	-0.13	-0.09	0.01
Q8BT07	Centrosomal protein of 55 kDa OS=Mus musculus GN=Cep55 PE=1 SV=2 - [CEP55_MOUSE]	-0.22	-0.24	-0.13	-0.15	0.01
F6VVW4	Actin-binding LIM protein 2 (Fragment) OS=Mus musculus GN=Ablim2 PE=4 SV=1 - [F6VVW4_MOUSE]	-0.66	-0.88	-0.46	-0.57	0.01
Q9JJ66	Cell division cycle protein 20 homolog OS=Mus musculus GN=Cdc20 PE=1 SV=2 - [CDC20_MOUSE]	0.82	0.65	0.58	0.41	0.01
Q5U4C2	Dipeptidyl aminopeptidase-like protein 6 OS=Mus musculus GN=Dpp6 PE=2 SV=1 - [Q5U4C2_MOUSE]	-0.48	-0.32	-0.64	-0.48	0.01
G5E8C3	G protein-coupled receptor, family C, group 5, member A OS=Mus musculus GN=Gprc5a PE=4 SV=1 - [G5E8C3_MOUSE]	0.55	0.32	0.45	0.31	0.01
D3YXQ4	Beta-chimaerin (Fragment) OS=Mus musculus GN=Chn2 PE=2 SV=1 - [D3YXQ4_MOUSE]	-1.54	-1.14	-2.24	-1.84	0.01
Q8VEA4	Mitochondrial intermembrane space import and assembly protein 40 OS=Mus musculus GN=Chchd4 PE=1 SV=1 - [MIA40_MOUSE]	-0.21	-0.24	-0.34	-0.38	0.01
Q3UT76	Protein Zfp957 OS=Mus musculus GN=Zfp957 PE=2 SV=1 - [Q3UT76_MOUSE]	-1.48	-0.95	-1.34	-0.81	0.01
Q80X90	Filamin-B OS=Mus musculus GN=Flnb PE=1 SV=3 - [FLNB_MOUSE]	0.12	0.18	0.09	0.13	0.01

B9EJ54	MCG21756, isoform CRA_b OS=Mus musculus GN=Nup205 PE=2 SV=1 - [B9EJ54_MOUSE]	0.37	0.32	0.24	0.19	0.01
P10404	MLV-related proviral Env polyprotein OS=Mus musculus PE=2 SV=3 - [ENV1_MOUSE]	0.60	0.45	0.64	0.34	0.01
Q9Z0R9	Fatty acid desaturase 2 OS=Mus musculus GN=Fads2 PE=2 SV=1 - [FADS2_MOUSE]	0.74	0.55	0.55	0.36	0.01
D3YXA1	Lebercilin (Fragment) OS=Mus musculus GN=Lca5 PE=2 SV=1 - [D3YXA1_MOUSE]	0.67	0.52	1.00	0.86	0.01
Q922P9	Putative oxidoreductase GLYR1 OS=Mus musculus GN=Glyr1 PE=2 SV=1 - [GLYR1_MOUSE]	0.22	0.23	0.12	0.17	0.01
Q9JKC6	Cell cycle exit and neuronal differentiation protein 1 OS=Mus musculus GN=Cend1 PE=1 SV=1 - [CEND_MOUSE]	-0.60	-0.36	-0.67	-0.44	0.01
Q11127-2	Isoform Short of Alpha-(1,3)-fucosyltransferase OS=Mus musculus GN=Fut4 - [FUT4_MOUSE]	0.30	0.28	0.49	0.33	0.01
F8WIX8	Histone H2A OS=Mus musculus GN=Hist1h2al PE=2 SV=1 - [F8WIX8_MOUSE]	-0.91	-0.86	-0.66	-0.47	0.01
Q8BT51	Cytochrome c oxidase assembly factor 4 homolog, mitochondrial OS=Mus musculus GN=COA4 PE=3 SV=1 - [COA4_MOUSE]	-0.15	-0.12	-0.07	-0.12	0.01
Q921H8	3-ketoacyl-CoA thiolase A, peroxisomal OS=Mus musculus GN=Acaa1a PE=2 SV=1 - [THIKA_MOUSE]	-0.24	-0.15	-0.27	-0.16	0.01
Q8VCZ6	Small G protein signaling modulator 3 OS=Mus musculus GN=Sgsm3 PE=1 SV=1 - [SGSM3_MOUSE]	0.14	0.16	0.26	0.19	0.01
E9Q4P1	Protein Wdfy1 OS=Mus musculus GN=Wdfy1 PE=4 SV=1 - [E9Q4P1_MOUSE]	0.34	0.27	0.32	0.17	0.01
P97478	Ubiquinone biosynthesis protein COQ7 homolog OS=Mus musculus GN=Coq7 PE=2 SV=3 - [COQ7_MOUSE]	0.20	0.27	0.36	0.38	0.01

Q9CR13	UPF0562 protein C7orf55 homolog OS=Mus musculus PE=3 SV=1 - [CG055_MOUSE]	-0.46	-0.76	-0.60	-0.90	0.01
Q99JI4	26S proteasome non-ATPase regulatory subunit 6 OS=Mus musculus GN=Psmd6 PE=1 SV=1 - [PSMD6_MOUSE]	0.24	0.19	0.33	0.36	0.01
Q99MV1	Tudor domain-containing protein 1 OS=Mus musculus GN=Tdrd1 PE=1 SV=2 [TDRD1_MOUSE]	-0.33	-0.56	-0.33	-0.50	0.01
Q62167	ATP-dependent RNA helicase DDX3X OS=Mus musculus GN=Ddx3x PE=1 SV=3 - [DDX3X_MOUSE]	0.32	0.36	0.17	0.29	0.01
Q9D906	Ubiquitin-like modifier-activating enzyme ATG7 OS=Mus musculus GN=Atg7 PE=1 SV=1 - [ATG7_MOUSE]	0.51	0.44	0.33	0.26	0.01
Q9D136	2-oxoglutarate and iron-dependent oxygenase domain-containing protein 3 OS=Mus musculus GN=Ogfod3 PE=2 SV=1 - [OGFD3_MOUSE]	0.17	0.17	0.28	0.27	0.01
A2A5F7	Potassium voltage-gated channel, subfamily H (Eag-related), member 4 OS=Mus musculus GN=Kcnh4 PE=2 SV=1 - [A2A5F7_MOUSE]	-1.97	-2.96	-1.52	-2.51	0.01
D3Z734	E3 ubiquitin-protein ligase RAD18 OS=Mus musculus GN=Rad18 PE=2 SV=1 - [D3Z734_MOUSE]	-0.41	-0.30	-0.60	-0.49	0.01
G3UYF9	Prefoldin subunit 6 OS=Mus musculus GN=H2-Ke2 PE=2 SV=1 - [G3UYF9_MOUSE]	-0.11	-0.10	-0.18	-0.17	0.01
Q06890	Clusterin OS=Mus musculus GN=Clu PE=1 SV=1 - [CLUS_MOUSE]	-0.41	-0.39	-0.30	-0.21	0.01
Q93092	Transaldolase OS=Mus musculus GN=Taldo1 PE=1 SV=2 - [TALDO_MOUSE]	-0.31	-0.19	-0.35	-0.22	0.01
Q80WV7-2	Isoform 2 of Serine/arginine repetitive matrix protein 3 OS=Mus musculus GN=Srrm3 - [SRRM3_MOUSE]	0.27	0.45	0.29	0.46	0.01
Q91VX9	Transmembrane protein 168 OS=Mus musculus GN=Tmem168 PE=2 SV=1 - [TM168_MOUSE]	0.61	0.37	0.75	0.52	0.01

Q9CZE3	Ras-related protein Rab-32 OS=Mus musculus GN=Rab32 PE=2 SV=3 - [RAB32_MOUSE]	0.13	0.11	0.10	0.06	0.01
H7BWZ1	Ubiquitin-fold modifier 1 OS=Mus musculus GN=Ufm1 PE=2 SV=1 - [H7BWZ1_MOUSE]	0.15	0.08	0.16	0.11	0.01
Q9Z2Y8	Proline synthase co-transcribed bacterial homolog protein OS=Mus musculus GN=Prosc PE=1 SV=1 [PROSC_MOUSE]	-0.27	-0.14	-0.18	-0.21	0.01
Q8R016	Bleomycin hydrolase OS=Mus musculus GN=Blmh PE=2 SV=1 - [BLMH_MOUSE]	0.16	0.16	0.09	0.18	0.01
Q8VC49	Interferon alpha-inducible protein 27-like protein 2B OS=Mus musculus GN=Ifi27l2b PE=2 SV=1 - [IF27B_MOUSE]	-0.17	-0.19	-0.18	-0.09	0.01
Q8BKE6	Cytochrome P450 20A1 OS=Mus musculus GN=Cyp20a1 PE=2 SV=1 - [CP20A_MOUSE]	0.22	0.26	0.26	0.41	0.01
P59113	Fermitin family homolog 1 OS=Mus musculus GN=Fermt1 PE=1 SV=4 - [FERM1_MOUSE]	0.53	0.32	0.61	0.39	0.01
O08638	Myosin-11 OS=Mus musculus GN=Myh11 PE=1 SV=1 - [MYH11_MOUSE]	0.26	0.22	0.34	0.17	0.01
P0C0S6	Histone H2A.Z OS=Mus musculus GN=H2afz PE=1 SV=2 [H2AZ_MOUSE]	-0.61	-0.40	-0.80	-0.78	0.01
P39039	Mannose-binding protein A OS=Mus musculus GN=Mbl1 PE=2 SV=1 - [MBL1_MOUSE]	-0.67	-0.76	-0.40	-0.49	0.01
Q9D571	MCG3887, isoform CRA_a OS=Mus musculus GN=Mtl5 PE=2 SV=1 - [Q9D571_MOUSE]	-0.33	-0.35	-0.18	-0.25	0.01
Q6NXL1	Protein Sec24d OS=Mus musculus GN=Sec24d PE=2 SV=1 - [Q6NXL1_MOUSE]	0.28	0.49	0.57	0.56	0.01
Q9Z2H7	PDZ domain-containing protein GIPC2 OS=Mus musculus GN=Gipc2 PE=1 SV=1 - [GIPC2_MOUSE]	-0.38	-0.21	-0.44	-0.42	0.01
Q9DBE9	pre-rRNA processing protein FTSJ3 OS=Mus musculus GN=Ftsj3 PE=1 SV=1 - [SPB1_MOUSE]	0.81	1.10	0.53	0.81	0.01

Q8R2G4-8	Isoform 7 of Ecto-ADP-ribosyltransferase 3 OS=Mus musculus GN=Art3 - [NAR3_MOUSE]	-0.80	-0.40	-0.77	-0.63	0.01
Q8BYA0	Tubulin-specific chaperone D OS=Mus musculus GN=Tbcd PE=2 SV=1 - [TBCD_MOUSE]	0.36	0.70	0.46	0.59	0.01
Q9D8W7	OCIA domain-containing protein 2 OS=Mus musculus GN=Ociad2 PE=2 SV=1 - [OCAD2_MOUSE]	0.35	0.55	0.29	0.48	0.01
Q9Z110-2	Isoform Short of Delta-1-pyrroline-5-carboxylate synthase OS=Mus musculus GN=Aldh18a1 - [P5CS_MOUSE]	0.11	0.09	0.16	0.16	0.01
Q8BP71-6	Isoform 6 of RNA binding protein fox-1 homolog 2 OS=Mus musculus GN=Rbfox2 - [RFOX2_MOUSE]	-0.40	-0.40	-0.20	-0.31	0.01
O70551	SRSF protein kinase 1 OS=Mus musculus GN=Srpk1 PE=1 SV=2 [SRPK1_MOUSE]	0.41	0.39	0.66	0.66	0.01
O54782	Epididymis-specific alpha-mannosidase OS=Mus musculus GN=Man2b2 PE=2 SV=2 - [MA2B2_MOUSE]	-0.49	-0.33	-0.68	-0.50	0.01
Q61152	Tyrosine-protein phosphatase non-receptor type 18 OS=Mus musculus GN=Ptpn18 PE=1 SV=1 - [PTN18_MOUSE]	-0.18	-0.11	-0.22	-0.18	0.01
P29594	Caspase-2 OS=Mus musculus GN=Casp2 PE=1 SV=5 - [CASP2_MOUSE]	0.35	0.36	0.58	0.59	0.01
O88685	26S protease regulatory subunit 6A OS=Mus musculus GN=Psmc3 PE=1 SV=2 - [PRS6A_MOUSE]	0.15	0.21	0.11	0.17	0.01
Q6KCD5-2	Isoform 2 of Nipped-B-like protein OS=Mus musculus GN=Nipbl - [NIPBL_MOUSE]	0.23	0.20	0.32	0.16	0.01
E9PXJ0	Neuronal tyrosine-phosphorylated phosphoinositide-3-kinase adapter 1 (Fragment) OS=Mus musculus GN=Nyap1 PE=2 SV=1 - [E9PXJ0_MOUSE]	-0.19	-0.29	-0.14	-0.24	0.01
Q9CQB5	CDGSH iron-sulfur domain-containing protein 2 OS=Mus musculus GN=Cisd2 PE=1 SV=1 - [CISD2_MOUSE]	0.09	0.18	0.12	0.13	0.01

Q8CGB3	Uveal autoantigen with coiled-coil domains and ankyrin repeats OS=Mus musculus GN=Uaca PE=1 SV=2 - [UACA_MOUSE]	-0.47	-0.28	-0.56	-0.39	0.01
Q6UKZ0	MCG8992 OS=Mus musculus GN=Serpinb3d PE=2 SV=1 - [Q6UKZ0_MOUSE]	-0.60	-0.57	-0.37	-0.34	0.01
Q9D0B0	Serine/arginine-rich splicing factor 9 OS=Mus musculus GN=Srsf9 PE=1 SV=1 - [SRSF9_MOUSE]	-0.36	-0.45	-0.24	-0.27	0.01
Q91W50	Cold shock domain-containing protein E1 OS=Mus musculus GN=Csde1 PE=2 SV=1 - [CSDE1_MOUSE]	0.09	0.06	0.11	0.07	0.01
Q62084	Protein phosphatase 1 regulatory subunit 14B OS=Mus musculus GN=Ppp1r14b PE=1 SV=2 - [PP14B_MOUSE]	-0.12	-0.19	-0.15	-0.10	0.01
G5E8J4	Protein tyrosine phosphatase, non-receptor type 21, isoform CRA_a OS=Mus musculus GN=Ptpn21 PE=4 SV=1 - [G5E8J4_MOUSE]	0.84	0.57	0.69	0.42	0.01
A2RSX7	tRNA wybutosine-synthesizing protein 5 OS=Mus musculus GN=Tyw5 PE=2 SV=2 - [TYW5_MOUSE]	-0.47	-0.37	-0.33	-0.23	0.01
Q9D020-1	Isoform 1 of Cytosolic 5'-nucleotidase 3A OS=Mus musculus GN=Nt5c3a - [5NT3A_MOUSE]	0.17	0.22	0.32	0.31	0.01
Q60936	Chaperone activity of bc1 complex-like, mitochondrial OS=Mus musculus GN=Adck3 PE=2 SV=2 - [ADCK3_MOUSE]	-0.31	-0.47	-0.50	-0.65	0.01
Q8BK62	Olfactomedin-like protein 3 OS=Mus musculus GN=Olfml3 PE=2 SV=2 - [OLFL3_MOUSE]	0.91	0.80	0.59	0.47	0.01
P62264	40S ribosomal protein S14 OS=Mus musculus GN=Rps14 PE=2 SV=3 - [RS14_MOUSE]	0.19	0.27	0.26	0.14	0.01
G5E8R3	Pyruvate carboxylase OS=Mus musculus GN=Pcx PE=3 SV=1 - [G5E8R3_MOUSE]	-0.25	-0.31	-0.15	-0.28	0.01
Q9CQ45	Neudesin OS=Mus musculus GN=Nenf PE=1 SV=1 - [NENF_MOUSE]	-0.37	-0.21	-0.45	-0.39	0.01
E9PZD2	Protein Micall2 OS=Mus musculus GN=Micall2 PE=2 SV=1 - [E9PZD2_MOUSE]	-0.35	-0.20	-0.39	-0.25	0.01

Q9Z2U0	Proteasome subunit alpha type-7 OS=Mus musculus GN=Psma7 PE=1 SV=1 - [PSA7_MOUSE]	-0.19	-0.14	-0.27	-0.18	0.01
P51125-3	Isoform 3 of Calpastatin OS=Mus musculus GN=Cast - [ICAL_MOUSE]	-0.17	-0.13	-0.23	-0.13	0.01
P08228	Superoxide dismutase [Cu-Zn] OS=Mus musculus GN=Sod1 PE=1 SV=2 - [SODC_MOUSE]	-0.31	-0.18	-0.36	-0.24	0.01
F8WJI3	Coiled-coil domain-containing protein 58 OS=Mus musculus GN=Ccdc58 PE=2 SV=1 - [F8WJI3_MOUSE]	-0.22	-0.22	-0.25	-0.37	0.01
Q9DD02-2	Isoform 2 of Protein Hikeshi OS=Mus musculus GN=L7rn6 - [HIKES_MOUSE]	0.39	0.29	0.30	0.19	0.01
Q9QZ06	Toll-interacting protein OS=Mus musculus GN=Tollip PE=1 SV=1 - [TOLIP_MOUSE]	-0.14	-0.29	-0.21	-0.27	0.01
Q9CY00	Tetratricopeptide repeat protein 30B OS=Mus musculus GN=Ttc30b PE=2 SV=1 - [TT30B_MOUSE]	0.35	0.58	0.36	0.60	0.01
Q922Q9	Chitinase domain-containing protein 1 OS=Mus musculus GN=Chid1 PE=2 SV=1 [CHID1_MOUSE]	-0.25	-0.15	-0.20	-0.14	0.01
Q6PGG2-2	Isoform 2 of GEM-interacting protein OS=Mus musculus GN=Gmip - [GMIP_MOUSE]	-0.30	-0.43	-0.22	-0.38	0.01
P81117	Nucleobindin-2 OS=Mus musculus GN=Nucb2 PE=1 SV=2 - [NUCB2_MOUSE]	0.22	0.39	0.23	0.35	0.01
Q8BMS7	Calcium-transporting ATPase type 2C member 1 OS=Mus musculus GN=Atp2c1 PE=2 SV=1 - [Q8BMS7_MOUSE]	0.15	0.13	0.24	0.22	0.01
P20029	78 kDa glucose-regulated protein OS=Mus musculus GN=Hspa5 PE=1 SV=3 - [GRP78_MOUSE]	0.17	0.27	0.14	0.25	0.01
P97352	Protein S100-A13 OS=Mus musculus GN=S100a13 PE=1 SV=1 - [S10AD_MOUSE]	-0.20	-0.12	-0.24	-0.17	0.01
H3BIX4	Neuroplastin (Fragment) OS=Mus musculus GN=Nptn PE=4 SV=1 - [H3BIX4_MOUSE]	-0.11	-0.24	-0.18	-0.21	0.01
P04925	Major prion protein OS=Mus musculus GN=Prnp PE=1 SV=2 - [PRIO_MOUSE]	0.17	0.15	0.10	0.09	0.01

Q8BI29	Specifically androgen-regulated gene protein OS=Mus musculus GN=Sarg PE=1 SV=2 - [SARG_MOUSE]	-0.58	-0.39	-0.46	-0.28	0.01
Q80Y56	Rabenosyn-5 OS=Mus musculus GN=Zfyve20 PE=2 SV=1 - [RBNS5_MOUSE]	-0.16	-0.16	-0.09	-0.10	0.01
Q9JHH6	Carboxypeptidase B2 OS=Mus musculus GN=Cpb2 PE=1 SV=1 - [CBPB2_MOUSE]	-0.90	-0.73	-0.61	-0.44	0.01
E9Q8L9	Rab11 family-interacting protein 1 OS=Mus musculus GN=Rab11fip1 PE=2 SV=1 - [E9Q8L9_MOUSE]	-0.55	-0.73	-0.35	-0.67	0.01
Q9D5Y1	Coiled-coil domain-containing protein 39 OS=Mus musculus GN=Ccdc39 PE=2 SV=1 - [CCD39_MOUSE]	-0.56	-0.43	-0.39	-0.27	0.01
O55234	Proteasome subunit beta type-5 OS=Mus musculus GN=Psmb5 PE=1 SV=3 - [PSB5_MOUSE]	-0.30	-0.31	-0.15	-0.25	0.01
Q7JJ13-2	Isoform 2 of Bromodomain-containing protein 2 OS=Mus musculus GN=Brd2 - [BRD2_MOUSE]	-0.26	-0.44	-0.30	-0.48	0.01
Q8BYQ0	Arylacetamide deacetylase-like 1, isoform CRA_c OS=Mus musculus GN=Nceh1 PE=2 SV=1 - [Q8BYQ0_MOUSE]	0.49	0.75	0.38	0.64	0.01
P70671	Interferon regulatory factor 3 OS=Mus musculus GN=Irf3 PE=1 SV=1 - [IRF3_MOUSE]	0.45	0.38	0.30	0.23	0.01
Q8VCI5	Peroxisomal biogenesis factor 19 OS=Mus musculus GN=Pex19 PE=1 SV=1 - [PEX19_MOUSE]	-0.21	-0.27	-0.28	-0.41	0.01
Q8VI24-2	Isoform 2 of DNA-binding protein SATB2 OS=Mus musculus GN=Satb2 - [SATB2_MOUSE]	0.25	0.25	0.41	0.24	0.01
Q9CQT1	Methylthioribose-1-phosphate isomerase OS=Mus musculus GN=Mri1 PE=2 SV=1 - [MTNA_MOUSE]	0.16	0.22	0.18	0.30	0.01
Q60605	Myosin light polypeptide 6 OS=Mus musculus GN=Myl6 PE=1 SV=3 - [MYL6_MOUSE]	-0.40	-0.21	-0.34	-0.43	0.01
Q8CGP2	Histone H2B type 1-P OS=Mus musculus GN=Hist1h2bp PE=1 SV=3 - [H2B1P_MOUSE]	0.26	0.37	0.18	0.31	0.01

E0CZ04	Gamma-glutamylaminecyclotransferase (Fragment) OS=Mus musculus GN=Ggact PE=2 SV=1 - [E0CZ04_MOUSE]	-0.29	-0.31	-0.24	-0.15	0.01
Q8VCN9	Tubulin-specific chaperone C OS=Mus musculus GN=Tbcc PE=2 SV=1 - [TBCC_MOUSE]	0.43	0.32	0.33	0.20	0.01
Q8VCU2	Glycosylphosphatidylinositol specific phospholipase D1 OS=Mus musculus GN=Gpld1 PE=2 SV=1 - [Q8VCU2_MOUSE]	-0.65	-1.02	-0.52	-0.72	0.01
Q6P8N8	ATP-dependent Clp protease ATP-binding subunit clpX-like, mitochondrial OS=Mus musculus GN=Clpx PE=2 SV=1 - [Q6P8N8_MOUSE]	0.13	0.08	0.18	0.16	0.01
P27773	Protein disulfide-isomerase A3 OS=Mus musculus GN=Pdia3 PE=1 SV=2 - [PDIA3_MOUSE]	0.18	0.27	0.13	0.23	0.01
G8JL83	YrdC domain-containing protein, mitochondrial (Fragment) OS=Mus musculus GN=Yrdc PE=2 SV=1 - [G8JL83_MOUSE]	0.32	0.55	0.37	0.60	0.01
Q3TKT4	Transcription activator BRG1 OS=Mus musculus GN=Smarca4 PE=1 SV=1 - [SMCA4_MOUSE]	0.32	0.40	0.45	0.63	0.01
Q3UFB2-2	Isoform 2 of Box C/D snoRNA protein 1 OS=Mus musculus GN=Znhit6 - [BCD1_MOUSE]	-0.48	-0.30	-0.62	-0.43	0.01
P97384	Annexin A11 OS=Mus musculus GN=Anxa11 PE=1 SV=2 - [ANX11_MOUSE]	0.12	0.08	0.09	0.06	0.01
Q8K0D5	Elongation factor G, mitochondrial OS=Mus musculus GN=Gfm1 PE=2 SV=1 - [EFGM_MOUSE]	-0.13	-0.12	-0.14	-0.22	0.01
A1Y9I9	Transmembrane O-methyltransferase OS=Mus musculus GN=Tomt PE=1 SV=1 - [TOMT_MOUSE]	-0.71	-0.66	-0.43	-0.39	0.01
P01638	Ig kappa chain V-V region L6 (Fragment) OS=Mus musculus PE=4 SV=1 - [KV5A6_MOUSE]	-0.51	-0.79	-0.54	-0.40	0.01
E9PWV3	Ribosomal protein S6 kinase OS=Mus musculus GN=Rps6ka1 PE=2 SV=1 - [E9PWV3_MOUSE]	0.22	0.39	0.22	0.26	0.01

J3QP68	Uncharacterized protein OS=Mus musculus GN=Gm4204 PE=3 SV=1 - [J3QP68_MOUSE]	0.20	0.23	0.28	0.39	0.01
Q8C845	EF-hand domain-containing protein D2 OS=Mus musculus GN=Efhd2 PE=2 SV=1 [Q8C845_MOUSE]	-0.29	-0.22	-0.23	-0.14	0.01
D3Z479	Sorting nexin-15 OS=Mus musculus GN=Snx15 PE=2 SV=1 - [D3Z479_MOUSE]	-0.56	-0.33	-0.56	-0.33	0.01
P18608	Non-histone chromosomal protein HMG-14 OS=Mus musculus GN=Hmgn1 PE=1 SV=2 - [HMGN1_MOUSE]	0.44	0.63	0.31	0.59	0.01
Q6P9R2	Serine/threonine-protein kinase OSR1 OS=Mus musculus GN=Oxsr1 PE=1 SV=1 [OXSR1_MOUSE]	-0.12	-0.14	-0.15	-0.07	0.01
Q04447	Creatine kinase B-type OS=Mus musculus GN=Ckb PE=1 SV=1 - [KCRB_MOUSE]	0.14	0.20	0.09	0.15	0.01
Q9CQH3	NADH dehydrogenase [ubiquinone] 1 beta subcomplex subunit 5, mitochondrial OS=Mus musculus GN=Ndufb5 PE=1 SV=1 - [NDUB5_MOUSE]	0.04	0.05	0.04	0.07	0.01
P22599	Alpha-1-antitrypsin 1-2 OS=Mus musculus GN=Serpina1b PE=1 SV=2 - [A1AT2_MOUSE]	-0.40	-0.33	-0.63	-0.56	0.01
Q0P543-2	Isoform 2 of Gastric inhibitory polypeptide receptor OS=Mus musculus GN=Gipr - [GIPR_MOUSE]	0.36	0.55	0.57	0.76	0.01
D3YXK2	Scaffold attachment factor B1 OS=Mus musculus GN=Safb PE=1 SV=2 - [SAFB1_MOUSE]	0.35	0.21	0.19	0.33	0.01
Q5SS80-2	Isoform 2 of Dehydrogenase/reductase SDR family member 13 OS=Mus musculus GN=Dhrs13 - [DHR13_MOUSE]	0.57	0.72	0.35	0.50	0.01
P61939	Thyroxine-binding globulin OS=Mus musculus GN=Serpina7 PE=1 SV=1 - [THBG_MOUSE]	-0.57	-0.73	-0.35	-0.50	0.01
G3UXX3	Sepiapterin reductase OS=Mus musculus GN=Spr PE=2 SV=1 - [G3UXX3_MOUSE]	0.14	0.18	0.08	0.17	0.01

Q922Q1	MOSC domain-containing protein 2, mitochondrial OS=Mus musculus GN=Marc2 PE=1 SV=1 - [MOSC2_MOUSE]	-0.29	-0.22	-0.16	-0.16	0.01
P43247	DNA mismatch repair protein Msh2 OS=Mus musculus GN=Msh2 PE=2 SV=1 - [MSH2_MOUSE]	0.14	0.20	0.22	0.29	0.01
Q9JLJ2	4-trimethylaminobutyraldehyde dehydrogenase OS=Mus musculus GN=Aldh9a1 PE=1 SV=1 - [AL9A1_MOUSE]	-0.37	-0.28	-0.30	-0.17	0.01
Q8R1X6	Spartin OS=Mus musculus GN=Spg20 PE=2 SV=1 - [SPG20_MOUSE]	0.32	0.39	0.19	0.26	0.01
O35066	Kinesin-like protein KIF3C OS=Mus musculus GN=Kif3c PE=2 SV=3 - [KIF3C_MOUSE]	-0.14	-0.25	-0.17	-0.27	0.01
E9QMC1	Cingulin OS=Mus musculus GN=Cgn PE=2 SV=1 - [E9QMC1_MOUSE]	-0.11	-0.11	-0.14	-0.07	0.01
P08775	DNA-directed RNA polymerase II subunit RPB1 OS=Mus musculus GN=Polr2a PE=1 SV=3 - [RPB1_MOUSE]	0.23	0.25	0.18	0.35	0.01
Q9WUG6	Insulin-like peptide INSL5 OS=Mus musculus GN=Insl5 PE=2 SV=1 - [INSL5_MOUSE]	1.08	0.58	1.22	0.87	0.01
Q9ESJ0	Exportin-4 OS=Mus musculus GN=Xpo4 PE=1 SV=2 - [XPO4_MOUSE]	0.63	0.37	0.76	0.51	0.01
Q91YI0	Argininosuccinate lyase OS=Mus musculus GN=Asl PE=2 SV=1 - [ARLY_MOUSE]	-0.20	-0.32	-0.16	-0.28	0.01
B8JK39	Protein Itga9 OS=Mus musculus GN=Itga9 PE=2 SV=1 - [B8JK39_MOUSE]	0.26	0.18	0.32	0.18	0.01
Q8K4T5	Dual specificity protein phosphatase 19 OS=Mus musculus GN=Dusp19 PE=2 SV=1 - [DUS19_MOUSE]	0.14	0.23	0.18	0.27	0.01
O55057	Retinal rod rhodopsin-sensitive cGMP 3',5'-cyclic phosphodiesterase subunit delta OS=Mus musculus GN=Pde6d PE=1 SV=1 - [PDE6D_MOUSE]	-0.67	-0.81	-0.40	-0.53	0.01
Q9CY50	Translocon-associated protein subunit alpha OS=Mus musculus GN=Ssr1 PE=1 SV=1 - [SSRA_MOUSE]	0.44	0.71	0.38	0.67	0.01

Q9JIK5	Nucleolar RNA helicase 2 OS=Mus musculus GN=Ddx21 PE=1 SV=3 - [DDX21_MOUSE]	0.32	0.44	0.21	0.39	0.01
H3BL23	Ubiquitin-conjugating enzyme E2 E2 (Fragment) OS=Mus musculus GN=Ube2e2 PE=2 SV=1 - [H3BL23_MOUSE]	0.26	0.44	0.36	0.54	0.01
Q9QY93	dCTP pyrophosphatase 1 OS=Mus musculus GN=Dctpp1 PE=1 SV=1 - [DCTP1_MOUSE]	-0.40	-0.47	-0.25	-0.54	0.01
Q8BKS9	Pumilio domain-containing protein KIAA0020 OS=Mus musculus GN=Kiaa0020 PE=2 SV=2 - [K0020_MOUSE]	0.63	0.40	0.55	0.32	0.01
Q920A5	Retinoid-inducible serine carboxypeptidase OS=Mus musculus GN=Scpep1 PE=2 SV=2 - [RISC_MOUSE]	0.24	0.36	0.25	0.44	0.01
Q91YS7	Dual-specificity mitogen-activated protein kinase kinase 2 OS=Mus musculus GN=Map2k2 PE=2 SV=1 - [Q91YS7_MOUSE]	0.38	0.35	0.18	0.39	0.01
Q8C166	Copine-1 OS=Mus musculus GN=Cpne1 PE=1 SV=1 - [CPNE1_MOUSE]	-0.41	-0.70	-0.43	-0.72	0.01
O55126	Protein NipSnap homolog 2 OS=Mus musculus GN=Gbas PE=2 SV=1 - [NIPS2_MOUSE]	0.28	0.35	0.21	0.43	0.01
P14148	60S ribosomal protein L7 OS=Mus musculus GN=Rpl7 PE=2 SV=2 - [RL7_MOUSE]	0.07	0.09	0.07	0.04	0.01
P01635	Ig kappa chain V-V region K2 (Fragment) OS=Mus musculus PE=1 SV=1 - [KV5A3_MOUSE]	0.31	0.58	0.34	0.49	0.01
A8Y5N4	17-beta-hydroxysteroid dehydrogenase 13 OS=Mus musculus GN=Hsd17b13 PE=2 SV=1 - [A8Y5N4_MOUSE]	-0.32	-0.37	-0.18	-0.24	0.01
Q9Z0H7	B-cell lymphoma/leukemia 10 OS=Mus musculus GN=Bcl10 PE=1 SV=1 - [BCL10_MOUSE]	0.21	0.26	0.37	0.42	0.01
Q9DA97	Septin-14 OS=Mus musculus GN=Sept14 PE=2 SV=3 - [SEP14_MOUSE]	0.30	0.38	0.18	0.26	0.01

Q99KJ8	Dynactin subunit 2 OS=Mus musculus GN=Dctn2 PE=1 SV=3 [DCTN2_MOUSE]	-0.06	-0.10	-0.09	-0.13	0.01
Q8BVA2	Transmembrane protein 222 OS=Mus musculus GN=Tmem222 PE=2 SV=1 - [TM222_MOUSE]	0.09	0.05	0.10	0.06	0.01
Q62468	Villin-1 OS=Mus musculus GN=Vil1 PE=1 SV=3 - [VILI_MOUSE]	0.03	0.06	0.05	0.07	0.01
Q9JLZ8	Single Ig IL-1-related receptor OS=Mus musculus GN=Sigirr PE=1 SV=2 - [SIGIR_MOUSE]	-0.30	-0.19	-0.40	-0.31	0.01
Q9Z247	Peptidyl-prolyl cis-trans isomerase FKBP9 OS=Mus musculus GN=Fkbp9 PE=1 SV=1 - [FKBP9_MOUSE]	-0.20	-0.16	-0.17	-0.30	0.01
P01674	Ig kappa chain V-III region PC 2154 OS=Mus musculus PE=1 SV=1 - [KV3AM_MOUSE]	1.75	1.02	1.90	1.16	0.01
Q8CIM3	D-2-hydroxyglutarate dehydrogenase, mitochondrial OS=Mus musculus GN=D2hgdh PE=2 SV=3 - [D2HDH_MOUSE]	0.95	0.61	0.81	0.47	0.01
Q9D0M3-2	Isoform 2 of Cytochrome c1, heme protein, mitochondrial OS=Mus musculus GN=Cyc1 - [CY1_MOUSE]	0.11	0.13	0.14	0.07	0.01
Q2KN98	Cytospin-A OS=Mus musculus GN=Specc1l PE=1 SV=1 - [CYTSA_MOUSE]	-0.25	-0.14	-0.31	-0.23	0.01
B2RT41	Protein Zfc3h1 OS=Mus musculus GN=Zfc3h1 PE=2 SV=1 - [B2RT41_MOUSE]	-1.69	-1.48	-1.07	-0.86	0.01
F7A8R0	N-alpha-acetyltransferase 35, NatC auxiliary subunit (Fragment) OS=Mus musculus GN=Naa35 PE=2 SV=1 - [F7A8R0_MOUSE]	0.57	0.34	0.70	0.47	0.01
Q9QUR7	Peptidyl-prolyl cis-trans isomerase NIMA-interacting 1 OS=Mus musculus GN=Pin1 PE=1 SV=1 - [PIN1_MOUSE]	-0.32	-0.28	-0.37	-0.53	0.01
Q80UJ7	Rab3 GTPase-activating protein catalytic subunit OS=Mus musculus GN=Rab3gap1 PE=2 SV=4 - [RB3GP_MOUSE]	0.16	0.27	0.18	0.30	0.01

I7HPW8	Protein Myo15b (Fragment) OS=Mus musculus GN=Myo15b PE=4 SV=1 - [I7HPW8_MOUSE]	0.25	0.18	0.19	0.12	0.01
P35505	Fumarylacetoacetase OS=Mus musculus GN=Fah PE=1 SV=2 - [FAAA_MOUSE]	-0.41	-0.42	-0.48	-0.22	0.01
O89086	Putative RNA-binding protein 3 OS=Mus musculus GN=Rbm3 PE=1 SV=1 - [RBM3_MOUSE]	-0.21	-0.28	-0.13	-0.21	0.01
P08905	Lysozyme C-2 OS=Mus musculus GN=Lyz2 PE=1 SV=2 - [LYZ2_MOUSE]	-0.38	-0.62	-0.33	-0.57	0.01
P09103	Protein disulfide-isomerase OS=Mus musculus GN=P4hb PE=1 SV=2 - [PDIA1_MOUSE]	0.16	0.24	0.12	0.22	0.01
Q9Z0H8-2	Isoform 2 of CAP-Gly domain-containing linker protein 2 OS=Mus musculus GN=Clip2 - [CLIP2_MOUSE]	-0.16	-0.21	-0.16	-0.10	0.01
P08249	Malate dehydrogenase, mitochondrial OS=Mus musculus GN=Mdh2 PE=1 SV=3 - [MDHM_MOUSE]	-0.16	-0.16	-0.29	-0.25	0.01
Q8BNQ3	Integral membrane protein GPR137B OS=Mus musculus GN=Gpr137b PE=1 SV=2 - [G137B_MOUSE]	-1.23	-1.12	-0.76	-0.65	0.01
Q8R5L1	Complement component 1 Q subcomponent-binding protein, mitochondrial OS=Mus musculus GN=C1qbp PE=2 SV=1 - [Q8R5L1_MOUSE]	-0.31	-0.16	-0.36	-0.31	0.01
P42703	Leukemia inhibitory factor receptor OS=Mus musculus GN=Lifr PE=1 SV=1 - [LIFR_MOUSE]	-0.49	-0.41	-0.73	-0.79	0.01
Q8R5F3	O-acetyl-ADP-ribose deacetylase 1 OS=Mus musculus GN=Oard1 PE=2 SV=2 - [OARD1_MOUSE]	0.49	0.46	0.30	0.26	0.01
Q1W617-2	Isoform 2 of Protein Shroom4 OS=Mus musculus GN=Shroom4 - [SHRM4_MOUSE]	-0.24	-0.41	-0.27	-0.44	0.01
Q8VEE4	Replication protein A 70 kDa DNA-binding subunit OS=Mus musculus GN=Rpa1 PE=2 SV=1 - [RFA1_MOUSE]	0.15	0.15	0.13	0.24	0.01

P70458	Plasma serine protease inhibitor OS=Mus musculus GN=Serpina5 PE=1 SV=2 - [IPSP_MOUSE]	0.97	1.34	0.62	0.99	0.01
Q9WTP7	GTP:AMP phosphotransferase AK3, mitochondrial OS=Mus musculus GN=Ak3 PE=1 SV=3 - [KAD3_MOUSE]	-0.09	-0.17	-0.10	-0.16	0.01
Q810D6	Glutamate-rich WD repeat-containing protein 1 OS=Mus musculus GN=Grwd1 PE=2 SV=2 - [GRWD1_MOUSE]	0.40	0.35	0.44	0.20	0.01
Q99N91	39S ribosomal protein L34, mitochondrial OS=Mus musculus GN=Mrpl34 PE=2 SV=1 - [RM34_MOUSE]	-0.24	-0.38	-0.39	-0.53	0.01
Q8BQB4	Four-jointed box protein 1 OS=Mus musculus GN=Fjx1 PE=1 SV=1 - [FJX1_MOUSE]	-0.94	-1.04	-0.54	-0.65	0.01
Q9DBG7	Signal recognition particle receptor subunit alpha OS=Mus musculus GN=Srpr PE=1 SV=1 - [SRPR_MOUSE]	0.47	0.26	0.56	0.39	0.01
F6SVV1	Protein Gm9493 OS=Mus musculus GN=Gm9493 PE=4 SV=1 - [F6SVV1_MOUSE]	0.24	0.38	0.22	0.21	0.01
Q5DTZ0	Protein NYNRIN OS=Mus musculus GN=Nynrin PE=1 SV=2 - [NYNRI_MOUSE]	0.31	0.18	0.36	0.23	0.01
Q9EPS3	D-glucuronyl C5-epimerase OS=Mus musculus GN=Glce PE=1 SV=2 - [GLCE_MOUSE]	0.41	0.77	0.46	0.70	0.01
Q9JJA2	Conserved oligomeric Golgi complex subunit 8 OS=Mus musculus GN=Cog8 PE=2 SV=3 - [COG8_MOUSE]	0.31	0.54	0.30	0.48	0.01
Q9DBX6	Cytochrome P450 2S1 OS=Mus musculus GN=Cyp2s1 PE=2 SV=1 - [CP2S1_MOUSE]	-0.29	-0.17	-0.38	-0.29	0.01
Q91W61	F-box/LRR-repeat protein 15 OS=Mus musculus GN=Fbxl15 PE=1 SV=2 - [FXL15_MOUSE]	0.42	0.34	0.68	0.60	0.01
P26369	Splicing factor U2AF 65 kDa subunit OS=Mus musculus GN=U2af2 PE=1 SV=3 - [U2AF2_MOUSE]	0.14	0.18	0.10	0.10	0.01
Q8BHA3	Probable D-tyrosyl-tRNA(Tyr) deacylase 2 OS=Mus musculus GN=Dtd2 PE=2 SV=1 - [DTD2_MOUSE]	-0.47	-0.84	-0.49	-0.80	0.01

Q7TRN4	Olfactory receptor 698 OS=Mus musculus GN=Olfr698 PE=3 SV=1 - [Q7TRN4_MOUSE]	0.16	0.15	0.27	0.26	0.01
Q9JLV5	Cullin-3 OS=Mus musculus GN=Cul3 PE=1 SV=1 - [CUL3_MOUSE]	0.14	0.20	0.10	0.18	0.01
Q8BR63	Protein FAM177A1 OS=Mus musculus GN=Fam177a1 PE=2 SV=1 - [F177A_MOUSE]	0.20	0.22	0.12	0.27	0.01
Q8CGM2	Retinitis pigmentosa 1-like 1 protein OS=Mus musculus GN=Rp1l1 PE=1 SV=1 - [RP1L1_MOUSE]	-0.33	-0.19	-0.39	-0.25	0.01
P10639	Thioredoxin OS=Mus musculus GN=Txn PE=1 SV=3 - [THIO_MOUSE]	0.23	0.35	0.19	0.36	0.01
Q9R0Y5	Adenylate kinase isoenzyme 1 OS=Mus musculus GN=Ak1 PE=1 SV=1 - [KAD1_MOUSE]	0.09	0.17	0.16	0.19	0.01
D3Z495	Protein spire homolog 1 OS=Mus musculus GN=Spire1 PE=2 SV=1 - [D3Z495_MOUSE]	-0.96	-1.47	-0.70	-1.21	0.01
P53994	Ras-related protein Rab-2A OS=Mus musculus GN=Rab2a PE=1 SV=1 - [RAB2A_MOUSE]	0.16	0.26	0.23	0.33	0.01
K7N6T3	Protein Gm3159 OS=Mus musculus GN=Gm3159 PE=4 SV=1 - [K7N6T3_MOUSE]	0.56	0.71	0.33	0.48	0.01
P47955	60S acidic ribosomal protein P1 OS=Mus musculus GN=Rplp1 PE=1 SV=1 - [RLA1_MOUSE]	0.10	0.19	0.17	0.23	0.01
P42567	Epidermal growth factor receptor substrate 15 OS=Mus musculus GN=Eps15 PE=1 SV=1 - [EPS15_MOUSE]	-0.27	-0.15	-0.32	-0.21	0.01
F7CXR1	Protein Gm9857 OS=Mus musculus GN=Gm9857 PE=2 SV=1 - [F7CXR1_MOUSE]	-0.30	-0.52	-0.35	-0.57	0.01
Q9CPU0	Lactoylglutathione lyase OS=Mus musculus GN=Glo1 PE=1 SV=3 - [LGUL_MOUSE]	0.05	0.07	0.03	0.06	0.01
Q14DK4-2	Isoform 2 of Glycerol-3-phosphate acyltransferase 2, mitochondrial OS=Mus musculus GN=Gpat2 - [GPAT2_MOUSE]	0.77	0.92	1.34	1.49	0.01
D3Z6I3	Lipoyl synthase, mitochondrial OS=Mus musculus GN=Lias PE=2 SV=1 - [D3Z6I3_MOUSE]	-0.16	-0.15	-0.27	-0.26	0.01

B1AYM9	Protein CK137956 OS=Mus musculus GN=CK137956 PE=2 SV=1 - [B1AYM9_MOUSE]	0.34	0.20	0.40	0.25	0.01
Q9D394-2	Isoform 2 of Protein RUFY3 OS=Mus musculus GN=Rufy3 - [RUFY3_MOUSE]	0.16	0.14	0.15	0.07	0.01
Q9Z0Y1	Dynactin subunit 3 OS=Mus musculus GN=Dctn3 PE=2 SV=2 [DCTN3_MOUSE]	0.20	0.34	0.29	0.43	0.01
P08003	Protein disulfide-isomerase A4 OS=Mus musculus GN=Pdia4 PE=1 SV=3 - [PDIA4_MOUSE]	0.18	0.22	0.10	0.16	0.01
A2AVJ7	Ribosome-binding protein 1 OS=Mus musculus GN=Rrbp1 PE=2 SV=1 - [A2AVJ7_MOUSE]	0.14	0.19	0.09	0.13	0.01
P99029-2	Isoform Cytoplasmic+peroxisomal of Peroxiredoxin-5, mitochondrial OS=Mus musculus GN=Prdx5 - [PRDX5_MOUSE]	-0.29	-0.16	-0.33	-0.22	0.01
Q8BZ20	Poly [ADP-ribose] polymerase 12 OS=Mus musculus GN=Parp12 PE=2 SV=3 - [PAR12_MOUSE]	0.58	0.38	0.81	0.53	0.01
Q99LS3	Phosphoserine phosphatase OS=Mus musculus GN=Psph PE=1 SV=1 - [SERB_MOUSE]	0.24	0.50	0.48	0.35	0.01
O88428	Bifunctional 3'-phosphoadenosine 5'-phosphosulfate synthase 2 OS=Mus musculus GN=Papss2 PE=1 SV=2 - [PAPS2_MOUSE]	-0.15	-0.13	-0.15	-0.07	0.01
A3KGQ6	Actin-related protein 2/3 complex subunit 5-like protein OS=Mus musculus GN=Arpc5l PE=2 SV=1 - [A3KGQ6_MOUSE]	0.18	0.22	0.36	0.30	0.01
E9Q6P4	Protein Tubgcp6 OS=Mus musculus GN=Tubgcp6 PE=2 SV=1 - [E9Q6P4_MOUSE]	-0.65	-0.55	-1.07	-0.97	0.01
Q8BU14	Translocation protein SEC62 OS=Mus musculus GN=Sec62 PE=1 SV=1 - [SEC62_MOUSE]	0.53	0.80	0.38	0.66	0.01
Q3UHD9-2	Isoform 2 of Arf-GAP with GTPase, ANK repeat and PH domain-containing protein 2 OS=Mus musculus GN=Agap2 - [AGAP2_MOUSE]	0.35	0.27	0.56	0.47	0.01

Q6DFY2	Opioid binding protein/cell adhesion molecule-like OS=Mus musculus GN=Opcml PE=2 SV=1 - [Q6DFY2_MOUSE]	-0.50	-0.86	-0.52	-0.89	0.01
O35711-2	Isoform 2 of Liprin-beta-2 OS=Mus musculus GN=Ppfibp2 - [LIPB2_MOUSE]	-0.22	-0.18	-0.10	-0.19	0.01
Q8BWS5	G protein-regulated inducer of neurite outgrowth 3 OS=Mus musculus GN=Gprin3 PE=1 SV=1 - [GRIN3_MOUSE]	-0.48	-0.53	-0.27	-0.32	0.01
E9PV12	Cullin-5 OS=Mus musculus GN=Cul5 PE=2 SV=1 - [E9PV12_MOUSE]	0.24	0.28	0.19	0.13	0.01
Q80U12	Small G protein signaling modulator 2 OS=Mus musculus GN=Sgsm2 PE=2 SV=2 - [SGSM2_MOUSE]	-0.51	-0.41	-0.34	-0.24	0.01
D6RIJ8	Protein 2700029M09Rik OS=Mus musculus GN=2700029M09Rik PE=4 SV=1 - [D6RIJ8_MOUSE]	0.46	0.78	0.43	0.74	0.01
F6RUV8	Protein AA386476 OS=Mus musculus GN=AA386476 PE=4 SV=2 - [F6RUV8_MOUSE]	0.21	0.19	0.36	0.34	0.01
E9QJV4	Pre-mRNA-processing factor 39 OS=Mus musculus GN=Prpf39 PE=2 SV=1 - [E9QJV4_MOUSE]	0.32	0.18	0.36	0.22	0.01
Q91WT8-2	Isoform 2 of RNA-binding protein 47 OS=Mus musculus GN=Rbm47 - [RBM47_MOUSE]	0.25	0.13	0.16	0.15	0.01
Q9D281	Protein Noxp20 OS=Mus musculus GN=Fam114a1 PE=2 SV=1 - [NXP20_MOUSE]	0.21	0.18	0.20	0.34	0.01
Q3UMU9-4	Isoform 4 of Hepatoma-derived growth factor-related protein 2 OS=Mus musculus GN=Hdgfrp2 - [HDGR2_MOUSE]	0.15	0.27	0.33	0.30	0.01
Q8BHC0	Lymphatic vessel endothelial hyaluronic acid receptor 1 OS=Mus musculus GN=Lyve1 PE=1 SV=1 [LYVE1_MOUSE]	-1.04	-0.69	-1.51	-1.16	0.01
Q9WVL0	Maleylacetoacetate isomerase OS=Mus musculus GN=Gstz1 PE=1 SV=1 - [MAAI_MOUSE]	-0.11	-0.12	-0.06	-0.13	0.01

Q3UZ39	Leucine-rich repeat flightless-interacting protein 1 OS=Mus musculus GN=Lrrfip1 PE=1 SV=2 - [LRRF1_MOUSE]	0.26	0.16	0.23	0.13	0.01
Q9CYW4	Haloacid dehalogenase like hydrolase domain-containing protein 3 OS=Mus musculus GN=Hdhd3 PE=2 SV=1 - [HDHD3_MOUSE]	-0.36	-0.48	-0.22	-0.45	0.01
Q60960	Importin subunit alpha-5 OS=Mus musculus GN=Kpna1 PE=1 SV=2 - [IMA5_MOUSE]	0.54	0.88	0.45	0.79	0.01
P13864	DNA (cytosine-5)-methyltransferase 1 OS=Mus musculus GN=Dnmt1 PE=1 SV=5 - [DNMT1_MOUSE]	0.20	0.27	0.16	0.13	0.01
O88593	Peptidoglycan recognition protein 1 OS=Mus musculus GN=Pglyrp1 PE=2 SV=1 - [PGRP1_MOUSE]	-0.57	-1.05	-0.63	-1.03	0.01
Q9D0S9	Histidine triad nucleotide-binding protein 2, mitochondrial OS=Mus musculus GN=Hint2 PE=2 SV=1 - [HINT2_MOUSE]	-0.58	-0.44	-0.92	-0.70	0.01
P0CW02	Lymphocyte antigen 6C1 OS=Mus musculus GN=Ly6c1 PE=2 SV=1 [LY6C1_MOUSE]	-0.43	-0.34	-0.71	-0.54	0.01
E9QNG1	Intersectin-2 OS=Mus musculus GN=Itsn2 PE=2 SV=1 - [E9QNG1_MOUSE]	-0.13	-0.22	-0.13	-0.13	0.01
Q9D5J6	Sedoheptulokinase OS=Mus musculus GN=Shpk PE=1 SV=1 - [SHPK_MOUSE]	-0.26	-0.44	-0.25	-0.44	0.01
P35293	Ras-related protein Rab-18 OS=Mus musculus GN=Rab18 PE=2 SV=2 - [RAB18_MOUSE]	0.13	0.06	0.12	0.13	0.01
Q6PHU5	Sortilin OS=Mus musculus GN=Sort1 PE=1 SV=1 - [SORT_MOUSE]	0.16	0.25	0.22	0.33	0.01
Q80UU9	Membrane-associated progesterone receptor component 2 OS=Mus musculus GN=Pgrmc2 PE=1 SV=2 - [PGRC2_MOUSE]	0.12	0.14	0.23	0.21	0.01
P82343-2	Isoform 2 of N-acylglucosamine 2-epimerase OS=Mus musculus GN=Renbp - [RENBP_MOUSE]	0.60	0.99	0.50	0.88	0.01

D3Z3R1	60S ribosomal protein L36 OS=Mus musculus GN=Gm5745 PE=3 SV=1 - [D3Z3R1_MOUSE]	0.09	0.12	0.13	0.18	0.01
F6TBK9	Supervillin (Fragment) OS=Mus musculus GN=Svil PE=4 SV=1 - [F6TBK9_MOUSE]	-0.14	-0.24	-0.14	-0.24	0.01
E9Q4M4	Coiled-coil-helix-coiled-coil-helix domain-containing protein 6, mitochondrial OS=Mus musculus GN=Chchd6 PE=2 SV=1 - [E9Q4M4_MOUSE]	-0.17	-0.27	-0.25	-0.37	0.01
D3YWT1	MCG11326, isoform CRA_b OS=Mus musculus GN=Hnrnph3 PE=4 SV=1 - [D3YWT1_MOUSE]	-0.12	-0.19	-0.10	-0.20	0.01
D3YYT0	Cadherin-2 OS=Mus musculus GN=Cdh2 PE=2 SV=1 - [D3YYT0_MOUSE]	-0.44	-0.25	-0.50	-0.31	0.01
Q3UM29	Conserved oligomeric Golgi complex subunit 7 OS=Mus musculus GN=Cog7 PE=2 SV=1 - [COG7_MOUSE]	0.16	0.21	0.25	0.34	0.01
Q8K0C9	GDP-mannose 4,6 dehydratase OS=Mus musculus GN=Gmds PE=2 SV=1 - [GMDS_MOUSE]	-0.11	-0.06	-0.14	-0.10	0.01
P61161	Actin-related protein 2 OS=Mus musculus GN=Actr2 PE=1 SV=1 - [ARP2_MOUSE]	0.08	0.04	0.07	0.06	0.01
Q8CG03	cGMP-specific 3',5'-cyclic phosphodiesterase OS=Mus musculus GN=Pde5a PE=1 SV=2 - [PDE5A_MOUSE]	0.29	0.20	0.40	0.23	0.01
E9PYK3	Protein Parp4 OS=Mus musculus GN=Parp4 PE=4 SV=1 - [E9PYK3_MOUSE]	0.23	0.11	0.23	0.23	0.01
P98203-4	Isoform 4 of Armadillo repeat protein deleted in velo-cardio-facial syndrome homolog OS=Mus musculus GN=Arvcf - [ARVC_MOUSE]	-0.28	-0.30	-0.16	-0.17	0.01
Q9DBC0	Selenoprotein O OS=Mus musculus GN=Selo PE=1 SV=4 - [SELO_MOUSE]	-0.25	-0.41	-0.22	-0.41	0.01
Q8VBV7	COP9 signalosome complex subunit 8 OS=Mus musculus GN=Cops8 PE=1 SV=1 - [CSN8_MOUSE]	0.07	0.10	0.10	0.15	0.01
P53702	Cytochrome c-type heme lyase OS=Mus musculus GN=Hccs PE=2 SV=2 - [CCHL_MOUSE]	0.15	0.19	0.32	0.25	0.01

Q922Q5	Adenosine 3'-phospho 5'-phosphosulfate transporter 2 OS=Mus musculus GN=Slc35b3 PE=2 SV=1 - [S35B3_MOUSE]	0.53	0.83	0.40	0.70	0.01
Q99JY9	Actin-related protein 3 OS=Mus musculus GN=Actr3 PE=1 SV=3 - [ARP3_MOUSE]	0.13	0.15	0.07	0.15	0.01
Q8BH00	Aldehyde dehydrogenase family 8 member A1 OS=Mus musculus GN=Aldh8a1 PE=1 SV=1 - [AL8A1_MOUSE]	-0.38	-0.66	-0.38	-0.66	0.01
B8JJ84	Syntaxin-binding protein 6 (Fragment) OS=Mus musculus GN=Stxbp6 PE=2 SV=1 - [B8JJ84_MOUSE]	0.31	0.55	0.41	0.65	0.01
Q80X95	Ras-related GTP-binding protein A OS=Mus musculus GN=Rraga PE=2 SV=1 - [RRAGA_MOUSE]	-0.15	-0.25	-0.13	-0.22	0.01
Q8BS95	Golgi pH regulator OS=Mus musculus GN=Gpr89a PE=2 SV=2 - [GPHR_MOUSE]	0.48	0.76	0.36	0.63	0.01
P59759	MKL/myocardin-like protein 2 OS=Mus musculus GN=Mkl2 PE=1 SV=1 - [MKL2_MOUSE]	0.16	0.24	0.11	0.19	0.01
P26516	26S proteasome non-ATPase regulatory subunit 7 OS=Mus musculus GN=Psmd7 PE=1 SV=2 - [PSMD7_MOUSE]	0.19	0.21	0.18	0.09	0.01
D3Z6T3	Cathepsin E OS=Mus musculus GN=Ctse PE=2 SV=1 - [D3Z6T3_MOUSE]	0.56	0.82	0.38	0.74	0.01
Q8K057-2	Isoform 2 of Intraflagellar transport protein 80 homolog OS=Mus musculus GN=Ift80 - [IFT80_MOUSE]	0.38	0.57	0.26	0.46	0.01
Q9D7Y9-2	Isoform 2 of Protein SLX4IP OS=Mus musculus GN=Slx4ip - [SLX4I_MOUSE]	0.17	0.27	0.30	0.39	0.01
Q3UR39	Rab proteins geranylgeranyltransferase component A 1 OS=Mus musculus GN=Chm PE=2 SV=1 - [Q3UR39_MOUSE]	0.54	0.95	0.55	0.96	0.01
A2RS43	Protein Pcdh7 OS=Mus musculus GN=Pcdh7 PE=2 SV=1 - [A2RS43_MOUSE]	-0.20	-0.35	-0.47	-0.36	0.01
P47911	60S ribosomal protein L6 OS=Mus musculus GN=Rpl6 PE=1 SV=3 - [RL6_MOUSE]	0.12	0.19	0.09	0.16	0.01

Q8BM54-2	Isoform 2 of E3 ubiquitin-protein ligase MYLIP OS=Mus musculus GN=Mylip - [MYLIP_MOUSE]	0.38	0.59	0.28	0.49	0.01
Q9Z1D1	Eukaryotic translation initiation factor 3 subunit G OS=Mus musculus GN=Eif3g PE=1 SV=2 - [EIF3G_MOUSE]	0.28	0.35	0.16	0.22	0.01
Q8R0H9	ADP-ribosylation factor-binding protein GGA1 OS=Mus musculus GN=Gga1 PE=1 SV=1 - [GGA1_MOUSE]	0.13	0.28	0.28	0.27	0.01
F8VQ54	Endoribonuclease Dicer OS=Mus musculus GN=Dicer1 PE=2 SV=1 - [F8VQ54_MOUSE]	0.31	0.22	0.24	0.14	0.01
A2CG77	Histone-lysine N-methyltransferase EHMT2 (Fragment) OS=Mus musculus GN=Ehmt2 PE=2 SV=1 - [A2CG77_MOUSE]	-0.58	-0.95	-0.92	-1.30	0.01
P58058	NAD kinase OS=Mus musculus GN=Nadk PE=1 SV=2 - [NADK_MOUSE]	-0.36	-0.42	-0.19	-0.41	0.01
Q80X85	28S ribosomal protein S7, mitochondrial OS=Mus musculus GN=Mrps7 PE=2 SV=1 - [RT07_MOUSE]	0.26	0.47	0.28	0.48	0.01
D3Z5V3	Uncharacterized protein (Fragment) OS=Mus musculus GN=Knop1 PE=2 SV=1 - [D3Z5V3_MOUSE]	-0.74	-0.85	-0.41	-0.52	0.01
Q9QXV0	ProSAAS OS=Mus musculus GN=Pcsk1n PE=1 SV=2 - [PCSK1_MOUSE]	-0.46	-0.72	-0.35	-0.45	0.01
A2AKH7	Leucine-rich repeat-containing protein 57 (Fragment) OS=Mus musculus GN=Lrrc57 PE=2 SV=1 - [A2AKH7_MOUSE]	0.17	0.15	0.16	0.28	0.01
G3X8Z1	Calcium-activated chloride channel regulator 4 OS=Mus musculus GN=Clca6 PE=4 SV=1 - [G3X8Z1_MOUSE]	0.68	0.85	0.65	1.24	0.01
E9PV44	ATPase inhibitor, mitochondrial OS=Mus musculus GN=Atpif1 PE=2 SV=1 - [E9PV44_MOUSE]	-0.37	-0.23	-0.51	-0.33	0.01
Q99M71-2	Isoform 2 of Mammalian ependymin-related protein 1 OS=Mus musculus GN=Epdr1 - [EPDR1_MOUSE]	-0.27	-0.21	-0.44	-0.38	0.01

Q8R1N0	Zinc finger protein 830 OS=Mus musculus GN=Znf830 PE=1 SV=1 - [ZN830_MOUSE]	0.20	0.32	0.15	0.26	0.01
Q8BG73	SH3 domain-binding glutamic acid-rich-like protein 2 OS=Mus musculus GN=Sh3bgrl2 PE=1 SV=1 - [SH3L2_MOUSE]	-0.13	-0.10	-0.13	-0.06	0.01
P20181	Neurotrophin-3 OS=Mus musculus GN=Ntf3 PE=1 SV=1 - [NTF3_MOUSE]	-0.27	-0.17	-0.34	-0.19	0.01
P01867-2	Isoform 2 of Ig gamma-2B chain C region OS=Mus musculus GN=Igh-3 - [IGG2B_MOUSE]	-0.27	-0.43	-0.58	-0.57	0.01
E9PVW4	V-set and immunoglobulin domain-containing protein 10 OS=Mus musculus GN=Vsig10 PE=2 SV=1 - [E9PVW4_MOUSE]	0.15	0.20	0.27	0.13	0.01
Q9D7T1	Rab15 effector protein OS=Mus musculus GN=Rep15 PE=2 SV=1 - [REP15_MOUSE]	0.12	0.08	0.17	0.13	0.01
Q9DBR7	Protein phosphatase 1 regulatory subunit 12A OS=Mus musculus GN=Ppp1r12a PE=1 SV=2 - [MYPT1_MOUSE]	0.20	0.38	0.22	0.35	0.01
Q91XC8	Death-associated protein 1 OS=Mus musculus GN=Dap PE=1 SV=3 - [DAP1_MOUSE]	0.24	0.39	0.19	0.35	0.01
E9QN99	Alpha/beta hydrolase domain-containing protein 14B OS=Mus musculus GN=Abhd14b PE=4 SV=1 - [E9QN99_MOUSE]	-0.08	-0.08	-0.15	-0.11	0.01
P18828-2	Isoform 2 of Syndecan-1 OS=Mus musculus GN=Sdc1 - [SDC1_MOUSE]	0.33	0.48	0.21	0.37	0.01
Q9Z0X1	Apoptosis-inducing factor 1, mitochondrial OS=Mus musculus GN=Aifm1 PE=1 SV=1 [AIFM1_MOUSE]	-0.22	-0.15	-0.20	-0.10	0.01
Q8BP92	Reticulocalbin-2 OS=Mus musculus GN=Rcn2 PE=2 SV=1 - [RCN2_MOUSE]	-0.31	-0.23	-0.47	-0.28	0.01
K7N652	Protein Vmn2r50 OS=Mus musculus GN=Vmn2r50 PE=3 SV=1 - [K7N652_MOUSE]	0.75	0.71	0.44	0.39	0.01
Q8CH18-3	Isoform 3 of Cell division cycle and apoptosis regulator protein 1 OS=Mus musculus GN=Ccar1 - [CCAR1_MOUSE]	0.06	0.14	0.12	0.12	0.01

Q9QXE7	F-box-like/WD repeat-containing protein TBL1X OS=Mus musculus GN=Tbl1x PE=2 SV=2 - [TBL1X_MOUSE]	-0.32	-0.36	-0.51	-0.63	0.01
Q9D311	Dual oxidase maturation factor 2 OS=Mus musculus GN=Duoxa2 PE=2 SV=1 - [DOXA2_MOUSE]	0.67	0.88	1.21	1.41	0.01
Q64521	Glycerol-3-phosphate dehydrogenase, mitochondrial OS=Mus musculus GN=Gpd2 PE=1 SV=2 - [GPDM_MOUSE]	-0.13	-0.23	-0.12	-0.18	0.01
Q91WK1	SPRY domain-containing protein 4 OS=Mus musculus GN=Spryd4 PE=2 SV=1 - [SPRY4_MOUSE]	-0.81	-0.61	-1.29	-1.09	0.01
Q8C0P5	Coronin-2A OS=Mus musculus GN=Coro2a PE=2 SV=1 - [COR2A_MOUSE]	-0.22	-0.40	-0.23	-0.40	0.01
Q9WU22	Tyrosine-protein phosphatase non-receptor type 4 OS=Mus musculus GN=Ptpn4 PE=1 SV=1 [PTN4_MOUSE]	0.36	0.39	0.65	0.68	0.01
E9PVG8	Protein 9530053A07Rik OS=Mus musculus GN=9530053A07Rik PE=4 SV=1 - [E9PVG8_MOUSE]	-0.17	-0.13	-0.28	-0.20	0.01
Q9QY33	Tetraspanin-3 OS=Mus musculus GN=Tspan3 PE=1 SV=1 - [TSN3_MOUSE]	0.07	0.11	0.11	0.16	0.01
P24668	Cation-dependent mannose-6-phosphate receptor OS=Mus musculus GN=M6pr PE=1 SV=1 - [MPRD_MOUSE]	0.10	0.11	0.07	0.15	0.01
Q6P5D8	Structural maintenance of chromosomes flexible hinge domain-containing protein 1 OS=Mus musculus GN=Smchd1 PE=1 SV=2 - [SMHD1_MOUSE]	0.22	0.20	0.39	0.32	0.01
D3Z414	Lymphocyte-specific helicase (Fragment) OS=Mus musculus GN=Hells PE=2 SV=1 - [D3Z414_MOUSE]	0.73	0.76	0.41	0.44	0.01
Q8C7D2-2	Isoform 2 of Protein cereblon OS=Mus musculus GN=Crbn - [CRBN_MOUSE]	-0.73	-1.02	-0.45	-0.73	0.01

P27600	Guanine nucleotide-binding protein subunit alpha-12 OS=Mus musculus GN=Gna12 PE=1 SV=3 - [GNA12_MOUSE]	-1.46	-0.96	-1.16	-0.66	0.01
M0QWS4	Ubiquitin-fold modifier conjugating enzyme 1, isoform CRA_c OS=Mus musculus GN=Ufc1 PE=4 SV=1 - [M0QWS4_MOUSE]	0.49	0.65	0.42	0.29	0.01
Q8VHE6	Dynein heavy chain 5, axonemal OS=Mus musculus GN=Dnah5 PE=1 SV=2 - [DYH5_MOUSE]	0.28	0.51	0.35	0.57	0.01
Q91V09	WD repeat-containing protein 13 OS=Mus musculus GN=Wdr13 PE=1 SV=1 - [WDR13_MOUSE]	-0.41	-0.26	-0.49	-0.26	0.01
P85094	Isochorismatase domain-containing protein 2A, mitochondrial OS=Mus musculus GN=Isoc2a PE=2 SV=1 - [ISC2A_MOUSE]	-0.33	-0.61	-0.38	-0.65	0.01
Q9DB16	Calcium-binding protein 39-like OS=Mus musculus GN=Cab39l PE=1 SV=3 - [CB39L_MOUSE]	0.59	1.01	0.53	0.95	0.01
Q9D892	Inosine triphosphate pyrophosphatase OS=Mus musculus GN=Itpa PE=1 SV=2 - [ITPA_MOUSE]	0.23	0.14	0.31	0.21	0.01
K7N759	Protein Gm8356 OS=Mus musculus GN=Gm8356 PE=4 SV=1 - [K7N759_MOUSE]	0.20	0.31	0.14	0.24	0.01
M0QWZ4	Protein Zfp142 OS=Mus musculus GN=Zfp142 PE=4 SV=1 - [M0QWZ4_MOUSE]	0.21	0.38	0.24	0.41	0.01
E9PZ87	Protein Pappa2 OS=Mus musculus GN=Pappa2 PE=4 SV=1 - [E9PZ87_MOUSE]	0.75	0.84	0.42	0.50	0.01
H3BKT0	Homeobox protein cut-like 1 OS=Mus musculus GN=Cux1 PE=2 SV=1 - [H3BKT0_MOUSE]	-0.20	-0.36	-0.31	-0.45	0.01
Q9CQX8	28S ribosomal protein S36, mitochondrial OS=Mus musculus GN=Mrps36 PE=1 SV=1 - [RT36_MOUSE]	-0.32	-0.20	-0.44	-0.29	0.01
Q3UNX5	Acyl-coenzyme A synthetase ACSM3, mitochondrial OS=Mus musculus GN=Acsm3 PE=1 SV=2 - [ACSM3_MOUSE]	0.21	0.09	0.14	0.17	0.01

Q7TPZ8	Carboxypeptidase A1 OS=Mus musculus GN=Cpa1 PE=2 SV=1 - [CBPA1_MOUSE]	1.40	2.36	1.31	2.42	0.01
Q9CYT6	Adenylyl cyclase-associated protein 2 OS=Mus musculus GN=Cap2 PE=1 SV=1 - [CAP2_MOUSE]	0.34	0.17	0.33	0.22	0.01
P38060	Hydroxymethylglutaryl-CoA lyase, mitochondrial OS=Mus musculus GN=Hmgcl PE=1 SV=2 - [HMGCL_MOUSE]	-0.32	-0.22	-0.37	-0.18	0.01
Q9WTR1	Transient receptor potential cation channel subfamily V member 2 OS=Mus musculus GN=Trpv2 PE=1 SV=2 - [TRPV2_MOUSE]	-0.46	-0.42	-0.27	-0.23	0.01
Q8R2U6	Diphosphoinositol polyphosphate phosphohydrolase 2 OS=Mus musculus GN=Nudt4 PE=1 SV=1 - [NUDT4_MOUSE]	-0.30	-0.26	-0.53	-0.41	0.01
Q8BLY2	Probable threonine--tRNA ligase 2, cytoplasmic OS=Mus musculus GN=Tarsl2 PE=2 SV=1 - [SYTC2_MOUSE]	0.16	0.29	0.18	0.31	0.01
P01648	Ig kappa chain V-V region HP 91A3 OS=Mus musculus PE=1 SV=1 - [KV5AF_MOUSE]	0.44	0.80	0.54	0.89	0.01
O09160	Galactoside 2-alpha-L-fucosyltransferase 1 OS=Mus musculus GN=Fut1 PE=2 SV=1 - [FUT1_MOUSE]	-0.99	-1.21	-0.51	-0.95	0.01
Q8CHH9	Septin-8 OS=Mus musculus GN=Sept8 PE=1 SV=4 - [SEPT8_MOUSE]	-0.25	-0.20	-0.39	-0.40	0.01
Q8R326-2	Isoform 2 of Paraspeckle component 1 OS=Mus musculus GN=Pspc1 - [PSPC1_MOUSE]	-0.20	-0.12	-0.10	-0.18	0.01
P16675	Lysosomal protective protein OS=Mus musculus GN=Ctsa PE=1 SV=1 - [PPGB_MOUSE]	-0.35	-0.49	-0.22	-0.47	0.01
Q924A2	Protein capicua homolog OS=Mus musculus GN=Cic PE=1 SV=2 - [CIC_MOUSE]	0.21	0.11	0.20	0.26	0.01
Q9DCC4	Pyrroline-5-carboxylate reductase 3 OS=Mus musculus GN=Pycrl PE=2 SV=2 - [P5CR3_MOUSE]	-0.08	-0.15	-0.18	-0.18	0.01
E9PX60	Fibroblast growth factor receptor (Fragment) OS=Mus musculus GN=Fgfr2 PE=2 SV=1 - [E9PX60_MOUSE]	-0.40	-0.80	-0.57	-0.86	0.01

P53395	Lipoamide acyltransferase component of branched chain alpha-keto acid dehydrogenase complex, mitochondrial OS=Mus musculus GN=Dbt PE=2 SV=2 - [ODB2_MOUSE]	-0.21	-0.10	-0.19	-0.13	0.01
H3BIY2	Protein 2510003E04Rik OS=Mus musculus GN=2510003E04Rik PE=2 SV=1 - [H3BIY2_MOUSE]	-0.23	-0.35	-0.41	-0.53	0.01
Q8CAK3-2	Isoform 2 of UPF0515 protein C19orf66 homolog OS=Mus musculus - [CS066_MOUSE]	-0.31	-0.34	-0.17	-0.20	0.01
Q9D2W5	GRAM domain-containing protein 3 OS=Mus musculus GN=Gramd3 PE=2 SV=1 - [Q9D2W5_MOUSE]	-0.08	-0.15	-0.09	-0.16	0.01
Q99KW3-1	Isoform 1 of TRIO and F-actin-binding protein OS=Mus musculus GN=Triobp - [TARA_MOUSE]	-0.23	-0.20	-0.10	-0.18	0.01
Q9CPW2	Adrenodoxin-like protein, mitochondrial OS=Mus musculus GN=Fdx1l PE=2 SV=1 - [ADXL_MOUSE]	-0.46	-0.44	-0.26	-0.25	0.01
P50427	Steryl-sulfatase OS=Mus musculus GN=Sts PE=2 SV=1 - [STS_MOUSE]	-0.72	-0.43	-0.64	-0.35	0.01
Q8BTE0	UPF0369 protein C6orf57 homolog OS=Mus musculus PE=2 SV=2 - [CF057_MOUSE]	-0.32	-0.22	-0.50	-0.40	0.01
Q6ZWM4	N-alpha-acetyltransferase 38, NatC auxiliary subunit OS=Mus musculus GN=Naa38 PE=3 SV=3 - [NAA38_MOUSE]	-0.36	-0.41	-0.20	-0.24	0.01
Q9JJJ7-4	Isoform 4 of Protein-cysteine N-palmitoyltransferase porcupine OS=Mus musculus GN=Porcn - [PORCN_MOUSE]	-0.43	-0.60	-0.74	-0.95	0.01
P70419	Polypeptide N-acetylgalactosaminyltransferase 3 OS=Mus musculus GN=Galnt3 PE=2 SV=3 - [GALT3_MOUSE]	0.42	0.32	0.26	0.19	0.01
F8VPK0	Protein Ttc37 OS=Mus musculus GN=Ttc37 PE=2 SV=1 - [F8VPK0_MOUSE]	0.24	0.44	0.33	0.52	0.01

J3QNB4	Uncharacterized protein OS=Mus musculus GN=Gm20091 PE=4 SV=1 - [J3QNB4_MOUSE]	-0.29	-0.31	-0.53	-0.54	0.01
P62918	60S ribosomal protein L8 OS=Mus musculus GN=Rpl8 PE=2 SV=2 - [RL8_MOUSE]	-0.05	-0.08	-0.10	-0.12	0.01
Q922Y1	UBX domain-containing protein 1 OS=Mus musculus GN=Ubxn1 PE=1 SV=1 - [UBXN1_MOUSE]	-0.06	-0.11	-0.08	-0.06	0.01
Q9EQI8	39S ribosomal protein L46, mitochondrial OS=Mus musculus GN=Mrpl46 PE=2 SV=1 - [RM46_MOUSE]	0.08	0.18	0.16	0.18	0.01
P56375	Acylphosphatase-2 OS=Mus musculus GN=Acyp2 PE=2 SV=2 - [ACYP2_MOUSE]	-0.44	-0.29	-0.42	-0.21	0.01
Q6P3Y9	Podocan-like protein 1 OS=Mus musculus GN=Podnl1 PE=2 SV=2 - [PONL1_MOUSE]	0.67	0.47	1.05	0.85	0.01
Q8C147	Dedicator of cytokinesis protein 8 OS=Mus musculus GN=Dock8 PE=1 SV=4 - [DOCK8_MOUSE]	-0.55	-0.73	-0.32	-0.50	0.01
Q9Z0U1	Tight junction protein ZO-2 OS=Mus musculus GN=Tjp2 PE=1 SV=2 - [ZO2_MOUSE]	0.11	0.23	0.14	0.22	0.01
O08716	Fatty acid-binding protein 9 OS=Mus musculus GN=Fabp9 PE=2 SV=2 - [FABP9_MOUSE]	0.53	0.50	0.30	0.28	0.01
D3Z3B0	Nicotinamide mononucleotide adenylyltransferase 3 OS=Mus musculus GN=Nmnat3 PE=2 SV=1 - [D3Z3B0_MOUSE]	-1.07	-0.58	-1.17	-0.69	0.01
Q9D7M8	DNA-directed RNA polymerase II subunit RPB4 OS=Mus musculus GN=Polr2d PE=2 SV=2 - [RPB4_MOUSE]	-0.47	-0.28	-0.46	-0.25	0.01
F8VPM7	ELKS/Rab6-interacting/CAST family member 1 OS=Mus musculus GN=Erc1 PE=2 SV=1 - [F8VPM7_MOUSE]	-0.40	-0.31	-0.44	-0.19	0.01
B1AZP2-2	Isoform 2 of Disks large associated protein 4 OS=Mus musculus GN=Dlgap4 - [DLGP4_MOUSE]	0.25	0.24	0.19	0.39	0.01
Q9JIY2-4	Isoform 4 of E3 ubiquitin-protein ligase Hakai OS=Mus musculus GN=Cbll1 - [HAKAI_MOUSE]	-0.04	-0.06	-0.06	-0.08	0.01

Q810A7	ATP-dependent RNA helicase DDX42 OS=Mus musculus GN=Ddx42 PE=1 SV=3 - [DDX42_MOUSE]	0.21	0.12	0.20	0.11	0.01
E9Q8T7	Protein Dnah1 OS=Mus musculus GN=Dnah1 PE=4 SV=1 - [E9Q8T7_MOUSE]	0.47	0.81	0.41	0.75	0.01
Q64674	Spermidine synthase OS=Mus musculus GN=Srm PE=2 SV=1 - [SPEE_MOUSE]	0.22	0.46	0.27	0.39	0.01
P61264	Syntaxin-1B OS=Mus musculus GN=Stx1b PE=1 SV=1 - [STX1B_MOUSE]	-0.14	-0.21	-0.24	-0.32	0.01
O89023	Tripeptidyl-peptidase 1 OS=Mus musculus GN=Tpp1 PE=1 SV=2 - [TPP1_MOUSE]	-0.44	-0.27	-0.62	-0.45	0.01
O35887	Calumenin OS=Mus musculus GN=Calu PE=1 SV=1 - [CALU_MOUSE]	-0.29	-0.27	-0.44	-0.54	0.01
P00683	Ribonuclease pancreatic OS=Mus musculus GN=Rnase1 PE=1 SV=2 - [RNAS1_MOUSE]	0.54	1.12	0.70	1.06	0.01
P11679	Keratin, type II cytoskeletal 8 OS=Mus musculus GN=Krt8 PE=1 SV=4 - [K2C8_MOUSE]	-0.09	-0.05	-0.10	-0.06	0.01
B2RY71	Protein Wdr63 OS=Mus musculus GN=Wdr63 PE=2 SV=1 - [B2RY71_MOUSE]	-0.62	-0.34	-0.72	-0.44	0.01
Q8CC12-3	Isoform 3 of Codanin-1 OS=Mus musculus GN=Cdan1 - [CDAN1_MOUSE]	-0.14	-0.07	-0.16	-0.10	0.01
Q4FZH1	Protein FAM189A2 OS=Mus musculus GN=Fam189a2 PE=1 SV=2 - [F1892_MOUSE]	0.73	0.54	0.50	0.31	0.01
F8WIB1	ADP-ribosylation factor-like protein 1 (Fragment) OS=Mus musculus GN=Arl1 PE=2 SV=1 - [F8WIB1_MOUSE]	0.09	0.20	0.20	0.21	0.01
Q3T9X3	Dynamin-2 OS=Mus musculus GN=Dnm2 PE=2 SV=1 - [Q3T9X3_MOUSE]	0.21	0.37	0.24	0.42	0.01
Q9D113	DNL-type zinc finger protein OS=Mus musculus GN=Dnlz PE=2 SV=1 - [DNLZ_MOUSE]	-0.29	-0.33	-0.53	-0.57	0.01
Q9JJG9	Nitric oxide-associated protein 1 OS=Mus musculus GN=Noa1 PE=1 SV=1 - [NOA1_MOUSE]	-0.49	-0.26	-0.58	-0.36	0.01

P62315	Small nuclear ribonucleoprotein Sm D1 OS=Mus musculus GN=Snrpd1 PE=1 SV=1 - [SMD1_MOUSE]	-0.29	-0.43	-0.22	-0.23	0.01
Q924M7	Mannose-6-phosphate isomerase OS=Mus musculus GN=Mpi PE=2 SV=1 - [MPI_MOUSE]	-0.11	-0.11	-0.20	-0.19	0.01
P57746	V-type proton ATPase subunit D OS=Mus musculus GN=Atp6v1d PE=1 SV=1 - [VATD_MOUSE]	0.33	0.17	0.23	0.17	0.01
P54729	NEDD8 ultimate buster 1 OS=Mus musculus GN=Nub1 PE=1 SV=2 - [NUB1_MOUSE]	0.14	0.23	0.12	0.21	0.01
P97471	Mothers against decapentaplegic homolog 4 OS=Mus musculus GN=Smad4 PE=1 SV=2 - [SMAD4_MOUSE]	0.96	0.63	0.74	0.41	0.01
D3YVN7	Elongation factor Tu OS=Mus musculus GN=Gm9755 PE=3 SV=1 - [D3YVN7_MOUSE]	0.08	0.10	0.05	0.07	0.01
Q9DBE0	Cysteine sulfinic acid decarboxylase OS=Mus musculus GN=Csad PE=2 SV=1 - [CSAD_MOUSE]	-0.55	-0.29	-0.68	-0.49	0.01
Q9QXM1-3	Isoform 3 of Junction-mediating and -regulatory protein OS=Mus musculus GN=Jmy - [JMY_MOUSE]	-1.03	-0.61	-0.78	-0.48	0.01
Q3UGC7	Eukaryotic translation initiation factor 3 subunit J-A OS=Mus musculus GN=Eif3j1 PE=2 SV=1 - [EI3JA_MOUSE]	0.15	0.26	0.12	0.18	0.01
Q8K003	Translation machinery-associated protein 7 OS=Mus musculus GN=Tma7 PE=2 SV=1 [TMA7_MOUSE]	0.25	0.57	0.40	0.52	0.01
Q99KI3	ER membrane protein complex subunit 3 OS=Mus musculus GN=Emc3 PE=2 SV=3 [EMC3_MOUSE]	0.08	0.11	0.05	0.10	0.01
Q8JZQ9	Eukaryotic translation initiation factor 3 subunit B OS=Mus musculus GN=Eif3b PE=1 SV=1 - [EIF3B_MOUSE]	0.22	0.11	0.26	0.26	0.01
Q571I9	Aldehyde dehydrogenase family 16 member A1 OS=Mus musculus GN=Aldh16a1 PE=2 SV=2 - [A16A1_MOUSE]	0.12	0.23	0.13	0.20	0.01

Q9QZK2-2	Isoform 2 of Breast cancer anti-estrogen resistance protein 3 OS=Mus musculus GN=Bcar3 - [BCAR3_MOUSE]	0.22	0.32	0.13	0.23	0.01
F6SQH7	Polymerase delta-interacting protein 2 (Fragment) OS=Mus musculus GN=Poldip2 PE=4 SV=1 - [F6SQH7_MOUSE]	0.28	0.39	0.23	0.18	0.01
Q9Z2I0	LETM1 and EF-hand domain-containing protein 1, mitochondrial OS=Mus musculus GN=Letm1 PE=2 SV=1 - [LETM1_MOUSE]	0.20	0.34	0.16	0.30	0.01
O09131	Glutathione S-transferase omega-1 OS=Mus musculus GN=Gsto1 PE=2 SV=2 [GSTO1_MOUSE]	-0.29	-0.13	-0.31	-0.22	0.01
P22315	Ferrochelatase, mitochondrial OS=Mus musculus GN=Fech PE=1 SV=2 - [HEMH_MOUSE]	-0.38	-0.32	-0.20	-0.20	0.01
O88559-2	Isoform 2 of Menin OS=Mus musculus GN=Men1 - [MEN1_MOUSE]	-0.46	-0.46	-0.25	-0.26	0.01
Q9JKN1	Zinc transporter 7 OS=Mus musculus GN=Slc30a7 PE=1 SV=1 - [ZNT7_MOUSE]	0.83	0.73	0.59	0.35	0.01
P19639	Glutathione S-transferase Mu 3 OS=Mus musculus GN=Gstm3 PE=1 SV=2 - [GSTM4_MOUSE]	-0.24	-0.33	-0.22	-0.14	0.01
Q3TEK6	Dual adapter for phosphotyrosine and 3-phosphotyrosine and 3-phosphoinositide OS=Mus musculus GN=Dapp1 PE=2 SV=1 - [Q3TEK6_MOUSE]	-0.35	-0.49	-0.21	-0.35	0.01
Q8CDG3-2	Isoform 2 of Deubiquitinating protein VCIP135 OS=Mus musculus GN=Vcpip1 - [VCIP1_MOUSE]	0.47	0.30	0.22	0.31	0.01
J3QNB1	La-related protein 1 OS=Mus musculus GN=Larp1 PE=4 SV=1 [J3QNB1_MOUSE]	0.21	0.16	0.09	0.14	0.01
E9Q2S9	Coiled-coil domain-containing protein 43 OS=Mus musculus GN=Ccdc43 PE=2 SV=1 - [E9Q2S9_MOUSE]	-0.67	-0.48	-1.06	-0.73	0.01

F6V5H4	Sushi, nidogen and EGF-like domain-containing protein 1 (Fragment) OS=Mus musculus GN=Sned1 PE=4 SV=1 - [F6V5H4_MOUSE]	-1.11	-0.71	-0.87	-0.48	0.01
Q8JZN5	Acyl-CoA dehydrogenase family member 9, mitochondrial OS=Mus musculus GN=Acad9 PE=2 SV=2 - [ACAD9_MOUSE]	-0.19	-0.16	-0.33	-0.23	0.01
B0QZV3	Protein Slc9a6 OS=Mus musculus GN=Slc9a6 PE=2 SV=1 - [B0QZV3_MOUSE]	-0.51	-0.29	-0.35	-0.25	0.01
P70372	ELAV-like protein 1 OS=Mus musculus GN=Elavl1 PE=1 SV=2 - [ELAV1_MOUSE]	0.04	0.08	0.09	0.07	0.01
P24369	Peptidyl-prolyl cis-trans isomerase B OS=Mus musculus GN=Ppib PE=2 SV=2 - [PPIB_MOUSE]	0.16	0.21	0.09	0.19	0.01
P01580	Interferon gamma OS=Mus musculus GN=Ifng PE=2 SV=1 - [IFNG_MOUSE]	-0.33	-0.53	-0.24	-0.45	0.01
P28033	CCAAT/enhancer-binding protein beta OS=Mus musculus GN=Cebpb PE=1 SV=1 - [CEBPB_MOUSE]	-0.85	-0.47	-0.84	-0.46	0.01
Q60716-2	Isoform IIa of Prolyl 4-hydroxylase subunit alpha-2 OS=Mus musculus GN=P4ha2 - [P4HA2_MOUSE]	-0.52	-0.98	-0.66	-1.11	0.01
Q8BFZ9	Erlin-2 OS=Mus musculus GN=Erlin2 PE=1 SV=1 - [ERLN2_MOUSE]	0.14	0.25	0.12	0.20	0.01
Q8BYC6	Serine/threonine-protein kinase TAO3 OS=Mus musculus GN=Taok3 PE=1 SV=2 [TAOK3_MOUSE]	-0.26	-0.46	-0.25	-0.46	0.01
P16125	L-lactate dehydrogenase B chain OS=Mus musculus GN=Ldhb PE=1 SV=2 - [LDHB_MOUSE]	0.17	0.24	0.10	0.19	0.01
Q5DTW7-2	Isoform 2 of Uncharacterized protein KIAA1551 OS=Mus musculus GN=Kiaa1551 - [K1551_MOUSE]	-0.70	-1.31	-0.94	-1.55	0.01
Q8BL97-3	Isoform 3 of Serine/arginine-rich splicing factor 7 OS=Mus musculus GN=Srsf7 - [SRSF7_MOUSE]	-0.32	-0.17	-0.34	-0.20	0.01

Q61235	Beta-2-syntrophin OS=Mus musculus GN=Sntb2 PE=1 SV=2 [SNTB2_MOUSE]	-0.27	-0.38	-0.25	-0.16	0.01
Q3UYC0	Protein phosphatase 1H OS=Mus musculus GN=Ppm1h PE=1 SV=1 - [PPM1H_MOUSE]	-0.48	-0.31	-0.35	-0.21	0.01
P62274	40S ribosomal protein S29 OS=Mus musculus GN=Rps29 PE=2 SV=2 - [RS29_MOUSE]	0.35	0.40	0.17	0.27	0.01
A2AV19	Exosome complex component RRP4 (Fragment) OS=Mus musculus GN=Exosc2 PE=2 SV=1 - [A2AV19_MOUSE]	-0.28	-0.51	-0.28	-0.28	0.01
Q9CYH2	Redox-regulatory protein FAM213A OS=Mus musculus GN=Fam213a PE=1 SV=2 - [F213A_MOUSE]	-0.36	-0.25	-0.22	-0.16	0.01
Q60932-2	Isoform Mt-VDAC1 of Voltage-dependent anion-selective channel protein 1 OS=Mus musculus GN=Vdac1 - [VDAC1_MOUSE]	-0.32	-0.20	-0.26	-0.14	0.01
Q8CIN4	Serine/threonine-protein kinase PAK 2 OS=Mus musculus GN=Pak2 PE=1 SV=1 - [PAK2_MOUSE]	0.13	0.17	0.10	0.22	0.01
Q5DID3-3	Isoform 3 of Uromodulin-like 1 OS=Mus musculus GN=Umodl1 - [UROL1_MOUSE]	-0.57	-0.53	-1.02	-0.97	0.01
Q91YT7	Protein Ythdf2 OS=Mus musculus GN=Ythdf2 PE=2 SV=1 - [Q91YT7_MOUSE]	-0.31	-0.23	-0.51	-0.43	0.01
Q9D2X6	Colon SVA-like protein OS=Mus musculus GN=Sval1 PE=2 SV=1 - [Q9D2X6_MOUSE]	0.50	0.50	0.21	0.41	0.01
Q4VC33	Macrophage erythroblast attacher OS=Mus musculus GN=Maea PE=1 SV=1 [MAEA_MOUSE]	1.59	0.94	1.41	0.75	0.01
Q62376	U1 small nuclear ribonucleoprotein 70 kDa OS=Mus musculus GN=Snrnp70 PE=1 SV=2 - [RU17_MOUSE]	-0.09	-0.18	-0.15	-0.22	0.01
P01630	Ig kappa chain V-II region 7S34.1 OS=Mus musculus PE=1 SV=1 - [KV2A6_MOUSE]	-0.44	-0.78	-0.41	-0.75	0.01

Q6P3D0	U8 snoRNA-decapping enzyme OS=Mus musculus GN=Nudt16 PE=1 SV=1 - [NUD16_MOUSE]	-0.14	-0.08	-0.19	-0.16	0.01
Q9D8W5	26S proteasome non-ATPase regulatory subunit 12 OS=Mus musculus GN=Psmd12 PE=1 SV=4 - [PSD12_MOUSE]	0.30	0.22	0.47	0.44	0.01
Q8K3A0	Iron-sulfur cluster co-chaperone protein HscB, mitochondrial OS=Mus musculus GN=Hscb PE=2 SV=2 - [HSC20_MOUSE]	-0.50	-0.43	-0.20	-0.37	0.01
E9Q5F1	Protein Olfr430 OS=Mus musculus GN=Olfr430 PE=2 SV=1 - [E9Q5F1_MOUSE]	0.28	0.41	0.17	0.30	0.01
Q64444	Carbonic anhydrase 4 OS=Mus musculus GN=Ca4 PE=1 SV=1 - [CAH4_MOUSE]	0.34	0.29	0.20	0.46	0.01
Q922S4	cGMP-dependent 3',5'-cyclic phosphodiesterase OS=Mus musculus GN=Pde2a PE=1 SV=3 - [PDE2A_MOUSE]	0.35	0.68	0.51	0.81	0.01
Q9Z2Q6	Septin-5 OS=Mus musculus GN=Sept5 PE=1 SV=2 - [SEPT5_MOUSE]	-0.23	-0.12	-0.17	-0.12	0.01
Q9D4H8	Cullin-2 OS=Mus musculus GN=Cul2 PE=1 SV=2 - [CUL2_MOUSE]	0.22	0.31	0.13	0.28	0.01
Q9CQ65	S-methyl-5'-thioadenosine phosphorylase OS=Mus musculus GN=Mtap PE=2 SV=1 - [MTAP_MOUSE]	-0.14	-0.22	-0.10	-0.18	0.01
Q8VIG0-2	Isoform 2 of Zinc finger CCHC domain-containing protein 14 OS=Mus musculus GN=Zcchc14 - [ZCH14_MOUSE]	-0.96	-0.52	-1.00	-0.56	0.01
Q6PI17	Metalloproteinase inhibitor 2 OS=Mus musculus GN=Timp2 PE=2 SV=1 - [Q6PI17_MOUSE]	-0.79	-0.45	-1.09	-0.75	0.01
P12961	Neuroendocrine protein 7B2 OS=Mus musculus GN=Scg5 PE=1 SV=1 - [7B2_MOUSE]	-0.42	-0.39	-0.75	-0.72	0.01
Q8CF89	TGF-beta-activated kinase 1 and MAP3K7-binding protein 1 OS=Mus musculus GN=Tab1 PE=1 SV=2 - [TAB1_MOUSE]	0.16	0.33	0.21	0.19	0.01
P60843	Eukaryotic initiation factor 4A-I OS=Mus musculus GN=Eif4a1 PE=2 SV=1 - [IF4A1_MOUSE]	0.34	0.53	0.23	0.46	0.01

P62869	Transcription elongation factor B polypeptide 2 OS=Mus musculus GN=Tceb2 PE=1 SV=1 - [ELOB_MOUSE]	-0.14	-0.10	-0.23	-0.19	0.01
P70441	Na(+)/H(+) exchange regulatory cofactor NHE-RF1 OS=Mus musculus GN=Slc9a3r1 PE=1 SV=3 - [NHRF1_MOUSE]	-0.29	-0.21	-0.39	-0.19	0.01
Q8BGS2	BolA-like protein 2 OS=Mus musculus GN=Bola2 PE=1 SV=1 [BOLA2_MOUSE]	-0.15	-0.20	-0.31	-0.32	0.01
Q99KD5	Protein unc-45 homolog A OS=Mus musculus GN=Unc45a PE=1 SV=2 - [UN45A_MOUSE]	0.41	0.19	0.48	0.40	0.01
Q921E6	Polycomb protein EED OS=Mus musculus GN=Eed PE=1 SV=1 - [EED_MOUSE]	0.32	0.52	0.56	0.77	0.01
Q922K9	Tyrosine-protein kinase FRK OS=Mus musculus GN=Frk PE=1 SV=3 - [FRK_MOUSE]	-0.08	-0.03	-0.08	-0.06	0.01
Q8C3X8	Lipase maturation factor 2 OS=Mus musculus GN=Lmf2 PE=2 SV=1 - [LMF2_MOUSE]	0.10	0.15	0.19	0.24	0.01
Q8BZW8	NHL repeat-containing protein 2 OS=Mus musculus GN=Nhlrc2 PE=2 SV=1 - [NHLC2_MOUSE]	0.20	0.30	0.25	0.13	0.01
Q91WE6-3	Isoform 3 of Threonylcarbamoyladenosine tRNA methylthiotransferase OS=Mus musculus GN=Cdkal1 - [CDKAL_MOUSE]	-0.75	-1.09	-0.45	-0.80	0.01
Q9JII6	Alcohol dehydrogenase [NADP(+)] OS=Mus musculus GN=Akr1a1 PE=1 SV=3 - [AK1A1_MOUSE]	-0.21	-0.21	-0.13	-0.10	0.01
F6WZH1	Dipeptidase 3 (Fragment) OS=Mus musculus GN=Mbd3 PE=4 SV=1 - [F6WZH1_MOUSE]	0.13	0.19	0.14	0.08	0.01
Q8CAA7	Glucose 1,6-bisphosphate synthase OS=Mus musculus GN=Pgm2l1 PE=1 SV=1 - [PGM2L_MOUSE]	-0.59	-0.95	-0.42	-0.78	0.01
Q9JM96	Cdc42 effector protein 4 OS=Mus musculus GN=Cdc42ep4 PE=1 SV=1 - [BORG4_MOUSE]	-0.23	-0.44	-0.24	-0.42	0.01

A2BE93	Protein SET (Fragment) OS=Mus musculus GN=Set PE=2 SV=1 - [A2BE93_MOUSE]	-0.15	-0.08	-0.20	-0.15	0.01
Q8CJ26	Death domain-containing membrane protein NRADD OS=Mus musculus GN=Nradd PE=1 SV=1 - [NRADD_MOUSE]	0.07	0.06	0.12	0.11	0.01
Q3TBA3	Antigen peptide transporter 1 OS=Mus musculus GN=Tap1 PE=2 SV=1 - [Q3TBA3_MOUSE]	0.23	0.10	0.23	0.16	0.01
Q9CR16	Peptidyl-prolyl cis-trans isomerase D OS=Mus musculus GN=Ppid PE=1 SV=3 - [PPID_MOUSE]	0.07	0.16	0.12	0.16	0.01
Q9CQ56	Vesicle transport protein USE1 OS=Mus musculus GN=Use1 PE=2 SV=1 - [USE1_MOUSE]	0.41	0.40	0.23	0.21	0.01
O08797	Protein Serpinb9 OS=Mus musculus GN=Serpinb9 PE=2 SV=1 - [O08797_MOUSE]	-0.13	-0.16	-0.25	-0.27	0.01
P26041	Moesin OS=Mus musculus GN=Msn PE=1 SV=3 - [MOES_MOUSE]	0.19	0.17	0.11	0.09	0.01
P13020-2	Isoform 2 of Gelsolin OS=Mus musculus GN=Gsn - [GELS_MOUSE]	-0.17	-0.11	-0.15	-0.07	0.01
O08842	GDNF family receptor alpha-2 OS=Mus musculus GN=Gfra2 PE=2 SV=1 - [GFRA2_MOUSE]	-0.46	-0.50	-0.24	-0.29	0.01
Q8R1F6-2	Isoform 2 of Protein HID1 OS=Mus musculus GN=Hid1 - [HID1_MOUSE]	0.31	0.14	0.21	0.17	0.01
F8WIG5	Protein diaphanous homolog 3 OS=Mus musculus GN=Diap3 PE=4 SV=2 - [F8WIG5_MOUSE]	0.87	1.43	1.56	2.12	0.01
P10637	Microtubule-associated protein tau OS=Mus musculus GN=Mapt PE=1 SV=3 - [TAU_MOUSE]	-0.16	-0.38	-0.28	-0.37	0.01
Q9Z1Y4	Thyroid receptor-interacting protein 6 OS=Mus musculus GN=Trip6 PE=1 SV=1 - [TRIP6_MOUSE]	0.18	0.22	0.36	0.37	0.01
G3UZ60	Small ubiquitin-related modifier 3 OS=Mus musculus GN=Sumo3 PE=2 SV=1 - [G3UZ60_MOUSE]	-0.09	-0.17	-0.09	-0.17	0.01
B1AS14	Uncharacterized protein (Fragment) OS=Mus musculus GN=Fam213b PE=2 SV=1 - [B1AS14_MOUSE]	-0.33	-0.59	-0.30	-0.56	0.01

Q3UM45	Protein phosphatase 1 regulatory subunit 7 OS=Mus musculus GN=Ppp1r7 PE=1 SV=2 - [PP1R7_MOUSE]	0.17	0.07	0.18	0.14	0.01
R4GML6	Centriolin (Fragment) OS=Mus musculus GN=Cep110 PE=4 SV=1 - [R4GML6_MOUSE]	0.37	0.58	0.85	0.81	0.01
Q8BH60-2	Isoform 2 of Golgi-associated PDZ and coiled-coil motif-containing protein OS=Mus musculus GN=Gopc - [GOPC_MOUSE]	-0.17	-0.36	-0.31	-0.42	0.01
A2AVA0-2	Isoform 2 of Sushi, von Willebrand factor type A, EGF and pentraxin domain-containing protein 1 OS=Mus musculus GN=Svep1 - [SVEP1_MOUSE]	0.95	0.54	1.33	0.92	0.01
Q923P0-2	Isoform 2 of Collagen alpha-1(XX) chain OS=Mus musculus GN=Col20a1 - [COKA1_MOUSE]	-0.29	-0.50	-0.23	-0.36	0.01
Q69ZK0	Phosphatidylinositol 3,4,5-trisphosphate-dependent Rac exchanger 1 protein OS=Mus musculus GN=Prex1 PE=1 SV=2 [PREX1_MOUSE]	0.09	0.19	0.10	0.16	0.01
P49615	Cyclin-dependent kinase 5 OS=Mus musculus GN=Cdk5 PE=1 SV=1 - [CDK5_MOUSE]	0.31	0.50	0.22	0.37	0.01
P48771	Cytochrome c oxidase subunit 7A2, mitochondrial OS=Mus musculus GN=Cox7a2 PE=1 SV=2 - [CX7A2_MOUSE]	0.20	0.19	0.09	0.12	0.01
Q9Z0Z4-2	Isoform 2 of Hephaestin OS=Mus musculus GN=Heph - [HEPH_MOUSE]	-0.17	-0.31	-0.14	-0.27	0.01
P35550	rRNA 2'-O-methyltransferase fibrillarin OS=Mus musculus GN=Fbl PE=1 SV=2 - [FBRL_MOUSE]	0.18	0.26	0.11	0.21	0.01
P70296	Phosphatidylethanolamine-binding protein 1 OS=Mus musculus GN=Pebp1 PE=1 SV=3 - [PEBP1_MOUSE]	-0.03	-0.06	-0.03	-0.03	0.01
O35405	Phospholipase D3 OS=Mus musculus GN=Pld3 PE=2 SV=1 - [PLD3_MOUSE]	-0.12	-0.20	-0.28	-0.17	0.01

Q91XD2	LIM and senescent cell antigen-like-containing domain protein 2 OS=Mus musculus GN=Lims2 PE=1 SV=1 - [LIMS2_MOUSE]	0.23	0.12	0.28	0.18	0.01
Q02819	Nucleobindin-1 OS=Mus musculus GN=Nucb1 PE=1 SV=2 - [NUCB1_MOUSE]	-0.17	-0.12	-0.10	-0.08	0.01
P21107	Tropomyosin alpha-3 chain OS=Mus musculus GN=Tpm3 PE=1 SV=3 - [TPM3_MOUSE]	-0.75	-0.73	-0.43	-0.38	0.01
Q02105	Complement C1q subcomponent subunit C OS=Mus musculus GN=C1qc PE=2 SV=2 - [C1QC_MOUSE]	-0.55	-0.85	-0.39	-0.51	0.01
Q8K0C5	Zymogen granule membrane protein 16 OS=Mus musculus GN=Zg16 PE=2 SV=1 - [ZG16_MOUSE]	0.11	0.15	0.09	0.07	0.01
Q8C3I8	Protein FAM203A OS=Mus musculus GN=Fam203a PE=2 SV=1 - [F203A_MOUSE]	0.38	0.22	0.35	0.18	0.01
Q9CQ86	Migration and invasion enhancer 1 OS=Mus musculus GN=Mien1 PE=1 SV=1 - [MIEN1_MOUSE]	-0.41	-0.26	-0.33	-0.17	0.01
G5E8T3	Signal recognition particle 19 kDa protein OS=Mus musculus GN=Srp19 PE=4 SV=1 - [G5E8T3_MOUSE]	0.63	0.93	0.38	0.67	0.01
D3Z6H3	Dynactin 6, isoform CRA_b OS=Mus musculus GN=Dctn6 PE=4 SV=1 - [D3Z6H3_MOUSE]	-0.11	-0.10	-0.18	-0.09	0.01
Q8VEN2-2	Isoform 2 of Placenta-expressed transcript 1 protein OS=Mus musculus GN=Plet1 - [PLET1_MOUSE]	0.59	0.88	0.36	0.65	0.01
Q3UVK0	Endoplasmic reticulum metallopeptidase 1 OS=Mus musculus GN=Ermp1 PE=1 SV=2 - [ERMP1_MOUSE]	-0.21	-0.47	-0.32	-0.47	0.01
Q9DCD2	Pre-mRNA-splicing factor SYF1 OS=Mus musculus GN=Xab2 PE=2 SV=1 - [SYF1_MOUSE]	-0.43	-0.34	-0.25	-0.19	0.01
Q8R2S1-2	Isoform 2 of Ribosomal protein S6 kinase-like 1 OS=Mus musculus GN=Rps6kl1 - [RPKL1_MOUSE]	-0.94	-0.75	-0.59	-0.40	0.01
Q9DBG3	AP-2 complex subunit beta OS=Mus musculus GN=Ap2b1 PE=1 SV=1 - [AP2B1_MOUSE]	0.06	0.11	0.07	0.13	0.01

Q91X83	S-adenosylmethionine synthase isoform type-1 OS=Mus musculus GN=Mat1a PE=2 SV=1 - [METK1_MOUSE]	0.30	0.35	0.16	0.21	0.01
O35465-2	Isoform 2 of Peptidyl-prolyl cis-trans isomerase FKBP8 OS=Mus musculus GN=Fkbp8 - [FKBP8_MOUSE]	0.23	0.16	0.17	0.09	0.01
Q6PFX9	Tankyrase-1 OS=Mus musculus GN=Tnks PE=1 SV=1 - [TNKS1_MOUSE]	0.29	0.15	0.33	0.19	0.01
O08997	Copper transport protein ATOX1 OS=Mus musculus GN=Atox1 PE=2 SV=1 [ATOX1_MOUSE]	0.24	0.35	0.14	0.27	0.01
Q62277	Synaptophysin OS=Mus musculus GN=Syp PE=1 SV=2 - [SYPH_MOUSE]	-0.16	-0.14	-0.29	-0.27	0.01
Q5XJF6	Ribosomal protein OS=Mus musculus GN=Rpl10a PE=2 SV=1 - [Q5XJF6_MOUSE]	0.11	0.13	0.05	0.12	0.01
P70404	Isocitrate dehydrogenase [NAD] subunit gamma 1, mitochondrial OS=Mus musculus GN=Idh3g PE=1 SV=1 - [IDHG1_MOUSE]	-0.22	-0.22	-0.09	-0.16	0.01
D3Z3A9	Reticulocalbin-3 (Fragment) OS=Mus musculus GN=Rcn3 PE=2 SV=1 - [D3Z3A9_MOUSE]	-0.09	-0.16	-0.16	-0.22	0.01
Q8K1J6	CCA tRNA nucleotidyltransferase 1, mitochondrial OS=Mus musculus GN=Trnt1 PE=2 SV=1 - [TRNT1_MOUSE]	0.34	0.41	0.16	0.31	0.01
Q8K2T8	RNA polymerase II-associated factor 1 homolog OS=Mus musculus GN=Paf1 PE=2 SV=1 - [PAF1_MOUSE]	-0.34	-0.27	-0.27	-0.13	0.01
Q811P8-2	Isoform 2 of Rho GTPase-activating protein 32 OS=Mus musculus GN=Arhgap32 - [RHG32_MOUSE]	-0.37	-0.20	-0.24	-0.18	0.01
G5E8C4	MCG142017, isoform CRA_a OS=Mus musculus GN=Tmtc3 PE=4 SV=1 - [G5E8C4_MOUSE]	0.16	0.12	0.24	0.13	0.01
Q61553	Fascin OS=Mus musculus GN=Fscn1 PE=1 SV=4 - [FSCN1_MOUSE]	-0.33	-0.26	-0.23	-0.13	0.01
O70252	Heme oxygenase 2 OS=Mus musculus GN=Hmox2 PE=2 SV=1 - [HMOX2_MOUSE]	0.24	0.17	0.27	0.11	0.01

Q9D1L0	Coiled-coil-helix-coiled-coil-helix domain-containing protein 2, mitochondrial OS=Mus musculus GN=Chchd2 PE=1 SV=1 - [CHCH2_MOUSE]	0.11	0.11	0.09	0.05	0.01
Q8BJ71	Nuclear pore complex protein Nup93 OS=Mus musculus GN=Nup93 PE=2 SV=1 - [NUP93_MOUSE]	0.26	0.18	0.24	0.11	0.01
P34928	Apolipoprotein C-I OS=Mus musculus GN=Apoc1 PE=2 SV=1 - [APOC1_MOUSE]	-0.66	-0.31	-0.63	-0.39	0.01
E9Q2I4	Uncharacterized protein OS=Mus musculus GN=Elmsan1 PE=4 SV=1 - [E9Q2I4_MOUSE]	0.09	0.17	0.08	0.16	0.01
Q80YW5	B box and SPRY domain-containing protein OS=Mus musculus GN=Bspry PE=1 SV=1 - [BSPRY_MOUSE]	0.26	0.48	0.25	0.47	0.01
B7ZDD9	Carnitine O-acetyltransferase OS=Mus musculus GN=Crat PE=2 SV=1 - [B7ZDD9_MOUSE]	-0.25	-0.32	-0.44	-0.56	0.01
Q9CQJ8	NADH dehydrogenase [ubiquinone] 1 beta subcomplex subunit 9 OS=Mus musculus GN=Ndufb9 PE=1 SV=3 - [NDUB9_MOUSE]	0.22	0.41	0.23	0.43	0.01
Q9CX86	Heterogeneous nuclear ribonucleoprotein A0 OS=Mus musculus GN=Hnrnpa0 PE=1 SV=1 - [ROA0_MOUSE]	0.38	0.36	0.32	0.15	0.01
Q9D2E2-2	Isoform 2 of Target of EGR1 protein 1 OS=Mus musculus GN=Toe1 - [TOE1_MOUSE]	0.22	0.37	0.41	0.56	0.01
P35980	60S ribosomal protein L18 OS=Mus musculus GN=Rpl18 PE=2 SV=3 [RL18_MOUSE]	-0.28	-0.35	-0.14	-0.23	0.01
Q8K199	COX assembly mitochondrial protein 2 homolog OS=Mus musculus GN=Cmc2 PE=2 SV=1 - [COXM2_MOUSE]	0.22	0.38	0.40	0.56	0.01
Q8C570	mRNA export factor OS=Mus musculus GN=Rae1 PE=1 SV=1 - [RAE1L_MOUSE]	-0.16	-0.10	-0.13	-0.07	0.01
F6WKE4	Epsilon-sarcoglycan (Fragment) OS=Mus musculus GN=Sgce PE=2 SV=1 - [F6WKE4_MOUSE]	-0.37	-0.58	-0.70	-0.34	0.01

Q3UJU9	Regulator of microtubule dynamics protein 3 OS=Mus musculus GN=Rmdn3 PE=1 SV=2 - [RMD3_MOUSE]	-0.15	-0.10	-0.10	-0.20	0.01
Q8BTS4	Nuclear pore complex protein Nup54 OS=Mus musculus GN=Nup54 PE=1 SV=1 - [NUP54_MOUSE]	0.45	0.24	0.32	0.20	0.01
Q922B1	O-acetyl-ADP-ribose deacetylase MACROD1 OS=Mus musculus GN=Macrod1 PE=2 SV=2 - [MACD1_MOUSE]	-0.59	-0.30	-0.35	-0.31	0.01
P25085-2	Isoform 2 of Interleukin-1 receptor antagonist protein OS=Mus musculus GN=Il1rn - [IL1RA_MOUSE]	0.53	0.28	0.34	0.25	0.01
Q61576	Peptidyl-prolyl cis-trans isomerase FKBP10 OS=Mus musculus GN=Fkbp10 PE=1 SV=2 - [FKB10_MOUSE]	0.03	0.03	0.07	0.05	0.01
E9Q3Q6	CD166 antigen OS=Mus musculus GN=Alcam PE=2 SV=1 - [E9Q3Q6_MOUSE]	-0.18	-0.22	-0.40	-0.30	0.01
Q91VR8	Protein BRICK1 OS=Mus musculus GN=Brk1 PE=2 SV=1 - [BRK1_MOUSE]	0.31	0.18	0.15	0.18	0.01
Q9WUA2	Phenylalanine--tRNA ligase beta subunit OS=Mus musculus GN=Farsb PE=2 SV=2 [SYFB_MOUSE]	0.20	0.23	0.14	0.33	0.01
Q9JK81	UPF0160 protein MYG1 mitochondrial OS=Mus musculus GN=Myg1 PE=2 SV=1 - [MYG1_MOUSE]	-0.11	-0.15	-0.10	-0.06	0.01
P68134	Actin, alpha skeletal muscle OS=Mus musculus GN=Acta1 PE=1 SV=1 - [ACTS_MOUSE]	0.19	0.39	0.21	0.36	0.01
Q9EPK7-2	Isoform 2 of Exportin-7 OS=Mus musculus GN=Xpo7 - [XPO7_MOUSE]	0.46	0.30	0.29	0.20	0.01
G5E843	Roundabout homolog 1 OS=Mus musculus GN=Robo1 PE=4 SV=1 - [G5E843_MOUSE]	-0.33	-0.51	-0.57	-0.26	0.01
Q9R0E1	Procollagen-lysine,2-oxoglutarate 5-dioxygenase 3 OS=Mus musculus GN=Plod3 PE=1 SV=1 [PLOD3_MOUSE]	-0.46	-0.53	-0.22	-0.35	0.01

Q9CSN1	SNW domain-containing protein 1 OS=Mus musculus GN=Snw1 PE=1 SV=3 [SNW1_MOUSE]	0.17	0.21	0.19	0.35	0.01
P47968	Ribose-5-phosphate isomerase OS=Mus musculus GN=Rpia PE=2 SV=2 - [RPIA_MOUSE]	-0.13	-0.20	-0.15	-0.28	0.01
Q5SXY1-3	Isoform 4 of Cytospin-B OS=Mus musculus GN=Specc1 - [CYTSB_MOUSE]	-0.38	-0.49	-0.20	-0.31	0.01
Q9ET01	Glycogen phosphorylase, liver form OS=Mus musculus GN=Pygl PE=1 SV=4 - [PYGL_MOUSE]	0.15	0.14	0.09	0.22	0.01
Q9EST5	Acidic leucine-rich nuclear phosphoprotein 32 family member B OS=Mus musculus GN=Anp32b PE=1 SV=1 - [AN32B_MOUSE]	-0.20	-0.13	-0.32	-0.24	0.01
P40142	Transketolase OS=Mus musculus GN=Tkt PE=1 SV=1 - [TKT_MOUSE]	-0.16	-0.10	-0.21	-0.12	0.01
Q8VDW0	ATP-dependent RNA helicase DDX39A OS=Mus musculus GN=Ddx39a PE=2 SV=1 - [DX39A_MOUSE]	0.31	0.70	0.51	0.75	0.01
Q6P4T2	U5 small nuclear ribonucleoprotein 200 kDa helicase OS=Mus musculus GN=Snrnp200 PE=2 SV=1 - [U520_MOUSE]	0.32	0.13	0.28	0.21	0.01
Q8C4Y1	Max protein OS=Mus musculus GN=Max PE=2 SV=1 - [Q8C4Y1_MOUSE]	-0.19	-0.07	-0.16	-0.19	0.01
B7ZNL3	Tpm1 protein OS=Mus musculus GN=Tpm1 PE=2 SV=1 - [B7ZNL3_MOUSE]	0.10	0.09	0.19	0.18	0.01
P10518	Delta-aminolevulinic acid dehydratase OS=Mus musculus GN=Alad PE=1 SV=1 - [HEM2_MOUSE]	-0.25	-0.14	-0.31	-0.17	0.01
E9Q9E8	SLAM family member 5 OS=Mus musculus GN=Cd84 PE=2 SV=1 - [E9Q9E8_MOUSE]	0.23	0.26	0.44	0.47	0.01
P42932	T-complex protein 1 subunit theta OS=Mus musculus GN=Cct8 PE=1 SV=3 - [TCPQ_MOUSE]	0.09	0.17	0.13	0.21	0.01
H9KUX9	Retinoid isomerohydrolase OS=Mus musculus GN=Rpe65 PE=2 SV=1 - [H9KUX9_MOUSE]	-0.08	-0.06	-0.06	-0.03	0.01

D3YU60	Microsomal glutathione S-transferase 1 OS=Mus musculus GN=Mgst1 PE=2 SV=1 - [D3YU60_MOUSE]	-0.24	-0.33	-0.52	-0.53	0.01
Q9CR20	Immediate early response 3-interacting protein 1 OS=Mus musculus GN=Ier3ip1 PE=2 SV=1 - [IR3IP_MOUSE]	0.39	0.54	0.75	0.91	0.01
Q60590	Alpha-1-acid glycoprotein 1 OS=Mus musculus GN=Orm1 PE=1 SV=1 - [A1AG1_MOUSE]	-0.20	-0.11	-0.29	-0.20	0.01
Q9JHL1	Na(+)/H(+) exchange regulatory cofactor NHE-RF2 OS=Mus musculus GN=Slc9a3r2 PE=1 SV=2 - [NHRF2_MOUSE]	-0.18	-0.15	-0.31	-0.32	0.01
O89053	Coronin-1A OS=Mus musculus GN=Coro1a PE=1 SV=5 - [COR1A_MOUSE]	0.08	0.06	0.14	0.12	0.01
Q62028	Secretory phospholipase A2 receptor OS=Mus musculus GN=Pla2r1 PE=1 SV=1 - [PLA2R_MOUSE]	-0.22	-0.16	-0.14	-0.09	0.01
P18524	Ig heavy chain V region RF OS=Mus musculus PE=1 SV=1 - [HVM53_MOUSE]	0.38	0.28	0.25	0.15	0.01
P62962	Profilin-1 OS=Mus musculus GN=Pfn1 PE=1 SV=2 - [PROF1_MOUSE]	-0.13	-0.09	-0.13	-0.06	0.01
Q9CXW3	Calcyclin-binding protein OS=Mus musculus GN=Cacybp PE=1 SV=1 - [CYBP_MOUSE]	0.22	0.09	0.21	0.24	0.01
E9Q6U4	Protein Pfdn4 OS=Mus musculus GN=Pfdn4 PE=2 SV=1 - [E9Q6U4_MOUSE]	-0.11	-0.14	-0.15	-0.24	0.01
Q80W00-2	Isoform 2 of Serine/threonine-protein phosphatase 1 regulatory subunit 10 OS=Mus musculus GN=Ppp1r10 - [PP1RA_MOUSE]	0.21	0.11	0.28	0.18	0.01
Q9JLI6	Selenocysteine lyase OS=Mus musculus GN=Scly PE=1 SV=1 - [SCLY_MOUSE]	-0.43	-0.17	-0.30	-0.37	0.01
Q149L6-3	Isoform 3 of DnaJ homolog subfamily B member 14 OS=Mus musculus GN=Dnajb14 - [DJB14_MOUSE]	0.44	0.40	0.25	0.20	0.01
O08915	AH receptor-interacting protein OS=Mus musculus GN=Aip PE=1 SV=1 - [AIP_MOUSE]	0.26	0.46	0.27	0.22	0.01

Q9CZL5	Pterin-4-alpha-carbinolamine dehydratase 2 OS=Mus musculus GN=Pcbd2 PE=1 SV=2 - [PHS2_MOUSE]	-0.48	-0.25	-0.32	-0.23	0.01
Q9R1P1	Proteasome subunit beta type-3 OS=Mus musculus GN=Psmb3 PE=1 SV=1 - [PSB3_MOUSE]	-0.19	-0.08	-0.10	-0.15	0.01
P60041	Somatostatin OS=Mus musculus GN=Sst PE=2 SV=1 - [SMS_MOUSE]	-0.51	-0.31	-0.78	-0.59	0.01
E9Q0E2	Protein 2700049A03Rik OS=Mus musculus GN=2700049A03Rik PE=2 SV=1 - [E9Q0E2_MOUSE]	0.46	0.40	0.84	0.78	0.01
P37889-2	Isoform 2 of Fibulin-2 OS=Mus musculus GN=Fbln2 - [FBLN2_MOUSE]	-0.35	-0.27	-0.30	-0.13	0.01
Q8VCE1	DnaJ homolog subfamily C member 28 OS=Mus musculus GN=Dnajc28 PE=2 SV=2 - [DJC28_MOUSE]	0.37	0.21	0.28	0.16	0.01
Q3U2G2	Heat shock 70 kDa protein 4 OS=Mus musculus GN=Hspa4 PE=2 SV=1 - [Q3U2G2_MOUSE]	-0.15	-0.09	-0.20	-0.11	0.01
Q9Z0N1	Eukaryotic translation initiation factor 2 subunit 3, X-linked OS=Mus musculus GN=Eif2s3x PE=1 SV=2 - [IF2G_MOUSE]	0.27	0.16	0.42	0.31	0.01
P55284	Cadherin-5 OS=Mus musculus GN=Cdh5 PE=1 SV=2 - [CADH5_MOUSE]	-0.48	-0.46	-0.27	-0.23	0.01
D3Z4D9	C2 domain-containing protein 3 (Fragment) OS=Mus musculus GN=C2cd3 PE=2 SV=1 - [D3Z4D9_MOUSE]	0.27	0.37	0.14	0.24	0.01
Q9JME7	Trafficking protein particle complex subunit 2-like protein OS=Mus musculus GN=Trappc2l PE=2 SV=1 - [TPC2L_MOUSE]	0.15	0.27	0.27	0.39	0.01
Q9CPU2	NADH dehydrogenase [ubiquinone] 1 beta subcomplex subunit 2, mitochondrial OS=Mus musculus GN=Ndufb2 PE=3 SV=1 - [NDUB2_MOUSE]	-0.06	-0.09	-0.13	-0.15	0.01
Q8K3J1	NADH dehydrogenase [ubiquinone] iron-sulfur protein 8, mitochondrial OS=Mus musculus GN=Ndufs8 PE=1 SV=1 - [NDUS8_MOUSE]	0.21	0.21	0.08	0.21	0.01

Q5NCM6	Epsin-2 OS=Mus musculus GN=Epn2 PE=2 SV=1 - [Q5NCM6_MOUSE]	-0.28	-0.25	-0.51	-0.50	0.01
P19783	Cytochrome c oxidase subunit 4 isoform 1, mitochondrial OS=Mus musculus GN=Cox4i1 PE=1 SV=2 - [COX41_MOUSE]	0.08	0.11	0.05	0.06	0.01
Q99LC8	Translation initiation factor eIF-2B subunit alpha OS=Mus musculus GN=Eif2b1 PE=2 SV=1 - [EI2BA_MOUSE]	0.49	0.20	0.52	0.41	0.01
F8VQ29	Protein Iqgap3 OS=Mus musculus GN=Iqgap3 PE=2 SV=1 - [F8VQ29_MOUSE]	0.11	0.20	0.20	0.29	0.01
Q9QYI3	DnaJ homolog subfamily C member 7 OS=Mus musculus GN=Dnajc7 PE=1 SV=2 - [DNJC7_MOUSE]	0.23	0.17	0.40	0.27	0.01
Q9CQC9	GTP-binding protein SAR1b OS=Mus musculus GN=Sar1b PE=1 SV=1 - [SAR1B_MOUSE]	-0.53	-0.32	-0.31	-0.23	0.01
Q8VCH8	UBX domain-containing protein 4 OS=Mus musculus GN=Ubxn4 PE=1 SV=1 - [UBXN4_MOUSE]	0.20	0.45	0.38	0.54	0.01
Q80WG5	Leucine-rich repeat-containing protein 8A OS=Mus musculus GN=Lrrc8a PE=1 SV=1 - [LRC8A_MOUSE]	0.58	0.57	0.31	0.29	0.01
Q80V94	AP-4 complex subunit epsilon-1 OS=Mus musculus GN=Ap4e1 PE=2 SV=3 - [AP4E1_MOUSE]	-0.23	-0.46	-0.25	-0.48	0.01
P32921	Tryptophan--tRNA ligase, cytoplasmic OS=Mus musculus GN=Wars PE=1 SV=2 - [SYWC_MOUSE]	0.33	0.39	0.17	0.23	0.01
Q61301-2	Isoform 2 of Catenin alpha-2 OS=Mus musculus GN=Ctnna2 - [CTNA2_MOUSE]	-0.38	-0.60	-0.24	-0.38	0.01
Q9Z0M3	Cadherin-20 OS=Mus musculus GN=Cdh20 PE=2 SV=1 - [CAD20_MOUSE]	-0.79	-1.41	-0.63	-1.26	0.01
Q99LC5	Electron transfer flavoprotein subunit alpha, mitochondrial OS=Mus musculus GN=Etfa PE=1 SV=2 - [ETFA_MOUSE]	-0.26	-0.16	-0.40	-0.26	0.01
A2A7U2	Protein Zmym6 OS=Mus musculus GN=Zmym6 PE=2 SV=1 - [A2A7U2_MOUSE]	-0.46	-0.25	-0.64	-0.42	0.01

Q9D6F9	Tubulin beta-4A chain OS=Mus musculus GN=Tubb4a PE=1 SV=3 - [TBB4A_MOUSE]	0.08	0.13	0.09	0.18	0.01
P31809-3	Isoform 3 of Carcinoembryonic antigen-related cell adhesion molecule 1 OS=Mus musculus GN=Ceacam1 - [CEAM1_MOUSE]	0.20	0.25	0.11	0.14	0.01
Q9CQE7	Endoplasmic reticulum-Golgi intermediate compartment protein 3 OS=Mus musculus GN=Ergic3 PE=2 SV=1 - [ERGI3_MOUSE]	0.45	0.35	0.19	0.25	0.01
P31786	Acyl-CoA-binding protein OS=Mus musculus GN=Dbi PE=1 SV=2 - [ACBP_MOUSE]	-0.16	-0.12	-0.29	-0.22	0.01
Q9QXP7	Complement C1q tumor necrosis factor-related protein 1 OS=Mus musculus GN=C1qtnf1 PE=2 SV=1 - [C1QT1_MOUSE]	0.43	0.82	0.40	0.78	0.01
Q9Z2V4	Phosphoenolpyruvate carboxykinase, cytosolic [GTP] OS=Mus musculus GN=Pck1 PE=2 SV=1 - [PCKGC_MOUSE]	-0.49	-0.23	-0.38	-0.24	0.01
P58774-2	Isoform 2 of Tropomyosin beta chain OS=Mus musculus GN=Tpm2 - [TPM2_MOUSE]	0.13	0.22	0.09	0.16	0.01
Q3V1H3	Hephaestin-like protein 1 OS=Mus musculus GN=Hephl1 PE=2 SV=2 - [HPHL1_MOUSE]	-0.42	-0.31	-0.73	-0.62	0.01
Q9D1G1	Ras-related protein Rab-1B OS=Mus musculus GN=Rab1b PE=1 SV=1 - [RAB1B_MOUSE]	0.17	0.33	0.16	0.30	0.01
Q61578	NADPH:adrenodoxin oxidoreductase, mitochondrial OS=Mus musculus GN=Fdxr PE=2 SV=1 - [ADRO_MOUSE]	-0.34	-0.29	-0.12	-0.25	0.01
Q9WTS2	Alpha-(1,6)-fucosyltransferase OS=Mus musculus GN=Fut8 PE=2 SV=2 - [FUT8_MOUSE]	0.28	0.47	0.22	0.48	0.01
O35595	Protein patched homolog 2 OS=Mus musculus GN=Ptch2 PE=2 SV=2 - [PTC2_MOUSE]	-0.36	-0.70	-0.38	-0.72	0.01
Q03963	Interferon-induced, double-stranded RNA-activated protein kinase OS=Mus musculus GN=Eif2ak2 PE=1 SV=2 - [E2AK2_MOUSE]	0.25	0.29	0.46	0.54	0.01

P61164	Alpha-centractin OS=Mus musculus GN=Actr1a PE=2 SV=1 - [ACTZ_MOUSE]	-0.11	-0.12	-0.23	-0.21	0.01
Q9D9V3-2	Isoform 2 of Ethylmalonyl-CoA decarboxylase OS=Mus musculus GN=Echdc1 - [ECHD1_MOUSE]	-0.08	-0.11	-0.19	-0.16	0.01
Q9CR26	Vacuolar protein sorting associated protein VTA1 homolog OS=Mus musculus GN=Vta1 PE=1 SV=1 - [VTA1_MOUSE]	0.11	0.25	0.26	0.17	0.01
P62077	Mitochondrial import inner membrane translocase subunit Tim8 B OS=Mus musculus GN=Timm8b PE=3 SV=1 - [TIM8B_MOUSE]	-0.44	-0.32	-0.37	-0.16	0.01
Q9D8U8	Sorting nexin-5 OS=Mus musculus GN=Snx5 PE=1 SV=1 - [SNX5_MOUSE]	0.15	0.31	0.17	0.30	0.01
F6UCX4	Protein Sptbn5 (Fragment) OS=Mus musculus GN=Sptbn5 PE=4 SV=1 - [F6UCX4_MOUSE]	0.22	0.44	0.36	0.57	0.01
Q61080	Forkhead box protein F1 OS=Mus musculus GN=Foxf1 PE=2 SV=2 - [FOXF1_MOUSE]	0.16	0.30	0.15	0.29	0.01
D3YVR7	L-lactate dehydrogenase (Fragment) OS=Mus musculus GN=Ldhc PE=2 SV=1 - [D3YVR7_MOUSE]	0.16	0.12	0.06	0.17	0.01
P05063	Fructose-bisphosphate aldolase C OS=Mus musculus GN=Aldoc PE=1 SV=4 - [ALDOC_MOUSE]	-0.30	-0.41	-0.21	-0.19	0.01
Q7TQI7	Ankyrin repeat and BTB/POZ domain-containing protein 2 OS=Mus musculus GN=Abtb2 PE=2 SV=1 [ABTB2_MOUSE]	-0.72	-1.45	-0.91	-1.65	0.01
Q80VQ1	Leucine-rich repeat-containing protein 1 OS=Mus musculus GN=Lrrc1 PE=2 SV=2 - [LRRC1_MOUSE]	0.06	0.15	0.15	0.14	0.01
B1AY10	Transcriptional repressor NF-X1 OS=Mus musculus GN=Nfx1 PE=2 SV=1 - [NFX1_MOUSE]	0.26	0.13	0.26	0.13	0.01
O09110-2	Isoform 1 of Dual specificity mitogen-activated protein kinase kinase 3 OS=Mus musculus GN=Map2k3 [MP2K3_MOUSE]	0.25	0.41	0.32	0.58	0.01

O54724	Polymerase I and transcript release factor OS=Mus musculus GN=Ptrf PE=1 SV=1 - [PTRF_MOUSE]	-0.13	-0.08	-0.21	-0.13	0.01
Q8CGY6-2	Isoform 2 of Protein unc-45 homolog B OS=Mus musculus GN=Unc45b - [UN45B_MOUSE]	0.44	0.81	0.37	0.74	0.01
D3YVU1	Ubiquitin carboxyl-terminal hydrolase 16 OS=Mus musculus GN=Usp16 PE=2 SV=1 - [D3YVU1_MOUSE]	-0.51	-0.69	-0.25	-0.60	0.01
Q8R2K3	Single-stranded DNA-binding protein OS=Mus musculus GN=Ssbp1 PE=2 SV=1 - [Q8R2K3_MOUSE]	-0.32	-0.13	-0.30	-0.20	0.01
Q9WVL3	Solute carrier family 12 member 7 OS=Mus musculus GN=Slc12a7 PE=1 SV=1 - [S12A7_MOUSE]	-0.23	-0.14	-0.18	-0.09	0.01
P09925	Surfeit locus protein 1 OS=Mus musculus GN=Surf1 PE=2 SV=3 - [SURF1_MOUSE]	-0.17	-0.29	-0.11	-0.21	0.01
E9Q8G5	Protein A430033K04Rik OS=Mus musculus GN=A430033K04Rik PE=2 SV=1 - [E9Q8G5_MOUSE]	-0.47	-0.36	-0.29	-0.18	0.01
P30412	Peptidyl-prolyl cis-trans isomerase C OS=Mus musculus GN=Ppic PE=1 SV=1 - [PPIC_MOUSE]	-0.10	-0.12	-0.07	-0.05	0.01
P57759	Endoplasmic reticulum resident protein 29 OS=Mus musculus GN=Erp29 PE=1 SV=2 [ERP29_MOUSE]	0.08	0.03	0.07	0.06	0.01
Q922U1	U4/U6 small nuclear ribonucleoprotein Prp3 OS=Mus musculus GN=Prpf3 PE=1 SV=1 - [PRPF3_MOUSE]	-0.06	-0.09	-0.14	-0.08	0.01
Q9Z160	Conserved oligomeric Golgi complex subunit 1 OS=Mus musculus GN=Cog1 PE=2 SV=3 - [COG1_MOUSE]	0.19	0.33	0.44	0.24	0.01
Q9DCJ5	NADH dehydrogenase [ubiquinone] 1 alpha subcomplex subunit 8 OS=Mus musculus GN=Ndufa8 PE=1 SV=3 - [NDUA8_MOUSE]	0.30	0.34	0.24	0.13	0.01
Q9QYC0	Alpha-adducin OS=Mus musculus GN=Add1 PE=1 SV=2 - [ADDA_MOUSE]	-0.21	-0.29	-0.11	-0.26	0.01
P82347	Delta-sarcoglycan OS=Mus musculus GN=Sgcd PE=1 SV=1 - [SGCD_MOUSE]	-0.31	-0.45	-0.31	-0.17	0.01

F6WNY1	Protein Tex16 (Fragment) OS=Mus musculus GN=Tex16 PE=2 SV=1 - [F6WNY1_MOUSE]	0.72	0.42	1.11	0.81	0.01
D3YWC9	MTSS1-like protein OS=Mus musculus GN=Mtss1l PE=2 SV=1 - [D3YWC9_MOUSE]	-0.70	-0.71	-0.36	-0.37	0.01
Q8R0S2	IQ motif and SEC7 domain-containing protein 1 OS=Mus musculus GN=Iqsec1 PE=1 SV=2 - [IQEC1_MOUSE]	-0.34	-0.52	-0.20	-0.38	0.01
O70503	Estradiol 17-beta-dehydrogenase 12 OS=Mus musculus GN=Hsd17b12 PE=2 SV=1 - [DHB12_MOUSE]	0.23	0.39	0.16	0.26	0.01
Q9EPM5-3	Isoform 3 of Syncoilin OS=Mus musculus GN=Sync - [SYNCI_MOUSE]	-0.18	-0.36	-0.19	-0.37	0.01
D3Z440	COP9 signalosome complex subunit 7a (Fragment) OS=Mus musculus GN=Cops7a PE=2 SV=1 - [D3Z440_MOUSE]	0.16	0.15	0.06	0.11	0.01
P04919-2	Isoform Kidney of Band 3 anion transport protein OS=Mus musculus GN=Slc4a1 - [B3AT_MOUSE]	-0.14	-0.25	-0.24	-0.36	0.01
Q6GTM0	Ifit2 protein OS=Mus musculus GN=Ifit2 PE=2 SV=1 - [Q6GTM0_MOUSE]	0.28	0.28	0.54	0.56	0.01
Q9JKF7	39S ribosomal protein L39, mitochondrial OS=Mus musculus GN=Mrpl39 PE=2 SV=4 - [RM39_MOUSE]	-0.16	-0.29	-0.31	-0.43	0.01
Q7TMC8	L-fucose kinase OS=Mus musculus GN=Fuk PE=2 SV=1 - [Q7TMC8_MOUSE]	0.41	0.27	0.37	0.16	0.01
Q8VCQ3	Nuclear receptor-binding factor 2 OS=Mus musculus GN=Nrbf2 PE=1 SV=1 - [NRBF2_MOUSE]	0.23	0.12	0.21	0.11	0.01
Q91V24	ATP-binding cassette sub-family A member 7 OS=Mus musculus GN=Abca7 PE=1 SV=1 - [ABCA7_MOUSE]	-0.40	-0.25	-0.66	-0.51	0.01
F6YU83	Serine/threonine-protein kinase PDIK1L (Fragment) OS=Mus musculus GN=Pdik1l PE=2 SV=1 - [F6YU83_MOUSE]	-0.18	-0.09	-0.24	-0.16	0.01
Q8C669	E3 ubiquitin-protein ligase pellino homolog 1 OS=Mus musculus GN=Peli1 PE=1 SV=2 - [PELI1_MOUSE]	-0.23	-0.14	-0.37	-0.28	0.01

O88792	Junctional adhesion molecule A OS=Mus musculus GN=F11r PE=1 SV=2 - [JAM1_MOUSE]	0.15	0.27	0.17	0.11	0.01
Q3T9A3	Serine/threonine-protein kinase MARK2 OS=Mus musculus GN=Mark2 PE=2 SV=1 - [Q3T9A3_MOUSE]	-0.19	-0.20	-0.24	-0.09	0.01
P63038	60 kDa heat shock protein, mitochondrial OS=Mus musculus GN=Hspd1 PE=1 SV=1 - [CH60_MOUSE]	0.10	0.15	0.06	0.09	0.01
P14431	H-2 class I histocompatibility antigen, Q9 alpha chain (Fragment) OS=Mus musculus GN=H2-Q9 PE=3 SV=1 - [HA19_MOUSE]	-0.92	-1.36	-0.50	-0.94	0.01
Q6LC96	RXR alpha 2 OS=Mus musculus GN=Rxra PE=2 SV=1 - [Q6LC96_MOUSE]	-0.11	-0.23	-0.15	-0.27	0.01
Q9ERR1-2	Isoform 2 of Nuclear distribution protein nudE-like 1 OS=Mus musculus GN=Ndel1 - [NDEL1_MOUSE]	0.20	0.11	0.29	0.19	0.01
Q9Z1G3	V-type proton ATPase subunit C 1 OS=Mus musculus GN=Atp6v1c1 PE=1 SV=4 - [VATC1_MOUSE]	0.33	0.20	0.23	0.13	0.01
O09167	60S ribosomal protein L21 OS=Mus musculus GN=Rpl21 PE=2 SV=3 [RL21_MOUSE]	0.46	0.64	0.23	0.49	0.01
P06745	Glucose-6-phosphate isomerase OS=Mus musculus GN=Gpi PE=1 SV=4 - [G6PI_MOUSE]	-0.15	-0.08	-0.18	-0.10	0.01
Q8R3N6	THO complex subunit 1 OS=Mus musculus GN=Thoc1 PE=1 SV=1 - [THOC1_MOUSE]	0.44	0.51	0.18	0.38	0.01
Q80YS6	Actin filament-associated protein 1 OS=Mus musculus GN=Afap1 PE=1 SV=1 [AFAP1_MOUSE]	0.12	0.21	0.23	0.32	0.01
Q8R0W0	Epiplakin OS=Mus musculus GN=Eppk1 PE=1 SV=2 - [EPIPL_MOUSE]	0.18	0.10	0.09	0.08	0.01
Q8VE88-2	Isoform 2 of Protein FAM114A2 OS=Mus musculus GN=Fam114a2 - [F1142_MOUSE]	0.19	0.08	0.21	0.23	0.01
P61600	N-alpha-acetyltransferase 20 OS=Mus musculus GN=Naa20 PE=2 SV=1 - [NAA20_MOUSE]	1.06	0.63	1.08	0.48	0.01

Q9CY66-3	Isoform 3 of H/ACA ribonucleoprotein complex subunit 1 OS=Mus musculus GN=Gar1 - [GAR1_MOUSE]	-0.33	-0.24	-0.58	-0.49	0.01
O35683	NADH dehydrogenase [ubiquinone] 1 alpha subcomplex subunit 1 OS=Mus musculus GN=Ndufa1 PE=2 SV=1 - [NDUA1_MOUSE]	-0.40	-0.50	-0.18	-0.34	0.01
A6ZI44	Fructose-bisphosphate aldolase OS=Mus musculus GN=Aldoa PE=2 SV=1 - [A6ZI44_MOUSE]	-0.09	-0.15	-0.12	-0.22	0.01
P97363	Serine palmitoyltransferase 2 OS=Mus musculus GN=Sptlc2 PE=2 SV=2 - [SPTC2_MOUSE]	0.24	0.57	0.31	0.46	0.01
A3KGU7	Spectrin alpha chain, non-erythrocytic 1 OS=Mus musculus GN=Sptan1 PE=2 SV=1 - [A3KGU7_MOUSE]	-0.64	-0.30	-0.73	-0.42	0.01
P70122	Ribosome maturation protein SBDS OS=Mus musculus GN=Sbds PE=1 SV=4 - [SBDS_MOUSE]	0.38	0.22	0.20	0.18	0.01
Q9WVC3	Caveolin-2 OS=Mus musculus GN=Cav2 PE=1 SV=1 - [CAV2_MOUSE]	-0.47	-0.97	-0.60	-1.09	0.01
B2RUF0	Y box protein 2 OS=Mus musculus GN=Ybx2 PE=2 SV=1 - [B2RUF0_MOUSE]	-1.15	-1.27	-0.47	-0.88	0.01
Q920Q8-4	Isoform 4 of Influenza virus NS1A-binding protein homolog OS=Mus musculus GN=Ivns1abp - [NS1BP_MOUSE]	0.57	0.31	0.31	0.28	0.01
Q9JLZ6-2	Isoform 2 of Hypermethylated in cancer 2 protein OS=Mus musculus GN=Hic2 - [HIC2_MOUSE]	-0.18	-0.28	-0.10	-0.20	0.01
P23116	Eukaryotic translation initiation factor 3 subunit A OS=Mus musculus GN=Eif3a PE=1 SV=5 - [EIF3A_MOUSE]	0.20	0.17	0.17	0.07	0.01
Q9DC63-3	Isoform 3 of F-box only protein 3 OS=Mus musculus GN=Fbxo3 - [FBX3_MOUSE]	-0.10	-0.11	-0.08	-0.04	0.01
Q8R480	Nuclear pore complex protein Nup85 OS=Mus musculus GN=Nup85 PE=1 SV=1 - [NUP85_MOUSE]	-0.69	-0.45	-0.52	-0.25	0.01
F8WI30	Sorting nexin-7 OS=Mus musculus GN=Snx7 PE=2 SV=1 - [F8WI30_MOUSE]	0.08	0.18	0.12	0.08	0.01

Q8VF20	MCG142237 OS=Mus musculus GN=Olfr509 PE=2 SV=1 - [Q8VF20_MOUSE]	0.52	0.26	0.63	0.36	0.01
Q8R238	Serine dehydratase-like OS=Mus musculus GN=Sdsl PE=2 SV=1 - [SDSL_MOUSE]	0.32	0.65	0.41	0.74	0.01
Q9DAT5	Mitochondrial tRNA-specific 2-thiouridylase 1 OS=Mus musculus GN=Trmu PE=2 SV=1 - [MTU1_MOUSE]	-0.19	-0.28	-0.38	-0.48	0.01
P56391	Cytochrome c oxidase subunit 6B1 OS=Mus musculus GN=Cox6b1 PE=1 SV=2 - [CX6B1_MOUSE]	0.17	0.25	0.09	0.23	0.01
G3UW90	Trichorhinophalangeal syndrome I (Human), isoform CRA_b OS=Mus musculus GN=Trps1 PE=4 SV=1 [G3UW90_MOUSE]	-1.02	-0.60	-0.81	-0.40	0.01
Q80UK7-2	Isoform 2 of Spindle assembly abnormal protein 6 homolog OS=Mus musculus GN=Sass6 - [SAS6_MOUSE]	0.30	0.46	0.17	0.34	0.01
A2AS86	Glucagon (Fragment) OS=Mus musculus GN=Gcg PE=2 SV=1 - [A2AS86_MOUSE]	-0.23	-0.36	-0.14	-0.31	0.01
P12023-3	Isoform APP751 of Amyloid beta A4 protein OS=Mus musculus GN=App - [A4_MOUSE]	0.27	0.17	0.21	0.10	0.01
P62270	40S ribosomal protein S18 OS=Mus musculus GN=Rps18 PE=1 SV=3 - [RS18_MOUSE]	-0.04	-0.07	-0.11	-0.09	0.01
A2BH40-3	Isoform 3 of AT-rich interactive domain-containing protein 1A OS=Mus musculus GN=Arid1a - [ARI1A_MOUSE]	0.11	0.20	0.17	0.08	0.01
Q6P5F9	Exportin-1 OS=Mus musculus GN=Xpo1 PE=1 SV=1 - [XPO1_MOUSE]	0.13	0.32	0.20	0.32	0.01
E9QK16	Protocadherin Fat 3 OS=Mus musculus GN=Fat3 PE=2 SV=1 - [E9QK16_MOUSE]	0.38	0.47	0.19	0.27	0.01
Q91XV3	Brain acid soluble protein 1 OS=Mus musculus GN=Basp1 PE=1 SV=3 - [BASP1_MOUSE]	-0.48	-0.29	-0.75	-0.49	0.01
P70202	Latexin OS=Mus musculus GN=Lxn PE=1 SV=2 - [LXN_MOUSE]	0.52	0.51	0.18	0.45	0.01
P50428	Arylsulfatase A OS=Mus musculus GN=Arsa PE=2 SV=2 - [ARSA_MOUSE]	-0.27	-0.26	-0.43	-0.18	0.01

G3X924	Plasmalemma vesicle associated protein OS=Mus musculus GN=Plvap PE=4 SV=1 - [G3X924_MOUSE]	-0.23	-0.36	-0.13	-0.24	0.01
P17156	Heat shock-related 70 kDa protein 2 OS=Mus musculus GN=Hspa2 PE=1 SV=2 - [HSP72_MOUSE]	0.07	0.02	0.06	0.06	0.01
P63325	40S ribosomal protein S10 OS=Mus musculus GN=Rps10 PE=1 SV=1 - [RS10_MOUSE]	0.17	0.19	0.11	0.08	0.01
P60521	Gamma-aminobutyric acid receptor-associated protein-like 2 OS=Mus musculus GN=Gabarapl2 PE=1 SV=1 - [GBRL2_MOUSE]	-0.27	-0.18	-0.39	-0.18	0.01
Q9ERF3	WD repeat-containing protein 61 OS=Mus musculus GN=Wdr61 PE=2 SV=1 - [WDR61_MOUSE]	-0.48	-0.19	-0.31	-0.30	0.01
F6VUN6	MICAL C-terminal-like protein (Fragment) OS=Mus musculus GN=Micalcl PE=4 SV=1 - [F6VUN6_MOUSE]	-0.10	-0.15	-0.22	-0.26	0.01
Q91WC0	Histone-lysine N-methyltransferase setd3 OS=Mus musculus GN=Setd3 PE=1 SV=1 - [SETD3_MOUSE]	0.19	0.11	0.30	0.21	0.01
O88532	Zinc finger RNA-binding protein OS=Mus musculus GN=Zfr PE=1 SV=2 - [ZFR_MOUSE]	0.13	0.10	0.08	0.05	0.01
Q91WG4	Elongator complex protein 2 OS=Mus musculus GN=Elp2 PE=1 SV=1 - [ELP2_MOUSE]	-0.86	-1.71	-1.09	-2.04	0.01
Q6P6M7	O-phosphoseryl-tRNA(Sec) selenium transferase OS=Mus musculus GN=Sepsecs PE=1 SV=2 - [SPCS_MOUSE]	-0.16	-0.25	-0.39	-0.39	0.01
B9EKI3	TATA element modulatory factor OS=Mus musculus GN=Tmf1 PE=1 SV=2 - [TMF1_MOUSE]	0.12	0.09	0.10	0.04	0.01
Q62469	Integrin alpha-2 OS=Mus musculus GN=Itga2 PE=1 SV=2 - [ITA2_MOUSE]	0.20	0.12	0.16	0.08	0.01
Q9DC71	28S ribosomal protein S15, mitochondrial OS=Mus musculus GN=Mrps15 PE=2 SV=2 - [RT15_MOUSE]	0.13	0.15	0.27	0.28	0.01

Q80TY0-2	Isoform 2 of Formin-binding protein 1 OS=Mus musculus GN=Fnbp1 - [FNBP1_MOUSE]	0.16	0.23	0.11	0.11	0.01
Q61189	Methylosome subunit pICln OS=Mus musculus GN=Clns1a PE=1 SV=1 - [ICLN_MOUSE]	-0.11	-0.15	-0.06	-0.09	0.01
Q91VM9	Inorganic pyrophosphatase 2, mitochondrial OS=Mus musculus GN=Ppa2 PE=2 SV=1 - [IPYR2_MOUSE]	-0.30	-0.19	-0.42	-0.21	0.01
Q8CF93	Polypeptide N-acetylgalactosaminyltransferase 13 OS=Mus musculus GN=Galnt13 PE=2 SV=1 - [GLT13_MOUSE]	-0.53	-0.26	-0.72	-0.45	0.01
D6RIN4	Dynein light chain 2, cytoplasmic OS=Mus musculus GN=Dynll2 PE=2 SV=1 - [D6RIN4_MOUSE]	-0.40	-0.18	-0.45	-0.26	0.01
P15306	Thrombomodulin OS=Mus musculus GN=Thbd PE=1 SV=1 - [TRBM_MOUSE]	0.31	0.64	0.38	0.71	0.01
Q91YL3	Uridine-cytidine kinase-like 1 OS=Mus musculus GN=Uckl1 PE=1 SV=1 - [UCKL1_MOUSE]	0.60	0.26	0.77	0.60	0.01
Q9ESY9	Gamma-interferon-inducible lysosomal thiol reductase OS=Mus musculus GN=Ifi30 PE=1 SV=3 - [GILT_MOUSE]	0.28	0.21	0.31	0.11	0.01
Q8VEE1	LIM and cysteine-rich domains protein 1 OS=Mus musculus GN=Lmcd1 PE=1 SV=1 - [LMCD1_MOUSE]	0.12	0.19	0.11	0.07	0.01
Q99K28	ADP-ribosylation factor GTPase-activating protein 2 OS=Mus musculus GN=Arfgap2 PE=1 SV=1 - [ARFG2_MOUSE]	0.16	0.08	0.23	0.20	0.01
P17182	Alpha-enolase OS=Mus musculus GN=Eno1 PE=1 SV=3 - [ENOA_MOUSE]	0.19	0.31	0.11	0.24	0.01
Q3UEG6	Alanine--glyoxylate aminotransferase 2, mitochondrial OS=Mus musculus GN=Agxt2 PE=2 SV=1 - [AGT2_MOUSE]	-0.07	-0.13	-0.11	-0.18	0.01
G3UW57	Protein Rab11fip2 OS=Mus musculus GN=Rab11fip2 PE=4 SV=1 - [G3UW57_MOUSE]	-0.37	-0.54	-0.19	-0.36	0.01

Q8K268	ATP-binding cassette sub-family F member 3 OS=Mus musculus GN=Abcf3 PE=1 SV=1 [ABCF3_MOUSE]	0.41	0.33	0.25	0.16	0.01
Q9QXC1	Fetuin-B OS=Mus musculus GN=Fetub PE=1 SV=1 - [FETUB_MOUSE]	-0.24	-0.17	-0.31	-0.13	0.01
F7DBB3	Protein Ahnak2 (Fragment) OS=Mus musculus GN=Ahnak2 PE=2 SV=1 - [F7DBB3_MOUSE]	-0.57	-0.48	-1.07	-1.02	0.01
P48722	Heat shock 70 kDa protein 4L OS=Mus musculus GN=Hspa4l PE=1 SV=2 - [HS74L_MOUSE]	0.10	0.07	0.17	0.13	0.01
Q8K385	Ferric-chelate reductase 1 OS=Mus musculus GN=FRRS1 PE=1 SV=1 - [FRRS1_MOUSE]	-0.39	-0.23	-0.63	-0.48	0.01
Q3UW98	Chloride channel calcium activated 7 OS=Mus musculus GN=AI747448 PE=2 SV=1 - [Q3UW98_MOUSE]	-0.46	-0.78	-0.30	-0.62	0.01
Q3UVL4	Vacuolar protein sorting associated protein 51 homolog OS=Mus musculus GN=Vps51 PE=2 SV=2 - [VPS51_MOUSE]	0.35	0.25	0.41	0.15	0.01
Q7TMK6	Protein Hook homolog 2 OS=Mus musculus GN=Hook2 PE=1 SV=3 - [HOOK2_MOUSE]	0.49	0.91	0.39	0.58	0.01
E9Q8E3	Protein Abi3bp OS=Mus musculus GN=Abi3bp PE=2 SV=1 - [E9Q8E3_MOUSE]	0.18	0.27	0.11	0.28	0.01
Q8K4L0	ATP-dependent RNA helicase DDX54 OS=Mus musculus GN=Ddx54 PE=1 SV=1 - [DDX54_MOUSE]	0.20	0.43	0.26	0.48	0.01
O88952	Protein lin-7 homolog C OS=Mus musculus GN=Lin7c PE=1 SV=2 - [LIN7C_MOUSE]	-0.15	-0.06	-0.16	-0.09	0.01
F6VVY4	Protein Slc25a1 (Fragment) OS=Mus musculus GN=Slc25a1 PE=3 SV=1 - [F6VVY4_MOUSE]	-0.06	-0.17	-0.11	-0.16	0.01
Q9CWG9	Biogenesis of lysosome related organelles complex 1 subunit 2 OS=Mus musculus GN=Bloc1s2 PE=1 SV=1 - [BL1S2_MOUSE]	-0.11	-0.15	-0.24	-0.28	0.01
K3W4Q5	Protein Fam186a OS=Mus musculus GN=Fam186a PE=4 SV=1 - [K3W4Q5_MOUSE]	-0.26	-0.53	-0.45	-0.73	0.01

P37913	DNA ligase 1 OS=Mus musculus GN=Lig1 PE=1 SV=2 - [DNLI1_MOUSE]	0.40	0.35	0.34	0.13	0.01
Q60989	E3 ubiquitin-protein ligase XIAP OS=Mus musculus GN=Xiap PE=1 SV=2 - [XIAP_MOUSE]	0.17	0.15	0.08	0.08	0.01
Q3UWA6-2	Isoform 2 of Heat- stable enterotoxin receptor OS=Mus musculus GN=Gucy2c - [GUC2C_MOUSE]	-0.10	-0.19	-0.11	-0.22	0.01
Q3TZ89-2	Isoform 2 of Protein transport protein Sec31B OS=Mus musculus GN=Sec31b - [SC31B_MOUSE]	0.29	0.60	0.42	0.74	0.01
Q3TW96	UDP-N-acetylhexosamine pyrophosphorylase-like protein 1 OS=Mus musculus GN=Uap1l1 PE=2 SV=1 - [UAP1L_MOUSE]	-0.12	-0.13	-0.12	-0.24	0.01
Q8BKU8-2	Isoform 2 of Transmembrane protein 87B OS=Mus musculus GN=Tmem87b - [TM87B_MOUSE]	0.18	0.36	0.18	0.36	0.01
A8Y5N8	Pleckstrin homology domain-containing family F member 2 (Fragment) OS=Mus musculus GN=Plekhf2 PE=2 SV=1 - [A8Y5N8_MOUSE]	-0.71	-1.17	-0.43	-0.90	0.01
Q9CY34	NEDD8-conjugating enzyme UBE2F OS=Mus musculus GN=Ube2f PE=1 SV=1 - [UB2FA_MOUSE]	0.30	0.39	0.13	0.36	0.01
Q8BVL9	Janus kinase and microtubule-interacting protein 1 OS=Mus musculus GN=Jakmip1 PE=1 SV=2 - [JKIP1_MOUSE]	-0.23	-0.39	-0.15	-0.32	0.01
Q99JF8	PC4 and SFRS1-interacting protein OS=Mus musculus GN=Psip1 PE=1 SV=1 [PSIP1_MOUSE]	0.19	0.38	0.26	0.48	0.01
L7N466	Protein Gm2382 OS=Mus musculus GN=Gm2382 PE=4 SV=1 - [L7N466_MOUSE]	0.21	0.18	0.08	0.13	0.01
A2A4A1	DnaJ homolog subfamily C member 24 OS=Mus musculus GN=Dnajc24 PE=2 SV=1 - [A2A4A1_MOUSE]	-0.19	-0.48	-0.31	-0.26	0.01
A2ALS5	Protein Rap1gap OS=Mus musculus GN=Rap1gap PE=2 SV=1 - [A2ALS5_MOUSE]	-0.19	-0.39	-0.25	-0.46	0.01

E9PZJ7	Protein Vmn2r107 OS=Mus musculus GN=Vmn2r107 PE=2 SV=1 - [E9PZJ7_MOUSE]	0.21	0.17	0.39	0.35	0.01
Q60759	Glutaryl-CoA dehydrogenase, mitochondrial OS=Mus musculus GN=Gcdh PE=2 SV=2 - [GCDH_MOUSE]	-0.14	-0.27	-0.17	-0.12	0.01
O35295	Transcriptional activator protein Pur-beta OS=Mus musculus GN=Purb PE=1 SV=3 - [PURB_MOUSE]	0.12	0.25	0.13	0.25	0.01
P62746	Rho-related GTP-binding protein RhoB OS=Mus musculus GN=Rhob PE=1 SV=1 - [RHOB_MOUSE]	-0.31	-0.67	-0.30	-0.50	0.01
Q07139	Protein ECT2 OS=Mus musculus GN=Ect2 PE=1 SV=2 - [ECT2_MOUSE]	0.61	0.46	0.38	0.22	0.01
A6H630	UPF0364 protein C6orf211 homolog OS=Mus musculus PE=2 SV=1 - [CF211_MOUSE]	0.14	0.14	0.12	0.26	0.01
Q8K352	SAM and SH3 domain-containing protein 3 OS=Mus musculus GN=Sash3 PE=2 SV=2 - [SASH3_MOUSE]	0.43	0.72	0.30	0.40	0.01
Q80VC9	Calmodulin-regulated spectrin-associated protein 3 OS=Mus musculus GN=Camsap3 PE=1 SV=1 - [CAMP3_MOUSE]	0.19	0.15	0.11	0.07	0.01
Q9ESN6	Tripartite motif-containing protein 2 OS=Mus musculus GN=Trim2 PE=1 SV=1 [TRIM2_MOUSE]	-0.15	-0.11	-0.27	-0.19	0.01
Q80V85	Tripartite motif-containing protein 62 OS=Mus musculus GN=Trim62 PE=2 SV=1 - [TRI62_MOUSE]	-1.27	-0.61	-1.69	-1.03	0.01
B1AYG7	Integrin beta-6 (Fragment) OS=Mus musculus GN=Itgb6 PE=2 SV=1 - [B1AYG7_MOUSE]	0.21	0.39	0.17	0.35	0.01
P10630	Eukaryotic initiation factor 4A-II OS=Mus musculus GN=Eif4a2 PE=2 SV=2 - [IF4A2_MOUSE]	0.24	0.35	0.14	0.18	0.01
D3YX85	Arf-GAP with SH3 domain, ANK repeat and PH domain-containing protein 2 OS=Mus musculus GN=Asap2 PE=2 SV=2 - [D3YX85_MOUSE]	-0.56	-0.34	-0.22	-0.54	0.01

Q501J2	Protein FAM173A OS=Mus musculus GN=Fam173a PE=2 SV=1 - [F173A_MOUSE]	0.34	0.30	0.18	0.14	0.01
A2AUG1	F-box/WD repeat-containing protein 2 OS=Mus musculus GN=Fbxw2 PE=2 SV=1 - [A2AUG1_MOUSE]	-0.32	-0.31	-0.64	-0.64	0.01
Q80WQ8-3	Isoform 3 of Mis18-binding protein 1 OS=Mus musculus GN=Mis18bp1 - [M18BP_MOUSE]	0.69	0.36	0.65	0.31	0.01
Q99LD9	Translation initiation factor eIF-2B subunit beta OS=Mus musculus GN=Eif2b2 PE=2 SV=1 - [EI2BB_MOUSE]	0.06	0.14	0.18	0.16	0.01
Q8VDP4	DBIRD complex subunit KIAA1967 homolog OS=Mus musculus PE=1 SV=2 - [K1967_MOUSE]	0.12	0.11	0.04	0.10	0.01
Q8BTG6	UPF0454 protein C12orf49 homolog OS=Mus musculus PE=2 SV=1 - [CL049_MOUSE]	-0.15	-0.28	-0.29	-0.43	0.01
P68040	Guanine nucleotide-binding protein subunit beta-2-like 1 OS=Mus musculus GN=Gnb2l1 PE=1 SV=3 - [GBLP_MOUSE]	-0.15	-0.05	-0.13	-0.13	0.01
Q76LS9-2	Isoform 2 of Protein FAM63A OS=Mus musculus GN=Fam63a - [FA63A_MOUSE]	0.97	0.98	0.48	0.49	0.01
Q64514-2	Isoform Short of Tripeptidyl-peptidase 2 OS=Mus musculus GN=Tpp2 - [TPP2_MOUSE]	0.31	0.17	0.46	0.27	0.01
P52800	Ephrin-B2 OS=Mus musculus GN=Efnb2 PE=1 SV=1 - [EFNB2_MOUSE]	-0.37	-0.19	-0.39	-0.18	0.01
Q8BKA3	Serine/arginine repetitive matrix protein 4 OS=Mus musculus GN=Srrm4 PE=1 SV=1 - [SRRM4_MOUSE]	0.67	1.16	0.43	0.92	0.01
P23492	Purine nucleoside phosphorylase OS=Mus musculus GN=Pnp PE=1 SV=2 - [PNPH_MOUSE]	-0.45	-0.51	-0.16	-0.44	0.01
D3YWT0	Signal peptidase complex catalytic subunit SEC11A OS=Mus musculus GN=Sec11a PE=2 SV=1 - [D3YWT0_MOUSE]	0.36	0.20	0.30	0.14	0.01
O35988	Syndecan-4 OS=Mus musculus GN=Sdc4 PE=1 SV=1 - [SDC4_MOUSE]	-0.38	-0.18	-0.54	-0.37	0.01

H3BJ37	Catechol O-methyltransferase domain-containing protein 1 OS=Mus musculus GN=Comtd1 PE=2 SV=1 - [H3BJ37_MOUSE]	0.07	0.13	0.05	0.10	0.01
Q8C407	Protein YIPF4 OS=Mus musculus GN=Yipf4 PE=2 SV=1 - [YIPF4_MOUSE]	0.51	0.24	0.63	0.36	0.01
A2AMS2	Galactose-1-phosphate uridylyltransferase OS=Mus musculus GN=Galt PE=3 SV=1 - [A2AMS2_MOUSE]	-0.64	-0.41	-0.57	-0.23	0.01
P63321	Ras-related protein Ral-A OS=Mus musculus GN=Rala PE=1 SV=1 - [RALA_MOUSE]	0.10	0.26	0.13	0.21	0.01
A7DTG3	SERTA domain-containing protein 4 OS=Mus musculus GN=Sertad4 PE=2 SV=1 - [SRTD4_MOUSE]	-1.02	-0.64	-0.74	-0.36	0.01
A1L341	Dual-specificity tyrosine phosphorylation-regulated kinase 1A OS=Mus musculus GN=Dyrk1a PE=2 SV=1 - [A1L341_MOUSE]	0.36	0.16	0.34	0.19	0.01
Q9CPW4	Actin-related protein 2/3 complex subunit 5 OS=Mus musculus GN=Arpc5 PE=2 SV=3 [ARPC5_MOUSE]	0.15	0.16	0.13	0.05	0.01
Q9WU79	Proline dehydrogenase 1, mitochondrial OS=Mus musculus GN=Prodh PE=2 SV=2 [PROD_MOUSE]	-0.57	-0.30	-0.37	-0.23	0.01
Q99KK1	Receptor expression-enhancing protein 3 OS=Mus musculus GN=Reep3 PE=1 SV=1 - [REEP3_MOUSE]	-0.39	-0.34	-0.77	-0.72	0.01
P48025	Tyrosine-protein kinase SYK OS=Mus musculus GN=Syk PE=1 SV=2 - [KSYK_MOUSE]	0.20	0.22	0.13	0.09	0.01
Q9DBD5	Proline-, glutamic acid- and leucine-rich protein 1 OS=Mus musculus GN=Pelp1 PE=1 SV=2 [PELP1_MOUSE]	0.08	0.22	0.16	0.23	0.01
Q78ZM0	Sorting nexin 3 OS=Mus musculus GN=Snx3 PE=2 SV=1 - [Q78ZM0_MOUSE]	0.18	0.39	0.20	0.37	0.01

Q9DBL1	Short/branched chain specific acyl-CoA dehydrogenase, mitochondrial OS=Mus musculus GN=Acadsb PE=1 SV=1 - [ACDSB_MOUSE]	-0.21	-0.49	-0.32	-0.56	0.01
A2A8L5	Receptor-type tyrosine-protein phosphatase F OS=Mus musculus GN=Ptprf PE=1 SV=1 - [PTPRF_MOUSE]	-0.47	-0.63	-0.25	-0.33	0.01
B1ARH1	Diphthamide biosynthesis protein 1 (Fragment) OS=Mus musculus GN=Dph1 PE=2 SV=1 - [B1ARH1_MOUSE]	0.30	0.65	0.44	0.79	0.01
D3YZ62	Unconventional myosin-Va OS=Mus musculus GN=Myo5a PE=2 SV=1 - [D3YZ62_MOUSE]	-0.15	-0.18	-0.37	-0.26	0.01
Q8BW70	Ubiquitin carboxyl-terminal hydrolase 38 OS=Mus musculus GN=Usp38 PE=2 SV=2 - [UBP38_MOUSE]	-0.21	-0.44	-0.24	-0.48	0.02
Q91WA3	Histone deacetylase 11 OS=Mus musculus GN=Hdac11 PE=2 SV=1 - [HDA11_MOUSE]	0.24	0.50	0.26	0.53	0.02
Q5SUA5	Unconventional myosin-Ig OS=Mus musculus GN=Myo1g PE=2 SV=1 - [MYO1G_MOUSE]	0.25	0.32	0.27	0.10	0.02
Q9WUK4	Replication factor C subunit 2 OS=Mus musculus GN=Rfc2 PE=2 SV=1 - [RFC2_MOUSE]	0.16	0.42	0.24	0.38	0.02
Q9Z2G9	Oxidoreductase HTATIP2 OS=Mus musculus GN=Htatip2 PE=1 SV=3 - [HTAI2_MOUSE]	-0.18	-0.33	-0.13	-0.28	0.02
P01837	Ig kappa chain C region OS=Mus musculus PE=1 SV=1 - [IGKC_MOUSE]	0.17	0.36	0.17	0.34	0.02
Q99LB6-2	Isoform 2 of Methionine adenosyltransferase 2 subunit beta OS=Mus musculus GN=Mat2b - [MAT2B_MOUSE]	-0.07	-0.20	-0.23	-0.20	0.02
Q921J2	GTP-binding protein Rheb OS=Mus musculus GN=Rheb PE=1 SV=1 - [RHEB_MOUSE]	0.32	0.53	0.20	0.33	0.02
Q99MN9	Propionyl-CoA carboxylase beta chain, mitochondrial OS=Mus musculus GN=Pccb PE=1 SV=2 - [PCCB_MOUSE]	-0.24	-0.10	-0.31	-0.23	0.02

Q9WVJ2	26S proteasome non-ATPase regulatory subunit 13 OS=Mus musculus GN=Psmd13 PE=1 SV=1 - [PSD13_MOUSE]	0.22	0.19	0.33	0.12	0.02
O88935-1	Isoform Ib of Synapsin-1 OS=Mus musculus GN=Syn1 - [SYN1_MOUSE]	-0.68	-0.34	-0.43	-0.28	0.02
Q8CD92-2	Isoform 2 of Tetratricopeptide repeat protein 27 OS=Mus musculus GN=Ttc27 - [TTC27_MOUSE]	0.43	0.39	0.86	0.83	0.02
G3X9K8	TP53RK-binding protein OS=Mus musculus GN=Tprkb PE=3 SV=1 - [G3X9K8_MOUSE]	-0.63	-1.27	-0.58	-1.21	0.02
Q8VCA8	Secernin-2 OS=Mus musculus GN=Scrn2 PE=2 SV=1 - [SCRN2_MOUSE]	-0.53	-0.49	-0.34	-0.19	0.02
Q9QYR9	Acyl-coenzyme A thioesterase 2, mitochondrial OS=Mus musculus GN=Acot2 PE=1 SV=2 - [ACOT2_MOUSE]	-0.32	-0.64	-0.26	-0.45	0.02
D3YY07	Palmitoyltransferase ZDHHC13 (Fragment) OS=Mus musculus GN=Zdhhc13 PE=2 SV=1 - [D3YY07_MOUSE]	0.40	0.26	0.23	0.15	0.02
Q7TPD0	Integrator complex subunit 3 OS=Mus musculus GN=Ints3 PE=1 SV=2 - [INT3_MOUSE]	0.69	0.45	0.37	0.27	0.02
Q9ER73	Elongator complex protein 4 OS=Mus musculus GN=Elp4 PE=2 SV=2 - [ELP4_MOUSE]	0.33	0.30	0.33	0.11	0.02
P97864	Caspase-7 OS=Mus musculus GN=Casp7 PE=1 SV=2 - [CASP7_MOUSE]	0.09	0.23	0.13	0.14	0.02
O54825	Bystin OS=Mus musculus GN=Bysl PE=1 SV=3 - [BYST_MOUSE]	0.25	0.19	0.23	0.08	0.02
A2APY7	NADH dehydrogenase [ubiquinone] 1 alpha subcomplex assembly factor 5 OS=Mus musculus GN=Ndufaf5 PE=2 SV=1 - [NDUF5_MOUSE]	-0.48	-0.28	-0.36	-0.17	0.02
D3Z4E3	DEAH (Asp-Glu-Ala-His) box polypeptide 32, isoform CRA_b OS=Mus musculus GN=Dhx32 PE=4 SV=1 - [D3Z4E3_MOUSE]	0.21	0.28	0.54	0.38	0.02

Q8C052	Microtubule-associated protein 1S OS=Mus musculus GN=Map1s PE=1 SV=2 - [MAP1S_MOUSE]	0.08	0.19	0.15	0.23	0.02
Q9JKK1	Syntaxin-6 OS=Mus musculus GN=Stx6 PE=1 SV=1 - [STX6_MOUSE]	0.06	0.10	0.12	0.17	0.02
P46638	Ras-related protein Rab-11B OS=Mus musculus GN=Rab11b PE=1 SV=3 - [RB11B_MOUSE]	-0.11	-0.20	-0.08	-0.11	0.02
Q64511	DNA topoisomerase 2-beta OS=Mus musculus GN=Top2b PE=1 SV=2 - [TOP2B_MOUSE]	0.22	0.37	0.14	0.22	0.02
P14824	Annexin A6 OS=Mus musculus GN=Anxa6 PE=1 SV=3 - [ANXA6_MOUSE]	-0.16	-0.09	-0.19	-0.09	0.02
F6YTG0	Enoyl-CoA hydratase domain-containing protein 2, mitochondrial (Fragment) OS=Mus musculus GN=Echdc2 PE=4 SV=1 - [F6YTG0_MOUSE]	-0.21	-0.43	-0.33	-0.58	0.02
Q9CPY1	39S ribosomal protein L51, mitochondrial OS=Mus musculus GN=Mrpl51 PE=2 SV=1 - [RM51_MOUSE]	0.30	0.19	0.53	0.42	0.02
O35400	Sulfotransferase family cytosolic 2B member 1 OS=Mus musculus GN=Sult2b1 PE=2 SV=2 - [ST2B1_MOUSE]	0.28	0.49	0.18	0.41	0.02
O08966	Solute carrier family 22 member 1 OS=Mus musculus GN=Slc22a1 PE=2 SV=2 - [S22A1_MOUSE]	-0.65	-0.68	-0.30	-0.34	0.02
Q8VH51-2	Isoform 2 of RNA-binding protein 39 OS=Mus musculus GN=Rbm39 - [RBM39_MOUSE]	0.27	0.50	0.19	0.39	0.02
E9PUN5	Tyrosine-protein kinase transmembrane receptor OS=Mus musculus GN=Ror2 PE=2 SV=1 - [E9PUN5_MOUSE]	-0.56	-0.29	-0.42	-0.22	0.02
Q9DCS2	UPF0585 protein C16orf13 homolog OS=Mus musculus PE=1 SV=1 - [CP013_MOUSE]	-0.21	-0.16	-0.29	-0.11	0.02
P60766	Cell division control protein 42 homolog OS=Mus musculus GN=Cdc42 PE=1 SV=2 - [CDC42_MOUSE]	0.09	0.16	0.18	0.27	0.02

O35704	Serine palmitoyltransferase 1 OS=Mus musculus GN=Sptlc1 PE=2 SV=2 - [SPTC1_MOUSE]	0.40	0.41	0.24	0.16	0.02
D3Z3F4	Metaxin-1 (Fragment) OS=Mus musculus GN=Mtx1 PE=4 SV=2 - [D3Z3F4_MOUSE]	0.42	0.56	0.18	0.40	0.02
Q8K3I6	Interleukin-27 subunit alpha OS=Mus musculus GN=Il27 PE=1 SV=1 - [IL27A_MOUSE]	-0.56	-1.03	-1.18	-1.65	0.02
Q68EF0	Rab-3A-interacting protein OS=Mus musculus GN=Rab3ip PE=1 SV=1 - [RAB3I_MOUSE]	0.37	0.66	0.24	0.50	0.02
D3Z108	Transmembrane and ubiquitin-like domain-containing protein 1 (Fragment) OS=Mus musculus GN=Tmub1 PE=2 SV=1 - [D3Z108_MOUSE]	0.18	0.11	0.31	0.24	0.02
Q9D3P8	Plasminogen receptor (KT) OS=Mus musculus GN=Plgrkt PE=1 SV=1 - [PLRKT_MOUSE]	-0.16	-0.05	-0.14	-0.14	0.02
F7BJB9	Protein Morc3 OS=Mus musculus GN=Morc3 PE=4 SV=1 - [F7BJB9_MOUSE]	-0.15	-0.32	-0.17	-0.34	0.02
F6S522	Claspin (Fragment) OS=Mus musculus GN=Clspn PE=4 SV=1 [F6S522_MOUSE]	-0.37	-0.34	-0.76	-0.73	0.02
Q9CQW5	Galectin-2 OS=Mus musculus GN=Lgals2 PE=2 SV=1 - [LEG2_MOUSE]	-0.60	-0.82	-0.29	-0.48	0.02
Q3TNA1	Xylulose kinase OS=Mus musculus GN=Xylb PE=2 SV=1 - [XYLB_MOUSE]	-0.57	-0.59	-0.27	-0.29	0.02
Q9CXT8	Mitochondrial-processing peptidase subunit beta OS=Mus musculus GN=Pmpcb PE=2 SV=1 - [MPPB_MOUSE]	-0.14	-0.23	-0.13	-0.30	0.02
O35245-2	Isoform 2 of Polycystin-2 OS=Mus musculus GN=Pkd2 - [PKD2_MOUSE]	-0.30	-0.37	-0.46	-0.73	0.02
Q922V4	Pleiotropic regulator 1 OS=Mus musculus GN=Plrg1 PE=2 SV=1 - [PLRG1_MOUSE]	-0.12	-0.11	-0.04	-0.10	0.02
Q8BHK9	DNA excision repair protein ERCC-6-like OS=Mus musculus GN=Ercc6l PE=1 SV=1 - [ERC6L_MOUSE]	0.35	0.67	0.72	1.04	0.02
P10810	Monocyte differentiation antigen CD14 OS=Mus musculus GN=Cd14 PE=1 SV=1 - [CD14_MOUSE]	-0.58	-1.23	-0.63	-1.28	0.02

E9Q828	Protein Atp2b4 OS=Mus musculus GN=Atp2b4 PE=2 SV=1 - [E9Q828_MOUSE]	-0.10	-0.19	-0.07	-0.15	0.02
P27808	Alpha-1,3-mannosyl-glycoprotein 2-beta-N-acetylglucosaminyltransferase OS=Mus musculus GN=Mgat1 PE=2 SV=1 - [MGAT1_MOUSE]	0.41	0.22	0.46	0.20	0.02
O55042-2	Isoform 2 of Alpha-synuclein OS=Mus musculus GN=Snca - [SYUA_MOUSE]	-0.33	-0.19	-0.26	-0.12	0.02
E9Q7Z8	Protein Ythdc1 OS=Mus musculus GN=Ythdc1 PE=2 SV=1 - [E9Q7Z8_MOUSE]	-0.08	-0.16	-0.07	-0.16	0.02
Q8BGD7	Neuronal PAS domain-containing protein 4 OS=Mus musculus GN=Npas4 PE=1 SV=1 - [NPAS4_MOUSE]	0.09	0.09	0.18	0.19	0.02
Q8BGY3	Leucine zipper protein 2 OS=Mus musculus GN=Luzp2 PE=2 SV=1 - [LUZP2_MOUSE]	0.08	0.16	0.07	0.16	0.02
Q812A5-2	Isoform 2 of Proline-rich protein 5 OS=Mus musculus GN=Prr5 - [PRR5_MOUSE]	0.61	1.28	0.62	1.28	0.02
Q8BUE4	Apoptosis-inducing factor 2 OS=Mus musculus GN=Aifm2 PE=2 SV=1 - [AIFM2_MOUSE]	0.31	0.31	0.15	0.15	0.02
O08796	Eukaryotic elongation factor 2 kinase OS=Mus musculus GN=Eef2k PE=1 SV=1 [EF2K_MOUSE]	0.37	0.52	0.18	0.32	0.02
Q3V3R4	Integrin alpha-1 OS=Mus musculus GN=Itga1 PE=2 SV=2 - [ITA1_MOUSE]	-0.21	-0.13	-0.15	-0.07	0.02
Q6PFH3	DDB1- and CUL4-associated factor 15 OS=Mus musculus GN=Dcaf15 PE=2 SV=1 - [DCA15_MOUSE]	-0.35	-0.77	-0.45	-0.87	0.02
B1AYL1	Protein Scn7a OS=Mus musculus GN=Scn7a PE=2 SV=1 - [B1AYL1_MOUSE]	-0.26	-0.17	-0.14	-0.10	0.02
Q9EP69	Phosphatidylinositide phosphatase SAC1 OS=Mus musculus GN=Sacm1l PE=2 SV=1 - [SAC1_MOUSE]	0.17	0.14	0.06	0.18	0.02
Q8R3F5	Malonyl-CoA-acyl carrier protein transacylase, mitochondrial OS=Mus musculus GN=Mcat PE=2 SV=3 - [FABD_MOUSE]	-0.77	-0.61	-0.44	-0.28	0.02

Q4LDD4-3	Isoform 3 of Arf-GAP with Rho-GAP domain, ANK repeat and PH domain-containing protein 1 OS=Mus musculus GN=Arap1 - [ARAP1_MOUSE]	0.49	0.60	0.22	0.33	0.02
Q9Z1T1	AP-3 complex subunit beta-1 OS=Mus musculus GN=Ap3b1 PE=1 SV=2 - [AP3B1_MOUSE]	0.21	0.12	0.26	0.11	0.02
Q05117	Tartrate-resistant acid phosphatase type 5 OS=Mus musculus GN=Acp5 PE=2 SV=2 - [PPA5_MOUSE]	-0.23	-0.21	-0.07	-0.23	0.02
Q9EQQ2	Protein YIPF5 OS=Mus musculus GN=Yipf5 PE=2 SV=1 - [YIPF5_MOUSE]	0.44	0.97	0.55	1.07	0.02
P03958	Adenosine deaminase OS=Mus musculus GN=Ada PE=1 SV=3 - [ADA_MOUSE]	0.19	0.09	0.08	0.14	0.02
P59999	Actin-related protein 2/3 complex subunit 4 OS=Mus musculus GN=Arpc4 PE=1 SV=3 [ARPC4_MOUSE]	-0.17	-0.08	-0.15	-0.07	0.02
P97376	Protein FRG1 OS=Mus musculus GN=Frg1 PE=1 SV=2 - [FRG1_MOUSE]	0.37	0.15	0.29	0.17	0.02
Q61493	DNA polymerase zeta catalytic subunit OS=Mus musculus GN=Rev3l PE=1 SV=3 [DPOLZ_MOUSE]	0.59	0.44	0.28	0.25	0.02
Q5NCU4	SPARC OS=Mus musculus GN=Sparc PE=2 SV=1 - [Q5NCU4_MOUSE]	-0.17	-0.35	-0.16	-0.34	0.02
A2AQV2	Carbohydrate sulfotransferase 14 OS=Mus musculus GN=Chst14 PE=2 SV=1 - [A2AQV2_MOUSE]	-0.31	-0.16	-0.27	-0.12	0.02
Q711T7	Glutamine-dependent NAD(+) synthetase OS=Mus musculus GN=Nadsyn1 PE=2 SV=1 - [NADE_MOUSE]	-0.28	-0.41	-0.16	-0.21	0.02
Q9CS72	Filamin-A-interacting protein 1 OS=Mus musculus GN=Filip1 PE=1 SV=2 - [FLIP1_MOUSE]	-0.56	-0.71	-0.25	-0.41	0.02
A2ATL1	MCG17280 OS=Mus musculus GN=Fam171a1 PE=4 SV=1 - [A2ATL1_MOUSE]	-0.14	-0.21	-0.31	-0.39	0.02
P46062	Signal-induced proliferation-associated protein 1 OS=Mus musculus GN=Sipa1 PE=2 SV=2 - [SIPA1_MOUSE]	-0.44	-0.98	-0.69	-1.23	0.02

Q5SW46	Protein Lpo OS=Mus musculus GN=Lpo PE=2 SV=1 - [Q5SW46_MOUSE]	-0.63	-0.74	-0.33	-0.33	0.02
P49312	Heterogeneous nuclear ribonucleoprotein A1 OS=Mus musculus GN=Hnrnpa1 PE=1 SV=2 - [ROA1_MOUSE]	-0.17	-0.08	-0.24	-0.15	0.02
Q9D711	Pirin OS=Mus musculus GN=Pir PE=2 SV=1 - [PIR_MOUSE]	-0.77	-0.64	-0.42	-0.29	0.02
B1AT92	Growth factor receptor-bound protein 2 OS=Mus musculus GN=Grb2 PE=2 SV=1 - [B1AT92_MOUSE]	-0.25	-0.09	-0.31	-0.24	0.02
Q9CWK8	Sorting nexin-2 OS=Mus musculus GN=Snx2 PE=1 SV=2 - [SNX2_MOUSE]	0.17	0.07	0.20	0.12	0.02
E9PYS3	Protein Adam3 OS=Mus musculus GN=Adam3 PE=2 SV=1 - [E9PYS3_MOUSE]	0.47	0.89	0.34	0.75	0.02
P01747	Ig heavy chain V region 36-65 OS=Mus musculus PE=1 SV=1 - [HVM03_MOUSE]	-0.16	-0.31	-0.32	-0.48	0.02
Q99JW2	Aminoacylase-1 OS=Mus musculus GN=Acy1 PE=1 SV=1 - [ACY1_MOUSE]	-0.25	-0.60	-0.27	-0.45	0.02
P49586	Choline-phosphate cytidylyltransferase A OS=Mus musculus GN=Pcyt1a PE=1 SV=1 - [PCY1A_MOUSE]	0.29	0.24	0.27	0.09	0.02
Q6PAR5-2	Isoform 2 of GTPase-activating protein and VPS9 domain-containing protein 1 OS=Mus musculus GN=Gapvd1 - [GAPD1_MOUSE]	0.35	0.13	0.27	0.19	0.02
B7ZNG0-2	Isoform 2 of Kinesin-like protein KIF7 OS=Mus musculus GN=Kif7 - [KIF7_MOUSE]	0.33	0.16	0.50	0.33	0.02
Q9DAW6	U4/U6 small nuclear ribonucleoprotein Prp4 OS=Mus musculus GN=Prpf4 PE=2 SV=1 - [PRP4_MOUSE]	-0.03	-0.09	-0.08	-0.09	0.02
Q9D7S9	Charged multivesicular body protein 5 OS=Mus musculus GN=Chmp5 PE=2 SV=1 - [CHMP5_MOUSE]	0.40	0.47	0.15	0.49	0.02
D3YWP3	MCG1034428 OS=Mus musculus GN=Rpl23a-ps3 PE=3 SV=1 - [D3YWP3_MOUSE]	0.10	0.24	0.13	0.23	0.02

P97427	Dihydropyrimidinase-related protein 1 OS=Mus musculus GN=Crmp1 PE=1 SV=1 - [DPYL1_MOUSE]	-0.23	-0.30	-0.48	-0.59	0.02
P21126	Ubiquitin-like protein 4A OS=Mus musculus GN=Ubl4a PE=2 SV=1 [UBL4A_MOUSE]	-0.11	-0.16	-0.05	-0.14	0.02
Q8CI78	Required for meiotic nuclear division protein 1 homolog OS=Mus musculus GN=Rmnd1 PE=2 SV=1 - [RMND1_MOUSE]	-0.37	-0.17	-0.40	-0.20	0.02
Q6P2L6	Histone-lysine N-methyltransferase NSD3 OS=Mus musculus GN=Whsc1l1 PE=1 SV=2 - [NSD3_MOUSE]	-0.33	-0.43	-0.13	-0.37	0.02
Q6PAV2-2	Isoform 2 of Probable E3 ubiquitin-protein ligase HERC4 OS=Mus musculus GN=Herc4 - [HERC4_MOUSE]	0.14	0.09	0.24	0.24	0.02
Q8R502	Leucine-rich repeat-containing protein 8C OS=Mus musculus GN=Lrrc8c PE=1 SV=1 - [LRC8C_MOUSE]	-0.33	-0.51	-0.17	-0.35	0.02
Q3U5Q7	UMP-CMP kinase 2, mitochondrial OS=Mus musculus GN=Cmpk2 PE=2 SV=2 - [CMPK2_MOUSE]	-0.30	-0.32	-0.16	-0.14	0.02
Q6PDK8	E3 ubiquitin-protein ligase DTX4 OS=Mus musculus GN=Dtx4 PE=1 SV=2 - [DTX4_MOUSE]	-0.89	-1.20	-0.40	-0.71	0.02
E9PWK1	Epoxide hydrolase 1 OS=Mus musculus GN=Ephx1 PE=2 SV=1 - [E9PWK1_MOUSE]	-0.08	-0.03	-0.11	-0.10	0.02
P58044	Isopentenyl-diphosphate Delta-isomerase 1 OS=Mus musculus GN=Idi1 PE=2 SV=1 - [IDI1_MOUSE]	0.18	0.45	0.22	0.41	0.02
D3YZ05	Translation initiation factor eIF-2B subunit alpha (Fragment) OS=Mus musculus GN=Eif2b1 PE=2 SV=1 - [D3YZ05_MOUSE]	0.37	0.51	0.15	0.36	0.02
P21278	Guanine nucleotide-binding protein subunit alpha-11 OS=Mus musculus GN=Gna11 PE=1 SV=1 - [GNA11_MOUSE]	-0.11	-0.29	-0.15	-0.24	0.02

E9QAS9	Fragile X mental retardation protein 1 homolog OS=Mus musculus GN=Fmr1 PE=2 SV=1 - [E9QAS9_MOUSE]	0.15	0.34	0.22	0.41	0.02
Q91VR7	Microtubule-associated proteins 1A/1B light chain 3A OS=Mus musculus GN=Map1lc3a PE=1 SV=1 - [MLP3A_MOUSE]	-0.33	-0.26	-0.26	-0.10	0.02
E9Q743	Protein Wdr66 OS=Mus musculus GN=Wdr66 PE=2 SV=1 - [E9Q743_MOUSE]	-0.26	-0.50	-0.19	-0.43	0.02
G5E8T9	Hydroxyacyl glutathione hydrolase OS=Mus musculus GN=Hagh PE=3 SV=1 - [G5E8T9_MOUSE]	-0.20	-0.31	-0.17	-0.43	0.02
Q3UN00	P2X purinoceptor OS=Mus musculus GN=P2rx7 PE=2 SV=1 [Q3UN00_MOUSE]	-0.90	-0.40	-1.11	-0.60	0.02
Q9D2G9	HHIP-like protein 2 OS=Mus musculus GN=Hhipl2 PE=2 SV=2 - [HIPL2_MOUSE]	0.98	0.43	1.23	0.68	0.02
Q7TMI3	E3 ubiquitin-protein ligase UHRF2 OS=Mus musculus GN=Uhrf2 PE=1 SV=1 - [UHRF2_MOUSE]	-0.11	-0.17	-0.25	-0.31	0.02
F6TZU3	Gigaxonin (Fragment) OS=Mus musculus GN=Gan PE=4 SV=1 - [F6TZU3_MOUSE]	-0.11	-0.33	-0.36	-0.35	0.02
Q8BH15-2	Isoform 2 of CCR4-NOT transcription complex subunit 10 OS=Mus musculus GN=Cnot10 - [CNO10_MOUSE]	-0.48	-0.48	-0.23	-0.22	0.02
Q9CRB2	H/ACA ribonucleoprotein complex subunit 2 OS=Mus musculus GN=Nhp2 PE=2 SV=1 - [NHP2_MOUSE]	0.15	0.41	0.34	0.49	0.02
Q9ES97-3	Isoform 3 of Reticulon-3 OS=Mus musculus GN=Rtn3 - [RTN3_MOUSE]	0.45	0.25	0.29	0.16	0.02
G3X9U9	Fission 1 (Mitochondrial outer membrane) homolog (Yeast), isoform CRA_c OS=Mus musculus GN=Fis1 PE=4 SV=1 - [G3X9U9_MOUSE]	-0.22	-0.08	-0.14	-0.12	0.02
P23475	X-ray repair cross-complementing protein 6 OS=Mus musculus GN=Xrcc6 PE=1 SV=5 [XRCC6_MOUSE]	0.14	0.05	0.16	0.18	0.02
Q61160	Protein FADD OS=Mus musculus GN=Fadd PE=1 SV=1 - [FADD_MOUSE]	0.17	0.21	0.20	0.06	0.02

Q8VBT0	Thioredoxin-related transmembrane protein 1 OS=Mus musculus GN=Tmx1 PE=1 SV=1 - [TMX1_MOUSE]	0.16	0.33	0.14	0.19	0.02
F7BWT7-2	Isoform 2 of Tetraspanin-15 OS=Mus musculus GN=Tspan15 - [TSN15_MOUSE]	0.17	0.38	0.27	0.48	0.02
P50446	Keratin, type II cytoskeletal 6A OS=Mus musculus GN=Krt6a PE=2 SV=3 - [K2C6A_MOUSE]	0.82	0.38	0.84	0.40	0.02
P33434-2	Isoform 2 of 72 kDa type IV collagenase OS=Mus musculus GN=Mmp2 - [MMP2_MOUSE]	-0.62	-0.70	-0.22	-0.47	0.02
Q9DB15	39S ribosomal protein L12, mitochondrial OS=Mus musculus GN=Mrpl12 PE=1 SV=2 - [RM12_MOUSE]	-0.19	-0.09	-0.28	-0.17	0.02
Q571F5	SPRY domain-containing SOCS box protein 3 OS=Mus musculus GN=Spsb3 PE=2 SV=2 - [SPSB3_MOUSE]	-0.43	-0.20	-0.42	-0.20	0.02
Q8BXA1	Golgi integral membrane protein 4 OS=Mus musculus GN=Golim4 PE=1 SV=1 - [GOLI4_MOUSE]	-0.12	-0.26	-0.12	-0.15	0.02
Q99N28	Cell adhesion molecule 3 OS=Mus musculus GN=Cadm3 PE=1 SV=1 - [CADM3_MOUSE]	-0.45	-0.20	-0.52	-0.27	0.02
Q1ERP8-4	Isoform 4 of CMRF35-like molecule 9 OS=Mus musculus GN=Cd300lg - [CLM9_MOUSE]	-0.39	-0.46	-0.87	-0.95	0.02
Q9QZM8-2	Isoform 2 of F-box only protein 17 OS=Mus musculus GN=Fbxo17 - [FBX17_MOUSE]	-0.54	-0.24	-0.70	-0.40	0.02
P56393	Cytochrome c oxidase subunit 7B, mitochondrial OS=Mus musculus GN=Cox7b PE=2 SV=1 - [COX7B_MOUSE]	-0.34	-0.36	-0.74	-0.77	0.02
Q8BGB7	Enolase-phosphatase E1 OS=Mus musculus GN=Enoph1 PE=2 SV=1 - [ENOPH_MOUSE]	-0.12	-0.27	-0.11	-0.22	0.02
A7XUY5-2	Isoform 2 of Selection and upkeep of intraepithelial T-cells protein 5 OS=Mus musculus GN=Skint5 - [SKIT5_MOUSE]	-0.51	-0.25	-0.48	-0.21	0.02
Q61703	Inter-alpha-trypsin inhibitor heavy chain H2 OS=Mus musculus GN=Itih2 PE=1 SV=1 - [ITIH2_MOUSE]	-0.56	-0.36	-0.19	-0.32	0.02

P29595	NEDD8 OS=Mus musculus GN=Nedd8 PE=1 SV=2 - [NEDD8_MOUSE]	-0.15	-0.14	-0.32	-0.30	0.02
P62830	60S ribosomal protein L23 OS=Mus musculus GN=Rpl23 PE=1 SV=1 [RL23_MOUSE]	0.45	0.20	0.34	0.19	0.02
Q8BHS3	Pre-mRNA-splicing factor RBM22 OS=Mus musculus GN=Rbm22 PE=2 SV=1 - [RBM22_MOUSE]	-0.22	-0.41	-0.15	-0.35	0.02
P46414	Cyclin-dependent kinase inhibitor 1B OS=Mus musculus GN=Cdkn1b PE=1 SV=2 - [CDN1B_MOUSE]	-0.49	-0.91	-0.33	-0.75	0.02
P16872	Interleukin-7 receptor subunit alpha OS=Mus musculus GN=Il7r PE=2 SV=2 - [IL7RA_MOUSE]	-0.69	-1.45	-1.40	-2.17	0.02
Q8C0I1	Alkyldihydroxyacetonephosphate synthase, peroxisomal OS=Mus musculus GN=Agps PE=1 SV=1 - [ADAS_MOUSE]	-0.27	-0.28	-0.09	-0.32	0.02
E9Q504	Histone demethylase UTY OS=Mus musculus GN=Uty PE=2 SV=1 - [E9Q504_MOUSE]	-0.20	-0.09	-0.30	-0.19	0.02
Q8WTY4	Anamorsin OS=Mus musculus GN=Ciapin1 PE=1 SV=1 - [CPIN1_MOUSE]	-0.13	-0.18	-0.13	-0.29	0.02
P52480	Pyruvate kinase PKM OS=Mus musculus GN=Pkm PE=1 SV=4 - [KPYM_MOUSE]	-0.11	-0.13	-0.08	-0.21	0.02
E9QKV6	Unconventional myosin-IXb OS=Mus musculus GN=Myo9b PE=2 SV=1 - [E9QKV6_MOUSE]	0.37	0.18	0.35	0.15	0.02
P42227-2	Isoform Stat3B of Signal transducer and activator of transcription 3 OS=Mus musculus GN=Stat3 - [STAT3_MOUSE]	0.19	0.10	0.32	0.27	0.02
P52480-2	Isoform M1 of Pyruvate kinase PKM OS=Mus musculus GN=Pkm - [KPYM_MOUSE]	0.15	0.33	0.15	0.32	0.02
Q7TPR4	Alpha-actinin-1 OS=Mus musculus GN=Actn1 PE=1 SV=1 [ACTN1_MOUSE]	0.31	0.25	0.18	0.10	0.02
Q9CWU6-2	Isoform 2 of Ubiquinol-cytochrome c reductase complex chaperone CBP3 homolog OS=Mus musculus GN=Uqcc - [UQCC_MOUSE]	-0.34	-0.24	-0.65	-0.56	0.02

Q9Z0G7	Zinc finger and BTB domain-containing protein 22 OS=Mus musculus GN=Zbtb22 PE=2 SV=1 - [ZBT22_MOUSE]	-1.09	-2.28	-0.98	-2.17	0.02
Q99JP0	Mitogen-activated protein kinase kinase kinase kinase 3 OS=Mus musculus GN=Map4k3 PE=1 SV=4 - [M4K3_MOUSE]	0.40	0.59	0.19	0.37	0.02
P56371	Ras-related protein Rab-4A OS=Mus musculus GN=Rab4a PE=1 SV=2 - [RAB4A_MOUSE]	-0.35	-0.54	-0.17	-0.39	0.02
Q9R1P4	Proteasome subunit alpha type-1 OS=Mus musculus GN=Psma1 PE=1 SV=1 - [PSA1_MOUSE]	-0.19	-0.43	-0.18	-0.30	0.02
Q80UQ2	Ras association domain-containing protein 6 OS=Mus musculus GN=Rassf6 PE=1 SV=1 - [RASF6_MOUSE]	0.47	0.68	0.21	0.60	0.02
P63254	Cysteine-rich protein 1 OS=Mus musculus GN=Crip1 PE=2 SV=2 - [CRIP1_MOUSE]	-0.07	-0.18	-0.10	-0.16	0.02
A2A7R1	Transmembrane protein 54 OS=Mus musculus GN=Tmem54 PE=4 SV=1 - [A2A7R1_MOUSE]	-0.21	-0.40	-0.47	-0.66	0.02
Q3UL36	Arginine and glutamate-rich protein 1 OS=Mus musculus GN=Arglu1 PE=1 SV=2 - [ARGL1_MOUSE]	0.23	0.09	0.20	0.31	0.02
P62743	AP-2 complex subunit sigma OS=Mus musculus GN=Ap2s1 PE=1 SV=1 - [AP2S1_MOUSE]	-0.14	-0.05	-0.16	-0.15	0.02
A2AUY4	Protein Baz2b OS=Mus musculus GN=Baz2b PE=4 SV=1 - [A2AUY4_MOUSE]	0.17	0.39	0.27	0.49	0.02
Q9CQV5	28S ribosomal protein S24, mitochondrial OS=Mus musculus GN=Mrps24 PE=2 SV=1 - [RT24_MOUSE]	0.11	0.19	0.06	0.14	0.02
E9Q993	Aryl hydrocarbon receptor nuclear translocator-like protein 2 OS=Mus musculus GN=Arntl2 PE=2 SV=1 [E9Q993_MOUSE]	-0.62	-1.07	-1.43	-1.88	0.02
Q3U3J1	2-oxoisovalerate dehydrogenase subunit alpha, mitochondrial OS=Mus musculus GN=Bckdha PE=2 SV=1 - [Q3U3J1_MOUSE]	-0.26	-0.51	-0.23	-0.55	0.02

Q8BXZ1	Protein disulfide-isomerase TMX3 OS=Mus musculus GN=Tmx3 PE=1 SV=2 - [TMX3_MOUSE]	0.17	0.29	0.13	0.35	0.02
P0C605-2	Isoform Beta of cGMP-dependent protein kinase 1 OS=Mus musculus GN=Prkg1 - [KGP1_MOUSE]	0.14	0.43	0.43	0.50	0.02
Q7TQ62	Podocan OS=Mus musculus GN=Podn PE=2 SV=1 - [PODN_MOUSE]	0.93	0.92	0.43	0.42	0.02
Q8VDQ8-2	Isoform 2 of NAD-dependent protein deacetylase sirtuin-2 OS=Mus musculus GN=Sirt2 - [SIR2_MOUSE]	0.11	0.35	0.22	0.24	0.02
Q8CHW4	Translation initiation factor eIF-2B subunit epsilon OS=Mus musculus GN=Eif2b5 PE=1 SV=1 - [EI2BE_MOUSE]	-0.47	-0.59	-0.57	-0.17	0.02
Q99KR3	Beta-lactamase-like protein 2 OS=Mus musculus GN=Lactb2 PE=1 SV=1 - [LACB2_MOUSE]	0.34	0.12	0.38	0.23	0.02
D3Z7B5	Protein C330027C09Rik OS=Mus musculus GN=C330027C09Rik PE=2 SV=1 - [D3Z7B5_MOUSE]	-1.12	-1.40	-0.48	-0.76	0.02
Q9D154	Leukocyte elastase inhibitor A OS=Mus musculus GN=Serpinb1a PE=1 SV=1 - [ILEUA_MOUSE]	0.15	0.10	0.06	0.06	0.02
Q8K3K7	1-acyl-sn-glycerol-3-phosphate acyltransferase beta OS=Mus musculus GN=Agpat2 PE=1 SV=1 - [PLCB_MOUSE]	-0.46	-0.57	-0.20	-0.31	0.02
Q58A65-2	Isoform 2 of C-Jun-amino-terminal kinase-interacting protein 4 OS=Mus musculus GN=Spag9 - [JIP4_MOUSE]	0.13	0.08	0.24	0.20	0.02
Q8R1Q9	Ribokinase OS=Mus musculus GN=Rbks PE=1 SV=1 - [RBSK_MOUSE]	-0.60	-0.37	-0.35	-0.21	0.02
E9Q9Q2	Protein R3hdm1 OS=Mus musculus GN=R3hdm1 PE=2 SV=1 - [E9Q9Q2_MOUSE]	-0.52	-0.56	-0.23	-0.27	0.02
Q9DBD0	Inhibitor of carbonic anhydrase OS=Mus musculus GN=Ica PE=1 SV=1 - [ICA_MOUSE]	-0.21	-0.24	-0.07	-0.20	0.02

Q8R1V4	Transmembrane emp24 domain-containing protein 4 OS=Mus musculus GN=Tmed4 PE=2 SV=1 - [TMED4_MOUSE]	0.25	0.38	0.11	0.29	0.02
Q9QUH0	Glutaredoxin-1 OS=Mus musculus GN=Glrx PE=1 SV=3 - [GLRX1_MOUSE]	-0.15	-0.19	-0.34	-0.39	0.02
A2ARI0	Thioredoxin-related transmembrane protein 4 OS=Mus musculus GN=Tmx4 PE=2 SV=1 - [A2ARI0_MOUSE]	0.16	0.33	0.34	0.51	0.02
Q5SUH7	Clathrin interactor 1 OS=Mus musculus GN=Clint1 PE=2 SV=1 [Q5SUH7_MOUSE]	0.21	0.41	0.17	0.24	0.02
F8VPR1	Leiomodin-1 OS=Mus musculus GN=Lmod1 PE=2 SV=1 - [F8VPR1_MOUSE]	0.03	0.10	0.06	0.09	0.02
Q9WTL8-3	Isoform 3 of Aryl hydrocarbon receptor nuclear translocator-like protein 1 OS=Mus musculus GN=Arntl - [BMAL1_MOUSE]	-0.18	-0.33	-0.11	-0.26	0.02
O54865	Guanylate cyclase soluble subunit beta-1 OS=Mus musculus GN=Gucy1b3 PE=2 SV=1 - [GCYB1_MOUSE]	0.22	0.10	0.11	0.10	0.02
G3UYD0	General transcription factor II-I OS=Mus musculus GN=Gtf2i PE=2 SV=1 - [G3UYD0_MOUSE]	-0.20	-0.09	-0.14	-0.08	0.02
H7BWY2	AP-3 complex subunit mu-1 OS=Mus musculus GN=Ap3m1 PE=2 SV=1 - [H7BWY2_MOUSE]	0.26	0.35	0.10	0.26	0.02
Q8CJG0	Protein argonaute-2 OS=Mus musculus GN=Ago2 PE=1 SV=3 - [AGO2_MOUSE]	-0.26	-0.08	-0.25	-0.20	0.02
Q69ZX8	Actin-binding LIM protein 3 OS=Mus musculus GN=Ablim3 PE=1 SV=2 - [ABLM3_MOUSE]	0.51	0.22	0.63	0.33	0.02
Q9WTL7	Acyl-protein thioesterase 2 OS=Mus musculus GN=Lypla2 PE=1 SV=1 - [LYPA2_MOUSE]	0.16	0.12	0.05	0.12	0.02
B1AUC4	Protein Ube2u OS=Mus musculus GN=Ube2u PE=2 SV=1 - [B1AUC4_MOUSE]	0.08	0.12	0.19	0.22	0.02
Q6P542	ATP-binding cassette sub-family F member 1 OS=Mus musculus GN=Abcf1 PE=1 SV=1 [ABCF1_MOUSE]	0.23	0.53	0.22	0.43	0.02

P70677	Caspase-3 OS=Mus musculus GN=Casp3 PE=1 SV=1 - [CASP3_MOUSE]	-0.10	-0.11	-0.24	-0.23	0.02
Q8VHQ9	Acyl-coenzyme A thioesterase 11 OS=Mus musculus GN=Acot11 PE=2 SV=1 - [ACO11_MOUSE]	0.30	0.36	0.16	0.15	0.02
P50580	Proliferation-associated protein 2G4 OS=Mus musculus GN=Pa2g4 PE=1 SV=3 - [PA2G4_MOUSE]	0.11	0.19	0.06	0.15	0.02
Q9QYB2-2	Isoform 2 of Dachshund homolog 1 OS=Mus musculus GN=Dach1 - [DACH1_MOUSE]	-0.22	-0.10	-0.22	-0.10	0.02
P54818	Galactocerebrosidase OS=Mus musculus GN=Galc PE=1 SV=2 - [GALC_MOUSE]	0.11	0.22	0.08	0.18	0.02
F6QA74	DNA-(apurinic or apyrimidinic site) lyase (Fragment) OS=Mus musculus GN=Apex1 PE=2 SV=1 - [F6QA74_MOUSE]	0.24	0.50	0.20	0.45	0.02
Q9D1L9	Ragulator complex protein LAMTOR5 OS=Mus musculus GN=Lamtor5 PE=2 SV=1 - [LTOR5_MOUSE]	-0.19	-0.46	-0.21	-0.41	0.02
E9PYT3	Atlastin-3 OS=Mus musculus GN=Atl3 PE=2 SV=1 - [E9PYT3_MOUSE]	0.21	0.24	0.15	0.07	0.02
Q9WU03-2	Isoform 2 of Kunitz-type protease inhibitor 2 OS=Mus musculus GN=Spint2 - [SPIT2_MOUSE]	0.46	0.27	0.35	0.15	0.02
P35831	Tyrosine-protein phosphatase non-receptor type 12 OS=Mus musculus GN=Ptpn12 PE=1 SV=3 - [PTN12_MOUSE]	0.25	0.36	0.11	0.22	0.02
Q62418-3	Isoform 3 of Drebrin-like protein OS=Mus musculus GN=Dbnl - [DBNL_MOUSE]	-0.19	-0.18	-0.15	-0.05	0.02
Q9CZH8-2	Isoform 2 of Coiled-coil domain-containing protein 77 OS=Mus musculus GN=Ccdc77 - [CCD77_MOUSE]	-0.70	-0.30	-0.83	-0.43	0.02
P01887	Beta-2-microglobulin OS=Mus musculus GN=B2m PE=1 SV=2 - [B2MG_MOUSE]	-0.27	-0.18	-0.14	-0.09	0.02
P28661-5	Isoform 5 of Septin-4 OS=Mus musculus GN=Sept4 - [SEPT4_MOUSE]	-0.50	-0.73	-0.23	-0.45	0.02
Q9JII5-2	Isoform 2 of DAZ-associated protein 1 OS=Mus musculus GN=Dazap1 - [DAZP1_MOUSE]	-0.35	-0.35	-0.10	-0.30	0.02

Q9QXD1	Peroxisomal acyl-coenzyme A oxidase 2 OS=Mus musculus GN=Acox2 PE=1 SV=2 - [ACOX2_MOUSE]	-0.17	-0.15	-0.09	-0.06	0.02
Q91ZM2-6	Isoform 6 of SH2B adapter protein 1 OS=Mus musculus GN=Sh2b1 - [SH2B1_MOUSE]	-0.26	-0.60	-0.47	-0.81	0.02
Q3TC93	HCLS1-binding protein 3 OS=Mus musculus GN=Hs1bp3 PE=1 SV=2 - [H1BP3_MOUSE]	-0.26	-0.26	-0.17	-0.08	0.02
Q9JHJ0	Tropomodulin-3 OS=Mus musculus GN=Tmod3 PE=1 SV=1 - [TMOD3_MOUSE]	-0.27	-0.10	-0.26	-0.15	0.02
Q5UAK0-4	Isoform 4 of Mesoderm induction early response protein 1 OS=Mus musculus GN=Mier1 - [MIER1_MOUSE]	-0.76	-0.33	-1.08	-0.66	0.02
Q921K2	Poly (ADP-ribose) polymerase family, member 1 OS=Mus musculus GN=Parp1 PE=2 SV=1 - [Q921K2_MOUSE]	0.19	0.15	0.06	0.13	0.02
E9QLB8	Uncharacterized aarF domain-containing protein kinase 4 OS=Mus musculus GN=Adck4 PE=4 SV=1 - [E9QLB8_MOUSE]	-0.29	-0.69	-0.39	-0.78	0.02
Q8C0E3	Tripartite motif-containing protein 47 OS=Mus musculus GN=Trim47 PE=2 SV=2 - [TRI47_MOUSE]	0.56	0.28	0.51	0.21	0.02
Q9CZW5	Mitochondrial import receptor subunit TOM70 OS=Mus musculus GN=Tomm70a PE=1 SV=2 - [TOM70_MOUSE]	0.05	0.15	0.11	0.09	0.02
O70591	Prefoldin subunit 2 OS=Mus musculus GN=Pfdn2 PE=2 SV=2 [PFD2_MOUSE]	-0.16	-0.09	-0.13	-0.25	0.02
P54823	Probable ATP-dependent RNA helicase DDX6 OS=Mus musculus GN=Ddx6 PE=2 SV=1 - [DDX6_MOUSE]	0.34	0.11	0.34	0.21	0.02
Q91Z31	Polypyrimidine tract-binding protein 2 OS=Mus musculus GN=Ptbp2 PE=1 SV=2 [PTBP2_MOUSE]	0.41	0.50	0.17	0.26	0.02
Q9CXW2	28S ribosomal protein S22, mitochondrial OS=Mus musculus GN=Mrps22 PE=2 SV=1 - [RT22_MOUSE]	-0.08	-0.07	-0.17	-0.18	0.02

Q8QZY9	Splicing factor 3B subunit 4 OS=Mus musculus GN=Sf3b4 PE=2 SV=1 - [SF3B4_MOUSE]	-0.14	-0.14	-0.07	-0.22	0.02
E9Q6R7	Protein Utrn OS=Mus musculus GN=Utrn PE=2 SV=1 - [E9Q6R7_MOUSE]	-0.04	-0.02	-0.07	-0.05	0.02
Q99MB1	Toll-like receptor 3 OS=Mus musculus GN=Tlr3 PE=1 SV=2 - [TLR3_MOUSE]	0.10	0.09	0.11	0.21	0.02
Q8BHJ6	Serine incorporator 5 OS=Mus musculus GN=Serinc5 PE=2 SV=1 - [SERC5_MOUSE]	-0.66	-0.28	-0.74	-0.36	0.02
Q91W53	Golgin subfamily A member 7 OS=Mus musculus GN=Golga7 PE=2 SV=1 - [GOGA7_MOUSE]	-0.11	-0.11	-0.25	-0.25	0.02
E9Q066	La-related protein 4 OS=Mus musculus GN=Larp4 PE=2 SV=1 [E9Q066_MOUSE]	0.23	0.15	0.32	0.43	0.02
Q9EPL8	Importin-7 OS=Mus musculus GN=Ipo7 PE=1 SV=2 - [IPO7_MOUSE]	0.36	0.14	0.26	0.16	0.02
Q9Z2X2	26S proteasome non-ATPase regulatory subunit 10 OS=Mus musculus GN=Psmd10 PE=1 SV=3 - [PSD10_MOUSE]	-0.12	-0.13	-0.27	-0.28	0.02
O55229	Choline/ethanolamine kinase OS=Mus musculus GN=Chkb PE=1 SV=3 - [CHKB_MOUSE]	0.11	0.24	0.10	0.19	0.02
P97789-3	Isoform 3 of 5'-3' exoribonuclease 1 OS=Mus musculus GN=Xrn1 - [XRN1_MOUSE]	0.24	0.32	0.15	0.44	0.02
Q91WM3	U3 small nucleolar RNA-interacting protein 2 OS=Mus musculus GN=Rrp9 PE=1 SV=1 - [U3IP2_MOUSE]	0.26	0.31	0.59	0.65	0.02
Q8C3Y4	Kinetochore-associated protein 1 OS=Mus musculus GN=Kntc1 PE=1 SV=2 - [KNTC1_MOUSE]	0.28	0.62	0.60	0.94	0.02
Q99LL3	Carbohydrate sulfotransferase 12 OS=Mus musculus GN=Chst12 PE=2 SV=2 - [CHSTC_MOUSE]	0.45	0.48	0.20	0.22	0.02
B1ARW4	NADH dehydrogenase [ubiquinone] iron-sulfur protein 5 (Fragment) OS=Mus musculus GN=Ndufs5 PE=2 SV=1 - [B1ARW4_MOUSE]	0.05	0.13	0.19	0.14	0.02

Q9WVL6	Exostosin-like 3 OS=Mus musculus GN=Extl3 PE=2 SV=2 - [EXTL3_MOUSE]	-0.39	-0.67	-0.21	-0.49	0.02
Q8BVE3	V-type proton ATPase subunit H OS=Mus musculus GN=Atp6v1h PE=1 SV=1 - [VATH_MOUSE]	0.21	0.37	0.12	0.25	0.02
Q6PNC0	DmX-like protein 1 OS=Mus musculus GN=Dmxl1 PE=1 SV=1 - [DMXL1_MOUSE]	-1.06	-0.85	-0.57	-0.36	0.02
E0CZ73	Occludin OS=Mus musculus GN=Ocln PE=2 SV=1 - [E0CZ73_MOUSE]	-0.21	-0.07	-0.24	-0.14	0.02
Q3UV66	Protein Slfn4 OS=Mus musculus GN=Slfn4 PE=2 SV=1 - [Q3UV66_MOUSE]	0.28	0.41	0.69	0.79	0.02
F7B6K4	Pantothenate kinase 4 (Fragment) OS=Mus musculus GN=Pank4 PE=4 SV=1 - [F7B6K4_MOUSE]	-0.16	-0.07	-0.22	-0.14	0.02
Q91VS8	FERM, RhoGEF and pleckstrin domain-containing protein 2 OS=Mus musculus GN=Farp2 PE=1 SV=2 [FARP2_MOUSE]	-0.68	-0.23	-0.60	-0.37	0.02
E9Q1F5	Protein Myo5c OS=Mus musculus GN=Myo5c PE=2 SV=1 - [E9Q1F5_MOUSE]	0.28	0.42	0.18	0.54	0.02
Q8VDD8	WAS protein family homolog 1 OS=Mus musculus GN=Wash1 PE=2 SV=1 - [WASH1_MOUSE]	0.40	0.41	0.21	0.16	0.02
F6ZJ37	Zinc finger protein 827 (Fragment) OS=Mus musculus GN=Zfp827 PE=4 SV=1 - [F6ZJ37_MOUSE]	0.27	0.61	0.27	0.61	0.02
Q6NVE8	WD repeat-containing protein 44 OS=Mus musculus GN=Wdr44 PE=1 SV=1 - [WDR44_MOUSE]	0.33	0.31	0.28	0.09	0.02
Q99MR8	Methylcrotonoyl-CoA carboxylase subunit alpha, mitochondrial OS=Mus musculus GN=Mccc1 PE=2 SV=2 - [MCCA_MOUSE]	-0.13	-0.27	-0.10	-0.17	0.02
Q80UG5	Septin-9 OS=Mus musculus GN=Sept9 PE=1 SV=1 - [SEPT9_MOUSE]	-0.09	-0.08	-0.08	-0.18	0.02
G3UXW3	Protein Ccdc28a (Fragment) OS=Mus musculus GN=Ccdc28a PE=4 SV=1 - [G3UXW3_MOUSE]	-1.52	-0.72	-1.40	-0.60	0.02
E9Q043	Protein Fndc1 OS=Mus musculus GN=Fndc1 PE=2 SV=1 - [E9Q043_MOUSE]	0.39	0.34	0.19	0.14	0.02

E9Q421	GPI-anchor transamidase OS=Mus musculus GN=Pigk PE=2 SV=1 - [E9Q421_MOUSE]	0.09	0.04	0.12	0.07	0.02
Q3TKR3	NACHT, LRR and PYD domains-containing protein 4C OS=Mus musculus GN=Nlrp4c PE=2 SV=1 - [NAL4C_MOUSE]	0.41	0.69	0.21	0.49	0.02
Q8BZ33	Tensin-4 OS=Mus musculus GN=Tns4 PE=2 SV=2 - [TENS4_MOUSE]	-0.42	-0.29	-0.25	-0.13	0.02
O35435	Dihydroorotate dehydrogenase (quinone), mitochondrial OS=Mus musculus GN=Dhodh PE=2 SV=2 - [PYRD_MOUSE]	0.25	0.21	0.41	0.15	0.02
Q3ZAS0	Sodium/hydrogen exchanger OS=Mus musculus GN=Slc9a2 PE=2 SV=1 - [Q3ZAS0_MOUSE]	-0.14	-0.32	-0.27	-0.45	0.02
Q9DCT5	Stromal cell-derived factor 2 OS=Mus musculus GN=Sdf2 PE=2 SV=1 - [SDF2_MOUSE]	-0.22	-0.19	-0.09	-0.09	0.02
Q80ZM8	Cardiolipin synthase OS=Mus musculus GN=Crls1 PE=2 SV=1 - [CRLS1_MOUSE]	0.41	0.27	0.26	0.12	0.02
E9PVU9	Bis(5'-adenosyl)-triphosphatase (Fragment) OS=Mus musculus GN=Fhit PE=2 SV=1 - [E9PVU9_MOUSE]	-0.47	-0.62	-0.20	-0.34	0.02
Q9CPX6	Ubiquitin-like-conjugating enzyme ATG3 OS=Mus musculus GN=Atg3 PE=1 SV=1 - [ATG3_MOUSE]	0.14	0.41	0.24	0.42	0.02
Q9D7H3	RNA 3'-terminal phosphate cyclase OS=Mus musculus GN=RtcA PE=2 SV=2 - [RTCA_MOUSE]	-0.21	-0.13	-0.29	-0.11	0.02
Q9CQR4	Acyl-coenzyme A thioesterase 13 OS=Mus musculus GN=Acot13 PE=1 SV=1 - [ACO13_MOUSE]	-0.73	-0.35	-0.91	-0.41	0.02
P27046	Alpha-mannosidase 2 OS=Mus musculus GN=Man2a1 PE=1 SV=2 - [MA2A1_MOUSE]	0.06	0.11	0.19	0.15	0.02
B1AW21	Myotubularin OS=Mus musculus GN=Mtm1 PE=4 SV=1 - [B1AW21_MOUSE]	-0.43	-0.23	-0.18	-0.21	0.02
H3BKM2	Nuclear-interacting partner of ALK OS=Mus musculus GN=Zc3hc1 PE=2 SV=1 - [H3BKM2_MOUSE]	0.27	0.18	0.52	0.43	0.02

Q8BLU2	Protein-lysine methyltransferase METTL21C OS=Mus musculus GN=Mettl21c PE=2 SV=1 - [MT21C_MOUSE]	0.33	0.51	0.15	0.34	0.02
Q9DCL2	MIP18 family protein FAM96A OS=Mus musculus GN=Fam96a PE=2 SV=1 - [FA96A_MOUSE]	-0.07	-0.09	-0.17	-0.18	0.02
Q6P4T1	Protein Snx19 OS=Mus musculus GN=Snx19 PE=2 SV=1 - [Q6P4T1_MOUSE]	-0.66	-1.46	-0.61	-1.41	0.02
Q8K4X7	1-acyl-sn-glycerol-3-phosphate acyltransferase delta OS=Mus musculus GN=Agpat4 PE=2 SV=1 - [PLCD_MOUSE]	0.22	0.28	0.07	0.25	0.02
Q69ZT1-2	Isoform 2 of Fanconi-associated nuclease 1 OS=Mus musculus GN=Fan1 - [FAN1_MOUSE]	-0.23	-0.13	-0.41	-0.31	0.02
Q8CBC4	Consortin OS=Mus musculus GN=Cnst PE=1 SV=1 - [CNST_MOUSE]	-0.45	-0.72	-0.21	-0.60	0.02
Q6PB44-2	Isoform 2 of Tyrosine-protein phosphatase non-receptor type 23 OS=Mus musculus GN=Ptpn23 - [PTN23_MOUSE]	0.20	0.52	0.28	0.53	0.02
Q8CIE4	Plec1 protein OS=Mus musculus GN=Parp10 PE=2 SV=1 - [Q8CIE4_MOUSE]	0.26	0.14	0.46	0.33	0.02
Q9R060	Cytosolic Fe-S cluster assembly factor NUBP1 OS=Mus musculus GN=Nubp1 PE=1 SV=1 - [NUBP1_MOUSE]	0.65	0.48	0.39	0.19	0.02
A1L317	Keratin, type I cytoskeletal 24 OS=Mus musculus GN=Krt24 PE=2 SV=2 - [K1C24_MOUSE]	-1.02	-0.50	-0.90	-0.37	0.02
P70261	Paladin OS=Mus musculus GN=Pald1 PE=1 SV=1 - [PALD_MOUSE]	-0.52	-0.24	-0.83	-0.54	0.02
Q8C163	Nuclease EXOG, mitochondrial OS=Mus musculus GN=Exog PE=2 SV=1 - [EXOG_MOUSE]	-0.46	-0.66	-0.20	-0.39	0.02
Q9QYR6	Microtubule-associated protein 1A OS=Mus musculus GN=Map1a PE=1 SV=2 - [MAP1A_MOUSE]	-0.09	-0.14	-0.20	-0.27	0.02
F8WGM8	Actin, cytoplasmic 2 (Fragment) OS=Mus musculus GN=Actg1 PE=2 SV=1 - [F8WGM8_MOUSE]	0.07	0.13	0.16	0.22	0.02

Q9WVA2	Mitochondrial import inner membrane translocase subunit Tim8 A OS=Mus musculus GN=Timm8a1 PE=1 SV=1 - [TIM8A_MOUSE]	-0.39	-0.15	-0.48	-0.26	0.02
Q9D1P4	Cysteine and histidine-rich domain-containing protein 1 OS=Mus musculus GN=Chordc1 PE=1 SV=1 - [CHRD1_MOUSE]	0.25	0.39	0.11	0.33	0.02
Q8R4P9-2	Isoform 2 of Multidrug resistance-associated protein 7 OS=Mus musculus GN=Abcc10 - [MRP7_MOUSE]	-0.41	-0.20	-0.68	-0.47	0.02
Q8BTI8-3	Isoform 3 of Serine/arginine repetitive matrix protein 2 OS=Mus musculus GN=Srrm2 - [SRRM2_MOUSE]	-0.07	-0.03	-0.09	-0.05	0.02
B0QZL2	Nicotinamide mononucleotide adenylyltransferase 1 OS=Mus musculus GN=Nmnat1 PE=2 SV=1 - [B0QZL2_MOUSE]	-0.83	-0.56	-0.51	-0.24	0.02
Q9CPR5	39S ribosomal protein L15, mitochondrial OS=Mus musculus GN=Mrpl15 PE=1 SV=1 - [RM15_MOUSE]	-0.27	-0.28	-0.18	-0.09	0.02
Q8CE96-3	Isoform 3 of tRNA (adenine(58)-N(1))-methyltransferase non-catalytic subunit TRM6 OS=Mus musculus GN=Trmt6 - [TRM6_MOUSE]	0.27	0.11	0.33	0.16	0.02
Q9CZW4	Long-chain-fatty-acid--CoA ligase 3 OS=Mus musculus GN=Acsl3 PE=2 SV=2 - [ACSL3_MOUSE]	0.27	0.59	0.25	0.59	0.02
Q9CWD3	Nucleoside diphosphate-linked moiety X motif 17 OS=Mus musculus GN=Nudt17 PE=2 SV=1 - [NUD17_MOUSE]	-0.24	-0.11	-0.22	-0.09	0.02
Q6P1E8-3	Isoform 2 of EF-hand calcium-binding domain containing protein 6 OS=Mus musculus GN=Efcab6 - [EFCB6_MOUSE]	-1.29	-2.78	-1.10	-2.59	0.02
P56873	Sjoegren syndrome/scleroderma autoantigen 1 homolog OS=Mus musculus GN=Sssca1 PE=2 SV=1 - [SSA27_MOUSE]	-0.13	-0.09	-0.26	-0.23	0.02
Q9D554	Splicing factor 3A subunit 3 OS=Mus musculus GN=Sf3a3 PE=2 SV=2 - [SF3A3_MOUSE]	0.14	0.22	0.09	0.09	0.02

E9Q8Q1	BRCA1-A complex subunit BRE OS=Mus musculus GN=Bre PE=2 SV=1 - [E9Q8Q1_MOUSE]	0.23	0.21	0.51	0.49	0.02
Q8VD75	Huntingtin-interacting protein 1 OS=Mus musculus GN=Hip1 PE=1 SV=2 - [HIP1_MOUSE]	0.15	0.31	0.11	0.23	0.02
D3Z7Q2	Uncharacterized protein OS=Mus musculus GN=Smim20 PE=2 SV=1 - [D3Z7Q2_MOUSE]	0.39	0.16	0.46	0.23	0.02
Q9D1H8	39S ribosomal protein L53, mitochondrial OS=Mus musculus GN=Mrpl53 PE=2 SV=1 - [RM53_MOUSE]	0.45	0.27	0.28	0.14	0.02
E9QAD4	Coiled-coil domain-containing protein 93 OS=Mus musculus GN=Ccdc93 PE=2 SV=1 - [E9QAD4_MOUSE]	0.18	0.23	0.33	0.10	0.02
Q8R395	COMM domain-containing protein 5 OS=Mus musculus GN=Commd5 PE=2 SV=1 - [COMD5_MOUSE]	0.34	0.14	0.47	0.27	0.02
Q9JMA1	Ubiquitin carboxyl-terminal hydrolase 14 OS=Mus musculus GN=Usp14 PE=1 SV=3 - [UBP14_MOUSE]	0.23	0.40	0.12	0.27	0.02
E9Q2X6	Structural maintenance of chromosomes protein OS=Mus musculus GN=Smc4 PE=2 SV=1 - [E9Q2X6_MOUSE]	0.43	0.21	0.41	0.17	0.02
Q5ND34-3	Isoform 3 of WD repeat-containing protein 81 OS=Mus musculus GN=Wdr81 - [WDR81_MOUSE]	0.13	0.24	0.33	0.44	0.02
Q8BIV3	Ran-binding protein 6 OS=Mus musculus GN=Ranbp6 PE=2 SV=3 - [RNBP6_MOUSE]	-0.32	-0.12	-0.36	-0.19	0.02
P14483	H-2 class II histocompatibility antigen, A beta chain OS=Mus musculus GN=H2-Ab1 PE=1 SV=1 - [HB2A_MOUSE]	-0.14	-0.04	-0.12	-0.15	0.02
Q9ER41	Torsin-1B OS=Mus musculus GN=Tor1b PE=2 SV=2 - [TOR1B_MOUSE]	-0.23	-0.16	-0.32	-0.47	0.02
P97500-4	Isoform 4 of Myelin transcription factor 1-like protein OS=Mus musculus GN=Myt1l - [MYT1L_MOUSE]	-0.63	-0.41	-0.39	-0.18	0.02

Q9EQG9-2	Isoform 2 of Collagen type IV alpha-3-binding protein OS=Mus musculus GN=Col4a3bp - [C43BP_MOUSE]	0.12	0.08	0.15	0.24	0.02
G3X999	Protein A330021E22Rik (Fragment) OS=Mus musculus GN=A330021E22Rik PE=4 SV=1 - [G3X999_MOUSE]	-0.09	-0.17	-0.06	-0.14	0.02
E9PXY1	Cullin-4B OS=Mus musculus GN=Cul4b PE=3 SV=2 - [E9PXY1_MOUSE]	0.34	0.16	0.27	0.12	0.02
O35685	Nuclear migration protein nudC OS=Mus musculus GN=Nudc PE=1 SV=1 - [NUDC_MOUSE]	0.22	0.32	0.09	0.24	0.02
P41105	60S ribosomal protein L28 OS=Mus musculus GN=Rpl28 PE=1 SV=2 [RL28_MOUSE]	0.07	0.14	0.05	0.13	0.02
Q91WJ7-2	Isoform 2 of SPATS2-like protein OS=Mus musculus GN=Spats2l - [SPS2L_MOUSE]	1.54	1.05	1.35	0.42	0.02
P70697	Uroporphyrinogen decarboxylase OS=Mus musculus GN=Urod PE=1 SV=2 - [DCUP_MOUSE]	-0.20	-0.39	-0.15	-0.20	0.02
Q05860-4	Isoform 4 of Formin-1 OS=Mus musculus GN=Fmn1 - [FMN1_MOUSE]	-0.13	-0.20	-0.32	-0.39	0.02
Q3V117	ATP-citrate synthase OS=Mus musculus GN=Acly PE=2 SV=1 - [Q3V117_MOUSE]	0.41	1.12	0.54	1.01	0.02
Q8C9S4	Uncharacterized protein C10orf118 homolog OS=Mus musculus GN=Otg1 PE=2 SV=2 - [CJ118_MOUSE]	-0.26	-0.11	-0.40	-0.31	0.02
Q60767	Lymphocyte antigen 75 OS=Mus musculus GN=Ly75 PE=1 SV=2 - [LY75_MOUSE]	-0.29	-0.40	-0.12	-0.23	0.02
Q921M7	Protein FAM49B OS=Mus musculus GN=Fam49b PE=2 SV=1 - [FA49B_MOUSE]	0.44	0.91	0.32	0.61	0.02
Q8VFZ8	MCG1048721 OS=Mus musculus GN=Olfr781 PE=3 SV=1 - [Q8VFZ8_MOUSE]	-0.39	-0.74	-0.24	-0.60	0.02
Q7TPS5-3	Isoform 3 of C2 domain containing protein 5 OS=Mus musculus GN=C2cd5 - [C2CD5_MOUSE]	-0.62	-0.48	-0.34	-0.20	0.02
Q8BQX0	RAB37, member of RAS oncogene family, isoform CRA_a OS=Mus musculus GN=Rab37 PE=2 SV=1 - [Q8BQX0_MOUSE]	-0.38	-0.62	-0.18	-0.42	0.02

Q6P9Q4	FH1/FH2 domain-containing protein 1 OS=Mus musculus GN=Fhod1 PE=2 SV=3 - [FHOD1_MOUSE]	-0.09	-0.20	-0.08	-0.12	0.02
P63094-2	Isoform Gnas-2 of Guanine nucleotide-binding protein G(s) subunit alpha isoforms short OS=Mus musculus GN=Gnas - [GNAS2_MOUSE]	-0.10	-0.22	-0.27	-0.36	0.02
Q9JJF0	Nucleosome assembly protein 1-like 5 OS=Mus musculus GN=Nap1l5 PE=2 SV=1 - [NP1L5_MOUSE]	0.57	0.26	0.52	0.22	0.02
Q7TPS0-2	Isoform 2 of Ribosomal protein S6 kinase alpha 6 OS=Mus musculus GN=Rps6ka6 - [KS6A6_MOUSE]	0.41	1.00	0.51	1.09	0.02
Q7TMR0	Lysosomal Pro-X carboxypeptidase OS=Mus musculus GN=Prcp PE=2 SV=2 - [PCP_MOUSE]	-0.19	-0.12	-0.36	-0.31	0.02
Q9CVD2	Ataxin-3 OS=Mus musculus GN=Atxn3 PE=1 SV=2 - [ATX3_MOUSE]	-0.18	-0.31	-0.09	-0.22	0.02
D3YTN4	GTPase IMAP family member 4 OS=Mus musculus GN=Gimap4 PE=2 SV=1 - [D3YTN4_MOUSE]	0.27	0.50	0.16	0.43	0.02
Q8CID0	Cysteine-rich protein 2-binding protein OS=Mus musculus GN=Csrp2bp PE=2 SV=2 - [CSR2B_MOUSE]	-0.34	-0.37	-0.14	-0.18	0.02
F6ZSM3	Annexin OS=Mus musculus GN=Anxa13 PE=3 SV=2 - [F6ZSM3_MOUSE]	0.53	0.44	0.33	0.15	0.02
Q9NYQ2	Hydroxyacid oxidase 2 OS=Mus musculus GN=Hao2 PE=2 SV=1 - [HAOX2_MOUSE]	-0.03	-0.10	-0.08	-0.11	0.02
P17427	AP-2 complex subunit alpha-2 OS=Mus musculus GN=Ap2a2 PE=1 SV=2 - [AP2A2_MOUSE]	-0.06	-0.17	-0.16	-0.24	0.02
Q921B4	Neurotrophin receptor-interacting factor 2 OS=Mus musculus GN=Nrif2 PE=1 SV=1 - [NRIF2_MOUSE]	-0.63	-0.27	-0.65	-0.29	0.02
Q6P9J9	Anoctamin-6 OS=Mus musculus GN=Ano6 PE=1 SV=1 - [ANO6_MOUSE]	-0.19	-0.23	-0.32	-0.09	0.02
Q3TVF4	Geranylgeranyl transferase type-2 subunit beta OS=Mus musculus GN=Rabggtb PE=2 SV=1 - [Q3TVF4_MOUSE]	0.06	0.15	0.09	0.18	0.02

Q91W59-2	Isoform 2 of RNA-binding motif, single-stranded-interacting protein 1 OS=Mus musculus GN=Rbms1 - [RBMS1_MOUSE]	-0.18	-0.10	-0.05	-0.16	0.02
P29341	Polyadenylate-binding protein 1 OS=Mus musculus GN=Pabpc1 PE=1 SV=2 - [PABP1_MOUSE]	0.03	0.04	0.01	0.05	0.02
D3YYU8	Protein Obsl1 OS=Mus musculus GN=Obsl1 PE=2 SV=1 - [D3YYU8_MOUSE]	-0.13	-0.26	-0.32	-0.45	0.02
Q8BP27-2	Isoform 2 of Swi5-dependent recombination DNA repair protein 1 homolog OS=Mus musculus GN=Sfr1 - [SFR1_MOUSE]	0.07	0.17	0.08	0.18	0.02
Q922Q4	Pyrroline-5-carboxylate reductase 2 OS=Mus musculus GN=Pycr2 PE=2 SV=1 - [P5CR2_MOUSE]	0.16	0.12	0.13	0.04	0.02
Q6P8X1	Sorting nexin-6 OS=Mus musculus GN=Snx6 PE=1 SV=2 - [SNX6_MOUSE]	0.11	0.30	0.17	0.32	0.02
Q9R0X4	Acyl-coenzyme A thioesterase 9, mitochondrial OS=Mus musculus GN=Acot9 PE=1 SV=1 - [ACOT9_MOUSE]	-0.17	-0.05	-0.21	-0.18	0.02
G3X8U3	MCG6895 OS=Mus musculus GN=2210016F16Rik PE=4 SV=1 - [G3X8U3_MOUSE]	0.02	0.05	0.05	0.07	0.02
Q9D7N9	Adipocyte plasma membrane-associated protein OS=Mus musculus GN=Apmap PE=1 SV=1 - [APMAP_MOUSE]	-0.17	-0.07	-0.20	-0.10	0.02
Q62086	Serum paraoxonase/arylesterase 2 OS=Mus musculus GN=Pon2 PE=1 SV=2 - [PON2_MOUSE]	0.16	0.32	0.11	0.28	0.02
D3YVL0	Putative helicase MOV-10 OS=Mus musculus GN=Mov10 PE=4 SV=1 - [D3YVL0_MOUSE]	0.18	0.28	0.13	0.38	0.02
Q8BP48	Methionine aminopeptidase 1 OS=Mus musculus GN=Metap1 PE=2 SV=1 - [MAP11_MOUSE]	0.14	0.24	0.09	0.26	0.02
P83870	PHD finger-like domain-containing protein 5A OS=Mus musculus GN=Phf5a PE=1 SV=1 [PHF5A_MOUSE]	-0.12	-0.06	-0.04	-0.12	0.02

Q8R3H7	Heparan sulfate 2-O-sulfotransferase 1 OS=Mus musculus GN=Hs2st1 PE=1 SV=2 - [HS2ST_MOUSE]	0.28	0.11	0.37	0.19	0.02
E9Q9I2	Protein Dlg5 OS=Mus musculus GN=Dlg5 PE=2 SV=1 - [E9Q9I2_MOUSE]	-0.58	-0.31	-0.53	-0.19	0.02
Q9JHU9	Inositol-3-phosphate synthase 1 OS=Mus musculus GN=Isyna1 PE=2 SV=1 - [INO1_MOUSE]	-0.16	-0.07	-0.23	-0.12	0.02
Q9DCL9	Multifunctional protein ADE2 OS=Mus musculus GN=Paics PE=1 SV=4 - [PUR6_MOUSE]	0.14	0.08	0.24	0.12	0.02
P97390	Vacuolar protein sorting associated protein 45 OS=Mus musculus GN=Vps45 PE=1 SV=1 - [VPS45_MOUSE]	0.10	0.26	0.11	0.25	0.02
O09174	Alpha-methylacyl-CoA racemase OS=Mus musculus GN=Amacr PE=1 SV=4 - [AMACR_MOUSE]	-0.25	-0.49	-0.15	-0.39	0.02
Q9CQY1	Ubiquitin-like protein ATG12 OS=Mus musculus GN=Atg12 PE=1 SV=1 - [ATG12_MOUSE]	0.22	0.46	0.17	0.41	0.02
Q9D1M0	Protein SEC13 homolog OS=Mus musculus GN=Sec13 PE=2 SV=3 - [SEC13_MOUSE]	-0.05	-0.10	-0.07	-0.03	0.02
B1AUY7	N-alpha-acetyltransferase 10 OS=Mus musculus GN=Naa10 PE=2 SV=1 - [B1AUY7_MOUSE]	-0.58	-1.16	-0.39	-0.97	0.02
Q9DAK8-2	Isoform 2 of Leucine-rich repeat-containing protein 51 OS=Mus musculus GN=Lrrc51 - [LRC51_MOUSE]	0.29	0.12	0.41	0.24	0.02
Q8VFI7	Olfactory receptor 866 OS=Mus musculus GN=Olfr866 PE=2 SV=1 - [Q8VFI7_MOUSE]	0.15	0.33	0.12	0.30	0.02
Q91ZU6-5	Isoform 5 of Dystonin OS=Mus musculus GN=Dst - [DYST_MOUSE]	-1.15	-1.13	-0.50	-0.48	0.02
Q9CPZ8	COX assembly mitochondrial protein homolog OS=Mus musculus GN=Cmc1 PE=3 SV=1 - [COXM1_MOUSE]	0.07	0.04	0.06	0.02	0.02
Q80XU2	Neuronal proto-oncogene tyrosine-protein kinase Src OS=Mus musculus GN=Src PE=2 SV=1 - [Q80XU2_MOUSE]	-0.25	-0.18	-0.42	-0.17	0.02

Q63810-2	Isoform 2 of Calcineurin subunit B type 1 OS=Mus musculus GN=Ppp3r1 - [CANB1_MOUSE]	0.13	0.32	0.16	0.33	0.02
Q8BH49	Sesquipedalian-1 OS=Mus musculus GN=Fam109a PE=2 SV=1 - [SESQ1_MOUSE]	-0.56	-1.06	-0.33	-0.83	0.02
Q8BPU7	Engulfment and cell motility protein 1 OS=Mus musculus GN=Elmo1 PE=1 SV=2 - [ELMO1_MOUSE]	0.08	0.10	0.21	0.20	0.02
Q8CCK0	Core histone macro-H2A.2 OS=Mus musculus GN=H2afy2 PE=1 SV=3 - [H2AW_MOUSE]	-0.15	-0.44	-0.21	-0.30	0.02
E9QNN1	ATP-dependent RNA helicase A OS=Mus musculus GN=Dhx9 PE=2 SV=1 - [E9QNN1_MOUSE]	0.03	0.10	0.11	0.14	0.02
P55012	Solute carrier family 12 member 2 OS=Mus musculus GN=Slc12a2 PE=1 SV=2 - [S12A2_MOUSE]	0.08	0.20	0.15	0.27	0.02
Q9QXJ2	Signal transducer and activator of transcription 2 OS=Mus musculus GN=Stat2 PE=1 SV=1 - [Q9QXJ2_MOUSE]	0.07	0.10	0.21	0.16	0.02
Q8C257	Il18r1 protein OS=Mus musculus GN=Il18r1 PE=2 SV=1 - [Q8C257_MOUSE]	-0.48	-0.38	-0.24	-0.15	0.02
P61971	Nuclear transport factor 2 OS=Mus musculus GN=Nutf2 PE=2 SV=1 - [NTF2_MOUSE]	0.05	0.14	0.07	0.15	0.02
Q8VC30	Bifunctional ATP-dependent dihydroxyacetone kinase/FAD-AMP lyase (cyclizing) OS=Mus musculus GN=Dak PE=2 SV=1 - [DHAK_MOUSE]	-0.59	-0.30	-0.37	-0.20	0.02
P09411	Phosphoglycerate kinase 1 OS=Mus musculus GN=Pgk1 PE=1 SV=4 - [PGK1_MOUSE]	0.20	0.35	0.10	0.26	0.02
F7CFC7	Protein 2010015L04Rik (Fragment) OS=Mus musculus GN=2010015L04Rik PE=2 SV=1 - [F7CFC7_MOUSE]	-0.72	-0.29	-1.03	-0.61	0.02
Q9D0K2	Succinyl-CoA:3-ketoacid coenzyme A transferase 1, mitochondrial OS=Mus musculus GN=Oxct1 PE=1 SV=1 - [SCOT1_MOUSE]	-0.09	-0.08	-0.05	-0.02	0.02

Q6ZQ73	Cullin-associated NEDD8-dissociated protein 2 OS=Mus musculus GN=Cand2 PE=1 SV=2 - [CAND2_MOUSE]	0.27	0.12	0.27	0.11	0.02
Q99K99	Uncharacterized protein C4orf19 homolog OS=Mus musculus PE=1 SV=2 - [CD019_MOUSE]	-0.12	-0.06	-0.22	-0.16	0.02
A6H8H2	DENN domain-containing protein 4C OS=Mus musculus GN=Dennd4c PE=1 SV=1 - [DEN4C_MOUSE]	0.21	0.31	0.08	0.22	0.02
P61166	Transmembrane protein 258 OS=Mus musculus GN=Tmem258 PE=2 SV=1 - [TM258_MOUSE]	0.10	0.13	0.03	0.11	0.02
Q69Z37	Sterile alpha motif domain-containing protein 9-like OS=Mus musculus GN=Samd9l PE=2 SV=2 - [SAM9L_MOUSE]	0.42	0.10	0.37	0.40	0.02
D3YZB3	Synaptojanin-2 OS=Mus musculus GN=Synj2 PE=2 SV=1 [D3YZB3_MOUSE]	-0.18	-0.13	-0.05	-0.17	0.02
O88455	7-dehydrocholesterol reductase OS=Mus musculus GN=Dhcr7 PE=2 SV=1 - [DHCR7_MOUSE]	0.29	0.53	0.18	0.53	0.02
A2CF80	Protein 0610010K14Rik OS=Mus musculus GN=0610010K14Rik PE=2 SV=1 - [A2CF80_MOUSE]	0.15	0.11	0.04	0.14	0.02
Q8BX09-2	Isoform 2 of Retinoblastoma-binding protein 5 OS=Mus musculus GN=Rbbp5 - [RBBP5_MOUSE]	-0.17	-0.27	-0.10	-0.12	0.02
Q9WUM4	Coronin-1C OS=Mus musculus GN=Coro1c PE=1 SV=2 - [COR1C_MOUSE]	0.17	0.30	0.09	0.22	0.02
Q9D975	Sulfiredoxin-1 OS=Mus musculus GN=Srxn1 PE=2 SV=1 - [SRXN1_MOUSE]	-0.35	-0.87	-0.47	-0.99	0.02
P80560	Receptor-type tyrosine-protein phosphatase N2 OS=Mus musculus GN=Ptprn2 PE=1 SV=2 - [PTPR2_MOUSE]	-0.39	-0.11	-0.36	-0.24	0.02
E9Q357	Protein Gm17673 OS=Mus musculus GN=Gm17673 PE=4 SV=2 - [E9Q357_MOUSE]	0.67	0.88	0.26	0.47	0.02

E9Q714	Putative polypeptide N-acetylgalactosaminyltransferase-like protein 3 OS=Mus musculus GN=Wbscr17 PE=2 SV=1 - [E9Q714_MOUSE]	-0.62	-0.77	-1.53	-1.68	0.02
Q60872	Eukaryotic translation initiation factor 1A OS=Mus musculus GN=Eif1a PE=2 SV=3 - [IF1A_MOUSE]	0.44	0.66	0.17	0.48	0.02
A2A5R2	Brefeldin A-inhibited guanine nucleotide-exchange protein 2 OS=Mus musculus GN=Arfgef2 PE=1 SV=1 - [BIG2_MOUSE]	0.22	0.38	0.16	0.47	0.02
P35278	Ras-related protein Rab-5C OS=Mus musculus GN=Rab5c PE=1 SV=2 - [RAB5C_MOUSE]	0.31	0.18	0.11	0.15	0.02
Q9QXB9	Developmentally-regulated GTP-binding protein 2 OS=Mus musculus GN=Drg2 PE=1 SV=1 - [DRG2_MOUSE]	0.42	0.69	0.19	0.48	0.02
E0CY15	DNA-directed RNA polymerase, mitochondrial OS=Mus musculus GN=Polrmt PE=2 SV=1 - [E0CY15_MOUSE]	0.73	0.95	0.28	0.50	0.02
Q9CPW3	39S ribosomal protein L54, mitochondrial OS=Mus musculus GN=Mrpl54 PE=2 SV=1 - [RM54_MOUSE]	0.75	1.50	0.48	1.24	0.02
E9Q9T1	Leucine-rich repeat flightless-interacting protein 1 OS=Mus musculus GN=Lrrfip1 PE=4 SV=2 - [E9Q9T1_MOUSE]	-0.21	-0.08	-0.29	-0.17	0.02
Q8R404	Protein QIL1 OS=Mus musculus GN=Qil1 PE=2 SV=1 - [QIL1_MOUSE]	0.27	0.39	0.11	0.23	0.02
B1AXP6	Mitochondrial import receptor subunit TOM5 homolog OS=Mus musculus GN=Tomm5 PE=3 SV=1 - [TOM5_MOUSE]	-0.21	-0.41	-0.18	-0.49	0.02
P50518	V-type proton ATPase subunit E 1 OS=Mus musculus GN=Atp6v1e1 PE=1 SV=2 - [VATE1_MOUSE]	0.09	0.19	0.06	0.14	0.02
Q5SRX1-3	Isoform 3 of TOM1-like protein 2 OS=Mus musculus GN=Tom1l2 - [TM1L2_MOUSE]	-0.31	-0.59	-0.18	-0.36	0.02
A2AJK8	Tetratricopeptide repeat protein 1 OS=Mus musculus GN=Ttc1 PE=2 SV=1 - [A2AJK8_MOUSE]	0.15	0.39	0.16	0.26	0.02

Q8BR93-2	Isoform 2 of Putative nuclease HARBI1 OS=Mus musculus GN=Harbi1 - [HARB1_MOUSE]	0.68	0.96	0.27	0.54	0.02
P35487	Pyruvate dehydrogenase E1 component subunit alpha, testis-specific form, mitochondrial OS=Mus musculus GN=Pdha2 PE=2 SV=1 - [ODPAT_MOUSE]	-0.77	-1.74	-0.82	-1.98	0.02
A2BI14	Coiled-coil domain-containing protein 171 (Fragment) OS=Mus musculus GN=Ccdc171 PE=2 SV=1 - [A2BI14_MOUSE]	0.25	0.72	0.34	0.63	0.02
P01631	Ig kappa chain V-II region 26-10 OS=Mus musculus PE=1 SV=1 - [KV2A7_MOUSE]	0.20	0.33	0.20	0.50	0.02
O35945	Aldehyde dehydrogenase, cytosolic 1 OS=Mus musculus GN=Aldh1a7 PE=2 SV=1 - [AL1A7_MOUSE]	-0.37	-0.30	-0.18	-0.12	0.02
Q08024	Core-binding factor subunit beta OS=Mus musculus GN=Cbfb PE=1 SV=1 - [PEBB_MOUSE]	0.15	0.40	0.18	0.37	0.02
Q8BVL3	Sorting nexin-17 OS=Mus musculus GN=Snx17 PE=1 SV=2 - [SNX17_MOUSE]	0.32	0.66	0.22	0.58	0.02
Q91WD0-2	Isoform 2 of Protein GPR108 OS=Mus musculus GN=Gpr108 - [GP108_MOUSE]	0.63	0.39	0.42	0.17	0.02
Q3TQI7	Uncharacterized protein C9orf78 homolog OS=Mus musculus PE=1 SV=2 - [CI078_MOUSE]	-0.34	-0.14	-0.38	-0.18	0.02
A2ASS6	Titin OS=Mus musculus GN=Ttn PE=1 SV=1 - [TITIN_MOUSE]	0.04	0.16	0.14	0.15	0.02
Q9JIA7	Sphingosine kinase 2 OS=Mus musculus GN=Sphk2 PE=1 SV=2 - [SPHK2_MOUSE]	0.10	0.08	0.21	0.19	0.02
Q80YQ2	Mediator of RNA polymerase II transcription subunit 23 OS=Mus musculus GN=Med23 PE=1 SV=2 - [MED23_MOUSE]	-0.12	-0.28	-0.11	-0.27	0.02
Q8K154	Protein Ugt2b34 OS=Mus musculus GN=Ugt2b34 PE=2 SV=1 - [Q8K154_MOUSE]	-0.19	-0.05	-0.15	-0.11	0.02
Q9Z2D6	Methyl-CpG-binding protein 2 OS=Mus musculus GN=Mecp2 PE=1 SV=1 - [MECP2_MOUSE]	-0.04	-0.09	-0.05	-0.12	0.02

Q7TQK1	Integrator complex subunit 7 OS=Mus musculus GN=Ints7 PE=1 SV=1 - [INT7_MOUSE]	0.15	0.26	0.45	0.48	0.02
P18419	Seminal vesicle secretory protein 4 OS=Mus musculus GN=Svs4 PE=1 SV=2 - [SVS4_MOUSE]	1.77	0.77	1.64	0.67	0.02
Q9CQW9	Interferon-induced transmembrane protein 3 OS=Mus musculus GN=Ifitm3 PE=1 SV=1 - [IFM3_MOUSE]	-0.36	-0.45	-0.19	-0.16	0.02
H3BKD4	Arf-GAP with SH3 domain, ANK repeat and PH domain-containing protein 1 OS=Mus musculus GN=Asap1 PE=2 SV=1 - [H3BKD4_MOUSE]	-0.77	-0.90	-0.22	-0.60	0.02
Q64332	Synapsin-2 OS=Mus musculus GN=Syn2 PE=1 SV=2 - [SYN2_MOUSE]	-0.33	-0.41	-0.82	-0.91	0.02
A2ALN0	C-C motif chemokine 22 OS=Mus musculus GN=Abcd1 PE=2 SV=1 - [A2ALN0_MOUSE]	-0.27	-0.43	-0.11	-0.27	0.02
P35822	Receptor-type tyrosine-protein phosphatase kappa OS=Mus musculus GN=Ptprk PE=1 SV=1 - [PTPRK_MOUSE]	-0.12	-0.17	-0.36	-0.24	0.02
P41183	B-cell lymphoma 6 protein homolog OS=Mus musculus GN=Bcl6 PE=1 SV=1 - [BCL6_MOUSE]	0.96	0.37	1.20	0.61	0.02
Q9R1A8	E3 ubiquitin-protein ligase RFWD2 OS=Mus musculus GN=Rfwd2 PE=1 SV=2 - [RFWD2_MOUSE]	0.43	0.16	0.56	0.29	0.02
Q9D3T1	Protein 4933436I01Rik OS=Mus musculus GN=4933436I01Rik PE=2 SV=1 - [Q9D3T1_MOUSE]	-0.87	-1.20	-0.33	-0.66	0.02
D3Z3U6	Activated CDC42 kinase 1 OS=Mus musculus GN=Tnk2 PE=2 SV=1 - [D3Z3U6_MOUSE]	-0.70	-0.29	-0.57	-0.26	0.02
B1AY85	Plexin domain-containing protein 2 OS=Mus musculus GN=Plxdc2 PE=2 SV=1 - [B1AY85_MOUSE]	-0.15	-0.31	-0.40	-0.56	0.02
Q8K1G2	Limbin OS=Mus musculus GN=Evc2 PE=1 SV=1 - [LBN_MOUSE]	0.41	0.15	0.44	0.22	0.02
P13516	Acyl-CoA desaturase 1 OS=Mus musculus GN=Scd1 PE=1 SV=2 - [ACOD1_MOUSE]	-0.27	-0.13	-0.22	-0.09	0.02

P97825	Hematological and neurological expressed 1 protein OS=Mus musculus GN=Hn1 PE=1 SV=3 - [HN1_MOUSE]	-0.20	-0.15	-0.37	-0.15	0.02
P01899	H-2 class I histocompatibility antigen, D-B alpha chain OS=Mus musculus GN=H2-D1 PE=1 SV=2 - [HA11_MOUSE]	0.34	0.22	0.15	0.12	0.02
Q62504-3	Isoform 3 of Msx2-interacting protein OS=Mus musculus GN=Spen - [MINT_MOUSE]	-0.29	-0.57	-0.27	-0.21	0.02
Q8BFW7-4	Isoform 4 of Lipoma-preferred partner homolog OS=Mus musculus GN=Lpp - [LPP_MOUSE]	0.37	0.77	0.24	0.64	0.02
P10852	4F2 cell-surface antigen heavy chain OS=Mus musculus GN=Slc3a2 PE=1 SV=1 - [4F2_MOUSE]	0.24	0.40	0.11	0.24	0.02
Q3THK3	General transcription factor IIF subunit 1 OS=Mus musculus GN=Gtf2f1 PE=1 SV=2 - [T2FA_MOUSE]	0.25	0.70	0.31	0.62	0.02
P61222	ATP-binding cassette sub-family E member 1 OS=Mus musculus GN=Abce1 PE=2 SV=1 - [ABCE1_MOUSE]	0.18	0.24	0.06	0.14	0.02
Q6ZWZ6	40S ribosomal protein S12 OS=Mus musculus GN=Rps12 PE=2 SV=1 - [Q6ZWZ6_MOUSE]	0.19	0.26	0.06	0.24	0.02
P00158	Cytochrome b OS=Mus musculus GN=Mt-Cyb PE=2 SV=1 - [CYB_MOUSE]	0.46	0.79	0.21	0.55	0.02
Q9JJZ6	Krueppel-like factor 13 OS=Mus musculus GN=Klf13 PE=2 SV=2 - [KLF13_MOUSE]	-0.29	-0.14	-0.26	-0.10	0.02
Q62420	Endophilin-A1 OS=Mus musculus GN=Sh3gl2 PE=1 SV=2 - [SH3G2_MOUSE]	0.16	0.29	0.08	0.21	0.02
Q05DV2	Dihydroxyacetone phosphate acyltransferase (Fragment) OS=Mus musculus GN=Gnpat PE=2 SV=1 - [Q05DV2_MOUSE]	-0.06	-0.17	-0.09	-0.19	0.02
Q9JIG7	Coiled-coil domain-containing protein 22 OS=Mus musculus GN=Ccdc22 PE=1 SV=1 - [CCD22_MOUSE]	0.09	0.07	0.20	0.12	0.02
B1AU76	Nuclear autoantigenic sperm protein OS=Mus musculus GN=Nasp PE=2 SV=1 - [B1AU76_MOUSE]	-0.06	-0.04	-0.04	-0.02	0.02

Q8VDG7	Platelet-activating factor acetylhydrolase 2, cytoplasmic OS=Mus musculus GN=Pafah2 PE=2 SV=2 - [PAFA2_MOUSE]	0.12	0.19	0.15	0.05	0.02
Q8C178	Acyl-coenzyme A oxidase OS=Mus musculus GN=Acox3 PE=2 SV=1 - [Q8C178_MOUSE]	0.75	0.32	0.72	0.29	0.02
P63330	Serine/threonine-protein phosphatase 2A catalytic subunit alpha isoform OS=Mus musculus GN=Ppp2ca PE=1 SV=1 - [PP2AA_MOUSE]	-0.10	-0.17	-0.28	-0.33	0.02
Q9WUQ2	Prolactin regulatory element-binding protein OS=Mus musculus GN=Preb PE=1 SV=1 - [PREB_MOUSE]	0.12	0.38	0.21	0.39	0.02
B1AUP0	Serine/threonine-protein kinase A-Raf (Fragment) OS=Mus musculus GN=Araf PE=2 SV=1 - [B1AUP0_MOUSE]	0.31	0.12	0.38	0.18	0.02
P58389	Serine/threonine-protein phosphatase 2A activator OS=Mus musculus GN=Ppp2r4 PE=1 SV=1 - [PTPA_MOUSE]	0.24	0.13	0.25	0.08	0.02
Q62417-7	Isoform 7 of Sorbin and SH3 domain-containing protein 1 OS=Mus musculus GN=Sorbs1 - [SRBS1_MOUSE]	0.07	0.23	0.18	0.28	0.02
O88351	Inhibitor of nuclear factor kappa-B kinase subunit beta OS=Mus musculus GN=Ikbkb PE=1 SV=1 - [IKKB_MOUSE]	0.34	0.63	0.25	0.74	0.02
D3YUG5	MKL/myocardin-like protein 1 (Fragment) OS=Mus musculus GN=Mkl1 PE=2 SV=1 - [D3YUG5_MOUSE]	-0.70	-0.54	-0.36	-0.20	0.02
Q91WS0	CDGSH iron-sulfur domain-containing protein 1 OS=Mus musculus GN=Cisd1 PE=1 SV=1 - [CISD1_MOUSE]	-0.07	-0.06	-0.16	-0.11	0.02
Q8CD10	Calcium uptake protein 2, mitochondrial OS=Mus musculus GN=Micu2 PE=1 SV=2 - [MICU2_MOUSE]	0.21	0.41	0.13	0.25	0.03
P62874	Guanine nucleotide-binding protein G(I)/G(S)/G(T) subunit beta-1 OS=Mus musculus GN=Gnb1 PE=1 SV=3 - [GBB1_MOUSE]	-0.09	-0.27	-0.14	-0.27	0.03

P19324	Serpin H1 OS=Mus musculus GN=Serpinh1 PE=1 SV=3 - [SERPH_MOUSE]	0.06	0.07	0.09	0.16	0.03
Q8K0U4	Heat shock 70 kDa protein 12A OS=Mus musculus GN=Hspa12a PE=1 SV=1 - [HS12A_MOUSE]	-0.37	-0.29	-0.16	-0.12	0.03
Q8CGP0	Histone H2B type 3-B OS=Mus musculus GN=Hist3h2bb PE=1 SV=3 - [H2B3B_MOUSE]	-0.08	-0.17	-0.23	-0.30	0.03
Q9D6U8	Protein FAM162A OS=Mus musculus GN=Fam162a PE=2 SV=1 - [F162A_MOUSE]	-0.23	-0.38	-0.13	-0.43	0.03
O35075	Down syndrome critical region protein 3 homolog OS=Mus musculus GN=Dscr3 PE=2 SV=1 - [DSCR3_MOUSE]	-0.54	-1.02	-0.28	-0.76	0.03
Q8C2L6	Transmembrane protein 161B OS=Mus musculus GN=Tmem161b PE=2 SV=1 - [T161B_MOUSE]	-1.19	-0.44	-1.48	-0.74	0.03
P00416	Cytochrome c oxidase subunit 3 OS=Mus musculus GN=Mtco3 PE=3 SV=1 - [COX3_MOUSE]	-0.41	-0.10	-0.45	-0.41	0.03
Q8K2I4	Beta-mannosidase OS=Mus musculus GN=Manba PE=2 SV=1 - [MANBA_MOUSE]	-0.28	-0.14	-0.48	-0.49	0.03
Q9DBB8	Trans-1,2-dihydrobenzene-1,2-diol dehydrogenase OS=Mus musculus GN=Dhdh PE=2 SV=1 - [DHDH_MOUSE]	-0.06	-0.08	-0.14	-0.18	0.03
Q9Z1A1	Protein Tfg OS=Mus musculus GN=Tfg PE=2 SV=1 - [Q9Z1A1_MOUSE]	-0.04	-0.07	-0.14	-0.08	0.03
Q9Z277	Tyrosine-protein kinase BAZ1B OS=Mus musculus GN=Baz1b PE=1 SV=2 - [BAZ1B_MOUSE]	0.07	0.20	0.16	0.28	0.03
Q99LC2	Cleavage stimulation factor subunit 1 OS=Mus musculus GN=Cstf1 PE=2 SV=1 - [CSTF1_MOUSE]	-0.31	-0.15	-0.55	-0.39	0.03
E9Q4T2	Protein Gm17511 OS=Mus musculus GN=Gm17511 PE=3 SV=1 - [E9Q4T2_MOUSE]	-0.72	-1.33	-0.36	-0.97	0.03
E9QKT1	Ubiquitin-protein ligase E3A OS=Mus musculus GN=Ube3a PE=2 SV=1 - [E9QKT1_MOUSE]	0.28	0.35	0.08	0.25	0.03

Q8BL48	RING finger protein unkempt homolog OS=Mus musculus GN=Unk PE=1 SV=1 - [UNK_MOUSE]	-0.77	-1.01	-0.28	-0.53	0.03
E9Q575	Caspase OS=Mus musculus GN=Casp12 PE=2 SV=1 - [E9Q575_MOUSE]	0.55	0.24	0.51	0.20	0.03
Q3U2P1	Protein transport protein Sec24A OS=Mus musculus GN=Sec24a PE=1 SV=1 - [SC24A_MOUSE]	0.39	0.12	0.43	0.24	0.03
Q9WVE8	Protein kinase C and casein kinase substrate in neurons protein 2 OS=Mus musculus GN=Pacsin2 PE=1 SV=1 - [PACN2_MOUSE]	0.07	0.18	0.08	0.19	0.03
Q9CQE1	Protein NipSnap homolog 3B OS=Mus musculus GN=Nipsnap3b PE=1 SV=1 - [NPS3B_MOUSE]	-0.32	-0.16	-0.58	-0.36	0.03
D3Z7C6	Prostaglandin E synthase 3 OS=Mus musculus GN=Ptges3 PE=2 SV=1 - [D3Z7C6_MOUSE]	0.11	0.10	0.04	0.05	0.03
A2AIW0-6	Isoform 6 of Serologically defined colon cancer antigen 3 homolog OS=Mus musculus GN=Sdccag3 - [SDCG3_MOUSE]	-0.90	-2.32	-1.13	-2.55	0.03
Q6PEV3	WAS/WASL-interacting protein family member 2 OS=Mus musculus GN=Wipf2 PE=2 SV=1 [WIPF2_MOUSE]	-0.23	-0.17	-0.51	-0.32	0.03
H7BWZ3	Actin-related protein 2/3 complex subunit 3 OS=Mus musculus GN=Arpc3 PE=2 SV=1 [H7BWZ3_MOUSE]	0.11	0.31	0.15	0.16	0.03
G5E8S8	MAGUK p55 subfamily member 7 OS=Mus musculus GN=Mpp7 PE=4 SV=1 - [G5E8S8_MOUSE]	-0.12	-0.38	-0.17	-0.25	0.03
Q0VBM2	Protein FAM83B OS=Mus musculus GN=Fam83b PE=1 SV=1 - [FA83B_MOUSE]	-0.06	-0.14	-0.06	-0.15	0.03
P69744	Transient receptor potential cation channel subfamily V member 5 OS=Mus musculus GN=Trpv5 PE=1 SV=2 - [TRPV5_MOUSE]	0.62	1.28	0.39	1.06	0.03

Q8BXK9	Chloride intracellular channel protein 5 OS=Mus musculus GN=Clic5 PE=1 SV=1 - [CLIC5_MOUSE]	0.03	0.07	0.06	0.11	0.03
P97393	Rho GTPase-activating protein 5 OS=Mus musculus GN=Arhgap5 PE=2 SV=2 - [RHG05_MOUSE]	0.55	0.25	0.43	0.18	0.03
Q9D5V2	Kelch-like protein 10 OS=Mus musculus GN=Klhl10 PE=1 SV=1 - [KLH10_MOUSE]	0.64	1.48	0.53	1.37	0.03
Q3UKX1	Protein arginine N-methyltransferase 2 OS=Mus musculus GN=Prmt2 PE=2 SV=1 - [Q3UKX1_MOUSE]	0.21	0.10	0.37	0.26	0.03
P70195	Proteasome subunit beta type-7 OS=Mus musculus GN=Psmb7 PE=1 SV=1 - [PSB7_MOUSE]	0.05	0.17	0.12	0.19	0.03
Q9JHI7	Exosome complex component RRP45 OS=Mus musculus GN=Exosc9 PE=2 SV=1 - [EXOS9_MOUSE]	-0.24	-0.10	-0.24	-0.10	0.03
Q8C6H0	Galectin-8 OS=Mus musculus GN=Lgals8 PE=2 SV=1 - [Q8C6H0_MOUSE]	0.13	0.17	0.34	0.38	0.03
Q62433	Protein NDRG1 OS=Mus musculus GN=Ndrg1 PE=1 SV=1 - [NDRG1_MOUSE]	0.13	0.37	0.15	0.23	0.03
Q64314-2	Isoform Short of Hematopoietic progenitor cell antigen CD34 OS=Mus musculus GN=Cd34 - [CD34_MOUSE]	-0.48	-0.20	-0.80	-0.53	0.03
Q8CI51	PDZ and LIM domain protein 5 OS=Mus musculus GN=Pdlim5 PE=1 SV=4 - [PDLI5_MOUSE]	-0.11	-0.33	-0.18	-0.35	0.03
Q8K0Q5	Rho GTPase-activating protein 18 OS=Mus musculus GN=Arhgap18 PE=2 SV=1 - [RHG18_MOUSE]	0.07	0.08	0.17	0.19	0.03
Q3TVI8	Pre-B-cell leukemia transcription factor-interacting protein 1 OS=Mus musculus GN=Pbxip1 PE=1 SV=2 - [PBIP1_MOUSE]	0.14	0.03	0.13	0.14	0.03
F8VQM4	Oxysterol-binding protein OS=Mus musculus GN=Osbpl7 PE=2 SV=1 - [F8VQM4_MOUSE]	0.31	0.54	0.14	0.37	0.03
Q80WW9	DDRGK domain-containing protein 1 OS=Mus musculus GN=Ddrgk1 PE=1 SV=2 - [DDRGK_MOUSE]	0.39	0.51	0.14	0.26	0.03

P54923	[Protein ADP-ribosylarginine] hydrolase OS=Mus musculus GN=Adprh PE=2 SV=1 - [ADPRH_MOUSE]	0.10	0.06	0.20	0.15	0.03
D3Z723	Nuclear transcription factor Y subunit beta (Fragment) OS=Mus musculus GN=Nfyb PE=2 SV=1 - [D3Z723_MOUSE]	0.36	0.16	0.63	0.43	0.03
Q3KNY0	Immunoglobulin-like and fibronectin type III domain-containing protein 1 OS=Mus musculus GN=Igfn1 PE=1 SV=3 - [IGFN1_MOUSE]	0.39	0.16	0.40	0.16	0.03
Q8C8I3	Eukaryotic translation initiation factor 2D OS=Mus musculus GN=Eif2d PE=2 SV=1 - [Q8C8I3_MOUSE]	0.60	0.39	0.37	0.15	0.03
Q9WV91	Prostaglandin F2 receptor negative regulator OS=Mus musculus GN=Ptgfrn PE=1 SV=2 - [FPRP_MOUSE]	-0.10	-0.05	-0.10	-0.18	0.03
F8WI14	Extracellular matrix protein 1 OS=Mus musculus GN=Ecm1 PE=2 SV=1 - [F8WI14_MOUSE]	-0.70	-0.26	-0.39	-0.30	0.03
D3YTK6	NAD-dependent protein deacetylase sirtuin-3 (Fragment) OS=Mus musculus GN=Sirt3 PE=2 SV=2 - [D3YTK6_MOUSE]	-0.70	-0.72	-0.28	-0.30	0.03
Q8K273	Membrane magnesium transporter 1 OS=Mus musculus GN=Mmgt1 PE=2 SV=1 - [MMGT1_MOUSE]	0.41	0.25	0.25	0.10	0.03
Q91Z53	Glyoxylate reductase/hydroxypyruvate reductase OS=Mus musculus GN=Grhpr PE=1 SV=1 [GRHPR_MOUSE]	-0.35	-0.33	-0.11	-0.16	0.03
Q80X80	C2 domain-containing protein 2-like OS=Mus musculus GN=C2cd2l PE=1 SV=3 - [C2C2L_MOUSE]	-0.40	-0.13	-0.29	-0.18	0.03
Q99J27	Acetyl-coenzyme A transporter 1 OS=Mus musculus GN=Slc33a1 PE=1 SV=1 - [ACATN_MOUSE]	0.29	0.09	0.36	0.22	0.03
E9Q4K7	Protein Kif13b OS=Mus musculus GN=Kif13b PE=2 SV=1 - [E9Q4K7_MOUSE]	0.10	0.04	0.04	0.09	0.03
Q8BHJ5	F-box-like/WD repeat-containing protein TBL1XR1 OS=Mus musculus GN=Tbl1xr1 PE=2 SV=1 - [TBL1R_MOUSE]	-0.45	-0.22	-0.82	-0.59	0.03

Q9EPK2	Protein XRP2 OS=Mus musculus GN=Rp2 PE=1 SV=3 - [XRP2_MOUSE]	-0.09	-0.03	-0.12	-0.12	0.03
Q8R3W2	MCG6979, isoform CRA_a OS=Mus musculus GN=0610009B22Rik PE=2 SV=1 - [Q8R3W2_MOUSE]	-0.99	-1.11	-0.39	-0.47	0.03
Q80YA7	Dipeptidyl peptidase 8 OS=Mus musculus GN=Dpp8 PE=1 SV=1 - [DPP8_MOUSE]	-0.37	-0.27	-0.20	-0.10	0.03
Q6PFY1	FCH and double SH3 domains protein 1 OS=Mus musculus GN=Fchsd1 PE=2 SV=1 - [FCSD1_MOUSE]	0.90	0.54	0.58	0.22	0.03
F6RGK4	MMS19 nucleotide excision repair protein homolog (Fragment) OS=Mus musculus GN=Mms19 PE=2 SV=1 - [F6RGK4_MOUSE]	-0.22	-0.12	-0.38	-0.19	0.03
P29699	Alpha-2-HS-glycoprotein OS=Mus musculus GN=Ahsg PE=1 SV=1 - [FETUA_MOUSE]	-0.04	-0.06	-0.09	-0.14	0.03
O54951	Semaphorin-6B OS=Mus musculus GN=Sema6b PE=2 SV=1 - [SEM6B_MOUSE]	-0.20	-0.17	-0.09	-0.06	0.03
P22366	Myeloid differentiation primary response protein MyD88 OS=Mus musculus GN=Myd88 PE=1 SV=3 - [MYD88_MOUSE]	-0.05	-0.08	-0.15	-0.17	0.03
P14428	H-2 class I histocompatibility antigen, K-Q alpha chain (Fragment) OS=Mus musculus GN=H2-K1 PE=2 SV=1 - [HA1Q_MOUSE]	-0.18	-0.45	-0.44	-0.71	0.03
Q9CV28-2	Isoform 2 of Protein FAM188A OS=Mus musculus GN=Fam188a - [F188A_MOUSE]	-0.45	-0.91	-0.25	-0.71	0.03
P0C0A3	Charged multivesicular body protein 6 OS=Mus musculus GN=Chmp6 PE=2 SV=2 - [CHMP6_MOUSE]	-0.25	-0.45	-0.12	-0.31	0.03
D3Z7U6	Calpain-9 (Fragment) OS=Mus musculus GN=Capn9 PE=2 SV=1 - [D3Z7U6_MOUSE]	1.49	0.53	1.88	0.93	0.03
Q9D939	Sulfotransferase 1C2 OS=Mus musculus GN=Sult1c2 PE=1 SV=1 - [ST1C2_MOUSE]	-0.29	-0.12	-0.28	-0.11	0.03

P63166	Small ubiquitin-related modifier 1 OS=Mus musculus GN=Sumo1 PE=1 SV=1 - [SUMO1_MOUSE]	-0.28	-0.12	-0.38	-0.16	0.03
Q4VA53	Sister chromatid cohesion protein PDS5 homolog B OS=Mus musculus GN=Pds5b PE=1 SV=1 - [PDS5B_MOUSE]	0.37	0.50	0.18	0.19	0.03
B2RVL6	Zinc finger CCHC domain-containing protein 24 OS=Mus musculus GN=Zcchc24 PE=2 SV=1 - [ZCH24_MOUSE]	-0.61	-1.40	-0.46	-1.25	0.03
D3YYD5	Vacuolar protein sorting associated protein 29 (Fragment) OS=Mus musculus GN=Vps29 PE=2 SV=1 - [D3YYD5_MOUSE]	-0.25	-0.10	-0.34	-0.17	0.03
P97470	Serine/threonine-protein phosphatase 4 catalytic subunit OS=Mus musculus GN=Ppp4c PE=1 SV=2 - [PP4C_MOUSE]	-0.24	-0.10	-0.40	-0.26	0.03
A2RTH5	Leucine carboxyl methyltransferase 1 OS=Mus musculus GN=Lcmt1 PE=2 SV=1 - [A2RTH5_MOUSE]	0.33	0.13	0.52	0.32	0.03
Q8BGA9	Mitochondrial inner membrane protein OXA1L OS=Mus musculus GN=Oxa1l PE=2 SV=1 - [OXA1L_MOUSE]	-0.39	-0.60	-0.15	-0.36	0.03
D6RCW3	Cholinephosphotransferase 1 OS=Mus musculus GN=Chpt1 PE=2 SV=1 - [D6RCW3_MOUSE]	0.80	0.31	1.28	0.80	0.03
Q8BWR8	Rhophilin-2 OS=Mus musculus GN=Rhpn2 PE=1 SV=2 - [RHPN2_MOUSE]	0.17	0.07	0.29	0.18	0.03
D3YYW8	RNA-binding protein 42 OS=Mus musculus GN=Rbm42 PE=2 SV=1 - [D3YYW8_MOUSE]	0.08	0.13	0.03	0.08	0.03
Q571I4	Tyrosine-protein kinase SgK223 OS=Mus musculus GN=Sgk223 PE=2 SV=2 - [SG223_MOUSE]	0.16	0.21	0.05	0.21	0.03
Q8C0E2	Vacuolar protein sorting associated protein 26B OS=Mus musculus GN=Vps26b PE=1 SV=1 - [VP26B_MOUSE]	-0.17	-0.11	-0.13	-0.04	0.03
P28659-2	Isoform 2 of CUGBP Elav-like family member 1 OS=Mus musculus GN=Celf1 - [CELF1_MOUSE]	-0.06	-0.21	-0.09	-0.17	0.03

E9QAN4	Kinesin-like protein KIF1A OS=Mus musculus GN=Kif1a PE=2 SV=1 - [E9QAN4_MOUSE]	0.55	0.84	0.21	0.49	0.03
P12265	Beta-glucuronidase OS=Mus musculus GN=Gusb PE=2 SV=2 - [BGLR_MOUSE]	-0.18	-0.23	-0.09	-0.09	0.03
E9QP59	Inner nuclear membrane protein Man1 OS=Mus musculus GN=Lemd3 PE=2 SV=1 - [E9QP59_MOUSE]	0.30	0.31	0.17	0.09	0.03
F6TLV3	Fibronectin type-III domain-containing protein 3A (Fragment) OS=Mus musculus GN=Fndc3a PE=4 SV=1 - [F6TLV3_MOUSE]	0.40	0.41	0.09	0.48	0.03
Q8BFR4	N-acetylglucosamine-6-sulfatase OS=Mus musculus GN=Gns PE=2 SV=1 - [GNS_MOUSE]	-0.17	-0.47	-0.47	-0.72	0.03
P97431	Interferon regulatory factor 6 OS=Mus musculus GN=Irf6 PE=1 SV=2 - [IRF6_MOUSE]	0.28	0.40	0.11	0.20	0.03
P70399	Tumor suppressor p53-binding protein 1 OS=Mus musculus GN=Tp53bp1 PE=1 SV=2 - [TP53B_MOUSE]	0.08	0.27	0.17	0.14	0.03
E9Q740	Signal recognition particle subunit SRP72 OS=Mus musculus GN=Srp72 PE=2 SV=1 - [E9Q740_MOUSE]	0.14	0.20	0.11	0.05	0.03
Q64433	10 kDa heat shock protein, mitochondrial OS=Mus musculus GN=Hspe1 PE=1 SV=2 - [CH10_MOUSE]	-0.19	-0.07	-0.27	-0.15	0.03
Q9CRB6	Tubulin polymerization-promoting protein family member 3 OS=Mus musculus GN=Tppp3 PE=1 SV=1 - [TPPP3_MOUSE]	-0.11	-0.07	-0.23	-0.17	0.03
Q9Z0J1	Reversion-inducing cysteine-rich protein with Kazal motifs OS=Mus musculus GN=Reck PE=2 SV=2 - [RECK_MOUSE]	-0.24	-0.22	-0.58	-0.57	0.03
Q10470	Beta-1,4-mannosyl-glycoprotein 4-beta-N-acetylglucosaminyltransferase OS=Mus musculus GN=Mgat3 PE=1 SV=2 - [MGAT3_MOUSE]	0.30	0.15	0.37	0.12	0.03
Q9WTP6-2	Isoform 2 of Adenylate kinase 2, mitochondrial OS=Mus musculus GN=Ak2 - [KAD2_MOUSE]	0.19	0.28	0.06	0.21	0.03

P03921	NADH-ubiquinone oxidoreductase chain 5 OS=Mus musculus GN=Mtnd5 PE=3 SV=2 - [NU5M_MOUSE]	-0.21	-0.18	-0.44	-0.51	0.03
Q8BGB8	Ubiquinone biosynthesis protein COQ4 homolog, mitochondrial OS=Mus musculus GN=Coq4 PE=2 SV=1 - [COQ4_MOUSE]	0.33	0.15	0.58	0.40	0.03
Q8CCJ3	E3 UFM1-protein ligase 1 OS=Mus musculus GN=Ufl1 PE=1 SV=2 - [UFL1_MOUSE]	0.30	0.22	0.27	0.06	0.03
Q9CY16	28S ribosomal protein S28, mitochondrial OS=Mus musculus GN=Mrps28 PE=2 SV=1 - [RT28_MOUSE]	-0.09	-0.13	-0.29	-0.20	0.03
Q99KK2	N-acylneuraminate cytidylyltransferase OS=Mus musculus GN=Cmas PE=1 SV=2 [NEUA_MOUSE]	0.09	0.04	0.10	0.03	0.03
Q9R1T2	SUMO-activating enzyme subunit 1 OS=Mus musculus GN=Sae1 PE=2 SV=1 - [SAE1_MOUSE]	0.33	0.22	0.31	0.07	0.03
P80315	T-complex protein 1 subunit delta OS=Mus musculus GN=Cct4 PE=1 SV=3 - [TCPD_MOUSE]	0.03	0.09	0.10	0.14	0.03
E9Q1Q0	Protein Slc4a1ap OS=Mus musculus GN=Slc4a1ap PE=2 SV=1 - [E9Q1Q0_MOUSE]	1.76	1.46	0.82	0.52	0.03
Q8BUV3	Gephyrin OS=Mus musculus GN=Gphn PE=1 SV=2 - [GEPH_MOUSE]	0.16	0.36	0.11	0.27	0.03
Q8R4P4	Transmembrane channel-like protein 2 OS=Mus musculus GN=Tmc2 PE=2 SV=1 [TMC2_MOUSE]	-0.09	-0.09	-0.04	-0.04	0.03
D3Z5J3	Sorbin and SH3 domain-containing protein 1 OS=Mus musculus GN=Sorbs1 PE=2 SV=1 - [D3Z5J3_MOUSE]	0.09	0.18	0.10	0.26	0.03
E9PZ36	Protein Pkhd1 OS=Mus musculus GN=Pkhd1 PE=4 SV=1 - [E9PZ36_MOUSE]	-0.24	-0.68	-0.43	-0.87	0.03
P31725	Protein S100-A9 OS=Mus musculus GN=S100a9 PE=1 SV=3 - [S10A9_MOUSE]	0.04	0.05	0.09	0.11	0.03
Q9R0P9	Ubiquitin carboxyl-terminal hydrolase isozyme L1 OS=Mus musculus GN=Uchl1 PE=1 SV=1 - [UCHL1_MOUSE]	-0.22	-0.11	-0.41	-0.28	0.03

Q9DAI2	Rab-like protein 5 OS=Mus musculus GN=Rabl5 PE=2 SV=1 [RABL5_MOUSE]	-0.18	-0.06	-0.25	-0.14	0.03
Q8BWM0	Prostaglandin E synthase 2 OS=Mus musculus GN=Ptges2 PE=1 SV=3 - [PGES2_MOUSE]	0.22	0.45	0.24	0.63	0.03
Q9CX80	Cytoglobin OS=Mus musculus GN=Cygb PE=2 SV=1 - [CYGB_MOUSE]	0.18	0.13	0.42	0.27	0.03
Q8BHC9	Alpha-(1,3)-fucosyltransferase 11 OS=Mus musculus GN=Fut11 PE=2 SV=1 - [FUT11_MOUSE]	-0.32	-0.08	-0.18	-0.27	0.03
Q9CPT0	Apoptosis facilitator Bcl-2-like protein 14 OS=Mus musculus GN=Bcl2l14 PE=2 SV=1 - [B2L14_MOUSE]	0.03	0.18	0.15	0.13	0.03
Q7TT52	Protein phosphatase 1 regulatory subunit 14D OS=Mus musculus GN=Ppp1r14d PE=2 SV=2 - [PP14D_MOUSE]	0.17	0.07	0.25	0.12	0.03
E9QP46	Nesprin-2 OS=Mus musculus GN=Syne2 PE=2 SV=1 - [E9QP46_MOUSE]	0.38	0.15	0.45	0.18	0.03
P15208	Insulin receptor OS=Mus musculus GN=Insr PE=1 SV=2 - [INSR_MOUSE]	-0.19	-0.42	-0.13	-0.36	0.03
Q8VD65	Phosphoinositide 3-kinase regulatory subunit 4 OS=Mus musculus GN=Pik3r4 PE=1 SV=3 - [PI3R4_MOUSE]	0.41	0.14	0.53	0.26	0.03
Q8BLN6	Protein unc-80 homolog OS=Mus musculus GN=Unc80 PE=1 SV=2 - [UNC80_MOUSE]	0.15	0.32	0.09	0.24	0.03
Q6DFV3	Rho GTPase-activating protein 21 OS=Mus musculus GN=Arhgap21 PE=1 SV=1 - [RHG21_MOUSE]	-0.08	-0.38	-0.30	-0.37	0.03
Q91V01	Lysophospholipid acyltransferase 5 OS=Mus musculus GN=Lpcat3 PE=1 SV=1 - [MBOA5_MOUSE]	-0.12	-0.04	-0.18	-0.11	0.03
Q9Z0Z7	Krueppel-like factor 5 OS=Mus musculus GN=Klf5 PE=2 SV=2 - [KLF5_MOUSE]	-0.17	-0.07	-0.28	-0.18	0.03
Q99LB2	Dehydrogenase/reductase SDR family member 4 OS=Mus musculus GN=Dhrs4 PE=2 SV=3 - [DHRS4_MOUSE]	-0.17	-0.35	-0.13	-0.40	0.03

Q9QUR6	Prolyl endopeptidase OS=Mus musculus GN=Prep PE=2 SV=1 - [PPCE_MOUSE]	0.13	0.17	0.03	0.13	0.03
Q9CQU1	Microfibrillar-associated protein 1 OS=Mus musculus GN=Mfap1 PE=1 SV=1 - [MFAP1_MOUSE]	0.12	0.18	0.38	0.38	0.03
B1AXN9	Ribosomal protein S6 kinase alpha-3 OS=Mus musculus GN=Rps6ka3 PE=2 SV=1 - [B1AXN9_MOUSE]	0.23	0.06	0.20	0.12	0.03
O08919	Numb-like protein OS=Mus musculus GN=Numbl PE=1 SV=3 - [NUMBL_MOUSE]	-0.19	-0.42	-0.13	-0.36	0.03
Q9QZC7-2	Isoform 2 of Pleckstrin homology domain-containing family B member 2 OS=Mus musculus GN=Plekhb2 [PKHB2_MOUSE]	0.10	0.29	0.16	0.34	0.03
Q8R0Y6	Cytosolic 10-formyltetrahydrofolate dehydrogenase OS=Mus musculus GN=Aldh1l1 PE=2 SV=1 - [AL1L1_MOUSE]	0.05	0.08	0.02	0.07	0.03
Q9CZC8	Secernin-1 OS=Mus musculus GN=Scrn1 PE=1 SV=1 - [SCRN1_MOUSE]	-0.56	-0.44	-0.28	-0.15	0.03
P50516	V-type proton ATPase catalytic subunit A OS=Mus musculus GN=Atp6v1a PE=1 SV=2 - [VATA_MOUSE]	-0.07	-0.15	-0.15	-0.26	0.03
P62307	Small nuclear ribonucleoprotein F OS=Mus musculus GN=Snrpf PE=2 SV=1 - [RUXF_MOUSE]	-0.56	-0.62	-0.27	-0.20	0.03
Q9EQH3	Vacuolar protein sorting associated protein 35 OS=Mus musculus GN=Vps35 PE=1 SV=1 - [VPS35_MOUSE]	-0.07	-0.14	-0.04	-0.11	0.03
P50429	Arylsulfatase B OS=Mus musculus GN=Arsb PE=2 SV=3 - [ARSB_MOUSE]	-0.42	-0.43	-0.16	-0.17	0.03
P59672	Ankyrin repeat and SAM domain-containing protein 1A OS=Mus musculus GN=Anks1a PE=1 SV=3 - [ANS1A_MOUSE]	0.07	0.35	0.28	0.37	0.03
Q8VEE0	Ribulose-phosphate 3-epimerase OS=Mus musculus GN=Rpe PE=2 SV=1 - [RPE_MOUSE]	0.05	0.04	0.13	0.10	0.03
E9Q8U3	Protein Gcom1 OS=Mus musculus GN=Gcom1 PE=2 SV=1 - [E9Q8U3_MOUSE]	-0.28	-0.29	-0.13	-0.10	0.03

Q9R0U9	Protein Tex21 OS=Mus musculus GN=Tex21 PE=2 SV=1 - [Q9R0U9_MOUSE]	-0.44	-0.26	-0.28	-0.10	0.03
Q9D0M0	Exosome complex exonuclease RRP42 OS=Mus musculus GN=Exosc7 PE=2 SV=2 - [EXOS7_MOUSE]	-0.37	-0.17	-0.27	-0.59	0.03
Q8CG70	Prolyl 3-hydroxylase 3 OS=Mus musculus GN=Leprel2 PE=2 SV=1 - [P3H3_MOUSE]	-0.26	-0.15	-0.54	-0.43	0.03
P70460	Vasodilator-stimulated phosphoprotein OS=Mus musculus GN=Vasp PE=1 SV=4 - [VASP_MOUSE]	0.13	0.25	0.06	0.21	0.03
F8VPU6	Ubiquitin carboxyl-terminal hydrolase OS=Mus musculus GN=Usp9y PE=3 SV=1 - [F8VPU6_MOUSE]	-1.74	-4.09	-1.32	-3.67	0.03
P62075	Mitochondrial import inner membrane translocase subunit Tim13 OS=Mus musculus GN=Timm13 PE=1 SV=1 - [TIM13_MOUSE]	0.14	0.16	0.03	0.14	0.03
Q9D0D3	Poly(A) RNA polymerase, mitochondrial OS=Mus musculus GN=Mtpap PE=2 SV=1 - [PAPD1_MOUSE]	0.49	0.22	0.36	0.14	0.03
Q8CEC0-3	Isoform 3 of Nuclear pore complex protein Nup88 OS=Mus musculus GN=Nup88 - [NUP88_MOUSE]	0.33	0.57	0.13	0.38	0.03
P30933	Seminal vesicle secretory protein 5 OS=Mus musculus GN=Svs5 PE=2 SV=1 - [SVS5_MOUSE]	3.05	0.96	2.92	1.40	0.03
Q91ZT5-2	Isoform 2 of FYVE, RhoGEF and PH domain-containing protein 4 OS=Mus musculus GN=Fgd4 - [FGD4_MOUSE]	0.28	0.57	0.26	0.74	0.03
P61087	Ubiquitin-conjugating enzyme E2 K OS=Mus musculus GN=Ube2k PE=1 SV=3 - [UBE2K_MOUSE]	0.14	0.12	0.23	0.36	0.03
P11032-2	Isoform HF2 of Granzyme A OS=Mus musculus GN=Gzma - [GRAA_MOUSE]	-0.37	-0.17	-0.71	-0.51	0.03
Q8C1E7	Transmembrane protein 120A OS=Mus musculus GN=Tmem120a PE=2 SV=1 - [T120A_MOUSE]	-0.53	-0.43	-0.24	-0.15	0.03

Q8BND5-2	Isoform 2 of Sulfhydryl oxidase 1 OS=Mus musculus GN=Qsox1 - [QSOX1_MOUSE]	-0.22	-0.42	-0.11	-0.27	0.03
Q8BNA6-3	Isoform 3 of Protocadherin Fat 3 OS=Mus musculus GN=Fat3 - [FAT3_MOUSE]	0.30	0.14	0.56	0.39	0.03
B1AUN2	MCG14442 OS=Mus musculus GN=Eif2b3 PE=4 SV=1 - [B1AUN2_MOUSE]	0.10	0.20	0.05	0.14	0.03
P34914	Bifunctional epoxide hydrolase 2 OS=Mus musculus GN=Ephx2 PE=1 SV=2 - [HYES_MOUSE]	-0.53	-0.34	-0.43	-0.11	0.03
E9Q616	Protein Ahnak OS=Mus musculus GN=Ahnak PE=2 SV=1 - [E9Q616_MOUSE]	-0.16	-0.06	-0.23	-0.12	0.03
O55100-2	Isoform 1B of Synaptogyrin-1 OS=Mus musculus GN=Syngr1 - [SNG1_MOUSE]	-0.44	-0.90	-0.23	-0.69	0.03
Q3TDT0	Tripartite motif-containing protein 3 OS=Mus musculus GN=Trim3 PE=2 SV=1 [Q3TDT0_MOUSE]	0.06	0.01	0.08	0.07	0.03
Q3UMR5	Calcium uniporter protein, mitochondrial OS=Mus musculus GN=Mcu PE=1 SV=2 - [MCU_MOUSE]	-0.15	-0.23	-0.07	-0.28	0.03
E9QQ10	A-kinase anchor protein 9 OS=Mus musculus GN=Akap9 PE=2 SV=1 - [E9QQ10_MOUSE]	0.10	0.23	0.14	0.08	0.03
Q924C1	Exportin-5 OS=Mus musculus GN=Xpo5 PE=2 SV=1 - [XPO5_MOUSE]	0.47	0.15	0.65	0.35	0.03
P68433	Histone H3.1 OS=Mus musculus GN=Hist1h3a PE=1 SV=2 - [H31_MOUSE]	0.28	0.43	0.12	0.19	0.03
Q91YD3	mRNA-decapping enzyme 1A OS=Mus musculus GN=Dcp1a PE=1 SV=1 - [DCP1A_MOUSE]	-0.16	-0.40	-0.14	-0.38	0.03
Q9WTI7-3	Isoform 3 of Unconventional myosin-Ic OS=Mus musculus GN=Myo1c - [MYO1C_MOUSE]	-0.03	-0.10	-0.04	-0.09	0.03
E9QMK6	Uncharacterized protein OS=Mus musculus GN=Ptchd4 PE=4 SV=1 - [E9QMK6_MOUSE]	-1.04	-1.78	-0.40	-1.15	0.03
P19426-2	Isoform 2 of Negative elongation factor E OS=Mus musculus GN=Nelfe - [NELFE_MOUSE]	0.16	0.20	0.15	0.04	0.03
Q9CQ25	Mitotic-spindle organizing protein 2 OS=Mus musculus GN=Mzt2 PE=1 SV=1 - [MZT2_MOUSE]	1.13	0.38	1.62	0.87	0.03

F7AC58	Piezo-type mechanosensitive ion channel component 1 (Fragment) OS=Mus musculus GN=Piezo1 PE=4 SV=1 - [F7AC58_MOUSE]	0.43	0.15	0.48	0.21	0.03
Q8BKN5-3	Isoform 3 of Gamma-tubulin complex component 5 OS=Mus musculus GN=Tubgcp5 - [GCP5_MOUSE]	-0.22	-0.23	-0.59	-0.60	0.03
Q8BV13	COP9 signalosome complex subunit 7b OS=Mus musculus GN=Cops7b PE=1 SV=1 - [CSN7B_MOUSE]	-0.35	-0.11	-0.42	-0.21	0.03
Q924C5	Alpha-protein kinase 3 OS=Mus musculus GN=Alpk3 PE=1 SV=1 - [ALPK3_MOUSE]	0.56	1.52	0.64	1.60	0.03
O88531	Palmitoyl-protein thioesterase 1 OS=Mus musculus GN=Ppt1 PE=2 SV=2 - [PPT1_MOUSE]	-0.35	-0.29	-0.17	-0.09	0.03
Q80U95	Ubiquitin-protein ligase E3C OS=Mus musculus GN=Ube3c PE=2 SV=2 - [UBE3C_MOUSE]	0.56	0.53	0.23	0.19	0.03
O88958	Glucosamine-6-phosphate isomerase 1 OS=Mus musculus GN=Gnpda1 PE=2 SV=3 - [GNPI1_MOUSE]	-0.44	-0.12	-0.56	-0.34	0.03
P11438	Lysosome-associated membrane glycoprotein 1 OS=Mus musculus GN=Lamp1 PE=1 SV=2 - [LAMP1_MOUSE]	-0.29	-0.17	-0.17	-0.07	0.03
Q8K274	Ketosamine-3-kinase OS=Mus musculus GN=Fn3krp PE=2 SV=2 - [KT3K_MOUSE]	-0.27	-0.10	-0.39	-0.20	0.03
Q8BVY0	Protein Rsl1d1 OS=Mus musculus GN=Rsl1d1 PE=2 SV=1 - [Q8BVY0_MOUSE]	0.50	0.32	0.44	0.10	0.03
Q60865	Caprin-1 OS=Mus musculus GN=Caprin1 PE=1 SV=2 - [CAPR1_MOUSE]	0.14	0.22	0.05	0.12	0.03
Q9R0M4	Podocalyxin OS=Mus musculus GN=Podxl PE=1 SV=2 - [PODXL_MOUSE]	-0.19	-0.41	-0.11	-0.28	0.03
A2AC80	Metalloendopeptidase homolog PEX (Fragment) OS=Mus musculus GN=Phex PE=2 SV=1 - [A2AC80_MOUSE]	0.48	1.27	0.48	1.27	0.03

P61458	Pterin-4-alpha-carbinolamine dehydratase OS=Mus musculus GN=Pcbd1 PE=1 SV=2 - [PHS_MOUSE]	-0.17	-0.39	-0.12	-0.21	0.03
Q9JHI0	Matrix metalloproteinase-19 OS=Mus musculus GN=Mmp19 PE=2 SV=1 - [MMP19_MOUSE]	0.16	0.44	0.19	0.47	0.03
P97863-3	Isoform 3 of Nuclear factor 1 B-type OS=Mus musculus GN=Nfib - [NFIB_MOUSE]	-0.05	-0.09	-0.16	-0.20	0.03
Q9D1J1	Adaptin ear-binding coat-associated protein 2 OS=Mus musculus GN=Necap2 PE=1 SV=1 - [NECP2_MOUSE]	-0.13	-0.08	-0.06	-0.03	0.03
Q923S9	Ras-related protein Rab-30 OS=Mus musculus GN=Rab30 PE=2 SV=1 - [RAB30_MOUSE]	0.11	0.32	0.23	0.45	0.03
P61924	Coatomer subunit zeta-1 OS=Mus musculus GN=Copz1 PE=2 SV=1 - [COPZ1_MOUSE]	0.04	0.04	0.04	0.09	0.03
Q9DBE8	Alpha-1,3/1,6-mannosyltransferase ALG2 OS=Mus musculus GN=Alg2 PE=2 SV=2 - [ALG2_MOUSE]	-0.04	-0.15	-0.15	-0.07	0.03
P46938	Yorkie homolog OS=Mus musculus GN=Yap1 PE=1 SV=2 - [YAP1_MOUSE]	-0.15	-0.17	-0.07	-0.28	0.03
Q3TLS3	GDP-D-glucose phosphorylase 1 OS=Mus musculus GN=Gdpgp1 PE=2 SV=2 - [GDPP1_MOUSE]	-0.38	-0.08	-0.45	-0.35	0.03
Q99M07	Cytochrome c oxidase assembly factor 5 OS=Mus musculus GN=Coa5 PE=2 SV=1 - [COA5_MOUSE]	0.43	0.59	0.11	0.59	0.03
P21784	V(D)J recombination-activating protein 2 OS=Mus musculus GN=Rag2 PE=1 SV=2 - [RAG2_MOUSE]	-0.10	-0.25	-0.09	-0.25	0.03
P63028	Translationally-controlled tumor protein OS=Mus musculus GN=Tpt1 PE=1 SV=1 - [TCTP_MOUSE]	-0.08	-0.09	-0.08	-0.20	0.03
E9Q3Y4	Lipopolysaccharide-responsive and beige-like anchor protein OS=Mus musculus GN=Lrba PE=2 SV=1 - [E9Q3Y4_MOUSE]	0.34	0.08	0.41	0.25	0.03
O88895	Histone deacetylase 3 OS=Mus musculus GN=Hdac3 PE=1 SV=1 - [HDAC3_MOUSE]	0.12	0.19	0.24	0.42	0.03

Q9QYJ3	DnaJ homolog subfamily B member 1 OS=Mus musculus GN=Dnajb1 PE=2 SV=3 - [DNJB1_MOUSE]	0.30	0.17	0.15	0.08	0.03
Q8BU25	Inactive serine protease PAMR1 OS=Mus musculus GN=Pamr1 PE=2 SV=3 - [PAMR1_MOUSE]	-0.93	-0.77	-0.42	-0.26	0.03
Q8CG14	Complement C1s-A subcomponent OS=Mus musculus GN=C1sa PE=2 SV=2 - [CS1A_MOUSE]	-0.32	-0.92	-0.43	-1.03	0.03
Q8VCP8	Adenylate kinase isoenzyme 6 OS=Mus musculus GN=Taf9 PE=2 SV=1 - [KAD6_MOUSE]	-0.31	-0.54	-0.12	-0.34	0.03
D3Z6F5	ATP synthase subunit alpha OS=Mus musculus GN=Atp5a1 PE=3 SV=1 - [D3Z6F5_MOUSE]	-0.20	-0.38	-0.61	-0.80	0.03
Q9EQC8	Papillary renal cell carcinoma (Translocation-associated) OS=Mus musculus GN=Prcc PE=2 SV=1 - [Q9EQC8_MOUSE]	-0.68	-0.62	-0.28	-0.22	0.03
O88696	Putative ATP-dependent Clp protease proteolytic subunit, mitochondrial OS=Mus musculus GN=Clpp PE=2 SV=1 - [CLPP_MOUSE]	-0.16	-0.06	-0.17	-0.06	0.03
F8WIV2	Serpin B6 OS=Mus musculus GN=Serpinb6a PE=2 SV=1 - [F8WIV2_MOUSE]	-0.11	-0.03	-0.14	-0.08	0.03
P52482	Ubiquitin-conjugating enzyme E2 E1 OS=Mus musculus GN=Ube2e1 PE=2 SV=1 - [UB2E1_MOUSE]	0.13	0.40	0.25	0.53	0.03
P61079	Ubiquitin-conjugating enzyme E2 D3 OS=Mus musculus GN=Ube2d3 PE=1 SV=1 - [UB2D3_MOUSE]	0.43	1.15	0.40	0.66	0.03
E9Q9X4	Nitric oxide synthase OS=Mus musculus GN=Nos3 PE=2 SV=1 - [E9Q9X4_MOUSE]	-0.21	-0.61	-0.31	-0.72	0.03
Q64324	Syntaxin-binding protein 2 OS=Mus musculus GN=Stxbp2 PE=2 SV=1 - [STXB2_MOUSE]	0.24	0.06	0.32	0.28	0.03
Q8CI32	BAG family molecular chaperone regulator 5 OS=Mus musculus GN=Bag5 PE=1 SV=1 - [BAG5_MOUSE]	0.38	0.33	0.18	0.10	0.03
P15092-2	Isoform 2 of Interferon-activable protein 204 OS=Mus musculus GN=Ifi204 - [IFI4_MOUSE]	-0.10	-0.05	-0.05	-0.02	0.03

O35375-5	Isoform B0 of Neuropilin-2 OS=Mus musculus GN=Nrp2 - [NRP2_MOUSE]	0.06	0.17	0.23	0.10	0.03
D6RFB9	Syntaxin-1A OS=Mus musculus GN=Stx1a PE=2 SV=1 - [D6RFB9_MOUSE]	-0.36	-0.61	-0.13	-0.41	0.03
D3YXP6	Phosphomevalonate kinase OS=Mus musculus GN=Pmvk PE=2 SV=1 - [D3YXP6_MOUSE]	1.01	0.40	0.95	0.34	0.03
Q6PFQ7-2	Isoform 2 of Ras GTPase-activating protein 4 OS=Mus musculus GN=Rasa4 - [RASL2_MOUSE]	0.28	0.29	0.75	0.77	0.03
O09044	Synaptosomal-associated protein 23 OS=Mus musculus GN=Snap23 PE=1 SV=1 - [SNP23_MOUSE]	-0.18	-0.12	-0.24	-0.05	0.03
Q99LF4	tRNA-splicing ligase RtcB homolog OS=Mus musculus GN=D10Wsu52e PE=2 SV=1 - [RTCB_MOUSE]	0.01	0.04	0.05	0.06	0.03
Q689Z5	Protein strawberry notch homolog 1 OS=Mus musculus GN=Sbno1 PE=1 SV=2 - [SBNO1_MOUSE]	0.30	0.60	0.14	0.44	0.03
D3YXY5	DET1- and DDB1-associated protein 1 OS=Mus musculus GN=Dda1 PE=2 SV=1 - [D3YXY5_MOUSE]	-0.15	-0.45	-0.24	-0.54	0.03
P58252	Elongation factor 2 OS=Mus musculus GN=Eef2 PE=1 SV=2 - [EF2_MOUSE]	0.04	0.08	0.02	0.08	0.03
Q60953	Protein PML OS=Mus musculus GN=Pml PE=1 SV=3 - [PML_MOUSE]	-0.64	-0.48	-0.11	-0.45	0.03
P97742	Carnitine O-palmitoyltransferase 1, liver isoform OS=Mus musculus GN=Cpt1a PE=1 SV=4 - [CPT1A_MOUSE]	-0.15	-0.04	-0.17	-0.10	0.03
P10649	Glutathione S-transferase Mu 1 OS=Mus musculus GN=Gstm1 PE=1 SV=2 - [GSTM1_MOUSE]	-0.10	-0.03	-0.14	-0.08	0.03
M0QW65	Asparagine synthetase domain-containing protein 1 OS=Mus musculus GN=Asnsd1 PE=4 SV=1 - [M0QW65_MOUSE]	-0.40	-0.14	-0.64	-0.38	0.03
Q9Z109	V-set and immunoglobulin domain-containing protein 2 OS=Mus musculus GN=Vsig2 PE=2 SV=2 - [VSIG2_MOUSE]	0.17	0.48	0.21	0.52	0.03

P52479-2	Isoform 2 of Ubiquitin carboxyl-terminal hydrolase 10 OS=Mus musculus GN=Usp10 - [UBP10_MOUSE]	-0.65	-0.43	-0.42	-0.13	0.03
Q3TEA8-2	Isoform 2 of Heterochromatin protein 1-binding protein 3 OS=Mus musculus GN=Hp1bp3 - [HP1B3_MOUSE]	0.18	0.18	0.14	0.03	0.03
Q9JKB3-2	Isoform 2 of Y-box-binding protein 3 OS=Mus musculus GN=Ybx3 - [YBOX3_MOUSE]	-0.04	-0.12	-0.11	-0.19	0.03
O89079	Coatomer subunit epsilon OS=Mus musculus GN=Cope PE=2 SV=3 - [COPE_MOUSE]	0.11	0.04	0.15	0.07	0.03
Q9Z0D9	CX3C chemokine receptor 1 OS=Mus musculus GN=Cx3cr1 PE=2 SV=1 - [CX3C1_MOUSE]	0.30	0.38	0.86	0.94	0.03
Q6P1F6	Serine/threonine-protein phosphatase 2A 55 kDa regulatory subunit B alpha isoform OS=Mus musculus GN=Ppp2r2a PE=1 SV=1 - [2ABA_MOUSE]	-0.03	-0.07	-0.03	-0.09	0.03
Q9D5V6	Synapse-associated protein 1 OS=Mus musculus GN=Syap1 PE=1 SV=1 - [SYAP1_MOUSE]	-0.42	-0.20	-0.32	-0.10	0.03
Q80X50	Ubiquitin-associated protein 2-like OS=Mus musculus GN=Ubap2l PE=1 SV=1 - [UBP2L_MOUSE]	0.07	0.11	0.03	0.14	0.03
E9Q933	Transmembrane protein 11, mitochondrial OS=Mus musculus GN=Tmem11 PE=2 SV=1 - [E9Q933_MOUSE]	-0.21	-0.57	-0.22	-0.59	0.03
Q9Z2E3	Serine/threonine-protein kinase/endoribonuclease IRE2 OS=Mus musculus GN=Ern2 PE=2 SV=2 - [ERN2_MOUSE]	0.36	1.11	0.41	0.89	0.03
E9PZR3	Poly(A) polymerase alpha OS=Mus musculus GN=Papola PE=2 SV=1 - [E9PZR3_MOUSE]	0.17	0.30	0.06	0.24	0.03
Q9D306	Alpha-1,3-mannosyl-glycoprotein 4-beta-N-acetylglucosaminyltransferase C OS=Mus musculus GN=Mgat4c PE=2 SV=1 - [MGT4C_MOUSE]	0.24	0.48	0.12	0.28	0.03

Q9R078	5'-AMP-activated protein kinase subunit beta-1 OS=Mus musculus GN=Prkab1 PE=1 SV=2 - [AAKB1_MOUSE]	0.31	0.28	0.12	0.10	0.03
Q8BFQ8	Parkinson disease 7 domain-containing protein 1 OS=Mus musculus GN=Pddc1 PE=1 SV=1 - [PDDC1_MOUSE]	-0.41	-0.15	-0.33	-0.14	0.03
Q8C129	Leucyl-cystinyl aminopeptidase OS=Mus musculus GN=Lnpep PE=1 SV=1 - [LCAP_MOUSE]	-0.07	-0.26	-0.15	-0.28	0.03
D3Z131	THO complex subunit 6 homolog OS=Mus musculus GN=Thoc6 PE=2 SV=1 - [D3Z131_MOUSE]	-0.30	-0.84	-0.34	-0.88	0.03
Q9Z2U2-2	Isoform 2 of Zinc finger protein 292 OS=Mus musculus GN=Zfp292 - [ZN292_MOUSE]	-2.51	-2.57	-0.91	-0.97	0.03
K3W4T2	Uncharacterized protein OS=Mus musculus GN=Gm6632 PE=4 SV=1 - [K3W4T2_MOUSE]	-0.11	-0.06	-0.24	-0.19	0.03
P62960	Nuclease-sensitive element-binding protein 1 OS=Mus musculus GN=Ybx1 PE=1 SV=3 - [YBOX1_MOUSE]	-0.35	-0.12	-0.60	-0.49	0.03
Q08731	Lithostathine-2 OS=Mus musculus GN=Reg2 PE=2 SV=1 - [LIT2_MOUSE]	0.61	0.29	1.14	0.61	0.03
Q8HWE6	MHC I like leukocyte 2 OS=Mus musculus GN=Mill2 PE=2 SV=1 - [Q8HWE6_MOUSE]	-0.29	-0.84	-0.79	-1.35	0.03
Q99KK7	Dipeptidyl peptidase 3 OS=Mus musculus GN=Dpp3 PE=2 SV=2 - [DPP3_MOUSE]	-0.01	-0.04	-0.05	-0.03	0.03
Q8VCC1	15-hydroxyprostaglandin dehydrogenase [NAD(+)] OS=Mus musculus GN=Hpgd PE=2 SV=1 - [PGDH_MOUSE]	-0.46	-1.13	-0.33	-0.92	0.03
E9Q5N7	Protein Nrxn2 OS=Mus musculus GN=Nrxn2 PE=4 SV=2 - [E9Q5N7_MOUSE]	-0.54	-0.45	-0.24	-0.15	0.03
Q91VN6	Probable ATP-dependent RNA helicase DDX41 OS=Mus musculus GN=Ddx41 PE=1 SV=2 - [DDX41_MOUSE]	0.42	0.71	0.15	0.44	0.03
Q8R3I3	Conserved oligomeric Golgi complex subunit 6 OS=Mus musculus GN=Cog6 PE=2 SV=2 - [COG6_MOUSE]	0.44	0.54	0.14	0.24	0.03

Q91WE2	Protein FAM192A OS=Mus musculus GN=Fam192a PE=2 SV=1 - [F192A_MOUSE]	-0.15	-0.17	-0.05	-0.07	0.03
Q3UFS4	G patch domain-containing protein 11 OS=Mus musculus GN=Gpatch11 PE=2 SV=2 - [GPT11_MOUSE]	-0.11	-0.09	-0.29	-0.27	0.03
A9C437	Chloride channel protein 2 OS=Mus musculus GN=Clcn2 PE=2 SV=1 - [A9C437_MOUSE]	0.52	0.19	0.37	0.17	0.03
P41539	Protachykinin-1 OS=Mus musculus GN=Tac1 PE=2 SV=1 - [TKN1_MOUSE]	-0.43	-0.38	-0.90	-1.19	0.03
Q9QWK5	Baculoviral IAP repeat-containing protein 1a OS=Mus musculus GN=Naip1 PE=2 SV=3 [BIR1A_MOUSE]	0.14	0.19	0.41	0.46	0.03
Q9JK38	Glucosamine 6-phosphate N-acetyltransferase OS=Mus musculus GN=Gnpnat1 PE=2 SV=1 - [GNA1_MOUSE]	-0.03	-0.07	-0.04	-0.10	0.03
I6L974	TBC1 domain family member 17 OS=Mus musculus GN=Tbc1d17 PE=2 SV=1 - [I6L974_MOUSE]	-0.07	-0.23	-0.12	-0.28	0.03
Q8BZN6	Dedicator of cytokinesis protein 10 OS=Mus musculus GN=Dock10 PE=1 SV=3 - [DOC10_MOUSE]	-0.04	-0.28	-0.25	-0.26	0.03
Q8BP00-2	Isoform 2 of IQ calmodulin-binding motif-containing protein 1 OS=Mus musculus GN=Iqcb1 - [IQCB1_MOUSE]	-0.91	-0.48	-0.62	-0.20	0.03
Q9R013	Cathepsin F OS=Mus musculus GN=Ctsf PE=2 SV=1 - [CATF_MOUSE]	0.09	0.15	0.30	0.34	0.03
Q3TML4	Gene model 773, (NCBI) OS=Mus musculus GN=Gm773 PE=2 SV=1 - [Q3TML4_MOUSE]	-0.16	-0.47	-0.22	-0.53	0.03
D3Z7C8	Branched-chain-amino-acid aminotransferase OS=Mus musculus GN=Bcat2 PE=2 SV=1 [D3Z7C8_MOUSE]	-0.31	-0.09	-0.25	-0.12	0.03
Q569Z5	Probable ATP-dependent RNA helicase DDX46 OS=Mus musculus GN=Ddx46 PE=1 SV=2 - [DDX46_MOUSE]	0.05	0.30	0.20	0.27	0.03

Q9D9D9	Uncharacterized protein C1orf189 homolog OS=Mus musculus PE=2 SV=2 - [CA189_MOUSE]	-0.06	-0.04	-0.04	-0.01	0.03
Q91VB8	Alpha globin 1 OS=Mus musculus GN=Hba-a1 PE=2 SV=1 - [Q91VB8_MOUSE]	0.07	0.02	0.07	0.04	0.03
Q3TIV5	Zinc finger CCCH domain-containing protein 15 OS=Mus musculus GN=Zc3h15 PE=1 SV=2 - [ZC3HF_MOUSE]	0.30	0.37	0.09	0.48	0.03
Q9CPP0	Nucleoplasmin-3 OS=Mus musculus GN=Npm3 PE=2 SV=3 [NPM3_MOUSE]	-0.47	-0.18	-0.43	-0.15	0.03
P09803	Cadherin-1 OS=Mus musculus GN=Cdh1 PE=1 SV=1 - [CADH1_MOUSE]	-0.03	-0.10	-0.04	-0.08	0.03
Q8C102	Polypeptide N-acetylgalactosaminyltransferase 5 OS=Mus musculus GN=Galnt5 PE=2 SV=2 - [GALT5_MOUSE]	-0.07	-0.20	-0.09	-0.22	0.03
A2AQA7	Intron-binding protein aquarius OS=Mus musculus GN=Aqr PE=2 SV=1 - [A2AQA7_MOUSE]	0.09	0.16	0.15	0.32	0.03
B1ATW6	EF-hand calcium-binding domain-containing protein 9 OS=Mus musculus GN=Efcab9 PE=2 SV=1 - [B1ATW6_MOUSE]	-1.43	-3.42	-0.96	-2.95	0.03
O08686	Homeobox protein BarH-like 2 OS=Mus musculus GN=Barx2 PE=2 SV=2 - [BARX2_MOUSE]	-0.05	-0.05	-0.13	-0.14	0.03
O08539-2	Isoform 2 of Myc box-dependent-interacting protein 1 OS=Mus musculus GN=Bin1 - [BIN1_MOUSE]	-0.14	-0.13	-0.14	-0.34	0.03
Q811D0-2	Isoform 2 of Disks large homolog 1 OS=Mus musculus GN=Dlg1 - [DLG1_MOUSE]	-0.13	-0.19	-0.09	-0.05	0.03
P84089	Enhancer of rudimentary homolog OS=Mus musculus GN=Erh PE=1 SV=1 - [ERH_MOUSE]	-0.14	-0.06	-0.25	-0.15	0.03
A2AEC2	Transcription elongation factor A protein-like 3 (Fragment) OS=Mus musculus GN=Tceal3 PE=2 SV=1 - [A2AEC2_MOUSE]	-0.16	-0.23	-0.48	-0.55	0.03
Q9JJ28	Protein flightless-1 homolog OS=Mus musculus GN=Flii PE=1 SV=1 - [FLII_MOUSE]	0.14	0.02	0.16	0.14	0.03

E9Q5C9	Protein Nolc1 OS=Mus musculus GN=Nolc1 PE=2 SV=1 - [E9Q5C9_MOUSE]	0.27	0.26	0.21	0.04	0.03
Q9CQK7	RWD domain-containing protein 1 OS=Mus musculus GN=Rwdd1 PE=1 SV=1 - [RWDD1_MOUSE]	-0.05	-0.07	-0.15	-0.17	0.03
O88587-2	Isoform Soluble of Catechol O-methyltransferase OS=Mus musculus GN=Comt - [COMT_MOUSE]	-0.24	-0.06	-0.30	-0.16	0.03
Q99PL6-2	Isoform 2 of UBX domain-containing protein 6 OS=Mus musculus GN=Ubxn6 - [UBXN6_MOUSE]	0.12	0.28	0.56	0.40	0.03
Q8R2U0-2	Isoform 2 of Nucleoporin SEH1 OS=Mus musculus GN=Seh1l - [SEH1_MOUSE]	-0.07	-0.13	-0.04	-0.05	0.03
E9Q6Q8	TBC1 domain family member 4 OS=Mus musculus GN=Tbc1d4 PE=2 SV=1 - [E9Q6Q8_MOUSE]	0.24	0.08	0.18	0.09	0.03
Q9JKK8	Serine/threonine-protein kinase ATR OS=Mus musculus GN=Atr PE=1 SV=2 - [ATR_MOUSE]	-0.91	-0.34	-1.66	-1.09	0.03
P49722	Proteasome subunit alpha type-2 OS=Mus musculus GN=Psma2 PE=1 SV=3 - [PSA2_MOUSE]	-0.05	-0.03	-0.10	-0.13	0.03
Q6DI94	DNA excision repair protein ERCC-6-like 2 OS=Mus musculus GN=Ercc6l2 PE=2 SV=1 - [ER6L2_MOUSE]	0.30	0.73	0.20	0.62	0.03
E9Q9D8	Protein Ankrd35 OS=Mus musculus GN=Ankrd35 PE=2 SV=1 - [E9Q9D8_MOUSE]	-0.50	-0.42	-0.22	-0.13	0.03
Q9EQ09	Oxidized low-density lipoprotein receptor 1 OS=Mus musculus GN=Olr1 PE=2 SV=2 - [OLR1_MOUSE]	0.19	0.32	0.06	0.19	0.03
A2RSQ0	DENN domain-containing protein 5B OS=Mus musculus GN=Dennd5b PE=1 SV=2 - [DEN5B_MOUSE]	-0.14	-0.18	-0.42	-0.46	0.03
F7B6N2	SID1 transmembrane family member 2 (Fragment) OS=Mus musculus GN=Sidt2 PE=4 SV=1 - [F7B6N2_MOUSE]	-0.35	-0.66	-0.14	-0.45	0.03
Q9CRB8	Mitochondrial fission process protein 1 OS=Mus musculus GN=Mtfp1 PE=1 SV=1 - [MTFP1_MOUSE]	-0.32	-0.64	-0.14	-0.47	0.03

Q9EPR5	VPS10 domain-containing receptor SorCS2 OS=Mus musculus GN=Sorcs2 PE=1 SV=2 - [SORC2_MOUSE]	-0.28	-0.61	-0.18	-0.30	0.04
Q8BGN8-2	Isoform 2 of Synaptoporin OS=Mus musculus GN=Synpr - [SYNPR_MOUSE]	0.59	0.13	0.57	0.33	0.04
O08688	Calpain-5 OS=Mus musculus GN=Capn5 PE=2 SV=1 - [CAN5_MOUSE]	0.18	0.46	0.24	0.63	0.04
Q9JMH9-1	Isoform 1 of Unconventional myosin-XVIIIa OS=Mus musculus GN=Myo18a [MY18A_MOUSE]	0.08	0.10	0.03	0.04	0.04
G3UW75	Kelch-like 7 (Drosophila), isoform CRA_a OS=Mus musculus GN=Klhl7 PE=4 SV=1 - [G3UW75_MOUSE]	0.34	0.15	0.69	0.50	0.04
Q60972	Histone-binding protein RBBP4 OS=Mus musculus GN=Rbbp4 PE=1 SV=5 - [RBBP4_MOUSE]	-0.24	-0.17	-0.61	-0.46	0.04
Q06770	Corticosteroid-binding globulin OS=Mus musculus GN=Serpina6 PE=1 SV=1 - [CBG_MOUSE]	0.44	0.28	0.18	0.12	0.04
Q9R190	Metastasis-associated protein MTA2 OS=Mus musculus GN=Mta2 PE=1 SV=1 - [MTA2_MOUSE]	0.06	0.08	0.22	0.17	0.04
Q9QXK7	Cleavage and polyadenylation specificity factor subunit 3 OS=Mus musculus GN=Cpsf3 PE=1 SV=2 - [CPSF3_MOUSE]	0.15	0.08	0.33	0.27	0.04
Q91YZ2	C-terminal binding protein 2, isoform CRA_b OS=Mus musculus GN=Ctbp2 PE=2 SV=1 - [Q91YZ2_MOUSE]	0.28	0.17	0.61	0.32	0.04
Q64437	Alcohol dehydrogenase class 4 mu/sigma chain OS=Mus musculus GN=Adh7 PE=2 SV=2 - [ADH7_MOUSE]	0.15	0.48	0.27	0.60	0.04
O08739	AMP deaminase 3 OS=Mus musculus GN=Ampd3 PE=2 SV=2 - [AMPD3_MOUSE]	-0.09	-0.30	-0.17	-0.38	0.04
P70665-3	Isoform 3 of Sialate O-acetylesterase OS=Mus musculus GN=Siae - [SIAE_MOUSE]	-0.05	-0.29	-0.27	-0.36	0.04

Q8VI40	Tripartite motif-containing protein 60 OS=Mus musculus GN=Trim60 PE=2 SV=1 - [TRI60_MOUSE]	-0.11	-0.11	-0.31	-0.32	0.04
A0JLY1	Coiled-coil domain-containing protein 173 OS=Mus musculus GN=Ccdc173 PE=2 SV=1 - [CC173_MOUSE]	-0.08	-0.20	-0.05	-0.17	0.04
E9QKZ2	Importin-9 OS=Mus musculus GN=Ipo9 PE=2 SV=1 - [E9QKZ2_MOUSE]	0.48	0.15	0.38	0.17	0.04
Q9CQU3	Protein RER1 OS=Mus musculus GN=Rer1 PE=1 SV=1 - [RER1_MOUSE]	0.67	0.21	0.60	0.25	0.04
Q8CAS9-3	Isoform 3 of Poly [ADP-ribose] polymerase 9 OS=Mus musculus GN=Parp9 - [PARP9_MOUSE]	0.29	0.05	0.31	0.23	0.04
A2A3V2	Solute carrier family 25 member 43 OS=Mus musculus GN=Slc25a43 PE=2 SV=1 - [S2543_MOUSE]	0.51	0.25	0.38	0.11	0.04
Q9DBR0	A-kinase anchor protein 8 OS=Mus musculus GN=Akap8 PE=1 SV=1 - [AKAP8_MOUSE]	0.09	0.18	0.04	0.16	0.04
Q3UMW7	MAP kinase-activated protein kinase 3 OS=Mus musculus GN=Mapkapk3 PE=1 SV=2 - [MAPK3_MOUSE]	-0.06	-0.03	-0.04	-0.01	0.04
E9Q3I3	Spermatogenesis-associated protein 13 OS=Mus musculus GN=Spata13 PE=4 SV=1 - [E9Q3I3_MOUSE]	-0.39	-0.34	-0.15	-0.11	0.04
D3YU96	Cytohesin-2 OS=Mus musculus GN=Cyth2 PE=2 SV=1 - [D3YU96_MOUSE]	-0.08	-0.12	-0.10	-0.25	0.04
P97454	Mothers against decapentaplegic homolog 5 OS=Mus musculus GN=Smad5 PE=1 SV=2 - [SMAD5_MOUSE]	0.40	1.03	0.30	0.93	0.04
A2APF7	Z-DNA-binding protein 1 OS=Mus musculus GN=Zbp1 PE=1 SV=1 - [A2APF7_MOUSE]	0.44	0.39	0.15	0.15	0.04
F8VPX2	Protein Fbxw9 OS=Mus musculus GN=Fbxw9 PE=2 SV=1 - [F8VPX2_MOUSE]	0.03	0.09	0.04	0.11	0.04
F6RPD4	Phosphorylated adapter RNA export protein (Fragment) OS=Mus musculus GN=Phax PE=4 SV=1 - [F6RPD4_MOUSE]	0.04	0.12	0.04	0.12	0.04

Q8K1Z0	Ubiquinone biosynthesis protein COQ9, mitochondrial OS=Mus musculus GN=Coq9 PE=1 SV=1 - [COQ9_MOUSE]	-0.32	-0.27	-0.10	-0.11	0.04
Q9DBJ1	Phosphoglycerate mutase 1 OS=Mus musculus GN=Pgam1 PE=1 SV=3 - [PGAM1_MOUSE]	0.07	0.14	0.04	0.07	0.04
Q9Z2A7	Diacylglycerol O-acyltransferase 1 OS=Mus musculus GN=Dgat1 PE=1 SV=1 [DGAT1_MOUSE]	-0.28	-0.09	-0.51	-0.39	0.04
Q9EQ61	Pescadillo homolog OS=Mus musculus GN=Pes1 PE=1 SV=1 - [PESC_MOUSE]	0.18	0.09	0.39	0.30	0.04
Q8C156	Condensin complex subunit 2 OS=Mus musculus GN=Ncaph PE=2 SV=1 - [CND2_MOUSE]	0.60	0.58	0.22	0.20	0.04
P50540-2	Isoform Short of Max-interacting protein 1 OS=Mus musculus GN=Mxi1 - [MXI1_MOUSE]	-0.20	-0.38	-0.08	-0.25	0.04
Q61035	Histidine--tRNA ligase, cytoplasmic OS=Mus musculus GN=Hars PE=2 SV=2 - [SYHC_MOUSE]	-0.01	-0.03	-0.02	-0.01	0.04
P55096	ATP-binding cassette sub-family D member 3 OS=Mus musculus GN=Abcd3 PE=1 SV=2 - [ABCD3_MOUSE]	0.19	0.35	0.08	0.18	0.04
Q0P678	Zinc finger CCCH domain-containing protein 18 OS=Mus musculus GN=Zc3h18 PE=1 SV=1 - [ZCH18_MOUSE]	0.20	0.27	0.08	0.09	0.04
E9QL65	Conserved oligomeric Golgi complex subunit 3 OS=Mus musculus GN=Cog3 PE=4 SV=1 - [E9QL65_MOUSE]	0.64	0.21	0.68	0.26	0.04
Q61029	Lamina-associated polypeptide 2, isoforms beta/delta/epsilon/gamma OS=Mus musculus GN=Tmpo PE=1 SV=4 [LAP2B_MOUSE]	0.05	0.16	0.05	0.13	0.04
E9PVU0	Unconventional myosin-VI OS=Mus musculus GN=Myo6 PE=2 SV=1 - [E9PVU0_MOUSE]	0.55	0.16	0.68	0.29	0.04
Q9R187	Tumor necrosis factor receptor superfamily member EDAR OS=Mus musculus GN=Edar PE=1 SV=1 - [EDAR_MOUSE]	-0.40	-0.18	-0.31	-0.09	0.04

Q62189	U1 small nuclear ribonucleoprotein A OS=Mus musculus GN=Snrpa PE=2 SV=3 - [SNRPA_MOUSE]	-0.08	-0.18	-0.06	-0.21	0.04
Q9DD06	Retinoic acid receptor responder protein 2 OS=Mus musculus GN=Rarres2 PE=2 SV=1 - [RARR2_MOUSE]	-0.16	-0.55	-0.39	-0.78	0.04
Q9D6Y9	1,4-alpha-glucan-branching enzyme OS=Mus musculus GN=Gbe1 PE=2 SV=1 - [GLGB_MOUSE]	-0.27	-0.52	-0.10	-0.34	0.04
Q9Z0F4	Calcium and integrin-binding protein 1 OS=Mus musculus GN=Cib1 PE=1 SV=3 - [CIB1_MOUSE]	-0.16	-0.33	-0.11	-0.12	0.04
Q9Z1X4	Interleukin enhancer-binding factor 3 OS=Mus musculus GN=Ilf3 PE=1 SV=2 - [ILF3_MOUSE]	0.10	0.26	0.09	0.11	0.04
O35972	39S ribosomal protein L23, mitochondrial OS=Mus musculus GN=Mrpl23 PE=2 SV=1 - [RM23_MOUSE]	-0.84	-0.25	-1.08	-0.48	0.04
Q8VC03	Echinoderm microtubule-associated protein-like 3 OS=Mus musculus GN=Eml3 PE=2 SV=1 - [EMAL3_MOUSE]	-0.04	-0.22	-0.27	-0.28	0.04
Q9R1R8	Retinol dehydrogenase 11 OS=Mus musculus GN=Rdh11 PE=2 SV=1 - [Q9R1R8_MOUSE]	0.62	0.16	0.36	0.27	0.04
Q9CZN7	Serine hydroxymethyltransferase OS=Mus musculus GN=Shmt2 PE=2 SV=1 - [Q9CZN7_MOUSE]	0.10	0.07	0.06	0.02	0.04
E9Q8F9	Protein 6030445D17Rik OS=Mus musculus GN=6030445D17Rik PE=4 SV=1 - [E9Q8F9_MOUSE]	-0.29	-0.46	-0.09	-0.26	0.04
Q9CQR2	40S ribosomal protein S21 OS=Mus musculus GN=Rps21 PE=2 SV=1 - [RS21_MOUSE]	-0.05	-0.03	-0.10	-0.04	0.04
D3YZP9	Coiled-coil domain-containing protein 6 OS=Mus musculus GN=Ccdc6 PE=3 SV=1 - [CCDC6_MOUSE]	0.05	0.08	0.14	0.19	0.04
E9Q8Q5	Breast carcinoma-amplified sequence 1 homolog OS=Mus musculus GN=Bcas1 PE=2 SV=1 - [E9Q8Q5_MOUSE]	0.08	0.37	0.22	0.18	0.04

Q811J3	Iron-responsive element-binding protein 2 OS=Mus musculus GN=Ireb2 PE=2 SV=2 - [IREB2_MOUSE]	0.25	0.11	0.53	0.39	0.04
Q3V1V3	ESF1 homolog OS=Mus musculus GN=Esf1 PE=1 SV=1 - [ESF1_MOUSE]	0.12	0.10	0.33	0.30	0.04
E9Q3Z5	Supervillin OS=Mus musculus GN=Svil PE=2 SV=1 - [E9Q3Z5_MOUSE]	0.11	0.36	0.19	0.44	0.04
E9PUA2	Terminal uridylyltransferase 7 OS=Mus musculus GN=Zcchc6 PE=4 SV=1 - [E9PUA2_MOUSE]	0.22	0.13	0.04	0.19	0.04
P54071	Isocitrate dehydrogenase [NADP], mitochondrial OS=Mus musculus GN=Idh2 PE=1 SV=3 - [IDHP_MOUSE]	-0.10	-0.02	-0.11	-0.06	0.04
Q8R127	Saccharopine dehydrogenase-like oxidoreductase OS=Mus musculus GN=Sccpdh PE=2 SV=1 - [SCPDL_MOUSE]	0.13	0.32	0.09	0.27	0.04
P23953	Carboxylesterase 1C OS=Mus musculus GN=Ces1c PE=1 SV=4 - [EST1C_MOUSE]	-0.22	-0.06	-0.23	-0.10	0.04
D6RDS1	Lipase maturation factor 1 OS=Mus musculus GN=Lmf1 PE=2 SV=1 - [D6RDS1_MOUSE]	0.12	0.42	0.24	0.53	0.04
Q9Z2G6	Protein sel-1 homolog 1 OS=Mus musculus GN=Sel1l PE=2 SV=2 - [SE1L1_MOUSE]	0.23	0.04	0.18	0.14	0.04
Q3U487	E3 ubiquitin-protein ligase HECTD3 OS=Mus musculus GN=Hectd3 PE=1 SV=2 - [HECD3_MOUSE]	0.17	0.05	0.29	0.21	0.04
O35071	Kinesin-like protein KIF1C OS=Mus musculus GN=Kif1c PE=1 SV=2 - [KIF1C_MOUSE]	0.54	0.16	0.41	0.18	0.04
Q8CHP8	Phosphoglycolate phosphatase OS=Mus musculus GN=Pgp PE=2 SV=1 - [PGP_MOUSE]	0.16	0.19	0.03	0.13	0.04
Q99JT9	1,2-dihydroxy-3-keto-5-methylthiopentene dioxygenase OS=Mus musculus GN=Adi1 PE=1 SV=1 - [MTND_MOUSE]	-1.22	-1.23	-0.42	-0.43	0.04
O70582	Arachidonate 12-lipoxygenase, 12R-type OS=Mus musculus GN=Alox12b PE=2 SV=1 - [LX12B_MOUSE]	-0.02	-0.06	-0.08	-0.12	0.04

A2AWI9	Endophilin-B2 OS=Mus musculus GN=Sh3glb2 PE=4 SV=1 - [A2AWI9_MOUSE]	0.36	0.09	0.33	0.54	0.04
D3YU80	Cold-inducible RNA-binding protein (Fragment) OS=Mus musculus GN=Cirbp PE=2 SV=1 - [D3YU80_MOUSE]	-0.18	-0.52	-0.40	-0.83	0.04
Q62241	U1 small nuclear ribonucleoprotein C OS=Mus musculus GN=Snrpc PE=2 SV=1 [RU1C_MOUSE]	-0.33	-0.25	-0.16	-0.07	0.04
Q99L13	3-hydroxyisobutyrate dehydrogenase, mitochondrial OS=Mus musculus GN=Hibadh PE=2 SV=1 - [3HIDH_MOUSE]	-0.29	-0.11	-0.48	-0.22	0.04
D6RCG2	Dolichyl-phosphate beta-glucosyltransferase OS=Mus musculus GN=Alg5 PE=4 SV=1 - [D6RCG2_MOUSE]	0.13	0.44	0.19	0.20	0.04
Q9D824-4	Isoform 4 of Pre-mRNA 3'-end-processing factor FIP1 OS=Mus musculus GN=Fip1l1 - [FIP1_MOUSE]	-0.39	-0.16	-0.48	-0.15	0.04
A2AGG1	Protein Ttc17 OS=Mus musculus GN=Ttc17 PE=2 SV=1 - [A2AGG1_MOUSE]	-1.09	-1.00	-0.41	-0.32	0.04
Q9JKV5	Secretory carrier-associated membrane protein 4 OS=Mus musculus GN=Scamp4 PE=2 SV=1 - [SCAM4_MOUSE]	0.13	0.11	0.35	0.32	0.04
Q5M8N4-2	Isoform 2 of Epimerase family protein SDR39U1 OS=Mus musculus GN=Sdr39u1 - [D39U1_MOUSE]	-0.26	-0.66	-0.18	-0.57	0.04
Q80U35	Rho guanine nucleotide exchange factor 17 OS=Mus musculus GN=Arhgef17 PE=1 SV=2 - [ARHGH_MOUSE]	-0.17	-0.12	-0.21	-0.41	0.04
B8JK50	HMG box transcription factor BBX OS=Mus musculus GN=Bbx PE=2 SV=1 - [B8JK50_MOUSE]	-0.12	-0.40	-0.21	-0.49	0.04
P10711	Transcription elongation factor A protein 1 OS=Mus musculus GN=Tcea1 PE=1 SV=2 - [TCEA1_MOUSE]	-0.23	-0.38	-0.08	-0.20	0.04

G3UZL7	H-2 class I histocompatibility antigen, TLA(B) alpha chain OS=Mus musculus GN=H2-T3 PE=2 SV=1 - [G3UZL7_MOUSE]	-0.53	-0.37	-1.22	-1.35	0.04
P62911	60S ribosomal protein L32 OS=Mus musculus GN=Rpl32 PE=2 SV=2 [RL32_MOUSE]	-0.19	-0.10	-0.09	-0.04	0.04
Q8VHM5	Heterogeneous nuclear ribonucleoprotein R OS=Mus musculus GN=Hnrnpr PE=2 SV=1 - [Q8VHM5_MOUSE]	0.07	0.07	0.04	0.14	0.04
Q8CIH9	Amidophosphoribosyltransferase OS=Mus musculus GN=Ppat PE=2 SV=1 - [Q8CIH9_MOUSE]	0.29	0.15	0.08	0.13	0.04
Q9WV84	Nucleoside diphosphate kinase, mitochondrial OS=Mus musculus GN=Nme4 PE=1 SV=1 - [NDKM_MOUSE]	0.04	0.27	0.19	0.26	0.04
P20352	Tissue factor OS=Mus musculus GN=F3 PE=1 SV=2 - [TF_MOUSE]	0.07	0.13	0.16	0.28	0.04
P97370	Sodium/potassium-transporting ATPase subunit beta-3 OS=Mus musculus GN=Atp1b3 PE=1 SV=1 - [AT1B3_MOUSE]	-0.07	-0.17	-0.30	-0.33	0.04
Q99LL5	Periodic tryptophan protein 1 homolog OS=Mus musculus GN=Pwp1 PE=1 SV=1 - [PWP1_MOUSE]	0.07	0.19	0.15	0.05	0.04
Q8CG72	Poly(ADP-ribose) glycohydrolase ARH3 OS=Mus musculus GN=Adprhl2 PE=1 SV=1 - [ARHL2_MOUSE]	-0.09	-0.38	-0.16	-0.34	0.04
P17095	High mobility group protein HMG-I/HMG-Y OS=Mus musculus GN=Hmga1 PE=1 SV=4 - [HMGA1_MOUSE]	0.39	0.25	0.09	0.17	0.04
B0R105	Proline-rich protein 5-like (Fragment) OS=Mus musculus GN=Prr5l PE=2 SV=1 - [B0R105_MOUSE]	-0.12	-0.37	-0.15	-0.40	0.04
Q9Z2L6	Multiple inositol polyphosphate phosphatase 1 OS=Mus musculus GN=Minpp1 PE=1 SV=3 - [MINP1_MOUSE]	-0.28	-0.06	-0.39	-0.25	0.04

P06151	L-lactate dehydrogenase A chain OS=Mus musculus GN=Ldha PE=1 SV=3 - [LDHA_MOUSE]	-0.11	-0.02	-0.15	-0.08	0.04
Q6TXD4	Dynamin-binding protein OS=Mus musculus GN=Dnmbp PE=1 SV=2 - [DNMBP_MOUSE]	-0.04	-0.18	-0.23	-0.12	0.04
Q9D1P0	39S ribosomal protein L13, mitochondrial OS=Mus musculus GN=Mrpl13 PE=2 SV=1 - [RM13_MOUSE]	0.19	0.05	0.08	0.11	0.04
Q9JLJ0	Lipopolysaccharide-induced tumor necrosis factor-alpha factor homolog OS=Mus musculus GN=Litaf PE=1 SV=1 - [LITAF_MOUSE]	0.10	0.34	0.12	0.28	0.04
Q9CWV4	Melanoma-associated antigen B16 OS=Mus musculus GN=Mageb16 PE=2 SV=1 - [MAGBG_MOUSE]	0.64	0.20	0.72	0.28	0.04
Q9R0M6	Ras-related protein Rab-9A OS=Mus musculus GN=Rab9a PE=1 SV=1 - [RAB9A_MOUSE]	0.06	0.14	0.27	0.28	0.04
Q99NF3	Centrosomal protein of 41 kDa OS=Mus musculus GN=Cep41 PE=1 SV=1 - [CEP41_MOUSE]	-1.31	-0.51	-1.13	-0.34	0.04
Q99K74-3	Isoform 3 of Mediator of RNA polymerase II transcription subunit 24 OS=Mus musculus GN=Med24 - [MED24_MOUSE]	0.46	0.27	1.13	0.94	0.04
F6W623	Histone-lysine N-methyltransferase MLL4 (Fragment) OS=Mus musculus GN=Kmt2b PE=4 SV=1 - [F6W623_MOUSE]	0.14	0.05	0.28	0.18	0.04
Q8CDN6	Thioredoxin-like protein 1 OS=Mus musculus GN=Txnl1 PE=1 SV=3 - [TXNL1_MOUSE]	-0.09	-0.01	-0.08	-0.06	0.04
Q9CQJ6	Density-regulated protein OS=Mus musculus GN=Denr PE=2 SV=1 - [DENR_MOUSE]	-0.04	-0.04	-0.13	-0.08	0.04
A2AMD2	Protein Ankrd60 OS=Mus musculus GN=Ankrd60 PE=2 SV=2 - [A2AMD2_MOUSE]	-1.10	-0.35	-1.17	-0.43	0.04
E9Q6B2	Coiled-coil domain-containing protein 85C OS=Mus musculus GN=Ccdc85c PE=2 SV=1 - [CC85C_MOUSE]	-0.12	-0.18	-0.31	-0.46	0.04

Q9WUP7-2	Isoform 2 of Ubiquitin carboxyl-terminal hydrolase isozyme L5 OS=Mus musculus GN=Uchl5 - [UCHL5_MOUSE]	0.57	0.43	0.18	0.17	0.04
Q9CQU0	Thioredoxin domain-containing protein 12 OS=Mus musculus GN=Txndc12 PE=2 SV=1 - [TXD12_MOUSE]	-0.10	-0.06	-0.15	-0.04	0.04
Q9CPV4	Glyoxalase domain-containing protein 4 OS=Mus musculus GN=Glod4 PE=2 SV=1 [GLOD4_MOUSE]	0.05	0.08	0.01	0.07	0.04
Q61598	Rab GDP dissociation inhibitor beta OS=Mus musculus GN=Gdi2 PE=1 SV=1 - [GDIB_MOUSE]	0.09	0.17	0.04	0.09	0.04
E9PXM9	Protein Fbxw24 OS=Mus musculus GN=Fbxw24 PE=4 SV=1 - [E9PXM9_MOUSE]	0.19	0.29	0.63	0.73	0.04
Q9QYJ0	DnaJ homolog subfamily A member 2 OS=Mus musculus GN=Dnaja2 PE=1 SV=1 - [DNJA2_MOUSE]	0.11	0.23	0.04	0.15	0.04
Q8VHI3	GDP-fucose protein O-fucosyltransferase 2 OS=Mus musculus GN=Pofut2 PE=1 SV=1 - [OFUT2_MOUSE]	-0.07	-0.27	-0.10	-0.21	0.04
Q9QWR8	Alpha-N-acetylgalactosaminidase OS=Mus musculus GN=Naga PE=2 SV=2 - [NAGAB_MOUSE]	-0.18	-0.09	-0.08	-0.04	0.04
P97411-2	Isoform 2 of Islet cell autoantigen 1 OS=Mus musculus GN=Ica1 - [ICA69_MOUSE]	0.95	0.70	0.44	0.19	0.04
P35951	Low-density lipoprotein receptor OS=Mus musculus GN=Ldlr PE=1 SV=2 - [LDLR_MOUSE]	0.25	0.49	0.09	0.33	0.04
P63168	Dynein light chain 1, cytoplasmic OS=Mus musculus GN=Dynll1 PE=1 SV=1 - [DYL1_MOUSE]	0.09	0.42	0.33	0.58	0.04
Q9QYS9-4	Isoform 4 of Protein quaking OS=Mus musculus GN=Qki - [QKI_MOUSE]	-0.04	-0.27	-0.22	-0.17	0.04
Q8C2E7	WASH complex subunit strumpellin OS=Mus musculus GN=Kiaa0196 PE=2 SV=2 - [STRUM_MOUSE]	0.08	0.20	0.42	0.34	0.04
Q9D1Q6	Endoplasmic reticulum resident protein 44 OS=Mus musculus GN=Erp44 PE=1 SV=1 [ERP44_MOUSE]	0.06	0.13	0.03	0.13	0.04

Q9CR67	Transmembrane protein 33 OS=Mus musculus GN=Tmem33 PE=2 SV=1 - [TMM33_MOUSE]	-0.20	-0.37	-0.09	-0.45	0.04
Q921K9-2	Isoform 2 of B-cell CLL/lymphoma 7 protein family member B OS=Mus musculus GN=Bcl7b - [BCL7B_MOUSE]	0.26	0.17	0.14	0.05	0.04
H7BXB9	Protein 1700037H04Rik (Fragment) OS=Mus musculus GN=1700037H04Rik PE=2 SV=1 - [H7BXB9_MOUSE]	-0.44	-0.31	-0.07	-0.26	0.04
Q9Z2I8-2	Isoform 2 of Succinyl-CoA ligase [GDP-forming] subunit beta, mitochondrial OS=Mus musculus GN=Suclg2 - [SUCB2_MOUSE]	-0.14	-0.05	-0.13	-0.04	0.04
Q3UKW2	Calmodulin OS=Mus musculus GN=Calm1 PE=2 SV=1 - [Q3UKW2_MOUSE]	-0.33	-0.10	-0.35	-0.13	0.04
J3QP71	Basigin (Fragment) OS=Mus musculus GN=Bsg PE=4 SV=1 - [J3QP71_MOUSE]	0.46	1.39	0.47	1.39	0.04
Q9D967	Magnesium-dependent phosphatase 1 OS=Mus musculus GN=Mdp1 PE=1 SV=1 - [MGDP1_MOUSE]	0.04	0.05	0.13	0.07	0.04
Q99M87-2	Isoform 2 of DnaJ homolog subfamily A member 3, mitochondrial OS=Mus musculus GN=Dnaja3 - [DNJA3_MOUSE]	-0.02	-0.07	-0.03	-0.04	0.04
Q9WUR2-2	Isoform 2 of Enoyl-CoA delta isomerase 2, mitochondrial OS=Mus musculus GN=Eci2 - [ECI2_MOUSE]	-0.10	-0.18	-0.18	-0.03	0.04
Q3TYX3	SET and MYND domain-containing protein 5 OS=Mus musculus GN=Smyd5 PE=2 SV=2 - [SMYD5_MOUSE]	0.29	0.62	0.12	0.44	0.04
O09049	Regenerating islet-derived protein 3-gamma OS=Mus musculus GN=Reg3g PE=1 SV=1 - [REG3G_MOUSE]	-0.71	-0.47	-0.35	-0.13	0.04
Q9WUR9	GTP:AMP phosphotransferase AK4, mitochondrial OS=Mus musculus GN=Ak4 PE=2 SV=1 - [KAD4_MOUSE]	-0.05	-0.08	-0.17	-0.20	0.04
Q9D7E4	UPF0449 protein C19orf25 homolog OS=Mus musculus PE=1 SV=1 - [CS025_MOUSE]	0.22	0.04	0.13	0.11	0.04

Q99KR7	Peptidyl-prolyl cis-trans isomerase F, mitochondrial OS=Mus musculus GN=Ppif PE=1 SV=1 - [PPIF_MOUSE]	-0.27	-0.08	-0.42	-0.20	0.04
E9PV26	Protein BC068157 OS=Mus musculus GN=BC068157 PE=2 SV=1 - [E9PV26_MOUSE]	-1.47	-2.04	-0.39	-0.96	0.04
Q31094	Class II histocompatibility antigen, M beta 1 chain OS=Mus musculus GN=H2-DMb1 PE=2 SV=2 - [Q31094_MOUSE]	-0.33	-0.20	-0.11	-0.10	0.04
Q7M6Z4-2	Isoform 2 of Kinesin-like protein KIF27 OS=Mus musculus GN=Kif27 - [KIF27_MOUSE]	-0.32	-0.25	-0.84	-0.42	0.04
Q8BK72	28S ribosomal protein S27, mitochondrial OS=Mus musculus GN=Mrps27 PE=2 SV=2 - [RT27_MOUSE]	0.03	0.05	0.06	0.12	0.04
Q8R5I6	Glutathione S-transferase mu 4 OS=Mus musculus GN=Gstm4 PE=2 SV=1 - [Q8R5I6_MOUSE]	-0.37	-0.33	-0.04	-0.26	0.04
Q5UE59	Kinesin light chain 1 OS=Mus musculus GN=Klc1 PE=2 SV=1 - [Q5UE59_MOUSE]	0.06	0.02	0.10	0.06	0.04
E9QPE8	Plectin OS=Mus musculus GN=Plec PE=2 SV=1 - [E9QPE8_MOUSE]	0.59	0.18	1.08	0.67	0.04
E0CZE0	NEDD8-activating enzyme E1 regulatory subunit OS=Mus musculus GN=Nae1 PE=2 SV=1 - [E0CZE0_MOUSE]	0.09	0.24	0.17	0.40	0.04
J3QMQ6	Protein Gm3336 OS=Mus musculus GN=Gm3336 PE=4 SV=1 - [J3QMQ6_MOUSE]	0.39	0.12	0.41	0.14	0.04
P49769	Presenilin-1 OS=Mus musculus GN=Psen1 PE=1 SV=1 - [PSN1_MOUSE]	0.12	0.54	0.44	0.78	0.04
O70479	BTB/POZ domain-containing adapter for CUL3-mediated RhoA degradation protein 2 OS=Mus musculus GN=Tnfaip1 PE=1 SV=1 - [BACD2_MOUSE]	0.70	0.29	0.57	0.16	0.04
Q8BT60	Copine-3 OS=Mus musculus GN=Cpne3 PE=1 SV=2 - [CPNE3_MOUSE]	-0.07	-0.16	-0.03	-0.12	0.04
H7BX02	Probable E3 ubiquitin-protein ligase Roquin OS=Mus musculus GN=Rc3h1 PE=2 SV=1 - [H7BX02_MOUSE]	0.62	0.22	0.58	0.18	0.04

A2AQ92	Protein Duox1 OS=Mus musculus GN=Duox1 PE=4 SV=1 - [A2AQ92_MOUSE]	-0.68	-0.79	-0.19	-0.31	0.04
Q61179	Interferon regulatory factor 9 OS=Mus musculus GN=Irf9 PE=1 SV=1 - [IRF9_MOUSE]	-0.13	-0.10	-0.06	-0.03	0.04
Q8BK67	Protein RCC2 OS=Mus musculus GN=Rcc2 PE=2 SV=1 - [RCC2_MOUSE]	-0.06	-0.11	-0.05	-0.19	0.04
P61979-3	Isoform 3 of Heterogeneous nuclear ribonucleoprotein K OS=Mus musculus GN=Hnrnpk - [HNRPK_MOUSE]	0.10	0.31	0.22	0.49	0.04
Q9WVA3	Mitotic checkpoint protein BUB3 OS=Mus musculus GN=Bub3 PE=2 SV=2 - [BUB3_MOUSE]	-0.14	-0.03	-0.13	-0.22	0.04
Q7TME2	Sperm-associated antigen 5 OS=Mus musculus GN=Spag5 PE=1 SV=1 - [SPAG5_MOUSE]	0.35	0.18	0.16	0.08	0.04
Q91VJ1	Interferon-inducible protein AIM2 OS=Mus musculus GN=Aim2 PE=1 SV=2 - [AIM2_MOUSE]	-0.25	-0.66	-0.17	-0.58	0.04
H3BLR8	Diphosphoinositol polyphosphate phosphohydrolase 1 OS=Mus musculus GN=Nudt3 PE=2 SV=1 - [H3BLR8_MOUSE]	-0.27	-0.27	-0.09	-0.09	0.04
D3YUU6	Protein Crnn OS=Mus musculus GN=Crnn PE=4 SV=1 - [D3YUU6_MOUSE]	0.25	0.65	0.16	0.56	0.04
Q8VD62	UPF0696 protein C11orf68 homolog OS=Mus musculus GN=Bles03 PE=2 SV=1 - [CK068_MOUSE]	-0.06	-0.29	-0.12	-0.22	0.04
D3Z0Y2	Peroxiredoxin-6 OS=Mus musculus GN=Prdx6 PE=2 SV=1 [D3Z0Y2_MOUSE]	-0.15	-0.03	-0.15	-0.08	0.04
Q8BGC4	Zinc-binding alcohol dehydrogenase domain containing protein 2 OS=Mus musculus GN=Zadh2 PE=2 SV=1 - [ZADH2_MOUSE]	-0.14	-0.32	-0.54	-0.20	0.04
Q9Z1Q2	Abhydrolase domain-containing protein 16A OS=Mus musculus GN=Abhd16a PE=1 SV=3 - [ABHGA_MOUSE]	-0.10	-0.24	-0.07	-0.28	0.04
E9QAF2	Protein Abca16 OS=Mus musculus GN=Abca16 PE=2 SV=1 - [E9QAF2_MOUSE]	-0.20	-0.06	-0.37	-0.24	0.04

Q8K354	Carbonyl reductase [NADPH] 3 OS=Mus musculus GN=Cbr3 PE=2 SV=1 - [CBR3_MOUSE]	-0.01	-0.05	-0.05	-0.04	0.04
Q61878	Bone marrow proteoglycan OS=Mus musculus GN=Prg2 PE=1 SV=1 - [PRG2_MOUSE]	0.59	0.50	0.23	0.14	0.04
E9Q7Q0	Mucin-4 OS=Mus musculus GN=Muc4 PE=2 SV=1 - [E9Q7Q0_MOUSE]	0.69	1.10	0.14	0.94	0.04
Q8BZR9	Uncharacterized protein C17orf85 homolog OS=Mus musculus PE=1 SV=1 - [CQ085_MOUSE]	0.51	0.14	0.66	0.29	0.04
P38647	Stress-70 protein, mitochondrial OS=Mus musculus GN=Hspa9 PE=1 SV=3 - [GRP75_MOUSE]	-0.12	-0.04	-0.19	-0.09	0.04
Q9CQQ8	U6 snRNA-associated Sm-like protein LSm7 OS=Mus musculus GN=Lsm7 PE=3 SV=1 - [LSM7_MOUSE]	-0.20	-0.07	-0.18	-0.05	0.04
P51450	Nuclear receptor ROR-gamma OS=Mus musculus GN=Rorc PE=1 SV=1 - [RORG_MOUSE]	-0.57	-1.31	-0.27	-1.01	0.04
Q8BH95	Enoyl-CoA hydratase, mitochondrial OS=Mus musculus GN=Echs1 PE=1 SV=1 - [ECHM_MOUSE]	-0.03	-0.07	-0.06	-0.02	0.04
Q80U38	Protein KHNYN OS=Mus musculus GN=Khnyn PE=2 SV=2 - [KHNYN_MOUSE]	0.60	0.23	0.41	0.14	0.04
Q9R0X2	ADAM DEC1 OS=Mus musculus GN=Adamdec1 PE=2 SV=1 - [ADEC1_MOUSE]	0.17	0.06	0.17	0.06	0.04
H3BL88	Protein 9930021J03Rik (Fragment) OS=Mus musculus GN=9930021J03Rik PE=4 SV=1 - [H3BL88_MOUSE]	-0.29	-1.13	-0.83	-1.67	0.04
Q8R2M2	Deoxynucleotidyltransferase terminal-interacting protein 2 OS=Mus musculus GN=Dnttip2 PE=1 SV=1 - [TDIF2_MOUSE]	-0.18	-0.56	-0.18	-0.55	0.04
Q99K95	Protein RTF2 homolog OS=Mus musculus GN=Rtfdc1 PE=2 SV=1 - [RTF2_MOUSE]	-0.31	-0.09	-0.52	-0.30	0.04
Q6ZWX6	Eukaryotic translation initiation factor 2 subunit 1 OS=Mus musculus GN=Eif2s1 PE=1 SV=3 - [IF2A_MOUSE]	0.11	0.04	0.11	0.04	0.04

F6U4D1	Glycosyltransferase 8 domain-containing protein 2 (Fragment) OS=Mus musculus GN=Glt8d2 PE=4 SV=2 - [F6U4D1_MOUSE]	0.31	0.92	0.29	0.91	0.04
Q9CZV8-4	Isoform 2 of F-box/LRR repeat protein 20 OS=Mus musculus GN=Fbxl20 - [FXL20_MOUSE]	-0.56	-0.37	-0.29	-0.09	0.04
Q6GQR8	Zinc finger protein 329 OS=Mus musculus GN=Znf329 PE=2 SV=2 - [ZN329_MOUSE]	-0.49	-0.18	-0.48	-0.14	0.04
P70444	BH3-interacting domain death agonist OS=Mus musculus GN=Bid PE=1 SV=2 - [BID_MOUSE]	-0.10	-0.22	-0.04	-0.16	0.04
Q8BGF3	WD repeat-containing protein 92 OS=Mus musculus GN=Wdr92 PE=2 SV=1 - [WDR92_MOUSE]	-0.75	-0.25	-0.72	-0.23	0.04
Q61635	GTP-binding protein OS=Mus musculus GN=Ifi47 PE=2 SV=1 - [Q61635_MOUSE]	0.22	0.49	0.10	0.42	0.04
E9Q3K0	Protein Gm6588 OS=Mus musculus GN=Gm6588 PE=4 SV=1 - [E9Q3K0_MOUSE]	0.12	0.35	0.11	0.35	0.04
Q8VDG3	Poly(A)-specific ribonuclease PARN OS=Mus musculus GN=Parn PE=1 SV=1 - [PARN_MOUSE]	0.27	0.10	0.19	0.07	0.04
Q925I1	ATPase family AAA domain-containing protein 3 OS=Mus musculus GN=Atad3 PE=1 SV=1 - [ATAD3_MOUSE]	-0.03	-0.11	-0.13	-0.05	0.04
P98191	Phosphatidate cytidylyltransferase 1 OS=Mus musculus GN=Cds1 PE=1 SV=2 - [CDS1_MOUSE]	0.06	0.37	0.40	0.23	0.04
Q8BW49	Tetratricopeptide repeat protein 12 OS=Mus musculus GN=Ttc12 PE=2 SV=1 - [TTC12_MOUSE]	-0.08	-0.07	-0.23	-0.23	0.04
Q9CPP6	NADH dehydrogenase [ubiquinone] 1 alpha subcomplex subunit 5 OS=Mus musculus GN=Ndufa5 PE=1 SV=3 - [NDUA5_MOUSE]	0.25	0.40	0.06	0.25	0.04
Q8C6B0	MCG20149, isoform CRA_a OS=Mus musculus GN=Mettl7a1 PE=2 SV=1 - [Q8C6B0_MOUSE]	-0.18	-0.04	-0.28	-0.16	0.04
E9Q7G0	Protein Numa1 OS=Mus musculus GN=Numa1 PE=2 SV=1 - [E9Q7G0_MOUSE]	-0.08	-0.05	-0.11	-0.02	0.04

Q3TCV3	Gamma-secretase-activating protein OS=Mus musculus GN=Gsap PE=2 SV=1 - [GSAP_MOUSE]	-0.18	-0.51	-0.15	-0.48	0.04
E9QNR5	Thrombospondin type-1 domain-containing protein 7A OS=Mus musculus GN=Thsd7a PE=2 SV=1 - [E9QNR5_MOUSE]	-0.03	-0.32	-0.32	-0.24	0.04
Q3V3Q7	Phosphofurin acidic cluster sorting protein 2 OS=Mus musculus GN=Pacs2 PE=1 SV=2 - [PACS2_MOUSE]	0.27	0.69	0.16	0.58	0.04
Q9WTL2	Ras-related protein Rab-25 OS=Mus musculus GN=Rab25 PE=1 SV=2 - [RAB25_MOUSE]	-0.05	-0.18	-0.07	-0.09	0.04
Q61371	Intraflagellar transport protein 88 homolog OS=Mus musculus GN=Ift88 PE=1 SV=2 - [IFT88_MOUSE]	-0.74	-0.36	-0.51	-0.13	0.04
E0CYJ0	MOB-like protein phocein (Fragment) OS=Mus musculus GN=Mob4 PE=2 SV=1 - [E0CYJ0_MOUSE]	-0.25	-0.20	-0.05	-0.35	0.04
P39053-3	Isoform 3 of Dynamin-1 OS=Mus musculus GN=Dnm1 - [DYN1_MOUSE]	-0.24	-0.49	-0.09	-0.34	0.04
O88487	Cytoplasmic dynein 1 intermediate chain 2 OS=Mus musculus GN=Dync1i2 PE=2 SV=1 - [DC1I2_MOUSE]	-0.22	-0.22	-0.13	-0.03	0.04
Q8C788	Sorting nexin 18 OS=Mus musculus GN=Snx18 PE=2 SV=1 - [Q8C788_MOUSE]	-0.31	-0.41	-0.07	-0.20	0.04
E9PXC2	Upstream stimulatory factor 2 OS=Mus musculus GN=Usf2 PE=2 SV=1 - [E9PXC2_MOUSE]	0.36	0.09	0.49	0.22	0.04
Q148W8	Inactive dual specificity phosphatase 27 OS=Mus musculus GN=Dusp27 PE=2 SV=1 - [DUS27_MOUSE]	-0.35	-0.66	-0.11	-0.42	0.04
P00405	Cytochrome c oxidase subunit 2 OS=Mus musculus GN=Mtco2 PE=1 SV=1 - [COX2_MOUSE]	-0.07	-0.07	-0.04	-0.01	0.04
Q9DC29	ATP-binding cassette sub-family B member 6, mitochondrial OS=Mus musculus GN=Abcb6 PE=1 SV=1 - [ABCB6_MOUSE]	-0.21	-0.16	-0.28	-0.04	0.04
D3Z420	MCG18394 OS=Mus musculus GN=Fam186b PE=4 SV=1 - [D3Z420_MOUSE]	-0.31	-0.09	-0.59	-0.38	0.04

O08553	Dihydropyrimidinase-related protein 2 OS=Mus musculus GN=Dpysl2 PE=1 SV=2 - [DPYL2_MOUSE]	-0.02	-0.08	-0.09	-0.14	0.04
Q9D051	Pyruvate dehydrogenase E1 component subunit beta, mitochondrial OS=Mus musculus GN=Pdhb PE=1 SV=1 - [ODPB_MOUSE]	-0.06	-0.04	-0.14	-0.15	0.04
P27661	Histone H2A.x OS=Mus musculus GN=H2afx PE=1 SV=2 [H2AX_MOUSE]	-0.30	-0.08	-0.47	-0.24	0.04
G3X940	Histone acetyltransferase KAT6A OS=Mus musculus GN=Kat6a PE=4 SV=1 - [G3X940_MOUSE]	-1.10	-2.96	-0.71	-2.57	0.04
Q920H4	Muscarinic acetylcholine receptor M5 OS=Mus musculus GN=Chrm5 PE=2 SV=2 - [ACM5_MOUSE]	-0.10	-0.24	-0.05	-0.19	0.04
Q2YDW2-2	Isoform 2 of Protein misato homolog 1 OS=Mus musculus GN=Msto1 - [MSTO1_MOUSE]	0.06	0.21	0.25	0.40	0.04
Q0KK56-2	Isoform 2 of Protein FAM184B OS=Mus musculus GN=Fam184b - [F184B_MOUSE]	-0.31	-1.18	-0.57	-1.44	0.04
Q5SSP3	Neutral amino acid transporter A OS=Mus musculus GN=Slc1a4 PE=2 SV=1 - [Q5SSP3_MOUSE]	0.08	0.30	0.27	0.50	0.04
Q8VDK1-2	Isoform 2 of Nitrilase homolog 1 OS=Mus musculus GN=Nit1 - [NIT1_MOUSE]	-0.08	-0.36	-0.14	-0.26	0.04
Q9D1F9	Protein Slc37a4 OS=Mus musculus GN=Slc37a4 PE=2 SV=1 - [Q9D1F9_MOUSE]	-1.02	-1.50	-0.25	-0.73	0.04
F8VPX6	Protein Speer4a OS=Mus musculus GN=Speer4a PE=4 SV=1 - [F8VPX6_MOUSE]	-0.87	-3.07	-1.24	-3.45	0.04
G3UYY2	Selenium-binding protein 2 OS=Mus musculus GN=Selenbp2 PE=2 SV=1 - [G3UYY2_MOUSE]	-0.44	-0.46	-0.13	-0.15	0.04
B7ZC85	Serine/threonine-protein kinase tousled-like 2 OS=Mus musculus GN=Tlk2 PE=2 SV=1 - [B7ZC85_MOUSE]	0.15	0.29	0.05	0.18	0.04
Q9CWL8	Beta-catenin-like protein 1 OS=Mus musculus GN=Ctnnbl1 PE=1 SV=1 - [CTBL1_MOUSE]	0.12	0.71	0.33	0.60	0.04

Q9R1T4-2	Isoform I of Septin-6 OS=Mus musculus GN=Sept6 - [SEPT6_MOUSE]	-0.05	-0.16	-0.04	-0.11	0.04
P70698	CTP synthase 1 OS=Mus musculus GN=Ctps1 PE=1 SV=2 [PYRG1_MOUSE]	0.33	0.28	0.15	0.06	0.04
Q78IK4	Apolipoprotein O-like OS=Mus musculus GN=Apool PE=2 SV=1 [APOOL_MOUSE]	-0.18	-0.05	-0.31	-0.15	0.04
D3Z3E2	Glycerophosphodiester phosphodiesterase 1 (Fragment) OS=Mus musculus GN=Gde1 PE=2 SV=1 - [D3Z3E2_MOUSE]	0.33	0.15	0.25	0.06	0.04
A2AQ25	Sickle tail protein OS=Mus musculus GN=Skt PE=1 SV=1 - [SKT_MOUSE]	-0.06	-0.23	-0.09	-0.25	0.04
P08207	Protein S100-A10 OS=Mus musculus GN=S100a10 PE=2 SV=2 - [S10AA_MOUSE]	-0.20	-0.08	-0.41	-0.21	0.04
A6PWX1	Calcium channel flower homolog OS=Mus musculus GN=Cacfd1 PE=2 SV=1 - [A6PWX1_MOUSE]	0.23	0.39	0.06	0.22	0.04
Q63844	Mitogen-activated protein kinase 3 OS=Mus musculus GN=Mapk3 PE=1 SV=5 - [MK03_MOUSE]	-0.04	-0.06	-0.02	-0.02	0.04
Q8BKR5	Protein phosphatase 1 regulatory subunit 37 OS=Mus musculus GN=Ppp1r37 PE=2 SV=1 - [PPR37_MOUSE]	0.25	0.68	0.16	0.59	0.05
E9Q8X5	Protein Zbtb10 OS=Mus musculus GN=Zbtb10 PE=4 SV=1 - [E9Q8X5_MOUSE]	-0.44	-0.67	-0.11	-0.34	0.05
Q9CZP3	Protein Zfp655 OS=Mus musculus GN=Zfp655 PE=2 SV=1 - [Q9CZP3_MOUSE]	-0.43	-0.81	-0.12	-0.50	0.05
D6RCX5	Mitochondrial fission regulator 1-like OS=Mus musculus GN=Mtfr1l PE=2 SV=1 - [D6RCX5_MOUSE]	-0.07	-0.18	-0.04	-0.14	0.05
F6ZK01	Myeloblastin (Fragment) OS=Mus musculus GN=Prtn3 PE=3 SV=1 - [F6ZK01_MOUSE]	1.61	0.58	1.43	0.40	0.05
Q06335	Amyloid-like protein 2 OS=Mus musculus GN=Aplp2 PE=1 SV=4 [APLP2_MOUSE]	-0.26	-0.43	-0.07	-0.24	0.05
P07356	Annexin A2 OS=Mus musculus GN=Anxa2 PE=1 SV=2 - [ANXA2_MOUSE]	-0.08	-0.02	-0.13	-0.09	0.05

Q8BTJ4-2	Isoform 2 of Bis(5'-adenosyl)-triphosphatase enpp4 OS=Mus musculus GN=Enpp4 - [ENPP4_MOUSE]	-0.12	-0.20	-0.45	-0.53	0.05
Q9DBG1	Sterol 26-hydroxylase, mitochondrial OS=Mus musculus GN=Cyp27a1 PE=1 SV=1 - [CP27A_MOUSE]	-0.29	-1.04	-0.41	-1.16	0.05
Q91YE7-2	Isoform 2 of RNA-binding protein 5 OS=Mus musculus GN=Rbm5 - [RBM5_MOUSE]	0.03	0.26	0.36	0.26	0.05
Q8R3P8	Protein Rnf113a1 OS=Mus musculus GN=Rnf113a1 PE=2 SV=1 - [Q8R3P8_MOUSE]	0.15	0.18	0.50	0.54	0.05
P61358	60S ribosomal protein L27 OS=Mus musculus GN=Rpl27 PE=2 SV=2 [RL27_MOUSE]	-0.18	-0.08	-0.15	-0.03	0.05
Q9CQ01	Ribonuclease T2 OS=Mus musculus GN=Rnaset2 PE=2 SV=1 - [RNT2_MOUSE]	-0.16	-0.03	-0.24	-0.18	0.05
Q64151	Semaphorin-4C OS=Mus musculus GN=Sema4c PE=1 SV=1 - [SEM4C_MOUSE]	0.15	0.42	0.10	0.36	0.05
Q9DBT5	AMP deaminase 2 OS=Mus musculus GN=Ampd2 PE=1 SV=1 - [AMPD2_MOUSE]	-0.16	-0.38	-0.11	-0.16	0.05
P14206	40S ribosomal protein SA OS=Mus musculus GN=Rpsa PE=1 SV=4 - [RSSA_MOUSE]	-0.03	-0.11	-0.12	-0.20	0.05
Q792Z1	MCG140784 OS=Mus musculus GN=Try10 PE=2 SV=1 - [Q792Z1_MOUSE]	-0.14	-0.03	-0.25	-0.23	0.05
O70433	Four and a half LIM domains protein 2 OS=Mus musculus GN=Fhl2 PE=1 SV=1 - [FHL2_MOUSE]	-0.09	-0.41	-0.14	-0.28	0.05
P23198	Chromobox protein homolog 3 OS=Mus musculus GN=Cbx3 PE=1 SV=2 - [CBX3_MOUSE]	0.11	0.27	0.06	0.17	0.05
Q62465	Synaptic vesicle membrane protein VAT-1 homolog OS=Mus musculus GN=Vat1 PE=1 SV=3 - [VAT1_MOUSE]	-0.05	-0.04	-0.16	-0.13	0.05
D3Z2Y6	Protein S100a16 (Fragment) OS=Mus musculus GN=S100a16 PE=2 SV=1 - [D3Z2Y6_MOUSE]	0.22	0.16	0.22	0.02	0.05

Q8C0C7	Phenylalanine--tRNA ligase alpha subunit OS=Mus musculus GN=Farsa PE=2 SV=1 [SYFA_MOUSE]	0.36	0.22	0.33	0.04	0.05
F8WIE5	E3 ubiquitin-protein ligase HECTD1 OS=Mus musculus GN=Hectd1 PE=2 SV=1 - [F8WIE5_MOUSE]	0.40	0.04	0.44	0.29	0.05
Q8BS28	Protein Gm10032 OS=Mus musculus GN=Gm10032 PE=2 SV=1 - [Q8BS28_MOUSE]	-0.58	-0.14	-0.82	-0.38	0.05
Q3USJ8-3	Isoform 3 of FCH and double SH3 domains protein 2 OS=Mus musculus GN=Fchsd2 - [FCSD2_MOUSE]	-0.26	-0.12	-0.18	-0.04	0.05
Q8R2V5	Arf-GAP with dual PH domain-containing protein 2 OS=Mus musculus GN=Adap2 PE=2 SV=1 - [ADAP2_MOUSE]	0.10	0.17	0.03	0.10	0.05
B1AZA5	Transmembrane protein 245 OS=Mus musculus GN=Tmem245 PE=1 SV=1 - [TM245_MOUSE]	-0.45	-0.17	-0.38	-0.10	0.05
Q80Z19	Mucin-2 (Fragments) OS=Mus musculus GN=Muc2 PE=1 SV=2 - [MUC2_MOUSE]	0.07	0.09	0.03	0.02	0.05
Q9R257	Heme-binding protein 1 OS=Mus musculus GN=Hebp1 PE=1 SV=2 - [HEBP1_MOUSE]	0.13	0.99	0.62	0.57	0.05
D3Z3D2	Nuclear cap-binding protein subunit 2 OS=Mus musculus GN=Ncbp2 PE=2 SV=1 - [D3Z3D2_MOUSE]	0.06	0.08	0.01	0.05	0.05
E9Q5I7	Endoglin OS=Mus musculus GN=Eng PE=2 SV=1 - [E9Q5I7_MOUSE]	-0.60	-0.42	-0.25	-0.12	0.05
M0QWG8	CAS1 domain-containing protein 1 (Fragment) OS=Mus musculus GN=Casd1 PE=4 SV=1 - [M0QWG8_MOUSE]	0.30	0.17	0.18	0.04	0.05
Q9DA64	CMT1A duplicated region transcript 4 protein homolog OS=Mus musculus GN=Cdrt4 PE=2 SV=1 - [CDRT4_MOUSE]	0.28	0.09	0.28	0.09	0.05
P32883-2	Isoform 2B of GTPase KRas OS=Mus musculus GN=Kras - [RASK_MOUSE]	-0.17	-0.07	-0.27	-0.09	0.05
P15261	Interferon gamma receptor 1 OS=Mus musculus GN=Ifngr1 PE=1 SV=3 - [INGR1_MOUSE]	-0.09	-0.04	-0.22	-0.17	0.05

D3YWD5	Major facilitator superfamily domain-containing protein 10 (Fragment) OS=Mus musculus GN=Mfsd10 PE=2 SV=1 - [D3YWD5_MOUSE]	-0.05	-0.17	-0.05	-0.16	0.05
P50543	Protein S100-A11 OS=Mus musculus GN=S100a11 PE=2 SV=1 - [S10AB_MOUSE]	-0.09	-0.15	-0.02	-0.11	0.05
Q91WG2-1	Isoform 2 of Rab GTPase-binding effector protein 2 OS=Mus musculus GN=Rabep2 - [RABE2_MOUSE]	-0.01	-0.09	-0.06	-0.11	0.05
Q8CES0	N-alpha-acetyltransferase 30 OS=Mus musculus GN=Naa30 PE=2 SV=2 - [NAA30_MOUSE]	-0.21	-0.17	-0.42	-0.10	0.05
Q62230	Sialoadhesin OS=Mus musculus GN=Siglec1 PE=1 SV=2 - [SN_MOUSE]	-0.36	-0.47	-0.04	-0.32	0.05
Q8VHL1	Histone-lysine N-methyltransferase SETD7 OS=Mus musculus GN=Setd7 PE=2 SV=2 - [SETD7_MOUSE]	-0.05	-0.08	-0.05	-0.01	0.05
D3Z0M9	MCG18410, isoform CRA_a OS=Mus musculus GN=Ddx23 PE=3 SV=1 - [D3Z0M9_MOUSE]	0.06	0.14	0.31	0.31	0.05
Q9CQ73	Plakophilin 2 OS=Mus musculus GN=Pkp2 PE=2 SV=1 - [Q9CQ73_MOUSE]	0.16	0.02	0.11	0.09	0.05
Q9QYI4	DnaJ homolog subfamily B member 12 OS=Mus musculus GN=Dnajb12 PE=2 SV=2 - [DJB12_MOUSE]	0.12	0.41	0.12	0.35	0.05
A2AP32	NADH dehydrogenase [ubiquinone] 1 beta subcomplex subunit 6 OS=Mus musculus GN=Ndufb6 PE=2 SV=1 - [A2AP32_MOUSE]	-0.07	-0.02	-0.02	-0.05	0.05
Q9CQ52	Chymotrypsin-like elastase family member 3B OS=Mus musculus GN=Cela3b PE=2 SV=1 - [CEL3B_MOUSE]	0.89	0.27	1.24	0.45	0.05
Q91VH2	Sorting nexin-9 OS=Mus musculus GN=Snx9 PE=1 SV=1 - [SNX9_MOUSE]	0.15	0.02	0.21	0.16	0.05
Q99LP6	GrpE protein homolog 1, mitochondrial OS=Mus musculus GN=Grpel1 PE=1 SV=1 - [GRPE1_MOUSE]	-0.17	-0.06	-0.08	-0.05	0.05

Q8VEH6	COBW domain-containing protein 1 OS=Mus musculus GN=Cbwd1 PE=2 SV=1 - [CBWD1_MOUSE]	0.12	0.09	0.15	0.02	0.05
Q8VDM6-2	Isoform 2 of Heterogeneous nuclear ribonucleoprotein U-like protein 1 OS=Mus musculus GN=Hnrnpul1 - [HNRL1_MOUSE]	-0.20	-0.20	-0.08	-0.05	0.05
Q5SSH7-2	Isoform 2 of Zinc finger ZZ-type and EF-hand domain-containing protein 1 OS=Mus musculus GN=Zzef1 - [ZZEF1_MOUSE]	0.40	0.44	0.16	0.10	0.05
Q8C079-4	Isoform 4 of Striatin-interacting protein 1 OS=Mus musculus GN=Strip1 - [STRP1_MOUSE]	0.04	0.17	0.31	0.18	0.05
P70699	Lysosomal alpha-glucosidase OS=Mus musculus GN=Gaa PE=1 SV=2 - [LYAG_MOUSE]	-0.12	-0.17	-0.02	-0.10	0.05
Q80TC6	Zinc finger SWIM domain-containing protein 5 OS=Mus musculus GN=Zswim5 PE=2 SV=2 - [ZSWM5_MOUSE]	0.33	0.25	0.14	0.06	0.05
D3YXJ5	Protein Fam84b OS=Mus musculus GN=Fam84b PE=4 SV=1 - [D3YXJ5_MOUSE]	-0.28	-0.07	-0.45	-0.22	0.05
Q1RLL5	Predicted gene, EG331529 OS=Mus musculus GN=Gm5128 PE=2 SV=1 - [Q1RLL5_MOUSE]	-1.17	-0.50	-0.77	-0.21	0.05
Q9CS84-4	Isoform 4a of Neurexin-1 OS=Mus musculus GN=Nrxn1 - [NRX1A_MOUSE]	-0.22	-0.72	-0.21	-0.71	0.05
Q3UI43	BRISC and BRCA1-A complex member 1 OS=Mus musculus GN=Babam1 PE=2 SV=1 - [BABA1_MOUSE]	0.12	0.06	0.14	0.03	0.05
Q9D902	General transcription factor IIE subunit 2 OS=Mus musculus GN=Gtf2e2 PE=2 SV=2 - [T2EB_MOUSE]	0.08	0.37	0.25	0.53	0.05
Q05AA6	Dystrophin-related protein 2 OS=Mus musculus GN=Drp2 PE=1 SV=1 - [DRP2_MOUSE]	0.45	0.64	0.10	0.29	0.05
Q61070	Etoposide-induced protein 2.4 OS=Mus musculus GN=Ei24 PE=1 SV=3 - [EI24_MOUSE]	0.16	0.06	0.36	0.26	0.05

Q61091	Frizzled-8 OS=Mus musculus GN=Fzd8 PE=1 SV=2 - [FZD8_MOUSE]	-0.18	-0.44	-0.08	-0.34	0.05
Q6DFW5	ATPase, Class VI, type 11B OS=Mus musculus GN=Atp11b PE=2 SV=1 - [Q6DFW5_MOUSE]	0.22	0.34	0.05	0.17	0.05
F6X4A2	Regulator of G-protein-signaling 3 (Fragment) OS=Mus musculus GN=Rgs3 PE=4 SV=1 - [F6X4A2_MOUSE]	-0.05	-0.13	-0.22	-0.30	0.05
P70186	Epiphycan OS=Mus musculus GN=Epyc PE=2 SV=1 - [EPYC_MOUSE]	0.53	0.94	2.12	2.52	0.05
P28650	Adenylosuccinate synthetase isozyme 1 OS=Mus musculus GN=Adssl1 PE=1 SV=2 - [PURA1_MOUSE]	0.14	0.51	0.16	0.30	0.05
Q8R3Q0	Store-operated calcium entry-associated regulatory factor OS=Mus musculus GN=Tmem66 PE=2 SV=2 - [SARAF_MOUSE]	0.18	0.16	0.57	0.55	0.05
Q3U422	NADH dehydrogenase [ubiquinone] flavoprotein 3, mitochondrial OS=Mus musculus GN=Ndufv3 PE=2 SV=1 - [Q3U422_MOUSE]	-0.42	-0.06	-0.50	-0.27	0.05
Q3UGR5	Haloacid dehalogenase like hydrolase domain-containing protein 2 OS=Mus musculus GN=Hdhd2 PE=1 SV=2 - [HDHD2_MOUSE]	-0.21	-0.17	-0.14	-0.02	0.05
Q8BYZ7-2	Isoform 2 of Engulfment and cell motility protein 3 OS=Mus musculus GN=Elmo3 - [ELMO3_MOUSE]	0.51	0.42	0.10	0.19	0.05
Q8CHQ0	F-box only protein 4 OS=Mus musculus GN=Fbxo4 PE=1 SV=2 - [FBX4_MOUSE]	0.09	0.37	0.17	0.44	0.05
O54851	Gap junction delta-2 protein OS=Mus musculus GN=Gjd2 PE=2 SV=2 - [CXD2_MOUSE]	-0.11	-0.03	-0.13	-0.05	0.05
Q9WUN2	Serine/threonine-protein kinase TBK1 OS=Mus musculus GN=Tbk1 PE=1 SV=1 - [TBK1_MOUSE]	-0.09	-0.30	-0.10	-0.32	0.05
Q6ZPY7-2	Isoform 2 of Lysine-specific demethylase 3B OS=Mus musculus GN=Kdm3b - [KDM3B_MOUSE]	-0.51	-0.32	-0.26	-0.07	0.05

Q9D142	Uridine diphosphate glucose pyrophosphatase OS=Mus musculus GN=Nudt14 PE=2 SV=1 - [NUD14_MOUSE]	-0.13	-0.48	-0.18	-0.53	0.05
Q8BFS6-4	Isoform 4 of Calcineurin like phosphoesterase domain-containing protein 1 OS=Mus musculus GN=Cpped1 - [CPPED_MOUSE]	-0.01	-0.05	-0.07	-0.08	0.05
Q9WUK6	Zinc finger and BTB domain-containing protein 18 OS=Mus musculus GN=Zbtb18 PE=2 SV=1 - [ZBT18_MOUSE]	0.60	0.40	0.29	0.09	0.05
Q9WV27	Sodium/potassium-transporting ATPase subunit alpha-4 OS=Mus musculus GN=Atp1a4 PE=1 SV=3 - [AT1A4_MOUSE]	0.06	0.06	0.22	0.21	0.05
P26150	3 beta-hydroxysteroid dehydrogenase/Delta 5-->4-isomerase type 3 OS=Mus musculus GN=Hsd3b3 PE=2 SV=3 - [3BHS3_MOUSE]	-0.08	-0.24	-0.22	-0.45	0.05
Q8VDL4-3	Isoform 3 of ADP-dependent glucokinase OS=Mus musculus GN=Adpgk - [ADPGK_MOUSE]	-0.12	-0.35	-0.08	-0.32	0.05
Q8BHL5-2	Isoform 2 of Engulfment and cell motility protein 2 OS=Mus musculus GN=Elmo2 - [ELMO2_MOUSE]	-0.04	-0.18	-0.09	-0.23	0.05
O54998	Peptidyl-prolyl cis-trans isomerase FKBP7 OS=Mus musculus GN=Fkbp7 PE=1 SV=1 - [FKBP7_MOUSE]	-1.05	-0.55	-0.64	-0.14	0.05
Q8R1I1	Cytochrome b-c1 complex subunit 9 OS=Mus musculus GN=Uqcr10 PE=1 SV=1 - [QCR9_MOUSE]	-0.12	-0.02	-0.18	-0.09	0.05
Q4PZA2-3	Isoform C of Endothelin converting enzyme 1 OS=Mus musculus GN=Ece1 - [ECE1_MOUSE]	-0.10	-0.35	-0.11	-0.19	0.05
H7BXB6	Protein CLEC16A OS=Mus musculus GN=Clec16a PE=2 SV=1 - [H7BXB6_MOUSE]	-0.20	-0.05	-0.23	-0.08	0.05
A2AAA9	C-type mannose receptor 2 OS=Mus musculus GN=Mrc2 PE=2 SV=1 - [A2AAA9_MOUSE]	-0.25	-0.15	-0.09	-0.06	0.05

Q9JHW9	Aldehyde dehydrogenase family 1 member A3 OS=Mus musculus GN=Aldh1a3 PE=2 SV=1 - [AL1A3_MOUSE]	-0.39	-0.04	-0.55	-0.57	0.05
Q8BY79	Solute carrier family 35 member G1 OS=Mus musculus GN=Slc35g1 PE=2 SV=1 - [S35G1_MOUSE]	0.12	0.05	0.29	0.22	0.05
Q64337-2	Isoform 2 of Sequestosome-1 OS=Mus musculus GN=Sqstm1 - [SQSTM_MOUSE]	0.08	0.33	0.22	0.49	0.05
Q61074	Protein phosphatase 1G OS=Mus musculus GN=Ppm1g PE=2 SV=3 - [PPM1G_MOUSE]	-0.07	-0.01	-0.08	-0.11	0.05
Q8CHS8-2	Isoform 2 of Vacuolar protein sorting-associated protein 37A OS=Mus musculus GN=Vps37a - [VP37A_MOUSE]	0.26	0.84	0.22	0.79	0.05
Q61771	Kinesin-like protein KIF3B OS=Mus musculus GN=Kif3b PE=1 SV=1 - [KIF3B_MOUSE]	0.06	0.25	0.09	0.12	0.05
D3Z6W2	Tyrosine-protein phosphatase non-receptor type OS=Mus musculus GN=Ptpn2 PE=2 SV=1 - [D3Z6W2_MOUSE]	0.16	0.18	0.57	0.58	0.05
P27601	Guanine nucleotide-binding protein subunit alpha-13 OS=Mus musculus GN=Gna13 PE=1 SV=1 - [GNA13_MOUSE]	0.05	0.05	0.06	0.00	0.05
Q8BH65-2	Isoform 2 of Protein DENND6A OS=Mus musculus GN=Dennd6a - [DEN6A_MOUSE]	0.84	2.20	0.42	1.79	0.05
D3Z598	Latent-transforming growth factor beta-binding protein 4 OS=Mus musculus GN=Ltbp4 PE=2 SV=1 [D3Z598_MOUSE]	-0.04	-0.07	-0.13	-0.19	0.05
F8WJG3	Transformer-2 protein homolog beta OS=Mus musculus GN=Tra2b PE=2 SV=1 - [F8WJG3_MOUSE]	-0.32	-0.07	-0.39	-0.16	0.05
Q8R1F0	Leydig cell tumor 10 kDa protein homolog OS=Mus musculus GN=D8Ertd738e PE=2 SV=1 - [L10K_MOUSE]	0.12	0.39	0.57	0.84	0.05
O35250-2	Isoform 2 of Exocyst complex component 7 OS=Mus musculus GN=Exoc7 - [EXOC7_MOUSE]	0.05	0.23	0.13	0.09	0.05

E9QPI5	Sister chromatid cohesion protein PDS5 homolog A OS=Mus musculus GN=Pds5a PE=4 SV=1 - [E9QPI5_MOUSE]	0.19	0.10	0.49	0.45	0.05
A0A4W9	Neuronal growth regulator 1 OS=Mus musculus GN=Negr1 PE=2 SV=1 - [A0A4W9_MOUSE]	-1.98	-0.81	-0.52	-0.81	0.05
Q924D0	Reticulon-4-interacting protein 1, mitochondrial OS=Mus musculus GN=Rtn4ip1 PE=1 SV=2 - [RT4I1_MOUSE]	0.77	0.23	0.25	0.35	0.05
Q8BZ97	PR domain zinc finger protein 8 OS=Mus musculus GN=Prdm8 PE=2 SV=1 - [PRDM8_MOUSE]	-0.34	-0.80	-0.13	-0.59	0.05
Q9CXX9	CUE domain-containing protein 2 OS=Mus musculus GN=Cuedc2 PE=2 SV=1 - [CUED2_MOUSE]	0.16	0.21	0.01	0.23	0.05
Q8K1J5	Protein SDE2 homolog OS=Mus musculus GN=Sde2 PE=1 SV=1 - [SDE2_MOUSE]	-0.19	-0.09	-0.13	-0.03	0.05
Q3UHB1	5'-nucleotidase domain-containing protein 3 OS=Mus musculus GN=Nt5dc3 PE=2 SV=1 - [NT5D3_MOUSE]	-0.28	-0.65	-0.10	-0.47	0.05
H3BLK8	Zinc finger homeobox protein 4 OS=Mus musculus GN=Zfhx4 PE=2 SV=1 - [H3BLK8_MOUSE]	0.39	0.65	0.08	0.34	0.05
E9PUV7	Protein Gm872 OS=Mus musculus GN=Gm872 PE=2 SV=1 - [E9PUV7_MOUSE]	0.28	0.24	0.10	0.06	0.05
Q8CAQ8-3	Isoform 3 of Mitochondrial inner membrane protein OS=Mus musculus GN=Immt - [IMMT_MOUSE]	-0.14	-0.55	-0.18	-0.53	0.05
F6ZH76	Hepatocyte nuclear factor 1-beta (Fragment) OS=Mus musculus GN=Hnf1b PE=2 SV=1 - [F6ZH76_MOUSE]	-0.53	-2.51	-1.58	-3.56	0.05
Q8BKT7	THO complex subunit 5 homolog OS=Mus musculus GN=Thoc5 PE=1 SV=2 - [THOC5_MOUSE]	0.36	0.48	0.03	0.37	0.05
B2RXC2	Inositol 1,4,5-trisphosphate 3-kinase B OS=Mus musculus GN=Itpkb PE=2 SV=1 - [B2RXC2_MOUSE]	-0.34	-0.37	-0.05	-0.18	0.05

P50396	Rab GDP dissociation inhibitor alpha OS=Mus musculus GN=Gdi1 PE=1 SV=3 - [GDIA_MOUSE]	-0.07	-0.03	-0.11	-0.03	0.05
F6VS49	Protein Apol8 OS=Mus musculus GN=Apol8 PE=4 SV=2 - [F6VS49_MOUSE]	-0.07	-0.30	-0.30	-0.53	0.05
O35864	COP9 signalosome complex subunit 5 OS=Mus musculus GN=Cops5 PE=1 SV=3 - [CSN5_MOUSE]	0.12	0.41	0.10	0.29	0.05
D3Z4C9	Uncharacterized protein OS=Mus musculus GN=Mnf1 PE=2 SV=1 - [D3Z4C9_MOUSE]	0.44	0.48	0.08	0.19	0.05
Q8C4Q6	Axin interactor, dorsalization-associated protein OS=Mus musculus GN=Aida PE=1 SV=1 - [AIDA_MOUSE]	0.33	0.16	0.22	0.04	0.05
Q9CZD0	Methylmalonic aciduria and homocystinuria type C protein homolog OS=Mus musculus GN=Mmachc PE=2 SV=2 - [MMAC_MOUSE]	-0.11	-0.52	-0.39	-0.80	0.05
A6H584	Collagen alpha-5(VI) chain OS=Mus musculus GN=Col6a5 PE=1 SV=2 - [CO6A5_MOUSE]	0.50	0.26	0.52	0.07	0.05
Q9Z315	U4/U6.U5 tri-snRNP-associated protein 1 OS=Mus musculus GN=Sart1 PE=2 SV=1 - [SNUT1_MOUSE]	0.09	0.22	0.06	0.09	0.05
P08071	Lactotransferrin OS=Mus musculus GN=Ltf PE=2 SV=4 - [TRFL_MOUSE]	-0.04	-0.13	-0.17	-0.27	0.05
Q8BHG3	Cell cycle control protein 50B OS=Mus musculus GN=Tmem30b PE=2 SV=1 - [CC50B_MOUSE]	0.27	0.06	0.44	0.23	0.05
B1ART1	Protein Vps13d OS=Mus musculus GN=Vps13d PE=2 SV=1 - [B1ART1_MOUSE]	-0.80	-0.99	-0.18	-0.37	0.05
Q9CQJ4	E3 ubiquitin-protein ligase RING2 OS=Mus musculus GN=Rnf2 PE=1 SV=1 - [RING2_MOUSE]	-0.34	-0.46	-0.07	-0.19	0.05
Q9D7S7-2	Isoform 2 of 60S ribosomal protein L22-like 1 OS=Mus musculus GN=Rpl22l1 - [RL22L_MOUSE]	0.58	0.18	0.51	0.14	0.05
Q9QXK3-4	Isoform 4 of Coatomer subunit gamma-2 OS=Mus musculus GN=Copg2 - [COPG2_MOUSE]	0.52	0.36	0.44	0.03	0.05

O08789	Max-binding protein MNT OS=Mus musculus GN=Mnt PE=2 SV=2 - [MNT_MOUSE]	0.04	0.05	0.14	0.15	0.05
Q8BSN5	PHD finger protein 23 OS=Mus musculus GN=Phf23 PE=2 SV=2 [PHF23_MOUSE]	0.05	0.06	0.19	0.20	0.05
Q99LC3	NADH dehydrogenase [ubiquinone] 1 alpha subcomplex subunit 10, mitochondrial OS=Mus musculus GN=Ndufa10 PE=1 SV=1 - [NDUAA_MOUSE]	-0.16	-0.05	-0.15	-0.04	0.05
F6VIG0	Protein RMD5 homolog B (Fragment) OS=Mus musculus GN=Rmnd5b PE=4 SV=1 - [F6VIG0_MOUSE]	-0.04	-0.32	-0.18	-0.32	0.05
P70288	Histone deacetylase 2 OS=Mus musculus GN=Hdac2 PE=1 SV=1 - [HDAC2_MOUSE]	-0.38	-0.15	-0.38	-0.08	0.05
Q8K2Y7	39S ribosomal protein L47, mitochondrial OS=Mus musculus GN=Mrpl47 PE=2 SV=2 - [RM47_MOUSE]	-0.52	-0.84	-0.10	-0.45	0.05
P07361	Alpha-1-acid glycoprotein 2 OS=Mus musculus GN=Orm2 PE=1 SV=1 - [A1AG2_MOUSE]	-0.30	-0.73	-0.12	-0.56	0.05
Q0VGB7	Serine/threonine-protein phosphatase 4 regulatory subunit 2 OS=Mus musculus GN=Ppp4r2 PE=1 SV=1 - [PP4R2_MOUSE]	0.17	0.58	0.14	0.41	0.05
Q61324-2	Isoform 2 of Aryl hydrocarbon receptor nuclear translocator 2 OS=Mus musculus GN=Arnt2 - [ARNT2_MOUSE]	-0.08	-0.25	-0.37	-0.55	0.05
Q62219-2	Isoform 2 of Transforming growth factor beta-1-induced transcript 1 protein OS=Mus musculus GN=Tgfb1i1 - [TGFI1_MOUSE]	0.03	0.18	0.07	0.15	0.05
P08121	Collagen alpha-1(III) chain OS=Mus musculus GN=Col3a1 PE=1 SV=4 - [CO3A1_MOUSE]	-0.41	-0.15	-0.45	-0.10	0.05
Q6PDD0	UDP-glucuronosyltransferase 2A2 OS=Mus musculus GN=Ugt2a2 PE=2 SV=1 - [UD2A2_MOUSE]	-0.45	-1.78	-0.65	-1.98	0.05
Q8CH77-4	Isoform 4 of Neuron navigator 1 OS=Mus musculus GN=Nav1 - [NAV1_MOUSE]	-0.15	-0.06	-0.33	-0.37	0.05

D3YYA0	Set1/Ash2 histone methyltransferase complex subunit ASH2 OS=Mus musculus GN=Ash2l PE=2 SV=1 [D3YYA0_MOUSE]	-0.26	-0.72	-0.23	-0.92	0.05
O88874	Cyclin-K OS=Mus musculus GN=Ccnk PE=1 SV=3 - [CCNK_MOUSE]	0.23	0.07	0.51	0.36	0.05
P47941	Crk-like protein OS=Mus musculus GN=Crkl PE=1 SV=2 - [CRKL_MOUSE]	-0.30	-0.22	-0.44	-0.05	0.05
O54983	Thiomorpholine-carboxylate dehydrogenase OS=Mus musculus GN=Crym PE=1 SV=1 - [CRYM_MOUSE]	-0.16	-0.48	-0.11	-0.45	0.05
Q8BJZ4	28S ribosomal protein S35, mitochondrial OS=Mus musculus GN=Mrps35 PE=2 SV=2 - [RT35_MOUSE]	0.04	0.08	0.10	0.19	0.05
Q62440-3	Isoform 3 of Transducin like enhancer protein 1 OS=Mus musculus GN=Tle1 - [TLE1_MOUSE]	-0.32	-0.52	-0.05	-0.32	0.05
E9PYJ8	Histone acetyltransferase p300 OS=Mus musculus GN=Ep300 PE=4 SV=1 - [E9PYJ8_MOUSE]	0.15	0.18	0.56	0.59	0.05
Q5SWP3	NAC-alpha domain-containing protein 1 OS=Mus musculus GN=Nacad PE=1 SV=1 - [NACAD_MOUSE]	-0.23	-0.06	-0.47	-0.30	0.05
Q8C0M2	Abhydrolase domain-containing protein 4 OS=Mus musculus GN=Abhd4 PE=2 SV=1 - [Q8C0M2_MOUSE]	-0.25	-0.60	-0.09	-0.44	0.05
Q8C0T5-2	Isoform 2 of Signal-induced proliferation-associated 1-like protein 1 OS=Mus musculus GN=Sipa1l1 - [SI1L1_MOUSE]	-0.40	-0.09	-0.74	-0.42	0.05
Q9CQD4	Charged multivesicular body protein 1b-2 OS=Mus musculus GN=Chmp1b2 PE=2 SV=2 - [CH1B2_MOUSE]	-0.16	-0.51	-0.13	-0.48	0.05
Q6ZQL4	WD repeat-containing protein 43 OS=Mus musculus GN=Wdr43 PE=1 SV=2 - [WDR43_MOUSE]	-0.16	-0.50	-0.12	-0.47	0.05
Q8R373	CXADR-like membrane protein OS=Mus musculus GN=Clmp PE=1 SV=1 - [CLMP_MOUSE]	-0.38	-0.74	-0.09	-0.44	0.05

Q80Y55	BSD domain-containing protein 1 OS=Mus musculus GN=Bsdc1 PE=2 SV=1 - [BSDC1_MOUSE]	0.14	0.57	0.20	0.63	0.05
Q8BZR6	TOM1-like protein 1 OS=Mus musculus GN=Tom1l1 PE=2 SV=1 - [Q8BZR6_MOUSE]	0.13	0.13	0.26	0.04	0.05
Q08509	Epidermal growth factor receptor kinase substrate 8 OS=Mus musculus GN=Eps8 PE=1 SV=2 - [EPS8_MOUSE]	0.19	0.03	0.26	0.14	0.05
P20033-2	Isoform Short of Platelet-derived growth factor subunit A OS=Mus musculus GN=Pdgfa - [PDGFA_MOUSE]	-1.44	-0.55	-1.14	-0.25	0.05
Q9ET22	Dipeptidyl peptidase 2 OS=Mus musculus GN=Dpp7 PE=2 SV=2 - [DPP2_MOUSE]	-1.52	-0.44	-1.48	-0.41	0.05
Q8R550-5	Isoform 5 of SH3 domain-containing kinase-binding protein 1 OS=Mus musculus GN=Sh3kbp1 - [SH3K1_MOUSE]	-0.67	-0.14	-0.91	-0.37	0.05
Q6PGF5	BMS1 homolog, ribosome assembly protein (Yeast) OS=Mus musculus GN=Bms1 PE=2 SV=1 [Q6PGF5_MOUSE]	-0.56	-0.41	-0.23	-0.09	0.05
D3Z3H4	Cingulin-like protein 1 OS=Mus musculus GN=Cgnl1 PE=2 SV=1 [D3Z3H4_MOUSE]	0.06	0.24	0.30	0.47	0.05
G5E8A7	Protein Smaug homolog 2 OS=Mus musculus GN=Samd4b PE=4 SV=1 - [G5E8A7_MOUSE]	0.18	0.14	0.31	0.56	0.05
Q8CCS6-2	Isoform 2 of Polyadenylate-binding protein 2 OS=Mus musculus GN=Pabpn1 - [PABP2_MOUSE]	-0.04	-0.01	-0.07	-0.03	0.05
Q8K2K6-3	Isoform 3 of Arf-GAP domain and FG repeat-containing protein 1 OS=Mus musculus GN=Agfg1 - [AGFG1_MOUSE]	-0.27	-0.09	-0.59	-0.32	0.05
Q8R086	Sulfite oxidase, mitochondrial OS=Mus musculus GN=Suox PE=1 SV=2 - [SUOX_MOUSE]	-0.13	-0.12	-0.46	-0.45	0.05
Q8CFX3	Protein Pcdh1 OS=Mus musculus GN=Pcdh1 PE=2 SV=1 - [Q8CFX3_MOUSE]	-0.22	-0.35	-0.05	-0.16	0.05

A2AUR3	Phosphotriesterase-related protein (Fragment) OS=Mus musculus GN=Pter PE=2 SV=1 - [A2AUR3_MOUSE]	0.23	0.12	0.17	0.02	0.05
Q924W5	Structural maintenance of chromosomes protein 6 OS=Mus musculus GN=Smc6 PE=2 SV=1 - [SMC6_MOUSE]	-0.16	-0.04	-0.19	-0.07	0.05
Q91Z58	Uncharacterized protein C6orf132 homolog OS=Mus musculus PE=1 SV=2 - [CF132_MOUSE]	-0.16	-0.65	-0.19	-0.51	0.05
Q149F1	RNA pseudouridylate synthase domain-containing protein 2 OS=Mus musculus GN=Rpusd2 PE=2 SV=2 - [RUSD2_MOUSE]	0.03	0.15	0.16	0.27	0.05
Q8BHP7	Olfactomedin-like protein 2A OS=Mus musculus GN=Olfml2a PE=1 SV=1 - [OLM2A_MOUSE]	1.42	0.43	1.34	0.35	0.05
Q9D0R8	Protein LSM12 homolog OS=Mus musculus GN=Lsm12 PE=1 SV=1 - [LSM12_MOUSE]	-0.15	-0.07	-0.38	-0.20	0.05
Q9D809	MCG22941 OS=Mus musculus GN=2200002D01Rik PE=4 SV=1 - [Q9D809_MOUSE]	-0.07	-0.46	-0.77	-0.47	0.05
Q8BJ03-2	Isoform 2 of Cytochrome c oxidase assembly protein COX15 homolog OS=Mus musculus GN=Cox15 - [COX15_MOUSE]	-0.23	-0.09	-0.22	-0.04	0.05
Q62422	Osteoclast-stimulating factor 1 OS=Mus musculus GN=Ostf1 PE=1 SV=2 - [OSTF1_MOUSE]	-0.21	-0.15	-0.14	-0.01	0.05
Q3U9G9	Lamin-B receptor OS=Mus musculus GN=Lbr PE=1 SV=2 - [LBR_MOUSE]	0.16	0.08	0.26	0.06	0.05
Q80TP3	E3 ubiquitin-protein ligase UBR5 OS=Mus musculus GN=Ubr5 PE=1 SV=2 - [UBR5_MOUSE]	0.19	0.22	0.75	0.47	0.05
Q61599	Rho GDP-dissociation inhibitor 2 OS=Mus musculus GN=Arhgdib PE=1 SV=3 - [GDIR2_MOUSE]	0.09	0.20	0.03	0.17	0.05
Q60855	Receptor-interacting serine/threonine-protein kinase 1 OS=Mus musculus GN=Ripk1 PE=1 SV=1 [RIPK1_MOUSE]	0.01	0.12	0.16	0.15	0.05

Q8R574	Phosphoribosyl pyrophosphate synthase-associated protein 2 OS=Mus musculus GN=Prpsap2 PE=1 SV=1 - [KPRB_MOUSE]	-0.20	-0.31	-0.12	-0.05	0.05
Q8BL99-3	Isoform 3 of Protein dopey-1 OS=Mus musculus GN=Dopey1 - [DOP1_MOUSE]	0.10	0.37	0.10	0.36	0.05
Q9QYG0	Protein NDRG2 OS=Mus musculus GN=Ndrg2 PE=1 SV=1 - [NDRG2_MOUSE]	-0.05	-0.14	-0.19	-0.32	0.05
Q9R0M5	Thiamin pyrophosphokinase 1 OS=Mus musculus GN=Tpk1 PE=1 SV=1 - [TPK1_MOUSE]	-0.26	-0.23	-0.17	-0.01	0.05
Q9R0Q6	Actin-related protein 2/3 complex subunit 1A OS=Mus musculus GN=Arpc1a PE=1 SV=1 - [ARC1A_MOUSE]	0.21	0.09	0.07	0.06	0.05
Q9WTX5	S-phase kinase-associated protein 1 OS=Mus musculus GN=Skp1 PE=1 SV=3 - [SKP1_MOUSE]	-0.10	-0.01	-0.15	-0.08	0.05
B2RUJ8	Rho GTPase activating protein 12 OS=Mus musculus GN=Arhgap12 PE=2 SV=1 - [B2RUJ8_MOUSE]	-0.06	-0.02	-0.05	-0.01	0.05
Q9CPT5	Nucleolar protein 16 OS=Mus musculus GN=Nop16 PE=2 SV=1 - [NOP16_MOUSE]	0.12	0.07	0.06	0.01	0.05

Supplementary Table 3. Significantly modulated proteins in the intestine of Crh-/- 7d DSS vs. WT 7d DSS mice (T-Test p-value ≤ 0.05)

Accession	Description	Crh DSS (1)/WT DSS (1)	Crh DSS (1)/ WT DSS (2)	Crh DSS (2)/WT DSS (1)	Crh DSS (2)/ WT DSS (2)	ttest
Q9JKF1	Ras GTPase-activating-like protein IQGAP1 OS=Mus musculus GN=Iqgap1 PE=1 SV=2 - [IQGA1_MOUSE]	-0.65	-0.66	-0.65	-0.66	0.00
G3UVV4	Hexokinase 1, isoform CRA_f OS=Mus musculus GN=Hk1 PE=3 SV=1 - [G3UVV4_MOUSE]	-0.32	-0.31	-0.32	-0.31	0.00
P55292	Desmocollin-2 OS=Mus musculus GN=Dsc2 PE=2 SV=1 - [DSC2_MOUSE]	0.40	0.41	0.41	0.41	0.00
Q8VDN2	Sodium/potassium-transporting ATPase subunit alpha-1 OS=Mus musculus GN=Atp1a1 PE=1 SV=1 - [AT1A1_MOUSE]	-1.03	-1.05	-1.03	-1.03	0.00
P97425	Eosinophil cationic protein 2 OS=Mus musculus GN=Ear2 PE=2 SV=1 - [ECP2_MOUSE]	2.10	2.13	2.08	2.07	0.00
Q8K0E8	Fibrinogen beta chain OS=Mus musculus GN=Fgb PE=2 SV=1 - [FIBB_MOUSE]	1.52	1.51	1.51	1.47	0.00
O35452	Protein Tnxb OS=Mus musculus GN=Tnxb PE=4 SV=1 - [O35452_MOUSE]	-0.25	-0.25	-0.26	-0.25	0.00
Q9Z1W8	Potassium-transporting ATPase alpha chain 2 OS=Mus musculus GN=Atp12a PE=1 SV=3 - [AT12A_MOUSE]	-2.49	-2.42	-2.49	-2.46	0.00
P49290	Eosinophil peroxidase OS=Mus musculus GN=Epx PE=1 SV=2 - [PERE_MOUSE]	1.76	1.77	1.80	1.82	0.00
P31809-3	Isoform 3 of Carcinoembryonic antigen-related cell adhesion molecule 1 OS=Mus musculus GN=Ceacam1 - [CEAM1_MOUSE]	0.35	0.35	0.36	0.35	0.00
Q3UNX5	Acyl-coenzyme A synthetase ACSM3, mitochondrial OS=Mus musculus GN=Acsm3 PE=1 SV=2 - [ACSM3_MOUSE]	-1.73	-1.73	-1.73	-1.67	0.00
A6X935-2	Isoform 2 of Inter alpha-trypsin inhibitor, heavy chain 4 OS=Mus musculus GN=Itih4 - [ITIH4_MOUSE]	1.45	1.51	1.50	1.49	0.00
P09036	Serine protease inhibitor Kazal-type 3 OS=Mus musculus GN=Spink3 PE=1 SV=1 - [ISK3_MOUSE]	-2.21	-2.27	-2.30	-2.31	0.00
P03995-2	Isoform 2 of Glial fibrillary acidic protein OS=Mus musculus GN=Gfap - [GFAP_MOUSE]	1.09	1.13	1.08	1.11	0.00
Q8R0Y6	Cytosolic 10-formyltetrahydrofolate dehydrogenase OS=Mus musculus GN=Aldh1l1 PE=2 SV=1 - [AL1L1_MOUSE]	-1.14	-1.12	-1.12	-1.09	0.00
P48758	Carbonyl reductase [NADPH] 1 OS=Mus musculus GN=Cbr1 PE=1 SV=3 - [CBR1_MOUSE]	-0.53	-0.53	-0.55	-0.54	0.00
A2A5G4	WAP four-disulfide core domain protein 2 OS=Mus musculus GN=Wfdc2 PE=2 SV=1 - [A2A5G4_MOUSE]	-1.88	-1.81	-1.89	-1.83	0.00

Q9CQ75	NADH dehydrogenase [ubiquinone] 1 alpha subcomplex subunit 2 OS=Mus musculus GN=Ndufa2 PE=1 SV=3 - [NDUA2_MOUSE]	-0.62	-0.65	-0.64	-0.63	0.00
Q9WVC8	Chloride anion exchanger OS=Mus musculus GN=Slc26a3 PE=2 SV=1 - [S26A3_MOUSE]	-1.99	-2.05	-2.05	-1.96	0.00
O88322	Nidogen-2 OS=Mus musculus GN=Nid2 PE=1 SV=2 - [NID2_MOUSE]	0.49	0.46	0.49	0.48	0.00
O35387	HCLS1-associated protein X-1 OS=Mus musculus GN=Hax1 PE=1 SV=1 - [HAX1_MOUSE]	0.11	0.11	0.10	0.10	0.00
P47856-2	Isoform 2 of Glutamine--fructose-6-phosphate aminotransferase [isomerizing] 1 OS=Mus musculus GN=Gfpt1 - [GFPT1_MOUSE]	-1.03	-1.03	-0.98	-0.99	0.00
P19221	Prothrombin OS=Mus musculus GN=F2 PE=1 SV=1 - [THRB_MOUSE]	1.03	0.98	1.00	0.98	0.00
Q99KI0	Aconitate hydratase, mitochondrial OS=Mus musculus GN=Aco2 PE=1 SV=1 - [ACON_MOUSE]	-0.27	-0.26	-0.26	-0.27	0.00
Q9JM13	Rab5 GDP/GTP exchange factor OS=Mus musculus GN=Rabgef1 PE=1 SV=1 - [RABX5_MOUSE]	0.40	0.39	0.41	0.39	0.00
Q8CCA5	Protein Apol10a OS=Mus musculus GN=Apol10a PE=2 SV=1 - [Q8CCA5_MOUSE]	1.13	1.08	1.07	1.08	0.00
E9PV24	Protein Fga OS=Mus musculus GN=Fga PE=2 SV=1 - [E9PV24_MOUSE]	1.48	1.49	1.51	1.57	0.00
Q80XU2	Neuronal proto-oncogene tyrosine-protein kinase Src OS=Mus musculus GN=Src PE=2 SV=1 - [Q80XU2_MOUSE]	-0.29	-0.28	-0.28	-0.29	0.00
Q91WG2-1	Isoform 2 of Rab GTPase-binding effector protein 2 OS=Mus musculus GN=Rabep2 - [RABE2_MOUSE]	0.37	0.39	0.39	0.39	0.00
P11087-2	Isoform 2 of Collagen alpha-1(I) chain OS=Mus musculus GN=Col1a1 - [CO1A1_MOUSE]	0.50	0.47	0.48	0.47	0.00
Q9D020-1	Isoform 1 of Cytosolic 5'-nucleotidase 3A OS=Mus musculus GN=Nt5c3a - [5NT3A_MOUSE]	-0.67	-0.70	-0.69	-0.72	0.00
Q61941	NAD(P) transhydrogenase, mitochondrial OS=Mus musculus GN=Nnt PE=1 SV=2 - [NNTM_MOUSE]	-1.06	-1.01	-1.03	-1.08	0.00
Q9D312	Keratin, type I cytoskeletal 20 OS=Mus musculus GN=Krt20 PE=1 SV=1 - [K1C20_MOUSE]	1.49	1.46	1.56	1.50	0.00
D3Z6T3	Cathepsin E OS=Mus musculus GN=Ctse PE=2 SV=1 - [D3Z6T3_MOUSE]	-1.60	-1.52	-1.50	-1.51	0.00
Q8R1F1	Niban-like protein 1 OS=Mus musculus GN=Fam129b PE=1 SV=2 - [NIBL1_MOUSE]	-0.58	-0.55	-0.54	-0.57	0.00

Q08189	Protein-glutamine gamma-glutamyltransferase E OS=Mus musculus GN=Tgm3 PE=1 SV=2 - [TGM3_MOUSE]	-2.75	-2.68	-2.85	-2.86	0.00
Q9WTL8-3	Isoform 3 of Aryl hydrocarbon receptor nuclear translocator-like protein 1 OS=Mus musculus GN=Arntl [BMAL1_MOUSE]	0.46	0.48	0.46	0.48	0.00
F6ZCF0	Cytosolic carboxypeptidase 1 (Fragment) OS=Mus musculus GN=Agtpbp1 PE=4 SV=1 - [F6ZCF0_MOUSE]	-0.44	-0.46	-0.44	-0.46	0.00
Q9QZ85	Interferon-inducible GTPase 1 OS=Mus musculus GN=Iigp1 PE=1 SV=2 - [IIGP1_MOUSE]	0.61	0.57	0.57	0.58	0.00
D3YTT4	Isobutyryl-CoA dehydrogenase, mitochondrial OS=Mus musculus GN=Acad8 PE=2 SV=1 - [D3YTT4_MOUSE]	-0.57	-0.54	-0.57	-0.54	0.00
O70423	Membrane primary amine oxidase OS=Mus musculus GN=Aoc3 PE=2 SV=3 - [AOC3_MOUSE]	0.44	0.44	0.44	0.47	0.00
Q7TPW1	Nexilin OS=Mus musculus GN=Nexn PE=1 SV=3 - [NEXN_MOUSE]	0.16	0.16	0.16	0.17	0.00
O35639	Annexin A3 OS=Mus musculus GN=Anxa3 PE=1 SV=4 - [ANXA3_MOUSE]	-1.01	-0.94	-1.00	-0.99	0.00
O08528	Hexokinase-2 OS=Mus musculus GN=Hk2 PE=1 SV=1 - [HXK2_MOUSE]	-0.80	-0.84	-0.79	-0.79	0.00
Q99KJ8	Dynactin subunit 2 OS=Mus musculus GN=Dctn2 PE=1 SV=3 - [DCTN2_MOUSE]	0.34	0.32	0.34	0.33	0.00
Q9QWG7	Sulfotransferase family cytosolic 1B member 1 OS=Mus musculus GN=Sult1b1 PE=2 SV=2 - [ST1B1_MOUSE]	-1.22	-1.27	-1.18	-1.22	0.00
Q00623	Apolipoprotein A-I OS=Mus musculus GN=Apoa1 PE=1 SV=2 - [APOA1_MOUSE]	1.00	1.00	1.04	1.07	0.00
Q60604	Adseverin OS=Mus musculus GN=Scin PE=1 SV=3 - [ADSV_MOUSE]	-0.91	-0.93	-0.97	-0.97	0.00
Q99J77	N-acetylneuraminic acid synthase (Sialic acid synthase) OS=Mus musculus GN=Nans PE=2 SV=1 - [Q99J77_MOUSE]	-0.76	-0.79	-0.78	-0.82	0.00
Q501J7-2	Isoform 2 of Phosphatase and actin regulator 4 OS=Mus musculus GN=Phactr4 - [PHAR4_MOUSE]	0.78	0.82	0.78	0.83	0.00
Q8BGH2	Sorting and assembly machinery component 50 homolog OS=Mus musculus GN=Samm50 PE=1 SV=1 - [SAM50_MOUSE]	-0.69	-0.64	-0.66	-0.66	0.00
Q9D6R2	Isocitrate dehydrogenase [NAD] subunit alpha, mitochondrial OS=Mus musculus GN=Idh3a PE=1 SV=1 - [IDH3A_MOUSE]	-0.90	-0.87	-0.93	-0.93	0.00
Q06890	Clusterin OS=Mus musculus GN=Clu PE=1 SV=1 - [CLUS_MOUSE]	0.86	0.91	0.91	0.93	0.00

P08074	Carbonyl reductase [NADPH] 2 OS=Mus musculus GN=Cbr2 PE=1 SV=1 - [CBR2_MOUSE]	-0.24	-0.24	-0.24	-0.22	0.00
F8VQC9	NBCn1-G OS=Mus musculus GN=Slc4a7 PE=2 SV=1 - [F8VQC9_MOUSE]	0.45	0.45	0.43	0.46	0.00
P28651	Carbonic anhydrase-related protein OS=Mus musculus GN=Ca8 PE=1 SV=5 - [CAH8_MOUSE]	1.00	1.01	1.07	0.99	0.00
Q8CI94	Glycogen phosphorylase, brain form OS=Mus musculus GN=Pygb PE=1 SV=3 - [PYGB_MOUSE]	-0.76	-0.75	-0.80	-0.80	0.00
Q9D2Q8	Protein S100-A14 OS=Mus musculus GN=S100a14 PE=2 SV=1 - [S10AE_MOUSE]	-1.58	-1.58	-1.51	-1.65	0.00
E9QMC1	Cingulin OS=Mus musculus GN=Cgn PE=2 SV=1 - [E9QMC1_MOUSE]	0.78	0.77	0.83	0.82	0.00
Q9DC69	NADH dehydrogenase [ubiquinone] 1 alpha subcomplex subunit 9, mitochondrial OS=Mus musculus GN=Ndufa9 PE=1 SV=2 - [NDUA9_MOUSE]	-0.68	-0.65	-0.71	-0.70	0.00
P04104	Keratin, type II cytoskeletal 1 OS=Mus musculus GN=Krt1 PE=1 SV=4 - [K2C1_MOUSE]	-1.32	-1.41	-1.34	-1.42	0.00
Q9ER71-2	Isoform 2 of Rho-related GTP-binding protein RhoJ OS=Mus musculus GN=Rhoj - [RHOJ_MOUSE]	-1.00	-1.06	-1.02	-1.08	0.00
Q9JIP7	Solute carrier family 15 member 1 OS=Mus musculus GN=Slc15a1 PE=1 SV=2 - [S15A1_MOUSE]	-2.86	-2.71	-2.95	-2.81	0.00
Q14C51	Pentatricopeptide repeat domain-containing protein 3, mitochondrial OS=Mus musculus GN=Ptcd3 PE=2 SV=2 - [PTCD3_MOUSE]	-0.40	-0.37	-0.41	-0.38	0.00
G5E8R3	Pyruvate carboxylase OS=Mus musculus GN=Pcx PE=3 SV=1 - [G5E8R3_MOUSE]	-1.48	-1.44	-1.57	-1.48	0.00
Q91XL1	Leucine-rich HEV glycoprotein (Precursor) OS=Mus musculus GN=Lrg1 PE=2 SV=1 - [Q91XL1_MOUSE]	1.21	1.22	1.30	1.30	0.00
Q8BIH0-2	Isoform 2 of Histone deacetylase complex subunit SAP130 OS=Mus musculus GN=Sap130 - [SP130_MOUSE]	0.65	0.69	0.65	0.69	0.00
Q62261	Spectrin beta chain, non-erythrocytic 1 OS=Mus musculus GN=Sptbn1 PE=1 SV=2 - [SPTB2_MOUSE]	0.39	0.36	0.39	0.37	0.00
P14094	Sodium/potassium-transporting ATPase subunit beta-1 OS=Mus musculus GN=Atp1b1 PE=1 SV=1 - [AT1B1_MOUSE]	-1.20	-1.31	-1.24	-1.29	0.00
Q9D2C2	Protein SAAL1 OS=Mus musculus GN=Saal1 PE=1 SV=1 - [SAAL1_MOUSE]	-0.28	-0.26	-0.28	-0.27	0.00
P00405	Cytochrome c oxidase subunit 2 OS=Mus musculus GN=Mtco2 PE=1 SV=1 - [COX2_MOUSE]	-0.90	-0.87	-0.89	-0.82	0.00

Q6NVF9	Cleavage and polyadenylation specificity factor subunit 6 OS=Mus musculus GN=Cpsf6 PE=1 SV=1 - [CPSF6_MOUSE]	0.53	0.53	0.54	0.49	0.00
D6RIM8	Harmonin OS=Mus musculus GN=Ush1c PE=2 SV=1 - [D6RIM8_MOUSE]	0.43	0.41	0.45	0.42	0.00
Q8CIE6	Coatomer subunit alpha OS=Mus musculus GN=Copa PE=1 SV=2 - [COPA_MOUSE]	-0.50	-0.46	-0.50	-0.47	0.00
P20060	Beta-hexosaminidase subunit beta OS=Mus musculus GN=Hexb PE=2 SV=2 - [HEXB_MOUSE]	-1.52	-1.42	-1.52	-1.41	0.00
E0CYX7	FXYD domain-containing ion transport regulator 5 (Fragment) OS=Mus musculus GN=Fxyd5 PE=2 SV=1 [E0CYX7_MOUSE]	0.89	0.84	0.93	0.87	0.00
P28650	Adenylosuccinate synthetase isozyme 1 OS=Mus musculus GN=Adssl1 PE=1 SV=2 - [PURA1_MOUSE]	0.87	0.80	0.85	0.82	0.00
Q9Z0H8-2	Isoform 2 of CAP-Gly domain-containing linker protein 2 OS=Mus musculus GN=Clip2 - [CLIP2_MOUSE]	0.60	0.57	0.54	0.57	0.00
Q8R0A0	General transcription factor IIF subunit 2 OS=Mus musculus GN=Gtf2f2 PE=1 SV=1 - [T2FB_MOUSE]	0.73	0.74	0.79	0.79	0.00
Q8CC88	von Willebrand factor A domain-containing protein 8 OS=Mus musculus GN=Vwa8 PE=2 SV=2 - [VWA8_MOUSE]	-0.76	-0.73	-0.80	-0.74	0.00
D3Z3F4	Metaxin-1 (Fragment) OS=Mus musculus GN=Mtx1 PE=4 SV=2 - [D3Z3F4_MOUSE]	-0.72	-0.66	-0.67	-0.67	0.00
Q8R0L1-2	Isoform 2 of Signal-transducing adaptor protein 2 OS=Mus musculus GN=Stap2 - [STAP2_MOUSE]	0.19	0.20	0.18	0.18	0.00
Q99K48	Non-POU domain-containing octamer-binding protein OS=Mus musculus GN=Nono PE=1 SV=3 - [NONO_MOUSE]	1.05	0.97	1.02	0.97	0.00
Q8BKC5-2	Isoform 2 of Importin-5 OS=Mus musculus GN=Ipo5 - [IPO5_MOUSE]	-0.80	-0.81	-0.84	-0.76	0.00
P21550	Beta-enolase OS=Mus musculus GN=Eno3 PE=1 SV=3 - [ENOB_MOUSE]	-1.90	-2.00	-1.81	-1.86	0.00
Q8C3V4	Signal transducer and activator of transcription 1 OS=Mus musculus GN=Stat1 PE=2 SV=1 - [Q8C3V4_MOUSE]	0.83	0.80	0.76	0.83	0.00
Q8K0C5	Zymogen granule membrane protein 16 OS=Mus musculus GN=Zg16 PE=2 SV=1 - [ZG16_MOUSE]	-0.88	-0.90	-0.97	-0.92	0.00
P08226	Apolipoprotein E OS=Mus musculus GN=Apoe PE=1 SV=2 - [APOE_MOUSE]	0.66	0.69	0.63	0.65	0.00
P70362	Ubiquitin fusion degradation protein 1 homolog OS=Mus musculus GN=Ufd1l PE=1 SV=2 - [UFD1_MOUSE]	0.33	0.33	0.31	0.35	0.00

K7N6C2	Protein Cyp2c68 OS=Mus musculus GN=Cyp2c68 PE=3 SV=1 - [K7N6C2_MOUSE]	-2.14	-1.95	-2.05	-1.98	0.00
Q04207-2	Isoform p65 delta of Transcription factor p65 OS=Mus musculus GN=Rela - [TF65_MOUSE]	0.53	0.51	0.56	0.54	0.00
B1ARW4	NADH dehydrogenase [ubiquinone] iron-sulfur protein 5 (Fragment) OS=Mus musculus GN=Ndufs5 PE=2 SV=1 - [B1ARW4_MOUSE]	-0.40	-0.43	-0.44	-0.40	0.00
P47738	Aldehyde dehydrogenase, mitochondrial OS=Mus musculus GN=Aldh2 PE=1 SV=1 - [ALDH2_MOUSE]	-0.91	-0.96	-0.87	-0.90	0.00
P30275	Creatine kinase U-type, mitochondrial OS=Mus musculus GN=Ckmt1 PE=1 SV=1 - [KCRU_MOUSE]	-1.82	-1.93	-1.83	-1.98	0.00
Q9Z0L8-2	Isoform II of Gamma-glutamyl hydrolase OS=Mus musculus GN=Ggh - [GGH_MOUSE]	-2.28	-2.16	-2.39	-2.22	0.00
P11679	Keratin, type II cytoskeletal 8 OS=Mus musculus GN=Krt8 PE=1 SV=4 - [K2C8_MOUSE]	1.33	1.23	1.36	1.28	0.00
P45952	Medium-chain specific acyl-CoA dehydrogenase, mitochondrial OS=Mus musculus GN=Acadm PE=1 SV=1 - [ACADM_MOUSE]	-0.21	-0.20	-0.20	-0.20	0.00
P52196	Thiosulfate sulfurtransferase OS=Mus musculus GN=Tst PE=1 SV=3 - [THTR_MOUSE]	-0.29	-0.27	-0.29	-0.30	0.00
Q3V156	Oxysterol-binding protein OS=Mus musculus GN=Osbpl1a PE=2 SV=1 - [Q3V156_MOUSE]	-0.15	-0.14	-0.15	-0.14	0.00
F7AA26	Protein Gm20459 (Fragment) OS=Mus musculus GN=Gm20459 PE=4 SV=1 - [F7AA26_MOUSE]	0.50	0.49	0.52	0.46	0.00
Q9JII5-2	Isoform 2 of DAZ-associated protein 1 OS=Mus musculus GN=Dazap1 - [DAZP1_MOUSE]	0.62	0.56	0.59	0.57	0.00
Q8CFX1	GDH/6PGL endoplasmic bifunctional protein OS=Mus musculus GN=H6pd PE=2 SV=2 - [G6PE_MOUSE]	0.64	0.71	0.68	0.68	0.00
P00329	Alcohol dehydrogenase 1 OS=Mus musculus GN=Adh1 PE=2 SV=2 - [ADH1_MOUSE]	-2.57	-2.47	-2.74	-2.67	0.00
E9Q912	Protein Rap1gds1 OS=Mus musculus GN=Rap1gds1 PE=2 SV=1 - [E9Q912_MOUSE]	-0.46	-0.51	-0.50	-0.52	0.00
Q8R123	FAD synthase OS=Mus musculus GN=Flad1 PE=1 SV=1 - [FAD1_MOUSE]	-1.05	-1.03	-0.98	-0.95	0.00
Q64516-1	Isoform 1 of Glycerol kinase OS=Mus musculus GN=Gk - [GLPK_MOUSE]	-1.95	-1.76	-1.82	-1.82	0.00
Q9D7Z3-2	Isoform 2 of Nucleolar protein 7 OS=Mus musculus GN=Nol7 - [NOL7_MOUSE]	0.45	0.43	0.48	0.46	0.00

Q9DCS2	UPF0585 protein C16orf13 homolog OS=Mus musculus PE=1 SV=1 - [CP013_MOUSE]	-0.53	-0.49	-0.52	-0.48	0.00
P06909	Complement factor H OS=Mus musculus GN=Cfh PE=1 SV=2 - [CFAH_MOUSE]	1.43	1.42	1.35	1.51	0.00
A8DUK4	Beta-globin OS=Mus musculus GN=Hbb-b1 PE=2 SV=1 - [A8DUK4_MOUSE]	-1.35	-1.35	-1.48	-1.38	0.00
P61922	4-aminobutyrate aminotransferase, mitochondrial OS=Mus musculus GN=Abat PE=1 SV=1 - [GABT_MOUSE]	-0.93	-0.94	-1.00	-1.02	0.00
P23780	Beta-galactosidase OS=Mus musculus GN=Glb1 PE=2 SV=1 - [BGAL_MOUSE]	-0.70	-0.71	-0.74	-0.77	0.00
Q68FD5	Clathrin heavy chain 1 OS=Mus musculus GN=Cltc PE=1 SV=3 - [CLH1_MOUSE]	-0.31	-0.31	-0.33	-0.34	0.00
Q6ZWY8	Thymosin beta-10 OS=Mus musculus GN=Tmsb10 PE=2 SV=3 - [TYB10_MOUSE]	1.13	1.08	1.21	1.16	0.00
Q9QX47-3	Isoform 3 of Protein SON OS=Mus musculus GN=Son - [SON_MOUSE]	0.63	0.68	0.69	0.71	0.00
P56392	Cytochrome c oxidase subunit 7A1, mitochondrial OS=Mus musculus GN=Cox7a1 PE=2 SV=1 - [CX7A1_MOUSE]	-1.07	-1.14	-1.12	-1.20	0.00
P14733	Lamin-B1 OS=Mus musculus GN=Lmnb1 PE=1 SV=3 - [LMNB1_MOUSE]	0.46	0.45	0.48	0.50	0.00
P20152	Vimentin OS=Mus musculus GN=Vim PE=1 SV=3 - [VIME_MOUSE]	0.70	0.68	0.76	0.70	0.00
O88312	Anterior gradient protein 2 homolog OS=Mus musculus GN=Agr2 PE=2 SV=1 - [AGR2_MOUSE]	0.38	0.35	0.35	0.35	0.00
Q8CIB5	Fermitin family homolog 2 OS=Mus musculus GN=Fermt2 PE=1 SV=1 - [FERM2_MOUSE]	-0.51	-0.47	-0.53	-0.51	0.00
D3Z0Y2	Peroxiredoxin-6 OS=Mus musculus GN=Prdx6 PE=2 SV=1 - [D3Z0Y2_MOUSE]	-1.52	-1.60	-1.43	-1.53	0.00
P06801	NADP-dependent malic enzyme OS=Mus musculus GN=Me1 PE=1 SV=2 - [MAOX_MOUSE]	0.81	0.79	0.77	0.86	0.00
Q61152	Tyrosine-protein phosphatase non-receptor type 18 OS=Mus musculus GN=Ptpn18 PE=1 SV=1 - [PTN18_MOUSE]	1.07	1.01	1.13	1.06	0.00
Q9WUG6	Insulin-like peptide INSL5 OS=Mus musculus GN=Insl5 PE=2 SV=1 - [INSL5_MOUSE]	-2.54	-2.33	-2.61	-2.50	0.00
P19001	Keratin, type I cytoskeletal 19 OS=Mus musculus GN=Krt19 PE=1 SV=1 - [K1C19_MOUSE]	0.96	0.90	1.01	0.97	0.00

Q07456	Protein AMBP OS=Mus musculus GN=Ambp PE=1 SV=2 - [AMBP_MOUSE]	1.51	1.38	1.37	1.43	0.00
E9Q7G0	Protein Numa1 OS=Mus musculus GN=Numa1 PE=2 SV=1 - [E9Q7G0_MOUSE]	0.79	0.83	0.88	0.88	0.00
Q8K310	Matrin-3 OS=Mus musculus GN=Matr3 PE=1 SV=1 - [MATR3_MOUSE]	0.42	0.42	0.46	0.42	0.00
Q9EQG9-2	Isoform 2 of Collagen type IV alpha-3-binding protein OS=Mus musculus GN=Col4a3bp - [C43BP_MOUSE]	-0.54	-0.54	-0.48	-0.52	0.00
Q3TA68	Protein Wdr36 OS=Mus musculus GN=Wdr36 PE=2 SV=1 - [Q3TA68_MOUSE]	0.48	0.46	0.52	0.49	0.00
F8WJ93	Echinoderm microtubule-associated protein-like 4 OS=Mus musculus GN=Eml4 PE=2 SV=1 - [F8WJ93_MOUSE]	0.38	0.35	0.36	0.33	0.00
Q5SVG5	AP-1 complex subunit beta-1 OS=Mus musculus GN=Ap1b1 PE=2 SV=1 - [Q5SVG5_MOUSE]	-0.32	-0.29	-0.29	-0.31	0.00
Q91X72	Hemopexin OS=Mus musculus GN=Hpx PE=1 SV=2 - [HEMO_MOUSE]	1.21	1.24	1.31	1.34	0.00
Q9JLB0-2	Isoform Alpha of MAGUK p55 subfamily member 6 OS=Mus musculus GN=Mpp6 - [MPP6_MOUSE]	1.01	1.01	0.93	0.92	0.00
Q9EPU4	Cleavage and polyadenylation specificity factor subunit 1 OS=Mus musculus GN=Cpsf1 PE=1 SV=1 - [CPSF1_MOUSE]	0.91	0.98	0.87	0.93	0.00
G5E8J4	Protein tyrosine phosphatase, non-receptor type 21, isoform CRA_a OS=Mus musculus GN=Ptpn21 PE=4 SV=1 - [G5E8J4_MOUSE]	-1.08	-1.17	-1.11	-1.20	0.00
Q8R071	Inositol-trisphosphate 3-kinase A OS=Mus musculus GN=Itpka PE=2 SV=1 - [IP3KA_MOUSE]	-0.90	-0.98	-0.92	-1.00	0.00
Q9D6J5	NADH dehydrogenase [ubiquinone] 1 beta subcomplex subunit 8, mitochondrial OS=Mus musculus GN=Ndufb8 PE=1 SV=1 - [NDUB8_MOUSE]	-0.45	-0.41	-0.40	-0.42	0.00
Q64444	Carbonic anhydrase 4 OS=Mus musculus GN=Ca4 PE=1 SV=1 - [CAH4_MOUSE]	-1.76	-1.68	-1.89	-1.76	0.00
P11688	Integrin alpha-5 OS=Mus musculus GN=Itga5 PE=1 SV=3 - [ITA5_MOUSE]	-0.66	-0.70	-0.65	-0.62	0.00
Q9D939	Sulfotransferase 1C2 OS=Mus musculus GN=Sult1c2 PE=1 SV=1 - [ST1C2_MOUSE]	-0.56	-0.58	-0.59	-0.53	0.00
Q3UW53	Protein Niban OS=Mus musculus GN=Fam129a PE=1 SV=2 - [NIBAN_MOUSE]	-0.56	-0.50	-0.51	-0.54	0.00
Q9ERS2	NADH dehydrogenase [ubiquinone] 1 alpha subcomplex subunit 13 OS=Mus musculus GN=Ndufa13 PE=1 SV=3 - [NDUAD_MOUSE]	-0.67	-0.74	-0.73	-0.75	0.00

Q9CZ13	Cytochrome b-c1 complex subunit 1, mitochondrial OS=Mus musculus GN=Uqcrc1 PE=1 SV=2 - [QCR1_MOUSE]	-0.43	-0.38	-0.40	-0.38	0.00
Q4FZH1	Protein FAM189A2 OS=Mus musculus GN=Fam189a2 PE=1 SV=2 - [F1892_MOUSE]	-0.48	-0.44	-0.49	-0.46	0.00
P16301	Phosphatidylcholine-sterol acyltransferase OS=Mus musculus GN=Lcat PE=1 SV=2 - [LCAT_MOUSE]	0.76	0.83	0.76	0.83	0.00
P01029	Complement C4-B OS=Mus musculus GN=C4b PE=1 SV=3 - [CO4B_MOUSE]	0.93	1.05	0.97	1.01	0.00
Q8R088	Golgi phosphoprotein 3-like OS=Mus musculus GN=Golph3l PE=2 SV=1 - [GLP3L_MOUSE]	-0.52	-0.54	-0.50	-0.57	0.00
Q923L7	Ear6 protein OS=Mus musculus GN=Ear6 PE=2 SV=1 - [Q923L7_MOUSE]	2.95	2.72	3.06	2.83	0.00
Q6ZQ38	Cullin-associated NEDD8-dissociated protein 1 OS=Mus musculus GN=Cand1 PE=2 SV=2 - [CAND1_MOUSE]	-0.91	-0.88	-0.84	-0.81	0.00
Q8BL66	Early endosome antigen 1 OS=Mus musculus GN=Eea1 PE=1 SV=2 - [EEA1_MOUSE]	0.32	0.33	0.34	0.36	0.00
Q3TBT3-3	Isoform 3 of Stimulator of interferon genes protein OS=Mus musculus GN=Tmem173 - [STING_MOUSE]	0.80	0.72	0.79	0.74	0.00
Q8BI72	CDKN2A-interacting protein OS=Mus musculus GN=Cdkn2aip PE=2 SV=1 - [CARF_MOUSE]	0.98	0.90	0.96	0.88	0.00
Q9NYQ2	Hydroxyacid oxidase 2 OS=Mus musculus GN=Hao2 PE=2 SV=1 - [HAOX2_MOUSE]	-0.81	-0.74	-0.73	-0.72	0.00
B7ZNH7	Collagen alpha-1(XIV) chain OS=Mus musculus GN=Col14a1 PE=2 SV=1 - [B7ZNH7_MOUSE]	1.03	1.16	1.08	1.11	0.00
Q56A08	G patch domain and KOW motifs-containing protein OS=Mus musculus GN=Gpkow PE=2 SV=2 - [GPKOW_MOUSE]	0.73	0.72	0.69	0.65	0.00
Q9JKS4-3	Isoform 3 of LIM domain-binding protein 3 OS=Mus musculus GN=Ldb3 - [LDB3_MOUSE]	0.37	0.33	0.34	0.34	0.00
Q9CZ96-2	Isoform 2 of Zinc finger CCHC-type and RNA-binding motif-containing protein 1 OS=Mus musculus GN=Zcrb1 - [ZCRB1_MOUSE]	0.54	0.51	0.50	0.47	0.00
P08905	Lysozyme C-2 OS=Mus musculus GN=Lyz2 PE=1 SV=2 - [LYZ2_MOUSE]	1.10	1.02	1.13	1.15	0.00
Q8BQB4	Four-jointed box protein 1 OS=Mus musculus GN=Fjx1 PE=1 SV=1 - [FJX1_MOUSE]	0.97	0.90	0.93	0.85	0.00
O70209	PDZ and LIM domain protein 3 OS=Mus musculus GN=Pdlim3 PE=1 SV=1 - [PDLI3_MOUSE]	0.33	0.37	0.33	0.35	0.00

O54941	SWI/SNF-related matrix-associated actin-dependent regulator of chromatin subfamily E member 1 OS=Mus musculus GN=Smarce1 PE=1 SV=1 - [SMCE1_MOUSE]	0.84	0.76	0.78	0.75	0.00
Q08879	Fibulin-1 OS=Mus musculus GN=Fbln1 PE=1 SV=2 - [FBLN1_MOUSE]	-0.92	-0.85	-0.88	-0.81	0.00
Q8BG07-2	Isoform 2 of Phospholipase D4 OS=Mus musculus GN=Pld4 - [PLD4_MOUSE]	0.40	0.37	0.42	0.41	0.00
Q8CIM3	D-2-hydroxyglutarate dehydrogenase, mitochondrial OS=Mus musculus GN=D2hgdh PE=2 SV=3 - [D2HDH_MOUSE]	-0.97	-1.02	-0.90	-0.95	0.00
Q3UW68	Calpain-13 OS=Mus musculus GN=Capn13 PE=2 SV=1 - [CAN13_MOUSE]	-2.06	-2.12	-1.89	-1.92	0.00
Q8VCC2	Liver carboxylesterase 1 OS=Mus musculus GN=Ces1 PE=2 SV=1 - [EST1_MOUSE]	1.32	1.44	1.27	1.38	0.00
Q7TMF3	NADH dehydrogenase [ubiquinone] 1 alpha subcomplex subunit 12 OS=Mus musculus GN=Ndufa12 PE=1 SV=2 - [NDUAC_MOUSE]	-0.62	-0.61	-0.69	-0.64	0.00
G3UW94	MCG14259, isoform CRA_b OS=Mus musculus GN=U2af1 PE=4 SV=1 - [G3UW94_MOUSE]	0.49	0.47	0.51	0.45	0.00
P47963	60S ribosomal protein L13 OS=Mus musculus GN=Rpl13 PE=2 SV=3 - [RL13_MOUSE]	0.74	0.75	0.79	0.84	0.00
P05784	Keratin, type I cytoskeletal 18 OS=Mus musculus GN=Krt18 PE=1 SV=5 - [K1C18_MOUSE]	0.96	0.92	0.85	0.95	0.00
O88329	Unconventional myosin-Ia OS=Mus musculus GN=Myo1a PE=2 SV=2 - [MYO1A_MOUSE]	-0.76	-0.70	-0.79	-0.79	0.00
Q504M2	MCG53395 OS=Mus musculus GN=Pdp2 PE=2 SV=1 - [Q504M2_MOUSE]	-1.46	-1.34	-1.40	-1.28	0.00
Q3UP87	Neutrophil elastase OS=Mus musculus GN=Elane PE=2 SV=1 - [ELNE_MOUSE]	2.57	2.75	2.39	2.56	0.00
Q9DCV7	Keratin, type II cytoskeletal 7 OS=Mus musculus GN=Krt7 PE=1 SV=1 - [K2C7_MOUSE]	1.08	1.02	1.15	1.16	0.00
Q9WV91	Prostaglandin F2 receptor negative regulator OS=Mus musculus GN=Ptgfrn PE=1 SV=2 - [FPRP_MOUSE]	-0.33	-0.30	-0.33	-0.34	0.00
O55222	Integrin-linked protein kinase OS=Mus musculus GN=Ilk PE=1 SV=2 - [ILK_MOUSE]	-0.65	-0.70	-0.61	-0.65	0.00
Q9JLV5	Cullin-3 OS=Mus musculus GN=Cul3 PE=1 SV=1 - [CUL3_MOUSE]	-0.35	-0.41	-0.37	-0.38	0.00
Q9EQP2	EH domain-containing protein 4 OS=Mus musculus GN=Ehd4 PE=1 SV=1 - [EHD4_MOUSE]	-0.37	-0.40	-0.40	-0.42	0.00

Q9CY64	Biliverdin reductase A OS=Mus musculus GN=Blvra PE=2 SV=1 - [BIEA_MOUSE]	-0.59	-0.58	-0.66	-0.62	0.00
Q9DCJ5	NADH dehydrogenase [ubiquinone] 1 alpha subcomplex subunit 8 OS=Mus musculus GN=Ndufa8 PE=1 SV=3 - [NDUA8_MOUSE]	-0.84	-0.85	-0.80	-0.75	0.00
Q6P2K6	Serine/threonine-protein phosphatase 4 regulatory subunit 3A OS=Mus musculus GN=Smek1 PE=1 SV=1 [P4R3A_MOUSE]	1.64	1.66	1.81	1.83	0.00
Q9D2X6	Colon SVA-like protein OS=Mus musculus GN=Sval1 PE=2 SV=1 - [Q9D2X6_MOUSE]	-3.14	-2.82	-3.05	-2.79	0.00
Q62395	Trefoil factor 3 OS=Mus musculus GN=Tff3 PE=1 SV=2 - [TFF3_MOUSE]	-0.91	-0.92	-0.99	-1.03	0.00
Q8K0J2	UDP-GlcNAc:betaGal beta-1,3-N-acetylglucosaminyltransferase 7 OS=Mus musculus GN=B3gnt7 PE=2 SV=2 - [B3GN7_MOUSE]	-2.10	-2.13	-2.28	-2.38	0.00
Q8BH59	Calcium-binding mitochondrial carrier protein Aralar1 OS=Mus musculus GN=Slc25a12 PE=1 SV=1 - [CMC1_MOUSE]	-0.56	-0.53	-0.59	-0.52	0.00
Q9CPP6	NADH dehydrogenase [ubiquinone] 1 alpha subcomplex subunit 5 OS=Mus musculus GN=Ndufa5 PE=1 SV=3 - [NDUA5_MOUSE]	-0.88	-0.99	-0.95	-0.89	0.00
Q61703	Inter-alpha-trypsin inhibitor heavy chain H2 OS=Mus musculus GN=Itih2 PE=1 SV=1 - [ITIH2_MOUSE]	1.38	1.37	1.21	1.36	0.00
Q9ESB3	Histidine-rich glycoprotein OS=Mus musculus GN=Hrg PE=1 SV=2 - [HRG_MOUSE]	0.94	1.03	0.93	1.04	0.00
Q8R3K3	Pentatricopeptide repeat-containing protein 2, mitochondrial OS=Mus musculus GN=Ptcd2 PE=2 SV=1 - [PTCD2_MOUSE]	-0.39	-0.41	-0.36	-0.38	0.00
Q8K154	Protein Ugt2b34 OS=Mus musculus GN=Ugt2b34 PE=2 SV=1 - [Q8K154_MOUSE]	-0.66	-0.60	-0.60	-0.57	0.00
P10404	MLV-related proviral Env polyprotein OS=Mus musculus PE=2 SV=3 - [ENV1_MOUSE]	-1.63	-1.49	-1.72	-1.60	0.00
Q8BSZ2	AP-3 complex subunit sigma-2 OS=Mus musculus GN=Ap3s2 PE=1 SV=1 - [AP3S2_MOUSE]	0.36	0.32	0.35	0.31	0.00
Q148B1	Cytochrome P450, family 2, subfamily c, polypeptide 65 OS=Mus musculus GN=Cyp2c65 PE=2 SV=1 - [Q148B1_MOUSE]	1.53	1.63	1.51	1.72	0.00
Q69Z69	N-acetyltransferase ESCO1 OS=Mus musculus GN=Esco1 PE=2 SV=2 - [ESCO1_MOUSE]	-0.72	-0.76	-0.81	-0.72	0.00
Q922Q1	MOSC domain-containing protein 2, mitochondrial OS=Mus musculus GN=Marc2 PE=1 SV=1 - [MOSC2_MOUSE]	-0.84	-0.80	-0.89	-0.78	0.00
Q8K419	Galectin-4 OS=Mus musculus GN=Lgals4 PE=1 SV=2 - [LEG4_MOUSE]	-0.74	-0.66	-0.75	-0.68	0.00

Q9EQW7	Kinesin-like protein KIF13A OS=Mus musculus GN=Kif13a PE=1 SV=1 - [KI13A_MOUSE]	-1.12	-1.00	-1.01	-0.98	0.00
P08071	Lactotransferrin OS=Mus musculus GN=Ltf PE=2 SV=4 - [TRFL_MOUSE]	1.74	1.84	1.97	1.98	0.00
P12246	Serum amyloid P-component OS=Mus musculus GN=Apcs PE=1 SV=2 - [SAMP_MOUSE]	2.49	2.24	2.51	2.26	0.00
G5E8L8	Mitogen activated protein kinase kinase kinase 2 OS=Mus musculus GN=Map3k2 PE=4 SV=1 - [G5E8L8_MOUSE]	-1.18	-1.25	-1.08	-1.15	0.00
Q99P91	Transmembrane glycoprotein NMB OS=Mus musculus GN=Gpnmb PE=1 SV=2 - [GPNMB_MOUSE]	-1.55	-1.39	-1.57	-1.42	0.00
Q80YC5	Coagulation factor XII OS=Mus musculus GN=F12 PE=2 SV=2 - [FA12_MOUSE]	0.92	0.93	1.02	1.03	0.00
P41245	Matrix metalloproteinase-9 OS=Mus musculus GN=Mmp9 PE=2 SV=2 - [MMP9_MOUSE]	2.23	2.02	2.16	1.95	0.00
Q8BW75	Amine oxidase [flavin-containing] B OS=Mus musculus GN=Maob PE=1 SV=4 - [AOFB_MOUSE]	-0.36	-0.40	-0.35	-0.35	0.00
Q9CQA6	Coiled-coil-helix-coiled-coil-helix domain-containing protein 1 OS=Mus musculus GN=Chchd1 PE=2 SV=1 - [CHCH1_MOUSE]	-0.49	-0.51	-0.44	-0.46	0.00
D3Z5X8	Cell growth-regulating nucleolar protein (Fragment) OS=Mus musculus GN=Lyar PE=2 SV=1 - [D3Z5X8_MOUSE]	0.39	0.40	0.34	0.37	0.00
Q9DBT4-2	Isoform 2 of Ectonucleoside triphosphate diphosphohydrolase 4 OS=Mus musculus GN=Entpd4 - [ENTP4_MOUSE]	-0.63	-0.65	-0.60	-0.56	0.00
P19096	Fatty acid synthase OS=Mus musculus GN=Fasn PE=1 SV=2 - [FAS_MOUSE]	-0.54	-0.46	-0.51	-0.48	0.00
Q3ULJ0-2	Isoform 2 of Glycerol-3-phosphate dehydrogenase 1-like protein OS=Mus musculus GN=Gpd1l - [GPD1L_MOUSE]	-0.77	-0.71	-0.67	-0.69	0.00
Q8CGN5	Perilipin-1 OS=Mus musculus GN=Plin1 PE=1 SV=2 - [PLIN1_MOUSE]	1.51	1.43	1.38	1.30	0.00
Q8BTI8-3	Isoform 3 of Serine/arginine repetitive matrix protein 2 OS=Mus musculus GN=Srrm2 - [SRRM2_MOUSE]	0.83	0.72	0.82	0.81	0.00
Q8VHX6	Filamin-C OS=Mus musculus GN=Flnc PE=1 SV=3 - [FLNC_MOUSE]	-0.63	-0.66	-0.71	-0.73	0.00
Q9CQW1	Synaptobrevin homolog YKT6 OS=Mus musculus GN=Ykt6 PE=2 SV=1 - [YKT6_MOUSE]	-0.31	-0.27	-0.31	-0.28	0.00
P09242	Alkaline phosphatase, tissue-nonspecific isozyme OS=Mus musculus GN=Alpl PE=1 SV=2 - [PPBT_MOUSE]	1.11	1.14	1.24	1.26	0.00

Q9Z1N5	Spliceosome RNA helicase Ddx39b OS=Mus musculus GN=Ddx39b PE=1 SV=1 - [DX39B_MOUSE]	-0.52	-0.47	-0.45	-0.49	0.00
Q9CY57-3	Isoform 3 of Chromatin target of PRMT1 protein OS=Mus musculus GN=Chtop - [CHTOP_MOUSE]	0.81	0.92	0.86	0.93	0.00
Q9DB20	ATP synthase subunit O, mitochondrial OS=Mus musculus GN=Atp5o PE=1 SV=1 - [ATPO_MOUSE]	-0.41	-0.47	-0.42	-0.43	0.00
Q9D8C9	Purine nucleoside phosphorylase OS=Mus musculus GN=Pnp2 PE=2 SV=1 - [Q9D8C9_MOUSE]	1.09	1.14	1.12	1.25	0.00
Q8BJ64	Choline dehydrogenase, mitochondrial OS=Mus musculus GN=Chdh PE=1 SV=1 - [CHDH_MOUSE]	-0.82	-0.77	-0.82	-0.90	0.00
Q922U1	U4/U6 small nuclear ribonucleoprotein Prp3 OS=Mus musculus GN=Prpf3 PE=1 SV=1 - [PRPF3_MOUSE]	0.47	0.41	0.46	0.44	0.00
A2ARZ3	Fibrous sheath-interacting protein 2 OS=Mus musculus GN=Fsip2 PE=1 SV=3 - [FSIP2_MOUSE]	-0.23	-0.25	-0.22	-0.22	0.00
Q9JMH9-1	Isoform 1 of Unconventional myosin-XVIIIa OS=Mus musculus GN=Myo18a - [MY18A_MOUSE]	0.43	0.40	0.43	0.38	0.00
Q6ZQK5	Arf-GAP with coiled-coil, ANK repeat and PH domain-containing protein 2 OS=Mus musculus GN=Acap2 PE=1 SV=2 - [ACAP2_MOUSE]	-0.33	-0.33	-0.30	-0.35	0.00
Q9Z2M7	Phosphomannomutase 2 OS=Mus musculus GN=Pmm2 PE=2 SV=1 - [PMM2_MOUSE]	-0.43	-0.38	-0.38	-0.37	0.00
O08692	Myeloid bactenecin (F1) OS=Mus musculus GN=Ngp PE=2 SV=1 - [O08692_MOUSE]	1.70	1.92	1.76	1.94	0.00
Q9ER73	Elongator complex protein 4 OS=Mus musculus GN=Elp4 PE=2 SV=2 - [ELP4_MOUSE]	0.66	0.73	0.76	0.77	0.00
Q9Z1A1	Protein Tfg OS=Mus musculus GN=Tfg PE=2 SV=1 - [Q9Z1A1_MOUSE]	0.34	0.37	0.34	0.38	0.00
Q9R1C7	Pre-mRNA-processing factor 40 homolog A OS=Mus musculus GN=Prpf40a PE=1 SV=1 - [PR40A_MOUSE]	0.71	0.76	0.83	0.78	0.00
P61759	Prefoldin subunit 3 OS=Mus musculus GN=Vbp1 PE=2 SV=2 - [PFD3_MOUSE]	0.24	0.28	0.27	0.28	0.00
Q07813	Apoptosis regulator BAX OS=Mus musculus GN=Bax PE=1 SV=1 - [BAX_MOUSE]	-0.39	-0.35	-0.35	-0.40	0.00
Q02819	Nucleobindin-1 OS=Mus musculus GN=Nucb1 PE=1 SV=2 - [NUCB1_MOUSE]	0.23	0.22	0.25	0.22	0.00
A2APM2	CD44 antigen OS=Mus musculus GN=Cd44 PE=2 SV=1 - [A2APM2_MOUSE]	0.53	0.60	0.56	0.52	0.00

Q91VW5	Golgin subfamily A member 4 OS=Mus musculus GN=Golga4 PE=1 SV=2 - [GOGA4_MOUSE]	0.24	0.22	0.24	0.21	0.00
G5E850	Cytochrome b-5, isoform CRA_a OS=Mus musculus GN=Cyb5 PE=3 SV=1 - [G5E850_MOUSE]	0.39	0.39	0.43	0.44	0.00
Q5SS80-2	Isoform 2 of Dehydrogenase/reductase SDR family member 13 OS=Mus musculus GN=Dhrs13 - [DHR13_MOUSE]	1.23	1.23	1.10	1.10	0.00
Q8BML9	Glutaminyl-tRNA synthetase OS=Mus musculus GN=Qars PE=2 SV=1 - [Q8BML9_MOUSE]	-0.27	-0.23	-0.25	-0.27	0.00
P70124	Serpin B5 OS=Mus musculus GN=Serpinb5 PE=2 SV=1 - [SPB5_MOUSE]	-0.96	-0.98	-0.84	-0.92	0.00
Q8CH18-3	Isoform 3 of Cell division cycle and apoptosis regulator protein 1 OS=Mus musculus GN=Ccar1 - [CCAR1_MOUSE]	0.52	0.60	0.52	0.54	0.00
Q9QXX4	Calcium-binding mitochondrial carrier protein Aralar2 OS=Mus musculus GN=Slc25a13 PE=1 SV=1 - [CMC2_MOUSE]	-0.82	-0.72	-0.83	-0.78	0.00
O88512	AP-1 complex subunit gamma-like 2 OS=Mus musculus GN=Ap1g2 PE=2 SV=2 - [AP1G2_MOUSE]	-0.27	-0.24	-0.28	-0.25	0.00
Q8VIJ6	Splicing factor, proline- and glutamine-rich OS=Mus musculus GN=Sfpq PE=1 SV=1 - [SFPQ_MOUSE]	1.01	0.98	1.14	1.07	0.00
O70404	Vesicle-associated membrane protein 8 OS=Mus musculus GN=Vamp8 PE=1 SV=1 - [VAMP8_MOUSE]	0.89	0.80	0.90	0.80	0.00
E9Q421	GPI-anchor transamidase OS=Mus musculus GN=Pigk PE=2 SV=1 - [E9Q421_MOUSE]	-0.27	-0.30	-0.30	-0.32	0.00
A3KGB4	TBC1 domain family member 8B OS=Mus musculus GN=Tbc1d8b PE=2 SV=1 - [TBC8B_MOUSE]	-0.43	-0.50	-0.48	-0.49	0.00
G3UX26	Voltage-dependent anion-selective channel protein 2 (Fragment) OS=Mus musculus GN=Vdac2 PE=4 SV=1 [G3UX26_MOUSE]	-0.84	-0.81	-0.72	-0.77	0.00
D3YXV1	Indoleamine 2,3-dioxygenase 1 OS=Mus musculus GN=Ido1 PE=2 SV=1 - [D3YXV1_MOUSE]	0.92	0.98	0.87	1.01	0.00
D3YZN4	Paraplegin OS=Mus musculus GN=Spg7 PE=2 SV=1 - [D3YZN4_MOUSE]	-0.61	-0.71	-0.70	-0.68	0.00
Q8VEM8	Phosphate carrier protein, mitochondrial OS=Mus musculus GN=Slc25a3 PE=1 SV=1 - [MPCP_MOUSE]	-0.67	-0.79	-0.72	-0.74	0.00
Q1HFZ0	tRNA (cytosine(34)-C(5))-methyltransferase OS=Mus musculus GN=Nsun2 PE=1 SV=2 - [NSUN2_MOUSE]	-0.17	-0.20	-0.20	-0.19	0.00
Q8BX02	KN motif and ankyrin repeat domain-containing protein 2 OS=Mus musculus GN=Kank2 PE=1 SV=1 - [KANK2_MOUSE]	-0.36	-0.37	-0.42	-0.36	0.00

M0QWG8	CAS1 domain-containing protein 1 (Fragment) OS=Mus musculus GN=Casd1 PE=4 SV=1 - [M0QWG8_MOUSE]	-0.82	-0.73	-0.85	-0.77	0.00
Q99K01-2	Isoform 2 of Pyridoxal-dependent decarboxylase domain-containing protein 1 OS=Mus musculus GN=Pdxdc1 - [PDXD1_MOUSE]	-0.17	-0.15	-0.15	-0.15	0.00
Q62159	Rho-related GTP-binding protein RhoC OS=Mus musculus GN=Rhoc PE=1 SV=2 - [RHOC_MOUSE]	-0.95	-0.99	-0.85	-0.89	0.00
B1ATZ0	Hepatocyte growth factor-regulated tyrosine kinase substrate OS=Mus musculus GN=Hgs PE=2 SV=1 - [B1ATZ0_MOUSE]	0.30	0.30	0.27	0.32	0.00
Q8VCN5	Cystathionine gamma-lyase OS=Mus musculus GN=Cth PE=1 SV=1 - [CGL_MOUSE]	0.25	0.21	0.24	0.24	0.00
Q8VDM6-2	Isoform 2 of Heterogeneous nuclear ribonucleoprotein U-like protein 1 OS=Mus musculus GN=Hnrnpul1 - [HNRL1_MOUSE]	0.60	0.65	0.59	0.68	0.00
Q99JY8	Lipid phosphate phosphohydrolase 3 OS=Mus musculus GN=Ppap2b PE=1 SV=1 - [LPP3_MOUSE]	0.53	0.48	0.52	0.46	0.00
O88533	Aromatic-L-amino-acid decarboxylase OS=Mus musculus GN=Ddc PE=2 SV=1 - [DDC_MOUSE]	-1.29	-1.16	-1.29	-1.14	0.00
P01027	Complement C3 OS=Mus musculus GN=C3 PE=1 SV=3 - [CO3_MOUSE]	1.32	1.34	1.46	1.52	0.00
Q9D3P8	Plasminogen receptor (KT) OS=Mus musculus GN=Plgrkt PE=1 SV=1 - [PLRKT_MOUSE]	-1.13	-1.22	-1.09	-1.03	0.00
F8VQH0	Abelson tyrosine-protein kinase 2 OS=Mus musculus GN=Abl2 PE=2 SV=1 - [F8VQH0_MOUSE]	-0.47	-0.51	-0.43	-0.47	0.00
A2A7R1	Transmembrane protein 54 OS=Mus musculus GN=Tmem54 PE=4 SV=1 - [A2A7R1_MOUSE]	-0.93	-0.90	-1.04	-1.01	0.00
Q80XN0	D-beta-hydroxybutyrate dehydrogenase, mitochondrial OS=Mus musculus GN=Bdh1 PE=1 SV=2 - [BDH_MOUSE]	-0.55	-0.49	-0.48	-0.47	0.00
Q9CQY5-2	Isoform 2 of Magnesium transporter protein 1 OS=Mus musculus GN=Magt1 - [MAGT1_MOUSE]	-0.33	-0.29	-0.34	-0.32	0.00
Q6IFX2	Keratin, type I cytoskeletal 42 OS=Mus musculus GN=Krt42 PE=1 SV=1 - [K1C42_MOUSE]	1.00	1.01	0.89	0.90	0.00
Q8VBT6	Apolipoprotein B receptor OS=Mus musculus GN=Apobr PE=1 SV=1 - [APOBR_MOUSE]	0.16	0.18	0.15	0.16	0.00
Q05117	Tartrate-resistant acid phosphatase type 5 OS=Mus musculus GN=Acp5 PE=2 SV=2 - [PPA5_MOUSE]	-1.19	-1.07	-1.18	-1.04	0.00
B2RXS4	Plexin-B2 OS=Mus musculus GN=Plxnb2 PE=1 SV=1 - [PLXB2_MOUSE]	-0.41	-0.44	-0.39	-0.46	0.00

Q11127-2	Isoform Short of Alpha-(1,3)-fucosyltransferase OS=Mus musculus GN=Fut4 - [FUT4_MOUSE]	-0.94	-0.79	-0.89	-0.90	0.00
Q9D279	Mitotic interactor and substrate of PLK1 OS=Mus musculus GN=Misp PE=2 SV=1 - [MISP_MOUSE]	0.82	0.76	0.88	0.77	0.00
Q9CQQ7	ATP synthase subunit b, mitochondrial OS=Mus musculus GN=Atp5f1 PE=1 SV=1 - [AT5F1_MOUSE]	-0.39	-0.45	-0.43	-0.39	0.00
Q9CS42	Ribose-phosphate pyrophosphokinase 2 OS=Mus musculus GN=Prps2 PE=1 SV=4 - [PRPS2_MOUSE]	-0.34	-0.30	-0.29	-0.32	0.00
Q9CYN9	Renin receptor OS=Mus musculus GN=Atp6ap2 PE=2 SV=2 - [RENR_MOUSE]	-0.61	-0.56	-0.62	-0.54	0.00
Q7M739	Nuclear pore complex-associated intranuclear coiled-coil protein TPR OS=Mus musculus GN=Tpr PE=2 SV=1 - [Q7M739_MOUSE]	0.71	0.78	0.79	0.85	0.00
Q8R0X7	Sphingosine-1-phosphate lyase 1 OS=Mus musculus GN=Sgpl1 PE=2 SV=1 - [SGPL1_MOUSE]	-0.31	-0.37	-0.36	-0.36	0.00
A1YIY0	Fc receptor-like protein 6 OS=Mus musculus GN=Fcrl6 PE=2 SV=1 - [FCRL6_MOUSE]	0.48	0.44	0.44	0.40	0.00
Q6P9R2	Serine/threonine-protein kinase OSR1 OS=Mus musculus GN=Oxsr1 PE=1 SV=1 - [OXSR1_MOUSE]	0.38	0.38	0.33	0.35	0.00
D3Z5G7	Protein Ces1b OS=Mus musculus GN=Ces1b PE=4 SV=1 - [D3Z5G7_MOUSE]	0.97	1.03	1.10	1.14	0.00
P07361	Alpha-1-acid glycoprotein 2 OS=Mus musculus GN=Orm2 PE=1 SV=1 - [A1AG2_MOUSE]	1.97	1.89	2.20	2.12	0.00
E9QLD5	Serine protease 29 OS=Mus musculus GN=Prss29 PE=3 SV=1 - [E9QLD5_MOUSE]	-1.33	-1.50	-1.35	-1.52	0.00
A8Y5N4	17-beta-hydroxysteroid dehydrogenase 13 OS=Mus musculus GN=Hsd17b13 PE=2 SV=1 - [A8Y5N4_MOUSE]	1.58	1.61	1.40	1.43	0.00
Q8R4P9-2	Isoform 2 of Multidrug resistance-associated protein 7 OS=Mus musculus GN=Abcc10 - [MRP7_MOUSE]	0.48	0.53	0.45	0.49	0.00
Q8CFB4	Guanylate-binding protein 5 OS=Mus musculus GN=Gbp5 PE=1 SV=2 - [GBP5_MOUSE]	1.24	1.04	1.14	1.16	0.00
P28660-2	Isoform 2 of Nck-associated protein 1 OS=Mus musculus GN=Nckap1 - [NCKP1_MOUSE]	-0.83	-0.78	-0.77	-0.70	0.00
Q9Z2Q6	Septin-5 OS=Mus musculus GN=Sept5 PE=1 SV=2 - [SEPT5_MOUSE]	0.91	0.79	0.93	0.85	0.00
E9Q0S6	Protein Tns1 OS=Mus musculus GN=Tns1 PE=2 SV=1 - [E9Q0S6_MOUSE]	-0.32	-0.37	-0.37	-0.37	0.00

H3BJT2	Insulin-like growth factor-binding protein 4 (Fragment) OS=Mus musculus GN=Igfbp4 PE=4 SV=1 - [H3BJT2_MOUSE]	1.35	1.23	1.25	1.13	0.00
Q61696	Heat shock 70 kDa protein 1A OS=Mus musculus GN=Hspa1a PE=1 SV=2 - [HS71A_MOUSE]	-0.51	-0.50	-0.59	-0.53	0.00
Q69ZK7	Protein FAM214A OS=Mus musculus GN=Fam214a PE=2 SV=3 - [F214A_MOUSE]	0.19	0.22	0.20	0.23	0.00
O88456	Calpain small subunit 1 OS=Mus musculus GN=Capns1 PE=2 SV=1 - [CPNS1_MOUSE]	-0.33	-0.28	-0.30	-0.28	0.00
Q9CRY7	Glycerophosphodiester phosphodiesterase domain-containing protein 1 OS=Mus musculus GN=Gdpd1 PE=2 SV=1 - [GDPD1_MOUSE]	-1.16	-1.27	-1.08	-1.11	0.00
Q64521	Glycerol-3-phosphate dehydrogenase, mitochondrial OS=Mus musculus GN=Gpd2 PE=1 SV=2 - [GPDM_MOUSE]	-0.56	-0.49	-0.56	-0.58	0.00
E9Q3V2	UDP-N-acetylglucosamine transporter (Fragment) OS=Mus musculus GN=Slc35a3 PE=2 SV=1 - [E9Q3V2_MOUSE]	-0.91	-1.04	-0.90	-0.91	0.00
Q9JHK4	Geranylgeranyl transferase type-2 subunit alpha OS=Mus musculus GN=Rabggta PE=1 SV=1 - [PGTA_MOUSE]	-0.41	-0.38	-0.44	-0.37	0.00
Q8R326-2	Isoform 2 of Paraspeckle component 1 OS=Mus musculus GN=Pspc1 - [PSPC1_MOUSE]	0.68	0.60	0.70	0.71	0.00
Q9CQN1	Heat shock protein 75 kDa, mitochondrial OS=Mus musculus GN=Trap1 PE=1 SV=1 - [TRAP1_MOUSE]	-0.50	-0.58	-0.55	-0.59	0.00
Q8R121-2	Isoform 2 of Protein Z-dependent protease inhibitor OS=Mus musculus GN=Serpina10 - [ZPI_MOUSE]	1.08	1.10	0.95	0.97	0.00
Q8JZQ2	AFG3-like protein 2 OS=Mus musculus GN=Afg3l2 PE=1 SV=1 - [AFG32_MOUSE]	-0.96	-0.89	-0.80	-0.92	0.00
Q62148	Retinal dehydrogenase 2 OS=Mus musculus GN=Aldh1a2 PE=1 SV=2 - [AL1A2_MOUSE]	1.01	0.88	0.96	0.87	0.00
E9PYP1	Protein Ces1a OS=Mus musculus GN=Ces1a PE=4 SV=1 - [E9PYP1_MOUSE]	1.02	0.93	1.12	1.03	0.00
G5E8Q0	Lysosomal trafficking regulator, isoform CRA_a OS=Mus musculus GN=Lyst PE=4 SV=1 - [G5E8Q0_MOUSE]	-1.12	-0.98	-1.12	-0.98	0.00
Q3UQ44	Ras GTPase-activating-like protein IQGAP2 OS=Mus musculus GN=Iqgap2 PE=1 SV=2 - [IQGA2_MOUSE]	-0.53	-0.44	-0.48	-0.50	0.00
Q61704	Inter-alpha-trypsin inhibitor heavy chain H3 OS=Mus musculus GN=Itih3 PE=1 SV=3 - [ITIH3_MOUSE]	1.21	1.25	1.36	1.42	0.00
Q8BMS1	Trifunctional enzyme subunit alpha, mitochondrial OS=Mus musculus GN=Hadha PE=1 SV=1 - [ECHA_MOUSE]	-0.62	-0.68	-0.59	-0.58	0.00

P51658	Estradiol 17-beta-dehydrogenase 2 OS=Mus musculus GN=Hsd17b2 PE=2 SV=2 - [DHB2_MOUSE]	-0.45	-0.40	-0.39	-0.39	0.00
Q8BWY7-3	Isoform 3 of Zinc transporter ZIP11 OS=Mus musculus GN=Slc39a11 - [S39AB_MOUSE]	-0.53	-0.47	-0.56	-0.51	0.00
Q9CSN1	SNW domain-containing protein 1 OS=Mus musculus GN=Snw1 PE=1 SV=3 - [SNW1_MOUSE]	1.15	0.99	1.09	0.99	0.00
Q61702	Inter-alpha-trypsin inhibitor heavy chain H1 OS=Mus musculus GN=Itih1 PE=1 SV=2 - [ITIH1_MOUSE]	1.00	1.04	1.15	1.16	0.00
Q9ERZ4	Muscarinic acetylcholine receptor M2 OS=Mus musculus GN=Chrm2 PE=1 SV=2 - [ACM2_MOUSE]	-1.01	-1.13	-0.95	-1.07	0.00
Q8CH77-4	Isoform 4 of Neuron navigator 1 OS=Mus musculus GN=Nav1 - [NAV1_MOUSE]	0.40	0.38	0.35	0.34	0.00
Q61646	Haptoglobin OS=Mus musculus GN=Hp PE=1 SV=1 - [HPT_MOUSE]	1.63	1.65	1.87	1.86	0.00
Q05186	Reticulocalbin-1 OS=Mus musculus GN=Rcn1 PE=1 SV=1 - [RCN1_MOUSE]	0.60	0.50	0.57	0.56	0.00
P37040	NADPH--cytochrome P450 reductase OS=Mus musculus GN=Por PE=1 SV=2 - [NCPR_MOUSE]	0.32	0.30	0.30	0.27	0.00
Q08331	Calretinin OS=Mus musculus GN=Calb2 PE=1 SV=3 - [CALB2_MOUSE]	0.39	0.39	0.44	0.45	0.00
E9Q4Q2	Splicing factor 1 OS=Mus musculus GN=Sf1 PE=2 SV=1 - [E9Q4Q2_MOUSE]	0.49	0.53	0.47	0.56	0.00
P54869	Hydroxymethylglutaryl-CoA synthase, mitochondrial OS=Mus musculus GN=Hmgcs2 PE=1 SV=2 - [HMCS2_MOUSE]	1.00	1.06	1.15	1.18	0.00
Q9D2Y4-2	Isoform 2 of Mixed lineage kinase domain-like protein OS=Mus musculus GN=Mlkl - [MLKL_MOUSE]	-0.20	-0.17	-0.17	-0.18	0.00
Q8BP00-2	Isoform 2 of IQ calmodulin-binding motif-containing protein 1 OS=Mus musculus GN=Iqcb1 - [IQCB1_MOUSE]	1.25	1.34	1.40	1.49	0.00
P62827	GTP-binding nuclear protein Ran OS=Mus musculus GN=Ran PE=1 SV=3 - [RAN_MOUSE]	-0.37	-0.36	-0.38	-0.32	0.00
Q91VD9	NADH-ubiquinone oxidoreductase 75 kDa subunit, mitochondrial OS=Mus musculus GN=Ndufs1 PE=1 SV=2 - [NDUS1_MOUSE]	-0.66	-0.66	-0.76	-0.75	0.00
Q03517	Secretogranin-2 OS=Mus musculus GN=Scg2 PE=1 SV=1 - [SCG2_MOUSE]	0.30	0.33	0.36	0.34	0.00
Q5NCE8	Magnesium transporter MRS2 homolog, mitochondrial OS=Mus musculus GN=Mrs2 PE=2 SV=2 - [MRS2_MOUSE]	-0.56	-0.63	-0.60	-0.67	0.00

Q9R0L6	Pericentriolar material 1 protein OS=Mus musculus GN=Pcm1 PE=1 SV=2 - [PCM1_MOUSE]	0.82	0.73	0.78	0.69	0.00
Q9R1T4-2	Isoform I of Septin-6 OS=Mus musculus GN=Sept6 - [SEPT6_MOUSE]	0.33	0.37	0.32	0.32	0.00
Q91X79	Chymotrypsin-like elastase family member 1 OS=Mus musculus GN=Cela1 PE=2 SV=1 - [CELA1_MOUSE]	-0.52	-0.52	-0.60	-0.58	0.00
F8VQ75	Kinesin-like protein KIF13A OS=Mus musculus GN=Kif13a PE=2 SV=1 - [F8VQ75_MOUSE]	-1.11	-1.04	-0.98	-0.92	0.00
Q4VAA2-2	Isoform 2 of Protein CDV3 OS=Mus musculus GN=Cdv3 - [CDV3_MOUSE]	0.81	0.71	0.81	0.85	0.00
Q9D7Z6	Calcium-activated chloride channel regulator 1 OS=Mus musculus GN=Clca1 PE=1 SV=2 - [CLCA1_MOUSE]	-1.50	-1.52	-1.69	-1.75	0.00
A2AKI5	Integrin alpha-V light chain OS=Mus musculus GN=Itgav PE=2 SV=1 - [A2AKI5_MOUSE]	-0.76	-0.72	-0.64	-0.66	0.00
Q9ERU9	E3 SUMO-protein ligase RanBP2 OS=Mus musculus GN=Ranbp2 PE=1 SV=2 - [RBP2_MOUSE]	0.34	0.34	0.32	0.39	0.00
Q9JHU4	Cytoplasmic dynein 1 heavy chain 1 OS=Mus musculus GN=Dync1h1 PE=1 SV=2 - [DYHC1_MOUSE]	-0.50	-0.46	-0.55	-0.47	0.00
O88451	Retinol dehydrogenase 7 OS=Mus musculus GN=Rdh7 PE=2 SV=1 - [RDH7_MOUSE]	-0.62	-0.60	-0.53	-0.63	0.00
Q8C3Y4	Kinetochore-associated protein 1 OS=Mus musculus GN=Kntc1 PE=1 SV=2 - [KNTC1_MOUSE]	-0.39	-0.42	-0.35	-0.38	0.00
P97429	Annexin A4 OS=Mus musculus GN=Anxa4 PE=2 SV=4 - [ANXA4_MOUSE]	0.21	0.21	0.23	0.24	0.00
E9PYS3	Protein Adam3 OS=Mus musculus GN=Adam3 PE=2 SV=1 - [E9PYS3_MOUSE]	-1.71	-1.79	-1.94	-2.02	0.00
H9H9T1	Protein FAM107B OS=Mus musculus GN=Fam107b PE=2 SV=1 - [H9H9T1_MOUSE]	0.87	0.80	0.97	0.89	0.00
Q3UGC7	Eukaryotic translation initiation factor 3 subunit J-A OS=Mus musculus GN=Eif3j1 PE=2 SV=1 - [EI3JA_MOUSE]	0.55	0.46	0.54	0.53	0.00
Q3UE37	Ubiquitin-conjugating enzyme E2 Z OS=Mus musculus GN=Ube2z PE=1 SV=2 - [UBE2Z_MOUSE]	-0.63	-0.54	-0.65	-0.62	0.00
P20918	Plasminogen OS=Mus musculus GN=Plg PE=1 SV=3 - [PLMN_MOUSE]	1.21	1.17	1.40	1.24	0.00
Q9CXR1	Dehydrogenase/reductase SDR family member 7 OS=Mus musculus GN=Dhrs7 PE=2 SV=2 - [DHRS7_MOUSE]	-0.15	-0.16	-0.15	-0.13	0.00

Q9QXG4	Acetyl-coenzyme A synthetase, cytoplasmic OS=Mus musculus GN=Acss2 PE=1 SV=2 - [ACSA_MOUSE]	-0.74	-0.76	-0.83	-0.87	0.00
O88569	Heterogeneous nuclear ribonucleoproteins A2/B1 OS=Mus musculus GN=Hnrnpa2b1 PE=1 SV=2 - [ROA2_MOUSE]	0.54	0.49	0.58	0.57	0.00
Q99K28	ADP-ribosylation factor GTPase-activating protein 2 OS=Mus musculus GN=Arfgap2 PE=1 SV=1 - [ARFG2_MOUSE]	0.45	0.44	0.43	0.51	0.00
P97363	Serine palmitoyltransferase 2 OS=Mus musculus GN=Sptlc2 PE=2 SV=2 - [SPTC2_MOUSE]	-0.40	-0.42	-0.37	-0.35	0.00
E9PWX8	Microtubule-associated serine/threonine-protein kinase 4 OS=Mus musculus GN=Mast4 PE=2 SV=1 - [E9PWX8_MOUSE]	0.61	0.60	0.51	0.61	0.00
D3YTY9	Kininogen-1 OS=Mus musculus GN=Kng1 PE=2 SV=1 [D3YTY9_MOUSE]	1.22	1.32	1.46	1.43	0.00
Q07797	Galectin-3-binding protein OS=Mus musculus GN=Lgals3bp PE=1 SV=1 - [LG3BP_MOUSE]	0.38	0.39	0.34	0.41	0.00
P43137	Lithostathine-1 OS=Mus musculus GN=Reg1 PE=2 SV=1 - [LIT1_MOUSE]	0.65	0.78	0.75	0.69	0.00
Q6XQH0	Galactose-3-O-sulfotransferase 2 OS=Mus musculus GN=Gal3st2 PE=2 SV=1 - [G3ST2_MOUSE]	-0.41	-0.38	-0.46	-0.44	0.00
P24270	Catalase OS=Mus musculus GN=Cat PE=1 SV=4 - [CATA_MOUSE]	0.49	0.44	0.52	0.45	0.00
P61600	N-alpha-acetyltransferase 20 OS=Mus musculus GN=Naa20 PE=2 SV=1 - [NAA20_MOUSE]	-0.68	-0.75	-0.80	-0.68	0.00
Q8CGK3	Lon protease homolog, mitochondrial OS=Mus musculus GN=Lonp1 PE=1 SV=2 - [LONM_MOUSE]	-0.60	-0.51	-0.59	-0.53	0.00
Q6PGF7	Exocyst complex component 8 OS=Mus musculus GN=Exoc8 PE=1 SV=1 - [EXOC8_MOUSE]	-0.31	-0.32	-0.28	-0.27	0.00
P29452	Caspase-1 OS=Mus musculus GN=Casp1 PE=1 SV=1 [CASP1_MOUSE]	0.21	0.24	0.20	0.20	0.00
Q9EP69	Phosphatidylinositide phosphatase SAC1 OS=Mus musculus GN=Sacm1l PE=2 SV=1 - [SAC1_MOUSE]	-0.59	-0.52	-0.63	-0.57	0.00
Q9DCN2-2	Isoform 2 of NADH-cytochrome b5 reductase 3 OS=Mus musculus GN=Cyb5r3 - [NB5R3_MOUSE]	-0.23	-0.21	-0.25	-0.24	0.00
H3BK86	Peptide YY OS=Mus musculus GN=Pyy PE=3 SV=1 - [H3BK86_MOUSE]	-1.99	-1.80	-2.20	-2.01	0.00
P14069	Protein S100-A6 OS=Mus musculus GN=S100a6 PE=1 SV=3 - [S10A6_MOUSE]	-1.04	-1.02	-0.88	-0.92	0.00

D3Z2Y6	Protein S100a16 (Fragment) OS=Mus musculus GN=S100a16 PE=2 SV=1 - [D3Z2Y6_MOUSE]	-0.71	-0.80	-0.87	-0.77	0.00
Q9Z0S1	3'(2'),5'-bisphosphate nucleotidase 1 OS=Mus musculus GN=Bpnt1 PE=1 SV=2 - [BPNT1_MOUSE]	-0.53	-0.47	-0.45	-0.52	0.00
P28667	MARCKS-related protein OS=Mus musculus GN=Marcksl1 PE=1 SV=2 - [MRP_MOUSE]	0.93	0.97	1.06	1.10	0.00
I7HPY1	Novel protein OS=Mus musculus GN=Fam3a PE=4 SV=1 - [I7HPY1_MOUSE]	-1.22	-1.27	-1.06	-1.11	0.00
Q91XA2	Golgi membrane protein 1 OS=Mus musculus GN=Golm1 PE=1 SV=2 - [GOLM1_MOUSE]	-0.46	-0.51	-0.52	-0.56	0.00
Q8BZ33	Tensin-4 OS=Mus musculus GN=Tns4 PE=2 SV=2 - [TENS4_MOUSE]	1.29	1.22	1.13	1.07	0.00
Q8C6I2-3	Isoform 3 of Succinate dehydrogenase assembly factor 2, mitochondrial OS=Mus musculus GN=Sdhaf2 - [SDHF2_MOUSE]	0.74	0.84	0.69	0.79	0.00
Q9D8S3	ADP-ribosylation factor GTPase-activating protein 3 OS=Mus musculus GN=Arfgap3 PE=2 SV=2 - [ARFG3_MOUSE]	0.40	0.44	0.38	0.36	0.00
E9PVX6	Protein Mki67 OS=Mus musculus GN=Mki67 PE=2 SV=1 - [E9PVX6_MOUSE]	0.66	0.73	0.60	0.69	0.00
P70168	Importin subunit beta-1 OS=Mus musculus GN=Kpnb1 PE=1 SV=2 - [IMB1_MOUSE]	-0.93	-0.80	-0.95	-0.83	0.00
Q9DBD0	Inhibitor of carbonic anhydrase OS=Mus musculus GN=Ica PE=1 SV=1 - [ICA_MOUSE]	0.56	0.60	0.68	0.60	0.00
Q62425	NADH dehydrogenase [ubiquinone] 1 alpha subcomplex subunit 4 OS=Mus musculus GN=Ndufa4 PE=1 SV=2 - [NDUA4_MOUSE]	-0.31	-0.31	-0.26	-0.28	0.00
O55125	Protein NipSnap homolog 1 OS=Mus musculus GN=Nipsnap1 PE=1 SV=1 - [NIPS1_MOUSE]	-0.44	-0.37	-0.43	-0.39	0.00
A2A4A1	DnaJ homolog subfamily C member 24 OS=Mus musculus GN=Dnajc24 PE=2 SV=1 - [A2A4A1_MOUSE]	0.24	0.26	0.23	0.27	0.00
Q8CIM7	Cytochrome P450 2D26 OS=Mus musculus GN=Cyp2d26 PE=1 SV=1 - [CP2DQ_MOUSE]	0.79	0.82	0.69	0.72	0.00
P09803	Cadherin-1 OS=Mus musculus GN=Cdh1 PE=1 SV=1 - [CADH1_MOUSE]	-0.20	-0.21	-0.18	-0.18	0.00
Q62312-2	Isoform RII-1 of TGF-beta receptor type-2 OS=Mus musculus GN=Tgfbr2 - [TGFR2_MOUSE]	0.80	0.87	0.91	0.98	0.00
Q8BI29	Specifically androgen-regulated gene protein OS=Mus musculus GN=Sarg PE=1 SV=2 - [SARG_MOUSE]	0.98	1.13	0.99	1.14	0.00

P82198	Transforming growth factor-beta-induced protein ig-h3 OS=Mus musculus GN=Tgfbi PE=2 SV=1 - [BGH3_MOUSE]	-0.24	-0.21	-0.20	-0.22	0.00
Q8BVE3	V-type proton ATPase subunit H OS=Mus musculus GN=Atp6v1h PE=1 SV=1 - [VATH_MOUSE]	-0.35	-0.40	-0.39	-0.34	0.00
Q9D3N8-3	Isoform 3 of Growth hormone-regulated TBC protein 1 OS=Mus musculus GN=Grtp1 - [GRTP1_MOUSE]	-0.78	-0.69	-0.81	-0.84	0.00
D3Z5F9	Protein YIF1B (Fragment) OS=Mus musculus GN=Yif1b PE=2 SV=1 - [D3Z5F9_MOUSE]	-0.12	-0.11	-0.12	-0.10	0.00
Q99LL3	Carbohydrate sulfotransferase 12 OS=Mus musculus GN=Chst12 PE=2 SV=2 - [CHSTC_MOUSE]	-0.88	-0.89	-0.76	-0.77	0.00
Q8R2N2	Cirhin OS=Mus musculus GN=Cirh1a PE=2 SV=3 - [CIR1A_MOUSE]	-0.31	-0.35	-0.28	-0.32	0.00
Q8K0Z7	Translational activator of cytochrome c oxidase 1 OS=Mus musculus GN=Taco1 PE=2 SV=1 - [TACO1_MOUSE]	-0.28	-0.30	-0.31	-0.34	0.00
P51881	ADP/ATP translocase 2 OS=Mus musculus GN=Slc25a5 PE=1 SV=3 - [ADT2_MOUSE]	-1.09	-0.98	-0.92	-0.91	0.00
Q3UH85	Protein 8430427H17Rik OS=Mus musculus GN=8430427H17Rik PE=2 SV=1 - [Q3UH85_MOUSE]	1.04	0.96	0.86	1.02	0.00
Q5JCS9	UDP-GlcNAc:betaGal beta-1,3-N-acetylglucosaminyltransferase 3 OS=Mus musculus GN=B3gnt3 PE=2 SV=1 - [B3GN3_MOUSE]	-1.16	-1.19	-1.38	-1.18	0.00
Q8K354	Carbonyl reductase [NADPH] 3 OS=Mus musculus GN=Cbr3 PE=2 SV=1 - [CBR3_MOUSE]	-0.33	-0.32	-0.34	-0.28	0.00
Q80T14	Extracellular matrix protein FRAS1 OS=Mus musculus GN=Fras1 PE=2 SV=2 - [FRAS1_MOUSE]	0.61	0.55	0.67	0.60	0.00
F6V9H6	Protein Muc3 (Fragment) OS=Mus musculus GN=Muc3 PE=4 SV=1 - [F6V9H6_MOUSE]	-1.36	-1.47	-1.20	-1.38	0.00
Q8R0W0	Epiplakin OS=Mus musculus GN=Eppk1 PE=1 SV=2 - [EPIPL_MOUSE]	-0.38	-0.31	-0.35	-0.33	0.00
Q9CXS4	Centromere protein V OS=Mus musculus GN=Cenpv PE=2 SV=2 - [CENPV_MOUSE]	0.37	0.43	0.41	0.36	0.00
Q3UW64	Bifunctional UDP-N-acetylglucosamine 2-epimerase/N-acetylmannosamine kinase OS=Mus musculus GN=Gne PE=2 SV=1 - [Q3UW64_MOUSE]	-0.50	-0.45	-0.51	-0.42	0.00
Q6A009	E3 ubiquitin-protein ligase listerin OS=Mus musculus GN=Ltn1 PE=1 SV=3 - [LTN1_MOUSE]	-0.25	-0.28	-0.23	-0.26	0.00
Q9QWR8	Alpha-N-acetylgalactosaminidase OS=Mus musculus GN=Naga PE=2 SV=2 - [NAGAB_MOUSE]	-0.38	-0.40	-0.43	-0.46	0.00

Q3TC93	HCLS1-binding protein 3 OS=Mus musculus GN=Hs1bp3 PE=1 SV=2 - [H1BP3_MOUSE]	0.31	0.30	0.36	0.35	0.00
Q8R1A8	Ornithine carbamoyltransferase, mitochondrial OS=Mus musculus GN=Otc PE=2 SV=1 - [Q8R1A8_MOUSE]	0.57	0.64	0.67	0.71	0.00
Q8K262	Placenta-specific protein 9 OS=Mus musculus GN=Plac9 PE=2 SV=1 - [PLAC9_MOUSE]	0.33	0.31	0.38	0.35	0.00
Q9D1P0	39S ribosomal protein L13, mitochondrial OS=Mus musculus GN=Mrpl13 PE=2 SV=1 - [RM13_MOUSE]	-0.18	-0.21	-0.17	-0.19	0.00
Q9D819	Inorganic pyrophosphatase OS=Mus musculus GN=Ppa1 PE=1 SV=1 - [IPYR_MOUSE]	-1.12	-1.02	-0.96	-0.93	0.00
Q9D8Y1	Transmembrane protein 126A OS=Mus musculus GN=Tmem126a PE=2 SV=1 - [T126A_MOUSE]	-0.31	-0.34	-0.36	-0.38	0.00
Q9ERG0	LIM domain and actin-binding protein 1 OS=Mus musculus GN=Lima1 PE=1 SV=3 - [LIMA1_MOUSE]	0.50	0.53	0.56	0.61	0.00
Q9D614	Syntaxin 17, isoform CRA_b OS=Mus musculus GN=Stx17 PE=2 SV=1 - [Q9D614_MOUSE]	0.31	0.30	0.36	0.34	0.00
Q9D0K2	Succinyl-CoA:3-ketoacid coenzyme A transferase 1, mitochondrial OS=Mus musculus GN=Oxct1 PE=1 SV=1 - [SCOT1_MOUSE]	-0.28	-0.33	-0.34	-0.33	0.00
O35459	Delta(3,5)-Delta(2,4)-dienoyl-CoA isomerase, mitochondrial OS=Mus musculus GN=Ech1 PE=2 SV=1 - [ECH1_MOUSE]	0.42	0.46	0.50	0.51	0.00
Q91VK1	Basic leucine zipper and W2 domain-containing protein 2 OS=Mus musculus GN=Bzw2 PE=1 SV=1 - [BZW2_MOUSE]	-0.21	-0.24	-0.23	-0.20	0.00
Q8BH24	Transmembrane 9 superfamily member 4 OS=Mus musculus GN=Tm9sf4 PE=2 SV=1 - [TM9S4_MOUSE]	-0.96	-0.83	-1.02	-0.92	0.00
Q9D6Y7-2	Isoform 2 of Mitochondrial peptide methionine sulfoxide reductase OS=Mus musculus GN=Msra - [MSRA_MOUSE]	0.67	0.61	0.64	0.54	0.00
P04186	Complement factor B OS=Mus musculus GN=Cfb PE=1 SV=2 - [CFAB_MOUSE]	1.00	1.09	0.90	1.08	0.00
Q8C8R3-2	Isoform 2 of Ankyrin-2 OS=Mus musculus GN=Ank2 - [ANK2_MOUSE]	0.37	0.40	0.34	0.33	0.00
Q9CZR2	N-acetylated-alpha-linked acidic dipeptidase 2 OS=Mus musculus GN=Naalad2 PE=1 SV=2 - [NALD2_MOUSE]	0.69	0.68	0.74	0.60	0.00
P51655	Glypican-4 OS=Mus musculus GN=Gpc4 PE=2 SV=2 - [GPC4_MOUSE]	-1.02	-0.91	-1.07	-0.90	0.00
E9PX70	Collagen alpha-1(XII) chain OS=Mus musculus GN=Col12a1 PE=2 SV=1 - [E9PX70_MOUSE]	-0.27	-0.25	-0.23	-0.28	0.00

E9QQ10	A-kinase anchor protein 9 OS=Mus musculus GN=Akap9 PE=2 SV=1 - [E9QQ10_MOUSE]	0.39	0.41	0.34	0.41	0.00
Q3UMT1	Protein phosphatase 1 regulatory subunit 12C OS=Mus musculus GN=Ppp1r12c PE=1 SV=1 - [PP12C_MOUSE]	0.39	0.31	0.34	0.34	0.00
Q5F2E7	Nuclear fragile X mental retardation-interacting protein 2 OS=Mus musculus GN=Nufip2 PE=1 SV=1 - [NUFP2_MOUSE]	0.79	0.80	0.90	0.94	0.00
Q8BU30	Isoleucine--tRNA ligase, cytoplasmic OS=Mus musculus GN=Iars PE=2 SV=2 - [SYIC_MOUSE]	-0.40	-0.47	-0.40	-0.46	0.00
G3X9V0	MCG22048, isoform CRA_a OS=Mus musculus GN=Psme2 PE=2 SV=1 - [G3X9V0_MOUSE]	-0.65	-0.62	-0.54	-0.57	0.00
P29699	Alpha-2-HS-glycoprotein OS=Mus musculus GN=Ahsg PE=1 SV=1 - [FETUA_MOUSE]	0.90	0.91	1.08	0.93	0.00
O88428	Bifunctional 3'-phosphoadenosine 5'-phosphosulfate synthase 2 OS=Mus musculus GN=Papss2 PE=1 SV=2 - [PAPS2_MOUSE]	0.52	0.50	0.60	0.58	0.00
P62311	U6 snRNA-associated Sm-like protein LSm3 OS=Mus musculus GN=Lsm3 PE=3 SV=2 - [LSM3_MOUSE]	0.47	0.51	0.41	0.47	0.00
Q5NC41	Protein 1810065E05Rik OS=Mus musculus GN=1810065E05Rik PE=4 SV=1 - [Q5NC41_MOUSE]	2.07	1.80	2.23	2.10	0.00
Q9D154	Leukocyte elastase inhibitor A OS=Mus musculus GN=Serpinb1a PE=1 SV=1 - [ILEUA_MOUSE]	-0.50	-0.46	-0.45	-0.40	0.00
Q64133	Amine oxidase [flavin-containing] A OS=Mus musculus GN=Maoa PE=1 SV=3 - [AOFA_MOUSE]	-0.53	-0.55	-0.47	-0.47	0.00
Q7JJ13-2	Isoform 2 of Bromodomain-containing protein 2 OS=Mus musculus GN=Brd2 - [BRD2_MOUSE]	0.58	0.50	0.56	0.48	0.00
Q6PGH2	Hematological and neurological expressed 1-like protein OS=Mus musculus GN=Hn1l PE=2 SV=1 - [HN1L_MOUSE]	0.40	0.48	0.46	0.41	0.00
I3ITR1	MCG50313 OS=Mus musculus GN=AK157302 PE=4 SV=1 - [I3ITR1_MOUSE]	-0.73	-0.83	-0.67	-0.77	0.00
P50285	Dimethylaniline monooxygenase [N-oxide-forming] 1 OS=Mus musculus GN=Fmo1 PE=1 SV=1 - [FMO1_MOUSE]	0.32	0.38	0.32	0.37	0.00
H9KUX9	Retinoid isomerohydrolase OS=Mus musculus GN=Rpe65 PE=2 SV=1 - [H9KUX9_MOUSE]	0.39	0.40	0.46	0.47	0.00
P21107-2	Isoform 2 of Tropomyosin alpha-3 chain OS=Mus musculus GN=Tpm3 - [TPM3_MOUSE]	0.34	0.35	0.33	0.40	0.00
Q8R105	Vacuolar protein sorting-associated protein 37C OS=Mus musculus GN=Vps37c PE=2 SV=1 - [VP37C_MOUSE]	0.52	0.45	0.54	0.46	0.00

Q8CID0	Cysteine-rich protein 2-binding protein OS=Mus musculus GN=Csrp2bp PE=2 SV=2 - [CSR2B_MOUSE]	1.10	1.00	0.98	0.88	0.00
Q9ERK4	Exportin-2 OS=Mus musculus GN=Cse1l PE=2 SV=1 - [XPO2_MOUSE]	-0.74	-0.85	-0.84	-0.92	0.00
Q80WE4-3	Isoform 3 of Kinesin-like protein KIF20B OS=Mus musculus GN=Kif20b - [KI20B_MOUSE]	-0.57	-0.55	-0.49	-0.47	0.00
Q9Z0R9	Fatty acid desaturase 2 OS=Mus musculus GN=Fads2 PE=2 SV=1 - [FADS2_MOUSE]	-1.24	-1.32	-1.07	-1.15	0.00
P62908	40S ribosomal protein S3 OS=Mus musculus GN=Rps3 PE=1 SV=1 - [RS3_MOUSE]	-0.45	-0.42	-0.36	-0.39	0.00
F8VPU2	FERM, RhoGEF and pleckstrin domain-containing protein 1 OS=Mus musculus GN=Farp1 PE=1 SV=1 - [FARP1_MOUSE]	-0.41	-0.50	-0.49	-0.50	0.00
B1AU76	Nuclear autoantigenic sperm protein OS=Mus musculus GN=Nasp PE=2 SV=1 - [B1AU76_MOUSE]	0.34	0.33	0.31	0.38	0.00
Q9DCE9	Protein Igtp OS=Mus musculus GN=Igtp PE=2 SV=1 - [Q9DCE9_MOUSE]	0.84	0.88	0.77	0.72	0.00
J3QPE8	MCG16555 OS=Mus musculus GN=Vdac3-ps1 PE=4 SV=1 - [J3QPE8_MOUSE]	-0.67	-0.62	-0.63	-0.54	0.00
E9QL31	Disabled homolog 2 OS=Mus musculus GN=Dab2 PE=2 SV=1 - [E9QL31_MOUSE]	0.70	0.79	0.81	0.87	0.00
Q4KMM3-2	Isoform 2 of Oxidation resistance protein 1 OS=Mus musculus GN=Oxr1 - [OXR1_MOUSE]	0.54	0.64	0.56	0.53	0.00
A2AP32	NADH dehydrogenase [ubiquinone] 1 beta subcomplex subunit 6 OS=Mus musculus GN=Ndufb6 PE=2 SV=1 - [A2AP32_MOUSE]	-0.65	-0.60	-0.59	-0.52	0.00
Q3UW66	Sulfurtransferase OS=Mus musculus GN=Mpst PE=2 SV=1 - [Q3UW66_MOUSE]	1.07	1.23	1.27	1.33	0.00
Q9Z266	SNARE-associated protein Snapin OS=Mus musculus GN=Snapin PE=1 SV=1 - [SNAPN_MOUSE]	-0.26	-0.28	-0.22	-0.24	0.00
Q9R0E1	Procollagen-lysine,2-oxoglutarate 5-dioxygenase 3 OS=Mus musculus GN=Plod3 PE=1 SV=1 - [PLOD3_MOUSE]	0.36	0.43	0.39	0.35	0.00
Q61102	ATP-binding cassette sub-family B member 7, mitochondrial OS=Mus musculus GN=Abcb7 PE=1 SV=3 - [ABCB7_MOUSE]	-0.65	-0.56	-0.62	-0.70	0.00
Q05816	Fatty acid-binding protein, epidermal OS=Mus musculus GN=Fabp5 PE=1 SV=3 - [FABP5_MOUSE]	0.25	0.26	0.29	0.30	0.00
Q8K019-3	Isoform 3 of Bcl-2-associated transcription factor 1 OS=Mus musculus GN=Bclaf1 - [BCLF1_MOUSE]	1.02	0.87	1.08	1.05	0.00

Q91WU0	Expressed sequence AU018778 OS=Mus musculus GN=Ces1f PE=2 SV=1 - [Q91WU0_MOUSE]	0.76	0.76	0.89	0.90	0.00
P26039	Talin-1 OS=Mus musculus GN=Tln1 PE=1 SV=2 - [TLN1_MOUSE]	-0.60	-0.68	-0.66	-0.75	0.00
Q8BMJ2	Leucine--tRNA ligase, cytoplasmic OS=Mus musculus GN=Lars PE=2 SV=2 - [SYLC_MOUSE]	-0.19	-0.19	-0.15	-0.18	0.00
A2A9W7	ADP-ribosylation factor-binding protein GGA3 OS=Mus musculus GN=Gga3 PE=2 SV=1 - [A2A9W7_MOUSE]	2.03	2.03	1.73	1.73	0.00
A2AEX8	Four and a half LIM domains 1, isoform CRA_b OS=Mus musculus GN=Fhl1 PE=4 SV=1 - [A2AEX8_MOUSE]	-0.80	-0.68	-0.66	-0.68	0.00
Q99MQ4	Asporin OS=Mus musculus GN=Aspn PE=1 SV=1 - [ASPN_MOUSE]	-0.41	-0.33	-0.37	-0.35	0.00
Q61578	NADPH:adrenodoxin oxidoreductase, mitochondrial OS=Mus musculus GN=Fdxr PE=2 SV=1 - [ADRO_MOUSE]	-0.54	-0.48	-0.43	-0.48	0.00
Q8BP40	Lysophosphatidic acid phosphatase type 6 OS=Mus musculus GN=Acp6 PE=2 SV=1 - [PPA6_MOUSE]	-0.17	-0.18	-0.15	-0.15	0.00
P58281-2	Isoform 2 of Dynamin-like 120 kDa protein, mitochondrial OS=Mus musculus GN=Opa1 - [OPA1_MOUSE]	-0.61	-0.52	-0.58	-0.50	0.00
P09813	Apolipoprotein A-II OS=Mus musculus GN=Apoa2 PE=1 SV=2 - [APOA2_MOUSE]	0.59	0.66	0.70	0.74	0.00
Q3TQI7	Uncharacterized protein C9orf78 homolog OS=Mus musculus PE=1 SV=2 - [CI078_MOUSE]	0.19	0.19	0.23	0.23	0.00
Q62433	Protein NDRG1 OS=Mus musculus GN=Ndrg1 PE=1 SV=1 - [NDRG1_MOUSE]	0.43	0.46	0.54	0.46	0.00
Q921G7	Electron transfer flavoprotein-ubiquinone oxidoreductase, mitochondrial OS=Mus musculus GN=Etfdh PE=1 SV=1 - [ETFD_MOUSE]	-0.63	-0.57	-0.55	-0.50	0.00
F8WHP8	ATP synthase subunit f, mitochondrial OS=Mus musculus GN=Atp5j2 PE=2 SV=1 - [F8WHP8_MOUSE]	-0.33	-0.40	-0.38	-0.41	0.00
F8VQI8	Sodium/hydrogen exchanger 10 OS=Mus musculus GN=Slc9c1 PE=2 SV=1 - [F8VQI8_MOUSE]	1.95	2.21	2.19	2.45	0.00
Q6PIP5	NudC domain-containing protein 1 OS=Mus musculus GN=Nudcd1 PE=2 SV=2 - [NUDC1_MOUSE]	0.42	0.43	0.36	0.45	0.00
Q61079	Single-minded homolog 2 OS=Mus musculus GN=Sim2 PE=1 SV=1 - [SIM2_MOUSE]	1.11	1.25	0.99	1.12	0.00
E9Q357	Protein Gm17673 OS=Mus musculus GN=Gm17673 PE=4 SV=2 - [E9Q357_MOUSE]	0.43	0.49	0.39	0.45	0.00

P02469	Laminin subunit beta-1 OS=Mus musculus GN=Lamb1 PE=1 SV=3 - [LAMB1_MOUSE]	0.45	0.51	0.50	0.56	0.00
Q6PGF3	Mediator of RNA polymerase II transcription subunit 16 OS=Mus musculus GN=Med16 PE=2 SV=2 - [MED16_MOUSE]	0.89	1.02	0.81	0.93	0.00
Q9D824-4	Isoform 4 of Pre-mRNA 3'-end-processing factor FIP1 OS=Mus musculus GN=Fip1l1 - [FIP1_MOUSE]	1.54	1.78	1.52	1.82	0.00
Q80TJ7-2	Isoform 2 of Histone lysine demethylase PHF8 OS=Mus musculus GN=Phf8 - [PHF8_MOUSE]	0.87	0.90	0.74	0.77	0.00
P27046	Alpha-mannosidase 2 OS=Mus musculus GN=Man2a1 PE=1 SV=2 - [MA2A1_MOUSE]	-0.36	-0.32	-0.34	-0.28	0.00
Q5SWP3	NAC-alpha domain-containing protein 1 OS=Mus musculus GN=Nacad PE=1 SV=1 - [NACAD_MOUSE]	0.29	0.26	0.33	0.30	0.00
P63030	Mitochondrial pyruvate carrier 1 OS=Mus musculus GN=Mpc1 PE=1 SV=1 - [MPC1_MOUSE]	-0.64	-0.68	-0.80	-0.73	0.00
Q9JL62	Glycolipid transfer protein OS=Mus musculus GN=Gltp PE=2 SV=3 - [GLTP_MOUSE]	-0.79	-0.87	-0.71	-0.71	0.00
Q3V117	ATP-citrate synthase OS=Mus musculus GN=Acly PE=2 SV=1 - [Q3V117_MOUSE]	-1.15	-1.16	-1.20	-1.40	0.00
Q60598	Src substrate cortactin OS=Mus musculus GN=Cttn PE=1 SV=2 - [SRC8_MOUSE]	0.54	0.58	0.63	0.67	0.00
Q3TDQ1	Dolichyl-diphosphooligosaccharide--protein glycosyltransferase subunit STT3B OS=Mus musculus GN=Stt3b PE=1 SV=2 - [STT3B_MOUSE]	-0.25	-0.28	-0.23	-0.23	0.00
Q5PRE5	Proline and serine-rich protein 1 OS=Mus musculus GN=Proser1 PE=2 SV=1 - [PRSR1_MOUSE]	0.75	0.66	0.68	0.59	0.00
O35129	Prohibitin-2 OS=Mus musculus GN=Phb2 PE=1 SV=1 - [PHB2_MOUSE]	-0.49	-0.39	-0.47	-0.42	0.00
Q7TME2	Sperm-associated antigen 5 OS=Mus musculus GN=Spag5 PE=1 SV=1 - [SPAG5_MOUSE]	-2.70	-2.63	-3.18	-3.11	0.00
Q9CQB4	Cytochrome b-c1 complex subunit 7 OS=Mus musculus GN=Uqcrb PE=3 SV=1 - [Q9CQB4_MOUSE]	-0.60	-0.58	-0.50	-0.50	0.00
E9PZD2	Protein Micall2 OS=Mus musculus GN=Micall2 PE=2 SV=1 - [E9PZD2_MOUSE]	0.44	0.36	0.38	0.36	0.00
Q80ZJ6-3	Isoform 3 of Protein zer-1 homolog OS=Mus musculus GN=Zer1 - [ZER1_MOUSE]	0.23	0.27	0.25	0.29	0.00
P51437	Cathelin-related antimicrobial peptide OS=Mus musculus GN=Camp PE=2 SV=1 - [CRAMP_MOUSE]	1.61	1.57	1.91	1.84	0.00

Q8BJ05-2	Isoform 2 of Zinc finger CCCH domain-containing protein 14 OS=Mus musculus GN=Zc3h14 - [ZC3HE_MOUSE]	0.53	0.62	0.59	0.68	0.00
P34928	Apolipoprotein C-I OS=Mus musculus GN=Apoc1 PE=2 SV=1 - [APOC1_MOUSE]	1.99	2.04	1.67	2.09	0.00
Q9QXC1	Fetuin-B OS=Mus musculus GN=Fetub PE=1 SV=1 - [FETUB_MOUSE]	1.01	0.82	0.99	0.89	0.00
Q61179	Interferon regulatory factor 9 OS=Mus musculus GN=Irf9 PE=1 SV=1 - [IRF9_MOUSE]	1.07	1.04	1.26	1.23	0.00
F8WHL1	Zinc transporter 9 OS=Mus musculus GN=Slc30a9 PE=2 SV=1 - [F8WHL1_MOUSE]	-0.71	-0.77	-0.61	-0.67	0.00
Q9D816	Cytochrome P450 2C55 OS=Mus musculus GN=Cyp2c55 PE=2 SV=1 - [CP255_MOUSE]	0.54	0.44	0.51	0.46	0.00
Q91WE2	Protein FAM192A OS=Mus musculus GN=Fam192a PE=2 SV=1 - [F192A_MOUSE]	0.75	0.74	0.89	0.88	0.00
Q68FL6	Methionine--tRNA ligase, cytoplasmic OS=Mus musculus GN=Mars PE=2 SV=1 - [SYMC_MOUSE]	-0.36	-0.39	-0.32	-0.39	0.00
D3YW52	Alpha-2-macroglobulin OS=Mus musculus GN=Pzp PE=2 SV=1 - [D3YW52_MOUSE]	1.16	1.19	1.38	1.40	0.00
G3UXX3	Sepiapterin reductase OS=Mus musculus GN=Spr PE=2 SV=1 - [G3UXX3_MOUSE]	-0.30	-0.32	-0.27	-0.34	0.00
Q9JIH2	Nuclear pore complex protein Nup50 OS=Mus musculus GN=Nup50 PE=1 SV=3 - [NUP50_MOUSE]	0.62	0.56	0.53	0.50	0.00
P56812	Programmed cell death protein 5 OS=Mus musculus GN=Pdcd5 PE=1 SV=3 - [PDCD5_MOUSE]	0.47	0.37	0.43	0.41	0.00
Q91V12-2	Isoform A of Cytosolic acyl coenzyme A thioester hydrolase OS=Mus musculus GN=Acot7 - [BACH_MOUSE]	-0.45	-0.44	-0.41	-0.36	0.00
Q9D1M0	Protein SEC13 homolog OS=Mus musculus GN=Sec13 PE=2 SV=3 - [SEC13_MOUSE]	0.13	0.11	0.14	0.12	0.00
O08553	Dihydropyrimidinase-related protein 2 OS=Mus musculus GN=Dpysl2 PE=1 SV=2 - [DPYL2_MOUSE]	0.29	0.30	0.35	0.28	0.00
P48036	Annexin A5 OS=Mus musculus GN=Anxa5 PE=1 SV=1 - [ANXA5_MOUSE]	0.18	0.15	0.18	0.18	0.00
E9Q0B5	Protein Fcgbp OS=Mus musculus GN=Fcgbp PE=2 SV=1 - [E9Q0B5_MOUSE]	-0.80	-0.66	-0.82	-0.70	0.00
D3YY41	Phosphatidylinositol 3-kinase, catalytic, alpha polypeptide, isoform CRA_a OS=Mus musculus GN=Pik3ca PE=4 SV=1 - [D3YY41_MOUSE]	-0.89	-0.92	-0.75	-0.78	0.00

D3YVR9	MCG7316, isoform CRA_b OS=Mus musculus GN=1110004F10Rik PE=4 SV=1 - [D3YVR9_MOUSE]	1.48	1.83	1.51	1.65	0.00
P01644	Ig kappa chain V-V region HP R16.7 OS=Mus musculus PE=1 SV=1 - [KV5AB_MOUSE]	-0.69	-0.66	-0.56	-0.59	0.00
Q9Z2Z6	Mitochondrial carnitine/acylcarnitine carrier protein OS=Mus musculus GN=Slc25a20 PE=1 SV=1 - [MCAT_MOUSE]	-0.84	-0.72	-0.87	-0.72	0.00
Q91W59-2	Isoform 2 of RNA-binding motif, single-stranded-interacting protein 1 OS=Mus musculus GN=Rbms1 - [RBMS1_MOUSE]	0.75	0.65	0.81	0.67	0.00
Q62318	Transcription intermediary factor 1-beta OS=Mus musculus GN=Trim28 PE=1 SV=3 - [TIF1B_MOUSE]	0.19	0.16	0.20	0.20	0.00
P10833	Ras-related protein R-Ras OS=Mus musculus GN=Rras PE=2 SV=1 - [RRAS_MOUSE]	-0.35	-0.41	-0.43	-0.44	0.00
Q62383	Transcription elongation factor SPT6 OS=Mus musculus GN=Supt6h PE=1 SV=2 - [SPT6H_MOUSE]	0.45	0.52	0.41	0.48	0.00
F6TQW2	Protein Ighg2c OS=Mus musculus GN=Ighg2c PE=4 SV=1 - [F6TQW2_MOUSE]	-1.14	-1.27	-1.22	-1.44	0.00
P01843	Ig lambda-1 chain C region OS=Mus musculus PE=1 SV=1 - [LAC1_MOUSE]	1.48	1.60	1.74	1.87	0.00
Q9WU78	Programmed cell death 6-interacting protein OS=Mus musculus GN=Pdcd6ip PE=1 SV=3 - [PDC6I_MOUSE]	-0.53	-0.61	-0.50	-0.49	0.00
Q8BHP7	Olfactomedin-like protein 2A OS=Mus musculus GN=Olfml2a PE=1 SV=1 - [OLM2A_MOUSE]	-0.92	-0.78	-0.91	-0.76	0.00
E9Q7L0	Protein Ogdhl OS=Mus musculus GN=Ogdhl PE=2 SV=1 - [E9Q7L0_MOUSE]	-0.24	-0.22	-0.28	-0.26	0.00
D3Z5N4	Glycogenin-1 (Fragment) OS=Mus musculus GN=Gyg PE=2 SV=2 - [D3Z5N4_MOUSE]	2.37	2.09	2.67	2.38	0.00
Q9CQU1	Microfibrillar-associated protein 1 OS=Mus musculus GN=Mfap1 PE=1 SV=1 - [MFAP1_MOUSE]	0.80	0.98	0.82	0.82	0.00
G3X8Y3	N-alpha-acetyltransferase 15, NatA auxiliary subunit OS=Mus musculus GN=Naa15 PE=4 SV=1 - [G3X8Y3_MOUSE]	-0.41	-0.48	-0.49	-0.52	0.00
Q91Z53	Glyoxylate reductase/hydroxypyruvate reductase OS=Mus musculus GN=Grhpr PE=1 SV=1 - [GRHPR_MOUSE]	0.37	0.36	0.33	0.30	0.00
Q9D2E2-2	Isoform 2 of Target of EGR1 protein 1 OS=Mus musculus GN=Toe1 - [TOE1_MOUSE]	0.37	0.44	0.38	0.44	0.00
Q64727	Vinculin OS=Mus musculus GN=Vcl PE=1 SV=4 - [VINC_MOUSE]	-0.34	-0.39	-0.31	-0.37	0.00

Q4VA93	Protein kinase C OS=Mus musculus GN=Prkca PE=2 SV=1 - [Q4VA93_MOUSE]	-0.12	-0.13	-0.15	-0.14	0.00
Q2KN98	Cytospin-A OS=Mus musculus GN=Specc1l PE=1 SV=1 - [CYTSA_MOUSE]	0.70	0.57	0.58	0.57	0.00
Q9CQZ6	NADH dehydrogenase [ubiquinone] 1 beta subcomplex subunit 3 OS=Mus musculus GN=Ndufb3 PE=1 SV=1 - [NDUB3_MOUSE]	-0.45	-0.38	-0.36	-0.39	0.00
E9PYF7	AP1AR OS=Mus musculus GN=Ap1ar PE=4 SV=1 - [E9PYF7_MOUSE]	0.58	0.70	0.59	0.71	0.00
Q9JM62-2	Isoform 2 of Receptor expression-enhancing protein 6 OS=Mus musculus GN=Reep6 - [REEP6_MOUSE]	0.18	0.15	0.18	0.15	0.00
Q9CQ60	6-phosphogluconolactonase OS=Mus musculus GN=Pgls PE=2 SV=1 - [6PGL_MOUSE]	-0.33	-0.26	-0.27	-0.30	0.00
Q3USH5	Splicing factor, suppressor of white-apricot homolog OS=Mus musculus GN=Sfswap PE=1 SV=2 - [SFSWA_MOUSE]	0.60	0.71	0.63	0.75	0.00
Q80WQ8-3	Isoform 3 of Mis18-binding protein 1 OS=Mus musculus GN=Mis18bp1 - [M18BP_MOUSE]	-0.95	-0.87	-0.83	-0.74	0.00
Q8K2Q9	Shootin-1 OS=Mus musculus GN=Kiaa1598 PE=1 SV=1 - [SHOT1_MOUSE]	0.38	0.48	0.43	0.47	0.00
P81117	Nucleobindin-2 OS=Mus musculus GN=Nucb2 PE=1 SV=2 - [NUCB2_MOUSE]	0.67	0.58	0.74	0.70	0.00
P29788	Vitronectin OS=Mus musculus GN=Vtn PE=1 SV=2 - [VTNC_MOUSE]	0.53	0.59	0.64	0.67	0.00
P19783	Cytochrome c oxidase subunit 4 isoform 1, mitochondrial OS=Mus musculus GN=Cox4i1 PE=1 SV=2 - [COX41_MOUSE]	-0.56	-0.63	-0.49	-0.59	0.00
Q91W50	Cold shock domain-containing protein E1 OS=Mus musculus GN=Csde1 PE=2 SV=1 - [CSDE1_MOUSE]	0.30	0.34	0.38	0.37	0.00
P97868-2	Isoform 2 of E3 ubiquitin-protein ligase RBBP6 OS=Mus musculus GN=Rbbp6 - [RBBP6_MOUSE]	1.00	0.83	0.83	0.81	0.00
D3Z7U6	Calpain-9 (Fragment) OS=Mus musculus GN=Capn9 PE=2 SV=1 - [D3Z7U6_MOUSE]	-1.11	-1.21	-0.94	-1.04	0.00
Q60932-2	Isoform Mt-VDAC1 of Voltage-dependent anion-selective channel protein 1 OS=Mus musculus GN=Vdac1 - [VDAC1_MOUSE]	-0.61	-0.76	-0.68	-0.77	0.00
Q61191	Host cell factor 1 OS=Mus musculus GN=Hcfc1 PE=1 SV=2 - [HCFC1_MOUSE]	0.56	0.64	0.58	0.71	0.00
P01867-2	Isoform 2 of Ig gamma-2B chain C region OS=Mus musculus GN=Igh-3 - [IGG2B_MOUSE]	-0.80	-0.72	-0.72	-0.62	0.00

O35955	Proteasome subunit beta type-10 OS=Mus musculus GN=Psmb10 PE=1 SV=1 - [PSB10_MOUSE]	0.45	0.58	0.51	0.52	0.00
Q9JJY3	Sphingomyelin phosphodiesterase 3 OS=Mus musculus GN=Smpd3 PE=1 SV=1 - [NSMA2_MOUSE]	-0.60	-0.50	-0.59	-0.50	0.00
Q9Z0U1	Tight junction protein ZO-2 OS=Mus musculus GN=Tjp2 PE=1 SV=2 - [ZO2_MOUSE]	0.27	0.26	0.29	0.22	0.00
A2AVJ7	Ribosome-binding protein 1 OS=Mus musculus GN=Rrbp1 PE=2 SV=1 - [A2AVJ7_MOUSE]	0.60	0.57	0.71	0.67	0.00
O09111	NADH dehydrogenase [ubiquinone] 1 beta subcomplex subunit 11, mitochondrial OS=Mus musculus GN=Ndufb11 PE=1 SV=2 - [NDUBB_MOUSE]	-0.55	-0.57	-0.67	-0.54	0.00
P51661	Corticosteroid 11-beta-dehydrogenase isozyme 2 OS=Mus musculus GN=Hsd11b2 PE=2 SV=2 - [DHI2_MOUSE]	-0.65	-0.68	-0.64	-0.53	0.00
Q09200	Beta-1,4 N-acetylgalactosaminyltransferase 1 OS=Mus musculus GN=B4galnt1 PE=2 SV=1 - [B4GN1_MOUSE]	0.98	1.16	1.04	1.24	0.00
Q9CZ44	NSFL1 cofactor p47 OS=Mus musculus GN=Nsfl1c PE=1 SV=1 - [NSF1C_MOUSE]	0.34	0.28	0.35	0.29	0.00
Q9Z2M6	Ubiquitin-like protein 3 OS=Mus musculus GN=Ubl3 PE=1 SV=1 - [UBL3_MOUSE]	-0.48	-0.54	-0.62	-0.54	0.00
Q9CQF3	Cleavage and polyadenylation specificity factor subunit 5 OS=Mus musculus GN=Nudt21 PE=2 SV=1 - [CPSF5_MOUSE]	0.87	1.05	0.86	0.85	0.00
Q76MZ3	Serine/threonine-protein phosphatase 2A 65 kDa regulatory subunit A alpha isoform OS=Mus musculus GN=Ppp2r1a PE=1 SV=3 - [2AAA_MOUSE]	-0.60	-0.71	-0.63	-0.76	0.00
Q0VEK2	MCG3056, isoform CRA_b OS=Mus musculus GN=Chp2 PE=2 SV=1 - [Q0VEK2_MOUSE]	-1.18	-0.99	-1.10	-0.93	0.00
Q1RME4	CAR-like soluble protein OS=Mus musculus GN=Gm1123 PE=2 SV=1 - [Q1RME4_MOUSE]	-0.59	-0.65	-0.74	-0.59	0.00
Q9D0A3	UPF0552 protein C15orf38 homolog OS=Mus musculus PE=2 SV=1 - [CO038_MOUSE]	0.28	0.27	0.34	0.28	0.00
Q91YQ5	Dolichyl-diphosphooligosaccharide--protein glycosyltransferase subunit 1 OS=Mus musculus GN=Rpn1 PE=2 SV=1 - [RPN1_MOUSE]	-0.30	-0.37	-0.31	-0.36	0.00
Q8BMD8	Calcium-binding mitochondrial carrier protein SCaMC-1 OS=Mus musculus GN=Slc25a24 PE=2 SV=1 - [SCMC1_MOUSE]	-0.53	-0.59	-0.46	-0.50	0.00
Q9CQC7	NADH dehydrogenase [ubiquinone] 1 beta subcomplex subunit 4 OS=Mus musculus GN=Ndufb4 PE=1 SV=3 - [NDUB4_MOUSE]	-0.50	-0.40	-0.43	-0.50	0.00
Q9Z315	U4/U6.U5 tri-snRNP-associated protein 1 OS=Mus musculus GN=Sart1 PE=2 SV=1 - [SNUT1_MOUSE]	0.96	0.92	1.04	0.80	0.00

Q9JIF7	Coatomer subunit beta OS=Mus musculus GN=Copb1 PE=1 SV=1 - [COPB_MOUSE]	-0.42	-0.50	-0.50	-0.40	0.00
Q62073	Mitogen-activated protein kinase kinase kinase 7 OS=Mus musculus GN=Map3k7 PE=1 SV=1 - [M3K7_MOUSE]	1.15	0.95	1.10	0.92	0.00
Q8K4Z5	Splicing factor 3A subunit 1 OS=Mus musculus GN=Sf3a1 PE=1 SV=1 - [SF3A1_MOUSE]	0.73	0.60	0.76	0.75	0.00
Q8BG94	COMM domain-containing protein 7 OS=Mus musculus GN=Commd7 PE=2 SV=1 - [COMD7_MOUSE]	-0.63	-0.59	-0.53	-0.49	0.00
Q62470-3	Isoform 3 of Integrin alpha-3 OS=Mus musculus GN=Itga3 - [ITA3_MOUSE]	-0.53	-0.49	-0.64	-0.54	0.00
F6RJ39	Apoptotic chromatin condensation inducer in the nucleus (Fragment) OS=Mus musculus GN=Acin1 PE=4 SV=1 - [F6RJ39_MOUSE]	0.84	0.71	0.93	0.86	0.00
P04117	Fatty acid-binding protein, adipocyte OS=Mus musculus GN=Fabp4 PE=1 SV=3 - [FABP4_MOUSE]	0.67	0.70	0.85	0.80	0.00
Q60767	Lymphocyte antigen 75 OS=Mus musculus GN=Ly75 PE=1 SV=2 - [LY75_MOUSE]	-1.10	-1.01	-1.31	-1.18	0.00
Q5SYD0	Unconventional myosin-Id OS=Mus musculus GN=Myo1d PE=1 SV=1 - [MYO1D_MOUSE]	-0.63	-0.58	-0.57	-0.48	0.00
G3UVU2	Splicing factor 3A subunit 2 OS=Mus musculus GN=Sf3a2 PE=4 SV=1 - [G3UVU2_MOUSE]	0.76	0.67	0.82	0.65	0.00
Q91WS0	CDGSH iron-sulfur domain-containing protein 1 OS=Mus musculus GN=Cisd1 PE=1 SV=1 - [CISD1_MOUSE]	-0.35	-0.31	-0.36	-0.28	0.00
Q921M7	Protein FAM49B OS=Mus musculus GN=Fam49b PE=2 SV=1 - [FA49B_MOUSE]	-0.34	-0.36	-0.29	-0.38	0.00
Q99KD5	Protein unc-45 homolog A OS=Mus musculus GN=Unc45a PE=1 SV=2 - [UN45A_MOUSE]	-0.87	-0.75	-0.67	-0.73	0.00
Q8VHQ9	Acyl-coenzyme A thioesterase 11 OS=Mus musculus GN=Acot11 PE=2 SV=1 - [ACO11_MOUSE]	-0.45	-0.39	-0.48	-0.38	0.00
E9Q586	Dynactin subunit 1 OS=Mus musculus GN=Dctn1 PE=2 SV=1 - [E9Q586_MOUSE]	-0.29	-0.26	-0.26	-0.22	0.00
P27601	Guanine nucleotide-binding protein subunit alpha-13 OS=Mus musculus GN=Gna13 PE=1 SV=1 - [GNA13_MOUSE]	-0.48	-0.43	-0.47	-0.56	0.00
P19467	Mucin-13 OS=Mus musculus GN=Muc13 PE=2 SV=1 - [MUC13_MOUSE]	-0.60	-0.57	-0.54	-0.46	0.00
P62702	40S ribosomal protein S4, X isoform OS=Mus musculus GN=Rps4x PE=2 SV=2 - [RS4X_MOUSE]	-0.50	-0.44	-0.43	-0.38	0.00

B2RUF0	Y box protein 2 OS=Mus musculus GN=Ybx2 PE=2 SV=1 - [B2RUF0_MOUSE]	-2.18	-2.49	-1.89	-2.20	0.00
P25085-2	Isoform 2 of Interleukin-1 receptor antagonist protein OS=Mus musculus GN=Il1rn - [IL1RA_MOUSE]	-1.16	-1.52	-1.29	-1.31	0.00
P48771	Cytochrome c oxidase subunit 7A2, mitochondrial OS=Mus musculus GN=Cox7a2 PE=1 SV=2 - [CX7A2_MOUSE]	-0.66	-0.79	-0.62	-0.63	0.00
Q9JMH6	Thioredoxin reductase 1, cytoplasmic OS=Mus musculus GN=Txnrd1 PE=1 SV=3 - [TRXR1_MOUSE]	0.13	0.12	0.14	0.16	0.00
F8VQ28	Paxillin OS=Mus musculus GN=Pxn PE=2 SV=1 - [F8VQ28_MOUSE]	0.28	0.31	0.34	0.36	0.00
Q9D1J3	SAP domain-containing ribonucleoprotein OS=Mus musculus GN=Sarnp PE=1 SV=3 - [SARNP_MOUSE]	0.58	0.56	0.63	0.48	0.00
Q8BH61	Coagulation factor XIII A chain OS=Mus musculus GN=F13a1 PE=2 SV=3 - [F13A_MOUSE]	0.60	0.59	0.57	0.72	0.00
Q8K3X6	Ankyrin repeat and SAM domain-containing protein 4B OS=Mus musculus GN=Anks4b PE=1 SV=1 - [ANS4B_MOUSE]	0.52	0.57	0.60	0.68	0.00
E9Q066	La-related protein 4 OS=Mus musculus GN=Larp4 PE=2 SV=1 - [E9Q066_MOUSE]	0.50	0.64	0.53	0.60	0.00
Q9D024	Coiled-coil domain-containing protein 47 OS=Mus musculus GN=Ccdc47 PE=2 SV=2 - [CCD47_MOUSE]	-0.18	-0.19	-0.20	-0.15	0.00
I7HLV2	60S ribosomal protein L10 (Fragment) OS=Mus musculus GN=Rpl10 PE=4 SV=1 - [I7HLV2_MOUSE]	-0.33	-0.43	-0.40	-0.38	0.00
P06728	Apolipoprotein A-IV OS=Mus musculus GN=Apoa4 PE=2 SV=3 - [APOA4_MOUSE]	0.76	0.63	0.78	0.64	0.00
P23475	X-ray repair cross-complementing protein 6 OS=Mus musculus GN=Xrcc6 PE=1 SV=5 - [XRCC6_MOUSE]	-0.50	-0.48	-0.39	-0.43	0.00
Q9WUK2-2	Isoform Short of Eukaryotic translation initiation factor 4H OS=Mus musculus GN=Eif4h - [IF4H_MOUSE]	0.35	0.37	0.40	0.46	0.00
P49710	Hematopoietic lineage cell-specific protein OS=Mus musculus GN=Hcls1 PE=1 SV=2 - [HCLS1_MOUSE]	0.73	0.60	0.64	0.76	0.00
P13597-2	Isoform 2 of Intercellular adhesion molecule 1 OS=Mus musculus GN=Icam1 - [ICAM1_MOUSE]	0.65	0.66	0.79	0.80	0.00
Q61247	Alpha-2-antiplasmin OS=Mus musculus GN=Serpinf2 PE=1 SV=1 - [A2AP_MOUSE]	0.96	1.08	1.20	1.24	0.00
P63024	Vesicle-associated membrane protein 3 OS=Mus musculus GN=Vamp3 PE=1 SV=1 - [VAMP3_MOUSE]	1.12	0.97	1.01	0.85	0.00

Q9D1A2	Cytosolic non-specific dipeptidase OS=Mus musculus GN=Cndp2 PE=1 SV=1 - [CNDP2_MOUSE]	-0.22	-0.22	-0.19	-0.26	0.00
P55050	Fatty acid-binding protein, intestinal OS=Mus musculus GN=Fabp2 PE=2 SV=2 - [FABPI_MOUSE]	1.03	0.90	1.18	1.12	0.00
Q921L5	Conserved oligomeric Golgi complex subunit 2 OS=Mus musculus GN=Cog2 PE=2 SV=2 - [COG2_MOUSE]	-0.48	-0.42	-0.54	-0.54	0.00
G3X9T8	Ceruloplasmin OS=Mus musculus GN=Cp PE=4 SV=1 - [G3X9T8_MOUSE]	0.75	0.92	0.85	0.98	0.00
Q8CAQ8	Mitochondrial inner membrane protein OS=Mus musculus GN=Immt PE=1 SV=1 - [IMMT_MOUSE]	-0.32	-0.37	-0.36	-0.42	0.00
Q6PD03	Serine/threonine-protein phosphatase 2A 56 kDa regulatory subunit alpha isoform OS=Mus musculus GN=Ppp2r5a PE=1 SV=1 - [2A5A_MOUSE]	-0.69	-0.79	-0.91	-0.84	0.00
Q60714	Long-chain fatty acid transport protein 1 OS=Mus musculus GN=Slc27a1 PE=1 SV=1 - [S27A1_MOUSE]	0.92	0.70	0.83	0.89	0.00
Q91WG0	Carboxylesterase 2 OS=Mus musculus GN=Ces2c PE=2 SV=1 - [Q91WG0_MOUSE]	-0.28	-0.22	-0.27	-0.24	0.00
Q6PB89	Adarb2 protein OS=Mus musculus GN=Adarb2 PE=2 SV=1 - [Q6PB89_MOUSE]	0.63	0.69	0.76	0.82	0.00
Q8VCT4	Carboxylesterase 1D OS=Mus musculus GN=Ces1d PE=1 SV=1 - [CES1D_MOUSE]	1.28	1.28	1.57	1.56	0.00
O08715-2	Isoform 1 of A-kinase anchor protein 1, mitochondrial OS=Mus musculus GN=Akap1 - [AKAP1_MOUSE]	0.69	0.88	0.75	0.88	0.00
Q9JHJ0	Tropomodulin-3 OS=Mus musculus GN=Tmod3 PE=1 SV=1 - [TMOD3_MOUSE]	0.32	0.28	0.26	0.24	0.00
Q8VDP6	CDP-diacylglycerol--inositol 3-phosphatidyltransferase OS=Mus musculus GN=Cdipt PE=1 SV=1 - [CDIPT_MOUSE]	-0.57	-0.70	-0.63	-0.75	0.00
Q9JLQ0	CD2-associated protein OS=Mus musculus GN=Cd2ap PE=1 SV=3 - [CD2AP_MOUSE]	0.37	0.49	0.42	0.45	0.00
Q6P9Q6	FK506-binding protein 15 OS=Mus musculus GN=Fkbp15 PE=1 SV=2 - [FKB15_MOUSE]	0.63	0.62	0.53	0.50	0.00
Q8VHR5	Transcriptional repressor p66-beta OS=Mus musculus GN=Gatad2b PE=1 SV=1 - [P66B_MOUSE]	1.39	1.19	1.58	1.38	0.00
Q9EQH2	Endoplasmic reticulum aminopeptidase 1 OS=Mus musculus GN=Erap1 PE=2 SV=2 - [ERAP1_MOUSE]	-0.32	-0.41	-0.34	-0.33	0.00
Q9DB77	Cytochrome b-c1 complex subunit 2, mitochondrial OS=Mus musculus GN=Uqcrc2 PE=1 SV=1 - [QCR2_MOUSE]	-0.47	-0.37	-0.45	-0.39	0.00

B0QZL2	Nicotinamide mononucleotide adenylyltransferase 1 OS=Mus musculus GN=Nmnat1 PE=2 SV=1 - [B0QZL2_MOUSE]	0.74	0.83	0.62	0.71	0.00
P11499	Heat shock protein HSP 90-beta OS=Mus musculus GN=Hsp90ab1 PE=1 SV=3 - [HS90B_MOUSE]	-0.44	-0.49	-0.37	-0.43	0.00
Q4PZA2-3	Isoform C of Endothelin-converting enzyme 1 OS=Mus musculus GN=Ece1 - [ECE1_MOUSE]	-0.49	-0.45	-0.37	-0.44	0.00
E9Q0A3	Protein Arhgef11 (Fragment) OS=Mus musculus GN=Arhgef11 PE=2 SV=1 - [E9Q0A3_MOUSE]	0.69	0.67	0.57	0.55	0.00
Q9D892	Inosine triphosphate pyrophosphatase OS=Mus musculus GN=Itpa PE=1 SV=2 - [ITPA_MOUSE]	-0.43	-0.34	-0.45	-0.38	0.00
Q9D3W4	GPN-loop GTPase 3 OS=Mus musculus GN=Gpn3 PE=2 SV=1 - [GPN3_MOUSE]	-0.37	-0.39	-0.30	-0.33	0.00
A1BN54	Alpha actinin 1a OS=Mus musculus GN=Actn1 PE=2 SV=1 - [A1BN54_MOUSE]	-0.59	-0.50	-0.67	-0.58	0.00
E9Q7Z8	Protein Ythdc1 OS=Mus musculus GN=Ythdc1 PE=2 SV=1 - [E9Q7Z8_MOUSE]	0.52	0.61	0.46	0.55	0.00
Q9D2R6	Cytochrome c oxidase assembly protein 3 homolog, mitochondrial OS=Mus musculus GN=Coa3 PE=2 SV=1 - [COA3_MOUSE]	-0.48	-0.53	-0.42	-0.41	0.00
O09160	Galactoside 2-alpha-L-fucosyltransferase 1 OS=Mus musculus GN=Fut1 PE=2 SV=1 - [FUT1_MOUSE]	0.47	0.39	0.44	0.36	0.00
O70145	Neutrophil cytosol factor 2 OS=Mus musculus GN=Ncf2 PE=2 SV=1 - [NCF2_MOUSE]	0.59	0.69	0.63	0.77	0.00
B7ZC19	GPI transamidase component PIG-T OS=Mus musculus GN=Pigt PE=2 SV=1 - [B7ZC19_MOUSE]	-0.51	-0.50	-0.43	-0.40	0.00
O35744	Chitinase-3-like protein 3 OS=Mus musculus GN=Chi3l3 PE=1 SV=2 - [CH3L3_MOUSE]	1.12	1.43	1.29	1.46	0.00
Q9EPB4	Apoptosis-associated speck-like protein containing a CARD OS=Mus musculus GN=Pycard PE=1 SV=1 - [ASC_MOUSE]	0.45	0.39	0.52	0.45	0.00
Q6NZR5	Protein Skiv2l OS=Mus musculus GN=Skiv2l PE=2 SV=1 - [Q6NZR5_MOUSE]	-0.33	-0.26	-0.34	-0.32	0.00
F6V6T4	Transmembrane emp24 domain-containing protein 2 (Fragment) OS=Mus musculus GN=Tmed2 PE=3 SV=1 - [F6V6T4_MOUSE]	-0.15	-0.16	-0.12	-0.13	0.00
Q80YX1-2	Isoform 2 of Tenascin OS=Mus musculus GN=Tnc - [TENA_MOUSE]	-0.22	-0.18	-0.23	-0.24	0.00
Q9DBB8	Trans-1,2-dihydrobenzene-1,2-diol dehydrogenase OS=Mus musculus GN=Dhdh PE=2 SV=1 - [DHDH_MOUSE]	0.33	0.34	0.30	0.26	0.00

A2AMD2	Protein Ankrd60 OS=Mus musculus GN=Ankrd60 PE=2 SV=2 - [A2AMD2_MOUSE]	-0.81	-0.71	-0.94	-0.85	0.00
Q02053	Ubiquitin-like modifier-activating enzyme 1 OS=Mus musculus GN=Uba1 PE=1 SV=1 - [UBA1_MOUSE]	-0.16	-0.14	-0.13	-0.13	0.00
D3YVR5	DDB1- and CUL4-associated factor 11 (Fragment) OS=Mus musculus GN=Dcaf11 PE=2 SV=1 - [D3YVR5_MOUSE]	0.22	0.20	0.19	0.16	0.00
P13439	Uridine 5'-monophosphate synthase OS=Mus musculus GN=Umps PE=2 SV=3 - [UMPS_MOUSE]	-0.48	-0.49	-0.58	-0.59	0.00
P09055	Integrin beta-1 OS=Mus musculus GN=Itgb1 PE=1 SV=1 - [ITB1_MOUSE]	-0.38	-0.45	-0.38	-0.48	0.00
P31532	Serum amyloid A-4 protein OS=Mus musculus GN=Saa4 PE=1 SV=2 - [SAA4_MOUSE]	0.60	0.49	0.64	0.53	0.00
Q8CGF7-2	Isoform 2 of Transcription elongation regulator 1 OS=Mus musculus GN=Tcerg1 - [TCRG1_MOUSE]	0.52	0.50	0.62	0.48	0.00
Q9EPQ7	StAR-related lipid transfer protein 5 OS=Mus musculus GN=Stard5 PE=2 SV=2 - [STAR5_MOUSE]	0.54	0.45	0.59	0.48	0.00
Q91V92	ATP-citrate synthase OS=Mus musculus GN=Acly PE=1 SV=1 - [ACLY_MOUSE]	-1.01	-1.05	-1.24	-1.28	0.00
E9PY90	cTAGE family member 5 OS=Mus musculus GN=Ctage5 PE=2 SV=1 - [E9PY90_MOUSE]	0.20	0.21	0.16	0.20	0.00
P43024	Cytochrome c oxidase subunit 6A1, mitochondrial OS=Mus musculus GN=Cox6a1 PE=1 SV=2 - [CX6A1_MOUSE]	-0.47	-0.42	-0.35	-0.42	0.00
A2A863-2	Isoform 2 of Integrin beta-4 OS=Mus musculus GN=Itgb4 - [ITB4_MOUSE]	-0.50	-0.56	-0.64	-0.64	0.00
Q3TDT0	Tripartite motif-containing protein 3 OS=Mus musculus GN=Trim3 PE=2 SV=1 - [Q3TDT0_MOUSE]	-0.26	-0.23	-0.29	-0.22	0.00
Q91WT9-2	Isoform 2 of Cystathionine beta-synthase OS=Mus musculus GN=Cbs - [CBS_MOUSE]	0.43	0.37	0.49	0.45	0.00
P07901	Heat shock protein HSP 90-alpha OS=Mus musculus GN=Hsp90aa1 PE=1 SV=4 - [HS90A_MOUSE]	-0.67	-0.68	-0.53	-0.57	0.00
Q9CZW4	Long-chain-fatty-acid--CoA ligase 3 OS=Mus musculus GN=Acsl3 PE=2 SV=2 - [ACSL3_MOUSE]	-0.93	-0.89	-0.71	-0.79	0.00
E9Q9C7	Actin-binding LIM protein 1 OS=Mus musculus GN=Ablim1 PE=2 SV=1 - [E9Q9C7_MOUSE]	0.68	0.89	0.89	0.84	0.00
Q8K3X4	Interferon regulatory factor 2-binding protein-like OS=Mus musculus GN=Irf2bpl PE=1 SV=1 - [I2BPL_MOUSE]	0.36	0.36	0.31	0.28	0.00

Q62418-3	Isoform 3 of Drebrin-like protein OS=Mus musculus GN=Dbnl - [DBNL_MOUSE]	0.56	0.49	0.61	0.47	0.00
P56528	ADP-ribosyl cyclase 1 OS=Mus musculus GN=Cd38 PE=1 SV=2 - [CD38_MOUSE]	-0.97	-0.83	-0.85	-0.72	0.00
Q9D3S3	Sorting nexin-29 OS=Mus musculus GN=Snx29 PE=1 SV=2 - [SNX29_MOUSE]	1.52	1.57	1.88	1.92	0.00
P20491	High affinity immunoglobulin epsilon receptor subunit gamma OS=Mus musculus GN=Fcer1g PE=1 SV=1 - [FCERG_MOUSE]	0.58	0.71	0.61	0.74	0.00
Q9D662	Protein transport protein Sec23B OS=Mus musculus GN=Sec23b PE=2 SV=1 - [SC23B_MOUSE]	0.17	0.21	0.23	0.20	0.00
P97290	Plasma protease C1 inhibitor OS=Mus musculus GN=Serping1 PE=1 SV=3 - [IC1_MOUSE]	0.75	0.90	0.99	0.98	0.00
Q99KR8	Plasma alpha-L-fucosidase OS=Mus musculus GN=Fuca2 PE=2 SV=1 - [FUCO2_MOUSE]	-0.35	-0.46	-0.38	-0.38	0.00
Q58A65-2	Isoform 2 of C-Jun-amino-terminal kinase-interacting protein 4 OS=Mus musculus GN=Spag9 - [JIP4_MOUSE]	0.42	0.42	0.41	0.52	0.00
Q5NCM6	Epsin-2 OS=Mus musculus GN=Epn2 PE=2 SV=1 - [Q5NCM6_MOUSE]	0.77	0.90	0.89	1.03	0.00
P31938	Dual specificity mitogen-activated protein kinase kinase 1 OS=Mus musculus GN=Map2k1 PE=1 SV=2 - [MP2K1_MOUSE]	-1.03	-0.91	-0.89	-0.77	0.00
A2AFR3-2	Isoform 2 of FERM and PDZ domain-containing protein 4 OS=Mus musculus GN=Frmpd4 - [FRPD4_MOUSE]	-0.14	-0.17	-0.16	-0.19	0.00
O09131	Glutathione S-transferase omega-1 OS=Mus musculus GN=Gsto1 PE=2 SV=2 - [GSTO1_MOUSE]	-0.59	-0.50	-0.49	-0.45	0.00
Q9JKB3-2	Isoform 2 of Y-box-binding protein 3 OS=Mus musculus GN=Ybx3 - [YBOX3_MOUSE]	0.31	0.29	0.26	0.24	0.00
P97927	Laminin subunit alpha-4 OS=Mus musculus GN=Lama4 PE=1 SV=2 - [LAMA4_MOUSE]	0.30	0.32	0.35	0.40	0.00
O88952	Protein lin-7 homolog C OS=Mus musculus GN=Lin7c PE=1 SV=2 - [LIN7C_MOUSE]	0.43	0.42	0.54	0.44	0.00
Q91YL3	Uridine-cytidine kinase-like 1 OS=Mus musculus GN=Uckl1 PE=1 SV=1 - [UCKL1_MOUSE]	0.26	0.21	0.28	0.24	0.00
O88532	Zinc finger RNA-binding protein OS=Mus musculus GN=Zfr PE=1 SV=2 - [ZFR_MOUSE]	0.32	0.29	0.26	0.25	0.00
O54990-6	Isoform 6 of Prominin-1 OS=Mus musculus GN=Prom1 - [PROM1_MOUSE]	0.92	0.72	0.83	0.71	0.00

F8VPK0	Protein Ttc37 OS=Mus musculus GN=Ttc37 PE=2 SV=1 - [F8VPK0_MOUSE]	-0.53	-0.40	-0.46	-0.51	0.00
Q3UMF0-3	Isoform 3 of Cordon-bleu protein-like 1 OS=Mus musculus GN=Cobll1 - [COBL1_MOUSE]	0.64	0.56	0.50	0.50	0.00
P29416	Beta-hexosaminidase subunit alpha OS=Mus musculus GN=Hexa PE=2 SV=2 - [HEXA_MOUSE]	-0.40	-0.37	-0.46	-0.48	0.00
Q3U319	E3 ubiquitin-protein ligase BRE1B OS=Mus musculus GN=Rnf40 PE=2 SV=2 - [BRE1B_MOUSE]	0.51	0.63	0.50	0.49	0.00
Q3TLH4	Protein PRRC2C OS=Mus musculus GN=Prrc2c PE=1 SV=3 - [PRC2C_MOUSE]	0.45	0.37	0.46	0.37	0.00
Q91WT7	3-alpha-hydroxysteroid dehydrogenase type 1 OS=Mus musculus GN=Akr1c14 PE=2 SV=1 - [Q91WT7_MOUSE]	-0.47	-0.51	-0.40	-0.40	0.00
P70404	Isocitrate dehydrogenase [NAD] subunit gamma 1, mitochondrial OS=Mus musculus GN=Idh3g PE=1 SV=1 - [IDHG1_MOUSE]	-0.70	-0.58	-0.78	-0.73	0.00
B7ZNG0-2	Isoform 2 of Kinesin-like protein KIF7 OS=Mus musculus GN=Kif7 - [KIF7_MOUSE]	-0.45	-0.38	-0.41	-0.34	0.00
F7CIB0	Centrosomal protein of 128 kDa (Fragment) OS=Mus musculus GN=Cep128 PE=2 SV=1 - [F7CIB0_MOUSE]	0.89	0.77	1.03	0.91	0.00
P46414	Cyclin-dependent kinase inhibitor 1B OS=Mus musculus GN=Cdkn1b PE=1 SV=2 - [CDN1B_MOUSE]	0.53	0.66	0.51	0.63	0.00
G5E8T0	DnaJ (Hsp40) homolog, subfamily C, member 5, isoform CRA_b OS=Mus musculus GN=Dnajc5 PE=4 SV=1 - [G5E8T0_MOUSE]	-0.46	-0.58	-0.46	-0.46	0.00
Q6PB66	Leucine-rich PPR motif-containing protein, mitochondrial OS=Mus musculus GN=Lrpprc PE=1 SV=2 - [LPPRC_MOUSE]	-0.85	-0.69	-0.85	-0.68	0.00
Q5I0W6	Methyltransferase like 7A2 OS=Mus musculus GN=Ubie PE=2 SV=1 - [Q5I0W6_MOUSE]	-1.82	-1.90	-2.25	-2.33	0.00
E9QNG1	Intersectin-2 OS=Mus musculus GN=Itsn2 PE=2 SV=1 - [E9QNG1_MOUSE]	0.44	0.44	0.40	0.53	0.00
Q99L20	Glutathione S-transferase theta 3 OS=Mus musculus GN=Gstt3 PE=2 SV=1 - [Q99L20_MOUSE]	-0.59	-0.61	-0.73	-0.76	0.00
Q8CFE3	REST corepressor 1 OS=Mus musculus GN=Rcor1 PE=1 SV=2 - [RCOR1_MOUSE]	0.69	0.84	0.64	0.78	0.00
P43241	Homeobox protein CDX-2 OS=Mus musculus GN=Cdx2 PE=1 SV=1 - [CDX2_MOUSE]	0.83	0.90	1.02	1.09	0.00
P67984	60S ribosomal protein L22 OS=Mus musculus GN=Rpl22 PE=2 SV=2 - [RL22_MOUSE]	0.23	0.26	0.31	0.27	0.00

P13634	Carbonic anhydrase 1 OS=Mus musculus GN=Ca1 PE=2 SV=4 - [CAH1_MOUSE]	0.92	0.86	1.12	1.06	0.00
Q61739	Integrin alpha-6 OS=Mus musculus GN=Itga6 PE=1 SV=3 - [ITA6_MOUSE]	-0.58	-0.49	-0.66	-0.60	0.00
Q63886	UDP-glucuronosyltransferase 1-1 OS=Mus musculus GN=Ugt1a1 PE=2 SV=2 - [UD11_MOUSE]	0.44	0.52	0.58	0.47	0.00
Q9WUA2	Phenylalanine--tRNA ligase beta subunit OS=Mus musculus GN=Farsb PE=2 SV=2 - [SYFB_MOUSE]	-0.45	-0.35	-0.37	-0.35	0.00
Q2KHK6	Gasdermin-C2 OS=Mus musculus GN=Gsdmc2 PE=2 SV=2 - [GSDC2_MOUSE]	0.65	0.59	0.76	0.77	0.00
D6RCG2	Dolichyl-phosphate beta-glucosyltransferase OS=Mus musculus GN=Alg5 PE=4 SV=1 - [D6RCG2_MOUSE]	-0.22	-0.26	-0.19	-0.22	0.00
F6YU83	Serine/threonine-protein kinase PDIK1L (Fragment) OS=Mus musculus GN=Pdik1l PE=2 SV=1 - [F6YU83_MOUSE]	0.18	0.22	0.19	0.23	0.00
Q6RHR9-2	Isoform 2 of Membrane-associated guanylate kinase, WW and PDZ domain-containing protein 1 OS=Mus musculus GN=Magi1 - [MAGI1_MOUSE]	1.04	1.13	0.84	1.07	0.00
Q9Z1T2	Thrombospondin-4 OS=Mus musculus GN=Thbs4 PE=1 SV=1 - [TSP4_MOUSE]	-1.42	-1.33	-1.76	-1.46	0.00
Q9R190	Metastasis-associated protein MTA2 OS=Mus musculus GN=Mta2 PE=1 SV=1 - [MTA2_MOUSE]	0.60	0.50	0.45	0.49	0.00
P70695	Fructose-1,6-bisphosphatase isozyme 2 OS=Mus musculus GN=Fbp2 PE=2 SV=2 - [F16P2_MOUSE]	-0.32	-0.37	-0.28	-0.29	0.00
G3X9Y6	Aldo-keto reductase family 1, member C19 OS=Mus musculus GN=Akr1c19 PE=4 SV=1 - [G3X9Y6_MOUSE]	0.52	0.62	0.52	0.66	0.00
P21570	Angiogenin OS=Mus musculus GN=Ang PE=1 SV=1 - [ANGI_MOUSE]	1.47	1.19	1.62	1.46	0.00
A2AQ53	Fibrillin-1 OS=Mus musculus GN=Fbn1 PE=4 SV=1 - [A2AQ53_MOUSE]	0.47	0.55	0.42	0.53	0.00
Q4ZJM7	Otolin-1 OS=Mus musculus GN=Otol1 PE=1 SV=2 - [OTOL1_MOUSE]	-0.30	-0.31	-0.25	-0.24	0.00
E9Q035	Protein Gm20425 OS=Mus musculus GN=Gm20425 PE=4 SV=1 - [E9Q035_MOUSE]	0.74	0.72	0.92	0.89	0.00
Q3UFK1	Coiled-coil domain containing 28A OS=Mus musculus GN=Ccdc28a PE=2 SV=1 - [Q3UFK1_MOUSE]	2.28	1.84	2.01	1.71	0.00
Q80US4	Actin-related protein 5 OS=Mus musculus GN=Actr5 PE=2 SV=3 - [ARP5_MOUSE]	0.12	0.15	0.11	0.13	0.00

O55060	Thiopurine S-methyltransferase OS=Mus musculus GN=Tpmt PE=1 SV=1 - [TPMT_MOUSE]	-0.40	-0.38	-0.31	-0.32	0.00
E9QP99	Golgin subfamily A member 3 OS=Mus musculus GN=Golga3 PE=2 SV=1 - [E9QP99_MOUSE]	0.36	0.34	0.39	0.45	0.00
Q9D2R0	Acetoacetyl-CoA synthetase OS=Mus musculus GN=Aacs PE=1 SV=1 - [AACS_MOUSE]	-0.42	-0.32	-0.40	-0.34	0.00
Q3URN6	Pregnancy-specific glycoprotein 29 OS=Mus musculus GN=Psg29 PE=2 SV=1 - [Q3URN6_MOUSE]	-0.96	-1.06	-1.19	-1.29	0.00
D3YXZ7	UDP-N-acetylglucosamine transporter (Fragment) OS=Mus musculus GN=Slc35a3 PE=2 SV=1 - [D3YXZ7_MOUSE]	-1.03	-1.05	-0.82	-0.84	0.00
Q148R4	Protein Spink5 OS=Mus musculus GN=Spink5 PE=2 SV=1 - [Q148R4_MOUSE]	-0.45	-0.45	-0.36	-0.36	0.00
Q925I1	ATPase family AAA domain-containing protein 3 OS=Mus musculus GN=Atad3 PE=1 SV=1 - [ATAD3_MOUSE]	-0.35	-0.30	-0.30	-0.26	0.00
Q3U0V1	Far upstream element-binding protein 2 OS=Mus musculus GN=Khsrp PE=1 SV=2 - [FUBP2_MOUSE]	0.50	0.45	0.61	0.54	0.00
E9Q3G8	Protein Nup153 OS=Mus musculus GN=Nup153 PE=2 SV=1 - [E9Q3G8_MOUSE]	0.76	0.94	1.02	1.00	0.00
E9PWI6	GRIP and coiled-coil domain-containing protein 2 OS=Mus musculus GN=Gcc2 PE=2 SV=1 - [E9PWI6_MOUSE]	0.65	0.52	0.51	0.50	0.00
F6QRE9	Protein BC007180 (Fragment) OS=Mus musculus GN=BC007180 PE=4 SV=2 - [F6QRE9_MOUSE]	0.85	0.69	0.92	0.75	0.00
O88713	Killer cell lectin-like receptor subfamily G member 1 OS=Mus musculus GN=Klrg1 PE=1 SV=2 - [KLRG1_MOUSE]	-0.31	-0.24	-0.33	-0.30	0.00
F6SMY7	Probable E3 ubiquitin-protein ligase MYCBP2 (Fragment) OS=Mus musculus GN=Mycbp2 PE=2 SV=1 - [F6SMY7_MOUSE]	1.29	1.20	1.59	1.50	0.00
Q91VN0	Low-density lipoprotein receptor-related protein 5 OS=Mus musculus GN=Lrp5 PE=1 SV=3 - [LRP5_MOUSE]	0.23	0.22	0.19	0.18	0.00
P56391	Cytochrome c oxidase subunit 6B1 OS=Mus musculus GN=Cox6b1 PE=1 SV=2 - [CX6B1_MOUSE]	-0.51	-0.46	-0.41	-0.38	0.00
Q9QZZ6	Dermatopontin OS=Mus musculus GN=Dpt PE=2 SV=1 - [DERM_MOUSE]	0.51	0.53	0.40	0.44	0.00
Q6DFW4	Nucleolar protein 58 OS=Mus musculus GN=Nop58 PE=1 SV=1 - [NOP58_MOUSE]	-0.37	-0.31	-0.34	-0.27	0.00
G3X8U3	MCG6895 OS=Mus musculus GN=2210016F16Rik PE=4 SV=1 - [G3X8U3_MOUSE]	-0.72	-0.55	-0.75	-0.66	0.00

J3QP68	Uncharacterized protein OS=Mus musculus GN=Gm4204 PE=3 SV=1 - [J3QP68_MOUSE]	0.40	0.43	0.54	0.46	0.00
P63073	Eukaryotic translation initiation factor 4E OS=Mus musculus GN=Eif4e PE=1 SV=1 - [IF4E_MOUSE]	0.47	0.42	0.51	0.37	0.00
P28740-2	Isoform 2 of Kinesin-like protein KIF2A OS=Mus musculus GN=Kif2a - [KIF2A_MOUSE]	0.26	0.29	0.25	0.21	0.00
Q8VCM7	Fibrinogen gamma chain OS=Mus musculus GN=Fgg PE=2 SV=1 - [FIBG_MOUSE]	1.65	1.52	1.97	1.99	0.00
Q6PDI5	Proteasome-associated protein ECM29 homolog OS=Mus musculus GN=Ecm29 PE=1 SV=3 - [ECM29_MOUSE]	-0.63	-0.73	-0.54	-0.70	0.00
Q3TCH7	Cullin-4A OS=Mus musculus GN=Cul4a PE=1 SV=1 - [CUL4A_MOUSE]	-0.56	-0.44	-0.54	-0.44	0.00
P01902	H-2 class I histocompatibility antigen, K-D alpha chain OS=Mus musculus GN=H2-K1 PE=1 SV=1 - [HA1D_MOUSE]	-0.40	-0.50	-0.41	-0.51	0.00
A2BFL2	Protein zyg-11 homolog A OS=Mus musculus GN=Zyg11a PE=2 SV=1 - [ZY11A_MOUSE]	-0.67	-0.61	-0.82	-0.76	0.00
P62196	26S protease regulatory subunit 8 OS=Mus musculus GN=Psmc5 PE=1 SV=1 - [PRS8_MOUSE]	-0.24	-0.19	-0.18	-0.20	0.00
Q3UVL4	Vacuolar protein sorting-associated protein 51 homolog OS=Mus musculus GN=Vps51 PE=2 SV=2 - [VPS51_MOUSE]	-0.67	-0.68	-0.85	-0.65	0.00
Q64514-2	Isoform Short of Tripeptidyl-peptidase 2 OS=Mus musculus GN=Tpp2 - [TPP2_MOUSE]	-0.49	-0.41	-0.42	-0.35	0.00
E9QAH1	Protein Golgb1 OS=Mus musculus GN=Golgb1 PE=2 SV=1 - [E9QAH1_MOUSE]	0.44	0.56	0.46	0.57	0.00
Q9DCS9	NADH dehydrogenase [ubiquinone] 1 beta subcomplex subunit 10 OS=Mus musculus GN=Ndufb10 PE=1 SV=3 - [NDUBA_MOUSE]	-0.66	-0.81	-0.63	-0.81	0.00
Q9CQ54	NADH dehydrogenase [ubiquinone] 1 subunit C2 OS=Mus musculus GN=Ndufc2 PE=1 SV=1 - [NDUC2_MOUSE]	-0.58	-0.47	-0.65	-0.57	0.00
Q8CGC7	Bifunctional glutamate/proline--tRNA ligase OS=Mus musculus GN=Eprs PE=1 SV=4 - [SYEP_MOUSE]	-0.51	-0.52	-0.40	-0.42	0.00
P08113	Endoplasmin OS=Mus musculus GN=Hsp90b1 PE=1 SV=2 - [ENPL_MOUSE]	-0.19	-0.22	-0.16	-0.17	0.00
P21107	Tropomyosin alpha-3 chain OS=Mus musculus GN=Tpm3 PE=1 SV=3 - [TPM3_MOUSE]	0.66	0.61	0.81	0.77	0.00
P50543	Protein S100-A11 OS=Mus musculus GN=S100a11 PE=2 SV=1 - [S10AB_MOUSE]	0.91	0.74	0.77	0.67	0.00

P17426-2	Isoform B of AP-2 complex subunit alpha-1 OS=Mus musculus GN=Ap2a1 - [AP2A1_MOUSE]	-0.39	-0.43	-0.37	-0.50	0.00
Q3TC46	Protein PAT1 homolog 1 OS=Mus musculus GN=Patl1 PE=1 SV=2 - [PATL1_MOUSE]	0.65	0.59	0.53	0.48	0.00
A2AMY5	Ubiquitin-associated protein 2 OS=Mus musculus GN=Ubap2 PE=2 SV=1 - [A2AMY5_MOUSE]	0.62	0.59	0.45	0.60	0.00
Q9Z1Z0	General vesicular transport factor p115 OS=Mus musculus GN=Uso1 PE=1 SV=2 - [USO1_MOUSE]	-0.47	-0.39	-0.35	-0.43	0.00
Q61937	Nucleophosmin OS=Mus musculus GN=Npm1 PE=1 SV=1 - [NPM_MOUSE]	0.40	0.34	0.47	0.40	0.00
Q9WTP7	GTP:AMP phosphotransferase AK3, mitochondrial OS=Mus musculus GN=Ak3 PE=1 SV=3 - [KAD3_MOUSE]	-0.79	-0.82	-0.60	-0.71	0.00
Q61550	Double-strand-break repair protein rad21 homolog OS=Mus musculus GN=Rad21 PE=1 SV=3 - [RAD21_MOUSE]	0.40	0.34	0.47	0.44	0.00
Q9QXX8	Nuclear fragile X mental retardation-interacting protein 1 OS=Mus musculus GN=Nufip1 PE=1 SV=1 - [NUFP1_MOUSE]	-1.75	-1.42	-1.62	-1.30	0.00
Q8R1M8	Mucosal pentraxin OS=Mus musculus GN=Mptx1 PE=2 SV=1 - [MPTX_MOUSE]	-1.39	-1.22	-1.65	-1.59	0.00
Q8BH95	Enoyl-CoA hydratase, mitochondrial OS=Mus musculus GN=Echs1 PE=1 SV=1 - [ECHM_MOUSE]	-0.31	-0.40	-0.39	-0.43	0.00
Q68FF0-2	Isoform 2 of Uncharacterized protein KIAA1841 OS=Mus musculus GN=Kiaa1841 - [K1841_MOUSE]	-0.62	-0.71	-0.51	-0.61	0.00
E9Q174	Unconventional myosin-VI OS=Mus musculus GN=Myo6 PE=2 SV=1 - [E9Q174_MOUSE]	0.47	0.53	0.58	0.64	0.00
P47962	60S ribosomal protein L5 OS=Mus musculus GN=Rpl5 PE=1 SV=3 - [RL5_MOUSE]	-0.38	-0.36	-0.49	-0.42	0.00
P21614	Vitamin D-binding protein OS=Mus musculus GN=Gc PE=1 SV=2 - [VTDB_MOUSE]	0.67	0.66	0.77	0.87	0.00
E9Q1T4	Sphingomyelin phosphodiesterase 4 (Fragment) OS=Mus musculus GN=Smpd4 PE=2 SV=1 - [E9Q1T4_MOUSE]	0.45	0.43	0.36	0.34	0.00
A2AW05	FACT complex subunit SSRP1 (Fragment) OS=Mus musculus GN=Ssrp1 PE=2 SV=1 - [A2AW05_MOUSE]	-0.13	-0.14	-0.17	-0.17	0.00
O09105	Neurogenic differentiation factor 4 OS=Mus musculus GN=Neurod4 PE=1 SV=1 - [NDF4_MOUSE]	-0.33	-0.37	-0.27	-0.30	0.00
H3BLA9	Sentrin-specific protease 6 (Fragment) OS=Mus musculus GN=Senp6 PE=2 SV=1 - [H3BLA9_MOUSE]	0.49	0.59	0.67	0.65	0.00

Q99LC3	NADH dehydrogenase [ubiquinone] 1 alpha subcomplex subunit 10, mitochondrial OS=Mus musculus GN=Ndufa10 PE=1 SV=1 - [NDUAA_MOUSE]	-0.36	-0.41	-0.48	-0.48	0.00
Q62472	Vomeronasal secretory protein 2 OS=Mus musculus GN=Lcn4 PE=2 SV=1 - [VNS2_MOUSE]	-2.76	-2.41	-2.33	-1.98	0.00
G3XA21	MCG134445, isoform CRA_a OS=Mus musculus GN=Mroh1 PE=4 SV=1 - [G3XA21_MOUSE]	-1.17	-0.97	-1.06	-0.85	0.00
Q8C7X2-2	Isoform 2 of ER membrane protein complex subunit 1 OS=Mus musculus GN=Emc1 - [EMC1_MOUSE]	-0.32	-0.37	-0.44	-0.41	0.00
Q9D1I2	Bcl10-interacting CARD protein OS=Mus musculus PE=2 SV=1 - [BINCA_MOUSE]	-0.44	-0.54	-0.58	-0.61	0.00
F6TFN2	Protein Lmo7 (Fragment) OS=Mus musculus GN=Lmo7 PE=4 SV=1 - [F6TFN2_MOUSE]	0.74	0.79	0.58	0.79	0.00
Q8BZ00	Sodium/hydrogen exchanger 9 OS=Mus musculus GN=Slc9a9 PE=2 SV=1 - [SL9A9_MOUSE]	1.44	1.24	1.72	1.51	0.00
P21619	Lamin-B2 OS=Mus musculus GN=Lmnb2 PE=1 SV=2 - [LMNB2_MOUSE]	0.22	0.26	0.21	0.19	0.00
D3YY50	Copper homeostasis protein cutC homolog (Fragment) OS=Mus musculus GN=Cutc PE=2 SV=1 - [D3YY50_MOUSE]	0.70	0.85	0.83	0.97	0.00
F6RPD4	Phosphorylated adapter RNA export protein (Fragment) OS=Mus musculus GN=Phax PE=4 SV=1 - [F6RPD4_MOUSE]	0.41	0.33	0.44	0.35	0.00
Q9DAW6	U4/U6 small nuclear ribonucleoprotein Prp4 OS=Mus musculus GN=Prpf4 PE=2 SV=1 - [PRP4_MOUSE]	0.41	0.39	0.49	0.50	0.00
Q9JLI0	Aldo-keto reductase a OS=Mus musculus GN=Akr1c12 PE=2 SV=1 - [Q9JLI0_MOUSE]	0.48	0.65	0.54	0.59	0.00
E9PYT3	Atlastin-3 OS=Mus musculus GN=Atl3 PE=2 SV=1 - [E9PYT3_MOUSE]	-0.53	-0.44	-0.52	-0.40	0.00
P27005	Protein S100-A8 OS=Mus musculus GN=S100a8 PE=1 SV=3 - [S10A8_MOUSE]	1.49	1.24	1.73	1.48	0.00
P70271	PDZ and LIM domain protein 4 OS=Mus musculus GN=Pdlim4 PE=1 SV=3 - [PDLI4_MOUSE]	0.30	0.31	0.23	0.28	0.00
P29351	Tyrosine-protein phosphatase non-receptor type 6 OS=Mus musculus GN=Ptpn6 PE=1 SV=2 - [PTN6_MOUSE]	0.26	0.21	0.30	0.26	0.00
G3UZ34	116 kDa U5 small nuclear ribonucleoprotein component OS=Mus musculus GN=Eftud2 PE=2 SV=1 - [G3UZ34_MOUSE]	0.25	0.35	0.32	0.31	0.00
Q8R1F6-2	Isoform 2 of Protein HID1 OS=Mus musculus GN=Hid1 - [HID1_MOUSE]	-0.75	-0.64	-0.89	-0.78	0.00

Q6S9I0	Kng2 protein OS=Mus musculus GN=Kng2 PE=2 SV=1 - [Q6S9I0_MOUSE]	0.57	0.73	0.59	0.75	0.00
Q9Z2I0	LETM1 and EF-hand domain-containing protein 1, mitochondrial OS=Mus musculus GN=Letm1 PE=2 SV=1 - [LETM1_MOUSE]	-0.65	-0.58	-0.52	-0.48	0.00
P26040	Ezrin OS=Mus musculus GN=Ezr PE=1 SV=3 - [EZRI_MOUSE]	-0.50	-0.55	-0.39	-0.46	0.00
Q8K297	Procollagen galactosyltransferase 1 OS=Mus musculus GN=Colgalt1 PE=1 SV=2 - [GT251_MOUSE]	0.31	0.40	0.30	0.37	0.00
P84084	ADP-ribosylation factor 5 OS=Mus musculus GN=Arf5 PE=2 SV=2 - [ARF5_MOUSE]	-0.61	-0.46	-0.48	-0.47	0.00
Q69ZQ2	Pre-mRNA-splicing factor ISY1 homolog OS=Mus musculus GN=Isy1 PE=1 SV=2 - [ISY1_MOUSE]	0.38	0.46	0.40	0.52	0.00
P58404-2	Isoform 2 of Striatin-4 OS=Mus musculus GN=Strn4 - [STRN4_MOUSE]	-0.59	-0.61	-0.46	-0.49	0.00
P07310	Creatine kinase M-type OS=Mus musculus GN=Ckm PE=1 SV=1 - [KCRM_MOUSE]	0.46	0.43	0.56	0.56	0.00
J3QQ16	Protein Col6a3 OS=Mus musculus GN=Col6a3 PE=4 SV=1 - [J3QQ16_MOUSE]	-0.49	-0.36	-0.40	-0.42	0.00
Q64464	Cytochrome P450 3A13 OS=Mus musculus GN=Cyp3a13 PE=2 SV=1 - [CP3AD_MOUSE]	-1.05	-1.04	-0.79	-0.86	0.00
Q8BL99-3	Isoform 3 of Protein dopey-1 OS=Mus musculus GN=Dopey1 - [DOP1_MOUSE]	1.06	1.35	1.12	1.40	0.00
Q8VDJ3	Vigilin OS=Mus musculus GN=Hdlbp PE=1 SV=1 - [VIGLN_MOUSE]	0.25	0.20	0.28	0.26	0.00
Q8K3J1	NADH dehydrogenase [ubiquinone] iron-sulfur protein 8, mitochondrial OS=Mus musculus GN=Ndufs8 PE=1 SV=1 - [NDUS8_MOUSE]	-0.70	-0.79	-0.56	-0.67	0.00
Q8VCL2	Protein SCO2 homolog, mitochondrial OS=Mus musculus GN=Sco2 PE=2 SV=1 - [SCO2_MOUSE]	-0.50	-0.63	-0.53	-0.67	0.00
G3X8T9	Serine (Or cysteine) peptidase inhibitor, clade A, member 3N, isoform CRA_a OS=Mus musculus GN=Serpina3n PE=3 SV=1 - [G3X8T9_MOUSE]	1.26	1.14	1.56	1.44	0.00
Q9CQY1	Ubiquitin-like protein ATG12 OS=Mus musculus GN=Atg12 PE=1 SV=1 - [ATG12_MOUSE]	-0.35	-0.36	-0.28	-0.28	0.00
O55128	Histone deacetylase complex subunit SAP18 OS=Mus musculus GN=Sap18 PE=1 SV=1 - [SAP18_MOUSE]	0.38	0.31	0.28	0.31	0.00
Q9Z2I8-2	Isoform 2 of Succinyl-CoA ligase [GDP-forming] subunit beta, mitochondrial OS=Mus musculus GN=Suclg2 - [SUCB2_MOUSE]	-0.42	-0.44	-0.35	-0.33	0.00

Q99KC8	von Willebrand factor A domain-containing protein 5A OS=Mus musculus GN=Vwa5a PE=1 SV=2 - [VMA5A_MOUSE]	0.29	0.34	0.26	0.35	0.00
F8WIV2	Serpin B6 OS=Mus musculus GN=Serpinb6a PE=2 SV=1 - [F8WIV2_MOUSE]	-0.53	-0.60	-0.43	-0.50	0.00
M0QW65	Asparagine synthetase domain-containing protein 1 OS=Mus musculus GN=Asnsd1 PE=4 SV=1 - [M0QW65_MOUSE]	0.71	0.80	0.57	0.66	0.00
Q9WVH9	Fibulin-5 OS=Mus musculus GN=Fbln5 PE=2 SV=1 - [FBLN5_MOUSE]	-0.34	-0.33	-0.27	-0.26	0.00
Q8R1N0	Zinc finger protein 830 OS=Mus musculus GN=Znf830 PE=1 SV=1 - [ZN830_MOUSE]	1.91	1.61	2.26	1.96	0.00
Q9CRD0	OCIA domain-containing protein 1 OS=Mus musculus GN=Ociad1 PE=1 SV=1 - [OCAD1_MOUSE]	0.39	0.36	0.49	0.45	0.00
Q9QXY6	EH domain-containing protein 3 OS=Mus musculus GN=Ehd3 PE=1 SV=2 - [EHD3_MOUSE]	0.37	0.46	0.42	0.52	0.00
Q9D853	Methyltransferase-like protein 10 OS=Mus musculus GN=Mettl10 PE=2 SV=1 - [MET10_MOUSE]	-0.81	-0.69	-0.97	-0.85	0.00
Q91WA6-2	Isoform 2 of Sharpin OS=Mus musculus GN=Sharpin - [SHRPN_MOUSE]	0.21	0.22	0.27	0.27	0.00
Q3UQ28	Peroxidasin homolog OS=Mus musculus GN=Pxdn PE=2 SV=2 - [PXDN_MOUSE]	-0.30	-0.23	-0.32	-0.29	0.00
O08529	Calpain-2 catalytic subunit OS=Mus musculus GN=Capn2 PE=2 SV=4 - [CAN2_MOUSE]	-0.29	-0.25	-0.35	-0.29	0.00
O70252	Heme oxygenase 2 OS=Mus musculus GN=Hmox2 PE=2 SV=1 - [HMOX2_MOUSE]	-0.36	-0.37	-0.27	-0.34	0.00
E9Q4K7	Protein Kif13b OS=Mus musculus GN=Kif13b PE=2 SV=1 - [E9Q4K7_MOUSE]	-0.21	-0.26	-0.28	-0.28	0.00
Q80WK2	Organic solute transporter subunit beta OS=Mus musculus GN=Slc51b PE=1 SV=1 - [OSTB_MOUSE]	0.40	0.37	0.33	0.29	0.00
Q569Z6	Thyroid hormone receptor-associated protein 3 OS=Mus musculus GN=Thrap3 PE=1 SV=1 - [TR150_MOUSE]	1.29	1.07	1.52	1.33	0.00
Q5FWK3	Rho GTPase-activating protein 1 OS=Mus musculus GN=Arhgap1 PE=1 SV=1 - [RHG01_MOUSE]	-0.41	-0.44	-0.31	-0.39	0.00
Q9Z109	V-set and immunoglobulin domain-containing protein 2 OS=Mus musculus GN=Vsig2 PE=2 SV=2 - [VSIG2_MOUSE]	0.86	0.69	0.95	0.78	0.00
Q9WU81-2	Isoform 2 of Sugar phosphate exchanger 2 OS=Mus musculus GN=Slc37a2 - [SPX2_MOUSE]	-1.07	-1.35	-1.03	-1.32	0.00

Q8BXK9	Chloride intracellular channel protein 5 OS=Mus musculus GN=Clic5 PE=1 SV=1 - [CLIC5_MOUSE]	-0.98	-1.14	-1.20	-1.37	0.00
Q08024	Core-binding factor subunit beta OS=Mus musculus GN=Cbfb PE=1 SV=1 - [PEBB_MOUSE]	0.27	0.29	0.36	0.35	0.00
P07759	Serine protease inhibitor A3K OS=Mus musculus GN=Serpina3k PE=1 SV=2 - [SPA3K_MOUSE]	-0.86	-0.95	-0.71	-0.72	0.00
Q8R1Q9	Ribokinase OS=Mus musculus GN=Rbks PE=1 SV=1 - [RBSK_MOUSE]	-0.33	-0.24	-0.29	-0.26	0.00
Q9WTL2	Ras-related protein Rab-25 OS=Mus musculus GN=Rab25 PE=1 SV=2 - [RAB25_MOUSE]	-0.38	-0.29	-0.36	-0.41	0.00
Q99JY9	Actin-related protein 3 OS=Mus musculus GN=Actr3 PE=1 SV=3 - [ARP3_MOUSE]	-0.20	-0.22	-0.18	-0.16	0.00
Q3UL36	Arginine and glutamate-rich protein 1 OS=Mus musculus GN=Arglu1 PE=1 SV=2 - [ARGL1_MOUSE]	0.77	0.57	0.79	0.73	0.00
P63001	Ras-related C3 botulinum toxin substrate 1 OS=Mus musculus GN=Rac1 PE=1 SV=1 - [RAC1_MOUSE]	-0.23	-0.21	-0.22	-0.28	0.00
P21981	Protein-glutamine gamma-glutamyltransferase 2 OS=Mus musculus GN=Tgm2 PE=1 SV=4 - [TGM2_MOUSE]	0.27	0.34	0.25	0.27	0.00
B1ATS4	Sarcoplasmic/endoplasmic reticulum calcium ATPase 3 OS=Mus musculus GN=Atp2a3 PE=2 SV=1 - [B1ATS4_MOUSE]	-0.63	-0.60	-0.77	-0.80	0.00
P70288	Histone deacetylase 2 OS=Mus musculus GN=Hdac2 PE=1 SV=1 - [HDAC2_MOUSE]	0.54	0.45	0.64	0.59	0.00
P60766	Cell division control protein 42 homolog OS=Mus musculus GN=Cdc42 PE=1 SV=2 - [CDC42_MOUSE]	-0.19	-0.24	-0.18	-0.20	0.00
Q64374	Regucalcin OS=Mus musculus GN=Rgn PE=1 SV=1 - [RGN_MOUSE]	-0.77	-0.62	-0.88	-0.73	0.00
O88441	Metaxin-2 OS=Mus musculus GN=Mtx2 PE=1 SV=1 - [MTX2_MOUSE]	-0.51	-0.42	-0.58	-0.45	0.00
Q91WT8-2	Isoform 2 of RNA-binding protein 47 OS=Mus musculus GN=Rbm47 - [RBM47_MOUSE]	0.33	0.26	0.34	0.37	0.00
Q7TQ62	Podocan OS=Mus musculus GN=Podn PE=2 SV=1 - [PODN_MOUSE]	-1.33	-1.05	-1.27	-0.99	0.00
P60710	Actin, cytoplasmic 1 OS=Mus musculus GN=Actb PE=1 SV=1 - [ACTB_MOUSE]	-0.25	-0.23	-0.30	-0.31	0.00
Q9CX60	Protein LBH OS=Mus musculus GN=Lbh PE=1 SV=1 - [LBH_MOUSE]	0.43	0.50	0.57	0.43	0.00

Q8BH64	EH domain-containing protein 2 OS=Mus musculus GN=Ehd2 PE=1 SV=1 - [EHD2_MOUSE]	-0.38	-0.34	-0.45	-0.34	0.00
Q6A028	Switch-associated protein 70 OS=Mus musculus GN=Swap70 PE=1 SV=2 - [SWP70_MOUSE]	0.73	0.58	0.60	0.53	0.00
Q80TL7-2	Isoform 2 of Protein MON2 homolog OS=Mus musculus GN=Mon2 - [MON2_MOUSE]	-0.68	-0.55	-0.62	-0.49	0.00
Q91VX9	Transmembrane protein 168 OS=Mus musculus GN=Tmem168 PE=2 SV=1 - [TM168_MOUSE]	-0.87	-0.70	-1.00	-0.86	0.00
Q8R3B1	1-phosphatidylinositol 4,5-bisphosphate phosphodiesterase delta-1 OS=Mus musculus GN=Plcd1 PE=2 SV=2 - [PLCD1_MOUSE]	-0.27	-0.32	-0.34	-0.39	0.00
G3UYV7	40S ribosomal protein S28 (Fragment) OS=Mus musculus GN=Rps28 PE=2 SV=1 - [G3UYV7_MOUSE]	0.25	0.22	0.17	0.21	0.00
Q91VA1	Choline transporter-like protein 4 OS=Mus musculus GN=Slc44a4 PE=1 SV=1 - [CTL4_MOUSE]	-0.45	-0.38	-0.40	-0.32	0.00
Q9QZQ8	Core histone macro-H2A.1 OS=Mus musculus GN=H2afy PE=1 SV=3 - [H2AY_MOUSE]	-0.28	-0.21	-0.24	-0.29	0.00
Q6P9S7	Polypeptide N-acetylgalactosaminyltransferase 10 OS=Mus musculus GN=Galnt10 PE=2 SV=1 - [GLT10_MOUSE]	-0.24	-0.29	-0.21	-0.28	0.00
Q99JW5	Epithelial cell adhesion molecule OS=Mus musculus GN=Epcam PE=2 SV=1 - [EPCAM_MOUSE]	-0.62	-0.69	-0.50	-0.54	0.00
Q6PF93	Phosphatidylinositol 3-kinase catalytic subunit type 3 OS=Mus musculus GN=Pik3c3 PE=1 SV=1 - [PK3C3_MOUSE]	-0.32	-0.40	-0.36	-0.44	0.00
Q9D7S9	Charged multivesicular body protein 5 OS=Mus musculus GN=Chmp5 PE=2 SV=1 - [CHMP5_MOUSE]	0.45	0.42	0.58	0.49	0.00
P53986	Monocarboxylate transporter 1 OS=Mus musculus GN=Slc16a1 PE=1 SV=1 - [MOT1_MOUSE]	-0.79	-0.81	-0.76	-0.58	0.00
Q9CZW5	Mitochondrial import receptor subunit TOM70 OS=Mus musculus GN=Tomm70a PE=1 SV=2 - [TOM70_MOUSE]	-0.39	-0.54	-0.44	-0.47	0.00
D3YVJ8	Retinol dehydrogenase 13 (Fragment) OS=Mus musculus GN=Rdh13 PE=2 SV=1 - [D3YVJ8_MOUSE]	-0.82	-0.93	-0.66	-0.75	0.00
Q6URW6	Myosin-14 OS=Mus musculus GN=Myh14 PE=1 SV=1 - [MYH14_MOUSE]	0.15	0.18	0.17	0.21	0.00
Q6PFD9	Nuclear pore complex protein Nup98-Nup96 OS=Mus musculus GN=Nup98 PE=1 SV=2 - [NUP98_MOUSE]	0.62	0.52	0.72	0.56	0.00
E9QNL5	Sulfotransferase 1A1 OS=Mus musculus GN=Sult1a1 PE=2 SV=1 - [E9QNL5_MOUSE]	-0.35	-0.43	-0.46	-0.50	0.00

P14206	40S ribosomal protein SA OS=Mus musculus GN=Rpsa PE=1 SV=4 - [RSSA_MOUSE]	0.32	0.40	0.31	0.41	0.00
Q8C181-3	Isoform 3 of Muscleblind-like protein 2 OS=Mus musculus GN=Mbnl2 - [MBNL2_MOUSE]	0.58	0.64	0.45	0.60	0.00
F8VPM0	Bromodomain adjacent to zinc finger domain protein 2A OS=Mus musculus GN=Baz2a PE=2 SV=1 - [F8VPM0_MOUSE]	0.83	0.66	0.78	0.61	0.00
O70310	Glycylpeptide N-tetradecanoyltransferase 1 OS=Mus musculus GN=Nmt1 PE=1 SV=1 - [NMT1_MOUSE]	0.13	0.16	0.17	0.18	0.00
Q01339	Beta-2-glycoprotein 1 OS=Mus musculus GN=Apoh PE=1 SV=1 - [APOH_MOUSE]	0.81	0.71	1.00	0.90	0.00
P15947	Kallikrein-1 OS=Mus musculus GN=Klk1 PE=1 SV=3 - [KLK1_MOUSE]	-1.08	-0.86	-1.05	-0.81	0.00
D0QMC3	Myeloid cell nuclear differentiation antigen-like protein OS=Mus musculus GN=Mndal PE=2 SV=1 - [MNDAL_MOUSE]	0.29	0.25	0.23	0.21	0.00
Q9CPU2	NADH dehydrogenase [ubiquinone] 1 beta subcomplex subunit 2, mitochondrial OS=Mus musculus GN=Ndufb2 PE=3 SV=1 - [NDUB2_MOUSE]	-0.24	-0.31	-0.24	-0.31	0.00
Q05BQ1	Protein turtle homolog A OS=Mus musculus GN=Igsf9 PE=1 SV=2 - [TUTLA_MOUSE]	-0.38	-0.43	-0.30	-0.36	0.00
P97369	Neutrophil cytosol factor 4 OS=Mus musculus GN=Ncf4 PE=2 SV=2 - [NCF4_MOUSE]	0.87	0.89	0.65	0.73	0.00
P70677	Caspase-3 OS=Mus musculus GN=Casp3 PE=1 SV=1 [CASP3_MOUSE]	0.52	0.65	0.66	0.75	0.00
Q61753	D-3-phosphoglycerate dehydrogenase OS=Mus musculus GN=Phgdh PE=1 SV=3 - [SERA_MOUSE]	0.14	0.16	0.19	0.20	0.00
Q8BJF9	Charged multivesicular body protein 2b OS=Mus musculus GN=Chmp2b PE=2 SV=1 - [CHM2B_MOUSE]	0.75	0.97	1.08	0.95	0.00
Q64442	Sorbitol dehydrogenase OS=Mus musculus GN=Sord PE=1 SV=3 - [DHSO_MOUSE]	-0.38	-0.29	-0.27	-0.31	0.00
P61255	60S ribosomal protein L26 OS=Mus musculus GN=Rpl26 PE=2 SV=1 - [RL26_MOUSE]	-0.29	-0.30	-0.22	-0.26	0.00
Q64685	Beta-galactoside alpha-2,6-sialyltransferase 1 OS=Mus musculus GN=St6gal1 PE=1 SV=2 - [SIAT1_MOUSE]	-0.90	-0.80	-1.12	-1.02	0.00
P35293	Ras-related protein Rab-18 OS=Mus musculus GN=Rab18 PE=2 SV=2 - [RAB18_MOUSE]	-0.32	-0.28	-0.28	-0.22	0.00
P50446	Keratin, type II cytoskeletal 6A OS=Mus musculus GN=Krt6a PE=2 SV=3 - [K2C6A_MOUSE]	-0.62	-0.75	-0.53	-0.67	0.00

P18528	Ig heavy chain V region 6.96 OS=Mus musculus PE=4 SV=1 - [HVM57_MOUSE]	-0.84	-0.69	-0.74	-0.59	0.00
Q7TT16	Inositol polyphosphate multikinase OS=Mus musculus GN=Ipmk PE=2 SV=1 - [IPMK_MOUSE]	0.86	0.67	0.87	0.68	0.00
Q9Z0J0	Epididymal secretory protein E1 OS=Mus musculus GN=Npc2 PE=2 SV=1 - [NPC2_MOUSE]	-0.63	-0.69	-0.50	-0.55	0.00
P52480-2	Isoform M1 of Pyruvate kinase PKM OS=Mus musculus GN=Pkm - [KPYM_MOUSE]	-0.16	-0.12	-0.17	-0.14	0.00
Q8C2Q3	RNA-binding protein 14 OS=Mus musculus GN=Rbm14 PE=1 SV=1 - [RBM14_MOUSE]	0.47	0.63	0.49	0.61	0.00
Q9EQJ0	Two pore calcium channel protein 1 OS=Mus musculus GN=Tpcn1 PE=2 SV=1 - [TPC1_MOUSE]	-0.83	-0.67	-0.96	-0.81	0.00
Q60590	Alpha-1-acid glycoprotein 1 OS=Mus musculus GN=Orm1 PE=1 SV=1 - [A1AG1_MOUSE]	1.29	1.16	1.62	1.49	0.00
Q62523	Zyxin OS=Mus musculus GN=Zyx PE=1 SV=2 - [ZYX_MOUSE]	0.39	0.40	0.44	0.53	0.00
F6TYB7	Myelin basic protein (Fragment) OS=Mus musculus GN=Mbp PE=4 SV=1 - [F6TYB7_MOUSE]	1.25	1.10	1.04	0.88	0.00
Q8K0C4	Lanosterol 14-alpha demethylase OS=Mus musculus GN=Cyp51a1 PE=2 SV=1 - [CP51A_MOUSE]	-0.42	-0.43	-0.56	-0.54	0.00
Q8BGE6	Cysteine protease ATG4B OS=Mus musculus GN=Atg4b PE=1 SV=2 - [ATG4B_MOUSE]	-0.54	-0.60	-0.49	-0.43	0.00
P01897	H-2 class I histocompatibility antigen, L-D alpha chain OS=Mus musculus GN=H2-L PE=1 SV=2 - [HA1L_MOUSE]	0.65	0.78	0.82	0.93	0.00
P03911	NADH-ubiquinone oxidoreductase chain 4 OS=Mus musculus GN=Mtnd4 PE=1 SV=1 - [NU4M_MOUSE]	-1.82	-1.44	-2.05	-1.67	0.00
B0V2N1-2	Isoform 2 of Receptor-type tyrosine-protein phosphatase S OS=Mus musculus GN=Ptprs - [PTPRS_MOUSE]	-0.08	-0.09	-0.09	-0.11	0.00
H3BLI9	TSC22 domain family protein 1 OS=Mus musculus GN=Tsc22d1 PE=4 SV=1 - [H3BLI9_MOUSE]	0.70	0.61	0.81	0.59	0.00
A2A6Q8	Myosin light chain 4 (Fragment) OS=Mus musculus GN=Myl4 PE=2 SV=1 - [A2A6Q8_MOUSE]	0.52	0.67	0.74	0.61	0.00
Q99LD9	Translation initiation factor eIF-2B subunit beta OS=Mus musculus GN=Eif2b2 PE=2 SV=1 - [EI2BB_MOUSE]	-0.62	-0.46	-0.53	-0.46	0.00
P46471	26S protease regulatory subunit 7 OS=Mus musculus GN=Psmc2 PE=1 SV=5 - [PRS7_MOUSE]	-0.33	-0.36	-0.25	-0.31	0.00

Q5BKQ4	Inactive pancreatic lipase-related protein 1 OS=Mus musculus GN=Pnliprp1 PE=2 SV=2 - [LIPR1_MOUSE]	0.51	0.66	0.56	0.70	0.00
A2AI08	Taperin OS=Mus musculus GN=Tprn PE=1 SV=1 - [TPRN_MOUSE]	0.83	0.68	0.75	0.59	0.00
Q7TQF7	Amphiphysin OS=Mus musculus GN=Amph PE=1 SV=1 - [AMPH_MOUSE]	0.60	0.69	0.48	0.57	0.00
Q8CHC4	Synaptojanin-1 OS=Mus musculus GN=Synj1 PE=1 SV=3 - [SYNJ1_MOUSE]	0.36	0.39	0.49	0.47	0.00
P03953-2	Isoform 2 of Complement factor D OS=Mus musculus GN=Cfd - [CFAD_MOUSE]	0.90	0.75	1.04	1.03	0.00
O54839-2	Isoform 2 of Eomesodermin homolog OS=Mus musculus GN=Eomes - [EOMES_MOUSE]	1.46	1.19	1.71	1.44	0.00
Q8JZR4	Excitatory amino acid transporter 5 OS=Mus musculus GN=Slc1a7 PE=2 SV=1 - [EAA5_MOUSE]	-0.81	-0.89	-0.63	-0.71	0.00
P10493	Nidogen-1 OS=Mus musculus GN=Nid1 PE=1 SV=2 - [NID1_MOUSE]	0.28	0.22	0.24	0.20	0.00
P30204-2	Isoform II of Macrophage scavenger receptor types I and II OS=Mus musculus GN=Msr1 - [MSRE_MOUSE]	0.42	0.50	0.35	0.43	0.00
O35295	Transcriptional activator protein Pur-beta OS=Mus musculus GN=Purb PE=1 SV=3 - [PURB_MOUSE]	-0.28	-0.30	-0.23	-0.22	0.00
Q91WQ9	Calmodulin-like protein 4 OS=Mus musculus GN=Calml4 PE=2 SV=2 - [CALL4_MOUSE]	0.37	0.48	0.45	0.53	0.00
A2AI93	DMRT-like family C1b OS=Mus musculus GN=Dmrtc1b PE=2 SV=1 - [A2AI93_MOUSE]	-0.28	-0.21	-0.26	-0.21	0.00
P70671	Interferon regulatory factor 3 OS=Mus musculus GN=Irf3 PE=1 SV=1 - [IRF3_MOUSE]	-0.31	-0.24	-0.30	-0.23	0.00
Q8VCI0	Phospholipase B-like 1 OS=Mus musculus GN=Plbd1 PE=1 SV=1 - [PLBL1_MOUSE]	-0.88	-0.70	-0.70	-0.62	0.00
E9Q4G7	Casein kinase I isoform alpha OS=Mus musculus GN=Csnk1a1 PE=2 SV=1 - [E9Q4G7_MOUSE]	0.21	0.18	0.22	0.16	0.00
O70172	Phosphatidylinositol 5-phosphate 4-kinase type-2 alpha OS=Mus musculus GN=Pip4k2a PE=1 SV=1 - [PI42A_MOUSE]	0.31	0.43	0.35	0.41	0.00
Q8BZF8	Phosphoglucomutase-like protein 5 OS=Mus musculus GN=Pgm5 PE=1 SV=2 - [PGM5_MOUSE]	-0.39	-0.30	-0.42	-0.33	0.00
O88572	Low-density lipoprotein receptor-related protein 6 OS=Mus musculus GN=Lrp6 PE=1 SV=1 - [LRP6_MOUSE]	0.33	0.35	0.25	0.27	0.00

P52927	High mobility group protein HMGI-C OS=Mus musculus GN=Hmga2 PE=1 SV=1 - [HMGA2_MOUSE]	0.66	0.80	0.82	0.95	0.00
Q9QZ23	NFU1 iron-sulfur cluster scaffold homolog, mitochondrial OS=Mus musculus GN=Nfu1 PE=1 SV=2 - [NFU1_MOUSE]	0.27	0.26	0.36	0.32	0.00
Q9ER72	Cysteine--tRNA ligase, cytoplasmic OS=Mus musculus GN=Cars PE=1 SV=2 - [SYCC_MOUSE]	0.07	0.08	0.10	0.09	0.00
Q9DC70	NADH dehydrogenase [ubiquinone] iron-sulfur protein 7, mitochondrial OS=Mus musculus GN=Ndufs7 PE=1 SV=1 - [NDUS7_MOUSE]	-0.86	-0.93	-0.66	-0.76	0.00
Q9Z2L6	Multiple inositol polyphosphate phosphatase 1 OS=Mus musculus GN=Minpp1 PE=1 SV=3 - [MINP1_MOUSE]	-0.59	-0.45	-0.43	-0.54	0.00
F6SMJ7	GTP-binding protein 8 (Fragment) OS=Mus musculus GN=Gtpbp8 PE=4 SV=1 - [F6SMJ7_MOUSE]	0.22	0.25	0.26	0.18	0.00
G3X924	Plasmalemma vesicle associated protein OS=Mus musculus GN=Plvap PE=4 SV=1 - [G3X924_MOUSE]	-0.54	-0.76	-0.60	-0.71	0.00
Q8CG14	Complement C1s-A subcomponent OS=Mus musculus GN=C1sa PE=2 SV=2 - [CS1A_MOUSE]	0.86	0.91	1.13	1.16	0.00
O09044	Synaptosomal-associated protein 23 OS=Mus musculus GN=Snap23 PE=1 SV=1 - [SNP23_MOUSE]	0.35	0.30	0.37	0.27	0.00
Q5SUF2	Luc7-like protein 3 OS=Mus musculus GN=Luc7l3 PE=1 SV=1 - [LC7L3_MOUSE]	0.27	0.38	0.37	0.38	0.00
E9Q6H8	Protein Plekha5 OS=Mus musculus GN=Plekha5 PE=2 SV=1 - [E9Q6H8_MOUSE]	0.36	0.30	0.43	0.39	0.00
E9QPX3	NADH dehydrogenase [ubiquinone] iron-sulfur protein 4, mitochondrial OS=Mus musculus GN=Ndufs4 PE=4 SV=1 - [E9QPX3_MOUSE]	-0.57	-0.79	-0.62	-0.72	0.00
O35435	Dihydroorotate dehydrogenase (quinone), mitochondrial OS=Mus musculus GN=Dhodh PE=2 SV=2 - [PYRD_MOUSE]	-0.26	-0.33	-0.24	-0.26	0.00
P62242	40S ribosomal protein S8 OS=Mus musculus GN=Rps8 PE=1 SV=2 - [RS8_MOUSE]	-0.33	-0.38	-0.28	-0.28	0.00
F7AFM3	Threonine synthase-like 2 (Fragment) OS=Mus musculus GN=Thnsl2 PE=4 SV=1 - [F7AFM3_MOUSE]	-0.43	-0.45	-0.56	-0.58	0.00
Q9WVJ3-2	Isoform 2 of Carboxypeptidase Q OS=Mus musculus GN=Cpq - [CBPQ_MOUSE]	-0.66	-0.47	-0.65	-0.64	0.00
P54751	CMP-N-acetylneuraminate-beta-galactosamide-alpha-2,3-sialyltransferase 1 OS=Mus musculus GN=St3gal1 PE=2 SV=1 - [SIA4A_MOUSE]	0.68	0.81	0.56	0.69	0.00
P80313	T-complex protein 1 subunit eta OS=Mus musculus GN=Cct7 PE=1 SV=1 - [TCPH_MOUSE]	-0.25	-0.22	-0.22	-0.17	0.00

Q8VD69	Arrestin domain containing 4 OS=Mus musculus GN=Arrdc4 PE=2 SV=1 - [Q8VD69_MOUSE]	0.24	0.24	0.18	0.18	0.00
Q922D8	C-1-tetrahydrofolate synthase, cytoplasmic OS=Mus musculus GN=Mthfd1 PE=1 SV=4 - [C1TC_MOUSE]	-0.45	-0.45	-0.34	-0.35	0.00
Q99JR1	Sideroflexin-1 OS=Mus musculus GN=Sfxn1 PE=1 SV=3 - [SFXN1_MOUSE]	-0.48	-0.42	-0.56	-0.60	0.00
P29758	Ornithine aminotransferase, mitochondrial OS=Mus musculus GN=Oat PE=1 SV=1 - [OAT_MOUSE]	0.41	0.37	0.50	0.50	0.00
Q8K2J1	Car12 protein OS=Mus musculus GN=Car12 PE=2 SV=1 - [Q8K2J1_MOUSE]	1.09	0.84	1.12	0.87	0.00
Q3U4F0	Sideroflexin-3 OS=Mus musculus GN=Sfxn3 PE=2 SV=1 - [Q3U4F0_MOUSE]	-0.73	-0.53	-0.66	-0.56	0.00
Q9D5Y1	Coiled-coil domain-containing protein 39 OS=Mus musculus GN=Ccdc39 PE=2 SV=1 - [CCD39_MOUSE]	0.48	0.63	0.50	0.64	0.00
G3UXL2	Ribose-phosphate pyrophosphokinase OS=Mus musculus GN=Prps1l3 PE=2 SV=1 - [G3UXL2_MOUSE]	-0.44	-0.44	-0.40	-0.31	0.00
E9PZ36	Protein Pkhd1 OS=Mus musculus GN=Pkhd1 PE=4 SV=1 - [E9PZ36_MOUSE]	-0.24	-0.19	-0.26	-0.21	0.00
Q7TSJ2	Microtubule-associated protein 6 OS=Mus musculus GN=Map6 PE=1 SV=2 - [MAP6_MOUSE]	0.34	0.30	0.42	0.32	0.00
E9PV66	Protein Myo18b OS=Mus musculus GN=Myo18b PE=4 SV=1 - [E9PV66_MOUSE]	1.85	1.58	2.28	2.01	0.00
A2AFS3	UPF0577 protein KIAA1324 OS=Mus musculus GN=Kiaa1324 PE=2 SV=1 - [K1324_MOUSE]	0.34	0.42	0.31	0.43	0.00
Q61699-2	Isoform HSP105-beta of Heat shock protein 105 kDa OS=Mus musculus GN=Hsph1 - [HS105_MOUSE]	0.34	0.29	0.38	0.27	0.00
P56480	ATP synthase subunit beta, mitochondrial OS=Mus musculus GN=Atp5b PE=1 SV=2 - [ATPB_MOUSE]	-0.07	-0.09	-0.08	-0.09	0.00
Q9R0H0	Peroxisomal acyl-coenzyme A oxidase 1 OS=Mus musculus GN=Acox1 PE=1 SV=5 - [ACOX1_MOUSE]	0.35	0.44	0.46	0.51	0.00
Q9D706	RNA polymerase II-associated protein 3 OS=Mus musculus GN=Rpap3 PE=1 SV=1 - [RPAP3_MOUSE]	0.33	0.47	0.42	0.45	0.00
P21447	Multidrug resistance protein 1A OS=Mus musculus GN=Abcb1a PE=1 SV=3 - [MDR1A_MOUSE]	-0.64	-0.48	-0.56	-0.46	0.00
Q91WP6	Serine protease inhibitor A3N OS=Mus musculus GN=Serpina3n PE=1 SV=1 - [SPA3N_MOUSE]	-1.99	-1.72	-1.64	-1.37	0.00

D3Z3A9	Reticulocalbin-3 (Fragment) OS=Mus musculus GN=Rcn3 PE=2 SV=1 - [D3Z3A9_MOUSE]	0.38	0.37	0.52	0.43	0.00
D3YU12	NmrA-like family domain-containing protein 1 OS=Mus musculus GN=Nmral1 PE=2 SV=1 - [D3YU12_MOUSE]	-0.62	-0.54	-0.45	-0.47	0.00
Q8BI84	Melanoma inhibitory activity protein 3 OS=Mus musculus GN=Mia3 PE=1 SV=2 - [MIA3_MOUSE]	0.35	0.32	0.45	0.38	0.00
Q5SWZ5	Myosin phosphatase Rho-interacting protein OS=Mus musculus GN=Mprip PE=2 SV=1 - [Q5SWZ5_MOUSE]	0.35	0.43	0.30	0.37	0.00
Q91YS7	Dual-specificity mitogen-activated protein kinase kinase 2 OS=Mus musculus GN=Map2k2 PE=2 SV=1 - [Q91YS7_MOUSE]	-0.90	-0.66	-0.93	-0.78	0.00
P35279	Ras-related protein Rab-6A OS=Mus musculus GN=Rab6a PE=1 SV=4 - [RAB6A_MOUSE]	-0.37	-0.26	-0.30	-0.28	0.00
Q5U430	E3 ubiquitin-protein ligase UBR3 OS=Mus musculus GN=Ubr3 PE=1 SV=3 - [UBR3_MOUSE]	-0.27	-0.22	-0.23	-0.18	0.00
Q3UG93	Heat shock factor protein 4 OS=Mus musculus GN=Hsf4 PE=2 SV=1 - [Q3UG93_MOUSE]	-1.12	-0.99	-0.90	-0.77	0.00
Q80U87	Ubiquitin carboxyl-terminal hydrolase 8 OS=Mus musculus GN=Usp8 PE=1 SV=2 - [UBP8_MOUSE]	0.52	0.49	0.48	0.66	0.00
A2ADY9	Protein DDI1 homolog 2 OS=Mus musculus GN=Ddi2 PE=1 SV=1 - [DDI2_MOUSE]	0.72	0.70	0.55	0.54	0.00
P21278	Guanine nucleotide-binding protein subunit alpha-11 OS=Mus musculus GN=Gna11 PE=1 SV=1 - [GNA11_MOUSE]	-0.40	-0.33	-0.48	-0.45	0.00
B9EJ54	MCG21756, isoform CRA_b OS=Mus musculus GN=Nup205 PE=2 SV=1 - [B9EJ54_MOUSE]	-0.50	-0.41	-0.41	-0.35	0.00
P52624	Uridine phosphorylase 1 OS=Mus musculus GN=Upp1 PE=1 SV=2 - [UPP1_MOUSE]	-1.43	-1.19	-1.64	-1.22	0.00
P17563	Selenium-binding protein 1 OS=Mus musculus GN=Selenbp1 PE=1 SV=2 - [SBP1_MOUSE]	-0.36	-0.42	-0.29	-0.34	0.00
Q80X50	Ubiquitin-associated protein 2-like OS=Mus musculus GN=Ubap2l PE=1 SV=1 - [UBP2L_MOUSE]	0.77	0.64	0.92	0.73	0.00
P41317	Mannose-binding protein C OS=Mus musculus GN=Mbl2 PE=2 SV=2 - [MBL2_MOUSE]	1.41	1.19	1.73	1.50	0.00
A2AD97	Matrix-remodeling-associated protein 8 (Fragment) OS=Mus musculus GN=Mxra8 PE=2 SV=1 - [A2AD97_MOUSE]	-0.38	-0.43	-0.31	-0.32	0.00
D3YXK2	Scaffold attachment factor B1 OS=Mus musculus GN=Safb PE=1 SV=2 - [SAFB1_MOUSE]	0.41	0.49	0.59	0.54	0.00

Q9Z2U0	Proteasome subunit alpha type-7 OS=Mus musculus GN=Psma7 PE=1 SV=1 - [PSA7_MOUSE]	0.15	0.17	0.11	0.14	0.00
F6VY64	U3 small nucleolar ribonucleoprotein protein IMP4 (Fragment) OS=Mus musculus GN=Imp4 PE=2 SV=1 - [F6VY64_MOUSE]	-0.87	-0.69	-0.79	-0.61	0.00
Q80VA0	N-acetylgalactosaminyltransferase 7 OS=Mus musculus GN=Galnt7 PE=2 SV=2 - [GALT7_MOUSE]	-0.24	-0.30	-0.23	-0.21	0.00
P22892	AP-1 complex subunit gamma-1 OS=Mus musculus GN=Ap1g1 PE=1 SV=3 - [AP1G1_MOUSE]	-0.62	-0.45	-0.50	-0.45	0.00
Q9Z2H7	PDZ domain-containing protein GIPC2 OS=Mus musculus GN=Gipc2 PE=1 SV=1 - [GIPC2_MOUSE]	0.29	0.33	0.40	0.41	0.00
Q3UKC1	Tax1-binding protein 1 homolog OS=Mus musculus GN=Tax1bp1 PE=1 SV=2 - [TAXB1_MOUSE]	0.54	0.55	0.74	0.63	0.00
Q5RIM6	Nuclear receptor co-repressor 1, isoform CRA_a OS=Mus musculus GN=Ncor1 PE=4 SV=1 - [Q5RIM6_MOUSE]	1.16	0.85	1.04	0.85	0.00
Q9JJF3	Bifunctional lysine-specific demethylase and histidyl-hydroxylase NO66 OS=Mus musculus GN=No66 PE=1 SV=2 - [NO66_MOUSE]	0.25	0.30	0.32	0.37	0.00
E9QKA4	Serine/arginine repetitive matrix protein 1 OS=Mus musculus GN=Srrm1 PE=2 SV=1 - [E9QKA4_MOUSE]	0.72	0.68	0.81	0.96	0.00
Q3TIU4	2',5'-phosphodiesterase 12 OS=Mus musculus GN=Pde12 PE=2 SV=2 - [PDE12_MOUSE]	0.24	0.25	0.26	0.18	0.00
A2ARJ3	Transmembrane protein 236 OS=Mus musculus GN=Tmem236 PE=3 SV=1 - [TM236_MOUSE]	-1.30	-1.18	-1.56	-1.11	0.00
D3Z5I9	RNA-binding protein 33 OS=Mus musculus GN=Rbm33 PE=2 SV=1 - [D3Z5I9_MOUSE]	0.72	0.95	0.71	0.93	0.00
Q91VA7	Isocitrate dehydrogenase 3 (NAD+) beta OS=Mus musculus GN=Idh3b PE=2 SV=1 - [Q91VA7_MOUSE]	-0.70	-0.79	-0.54	-0.65	0.00
Q07076	Annexin A7 OS=Mus musculus GN=Anxa7 PE=2 SV=2 - [ANXA7_MOUSE]	-0.21	-0.17	-0.17	-0.23	0.00
Q63739	Protein tyrosine phosphatase type IVA 1 OS=Mus musculus GN=Ptp4a1 PE=1 SV=1 - [TP4A1_MOUSE]	-0.46	-0.56	-0.56	-0.68	0.00
P70665-3	Isoform 3 of Sialate O-acetylesterase OS=Mus musculus GN=Siae - [SIAE_MOUSE]	-0.32	-0.38	-0.42	-0.46	0.00
D3YVX2	Coiled-coil domain-containing protein 7 (Fragment) OS=Mus musculus GN=Ccdc7 PE=2 SV=1 - [D3YVX2_MOUSE]	-0.55	-0.58	-0.48	-0.40	0.00
Q8BHJ9	Pre-mRNA-splicing factor SLU7 OS=Mus musculus GN=Slu7 PE=1 SV=1 - [SLU7_MOUSE]	0.27	0.30	0.36	0.39	0.00

Q9JI67	Beta-1,3-galactosyltransferase 5 OS=Mus musculus GN=B3galt5 PE=2 SV=1 - [B3GT5_MOUSE]	-1.03	-0.76	-1.05	-0.82	0.00
Q569Z5	Probable ATP-dependent RNA helicase DDX46 OS=Mus musculus GN=Ddx46 PE=1 SV=2 - [DDX46_MOUSE]	0.35	0.38	0.35	0.26	0.00
Q8K4P0	pre-mRNA 3' end processing protein WDR33 OS=Mus musculus GN=Wdr33 PE=2 SV=1 - [WDR33_MOUSE]	0.51	0.50	0.35	0.47	0.00
P16015	Carbonic anhydrase 3 OS=Mus musculus GN=Ca3 PE=1 SV=3 - [CAH3_MOUSE]	0.64	0.78	0.85	0.94	0.00
P28063	Proteasome subunit beta type-8 OS=Mus musculus GN=Psmb8 PE=1 SV=2 - [PSB8_MOUSE]	0.24	0.30	0.29	0.35	0.00
Q9DCT2	NADH dehydrogenase [ubiquinone] iron-sulfur protein 3, mitochondrial OS=Mus musculus GN=Ndufs3 PE=1 SV=2 - [NDUS3_MOUSE]	-0.61	-0.73	-0.50	-0.67	0.00
E9PUI0	Alpha-N-acetylgalactosaminide alpha-2,6-sialyltransferase 6 OS=Mus musculus GN=St6galnac6 PE=2 SV=1 - [E9PUI0_MOUSE]	-0.87	-0.69	-1.02	-0.84	0.00
Q64669	NAD(P)H dehydrogenase [quinone] 1 OS=Mus musculus GN=Nqo1 PE=1 SV=3 - [NQO1_MOUSE]	0.35	0.33	0.45	0.44	0.00
Q3TAI4	Tumor protein D52-like 2, isoform CRA_c OS=Mus musculus GN=Tpd52l2 PE=2 SV=1 - [Q3TAI4_MOUSE]	0.22	0.20	0.28	0.27	0.00
Q3UJB0	Protein Sf3b2 OS=Mus musculus GN=Sf3b2 PE=2 SV=1 - [Q3UJB0_MOUSE]	0.70	0.54	0.79	0.67	0.00
Q60967	Bifunctional 3'-phosphoadenosine 5'-phosphosulfate synthase 1 OS=Mus musculus GN=Papss1 PE=2 SV=1 - [PAPS1_MOUSE]	-0.29	-0.43	-0.37	-0.34	0.00
Q8BMD5-3	Isoform 3 of Cytidine and dCMP deaminase domain-containing protein 1 OS=Mus musculus GN=Cdadc1 - [CDAC1_MOUSE]	0.39	0.49	0.34	0.43	0.00
E9Q641	Nebulin-related-anchoring protein OS=Mus musculus GN=Nrap PE=2 SV=1 - [E9Q641_MOUSE]	0.46	0.38	0.39	0.31	0.00
Q8BLV3-2	Isoform 2 of Sodium/hydrogen exchanger 7 OS=Mus musculus GN=Slc9a7 - [SL9A7_MOUSE]	0.80	0.78	0.61	0.59	0.00
Q501J6	Probable ATP-dependent RNA helicase DDX17 OS=Mus musculus GN=Ddx17 PE=2 SV=1 - [DDX17_MOUSE]	0.25	0.31	0.22	0.25	0.00
Q64332	Synapsin-2 OS=Mus musculus GN=Syn2 PE=1 SV=2 - [SYN2_MOUSE]	1.11	1.04	1.47	1.31	0.00
Q8VI40	Tripartite motif-containing protein 60 OS=Mus musculus GN=Trim60 PE=2 SV=1 - [TRI60_MOUSE]	0.59	0.76	0.68	0.86	0.00
E9Q3Z4	Hexokinase-3 OS=Mus musculus GN=Hk3 PE=2 SV=1 - [E9Q3Z4_MOUSE]	0.59	0.54	0.44	0.43	0.00

P39654	Arachidonate 12-lipoxygenase, leukocyte-type OS=Mus musculus GN=Alox12l PE=1 SV=4 - [LX12L_MOUSE]	1.06	0.78	1.14	0.98	0.00
Q91ZE6	Beta-spectrin 4 OS=Mus musculus GN=Sptbn4 PE=2 SV=1 - [Q91ZE6_MOUSE]	0.49	0.54	0.38	0.42	0.00
Q9ET30	Transmembrane 9 superfamily member 3 OS=Mus musculus GN=Tm9sf3 PE=1 SV=1 - [TM9S3_MOUSE]	-0.32	-0.32	-0.36	-0.24	0.00
Q8BYA0	Tubulin-specific chaperone D OS=Mus musculus GN=Tbcd PE=2 SV=1 - [TBCD_MOUSE]	-0.78	-0.64	-0.80	-0.56	0.00
Q8R0N6	Hydroxyacid-oxoacid transhydrogenase, mitochondrial OS=Mus musculus GN=Adhfe1 PE=2 SV=2 - [HOT_MOUSE]	0.65	0.91	0.72	0.67	0.00
Q61581	Insulin-like growth factor-binding protein 7 OS=Mus musculus GN=Igfbp7 PE=2 SV=3 - [IBP7_MOUSE]	0.49	0.45	0.55	0.64	0.00
P70261	Paladin OS=Mus musculus GN=Pald1 PE=1 SV=1 - [PALD_MOUSE]	0.25	0.32	0.30	0.37	0.00
E9QMZ5	Plectin OS=Mus musculus GN=Plec PE=2 SV=1 - [E9QMZ5_MOUSE]	0.86	0.79	0.67	0.60	0.00
P11247	Myeloperoxidase OS=Mus musculus GN=Mpo PE=2 SV=2 - [PERM_MOUSE]	1.31	1.83	1.50	1.84	0.00
Q61599	Rho GDP-dissociation inhibitor 2 OS=Mus musculus GN=Arhgdib PE=1 SV=3 - [GDIR2_MOUSE]	0.59	0.56	0.77	0.75	0.00
Q922B2	Aspartate--tRNA ligase, cytoplasmic OS=Mus musculus GN=Dars PE=2 SV=2 - [SYDC_MOUSE]	-0.36	-0.40	-0.35	-0.27	0.00
P63044	Vesicle-associated membrane protein 2 OS=Mus musculus GN=Vamp2 PE=1 SV=2 - [VAMP2_MOUSE]	0.57	0.75	0.53	0.57	0.00
Q99PV0	Pre-mRNA-processing-splicing factor 8 OS=Mus musculus GN=Prpf8 PE=1 SV=2 - [PRP8_MOUSE]	0.14	0.17	0.14	0.19	0.00
E9PYL9	Uncharacterized protein OS=Mus musculus GN=Gm10036 PE=3 SV=1 - [E9PYL9_MOUSE]	-0.44	-0.45	-0.34	-0.33	0.00
P14869	60S acidic ribosomal protein P0 OS=Mus musculus GN=Rplp0 PE=1 SV=3 - [RLA0_MOUSE]	-0.63	-0.59	-0.47	-0.45	0.00
Q99M71-2	Isoform 2 of Mammalian ependymin-related protein 1 OS=Mus musculus GN=Epdr1 - [EPDR1_MOUSE]	0.47	0.37	0.43	0.33	0.00
Q8BG15-6	Isoform 6 of CTD small phosphatase-like protein 2 OS=Mus musculus GN=Ctdspl2 - [CTSL2_MOUSE]	-0.35	-0.27	-0.35	-0.27	0.00
A6H5X4-2	Isoform 2 of PHD finger protein 11 OS=Mus musculus GN=Phf11 - [PHF11_MOUSE]	0.97	0.73	1.02	0.78	0.00

A2A547	60S ribosomal protein L19 OS=Mus musculus GN=Rpl19 PE=2 SV=1 - [A2A547_MOUSE]	0.22	0.22	0.16	0.19	0.00
Q9QZD9	Eukaryotic translation initiation factor 3 subunit I OS=Mus musculus GN=Eif3i PE=1 SV=1 - [EIF3I_MOUSE]	0.21	0.27	0.24	0.31	0.00
Q8VDZ4	Palmitoyltransferase ZDHHC5 OS=Mus musculus GN=Zdhhc5 PE=1 SV=1 - [ZDHC5_MOUSE]	0.44	0.54	0.65	0.60	0.00
Q6R891	Neurabin-2 OS=Mus musculus GN=Ppp1r9b PE=1 SV=1 - [NEB2_MOUSE]	0.36	0.53	0.47	0.49	0.00
Q9JKK8	Serine/threonine-protein kinase ATR OS=Mus musculus GN=Atr PE=1 SV=2 - [ATR_MOUSE]	1.00	0.80	1.19	1.00	0.00
J3QP43	Protein 6330403A02Rik OS=Mus musculus GN=6330403A02Rik PE=4 SV=1 - [J3QP43_MOUSE]	-0.17	-0.18	-0.19	-0.24	0.00
Q62293	Interferon-gamma-inducible GTPase Ifggb5 protein OS=Mus musculus GN=Tgtp PE=2 SV=1 - [Q62293_MOUSE]	0.46	0.58	0.61	0.69	0.00
Q7TSC1	Protein PRRC2A OS=Mus musculus GN=Prrc2a PE=1 SV=1 - [PRC2A_MOUSE]	0.46	0.35	0.52	0.42	0.00
P05201	Aspartate aminotransferase, cytoplasmic OS=Mus musculus GN=Got1 PE=1 SV=3 - [AATC_MOUSE]	-0.21	-0.17	-0.24	-0.18	0.00
Q80VQ1	Leucine-rich repeat-containing protein 1 OS=Mus musculus GN=Lrrc1 PE=2 SV=2 - [LRRC1_MOUSE]	-0.59	-0.74	-0.56	-0.77	0.00
E9Q8T1	Transforming acidic coiled-coil-containing protein 2 OS=Mus musculus GN=Tacc2 PE=2 SV=1 - [E9Q8T1_MOUSE]	-0.91	-0.62	-0.72	-0.71	0.00
Q9Z127	Large neutral amino acids transporter small subunit 1 OS=Mus musculus GN=Slc7a5 PE=1 SV=2 - [LAT1_MOUSE]	-0.33	-0.30	-0.43	-0.40	0.00
Q8BWM0	Prostaglandin E synthase 2 OS=Mus musculus GN=Ptges2 PE=1 SV=3 - [PGES2_MOUSE]	-0.75	-0.67	-0.73	-0.51	0.00
O09049	Regenerating islet-derived protein 3-gamma OS=Mus musculus GN=Reg3g PE=1 SV=1 - [REG3G_MOUSE]	1.55	2.16	1.67	2.10	0.00
D3YWH4	Transmembrane 9 superfamily member 1 (Fragment) OS=Mus musculus GN=Tm9sf1 PE=2 SV=1 - [D3YWH4_MOUSE]	-0.22	-0.29	-0.21	-0.21	0.00
Q8VDP4	DBIRD complex subunit KIAA1967 homolog OS=Mus musculus PE=1 SV=2 - [K1967_MOUSE]	0.49	0.50	0.58	0.39	0.00
P51125-3	Isoform 3 of Calpastatin OS=Mus musculus GN=Cast - [ICAL_MOUSE]	0.82	0.72	1.06	0.87	0.00
O35083	1-acyl-sn-glycerol-3-phosphate acyltransferase alpha OS=Mus musculus GN=Agpat1 PE=2 SV=1 - [PLCA_MOUSE]	-0.61	-0.54	-0.41	-0.49	0.00

P12787	Cytochrome c oxidase subunit 5A, mitochondrial OS=Mus musculus GN=Cox5a PE=1 SV=2 - [COX5A_MOUSE]	-0.59	-0.71	-0.50	-0.71	0.00
Q9DBS1	Transmembrane protein 43 OS=Mus musculus GN=Tmem43 PE=1 SV=1 - [TMM43_MOUSE]	-0.18	-0.13	-0.13	-0.12	0.00
Q6ZPI3	Ligand-dependent corepressor OS=Mus musculus GN=Lcor PE=2 SV=2 - [LCOR_MOUSE]	-0.18	-0.15	-0.22	-0.20	0.00
D3YWV8	Protein Sycp2l (Fragment) OS=Mus musculus GN=Sycp2l PE=2 SV=1 - [D3YWV8_MOUSE]	-0.22	-0.27	-0.28	-0.33	0.00
Q6PB44-2	Isoform 2 of Tyrosine-protein phosphatase non-receptor type 23 OS=Mus musculus GN=Ptpn23 - [PTN23_MOUSE]	-0.17	-0.20	-0.20	-0.14	0.00
D3YZP9	Coiled-coil domain-containing protein 6 OS=Mus musculus GN=Ccdc6 PE=3 SV=1 - [CCDC6_MOUSE]	0.27	0.34	0.23	0.26	0.00
Q8BMF4	Dihydrolipoyllysine-residue acetyltransferase component of pyruvate dehydrogenase complex, mitochondrial OS=Mus musculus GN=Dlat PE=1 SV=2 [ODP2_MOUSE]	-0.26	-0.31	-0.21	-0.26	0.00
P54822	Adenylosuccinate lyase OS=Mus musculus GN=Adsl PE=2 SV=2 - [PUR8_MOUSE]	-0.98	-0.84	-0.79	-0.66	0.00
P97742	Carnitine O-palmitoyltransferase 1, liver isoform OS=Mus musculus GN=Cpt1a PE=1 SV=4 - [CPT1A_MOUSE]	-0.83	-0.62	-0.64	-0.59	0.00
Q3V3R4	Integrin alpha-1 OS=Mus musculus GN=Itga1 PE=2 SV=2 - [ITA1_MOUSE]	-0.39	-0.30	-0.43	-0.44	0.00
G5E898	Periplakin OS=Mus musculus GN=Ppl PE=4 SV=1 - [G5E898_MOUSE]	0.58	0.44	0.59	0.66	0.00
Q7TMB8	Cytoplasmic FMR1-interacting protein 1 OS=Mus musculus GN=Cyfip1 PE=1 SV=1 - [CYFP1_MOUSE]	-0.86	-0.74	-1.07	-0.82	0.00
Q80YA8	Protein crumbs homolog 2 OS=Mus musculus GN=Crb2 PE=2 SV=3 - [CRUM2_MOUSE]	-0.74	-0.74	-0.98	-0.98	0.00
Q9QXD8	LIM domain-containing protein 1 OS=Mus musculus GN=Limd1 PE=1 SV=2 - [LIMD1_MOUSE]	0.39	0.59	0.50	0.54	0.00
P01680	Ig kappa chain V-IV region S107B OS=Mus musculus PE=4 SV=1 - [KV4A1_MOUSE]	-1.90	-1.85	-2.51	-2.47	0.00
Q9D0E1-2	Isoform 2 of Heterogeneous nuclear ribonucleoprotein M OS=Mus musculus GN=Hnrnpm - [HNRPM_MOUSE]	0.38	0.37	0.28	0.28	0.00
Q3UTH8-2	Isoform 2 of Rho guanine nucleotide exchange factor 9 OS=Mus musculus GN=Arhgef9 - [ARHG9_MOUSE]	-0.48	-0.42	-0.38	-0.32	0.00
Q3USF0	UDP-GlcNAc:betaGal beta-1,3-N-acetylglucosaminyltransferase 6 OS=Mus musculus GN=B3gnt6 PE=2 SV=2 - [B3GN6_MOUSE]	1.01	0.77	1.11	0.86	0.00

Q62388	Serine-protein kinase ATM OS=Mus musculus GN=Atm PE=1 SV=2 - [ATM_MOUSE]	-0.76	-0.57	-0.78	-0.59	0.00
Q69ZF7	Metal transporter CNNM4 OS=Mus musculus GN=Cnnm4 PE=1 SV=2 - [CNNM4_MOUSE]	-1.13	-0.78	-1.14	-1.07	0.00
D3YUM1	NADH dehydrogenase [ubiquinone] flavoprotein 1, mitochondrial OS=Mus musculus GN=Ndufv1 PE=2 SV=1 - [D3YUM1_MOUSE]	-0.63	-0.73	-0.51	-0.73	0.00
Q9Z1B7	Mitogen-activated protein kinase 13 OS=Mus musculus GN=Mapk13 PE=1 SV=2 - [MK13_MOUSE]	-0.23	-0.21	-0.15	-0.18	0.00
Q91YD9	Neural Wiskott-Aldrich syndrome protein OS=Mus musculus GN=Wasl PE=1 SV=1 - [WASL_MOUSE]	0.23	0.20	0.27	0.19	0.00
Q8BTW3	Exosome complex component MTR3 OS=Mus musculus GN=Exosc6 PE=1 SV=1 - [EXOS6_MOUSE]	0.52	0.36	0.54	0.50	0.00
O09117-2	Isoform 2 of Synaptophysin-like protein 1 OS=Mus musculus GN=Sypl1 - [SYPL1_MOUSE]	-0.87	-0.77	-0.80	-0.58	0.00
Q78IQ7	Zinc transporter ZIP4 OS=Mus musculus GN=Slc39a4 PE=1 SV=1 - [S39A4_MOUSE]	-0.43	-0.37	-0.51	-0.36	0.00
Q04447	Creatine kinase B-type OS=Mus musculus GN=Ckb PE=1 SV=1 - [KCRB_MOUSE]	0.25	0.21	0.30	0.23	0.00
Q924M7	Mannose-6-phosphate isomerase OS=Mus musculus GN=Mpi PE=2 SV=1 - [MPI_MOUSE]	-0.42	-0.42	-0.29	-0.35	0.00
Q9CQU3	Protein RER1 OS=Mus musculus GN=Rer1 PE=1 SV=1 - [RER1_MOUSE]	-0.59	-0.76	-0.61	-0.51	0.00
Q8K1R7	Serine/threonine-protein kinase Nek9 OS=Mus musculus GN=Nek9 PE=1 SV=2 - [NEK9_MOUSE]	0.12	0.11	0.09	0.08	0.00
Q60902-4	Isoform 4 of Epidermal growth factor receptor substrate 15-like 1 OS=Mus musculus GN=Eps15l1 - [EP15R_MOUSE]	0.39	0.51	0.51	0.60	0.00
F6RBL8	Intraflagellar transport protein 52 homolog (Fragment) OS=Mus musculus GN=Ift52 PE=2 SV=1 - [F6RBL8_MOUSE]	-0.81	-0.69	-0.65	-0.54	0.00
Q8R4U7	Leucine zipper protein 1 OS=Mus musculus GN=Luzp1 PE=1 SV=2 - [LUZP1_MOUSE]	0.58	0.57	0.62	0.80	0.00
Q9D6J6	NADH dehydrogenase [ubiquinone] flavoprotein 2, mitochondrial OS=Mus musculus GN=Ndufv2 PE=1 SV=2 - [NDUV2_MOUSE]	-0.44	-0.44	-0.61	-0.56	0.00
Q9WVJ2	26S proteasome non-ATPase regulatory subunit 13 OS=Mus musculus GN=Psmd13 PE=1 SV=1 - [PSD13_MOUSE]	-0.40	-0.29	-0.30	-0.31	0.00
Q8CHR6	Dihydropyrimidine dehydrogenase [NADP(+)] OS=Mus musculus GN=Dpyd PE=2 SV=1 - [DPYD_MOUSE]	0.31	0.32	0.31	0.22	0.00

Q8CG48	Structural maintenance of chromosomes protein 2 OS=Mus musculus GN=Smc2 PE=1 SV=2 - [SMC2_MOUSE]	-0.25	-0.24	-0.34	-0.25	0.00
O08784	Treacle protein OS=Mus musculus GN=Tcof1 PE=1 SV=1 - [TCOF_MOUSE]	0.73	0.54	0.60	0.51	0.00
Q9R1T2	SUMO-activating enzyme subunit 1 OS=Mus musculus GN=Sae1 PE=2 SV=1 - [SAE1_MOUSE]	-0.31	-0.31	-0.22	-0.26	0.00
P97807-2	Isoform Cytoplasmic of Fumarate hydratase, mitochondrial OS=Mus musculus GN=Fh - [FUMH_MOUSE]	-0.15	-0.18	-0.12	-0.14	0.00
Q9D6K5-4	Isoform 4 of Synaptojanin-2-binding protein OS=Mus musculus GN=Synj2bp - [SYJ2B_MOUSE]	-0.67	-0.99	-0.78	-0.78	0.00
D3Z2X2	Protein Dnhd1 OS=Mus musculus GN=Dnhd1 PE=2 SV=2 - [D3Z2X2_MOUSE]	-1.27	-0.95	-1.39	-1.08	0.00
P01638	Ig kappa chain V-V region L6 (Fragment) OS=Mus musculus PE=4 SV=1 - [KV5A6_MOUSE]	-0.67	-0.56	-0.78	-0.82	0.00
Q9WTQ5-2	Isoform 2 of A-kinase anchor protein 12 OS=Mus musculus GN=Akap12 - [AKA12_MOUSE]	0.54	0.63	0.71	0.81	0.00
Q99M15	Proline-serine-threonine phosphatase-interacting protein 2 OS=Mus musculus GN=Pstpip2 PE=1 SV=4 - [PPIP2_MOUSE]	0.95	1.06	1.36	0.99	0.00
Q64012-2	Isoform 1 of RNA-binding protein Raly OS=Mus musculus GN=Raly - [RALY_MOUSE]	0.29	0.30	0.32	0.41	0.00
Q8CGZ0	Calcium homeostasis endoplasmic reticulum protein OS=Mus musculus GN=Cherp PE=1 SV=1 - [CHERP_MOUSE]	0.73	1.04	0.81	1.00	0.00
Q78ZA7	Nucleosome assembly protein 1-like 4 OS=Mus musculus GN=Nap1l4 PE=1 SV=1 - [NP1L4_MOUSE]	0.39	0.32	0.47	0.45	0.00
Q8VC91	Ikbkg protein OS=Mus musculus GN=Ikbkg PE=2 SV=1 - [Q8VC91_MOUSE]	0.47	0.48	0.41	0.33	0.00
P19536	Cytochrome c oxidase subunit 5B, mitochondrial OS=Mus musculus GN=Cox5b PE=1 SV=1 - [COX5B_MOUSE]	-0.52	-0.55	-0.40	-0.40	0.00
Q60759	Glutaryl-CoA dehydrogenase, mitochondrial OS=Mus musculus GN=Gcdh PE=2 SV=2 - [GCDH_MOUSE]	-0.24	-0.20	-0.30	-0.27	0.00
Q9R1P4	Proteasome subunit alpha type-1 OS=Mus musculus GN=Psma1 PE=1 SV=1 - [PSA1_MOUSE]	0.21	0.22	0.15	0.19	0.00
P15626	Glutathione S-transferase Mu 2 OS=Mus musculus GN=Gstm2 PE=1 SV=2 - [GSTM2_MOUSE]	-0.70	-0.58	-0.52	-0.48	0.00
Q9CQE8	UPF0568 protein C14orf166 homolog OS=Mus musculus PE=2 SV=1 - [CN166_MOUSE]	0.09	0.09	0.10	0.12	0.00

Q60597	2-oxoglutarate dehydrogenase, mitochondrial OS=Mus musculus GN=Ogdh PE=1 SV=3 - [ODO1_MOUSE]	-0.13	-0.15	-0.12	-0.10	0.00
Q61074	Protein phosphatase 1G OS=Mus musculus GN=Ppm1g PE=2 SV=3 - [PPM1G_MOUSE]	0.23	0.31	0.23	0.30	0.00
P16675	Lysosomal protective protein OS=Mus musculus GN=Ctsa PE=1 SV=1 - [PPGB_MOUSE]	-0.46	-0.57	-0.46	-0.38	0.00
Q8K2T8	RNA polymerase II-associated factor 1 homolog OS=Mus musculus GN=Paf1 PE=2 SV=1 - [PAF1_MOUSE]	1.32	0.95	1.17	0.93	0.00
Q9CQH3	NADH dehydrogenase [ubiquinone] 1 beta subcomplex subunit 5, mitochondrial OS=Mus musculus GN=Ndufb5 PE=1 SV=1 - [NDUB5_MOUSE]	-0.51	-0.60	-0.41	-0.44	0.00
Q8R5A3	Amyloid beta A4 precursor protein-binding family B member 1-interacting protein OS=Mus musculus GN=Apbb1ip PE=1 SV=2 - [AB1IP_MOUSE]	0.45	0.44	0.32	0.34	0.00
Q8C4U3	Secreted frizzled-related protein 1 OS=Mus musculus GN=Sfrp1 PE=1 SV=3 - [SFRP1_MOUSE]	0.64	0.73	0.80	0.95	0.00
P10922	Histone H1.0 OS=Mus musculus GN=H1f0 PE=2 SV=4 - [H10_MOUSE]	-0.44	-0.35	-0.38	-0.29	0.00
P26041	Moesin OS=Mus musculus GN=Msn PE=1 SV=3 - [MOES_MOUSE]	0.25	0.22	0.31	0.32	0.00
B2ZRS5	DEP domain-containing mTOR-interacting protein OS=Mus musculus GN=Deptor PE=4 SV=1 - [B2ZRS5_MOUSE]	-0.34	-0.31	-0.32	-0.23	0.00
P22315	Ferrochelatase, mitochondrial OS=Mus musculus GN=Fech PE=1 SV=2 - [HEMH_MOUSE]	-0.35	-0.40	-0.28	-0.42	0.00
Q8K2I3	Dimethylaniline monooxygenase [N-oxide-forming] 2 OS=Mus musculus GN=Fmo2 PE=1 SV=3 - [FMO2_MOUSE]	-0.67	-0.65	-0.93	-0.81	0.00
Q91VN6	Probable ATP-dependent RNA helicase DDX41 OS=Mus musculus GN=Ddx41 PE=1 SV=2 - [DDX41_MOUSE]	0.49	0.56	0.65	0.72	0.00
A2AR02	Peptidyl-prolyl cis-trans isomerase G OS=Mus musculus GN=Ppig PE=1 SV=1 - [PPIG_MOUSE]	0.58	0.59	0.44	0.67	0.00
P54728	UV excision repair protein RAD23 homolog B OS=Mus musculus GN=Rad23b PE=1 SV=2 - [RD23B_MOUSE]	0.50	0.37	0.39	0.34	0.00
O89079	Coatomer subunit epsilon OS=Mus musculus GN=Cope PE=2 SV=3 - [COPE_MOUSE]	-0.34	-0.28	-0.43	-0.35	0.00
P31750	RAC-alpha serine/threonine-protein kinase OS=Mus musculus GN=Akt1 PE=1 SV=2 - [AKT1_MOUSE]	-0.75	-0.75	-0.55	-0.56	0.00
Q9BCZ4	Selenoprotein S OS=Mus musculus GN=Vimp PE=2 SV=3 - [SELS_MOUSE]	-0.14	-0.12	-0.18	-0.17	0.00

Q8C9B9	Death-inducer obliterator 1 OS=Mus musculus GN=Dido1 PE=1 SV=4 - [DIDO1_MOUSE]	0.98	0.96	0.85	0.66	0.00
E9Q7D5	Protein Arhgef5 OS=Mus musculus GN=Arhgef5 PE=2 SV=1 - [E9Q7D5_MOUSE]	0.72	0.83	0.54	0.71	0.00
Q920E5	Farnesyl pyrophosphate synthase OS=Mus musculus GN=Fdps PE=2 SV=1 - [FPPS_MOUSE]	-0.53	-0.61	-0.40	-0.49	0.00
F8WIB1	ADP-ribosylation factor-like protein 1 (Fragment) OS=Mus musculus GN=Arl1 PE=2 SV=1 - [F8WIB1_MOUSE]	-0.64	-0.43	-0.63	-0.57	0.00
G5E8Q4	Cytohesin-3 OS=Mus musculus GN=Cyth3 PE=4 SV=1 - [G5E8Q4_MOUSE]	0.34	0.45	0.31	0.41	0.00
O08583	THO complex subunit 4 OS=Mus musculus GN=Alyref PE=1 SV=3 - [THOC4_MOUSE]	0.50	0.33	0.40	0.39	0.00
Q9CR92	Coiled-coil domain-containing protein 96 OS=Mus musculus GN=Ccdc96 PE=2 SV=1 - [CCD96_MOUSE]	-0.75	-0.64	-0.96	-0.86	0.00
A6ZI44	Fructose-bisphosphate aldolase OS=Mus musculus GN=Aldoa PE=2 SV=1 - [A6ZI44_MOUSE]	0.27	0.21	0.18	0.23	0.00
Q9EQQ2	Protein YIPF5 OS=Mus musculus GN=Yipf5 PE=2 SV=1 - [YIPF5_MOUSE]	1.08	1.26	0.82	1.16	0.00
E9PUZ8	C4b-binding protein (Fragment) OS=Mus musculus GN=C4bp PE=2 SV=1 - [E9PUZ8_MOUSE]	0.79	0.52	0.65	0.65	0.00
Q91YJ5	Translation initiation factor IF-2, mitochondrial OS=Mus musculus GN=Mtif2 PE=1 SV=2 - [IF2M_MOUSE]	0.52	0.68	0.64	0.79	0.00
Q810Q5	Normal mucosa of esophagus-specific gene 1 protein OS=Mus musculus GN=Nmes1 PE=2 SV=1 - [NMES1_MOUSE]	-0.39	-0.47	-0.35	-0.32	0.00
P03930	ATP synthase protein 8 OS=Mus musculus GN=Mtatp8 PE=1 SV=1 - [ATP8_MOUSE]	-0.32	-0.40	-0.27	-0.39	0.00
Q9CWS0	N(G),N(G)-dimethylarginine dimethylaminohydrolase 1 OS=Mus musculus GN=Ddah1 PE=1 SV=3 - [DDAH1_MOUSE]	0.33	0.30	0.44	0.39	0.00
D3YUJ0	Ellis-van Creveld syndrome protein homolog OS=Mus musculus GN=Evc PE=2 SV=1 - [D3YUJ0_MOUSE]	-0.67	-0.91	-0.70	-0.94	0.00
P49813	Tropomodulin-1 OS=Mus musculus GN=Tmod1 PE=2 SV=2 - [TMOD1_MOUSE]	0.76	0.72	0.50	0.67	0.00
Q2M2N2	Speckle-type POZ protein-like OS=Mus musculus GN=Spopl PE=2 SV=3 - [SPOPL_MOUSE]	1.32	1.03	1.15	0.87	0.00
Q61151	Serine/threonine-protein phosphatase 2A 56 kDa regulatory subunit epsilon isoform OS=Mus musculus GN=Ppp2r5e PE=2 SV=3 - [2A5E_MOUSE]	-0.85	-0.71	-1.08	-0.85	0.00

E9QAD4	Coiled-coil domain-containing protein 93 OS=Mus musculus GN=Ccdc93 PE=2 SV=1 - [E9QAD4_MOUSE]	-0.47	-0.37	-0.35	-0.32	0.00
D3YZD6	Quinone oxidoreductase-like protein 1 OS=Mus musculus GN=Cryzl1 PE=2 SV=1 - [D3YZD6_MOUSE]	0.28	0.33	0.22	0.31	0.00
Q9EPC1	Alpha-parvin OS=Mus musculus GN=Parva PE=1 SV=1 - [PARVA_MOUSE]	-0.46	-0.38	-0.58	-0.52	0.00
H3BKG8	Putative adenosylhomocysteinase 3 (Fragment) OS=Mus musculus GN=Ahcyl2 PE=2 SV=1 - [H3BKG8_MOUSE]	0.47	0.35	0.53	0.41	0.00
Q3V384	Lactation elevated protein 1 OS=Mus musculus GN=Lace1 PE=2 SV=1 - [LACE1_MOUSE]	-0.47	-0.64	-0.44	-0.54	0.00
P53811	Phosphatidylinositol transfer protein beta isoform OS=Mus musculus GN=Pitpnb PE=1 SV=2 - [PIPNB_MOUSE]	-0.72	-0.64	-0.68	-0.47	0.00
P31725	Protein S100-A9 OS=Mus musculus GN=S100a9 PE=1 SV=3 - [S10A9_MOUSE]	1.42	1.19	1.81	1.55	0.00
O88543	COP9 signalosome complex subunit 3 OS=Mus musculus GN=Cops3 PE=1 SV=3 - [CSN3_MOUSE]	-0.18	-0.14	-0.20	-0.15	0.00
Q9QYA2	Mitochondrial import receptor subunit TOM40 homolog OS=Mus musculus GN=Tomm40 PE=1 SV=3 - [TOM40_MOUSE]	-0.73	-0.78	-0.53	-0.61	0.00
Q6P5F9	Exportin-1 OS=Mus musculus GN=Xpo1 PE=1 SV=1 - [XPO1_MOUSE]	-0.72	-0.77	-0.58	-0.53	0.00
Q3UHX2	28 kDa heat- and acid-stable phosphoprotein OS=Mus musculus GN=Pdap1 PE=1 SV=1 - [HAP28_MOUSE]	0.61	0.49	0.73	0.72	0.00
Q921K8	Protein FAM115C OS=Mus musculus GN=Fam115c PE=2 SV=1 - [F115C_MOUSE]	-0.60	-0.59	-0.85	-0.72	0.00
O88286-2	Isoform S of Protein Wiz OS=Mus musculus GN=Wiz - [WIZ_MOUSE]	0.64	0.50	0.76	0.59	0.00
G3X9V2	Catenin (Cadherin associated protein), delta 1, isoform CRA_a OS=Mus musculus GN=Ctnnd1 PE=4 SV=1 - [G3X9V2_MOUSE]	0.50	0.57	0.68	0.74	0.00
B9EKI3	TATA element modulatory factor OS=Mus musculus GN=Tmf1 PE=1 SV=2 - [TMF1_MOUSE]	0.52	0.60	0.43	0.42	0.00
E9PWF0	Thrombospondin-3 OS=Mus musculus GN=Thbs3 PE=2 SV=1 - [E9PWF0_MOUSE]	-0.49	-0.64	-0.61	-0.76	0.00
Q64092-3	Isoform 3 of Transcription factor E3 OS=Mus musculus GN=Tfe3 - [TFE3_MOUSE]	-0.51	-0.54	-0.37	-0.41	0.00
Q91WD5	NADH dehydrogenase [ubiquinone] iron-sulfur protein 2, mitochondrial OS=Mus musculus GN=Ndufs2 PE=1 SV=1 - [NDUS2_MOUSE]	-0.67	-0.51	-0.79	-0.65	0.00

Q8BJ71	Nuclear pore complex protein Nup93 OS=Mus musculus GN=Nup93 PE=2 SV=1 - [NUP93_MOUSE]	-0.22	-0.18	-0.19	-0.14	0.00
Q6GQT9	Nodal modulator 1 OS=Mus musculus GN=Nomo1 PE=1 SV=1 - [NOMO1_MOUSE]	-0.22	-0.19	-0.20	-0.15	0.00
Q9Z1D1	Eukaryotic translation initiation factor 3 subunit G OS=Mus musculus GN=Eif3g PE=1 SV=2 - [EIF3G_MOUSE]	0.77	0.54	0.84	0.74	0.00
Q8JZS0	Protein lin-7 homolog A OS=Mus musculus GN=Lin7a PE=1 SV=2 - [LIN7A_MOUSE]	0.37	0.48	0.46	0.57	0.00
Q9CQA3	Succinate dehydrogenase [ubiquinone] iron-sulfur subunit, mitochondrial OS=Mus musculus GN=Sdhb PE=1 SV=1 - [DHSB_MOUSE]	-0.46	-0.48	-0.34	-0.35	0.00
Q8K2F8	Protein LSM14 homolog A OS=Mus musculus GN=Lsm14a PE=1 SV=1 - [LS14A_MOUSE]	0.75	0.58	0.70	0.89	0.00
A2AQ25	Sickle tail protein OS=Mus musculus GN=Skt PE=1 SV=1 - [SKT_MOUSE]	0.42	0.44	0.31	0.48	0.00
Q04899	Cyclin-dependent kinase 18 OS=Mus musculus GN=Cdk18 PE=1 SV=1 - [CDK18_MOUSE]	0.77	0.62	0.64	0.50	0.00
P61089	Ubiquitin-conjugating enzyme E2 N OS=Mus musculus GN=Ube2n PE=1 SV=1 - [UBE2N_MOUSE]	-0.79	-0.62	-0.71	-0.52	0.00
Q8K157	Aldose 1-epimerase OS=Mus musculus GN=Galm PE=2 SV=1 - [GALM_MOUSE]	-0.80	-0.72	-0.52	-0.71	0.00
A2APB1	Ras association domain-containing protein 2 (Fragment) OS=Mus musculus GN=Rassf2 PE=2 SV=1 - [A2APB1_MOUSE]	0.39	0.36	0.53	0.49	0.00
P62806	Histone H4 OS=Mus musculus GN=Hist1h4a PE=1 SV=2 - [H4_MOUSE]	0.42	0.28	0.42	0.35	0.00
Q9CQW9	Interferon-induced transmembrane protein 3 OS=Mus musculus GN=Ifitm3 PE=1 SV=1 - [IFM3_MOUSE]	1.04	0.96	0.82	0.69	0.00
P70399	Tumor suppressor p53-binding protein 1 OS=Mus musculus GN=Tp53bp1 PE=1 SV=2 - [TP53B_MOUSE]	0.36	0.46	0.30	0.41	0.00
Q5SXY1-3	Isoform 4 of Cytospin-B OS=Mus musculus GN=Specc1 - [CYTSB_MOUSE]	0.33	0.23	0.28	0.35	0.00
P24638	Lysosomal acid phosphatase OS=Mus musculus GN=Acp2 PE=2 SV=2 - [PPAL_MOUSE]	-0.53	-0.46	-0.42	-0.34	0.00
P17156	Heat shock-related 70 kDa protein 2 OS=Mus musculus GN=Hspa2 PE=1 SV=2 - [HSP72_MOUSE]	-0.17	-0.20	-0.15	-0.22	0.00
Q8BT60	Copine-3 OS=Mus musculus GN=Cpne3 PE=1 SV=2 - [CPNE3_MOUSE]	0.36	0.41	0.52	0.51	0.00

Q61129	Complement factor I OS=Mus musculus GN=Cfi PE=1 SV=3 - [CFAI_MOUSE]	0.82	0.77	0.54	0.66	0.00
P61205	ADP-ribosylation factor 3 OS=Mus musculus GN=Arf3 PE=2 SV=2 - [ARF3_MOUSE]	-0.76	-0.53	-0.77	-0.60	0.00
Q05860-4	Isoform 4 of Formin-1 OS=Mus musculus GN=Fmn1 - [FMN1_MOUSE]	-0.37	-0.35	-0.27	-0.26	0.00
Q9WUU7	Cathepsin Z OS=Mus musculus GN=Ctsz PE=2 SV=1 - [CATZ_MOUSE]	-0.16	-0.13	-0.20	-0.18	0.00
E9PXV7	Protein diaphanous homolog 1 OS=Mus musculus GN=Diap1 PE=2 SV=1 - [E9PXV7_MOUSE]	-0.57	-0.38	-0.51	-0.41	0.00
Q06138	Calcium-binding protein 39 OS=Mus musculus GN=Cab39 PE=1 SV=2 - [CAB39_MOUSE]	-1.21	-0.94	-1.31	-0.92	0.00
Q8BG30	Negative elongation factor A OS=Mus musculus GN=Nelfa PE=1 SV=1 - [NELFA_MOUSE]	0.72	0.69	0.89	0.58	0.00
Q922S4	cGMP-dependent 3',5'-cyclic phosphodiesterase OS=Mus musculus GN=Pde2a PE=1 SV=3 - [PDE2A_MOUSE]	-0.57	-0.42	-0.48	-0.64	0.00
O88492	Perilipin-4 OS=Mus musculus GN=Plin4 PE=1 SV=2 - [PLIN4_MOUSE]	0.72	0.72	1.02	0.92	0.00
E9QNL4	Inhibitor of nuclear factor kappa-B kinase subunit alpha OS=Mus musculus GN=Chuk PE=2 SV=1 - [E9QNL4_MOUSE]	0.75	0.99	0.67	0.90	0.00
Q9CRC8	Leucine-rich repeat-containing protein 40 OS=Mus musculus GN=Lrrc40 PE=2 SV=2 - [LRC40_MOUSE]	-0.35	-0.35	-0.46	-0.50	0.00
B2RX12-3	Isoform 3 of Canalicular multispecific organic anion transporter 2 OS=Mus musculus GN=Abcc3 - [MRP3_MOUSE]	-0.22	-0.30	-0.30	-0.22	0.00
Q9DBX6	Cytochrome P450 2S1 OS=Mus musculus GN=Cyp2s1 PE=2 SV=1 - [CP2S1_MOUSE]	-0.60	-0.77	-0.84	-0.93	0.00
Q64735-2	Isoform 2 of Complement component receptor 1-like protein OS=Mus musculus GN=Cr1l - [CR1L_MOUSE]	-0.65	-0.63	-0.56	-0.43	0.00
P28663	Beta-soluble NSF attachment protein OS=Mus musculus GN=Napb PE=1 SV=2 - [SNAB_MOUSE]	-0.27	-0.35	-0.24	-0.33	0.00
Q8QZY1	Eukaryotic translation initiation factor 3 subunit L OS=Mus musculus GN=Eif3l PE=1 SV=1 - [EIF3L_MOUSE]	-0.16	-0.20	-0.24	-0.25	0.00
O70622-2	Isoform 2 of Reticulon-2 OS=Mus musculus GN=Rtn2 - [RTN2_MOUSE]	-0.63	-0.80	-0.51	-0.65	0.00
Q5PSV9	Mediator of DNA damage checkpoint protein 1 OS=Mus musculus GN=Mdc1 PE=1 SV=1 - [MDC1_MOUSE]	0.35	0.48	0.39	0.52	0.00

Q91VM9	Inorganic pyrophosphatase 2, mitochondrial OS=Mus musculus GN=Ppa2 PE=2 SV=1 - [IPYR2_MOUSE]	0.23	0.17	0.25	0.25	0.00
G3UZ26	Serine hydroxymethyltransferase (Fragment) OS=Mus musculus GN=Shmt1 PE=3 SV=1 - [G3UZ26_MOUSE]	-0.30	-0.34	-0.30	-0.22	0.00
A7DTG3	SERTA domain-containing protein 4 OS=Mus musculus GN=Sertad4 PE=2 SV=1 - [SRTD4_MOUSE]	0.16	0.16	0.22	0.22	0.00
Q9EQ08	Heparan N-sulfatase OS=Mus musculus GN=Sgsh PE=2 SV=1 - [Q9EQ08_MOUSE]	-0.71	-0.60	-0.58	-0.45	0.00
Q2LKU9	NACHT-, LRR-, and PYD-containing protein 1 paralog a OS=Mus musculus GN=Nlrp1a PE=2 SV=1 - [Q2LKU9_MOUSE]	-0.60	-0.80	-0.54	-0.75	0.00
Q921U8-2	Isoform L2 of Smoothelin OS=Mus musculus GN=Smtn - [SMTN_MOUSE]	-0.27	-0.33	-0.29	-0.40	0.00
Q8C2Q8	ATP synthase subunit gamma OS=Mus musculus GN=Atp5c1 PE=2 SV=1 - [Q8C2Q8_MOUSE]	-0.42	-0.34	-0.32	-0.27	0.00
Q8BK30	NADH dehydrogenase [ubiquinone] flavoprotein 3, mitochondrial OS=Mus musculus GN=Ndufv3 PE=2 SV=1 - [NDUV3_MOUSE]	-1.02	-0.85	-0.82	-0.65	0.00
Q9WTI7-3	Isoform 3 of Unconventional myosin-Ic OS=Mus musculus GN=Myo1c - [MYO1C_MOUSE]	-0.30	-0.27	-0.41	-0.34	0.00
B2RS76	Carboxypeptidase B1 (Tissue) OS=Mus musculus GN=Cpb1 PE=2 SV=1 - [B2RS76_MOUSE]	0.44	0.61	0.63	0.70	0.00
Q8VIB3	Type 2 lactosamine alpha-2,3-sialyltransferase OS=Mus musculus GN=St3gal6 PE=2 SV=3 - [SIA10_MOUSE]	-1.20	-1.26	-1.72	-1.66	0.00
Q9CR57	60S ribosomal protein L14 OS=Mus musculus GN=Rpl14 PE=2 SV=3 - [RL14_MOUSE]	-0.13	-0.10	-0.15	-0.11	0.00
Q9CWJ9	Bifunctional purine biosynthesis protein PURH OS=Mus musculus GN=Atic PE=1 SV=2 - [PUR9_MOUSE]	0.14	0.14	0.18	0.20	0.00
Q6ZWN5	40S ribosomal protein S9 OS=Mus musculus GN=Rps9 PE=2 SV=3 - [RS9_MOUSE]	-0.28	-0.22	-0.33	-0.25	0.00
Q91Y74	CMP-N-acetylneuraminate-beta-galactosamide-alpha-2,3-sialyltransferase 4 OS=Mus musculus GN=St3gal4 PE=2 SV=1 - [SIA4C_MOUSE]	-0.59	-0.63	-0.75	-0.87	0.00
F8VQF9	Trafficking protein particle complex subunit 10 OS=Mus musculus GN=Trappc10 PE=4 SV=1 - [F8VQF9_MOUSE]	-0.67	-0.89	-0.59	-0.80	0.00
O88736	3-keto-steroid reductase OS=Mus musculus GN=Hsd17b7 PE=2 SV=1 - [DHB7_MOUSE]	-0.42	-0.51	-0.64	-0.47	0.00
Q80SX0	StAR-related lipid transfer protein 4 OS=Mus musculus GN=Stard4 PE=2 SV=1 - [Q80SX0_MOUSE]	-0.92	-0.87	-0.68	-0.63	0.00

Q99KF1	Transmembrane emp24 domain-containing protein 9 OS=Mus musculus GN=Tmed9 PE=2 SV=2 - [TMED9_MOUSE]	-0.31	-0.30	-0.22	-0.23	0.00
Q9QXN3-2	Isoform 2 of Activating signal cointegrator 1 OS=Mus musculus GN=Trip4 - [TRIP4_MOUSE]	0.54	0.48	0.41	0.35	0.00
Q02257	Junction plakoglobin OS=Mus musculus GN=Jup PE=1 SV=3 - [PLAK_MOUSE]	-0.31	-0.31	-0.22	-0.24	0.00
E9Q8E3	Protein Abi3bp OS=Mus musculus GN=Abi3bp PE=2 SV=1 - [E9Q8E3_MOUSE]	-0.47	-0.39	-0.59	-0.56	0.00
E9PXB7	E3 ubiquitin-protein ligase NEDD4-like OS=Mus musculus GN=Nedd4l PE=2 SV=1 - [E9PXB7_MOUSE]	0.20	0.16	0.14	0.21	0.00
P14152	Malate dehydrogenase, cytoplasmic OS=Mus musculus GN=Mdh1 PE=1 SV=3 - [MDHC_MOUSE]	-0.33	-0.34	-0.24	-0.25	0.00
E9PVA8	Protein Gcn1l1 OS=Mus musculus GN=Gcn1l1 PE=2 SV=1 - [E9PVA8_MOUSE]	-0.21	-0.26	-0.29	-0.20	0.00
Q8BP92	Reticulocalbin-2 OS=Mus musculus GN=Rcn2 PE=2 SV=1 - [RCN2_MOUSE]	0.26	0.17	0.21	0.24	0.00
Q9CQ26	STAM-binding protein OS=Mus musculus GN=Stambp PE=2 SV=1 - [STABP_MOUSE]	0.45	0.38	0.43	0.58	0.00
Q3UFS4	G patch domain-containing protein 11 OS=Mus musculus GN=Gpatch11 PE=2 SV=2 - [GPT11_MOUSE]	0.74	0.89	1.00	1.14	0.00
Q9CPU4	Microsomal glutathione S-transferase 3 OS=Mus musculus GN=Mgst3 PE=1 SV=1 - [MGST3_MOUSE]	-0.97	-0.67	-0.96	-0.74	0.00
P62245	40S ribosomal protein S15a OS=Mus musculus GN=Rps15a PE=2 SV=2 - [RS15A_MOUSE]	-0.51	-0.38	-0.42	-0.33	0.00
O09174	Alpha-methylacyl-CoA racemase OS=Mus musculus GN=Amacr PE=1 SV=4 - [AMACR_MOUSE]	0.65	0.45	0.70	0.60	0.00
Q01853	Transitional endoplasmic reticulum ATPase OS=Mus musculus GN=Vcp PE=1 SV=4 - [TERA_MOUSE]	0.34	0.31	0.46	0.41	0.00
Q810V0	U3 small nucleolar ribonucleoprotein protein MPP10 OS=Mus musculus GN=Mphosph10 PE=1 SV=2 - [MPP10_MOUSE]	0.34	0.41	0.46	0.52	0.00
Q99LC2	Cleavage stimulation factor subunit 1 OS=Mus musculus GN=Cstf1 PE=2 SV=1 - [CSTF1_MOUSE]	0.50	0.69	0.68	0.80	0.00
Q9D8B6	Protein FAM210B OS=Mus musculus GN=Fam210b PE=2 SV=3 - [F210B_MOUSE]	-0.78	-0.73	-0.58	-0.53	0.00
A2A6J4	Lymphocyte-specific protein 1 OS=Mus musculus GN=Lsp1 PE=2 SV=1 - [A2A6J4_MOUSE]	0.34	0.53	0.43	0.48	0.00

P12382	6-phosphofructokinase, liver type OS=Mus musculus GN=Pfkl PE=1 SV=4 - [K6PL_MOUSE]	0.19	0.17	0.12	0.18	0.00
P26638	Serine--tRNA ligase, cytoplasmic OS=Mus musculus GN=Sars PE=2 SV=3 - [SYSC_MOUSE]	-0.51	-0.42	-0.52	-0.34	0.00
Q8BK48	Carboxylesterase 5, isoform CRA_b OS=Mus musculus GN=Ces2e PE=2 SV=1 - [Q8BK48_MOUSE]	0.44	0.54	0.61	0.69	0.00
Q61263	Sterol O-acyltransferase 1 OS=Mus musculus GN=Soat1 PE=2 SV=2 - [SOAT1_MOUSE]	-0.55	-0.54	-0.39	-0.40	0.00
Q80X60	EF-hand calcium-binding domain-containing protein 3 OS=Mus musculus GN=Efcab3 PE=2 SV=1 - [EFCB3_MOUSE]	2.72	2.02	2.53	1.83	0.00
E9QKZ2	Importin-9 OS=Mus musculus GN=Ipo9 PE=2 SV=1 - [E9QKZ2_MOUSE]	-0.47	-0.53	-0.67	-0.69	0.00
Q9JKN1	Zinc transporter 7 OS=Mus musculus GN=Slc30a7 PE=1 SV=1 - [ZNT7_MOUSE]	-0.35	-0.41	-0.48	-0.31	0.00
B2RQG2	PHD finger protein 3 OS=Mus musculus GN=Phf3 PE=2 SV=1 - [B2RQG2_MOUSE]	0.71	0.66	0.59	0.45	0.00
Q8BG46	Ankyrin repeat domain-containing protein 10 OS=Mus musculus GN=Ankrd10 PE=2 SV=1 - [Q8BG46_MOUSE]	-0.48	-0.64	-0.42	-0.58	0.00
Q9CYA6	Zinc finger CCHC domain-containing protein 8 OS=Mus musculus GN=Zcchc8 PE=2 SV=3 - [ZCHC8_MOUSE]	0.50	0.32	0.46	0.47	0.00
H3BIX4	Neuroplastin (Fragment) OS=Mus musculus GN=Nptn PE=4 SV=1 - [H3BIX4_MOUSE]	-0.19	-0.24	-0.15	-0.18	0.00
E9Q2C0	Protein 9330101J02Rik (Fragment) OS=Mus musculus GN=9330101J02Rik PE=2 SV=1 - [E9Q2C0_MOUSE]	1.01	0.88	1.34	1.20	0.00
D3Z479	Sorting nexin-15 OS=Mus musculus GN=Snx15 PE=2 SV=1 - [D3Z479_MOUSE]	0.54	0.51	0.40	0.37	0.00
Q8K377	Leucine-rich repeat transmembrane neuronal protein 1 OS=Mus musculus GN=Lrrtm1 PE=2 SV=1 - [LRRT1_MOUSE]	-1.10	-0.92	-0.89	-0.69	0.00
O35704	Serine palmitoyltransferase 1 OS=Mus musculus GN=Sptlc1 PE=2 SV=2 - [SPTC1_MOUSE]	-0.24	-0.27	-0.25	-0.17	0.00
Q61425	Hydroxyacyl-coenzyme A dehydrogenase, mitochondrial OS=Mus musculus GN=Hadh PE=1 SV=2 - [HCDH_MOUSE]	-0.14	-0.21	-0.16	-0.21	0.00
Q8BHB9	Chloride intracellular channel protein 6 OS=Mus musculus GN=Clic6 PE=2 SV=1 - [CLIC6_MOUSE]	-0.89	-1.08	-1.28	-1.37	0.00
P08122	Collagen alpha-2(IV) chain OS=Mus musculus GN=Col4a2 PE=2 SV=4 - [CO4A2_MOUSE]	0.31	0.26	0.31	0.40	0.00

Q922Y1	UBX domain-containing protein 1 OS=Mus musculus GN=Ubxn1 PE=1 SV=1 - [UBXN1_MOUSE]	0.28	0.32	0.38	0.42	0.00
E9PUL3	Calcium-activated chloride channel regulator 4 OS=Mus musculus GN=Clca4 PE=4 SV=1 - [E9PUL3_MOUSE]	-0.36	-0.52	-0.49	-0.56	0.00
Q6VN19	Ran-binding protein 10 OS=Mus musculus GN=Ranbp10 PE=1 SV=2 - [RBP10_MOUSE]	1.02	0.80	1.28	1.05	0.00
Q8VED2	Biogenesis of lysosome-related organelles complex 1 subunit 4 OS=Mus musculus GN=Bloc1s4 PE=1 SV=1 - [BL1S4_MOUSE]	-0.40	-0.40	-0.29	-0.29	0.00
A2AWA9	Rab GTPase-activating protein 1 OS=Mus musculus GN=Rabgap1 PE=2 SV=1 - [RBGP1_MOUSE]	-0.49	-0.44	-0.66	-0.62	0.00
Q8CB77	Transcription elongation factor B polypeptide 3 OS=Mus musculus GN=Tceb3 PE=1 SV=3 - [ELOA1_MOUSE]	0.59	0.52	0.62	0.40	0.00
Q8CIN4	Serine/threonine-protein kinase PAK 2 OS=Mus musculus GN=Pak2 PE=1 SV=1 - [PAK2_MOUSE]	0.36	0.29	0.30	0.22	0.00
P50429	Arylsulfatase B OS=Mus musculus GN=Arsb PE=2 SV=3 - [ARSB_MOUSE]	0.77	0.69	0.52	0.56	0.00
Q9JJ00	Phospholipid scramblase 1 OS=Mus musculus GN=Plscr1 PE=2 SV=1 - [PLS1_MOUSE]	0.39	0.58	0.49	0.59	0.00
H7BXB9	Protein 1700037H04Rik (Fragment) OS=Mus musculus GN=1700037H04Rik PE=2 SV=1 - [H7BXB9_MOUSE]	0.41	0.58	0.39	0.49	0.00
Q99N17	Cytochrome P450 CYP4F16 OS=Mus musculus GN=Cyp4f16 PE=2 SV=1 - [Q99N17_MOUSE]	-0.56	-0.44	-0.70	-0.58	0.00
P35486	Pyruvate dehydrogenase E1 component subunit alpha, somatic form, mitochondrial OS=Mus musculus GN=Pdha1 PE=1 SV=1 - [ODPA_MOUSE]	-0.34	-0.24	-0.24	-0.24	0.00
Q69ZN7	Myoferlin OS=Mus musculus GN=Myof PE=1 SV=2 - [MYOF_MOUSE]	-0.30	-0.30	-0.23	-0.21	0.00
Q00898	Alpha-1-antitrypsin 1-5 OS=Mus musculus GN=Serpina1e PE=1 SV=1 - [A1AT5_MOUSE]	1.64	2.06	1.34	1.96	0.00
A2AER8	Polyglutamine-binding protein 1 (Fragment) OS=Mus musculus GN=Pqbp1 PE=2 SV=1 - [A2AER8_MOUSE]	0.36	0.42	0.53	0.37	0.00
P01887	Beta-2-microglobulin OS=Mus musculus GN=B2m PE=1 SV=2 - [B2MG_MOUSE]	0.59	0.47	0.68	0.46	0.00
P01872	Ig mu chain C region secreted form OS=Mus musculus GN=Igh-6 PE=1 SV=2 - [IGHM_MOUSE]	-0.46	-0.30	-0.35	-0.35	0.00
Q8CHT0	Delta-1-pyrroline-5-carboxylate dehydrogenase, mitochondrial OS=Mus musculus GN=Aldh4a1 PE=1 SV=3 - [AL4A1_MOUSE]	-0.26	-0.29	-0.19	-0.21	0.00

Q9ESU7	Neutral amino acid transporter ASCT2 OS=Mus musculus GN=Slc1a5 PE=2 SV=1 - [Q9ESU7_MOUSE]	-0.39	-0.44	-0.60	-0.45	0.00
Q80X82	Symplekin OS=Mus musculus GN=Sympk PE=1 SV=1 - [SYMPK_MOUSE]	0.59	0.67	0.43	0.50	0.00
P58871	182 kDa tankyrase-1-binding protein OS=Mus musculus GN=Tnks1bp1 PE=1 SV=2 - [TB182_MOUSE]	0.47	0.39	0.41	0.29	0.00
Q8BT07	Centrosomal protein of 55 kDa OS=Mus musculus GN=Cep55 PE=1 SV=2 - [CEP55_MOUSE]	0.51	0.69	0.47	0.65	0.00
A8WHU2	Coiled-coil domain-containing protein 30 (Fragment) OS=Mus musculus GN=Ccdc30 PE=2 SV=1 - [A8WHU2_MOUSE]	-0.40	-0.41	-0.28	-0.30	0.00
Q8R1N4-2	Isoform 2 of NudC domain-containing protein 3 OS=Mus musculus GN=Nudcd3 - [NUDC3_MOUSE]	0.26	0.25	0.37	0.32	0.00
P24549	Retinal dehydrogenase 1 OS=Mus musculus GN=Aldh1a1 PE=1 SV=5 - [AL1A1_MOUSE]	0.39	0.44	0.59	0.55	0.00
D3YWT0	Signal peptidase complex catalytic subunit SEC11A OS=Mus musculus GN=Sec11a PE=2 SV=1 - [D3YWT0_MOUSE]	-0.67	-0.47	-0.54	-0.45	0.00
P14685	26S proteasome non-ATPase regulatory subunit 3 OS=Mus musculus GN=Psmd3 PE=1 SV=3 - [PSMD3_MOUSE]	-0.30	-0.43	-0.45	-0.33	0.00
Q9D4H8	Cullin-2 OS=Mus musculus GN=Cul2 PE=1 SV=2 - [CUL2_MOUSE]	-0.71	-0.74	-0.47	-0.62	0.00
Q8BGD7	Neuronal PAS domain-containing protein 4 OS=Mus musculus GN=Npas4 PE=1 SV=1 - [NPAS4_MOUSE]	-0.96	-0.83	-1.28	-1.15	0.00
Q8K4F5	Alpha/beta hydrolase domain-containing protein 11 OS=Mus musculus GN=Abhd11 PE=2 SV=1 - [ABHDB_MOUSE]	-0.34	-0.45	-0.35	-0.50	0.00
Q8R422	CD109 antigen OS=Mus musculus GN=Cd109 PE=2 SV=1 - [CD109_MOUSE]	0.33	0.24	0.24	0.22	0.00
Q9D903	Probable rRNA-processing protein EBP2 OS=Mus musculus GN=Ebna1bp2 PE=2 SV=1 - [EBP2_MOUSE]	0.74	0.64	0.58	0.47	0.00
O70200	Allograft inflammatory factor 1 OS=Mus musculus GN=Aif1 PE=1 SV=1 - [AIF1_MOUSE]	0.74	0.58	0.91	0.84	0.00
P62965	Cellular retinoic acid-binding protein 1 OS=Mus musculus GN=Crabp1 PE=1 SV=2 - [RABP1_MOUSE]	0.81	1.17	1.05	1.30	0.00
P22005	Proenkephalin-A OS=Mus musculus GN=Penk PE=2 SV=2 - [PENK_MOUSE]	0.71	0.65	0.97	0.91	0.00
D3Z442	Uncharacterized protein OS=Mus musculus GN=Aamdc PE=2 SV=1 - [D3Z442_MOUSE]	0.26	0.35	0.38	0.41	0.00

Q8C547	HEAT repeat-containing protein 5B OS=Mus musculus GN=Heatr5b PE=2 SV=3 - [HTR5B_MOUSE]	-0.34	-0.42	-0.53	-0.40	0.00
Q68FE8-2	Isoform 2 of Zinc finger protein 280D OS=Mus musculus GN=Znf280d - [Z280D_MOUSE]	0.44	0.33	0.52	0.41	0.00
O55029	Coatomer subunit beta' OS=Mus musculus GN=Copb2 PE=2 SV=2 - [COPB2_MOUSE]	0.10	0.10	0.14	0.11	0.00
Q9ET54-4	Isoform 4 of Palladin OS=Mus musculus GN=Palld - [PALLD_MOUSE]	0.15	0.17	0.22	0.16	0.00
Q6PDG5-2	Isoform 2 of SWI/SNF complex subunit SMARCC2 OS=Mus musculus GN=Smarcc2 - [SMRC2_MOUSE]	0.19	0.18	0.25	0.26	0.00
Q99JY0	Trifunctional enzyme subunit beta, mitochondrial OS=Mus musculus GN=Hadhb PE=1 SV=1 - [ECHB_MOUSE]	-0.40	-0.39	-0.27	-0.30	0.00
P17047	Lysosome-associated membrane glycoprotein 2 OS=Mus musculus GN=Lamp2 PE=2 SV=2 - [LAMP2_MOUSE]	-0.46	-0.44	-0.45	-0.29	0.00
Q9Z1M8	Protein Red OS=Mus musculus GN=Ik PE=2 SV=2 - [RED_MOUSE]	0.59	0.53	0.82	0.67	0.00
O08739	AMP deaminase 3 OS=Mus musculus GN=Ampd3 PE=2 SV=2 - [AMPD3_MOUSE]	0.70	0.62	0.53	0.45	0.00
Q9CQV5	28S ribosomal protein S24, mitochondrial OS=Mus musculus GN=Mrps24 PE=2 SV=1 - [RT24_MOUSE]	-0.65	-0.51	-0.54	-0.41	0.00
A2BI14	Coiled-coil domain-containing protein 171 (Fragment) OS=Mus musculus GN=Ccdc171 PE=2 SV=1 - [A2BI14_MOUSE]	0.15	0.14	0.19	0.21	0.00
Q8C754	Vacuolar protein sorting-associated protein 52 homolog OS=Mus musculus GN=Vps52 PE=2 SV=1 - [VPS52_MOUSE]	-0.41	-0.31	-0.42	-0.50	0.00
E9Q2B0	General transcription factor 3C polypeptide 4 OS=Mus musculus GN=Gtf3c4 PE=2 SV=1 - [E9Q2B0_MOUSE]	-0.54	-0.53	-0.75	-0.75	0.00
Q6PIU9	Uncharacterized protein FLJ45252 homolog OS=Mus musculus PE=1 SV=2 - [YJ005_MOUSE]	1.15	1.11	0.76	0.89	0.00
E9Q3X0	V-type proton ATPase 16 kDa proteolipid subunit OS=Mus musculus GN=Mvp PE=2 SV=1 - [E9Q3X0_MOUSE]	-0.15	-0.19	-0.14	-0.12	0.00
Q8BR63	Protein FAM177A1 OS=Mus musculus GN=Fam177a1 PE=2 SV=1 - [F177A_MOUSE]	0.27	0.27	0.17	0.24	0.00
Q8JZM2	Proline-rich protein 15-like protein OS=Mus musculus GN=Prr15l PE=2 SV=1 - [PR15L_MOUSE]	0.20	0.23	0.16	0.16	0.00
Q8VE70	Programmed cell death protein 10 OS=Mus musculus GN=Pdcd10 PE=1 SV=1 - [PDC10_MOUSE]	-0.78	-0.57	-0.59	-0.51	0.00

Q923T9-3	Isoform 3 of Calcium/calmodulin-dependent protein kinase type II subunit gamma OS=Mus musculus GN=Camk2g - [KCC2G_MOUSE]	-0.21	-0.19	-0.14	-0.23	0.00
Q91YD3	mRNA-decapping enzyme 1A OS=Mus musculus GN=Dcp1a PE=1 SV=1 - [DCP1A_MOUSE]	0.46	0.65	0.50	0.69	0.00
Q99PG2	Opioid growth factor receptor OS=Mus musculus GN=Ogfr PE=2 SV=1 - [OGFR_MOUSE]	0.54	0.45	0.71	0.56	0.00
Q00422	GA-binding protein alpha chain OS=Mus musculus GN=Gabpa PE=1 SV=2 - [GABPA_MOUSE]	0.67	0.78	0.91	1.04	0.00
O08917	Flotillin-1 OS=Mus musculus GN=Flot1 PE=1 SV=1 - [FLOT1_MOUSE]	0.29	0.31	0.20	0.24	0.00
Q8R2Y8	Peptidyl-tRNA hydrolase 2, mitochondrial OS=Mus musculus GN=Ptrh2 PE=2 SV=1 - [PTH2_MOUSE]	-0.35	-0.25	-0.40	-0.33	0.00
Q505F1-2	Isoform 2 of Nuclear receptor subfamily 2 group C member 1 OS=Mus musculus GN=Nr2c1 - [NR2C1_MOUSE]	0.65	0.83	0.52	0.70	0.00
Q99P58	Ras-related protein Rab-27B OS=Mus musculus GN=Rab27b PE=1 SV=3 - [RB27B_MOUSE]	-0.61	-0.48	-0.38	-0.53	0.00
Q8K1M3	Protein kinase, cAMP dependent regulatory, type II alpha OS=Mus musculus GN=Prkar2a PE=2 SV=1 - [Q8K1M3_MOUSE]	-0.13	-0.20	-0.15	-0.17	0.00
Q9D2M8	Ubiquitin-conjugating enzyme E2 variant 2 OS=Mus musculus GN=Ube2v2 PE=2 SV=4 - [UB2V2_MOUSE]	-0.64	-0.68	-0.45	-0.50	0.00
Q61024	Asparagine synthetase [glutamine-hydrolyzing] OS=Mus musculus GN=Asns PE=2 SV=3 - [ASNS_MOUSE]	0.33	0.38	0.51	0.46	0.00
L7N466	Protein Gm2382 OS=Mus musculus GN=Gm2382 PE=4 SV=1 - [L7N466_MOUSE]	-0.25	-0.39	-0.30	-0.37	0.00
F7CZ64	Voltage-dependent calcium channel gamma-8 subunit OS=Mus musculus GN=Cacng8 PE=4 SV=2 - [F7CZ64_MOUSE]	1.05	1.34	0.83	1.12	0.00
Q3UUI3-2	Isoform 2 of Acyl-coenzyme A thioesterase THEM4 OS=Mus musculus GN=Them4 - [THEM4_MOUSE]	-0.53	-0.72	-0.47	-0.66	0.00
Q08857	Platelet glycoprotein 4 OS=Mus musculus GN=Cd36 PE=1 SV=2 - [CD36_MOUSE]	0.75	0.55	0.90	0.82	0.00
Q61792	LIM and SH3 domain protein 1 OS=Mus musculus GN=Lasp1 PE=1 SV=1 - [LASP1_MOUSE]	0.45	0.70	0.52	0.64	0.00
P63254	Cysteine-rich protein 1 OS=Mus musculus GN=Crip1 PE=2 SV=2 - [CRIP1_MOUSE]	-0.69	-0.83	-0.56	-0.88	0.00
Q8C7R4	Ubiquitin-like modifier-activating enzyme 6 OS=Mus musculus GN=Uba6 PE=1 SV=1 - [UBA6_MOUSE]	-0.39	-0.27	-0.45	-0.41	0.00

Q3UYV9	Nuclear cap-binding protein subunit 1 OS=Mus musculus GN=Ncbp1 PE=1 SV=2 - [NCBP1_MOUSE]	-0.42	-0.39	-0.59	-0.53	0.00
E9PZJ8	Activating signal cointegrator 1 complex subunit 3 OS=Mus musculus GN=Ascc3 PE=2 SV=1 - [ASCC3_MOUSE]	-0.25	-0.34	-0.33	-0.40	0.00
E9PV12	Cullin-5 OS=Mus musculus GN=Cul5 PE=2 SV=1 - [E9PV12_MOUSE]	-0.26	-0.38	-0.26	-0.28	0.00
B2RW07	Ataxin 7-like 1 OS=Mus musculus GN=Atxn7l1 PE=2 SV=1 - [B2RW07_MOUSE]	-0.63	-0.68	-0.45	-0.50	0.00
Q3UEG6	Alanine--glyoxylate aminotransferase 2, mitochondrial OS=Mus musculus GN=Agxt2 PE=2 SV=1 - [AGT2_MOUSE]	-0.42	-0.48	-0.30	-0.36	0.00
P11438	Lysosome-associated membrane glycoprotein 1 OS=Mus musculus GN=Lamp1 PE=1 SV=2 - [LAMP1_MOUSE]	-0.26	-0.22	-0.18	-0.17	0.00
F6QL70	Protein Gm17669 OS=Mus musculus GN=Gm17669 PE=4 SV=1 - [F6QL70_MOUSE]	0.62	0.56	0.81	0.83	0.00
F7CFC7	Protein 2010015L04Rik (Fragment) OS=Mus musculus GN=2010015L04Rik PE=2 SV=1 - [F7CFC7_MOUSE]	0.68	0.63	0.94	0.89	0.00
Q62419	Endophilin-A2 OS=Mus musculus GN=Sh3gl1 PE=1 SV=1 - [SH3G1_MOUSE]	0.30	0.39	0.45	0.47	0.00
Q9D061	Acyl-CoA-binding domain-containing protein 6 OS=Mus musculus GN=Acbd6 PE=1 SV=2 - [ACBD6_MOUSE]	0.37	0.37	0.26	0.26	0.00
Q8BGT9	Polypeptide N-acetylgalactosaminyltransferase 12 OS=Mus musculus GN=Galnt12 PE=2 SV=1 - [GLT12_MOUSE]	0.28	0.31	0.19	0.25	0.00
Q8K2C9	Very-long-chain (3R)-3-hydroxyacyl-[acyl-carrier protein] dehydratase 3 OS=Mus musculus GN=ptplad1 PE=1 SV=2 - [HACD3_MOUSE]	-0.75	-0.86	-0.53	-0.66	0.00
P15092-2	Isoform 2 of Interferon-activable protein 204 OS=Mus musculus GN=Ifi204 - [IFI4_MOUSE]	0.46	0.70	0.51	0.66	0.00
H7BWZ3	Actin-related protein 2/3 complex subunit 3 OS=Mus musculus GN=Arpc3 PE=2 SV=1 - [H7BWZ3_MOUSE]	-0.65	-0.55	-0.50	-0.40	0.00
P14602-2	Isoform B of Heat shock protein beta-1 OS=Mus musculus GN=Hspb1 - [HSPB1_MOUSE]	-0.32	-0.44	-0.28	-0.38	0.00
P97384	Annexin A11 OS=Mus musculus GN=Anxa11 PE=1 SV=2 - [ANX11_MOUSE]	-0.38	-0.36	-0.33	-0.24	0.00
Q9EPL8	Importin-7 OS=Mus musculus GN=Ipo7 PE=1 SV=2 - [IPO7_MOUSE]	-0.74	-0.56	-0.91	-0.72	0.00
P70670	Nascent polypeptide-associated complex subunit alpha, muscle-specific form OS=Mus musculus GN=Naca PE=1 SV=2 - [NACAM_MOUSE]	0.30	0.25	0.37	0.25	0.00

Q80WG5	Leucine-rich repeat-containing protein 8A OS=Mus musculus GN=Lrrc8a PE=1 SV=1 - [LRC8A_MOUSE]	-0.36	-0.27	-0.31	-0.23	0.00
Q8BX70-3	Isoform 3 of Vacuolar protein sorting-associated protein 13C OS=Mus musculus GN=Vps13c - [VP13C_MOUSE]	-0.19	-0.29	-0.25	-0.30	0.00
Q8CH25-2	Isoform 2 of SAFB-like transcription modulator OS=Mus musculus GN=Sltm - [SLTM_MOUSE]	0.60	0.53	0.41	0.41	0.00
Q8VHF2-2	Isoform 2 of Cadherin-related family member 5 OS=Mus musculus GN=Cdhr5 - [CDHR5_MOUSE]	-0.36	-0.26	-0.24	-0.32	0.00
P00920	Carbonic anhydrase 2 OS=Mus musculus GN=Ca2 PE=1 SV=4 - [CAH2_MOUSE]	-0.68	-0.89	-0.55	-0.76	0.00
Q5SF07	Insulin-like growth factor 2 mRNA-binding protein 2 OS=Mus musculus GN=Igf2bp2 PE=1 SV=1 - [IF2B2_MOUSE]	0.50	0.79	0.58	0.68	0.00
O09012-2	Isoform 2 of Peroxisomal targeting signal 1 receptor OS=Mus musculus GN=Pex5 - [PEX5_MOUSE]	0.64	0.64	0.61	0.40	0.00
O70326	Gremlin-1 OS=Mus musculus GN=Grem1 PE=2 SV=1 - [GREM1_MOUSE]	0.25	0.21	0.33	0.30	0.00
P16460	Argininosuccinate synthase OS=Mus musculus GN=Ass1 PE=1 SV=1 - [ASSY_MOUSE]	-0.50	-0.56	-0.35	-0.44	0.00
P14438	H-2 class II histocompatibility antigen, A-U alpha chain (Fragment) OS=Mus musculus GN=H2-Aa PE=1 SV=1 [HA2U_MOUSE]	-0.25	-0.23	-0.35	-0.27	0.00
Q9QYG0	Protein NDRG2 OS=Mus musculus GN=Ndrg2 PE=1 SV=1 - [NDRG2_MOUSE]	-0.43	-0.46	-0.66	-0.58	0.00
Q04857	Collagen alpha-1(VI) chain OS=Mus musculus GN=Col6a1 PE=2 SV=1 - [CO6A1_MOUSE]	-0.22	-0.18	-0.27	-0.19	0.00
P45376	Aldose reductase OS=Mus musculus GN=Akr1b1 PE=1 SV=3 - [ALDR_MOUSE]	-0.29	-0.22	-0.19	-0.20	0.00
Q9DBL1	Short/branched chain specific acyl-CoA dehydrogenase, mitochondrial OS=Mus musculus GN=Acadsb PE=1 SV=1 - [ACDSB_MOUSE]	-0.49	-0.37	-0.55	-0.37	0.00
Q3TML0	Protein disulfide-isomerase A6 OS=Mus musculus GN=Pdia6 PE=2 SV=1 - [Q3TML0_MOUSE]	-0.37	-0.24	-0.30	-0.25	0.00
D3Z4E3	DEAH (Asp-Glu-Ala-His) box polypeptide 32, isoform CRA_b OS=Mus musculus GN=Dhx32 PE=4 SV=1 - [D3Z4E3_MOUSE]	-0.18	-0.28	-0.26	-0.29	0.00
P10649	Glutathione S-transferase Mu 1 OS=Mus musculus GN=Gstm1 PE=1 SV=2 - [GSTM1_MOUSE]	-0.34	-0.40	-0.26	-0.28	0.00
Q6ZQA6	Immunoglobulin superfamily member 3 OS=Mus musculus GN=Igsf3 PE=1 SV=2 - [IGSF3_MOUSE]	-0.41	-0.52	-0.57	-0.67	0.00

Q8BGD9	Eukaryotic translation initiation factor 4B OS=Mus musculus GN=Eif4b PE=1 SV=1 - [IF4B_MOUSE]	0.47	0.29	0.40	0.35	0.00
Q9R059	Four and a half LIM domains protein 3 OS=Mus musculus GN=Fhl3 PE=2 SV=2 - [FHL3_MOUSE]	0.34	0.34	0.21	0.32	0.00
Q9Z2E1	Methyl-CpG-binding domain protein 2 OS=Mus musculus GN=Mbd2 PE=2 SV=2 - [MBD2_MOUSE]	0.40	0.30	0.34	0.25	0.00
A2A998	Complement component C8 alpha chain OS=Mus musculus GN=C8a PE=2 SV=1 - [A2A998_MOUSE]	0.42	0.29	0.43	0.48	0.00
Q9QXL2-3	Isoform 3 of Kinesin-like protein KIF21A OS=Mus musculus GN=Kif21a - [KI21A_MOUSE]	-0.34	-0.48	-0.34	-0.48	0.00
E9Q7H5	Uncharacterized protein OS=Mus musculus GN=Gm8991 PE=4 SV=1 - [E9Q7H5_MOUSE]	0.56	0.37	0.58	0.45	0.00
O88792	Junctional adhesion molecule A OS=Mus musculus GN=F11r PE=1 SV=2 - [JAM1_MOUSE]	-0.44	-0.36	-0.30	-0.28	0.00
Q8BKE6	Cytochrome P450 20A1 OS=Mus musculus GN=Cyp20a1 PE=2 SV=1 - [CP20A_MOUSE]	-0.72	-0.48	-0.65	-0.50	0.00
Q8CIH9	Amidophosphoribosyltransferase OS=Mus musculus GN=Ppat PE=2 SV=1 - [Q8CIH9_MOUSE]	-0.84	-1.08	-0.68	-0.80	0.00
Q9DCD0	6-phosphogluconate dehydrogenase, decarboxylating OS=Mus musculus GN=Pgd PE=2 SV=3 - [6PGD_MOUSE]	0.20	0.24	0.29	0.31	0.00
O70433	Four and a half LIM domains protein 2 OS=Mus musculus GN=Fhl2 PE=1 SV=1 - [FHL2_MOUSE]	0.38	0.41	0.25	0.33	0.00
Q3UTJ2	Sorbin and SH3 domain-containing protein 2 OS=Mus musculus GN=Sorbs2 PE=1 SV=2 - [SRBS2_MOUSE]	1.01	0.73	1.11	0.78	0.00
Q923D5	WW domain-binding protein 11 OS=Mus musculus GN=Wbp11 PE=1 SV=2 - [WBP11_MOUSE]	0.67	0.56	0.80	0.51	0.00
Q91X84	CREB-regulated transcription coactivator 3 OS=Mus musculus GN=Crtc3 PE=2 SV=2 - [CRTC3_MOUSE]	0.74	1.07	0.80	1.12	0.00
Q8K003	Translation machinery-associated protein 7 OS=Mus musculus GN=Tma7 PE=2 SV=1 - [TMA7_MOUSE]	0.53	0.83	0.84	0.84	0.00
P67778	Prohibitin OS=Mus musculus GN=Phb PE=1 SV=1 - [PHB_MOUSE]	-0.27	-0.36	-0.26	-0.39	0.00
Q8BH00	Aldehyde dehydrogenase family 8 member A1 OS=Mus musculus GN=Aldh8a1 PE=1 SV=1 - [AL8A1_MOUSE]	-0.24	-0.20	-0.32	-0.28	0.00
Q9QY40	Plexin-B3 OS=Mus musculus GN=Plxnb3 PE=1 SV=2 - [PLXB3_MOUSE]	-1.78	-1.32	-1.57	-1.11	0.00

A2AQE4	COP9 signalosome complex subunit 2 OS=Mus musculus GN=Cops2 PE=2 SV=1 - [A2AQE4_MOUSE]	-0.51	-0.46	-0.39	-0.32	0.00
Q811P8-2	Isoform 2 of Rho GTPase-activating protein 32 OS=Mus musculus GN=Arhgap32 - [RHG32_MOUSE]	0.56	0.71	0.44	0.53	0.00
Q505D7	Optic atrophy 3 protein homolog OS=Mus musculus GN=Opa3 PE=1 SV=1 - [OPA3_MOUSE]	-0.61	-0.45	-0.42	-0.41	0.00
Q91ZU6-8	Isoform 7 of Dystonin OS=Mus musculus GN=Dst - [DYST_MOUSE]	0.28	0.24	0.38	0.35	0.00
P61082	NEDD8-conjugating enzyme Ubc12 OS=Mus musculus GN=Ube2m PE=2 SV=1 - [UBC12_MOUSE]	-0.22	-0.18	-0.26	-0.29	0.00
Q6PHU5	Sortilin OS=Mus musculus GN=Sort1 PE=1 SV=1 - [SORT_MOUSE]	-0.38	-0.39	-0.57	-0.46	0.00
P32883-2	Isoform 2B of GTPase KRas OS=Mus musculus GN=Kras - [RASK_MOUSE]	-0.39	-0.46	-0.28	-0.37	0.00
Q9Z2V4	Phosphoenolpyruvate carboxykinase, cytosolic [GTP] OS=Mus musculus GN=Pck1 PE=2 SV=1 - [PCKGC_MOUSE]	-0.47	-0.41	-0.55	-0.34	0.00
Q9QYB5-2	Isoform 1 of Gamma-adducin OS=Mus musculus GN=Add3 - [ADDG_MOUSE]	0.18	0.17	0.11	0.15	0.00
Q71FD5	E3 ubiquitin-protein ligase ZNRF2 OS=Mus musculus GN=Znrf2 PE=1 SV=1 - [ZNRF2_MOUSE]	0.58	0.62	0.83	0.87	0.00
A2ACP1-2	Isoform 2 of Tetratricopeptide repeat protein 39A OS=Mus musculus GN=Ttc39a - [TT39A_MOUSE]	-1.20	-0.82	-1.24	-0.91	0.00
G3UW34	MCG130981 OS=Mus musculus GN=Rpl9-ps6 PE=4 SV=1 - [G3UW34_MOUSE]	-0.44	-0.39	-0.57	-0.38	0.00
Q9CQR2	40S ribosomal protein S21 OS=Mus musculus GN=Rps21 PE=2 SV=1 - [RS21_MOUSE]	0.43	0.43	0.62	0.59	0.00
P35979	60S ribosomal protein L12 OS=Mus musculus GN=Rpl12 PE=1 SV=2 - [RL12_MOUSE]	-0.33	-0.26	-0.39	-0.41	0.00
O35857	Mitochondrial import inner membrane translocase subunit TIM44 OS=Mus musculus GN=Timm44 PE=2 SV=2 - [TIM44_MOUSE]	-0.32	-0.30	-0.20	-0.31	0.00
Q9CRB9	Coiled-coil-helix-coiled-coil-helix domain-containing protein 3, mitochondrial OS=Mus musculus GN=Chchd3 PE=1 SV=1 - [CHCH3_MOUSE]	-0.64	-0.58	-0.43	-0.44	0.00
Q60737	Casein kinase II subunit alpha OS=Mus musculus GN=Csnk2a1 PE=1 SV=2 - [CSK21_MOUSE]	0.27	0.39	0.31	0.25	0.00
G5E866	Splicing factor 3B subunit 1 OS=Mus musculus GN=Sf3b1 PE=4 SV=1 - [G5E866_MOUSE]	0.41	0.44	0.27	0.41	0.00

Q62241	U1 small nuclear ribonucleoprotein C OS=Mus musculus GN=Snrpc PE=2 SV=1 - [RU1C_MOUSE]	0.76	0.67	1.01	1.01	0.00
E0CXN5	Glycerol-3-phosphate dehydrogenase [NAD(+)], cytoplasmic OS=Mus musculus GN=Gpd1 PE=2 SV=1 [E0CXN5_MOUSE]	0.10	0.11	0.11	0.16	0.00
D3Z6P0-2	Isoform 2 of Protein disulfide-isomerase A2 OS=Mus musculus GN=Pdia2 - [PDIA2_MOUSE]	0.31	0.49	0.50	0.47	0.00
P29341	Polyadenylate-binding protein 1 OS=Mus musculus GN=Pabpc1 PE=1 SV=2 - [PABP1_MOUSE]	0.40	0.33	0.53	0.48	0.00
Q80XL6	Acyl-CoA dehydrogenase family member 11 OS=Mus musculus GN=Acad11 PE=1 SV=2 - [ACD11_MOUSE]	-0.95	-0.77	-1.24	-0.91	0.00
Q9D309	Protein FAM3B OS=Mus musculus GN=Fam3b PE=1 SV=1 - [FAM3B_MOUSE]	0.45	0.35	0.58	0.45	0.00
Q91YR7	Pre-mRNA-processing factor 6 OS=Mus musculus GN=Prpf6 PE=2 SV=1 - [PRP6_MOUSE]	0.12	0.17	0.12	0.12	0.00
Q8VCE1	DnaJ homolog subfamily C member 28 OS=Mus musculus GN=Dnajc28 PE=2 SV=2 - [DJC28_MOUSE]	-0.35	-0.25	-0.40	-0.40	0.00
Q9JKY7	Cytochrome P450 CYP2D22 OS=Mus musculus GN=Cyp2d22 PE=2 SV=1 - [Q9JKY7_MOUSE]	-0.56	-0.41	-0.50	-0.35	0.00
Q8K212	Phosphofurin acidic cluster sorting protein 1 OS=Mus musculus GN=Pacs1 PE=1 SV=2 - [PACS1_MOUSE]	0.39	0.29	0.44	0.30	0.00
Q8CHP5-2	Isoform 2 of Partner of Y14 and mago OS=Mus musculus GN=Wibg - [WIBG_MOUSE]	0.23	0.32	0.27	0.37	0.00
O70570	Polymeric immunoglobulin receptor OS=Mus musculus GN=Pigr PE=1 SV=1 - [PIGR_MOUSE]	-0.13	-0.14	-0.09	-0.14	0.00
Q99PT1	Rho GDP-dissociation inhibitor 1 OS=Mus musculus GN=Arhgdia PE=1 SV=3 - [GDIR1_MOUSE]	-0.48	-0.46	-0.34	-0.32	0.00
Q8BH15-2	Isoform 2 of CCR4-NOT transcription complex subunit 10 OS=Mus musculus GN=Cnot10 - [CNO10_MOUSE]	0.19	0.13	0.19	0.14	0.00
P28653	Biglycan OS=Mus musculus GN=Bgn PE=2 SV=1 - [PGS1_MOUSE]	-0.20	-0.31	-0.26	-0.32	0.00
E9QLU4	Tetratricopeptide repeat protein 29 OS=Mus musculus GN=Ttc29 PE=4 SV=1 - [E9QLU4_MOUSE]	0.60	0.85	0.58	0.83	0.00
Q4VA53	Sister chromatid cohesion protein PDS5 homolog B OS=Mus musculus GN=Pds5b PE=1 SV=1 - [PDS5B_MOUSE]	-0.45	-0.61	-0.38	-0.54	0.00
Q8BWZ3	N-alpha-acetyltransferase 25, NatB auxiliary subunit OS=Mus musculus GN=Naa25 PE=1 SV=1 - [NAA25_MOUSE]	-0.29	-0.27	-0.38	-0.24	0.00

Q9CYL5	Golgi-associated plant pathogenesis-related protein 1 OS=Mus musculus GN=Glipr2 PE=2 SV=3 - [GAPR1_MOUSE]	0.72	0.60	0.70	0.44	0.00
Q8VCX5	Calcium uptake protein 1, mitochondrial OS=Mus musculus GN=Micu1 PE=1 SV=1 - [MICU1_MOUSE]	-0.45	-0.60	-0.38	-0.54	0.00
Q80TP3	E3 ubiquitin-protein ligase UBR5 OS=Mus musculus GN=Ubr5 PE=1 SV=2 - [UBR5_MOUSE]	0.39	0.28	0.46	0.41	0.00
O35954	Membrane-associated phosphatidylinositol transfer protein 1 OS=Mus musculus GN=Pitpnm1 PE=1 SV=1 - [PITM1_MOUSE]	1.01	0.71	1.15	0.85	0.00
Q62376	U1 small nuclear ribonucleoprotein 70 kDa OS=Mus musculus GN=Snrnp70 PE=1 SV=2 - [RU17_MOUSE]	0.36	0.32	0.50	0.45	0.00
J3QN23	DNA repair and recombination protein RAD54B (Fragment) OS=Mus musculus GN=Rad54b PE=4 SV=1 - [J3QN23_MOUSE]	0.47	0.44	0.67	0.63	0.00
Q61292	Laminin subunit beta-2 OS=Mus musculus GN=Lamb2 PE=2 SV=2 - [LAMB2_MOUSE]	-0.18	-0.14	-0.16	-0.11	0.00
Q6PFX9	Tankyrase-1 OS=Mus musculus GN=Tnks PE=1 SV=1 - [TNKS1_MOUSE]	0.24	0.19	0.31	0.26	0.00
P09405	Nucleolin OS=Mus musculus GN=Ncl PE=1 SV=2 - [NUCL_MOUSE]	0.40	0.41	0.57	0.59	0.00
Q3V0K9	Plastin-1 OS=Mus musculus GN=Pls1 PE=2 SV=1 - [PLSI_MOUSE]	-0.31	-0.31	-0.21	-0.22	0.00
Q9QZ41-2	Isoform 2 of Protein DBF4 homolog A OS=Mus musculus GN=Dbf4 - [DBF4A_MOUSE]	0.40	0.31	0.34	0.24	0.00
Q8C7U7	Polypeptide N-acetylgalactosaminyltransferase 6 OS=Mus musculus GN=Galnt6 PE=2 SV=1 - [GALT6_MOUSE]	0.18	0.11	0.15	0.17	0.00
D3YWJ5	Tumor necrosis factor alpha-induced protein 8 OS=Mus musculus GN=Tnfaip8 PE=2 SV=1 - [D3YWJ5_MOUSE]	-0.50	-0.50	-0.73	-0.70	0.00
Q9JIK5	Nucleolar RNA helicase 2 OS=Mus musculus GN=Ddx21 PE=1 SV=3 - [DDX21_MOUSE]	-0.17	-0.12	-0.17	-0.11	0.00
Q9WVA3	Mitotic checkpoint protein BUB3 OS=Mus musculus GN=Bub3 PE=2 SV=2 - [BUB3_MOUSE]	0.19	0.23	0.28	0.31	0.00
Q9CQJ2	PIH1 domain-containing protein 1 OS=Mus musculus GN=Pih1d1 PE=2 SV=1 - [PIHD1_MOUSE]	0.72	0.52	0.66	0.46	0.00
E9Q3Y4	Lipopolysaccharide-responsive and beige-like anchor protein OS=Mus musculus GN=Lrba PE=2 SV=1 - [E9Q3Y4_MOUSE]	-0.58	-0.39	-0.66	-0.53	0.00
Q62393-3	Isoform 3 of Tumor protein D52 OS=Mus musculus GN=Tpd52 - [TPD52_MOUSE]	0.67	0.67	0.96	0.96	0.00

P35831	Tyrosine-protein phosphatase non-receptor type 12 OS=Mus musculus GN=Ptpn12 PE=1 SV=3 - [PTN12_MOUSE]	0.66	0.50	0.83	0.74	0.00
Q8BUU7	N-acetylgalactosamine kinase OS=Mus musculus GN=Galk2 PE=2 SV=1 - [Q8BUU7_MOUSE]	-0.58	-0.43	-0.40	-0.38	0.00
Q8R274	MCG142205 OS=Mus musculus GN=Vmn1r210 PE=4 SV=1 - [Q8R274_MOUSE]	-0.98	-0.70	-1.16	-0.88	0.00
Q6PEE2	CBP80/20-dependent translation initiation factor OS=Mus musculus GN=Ctif PE=1 SV=2 - [CTIF_MOUSE]	-0.62	-0.59	-0.44	-0.41	0.00
P25799	Nuclear factor NF-kappa-B p105 subunit OS=Mus musculus GN=Nfkb1 PE=1 SV=2 - [NFKB1_MOUSE]	0.41	0.63	0.47	0.63	0.00
Q9JHC0	Glutathione peroxidase 2 OS=Mus musculus GN=Gpx2 PE=2 SV=3 - [GPX2_MOUSE]	-0.19	-0.26	-0.19	-0.29	0.00
Q9WV92-2	Isoform 2 of Band 4.1-like protein 3 OS=Mus musculus GN=Epb41l3 - [E41L3_MOUSE]	0.44	0.38	0.56	0.60	0.00
O08663	Methionine aminopeptidase 2 OS=Mus musculus GN=Metap2 PE=1 SV=1 - [MAP2_MOUSE]	-0.28	-0.26	-0.37	-0.23	0.00
Q8VCI5	Peroxisomal biogenesis factor 19 OS=Mus musculus GN=Pex19 PE=1 SV=1 - [PEX19_MOUSE]	0.44	0.53	0.63	0.72	0.00
Q9R0X2	ADAM DEC1 OS=Mus musculus GN=Adamdec1 PE=2 SV=1 - [ADEC1_MOUSE]	0.55	0.78	0.67	0.90	0.00
D3Z1X8	SWI/SNF complex subunit SMARCC1 OS=Mus musculus GN=Smarcc1 PE=2 SV=1 - [D3Z1X8_MOUSE]	0.64	0.61	0.78	0.47	0.00
Q80SY4	E3 ubiquitin-protein ligase MIB1 OS=Mus musculus GN=Mib1 PE=1 SV=1 - [MIB1_MOUSE]	0.30	0.43	0.28	0.40	0.00
D3YU60	Microsomal glutathione S-transferase 1 OS=Mus musculus GN=Mgst1 PE=2 SV=1 - [D3YU60_MOUSE]	0.31	0.32	0.33	0.46	0.00
Q8BLF1	Neutral cholesterol ester hydrolase 1 OS=Mus musculus GN=Nceh1 PE=1 SV=1 - [NCEH1_MOUSE]	-0.34	-0.33	-0.51	-0.41	0.00
Q9CYR6	Phosphoacetylglucosamine mutase OS=Mus musculus GN=Pgm3 PE=1 SV=1 - [AGM1_MOUSE]	-0.55	-0.70	-0.49	-0.44	0.00
Q80V26	Inositol monophosphatase 3 OS=Mus musculus GN=Impad1 PE=1 SV=1 - [IMPA3_MOUSE]	-0.62	-0.47	-0.73	-0.77	0.00
Q91Z92	Beta-1,3-galactosyltransferase 6 OS=Mus musculus GN=B3galt6 PE=2 SV=1 - [B3GT6_MOUSE]	-0.17	-0.21	-0.23	-0.27	0.00
P97499	Telomerase protein component 1 OS=Mus musculus GN=Tep1 PE=1 SV=1 - [TEP1_MOUSE]	-0.63	-0.49	-0.80	-0.58	0.00

Q3UG37	Histone deacetylase 6 (Fragment) OS=Mus musculus GN=Hdac6 PE=2 SV=1 - [Q3UG37_MOUSE]	0.74	0.48	0.64	0.50	0.00
Q8CAQ8-3	Isoform 3 of Mitochondrial inner membrane protein OS=Mus musculus GN=Immt - [IMMT_MOUSE]	-0.79	-0.62	-0.77	-0.50	0.00
Q8BHH8	Clarin-3 OS=Mus musculus GN=Clrn3 PE=2 SV=1 - [CLRN3_MOUSE]	-1.05	-1.02	-0.73	-0.70	0.00
Q9CQQ8	U6 snRNA-associated Sm-like protein LSm7 OS=Mus musculus GN=Lsm7 PE=3 SV=1 - [LSM7_MOUSE]	0.31	0.35	0.46	0.48	0.00
Q9JKA5	Cell surface A33 antigen OS=Mus musculus GN=Gpa33 PE=2 SV=2 - [GPA33_MOUSE]	-0.88	-1.11	-0.66	-0.88	0.00
E9Q7N5	Ribosome-releasing factor 2, mitochondrial OS=Mus musculus GN=Gfm2 PE=2 SV=1 - [E9Q7N5_MOUSE]	-0.59	-0.54	-0.66	-0.39	0.00
Q80YR5	Scaffold attachment factor B2 OS=Mus musculus GN=Safb2 PE=1 SV=2 - [SAFB2_MOUSE]	0.69	0.52	0.61	0.42	0.00
Q61908	Cx9C motif-containing protein 4 OS=Mus musculus GN=Cmc4 PE=2 SV=1 - [CMC4_MOUSE]	0.41	0.29	0.45	0.33	0.00
P07146	Anionic trypsin-2 OS=Mus musculus GN=Prss2 PE=2 SV=1 - [TRY2_MOUSE]	0.31	0.21	0.34	0.34	0.00
Q6WVG3	BTB/POZ domain-containing protein KCTD12 OS=Mus musculus GN=Kctd12 PE=1 SV=1 - [KCD12_MOUSE]	0.47	0.54	0.37	0.34	0.00
P07091	Protein S100-A4 OS=Mus musculus GN=S100a4 PE=1 SV=1 - [S10A4_MOUSE]	0.27	0.26	0.39	0.37	0.00
Q9DCH4	Eukaryotic translation initiation factor 3 subunit F OS=Mus musculus GN=Eif3f PE=1 SV=2 - [EIF3F_MOUSE]	-0.19	-0.16	-0.14	-0.12	0.00
Q9Z2N8	Actin-like protein 6A OS=Mus musculus GN=Actl6a PE=1 SV=2 - [ACL6A_MOUSE]	0.29	0.31	0.20	0.23	0.00
E9Q1S3	Protein transport protein Sec23A OS=Mus musculus GN=Sec23a PE=2 SV=1 - [E9Q1S3_MOUSE]	0.18	0.14	0.22	0.16	0.00
P14131	40S ribosomal protein S16 OS=Mus musculus GN=Rps16 PE=2 SV=4 - [RS16_MOUSE]	-0.38	-0.48	-0.30	-0.33	0.00
Q9CZY3	Ubiquitin-conjugating enzyme E2 variant 1 OS=Mus musculus GN=Ube2v1 PE=1 SV=1 - [UB2V1_MOUSE]	-0.40	-0.31	-0.36	-0.51	0.00
Q7M759	Alpha/beta hydrolase domain-containing protein 17B OS=Mus musculus GN=Abhd17b PE=2 SV=1 - [AB17B_MOUSE]	-0.24	-0.17	-0.28	-0.22	0.00
Q91YE8	Synaptopodin-2 OS=Mus musculus GN=Synpo2 PE=1 SV=2 - [SYNP2_MOUSE]	-0.39	-0.27	-0.37	-0.47	0.00

P12804	Fibroleukin OS=Mus musculus GN=Fgl2 PE=1 SV=1 - [FGL2_MOUSE]	1.04	0.75	1.26	0.95	0.00
Q9D7E4	UPF0449 protein C19orf25 homolog OS=Mus musculus PE=1 SV=1 - [CS025_MOUSE]	0.51	0.39	0.59	0.38	0.00
P61087	Ubiquitin-conjugating enzyme E2 K OS=Mus musculus GN=Ube2k PE=1 SV=3 - [UBE2K_MOUSE]	-0.07	-0.07	-0.10	-0.06	0.00
Q9D8G5	Regenerating islet-derived protein 4 OS=Mus musculus GN=Reg4 PE=2 SV=1 - [REG4_MOUSE]	0.25	0.24	0.19	0.16	0.00
Q8K1G2	Limbin OS=Mus musculus GN=Evc2 PE=1 SV=1 - [LBN_MOUSE]	-0.46	-0.49	-0.65	-0.71	0.00
Q3TC72	Fumarylacetoacetate hydrolase domain-containing protein 2A OS=Mus musculus GN=Fahd2 PE=1 SV=1 - [FAHD2_MOUSE]	-0.16	-0.26	-0.18	-0.22	0.00
Q9D0I9	Arginine--tRNA ligase, cytoplasmic OS=Mus musculus GN=Rars PE=2 SV=2 - [SYRC_MOUSE]	-0.37	-0.29	-0.33	-0.22	0.00
Q9Z1Q9	Valine--tRNA ligase OS=Mus musculus GN=Vars PE=2 SV=1 - [SYVC_MOUSE]	-0.43	-0.32	-0.33	-0.26	0.00
Q8K4L4	Protein POF1B OS=Mus musculus GN=Pof1b PE=1 SV=3 - [POF1B_MOUSE]	0.72	0.50	0.81	0.59	0.00
Q80W54	CAAX prenyl protease 1 homolog OS=Mus musculus GN=Zmpste24 PE=1 SV=2 - [FACE1_MOUSE]	-0.56	-0.45	-0.41	-0.34	0.00
H3BKC5	U6 snRNA-associated Sm-like protein LSm5 (Fragment) OS=Mus musculus GN=Lsm5 PE=2 SV=1 - [H3BKC5_MOUSE]	-0.32	-0.43	-0.44	-0.55	0.00
Q6W8Q3	Purkinje cell protein 4-like protein 1 OS=Mus musculus GN=Pcp4l1 PE=2 SV=1 - [PC4L1_MOUSE]	0.45	0.50	0.60	0.72	0.00
Q9CZX9	ER membrane protein complex subunit 4 OS=Mus musculus GN=Emc4 PE=2 SV=1 - [EMC4_MOUSE]	-0.14	-0.16	-0.10	-0.11	0.00
P80315	T-complex protein 1 subunit delta OS=Mus musculus GN=Cct4 PE=1 SV=3 - [TCPD_MOUSE]	-0.12	-0.20	-0.15	-0.17	0.00
Q9CQG2	Methyltransferase-like protein 16 OS=Mus musculus GN=Mettl16 PE=1 SV=1 - [MET16_MOUSE]	3.77	3.24	2.80	2.27	0.00
Q5NCU4	SPARC OS=Mus musculus GN=Sparc PE=2 SV=1 - [Q5NCU4_MOUSE]	0.24	0.29	0.21	0.18	0.00
Q9ERD7	Tubulin beta-3 chain OS=Mus musculus GN=Tubb3 PE=1 SV=1 - [TBB3_MOUSE]	-0.36	-0.28	-0.23	-0.24	0.00
P21279	Guanine nucleotide-binding protein G(q) subunit alpha OS=Mus musculus GN=Gnaq PE=1 SV=4 - [GNAQ_MOUSE]	-0.31	-0.27	-0.41	-0.42	0.00

Q8BY79	Solute carrier family 35 member G1 OS=Mus musculus GN=Slc35g1 PE=2 SV=1 - [S35G1_MOUSE]	-0.90	-0.69	-1.17	-0.95	0.00
J3QMM7	ATP-dependent (S)-NAD(P)H-hydrate dehydratase OS=Mus musculus GN=Carkd PE=3 SV=1 - [J3QMM7_MOUSE]	-0.16	-0.18	-0.17	-0.25	0.00
P51827-2	Isoform 2 of AF4/FMR2 family member 3 OS=Mus musculus GN=Aff3 - [AFF3_MOUSE]	0.94	1.03	0.65	0.74	0.00
Q91XZ7	Protein Pcdhb3 OS=Mus musculus GN=Pcdhb3 PE=2 SV=1 - [Q91XZ7_MOUSE]	1.03	0.98	1.48	1.43	0.00
O55013	Trafficking protein particle complex subunit 3 OS=Mus musculus GN=Trappc3 PE=1 SV=1 - [TPPC3_MOUSE]	-0.37	-0.24	-0.40	-0.36	0.00
Q61635	GTP-binding protein OS=Mus musculus GN=Ifi47 PE=2 SV=1 - [Q61635_MOUSE]	0.32	0.31	0.48	0.42	0.00
Q9CW03	Structural maintenance of chromosomes protein 3 OS=Mus musculus GN=Smc3 PE=1 SV=2 - [SMC3_MOUSE]	-0.34	-0.26	-0.37	-0.24	0.00
Q70IV5	Synemin OS=Mus musculus GN=Synm PE=1 SV=2 - [SYNEM_MOUSE]	0.05	0.05	0.03	0.06	0.00
P57016	Ladinin-1 OS=Mus musculus GN=Lad1 PE=1 SV=1 - [LAD1_MOUSE]	0.81	0.72	1.17	0.98	0.00
G3UXZ5	Proteasome activator complex subunit 1 (Fragment) OS=Mus musculus GN=Psme1 PE=2 SV=1 - [G3UXZ5_MOUSE]	-0.60	-0.49	-0.52	-0.35	0.00
A2AQ99	Protein Duox2 OS=Mus musculus GN=Duox2 PE=4 SV=1 - [A2AQ99_MOUSE]	0.47	0.52	0.32	0.38	0.00
P10630	Eukaryotic initiation factor 4A-II OS=Mus musculus GN=Eif4a2 PE=2 SV=2 - [IF4A2_MOUSE]	-0.68	-0.73	-0.44	-0.57	0.00
Q91WQ3	Tyrosine--tRNA ligase, cytoplasmic OS=Mus musculus GN=Yars PE=2 SV=3 - [SYYC_MOUSE]	-0.45	-0.47	-0.31	-0.33	0.00
Q8BP71-6	Isoform 6 of RNA binding protein fox-1 homolog 2 OS=Mus musculus GN=Rbfox2 - [RFOX2_MOUSE]	0.25	0.34	0.35	0.42	0.00
Q3US41-4	Isoform 4 of Epithelial splicing regulatory protein 1 OS=Mus musculus GN=Esrp1 - [ESRP1_MOUSE]	0.19	0.17	0.17	0.26	0.00
F8WIE5	E3 ubiquitin-protein ligase HECTD1 OS=Mus musculus GN=Hectd1 PE=2 SV=1 - [F8WIE5_MOUSE]	-0.57	-0.38	-0.49	-0.64	0.00
Q99LD4	COP9 signalosome complex subunit 1 OS=Mus musculus GN=Gps1 PE=1 SV=1 - [CSN1_MOUSE]	-0.58	-0.37	-0.57	-0.44	0.00
Q9D7N9	Adipocyte plasma membrane-associated protein OS=Mus musculus GN=Apmap PE=1 SV=1 - [APMAP_MOUSE]	-0.26	-0.31	-0.18	-0.24	0.00

Q6TXD4	Dynamin-binding protein OS=Mus musculus GN=Dnmbp PE=1 SV=2 - [DNMBP_MOUSE]	-0.75	-0.58	-0.99	-0.85	0.00
Q9DB10	Single-pass membrane protein with aspartate-rich tail 1, mitochondrial OS=Mus musculus GN=Smdt1 PE=2 SV=1 - [SMDT1_MOUSE]	-0.93	-1.12	-0.66	-0.86	0.00
O55201-2	Isoform 2 of Transcription elongation factor SPT5 OS=Mus musculus GN=Supt5h - [SPT5H_MOUSE]	0.18	0.18	0.22	0.28	0.00
Q9R1P3	Proteasome subunit beta type-2 OS=Mus musculus GN=Psmb2 PE=1 SV=1 - [PSB2_MOUSE]	0.07	0.11	0.08	0.07	0.00
Q9D5T0	ATPase family AAA domain-containing protein 1 OS=Mus musculus GN=Atad1 PE=1 SV=1 - [ATAD1_MOUSE]	-0.46	-0.29	-0.44	-0.50	0.00
P70303	CTP synthase 2 OS=Mus musculus GN=Ctps2 PE=1 SV=1 - [PYRG2_MOUSE]	-0.44	-0.42	-0.31	-0.28	0.00
E9Q4Y1	Protein Adh6a OS=Mus musculus GN=Adh6a PE=2 SV=1 - [E9Q4Y1_MOUSE]	0.55	0.80	0.53	0.77	0.00
Q9CQU0	Thioredoxin domain-containing protein 12 OS=Mus musculus GN=Txndc12 PE=2 SV=1 - [TXD12_MOUSE]	0.35	0.24	0.39	0.29	0.00
O54890	Integrin beta-3 OS=Mus musculus GN=Itgb3 PE=1 SV=2 - [ITB3_MOUSE]	-0.66	-0.56	-0.86	-0.90	0.00
O55028	[3-methyl-2-oxobutanoate dehydrogenase [lipoamide]] kinase, mitochondrial OS=Mus musculus GN=Bckdk PE=1 SV=1 - [BCKD_MOUSE]	-0.48	-0.61	-0.36	-0.50	0.00
Q60596	DNA repair protein XRCC1 OS=Mus musculus GN=Xrcc1 PE=1 SV=2 - [XRCC1_MOUSE]	0.62	0.82	0.51	0.80	0.00
Q9D8I3	Glyoxalase domain-containing protein 5 OS=Mus musculus GN=Glod5 PE=2 SV=1 - [GLOD5_MOUSE]	-0.37	-0.33	-0.42	-0.53	0.00
Q8BSK8-2	Isoform Alpha II of Ribosomal protein S6 kinase beta-1 OS=Mus musculus GN=Rps6kb1 - [KS6B1_MOUSE]	-0.71	-0.50	-0.66	-0.45	0.00
P54823	Probable ATP-dependent RNA helicase DDX6 OS=Mus musculus GN=Ddx6 PE=2 SV=1 - [DDX6_MOUSE]	-0.26	-0.16	-0.27	-0.21	0.00
Q9JHS9	Spliceosome-associated protein CWC15 homolog OS=Mus musculus GN=Cwc15 PE=1 SV=1 - [CWC15_MOUSE]	0.38	0.31	0.35	0.50	0.00
Q9D787	Peptidyl-prolyl cis-trans isomerase-like 2 OS=Mus musculus GN=Ppil2 PE=2 SV=2 - [PPIL2_MOUSE]	0.39	0.50	0.60	0.66	0.00
Q8R3I3	Conserved oligomeric Golgi complex subunit 6 OS=Mus musculus GN=Cog6 PE=2 SV=2 - [COG6_MOUSE]	-0.42	-0.45	-0.26	-0.39	0.00
Q9CR68	Cytochrome b-c1 complex subunit Rieske, mitochondrial OS=Mus musculus GN=Uqcrfs1 PE=1 SV=1 - [UCRI_MOUSE]	-0.47	-0.32	-0.38	-0.29	0.00

P35276	Ras-related protein Rab-3D OS=Mus musculus GN=Rab3d PE=1 SV=1 - [RAB3D_MOUSE]	-0.65	-0.64	-0.40	-0.50	0.00
Q6PDL0	Cytoplasmic dynein 1 light intermediate chain 2 OS=Mus musculus GN=Dync1li2 PE=1 SV=2 - [DC1L2_MOUSE]	0.22	0.29	0.29	0.19	0.00
O89086	Putative RNA-binding protein 3 OS=Mus musculus GN=Rbm3 PE=1 SV=1 - [RBM3_MOUSE]	0.63	0.43	0.46	0.39	0.00
Q811G0-2	Isoform 2 of Protein PTHB1 OS=Mus musculus GN=Bbs9 - [PTHB1_MOUSE]	0.39	0.34	0.55	0.49	0.00
G3X982	Aldehyde oxidase 3, isoform CRA_a OS=Mus musculus GN=Aox3 PE=4 SV=1 - [G3X982_MOUSE]	1.29	1.29	1.89	1.89	0.00
Q91VI7	Ribonuclease inhibitor OS=Mus musculus GN=Rnh1 PE=1 SV=1 - [RINI_MOUSE]	0.13	0.20	0.13	0.17	0.00
H3BLB5	Uncharacterized protein OS=Mus musculus GN=Vmn2r ps128 PE=2 SV=1 - [H3BLB5_MOUSE]	0.77	0.54	0.71	0.48	0.00
P19324	Serpin H1 OS=Mus musculus GN=Serpinh1 PE=1 SV=3 - [SERPH_MOUSE]	0.08	0.11	0.13	0.14	0.00
O35218	Cleavage and polyadenylation specificity factor subunit 2 OS=Mus musculus GN=Cpsf2 PE=1 SV=1 - [CPSF2_MOUSE]	0.23	0.22	0.31	0.34	0.00
Q99JZ0	Syntenin-2 OS=Mus musculus GN=Sdcbp2 PE=2 SV=1 - [SDCB2_MOUSE]	1.32	1.26	1.04	0.79	0.00
P35991	Tyrosine-protein kinase BTK OS=Mus musculus GN=Btk PE=1 SV=4 - [BTK_MOUSE]	-0.39	-0.46	-0.31	-0.29	0.00
Q3V3R1	Monofunctional C1-tetrahydrofolate synthase, mitochondrial OS=Mus musculus GN=Mthfd1l PE=1 SV=2 - [C1TM_MOUSE]	-0.60	-0.45	-0.45	-0.35	0.00
Q9D0R8	Protein LSM12 homolog OS=Mus musculus GN=Lsm12 PE=1 SV=1 - [LSM12_MOUSE]	0.64	0.56	0.84	0.90	0.00
Q9QXK3-4	Isoform 4 of Coatomer subunit gamma-2 OS=Mus musculus GN=Copg2 - [COPG2_MOUSE]	-0.43	-0.26	-0.44	-0.38	0.00
Q5SVD0	Protein FAM101B OS=Mus musculus GN=Fam101b PE=2 SV=1 - [F101B_MOUSE]	0.18	0.27	0.19	0.28	0.00
Q9CQF7	Prefoldin 1 OS=Mus musculus GN=Pfdn1 PE=2 SV=1 - [Q9CQF7_MOUSE]	0.42	0.39	0.58	0.60	0.00
Q11011	Puromycin-sensitive aminopeptidase OS=Mus musculus GN=Npepps PE=1 SV=2 - [PSA_MOUSE]	-0.21	-0.26	-0.15	-0.22	0.00
O35709	Ectoderm-neural cortex protein 1 OS=Mus musculus GN=Enc1 PE=2 SV=2 - [ENC1_MOUSE]	0.45	0.43	0.32	0.29	0.00

Q8BHG9	CGG triplet repeat-binding protein 1 OS=Mus musculus GN=Cggbp1 PE=2 SV=1 - [CGBP1_MOUSE]	0.29	0.21	0.28	0.18	0.00
O35988	Syndecan-4 OS=Mus musculus GN=Sdc4 PE=1 SV=1 - [SDC4_MOUSE]	0.36	0.24	0.41	0.37	0.00
P52431	DNA polymerase delta catalytic subunit OS=Mus musculus GN=Pold1 PE=1 SV=2 - [DPOD1_MOUSE]	-0.21	-0.31	-0.36	-0.27	0.00
P01868	Ig gamma-1 chain C region secreted form OS=Mus musculus GN=Ighg1 PE=1 SV=1 - [IGHG1_MOUSE]	-0.72	-0.69	-0.48	-0.47	0.00
Q9D9D9	Uncharacterized protein C1orf189 homolog OS=Mus musculus PE=2 SV=2 - [CA189_MOUSE]	1.08	0.73	1.15	0.79	0.00
Q791V5	Mitochondrial carrier homolog 2 OS=Mus musculus GN=Mtch2 PE=1 SV=1 - [MTCH2_MOUSE]	-1.60	-1.46	-1.11	-1.00	0.00
Q91X52	L-xylulose reductase OS=Mus musculus GN=Dcxr PE=2 SV=2 - [DCXR_MOUSE]	-0.38	-0.32	-0.47	-0.29	0.00
P35700	Peroxiredoxin-1 OS=Mus musculus GN=Prdx1 PE=1 SV=1 - [PRDX1_MOUSE]	0.38	0.29	0.48	0.48	0.00
Q61165	Sodium/hydrogen exchanger 1 OS=Mus musculus GN=Slc9a1 PE=1 SV=1 - [SL9A1_MOUSE]	0.27	0.23	0.17	0.28	0.00
P35230	Regenerating islet-derived protein 3-beta OS=Mus musculus GN=Reg3b PE=1 SV=1 - [REG3B_MOUSE]	1.52	2.25	1.88	2.57	0.00
P16406	Glutamyl aminopeptidase OS=Mus musculus GN=Enpep PE=1 SV=1 - [AMPE_MOUSE]	-0.26	-0.24	-0.15	-0.27	0.00
Q9D6K8	FUN14 domain-containing protein 2 OS=Mus musculus GN=Fundc2 PE=2 SV=1 - [FUND2_MOUSE]	-0.69	-0.51	-0.57	-0.40	0.00
Q3V2J0	Protein FAM92B OS=Mus musculus GN=Fam92b PE=2 SV=2 - [FA92B_MOUSE]	-0.23	-0.34	-0.28	-0.39	0.00
Q3U0Y2	Uncharacterized protein ZMYM6NB OS=Mus musculus GN=Zmym6nb PE=2 SV=2 - [ZMYNB_MOUSE]	0.84	0.57	0.95	0.68	0.00
P32233	Developmentally-regulated GTP-binding protein 1 OS=Mus musculus GN=Drg1 PE=1 SV=1 - [DRG1_MOUSE]	-0.32	-0.47	-0.31	-0.45	0.00
J3QP71	Basigin (Fragment) OS=Mus musculus GN=Bsg PE=4 SV=1 - [J3QP71_MOUSE]	-0.55	-0.76	-0.45	-0.65	0.00
Q8R1F0	Leydig cell tumor 10 kDa protein homolog OS=Mus musculus GN=D8Ertd738e PE=2 SV=1 - [L10K_MOUSE]	0.50	0.41	0.39	0.29	0.00
P01675	Ig kappa chain V-VI region XRPC 44 OS=Mus musculus PE=1 SV=1 - [KV6A1_MOUSE]	0.38	0.38	0.31	0.23	0.00

P11672	Neutrophil gelatinase-associated lipocalin OS=Mus musculus GN=Lcn2 PE=1 SV=1 - [NGAL_MOUSE]	0.63	0.67	0.83	1.01	0.00
P08101-2	Isoform IIB2 of Low affinity immunoglobulin gamma Fc region receptor II OS=Mus musculus GN=Fcgr2 - [FCGR2_MOUSE]	0.23	0.25	0.36	0.34	0.00
D3Z5G0	Protein Pydc4 OS=Mus musculus GN=Pydc4 PE=2 SV=1 - [D3Z5G0_MOUSE]	-0.96	-1.24	-0.71	-0.99	0.00
Q9JL26-2	Isoform 2 of Formin-like protein 1 OS=Mus musculus GN=Fmnl1 - [FMNL_MOUSE]	0.28	0.21	0.34	0.22	0.00
Q9QXA5	U6 snRNA-associated Sm-like protein LSm4 OS=Mus musculus GN=Lsm4 PE=2 SV=1 - [LSM4_MOUSE]	0.51	0.46	0.32	0.34	0.00
G5E8C3	G protein-coupled receptor, family C, group 5, member A OS=Mus musculus GN=Gprc5a PE=4 SV=1 - [G5E8C3_MOUSE]	-1.42	-1.07	-1.27	-0.83	0.00
Q9D7S0	Ly6/PLAUR domain-containing protein 8 OS=Mus musculus GN=Lypd8 PE=2 SV=1 - [LYPD8_MOUSE]	-1.24	-1.43	-0.85	-1.03	0.00
Q8R5H1	Ubiquitin carboxyl-terminal hydrolase 15 OS=Mus musculus GN=Usp15 PE=2 SV=1 - [UBP15_MOUSE]	0.43	0.54	0.31	0.42	0.00
G3X922	MCG115602 OS=Mus musculus GN=Dnajc13 PE=4 SV=1 - [G3X922_MOUSE]	-0.41	-0.26	-0.35	-0.45	0.00
O55229	Choline/ethanolamine kinase OS=Mus musculus GN=Chkb PE=1 SV=3 - [CHKB_MOUSE]	-0.66	-0.43	-0.60	-0.44	0.00
P13020-2	Isoform 2 of Gelsolin OS=Mus musculus GN=Gsn - [GELS_MOUSE]	-0.21	-0.31	-0.22	-0.32	0.00
P61804	Dolichyl-diphosphooligosaccharide--protein glycosyltransferase subunit DAD1 OS=Mus musculus GN=Dad1 PE=2 SV=3 - [DAD1_MOUSE]	-0.69	-0.49	-0.77	-0.52	0.00
Q9QZ06	Toll-interacting protein OS=Mus musculus GN=Tollip PE=1 SV=1 - [TOLIP_MOUSE]	0.42	0.27	0.47	0.37	0.00
Q8K023-2	Isoform 2 of Aldo-keto reductase family 1 member C18 OS=Mus musculus GN=Akr1c18 - [AKC1H_MOUSE]	0.88	0.59	0.99	0.70	0.00
Q8K409	DNA polymerase beta OS=Mus musculus GN=Polb PE=2 SV=3 - [DPOLB_MOUSE]	0.36	0.40	0.25	0.26	0.00
Q6NV99	Protein Haus6 OS=Mus musculus GN=Haus6 PE=2 SV=1 - [Q6NV99_MOUSE]	0.71	0.52	0.62	0.42	0.00
A6H584	Collagen alpha-5(VI) chain OS=Mus musculus GN=Col6a5 PE=1 SV=2 - [CO6A5_MOUSE]	0.42	0.56	0.32	0.46	0.00
Q3U487	E3 ubiquitin-protein ligase HECTD3 OS=Mus musculus GN=Hectd3 PE=1 SV=2 - [HECD3_MOUSE]	-0.40	-0.39	-0.59	-0.58	0.00

Q9CQX8	28S ribosomal protein S36, mitochondrial OS=Mus musculus GN=Mrps36 PE=1 SV=1 - [RT36_MOUSE]	0.32	0.28	0.46	0.34	0.00
Q9Z1G3	V-type proton ATPase subunit C 1 OS=Mus musculus GN=Atp6v1c1 PE=1 SV=4 - [VATC1_MOUSE]	-0.69	-0.56	-0.48	-0.42	0.00
Q8R3Y8-2	Isoform 2 of Interferon regulatory factor 2-binding protein 1 OS=Mus musculus GN=Irf2bp1 - [I2BP1_MOUSE]	-0.49	-0.44	-0.31	-0.32	0.00
P31001	Desmin OS=Mus musculus GN=Des PE=1 SV=3 - [DESM_MOUSE]	0.12	0.08	0.11	0.08	0.00
J3QNK1	Synphilin-1 OS=Mus musculus GN=Sncaip PE=4 SV=1 - [J3QNK1_MOUSE]	-0.72	-0.74	-0.48	-0.51	0.00
O35927-2	Isoform 2 of Catenin delta-2 OS=Mus musculus GN=Ctnnd2 - [CTND2_MOUSE]	-0.72	-1.06	-0.88	-1.22	0.00
P62737	Actin, aortic smooth muscle OS=Mus musculus GN=Acta2 PE=1 SV=1 - [ACTA_MOUSE]	-0.28	-0.42	-0.38	-0.50	0.00
O35098	Dihydropyrimidinase-related protein 4 OS=Mus musculus GN=Dpysl4 PE=1 SV=1 - [DPYL4_MOUSE]	-0.22	-0.28	-0.31	-0.38	0.00
Q62468	Villin-1 OS=Mus musculus GN=Vil1 PE=1 SV=3 - [VILI_MOUSE]	-0.13	-0.12	-0.09	-0.08	0.00
Q9CR41	Huntingtin-interacting protein K OS=Mus musculus GN=Hypk PE=2 SV=2 - [HYPK_MOUSE]	0.17	0.24	0.29	0.29	0.00
O70194	Eukaryotic translation initiation factor 3 subunit D OS=Mus musculus GN=Eif3d PE=1 SV=2 - [EIF3D_MOUSE]	0.34	0.21	0.37	0.33	0.00
Q07417	Short-chain specific acyl-CoA dehydrogenase, mitochondrial OS=Mus musculus GN=Acads PE=2 SV=2 - [ACADS_MOUSE]	0.15	0.18	0.10	0.13	0.00
E9PUD6	Protein Zscan18 OS=Mus musculus GN=Zscan18 PE=4 SV=1 - [E9PUD6_MOUSE]	0.48	0.63	0.36	0.52	0.00
H3BJ37	Catechol O-methyltransferase domain-containing protein 1 OS=Mus musculus GN=Comtd1 PE=2 SV=1 - [H3BJ37_MOUSE]	-0.50	-0.33	-0.56	-0.40	0.00
O08797	Protein Serpinb9 OS=Mus musculus GN=Serpinb9 PE=2 SV=1 - [O08797_MOUSE]	0.20	0.16	0.14	0.12	0.00
P52479-2	Isoform 2 of Ubiquitin carboxyl-terminal hydrolase 10 OS=Mus musculus GN=Usp10 - [UBP10_MOUSE]	0.52	0.65	0.82	0.88	0.00
P54818	Galactocerebrosidase OS=Mus musculus GN=Galc PE=1 SV=2 - [GALC_MOUSE]	-0.23	-0.15	-0.26	-0.18	0.00
P15327	Bisphosphoglycerate mutase OS=Mus musculus GN=Bpgm PE=2 SV=2 - [PMGE_MOUSE]	0.30	0.44	0.27	0.33	0.00

Q9D6M3	Mitochondrial glutamate carrier 1 OS=Mus musculus GN=Slc25a22 PE=1 SV=1 - [GHC1_MOUSE]	-0.14	-0.18	-0.17	-0.23	0.00
P63054	Purkinje cell protein 4 OS=Mus musculus GN=Pcp4 PE=2 SV=2 - [PCP4_MOUSE]	1.06	0.71	1.27	1.08	0.00
Q99KY4-2	Isoform 2 of Cyclin-G-associated kinase OS=Mus musculus GN=Gak - [GAK_MOUSE]	0.45	0.34	0.29	0.47	0.00
P19639	Glutathione S-transferase Mu 3 OS=Mus musculus GN=Gstm3 PE=1 SV=2 - [GSTM4_MOUSE]	-0.14	-0.15	-0.20	-0.23	0.00
Q810D6	Glutamate-rich WD repeat-containing protein 1 OS=Mus musculus GN=Grwd1 PE=2 SV=2 - [GRWD1_MOUSE]	0.23	0.13	0.19	0.21	0.00
P19426-2	Isoform 2 of Negative elongation factor E OS=Mus musculus GN=Nelfe - [NELFE_MOUSE]	0.29	0.50	0.38	0.45	0.00
O35226-2	Isoform Rpn10B of 26S proteasome non-ATPase regulatory subunit 4 OS=Mus musculus GN=Psmd4 - [PSMD4_MOUSE]	0.40	0.50	0.29	0.39	0.00
O54865	Guanylate cyclase soluble subunit beta-1 OS=Mus musculus GN=Gucy1b3 PE=2 SV=1 - [GCYB1_MOUSE]	-0.33	-0.23	-0.20	-0.29	0.00
Q9D8Y7	Tumor necrosis factor alpha-induced protein 8-like protein 2 OS=Mus musculus GN=Tnfaip8l2 PE=1 SV=1 - [TP8L2_MOUSE]	0.67	0.54	0.38	0.51	0.00
D3YXA1	Lebercilin (Fragment) OS=Mus musculus GN=Lca5 PE=2 SV=1 - [D3YXA1_MOUSE]	-0.69	-0.47	-0.81	-0.60	0.00
Q9Z204-2	Isoform C1 of Heterogeneous nuclear ribonucleoproteins C1/C2 OS=Mus musculus GN=Hnrnpc - [HNRPC_MOUSE]	0.53	0.75	0.44	0.60	0.00
Q9Z0F7	Gamma-synuclein OS=Mus musculus GN=Sncg PE=1 SV=1 - [SYUG_MOUSE]	0.51	0.41	0.70	0.50	0.00
Q3THE2	Myosin regulatory light chain 12B OS=Mus musculus GN=Myl12b PE=1 SV=2 - [ML12B_MOUSE]	0.52	0.63	0.38	0.42	0.00
Q9EPT5	Solute carrier organic anion transporter family member 2A1 OS=Mus musculus GN=Slco2a1 PE=1 SV=2 - [SO2A1_MOUSE]	-1.14	-0.82	-0.99	-0.67	0.00
C6KI89	Cation channel sperm-associated protein subunit gamma 2 OS=Mus musculus GN=Catsperg2 PE=1 SV=1 - [CTSG2_MOUSE]	-1.11	-1.05	-0.76	-0.70	0.00
Q62077	1-phosphatidylinositol 4,5-bisphosphate phosphodiesterase gamma-1 OS=Mus musculus GN=Plcg1 PE=1 SV=2 - [PLCG1_MOUSE]	-0.16	-0.12	-0.20	-0.14	0.00
Q9R100	Cadherin-17 OS=Mus musculus GN=Cdh17 PE=1 SV=1 - [CAD17_MOUSE]	-0.39	-0.45	-0.25	-0.37	0.00
Q9Z103	Activity-dependent neuroprotector homeobox protein OS=Mus musculus GN=Adnp PE=2 SV=1 - [ADNP_MOUSE]	0.59	0.88	0.57	0.85	0.00

Q8R0K4	Coiled-coil domain-containing protein 137 OS=Mus musculus GN=Ccdc137 PE=2 SV=1 - [CC137_MOUSE]	0.51	0.48	0.76	0.69	0.00
P48722	Heat shock 70 kDa protein 4L OS=Mus musculus GN=Hspa4l PE=1 SV=2 - [HS74L_MOUSE]	-0.16	-0.16	-0.13	-0.23	0.00
Q9WVE8	Protein kinase C and casein kinase substrate in neurons protein 2 OS=Mus musculus GN=Pacsin2 PE=1 SV=1 - [PACN2_MOUSE]	-0.30	-0.31	-0.22	-0.20	0.00
P35428	Transcription factor HES-1 OS=Mus musculus GN=Hes1 PE=1 SV=1 - [HES1_MOUSE]	0.90	0.63	0.82	0.54	0.00
Q9D2G2	Dihydrolipoyllysine-residue succinyltransferase component of 2-oxoglutarate dehydrogenase complex, mitochondrial OS=Mus musculus GN=Dlst PE=1 SV=1 - [ODO2_MOUSE]	0.11	0.07	0.09	0.11	0.00
Q6PAM1	Alpha-taxilin OS=Mus musculus GN=Txlna PE=2 SV=1 - [TXLNA_MOUSE]	0.37	0.28	0.44	0.49	0.00
Q9EQ06	Estradiol 17-beta-dehydrogenase 11 OS=Mus musculus GN=Hsd17b11 PE=2 SV=1 - [DHB11_MOUSE]	-0.28	-0.32	-0.22	-0.19	0.00
Q925N0	Sideroflexin-5 OS=Mus musculus GN=Sfxn5 PE=1 SV=2 - [SFXN5_MOUSE]	-1.45	-1.32	-2.06	-2.06	0.00
E9Q394	Protein Akap13 OS=Mus musculus GN=Akap13 PE=2 SV=1 - [E9Q394_MOUSE]	0.63	0.61	0.36	0.54	0.00
Q00519	Xanthine dehydrogenase/oxidase OS=Mus musculus GN=Xdh PE=1 SV=5 - [XDH_MOUSE]	0.35	0.36	0.56	0.43	0.00
Q8BFP9	[Pyruvate dehydrogenase [lipoamide]] kinase isozyme 1, mitochondrial OS=Mus musculus GN=Pdk1 PE=2 SV=2 - [PDK1_MOUSE]	-0.51	-0.44	-0.73	-0.67	0.00
Q9D0R2	Threonine--tRNA ligase, cytoplasmic OS=Mus musculus GN=Tars PE=1 SV=2 - [SYTC_MOUSE]	0.18	0.23	0.20	0.30	0.00
Q91ZU6-5	Isoform 5 of Dystonin OS=Mus musculus GN=Dst - [DYST_MOUSE]	-0.67	-0.57	-0.49	-0.39	0.00
Q4LDD4-3	Isoform 3 of Arf-GAP with Rho-GAP domain, ANK repeat and PH domain-containing protein 1 OS=Mus musculus GN=Arap1 - [ARAP1_MOUSE]	-0.32	-0.46	-0.28	-0.42	0.00
Q9CQW5	Galectin-2 OS=Mus musculus GN=Lgals2 PE=2 SV=1 - [LEG2_MOUSE]	0.59	0.51	0.82	0.80	0.00
J3QN09	Arginyl aminopeptidase (Aminopeptidase B)-like 1, isoform CRA_a OS=Mus musculus GN=Rnpepl1 PE=4 SV=1 - [J3QN09_MOUSE]	0.13	0.20	0.14	0.21	0.00
Q60972	Histone-binding protein RBBP4 OS=Mus musculus GN=Rbbp4 PE=1 SV=5 - [RBBP4_MOUSE]	0.16	0.16	0.22	0.25	0.00
P26369	Splicing factor U2AF 65 kDa subunit OS=Mus musculus GN=U2af2 PE=1 SV=3 - [U2AF2_MOUSE]	0.40	0.40	0.58	0.60	0.00

O35074	Prostacyclin synthase OS=Mus musculus GN=Ptgis PE=2 SV=1 - [PTGIS_MOUSE]	-0.31	-0.24	-0.40	-0.39	0.00
Q8BTX9	Inactive hydroxysteroid dehydrogenase-like protein 1 OS=Mus musculus GN=Hsdl1 PE=2 SV=1 - [HSDL1_MOUSE]	-0.72	-0.71	-0.49	-0.47	0.00
Q9JM14	5'(3')-deoxyribonucleotidase, cytosolic type OS=Mus musculus GN=Nt5c PE=1 SV=1 - [NT5C_MOUSE]	-0.61	-0.88	-0.80	-1.08	0.00
P16294	Coagulation factor IX OS=Mus musculus GN=F9 PE=2 SV=3 - [FA9_MOUSE]	0.33	0.24	0.29	0.19	0.00
Q9D1E8	1-acyl-sn-glycerol-3-phosphate acyltransferase epsilon OS=Mus musculus GN=Agpat5 PE=2 SV=2 - [PLCE_MOUSE]	-0.68	-0.46	-0.64	-0.43	0.00
Q9QXJ2	Signal transducer and activator of transcription 2 OS=Mus musculus GN=Stat2 PE=1 SV=1 - [Q9QXJ2_MOUSE]	0.41	0.27	0.25	0.33	0.00
P99026	Proteasome subunit beta type-4 OS=Mus musculus GN=Psmb4 PE=1 SV=1 - [PSB4_MOUSE]	0.23	0.13	0.24	0.20	0.00
Q9JKJ9	24-hydroxycholesterol 7-alpha-hydroxylase OS=Mus musculus GN=Cyp39a1 PE=2 SV=1 - [CP39A_MOUSE]	-0.92	-0.77	-0.67	-0.53	0.00
Q80SZ7	Guanine nucleotide-binding protein G(I)/G(S)/G(O) subunit gamma-5 OS=Mus musculus GN=Gng5 PE=2 SV=2 - [GBG5_MOUSE]	0.22	0.37	0.27	0.35	0.00
Q8BY35-2	Isoform 2 of FYVE, RhoGEF and PH domain-containing protein 2 OS=Mus musculus GN=Fgd2 - [FGD2_MOUSE]	0.96	0.66	1.15	0.84	0.00
Q8BTS4	Nuclear pore complex protein Nup54 OS=Mus musculus GN=Nup54 PE=1 SV=1 - [NUP54_MOUSE]	0.63	0.43	0.38	0.45	0.00
Q9CYX7	RRP15-like protein OS=Mus musculus GN=Rrp15 PE=2 SV=2 - [RRP15_MOUSE]	0.82	0.60	1.07	0.84	0.00
G5E819	Serine/threonine-protein phosphatase OS=Mus musculus GN=Ppp5c PE=3 SV=1 - [G5E819_MOUSE]	-0.45	-0.48	-0.34	-0.29	0.00
Q6A068	Cell division cycle 5-like protein OS=Mus musculus GN=Cdc5l PE=1 SV=2 - [CDC5L_MOUSE]	0.65	0.44	0.79	0.70	0.00
Q9ESJ0	Exportin-4 OS=Mus musculus GN=Xpo4 PE=1 SV=2 - [XPO4_MOUSE]	-0.70	-0.77	-0.43	-0.65	0.00
Q9JHI5	Isovaleryl-CoA dehydrogenase, mitochondrial OS=Mus musculus GN=Ivd PE=1 SV=1 - [IVD_MOUSE]	-0.11	-0.14	-0.08	-0.12	0.00
Q9CR62	Mitochondrial 2-oxoglutarate/malate carrier protein OS=Mus musculus GN=Slc25a11 PE=1 SV=3 - [M2OM_MOUSE]	-0.25	-0.21	-0.36	-0.30	0.00
Q4V9Z5-3	Isoform 3 of Seizure 6-like protein 2 OS=Mus musculus GN=Sez6l2 - [SE6L2_MOUSE]	1.51	1.01	1.74	1.24	0.00

Q9JJZ6	Krueppel-like factor 13 OS=Mus musculus GN=Klf13 PE=2 SV=2 - [KLF13_MOUSE]	0.30	0.45	0.39	0.54	0.00
Q8VEE1	LIM and cysteine-rich domains protein 1 OS=Mus musculus GN=Lmcd1 PE=1 SV=1 - [LMCD1_MOUSE]	0.70	0.67	0.43	0.49	0.00
A2ADR8	Nuclear inhibitor of protein phosphatase 1 OS=Mus musculus GN=Ppp1r8 PE=2 SV=1 - [A2ADR8_MOUSE]	0.29	0.20	0.31	0.36	0.00
Q9D6K9	Ceramide synthase 5 OS=Mus musculus GN=Cers5 PE=1 SV=1 - [CERS5_MOUSE]	-0.49	-0.35	-0.42	-0.28	0.00
Q5SWU9	Acetyl-CoA carboxylase 1 OS=Mus musculus GN=Acaca PE=1 SV=1 - [ACACA_MOUSE]	-0.50	-0.37	-0.58	-0.36	0.00
Q8K1S3-2	Isoform 2 of Netrin receptor UNC5B OS=Mus musculus GN=Unc5b - [UNC5B_MOUSE]	-0.60	-0.58	-0.81	-0.92	0.00
B1AUQ7	Centrin-2 (Fragment) OS=Mus musculus GN=Cetn2 PE=2 SV=1 - [B1AUQ7_MOUSE]	0.53	0.37	0.65	0.48	0.00
P08121	Collagen alpha-1(III) chain OS=Mus musculus GN=Col3a1 PE=1 SV=4 - [CO3A1_MOUSE]	0.45	0.26	0.43	0.34	0.00
O54950	5'-AMP-activated protein kinase subunit gamma-1 OS=Mus musculus GN=Prkag1 PE=1 SV=2 - [AAKG1_MOUSE]	-0.25	-0.16	-0.27	-0.18	0.00
P57784	U2 small nuclear ribonucleoprotein A' OS=Mus musculus GN=Snrpa1 PE=1 SV=2 - [RU2A_MOUSE]	0.27	0.34	0.19	0.28	0.00
P97805	Protein FAM3D OS=Mus musculus GN=Fam3d PE=2 SV=2 - [FAM3D_MOUSE]	-0.70	-1.09	-0.70	-1.01	0.00
F6YRQ2	DnaJ homolog subfamily B member 6 (Fragment) OS=Mus musculus GN=Dnajb6 PE=4 SV=1 - [F6YRQ2_MOUSE]	-0.86	-1.03	-0.58	-0.76	0.00
Q3THK3	General transcription factor IIF subunit 1 OS=Mus musculus GN=Gtf2f1 PE=1 SV=2 - [T2FA_MOUSE]	0.42	0.65	0.62	0.77	0.00
D3YXQ4	Beta-chimaerin (Fragment) OS=Mus musculus GN=Chn2 PE=2 SV=1 - [D3YXQ4_MOUSE]	1.06	0.86	1.49	1.29	0.00
P49312	Heterogeneous nuclear ribonucleoprotein A1 OS=Mus musculus GN=Hnrnpa1 PE=1 SV=2 - [ROA1_MOUSE]	0.50	0.34	0.62	0.51	0.00
Q62000	Mimecan OS=Mus musculus GN=Ogn PE=2 SV=1 - [MIME_MOUSE]	-0.27	-0.21	-0.23	-0.15	0.00
Q8R4P4	Transmembrane channel-like protein 2 OS=Mus musculus GN=Tmc2 PE=2 SV=1 - [TMC2_MOUSE]	0.86	1.16	1.25	1.54	0.00
Q6ZWQ7	Signal peptidase complex subunit 3 OS=Mus musculus GN=Spcs3 PE=2 SV=1 - [Q6ZWQ7_MOUSE]	-0.45	-0.35	-0.46	-0.27	0.00

P35980	60S ribosomal protein L18 OS=Mus musculus GN=Rpl18 PE=2 SV=3 - [RL18_MOUSE]	0.20	0.15	0.26	0.23	0.00
G3UZA0	KRR1 small subunit processome component homolog OS=Mus musculus GN=Krr1 PE=2 SV=1 - [G3UZA0_MOUSE]	-0.39	-0.37	-0.58	-0.56	0.00
Q80U93	Nuclear pore complex protein Nup214 OS=Mus musculus GN=Nup214 PE=1 SV=2 - [NU214_MOUSE]	0.64	0.57	0.45	0.37	0.00
Q99K85	Phosphoserine aminotransferase OS=Mus musculus GN=Psat1 PE=1 SV=1 - [SERC_MOUSE]	0.36	0.64	0.55	0.63	0.00
O89053	Coronin-1A OS=Mus musculus GN=Coro1a PE=1 SV=5 - [COR1A_MOUSE]	0.31	0.28	0.45	0.44	0.00
Q3U3J1	2-oxoisovalerate dehydrogenase subunit alpha, mitochondrial OS=Mus musculus GN=Bckdha PE=2 SV=1 - [Q3U3J1_MOUSE]	-0.14	-0.20	-0.21	-0.13	0.00
Q61672	Equilibrative nucleoside transporter 2 OS=Mus musculus GN=Slc29a2 PE=1 SV=2 - [S29A2_MOUSE]	0.28	0.42	0.28	0.42	0.00
P54310	Hormone-sensitive lipase OS=Mus musculus GN=Lipe PE=1 SV=2 - [LIPS_MOUSE]	0.73	0.65	0.54	0.41	0.00
Q8CHW4	Translation initiation factor eIF-2B subunit epsilon OS=Mus musculus GN=Eif2b5 PE=1 SV=1 - [EI2BE_MOUSE]	-0.28	-0.25	-0.20	-0.16	0.00
Q9WV69-2	Isoform 2 of Dematin OS=Mus musculus GN=Dmtn - [DEMA_MOUSE]	0.23	0.34	0.28	0.40	0.00
Q8CC86	Nicotinate phosphoribosyltransferase OS=Mus musculus GN=Naprt1 PE=2 SV=1 - [PNCB_MOUSE]	-0.27	-0.29	-0.44	-0.34	0.00
P12265	Beta-glucuronidase OS=Mus musculus GN=Gusb PE=2 SV=2 - [BGLR_MOUSE]	0.08	0.09	0.07	0.13	0.00
P28867	Protein kinase C delta type OS=Mus musculus GN=Prkcd PE=1 SV=3 - [KPCD_MOUSE]	-0.31	-0.19	-0.19	-0.25	0.00
E9Q453	Tropomyosin alpha-1 chain OS=Mus musculus GN=Tpm1 PE=2 SV=1 - [E9Q453_MOUSE]	0.42	0.45	0.26	0.34	0.00
P97370	Sodium/potassium-transporting ATPase subunit beta-3 OS=Mus musculus GN=Atp1b3 PE=1 SV=1 - [AT1B3_MOUSE]	-0.34	-0.41	-0.27	-0.24	0.00
Q64459	Cytochrome P450 3A11 OS=Mus musculus GN=Cyp3a11 PE=1 SV=1 - [CP3AB_MOUSE]	-0.27	-0.41	-0.27	-0.41	0.00
Q91XQ0	Dynein heavy chain 8, axonemal OS=Mus musculus GN=Dnahc8 PE=2 SV=2 - [DYH8_MOUSE]	0.69	0.55	0.97	0.77	0.00
Q8CHP8	Phosphoglycolate phosphatase OS=Mus musculus GN=Pgp PE=2 SV=1 - [PGP_MOUSE]	-0.22	-0.22	-0.13	-0.16	0.00

Q9QYE6	Golgin subfamily A member 5 OS=Mus musculus GN=Golga5 PE=1 SV=2 - [GOGA5_MOUSE]	0.60	0.41	0.38	0.39	0.00
Q8R409	Protein HEXIM1 OS=Mus musculus GN=Hexim1 PE=1 SV=1 - [HEXI1_MOUSE]	0.64	0.42	0.59	0.40	0.00
Q9DBS5	Kinesin light chain 4 OS=Mus musculus GN=Klc4 PE=1 SV=1 - [KLC4_MOUSE]	0.32	0.40	0.37	0.55	0.00
Q64282	Interferon-induced protein with tetratricopeptide repeats 1 OS=Mus musculus GN=Ifit1 PE=1 SV=2 - [IFIT1_MOUSE]	0.20	0.24	0.31	0.34	0.00
A2AN84	55 kDa erythrocyte membrane protein OS=Mus musculus GN=Mpp1 PE=2 SV=1 - [A2AN84_MOUSE]	0.38	0.40	0.58	0.59	0.00
Q8BYQ0	Arylacetamide deacetylase-like 1, isoform CRA_c OS=Mus musculus GN=Nceh1 PE=2 SV=1 - [Q8BYQ0_MOUSE]	0.90	0.95	0.59	0.64	0.00
P29391	Ferritin light chain 1 OS=Mus musculus GN=Ftl1 PE=1 SV=2 - [FRIL1_MOUSE]	-1.13	-0.97	-0.77	-0.67	0.00
E9Q401	Ryanodine receptor 2 OS=Mus musculus GN=Ryr2 PE=1 SV=1 - [RYR2_MOUSE]	-0.81	-0.67	-0.58	-0.45	0.00
Q8JZM7	Parafibromin OS=Mus musculus GN=Cdc73 PE=2 SV=1 - [CDC73_MOUSE]	0.35	0.25	0.36	0.46	0.00
Q8BYC6	Serine/threonine-protein kinase TAO3 OS=Mus musculus GN=Taok3 PE=1 SV=2 - [TAOK3_MOUSE]	0.21	0.15	0.23	0.15	0.00
Q99LC8	Translation initiation factor eIF-2B subunit alpha OS=Mus musculus GN=Eif2b1 PE=2 SV=1 - [EI2BA_MOUSE]	-0.80	-0.78	-0.51	-0.54	0.00
Q99N91	39S ribosomal protein L34, mitochondrial OS=Mus musculus GN=Mrpl34 PE=2 SV=1 - [RM34_MOUSE]	0.28	0.31	0.43	0.45	0.00
Q91XD2	LIM and senescent cell antigen-like-containing domain protein 2 OS=Mus musculus GN=Lims2 PE=1 SV=1 - [LIMS2_MOUSE]	-0.24	-0.21	-0.35	-0.24	0.00
Q05921	2-5A-dependent ribonuclease OS=Mus musculus GN=Rnasel PE=2 SV=2 - [RN5A_MOUSE]	-0.35	-0.36	-0.50	-0.56	0.00
Q64430	Copper-transporting ATPase 1 OS=Mus musculus GN=Atp7a PE=1 SV=3 - [ATP7A_MOUSE]	-0.28	-0.20	-0.29	-0.18	0.00
G3X9K3	Brefeldin A-inhibited guanine nucleotide-exchange protein 1 OS=Mus musculus GN=Arfgef1 PE=2 SV=1 - [BIG1_MOUSE]	0.76	0.66	0.75	0.43	0.00
A2AA86	SUZ domain-containing protein 1 OS=Mus musculus GN=Szrd1 PE=2 SV=1 - [A2AA86_MOUSE]	0.86	0.70	1.07	0.63	0.00
E9PYG6	Protein Rasa1 OS=Mus musculus GN=Rasa1 PE=2 SV=1 - [E9PYG6_MOUSE]	-0.59	-0.66	-0.38	-0.46	0.00

Q8BJD1	Inter-alpha-trypsin inhibitor heavy chain H5 OS=Mus musculus GN=Itih5 PE=2 SV=1 - [ITIH5_MOUSE]	-0.80	-0.63	-1.13	-0.93	0.00
E9QAZ2	Ribosomal protein L15 OS=Mus musculus GN=Gm10020 PE=3 SV=1 - [E9QAZ2_MOUSE]	-0.32	-0.47	-0.28	-0.32	0.00
Q8QZY9	Splicing factor 3B subunit 4 OS=Mus musculus GN=Sf3b4 PE=2 SV=1 - [SF3B4_MOUSE]	0.56	0.44	0.68	0.40	0.00
Q8BGC4	Zinc-binding alcohol dehydrogenase domain-containing protein 2 OS=Mus musculus GN=Zadh2 PE=2 SV=1 - [ZADH2_MOUSE]	-0.85	-0.68	-1.15	-0.75	0.00
Q6ZQI3	Malectin OS=Mus musculus GN=Mlec PE=2 SV=2 - [MLEC_MOUSE]	-0.23	-0.35	-0.21	-0.24	0.00
Q80UU9	Membrane-associated progesterone receptor component 2 OS=Mus musculus GN=Pgrmc2 PE=1 SV=2 - [PGRC2_MOUSE]	-0.33	-0.23	-0.34	-0.21	0.00
H7BX26	Centrosomal protein of 170 kDa OS=Mus musculus GN=Cep170 PE=2 SV=1 - [H7BX26_MOUSE]	0.52	0.39	0.71	0.57	0.00
Q5NCB9	Tripartite motif-containing protein 7 (Fragment) OS=Mus musculus GN=Trim7 PE=2 SV=2 - [Q5NCB9_MOUSE]	0.53	0.61	0.42	0.35	0.00
Q8R323	Replication factor C subunit 3 OS=Mus musculus GN=Rfc3 PE=2 SV=1 - [RFC3_MOUSE]	-0.55	-0.48	-0.76	-0.47	0.00
Q9QYC0	Alpha-adducin OS=Mus musculus GN=Add1 PE=1 SV=2 - [ADDA_MOUSE]	0.12	0.19	0.16	0.22	0.00
Q8BWY3	Eukaryotic peptide chain release factor subunit 1 OS=Mus musculus GN=Etf1 PE=1 SV=4 - [ERF1_MOUSE]	-0.19	-0.33	-0.31	-0.35	0.00
Q9R0P5	Destrin OS=Mus musculus GN=Dstn PE=1 SV=3 - [DEST_MOUSE]	-0.27	-0.31	-0.20	-0.19	0.00
Q9DAR7	m7GpppX diphosphatase OS=Mus musculus GN=Dcps PE=1 SV=1 - [DCPS_MOUSE]	-0.34	-0.25	-0.19	-0.24	0.00
Q99NF7	Ppm1b protein OS=Mus musculus GN=Ppm1b PE=2 SV=1 - [Q99NF7_MOUSE]	-0.10	-0.08	-0.06	-0.10	0.00
P60843	Eukaryotic initiation factor 4A-I OS=Mus musculus GN=Eif4a1 PE=2 SV=1 - [IF4A1_MOUSE]	-0.66	-0.68	-0.40	-0.49	0.00
Q8BXC6	COMM domain-containing protein 2 OS=Mus musculus GN=Commd2 PE=1 SV=1 - [COMD2_MOUSE]	-0.34	-0.37	-0.35	-0.21	0.00
Q9WTP6-2	Isoform 2 of Adenylate kinase 2, mitochondrial OS=Mus musculus GN=Ak2 - [KAD2_MOUSE]	-0.29	-0.41	-0.23	-0.30	0.00
Q8K2Q7	BRO1 domain-containing protein BROX OS=Mus musculus GN=Brox PE=2 SV=1 - [BROX_MOUSE]	0.74	0.59	1.04	0.89	0.00

Q91WH2	MCG131371 OS=Mus musculus GN=Ugt2b38 PE=2 SV=1 - [Q91WH2_MOUSE]	0.87	1.18	0.65	0.96	0.00
Q9CYH2	Redox-regulatory protein FAM213A OS=Mus musculus GN=Fam213a PE=1 SV=2 - [F213A_MOUSE]	-0.26	-0.30	-0.41	-0.43	0.00
O35678	Monoglyceride lipase OS=Mus musculus GN=Mgll PE=1 SV=1 - [MGLL_MOUSE]	0.47	0.42	0.45	0.26	0.00
P27546-2	Isoform 2 of Microtubule-associated protein 4 OS=Mus musculus GN=Map4 - [MAP4_MOUSE]	0.52	0.43	0.76	0.61	0.00
Q9QXE7	F-box-like/WD repeat-containing protein TBL1X OS=Mus musculus GN=Tbl1x PE=2 SV=2 - [TBL1X_MOUSE]	0.37	0.38	0.50	0.61	0.00
E9Q1G8	Septin-7 OS=Mus musculus GN=Sept7 PE=3 SV=2 - [E9Q1G8_MOUSE]	0.33	0.20	0.38	0.33	0.00
G3X973	Stabilin 1, isoform CRA_a OS=Mus musculus GN=Stab1 PE=4 SV=1 - [G3X973_MOUSE]	-0.21	-0.27	-0.15	-0.19	0.00
Q8R2U6	Diphosphoinositol polyphosphate phosphohydrolase 2 OS=Mus musculus GN=Nudt4 PE=1 SV=1 - [NUDT4_MOUSE]	0.25	0.18	0.18	0.29	0.00
Q91WL0	Epidermal growth factor receptor kinase substrate 8-like protein 3 OS=Mus musculus GN=Eps8l3 PE=2 SV=1 - [ES8L3_MOUSE]	-0.32	-0.57	-0.40	-0.46	0.00
Q99L59	X-linked lymphocyte-regulated protein 3A OS=Mus musculus GN=Xlr3a PE=2 SV=1 - [Q99L59_MOUSE]	-1.22	-1.44	-0.80	-1.02	0.00
Q9R0U9	Protein Tex21 OS=Mus musculus GN=Tex21 PE=2 SV=1 - [Q9R0U9_MOUSE]	0.24	0.26	0.37	0.38	0.00
Q9D186	Apolipoprotein O OS=Mus musculus GN=Apoo PE=2 SV=1 - [Q9D186_MOUSE]	-0.53	-0.41	-0.72	-0.66	0.00
P47199	Quinone oxidoreductase OS=Mus musculus GN=Cryz PE=2 SV=1 - [QOR_MOUSE]	-0.34	-0.30	-0.21	-0.21	0.00
Q99KK2	N-acylneuraminate cytidylyltransferase OS=Mus musculus GN=Cmas PE=1 SV=2 - [NEUA_MOUSE]	-0.21	-0.19	-0.12	-0.16	0.00
D3YVP5	Glutathione S-transferase Mu 7 OS=Mus musculus GN=Gstm7 PE=2 SV=1 - [D3YVP5_MOUSE]	-0.61	-0.69	-0.93	-1.03	0.00
E9PWE8	Dihydropyrimidinase-related protein 3 OS=Mus musculus GN=Dpysl3 PE=2 SV=1 - [E9PWE8_MOUSE]	0.28	0.26	0.43	0.41	0.00
Q05CL8	La-related protein 7 OS=Mus musculus GN=Larp7 PE=1 SV=2 - [LARP7_MOUSE]	0.12	0.15	0.21	0.15	0.00
Q80VP1	Epsin-1 OS=Mus musculus GN=Epn1 PE=1 SV=3 - [EPN1_MOUSE]	0.21	0.29	0.39	0.32	0.00

Q9DAK9	14 kDa phosphohistidine phosphatase OS=Mus musculus GN=Phpt1 PE=1 SV=1 - [PHP14_MOUSE]	0.40	0.24	0.30	0.25	0.00
A9C445	Protein 2610015P09Rik OS=Mus musculus GN=2610015P09Rik PE=2 SV=1 - [A9C445_MOUSE]	-0.47	-0.72	-0.54	-0.79	0.00
Q91X51	Golgi reassembly-stacking protein 1 OS=Mus musculus GN=Gorasp1 PE=2 SV=3 - [GORS1_MOUSE]	0.33	0.40	0.49	0.58	0.00
E9Q557	Desmoplakin OS=Mus musculus GN=Dsp PE=2 SV=1 - [DESP_MOUSE]	0.26	0.46	0.31	0.39	0.00
Q6NXL1	Protein Sec24d OS=Mus musculus GN=Sec24d PE=2 SV=1 - [Q6NXL1_MOUSE]	0.20	0.12	0.21	0.20	0.00
Q8VCF0	Mitochondrial antiviral-signaling protein OS=Mus musculus GN=Mavs PE=1 SV=1 - [MAVS_MOUSE]	-0.07	-0.10	-0.13	-0.10	0.00
Q04736	Tyrosine-protein kinase Yes OS=Mus musculus GN=Yes1 PE=1 SV=3 - [YES_MOUSE]	-0.62	-0.65	-0.37	-0.50	0.00
Q9DBM2	Peroxisomal bifunctional enzyme OS=Mus musculus GN=Ehhadh PE=1 SV=4 - [ECHP_MOUSE]	-0.32	-0.21	-0.22	-0.19	0.00
B1AUP0	Serine/threonine-protein kinase A-Raf (Fragment) OS=Mus musculus GN=Araf PE=2 SV=1 - [B1AUP0_MOUSE]	-0.60	-0.70	-0.39	-0.49	0.00
P58021	Transmembrane 9 superfamily member 2 OS=Mus musculus GN=Tm9sf2 PE=2 SV=1 - [TM9S2_MOUSE]	-0.52	-0.32	-0.56	-0.42	0.00
Q9JKD3	Secretory carrier-associated membrane protein 5 OS=Mus musculus GN=Scamp5 PE=2 SV=1 - [SCAM5_MOUSE]	-0.26	-0.33	-0.18	-0.25	0.00
E0CY15	DNA-directed RNA polymerase, mitochondrial OS=Mus musculus GN=Polrmt PE=2 SV=1 - [E0CY15_MOUSE]	-1.30	-1.68	-0.91	-1.29	0.00
D3YVA2	Neurocalcin-delta (Fragment) OS=Mus musculus GN=Ncald PE=2 SV=1 - [D3YVA2_MOUSE]	-0.33	-0.20	-0.30	-0.36	0.00
Q8K0C9	GDP-mannose 4,6 dehydratase OS=Mus musculus GN=Gmds PE=2 SV=1 - [GMDS_MOUSE]	0.14	0.16	0.24	0.21	0.00
Q9CW46	Ribonucleoprotein PTB-binding 1 OS=Mus musculus GN=Raver1 PE=1 SV=2 - [RAVR1_MOUSE]	0.08	0.12	0.12	0.08	0.00
P41105	60S ribosomal protein L28 OS=Mus musculus GN=Rpl28 PE=1 SV=2 - [RL28_MOUSE]	-0.20	-0.18	-0.12	-0.13	0.00
Q61160	Protein FADD OS=Mus musculus GN=Fadd PE=1 SV=1 - [FADD_MOUSE]	-0.19	-0.14	-0.23	-0.24	0.00
Q9WTX8	Mitotic spindle assembly checkpoint protein MAD1 OS=Mus musculus GN=Mad1l1 PE=2 SV=1 - [MD1L1_MOUSE]	0.51	0.37	0.48	0.67	0.00

Q9CQ69	Cytochrome b-c1 complex subunit 8 OS=Mus musculus GN=Uqcrq PE=1 SV=3 - [QCR8_MOUSE]	-0.48	-0.45	-0.27	-0.35	0.00
P24604-2	Isoform 2 of Tyrosine-protein kinase Tec OS=Mus musculus GN=Tec - [TEC_MOUSE]	-1.00	-0.69	-0.89	-0.58	0.00
E9PZJ7	Protein Vmn2r107 OS=Mus musculus GN=Vmn2r107 PE=2 SV=1 - [E9PZJ7_MOUSE]	-0.37	-0.54	-0.51	-0.68	0.00
P00493	Hypoxanthine-guanine phosphoribosyltransferase OS=Mus musculus GN=Hprt1 PE=1 SV=3 - [HPRT_MOUSE]	-0.21	-0.37	-0.24	-0.28	0.00
Q8BGB7	Enolase-phosphatase E1 OS=Mus musculus GN=Enoph1 PE=2 SV=1 - [ENOPH_MOUSE]	-0.45	-0.35	-0.43	-0.25	0.00
Q8CCH2	NHL repeat-containing protein 3 OS=Mus musculus GN=Nhlrc3 PE=2 SV=1 - [NHLC3_MOUSE]	-0.33	-0.19	-0.27	-0.22	0.00
Q8BTV2-2	Isoform 2 of Cleavage and polyadenylation specificity factor subunit 7 OS=Mus musculus GN=Cpsf7 - [CPSF7_MOUSE]	0.38	0.23	0.34	0.24	0.00
Q8CD10	Calcium uptake protein 2, mitochondrial OS=Mus musculus GN=Micu2 PE=1 SV=2 - [MICU2_MOUSE]	-1.11	-0.75	-0.97	-0.64	0.00
O35887	Calumenin OS=Mus musculus GN=Calu PE=1 SV=1 - [CALU_MOUSE]	0.37	0.31	0.54	0.45	0.00
Q5SFM8-2	Isoform 2 of RNA-binding protein 27 OS=Mus musculus GN=Rbm27 - [RBM27_MOUSE]	0.90	0.78	0.99	0.54	0.00
B1B0C7	Basement membrane-specific heparan sulfate proteoglycan core protein OS=Mus musculus GN=Hspg2 PE=2 SV=1 - [B1B0C7_MOUSE]	0.18	0.17	0.12	0.11	0.00
Q09199	Beta-1,4 N-acetylgalactosaminyltransferase 2 OS=Mus musculus GN=B4galnt2 PE=2 SV=1 - [B4GN2_MOUSE]	0.48	0.74	0.44	0.67	0.00
P54071	Isocitrate dehydrogenase [NADP], mitochondrial OS=Mus musculus GN=Idh2 PE=1 SV=3 - [IDHP_MOUSE]	-0.35	-0.34	-0.24	-0.21	0.00
P40124	Adenylyl cyclase-associated protein 1 OS=Mus musculus GN=Cap1 PE=1 SV=4 - [CAP1_MOUSE]	-0.48	-0.50	-0.31	-0.32	0.00
J3QNB1	La-related protein 1 OS=Mus musculus GN=Larp1 PE=4 SV=1 - [J3QNB1_MOUSE]	0.26	0.27	0.40	0.41	0.00
Q8CI51	PDZ and LIM domain protein 5 OS=Mus musculus GN=Pdlim5 PE=1 SV=4 - [PDLI5_MOUSE]	0.25	0.26	0.41	0.37	0.00
Q61475	Complement decay-accelerating factor, GPI-anchored OS=Mus musculus GN=Cd55 PE=2 SV=2 - [DAF1_MOUSE]	-1.19	-0.83	-1.03	-0.67	0.00
Q91WD0-2	Isoform 2 of Protein GPR108 OS=Mus musculus GN=Gpr108 - [GP108_MOUSE]	-1.04	-0.58	-0.89	-0.72	0.00

P28293	Cathepsin G OS=Mus musculus GN=Ctsg PE=1 SV=2 - [CATG_MOUSE]	1.43	2.04	1.13	1.74	0.00
Q8K2B3	Succinate dehydrogenase [ubiquinone] flavoprotein subunit, mitochondrial OS=Mus musculus GN=Sdha PE=1 SV=1 - [DHSA_MOUSE]	-0.20	-0.17	-0.16	-0.10	0.00
Q6PGF5	BMS1 homolog, ribosome assembly protein (Yeast) OS=Mus musculus GN=Bms1 PE=2 SV=1 - [Q6PGF5_MOUSE]	0.32	0.26	0.46	0.40	0.00
Q61625	Glutamate receptor ionotropic, delta-2 OS=Mus musculus GN=Grid2 PE=1 SV=1 - [GRID2_MOUSE]	0.76	0.89	1.19	1.31	0.00
P61750	ADP-ribosylation factor 4 OS=Mus musculus GN=Arf4 PE=2 SV=2 - [ARF4_MOUSE]	-0.38	-0.37	-0.58	-0.58	0.00
Q6P4S8	Integrator complex subunit 1 OS=Mus musculus GN=Ints1 PE=1 SV=2 - [INT1_MOUSE]	1.37	0.95	1.19	0.77	0.00
Q8R1B4	Eukaryotic translation initiation factor 3 subunit C OS=Mus musculus GN=Eif3c PE=1 SV=1 - [EIF3C_MOUSE]	-0.26	-0.20	-0.24	-0.14	0.00
Q9DD20	Methyltransferase-like protein 7B OS=Mus musculus GN=Mettl7b PE=2 SV=2 - [MET7B_MOUSE]	0.44	0.49	0.27	0.48	0.00
Q8R5H1-5	Isoform 5 of Ubiquitin carboxyl-terminal hydrolase 15 OS=Mus musculus GN=Usp15 - [UBP15_MOUSE]	0.47	0.33	0.61	0.46	0.00
Q9Z2X1-2	Isoform 2 of Heterogeneous nuclear ribonucleoprotein F OS=Mus musculus GN=Hnrnpf - [HNRPF_MOUSE]	0.28	0.28	0.44	0.41	0.00
Q6P069-2	Isoform 2 of Sorcin OS=Mus musculus GN=Sri - [SORCN_MOUSE]	-0.22	-0.19	-0.25	-0.14	0.00
Q501J2	Protein FAM173A OS=Mus musculus GN=Fam173a PE=2 SV=1 - [F173A_MOUSE]	-0.29	-0.23	-0.41	-0.35	0.00
F6WZH1	Dipeptidase 3 (Fragment) OS=Mus musculus GN=Mbd3 PE=4 SV=1 - [F6WZH1_MOUSE]	0.42	0.32	0.58	0.40	0.00
P22366	Myeloid differentiation primary response protein MyD88 OS=Mus musculus GN=Myd88 PE=1 SV=3 - [MYD88_MOUSE]	0.40	0.32	0.57	0.49	0.00
Q7TPE5	Probable RNA polymerase II nuclear localization protein SLC7A6OS OS=Mus musculus GN=Slc7a6os PE=1 SV=1 - [S7A6O_MOUSE]	0.30	0.30	0.19	0.20	0.00
Q9QYB8	Beta-adducin OS=Mus musculus GN=Add2 PE=1 SV=4 - [ADDB_MOUSE]	0.73	1.02	1.07	1.36	0.00
E9Q1T1	Calcium/calmodulin-dependent protein kinase type II subunit beta OS=Mus musculus GN=Camk2d PE=2 SV=1 - [E9Q1T1_MOUSE]	-0.18	-0.19	-0.17	-0.10	0.00
Q3V1L4	Cytosolic purine 5'-nucleotidase OS=Mus musculus GN=Nt5c2 PE=1 SV=2 - [5NTC_MOUSE]	0.43	0.43	0.65	0.69	0.00

Q9DBU5	E3 ubiquitin-protein ligase RNF6 OS=Mus musculus GN=Rnf6 PE=1 SV=1 - [RNF6_MOUSE]	0.97	0.86	0.66	0.55	0.00
Q3TJD7	PDZ and LIM domain protein 7 OS=Mus musculus GN=Pdlim7 PE=2 SV=1 - [PDLI7_MOUSE]	0.16	0.17	0.12	0.10	0.00
P62748	Hippocalcin-like protein 1 OS=Mus musculus GN=Hpcal1 PE=2 SV=2 - [HPCL1_MOUSE]	-0.38	-0.21	-0.37	-0.34	0.00
P11928	2'-5'-oligoadenylate synthase 1A OS=Mus musculus GN=Oas1a PE=2 SV=2 - [OAS1A_MOUSE]	-0.83	-0.60	-0.85	-0.51	0.00
Q8VBT0	Thioredoxin-related transmembrane protein 1 OS=Mus musculus GN=Tmx1 PE=1 SV=1 - [TMX1_MOUSE]	-0.31	-0.28	-0.17	-0.30	0.00
B1AWQ3	Espin OS=Mus musculus GN=Espn PE=2 SV=1 - [B1AWQ3_MOUSE]	0.83	0.61	1.13	0.91	0.00
Q9Z0M1	Enamel matrix serine proteinase 1 (Precursor) OS=Mus musculus GN=Klk4 PE=2 SV=1 - [Q9Z0M1_MOUSE]	-0.75	-0.76	-0.48	-0.49	0.00
P46737	Lys-63-specific deubiquitinase BRCC36 OS=Mus musculus GN=Brcc3 PE=2 SV=1 - [BRCC3_MOUSE]	0.16	0.15	0.15	0.24	0.00
A2AG06-2	Isoform 2 of Uncharacterized protein C17orf104 homolog OS=Mus musculus GN=Gm1564 - [CQ104_MOUSE]	-1.30	-1.20	-1.98	-1.89	0.00
Q3UF95	Large proline-rich protein BAG6 OS=Mus musculus GN=Bag6 PE=2 SV=1 - [Q3UF95_MOUSE]	-0.20	-0.12	-0.20	-0.24	0.00
P62141	Serine/threonine-protein phosphatase PP1-beta catalytic subunit OS=Mus musculus GN=Ppp1cb PE=1 SV=3 - [PP1B_MOUSE]	0.35	0.44	0.26	0.28	0.00
J3QN79	MCG8345 OS=Mus musculus GN=Fabp3-ps1 PE=3 SV=1 - [J3QN79_MOUSE]	0.23	0.37	0.27	0.40	0.00
Q9CZE3	Ras-related protein Rab-32 OS=Mus musculus GN=Rab32 PE=2 SV=3 - [RAB32_MOUSE]	-0.26	-0.23	-0.14	-0.25	0.00
Q8BXN9	Transmembrane protein 87A OS=Mus musculus GN=Tmem87a PE=1 SV=1 - [TM87A_MOUSE]	-0.93	-0.81	-0.54	-0.60	0.00
P11032-2	Isoform HF2 of Granzyme A OS=Mus musculus GN=Gzma - [GRAA_MOUSE]	0.57	0.89	0.72	1.03	0.00
Q99JW2	Aminoacylase-1 OS=Mus musculus GN=Acy1 PE=1 SV=1 - [ACY1_MOUSE]	-0.48	-0.48	-0.72	-0.43	0.00
F6ZSM3	Annexin OS=Mus musculus GN=Anxa13 PE=3 SV=2 - [F6ZSM3_MOUSE]	-0.50	-0.27	-0.49	-0.47	0.00
P06537-3	Isoform 1-B of Glucocorticoid receptor OS=Mus musculus GN=Nr3c1 - [GCR_MOUSE]	0.38	0.55	0.31	0.47	0.00

Q8VC28	Aldo-keto reductase family 1 member C13 OS=Mus musculus GN=Akr1c13 PE=1 SV=2 - [AK1CD_MOUSE]	0.15	0.25	0.25	0.28	0.00
Q8K1M6-3	Isoform 3 of Dynamin-1-like protein OS=Mus musculus GN=Dnm1l - [DNM1L_MOUSE]	-0.11	-0.10	-0.15	-0.09	0.00
Q6ZPY7-2	Isoform 2 of Lysine-specific demethylase 3B OS=Mus musculus GN=Kdm3b - [KDM3B_MOUSE]	0.66	0.85	1.02	1.21	0.00
Q8BGB8	Ubiquinone biosynthesis protein COQ4 homolog, mitochondrial OS=Mus musculus GN=Coq4 PE=2 SV=1 - [COQ4_MOUSE]	-1.17	-0.82	-1.54	-1.28	0.00
P62317	Small nuclear ribonucleoprotein Sm D2 OS=Mus musculus GN=Snrpd2 PE=1 SV=1 - [SMD2_MOUSE]	0.26	0.21	0.38	0.32	0.00
P51150	Ras-related protein Rab-7a OS=Mus musculus GN=Rab7a PE=1 SV=2 - [RAB7A_MOUSE]	-0.09	-0.15	-0.12	-0.16	0.00
Q8BP47	Asparagine--tRNA ligase, cytoplasmic OS=Mus musculus GN=Nars PE=1 SV=2 - [SYNC_MOUSE]	-0.24	-0.32	-0.18	-0.22	0.00
P51855	Glutathione synthetase OS=Mus musculus GN=Gss PE=2 SV=1 - [GSHB_MOUSE]	-0.17	-0.10	-0.14	-0.12	0.00
Q9CQ91	NADH dehydrogenase [ubiquinone] 1 alpha subcomplex subunit 3 OS=Mus musculus GN=Ndufa3 PE=1 SV=1 - [NDUA3_MOUSE]	-0.82	-0.53	-0.88	-0.56	0.00
Q8BK58	HSPB1-associated protein 1 OS=Mus musculus GN=Hspbap1 PE=1 SV=2 - [HBAP1_MOUSE]	0.40	0.49	0.63	0.71	0.00
F6VIY4	Crossover junction endonuclease MUS81 (Fragment) OS=Mus musculus GN=Mus81 PE=4 SV=1 - [F6VIY4_MOUSE]	1.48	1.14	1.13	0.78	0.00
Q99JP0	Mitogen-activated protein kinase kinase kinase kinase 3 OS=Mus musculus GN=Map4k3 PE=1 SV=4 - [M4K3_MOUSE]	0.68	0.52	0.96	0.79	0.00
Q99NB8	Ubiquilin-4 OS=Mus musculus GN=Ubqln4 PE=1 SV=1 - [UBQL4_MOUSE]	0.67	0.37	0.59	0.46	0.00
Q61878	Bone marrow proteoglycan OS=Mus musculus GN=Prg2 PE=1 SV=1 - [PRG2_MOUSE]	1.81	1.19	1.98	1.26	0.00
Q9Z0Z4-2	Isoform 2 of Hephaestin OS=Mus musculus GN=Heph - [HEPH_MOUSE]	-0.23	-0.16	-0.28	-0.18	0.00
Q99LT0	Protein dpy-30 homolog OS=Mus musculus GN=Dpy30 PE=1 SV=1 - [DPY30_MOUSE]	0.32	0.48	0.46	0.61	0.00
F6ZDT4	Ribonuclease H2 subunit C (Fragment) OS=Mus musculus GN=Rnaseh2c PE=4 SV=1 - [F6ZDT4_MOUSE]	0.65	0.61	1.04	0.79	0.00
Q9DBR0	A-kinase anchor protein 8 OS=Mus musculus GN=Akap8 PE=1 SV=1 - [AKAP8_MOUSE]	0.28	0.27	0.38	0.46	0.00

P49182	Heparin cofactor 2 OS=Mus musculus GN=Serpind1 PE=1 SV=1 - [HEP2_MOUSE]	0.46	0.49	0.29	0.32	0.00
Q6NZF1	Zinc finger CCCH domain-containing protein 11A OS=Mus musculus GN=Zc3h11a PE=1 SV=1 - [ZC11A_MOUSE]	0.72	1.00	0.83	1.28	0.00
D3YVV1	Cell cycle control protein 50A OS=Mus musculus GN=Tmem30a PE=2 SV=1 - [D3YVV1_MOUSE]	0.34	0.23	0.30	0.19	0.00
P16858	Glyceraldehyde-3-phosphate dehydrogenase OS=Mus musculus GN=Gapdh PE=1 SV=2 - [G3P_MOUSE]	-0.37	-0.50	-0.27	-0.38	0.00
O35400	Sulfotransferase family cytosolic 2B member 1 OS=Mus musculus GN=Sult2b1 PE=2 SV=2 - [ST2B1_MOUSE]	-0.27	-0.27	-0.15	-0.20	0.00
Q9DBJ3	Brain-specific angiogenesis inhibitor 1-associated protein 2-like protein 1 OS=Mus musculus GN=Baiap2l1 PE=1 SV=1 - [BI2L1_MOUSE]	0.22	0.22	0.17	0.12	0.00
Q91YP0	L-2-hydroxyglutarate dehydrogenase, mitochondrial OS=Mus musculus GN=L2hgdh PE=2 SV=1 - [L2HDH_MOUSE]	-0.30	-0.27	-0.31	-0.46	0.00
Q8BTG6	UPF0454 protein C12orf49 homolog OS=Mus musculus PE=2 SV=1 - [CL049_MOUSE]	0.52	0.38	0.72	0.57	0.00
P28654	Decorin OS=Mus musculus GN=Dcn PE=2 SV=1 - [PGS2_MOUSE]	0.41	0.40	0.61	0.64	0.00
O88273	Gremlin-2 OS=Mus musculus GN=Grem2 PE=1 SV=1 - [GREM2_MOUSE]	-0.52	-0.35	-0.67	-0.51	0.00
E9QPZ4	TD and POZ domain-containing protein 1 OS=Mus musculus GN=Tdpoz1 PE=4 SV=1 - [E9QPZ4_MOUSE]	-1.30	-2.05	-1.51	-2.26	0.00
Q8BMS7	Calcium-transporting ATPase type 2C member 1 OS=Mus musculus GN=Atp2c1 PE=2 SV=1 - [Q8BMS7_MOUSE]	-0.14	-0.14	-0.22	-0.22	0.00
Q8BZN6	Dedicator of cytokinesis protein 10 OS=Mus musculus GN=Dock10 PE=1 SV=3 - [DOC10_MOUSE]	0.51	0.81	0.58	0.88	0.00
P57746	V-type proton ATPase subunit D OS=Mus musculus GN=Atp6v1d PE=1 SV=1 - [VATD_MOUSE]	-0.29	-0.30	-0.44	-0.47	0.00
Q6PGL7	WASH complex subunit FAM21 OS=Mus musculus GN=Fam21 PE=1 SV=1 - [FAM21_MOUSE]	0.75	0.69	1.20	0.93	0.00
Q8VEN2-2	Isoform 2 of Placenta-expressed transcript 1 protein OS=Mus musculus GN=Plet1 - [PLET1_MOUSE]	1.22	0.93	1.73	1.44	0.00
Q7TPS5-3	Isoform 3 of C2 domain-containing protein 5 OS=Mus musculus GN=C2cd5 - [C2CD5_MOUSE]	1.35	1.66	0.88	1.19	0.00
D3YUJ6	Protein Gm7792 OS=Mus musculus GN=Gm7792 PE=4 SV=2 - [D3YUJ6_MOUSE]	-0.67	-0.44	-0.82	-0.59	0.00

Q1W617-2	Isoform 2 of Protein Shroom4 OS=Mus musculus GN=Shroom4 - [SHRM4_MOUSE]	0.79	1.20	1.09	1.50	0.00
Q9CZU6	Citrate synthase, mitochondrial OS=Mus musculus GN=Cs PE=1 SV=1 - [CISY_MOUSE]	-0.29	-0.36	-0.19	-0.27	0.00
Q99JR8-2	Isoform 2 of SWI/SNF-related matrix-associated actin-dependent regulator of chromatin subfamily D member 2 OS=Mus musculus GN=Smarcd2 - [SMRD2_MOUSE]	0.58	0.72	0.38	0.52	0.00
Q99LJ0	CTTNBP2 N-terminal-like protein OS=Mus musculus GN=Cttnbp2nl PE=1 SV=1 - [CT2NL_MOUSE]	1.12	1.04	1.67	1.70	0.00
Q78IK4	Apolipoprotein O-like OS=Mus musculus GN=Apool PE=2 SV=1 - [APOOL_MOUSE]	-0.53	-0.50	-0.34	-0.32	0.00
Q8BP01	Vimentin-type intermediate filament-associated coiled-coil protein OS=Mus musculus GN=Vmac PE=2 SV=1 - [VMAC_MOUSE]	0.23	0.37	0.22	0.33	0.00
E9PVB3	Coiled-coil domain-containing protein 175 OS=Mus musculus GN=Ccdc175 PE=2 SV=1 - [CC175_MOUSE]	-0.36	-0.47	-0.25	-0.36	0.00
D3YZY4	Retinoic acid receptor gamma (Fragment) OS=Mus musculus GN=Rarg PE=2 SV=1 - [D3YZY4_MOUSE]	-0.73	-0.59	-0.52	-0.39	0.00
Q64337-2	Isoform 2 of Sequestosome-1 OS=Mus musculus GN=Sqstm1 - [SQSTM_MOUSE]	0.32	0.39	0.58	0.45	0.00
Q3UEB3-2	Isoform 2 of Poly(U)-binding-splicing factor PUF60 OS=Mus musculus GN=Puf60 - [PUF60_MOUSE]	0.28	0.41	0.40	0.54	0.00
P24547	Inosine-5'-monophosphate dehydrogenase 2 OS=Mus musculus GN=Impdh2 PE=1 SV=2 - [IMDH2_MOUSE]	0.19	0.18	0.10	0.20	0.00
Q61738-3	Isoform Alpha-7X1B of Integrin alpha-7 OS=Mus musculus GN=Itga7 - [ITA7_MOUSE]	-0.29	-0.25	-0.41	-0.43	0.00
Q61493	DNA polymerase zeta catalytic subunit OS=Mus musculus GN=Rev3l PE=1 SV=3 - [DPOLZ_MOUSE]	-0.41	-0.51	-0.27	-0.37	0.00
P58044	Isopentenyl-diphosphate Delta-isomerase 1 OS=Mus musculus GN=Idi1 PE=2 SV=1 - [IDI1_MOUSE]	-0.60	-0.42	-0.66	-0.39	0.00
P35487	Pyruvate dehydrogenase E1 component subunit alpha, testis-specific form, mitochondrial OS=Mus musculus GN=Pdha2 PE=2 SV=1 - [ODPAT_MOUSE]	0.92	1.10	0.57	1.02	0.00
P14847	C-reactive protein OS=Mus musculus GN=Crp PE=2 SV=2 - [CRP_MOUSE]	0.59	0.83	0.88	1.12	0.00
D3Z3D2	Nuclear cap-binding protein subunit 2 OS=Mus musculus GN=Ncbp2 PE=2 SV=1 - [D3Z3D2_MOUSE]	0.10	0.08	0.15	0.10	0.00
Q8R2M2	Deoxynucleotidyltransferase terminal-interacting protein 2 OS=Mus musculus GN=Dnttip2 PE=1 SV=1 - [TDIF2_MOUSE]	0.58	0.42	0.47	0.31	0.00

Q80UG5	Septin-9 OS=Mus musculus GN=Sept9 PE=1 SV=1 - [SEPT9_MOUSE]	0.38	0.31	0.48	0.27	0.00
Q80X95	Ras-related GTP-binding protein A OS=Mus musculus GN=Rraga PE=2 SV=1 - [RRAGA_MOUSE]	-0.27	-0.17	-0.33	-0.30	0.00
P28271	Cytoplasmic aconitate hydratase OS=Mus musculus GN=Aco1 PE=1 SV=3 - [ACOC_MOUSE]	-0.08	-0.08	-0.11	-0.13	0.00
Q8BFY6	Peflin OS=Mus musculus GN=Pef1 PE=2 SV=1 - [PEF1_MOUSE]	-0.24	-0.27	-0.27	-0.14	0.00
D3YUR8	Protein 0610007P14Rik (Fragment) OS=Mus musculus GN=0610007P14Rik PE=2 SV=1 - [D3YUR8_MOUSE]	-0.49	-0.61	-0.76	-0.89	0.00
O88895	Histone deacetylase 3 OS=Mus musculus GN=Hdac3 PE=1 SV=1 - [HDAC3_MOUSE]	0.33	0.28	0.44	0.26	0.00
Q8JZR0	Long-chain-fatty-acid--CoA ligase 5 OS=Mus musculus GN=Acsl5 PE=2 SV=1 - [ACSL5_MOUSE]	-0.64	-0.41	-0.53	-0.36	0.00
Q8CHY6	Transcriptional repressor p66 alpha OS=Mus musculus GN=Gatad2a PE=1 SV=2 - [P66A_MOUSE]	0.92	0.58	1.08	0.77	0.00
P0CW02	Lymphocyte antigen 6C1 OS=Mus musculus GN=Ly6c1 PE=2 SV=1 - [LY6C1_MOUSE]	0.44	0.29	0.27	0.26	0.00
Q9Z0X1	Apoptosis-inducing factor 1, mitochondrial OS=Mus musculus GN=Aifm1 PE=1 SV=1 - [AIFM1_MOUSE]	-0.28	-0.34	-0.18	-0.25	0.00
P49615	Cyclin-dependent kinase 5 OS=Mus musculus GN=Cdk5 PE=1 SV=1 - [CDK5_MOUSE]	-0.55	-0.47	-0.34	-0.32	0.00
Q9EP71	Ankycorbin OS=Mus musculus GN=Rai14 PE=1 SV=1 - [RAI14_MOUSE]	0.33	0.50	0.30	0.49	0.00
Q9CPP0	Nucleoplasmin-3 OS=Mus musculus GN=Npm3 PE=2 SV=3 - [NPM3_MOUSE]	0.43	0.57	0.30	0.44	0.00
Q8VHM5	Heterogeneous nuclear ribonucleoprotein R OS=Mus musculus GN=Hnrnpr PE=2 SV=1 - [Q8VHM5_MOUSE]	0.13	0.10	0.18	0.18	0.00
Q6GQS1	Calcium-binding mitochondrial carrier protein SCaMC-3 OS=Mus musculus GN=Slc25a23 PE=2 SV=1 - [SCMC3_MOUSE]	-0.35	-0.55	-0.35	-0.56	0.00
P47809	Dual specificity mitogen-activated protein kinase kinase 4 OS=Mus musculus GN=Map2k4 PE=1 SV=2 - [MP2K4_MOUSE]	-0.44	-0.25	-0.32	-0.29	0.00
Q9R098	Hepatocyte growth factor activator OS=Mus musculus GN=Hgfac PE=1 SV=1 - [HGFA_MOUSE]	-0.58	-0.38	-0.33	-0.43	0.00
E9Q512	Protein Trip11 OS=Mus musculus GN=Trip11 PE=2 SV=1 - [E9Q512_MOUSE]	0.59	0.50	0.32	0.41	0.00

Q9D8B3	Charged multivesicular body protein 4b OS=Mus musculus GN=Chmp4b PE=2 SV=2 - [CHM4B_MOUSE]	0.29	0.28	0.46	0.43	0.00
Q8CFQ9	Fusion, derived from t(1216) malignant liposarcoma (Human) OS=Mus musculus GN=Fus PE=2 SV=1 - [Q8CFQ9_MOUSE]	0.36	0.22	0.43	0.37	0.00
B8JK39	Protein Itga9 OS=Mus musculus GN=Itga9 PE=2 SV=1 - [B8JK39_MOUSE]	-0.58	-0.36	-0.69	-0.51	0.00
Q9JI39	ATP-binding cassette sub-family B member 10, mitochondrial OS=Mus musculus GN=Abcb10 PE=2 SV=1 - [ABCBA_MOUSE]	-0.47	-0.32	-0.47	-0.28	0.00
O70439	Syntaxin-7 OS=Mus musculus GN=Stx7 PE=1 SV=3 - [STX7_MOUSE]	0.25	0.18	0.13	0.19	0.00
P70349	Histidine triad nucleotide-binding protein 1 OS=Mus musculus GN=Hint1 PE=1 SV=3 - [HINT1_MOUSE]	-0.42	-0.38	-0.29	-0.23	0.00
P07724	Serum albumin OS=Mus musculus GN=Alb PE=1 SV=3 - [ALBU_MOUSE]	0.26	0.24	0.41	0.37	0.00
P58389	Serine/threonine-protein phosphatase 2A activator OS=Mus musculus GN=Ppp2r4 PE=1 SV=1 - [PTPA_MOUSE]	-0.36	-0.24	-0.36	-0.22	0.00
Q6A0A2	La-related protein 4B OS=Mus musculus GN=Larp4b PE=1 SV=2 - [LAR4B_MOUSE]	0.44	0.70	0.41	0.62	0.00
P70232-2	Isoform 2 of Neural cell adhesion molecule L1-like protein OS=Mus musculus GN=Chl1 - [CHL1_MOUSE]	0.31	0.41	0.57	0.52	0.00
Q9CX56	26S proteasome non-ATPase regulatory subunit 8 OS=Mus musculus GN=Psmd8 PE=1 SV=2 - [PSMD8_MOUSE]	-0.43	-0.32	-0.44	-0.25	0.00
Q8K2I4	Beta-mannosidase OS=Mus musculus GN=Manba PE=2 SV=1 - [MANBA_MOUSE]	-0.84	-0.95	-0.63	-0.53	0.00
E9PVU0	Unconventional myosin-VI OS=Mus musculus GN=Myo6 PE=2 SV=1 - [E9PVU0_MOUSE]	0.57	0.63	0.85	0.46	0.00
D3Z5T2	Armadillo repeat-containing protein 10 OS=Mus musculus GN=Armc10 PE=2 SV=1 - [D3Z5T2_MOUSE]	-0.62	-0.61	-0.46	-0.34	0.00
P16014	Secretogranin-1 OS=Mus musculus GN=Chgb PE=1 SV=2 - [SCG1_MOUSE]	0.20	0.37	0.37	0.39	0.00
D3YTN4	GTPase IMAP family member 4 OS=Mus musculus GN=Gimap4 PE=2 SV=1 - [D3YTN4_MOUSE]	-0.45	-0.43	-0.33	-0.25	0.00
Q8BHE8	Uncharacterized protein C2orf47 homolog, mitochondrial OS=Mus musculus PE=2 SV=1 - [CB047_MOUSE]	-0.62	-0.40	-0.75	-0.74	0.00
O35216	Histone H3-like centromeric protein A OS=Mus musculus GN=Cenpa PE=2 SV=1 - [CENPA_MOUSE]	0.28	0.40	0.21	0.33	0.00

Q8C5W0-2	Isoform 2 of Calmin OS=Mus musculus GN=Clmn - [CLMN_MOUSE]	0.50	0.47	0.26	0.43	0.00
Q9QZE5	Coatomer subunit gamma-1 OS=Mus musculus GN=Copg1 PE=2 SV=1 - [COPG1_MOUSE]	-0.16	-0.21	-0.28	-0.30	0.00
O54984	ATPase Asna1 OS=Mus musculus GN=Asna1 PE=1 SV=2 - [ASNA_MOUSE]	-0.28	-0.39	-0.45	-0.26	0.00
Q01149	Collagen alpha-2(I) chain OS=Mus musculus GN=Col1a2 PE=2 SV=2 - [CO1A2_MOUSE]	0.27	0.34	0.49	0.44	0.00
B1AXC8	Coiled-coil-helix-coiled-coil-helix domain-containing protein 7 (Fragment) OS=Mus musculus GN=Chchd7 PE=2 SV=1 - [B1AXC8_MOUSE]	-0.16	-0.21	-0.30	-0.28	0.00
Q91W34	UPF0420 protein C16orf58 homolog OS=Mus musculus PE=2 SV=1 - [CP058_MOUSE]	-0.16	-0.15	-0.26	-0.21	0.00
P98083-2	Isoform p52Shc of SHC-transforming protein 1 OS=Mus musculus GN=Shc1 - [SHC1_MOUSE]	0.19	0.18	0.18	0.29	0.00
Q8BPM0-2	Isoform 2 of Disheveled-associated activator of morphogenesis 1 OS=Mus musculus GN=Daam1 - [DAAM1_MOUSE]	-0.55	-0.57	-0.43	-0.80	0.00
P41241	Tyrosine-protein kinase CSK OS=Mus musculus GN=Csk PE=1 SV=2 - [CSK_MOUSE]	-0.15	-0.27	-0.19	-0.24	0.00
P61924	Coatomer subunit zeta-1 OS=Mus musculus GN=Copz1 PE=2 SV=1 - [COPZ1_MOUSE]	0.12	0.14	0.08	0.16	0.00
Q99NB1	Acetyl-coenzyme A synthetase 2-like, mitochondrial OS=Mus musculus GN=Acss1 PE=1 SV=1 - [ACS2L_MOUSE]	-0.31	-0.27	-0.48	-0.37	0.00
O88746	Target of Myb protein 1 OS=Mus musculus GN=Tom1 PE=1 SV=1 - [TOM1_MOUSE]	0.48	0.65	0.34	0.50	0.00
Q9ERE7	LDLR chaperone MESD OS=Mus musculus GN=Mesdc2 PE=1 SV=1 - [MESD_MOUSE]	0.23	0.18	0.33	0.30	0.00
P39039	Mannose-binding protein A OS=Mus musculus GN=Mbl1 PE=2 SV=1 - [MBL1_MOUSE]	0.70	0.94	0.48	0.71	0.00
Q91ZF4	Cathepsin Q (Precursor) OS=Mus musculus GN=Ctsq PE=2 SV=1 - [Q91ZF4_MOUSE]	0.34	0.22	0.33	0.20	0.00
P30678	Guanine nucleotide-binding protein subunit alpha-15 OS=Mus musculus GN=Gna15 PE=2 SV=1 - [GNA15_MOUSE]	0.57	0.91	0.56	0.90	0.00
Q6P8U6	Pancreatic triacylglycerol lipase OS=Mus musculus GN=Pnlip PE=1 SV=1 - [LIPP_MOUSE]	0.51	0.39	0.42	0.26	0.00
Q6PGB6-3	Isoform 3 of N-alpha-acetyltransferase 50 OS=Mus musculus GN=Naa50 - [NAA50_MOUSE]	-0.51	-0.29	-0.34	-0.34	0.00

O35691	Pinin OS=Mus musculus GN=Pnn PE=1 SV=4 - [PININ_MOUSE]	0.69	0.47	0.91	0.69	0.00
Q3LS21	Potassium channel subfamily K member 9 OS=Mus musculus GN=Kcnk9 PE=2 SV=1 - [KCNK9_MOUSE]	0.29	0.18	0.33	0.22	0.00
P26048	Gamma-aminobutyric acid receptor subunit alpha-2 OS=Mus musculus GN=Gabra2 PE=1 SV=1 - [GBRA2_MOUSE]	0.36	0.26	0.51	0.41	0.00
Q91ZA3	Propionyl-CoA carboxylase alpha chain, mitochondrial OS=Mus musculus GN=Pcca PE=2 SV=2 - [PCCA_MOUSE]	-0.21	-0.17	-0.18	-0.11	0.00
A2ABY3	Ethanolamine-phosphate cytidylyltransferase OS=Mus musculus GN=Pcyt2 PE=4 SV=1 - [A2ABY3_MOUSE]	0.36	0.31	0.18	0.27	0.00
P14873	Microtubule-associated protein 1B OS=Mus musculus GN=Map1b PE=1 SV=2 - [MAP1B_MOUSE]	0.50	0.37	0.63	0.37	0.00
Q60865	Caprin-1 OS=Mus musculus GN=Caprin1 PE=1 SV=2 - [CAPR1_MOUSE]	0.38	0.52	0.66	0.72	0.00
Q80XS7	Protein FAM83E OS=Mus musculus GN=Fam83e PE=2 SV=2 - [FA83E_MOUSE]	0.66	0.46	0.68	0.39	0.00
P21126	Ubiquitin-like protein 4A OS=Mus musculus GN=Ubl4a PE=2 SV=1 - [UBL4A_MOUSE]	0.40	0.30	0.43	0.57	0.00
P01631	Ig kappa chain V-II region 26-10 OS=Mus musculus PE=1 SV=1 - [KV2A7_MOUSE]	-0.73	-0.47	-0.90	-0.64	0.00
P63094-2	Isoform Gnas-2 of Guanine nucleotide-binding protein G(s) subunit alpha isoforms short OS=Mus musculus GN=Gnas - [GNAS2_MOUSE]	-0.58	-0.33	-0.58	-0.40	0.00
Q6PDN3-3	Isoform 3 of Myosin light chain kinase, smooth muscle OS=Mus musculus GN=Mylk - [MYLK_MOUSE]	0.20	0.17	0.31	0.27	0.00
P46978	Dolichyl-diphosphooligosaccharide--protein glycosyltransferase subunit STT3A OS=Mus musculus GN=Stt3a PE=1 SV=1 - [STT3A_MOUSE]	-0.42	-0.22	-0.42	-0.36	0.00
O08573-2	Isoform Short of Galectin-9 OS=Mus musculus GN=Lgals9 - [LEG9_MOUSE]	0.83	0.73	0.54	0.46	0.00
Q920R0	Alsin OS=Mus musculus GN=Als2 PE=1 SV=3 - [ALS2_MOUSE]	1.90	3.02	2.57	3.66	0.00
D3Z723	Nuclear transcription factor Y subunit beta (Fragment) OS=Mus musculus GN=Nfyb PE=2 SV=1 - [D3Z723_MOUSE]	0.56	0.67	0.35	0.46	0.00
Q99LA2	Protein Zfp207 OS=Mus musculus GN=Zfp207 PE=2 SV=1 - [Q99LA2_MOUSE]	0.34	0.32	0.21	0.41	0.00
Q8VDU4	Proto-oncogene vav OS=Mus musculus GN=Vav1 PE=2 SV=1 - [Q8VDU4_MOUSE]	-0.60	-0.45	-0.86	-0.71	0.00

Q8K273	Membrane magnesium transporter 1 OS=Mus musculus GN=Mmgt1 PE=2 SV=1 - [MMGT1_MOUSE]	-0.37	-0.40	-0.61	-0.62	0.00
P61971	Nuclear transport factor 2 OS=Mus musculus GN=Nutf2 PE=2 SV=1 - [NTF2_MOUSE]	-0.10	-0.10	-0.10	-0.05	0.00
Q91YW3	DnaJ homolog subfamily C member 3 OS=Mus musculus GN=Dnajc3 PE=1 SV=1 - [DNJC3_MOUSE]	0.24	0.24	0.16	0.31	0.00
P61079	Ubiquitin-conjugating enzyme E2 D3 OS=Mus musculus GN=Ube2d3 PE=1 SV=1 - [UB2D3_MOUSE]	-0.69	-0.62	-0.92	-0.48	0.00
Q921S7	39S ribosomal protein L37, mitochondrial OS=Mus musculus GN=Mrpl37 PE=2 SV=1 - [RM37_MOUSE]	-0.22	-0.14	-0.25	-0.28	0.00
A2AGR1	MAP kinase-activating death domain protein OS=Mus musculus GN=Madd PE=2 SV=1 - [A2AGR1_MOUSE]	0.56	0.66	0.35	0.45	0.00
Q148W0	Probable phospholipid-transporting ATPase IC OS=Mus musculus GN=Atp8b1 PE=2 SV=2 - [AT8B1_MOUSE]	-0.28	-0.20	-0.38	-0.27	0.00
P62264	40S ribosomal protein S14 OS=Mus musculus GN=Rps14 PE=2 SV=3 - [RS14_MOUSE]	-0.13	-0.13	-0.22	-0.19	0.00
Q8BJW6	Eukaryotic translation initiation factor 2A OS=Mus musculus GN=Eif2a PE=2 SV=2 - [EIF2A_MOUSE]	0.18	0.14	0.26	0.17	0.00
Q9QZD8	Mitochondrial dicarboxylate carrier OS=Mus musculus GN=Slc25a10 PE=2 SV=2 - [DIC_MOUSE]	-0.61	-0.54	-0.41	-0.33	0.00
Q99JX7	Nuclear RNA export factor 1 OS=Mus musculus GN=Nxf1 PE=1 SV=3 - [NXF1_MOUSE]	0.14	0.07	0.13	0.12	0.00
P46735	Unconventional myosin-Ib OS=Mus musculus GN=Myo1b PE=2 SV=3 - [MYO1B_MOUSE]	-0.11	-0.19	-0.11	-0.16	0.00
G5E846	Peripherin OS=Mus musculus GN=Prph PE=3 SV=1 - [G5E846_MOUSE]	0.11	0.05	0.09	0.08	0.00
D3YWD5	Major facilitator superfamily domain-containing protein 10 (Fragment) OS=Mus musculus GN=Mfsd10 PE=2 SV=1 - [D3YWD5_MOUSE]	0.45	0.58	0.30	0.42	0.00
Q8CHH9	Septin-8 OS=Mus musculus GN=Sept8 PE=1 SV=4 - [SEPT8_MOUSE]	0.43	0.51	0.27	0.53	0.00
Q8N7N5-2	Isoform 2 of DDB1- and CUL4-associated factor 8 OS=Mus musculus GN=Dcaf8 - [DCAF8_MOUSE]	0.38	0.34	0.59	0.56	0.00
Q9JJ26	Pyrin OS=Mus musculus GN=Mefv PE=2 SV=2 - [MEFV_MOUSE]	0.34	0.42	0.56	0.63	0.00
E9PUK8	Ephrin type-A receptor 6 OS=Mus musculus GN=Epha6 PE=2 SV=1 - [E9PUK8_MOUSE]	0.06	0.04	0.06	0.04	0.00

O70305-3	Isoform 3 of Ataxin-2 OS=Mus musculus GN=Atxn2 - [ATX2_MOUSE]	0.62	0.42	0.82	0.56	0.00
Q64737	Trifunctional purine biosynthetic protein adenosine-3 OS=Mus musculus GN=Gart PE=2 SV=3 - [PUR2_MOUSE]	-0.38	-0.36	-0.32	-0.19	0.00
Q922J3	CAP-Gly domain-containing linker protein 1 OS=Mus musculus GN=Clip1 PE=1 SV=1 - [CLIP1_MOUSE]	0.16	0.29	0.27	0.31	0.01
Q9JIA7	Sphingosine kinase 2 OS=Mus musculus GN=Sphk2 PE=1 SV=2 - [SPHK2_MOUSE]	-0.29	-0.23	-0.44	-0.31	0.01
Q9D051	Pyruvate dehydrogenase E1 component subunit beta, mitochondrial OS=Mus musculus GN=Pdhb PE=1 SV=1 - [ODPB_MOUSE]	-0.35	-0.31	-0.19	-0.23	0.01
J3QMQ6	Protein Gm3336 OS=Mus musculus GN=Gm3336 PE=4 SV=1 - [J3QMQ6_MOUSE]	-0.89	-0.57	-0.84	-0.51	0.01
Q99JI4	26S proteasome non-ATPase regulatory subunit 6 OS=Mus musculus GN=Psmd6 PE=1 SV=1 - [PSMD6_MOUSE]	-0.47	-0.32	-0.38	-0.24	0.01
Q60520	Paired amphipathic helix protein Sin3a OS=Mus musculus GN=Sin3a PE=1 SV=3 - [SIN3A_MOUSE]	0.19	0.24	0.14	0.27	0.01
A2ADZ8	IQ domain-containing protein C OS=Mus musculus GN=Iqcc PE=2 SV=1 - [IQCC_MOUSE]	-0.58	-0.49	-0.39	-0.31	0.01
P30115	Glutathione S-transferase A3 OS=Mus musculus GN=Gsta3 PE=1 SV=2 - [GSTA3_MOUSE]	-0.31	-0.44	-0.24	-0.27	0.01
P52825	Carnitine O-palmitoyltransferase 2, mitochondrial OS=Mus musculus GN=Cpt2 PE=1 SV=2 - [CPT2_MOUSE]	-0.08	-0.06	-0.08	-0.12	0.01
Q91YE7-2	Isoform 2 of RNA-binding protein 5 OS=Mus musculus GN=Rbm5 - [RBM5_MOUSE]	0.30	0.35	0.49	0.54	0.01
Q9CQH0	PDZK1-interacting protein 1 OS=Mus musculus GN=Pdzk1ip1 PE=2 SV=1 - [PDZ1I_MOUSE]	-0.86	-0.67	-1.27	-1.09	0.01
P31428	Dipeptidase 1 OS=Mus musculus GN=Dpep1 PE=1 SV=2 - [DPEP1_MOUSE]	0.11	0.21	0.14	0.18	0.01
O35309	N-myc-interactor OS=Mus musculus GN=Nmi PE=2 SV=1 - [NMI_MOUSE]	0.28	0.17	0.16	0.25	0.01
Q3UJ22	Gene model 648, (NCBI) OS=Mus musculus GN=Gm648 PE=2 SV=1 - [Q3UJ22_MOUSE]	0.72	0.86	0.86	1.30	0.01
Q9D7F7	Charged multivesicular body protein 4c OS=Mus musculus GN=Chmp4c PE=2 SV=1 - [CHM4C_MOUSE]	0.64	0.43	0.79	0.83	0.01
F8WI30	Sorting nexin-7 OS=Mus musculus GN=Snx7 PE=2 SV=1 - [F8WI30_MOUSE]	0.23	0.43	0.34	0.43	0.01

Q80V85	Tripartite motif-containing protein 62 OS=Mus musculus GN=Trim62 PE=2 SV=1 - [TRI62_MOUSE]	0.68	1.12	0.80	1.23	0.01
Q61081	Hsp90 co-chaperone Cdc37 OS=Mus musculus GN=Cdc37 PE=2 SV=1 - [CDC37_MOUSE]	-0.44	-0.49	-0.33	-0.27	0.01
Q921G8	Gamma-tubulin complex component 2 OS=Mus musculus GN=Tubgcp2 PE=2 SV=2 - [GCP2_MOUSE]	-0.17	-0.17	-0.20	-0.29	0.01
Q5NC83	Uncharacterized protein C7orf72 homolog OS=Mus musculus PE=2 SV=2 - [CG072_MOUSE]	-0.53	-0.42	-0.38	-0.27	0.01
Q99LI2	Chloride channel CLIC-like protein 1 OS=Mus musculus GN=Clcc1 PE=1 SV=1 - [CLCC1_MOUSE]	0.46	0.41	0.72	0.65	0.01
Q9DBG1	Sterol 26-hydroxylase, mitochondrial OS=Mus musculus GN=Cyp27a1 PE=1 SV=1 - [CP27A_MOUSE]	-1.17	-0.71	-1.31	-0.86	0.01
Q91V01	Lysophospholipid acyltransferase 5 OS=Mus musculus GN=Lpcat3 PE=1 SV=1 - [MBOA5_MOUSE]	-0.73	-0.52	-0.57	-0.37	0.01
Q8BH74	Nuclear pore complex protein Nup107 OS=Mus musculus GN=Nup107 PE=2 SV=1 - [NU107_MOUSE]	0.62	0.44	0.49	0.31	0.01
Q8VHE0	Translocation protein SEC63 homolog OS=Mus musculus GN=Sec63 PE=1 SV=4 - [SEC63_MOUSE]	-0.15	-0.09	-0.08	-0.12	0.01
Q8JZN1	Macrophage galactose N-acetyl-galactosamine specific lectin 2 OS=Mus musculus GN=Mgl2 PE=2 SV=1 - [Q8JZN1_MOUSE]	0.44	0.23	0.47	0.41	0.01
Q64378	Peptidyl-prolyl cis-trans isomerase FKBP5 OS=Mus musculus GN=Fkbp5 PE=1 SV=1 - [FKBP5_MOUSE]	-0.33	-0.32	-0.18	-0.23	0.01
Q8VCZ6	Small G protein signaling modulator 3 OS=Mus musculus GN=Sgsm3 PE=1 SV=1 - [SGSM3_MOUSE]	-0.55	-0.57	-0.96	-0.83	0.01
Q9CQZ5	NADH dehydrogenase [ubiquinone] 1 alpha subcomplex subunit 6 OS=Mus musculus GN=Ndufa6 PE=1 SV=1 - [NDUA6_MOUSE]	-1.07	-0.98	-0.65	-0.63	0.01
Q9DCL9	Multifunctional protein ADE2 OS=Mus musculus GN=Paics PE=1 SV=4 - [PUR6_MOUSE]	-0.20	-0.16	-0.22	-0.11	0.01
Q3U0V2	Tumor necrosis factor receptor type 1-associated DEATH domain protein OS=Mus musculus GN=Tradd PE=1 SV=1 - [TRADD_MOUSE]	-0.36	-0.54	-0.73	-0.55	0.01
P62717	60S ribosomal protein L18a OS=Mus musculus GN=Rpl18a PE=1 SV=1 - [RL18A_MOUSE]	-0.42	-0.26	-0.52	-0.36	0.01
Q9D8W5	26S proteasome non-ATPase regulatory subunit 12 OS=Mus musculus GN=Psmd12 PE=1 SV=4 - [PSD12_MOUSE]	-0.27	-0.23	-0.28	-0.14	0.01
E9PXJ0	Neuronal tyrosine-phosphorylated phosphoinositide-3-kinase adapter 1 (Fragment) OS=Mus musculus GN=Nyap1 PE=2 SV=1 - [E9PXJ0_MOUSE]	0.25	0.16	0.25	0.15	0.01

Q99MN9	Propionyl-CoA carboxylase beta chain, mitochondrial OS=Mus musculus GN=Pccb PE=1 SV=2 - [PCCB_MOUSE]	-0.23	-0.27	-0.16	-0.16	0.01
Q9Z1P6	NADH dehydrogenase [ubiquinone] 1 alpha subcomplex subunit 7 OS=Mus musculus GN=Ndufa7 PE=1 SV=3 - [NDUA7_MOUSE]	-0.53	-0.66	-0.34	-0.44	0.01
P84089	Enhancer of rudimentary homolog OS=Mus musculus GN=Erh PE=1 SV=1 - [ERH_MOUSE]	0.50	0.42	0.77	0.64	0.01
Q62469	Integrin alpha-2 OS=Mus musculus GN=Itga2 PE=1 SV=2 - [ITA2_MOUSE]	0.30	0.20	0.18	0.18	0.01
P14483	H-2 class II histocompatibility antigen, A beta chain OS=Mus musculus GN=H2-Ab1 PE=1 SV=1 - [HB2A_MOUSE]	-0.13	-0.07	-0.12	-0.10	0.01
Q61543	Golgi apparatus protein 1 OS=Mus musculus GN=Glg1 PE=1 SV=1 - [GSLG1_MOUSE]	-0.08	-0.12	-0.15	-0.16	0.01
Q6PHS9-4	Isoform 4 of Voltage-dependent calcium channel subunit alpha-2/delta-2 OS=Mus musculus GN=Cacna2d2 - [CA2D2_MOUSE]	0.27	0.26	0.17	0.16	0.01
P23116	Eukaryotic translation initiation factor 3 subunit A OS=Mus musculus GN=Eif3a PE=1 SV=5 - [EIF3A_MOUSE]	-0.10	-0.15	-0.20	-0.16	0.01
Q8R1G6	PDZ and LIM domain protein 2 OS=Mus musculus GN=Pdlim2 PE=1 SV=1 - [PDLI2_MOUSE]	1.49	1.17	1.07	0.75	0.01
A2AKH7	Leucine-rich repeat-containing protein 57 (Fragment) OS=Mus musculus GN=Lrrc57 PE=2 SV=1 - [A2AKH7_MOUSE]	-0.46	-0.42	-0.29	-0.26	0.01
O08807	Peroxiredoxin-4 OS=Mus musculus GN=Prdx4 PE=1 SV=1 - [PRDX4_MOUSE]	0.11	0.16	0.09	0.13	0.01
Q8K2F0	Bromodomain-containing protein 3 OS=Mus musculus GN=Brd3 PE=1 SV=2 - [BRD3_MOUSE]	0.52	0.45	0.79	0.79	0.01
Q9WTP9	Ventral anterior homeobox 2 OS=Mus musculus GN=Vax2 PE=1 SV=1 - [VAX2_MOUSE]	-0.10	-0.11	-0.16	-0.17	0.01
Q01341	Adenylate cyclase type 6 OS=Mus musculus GN=Adcy6 PE=1 SV=1 - [ADCY6_MOUSE]	-0.29	-0.50	-0.43	-0.59	0.01
Q9WUP7-2	Isoform 2 of Ubiquitin carboxyl-terminal hydrolase isozyme L5 OS=Mus musculus GN=Uchl5 - [UCHL5_MOUSE]	-0.23	-0.18	-0.31	-0.33	0.01
O88351	Inhibitor of nuclear factor kappa-B kinase subunit beta OS=Mus musculus GN=Ikbkb PE=1 SV=1 - [IKKB_MOUSE]	-0.36	-0.61	-0.48	-0.69	0.01
D6RFQ6	Mitochondrial fission regulator 1 OS=Mus musculus GN=Mtfr1 PE=2 SV=1 - [D6RFQ6_MOUSE]	-0.39	-0.46	-0.24	-0.31	0.01
Q6NZA9	Transcription initiation factor TFIID subunit 9B OS=Mus musculus GN=Taf9b PE=1 SV=2 - [TAF9B_MOUSE]	0.53	0.77	0.39	0.62	0.01

A2RTH5	Leucine carboxyl methyltransferase 1 OS=Mus musculus GN=Lcmt1 PE=2 SV=1 - [A2RTH5_MOUSE]	-0.57	-0.56	-0.97	-0.83	0.01
O89114	DnaJ homolog subfamily B member 5 OS=Mus musculus GN=Dnajb5 PE=2 SV=1 - [DNJB5_MOUSE]	-0.29	-0.22	-0.39	-0.41	0.01
Q9DCS3	Trans-2-enoyl-CoA reductase, mitochondrial OS=Mus musculus GN=Mecr PE=2 SV=2 - [MECR_MOUSE]	-0.49	-0.86	-0.51	-0.62	0.01
P32507-2	Isoform Alpha of Poliovirus receptor-related protein 2 OS=Mus musculus GN=Pvrl2 - [PVRL2_MOUSE]	-0.14	-0.16	-0.09	-0.18	0.01
D3YVL0	Putative helicase MOV-10 OS=Mus musculus GN=Mov10 PE=4 SV=1 - [D3YVL0_MOUSE]	0.51	0.29	0.50	0.33	0.01
Q62086	Serum paraoxonase/arylesterase 2 OS=Mus musculus GN=Pon2 PE=1 SV=2 - [PON2_MOUSE]	-0.66	-0.68	-0.38	-0.45	0.01
Q9JJF9	Signal peptide peptidase-like 2A OS=Mus musculus GN=Sppl2a PE=1 SV=2 - [SPP2A_MOUSE]	0.45	0.43	0.72	0.71	0.01
Q8BG32	26S proteasome non-ATPase regulatory subunit 11 OS=Mus musculus GN=Psmd11 PE=1 SV=3 - [PSD11_MOUSE]	-0.36	-0.31	-0.28	-0.18	0.01
P0C0A3	Charged multivesicular body protein 6 OS=Mus musculus GN=Chmp6 PE=2 SV=2 - [CHMP6_MOUSE]	0.17	0.29	0.23	0.33	0.01
D3Z7V3	Sarcolemmal membrane-associated protein OS=Mus musculus GN=Slmap PE=2 SV=1 - [D3Z7V3_MOUSE]	-0.21	-0.27	-0.15	-0.30	0.01
Q922V4	Pleiotropic regulator 1 OS=Mus musculus GN=Plrg1 PE=2 SV=1 - [PLRG1_MOUSE]	0.44	0.30	0.47	0.26	0.01
Q9D820-2	Isoform 2 of Prolyl-tRNA synthetase associated domain-containing protein 1 OS=Mus musculus GN=Prorsd1 - [PRXD1_MOUSE]	0.42	0.28	0.57	0.43	0.01
O70591	Prefoldin subunit 2 OS=Mus musculus GN=Pfdn2 PE=2 SV=2 - [PFD2_MOUSE]	0.67	0.33	0.53	0.48	0.01
Q60605-2	Isoform Smooth muscle of Myosin light polypeptide 6 OS=Mus musculus GN=Myl6 - [MYL6_MOUSE]	0.21	0.34	0.26	0.40	0.01
D3Z4C0	FERM, RhoGEF and pleckstrin domain-containing protein 2 OS=Mus musculus GN=Farp2 PE=2 SV=1 - [D3Z4C0_MOUSE]	0.77	0.81	0.47	0.50	0.01
P61027	Ras-related protein Rab-10 OS=Mus musculus GN=Rab10 PE=1 SV=1 - [RAB10_MOUSE]	-0.45	-0.45	-0.24	-0.33	0.01
E9QNN1	ATP-dependent RNA helicase A OS=Mus musculus GN=Dhx9 PE=2 SV=1 - [E9QNN1_MOUSE]	0.10	0.09	0.05	0.08	0.01
Q9D311	Dual oxidase maturation factor 2 OS=Mus musculus GN=Duoxa2 PE=2 SV=1 - [DOXA2_MOUSE]	0.66	0.88	1.08	1.30	0.01

Q8VDT9	39S ribosomal protein L50, mitochondrial OS=Mus musculus GN=Mrpl50 PE=2 SV=2 - [RM50_MOUSE]	-0.20	-0.26	-0.14	-0.16	0.01
Q99MR8	Methylcrotonoyl-CoA carboxylase subunit alpha, mitochondrial OS=Mus musculus GN=Mccc1 PE=2 SV=2 - [MCCA_MOUSE]	-0.32	-0.25	-0.49	-0.39	0.01
Q9QUK0-2	Isoform 2 of Cyclin-dependent kinase-like 2 OS=Mus musculus GN=Cdkl2 - [CDKL2_MOUSE]	0.94	0.58	1.16	0.80	0.01
Q921J2	GTP-binding protein Rheb OS=Mus musculus GN=Rheb PE=1 SV=1 - [RHEB_MOUSE]	-0.31	-0.47	-0.25	-0.31	0.01
Q8CIH5	1-phosphatidylinositol 4,5-bisphosphate phosphodiesterase gamma-2 OS=Mus musculus GN=Plcg2 PE=1 SV=1 - [PLCG2_MOUSE]	0.20	0.12	0.25	0.23	0.01
Q6NZQ2	DEAD/H (Asp-Glu-Ala-Asp/His) box polypeptide 31 OS=Mus musculus GN=Ddx31 PE=2 SV=2 - [Q6NZQ2_MOUSE]	-0.11	-0.13	-0.18	-0.20	0.01
E9Q704	Peptidyl-glycine alpha-amidating monooxygenase OS=Mus musculus GN=Pam PE=2 SV=1 - [E9Q704_MOUSE]	-0.33	-0.21	-0.41	-0.29	0.01
P61161	Actin-related protein 2 OS=Mus musculus GN=Actr2 PE=1 SV=1 - [ARP2_MOUSE]	-0.36	-0.29	-0.22	-0.19	0.01
B7ZNL3	Tpm1 protein OS=Mus musculus GN=Tpm1 PE=2 SV=1 - [B7ZNL3_MOUSE]	-0.12	-0.19	-0.22	-0.24	0.01
Q3UMR5	Calcium uniporter protein, mitochondrial OS=Mus musculus GN=Mcu PE=1 SV=2 - [MCU_MOUSE]	-0.38	-0.24	-0.46	-0.30	0.01
Q3TPE9-2	Isoform 2 of Ankyrin repeat and MYND domain-containing protein 2 OS=Mus musculus GN=Ankmy2 - [ANKY2_MOUSE]	-0.71	-0.43	-0.78	-0.49	0.01
Q80U12	Small G protein signaling modulator 2 OS=Mus musculus GN=Sgsm2 PE=2 SV=2 - [SGSM2_MOUSE]	0.60	0.86	0.43	0.68	0.01
P35505	Fumarylacetoacetase OS=Mus musculus GN=Fah PE=1 SV=2 - [FAAA_MOUSE]	0.19	0.40	0.31	0.34	0.01
A2BE93	Protein SET (Fragment) OS=Mus musculus GN=Set PE=2 SV=1 - [A2BE93_MOUSE]	0.29	0.26	0.45	0.44	0.01
Q6ZQH8	Nucleoporin NUP188 homolog OS=Mus musculus GN=Nup188 PE=1 SV=2 - [NU188_MOUSE]	-0.30	-0.55	-0.37	-0.52	0.01
Q8BYK6	YTH domain family protein 3 OS=Mus musculus GN=Ythdf3 PE=1 SV=2 - [YTHD3_MOUSE]	0.88	1.01	1.48	1.59	0.01
Q9WV84	Nucleoside diphosphate kinase, mitochondrial OS=Mus musculus GN=Nme4 PE=1 SV=1 - [NDKM_MOUSE]	-0.58	-0.42	-0.77	-0.47	0.01
Q9D8T2	Gasdermin-D OS=Mus musculus GN=Gsdmdc1 PE=2 SV=1 - [GSDMD_MOUSE]	-0.52	-0.38	-0.31	-0.29	0.01

P49586	Choline-phosphate cytidylyltransferase A OS=Mus musculus GN=Pcyt1a PE=1 SV=1 - [PCY1A_MOUSE]	0.34	0.33	0.21	0.20	0.01
P56388-2	Isoform Short of Cocaine- and amphetamine-regulated transcript protein OS=Mus musculus GN=Cartpt - [CART_MOUSE]	0.64	0.96	0.53	0.59	0.01
Q9Z247	Peptidyl-prolyl cis-trans isomerase FKBP9 OS=Mus musculus GN=Fkbp9 PE=1 SV=1 - [FKBP9_MOUSE]	-0.14	-0.12	-0.08	-0.17	0.01
Q4FE56	Ubiquitin carboxyl-terminal hydrolase OS=Mus musculus GN=Usp9x PE=2 SV=1 - [Q4FE56_MOUSE]	-0.34	-0.30	-0.29	-0.16	0.01
E9Q1T5	MARVEL domain-containing protein 2 OS=Mus musculus GN=Marveld2 PE=2 SV=1 - [E9Q1T5_MOUSE]	-0.36	-0.22	-0.35	-0.21	0.01
D3Z0F5	COP9 signalosome complex subunit 6 OS=Mus musculus GN=Cops6 PE=2 SV=1 - [D3Z0F5_MOUSE]	-0.47	-0.37	-0.51	-0.26	0.01
P40936	Indolethylamine N-methyltransferase OS=Mus musculus GN=Inmt PE=1 SV=1 - [INMT_MOUSE]	0.33	0.52	0.29	0.49	0.01
E9Q3I4	Intersectin-1 OS=Mus musculus GN=Itsn1 PE=2 SV=1 - [E9Q3I4_MOUSE]	0.61	0.30	0.59	0.49	0.01
P70444	BH3-interacting domain death agonist OS=Mus musculus GN=Bid PE=1 SV=2 - [BID_MOUSE]	0.32	0.25	0.23	0.16	0.01
Q6PD26	GPI transamidase component PIG-S OS=Mus musculus GN=Pigs PE=1 SV=3 - [PIGS_MOUSE]	-0.47	-0.62	-0.36	-0.70	0.01
Q8BJL1	F-box only protein 30 OS=Mus musculus GN=Fbxo30 PE=2 SV=2 - [FBX30_MOUSE]	0.69	0.55	0.48	0.34	0.01
E9Q1U2	Synaptopodin-2 OS=Mus musculus GN=Synpo2 PE=2 SV=1 - [E9Q1U2_MOUSE]	0.36	0.49	0.40	0.67	0.01
P83882	60S ribosomal protein L36a OS=Mus musculus GN=Rpl36a PE=2 SV=2 - [RL36A_MOUSE]	-0.89	-0.62	-0.66	-0.44	0.01
P00416	Cytochrome c oxidase subunit 3 OS=Mus musculus GN=Mtco3 PE=3 SV=1 - [COX3_MOUSE]	-2.41	-1.68	-1.94	-1.20	0.01
Q9D9M2-2	Isoform 2 of Ubiquitin carboxyl-terminal hydrolase 12 OS=Mus musculus GN=Usp12 - [UBP12_MOUSE]	-0.24	-0.22	-0.39	-0.37	0.01
P51949	CDK-activating kinase assembly factor MAT1 OS=Mus musculus GN=Mnat1 PE=2 SV=2 - [MAT1_MOUSE]	0.84	0.56	1.14	0.86	0.01
Q8K1E0	Syntaxin-5 OS=Mus musculus GN=Stx5 PE=1 SV=3 - [STX5_MOUSE]	0.39	0.29	0.40	0.21	0.01
Q3T9X3	Dynamin-2 OS=Mus musculus GN=Dnm2 PE=2 SV=1 - [Q3T9X3_MOUSE]	-0.52	-0.37	-0.30	-0.29	0.01

Q6ZQA0	Neurobeachin-like protein 2 OS=Mus musculus GN=Nbeal2 PE=2 SV=2 - [NBEL2_MOUSE]	-0.39	-0.30	-0.58	-0.41	0.01
Q9JJ06	Glycoprotein-N-acetylgalactosamine 3-beta-galactosyltransferase 1 OS=Mus musculus GN=C1galt1 PE=1 SV=1 - [C1GLT_MOUSE]	-0.21	-0.13	-0.19	-0.27	0.01
Q3UM45	Protein phosphatase 1 regulatory subunit 7 OS=Mus musculus GN=Ppp1r7 PE=1 SV=2 - [PP1R7_MOUSE]	-0.26	-0.19	-0.24	-0.37	0.01
Q9JIY8	Probable N-acetyltransferase CML3 OS=Mus musculus GN=Cml3 PE=2 SV=1 - [CMLO3_MOUSE]	-0.44	-0.61	-0.30	-0.47	0.01
Q3UB74	Transforming growth factor beta regulator 1 OS=Mus musculus GN=Tbrg1 PE=1 SV=2 - [TBRG1_MOUSE]	0.68	0.41	0.80	0.52	0.01
P58137	Acyl-coenzyme A thioesterase 8 OS=Mus musculus GN=Acot8 PE=1 SV=1 - [ACOT8_MOUSE]	-0.44	-0.34	-0.22	-0.40	0.01
Q9JMD3	PCTP-like protein OS=Mus musculus GN=Stard10 PE=1 SV=1 - [PCTL_MOUSE]	-0.15	-0.18	-0.24	-0.28	0.01
O54988-2	Isoform 2 of STE20-like serine/threonine-protein kinase OS=Mus musculus GN=Slk - [SLK_MOUSE]	0.21	0.14	0.29	0.22	0.01
Q91V64	Isochorismatase domain-containing protein 1 OS=Mus musculus GN=Isoc1 PE=2 SV=1 - [ISOC1_MOUSE]	0.13	0.19	0.16	0.25	0.01
D3YWQ8	Protein kinase C eta type OS=Mus musculus GN=Prkch PE=2 SV=1 - [D3YWQ8_MOUSE]	-0.25	-0.34	-0.17	-0.26	0.01
G3X999	Protein A330021E22Rik (Fragment) OS=Mus musculus GN=A330021E22Rik PE=4 SV=1 - [G3X999_MOUSE]	-0.33	-0.47	-0.54	-0.68	0.01
Q8CAS9-3	Isoform 3 of Poly [ADP-ribose] polymerase 9 OS=Mus musculus GN=Parp9 - [PARP9_MOUSE]	0.17	0.29	0.19	0.31	0.01
P01837	Ig kappa chain C region OS=Mus musculus PE=1 SV=1 - [IGKC_MOUSE]	-0.27	-0.53	-0.51	-0.55	0.01
D3Z108	Transmembrane and ubiquitin-like domain-containing protein 1 (Fragment) OS=Mus musculus GN=Tmub1 PE=2 SV=1 - [D3Z108_MOUSE]	-0.10	-0.07	-0.07	-0.13	0.01
P06330	Ig heavy chain V region AC38 205.12 OS=Mus musculus PE=1 SV=1 - [HVM51_MOUSE]	-0.44	-0.73	-0.44	-0.44	0.01
Q9WUZ9	Ectonucleoside triphosphate diphosphohydrolase 5 OS=Mus musculus GN=Entpd5 PE=1 SV=1 - [ENTP5_MOUSE]	-0.19	-0.24	-0.26	-0.37	0.01
P97762	Retinitis pigmentosa 9 protein homolog OS=Mus musculus GN=rp9 PE=1 SV=1 - [RP9_MOUSE]	1.07	0.64	1.09	0.66	0.01
Q9DC71	28S ribosomal protein S15, mitochondrial OS=Mus musculus GN=Mrps15 PE=2 SV=2 - [RT15_MOUSE]	-0.67	-0.43	-0.59	-0.35	0.01

Q80ZQ3	Uncharacterized protein C8orf74 homolog OS=Mus musculus PE=2 SV=1 - [CH074_MOUSE]	-0.58	-0.72	-0.35	-0.49	0.01
Q8C570	mRNA export factor OS=Mus musculus GN=Rae1 PE=1 SV=1 - [RAE1L_MOUSE]	0.28	0.27	0.44	0.47	0.01
Q6ZQB6-2	Isoform 2 of Inositol hexakisphosphate and diphosphoinositol-pentakisphosphate kinase 2 OS=Mus musculus GN=Ppip5k2 - [VIP2_MOUSE]	-0.57	-0.45	-0.49	-0.27	0.01
Q8C605	6-phosphofructokinase OS=Mus musculus GN=Pfkp PE=2 SV=1 - [Q8C605_MOUSE]	-0.41	-0.40	-0.28	-0.22	0.01
Q64435	UDP-glucuronosyltransferase 1-6 OS=Mus musculus GN=Ugt1a6 PE=1 SV=1 - [UD16_MOUSE]	0.51	0.35	0.68	0.45	0.01
B2RUP2-2	Isoform 2 of Protein unc-13 homolog D OS=Mus musculus GN=Unc13d - [UN13D_MOUSE]	-0.25	-0.15	-0.32	-0.23	0.01
Q8BHG3	Cell cycle control protein 50B OS=Mus musculus GN=Tmem30b PE=2 SV=1 - [CC50B_MOUSE]	-0.36	-0.59	-0.33	-0.56	0.01
P56389	Cytidine deaminase OS=Mus musculus GN=Cda PE=1 SV=2 - [CDD_MOUSE]	0.24	0.41	0.51	0.45	0.01
Q3TJ91	Lethal(2) giant larvae protein homolog 2 OS=Mus musculus GN=Llgl2 PE=2 SV=2 - [L2GL2_MOUSE]	0.10	0.22	0.19	0.17	0.01
Q91YT7	Protein Ythdf2 OS=Mus musculus GN=Ythdf2 PE=2 SV=1 - [Q91YT7_MOUSE]	0.85	0.51	1.03	0.69	0.01
P30306	M-phase inducer phosphatase 2 OS=Mus musculus GN=Cdc25b PE=2 SV=1 - [MPIP2_MOUSE]	-0.31	-0.20	-0.42	-0.33	0.01
F6TCF9	BAG family molecular chaperone regulator 1 OS=Mus musculus GN=Bag1 PE=2 SV=1 - [F6TCF9_MOUSE]	0.15	0.08	0.15	0.12	0.01
Q5DTM8	E3 ubiquitin-protein ligase BRE1A OS=Mus musculus GN=Rnf20 PE=1 SV=2 - [BRE1A_MOUSE]	0.47	0.24	0.50	0.39	0.01
P53702	Cytochrome c-type heme lyase OS=Mus musculus GN=Hccs PE=2 SV=2 - [CCHL_MOUSE]	-0.64	-0.47	-0.94	-0.80	0.01
Q9R0N9	Synaptotagmin-9 OS=Mus musculus GN=Syt9 PE=1 SV=2 - [SYT9_MOUSE]	-0.29	-0.47	-0.62	-0.49	0.01
D3Z166	Alpha-1,3-mannosyl-glycoprotein 4-beta-N-acetylglucosaminyltransferase A OS=Mus musculus GN=Mgat4a PE=2 SV=1 - [D3Z166_MOUSE]	-0.84	-0.52	-1.10	-0.81	0.01
E9QAF9	Protein TANC1 OS=Mus musculus GN=Tanc1 PE=2 SV=1 - [E9QAF9_MOUSE]	0.45	0.40	0.52	0.75	0.01
P46935	E3 ubiquitin-protein ligase NEDD4 OS=Mus musculus GN=Nedd4 PE=1 SV=3 - [NEDD4_MOUSE]	-0.08	-0.06	-0.09	-0.04	0.01

A2AX52	Collagen alpha-4(VI) chain OS=Mus musculus GN=Col6a4 PE=1 SV=2 - [CO6A4_MOUSE]	0.44	0.22	0.40	0.31	0.01
P70257-2	Isoform NFIX2 of Nuclear factor 1 X-type OS=Mus musculus GN=Nfix - [NFIX_MOUSE]	0.92	0.70	0.54	1.06	0.01
Q7TSQ8	Pyruvate dehydrogenase phosphatase regulatory subunit, mitochondrial OS=Mus musculus GN=Pdpr PE=2 SV=1 - [PDPR_MOUSE]	0.44	0.37	0.44	0.22	0.01
P53810	Phosphatidylinositol transfer protein alpha isoform OS=Mus musculus GN=Pitpna PE=1 SV=2 - [PIPNA_MOUSE]	-0.57	-0.40	-0.37	-0.29	0.01
D3YU17	Nicalin OS=Mus musculus GN=Ncln PE=2 SV=1 - [D3YU17_MOUSE]	-0.21	-0.14	-0.28	-0.19	0.01
Q8C6H0	Galectin-8 OS=Mus musculus GN=Lgals8 PE=2 SV=1 - [Q8C6H0_MOUSE]	0.53	0.32	0.64	0.42	0.01
Q5SQP1-2	Isoform 2 of HORMA domain-containing protein 2 OS=Mus musculus GN=Hormad2 - [HORM2_MOUSE]	0.44	0.29	0.38	0.22	0.01
Q8K3E5-2	Isoform 2 of Jouberin OS=Mus musculus GN=Ahi1 - [AHI1_MOUSE]	-0.24	-0.37	-0.37	-0.50	0.01
P70335	Rho-associated protein kinase 1 OS=Mus musculus GN=Rock1 PE=1 SV=1 - [ROCK1_MOUSE]	-0.09	-0.13	-0.15	-0.19	0.01
Q4VAE3	Transmembrane protein 65 OS=Mus musculus GN=Tmem65 PE=2 SV=1 - [TMM65_MOUSE]	-0.39	-0.37	-0.24	-0.22	0.01
P35550	rRNA 2'-O-methyltransferase fibrillarin OS=Mus musculus GN=Fbl PE=1 SV=2 - [FBRL_MOUSE]	-0.11	-0.18	-0.10	-0.11	0.01
P46061	Ran GTPase-activating protein 1 OS=Mus musculus GN=Rangap1 PE=1 SV=2 - [RAGP1_MOUSE]	-0.43	-0.35	-0.23	-0.26	0.01
Q8VE10	N-alpha-acetyltransferase 40 OS=Mus musculus GN=Naa40 PE=2 SV=1 - [NAA40_MOUSE]	-0.45	-0.75	-0.44	-0.74	0.01
P62830	60S ribosomal protein L23 OS=Mus musculus GN=Rpl23 PE=1 SV=1 - [RL23_MOUSE]	-0.46	-0.46	-0.26	-0.30	0.01
G3X9Q3	Histocompatibility (Minor) HA-1, isoform CRA_a OS=Mus musculus GN=Hmha1 PE=4 SV=1 - [G3X9Q3_MOUSE]	0.57	0.34	0.59	0.36	0.01
Q00493	Carboxypeptidase E OS=Mus musculus GN=Cpe PE=1 SV=2 - [CBPE_MOUSE]	-0.33	-0.15	-0.25	-0.26	0.01
F6RJC1	Teneurin-3 (Fragment) OS=Mus musculus GN=Tenm3 PE=4 SV=1 - [F6RJC1_MOUSE]	-1.03	-0.95	-0.51	-0.74	0.01
Q4KL52	Zfyve19 protein OS=Mus musculus GN=Zfyve19 PE=2 SV=1 - [Q4KL52_MOUSE]	1.02	1.27	0.62	0.87	0.01

Q8R2I1	GDP-fucose transporter 1 OS=Mus musculus GN=Slc35c1 PE=2 SV=1 - [Q8R2I1_MOUSE]	-0.64	-0.54	-0.79	-0.39	0.01
P20615	Homeobox protein Hox-B9 OS=Mus musculus GN=Hoxb9 PE=2 SV=3 - [HXB9_MOUSE]	-1.07	-0.64	-1.29	-0.86	0.01
Q8BWR8	Rhophilin-2 OS=Mus musculus GN=Rhpn2 PE=1 SV=2 - [RHPN2_MOUSE]	-0.72	-0.43	-0.86	-0.57	0.01
O35177	Enhancer of filamentation 1 OS=Mus musculus GN=Nedd9 PE=1 SV=2 - [CASL_MOUSE]	-0.29	-0.43	-0.21	-0.36	0.01
Q9DAW9	Calponin-3 OS=Mus musculus GN=Cnn3 PE=2 SV=1 - [CNN3_MOUSE]	0.37	0.53	0.27	0.34	0.01
P59113	Fermitin family homolog 1 OS=Mus musculus GN=Fermt1 PE=1 SV=4 - [FERM1_MOUSE]	-0.56	-0.36	-0.71	-0.45	0.01
Q91VH2	Sorting nexin-9 OS=Mus musculus GN=Snx9 PE=1 SV=1 - [SNX9_MOUSE]	0.21	0.25	0.13	0.27	0.01
Q8VHK1	Caskin-2 OS=Mus musculus GN=Caskin2 PE=1 SV=3 - [CSKI2_MOUSE]	0.25	0.22	0.40	0.37	0.01
Q9ESY9	Gamma-interferon-inducible lysosomal thiol reductase OS=Mus musculus GN=Ifi30 PE=1 SV=3 - [GILT_MOUSE]	-1.30	-0.95	-0.96	-0.62	0.01
A2A9X5	5'(3')-deoxyribonucleotidase, cytosolic type OS=Mus musculus GN=Nt5c PE=2 SV=1 - [A2A9X5_MOUSE]	-0.91	-0.54	-0.89	-0.54	0.01
P27808	Alpha-1,3-mannosyl-glycoprotein 2-beta-N-acetylglucosaminyltransferase OS=Mus musculus GN=Mgat1 PE=2 SV=1 - [MGAT1_MOUSE]	-0.57	-0.58	-0.58	-0.28	0.01
Q9JKW0	ADP-ribosylation factor-like protein 6-interacting protein 1 OS=Mus musculus GN=Arl6ip1 PE=2 SV=1 - [AR6P1_MOUSE]	-0.53	-0.38	-0.67	-0.37	0.01
Q9CXN7	Phenazine biosynthesis-like domain-containing protein 2 OS=Mus musculus GN=Pbld2 PE=2 SV=1 - [PBLD2_MOUSE]	-0.17	-0.20	-0.28	-0.32	0.01
Q9WTK3	Glycosylphosphatidylinositol anchor attachment 1 protein OS=Mus musculus GN=Gpaa1 PE=1 SV=3 - [GPAA1_MOUSE]	-0.82	-0.52	-0.73	-0.43	0.01
Q9JLJ2	4-trimethylaminobutyraldehyde dehydrogenase OS=Mus musculus GN=Aldh9a1 PE=1 SV=1 - [AL9A1_MOUSE]	0.17	0.20	0.27	0.32	0.01
Q5SVQ0-4	Isoform 4 of Histone acetyltransferase KAT7 OS=Mus musculus GN=Kat7 - [KAT7_MOUSE]	0.62	0.51	0.38	0.32	0.01
Q810B6	Ankyrin repeat and FYVE domain-containing protein 1 OS=Mus musculus GN=Ankfy1 PE=2 SV=2 - [ANFY1_MOUSE]	-0.16	-0.13	-0.26	-0.23	0.01
Q8BY89-2	Isoform 2 of Choline transporter-like protein 2 OS=Mus musculus GN=Slc44a2 - [CTL2_MOUSE]	-0.29	-0.30	-0.39	-0.53	0.01

Q3UDK1-2	Isoform 2 of TRAF-type zinc finger domain-containing protein 1 OS=Mus musculus GN=Trafd1 - [TRAD1_MOUSE]	0.93	1.44	0.72	1.22	0.01
Q542I8	Integrin beta OS=Mus musculus GN=Itgb2 PE=2 SV=1 [Q542I8_MOUSE]	0.27	0.37	0.31	0.51	0.01
P57759	Endoplasmic reticulum resident protein 29 OS=Mus musculus GN=Erp29 PE=1 SV=2 - [ERP29_MOUSE]	0.07	0.09	0.05	0.06	0.01
B2RQY6	BC035947 protein OS=Mus musculus GN=BC035947 PE=2 SV=1 - [B2RQY6_MOUSE]	-0.48	-0.40	-0.77	-0.69	0.01
P35385	Heat shock protein beta-7 OS=Mus musculus GN=Hspb7 PE=1 SV=3 - [HSPB7_MOUSE]	-0.34	-0.30	-0.56	-0.50	0.01
Q9QY81	Nuclear pore membrane glycoprotein 210 OS=Mus musculus GN=Nup210 PE=1 SV=2 - [PO210_MOUSE]	-0.17	-0.23	-0.35	-0.28	0.01
Q78JE5	F-box only protein 22 OS=Mus musculus GN=Fbxo22 PE=2 SV=2 - [FBX22_MOUSE]	-0.78	-0.47	-0.97	-0.66	0.01
Q8BWT1	3-ketoacyl-CoA thiolase, mitochondrial OS=Mus musculus GN=Acaa2 PE=1 SV=3 - [THIM_MOUSE]	0.22	0.25	0.41	0.38	0.01
Q8CHI8-3	Isoform 3 of E1A-binding protein p400 OS=Mus musculus GN=Ep400 - [EP400_MOUSE]	0.21	0.16	0.32	0.26	0.01
Q08091	Calponin-1 OS=Mus musculus GN=Cnn1 PE=2 SV=1 - [CNN1_MOUSE]	-0.22	-0.37	-0.20	-0.31	0.01
Q9EPM5-3	Isoform 3 of Syncoilin OS=Mus musculus GN=Sync - [SYNCI_MOUSE]	0.23	0.37	0.20	0.34	0.01
Q8R4C2	RUN and FYVE domain-containing protein 2 OS=Mus musculus GN=Rufy2 PE=1 SV=2 - [RUFY2_MOUSE]	-0.69	-0.61	-0.43	-0.36	0.01
Q8C436-2	Isoform 2 of Protein-lysine methyltransferase METTL21D OS=Mus musculus GN=Mettl21d - [MT21D_MOUSE]	0.74	0.61	1.17	1.03	0.01
Q8R2T8-2	Isoform 2 of General transcription factor 3C polypeptide 5 OS=Mus musculus GN=Gtf3c5 - [TF3C5_MOUSE]	0.78	0.55	0.48	0.40	0.01
Q9WTS2	Alpha-(1,6)-fucosyltransferase OS=Mus musculus GN=Fut8 PE=2 SV=2 - [FUT8_MOUSE]	-0.34	-0.56	-0.29	-0.49	0.01
O55143	Sarcoplasmic/endoplasmic reticulum calcium ATPase 2 OS=Mus musculus GN=Atp2a2 PE=1 SV=2 - [AT2A2_MOUSE]	-0.24	-0.18	-0.17	-0.11	0.01
Q6GTL7	6-phosphofructo-2-kinase/fructose-2, 6-biphosphatase 2 OS=Mus musculus GN=Pfkfb2 PE=2 SV=1 - [Q6GTL7_MOUSE]	-0.42	-0.64	-0.33	-0.42	0.01
Q8CIZ8	von Willebrand factor OS=Mus musculus GN=Vwf PE=1 SV=2 - [VWF_MOUSE]	0.28	0.31	0.16	0.19	0.01

P51863	V-type proton ATPase subunit d 1 OS=Mus musculus GN=Atp6v0d1 PE=1 SV=2 - [VA0D1_MOUSE]	-0.43	-0.34	-0.50	-0.24	0.01
Q923A2	Protein Spindly OS=Mus musculus GN=Spdl1 PE=1 SV=2 - [SPDLY_MOUSE]	0.45	0.46	0.77	0.77	0.01
Q9EQG7	Ectonucleotide pyrophosphatase/phosphodiesterase family member 5 OS=Mus musculus GN=Enpp5 PE=2 SV=3 - [ENPP5_MOUSE]	-1.16	-0.73	-1.00	-0.60	0.01
P31230	Aminoacyl tRNA synthase complex-interacting multifunctional protein 1 OS=Mus musculus GN=Aimp1 PE=1 SV=2 - [AIMP1_MOUSE]	0.31	0.23	0.35	0.18	0.01
P97821	Dipeptidyl peptidase 1 OS=Mus musculus GN=Ctsc PE=2 SV=1 - [CATC_MOUSE]	-0.14	-0.17	-0.08	-0.13	0.01
Q9CQ52	Chymotrypsin-like elastase family member 3B OS=Mus musculus GN=Cela3b PE=2 SV=1 - [CEL3B_MOUSE]	0.46	0.80	0.53	0.85	0.01
Q9Z2X8	Kelch-like ECH-associated protein 1 OS=Mus musculus GN=Keap1 PE=1 SV=1 - [KEAP1_MOUSE]	0.73	0.76	0.43	0.46	0.01
Q61553	Fascin OS=Mus musculus GN=Fscn1 PE=1 SV=4 - [FSCN1_MOUSE]	0.17	0.11	0.13	0.21	0.01
O54714-3	Isoform 3 of E3 SUMO-protein ligase PIAS3 OS=Mus musculus GN=Pias3 - [PIAS3_MOUSE]	-1.65	-1.61	-0.99	-0.95	0.01
Q8BFZ3	Beta-actin-like protein 2 OS=Mus musculus GN=Actbl2 PE=1 SV=1 - [ACTBL_MOUSE]	-0.25	-0.27	-0.46	-0.36	0.01
Q69ZP3-2	Isoform 2 of Probable hydrolase PNKD OS=Mus musculus GN=Pnkd - [PNKD_MOUSE]	-0.41	-0.24	-0.42	-0.25	0.01
Q91WR3-2	Isoform 2 of Activating signal cointegrator 1 complex subunit 2 OS=Mus musculus GN=Ascc2 - [ASCC2_MOUSE]	0.24	0.41	0.31	0.48	0.01
Q62266	Cornifin-A OS=Mus musculus GN=Sprr1a PE=2 SV=1 - [SPR1A_MOUSE]	2.61	2.31	1.75	1.29	0.01
Q80UK7-2	Isoform 2 of Spindle assembly abnormal protein 6 homolog OS=Mus musculus GN=Sass6 - [SAS6_MOUSE]	-0.78	-0.70	-0.49	-0.41	0.01
Q9QYJ3	DnaJ homolog subfamily B member 1 OS=Mus musculus GN=Dnajb1 PE=2 SV=3 - [DNJB1_MOUSE]	0.14	0.20	0.28	0.26	0.01
Q6NZB0	DnaJ homolog subfamily C member 8 OS=Mus musculus GN=Dnajc8 PE=2 SV=2 - [DNJC8_MOUSE]	0.31	0.28	0.52	0.44	0.01
O88327	Alpha-catulin OS=Mus musculus GN=Ctnnal1 PE=2 SV=1 - [CTNL1_MOUSE]	-0.81	-0.74	-1.02	-0.48	0.01
Q9D9V3-2	Isoform 2 of Ethylmalonyl-CoA decarboxylase OS=Mus musculus GN=Echdc1 - [ECHD1_MOUSE]	-0.16	-0.15	-0.08	-0.11	0.01

Q8K2Q0	COMM domain-containing protein 9 OS=Mus musculus GN=Commd9 PE=2 SV=3 - [COMD9_MOUSE]	-0.43	-0.29	-0.38	-0.22	0.01
Q8BJ03-2	Isoform 2 of Cytochrome c oxidase assembly protein COX15 homolog OS=Mus musculus GN=Cox15 - [COX15_MOUSE]	-0.50	-0.29	-0.53	-0.33	0.01
A2A3Z2	Transcriptional regulator Kaiso (Fragment) OS=Mus musculus GN=Zbtb33 PE=2 SV=1 - [A2A3Z2_MOUSE]	-0.95	-0.45	-0.68	-0.70	0.01
P97470	Serine/threonine-protein phosphatase 4 catalytic subunit OS=Mus musculus GN=Ppp4c PE=1 SV=2 - [PP4C_MOUSE]	0.48	0.32	0.59	0.34	0.01
Q3TJI8	Corticosteroid 11-beta-dehydrogenase isozyme 1 OS=Mus musculus GN=Hsd11b1 PE=2 SV=1 - [Q3TJI8_MOUSE]	0.46	0.68	0.70	0.96	0.01
Q9R069	Basal cell adhesion molecule OS=Mus musculus GN=Bcam PE=2 SV=1 - [BCAM_MOUSE]	0.05	0.08	0.05	0.04	0.01
Q9DCU6	39S ribosomal protein L4, mitochondrial OS=Mus musculus GN=Mrpl4 PE=2 SV=1 - [RM04_MOUSE]	-0.66	-0.59	-0.41	-0.34	0.01
Q91VR7	Microtubule-associated proteins 1A/1B light chain 3A OS=Mus musculus GN=Map1lc3a PE=1 SV=1 - [MLP3A_MOUSE]	0.22	0.17	0.34	0.32	0.01
Q80X90	Filamin-B OS=Mus musculus GN=Flnb PE=1 SV=3 - [FLNB_MOUSE]	-0.27	-0.30	-0.16	-0.18	0.01
Q9ES74	Serine/threonine-protein kinase Nek7 OS=Mus musculus GN=Nek7 PE=2 SV=1 - [NEK7_MOUSE]	-0.69	-0.49	-0.67	-0.35	0.01
Q9CSB4-2	Isoform 2 of Partitioning defective 3 homolog B OS=Mus musculus GN=Pard3b - [PAR3L_MOUSE]	0.66	0.59	0.42	0.34	0.01
Q61823	Programmed cell death protein 4 OS=Mus musculus GN=Pdcd4 PE=1 SV=1 - [PDCD4_MOUSE]	-0.12	-0.15	-0.13	-0.07	0.01
Q9CWK8	Sorting nexin-2 OS=Mus musculus GN=Snx2 PE=1 SV=2 - [SNX2_MOUSE]	-0.10	-0.05	-0.07	-0.10	0.01
Q9JIY2-4	Isoform 4 of E3 ubiquitin-protein ligase Hakai OS=Mus musculus GN=Cbll1 - [HAKAI_MOUSE]	0.53	0.77	0.36	0.60	0.01
Q8BNA6-3	Isoform 3 of Protocadherin Fat 3 OS=Mus musculus GN=Fat3 - [FAT3_MOUSE]	-0.71	-0.95	-1.20	-1.44	0.01
P15105	Glutamine synthetase OS=Mus musculus GN=Glul PE=1 SV=6 - [GLNA_MOUSE]	-0.26	-0.20	-0.13	-0.18	0.01
Q91Y97	Fructose-bisphosphate aldolase B OS=Mus musculus GN=Aldob PE=1 SV=3 - [ALDOB_MOUSE]	-0.27	-0.13	-0.29	-0.26	0.01
F7DAY5	Regulation of nuclear pre-mRNA domain-containing protein 1B (Fragment) OS=Mus musculus GN=Rprd1b PE=4 SV=1 - [F7DAY5_MOUSE]	0.31	0.22	0.42	0.25	0.01

E9PUM4	Talin-2 OS=Mus musculus GN=Tln2 PE=4 SV=1 - [E9PUM4_MOUSE]	-0.15	-0.25	-0.29	-0.33	0.01
Q9JI10	Serine/threonine-protein kinase 3 OS=Mus musculus GN=Stk3 PE=1 SV=1 - [STK3_MOUSE]	0.55	0.41	0.84	0.67	0.01
Q9JM96	Cdc42 effector protein 4 OS=Mus musculus GN=Cdc42ep4 PE=1 SV=1 - [BORG4_MOUSE]	0.33	0.67	0.43	0.56	0.01
O09110-2	Isoform 1 of Dual specificity mitogen-activated protein kinase kinase 3 OS=Mus musculus GN=Map2k3 - [MP2K3_MOUSE]	-0.23	-0.23	-0.25	-0.40	0.01
P62137	Serine/threonine-protein phosphatase PP1-alpha catalytic subunit OS=Mus musculus GN=Ppp1ca PE=1 SV=1 - [PP1A_MOUSE]	0.36	0.27	0.43	0.22	0.01
Q9CQW2	ADP-ribosylation factor-like protein 8B OS=Mus musculus GN=Arl8b PE=2 SV=1 - [ARL8B_MOUSE]	-0.35	-0.37	-0.20	-0.22	0.01
Q3TH34	Protein FAM45A OS=Mus musculus GN=Fam45a PE=2 SV=1 - [Q3TH34_MOUSE]	-0.17	-0.24	-0.11	-0.20	0.01
Q8R4R6	Nucleoporin NUP53 OS=Mus musculus GN=Nup35 PE=1 SV=2 - [NUP53_MOUSE]	0.36	0.47	0.36	0.65	0.01
F8VQ43	Laminin subunit alpha-2 OS=Mus musculus GN=Lama2 PE=2 SV=1 - [F8VQ43_MOUSE]	0.12	0.13	0.08	0.17	0.01
Q8BM75-2	Isoform 2 of AT-rich interactive domain-containing protein 5B OS=Mus musculus GN=Arid5b - [ARI5B_MOUSE]	-0.23	-0.20	-0.38	-0.34	0.01
Q9ES97-3	Isoform 3 of Reticulon-3 OS=Mus musculus GN=Rtn3 - [RTN3_MOUSE]	-0.73	-0.41	-0.85	-0.57	0.01
Q8K4S1	1-phosphatidylinositol 4,5-bisphosphate phosphodiesterase epsilon-1 OS=Mus musculus GN=Plce1 PE=1 SV=3 - [PLCE1_MOUSE]	-0.59	-0.57	-0.92	-0.48	0.01
O35841	Apoptosis inhibitor 5 OS=Mus musculus GN=Api5 PE=2 SV=2 - [API5_MOUSE]	0.16	0.10	0.22	0.17	0.01
Q91VB8	Alpha globin 1 OS=Mus musculus GN=Hba-a1 PE=2 SV=1 - [Q91VB8_MOUSE]	-0.17	-0.28	-0.15	-0.25	0.01
G5E870	E3 ubiquitin-protein ligase TRIP12 OS=Mus musculus GN=Trip12 PE=1 SV=1 - [TRIPC_MOUSE]	-0.21	-0.30	-0.18	-0.35	0.01
E9Q8N5	CLIP-associating protein 2 OS=Mus musculus GN=Clasp2 PE=2 SV=1 - [E9Q8N5_MOUSE]	-0.19	-0.18	-0.32	-0.30	0.01
Q9DBC3	Cap-specific mRNA (nucleoside-2'-O-)-methyltransferase 1 OS=Mus musculus GN=Ftsjd2 PE=1 SV=1 - [MTR1_MOUSE]	-0.61	-0.42	-0.48	-0.29	0.01
P01635	Ig kappa chain V-V region K2 (Fragment) OS=Mus musculus PE=1 SV=1 - [KV5A3_MOUSE]	-0.23	-0.37	-0.48	-0.49	0.01

F6XZQ5	CMP-N-acetylneuraminate-beta-1,4-galactoside alpha-2,3-sialyltransferase (Fragment) OS=Mus musculus GN=St3gal3 PE=4 SV=1 - [F6XZQ5_MOUSE]	0.59	0.40	0.51	0.29	0.01
Q810A7	ATP-dependent RNA helicase DDX42 OS=Mus musculus GN=Ddx42 PE=1 SV=3 - [DDX42_MOUSE]	0.25	0.32	0.45	0.25	0.01
Q9DCL2	MIP18 family protein FAM96A OS=Mus musculus GN=Fam96a PE=2 SV=1 - [FA96A_MOUSE]	-0.23	-0.36	-0.19	-0.23	0.01
F2Z3X3	CUE domain-containing protein 1 OS=Mus musculus GN=Cuedc1 PE=2 SV=1 - [F2Z3X3_MOUSE]	0.78	0.43	0.75	0.47	0.01
Q3U7R1	Extended synaptotagmin-1 OS=Mus musculus GN=Esyt1 PE=2 SV=2 - [ESYT1_MOUSE]	-0.26	-0.18	-0.14	-0.14	0.01
Q7TPV4	Myb-binding protein 1A OS=Mus musculus GN=Mybbp1a PE=1 SV=2 - [MBB1A_MOUSE]	-0.33	-0.20	-0.35	-0.21	0.01
E9Q3Q6	CD166 antigen OS=Mus musculus GN=Alcam PE=2 SV=1 - [E9Q3Q6_MOUSE]	-0.68	-0.66	-0.36	-0.43	0.01
Q8C2E7	WASH complex subunit strumpellin OS=Mus musculus GN=Kiaa0196 PE=2 SV=2 - [STRUM_MOUSE]	-0.39	-0.37	-0.68	-0.60	0.01
Q8JZP9	GAS2-like protein 1 OS=Mus musculus GN=Gas2l1 PE=2 SV=1 - [GA2L1_MOUSE]	-0.30	-0.24	-0.20	-0.14	0.01
Q9JJ28	Protein flightless-1 homolog OS=Mus musculus GN=Flii PE=1 SV=1 - [FLII_MOUSE]	-0.16	-0.21	-0.10	-0.20	0.01
H3BLK9	Espin-like protein OS=Mus musculus GN=Espnl PE=2 SV=1 - [H3BLK9_MOUSE]	-0.38	-0.27	-0.57	-0.46	0.01
Q8C6B0	MCG20149, isoform CRA_a OS=Mus musculus GN=Mettl7a1 PE=2 SV=1 - [Q8C6B0_MOUSE]	-0.42	-0.32	-0.25	-0.22	0.01
Q91VZ6	Stromal membrane-associated protein 1 OS=Mus musculus GN=Smap1 PE=1 SV=1 - [SMAP1_MOUSE]	0.29	0.22	0.46	0.33	0.01
Q810A3	Tetratricopeptide repeat protein 9C OS=Mus musculus GN=Ttc9c PE=2 SV=1 - [TTC9C_MOUSE]	-0.16	-0.24	-0.12	-0.20	0.01
P62281	40S ribosomal protein S11 OS=Mus musculus GN=Rps11 PE=2 SV=3 - [RS11_MOUSE]	-0.36	-0.38	-0.19	-0.25	0.01
Q8VHL1	Histone-lysine N-methyltransferase SETD7 OS=Mus musculus GN=Setd7 PE=2 SV=2 - [SETD7_MOUSE]	0.57	0.30	0.47	0.33	0.01
P80318	T-complex protein 1 subunit gamma OS=Mus musculus GN=Cct3 PE=1 SV=1 - [TCPG_MOUSE]	-0.06	-0.10	-0.06	-0.10	0.01
Q8K0U4	Heat shock 70 kDa protein 12A OS=Mus musculus GN=Hspa12a PE=1 SV=1 - [HS12A_MOUSE]	0.22	0.23	0.17	0.11	0.01

Q8VCG1	Deoxyuridine triphosphatase, isoform CRA_b OS=Mus musculus GN=Dut PE=2 SV=1 - [Q8VCG1_MOUSE]	-0.14	-0.24	-0.28	-0.18	0.01
Q8VDG7	Platelet-activating factor acetylhydrolase 2, cytoplasmic OS=Mus musculus GN=Pafah2 PE=2 SV=2 - [PAFA2_MOUSE]	-0.89	-0.59	-0.55	-0.45	0.01
O09172	Glutamate--cysteine ligase regulatory subunit OS=Mus musculus GN=Gclm PE=2 SV=1 - [GSH0_MOUSE]	-0.14	-0.09	-0.20	-0.15	0.01
P22437	Prostaglandin G/H synthase 1 OS=Mus musculus GN=Ptgs1 PE=2 SV=1 - [PGH1_MOUSE]	-0.33	-0.52	-0.33	-0.60	0.01
O09061	Proteasome subunit beta type-1 OS=Mus musculus GN=Psmb1 PE=1 SV=1 - [PSB1_MOUSE]	0.15	0.17	0.09	0.11	0.01
P00688	Pancreatic alpha-amylase OS=Mus musculus GN=Amy2 PE=1 SV=2 - [AMYP_MOUSE]	0.27	0.14	0.24	0.16	0.01
Q8BZW8	NHL repeat-containing protein 2 OS=Mus musculus GN=Nhlrc2 PE=2 SV=1 - [NHLC2_MOUSE]	-0.39	-0.24	-0.50	-0.34	0.01
P70298	Homeobox protein cut-like 2 OS=Mus musculus GN=Cux2 PE=2 SV=2 - [CUX2_MOUSE]	-1.38	-1.03	-0.98	-0.63	0.01
Q8CFE6	Sodium-coupled neutral amino acid transporter 2 OS=Mus musculus GN=Slc38a2 PE=1 SV=1 - [S38A2_MOUSE]	0.18	0.33	0.22	0.36	0.01
Q8BU11	TOX high mobility group box family member 4 OS=Mus musculus GN=Tox4 PE=1 SV=3 - [TOX4_MOUSE]	0.52	0.39	0.81	0.67	0.01
Q8K021	Secretory carrier-associated membrane protein 1 OS=Mus musculus GN=Scamp1 PE=1 SV=1 - [SCAM1_MOUSE]	-0.10	-0.22	-0.19	-0.20	0.01
P70429-2	Isoform 1 of Ena/VASP-like protein OS=Mus musculus GN=Evl - [EVL_MOUSE]	0.45	0.36	0.71	0.66	0.01
Q9QYF9	Protein NDRG3 OS=Mus musculus GN=Ndrg3 PE=1 SV=1 - [NDRG3_MOUSE]	-0.24	-0.37	-0.41	-0.53	0.01
P47740	Fatty aldehyde dehydrogenase OS=Mus musculus GN=Aldh3a2 PE=2 SV=2 - [AL3A2_MOUSE]	-0.35	-0.41	-0.19	-0.31	0.01
Q9CQL5	39S ribosomal protein L18, mitochondrial OS=Mus musculus GN=Mrpl18 PE=2 SV=1 - [RM18_MOUSE]	-0.37	-0.21	-0.45	-0.44	0.01
P97298	Pigment epithelium-derived factor OS=Mus musculus GN=Serpinf1 PE=1 SV=2 - [PEDF_MOUSE]	0.49	0.69	0.31	0.51	0.01
P09103	Protein disulfide-isomerase OS=Mus musculus GN=P4hb PE=1 SV=2 - [PDIA1_MOUSE]	0.31	0.17	0.37	0.34	0.01
D3YZR1	Protein TSSC4 (Fragment) OS=Mus musculus GN=Tssc4 PE=2 SV=1 - [D3YZR1_MOUSE]	0.68	0.45	0.53	0.32	0.01

Q8VCF1	Soluble calcium-activated nucleotidase 1 OS=Mus musculus GN=Cant1 PE=2 SV=1 - [CANT1_MOUSE]	-0.30	-0.16	-0.18	-0.29	0.01
Q6PFH3	DDB1- and CUL4-associated factor 15 OS=Mus musculus GN=Dcaf15 PE=2 SV=1 - [DCA15_MOUSE]	-0.74	-0.53	-1.13	-0.92	0.01
Q6NS46	Protein RRP5 homolog OS=Mus musculus GN=Pdcd11 PE=2 SV=2 - [RRP5_MOUSE]	-0.45	-0.34	-0.21	-0.29	0.01
Q9CXT8	Mitochondrial-processing peptidase subunit beta OS=Mus musculus GN=Pmpcb PE=2 SV=1 - [MPPB_MOUSE]	0.13	0.14	0.19	0.25	0.01
Q99LD8	N(G),N(G)-dimethylarginine dimethylaminohydrolase 2 OS=Mus musculus GN=Ddah2 PE=1 SV=1 - [DDAH2_MOUSE]	0.18	0.27	0.22	0.36	0.01
A2ARP1-7	Isoform 6 of Inositol hexakisphosphate and diphosphoinositol-pentakisphosphate kinase 1 OS=Mus musculus GN=Ppip5k1 - [VIP1_MOUSE]	-0.52	-0.30	-0.62	-0.40	0.01
Q91XU3	Phosphatidylinositol 5-phosphate 4-kinase type-2 gamma OS=Mus musculus GN=Pip4k2c PE=2 SV=1 - [PI42C_MOUSE]	0.20	0.30	0.24	0.40	0.01
Q8R3G9	Tetraspanin-8 OS=Mus musculus GN=Tspan8 PE=2 SV=1 - [TSN8_MOUSE]	-0.23	-0.23	-0.37	-0.41	0.01
Q99N05	Membrane-spanning 4-domains subfamily A member 4D OS=Mus musculus GN=Ms4a4d PE=2 SV=1 - [M4A4D_MOUSE]	0.28	0.20	0.42	0.34	0.01
Q8BY71	Histone acetyltransferase type B catalytic subunit OS=Mus musculus GN=Hat1 PE=2 SV=1 - [HAT1_MOUSE]	-0.47	-0.28	-0.56	-0.61	0.01
A2AMH5	Choline transporter-like protein 1 OS=Mus musculus GN=Slc44a1 PE=2 SV=1 - [A2AMH5_MOUSE]	-1.39	-0.76	-1.48	-0.93	0.01
Q3TMP1	General transcription factor IIIC, polypeptide 3 OS=Mus musculus GN=Gtf3c3 PE=2 SV=1 - [Q3TMP1_MOUSE]	0.33	0.38	0.17	0.27	0.01
Q689Z5	Protein strawberry notch homolog 1 OS=Mus musculus GN=Sbno1 PE=1 SV=2 - [SBNO1_MOUSE]	-0.77	-0.44	-0.83	-0.50	0.01
Q9Z0P4-2	Isoform 2 of Paralemmin-1 OS=Mus musculus GN=Palm - [PALM_MOUSE]	0.23	0.22	0.35	0.41	0.01
Q5Q9H7	DNA polymerase kappa OS=Mus musculus GN=Polk PE=2 SV=1 - [Q5Q9H7_MOUSE]	0.13	0.23	0.16	0.12	0.01
D3Z141	Galectin-7 (Fragment) OS=Mus musculus GN=Lgals7 PE=2 SV=1 - [D3Z141_MOUSE]	0.86	0.50	1.05	0.68	0.01
Q3U1U4	Integrin alpha-M OS=Mus musculus GN=Itgam PE=2 SV=1 - [Q3U1U4_MOUSE]	0.54	0.95	0.58	0.98	0.01
Q61189	Methylosome subunit pICln OS=Mus musculus GN=Clns1a PE=1 SV=1 - [ICLN_MOUSE]	0.34	0.41	0.18	0.31	0.01

P61290	Proteasome activator complex subunit 3 OS=Mus musculus GN=Psme3 PE=1 SV=1 - [PSME3_MOUSE]	-0.33	-0.32	-0.56	-0.56	0.01
Q69ZC8	Uncharacterized protein KIAA1704 OS=Mus musculus GN=Kiaa1704 PE=1 SV=2 - [K1704_MOUSE]	0.36	0.50	0.23	0.36	0.01
Q8BUR4	Dedicator of cytokinesis protein 1 OS=Mus musculus GN=Dock1 PE=1 SV=3 - [DOCK1_MOUSE]	-0.43	-0.31	-0.63	-0.60	0.01
F6ZMJ4	Trinucleotide repeat-containing gene 6A protein OS=Mus musculus GN=Tnrc6a PE=2 SV=2 - [F6ZMJ4_MOUSE]	0.30	0.28	0.51	0.48	0.01
Q9JI11	Serine/threonine-protein kinase 4 OS=Mus musculus GN=Stk4 PE=1 SV=1 - [STK4_MOUSE]	0.37	0.37	0.43	0.19	0.01
P60840-2	Isoform 2 of Alpha-endosulfine OS=Mus musculus GN=Ensa - [ENSA_MOUSE]	0.96	0.55	1.09	0.67	0.01
Q9D115	Zinc finger protein 706 OS=Mus musculus GN=Znf706 PE=2 SV=1 - [ZN706_MOUSE]	0.48	0.26	0.56	0.38	0.01
Q91WS2-2	Isoform 2 of NACHT, LRR and PYD domains-containing protein 6 OS=Mus musculus GN=Nlrp6 - [NALP6_MOUSE]	-0.70	-0.61	-0.31	-0.56	0.01
Q5RL20	39S ribosomal protein L43, mitochondrial OS=Mus musculus GN=Mrpl43 PE=2 SV=1 - [Q5RL20_MOUSE]	-0.28	-0.21	-0.26	-0.13	0.01
Q9D4D5	Protein 4933414I15Rik OS=Mus musculus GN=4933414I15Rik PE=2 SV=1 - [Q9D4D5_MOUSE]	0.68	0.62	0.42	0.35	0.01
O08691	Arginase-2, mitochondrial OS=Mus musculus GN=Arg2 PE=1 SV=1 - [ARGI2_MOUSE]	0.24	0.14	0.26	0.16	0.01
Q60936	Chaperone activity of bc1 complex-like, mitochondrial OS=Mus musculus GN=Adck3 PE=2 SV=2 - [ADCK3_MOUSE]	-0.39	-0.26	-0.26	-0.19	0.01
Q3B7Z2	Oxysterol-binding protein 1 OS=Mus musculus GN=Osbp PE=1 SV=3 - [OSBP1_MOUSE]	-0.26	-0.18	-0.12	-0.18	0.01
Q8BH79	Anoctamin-10 OS=Mus musculus GN=Ano10 PE=2 SV=1 - [ANO10_MOUSE]	-0.59	-0.34	-0.43	-0.30	0.01
G8JL83	YrdC domain-containing protein, mitochondrial (Fragment) OS=Mus musculus GN=Yrdc PE=2 SV=1 - [G8JL83_MOUSE]	-0.34	-0.46	-0.21	-0.32	0.01
Q9Z1F9	SUMO-activating enzyme subunit 2 OS=Mus musculus GN=Uba2 PE=2 SV=1 - [SAE2_MOUSE]	-0.40	-0.26	-0.50	-0.28	0.01
Q5DTY9	BTB/POZ domain-containing protein KCTD16 OS=Mus musculus GN=Kctd16 PE=1 SV=2 - [KCD16_MOUSE]	0.78	1.39	0.95	1.55	0.01
O70318	Band 4.1-like protein 2 OS=Mus musculus GN=Epb41l2 PE=1 SV=2 - [E41L2_MOUSE]	0.12	0.10	0.20	0.14	0.01

A3KMP2	Tetratricopeptide repeat protein 38 OS=Mus musculus GN=Ttc38 PE=2 SV=2 - [TTC38_MOUSE]	-0.36	-0.17	-0.37	-0.32	0.01
Q8BVG4	Dipeptidyl peptidase 9 OS=Mus musculus GN=Dpp9 PE=2 SV=2 - [DPP9_MOUSE]	0.54	0.35	0.78	0.59	0.01
D3Z3J6	Polyadenylate-binding protein-interacting protein 1 OS=Mus musculus GN=Paip1 PE=2 SV=1 - [D3Z3J6_MOUSE]	0.40	0.50	0.22	0.37	0.01
Q811J3	Iron-responsive element-binding protein 2 OS=Mus musculus GN=Ireb2 PE=2 SV=2 - [IREB2_MOUSE]	0.46	0.67	0.80	1.00	0.01
Q6P8I4	PEST proteolytic signal-containing nuclear protein OS=Mus musculus GN=Pcnp PE=1 SV=1 - [PCNP_MOUSE]	0.19	0.23	0.36	0.37	0.01
Q9CQ19	Myosin regulatory light polypeptide 9 OS=Mus musculus GN=Myl9 PE=1 SV=3 - [MYL9_MOUSE]	0.36	0.25	0.33	0.17	0.01
Q6DID7-2	Isoform 2 of Protein wntless homolog OS=Mus musculus GN=Wls - [WLS_MOUSE]	-0.88	-0.60	-0.68	-0.40	0.01
Q80ZX0	Protein Sec24b OS=Mus musculus GN=Sec24b PE=2 SV=1 - [Q80ZX0_MOUSE]	0.24	0.33	0.19	0.17	0.01
E9Q5C9	Protein Nolc1 OS=Mus musculus GN=Nolc1 PE=2 SV=1 - [E9Q5C9_MOUSE]	0.31	0.30	0.44	0.56	0.01
Q9D6T0-2	Isoform 2 of Nitric oxide synthase-interacting protein OS=Mus musculus GN=Nosip - [NOSIP_MOUSE]	0.60	0.73	1.08	1.20	0.01
Q8CIB6	Transmembrane protein 230 OS=Mus musculus GN=Tmem230 PE=1 SV=1 - [TM230_MOUSE]	0.37	0.37	0.22	0.21	0.01
Q5UAK0-4	Isoform 4 of Mesoderm induction early response protein 1 OS=Mus musculus GN=Mier1 - [MIER1_MOUSE]	0.28	0.17	0.37	0.25	0.01
A2ADR5	Monocarboxylate transporter 8 (Fragment) OS=Mus musculus GN=Slc16a2 PE=2 SV=1 - [A2ADR5_MOUSE]	5.53	4.72	3.47	2.66	0.01
Q9WVA4	Transgelin-2 OS=Mus musculus GN=Tagln2 PE=1 SV=4 - [TAGL2_MOUSE]	0.16	0.10	0.20	0.12	0.01
Q9CR95	Adaptin ear-binding coat-associated protein 1 OS=Mus musculus GN=Necap1 PE=1 SV=2 - [NECP1_MOUSE]	-0.64	-0.40	-0.55	-0.31	0.01
B1AY13	Ubiquitin carboxyl-terminal hydrolase 24 OS=Mus musculus GN=Usp24 PE=1 SV=1 - [UBP24_MOUSE]	0.18	0.35	0.25	0.38	0.01
O08579	Emerin OS=Mus musculus GN=Emd PE=1 SV=1 - [EMD_MOUSE]	0.26	0.47	0.52	0.33	0.01
P56677	Suppressor of tumorigenicity 14 protein homolog OS=Mus musculus GN=St14 PE=1 SV=2 - [ST14_MOUSE]	-0.03	-0.05	-0.07	-0.04	0.01

Q3TB04	FERM domain-containing protein 4A OS=Mus musculus GN=Frmd4a PE=2 SV=1 - [Q3TB04_MOUSE]	0.91	0.76	0.58	0.43	0.01
Q8C048	Protein Atp2b3 OS=Mus musculus GN=Atp2b3 PE=2 SV=1 - [Q8C048_MOUSE]	-0.56	-0.46	-0.36	-0.26	0.01
Q9DCM0	Persulfide dioxygenase ETHE1, mitochondrial OS=Mus musculus GN=Ethe1 PE=1 SV=2 - [ETHE1_MOUSE]	-0.17	-0.10	-0.17	-0.09	0.01
P70699	Lysosomal alpha-glucosidase OS=Mus musculus GN=Gaa PE=1 SV=2 - [LYAG_MOUSE]	-0.17	-0.23	-0.22	-0.35	0.01
Q8BJH1-2	Isoform 2 of Zinc finger C2HC domain-containing protein 1A OS=Mus musculus GN=Zc2hc1a - [ZC21A_MOUSE]	0.62	0.38	0.79	0.49	0.01
P83940	Transcription elongation factor B polypeptide 1 OS=Mus musculus GN=Tceb1 PE=1 SV=1 - [ELOC_MOUSE]	0.16	0.12	0.25	0.19	0.01
J3QMK2	MCG22959, isoform CRA_a OS=Mus musculus GN=Bbip1 PE=4 SV=1 - [J3QMK2_MOUSE]	0.41	0.67	0.32	0.57	0.01
Q80YW5	B box and SPRY domain-containing protein OS=Mus musculus GN=Bspry PE=1 SV=1 - [BSPRY_MOUSE]	-0.29	-0.36	-0.51	-0.59	0.01
P97494	Glutamate--cysteine ligase catalytic subunit OS=Mus musculus GN=Gclc PE=2 SV=4 - [GSH1_MOUSE]	0.14	0.25	0.13	0.20	0.01
D3YW19	Protein 9030624J02Rik (Fragment) OS=Mus musculus GN=9030624J02Rik PE=2 SV=2 - [D3YW19_MOUSE]	-0.61	-0.44	-0.95	-0.81	0.01
Q9DAA4	Probable inactive serine protease 37 OS=Mus musculus GN=Prss37 PE=2 SV=1 - [PRS37_MOUSE]	0.87	0.49	0.98	0.59	0.01
Q8JZX4	Splicing factor 45 OS=Mus musculus GN=Rbm17 PE=1 SV=1 - [SPF45_MOUSE]	0.41	0.55	0.80	0.84	0.01
A2AGT5-2	Isoform 2 of Cytoskeleton-associated protein 5 OS=Mus musculus GN=Ckap5 - [CKAP5_MOUSE]	-0.24	-0.16	-0.29	-0.36	0.01
B2RXQ9	Sorbin and SH3 domain-containing protein 2 OS=Mus musculus GN=Sorbs2 PE=2 SV=1 - [B2RXQ9_MOUSE]	0.48	0.76	0.36	0.64	0.01
Q8K3I6	Interleukin-27 subunit alpha OS=Mus musculus GN=Il27 PE=1 SV=1 - [IL27A_MOUSE]	0.56	0.98	0.56	0.98	0.01
P63213	Guanine nucleotide-binding protein G(I)/G(S)/G(O) subunit gamma-2 OS=Mus musculus GN=Gng2 PE=1 SV=2 - [GBG2_MOUSE]	0.34	0.51	0.67	0.74	0.01
Q9CVB6	Actin-related protein 2/3 complex subunit 2 OS=Mus musculus GN=Arpc2 PE=1 SV=3 - [ARPC2_MOUSE]	-0.30	-0.24	-0.18	-0.15	0.01
B2RTM1	Transmembrane protein 158 OS=Mus musculus GN=Tmem158 PE=2 SV=1 - [B2RTM1_MOUSE]	-0.66	-0.72	-0.37	-0.43	0.01

D3YUG5	MKL/myocardin-like protein 1 (Fragment) OS=Mus musculus GN=Mkl1 PE=2 SV=1 - [D3YUG5_MOUSE]	1.16	0.70	1.06	0.59	0.01
Q62179	Semaphorin-4B OS=Mus musculus GN=Sema4b PE=1 SV=2 - [SEM4B_MOUSE]	0.55	0.38	0.27	0.33	0.01
O35405	Phospholipase D3 OS=Mus musculus GN=Pld3 PE=2 SV=1 - [PLD3_MOUSE]	-0.40	-0.29	-0.63	-0.45	0.01
D3Z3R1	60S ribosomal protein L36 OS=Mus musculus GN=Gm5745 PE=3 SV=1 - [D3Z3R1_MOUSE]	0.19	0.36	0.28	0.42	0.01
D3Z7J7	Deoxyhypusine hydroxylase (Fragment) OS=Mus musculus GN=Dohh PE=2 SV=1 - [D3Z7J7_MOUSE]	0.37	0.57	0.29	0.56	0.01
O09053	Werner syndrome ATP-dependent helicase homolog OS=Mus musculus GN=Wrn PE=1 SV=3 - [WRN_MOUSE]	0.33	0.32	0.55	0.29	0.01
Q3U2A8	Valine--tRNA ligase, mitochondrial OS=Mus musculus GN=Vars2 PE=2 SV=2 - [SYVM_MOUSE]	-0.42	-0.25	-0.55	-0.36	0.01
Q6YI28	EP1 OS=Mus musculus GN=2210407C18Rik PE=2 SV=1 - [Q6YI28_MOUSE]	1.58	0.97	1.36	0.77	0.01
B1AXP6	Mitochondrial import receptor subunit TOM5 homolog OS=Mus musculus GN=Tomm5 PE=3 SV=1 - [TOM5_MOUSE]	-0.39	-0.23	-0.32	-0.19	0.01
Q3TP05	Angiomotin-like protein 2 OS=Mus musculus GN=Amotl2 PE=2 SV=1 - [Q3TP05_MOUSE]	0.52	0.27	0.51	0.63	0.01
P55302	Alpha-2-macroglobulin receptor-associated protein OS=Mus musculus GN=Lrpap1 PE=1 SV=1 - [AMRP_MOUSE]	0.27	0.20	0.43	0.32	0.01
Q9EPU0	Regulator of nonsense transcripts 1 OS=Mus musculus GN=Upf1 PE=1 SV=2 - [RENT1_MOUSE]	0.18	0.28	0.13	0.22	0.01
G3X9S2	Ectonucleotide pyrophosphatase/phosphodiesterase 1, isoform CRA_d OS=Mus musculus GN=Enpp1 PE=1 SV=1 - [G3X9S2_MOUSE]	-0.31	-0.15	-0.22	-0.20	0.01
Q8K124	Pleckstrin homology domain-containing family O member 2 OS=Mus musculus GN=Plekho2 PE=1 SV=1 - [PKHO2_MOUSE]	0.60	0.52	0.85	1.04	0.01
Q812E0	Cytoplasmic polyadenylation element-binding protein 2 OS=Mus musculus GN=Cpeb2 PE=1 SV=1 - [CPEB2_MOUSE]	0.59	0.53	1.01	0.95	0.01
Q99PL6-2	Isoform 2 of UBX domain-containing protein 6 OS=Mus musculus GN=Ubxn6 - [UBXN6_MOUSE]	-0.25	-0.42	-0.28	-0.50	0.01
E9Q855	Secretory carrier-associated membrane protein 3 OS=Mus musculus GN=Scamp3 PE=2 SV=1 - [E9Q855_MOUSE]	0.38	0.19	0.37	0.25	0.01
F7BWT7-2	Isoform 2 of Tetraspanin-15 OS=Mus musculus GN=Tspan15 - [TSN15_MOUSE]	-0.15	-0.18	-0.27	-0.30	0.01

E9Q6U4	Protein Pfdn4 OS=Mus musculus GN=Pfdn4 PE=2 SV=1 - [E9Q6U4_MOUSE]	0.33	0.29	0.58	0.46	0.01
G3UW42	MCG49030 OS=Mus musculus GN=Gm6900 PE=4 SV=1 - [G3UW42_MOUSE]	0.37	0.23	0.48	0.29	0.01
Q9CQ39	Mediator of RNA polymerase II transcription subunit 21 OS=Mus musculus GN=Med21 PE=1 SV=1 - [MED21_MOUSE]	0.24	0.39	0.19	0.34	0.01
E9Q9M5	Ubiquitin carboxyl-terminal hydrolase OS=Mus musculus GN=Usp19 PE=2 SV=1 - [E9Q9M5_MOUSE]	0.71	0.45	0.99	0.66	0.01
O35375-5	Isoform B0 of Neuropilin-2 OS=Mus musculus GN=Nrp2 - [NRP2_MOUSE]	-0.49	-0.41	-0.84	-0.65	0.01
Q8CJ61	CKLF-like MARVEL transmembrane domain-containing protein 4 OS=Mus musculus GN=Cmtm4 PE=2 SV=1 - [CKLF4_MOUSE]	-0.76	-0.42	-0.86	-0.52	0.01
Q61749	Translation initiation factor eIF-2B subunit delta OS=Mus musculus GN=Eif2b4 PE=2 SV=2 - [EI2BD_MOUSE]	0.54	0.39	0.41	0.24	0.01
Q9JMG1	Endothelial differentiation-related factor 1 OS=Mus musculus GN=Edf1 PE=1 SV=1 - [EDF1_MOUSE]	0.14	0.15	0.14	0.25	0.01
B1AVH7	TBC1 domain family member 2A OS=Mus musculus GN=Tbc1d2 PE=2 SV=1 - [TBD2A_MOUSE]	-0.14	-0.07	-0.10	-0.14	0.01
Q9D735	Uncharacterized protein C19orf43 homolog OS=Mus musculus PE=2 SV=1 - [CS043_MOUSE]	0.61	0.87	0.42	0.87	0.01
Q80T06	Elongation factor 1-delta OS=Mus musculus GN=Eef1d PE=2 SV=1 - [Q80T06_MOUSE]	0.39	0.20	0.32	0.21	0.01
Q91WV0	Protein Dr1 OS=Mus musculus GN=Dr1 PE=2 SV=1 - [NC2B_MOUSE]	0.58	0.57	0.34	0.32	0.01
Q8C4Y1	Max protein OS=Mus musculus GN=Max PE=2 SV=1 - [Q8C4Y1_MOUSE]	0.22	0.22	0.37	0.20	0.01
Q6ZPS6	Ankyrin repeat and IBR domain-containing protein 1 OS=Mus musculus GN=Ankib1 PE=1 SV=2 - [AKIB1_MOUSE]	-0.18	-0.23	-0.33	-0.38	0.01
Q9CPN9	Protein 2210010C04Rik OS=Mus musculus GN=2210010C04Rik PE=2 SV=1 - [Q9CPN9_MOUSE]	0.34	0.59	0.31	0.56	0.01
Q9JII6	Alcohol dehydrogenase [NADP(+)] OS=Mus musculus GN=Akr1a1 PE=1 SV=3 - [AK1A1_MOUSE]	0.09	0.08	0.08	0.04	0.01
D3Z5F0	Cdc42 effector protein 5 (Fragment) OS=Mus musculus GN=Cdc42ep5 PE=2 SV=1 - [D3Z5F0_MOUSE]	1.04	0.60	0.99	0.55	0.01
Q9Z1G4-3	Isoform A1-III of V-type proton ATPase 116 kDa subunit a isoform 1 OS=Mus musculus GN=Atp6v0a1 - [VPP1_MOUSE]	-0.25	-0.15	-0.29	-0.16	0.01

E9PWK1	Epoxide hydrolase 1 OS=Mus musculus GN=Ephx1 PE=2 SV=1 - [E9PWK1_MOUSE]	-0.28	-0.17	-0.32	-0.17	0.01
P58774	Tropomyosin beta chain OS=Mus musculus GN=Tpm2 PE=1 SV=1 - [TPM2_MOUSE]	0.19	0.27	0.12	0.17	0.01
B1AY85	Plexin domain-containing protein 2 OS=Mus musculus GN=Plxdc2 PE=2 SV=1 - [B1AY85_MOUSE]	0.16	0.19	0.29	0.32	0.01
Q61324-2	Isoform 2 of Aryl hydrocarbon receptor nuclear translocator 2 OS=Mus musculus GN=Arnt2 - [ARNT2_MOUSE]	-0.60	-0.39	-0.47	-0.27	0.01
Q6DFY2	Opioid binding protein/cell adhesion molecule-like OS=Mus musculus GN=Opcml PE=2 SV=1 - [Q6DFY2_MOUSE]	0.63	0.50	1.04	0.91	0.01
F6YIY0	Protein YIPF3 (Fragment) OS=Mus musculus GN=Yipf3 PE=4 SV=1 - [F6YIY0_MOUSE]	-0.21	-0.44	-0.32	-0.46	0.01
P03921	NADH-ubiquinone oxidoreductase chain 5 OS=Mus musculus GN=Mtnd5 PE=3 SV=2 - [NU5M_MOUSE]	-1.20	-0.75	-0.86	-0.56	0.01
E9Q5L7	PHD finger protein 10 OS=Mus musculus GN=Phf10 PE=2 SV=2 - [E9Q5L7_MOUSE]	0.36	0.50	0.66	0.79	0.01
Q3UMU9-4	Isoform 4 of Hepatoma-derived growth factor-related protein 2 OS=Mus musculus GN=Hdgfrp2 - [HDGR2_MOUSE]	0.36	0.49	0.75	0.71	0.01
Q9DAS9	Guanine nucleotide-binding protein G(I)/G(S)/G(O) subunit gamma-12 OS=Mus musculus GN=Gng12 PE=1 SV=3 - [GBG12_MOUSE]	-0.49	-0.56	-0.33	-0.28	0.01
Q8BRM2	RAB6-interacting golgin OS=Mus musculus GN=Gorab PE=1 SV=1 - [GORAB_MOUSE]	0.72	0.79	0.54	0.36	0.01
A2AEC2	Transcription elongation factor A protein-like 3 (Fragment) OS=Mus musculus GN=Tceal3 PE=2 SV=1 - [A2AEC2_MOUSE]	0.47	0.30	0.67	0.50	0.01
Q8R2G4-8	Isoform 7 of Ecto-ADP-ribosyltransferase 3 OS=Mus musculus GN=Art3 - [NAR3_MOUSE]	0.44	0.48	0.51	0.22	0.01
Q8CF89	TGF-beta-activated kinase 1 and MAP3K7-binding protein 1 OS=Mus musculus GN=Tab1 PE=1 SV=2 - [TAB1_MOUSE]	0.33	0.38	0.45	0.19	0.01
P97314	Cysteine and glycine-rich protein 2 OS=Mus musculus GN=Csrp2 PE=1 SV=3 - [CSRP2_MOUSE]	0.44	0.47	0.36	0.20	0.01
Q9D0J8	Parathymosin OS=Mus musculus GN=Ptms PE=2 SV=3 - [PTMS_MOUSE]	0.20	0.14	0.31	0.25	0.01
Q99N69	Leupaxin OS=Mus musculus GN=Lpxn PE=1 SV=2 - [LPXN_MOUSE]	-0.20	-0.24	-0.11	-0.16	0.01
E9PVW4	V-set and immunoglobulin domain-containing protein 10 OS=Mus musculus GN=Vsig10 PE=2 SV=1 - [E9PVW4_MOUSE]	-0.72	-0.60	-0.34	-0.44	0.01

P62331	ADP-ribosylation factor 6 OS=Mus musculus GN=Arf6 PE=1 SV=2 - [ARF6_MOUSE]	-0.96	-0.45	-0.90	-0.63	0.01
E9Q9Q2	Protein R3hdm1 OS=Mus musculus GN=R3hdm1 PE=2 SV=1 - [E9Q9Q2_MOUSE]	0.48	0.84	0.71	1.08	0.01
Q9CYG7	Mitochondrial import receptor subunit TOM34 OS=Mus musculus GN=Tomm34 PE=2 SV=1 - [TOM34_MOUSE]	-0.25	-0.29	-0.50	-0.43	0.01
P46938	Yorkie homolog OS=Mus musculus GN=Yap1 PE=1 SV=2 - [YAP1_MOUSE]	0.36	0.30	0.16	0.22	0.01
Q99JI1	Musculoskeletal embryonic nuclear protein 1 OS=Mus musculus GN=Mustn1 PE=2 SV=1 - [MSTN1_MOUSE]	-0.32	-0.27	-0.14	-0.22	0.01
Q8VCH8	UBX domain-containing protein 4 OS=Mus musculus GN=Ubxn4 PE=1 SV=1 - [UBXN4_MOUSE]	0.28	0.34	0.14	0.25	0.01
Q61735	Leukocyte surface antigen CD47 OS=Mus musculus GN=Cd47 PE=1 SV=2 - [CD47_MOUSE]	-0.32	-0.38	-0.17	-0.24	0.01
Q99MZ6	Unconventional myosin-VIIb OS=Mus musculus GN=Myo7b PE=1 SV=3 - [MYO7B_MOUSE]	-0.19	-0.18	-0.08	-0.19	0.01
Q9DAX2	Lipid phosphate phosphohydrolase 2 OS=Mus musculus GN=Ppap2c PE=1 SV=1 - [LPP2_MOUSE]	-1.08	-0.62	-1.03	-0.57	0.01
D3Z183	Major facilitator superfamily domain-containing protein 6 OS=Mus musculus GN=Mfsd6 PE=2 SV=1 - [D3Z183_MOUSE]	0.57	0.38	0.44	0.25	0.01
Q3UHQ6	Protein dopey-2 OS=Mus musculus GN=Dopey2 PE=2 SV=3 - [DOP2_MOUSE]	-0.70	-0.41	-0.95	-0.66	0.01
Q8R059	UDP-glucose 4-epimerase OS=Mus musculus GN=Gale PE=2 SV=1 - [GALE_MOUSE]	-0.16	-0.07	-0.15	-0.14	0.01
O70572	Sphingomyelin phosphodiesterase 2 OS=Mus musculus GN=Smpd2 PE=1 SV=1 - [NSMA_MOUSE]	-0.59	-0.29	-0.61	-0.40	0.01
Q3UM29	Conserved oligomeric Golgi complex subunit 7 OS=Mus musculus GN=Cog7 PE=2 SV=1 - [COG7_MOUSE]	-0.23	-0.36	-0.36	-0.52	0.01
Q5SSG2	Protein Mmp28 OS=Mus musculus GN=Mmp28 PE=2 SV=2 - [Q5SSG2_MOUSE]	0.57	1.05	0.69	1.16	0.01
Q8K0P3	TLD domain-containing protein 1 OS=Mus musculus GN=Tldc1 PE=2 SV=1 - [TLDC1_MOUSE]	-0.14	-0.32	-0.27	-0.22	0.01
Q8BV13	COP9 signalosome complex subunit 7b OS=Mus musculus GN=Cops7b PE=1 SV=1 - [CSN7B_MOUSE]	0.03	0.07	0.08	0.06	0.01
B2KF50	Protein Uhrf1bp1 OS=Mus musculus GN=Uhrf1bp1 PE=2 SV=1 - [B2KF50_MOUSE]	0.63	0.38	0.87	0.61	0.01

Q9D902	General transcription factor IIE subunit 2 OS=Mus musculus GN=Gtf2e2 PE=2 SV=2 - [T2EB_MOUSE]	0.27	0.41	0.48	0.62	0.01
P62320	Small nuclear ribonucleoprotein Sm D3 OS=Mus musculus GN=Snrpd3 PE=1 SV=1 - [SMD3_MOUSE]	0.08	0.10	0.08	0.15	0.01
Q9QZ08	N-acetyl-D-glucosamine kinase OS=Mus musculus GN=Nagk PE=2 SV=3 - [NAGK_MOUSE]	-0.14	-0.16	-0.27	-0.26	0.01
Q9DBY8	Nuclear valosin-containing protein-like OS=Mus musculus GN=Nvl PE=1 SV=1 - [NVL_MOUSE]	1.19	2.14	1.73	2.67	0.01
Q8K352	SAM and SH3 domain-containing protein 3 OS=Mus musculus GN=Sash3 PE=2 SV=2 - [SASH3_MOUSE]	0.60	0.62	1.11	1.07	0.01
Q61133	Glutathione S-transferase theta-2 OS=Mus musculus GN=Gstt2 PE=2 SV=4 - [GSTT2_MOUSE]	-0.40	-0.47	-0.20	-0.32	0.01
Q6PD28	Protein Ppp2r5b OS=Mus musculus GN=Ppp2r5b PE=2 SV=1 - [Q6PD28_MOUSE]	-0.12	-0.15	-0.21	-0.25	0.01
Q99KG3-2	Isoform 2 of RNA-binding protein 10 OS=Mus musculus GN=Rbm10 - [RBM10_MOUSE]	0.67	0.36	0.70	0.42	0.01
P37804	Transgelin OS=Mus musculus GN=Tagln PE=1 SV=3 - [TAGL_MOUSE]	-0.27	-0.43	-0.19	-0.32	0.01
Q05421	Cytochrome P450 2E1 OS=Mus musculus GN=Cyp2e1 PE=1 SV=1 - [CP2E1_MOUSE]	0.15	0.27	0.19	0.31	0.01
Q99L04	Dehydrogenase/reductase SDR family member 1 OS=Mus musculus GN=Dhrs1 PE=2 SV=1 - [DHRS1_MOUSE]	-0.37	-0.32	-0.58	-0.64	0.01
P05202	Aspartate aminotransferase, mitochondrial OS=Mus musculus GN=Got2 PE=1 SV=1 - [AATM_MOUSE]	0.06	0.05	0.08	0.10	0.01
Q8BYZ7-2	Isoform 2 of Engulfment and cell motility protein 3 OS=Mus musculus GN=Elmo3 - [ELMO3_MOUSE]	-0.90	-0.51	-0.80	-0.46	0.01
Q5SU71	E3 ubiquitin/ISG15 ligase TRIM25 OS=Mus musculus GN=Trim25 PE=2 SV=1 - [Q5SU71_MOUSE]	0.47	0.46	0.40	0.20	0.01
A2A5R8	Double-stranded RNA-binding protein Staufen homolog 1 OS=Mus musculus GN=Stau1 PE=2 SV=1 - [A2A5R8_MOUSE]	0.21	0.35	0.30	0.47	0.01
Q9D554	Splicing factor 3A subunit 3 OS=Mus musculus GN=Sf3a3 PE=2 SV=2 - [SF3A3_MOUSE]	0.35	0.21	0.48	0.43	0.01
Q91VM5	RNA binding motif protein, X-linked-like-1 OS=Mus musculus GN=Rbmxl1 PE=1 SV=1 - [RMXL1_MOUSE]	0.41	0.25	0.57	0.36	0.01
Q9D0M3-2	Isoform 2 of Cytochrome c1, heme protein, mitochondrial OS=Mus musculus GN=Cyc1 - [CY1_MOUSE]	-0.31	-0.48	-0.24	-0.47	0.01

Q60772	Cyclin-dependent kinase 4 inhibitor C OS=Mus musculus GN=Cdkn2c PE=2 SV=1 - [CDN2C_MOUSE]	0.21	0.40	0.21	0.35	0.01
B1ART1	Protein Vps13d OS=Mus musculus GN=Vps13d PE=2 SV=1 - [B1ART1_MOUSE]	-0.95	-0.44	-1.04	-0.98	0.01
P84096	Rho-related GTP-binding protein RhoG OS=Mus musculus GN=Rhog PE=2 SV=1 - [RHOG_MOUSE]	0.19	0.11	0.24	0.26	0.01
Q9R0Y5	Adenylate kinase isoenzyme 1 OS=Mus musculus GN=Ak1 PE=1 SV=1 - [KAD1_MOUSE]	0.21	0.16	0.15	0.09	0.01
Q3U2G2	Heat shock 70 kDa protein 4 OS=Mus musculus GN=Hspa4 PE=2 SV=1 - [Q3U2G2_MOUSE]	0.18	0.10	0.24	0.20	0.01
P59708	Pre-mRNA branch site protein p14 OS=Mus musculus GN=Sf3b14 PE=2 SV=1 - [PM14_MOUSE]	0.28	0.20	0.20	0.39	0.01
D3YVN7	Elongation factor Tu OS=Mus musculus GN=Gm9755 PE=3 SV=1 - [D3YVN7_MOUSE]	-0.23	-0.23	-0.11	-0.17	0.01
Q8CGM2	Retinitis pigmentosa 1-like 1 protein OS=Mus musculus GN=Rp1l1 PE=1 SV=1 - [RP1L1_MOUSE]	-0.84	-0.89	-0.46	-0.51	0.01
Q3UIL6-2	Isoform 2 of Pleckstrin homology domain-containing family A member 7 OS=Mus musculus GN=Plekha7 - [PKHA7_MOUSE]	0.28	0.39	0.61	0.52	0.01
Q9D1D4	Transmembrane emp24 domain-containing protein 10 OS=Mus musculus GN=Tmed10 PE=2 SV=1 - [TMEDA_MOUSE]	-0.20	-0.16	-0.08	-0.19	0.01
Q8BG95	Protein phosphatase 1 regulatory subunit 12B OS=Mus musculus GN=Ppp1r12b PE=1 SV=2 - [MYPT2_MOUSE]	0.25	0.12	0.28	0.24	0.01
Q9JMG2	C1GALT1-specific chaperone 1 OS=Mus musculus GN=C1galt1c1 PE=2 SV=1 - [C1GLC_MOUSE]	-0.32	-0.27	-0.43	-0.19	0.01
Q3TXY2	Protein Tex264 OS=Mus musculus GN=Tex264 PE=2 SV=1 - [Q3TXY2_MOUSE]	-0.11	-0.24	-0.25	-0.28	0.01
P70158	Acid sphingomyelinase-like phosphodiesterase 3a OS=Mus musculus GN=Smpdl3a PE=2 SV=2 - [ASM3A_MOUSE]	-0.25	-0.45	-0.24	-0.44	0.01
Q8C3W1	Uncharacterized protein C1orf198 homolog OS=Mus musculus PE=1 SV=1 - [CA198_MOUSE]	0.15	0.24	0.11	0.15	0.01
Q63844	Mitogen-activated protein kinase 3 OS=Mus musculus GN=Mapk3 PE=1 SV=5 - [MK03_MOUSE]	-0.17	-0.11	-0.26	-0.20	0.01
Q91VF2	Histamine N-methyltransferase OS=Mus musculus GN=Hnmt PE=1 SV=1 - [HNMT_MOUSE]	0.13	0.14	0.06	0.15	0.01
Q3TTY5	Keratin, type II cytoskeletal 2 epidermal OS=Mus musculus GN=Krt2 PE=1 SV=1 - [K22E_MOUSE]	-0.49	-0.89	-0.60	-1.01	0.01

Q9WV52	Pleckstrin-2 OS=Mus musculus GN=Plek2 PE=1 SV=1 [PLEK2_MOUSE]	0.63	1.02	1.05	1.47	0.01
B0R058	Glycerophosphocholine phosphodiesterase GPCPD1 OS=Mus musculus GN=Gpcpd1 PE=2 SV=1 - [B0R058_MOUSE]	-0.46	-0.28	-0.39	-0.21	0.01
Q9JJV2-3	Isoform 3 of Profilin-2 OS=Mus musculus GN=Pfn2 - [PROF2_MOUSE]	0.27	0.14	0.33	0.27	0.01
Q78T54-2	Isoform 2 of Vacuolar ATPase assembly integral membrane protein VMA21 OS=Mus musculus GN=Vma21 - [VMA21_MOUSE]	-0.29	-0.14	-0.20	-0.16	0.01
Q8BPB0	MOB kinase activator 1B OS=Mus musculus GN=Mob1b PE=1 SV=3 - [MOB1B_MOUSE]	-0.46	-0.24	-0.54	-0.35	0.01
Q9JKX6	ADP-sugar pyrophosphatase OS=Mus musculus GN=Nudt5 PE=1 SV=1 - [NUDT5_MOUSE]	-0.22	-0.12	-0.15	-0.25	0.01
D3Z3A0	MCG126099, isoform CRA_b OS=Mus musculus GN=Ppp1r2 PE=4 SV=1 - [D3Z3A0_MOUSE]	0.90	0.49	0.65	0.45	0.01
P58058	NAD kinase OS=Mus musculus GN=Nadk PE=1 SV=2 - [NADK_MOUSE]	0.81	1.27	0.73	1.47	0.01
A2ALS5	Protein Rap1gap OS=Mus musculus GN=Rap1gap PE=2 SV=1 - [A2ALS5_MOUSE]	0.62	0.97	0.42	0.76	0.01
E9PXX7	Thioredoxin domain-containing protein 5 OS=Mus musculus GN=Txndc5 PE=2 SV=1 - [E9PXX7_MOUSE]	0.21	0.12	0.27	0.28	0.01
E9Q2C4	Type II inositol 3,4-bisphosphate 4-phosphatase (Fragment) OS=Mus musculus GN=Inpp4b PE=2 SV=1 - [E9Q2C4_MOUSE]	-1.28	-0.73	-1.21	-0.66	0.01
Q99J36	THUMP domain-containing protein 1 OS=Mus musculus GN=Thumpd1 PE=1 SV=1 - [THUM1_MOUSE]	-0.65	-0.36	-0.64	-0.36	0.01
P42859-2	Isoform Short of Huntingtin OS=Mus musculus GN=Htt - [HD_MOUSE]	-0.31	-0.30	-0.24	-0.13	0.01
Q9CT10	Ran-binding protein 3 OS=Mus musculus GN=Ranbp3 PE=1 SV=2 - [RANB3_MOUSE]	-0.51	-0.47	-0.24	-0.33	0.01
F6RG68	FCH domain only protein 2 OS=Mus musculus GN=Fcho2 PE=4 SV=2 - [F6RG68_MOUSE]	0.10	0.20	0.26	0.22	0.01
G3X8U7	Serine/threonine-protein phosphatase OS=Mus musculus GN=Ppp3cb PE=3 SV=1 - [G3X8U7_MOUSE]	-0.46	-0.46	-0.28	-0.24	0.01
Q9D5V6	Synapse-associated protein 1 OS=Mus musculus GN=Syap1 PE=1 SV=1 - [SYAP1_MOUSE]	0.35	0.42	0.58	0.74	0.01
O55091	Protein IMPACT OS=Mus musculus GN=Impact PE=1 SV=2 - [IMPCT_MOUSE]	-0.55	-0.35	-0.45	-0.24	0.01

P20029	78 kDa glucose-regulated protein OS=Mus musculus GN=Hspa5 PE=1 SV=3 - [GRP78_MOUSE]	0.23	0.12	0.29	0.20	0.01
P02088	Hemoglobin subunit beta-1 OS=Mus musculus GN=Hbb-b1 PE=1 SV=2 - [HBB1_MOUSE]	0.19	0.25	0.11	0.16	0.01
Q9CQ22	Ragulator complex protein LAMTOR1 OS=Mus musculus GN=Lamtor1 PE=1 SV=1 - [LTOR1_MOUSE]	0.34	0.16	0.40	0.37	0.01
Q91W39	Nuclear receptor coactivator 5 OS=Mus musculus GN=Ncoa5 PE=1 SV=1 - [NCOA5_MOUSE]	0.24	0.21	0.27	0.11	0.01
Q8BW66	Solute carrier family 25 member 48 OS=Mus musculus GN=Slc25a48 PE=2 SV=2 - [S2548_MOUSE]	-0.55	-0.32	-0.77	-0.55	0.01
Q9CQV8-2	Isoform Short of 14-3-3 protein beta/alpha OS=Mus musculus GN=Ywhab - [1433B_MOUSE]	-0.47	-0.28	-0.32	-0.23	0.01
P02089	Hemoglobin subunit beta-2 OS=Mus musculus GN=Hbb-b2 PE=1 SV=2 - [HBB2_MOUSE]	0.21	0.11	0.28	0.23	0.01
E9Q852	Afadin OS=Mus musculus GN=Mllt4 PE=4 SV=2 - [E9Q852_MOUSE]	0.26	0.16	0.37	0.27	0.01
Q9CX86	Heterogeneous nuclear ribonucleoprotein A0 OS=Mus musculus GN=Hnrnpa0 PE=1 SV=1 - [ROA0_MOUSE]	0.44	0.31	0.68	0.63	0.01
Q80YR4-2	Isoform 2 of Zinc finger protein 598 OS=Mus musculus GN=Znf598 - [ZN598_MOUSE]	0.83	0.67	0.37	0.51	0.01
Q8VH51-2	Isoform 2 of RNA-binding protein 39 OS=Mus musculus GN=Rbm39 - [RBM39_MOUSE]	0.19	0.08	0.20	0.16	0.01
P42227-2	Isoform Stat3B of Signal transducer and activator of transcription 3 OS=Mus musculus GN=Stat3 - [STAT3_MOUSE]	-0.11	-0.08	-0.07	-0.04	0.01
E9QJV4	Pre-mRNA-processing factor 39 OS=Mus musculus GN=Prpf39 PE=2 SV=1 - [E9QJV4_MOUSE]	-0.24	-0.27	-0.13	-0.16	0.01
Q7TQH0-2	Isoform 2 of Ataxin-2-like protein OS=Mus musculus GN=Atxn2l - [ATX2L_MOUSE]	0.29	0.58	0.30	0.42	0.01
Q9QYI3	DnaJ homolog subfamily C member 7 OS=Mus musculus GN=Dnajc7 PE=1 SV=2 - [DNJC7_MOUSE]	-0.24	-0.20	-0.29	-0.12	0.01
Q8R3N6	THO complex subunit 1 OS=Mus musculus GN=Thoc1 PE=1 SV=1 - [THOC1_MOUSE]	-0.36	-0.25	-0.57	-0.40	0.01
Q9D8C4	Interferon-induced 35 kDa protein homolog OS=Mus musculus GN=Ifi35 PE=2 SV=3 - [IN35_MOUSE]	0.64	0.70	0.61	0.28	0.01
Q8BG51	Mitochondrial Rho GTPase 1 OS=Mus musculus GN=Rhot1 PE=2 SV=1 - [MIRO1_MOUSE]	-0.46	-0.30	-0.35	-0.20	0.01

M0QWS4	Ubiquitin-fold modifier conjugating enzyme 1, isoform CRA_c OS=Mus musculus GN=Ufc1 PE=4 SV=1 - [M0QWS4_MOUSE]	-0.24	-0.54	-0.35	-0.47	0.01
Q9D1T5	Proline-rich protein 15 OS=Mus musculus GN=Prr15 PE=2 SV=1 - [PRR15_MOUSE]	1.02	0.64	0.83	0.45	0.01
D3Z030	Leucine-rich repeat-containing protein 16A OS=Mus musculus GN=Lrrc16a PE=2 SV=2 - [D3Z030_MOUSE]	-0.45	-0.46	-0.20	-0.34	0.01
Q9QY23	Plakophilin-3 OS=Mus musculus GN=Pkp3 PE=1 SV=2 - [PKP3_MOUSE]	0.42	0.45	0.20	0.30	0.01
Q8VCC1	15-hydroxyprostaglandin dehydrogenase [NAD(+)] OS=Mus musculus GN=Hpgd PE=2 SV=1 - [PGDH_MOUSE]	-0.17	-0.30	-0.15	-0.29	0.01
Q80UW8	DNA-directed RNA polymerases I, II, and III subunit RPABC1 OS=Mus musculus GN=Polr2e PE=2 SV=1 - [RPAB1_MOUSE]	0.27	0.16	0.20	0.12	0.01
Q8C0T5-2	Isoform 2 of Signal-induced proliferation-associated 1-like protein 1 OS=Mus musculus GN=Sipa1l1 - [SI1L1_MOUSE]	0.56	0.44	0.37	0.24	0.01
O70475	UDP-glucose 6-dehydrogenase OS=Mus musculus GN=Ugdh PE=1 SV=1 - [UGDH_MOUSE]	0.09	0.05	0.05	0.09	0.01
Q9Z0H3-2	Isoform B of SWI/SNF-related matrix-associated actin-dependent regulator of chromatin subfamily B member 1 OS=Mus musculus GN=Smarcb1 - [SNF5_MOUSE]	0.11	0.15	0.22	0.25	0.01
Q62189	U1 small nuclear ribonucleoprotein A OS=Mus musculus GN=Snrpa PE=2 SV=3 - [SNRPA_MOUSE]	0.34	0.39	0.17	0.26	0.01
Q9D1F9	Protein Slc37a4 OS=Mus musculus GN=Slc37a4 PE=2 SV=1 - [Q9D1F9_MOUSE]	-0.86	-0.50	-1.19	-0.83	0.01
E9Q4Y4	Protein Cep192 OS=Mus musculus GN=Cep192 PE=2 SV=1 - [E9Q4Y4_MOUSE]	0.28	0.53	0.38	0.63	0.01
Q9QY96	Extracellular calcium-sensing receptor OS=Mus musculus GN=Casr PE=2 SV=2 - [CASR_MOUSE]	0.37	0.46	0.20	0.29	0.01
Q61136	Serine/threonine-protein kinase PRP4 homolog OS=Mus musculus GN=Prpf4b PE=1 SV=3 - [PRP4B_MOUSE]	0.28	0.20	0.46	0.36	0.01
Q8VHY0	Chondroitin sulfate proteoglycan 4 OS=Mus musculus GN=Cspg4 PE=1 SV=3 - [CSPG4_MOUSE]	0.14	0.20	0.08	0.17	0.01
Q8K010	5-oxoprolinase OS=Mus musculus GN=Oplah PE=2 SV=1 - [OPLA_MOUSE]	0.14	0.07	0.16	0.15	0.01
Q6GQV7	Erythroid differentiation-related factor 1 OS=Mus musculus GN=Edrf1 PE=2 SV=1 - [EDRF1_MOUSE]	-0.30	-0.28	-0.54	-0.52	0.01
F8VQC7	Kinectin OS=Mus musculus GN=Ktn1 PE=2 SV=1 - [F8VQC7_MOUSE]	0.71	0.62	0.45	0.31	0.01

Q60770	Syntaxin-binding protein 3 OS=Mus musculus GN=Stxbp3 PE=1 SV=1 - [STXB3_MOUSE]	-0.31	-0.16	-0.39	-0.27	0.01
Q06770	Corticosteroid-binding globulin OS=Mus musculus GN=Serpina6 PE=1 SV=1 - [CBG_MOUSE]	0.68	0.60	1.23	1.03	0.01
F6U6U5	Serine/threonine-protein kinase SIK3 (Fragment) OS=Mus musculus GN=Sik3 PE=4 SV=1 - [F6U6U5_MOUSE]	-0.42	-0.31	-0.68	-0.45	0.01
O35465-2	Isoform 2 of Peptidyl-prolyl cis-trans isomerase FKBP8 OS=Mus musculus GN=Fkbp8 - [FKBP8_MOUSE]	-0.27	-0.19	-0.41	-0.41	0.01
Q9R257	Heme-binding protein 1 OS=Mus musculus GN=Hebp1 PE=1 SV=2 - [HEBP1_MOUSE]	0.38	0.19	0.48	0.35	0.01
F8VQB6	Unconventional myosin-X OS=Mus musculus GN=Myo10 PE=1 SV=1 - [MYO10_MOUSE]	0.57	0.87	1.05	1.34	0.01
Q62245	Son of sevenless homolog 1 OS=Mus musculus GN=Sos1 PE=1 SV=2 - [SOS1_MOUSE]	1.44	1.29	0.84	0.68	0.01
O55074	A-kinase anchor protein 7 isoform alpha OS=Mus musculus GN=Akap7 PE=1 SV=4 - [AKA7A_MOUSE]	-0.44	-0.81	-0.49	-0.87	0.01
F2Z4A3	Protein Fat1 OS=Mus musculus GN=Fat1 PE=2 SV=1 - [F2Z4A3_MOUSE]	-0.30	-0.45	-0.24	-0.50	0.01
Q6KCD5-2	Isoform 2 of Nipped-B-like protein OS=Mus musculus GN=Nipbl - [NIPBL_MOUSE]	0.44	0.33	0.68	0.71	0.01
A2AWS5	Histone deacetylase 5 OS=Mus musculus GN=Hdac5 PE=2 SV=1 - [A2AWS5_MOUSE]	0.38	0.36	0.22	0.19	0.01
P53395	Lipoamide acyltransferase component of branched-chain alpha-keto acid dehydrogenase complex, mitochondrial OS=Mus musculus GN=Dbt PE=2 SV=2 - [ODB2_MOUSE]	-0.12	-0.17	-0.07	-0.15	0.01
A2AGS2	Dual-specificity mitogen-activated protein kinase kinase 6 OS=Mus musculus GN=Map2k6 PE=2 SV=1 - [A2AGS2_MOUSE]	-0.35	-0.46	-0.67	-0.77	0.01
O55033	Cytoplasmic protein NCK2 OS=Mus musculus GN=Nck2 PE=1 SV=1 - [NCK2_MOUSE]	0.30	0.40	0.54	0.67	0.01
Q922E6	FAST kinase domain-containing protein 2 OS=Mus musculus GN=Fastkd2 PE=2 SV=2 - [FAKD2_MOUSE]	0.31	0.17	0.36	0.21	0.01
Q3UKX1	Protein arginine N-methyltransferase 2 OS=Mus musculus GN=Prmt2 PE=2 SV=1 - [Q3UKX1_MOUSE]	-0.63	-0.48	-1.06	-0.90	0.01
Q3UC65-3	Isoform 3 of UPF0471 protein C1orf63 homolog OS=Mus musculus GN=D4Wsu53e - [CA063_MOUSE]	2.10	1.17	1.99	1.07	0.01
J3QJV7	Rab GTPase-binding effector protein 1 OS=Mus musculus GN=Rabep1 PE=4 SV=1 - [J3QJV7_MOUSE]	0.39	0.42	0.22	0.22	0.01

Q9R1Z8	Vinexin OS=Mus musculus GN=Sorbs3 PE=1 SV=1 - [VINEX_MOUSE]	0.35	0.20	0.25	0.16	0.01
Q8K0F1-2	Isoform 2 of TBC1 domain family member 23 OS=Mus musculus GN=Tbc1d23 - [TBC23_MOUSE]	-0.81	-0.43	-0.82	-0.45	0.01
P23492	Purine nucleoside phosphorylase OS=Mus musculus GN=Pnp PE=1 SV=2 - [PNPH_MOUSE]	0.18	0.35	0.18	0.25	0.01
O54774	AP-3 complex subunit delta-1 OS=Mus musculus GN=Ap3d1 PE=1 SV=1 - [AP3D1_MOUSE]	-0.24	-0.17	-0.20	-0.36	0.01
P97930	Thymidylate kinase OS=Mus musculus GN=Dtymk PE=2 SV=2 - [KTHY_MOUSE]	-0.39	-0.22	-0.34	-0.19	0.01
P59997-2	Isoform 2 of Lysine-specific demethylase 2A OS=Mus musculus GN=Kdm2a - [KDM2A_MOUSE]	0.50	0.41	0.86	0.54	0.01
Q8K4L0	ATP-dependent RNA helicase DDX54 OS=Mus musculus GN=Ddx54 PE=1 SV=1 - [DDX54_MOUSE]	0.64	0.36	0.88	0.60	0.01
Q5U4C3	Splicing factor, arginine/serine-rich 19 OS=Mus musculus GN=Scaf1 PE=1 SV=1 - [SFR19_MOUSE]	0.24	0.52	0.54	0.60	0.01
P27659	60S ribosomal protein L3 OS=Mus musculus GN=Rpl3 PE=2 SV=3 - [RL3_MOUSE]	-0.32	-0.28	-0.16	-0.17	0.01
Q5SNZ0-3	Isoform 3 of Girdin OS=Mus musculus GN=Ccdc88a - [GRDN_MOUSE]	-1.01	-0.54	-1.07	-0.60	0.01
D3YZX8	Signal recognition particle 9 kDa protein OS=Mus musculus GN=Srp9 PE=2 SV=1 - [D3YZX8_MOUSE]	0.28	0.15	0.36	0.23	0.01
B1ATI9	Growth arrest-specific protein 7 OS=Mus musculus GN=Gas7 PE=4 SV=1 - [B1ATI9_MOUSE]	0.64	0.52	0.48	0.25	0.01
Q3TMX0	MCG4375, isoform CRA_b OS=Mus musculus GN=Sdcbp PE=2 SV=1 - [Q3TMX0_MOUSE]	0.22	0.31	0.24	0.46	0.01
Q9DBH5	Vesicular integral-membrane protein VIP36 OS=Mus musculus GN=Lman2 PE=2 SV=2 - [LMAN2_MOUSE]	-0.06	-0.08	-0.13	-0.13	0.01
Q8QZS1	3-hydroxyisobutyryl-CoA hydrolase, mitochondrial OS=Mus musculus GN=Hibch PE=1 SV=1 - [HIBCH_MOUSE]	-0.42	-0.57	-0.23	-0.45	0.01
P51660	Peroxisomal multifunctional enzyme type 2 OS=Mus musculus GN=Hsd17b4 PE=1 SV=3 - [DHB4_MOUSE]	-0.14	-0.11	-0.23	-0.14	0.01
Q9DB15	39S ribosomal protein L12, mitochondrial OS=Mus musculus GN=Mrpl12 PE=1 SV=2 - [RM12_MOUSE]	0.10	0.07	0.17	0.12	0.01
Q9R112	Sulfide:quinone oxidoreductase, mitochondrial OS=Mus musculus GN=Sqrdl PE=2 SV=3 - [SQRD_MOUSE]	-0.19	-0.16	-0.11	-0.08	0.01

Q9D967	Magnesium-dependent phosphatase 1 OS=Mus musculus GN=Mdp1 PE=1 SV=1 - [MGDP1_MOUSE]	-0.22	-0.10	-0.26	-0.20	0.01
Q8BS28	Protein Gm10032 OS=Mus musculus GN=Gm10032 PE=2 SV=1 - [Q8BS28_MOUSE]	0.54	0.95	0.46	0.86	0.01
O70503	Estradiol 17-beta-dehydrogenase 12 OS=Mus musculus GN=Hsd17b12 PE=2 SV=1 - [DHB12_MOUSE]	-0.36	-0.33	-0.16	-0.23	0.01
Q3TL44	NLR family member X1 OS=Mus musculus GN=Nlrx1 PE=2 SV=1 - [NLRX1_MOUSE]	-0.71	-0.39	-0.92	-0.58	0.01
Q9JIA6	Galactokinase OS=Mus musculus GN=Galk1 PE=2 SV=1 - [Q9JIA6_MOUSE]	-0.24	-0.24	-0.45	-0.42	0.01
P49722	Proteasome subunit alpha type-2 OS=Mus musculus GN=Psma2 PE=1 SV=3 - [PSA2_MOUSE]	0.19	0.11	0.23	0.26	0.01
E9PVP1	Aurora kinase B OS=Mus musculus GN=Aim1 PE=2 SV=1 - [E9PVP1_MOUSE]	0.38	0.32	0.67	0.62	0.01
E9Q6A7	Protein Bptf OS=Mus musculus GN=Bptf PE=2 SV=1 - [E9Q6A7_MOUSE]	0.59	0.32	0.80	0.67	0.01
O55106	Striatin OS=Mus musculus GN=Strn PE=1 SV=2 - [STRN_MOUSE]	0.58	0.87	0.36	0.63	0.01
B2RWE3	CDGSH iron sulfur domain 3 OS=Mus musculus GN=Cisd3 PE=2 SV=1 - [B2RWE3_MOUSE]	-0.84	-0.63	-0.40	-0.46	0.01
O88593	Peptidoglycan recognition protein 1 OS=Mus musculus GN=Pglyrp1 PE=2 SV=1 - [PGRP1_MOUSE]	0.56	0.32	0.46	0.25	0.01
O35737	Heterogeneous nuclear ribonucleoprotein H OS=Mus musculus GN=Hnrnph1 PE=1 SV=3 - [HNRH1_MOUSE]	0.40	0.33	0.16	0.30	0.01
Q91X91	Nicotinate-nucleotide pyrophosphorylase [carboxylating] OS=Mus musculus GN=Qprt PE=2 SV=1 - [NADC_MOUSE]	0.77	0.61	0.49	0.32	0.01
Q9DCI9	39S ribosomal protein L32, mitochondrial OS=Mus musculus GN=Mrpl32 PE=2 SV=1 - [RM32_MOUSE]	-0.49	-0.38	-0.23	-0.26	0.01
F2Z461	E3 ISG15--protein ligase Herc6 OS=Mus musculus GN=Herc6 PE=2 SV=1 - [HERC6_MOUSE]	1.63	0.92	2.27	1.55	0.01
Q9QZI9	Serine incorporator 3 OS=Mus musculus GN=Serinc3 PE=1 SV=2 - [SERC3_MOUSE]	-1.37	-0.74	-1.11	-0.64	0.01
Q3USN8	Protein Gm10654 OS=Mus musculus GN=Gm10654 PE=2 SV=1 - [Q3USN8_MOUSE]	0.28	0.33	0.54	0.58	0.01
Q8C845	EF-hand domain-containing protein D2 OS=Mus musculus GN=Efhd2 PE=2 SV=1 - [Q8C845_MOUSE]	0.23	0.20	0.43	0.31	0.01

E9Q8P5	PDZ and LIM domain protein 5 OS=Mus musculus GN=Pdlim5 PE=2 SV=2 - [E9Q8P5_MOUSE]	0.74	0.94	1.18	1.64	0.01
Q9QZC7-2	Isoform 2 of Pleckstrin homology domain-containing family B member 2 OS=Mus musculus GN=Plekhb2 - [PKHB2_MOUSE]	-0.14	-0.24	-0.23	-0.34	0.01
E9Q6R7	Protein Utrn OS=Mus musculus GN=Utrn PE=2 SV=1 - [E9Q6R7_MOUSE]	-0.09	-0.09	-0.11	-0.04	0.01
E9Q5F9	Protein Setd2 OS=Mus musculus GN=Setd2 PE=2 SV=1 - [E9Q5F9_MOUSE]	-0.96	-0.95	-0.52	-0.51	0.01
Q920D3	Mediator of RNA polymerase II transcription subunit 28 OS=Mus musculus GN=Med28 PE=2 SV=2 - [MED28_MOUSE]	1.03	0.47	1.08	0.72	0.01
P70452	Syntaxin-4 OS=Mus musculus GN=Stx4 PE=1 SV=1 - [STX4_MOUSE]	-0.22	-0.11	-0.29	-0.21	0.01
A3KGF9	Uncharacterized protein C15orf52 homolog OS=Mus musculus PE=1 SV=1 - [CO052_MOUSE]	-0.14	-0.32	-0.33	-0.34	0.01
D3Z091	Protein Pik3c2b (Fragment) OS=Mus musculus GN=Pik3c2b PE=2 SV=1 - [D3Z091_MOUSE]	0.32	0.40	0.62	0.69	0.01
Q921M4-2	Isoform 2 of Golgin subfamily A member 2 OS=Mus musculus GN=Golga2 - [GOGA2_MOUSE]	0.50	0.71	0.29	0.62	0.01
D3YXJ5	Protein Fam84b OS=Mus musculus GN=Fam84b PE=4 SV=1 - [D3YXJ5_MOUSE]	-0.41	-0.58	-0.34	-0.25	0.01
D3YXN9	MCG59255 OS=Mus musculus GN=5830403L16Rik PE=4 SV=1 - [D3YXN9_MOUSE]	0.67	0.47	1.09	0.88	0.01
Q9CVD2	Ataxin-3 OS=Mus musculus GN=Atxn3 PE=1 SV=2 - [ATX3_MOUSE]	0.89	0.53	0.76	0.40	0.01
Q9DA03	Complex III assembly factor LYRM7 OS=Mus musculus GN=Lyrm7 PE=2 SV=2 - [LYRM7_MOUSE]	0.19	0.20	0.36	0.37	0.01
P27641	X-ray repair cross-complementing protein 5 OS=Mus musculus GN=Xrcc5 PE=2 SV=4 - [XRCC5_MOUSE]	-0.51	-0.49	-0.20	-0.45	0.01
P14211	Calreticulin OS=Mus musculus GN=Calr PE=1 SV=1 - [CALR_MOUSE]	0.22	0.17	0.37	0.33	0.01
Q8VEK3	Heterogeneous nuclear ribonucleoprotein U OS=Mus musculus GN=Hnrnpu PE=1 SV=1 - [HNRPU_MOUSE]	0.20	0.12	0.30	0.24	0.01
Q9EPK7-2	Isoform 2 of Exportin-7 OS=Mus musculus GN=Xpo7 - [XPO7_MOUSE]	-1.23	-0.59	-1.25	-0.76	0.01
Q9Z1T1	AP-3 complex subunit beta-1 OS=Mus musculus GN=Ap3b1 PE=1 SV=2 - [AP3B1_MOUSE]	-0.41	-0.31	-0.19	-0.23	0.01

H3BJN8	RWD domain-containing protein 4 (Fragment) OS=Mus musculus GN=Rwdd4a PE=2 SV=1 - [H3BJN8_MOUSE]	0.39	0.51	0.20	0.41	0.01
P38060	Hydroxymethylglutaryl-CoA lyase, mitochondrial OS=Mus musculus GN=Hmgcl PE=1 SV=2 - [HMGCL_MOUSE]	0.22	0.14	0.23	0.10	0.01
Q9QYK4	Heparan-sulfate 6-O-sulfotransferase 3 OS=Mus musculus GN=Hs6st3 PE=1 SV=2 - [H6ST3_MOUSE]	0.43	0.83	0.55	0.95	0.01
E9QPG8	Deleted in malignant brain tumors 1 protein OS=Mus musculus GN=Dmbt1 PE=2 SV=1 - [E9QPG8_MOUSE]	0.69	0.78	0.35	0.45	0.01
Q5SWT3	Solute carrier family 25 member 35 OS=Mus musculus GN=Slc25a35 PE=2 SV=2 - [S2535_MOUSE]	-0.35	-0.18	-0.44	-0.29	0.01
Q9R097	Kunitz-type protease inhibitor 1 OS=Mus musculus GN=Spint1 PE=1 SV=2 - [SPIT1_MOUSE]	-0.37	-0.26	-0.35	-0.16	0.01
D3Z460	Protein Gm1330 OS=Mus musculus GN=Gm1330 PE=2 SV=1 - [D3Z460_MOUSE]	-0.16	-0.31	-0.21	-0.36	0.01
Q9QUM9	Proteasome subunit alpha type-6 OS=Mus musculus GN=Psma6 PE=1 SV=1 - [PSA6_MOUSE]	0.11	0.20	0.10	0.19	0.01
Q8C257	Il18r1 protein OS=Mus musculus GN=Il18r1 PE=2 SV=1 - [Q8C257_MOUSE]	-0.50	-0.26	-0.52	-0.28	0.01
Q8BLR5	PH and SEC7 domain-containing protein 4 OS=Mus musculus GN=Psd4 PE=2 SV=1 - [PSD4_MOUSE]	0.24	0.46	0.39	0.60	0.01
Q9DCH6	AN1-type zinc finger protein 6 OS=Mus musculus GN=Zfand6 PE=1 SV=1 - [ZFAN6_MOUSE]	0.46	0.66	0.89	1.09	0.01
Q9JLI8	Squamous cell carcinoma antigen recognized by T-cells 3 OS=Mus musculus GN=Sart3 PE=2 SV=1 - [SART3_MOUSE]	0.17	0.09	0.12	0.20	0.01
E9Q409	Protein Prss32 OS=Mus musculus GN=Prss32 PE=2 SV=1 - [E9Q409_MOUSE]	-0.23	-0.14	-0.22	-0.10	0.01
Q60692	Proteasome subunit beta type-6 OS=Mus musculus GN=Psmb6 PE=1 SV=3 - [PSB6_MOUSE]	0.15	0.14	0.28	0.26	0.01
P26443	Glutamate dehydrogenase 1, mitochondrial OS=Mus musculus GN=Glud1 PE=1 SV=1 - [DHE3_MOUSE]	0.14	0.09	0.22	0.18	0.01
Q6PCX9	E3 ubiquitin-protein ligase TRIM37 OS=Mus musculus GN=Trim37 PE=2 SV=1 - [TRI37_MOUSE]	0.25	0.34	0.14	0.22	0.01
P32067	Lupus La protein homolog OS=Mus musculus GN=Ssb PE=2 SV=1 - [LA_MOUSE]	-0.19	-0.21	-0.09	-0.13	0.01
Q9ER05	Chymopasin OS=Mus musculus GN=Ctrl PE=2 SV=1 - [Q9ER05_MOUSE]	0.38	0.30	0.15	0.26	0.01

E9PXY1	Cullin-4B OS=Mus musculus GN=Cul4b PE=3 SV=2 - [E9PXY1_MOUSE]	-0.38	-0.65	-0.37	-0.31	0.01
Q9DCH2	Ribonuclease P protein subunit p20 OS=Mus musculus GN=Pop7 PE=2 SV=1 - [POP7_MOUSE]	-0.14	-0.27	-0.17	-0.30	0.01
Q8BTM9-4	Isoform 4 of RAS guanyl-releasing protein 4 OS=Mus musculus GN=Rasgrp4 - [GRP4_MOUSE]	-0.07	-0.06	-0.04	-0.03	0.01
Q61329	Zinc finger homeobox protein 3 OS=Mus musculus GN=Zfhx3 PE=1 SV=1 - [ZFHX3_MOUSE]	-0.11	-0.11	-0.06	-0.06	0.01
P00158	Cytochrome b OS=Mus musculus GN=Mt-Cyb PE=2 SV=1 - [CYB_MOUSE]	-1.26	-1.78	-0.71	-1.22	0.01
A2AWT6	Nucleolar transcription factor 1 OS=Mus musculus GN=Ubtf PE=4 SV=1 - [A2AWT6_MOUSE]	0.27	0.18	0.40	0.23	0.01
Q9D880	Mitochondrial import inner membrane translocase subunit TIM50 OS=Mus musculus GN=Timm50 PE=1 SV=1 - [TIM50_MOUSE]	-0.58	-0.26	-0.48	-0.34	0.01
Q8VD26-3	Isoform 3 of Transmembrane protein 143 OS=Mus musculus GN=Tmem143 - [TM143_MOUSE]	0.61	0.37	0.58	0.28	0.01
P19157	Glutathione S-transferase P 1 OS=Mus musculus GN=Gstp1 PE=1 SV=2 - [GSTP1_MOUSE]	-0.12	-0.20	-0.17	-0.29	0.01
O09106	Histone deacetylase 1 OS=Mus musculus GN=Hdac1 PE=1 SV=1 - [HDAC1_MOUSE]	0.52	0.30	0.76	0.55	0.01
O70582	Arachidonate 12-lipoxygenase, 12R-type OS=Mus musculus GN=Alox12b PE=2 SV=1 - [LX12B_MOUSE]	0.21	0.14	0.32	0.20	0.01
Q80UK0	SEC14 domain and spectrin repeat-containing protein 1 OS=Mus musculus GN=Sestd1 PE=2 SV=1 - [SESD1_MOUSE]	-0.25	-0.45	-0.44	-0.64	0.01
Q8CI08	SLAIN motif-containing protein 2 OS=Mus musculus GN=Slain2 PE=1 SV=2 - [SLAI2_MOUSE]	0.68	0.88	0.69	0.33	0.01
Q9R0Q1	Synaptotagmin-like protein 4 OS=Mus musculus GN=Sytl4 PE=1 SV=1 - [SYTL4_MOUSE]	0.87	0.64	0.58	0.35	0.01
Q9D0M1	Phosphoribosyl pyrophosphate synthase-associated protein 1 OS=Mus musculus GN=Prpsap1 PE=1 SV=1 - [KPRA_MOUSE]	0.16	0.34	0.28	0.41	0.01
Q9D2F7	Nuclear pore membrane glycoprotein 210-like OS=Mus musculus GN=Nup210l PE=2 SV=2 - [P210L_MOUSE]	0.34	0.23	0.53	0.50	0.01
B7ZC85	Serine/threonine-protein kinase tousled-like 2 OS=Mus musculus GN=Tlk2 PE=2 SV=1 - [B7ZC85_MOUSE]	0.27	0.16	0.39	0.28	0.01
Q61239	Protein farnesyltransferase/geranylgeranyltransferase type-1 subunit alpha OS=Mus musculus GN=Fnta PE=1 SV=1 - [FNTA_MOUSE]	0.12	0.17	0.18	0.28	0.01

Q9JLN9	Serine/threonine-protein kinase mTOR OS=Mus musculus GN=Mtor PE=1 SV=2 - [MTOR_MOUSE]	-0.17	-0.24	-0.37	-0.22	0.01
D3YXC2	Mitochondrial carrier homolog 1 (Fragment) OS=Mus musculus GN=Mtch1 PE=2 SV=1 - [D3YXC2_MOUSE]	-0.50	-0.29	-0.72	-0.51	0.01
Q8K3Z9	Nuclear envelope pore membrane protein POM 121 OS=Mus musculus GN=Pom121 PE=1 SV=2 - [PO121_MOUSE]	0.26	0.49	0.24	0.44	0.01
Q8BGT7	Survival of motor neuron-related-splicing factor 30 OS=Mus musculus GN=Smndc1 PE=2 SV=1 - [SPF30_MOUSE]	0.26	0.18	0.41	0.25	0.01
O88673	Diacylglycerol kinase alpha OS=Mus musculus GN=Dgka PE=2 SV=2 - [DGKA_MOUSE]	-0.04	-0.05	-0.02	-0.03	0.01
Q61205	Platelet-activating factor acetylhydrolase IB subunit gamma OS=Mus musculus GN=Pafah1b3 PE=1 SV=1 - [PA1B3_MOUSE]	-0.20	-0.26	-0.16	-0.11	0.01
Q8C078	Calcium/calmodulin-dependent protein kinase kinase 2 OS=Mus musculus GN=Camkk2 PE=1 SV=2 - [KKCC2_MOUSE]	0.34	0.43	0.67	0.76	0.01
P00860	Ornithine decarboxylase OS=Mus musculus GN=Odc1 PE=1 SV=2 - [DCOR_MOUSE]	0.11	0.08	0.19	0.16	0.01
Q8K0T7	Protein unc-13 homolog C OS=Mus musculus GN=Unc13c PE=1 SV=3 - [UN13C_MOUSE]	-0.27	-0.33	-0.52	-0.59	0.01
Q32ME9	Desmocollin 1 OS=Mus musculus GN=Dsc1 PE=2 SV=1 - [Q32ME9_MOUSE]	-0.76	-0.39	-0.82	-0.46	0.01
Q9EQN3	TSC22 domain family protein 4 OS=Mus musculus GN=Tsc22d4 PE=2 SV=2 - [T22D4_MOUSE]	0.32	0.31	0.34	0.13	0.01
Q60649	Caseinolytic peptidase B protein homolog OS=Mus musculus GN=Clpb PE=1 SV=1 - [CLPB_MOUSE]	-0.16	-0.13	-0.29	-0.27	0.01
Q9QZM8-2	Isoform 2 of F-box only protein 17 OS=Mus musculus GN=Fbxo17 - [FBX17_MOUSE]	0.23	0.13	0.19	0.10	0.01
D6RDS1	Lipase maturation factor 1 OS=Mus musculus GN=Lmf1 PE=2 SV=1 - [D6RDS1_MOUSE]	0.12	0.22	0.10	0.20	0.01
Q8VHC3	Selenoprotein M OS=Mus musculus GN=Selm PE=1 SV=3 - [SELM_MOUSE]	0.13	0.13	0.26	0.19	0.01
H3BKM2	Nuclear-interacting partner of ALK OS=Mus musculus GN=Zc3hc1 PE=2 SV=1 - [H3BKM2_MOUSE]	0.77	0.50	0.59	0.31	0.01
Q9CPX6	Ubiquitin-like-conjugating enzyme ATG3 OS=Mus musculus GN=Atg3 PE=1 SV=1 - [ATG3_MOUSE]	0.24	0.13	0.34	0.27	0.01
A2BIN0	Major urinary proteins 11 and 8 OS=Mus musculus GN=Mup11 PE=2 SV=1 - [A2BIN0_MOUSE]	0.25	0.49	0.36	0.59	0.01

Q920A5	Retinoid-inducible serine carboxypeptidase OS=Mus musculus GN=Scpep1 PE=2 SV=2 - [RISC_MOUSE]	-0.06	-0.13	-0.12	-0.07	0.01
Q9Z211	Peroxisomal membrane protein 11A OS=Mus musculus GN=Pex11a PE=2 SV=1 - [PX11A_MOUSE]	-0.14	-0.07	-0.13	-0.07	0.01
E9QN52	Leucine-rich repeat flightless-interacting protein 2 OS=Mus musculus GN=Lrrfip2 PE=2 SV=1 - [E9QN52_MOUSE]	0.27	0.49	0.22	0.41	0.01
P16546-2	Isoform 2 of Spectrin alpha chain, non-erythrocytic 1 OS=Mus musculus GN=Sptan1 - [SPTN1_MOUSE]	0.41	0.27	0.65	0.44	0.01
P50295	Arylamine N-acetyltransferase 2 OS=Mus musculus GN=Nat2 PE=1 SV=1 - [ARY2_MOUSE]	0.35	0.14	0.31	0.36	0.01
A2A9K3	Centrosomal protein of 85 kDa OS=Mus musculus GN=Cep85 PE=2 SV=1 - [A2A9K3_MOUSE]	0.73	0.86	0.37	0.50	0.01
A2AN08-3	Isoform 3 of E3 ubiquitin-protein ligase UBR4 OS=Mus musculus GN=Ubr4 - [UBR4_MOUSE]	-0.27	-0.17	-0.22	-0.11	0.01
G5E8K5-3	Isoform 3 of Ankyrin-3 OS=Mus musculus GN=Ank3 - [ANK3_MOUSE]	0.15	0.25	0.12	0.26	0.01
Q9QXD1	Peroxisomal acyl-coenzyme A oxidase 2 OS=Mus musculus GN=Acox2 PE=1 SV=2 - [ACOX2_MOUSE]	0.67	0.50	0.44	0.26	0.01
Q5FWX7	Oxysterol-binding protein OS=Mus musculus GN=Osbpl9 PE=2 SV=1 - [Q5FWX7_MOUSE]	0.38	0.24	0.33	0.16	0.01
Q4VC33	Macrophage erythroblast attacher OS=Mus musculus GN=Maea PE=1 SV=1 - [MAEA_MOUSE]	-0.89	-0.45	-1.06	-0.63	0.01
Q8R344	Coiled-coil domain-containing protein 12 OS=Mus musculus GN=Ccdc12 PE=1 SV=2 - [CCD12_MOUSE]	0.36	0.69	0.42	0.77	0.01
E9Q467	Protein Abcc4 OS=Mus musculus GN=Abcc4 PE=2 SV=1 - [E9Q467_MOUSE]	-1.17	-0.83	-0.80	-0.46	0.01
Q9D2P4	Ubiquitin-related modifier 1 homolog OS=Mus musculus GN=Urm1 PE=1 SV=1 - [URM1_MOUSE]	0.20	0.36	0.15	0.30	0.01
F8VPX6	Protein Speer4a OS=Mus musculus GN=Speer4a PE=4 SV=1 - [F8VPX6_MOUSE]	-1.72	-0.91	-2.32	-1.52	0.01
Q9WTK5	Nuclear factor NF-kappa-B p100 subunit OS=Mus musculus GN=Nfkb2 PE=1 SV=1 - [NFKB2_MOUSE]	0.22	0.35	0.21	0.44	0.01
Q8BMK4	Cytoskeleton-associated protein 4 OS=Mus musculus GN=Ckap4 PE=2 SV=2 - [CKAP4_MOUSE]	-0.23	-0.11	-0.20	-0.12	0.01
Q9JMG7	Hepatoma-derived growth factor-related protein 3 OS=Mus musculus GN=Hdgfrp3 PE=1 SV=2 - [HDGR3_MOUSE]	0.23	0.21	0.45	0.32	0.01

B1AZI6	THO complex subunit 2 OS=Mus musculus GN=Thoc2 PE=2 SV=1 - [THOC2_MOUSE]	-0.74	-0.51	-0.52	-0.29	0.01
P49135	TFIIH basal transcription factor complex helicase XPB subunit OS=Mus musculus GN=Ercc3 PE=2 SV=1 - [ERCC3_MOUSE]	-0.36	-0.19	-0.46	-0.29	0.01
P23506	Protein-L-isoaspartate(D-aspartate) O-methyltransferase OS=Mus musculus GN=Pcmt1 PE=1 SV=3 - [PIMT_MOUSE]	-0.27	-0.30	-0.13	-0.18	0.01
Q60648	Ganglioside GM2 activator OS=Mus musculus GN=Gm2a PE=1 SV=2 - [SAP3_MOUSE]	-0.53	-0.46	-0.35	-0.21	0.01
Q6ZWX6	Eukaryotic translation initiation factor 2 subunit 1 OS=Mus musculus GN=Eif2s1 PE=1 SV=3 - [IF2A_MOUSE]	-0.37	-0.17	-0.43	-0.27	0.01
Q922H2	[Pyruvate dehydrogenase [lipoamide]] kinase isozyme 3, mitochondrial OS=Mus musculus GN=Pdk3 PE=2 SV=1 - [PDK3_MOUSE]	0.43	0.47	0.19	0.31	0.01
A2ALN0	C-C motif chemokine 22 OS=Mus musculus GN=Abcd1 PE=2 SV=1 - [A2ALN0_MOUSE]	-0.55	-0.28	-0.65	-0.38	0.01
Q8BRV5	Uncharacterized protein KIAA1671 OS=Mus musculus GN=Kiaa1671 PE=2 SV=1 - [K1671_MOUSE]	-0.13	-0.19	-0.26	-0.32	0.01
Q8R238	Serine dehydratase-like OS=Mus musculus GN=Sdsl PE=2 SV=1 - [SDSL_MOUSE]	-0.37	-0.20	-0.53	-0.36	0.01
Q9DBT5	AMP deaminase 2 OS=Mus musculus GN=Ampd2 PE=1 SV=1 - [AMPD2_MOUSE]	0.27	0.45	0.20	0.42	0.01
P61164	Alpha-centractin OS=Mus musculus GN=Actr1a PE=2 SV=1 - [ACTZ_MOUSE]	-0.17	-0.21	-0.18	-0.08	0.01
Q9D289	Trafficking protein particle complex subunit 6B OS=Mus musculus GN=Trappc6b PE=2 SV=1 - [TPC6B_MOUSE]	-0.84	-0.64	-0.61	-0.31	0.01
Q9Z2D6	Methyl-CpG-binding protein 2 OS=Mus musculus GN=Mecp2 PE=1 SV=1 - [MECP2_MOUSE]	0.22	0.27	0.44	0.48	0.01
Q8C156	Condensin complex subunit 2 OS=Mus musculus GN=Ncaph PE=2 SV=1 - [CND2_MOUSE]	0.22	0.44	0.34	0.55	0.01
Q3ZK22	Vezatin OS=Mus musculus GN=Vezt PE=1 SV=2 - [VEZA_MOUSE]	0.91	1.89	1.31	2.18	0.01
E0CY52	Uncharacterized protein OS=Mus musculus GN=Slc25a54 PE=2 SV=1 - [E0CY52_MOUSE]	-0.85	-0.65	-0.53	-0.34	0.01
Q62186	Translocon-associated protein subunit delta OS=Mus musculus GN=Ssr4 PE=2 SV=1 - [SSRD_MOUSE]	-0.25	-0.12	-0.33	-0.25	0.01
Q9CQ48	NudC domain-containing protein 2 OS=Mus musculus GN=Nudcd2 PE=1 SV=1 - [NUDC2_MOUSE]	0.34	0.18	0.17	0.19	0.01

P54987	Cis-aconitate decarboxylase OS=Mus musculus GN=Irg1 PE=1 SV=2 - [IRG1_MOUSE]	0.65	0.33	0.69	0.37	0.01
Q08EC7	Pla2g4c protein OS=Mus musculus GN=Pla2g4c PE=2 SV=1 - [Q08EC7_MOUSE]	0.22	0.27	0.15	0.12	0.01
Q8BYH8-2	Isoform 2 of Chromodomain-helicase-DNA-binding protein 9 OS=Mus musculus GN=Chd9 - [CHD9_MOUSE]	0.25	0.45	0.20	0.39	0.01
Q8BUG5-2	Isoform 2 of Calcyphosin-2 OS=Mus musculus GN=Caps2 - [CAYP2_MOUSE]	3.95	3.87	2.08	2.00	0.01
A3KGQ6	Actin-related protein 2/3 complex subunit 5-like protein OS=Mus musculus GN=Arpc5l PE=2 SV=1 - [A3KGQ6_MOUSE]	-0.49	-0.34	-0.50	-0.21	0.01
F7C090	Beclin-1 (Fragment) OS=Mus musculus GN=Becn1 PE=2 SV=1 - [F7C090_MOUSE]	0.46	0.23	0.53	0.30	0.01
Q9D7A8	Armadillo repeat-containing protein 1 OS=Mus musculus GN=Armc1 PE=2 SV=1 - [ARMC1_MOUSE]	-0.10	-0.12	-0.21	-0.11	0.01
Q6DVA0	LEM domain-containing protein 2 OS=Mus musculus GN=Lemd2 PE=1 SV=1 - [LEMD2_MOUSE]	-0.21	-0.30	-0.51	-0.36	0.01
Q80WJ7	Protein LYRIC OS=Mus musculus GN=Mtdh PE=1 SV=1 - [LYRIC_MOUSE]	0.44	0.30	0.68	0.70	0.01
M0QWQ9	Uncharacterized protein OS=Mus musculus GN=Gm17174 PE=4 SV=1 - [M0QWQ9_MOUSE]	0.60	0.70	1.19	1.29	0.01
Q8C5L3-3	Isoform 3 of CCR4-NOT transcription complex subunit 2 OS=Mus musculus GN=Cnot2 - [CNOT2_MOUSE]	0.53	0.38	0.84	0.87	0.01
Q6PAV2-2	Isoform 2 of Probable E3 ubiquitin-protein ligase HERC4 OS=Mus musculus GN=Herc4 - [HERC4_MOUSE]	-0.22	-0.24	-0.47	-0.41	0.01
P27612	Phospholipase A-2-activating protein OS=Mus musculus GN=Plaa PE=2 SV=4 - [PLAP_MOUSE]	0.40	0.50	0.23	0.25	0.01
Q8BNQ3	Integral membrane protein GPR137B OS=Mus musculus GN=Gpr137b PE=1 SV=2 - [G137B_MOUSE]	1.37	2.02	0.77	1.42	0.01
E9Q3E1	Aldehyde dehydrogenase OS=Mus musculus GN=Aldh3b2 PE=2 SV=1 - [E9Q3E1_MOUSE]	0.32	0.45	0.17	0.39	0.01
D6RIJ8	Protein 2700029M09Rik OS=Mus musculus GN=2700029M09Rik PE=4 SV=1 - [D6RIJ8_MOUSE]	-0.53	-0.74	-0.28	-0.50	0.01
Q9D1L0	Coiled-coil-helix-coiled-coil-helix domain-containing protein 2, mitochondrial OS=Mus musculus GN=Chchd2 PE=1 SV=1 - [CHCH2_MOUSE]	0.32	0.42	0.20	0.21	0.01
Q9WUX5-2	Isoform 2 of Protein MRVI1 OS=Mus musculus GN=Mrvi1 - [MRVI1_MOUSE]	0.37	0.36	0.34	0.14	0.01

Q9R0N5	Synaptotagmin-5 OS=Mus musculus GN=Syt5 PE=1 SV=1 - [SYT5_MOUSE]	-0.42	-0.54	-0.21	-0.33	0.01
Q9DCT1	1,5-anhydro-D-fructose reductase OS=Mus musculus GN=Akr1e2 PE=1 SV=1 - [AKCL2_MOUSE]	0.19	0.28	0.13	0.14	0.01
Q61990-2	Isoform 2 of Poly(rC)-binding protein 2 OS=Mus musculus GN=Pcbp2 - [PCBP2_MOUSE]	0.14	0.16	0.27	0.13	0.01
Q61233	Plastin-2 OS=Mus musculus GN=Lcp1 PE=1 SV=4 - [PLSL_MOUSE]	0.14	0.12	0.22	0.26	0.01
Q9CPQ3	Mitochondrial import receptor subunit TOM22 homolog OS=Mus musculus GN=Tomm22 PE=2 SV=3 - [TOM22_MOUSE]	0.40	0.89	0.55	0.91	0.01
Q3TPM3	Cullin-1 (Fragment) OS=Mus musculus GN=Cul1 PE=2 SV=1 - [Q3TPM3_MOUSE]	-0.38	-0.44	-0.18	-0.26	0.01
D3Z598	Latent-transforming growth factor beta-binding protein 4 OS=Mus musculus GN=Ltbp4 PE=2 SV=1 - [D3Z598_MOUSE]	-0.03	-0.04	-0.05	-0.02	0.01
O55142	60S ribosomal protein L35a OS=Mus musculus GN=Rpl35a PE=2 SV=2 - [RL35A_MOUSE]	-0.51	-0.36	-0.53	-0.21	0.01
P01849	T-cell receptor alpha chain C region OS=Mus musculus GN=Tcra PE=1 SV=1 - [TCA_MOUSE]	0.61	0.51	0.35	0.25	0.01
Q9D281	Protein Noxp20 OS=Mus musculus GN=Fam114a1 PE=2 SV=1 - [NXP20_MOUSE]	-0.18	-0.38	-0.20	-0.34	0.01
Q8BKL1	Protein SSXT OS=Mus musculus GN=Ss18 PE=2 SV=1 - [Q8BKL1_MOUSE]	0.68	0.40	1.04	0.76	0.01
Q9QZR5-4	Isoform 4 of Homeodomain-interacting protein kinase 2 OS=Mus musculus GN=Hipk2 - [HIPK2_MOUSE]	-1.75	-1.01	-1.48	-0.74	0.01
H3BJR7	WD repeat-containing protein 37 (Fragment) OS=Mus musculus GN=Wdr37 PE=2 SV=1 - [H3BJR7_MOUSE]	0.57	0.66	0.28	0.37	0.01
A2AJ15	Endoplasmic reticulum mannosyl-oligosaccharide 1,2-alpha-mannosidase OS=Mus musculus GN=Man1b1 PE=2 SV=1 - [MA1B1_MOUSE]	-0.28	-0.34	-0.59	-0.60	0.01
O88544	COP9 signalosome complex subunit 4 OS=Mus musculus GN=Cops4 PE=1 SV=1 - [CSN4_MOUSE]	-0.12	-0.21	-0.14	-0.27	0.01
P51885	Lumican OS=Mus musculus GN=Lum PE=1 SV=2 - [LUM_MOUSE]	0.20	0.16	0.34	0.33	0.01
Q8K2K6-3	Isoform 3 of Arf-GAP domain and FG repeat-containing protein 1 OS=Mus musculus GN=Agfg1 - [AGFG1_MOUSE]	0.73	0.42	0.88	0.43	0.01
Q91VC7	Protein phosphatase 1 regulatory subunit 14A OS=Mus musculus GN=Ppp1r14a PE=2 SV=1 - [PP14A_MOUSE]	0.27	0.17	0.38	0.20	0.01

Q80W00-2	Isoform 2 of Serine/threonine-protein phosphatase 1 regulatory subunit 10 OS=Mus musculus GN=Ppp1r10 - [PP1RA_MOUSE]	0.14	0.25	0.26	0.36	0.01
A2A8L5	Receptor-type tyrosine-protein phosphatase F OS=Mus musculus GN=Ptprf PE=1 SV=1 - [PTPRF_MOUSE]	0.13	0.18	0.09	0.22	0.01
Q923D2	Flavin reductase (NADPH) OS=Mus musculus GN=Blvrb PE=2 SV=3 - [BLVRB_MOUSE]	-0.08	-0.10	-0.16	-0.19	0.01
Q6PAR5-2	Isoform 2 of GTPase-activating protein and VPS9 domain-containing protein 1 OS=Mus musculus GN=Gapvd1 - [GAPD1_MOUSE]	-0.08	-0.12	-0.20	-0.17	0.01
Q8K2H2	OTU domain-containing protein 6B OS=Mus musculus GN=Otud6b PE=2 SV=1 - [OTU6B_MOUSE]	0.21	0.17	0.08	0.20	0.01
A2ANU3	Synapse differentiation-inducing gene protein 1 OS=Mus musculus GN=Syndig1 PE=1 SV=1 - [SYNG1_MOUSE]	-0.34	-0.65	-0.32	-0.63	0.01
Q8BMS4	Hexaprenyldihydroxybenzoate methyltransferase, mitochondrial OS=Mus musculus GN=Coq3 PE=2 SV=1 - [COQ3_MOUSE]	0.21	0.17	0.12	0.09	0.01
O55126	Protein NipSnap homolog 2 OS=Mus musculus GN=Gbas PE=2 SV=1 - [NIPS2_MOUSE]	-0.26	-0.27	-0.10	-0.18	0.01
Q9Z2D0	Myotubularin-related protein 9 OS=Mus musculus GN=Mtmr9 PE=2 SV=2 - [MTMR9_MOUSE]	-0.75	-0.55	-0.48	-0.29	0.01
Q8C4Y3	Negative elongation factor B OS=Mus musculus GN=Nelfb PE=2 SV=2 - [NELFB_MOUSE]	-0.39	-0.19	-0.36	-0.20	0.01
P56565	Protein S100-A1 OS=Mus musculus GN=S100a1 PE=1 SV=2 - [S10A1_MOUSE]	-0.58	-0.61	-0.23	-0.47	0.01
P10639	Thioredoxin OS=Mus musculus GN=Txn PE=1 SV=3 - [THIO_MOUSE]	-0.22	-0.35	-0.14	-0.23	0.01
Q63850	Nuclear pore glycoprotein p62 OS=Mus musculus GN=Nup62 PE=1 SV=2 - [NUP62_MOUSE]	0.60	0.33	0.76	0.88	0.01
P08730	Keratin, type I cytoskeletal 13 OS=Mus musculus GN=Krt13 PE=1 SV=2 - [K1C13_MOUSE]	0.45	0.85	0.40	0.80	0.01
Q9D394-2	Isoform 2 of Protein RUFY3 OS=Mus musculus GN=Rufy3 - [RUFY3_MOUSE]	0.16	0.20	0.15	0.07	0.01
P39447	Tight junction protein ZO-1 OS=Mus musculus GN=Tjp1 PE=1 SV=2 - [ZO1_MOUSE]	0.72	0.42	1.11	0.80	0.01
Q8K3H0	DCC-interacting protein 13-alpha OS=Mus musculus GN=Appl1 PE=1 SV=1 - [DP13A_MOUSE]	0.10	0.21	0.20	0.11	0.01
P33434-2	Isoform 2 of 72 kDa type IV collagenase OS=Mus musculus GN=Mmp2 - [MMP2_MOUSE]	1.05	0.45	0.93	0.60	0.01

Q9CWF2	Tubulin beta-2B chain OS=Mus musculus GN=Tubb2b PE=1 SV=1 - [TBB2B_MOUSE]	-0.49	-0.48	-0.25	-0.25	0.01
Q6NXI6-2	Isoform 2 of Regulation of nuclear pre-mRNA domain-containing protein 2 OS=Mus musculus GN=Rprd2 - [RPRD2_MOUSE]	0.39	0.21	0.35	0.18	0.01
Q9DBG6	Dolichyl-diphosphooligosaccharide--protein glycosyltransferase subunit 2 OS=Mus musculus GN=Rpn2 PE=2 SV=1 - [RPN2_MOUSE]	-0.32	-0.21	-0.50	-0.29	0.01
[truncated: 539,297 more chars]
